# Recurrent erosion of *COA1/MITRAC15* exemplifies conditional gene dispensability in oxidative phosphorylation

**DOI:** 10.1038/s41598-021-04077-y

**Published:** 2021-12-24

**Authors:** Sagar Sharad Shinde, Sandhya Sharma, Lokdeep Teekas, Ashutosh Sharma, Nagarjun Vijay

**Affiliations:** Computational Evolutionary Genomics Lab, Department of Biological Sciences, IISER Bhopal, Bhauri, Madhya Pradesh India

**Keywords:** Evolution, Molecular evolution

## Abstract

Skeletal muscle fibers rely upon either oxidative phosphorylation or the glycolytic pathway with much less reliance on oxidative phosphorylation to achieve muscular contractions that power mechanical movements. Species with energy-intensive adaptive traits that require sudden bursts of energy have a greater dependency on glycolytic fibers. Glycolytic fibers have decreased reliance on OXPHOS and lower mitochondrial content compared to oxidative fibers. Hence, we hypothesized that gene loss might have occurred within the OXPHOS pathway in lineages that largely depend on glycolytic fibers. The protein encoded by the *COA1/MITRAC15* gene with conserved orthologs found in budding yeast to humans promotes mitochondrial translation. We show that gene disrupting mutations have accumulated within the *COA1* gene in the cheetah, several species of galliform birds, and rodents. The genomic region containing *COA1* is a well-established evolutionary breakpoint region in mammals. Careful inspection of genome assemblies of closely related species of rodents and marsupials suggests two independent *COA1* gene loss events co-occurring with chromosomal rearrangements. Besides recurrent gene loss events, we document changes in *COA1* exon structure in primates and felids. The detailed evolutionary history presented in this study reveals the intricate link between skeletal muscle fiber composition and the occasional dispensability of the chaperone-like role of the *COA1* gene.

## Introduction

Skeletal muscles control numerous locomotor functions in vertebrates^1^. The hundreds of different muscles in the body consist of highly organized heterogeneous bundles of fibers. These fibers are classified based on the contractile properties, power source, and myosin component into type-1, 2A, 2B, and 2X^2^. Muscles with aerobic type-1 and 2A fibers rely on the oxidative phosphorylation (OXPHOS) pathway for sustained locomotion^3^. Locomotion that requires sudden energy bursts depends on the predominantly anaerobic 2B and 2X fibers^4^. OXPHOS is the energy-releasing electron transport chain (ETC) coupled with the energy-requiring chemiosmosis^5,6^. A chain of mitochondrial inner membrane-embedded proteins encoded by mitochondrial and nuclear genes forms four large complexes that transport electrons through redox reactions. The energy released results in a proton gradient, which uses a fifth membrane-embedded complex to generate ATP through chemiosmosis. The individual complexes are known to assemble into supercomplexes^7^. Optimization of the OXPHOS pathway leads to improved locomotor performance^8^. However, degeneration of locomotor abilities leads to a relaxed selective constraint on the OXPHOS pathway^9,10^. The special locomotory needs of galliform birds, rodents, marsupials, and felids lead to a greater reliance on anaerobic fast fibers for sudden bursts of energy^11–13^.

The ability to fly is a distinctive feature of birds except for lineages that have become entirely flightless or retain only a limited flying capacity^14–17^. The large amount of energy required for flight has necessitated a high metabolic rate in birds^18^. Increased ATP generation fulfills these energy demands through metabolic adaptations in the OXPHOS pathway^19^. Avian flight is possible through a combination of flight muscles that consist of anaerobic white (fast glycolytic), intermediate/red-pink (fast oxidative), and aerobic red (slow oxidative) fibers^20–22^. Birds with strong flight abilities, such as long-distance migrants and small passerines, contain fast oxidative fibers^23^. However, galliform species have mostly glycolytic fibers to allow short bursts of activity^24^. Hence, the OXPHOS pathway is under stronger selective constraint in non-galliform bird species than galliform birds due to the functional specialization of mitochondria to different muscle fibers^25^.

The cheetah (*Acinonyx jubatus*) epitomizes the relevance of speed and acceleration^26^. In general, felids are adept at sprinting and can accelerate more rapidly than canids but cannot sustain them for a prolonged period^27^. The predominance of type-2X fibers in felids provides the ability of rapid acceleration^28–30^. Compared to canids, felids have a greater reliance on glycolytic fibers. In smaller mammals such as rodents, the higher relative speed results from faster constriction by the higher proportion of glycolytic fibers (mostly 2X and 2B)^31^. For instance, rodent limbs have more abundant type 2B fibers compared to larger mammals (including humans, which have no type 2B fibers in the limbs)^32,33^. Marsupial species also attain high relative speeds with glycolytic fibers (2B and 2X)^34,35^. The higher proportion of fast glycolytic fibers in felids, rodents, and marsupials results in relaxed selection on the OXPHOS pathway genes in these species. Glycolytic fibers have decreased reliance on OXPHOS and lower mitochondrial content than oxidative fibers^25,36^.

Functional studies implicate *COA1* (also known as *MITRAC15*) in promoting mitochondrial translation and complex I and IV biogenesis^37,38^. However, overexpression of other genes easily compensates for the mild effect of *COA1* gene knockout^39,40^. Notably, the *COA1* gene was also identified as a positively selected gene in a genome-wide screen in primates^41^ and suggests that COA1 can contribute to fitness increases through its role as a chaperone despite its mild phenotype. Our study evaluates whether the protein encoded by the *COA1* gene, a mitochondrial complex I translation factor with a chaperone-like role, is dispensable when the OXPHOS pathway is under relaxed selective constraints. The integration of the mitochondria in the host cell after endosymbiosis has involved an initial recruitment phase for OXPHOS proteins that increased the number of components followed by loss and length reduction made possible by mutual functional compensation^42,43^. We hypothesized that the OXPHOS pathway might have experienced reduced purifying selection in felids, rodents, marsupials, and galliform birds based on an increased proportion of glycolytic fibers. Duplicate copies or alternative metabolic pathways compensate for gene function and decide gene dispensability^44^. Hence, to evaluate our hypothesis, we aim to (1) investigate whether *COA1* has any homologs that could compensate its function, (2) screen the genomes of vertebrate species to identify and track the evolutionary history of *COA1* orthologs, (3) identify evidence of gene disruptive changes within the *COA1* locus using several types of high-throughput datasets and (4) reconstruct the sequence of events associated with the potential erosion of the *COA1* locus due to chromosomal rearrangement (CR) events at the evolutionary breakpoint region (EBR) spanning the *COA1* gene. We extensively screened publicly available genomes and transcriptomes of more than 365 vertebrate species to establish recurrent loss of the widely conserved *COA1* gene.

## Results

### *COA1* is a distant homolog of *TIMM21*

**Figure 1:**
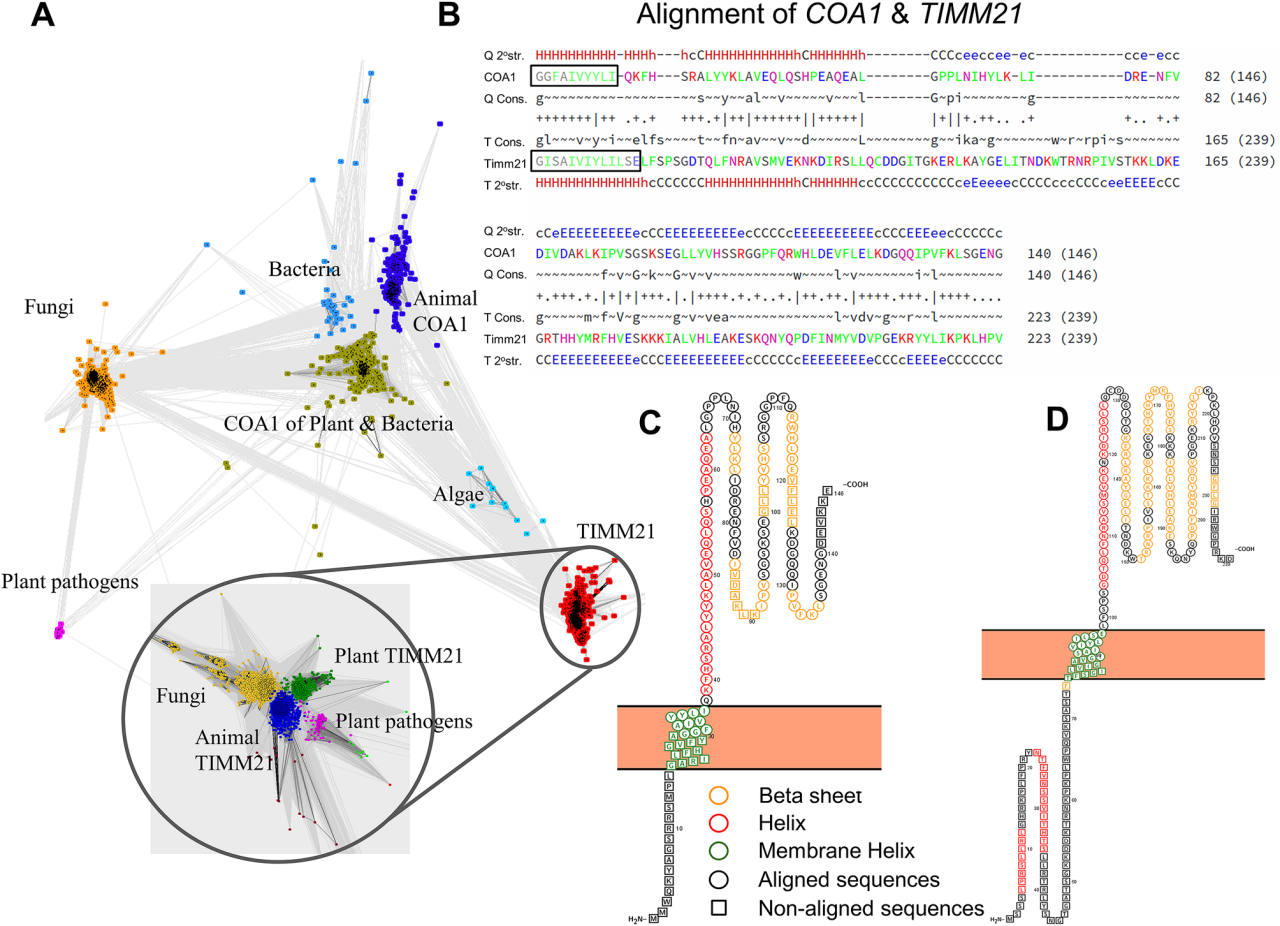
*COA1/MITRAC15* and *TIMM21* are distant homologs with similar amino acid sequence profiles and secondary structures. (**A**) CLANS generated a cluster map of *COA1* and *TIMM21* using amino acid sequences downloaded from the Pfam 34.0 database. Different colors represent distinct clusters: *COA1* is dark blue, *TIMM21* is red, homologs of *COA1* from species of fungi are in dark yellow, homologs of *COA1* from bacterial species are in the light blue cluster. *COA1* homologs in plants and bacteria are in forest green color, *COA1* from algae are in aqua blue, and homologs of *COA1* from plant pathogens are in magenta. The inset is the zoomed-in figure of *TIMM21* sequence clusters where the cluster of fungi is in yellow, animals in dark blue, plant pathogens in magenta, and the cluster of plants in dark green (Supplementary Fig. S5). (**B**) The output of HHpred shows the alignment of human *COA1* with yeast *TIMM21*. The region in the box highlights the predicted transmembrane helix. The ‘Q’ and ‘T’ refer to the query and template sequences in the alignment. The sequences, ‘2^0^ str.’ and ‘Cons.’ denote the PSI-PRED secondary structure prediction and confidence values where H = helix, E = strand, C = loops, and CAPS = strong prediction in secondary structure prediction. The upper- and lower-case amino acids in the consensus sequences indicate high (> ~ 60%) and moderate (> ~ 40%) conservation, respectively. Symbols indicating the quality of the column-column match: ‘|’ very good, ‘+’ good, ‘.’ neutral, ‘_’ bad and ‘=’ very bad. (**C**) The PROTEUS2 predicted secondary structure of human (*Homo sapiens) COA1* is displayed using PROTTER. (**D**) The PROTEUS2 predicted secondary structure of yeast (*Saccharomyces cerevisiae*) *TIMM21* is displayed using PROTTER.

We identified that the *TIMM21* gene is a distant homolog of *COA1* based on Position-specific iterative Basic local alignment search tool (PSI-Blast) and HMM-HMM–based lightning-fast iterative sequence search (HHblits) iterative profile-profile search of the Uniprot database. Clustering *COA1* and *TIMM21* homologous sequences from the Pfam 34.0 database using CLANS finds distinct swarms (see Fig. 1A). The *COA1* homologs form separate clusters for bacteria, fungi, plants, and animals genes. The inset in Fig. 1A shows the sub-clustering of fungi, plants, and animals within the *TIMM21* swarm (see Supplementary Text S1, Supplementary Figs. S1–S9, and Supplementary File S1–S5). The pairwise alignment of the human *COA1* protein sequence with the *TIMM21* protein (from *Saccharomyces cerevisiae*) shows that regions with the most substantial homology include the membrane-spanning domain and cover > 100 residues (see Fig. 1B and Supplementary Fig. S10). In addition to the primary sequence-homology detected, both *TIMM21* and *COA1* are known to play prominent roles in the mitochondria and have comparable secondary structures (see Fig. 1C,D). The strong homology between these proteins also allows for homology-based modeling of the tertiary structure of the *COA1* protein using *TIMM21* as a model (see Supplementary Fig. S9). The de novo predicted structures of *TIMM21* and *COA1* from the AlphaFold^45^ protein structure database are also highly similar (see Supplementary Figs. S11–S16). Despite the lack of well-conserved motifs, we found three well-matching columns (marked with a '|' sign in Fig. 1B) between residues 91 to 95 in *COA1*. Two consecutive conserved residues occur at positions 57–58, 64–65, and 67–68 of *COA1*. The similar sequence, structure, and function of *COA1* and *TIMM21* strongly support that these genes are homologs. The presence of distinct *COA1* and *TIMM21* orthologs in fungi, animals, plants, and protists suggests both homologs existed in the LECA (Last Eukaryotic Common Ancestor).

### *COA1* gene duplication, pseudogenisation, and exon reorganization

The *COA1* gene has undergone independent gene duplications followed by pseudogenisation and degeneration of the duplicated copy in primates, carnivores, and a few rodent species (see Supplementary Text S2A, Supplementary Tables S1–S3, and Supplementary Figs. S17–S48). However, intact transcriptionally active *COA1* gene copies are present in more than 300 vertebrate species. In the cheetah (*Acinonyx jubatus*), the open reading frame at the *COA1* locus identified based on conserved synteny is disrupted (see Fig. 2). The cheetah gene disrupting premature stop codon is due to a single base C → T substitution at the 27th base of exon-2 assembled at the *COA1* locus (see Supplementary Table S1 and Supplementary Fig. S49–S51). The duplicated copy of *COA1* also contains a premature stop codon at the 49th base of exon-2 caused by a single base insertion at the 11th base of exon-2 (see Supplementary Figs. S52–S54). The *COA1* gene transcripts are missing in the skin transcriptome of the cheetah (see Supplementary Figs. S55–S57). We evaluated the possibility of alternative splice isoforms and potential assembly errors in great detail to rule out the case of an intact reading frame. We find multiple lines of evidence support *COA1* gene loss in the cheetah (see Supplementary Text S2B and Supplementary Figs. S19 and S55–S77). Gene loss in the cheetah occurred between 2.63 and 3.06 MYA (Supplementary Table S4).

**Figure 2:**
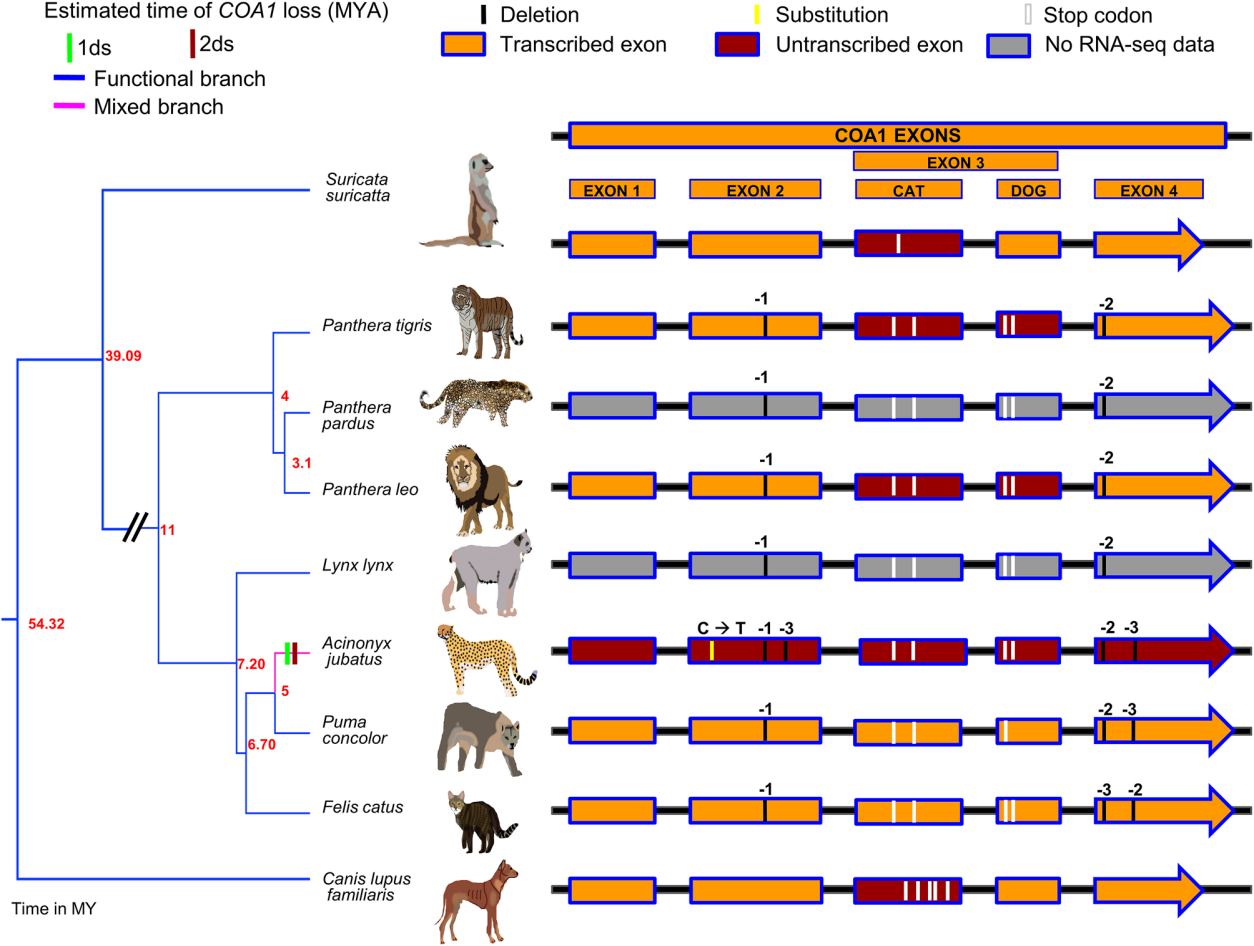
Loss of *COA1/MITRAC15* gene in feliforms. Gene loss event in cheetah *(Acinonyx jubatus*) is visualized using FigTree on a time-calibrated phylogenetic tree downloaded from the TimeTree website. The phylogenetic tree of the Feliformia group is obtained from the study by Johnson 2006^117^. Blue branches in the tree represent functional branches, the pink-colored branches represent mixed (functional + pseudogenic) branches. The method proposed by Meredith et al., 2009 was used to estimate the time of gene loss using two different substitution rate models (1ds and 2ds). Details of the estimated time of gene loss are provided in Supplementary Table S4. The evidence of transcription is shown in Supplementary Figs. S24–S42, S55–S57, S920–S924, S944–S949. The detailed figure showing gene order is in Supplementary Fig. S23.

### *COA1* gene loss in galliform species

**Figure 3:**
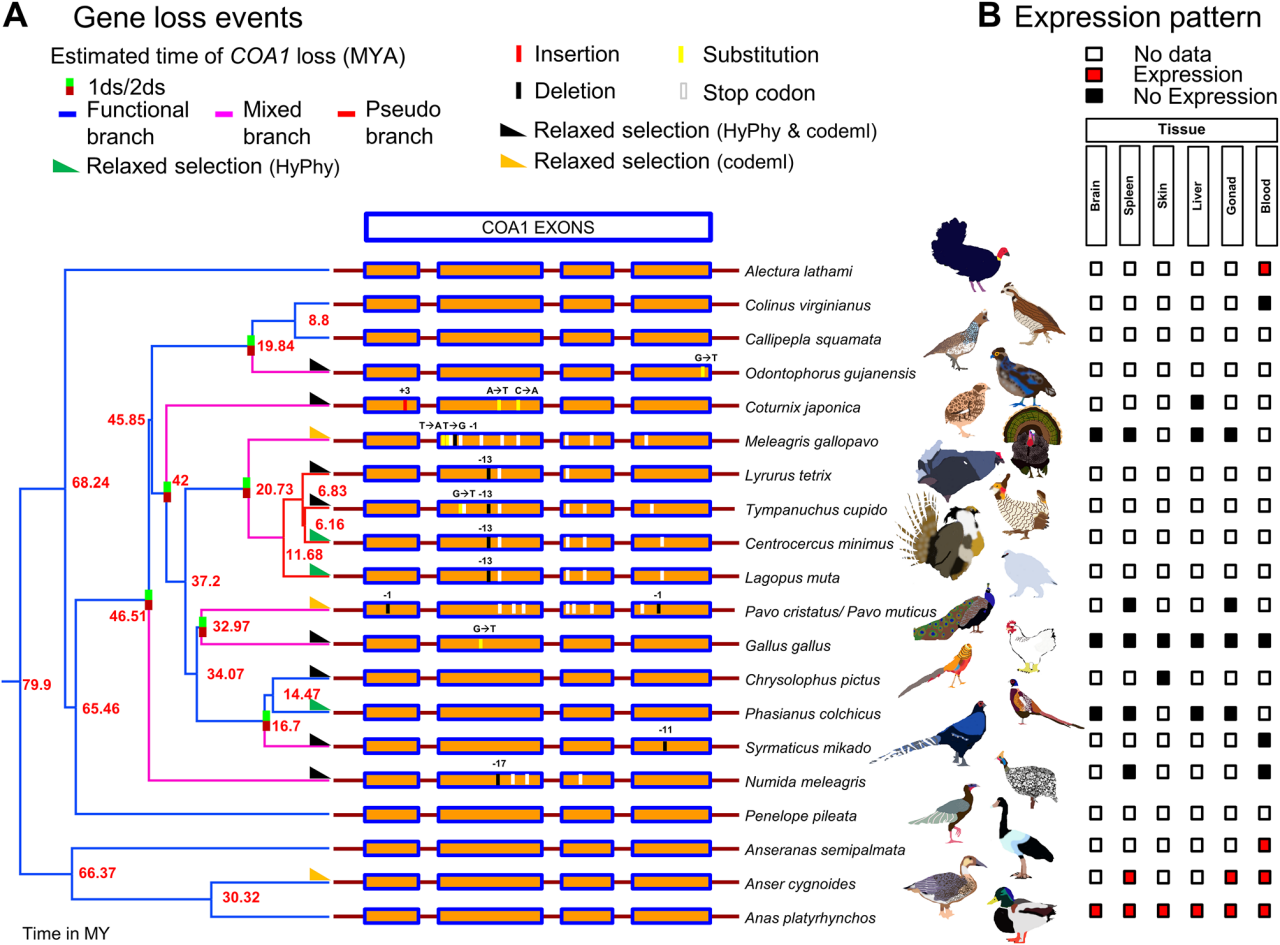
Recurrent loss of *COA1/MITRAC15* gene in galliform species. (**A**) Gene loss events in eleven galliform species along a time-calibrated phylogenetic tree from the TimeTree website are visualized in FigTree. Blue branches in the tree represent functional branches, the pink-colored branches represent mixed (functional + pseudogenic) branches, and red-colored branches represent pseudo branches. The method proposed by Meredith et al., 2009 was used to estimate the time of gene loss using two different substitution rate models (1ds and 2ds). Details of the estimated time of gene loss are provided in Supplementary Table S4. Short colored bars depict the locations of the gene disrupting mutations on the four exons of *COA1* (Supplementary Table S5). (**B**) The *COA1* gene expression pattern in six tissues (brain, spleen, skin, liver, gonad, and blood) was assessed by screening RNA-seq datasets (Supplementary Figs. S466–S626). The red-colored blocks depict the robust expression of the *COA1* gene, the black-colored blocks depict a lack of *COA1* gene expression in that particular tissue, and the white-colored blocks represent a lack of data for that tissue. A black right-angle triangle represents lineages detected to be under relaxed selection by both HyPhy RELAX and codeml. Those detected only by codeml are shown as orange right-angled triangles, and those detected only by HyPhy RELAX are shown as dark green right-angled triangles (Supplementary Tables S7, S8, and S9).

We found evidence of eight independent gene-disruption events in the *COA1* gene in the galliform group (see Fig. 3A). The chicken (*Gallus gallus*) and Amazonian wood quail (*Odontophorus gujanensis*) have single-base G → T substitutions at the 69th base of exon-2 and the 72nd base of exon-4 in the *COA1* gene, respectively (see Supplementary Table S5). These substitutions lead to (GAA → TAA) premature stop codons. Gene loss of *COA1* is estimated to be around 32.97 MYA in chicken and approximately 19.84 MYA in the Amazonian wood quail (see Supplementary Tables S4 and S5). In the Indian peafowl (*Pavo cristatus*), two single-base deletions, one at 37th base of exon-1 and another at 31st base of exon-4, introduce frameshifts resulting in premature stop codons in exons 2, 3, and 4. The gene disrupting mutations identified in the Indian peafowl (*Pavo cristatus*) also occur in the green peafowl (*Pavo muticus*). Loss of the *COA1* gene is estimated to have happened around 32.97 MYA in the peafowls (see Supplementary Tables S4 and S5). The exon-2 of pinnated grouse (*Tympanuchus cupido*) and helmeted guineafowl (*Numida meleagris*) have independent 13 and 17 base deletions, which lead to several frameshift induced premature stop codons (see Supplementary Table S5). The 13-base deletion in the exon-2 of the pinnated grouse (*Tympanuchus cupido*) also occurs in Gunnison grouse (*Centrocercus minimus*), rock ptarmigan (*Lagopus muta*), and the black grouse (*Lyrurus tetrix*) (see Supplementary Table S5). The estimated time of gene loss in these four species is around 20.73 MYA, and for helmeted guineafowl is around 46.51 MYA (see Supplementary Table S4).

In turkey (*Meleagris gallopavo*)*,* a two-base substitution at bases 7 and 8 and a single base deletion at the 37th base of exon-2 result in a frameshift in the COA1 gene leading to premature stop codons. Gene loss in turkey is estimated to have occurred around 20.73 MYA (see Supplementary Tables S4 and S5). Two closely spaced single base substitutions (**A**A**C** → **T**A**A**) at 48th and 50th positions of exon-2 result in a premature stop codon in the Japanese quail (*Coturnix japonica*). The time of gene loss in the Japanese quail is estimated at around 42 MYA (see Supplementary Tables S4 and S5). The mikado pheasant (*Syrmaticus mikado*) has a frame-disrupting 11-base deletion in exon-4, and the time of gene loss is around 16.7 MYA (see Supplementary Tables S4 and S5). Other galliform species such as Australian brushturkey (*Alectura lathami*)*,* northern bobwhite (*Colinus virginianus*), blue quail (*Callipepla squamata*)*,* ring-necked pheasant (*Phasianus colchicus*)*,* golden pheasant (*Chrysolophus pictus*), and white-crested guan (*Penelope pileata*) have intact *COA1* coding sequences. The coding region is intact in outgroup species such as swan goose (*Anser cygnoides*), duck (*Anas platyrhynchos*), and magpie goose (*Anseranas semipalmata*). Five genes upstream (*BLVRA*, *VOPP1*, *LANCL2*, *EGFR,* and *SEC61G*) and downstream (*STK17A*, *HECW1*, *MRPL32*, *PSMA2,* and *C7orf25*) from *COA1* retain a conserved order in birds (see Supplementary Table S6). We relied upon this conserved order to verify the 1-to-1 orthology of the *COA1* gene across species (see Supplementary Fig. S78).

Signatures of relaxed selection identified by molecular evolution tools (see “Materials and methods”) in galliform species with gene disrupting changes further support the loss of *COA1* in these lineages (see Supplementary Tables S7, S8, and S9). To evaluate the relevance of the gene disrupting mutations and signatures of relaxed selection identified in galliform species, the transcriptomes of Galloanserae species were screened to assess the transcriptional status of *COA1*. We analyzed RNA-seq datasets of chicken tissues and found that the *COA1* gene is not transcribed despite screening more than 20 tissues (see Fig. 3B). Other Galloanserae species have RNA-seq data available for very few tissues. We evaluated the RNA-seq datasets from six tissues (Brain, Spleen, Skin, Liver, Gonad, and Blood) available in several species for the presence of *COA1* transcripts. Our search consistently revealed transcription of *COA1* gene in Anseriformes species in contrast to lack of transcription in galliform species except for Australian brushturkey (*Alectura lathami*) and northern bobwhite (*Colinus virginianus*), which have intact *COA1* gene that is under strong purifying selection (see Fig. 3B).

Despite intact coding regions, the ring-necked pheasant (*Phasianus colchicus*) and golden pheasant (*Chrysolophus pictus*) *COA1* sequences have signatures of relaxed selection (see Supplementary Table S7). None of the four tissues (Brain, Spleen, Liver, and Gonad) for which RNA-seq data is available from the ring-necked pheasant shows any *COA1* transcripts. Similarly, the one tissue (Skin) for which RNA-seq data is available in the golden pheasant (*Chrysolophus pictus*) does not show *COA1* expression. The lack of gene expression and signatures of relaxed selection in the ring-necked pheasant (*Phasianus colchicus*) and golden pheasant (*Chrysolophus pictus*) suggests gene loss.

**Figure 4:**
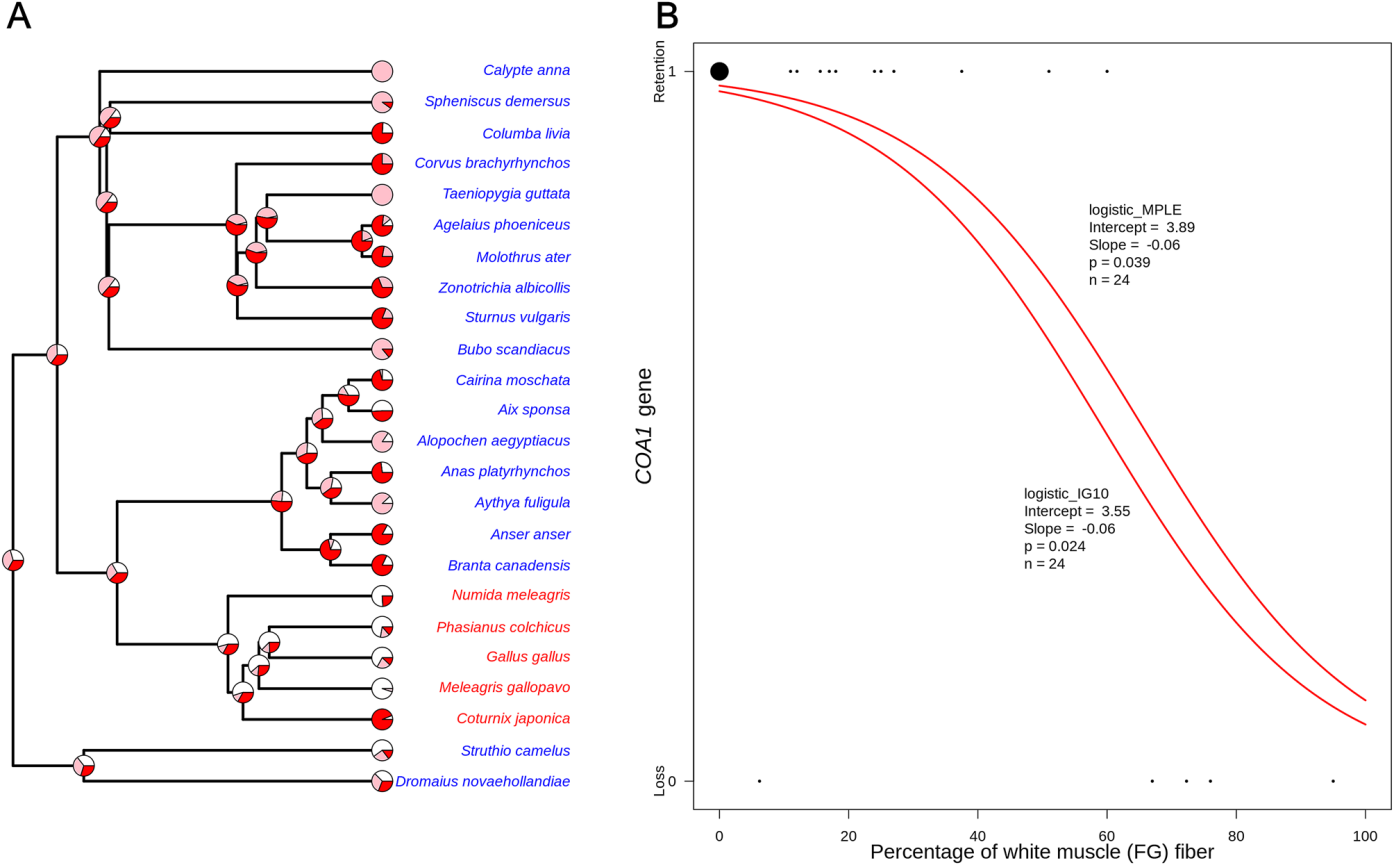
Phylogenetic correlation of skeletal muscle fiber types and loss/retention of *COA1*/*MITRAC15* gene in different bird species. (**A**) Proportion of white, pink, and red muscle fiber in pectoralis muscle depicted in pie charts (for detailed data, see Supplementary Table S10A). The ancestral state of muscle fiber type was estimated using the phytools package version 0.7–90^118^ in R version 4.1.0^114^. Species having lost *COA1* represented in red, species retaining *COA1* shown in blue. The tree is not time-calibrated and is downloaded from the TimeTree website and visualized in phytools package in R. (**B**) Two methods (logistic_MPLE and logistic_IG10) were used for phylogenetic regression analyses to evaluate the effect of the proportion of white fiber (fast glycolytic; FG) on the loss/retention of the *COA1* gene (n = 24). The 8 species without white fibers are represented by the big black dot, all others are shown as tiny individual dots along the FG.

We evaluated the link between gene loss and the percent of glycolytic muscle fibers using comparative quantitative phylogenetic methods (see “Materials and methods”). Galliform species with gene loss tend to have > 60% glycolytic fiber in the pectoralis muscle (see Fig. 4A and Supplementary Table S10A). However, despite losing the *COA1* gene, the Japanese quail (*Coturnix japonica*) has less glycolytic muscle fiber than other galliform birds. The Japanese quail is also the only galliform species with flapping flight^46^. Closer inspection of related species such as the common quail (*Coturnix coturnix*) and red-legged partridge (*Alectoris rufa*) suggests the *COA1* was potentially lost in an ancestral species with a higher glycolytic muscle fiber content (see Supplementary Table S10A). The lineage leading to the Japanese quail subsequently reduced the white fiber in the pectoralis muscle. However, the quail is unlikely to regain the gene due to Dollo's law of irreversibility^47^.

Our results recovered a significant negative correlation between the proportion of white-fast-glycolytic muscle fiber type in pectoralis muscle and *COA1* gene retention (see Fig. 4B and Supplementary Table S10B). This negative correlation suggests that a high proportion of white muscle fiber may have resulted in the loss of *COA1*. Notably, non-galliform flightless birds retain an intact *COA1* gene, are transcriptionally active, and lack signatures of relaxed selection (see Supplementary Text S3 and Supplementary Table S11)*.* Hence, the loss of the *COA1* gene is not associated with flying ability but rather with the proportion of white muscle fiber.

### *COA1* occurs in an evolutionary breakpoint region

**Figure 5:**
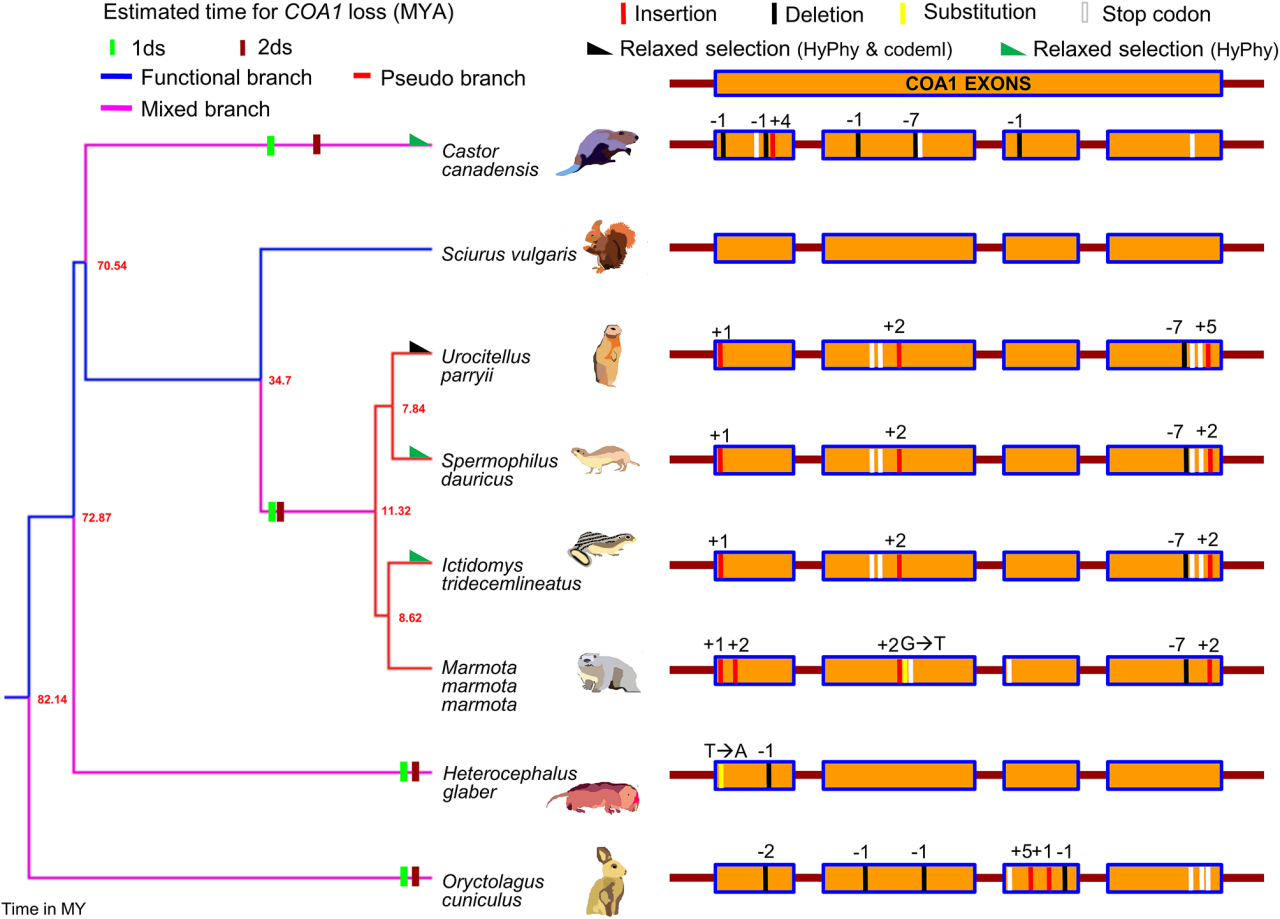
Recurrent loss of *COA1/MITRAC15* gene in rodent species. Gene loss events in seven rodent species through four events are represented exon-wise. Blue branches in the tree represent functional branches, pink-colored branches represent mixed (functional + pseudogenic) branches, and red-colored branches represent pseudo branches. The gene disrupting mutations are shown for these species in *COA1* exon-wise except for *Sciurus vulgaris,* where *COA1* is functional. Details of the estimated time of gene loss are provided in Supplementary Table S4. The events are provided in detail in Supplementary Table S12. A black right-angle triangle represents lineages detected to be under relaxed selection by both HyPhy RELAX and codeml. Those detected only by codeml are shown as orange right-angled triangles, and those detected only by HyPhy RELAX are shown as dark green right-angled triangles (Supplementary Tables S7, S8, and S9). The time-calibrated phylogenetic tree was obtained from the TimeTree website and visualized in FigTree.

We find evidence of *COA1* gene disrupting mutations and lack of gene expression in multiple RNA-seq datasets despite a conserved gene order in the rabbit (*Oryctolagus cuniculus*), naked mole-rat (*Heterocephalus glaber*), and four Sciuridae species (*Urocitellus parryii*, *Spermophilus dauricus*, *Ictidomys tridecemlineatus*, *Marmota marmota marmota*). The gene disrupting mutations identified in the rabbit *COA1* gene includes a two-base pair deletion at the 22nd codon of exon-1 and single base pair deletions in exon-2 at the 13th and 37th codons, respectively. Gene disrupting changes in the third exon consist of a five-base insertion between the 11th and 12th codon, one base insertion at the 17th codon, and one base deletion in the 23^rd^ codon (see Fig. 5 and Supplementary Table S12). These six gene-disrupting changes result in premature stop codons in exon-2 and exon-4. Gene loss in the rabbit is estimated to have occurred between 4.64 MYA and 6.39 MYA (see Fig. 5 and Supplementary Table S4). The lack of *COA1* RNA-seq reads in tissues such as the brain, liver, and testis that express *COA1* in closely related species supports the loss of the *COA1* gene in the naked mole-rat. Besides the lack of a start codon, a single gene disrupting mutation is found in the naked mole-rat *COA1* gene and consists of a single base deletion at the 21st codon of exon-1. Gene loss in the naked mole-rat is estimated between 4.53 MYA and 6.12 MYA (see Supplementary Tables S4, S12, and Fig. 5).

The presence of common gene disrupting changes such as the one base pair insertion at second codon of exon-1, two base pair insertion at 25th codon of exon-2, seven base pair deletion between 25 and 26th codon of exon-4, and a 2-base insertion at 33rd codon of exon-4 supports a shared gene loss in four Sciuridae species (*Urocitellus parryii*, *Spermophilus dauricus*, *Ictidomys tridecemlineatus*, *Marmota marmota marmota*). The *COA1* gene of alpine marmot has additional gene disrupting changes consisting of a 2-base insertion between the 8th and 9th codon of exon-1 and a single nucleotide substitution at the 26th codon of exon-2. The 2-base insertion at the 33rd codon of exon-4 has extended to a five-base pair insertion in the Daurian ground squirrel (*Spermophilus dauricus*). The estimated time of gene loss for this shared event is between 30.62 MYA and 31.74 MYA (see Supplementary Tables S4, S12, and Fig. 5). The presence of intact open reading frames robustly expressed at syntenic locations in closely related species (~ 30–50 million years) strongly supports at least three independent *COA1* gene loss events (i.e., one in rabbit, one in the four Sciuridae species, and one in the naked mole-rat; see Fig. 5).

Multiple gene-disrupting mutations in the *COA1* gene of the North American beaver (*Castor canadensis*) suggest a fourth independent gene loss event. Gene-disrupting mutations in the beaver result in at least two premature stop codons. In the first exon, single-base deletions occur in the 3^rd^ and 20th codon, a four-base insertion occurs between 33rd and 34th codon. The second exon has a single-base deletion in the 33^rd^ codon and a seven-base pair deletion between 29 and 30th codons. A single base deletion occurs at the 12th codon of exon-3 (see Supplementary Table S12 and Fig. 5). The Illumina sequencing raw reads support the gene disrupting mutations identified in the genome assembly (Supplementary File S6), and duplicate copies do not occur. The loss of the *COA1* gene in the beaver is estimated to have happened sometime between 26.30 MYA and 32.40 MYA (see Supplementary Table S4 and Fig. 5).

**Figure 6:**
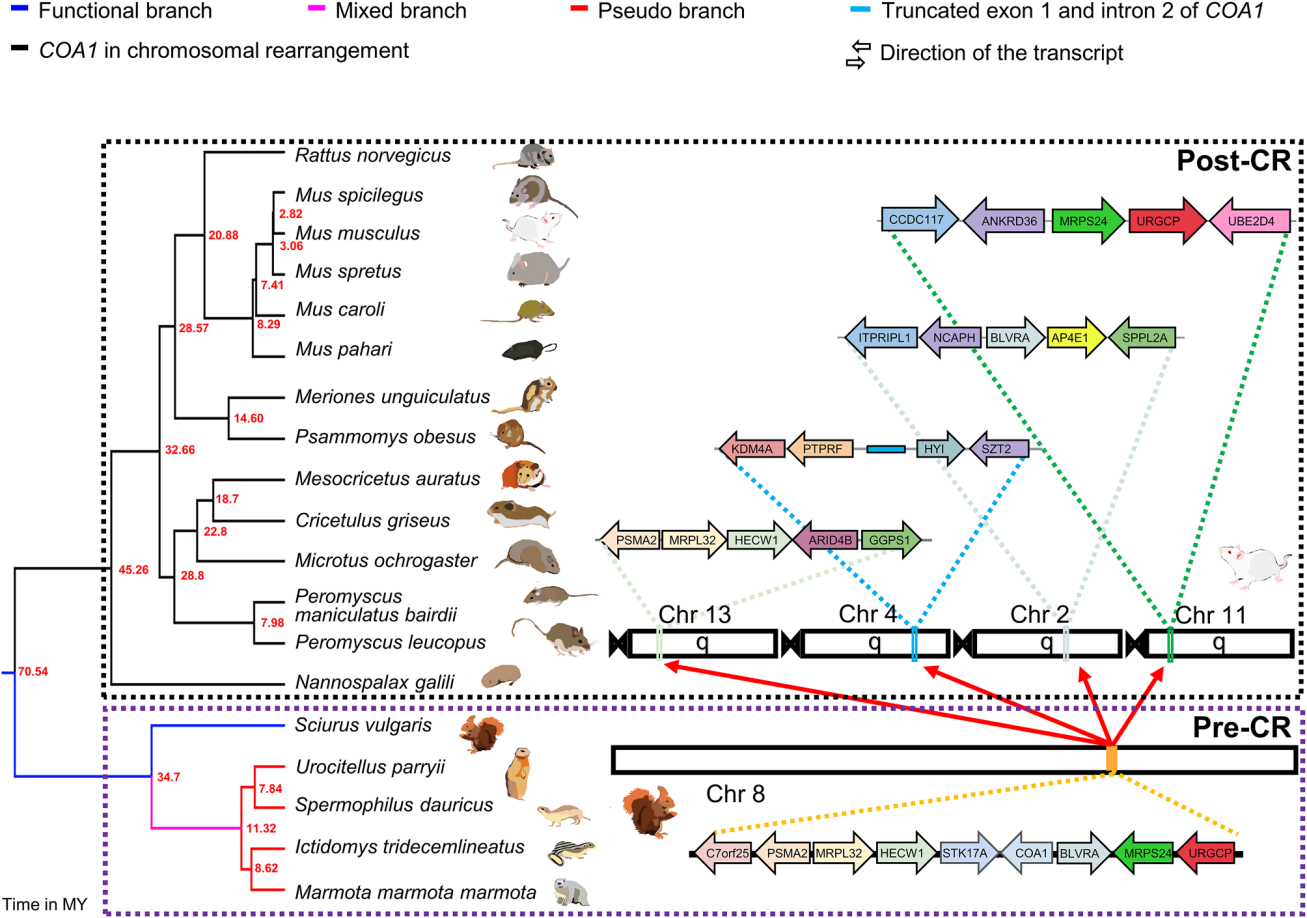
Recurrent loss of *COA1/MITRAC15* gene in rodent species. Gene loss events in fourteen rodent species are shown to occur through a chromosomal rearrangement (CR) event. Blue branches in the tree represent functional branches, the pink-colored branches represent mixed (functional + pseudogenic), and red-colored branches represent pseudogenic branches. The black branches correspond to the species that have lost the gene due to the rearrangement at the evolutionary breakpoint region (EBR). Gene order in the genomic region flanking the *COA1* gene in rodent species is shown with arrows that depict the direction of gene transcription relative to the *COA1* gene for consistency across species. Boxes represent the genes located on short scaffolds with unknown orientation. The black (post-CR) and purple (pre-CR) dotted boxes represent post- and pre-chromosomal rearrangement gene orders, respectively. The *Mus musculus* chromosomes are used to represent the post-chromosomal rearrangement. Chromosomes 4 and 2 of *Mus musculus* contain partial remains of the *COA1* gene and a functional *BLVRA* gene, respectively. A solid sky-blue colored line depicts the partial exon-1 and intron 2 of *COA1* located between the *PTPRF* and *HYI* genes. The pre-chromosomal rearrangement is represented by the *COA1* located at chromosome 8 of *Sciurus vulgaris*. The gene order conservation in each of the rodent species is shown in Supplementary Fig. S179. The time-calibrated phylogenetic tree was obtained from the TimeTree website and visualized in FigTree.

Repetitive elements and lack of long-read sequencing data in most rodent species prevent genome assembly verification. Hence, we have screened newly available unannotated genomes of several closely related rodent species. These genome assemblies were verified using long-read sequencing data or cloned fragments that cover parts of the genome when available (see Supplementary Text S4A, Supplementary Table S13, and Supplementary Figs. S79–S178). Gaps in the genome assembly also hamper the identification of the correct gene order. Previous reports that examined genome assemblies and EST data have claimed loss of the *STK17A* gene in mice due to a CR spanning this genomic region corresponding to chr7p13 in the human genome^48^. Detailed examination of gene order flanking the *COA1* locus in several rodent genomes revealed the occurrence of this previously reported CR event (see Fig. 6 and Supplementary Fig. S179). Example gene orders and their genomic locations for pre-CR (European red squirrel) and post-CR (mouse) species illustrate the movement of genes to four different chromosomes (see Fig. 6). Rather than a simple rearrangement, this appears to be a scattering of short genomic regions to other parts of the genome. We refer to this complex event that involved the movement of genes to more than two new chromosomes as a "gene-scattering" rearrangement.

Identifying gene loss events coinciding with Evolutionary Breakpoint Regions (EBRs) is notoriously challenging and has motivated nuanced inferences in bird^49^ and rodent species^50^ (see Supplementary Text S4B, Supplementary Tables S14–S17, and Supplementary Figs. S180–S228). Nonetheless, more than a dozen rodent species share the putative combined loss of *STK17A* and *COA1* (see Fig. 6). Based on the presence of adjacent genes, the rearranged regions could be tracked down to four different chromosomes (see Fig. 6 and Supplementary Figs. S179, O6, and O9). Genes on the left flank of *STK17A*-*COA1*-*BLVRA* consist of *PSMA2*, *MRPL32,* and *HECW1* in gene orders O1 to O5 (see Supplementary Fig. S179). After the CR, the same sequence of genes can be found in gene order O9 and occur adjacent to *ARID4B* and *GGPS1*. Genes on the right flank of *STK17A*-*COA1*-*BLVRA* consist of *MRPS24*, *URGCP,* and *UBE2D4* in gene order O4. Several other gene orders (O1–O5) occur on the right flank in various species. The sequence of genes found on the right flank in gene order O4 is also found sequentially in gene order O6 and occurs adjacent to *ANKRD36* and *CCDC117* after the CR. We found that the *BLVRA* gene has translocated to an entirely new location and does not co-occur with either the left or right flank. However, the new location of the *BLVRA* gene between the *NCAPH* and *ITPRIPL1* genes on the left flank and *AP4E1* and *SPPL2A* genes on the right flank is consistently conserved across all 14 post-CR species and corresponds to gene order O7. The remnants of *COA1* occur in a gene desert region between *PTPRF* and *HYI* genes in most post-CR species (see Supplementary Fig. S179, Supplementary Text S4C and Supplementary File S7).

**Figure 7:**
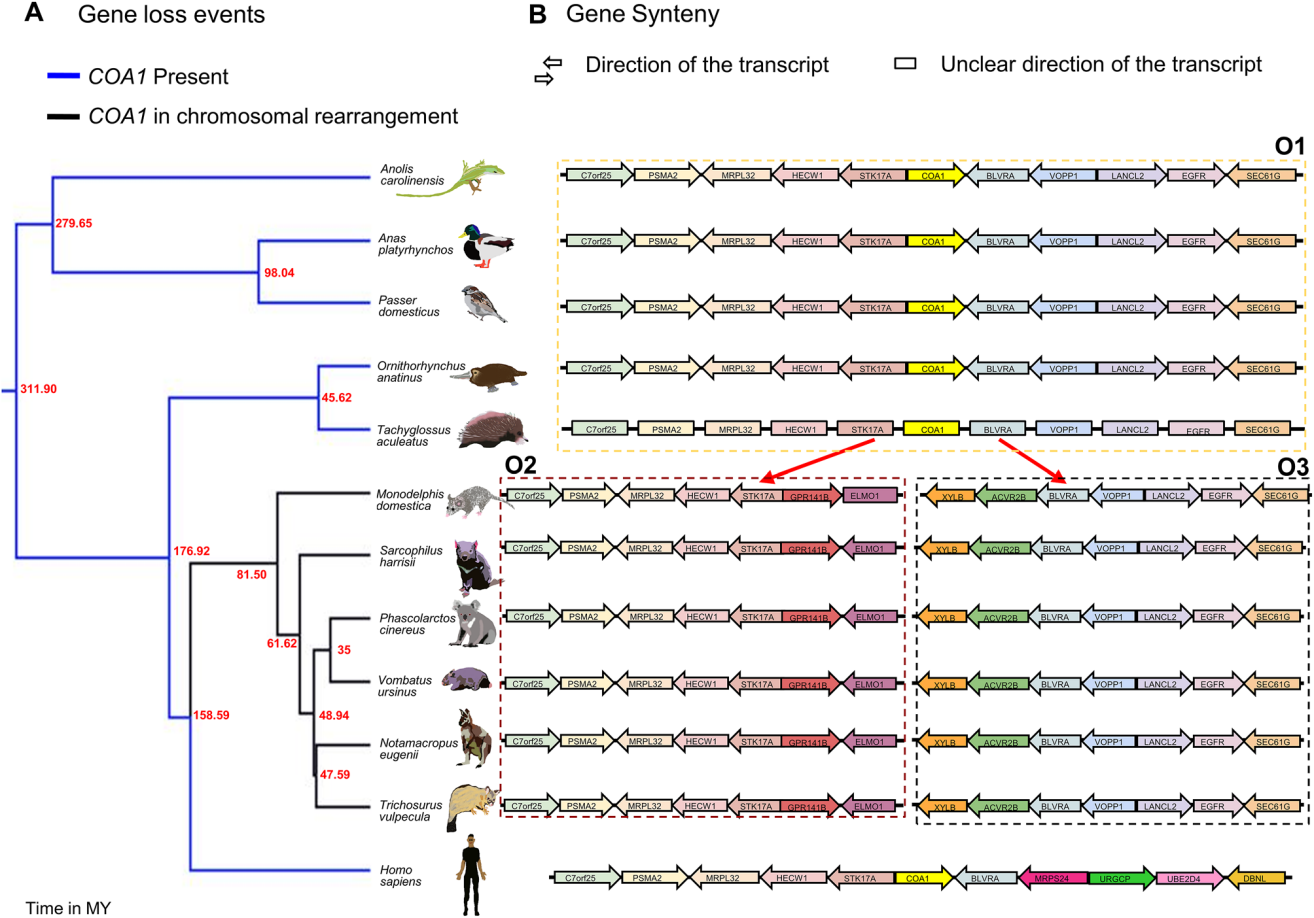
The genomic region spanning the *COA1/MITRAC15* gene coincides with an evolutionary breakpoint (EBR). (**A**) The phylogenetic relationship between marsupial species along with a few outgroup species. The time-calibrated phylogenetic tree is downloaded from the TimeTree website and visualized in FigTree. (**B**) The gene order in the region flanking the *COA1* gene. The arrows show the direction of gene transcription relative to the *COA1* gene for consistency across species. Each dotted box contains one type of gene order, and the red arrows from gene order O1 depict the chromosomal rearrangement (CR) event that leads to gene orders O2 and O3 in the six marsupial species. The outgroup species have the pre-CR gene order O1. In the post-CR species, we see gene orders O2 and O3. A functional *COA1* can be identified in the outgroup species but is presumably lost in marsupial species as it is missing in the genome assembly and raw read datasets.

**Figure 8:**
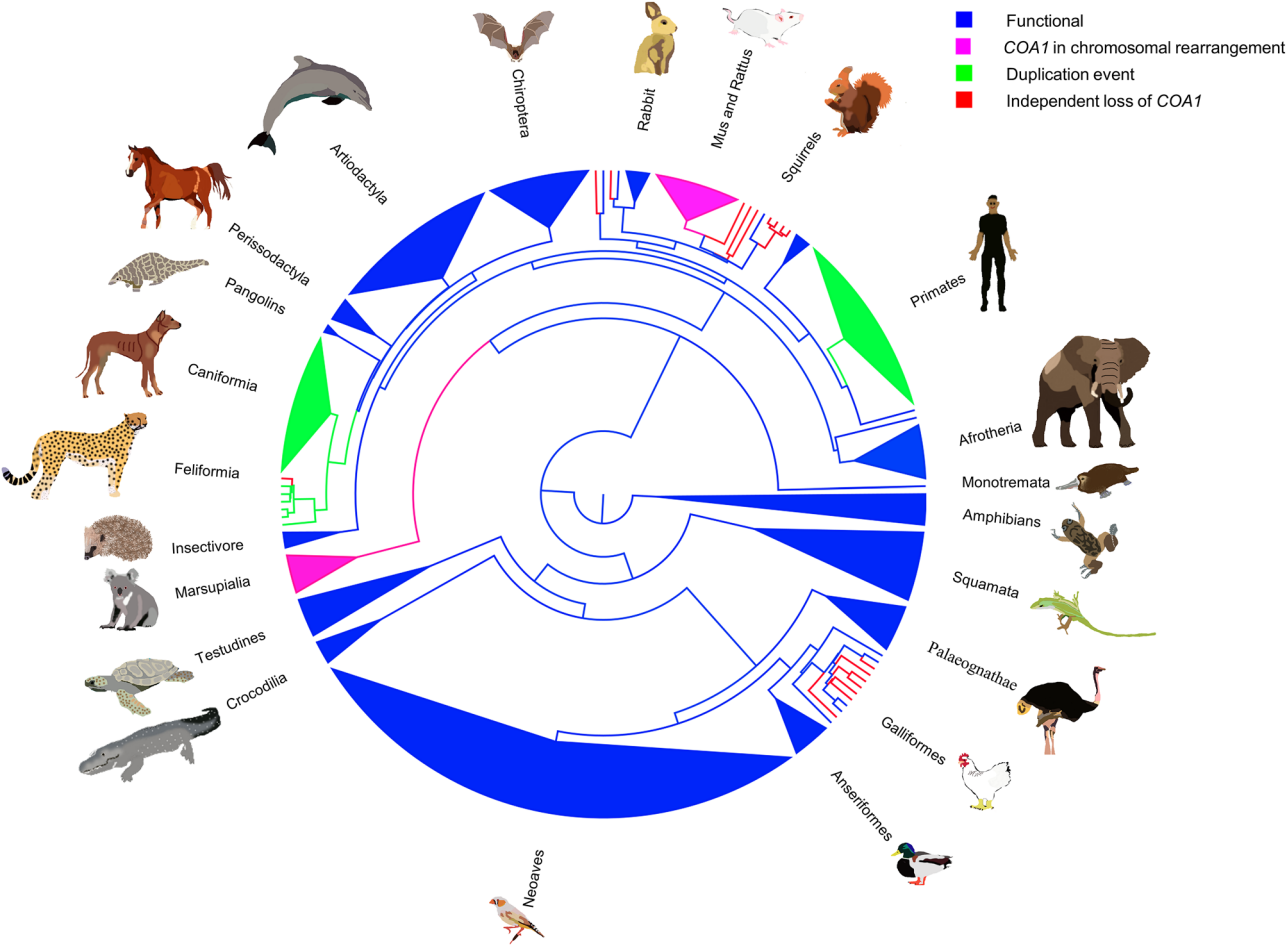
The status of the *COA1/MITRAC15* gene in various clades is shown in the phylogenetic tree obtained from the TimeTree website and visualized in FigTree. The total number of species used is 276. Different colors represent different types of events. Blue represents the intact *COA1* gene, and pink represents the *COA1* gene loss due to the chromosomal rearrangement (CR) at the evolutionary breakpoint region (EBR), green represents the duplication event of the *COA1* gene, and red represents the independent loss of the *COA1* gene.

Comparison of gene order in marsupial species with various outgroup species (including the platypus and short-beaked echidna from the order Monotremata) identified the presence of an independent CR event spanning the *COA1* locus (see Fig. 7). In contrast to the rodent-specific EBR, we found that the *STK17A* gene is intact in post-CR (gene order O2 and O3 in Fig. 7) marsupial species. However, an extensive search of marsupial genomes, transcriptomes, and sequencing datasets (including high coverage Pacbio datasets for the Koala) failed to find any evidence of *COA1* orthologs or their remnants. Lack of sequencing reads from *COA1* in marsupial species suggests either complete erosion of the gene or drastic change in sequence composition that eludes homology detection tools or sequencing with currently available technologies. We found the *COA1* gene is intact in most species, is independently lost in multiple lineages, has undergone duplication and pseudogenisation in different clades, and is potentially lost due to CRs (see Fig. 8).

## Discussion

Our search of the sequence databases identified that *COA1* and *TIMM21* are distant homologs with representative genes found in animals, plants, fungi, and protists. Earlier studies have noted that *COA1* resembles *TIMM21*, a subunit of the TIM23 complex^51^. Hence, it is plausible that *COA1* and *TIMM21* are the results of ancient gene duplication. *TIMM21* gene duplicates that interact with the mitochondrial import apparatus and respiratory chain complexes occur in Arabidopsis^52^. The *COA1* gene has undergone duplication events in carnivores, primates, and a few rodent species. The prevalence of such duplication events suggests that a higher *COA1* protein dosage is not harmful or that sophisticated regulatory machinery exists to maintain the correct dosage. Genes with duplicated copies have greater flexibility for subfunctionalization or neofunctionalization^53^. The ancient origin of *COA1* and *TIMM21* has resulted in considerable sequence-level divergence and changes in the protein structure, suggesting that they may not be able to compensate for each other completely.

In contrast to gene duplication, the origin of new splice-isoforms increases the transcriptome complexity without increasing the gene count. The evolution of phenotypic novelty through alternative splicing has received greater attention thanks to the availability of large-scale transcriptomic and proteomic datasets in diverse species^54^. While positive selection has a role in specific examples of alternative splicing^55,56^, the vast majority of splicing is probably noisy, and neutral processes may explain its evolution^57^. Alternative splicing also reduces premature protein truncation due to purifying selection^58^. In the case of felid species, the alternative splicing of the third exon (see Fig. 2) may have evolved in response to the gene-disrupting changes. Verifying the relevance of the alternative splicing observed at the transcriptional level would require further scrutiny of the protein level isoforms of the *COA1* gene in felid and canid species. In primate species, the potential addition of the extra coding-exon occurs by a shift of the start codon into the untranslated region. Such changes at the reading frame termini occur when the gene is under relaxed selective constraints^59^. Acquisition of novel protein-coding sequences through changes in the exon length is also known to occur^60^. We speculate that drastic lineage-specific changes in purifying selection have allowed for changes in intron–exon structure resulting in the evolution of new splice-isoforms of *COA1*.

The fast glycolytic (FG) fibers use carbohydrates, while fast oxidative glycolytic fibers (FOG) use lipids. Flight degenerate birds have a greater proportion of FG fibers, while the flying birds have mostly FOG fibers. Overall, flight degenerate birds such as galliform species utilize carbohydrates as substrates compared to lipids by flying species^61^. This switch of the primary energy source from lipids to carbohydrates has been linked to convergent amino acid changes in the *ATGL* and *ACOT7* genes of flight-degenerate birds^14^. This difference in the substrate used also leads to differing levels of reactive oxygen species (ROS).

In the OXPHOS pathway, the electrons from the electron transport chain end up in an oxygen molecule which is usually reduced to produce water at complex IV. However, a small fraction (0.1–2%) of the electrons leak at complexes I and III resulting in partial reduction of oxygen to form superoxide (O2^.−^)^62^, a precursor of most other ROS^63^. The FADH_2_/NADH or F/N ratio (ratio of electrons entering the respiratory chain via FADH_2_ and NADH) correlates with ROS production^64,65^. The breakdown of glucose results in a lower F/N ratio compared to the breakdown of fatty acids^64,66^. Both the F/N ratio and radical formation increase with an increase in the fatty acid length^65,66^. The generation of internal ROS in the eukaryotic cell has given rise to numerous adaptations to deal with damage caused by ROS^67^. Thus, lipid utilizing FOG muscle fibers have a higher ROS level than carbohydrate using FG fibers. The lower ROS levels in galliform species that predominantly have FG fibers would result in relaxed selective constraints on the machinery involved in coping with ROS. Hence, *COA1* may have a role in ROS detoxification.

Radical formation due to catabolism of substrates with a high F/N ratio is countered by adaptations such as peroxisomes, uncoupling proteins, mitophagy, carnitine shuttles, complex adjustments, and supercomplex formation^64,66,68^. One intriguing possibility is that changes in substrates lead to varying selection pressure on these processes. For instance, uncoupling protein 1 (*UCP1*) or thermogenin expressed in the inner mitochondrial membrane facilitates the regulated leakage of protons to generate heat in brown adipose tissue^69^. The *UCP1* gene is absent in all birds^70^ and some mammals^71,72^ despite its presence in fishes^73^, amphibians^74^, and marsupials^75^. The integration of *UCP1* in the thermogenic pathway is considered a eutherian-mammal-specific adaptation unrelated to its ancestral innate immune functions^76^. Repeated loss of *UCP1* in vertebrate lineages appears to result from changing functional roles^71,77^. Genes involved in other processes countering radical formation could also have experienced changing selection regimes. The higher-order organization of the mitochondrial electron transport chain forms supercomplexes (SCs)^78^. The ratio of SCs is known to vary between species and tissues^7^ and in response to metabolic demands of the cell^79^. While the SCs are highly stable in reptiles, they are unstable in birds and mammals^80^. We surmise that the loss of *COA1* in various bird and mammal species might reflect a change in the ratio of SCs.

Our computational analysis of more than 300 vertebrate genomes has found that the *COA1* gene is intact and transcribed in most (~ 90%) species, except for the cheetah, galliform, rodent, and marsupial species. Notably, the detailed investigation of the *COA1* gene in non-galliform bird species that are flightless or have a limited flying ability has found an intact transcribed gene. These non-galliform flightless birds tend to have a greater reliance on the hind limb muscles and have comparatively less white fiber in the skeletal muscle of the pectoralis. Therefore, the *COA1* gene loss appears to be associated with skeletal muscle fiber composition changes towards a higher proportion of white fiber in the pectoralis. Similar to galliform birds, the increase in the proportion of white fiber in the skeletal muscles of rodents compared to primates and felids compared to canids appears to be a result of changing locomotor needs. The prominent role of mitochondria in skeletal muscles is evident from locomotory disorders of the muscle tissue caused by defects in mitochondrial genes^81^. While our data support the loss of the *COA1* gene following relaxed selection on the OXPHOS pathway, other implications of gene loss also exist (see Supplementary Text S5).

EBRs are genomic regions that have undergone one or more structural changes resulting in altered karyotypes between lineages^82^. Recurrent non-random structural changes at the same genomic regions in multiple lineages potentially occur due to repeat elements^83,84^, chromosome fragile sites^85–87^, nucleotide composition, methylation level^88^, and chromatin state^89,90^. However, the prevalence of EBRs and their relevance to evolutionary processes has been the focus of considerable debate^91–93^. Several lineage-specific gene loss events near EBRs in rodents are due to CRs^48,94^. Notably, one of these lost genes, *STK17A,* is adjacent to the *COA1* gene. This genomic region containing *STK17A* and *COA1* genes has been implicated in a large-scale GWAS for longevity^95^. The co-occurrence of a rearrangement with putative *COA1* gene loss in rodents and marsupials is very intriguing. However, rodent genomes have mutational hotspots with high lineage-specific GC-biased gene conversion (gBGC), resulting in a substantial gene sequence divergence^50^. Such highly diverged orthologs can be challenging to identify due to difficulties in sequencing high GC regions. The overall magnitude of gBGC is relatively low in *COA1*, especially in rodents. Moreover, we find remnants of *COA1* in several post-CR rodent species that suggest actual gene loss. Several pre-CR rodent species have also independently accumulated gene disrupting mutations in the *COA1* gene. Hence, the *COA1* gene appears to be under relaxed selective constraint even before the rearrangement.

## Conclusions

*COA1* is a distant homolog of the *TIMM21* gene that has undergone recurrent gene loss in several galliform species (16.7–46.51 MYA) and rodent species (4.53–32.40 MYA). The gene loss occurs in species that rely primarily on glycolytic muscle fibers to achieve short bursts of activity. We show that *COA1* and the adjacent *STK17A* gene are located at an EBR and are missing from the genomes of several rodent species following CR. The *COA1* gene has undergone duplication in carnivores and primates, followed by pseudogenization of one copy and divergence of the other copy in its intron–exon structure. The prevalence of repeated gene loss and duplication events in the history of *COA1* demonstrates the occasional context-dependent dispensability of this gene.

## Materials and methods

### Finding homologs of *COA1*

The amino acid sequence of the human *COA1* gene was used as a query in PSI-BLAST^96^ against the non-redundant protein sequence database with eight iterations to identify homologs. Similarly, the human *COA1* amino acid sequence was used as the query in the program HHblits of HHsuite^97,98^ with the flags "-e 1e-3 -n 8 -p 20 -Z 5000 -z 1 -b 1 -B 5000 -d UniRef30_2020_06". The output from HHblits was used as an input to the CLANS program^99^ with an e-value cut-off of 1e−4 to cluster the blast hits using the MPI Bioinformatics Toolkit^100,101^. We ran the CLANS java application for more than 50,000 rounds on the webserver output to ensure stable clusters. Manual inspection of gene annotations allowed identification of each of the groups. Subsequently, we performed the HHblits search again with different settings such as "-glob" for global alignments and "-loc" for local alignments. Manually curated multiple sequence alignment of *COA1* open reading frames from 24 primate species was also separately used as a query for better sensitivity. The amino acid sequence of *TIMM21* provides a consistent hit with different search settings and databases.

### Validation of *COA1* annotation

Despite being a fast-evolving gene, orthologs of *COA1* can be identified based on gene synteny and sequence identity. However, identifying *COA1* orthologs between distantly related species is challenging^102^. We screened the genome assemblies and annotations available on NCBI and Ensembl for *COA1* (C7orf44 or *MITRAC15*) protein-coding transcripts. The *COA1* gene orthologs have been annotated in almost 300 vertebrate species (see Supplementary Table S18). However, the number of exons and the open reading frame (ORF) length vary between species. We validated the annotation of the *COA1* gene relying upon gene synteny in the genomic vicinity of the *COA1* gene, multiple sequence alignments, and RNA-seq data (see Supplementary Text S6, Supplementary Figs. S229–S462, and Supplementary Tables S6, S13, S18–S22).

### Verification of *COA1* gene disrupting changes in raw read data

We have extended a previously published 5-pass strategy^103^ to verify gene loss events by including Hi-C datasets for assembly validation. Briefly, to verify the correctness of the genome assembly nucleotide sequence, we used the *COA1* gene sequence of multiple species as a query for a blastn search of the short-read sequence database (Supplementary Table S23). Manual inspection of the blast search results ensured concordance between gene sequence and short-read data (Supplementary File S8). We also verified the correctness of genome assembly in the vicinity of the *COA1* gene by evaluating Pacbio long-read data when available (see Supplementary Figs. S463–S465). We used Hi-C datasets for assembly validation in chicken, naked-mole rat, mouse, human, macaque, rabbit, dog, and cheetah (see Supplementary Figs. S105–S178, Supplementary Text S6, Supplementary Tables S6, S13, S18–S22, and Supplementary Figs. S229–S462).

### Assessing the transcriptional status of *COA1*

We analyzed transcriptomic datasets for evidence of transcription of *COA1* locus. The RNA-seq reads were mapped to the genome assemblies using the STAR read mapper^104^. We visualized the resulting bam files using the Integrative Genomics Viewer (IGV) browser^105,106^. For consistent representation across tissues and species, we used three different views: (1) Positions of all four exons of *COA1* identified using blast search are shown as a bed record below the RNA-seq bam files, (2) Zoomed-in views of each of the four exons are presented in four panels within a single screenshot and (3) Zoomed-in view of the first and last exons of *COA1* are shown along with the adjacent genes on both sides. The adjacent genes in the IGV screenshot act as positive controls (Supplementary Text S7, Supplementary Figs. S18, S24–S43, S48, S55–S57, S466–S954, and Supplementary Tables S18, S24).

### Molecular evolutionary analyses

We performed molecular evolutionary analyses to identify signatures of relaxed selection using codeml from the PAML package and program RELAX from the HyPhy package. We also used the codeml program to estimate the time of gene loss using the method from Meredith et al.^107^. We evaluated the role of GC content range and K-mer abundance in determining the gene coverage in sequencing datasets and quantified the magnitude of gBGC. We used computational prediction methods to find changes in RNA binding sites (Supplementary Text S8, Supplementary Figs. S189–S222, S955–S957, and Supplementary Tables S1–S5, S7–S9, S12, S14–S17, S25–S26).

### Comparative phylogenetic logistic regression

The pectoralis muscle of birds helps in the movement of humerus bone around the shoulder and provides the power required for the wing's downstroke during flight. This muscle comprises around 8–11% of total body mass in adult flying birds^108^ and is regarded as a quintessential locomotory muscle in flying birds. Fortunately, data on the muscle fiber composition of 43 bird species are compiled in a single study^109^. Quantitative data is available for only 28 of these 43 species. Hence, we performed an additional search of the literature to identify other papers that measure muscle fiber composition using a comparable methodology and report the information in a similar quantitative format. Based on this search of literature^23,109–113^, we have compiled a dataset (Supplementary Tables S10 and S27) of the area percentage of red, pink, and white muscle fiber in the avian pectoralis muscle (see Supplementary Text S9). We were able to retrieve muscle fiber composition for major pectoralis muscle for a total of 24 bird species whose *COA1* gene status is also available (see Supplementary Table S27). We considered the *COA1* gene's status as a binary variable, with 0 implying loss of the functional copy of the gene while 1 representing retention of the gene. To test whether retention/loss of the *COA1* gene is dependent on muscle fiber type, we did a phylogenetically corrected logistic regression analysis on birds in R version 4.1.0^114^ using the phylolm package version 2.6.2^115,116^.

## Supplementary Information

Supplementary Information 1.

Supplementary Information 2.

Supplementary Information 3.

## Data Availability

All data associated with this study are available in the Supplementary Materials. The data is also available in an easy-to-view format on the Github repository: https://github.com/ceglabsagarshinde/COA1_GENE.
