# Supplementary material for: Recurrent erosion of COA1/MITRAC15 exemplifies conditional gene dispensability in oxidative phosphorylation
Source: Sci Rep. 2021 Dec 24;11:24437. doi: 10.1038/s41598-021-04077-y (PMC8709867; doi:10.1038/s41598-021-04077-y)
Supplement: Supplementary file 1 — Supplementary Information 1. [file 41598_2021_4077_MOESM1_ESM.pdf]

Supplementary Information

Recurrent erosion of *COA1/MITRAC15* exemplifies conditional gene dispensability in oxidative phosphorylation

Sagar Sharad Shinde, Sandhya Sharma, Lokdeep Teekas, Ashutosh Sharma, Nagarjun Vijay

Computational Evolutionary Genomics Lab, Department of Biological Sciences, IISER Bhopal, Bhauri, Madhya Pradesh, India

*Correspondence: nagarjun@iiserb.ac.in

S1 text: *COA1* is a distant homolog of *TIMM21*

We have tried to identify homologs of *COA1* and traced the history of this gene family. The rationale for determining the homologs of *COA1* is to evaluate the presence of potential paralogs that could compensate for the loss of the *COA1* gene. Our analysis finds that the closest homolog for *COA1* is *TIMM21*. Subsequently, we estimated when the *TIMM21* and *COA1* genes emerged through a duplication event. Demonstrating that both these genes are well conserved illustrates that the loss of *COA1* in various vertebrate species is striking and potentially functionally relevant. Distinct one-to-one orthologs of *COA1* and *TIMM21* can be identified based on sequence identity and gene order in mammals, birds, amphibians, lobe-finned fishes, and jawless fishes, which share the second round of whole-genome duplication (2R-WGD) and lack the third round of whole-genome-duplication (3R-WGD). Two copies of the *TIMM21* gene occur in several species of ray-finned fishes, which have undergone 3R-WGD. Pre-2R-WGD invertebrate species such as the fruit fly and oriental fly have homologs of *COA1* and *TIMM21*.

The presence of distinct copies of *COA1* and *TIMM21* in pre-2R-WGD species suggests that both genes existed in the LVCA (Last Vertebrate Common Ancestor) (**Supplementary Fig. S1**). Homologs of both *COA1* and *TIMM21* are present in *Schizosaccharomyces pombe* and *Saccharomyces cerevisiae*. Based on the presence of these homologs in fungi and animals, we can track the presence of these genes to the LOCA (Last Opisthokont Common Ancestor). A further search of *COA1* and *TIMM21* homologs revealed their presence in Amoebozoa (*Dictyostelium discoideum*), Chlorophyta (*Chlamydomonas reinhardtii*), Plantae (*Arabidopsis thaliana*), and Protista (**Supplementary Fig. S2**). The widespread presence of these homologs suggests both copies were present in the Last Eukaryotic Common Ancestor (LECA).

Identification of one-to-one orthologs relies on the conserved synteny of the region containing the focal gene. However, synteny is not conserved over very long (> 500 million years) timescales[1], and high confidence identification of one-to-one orthologs is not always feasible. In the case of such distantly related genes, the Pfam 34.0 database[2] of protein families provides a valuable resource. We found that *TIMM21* (PF08294) and *COA1* (PF08695) families are members of the MIM-OM_import (CL0455) clan, which is characterized as "mitochondrial membrane proteins with importing activities.". We used all proteins that form part of these protein families as input to clans to identify the clustering patterns. When both *TIMM21* (PF08294) and *COA1* (PF08695) families are concatenated and used as input for CLANS, we recover the clustering pattern (**Fig. 1A** and **Supplementary Fig. S3**) seen while running CLANS on the HHblits hits of the human *COA1* protein (**Supplementary Fig. S4**). The clustering pattern of *TIMM21* (PF08294) and *COA1* (PF08695) families suggests that all *TIMM21* proteins are much more similar to each other than *COA1* proteins. However, identical to *COA1,* we can see separate clusters for fungal, plant, and animal sequences within *TIMM21* proteins (**Fig. 1A** inset and **Supplementary Fig. S5**). Such independent clustering of *TIMM21* and *COA1* homologs suggests that lineage-specific duplications after the LECA are unlikely.

The orthology-based analysis and sequence clustering suggest that both *TIMM21* and *COA1* existed in the LECA. While we cannot conclusively exclude post-LECA lineage-specific duplications, the data does not seem to support this scenario. Earlier reports support the presence of *TIMM21* in the LECA[3,4]. However, the *COA1* gene and its origin are understudied. We can envisage the following simple scenarios that can result in the LECA containing both *TIMM21* and *COA1* homologs:

1. *TIMM21* from archaea + *COA1* from bacteria→FECA→LECA
2. *COA1* from archaea + *TIMM21* from bacteria→FECA→LECA
3. *COA1* from archaea →FECA—(Duplication event)→LECA
4. *COA1* from bacteria →FECA—(Duplication event)→LECA
5. *TIMM21* from archaea →FECA—(Duplication event)→LECA
6. *TIMM21* from bacteria →FECA—(Duplication event)→LECA

Many bacterial sequences belong to the *COA1* protein family in contrast to *TIMM21*, which has a minimal number of bacterial homologs. Hence, the data supports scenarios 1 and 4, which suggest that *COA1* has a bacterial origin. Scenarios 5 and 6, which indicate duplication of *TIMM21* within the FECA, will find it challenging to explain the large number of bacterial homologs of *COA1*. Scenarios 2 and 3 suggest that *COA1* has an archaeal origin and has minimal support as very few *COA1* sequences occur in archaea.

We used the human *COA1* sequence to perform an iterative profile-profile search of the uniport (UniRef30_2020_06) database using HHblits. The list of proteins identified as homologs of human *COA1* (**Supplementary File S1-S2**) and primate *COA1* orthologs (**Supplementary File S3**) contain several *TIMM21* like proteins. Iterative PSI-BLAST search identified *TIMM21* homologs from the second iteration onwards and found an increasing number of *TIMM21* hits in each subsequent iteration (**Supplementary File S4**). Out of the 500 top search results from HHblits, 59 have annotation as "Cytochrome C oxidase assembly factor" or "Cytochrome C oxidase assembly protein" or "*COA1*", and 120 as "*TIMM21*" homologs. The annotation of 13 proteins is "hypothetical", nine are "membrane" proteins, eight are "DUF1783 domain-containing" proteins, and 27 proteins are from diverse proteins. The remaining 264 of the 500 hits are "Uncharacterized". The large number of "Uncharacterized" proteins identified are challenging to interpret. Hence, to trace the relationships between the proteins identified as homologs of *COA1*, we investigated the sequence identity-based clusters established by CLANS (**Supplementary Fig. S6**). The large group of red dots consists of proteins annotated as *TIMM21,* and the collection of blue dots contains proteins annotated as *COA1*. Homologs of *COA1* from bacterial species form two clusters, a distinct light blue cluster consisting of predominantly Planctomycetes bacteria and a diffuse bunch of brown dots that consists of largely proteobacterial species. The group of orange dots consists of proteins annotated as *COA1* in fungal genomes. The *COA1* homologs in plants consist of a yellow cluster consisting of *Arabidopsis thaliana* homolog At2g20390 and the magenta cluster of *TIMM21*-like proteins containing *Arabidopsis thaliana* homolog At2g40800.

To further verify whether the database matches are homologous, we evaluated the biological function, secondary structure similarity, relationship among top hits, and occurrence of conserved motifs. To obtain secondary structure predictions for the proteins *COA1* and *TIMM21*, we used the PROTEUS (version 2.0) webserver[5]. The HeliQuest webserver[6] provided each predicted helix's physicochemical properties and amino acid compositions (**Supplementary Fig. S7-S8**). While the three-dimensional (3-D) structure of the *COA1* protein is not available yet, multiple structures of the *TIMM21* protein are available in the Protein Data Bank (PDB). It is possible to use comparative/homology modeling to predict the 3-D structure based on the protein structure of a related protein[7]. Hence, we used the comparative modeling approach implemented in Modeller (v10.0) software to model the structure of *COA1* based on the homologous structures available in PDB. The Phyre2[8] and Expasy Swiss-Model[9] webservers also predicted homologous 3-D structures of *COA1* (**Supplementary File S5**). All the top hits were from 3-D structures of the IMS (Inter Membrane Space) domain of *TIMM21* protein. The IMS domain of *TIMM21*, whose 3-D structures are available on PDB, contains only the part of the protein that occurs after the membrane-spanning helix. To model the structure of *COA1* using these existing 3-D structures, we used the *COA1* amino acid sequence that occurs after the membrane-spanning domain. We visualized (**Supplementary Fig. S9**) the structure of *TIMM21* and the predicted *COA1* structure using (UCSF Chimera v1.15) ChimeraX[10].

S2A text: *COA1* gene duplication, pseudogenisation, and exon reorganization

In species with functional and pseudogene copies of *COA1*, these copies have diverged considerably and formed distinct haplotypes. For example, the blastn search of sequencing short-read data from the human genome with a *COA1* gene sequence as a query results in two distinct haplotypes. One set of reads correspond to the intact *COA1* gene in humans, and the other set of reads are from the pseudogenic copy (**Supplementary Fig. S17**). Comparative analysis of Primate genome assemblies suggests that the pseudogenic copy results from a duplication of *COA1* within the primate lineage (**Supplementary Fig. S18**).

Independent duplication of *COA1* has occurred in carnivores (**Supplementary Fig. S19**). However, the duplicated copy has undergone pseudogenization and diverged from the functional gene sequence similar to Primates. For example, sequencing raw read data in the tiger consist of two distinct haplotypes corresponding to the intact and pseudogene copies (**Supplementary Fig. S20**). While the intact copy is located at a genomic region (*STK17A* & *HECW1* upstream and *BLVRA* & *VOPP1* downstream) with conserved synteny across other mammals, the pseudogene copy occurs adjacent to the *PRR32* gene. Outgroup species such as horse (*Equus caballus*) and pangolin (*Manis javanica*) have a single copy of the *COA1* gene with all raw reads supporting a single haplotype (**Supplementary Fig. S21-S22**). Both sub-orders (Caniformia and Feliformia) within Carnivora share this duplication of the *COA1* gene (**Supplementary Fig. S19**). The intact *COA1* copy is expressed in diverse transcriptomes among Caniformia species, while the pseudogene copy lacks expression. The first and second exons are orthologous; however, the genomic location of the transcribed third exon is different between Feliformia (cat-like-exon-3) and Caniformia species (dog-like-exon-3) (**Fig. 2** and **Supplementary Fig. S23**).

The final exon of the *COA1* gene in Feliformia extends to 163 base pairs (*Panthera tigris altaica, Panthera leo, Panthera pardus,* and *Lynx lynx*) and 160 base pairs (*Puma concolor* and *Felis catus*) compared to the 100 base pairs in Caniformia species. A single deletion event causes the difference of three base pairs between these two groups of Feliformia at the 24th base of exon-4 (**Supplementary Table S1**). The extended final exon shared by all Feliformia species results from a two-base frameshift deletion before the erstwhile stop codon in exon-4. Despite the extended last exon in Feliformia species, the full-length open reading frames of Feliformia (130/131 amino acids) and Caniformia (135 amino acids) are comparable.

The shorter reading frame in Feliformia results from most *COA1* transcripts skipping the dog-like-exon-3, whose inclusion results in premature stop codons in all the seven Feliformia species. The dog-like-exon-3 is present in all *COA1* transcripts of Caniformia species and does not contain gene-disrupting changes. A single base deletion in all Feliformia species changes the end phase of exon-2 to maintain an intact reading frame while skipping the dog-like-exon-3. Transcriptomes of the cat (*Felis catus*) from the spleen (**Supplementary Fig. S24-S34**) and puma (*Puma concolor*) from blood (**Supplementary Fig. S35-S42**) exhibit expression of a proto cat-like-exon-3 which gets spliced into some of the *COA1* transcripts. However, the majority of transcripts skip this proto cat-like-exon-3 which contains premature stop codons. These changes in exon splicing patterns between Caniformia and Feliformia species appear to result from changes in splice factor binding sites at the *COA1* locus (**Supplementary Fig. S43**).

In contrast to primates and carnivores, reads support multiple haplotypes of *COA1* only in the second exon of naked mole-rat (**Supplementary Fig. S44**). Hence, the duplicated copy of *COA1* in naked mole-rat appears to have mostly degraded. However, we cannot rule out the possibility that the reads from other haplotypes spanning the remaining three exons are missing due to high GC content. The sequencing reads support a single intact open reading frame in the red squirrel (**Supplementary Fig. S45**) and platypus (**Supplementary Fig. S46**). Although a single haplotype occurs in the raw read dataset of chicken, this haplotype has gene-disrupting changes (**Supplementary Fig. S47**). The gene-disrupting modifications identified in the chicken *COA1* gene were investigated further by screening long-read datasets, transcriptomes, and genomes of various galliform species.

After duplication of the *COA1* gene in Primates, an extension of the N-terminal region has occurred in Cercopithecidae and Catarrhini and is transcriptionally active (**Supplementary Fig. S48**). However, new world monkeys do not have this N-terminal extension denoted as exon-1a. Both Cercopithecidae and Catarrhini have an additional start codon in exon-1a upstream from the original start codon in the ancestral exon-1 denoted as exon-1b in species with N-terminal extension (**Supplementary Table S2)**. A striking difference between Cercopithecidae and Catarrhini is the lack of the internal start codon in Cercopithecidae, where Catarrhini has a start codon. Since proteome level data is not available for these species, we rely solely on the RNA-seq datasets and start and stop codons within the expressed transcripts to evaluate the exon/intron structure changes. Using these carefully

annotated Primate sequences of *COA1*, we verified a previous report[11] of positive selection in this gene among Primates (**Supplementary Table S3**).

S2B text: *COA1* gene duplication, pseudogenisation, and exon reorganization

RNA-seq data is available only from the skin tissue and one unknown tissue sample in the cheetah. Unfortunately, these RNA-seq datasets are of poor quality, evidenced by the lack of gene expression information for most genes (**Supplementary Fig. S55-S60)**. Nonetheless, to evaluate if the skin is a relevant tissue to screen for the expression level of *COA1*, we analyzed skin transcriptome datasets from closely related species. The *COA1* gene is robustly expressed in the skin tissue of cats and dogs (**Supplementary Fig. S61-S65 and S66-S67**). The presence or absence of evidence of transcription or translation is not conclusive evidence of gene loss. It is known that several pseudogenes are not only transcribed but are also translated. However, they may not form functional proteins as the full-length protein is not produced due to premature stop codons[12]. Hence, the presence of premature stop codons that disrupt the functional domains of the protein or truncate a significant fraction of the protein is considered more reliable evidence of gene loss.

Two versions of the cheetah genome (Aci_jub_2/ GCA_003709585.1 and aciJub1/ GCA_001443585.1) are currently available in the NCBI (National Center for Biotechnology Information) database. A third version of the cheetah genome generated by scaffolding the second version using Hi-C data is also available (aciJub1_HiC.fasta). While these multiple versions of the cheetah genome assembly have improved the contiguity (as seen by the increased N50), the actual base pair level sequence correctness cannot be assessed directly from the genome assembly. Some of the carnivore genome assemblies are known to have base-pair level errors that can affect the results of evolutionary analysis[13]. Hence, we rely upon the sequencing raw read datasets to ensure the validity of gene loss.

The *COA1* gene has undergone a duplication followed by pseudogenisation of one copy in the ancestor of all carnivore species (**Supplementary Fig. S19**). We used the *COA1* sequence of the puma (*Puma concolor*), which has an intact ORF, as a query to search for the *COA1* gene in cheetah. Based on the results of the blastn search of the sequencing raw read datasets, we found evidence of at least two haplotypes of *COA1* in each of the three cheetah individuals screened. Various scenarios that can result in an open reading frame among these haplotypes were evaluated to verify gene loss in the cheetah (**Supplementary Fig. S68-S70**). In the first haplotype, located in the syntenic region of *COA1*, one base change occurs in exon two position 27 (C → T), leading to a premature stop codon (TAG). The second haplotype (i.e., the duplicated copy of *COA1*) is located between PRR32 and DCAF12L2 (DDB1- and CUL4-associated factor 12-like protein 2 gene) on the scaffold NW_020836464.1. We found two single base insertions in exon two at positions 11 (T) and 89 (C), leading to an altered reading frame in haplotype two. A single base substitution (C → T) at position 59 in this new reading frame leads to a premature stop codon (TGA). Exon four has an insertion of CTT, TAT, AAACA, and A at positions 22, 82, 84, and 145, respectively, and it has two stop codons at positions 24-26 (TAA) and 84-86 (TAG).

In haplotype two, we found few reads that do not support T insertion at position 11 in exon two. This difference between the haplotype two reads could result from individual polymorphism (**Supplementary Fig. S71-S73**). However, even when the insertion at position 11 is missing in exon two, a premature stop codon occurs in exon four at position 31, where C → T substitution leads to a stop codon (TAA). Despite this, exon four contains two more stop codons at positions 79-81 (TGA) and 84-86 (TGA). For each of the three haplotypes, we tried different possible combinations of exons to evaluate if any combination produced an intact ORF. We found that none of the exon combinations lead to an intact ORF (**Supplementary Fig. S74-S77**). The conserved domain identified in each ORF is shown as a green line below the exons. In most cases, we can see that the truncated ORF covers only a fraction of the conserved domain. Functional evaluation of the short proteins would give a definitive answer about the relevance of these ORF's.

S3 text: *COA1* gene loss in galliform species

We find evidence of *COA1* gene loss in multiple galliform species. The loss of *COA1* could be verified through the presence of gene disrupting mutations, lack of gene expression in multiple tissues, and signatures of relaxed selection detected by molecular evolutionary analyses in galliform species consisting of the red-legged partridge (*Alectoris rufa*), Chinese bamboo partridge (*Bambusicola thoracicus*), common quail (*Coturnix coturnix*), Japanese quail (*Coturnix japonica*), lesser sage-grouse (*Centrocercus minimus*), chicken (*Gallus gallus*), rock ptarmigan (*Lagopus muta*), black grouse (*Lyrurus tetrix*), wild turkey (*Meleagris gallopavo*), helmeted guineafowl (*Numida meleagris*), marbled wood quail (*Odontophorus gujanensis*), Indian peafowl (*Pavo cristatus*), mikado pheasant (*Syrmaticus mikado*) and greater prairie chicken (*Tympanuchus cupido*). In addition to these species, the common pheasant (*Phasianus colchicus*) and golden pheasant (*Chrysolophus pictus*) may have acquired regulatory changes resulting in a lack of gene expression and relaxed selection. Although we see signatures of relaxed selection in wood duck or Carolina duck (*Aix sponsa*), Muscovy duck (*Cairina moschata*), and common crossbill (*Loxia curvirostra*), these species appear to have intact reading frames that are transcribed. However, despite being galliform birds, species such as Australian brushturkey (*Alectura lathami*), northern bobwhite (*Colinus virginianus*), scaled quail (*Callipepla squamata*), and white-crested guan (*Penelope pileata*) have intact gene sequences that are under purifying selection (see **Supplementary Table S11**). Retention of an intact *COA1* gene in these galliform species may result from a more ancestral duck-like muscle fiber distribution with a lower proportion of white fiber.

Several flight-degenerate species from diverse taxa[14] such as rifleman (*Acanthisitta chloris*), speckled mousebird (*Colius striatus*), sunbittern (*Eurypyga helias*), hoatzin (*Opisthocomus hoazin*), and South African ostrich (*Struthio camelus australis*) have retained intact *COA1* sequences. Various tinamou species such as the rufescent tinamou (*Crypturellus cinnamomeus*), little tinamou (*Crypturellus soui*), undulated tinamou (*Crypturellus undulatus*), martineta tinamou (*Eudromia elegans*), white-throated tinamou (*Tinamus guttatus*), tawny-breasted tinamou (*Nothocercus julius*), hooded tinamou (*Nothocercus

nigrocapillus), ornate tinamou (*Nothoprocta ornata*), Andean tinamou (*Nothoprocta pentlandii*) and Chilean tinamou (*Nothoprocta perdicaria*) also have intact *COA1* gene sequences. Other Palaeognathae flightless birds such as the North Island kiwi (*Apteryx australis mantelli*), Okarito kiwi (*Apteryx rowi*), southern cassowary (*Casuarius casuarius*), emu (*Dromaius novaehollandiae*), and greater rhea (*Rhea americana*) also have intact *COA1* gene sequences. The flightless cormorant (*Nannopterum harrisi*), as well as other species of cormorants such as double-crested cormorant (*Phalacrocorax auritus*), Neotropic cormorant (*Phalacrocorax brasilianus*), great cormorant (*Phalacrocorax carbo*), and pelagic cormorant (*Phalacrocorax pelagicus*), also have intact *COA1* gene sequences. The ground-dwelling brown mesite (*Mesitornis unicolor*), kākāpō also called owl parrot (*Strigops habroptila*), emperor penguin (*Aptenodytes forsteri*), Adélie penguin (*Pygoscelis adeliae*), and African penguin (*Spheniscus demersus*) are also considered flightless but retain the *COA1* gene. The greater roadrunner (*Geococcyx californianus*) has a limited flying ability but is capable of very fast running. Similarly, the kagu (*Rhynochetus jubatus*) is almost flightless and spends most of its time on the ground. Both the greater roadrunner and kagu have intact *COA1* sequences.

Overall, we see that the *COA1* gene loss is found in species with a high proportion of white muscle fiber in the pectoralis and is not simply a consequence of degeneration of flight abilities. Increased use of hind limb muscles in flightless birds and a lower fraction of white muscle fiber in the pectoralis muscle seem sufficient to retain an intact *COA1* gene. Although quantitative information is not available, the red-winged blackbird (*Agelaius phoeniceus*), snowy owl (*Bubo scandiacus*), and Canada geese (Branta canadensis) have predominantly red muscle fiber and have intact *COA1* gene sequence. Similarly, the cockatiel (*Nymphicus hollandicus*), dark-eyed juncos (*Junco hyemalis*), and species closely related to the Smithsonian gull (*Larus smithsonianus*) are reported[15] to have only red muscle fiber, and all of these species have intact *COA1* gene sequence. Hence, based on currently available data, we find a significant negative correlation pattern between loss of *COA1* gene and predominance of white muscle fiber.

S4A text: *COA1* occurs in an evolutionary breakpoint region

While verifying gene loss, we need to consider two aspects. First, we need to confirm whether the raw read dataset supports the genome assembly sequence and identified gene disrupting mutations. Second, we need to evaluate whether the gene order is conserved in the region adjacent to the focal gene, allowing for accurate identification of the ortholog. Hence, the first criteria require high coverage data from a sequencing technology with a low error rate (for instance, Illumina sequencing data). The second criteria require evaluation of gene order and the genome assemblies need to be of high contiguity. Additionally, long-range sequence information should be available from either long-read sequencing technologies like PacBio or complementary methods such as Hi-C or synthetic long reads. We describe how we have used both these criteria to verify gene loss events reported by us.

Genome assembly and raw read based verification of *COA1* in rodents

Eurasian red squirrel (*Sciurus vulgaris*):

We used short-read Illumina data to verify genome assembly correctness and conserved synteny for the Eurasian red squirrel (*Sciurus vulgaris*). But in Eurasian red squirrel, publicly available Illumina raw read data (~15 Gb) was not enough to verify the genome assembly. We used long-read PacBio raw read data (~67 Gb) and mapped it with the red squirrel genome (Sciurus_vulgaris.mSciVul1.1). We extracted the reads > 3kb from the mapped read file and loaded it in the UCSC genome browser as a custom track (**Supplementary Figures S79-S83**). We verified that long PacBio reads span from *COA1* to the genes adjacent to *COA1*, which provides confidence about genome assembly robustness and conserved synteny (see **Supplementary Table S13**).

American beaver (*Castor canadensis):*

In the old genome assembly (Castor_canadensis.C.can_genome_v1.0) of American beaver (*Castor canadensis),* the pseudogenized *COA1* is the only gene located on the scaffold MTKA01013026.1 (**Supplementary Figures S84**). The *COA1* adjacent genes are on different scaffolds. Due to the fragmented nature of this assembly, we are unable to confirm synteny in the American beaver. We used a new genome assembly (GCA_009822645.1_ASM982264v1) of the American beaver published by Zhou et al., 2020[16] to verify the gene order. We found that the *C7orf5*, *PSMA2*, *MRPL32*, *HECW1*, and *STK17A* are adjacent to *COA1* on the scaffold RPDE01003036.1. However, other nearby genes *BLVRA* and *MRPS24*, are present on scaffold RPDE01003922.1, while *URGCP* and *UBE2D4* are on scaffold RPDE01003294.1 (**Supplementary Table S13**). We further evaluated the correctness of the genome assembly using long-read PacBio data (**Supplementary Fig. S85-S94**). However, we observed that some regions showed no spanning reads, and gaps in coverage prevent verification of the assembly. Nonetheless, the newer genome assembly helps verify the conserved gene order on the left flank. It is possible that gene order has changed on the right flank and will need to be evaluated in the future using a better quality genome assembly.

Naked mole-rat (*Heterocephalus glaber*):

A total of three genome assemblies are available for naked mole-rat (*Heterocephalus glaber*). Out of the two older assemblies, one is from a male (Heterocephalus_glaber_male.HetGla_1.0), and another is from a female (Heterocephalus_glaber_female HetGla_female_1.0). The latest assembly has been generated by Zhou et al., 2020[16] (GCA_014060925.1_Heter_glaber.v1.7) based on long-read PacBio data. As the male naked mole-rat genome is relatively fragmented, we checked the syntenic region in the female naked mole-rat genome. We found all the genes adjacent to *COA1* (i.e., *C7orf25*, *PSMA2*, *MRPL32*, *HECW1*, *STK17A*, *BLVRA*, *CYP3A9*, *GJC3*, and *AZGP1*) are on a single scaffold, JH602053.1 (see **Supplementary Table S13**). To check the correctness of

the assembly, we used the raw PacBio data of naked mole-rat and mapped it to the female genome. After visualizing the mapped reads in the UCSC genome browser, we found that the reads do not span all regions. There are several coverage gaps in the regions adjacent to *COA1* (**Supplementary Fig. S95**).

We evaluated the synteny in the latest genome assembly generated by Zhou et al., 2020[16] and found that the genes *HECW1*, *STK17A*, *COA1*, *BLVRA*, *CYP3A9*, *GJC3*, and *AZP1* are present in a collinear fashion on the scaffold RPGA01000026.1. The genes *C7orf25*, *PSMA2*, and *MRPL32* are on the scaffold RPGA01005726.1 (**Supplementary Fig. S96-S104**). As an additional step to verify genome assembly correctness, we analyzed the Hi-C (High-throughput Chromosome Conformation Capture) data from naked mole-rat using the genome assembly of a female (Heterocephalus_glaber_female HetGla_female_1.0). We did not find any apparent patterns of genome assembly errors based on the frequency of interactions between genomic regions adjacent to *COA1* (**Supplementary Fig. S105-106**). Hence, all the available data (three genome assemblies, short read Illumina, PacBio, and Hi-C data) are consistent with a conserved gene order.

Our evaluation of Hi-C interaction maps of various species did not find any such obvious patterns of gaps in the *COA1* region of the genome. Even the duplicated *COA1* regions in chicken, carnivores, primates, and rodents did not have a gap in the interaction map (**Supplementary Fig. S107-S178**). These Hi-C maps support the long-range correctness of the genome assembly and help validate the synteny relationships identified. In the case of the mouse, we found that following the CR, the *COA1* and adjacent gene *BLVRA* are translocated to two different chromosomes. The regions flanking *COA1* and *BLVRA* are on two other chromosomes. We found the Hi-C map free of gaps or other patterns indicating assembly errors in all four chromosomes (**Supplementary Fig. S141-S156**).

S4B text: *COA1* occurs in an evolutionary breakpoint region

Search for the *COA1* gene in the mammoth genome demonstrated striking heterogeneity in coverage of the four exons based on the Illumina ancient DNA sequencing datasets analyzed (see **Supplementary Fig. S180A-F**). Despite having comparable genome-wide coverage, we could see that not all exons occur in all the datasets. For instance, the re-sequencing dataset from PRJEB29510 (162 Gb) does not have reads from any of the four *COA1* exons. However, the datasets from PRJEB7929 (88.34 Gb) and PRJNA397140 (155 Gb) have reads covering three exons each despite having much lower genome-wide coverage. The third exon of *COA1* was missing or had fewer reads than the other three exons in most datasets. The dataset from PRJEB42269 had no reads from the first exon but had a few reads from exons three and four. We reasoned that this heterogeneity in the coverage of various *COA1* exons was mainly a result of the well-established sequencing bias of Illumina that results in inadequate coverage of GC-rich regions[17]. Quantification of GC content in each of the four *COA1* exons and K-mer abundance in different GC content bins in each mammoth Illumina

re-sequencing dataset explains most of the coverage heterogeneity between datasets as well as exons (see **Supplementary Fig. S180G**).

In contrast to the *COA1* gene, we did not see heterogeneity in the sequencing coverage of *TIMM21* exons despite comparable GC content for some of the exons (see **Supplementary Fig. S180G** and **Supplementary Fig. 181-187**). The heterogeneity in sequencing coverage of *COA1* exons demonstrates the challenges of detecting its presence in Illumina sequencing datasets. GC-biased gene conversion (gBGC) plays a defining role in the base composition for any particular gene or genomic region. It preferentially fixes GC in AT/GC heterozygotes and increases the GC content. The GC content of the *COA1* exons can be driven to extreme values by gBGC. The magnitude of gBGC also varies across the genome within a species as well as between species. Therefore, *COA1* orthologs from closely related species or even duplicated copies of *COA1* within the same species can have very different GC content. Such differences in GC content can result in correspondingly different coverage of the gene sequence in Illumina data and masquerade as a gene loss event[18,19].

A well-known example for high GC content impeding sequencing is the gene *PDX1*, which has striking differences in GC content between closely related rodent species and requires dedicated GC-rich DNA enrichment protocols for sequencing. We contrasted *COA1* with the *PDX1* genes of rodents by comparing the minimum and maximum (see **Supplementary Table S14**) GC contents possible given their amino acid sequence. Although *COA1* had lower GC content levels than *PDX1*, we could not rule out the possibility of gBGC affecting some exons. The values of GC* (strong and weak convergence) across more than 200 vertebrate species with intact *COA1* reading frames suggested considerable heterogeneity between taxa (see **Supplementary Fig. S188** and **Supplementary Table S15)**. In each taxonomic group, the prevalence of gBGC was separately quantified (see **Supplementary Fig. S189-S222**). Strong patterns of gBGC occur in the *COA1* sequence of several species (see **Supplementary Fig. S189-S222**: elephant (*Loxodonta africana*), kagu (*Rhynochetus jubatus*), blue-crowned manakin (*Lepidothrix coronata*), Chilean tinamou (*Nothoprocta perdicaria*), American black bear (*Ursus americanus*), North American river otter *(Lontra canadensis)*, meerkat (*Suricata suricatta*), California sea lion (*Zalophus californianus*), little brown bat (*Myotis lucifugus*), large flying fox (*Pteropus vampyrus*), southern pig-tailed macaque (*Macaca nemestrina*), Brazilian guinea pig (*Cavia aperea*), sheep (*Ovis aries*), eastern brown snake (*Pseudonaja textilis*) and the Goode's thornscrub tortoise (*Gopherus evgoodei*)). However, no rodent species with intact *COA1* show any striking gBGC patterns (**Supplementary Table S16**). The GC content vs. K-mer abundance plots of PacBio, BGI-seq, and Illumina datasets spans the entire range of GC contents seen in *COA1* exons (see **Supplementary Fig. S223**). Since the GC content of individual *COA1* exons differs between species groups (see **Supplementary Fig. S224-S228** and **Supplementary Table S17**), the high GC content of certain regions might result in inadequate sequencing coverage of the *COA1* gene in some species. Hence, the lack of sequencing reads covering *COA1* cannot serve as definitive evidence of gene loss.

S4C text: *COA1* occurs in an evolutionary breakpoint region

Both *COA1* and *STK17A* are missing in the post-CR rodent genome assemblies. The search of the genome assemblies, sequencing raw read datasets, and RNA-seq datasets also failed to find evidence of an intact *COA1* or *STK17A* gene. All raw read and genome assembly hits for *STK17A* while using queries from pre-CR rodent genomes could be traced back to the *STK17B* gene that matches the *STK17A* gene at a short sequence stretch. The *STK17A* gene is lost or has sequence properties that prevent sequencing with currently available technologies. The exon-1 region of *COA1* occurs in a gene desert region between *PTPRF* and *HYI* genes in post-CR species. Using blast search of *COA1* introns, we found strong support for the existence of *COA1* intron-2 close to the exon-1 hit. Pairwise genome alignments support the presence of the *COA1* gene remains at this location (see **Supplementary File S7**). Notably, the *COA1* remnants of a truncated exon-1 and intron-2 occur in the gene desert between *PTPRF* and *HYI* genes only in post-CR species. None of the pre-CR species had any such remains. Hence, the *COA1* remnants between *PTPRF* and *HYI* genes are unlikely to have resulted from duplicated copies of *COA1*. The synteny of this region is well conserved with *KDM4A* and *PTPRF* on the left flank and *HYI* and *SZT2* on the right side and corresponds to gene order O8. Careful examination of this region in RNA-seq datasets found no evidence of transcripts.

S5 text: Implications of gene loss

Gene loss can be dealt with through compensation from another gene[20] or is associated with a biological pathway rewiring[21]. Large-scale changes in gene content are associated with major evolutionary transitions that drastically alter the fitness landscape. Prominent examples of such shifts are the origin of flight in birds[22] and the movement of mammals from land to water seen in cetaceans[23]. Recurrent gene loss events following relaxed selective constraint in various other lineages have also been documented[24–26]. Differences in the immune response of Galliformes and Anseriformes are linked to lineage-specific gene loss[27,28]. The *COA1* gene is not known to have any obvious immune functions, and its loss in galliform birds appears to be a consequence of relaxed selection on the OXPHOS pathway.

The correlation between recurrent gene loss and specific phenotypes has provided crucial insights into the evolution of traits. Stomach loss in gnathostomes co-occurs with the loss of several genes that code for digestive enzymes[29]. The loss of ketogenesis has occurred through the recurrent loss of the *HMGCS2* gene[30]. Gene losses associated with dietary composition, the patterns of feeding, and gut microbiomes have also been identified[31]. Recurrent loss of Toll-like receptors (TLRs), which play prominent roles in the innate immune system, is associated with impaired ability to detect extracellular flagellin[32]. The repeated loss of the *CORT* (cortistatin) gene is related to modifications in the circadian pathway[24]. In the *COA1* gene, we record the independent occurrence of gene disrupting changes in closely related species of galliforms and rodents. However, we cannot rule out the possibility of a common regulatory mutation that initially resulted in the loss of gene expression followed by the independent accumulation of the gene disrupting changes that we observe. Our hypothesis of relaxed selection on the OXPHOS pathway predicts gene loss following skeletal muscle fiber composition changes. The *COA1* gene does not alter the muscle fiber composition and might have experienced relaxed selective constraint due to increased fast glycolytic (FG) muscle

fibers. Hence, it is tempting to speculate that the independent gene disrupting changes reflect recurrent gene loss events. However, the mechanistic basis of changes in muscle fiber composition between species is yet to be understood. Identifying the genetic changes that determine muscle fiber composition and the sequence of events would clarify when and why the *COA1* gene loss occurred.

Species with exceptionally large body sizes or extremely long lifespans have a greater number of cell divisions. An increment in the number of cell divisions enhances cancer risk. However, paradoxically, large-bodied animals like elephants and whales do not have a higher cancer incidence[33,34]. Cancer resistance due to lineage-specific changes in gene content may explain this paradox[35–37]. While specific genetic changes in mammalian species lead to cancer resistance[38,39], the reasons for lower cancer incidence in birds compared to mammals are mostly unexplored[40].

Interestingly, the *COA1* gene is an oncogene with a role in colorectal cancer[41], and its loss could reduce cancer risk. Silencing of *COA1* by miRNAs strongly suppresses giant cell tumors of the bone[42,43]. Our discovery of *COA1* gene loss in galliforms sets a precedent for the indisputable identification of gene loss events in birds and might reveal other oncogenes which are lost. We also identify *COA1* gene loss in the beaver and naked mole-rat genomes, species that are models to study longevity[16]. High-quality near-complete vertebrate genomes with very few errors will further aid in the large-scale identification of gene loss events across the vertebrate phylogeny[44].

S6 text: Validation of *COA1* annotation

Annotation across most species endorses the existence of four coding exons that produce a ~130 to 140 amino acid (aa) protein. The *COA1* annotation in the human genome (see **Supplementary Fig. S229**) has multiple isoforms with seven exons. The additional three exons annotated in the human genome upstream from the widely conserved four exons need further investigation. Bird species such as *Nipponia nippon*, *Cuculus canorus*, *Pterocles gutturalis*, *Gavia stellata*, *Buceros rhinoceros silvestris*, *Anser cygnoides domesticus*, *Anas platyrhynchos* (corrected in XM_027451320.2), and *Fulmarus glacialis* have annotation for a fifth exon upstream from the widely conserved four exons. Annotation for multiple isoforms of the *COA1* gene also exists in *Athene cunicularia*, *Tyto alba*, *Calidris pugnax*, *Serinus canaria*, *Corvus moneduloides*, *Corvus brachyrhynchos*, *Egretta garzetta*, *Aquila chrysaetos*, *Pipra filicauda*, *Corvus cornix*, *Cygnus atratus*, and *Parus major* (see **Supplementary Table S18)**. We examined RNA-seq datasets of multiple species to evaluate the expression of the isoforms. RNA-seq data in *Colius striatus* and *Eurypyga helias* (which had partial sequences annotated) allowed the reconstruction of full-length open reading frames (ORFs). In addition to bird genomes, the *COA1* gene ortholog is annotated in lizards (*Zootoca vivipara*, *Podarcis muralis*, *Lacerta agilis*, *Anolis carolinensis*, *Gekko japonicus*, *Thamnophis sirtalis*, *Pantherophis guttatus*, *Notechis scutatus*, *Pseudonaja textilis* and *Python bivittatus*), turtles (*Trachemys scripta elegans*, *Chelonia mydas*, *Chelonoidis abingdonii*, *Chrysemys picta*, *Gopherus evgoodei* and *Pelodiscus sinensis*), alligators (*Gavialis gangeticus*, *Alligator sinensis*, *Alligator mississippiensis* and *Crocodylus porosus*), even-toed ungulates (*Bos*

taurus, *Sus scrofa*, *Odocoileus virginianus texanus*, *Bison bison bison*, *Bos indicus x Bos taurus*, *Bos mutus*, *Bubalus bubalis*, *Capra hircus*, *Ovis aries*, *Vicugna pacos*, *Camelus ferus*, *Camelus bactrianus*, *Camelus dromedarius*, *Neophocaena asiaeorientalis asiaeorientalis*, *Balaenoptera acutorostrata scammoni*, *Lipotes vexillifer*, *Lagenorhynchus obliquidens*, *Globicephala melas*, *Orcinus orca*, *Tursiops truncatus*, *Phocoena sinus*, *Monodon monoceros*, *Delphinapterus leucas*, *Physeter catodon* and *Balaenoptera musculus*), odd-toed ungulates (*Equus caballus*, *Equus asinus*, *Equus przewalskii* and *Ceratotherium simum simum*), pangolins (*Manis pentadactyla* and *Manis javanica*), *Galeopterus variegatus*, *Tupaia chinensis* and Primates (*Homo sapiens*, *Macaca mulatta*, *Pan troglodytes*, *Chlorocebus sabaeus*, *Callithrix jacchus*, *Colobus angolensis palliatus*, *Cercocebus atys*, *Macaca fascicularis*, *Macaca nemestrina*, *Papio anubis*, *Theropithecus gelada*, *Mandrillus leucophaeus*, *Trachypithecus francoisi*, *Rhinopithecus bieti*, *Rhinopithecus roxellana*, *Piliocolobus tephrosceles*, *Gorilla gorilla*, *Pan paniscus*, *Pongo abelii*, *Nomascus leucogenys*, *Hylobates moloch*, *Saimiri boliviensis*, *Sapajus apella*, *Cebus imitator*, *Aotus nancymaae*, *Carlito syrichta*, *Propithecus coquereli*, *Microcebus murinus* and *Otolemur garnettii*).

We screened the synteny pattern of the candidate *COA1* gene in Galliformes and Anseriformes using five upstream genes (*STK17A*, *HECW1*, *TNS3*, *PSMA2*, *MRPL32*) and the five downstream genes (*BLVRA*, *VOPP1*, *LANCL2*, *EGFR*, *SEC61G*) (see **Supplementary Table S6 and S19**). The chicken (*Gallus gallus*) has a chromosome level assembly, and the gene occurs on Chromosome 2, and its region is syntenic with human (*Homo sapiens*) chromosome 2 (**Supplementary Fig. S230-231**). The gene synteny is mostly conserved in these species and is present on the same scaffold/chromosome. The blast search of the genome using the query gene sequence of closely related species identified genes missing in the annotation. *Anas platyrhynchos* has chromosome level assembly with the same gene order as *Gallus gallus* (**Supplementary Fig. S232**). *Anser cygnoides* and *Anseranas semipalmata* also contain this conserved gene order. *Anas platyrhynchos*, *Numida meleagris, Coturnix japonica*, *Meleagris gallopavo*, *Aquila chrysaetos chrysaetos, Parus major, Strigops habroptila, Taeniopygia guttata, Felis catus, Canis lupus familiaris, Panthera leo, Pan troglodytes, Pan paniscus, Gorilla gorilla gorilla, Pongo abelii, Macaca mulatta, Ornithorhynchus anatinus, Balaenoptera musculus, Danio rerio, Podarcis muralis, and Chrysemys picta bellii* show syntenic blocks aligning with the human chromosome 7 (**Supplementary Fig. S233-S253**). Synteny-based verification was done clade-wise in birds (see **Supplementary Table S6**), rodents (**Supplementary Table S13**), Carnivores (**Supplementary Table S20**), Primates (**Supplementary Table S21**), and reptiles. Gene order and synteny relationships for representative species from each of the clades are in **Supplementary Fig. S254-S427**. For pairwise alignment of human *COA1* gene with different species, see **Supplementary Fig. S428-S457**.

Vertebrate species have a conserved *COA1* gene intron/exon organizational structure. However, two lineages (Primates and Carnivores) with evidence of intron/exon organization changes have also had *COA1* gene duplication events. To ensure that the observed differences were not a result of incorrect annotation, alignment artifacts, or duplicated copies, we

compared the *COA1* gene organization across diverse vertebrate species. Subsequently, we validated the annotations from NCBI and Ensembl using RNA-seq datasets. Sequencing read haplotypes from the functional and pseudogenized copy can be distinguished as their sequences have diverged.

<u>Analysis of Hi-C (High-throughput Chromosome Conformation Capture) datasets</u>

We downloaded the Hi-C data from ENA (European Nucleotide Archive) for human (*Homo sapiens*), rabbit (*Oryctolagus cuniculus*), naked mole-rat (*Heterocephalus glaber*), mouse (*Mus musculus*), chicken (*Gallus gallus*), cheetah (*Acinonyx jubatus*), rhesus macaque (*Macaca mulatta*) and dog (*Canis lupus familiaris*) (see **Supplementary Table S22)**. For human, rabbit, mouse, rhesus macaque, and dog genomes, we used the data from liver tissue. We used the data from blood tissue for the cheetah and in naked mole-rat the embryonic fibroblast cells. Embryonic tissue fibroblasts, mature erythrocytes, and immature erythrocytes tissue are used for chicken. To process Hi-C data, we used HiCUP (https://www.bioinformatics.babraham.ac.uk/projects/hicup/) with different genome digestion parameters of restriction enzymes (see **Supplementary Table S22**). We made a config file for each species which contains the details of input files and the required path of tools. HiCUP generates valid interaction pairs mapped bam files using bowtie2. The mapped files can be converted into prejuicer format using the hicup2juicer command available in HiCUP.

The prejuicer file lists valid interaction pair information for the contact map. This file was converted into a binary .hic file using juicer tools. We used the .hic format files as input to Juicebox to visualize the interaction map in the genomic region containing *COA1*. Assembly errors result in patterns of interaction between distantly located regions and gaps in the interaction map. We visualized these interaction maps using Juicebox. As a negative control, we simulated the effect of assembly gaps by removing the reads mapped to the genomic regions of different sizes (i.e., 10 kb, 50 kb, 100 kb, 500kb, and 1Mb) from the *COA1* and nearby regions using one human sample (SRR1501150). The visualization of these datasets appeared as gaps in the interaction map generated by Juicebox. When the 10kb region was subtracted, the gap in the interaction was not visible (**Supplementary Fig. S458**). However, when the 50 kb region was subtracted, a small gap is visible at the 25 kb resolution as a region lacking interaction (**Supplementary Fig. S459**). Similarly, when we increase the size of the region for which reads are subtracted to 100Kb, a more apparent gap is visible (**Supplementary Fig. S460**). For the 500kb and 1MB subtracted region, we found an apparent pattern with a large gap without any interaction (**Supplementary Fig. S461-S462**). Based on these results, we suggest that regions > 50kb with assembly errors can be identified by visual inspection of Hi-C interaction maps.

S7 text: Assessing the transcriptional status of *COA1*

No evidence for transcription of *COA1* gene in chicken exists in the RNA-seq data from 23 tissues consisting of blood, bone marrow, breast muscle, bursa, cerebellum, cerebrum, comb, eye, fascia, gallbladder, gizzard, gonad, heart, immature egg, kidney, liver, lung, mature egg,

pancreas, shank, skin, spleen, and uterus (**Supplementary Fig. S466-S534**). Among other galliform species, we found no evidence for the expression of the *COA1* gene. (The spleen and gonad of the peacock, the skin of golden pheasant, gonad, spleen, brain, muscle, liver, and heart of ring-necked pheasant, bursa, gonad spleen, blood and uterus of helmeted guineafowl, breast muscle, gonad, spleen, brain, liver, heart, and bursa of turkey, kidney, liver, muscle, lung, and heart of Japanese quail, the blood of *Colinus virginianus* and blood of *Syrmaticus Mikado*, see **Supplementary Fig. S535-603**). The only galliform species with a transcribed *COA1* gene was *Alectura lathami* in blood tissue (**Supplementary Fig. S604-S606**).

In contrast to Galliformes, the *COA1* gene is intact in the Anseriformes. However, the *COA1* gene annotation in duck (*Anas platyrhynchos platyrhynchos*) contains two isoforms. The more extended isoform codes for a 265 amino acid protein and consists of five exons. The shorter isoform (139 amino acids) is orthologous to the Galliformes ORF. Upon closer inspection of the first exon, only 24 of the 372 bases have RNA-seq read support **(Supplementary Fig. S607)**. Hence, this additional exon might be an annotation artifact or part of the untranslated region. The last four annotated exons, which correspond to the intact 139 amino acid encoding sequence, were found to be robustly expressed in the gonad, spleen, liver, brain, and skin (**Supplementary Fig. S608-S615**). A similar annotation of the fifth exon in *Anser cygnoides domesticus* appears to be an artifact. The gonad, liver, and spleen express the last four exons (see **Supplementary Fig. S616-S622**). The RNA-seq data from blood tissue for magpie goose (*Anseranas semipalmata*) and southern screamer (*Chauna torquata*) also supported the transcription of the *COA1* gene (**Supplementary Fig. S623-S626**).

Having verified the expression of the *COA1* gene in multiple Anseriformes, we screened additional bird RNA-seq datasets to evaluate the transcriptional activity of the intact ORF found in these species. Many other bird genomes have annotations for multiple isoforms of the *COA1* gene, like the duck genomes. These isoforms range in length from 136 to 265 amino acids and 4 to 7 exons. Based on careful examination of multiple RNA-seq datasets across several closely related species and sequence homology, we found that the four-exon transcript coding for a 139 amino acid protein was the only correct annotation in most cases. However, additional exons have a robust expression in rare cases and require further investigation. In the Corvidae group, annotation exists for transcripts of lengths 170 and 139 aa. The first exon of the longer transcript lacked expression.

In comparison, all four transcripts of the shorter transcript are present in the blood tissue of western jackdaw (*Corvus monedula*) as well as gonad, brain, spleen, and liver of hooded crow (*Corvus cornix*) **(Supplementary Fig. S627-S632)**. The common canary (*Serinus canaria*) has three transcripts with 177, 154, and 139 aa **(Supplementary Fig. S633-S634)**. We checked the expression using liver and skin tissue and found support for all three transcripts. However, the transcript with 139 aa was strongly expressed upon closer inspection, and the other two transcripts are potentially artifacts. The great tit (*Parus major*) has two transcripts of lengths 169 and 139 aa. While the kidney and liver express both

transcripts, the first exon has feeble expression and appears artefactual **(Supplementary Fig. S635-S636)**.

The golden eagle (*Aquila chrysaetos*) has four annotated transcripts with lengths of 219, 180, 159, and 139 aa. Transcript of 219 aa length contains six exons, transcripts of length 180 aa, and 159 aa have five exons, and 139 aa transcript contains four exons. We found that exon 1 showed negligible expression, and exons 2 to 6 have high expression levels. However, exon 1 and 2 both have an in-frame stop codon **(Supplementary Fig. S637-S641)**. Hence, we consider that the 139 aa long transcripts expressed in the liver and muscle are correct. Red-throated loon (*Gavia stellata*) has a single five exon transcript of length 155 aa annotated. We discovered a lack of expression in the first exon compared to the last four exons orthologous to the transcript of length 139 aa **(Supplementary Fig. S642-S643)**.

The ruff (*Calidris pugnax*) genome annotates three transcripts with lengths of 233, 229, and 139 aa. Transcript one and two contain seven exons each, and the third transcript contains four exons. Exons 1 and 2 lack expression in the first two transcripts, and the third exon did not have any start codon explaining the transcript. The last four exons have transcripts and are orthologous to the *COA1* gene in other species **(Supplementary Fig. 644-S648)**. In the little egret (*Egretta garzetta*), transcripts of lengths 212 and 203 are annotated and contain five exons. We found evidence of expression of *COA1* in blood tissue **(Supplementary Fig. S649-S650)**. Although the first exon has a lower expression level than the last four exons, the consistent occurrence of the fifth exon across many species suggests it might be part of the untranslated region. We annotated and verified the expression of *COA1* in *Phalacrocorax carbo, Phaethon lepturus, Opisthocomus hoazin,* and *Leptosomus discolor* (**Supplementary Fig. S651-S658)**. *Eurypyga helias* has an unverified transcript length of 121 aa. Hence, we screened the genome and RNA-seq data and found its transcript length is 139 aa **(Supplementary Fig. S659-S661)**. We verified the *COA1* gene expression using RNA-seq data in *Strigops habroptila* as it had less than 100 percent RNA-seq coverage **(Supplementary Fig. S662-S663)**. We also examined the RNA-seq data from a few other bird species to verify the *COA1* gene (see **Supplementary Fig. S664-S711**). Bird species share this conserved gene order (**Supplementary Fig. S712**). The Anolis lizard (*Anolis carolinensis*) liver also expresses the *COA1* gene (**Supplementary Fig. S713-S714**).

RNA-seq datasets from the European rabbit's (*Oryctolagus cuniculus*) heart and liver showed no evidence of transcription of *COA1* (see **Supplementary Fig. S715-S717**). In contrast to the rabbit, intact *COA1* gene is present in the Royle's pika (*Ochotona roylei*) and Daurian pika (*Ochotona dauurica*) with blood RNA-seq datasets showing robust expression (see **Supplementary Fig. S718-719**). Screening RNA-seq datasets from the root ganglion, spinal cord, ovary, liver, spleen, and testis in the naked mole-rat (*Heterocephalus glaber*) revealed no transcription of *COA1* locus (see **Supplementary Fig. S720-728**). The closely related Damaraland mole-rat (*Fukomys damarensis*) has robust *COA1* expression in the brain, liver, and testis (see **Supplementary Fig. S729-S734**). The Brazilian guinea pig (*Cavia aperea*), the guinea pig (*Cavia porcellus*), and the long-tailed chinchilla (*Chinchilla lanigera*) were all found to express the *COA1* gene robustly (see **Supplementary Fig. S735-S742**). The thirteen-lined ground squirrel (*Ictidomys tridecemlineatus*), the Arctic ground squirrel

(*Urocitellus parryii*), the groundhog (*Marmota monax*), and the Himalayan marmot (*Marmota himalayana*) do not express the *COA1* locus (see **Supplementary Fig. S743-S761**). In contrast to these species, the Eurasian red squirrel (*Sciurus vulgaris*) has an intact *COA1* expressed in the skin (see **Supplementary Fig. S762-S763**). Despite gene disrupting mutations, the North American beaver (*Castor canadensis*) *COA1* locus is expressed in the blood and spleen (see **Supplementary Fig. S764-S765**). Other tissues such as the brain, liver, stomach, ovarian follicle, skeletal muscle, and kidney do not show any expression at the *COA1* locus (see **Supplementary Fig. S766-S771**). The expressed transcript might represent a new long non-coding RNA that cannot produce a functional *COA1* protein due to the presence of premature stop codons.

Chromosomal rearrangement in rodent species has resulted in the relocation of genes flanking *COA1* to new locations. The *BLVRA* gene is transcriptionally active in the mouse's (*Mus musculus*) liver and heart even though it has translocated to an entirely different location between *AP4E1* and *NCAPH* (see **Supplementary Fig. S772**). Genes on the left flank consisting of *HECW1*, *PSMA2*, and *MRPL32* are now located beside *ARID4B* and are expressed in the mouse (see **Supplementary Fig. S773-S774**). The genes from the right flank (*MRPS24* and *URGCP*) are also transcriptionally active in the mouse at their new location beside *ANKRD36* (see **Supplementary Fig. S775**). Remnants of *COA1* occur between the *PTPRF* and *HYI* genes. However, no transcriptional activity is seen in the mouse in the region between *PTPRF* and *HYI* genes (see **Supplementary Fig. S776**). The new gene order and gene expression patterns are shared by rat (*Rattus norvegicus*) (see **Supplementary Fig. S777-S781**), steppe mouse (*Mus spicilegus*) (see **Supplementary Fig. S782-S786**), Gairdner's shrewmouse (*Mus pahari*) (see **Supplementary Fig. S787-S791**), Ryukyu mouse (*Mus caroli*) (see **Supplementary Fig. S792-S796**), Algerian mouse (*Mus spretus*) (see **Supplementary Fig. S797-801**), deer mouse (*Peromyscus maniculatus*) (see **Supplementary Fig. S802-S806**), prairie vole (*Microtus ochrogaster*) (see **Supplementary Fig. S807-S811**), golden hamster (*Mesocricetus auratus*) (see **Supplementary Fig. S812-S816**), Mongolian gerbil or Mongolian jird (*Meriones unguiculatus*) (see **Supplementary Fig. S817-S820**), Chinese hamster (Cricetulus griseus) (see **Supplementary Fig. S821-S825**), Northern Israeli blind subterranean mole-rat (*Nannospalax galili*) (see **Supplementary Fig. S826-S830**), white-footed mouse (*Peromyscus leucopus*) (see **Supplementary Fig. S831-S835**) and fat sand rat (*Psammomys obesus*) (see **Supplementary Fig. S836-S840**). The banner-tailed kangaroo rat (*Dipodomys spectabilis*) (see **Supplementary Fig. S841-S842**) has a different gene order and appears to represent one of the pre-CR species. However, we cannot rule out the possibility of genome assembly errors.

The genome assemblies of rodents such as the mouse and rat are well-curated and represent some of the highest-quality reference genomes[44]. To ensure that the CRs identified are correct, we evaluated the correctness of genome assemblies of the mouse (see **Supplementary Fig. S843-S849**) and white-footed mouse (*Peromyscus leucopus*) (see **Supplementary Fig. S850-S857**) using PacBio long-read sequencing datasets. The mouse genome assembly has been finished to a very high quality using artificial clones of genome fragments[45]. We further verified the mouse genome assembly by visualizing the coverage of

assembly fragments across the genomic regions of interest (see **Supplementary Fig. S858-S863**). Repeat regions occur at the boundaries of the EBRs (see the last row of the screenshots). Although repeat regions are a major contributing factor for the misassembly of genomes, the conserved gene orders across several species and concordance in the timing of the CR and support from long-read data support the presence of a genuine change in gene order.

The *COA1* gene is intact and robustly expressed in the platypus's (*Ornithorhynchus anatinus*) heart and brain (see **Supplementary Fig. S864-S866**). Gene order in the short-beaked echidna (*Tachyglossus aculeatus*) matches the platypus and other outgroup species (see **Supplementary Fig. S867)**. In contrast to the monotreme species, all marsupial genomes analyzed have a different gene order following CRs. The gray short-tailed opossum (*Monodelphis domestica*) has the gene *ACVR2B* beside the new location of the right flank genes of *COA1*. The left flank genes are beside *GPR141B*. No traces of the *COA1* gene are found either in the genome assembly or raw read datasets. The opossum's brain expresses these adjacent genes with no transcripts in the intergenic regions (see **Supplementary Fig. S868-S870**). The gene order and transcriptional activity were the same in the tammar wallaby (*Notamacropus eugenii*) (uterus: see **Supplementary Fig. S871-S872**), koala (*Phascolarctos cinereus*) (liver and PBMC (peripheral blood mononuclear cell): see **Supplementary Fig. S873-S875**), the Tasmanian devil (*Sarcophilus harrisii*) (lung and spleen: see **Supplementary Fig. S876-S878**), and the common brushtail (*Trichosurus vulpecula*) (liver: see **Supplementary Fig. S879-S881**). Long-read sequencing data in the koala supports the correctness of genome assembly (see **Supplementary Fig. S882-S884**).

The NCBI annotation documents the presence of transcripts, and the *COA1* gene is remarkably well conserved in ungulate species (see **Supplementary Table S18**). Within ungulate species, certain cervid species have remarkable sprinting abilities that allow them to escape from predators. However, in addition to sprinting ability, these species are resistant to fatigue. Hence, the prediction from our hypothesis is that gene loss would not occur in cervid species. The white-tailed deer (*Odocoileus virginianus*) liver and retropharyngeal lymph node and the red deer (*Cervus elaphus*) blood transcriptomes express *COA1* (see **Supplementary Fig. S885-S888**).

The *COA1* gene has undergone duplication within the primate lineage. We screened the genomes of 27 primate species to track down when the gene duplication event occurred. Based on the presence of the duplicate copies, the duplication event is estimated to have happened in the last 43 million years (see **Supplementary Fig. S18**). Subsequent duplications have also occurred in Nancy Ma's night monkey (*Aotus nancymaae*) and a shared duplication in the black-capped squirrel monkey (*Saimiri boliviensis*) and the Panamanian white-faced capuchin (*Cebus imitator*). Concurrent with the gene duplication, the intron-exon structure of the *COA1* gene has also changed (see **Supplementary Fig. S48**). The functional copy of the *COA1* gene is transcriptionally active in the gray mouse lemur (*Microcebus murinus*) (kidney and lung: see **Supplementary Fig. S889-S890**), the northern greater galago (*Otolemur garnettii*) (liver: see **Supplementary Fig. S891**), Coquerel's sifaka (*Propithecus coquereli*) (see **Supplementary Fig. S892**), Nancy Ma's night monkey (*Aotus*

nancymaae) (liver, heart, and kidney: see **Supplementary Fig. S893-S895**), the common marmoset (*Callithrix jacchus*) (lung, liver, and kidney: see **Supplementary Fig. S896-S897**), the Panamanian white-faced capuchin (*Cebus imitator*) (blood: see **Supplementary Fig. S898-S900**), the black-capped squirrel monkey (*Saimiri boliviensis*) (ovary and heart: see **Supplementary Fig. S901-904**), the sooty mangabey (*Cercocebus atys*) (liver: see **Supplementary Fig. S905-S906**), the olive baboon (*Papio anubis*) (kidney and heart: see **Supplementary Fig. S907-S908**), the crab-eating macaque (*Macaca fascicularis*) (blood and liver: see **Supplementary Fig. S909-S910**), the golden snub-nosed monkey (*Rhinopithecus roxellana*) (heart and blood: see **Supplementary Fig. S911-S912**), human (*Homo sapiens*) (liver : see **Supplementary Fig. S913-S918**), and the Philippine tarsier (*Carlito syrichta*) (see **Supplementary Fig. S919**).

The intron/exon structure of the *COA1* gene has undergone several changes in the carnivore lineage (see **Supplementary Fig. S48**). However, outgroup species such as the horse (*Equus caballus*) and pangolin (*Manis javanica*) lack intron/exon structure (see **Supplementary Fig. S48**). We screened the RNA-seq dataset of multiple carnivore species to validate the annotation and evaluate the intron/exon structure changes. Alternative exon usage was also carefully analyzed to quantify the transcriptional status of *COA1* in different carnivore species. The *COA1* gene is transcriptionally active in the meerkat (*Suricata suricatta*) (testis and liver: see **Supplementary Fig. S920-S922**), dog (*Canis lupus familiaris*) (spleen and skeletal muscle: see **Supplementary Fig. S923-S934**), ferret (*Mustela putorius furo*) (heart and kidney: see **Supplementary Fig. S935-S936**), the giant panda (*Ailuropoda melanoleuca*) (heart and liver: see **Supplementary Fig. S937-S938**), American black bear (*Ursus americanus*) (liver, kidney, and the brain: see **Supplementary Fig. S939-S940**), and Weddell seal (*Leptonychotes weddellii*) (lung and muscle: see **Supplementary Fig. S941-S943**). Detailed investigation of the splice junctions and actual positions of splice sites in dog transcriptome also supports the *COA1* gene annotation.

Skipping of the dog-like-exon-3 occurs in the transcriptomes of tiger (*Panthera tigris altaica*), lion (*Panthera leo persica*), cat (*Felis catus*), and puma (*Puma concolor*) (see **Supplementary Fig. S24-S42 and S944-S949**). Although annotation for the *COA1* locus exists in the cheetah (*Acinonyx jubatus*), we found no transcripts in the skin RNA-seq data (see **Supplementary Fig. S55-S57**). Closer inspection of the *COA1* locus in cheetah suggests gene loss. We further compared the splice isoforms found in canine and felid species through sashimi plots of the *COA1* locus. The sashimi plot shows the links between the splice sites and the number of reads that are splice mapped between these sites (see **Supplementary Fig. S950-S954**). Changes in the splice enhancers and splice silencer elements were also compared between cat and dog (see **Supplementary Fig. S43**).

Co-expressed genes tend to perform related functions and are lost together. Hence, to identify the loss of genes related to *COA1*, we identified the top 50 genes co-expressed with human ortholog based on the correlation values in COXPRESdb ver. 7.3[46]. The presence of orthologs of these co-expressed genes in the high-quality genomes of chicken and mouse using Ensembl BioMart suggests widespread conservation (**Supplementary Table S24**). None of these co-expressed genes appear lost in galliforms or rodents.

S8 text: Molecular evolutionary analyses

Relaxed selection signatures

Molecular signatures of relaxation in the degree of purifying selection generally accompany the loss of gene functionality and have been used as evidence of gene loss[26,47,48]. We relied upon multiple sequence alignments of Carnivores (see **Supplementary Table S1**), rodents (see **Supplementary Table S12**), and Primates (see **Supplementary Table S2**) to identify gene disrupting mutations and changes in intron-exon structure. We evaluated each taxonomic group for lineage-specific relaxed selection (see **Supplementary Table S7** and **S8**). Based on a previous report[11] of positive selection in primate species, we additionally identified positively selected sites using site models implemented in codeml (see **Supplementary Table S3**).

Based on the gene sequence of *COA1*, we could identify eleven galliform species with gene-disrupting mutations (see **Supplementary Table S4** and **S5**). Two other galliform species (*Chrysolophus pictus* and *Phasianus colchicus*) do not express the *COA1* gene in the RNA-seq datasets analyzed. Hence, we looked for signatures of relaxed selection in each of the terminal branches leading to each galliform species. We quantified branch-specific selection patterns using the program RELAX[49] from the HyPhy package and the codeml program from the PAML[50] package.

Identification of signatures of relaxed selection using HyPhy

We labeled the focal species as the foreground to test for relaxed selection in the terminal branches. We used the closely related species (identified based on sequence saturation test available in DAMBE[51] software) as the background species. We downloaded the phylogenetic tree with branch lengths from the TimeTree website. The codons in the ORF sequences were aligned using PRANK aligner using Guidance v2.02. We used RELAX to test for signatures of relaxed selection in each focal species using these alignments by labeling one species at a time as the foreground. The results are provided in **Supplementary Table S9**.

Identification of signatures of relaxed selection using codeml

The lineage-specific selection was inferred using the codeml program from the PAML package. PAML makes use of non-synonymous substitution rate/synonymous substitution rate (dN/dS or ω) to infer selection using three branch-specific models under maximum likelihood approach: M0 (null model), branch-free (alternate model), and branch-neutral (alternate model). We illustrate the various models used, config file settings and model testing procedure, and interpretation using whiteboard photographs and explanations.

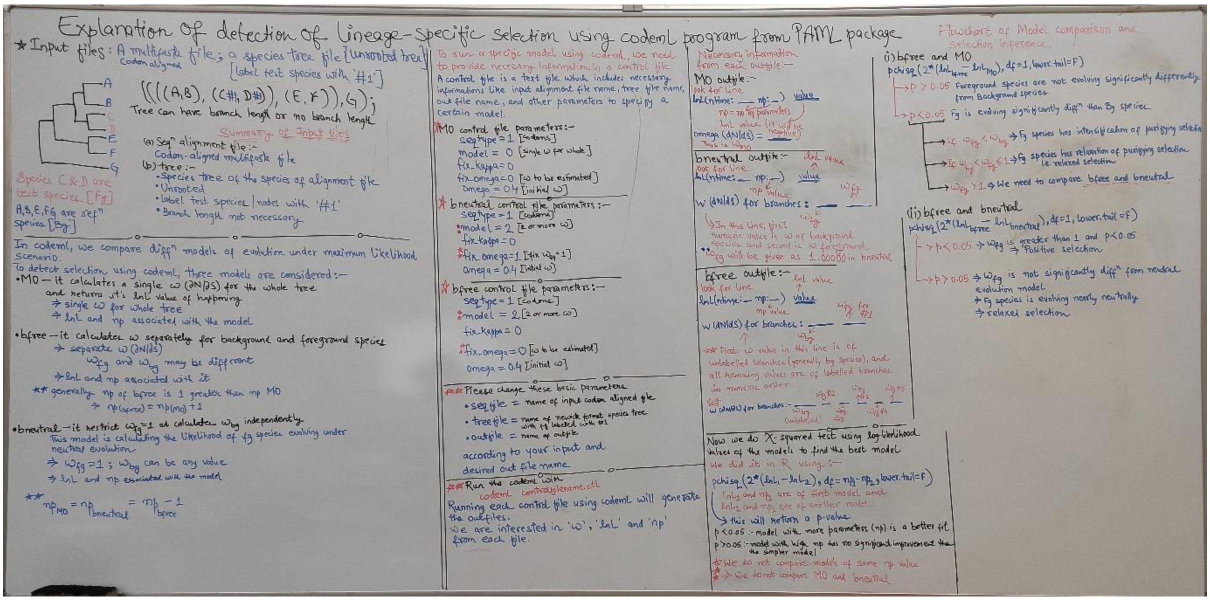

Photograph 1: Photograph of the entire whiteboard explaining the branch tests in codeml.

The M0 model assumes a single ω for all the species, while the branch-neutral model assumes the foreground species to evolve under neutral evolution ($\omega_{fg} = 1$). In contrast, the branch-free model calculates ω independently for foreground and background species. Details of each of these three models are shown in *Photograph 2*.

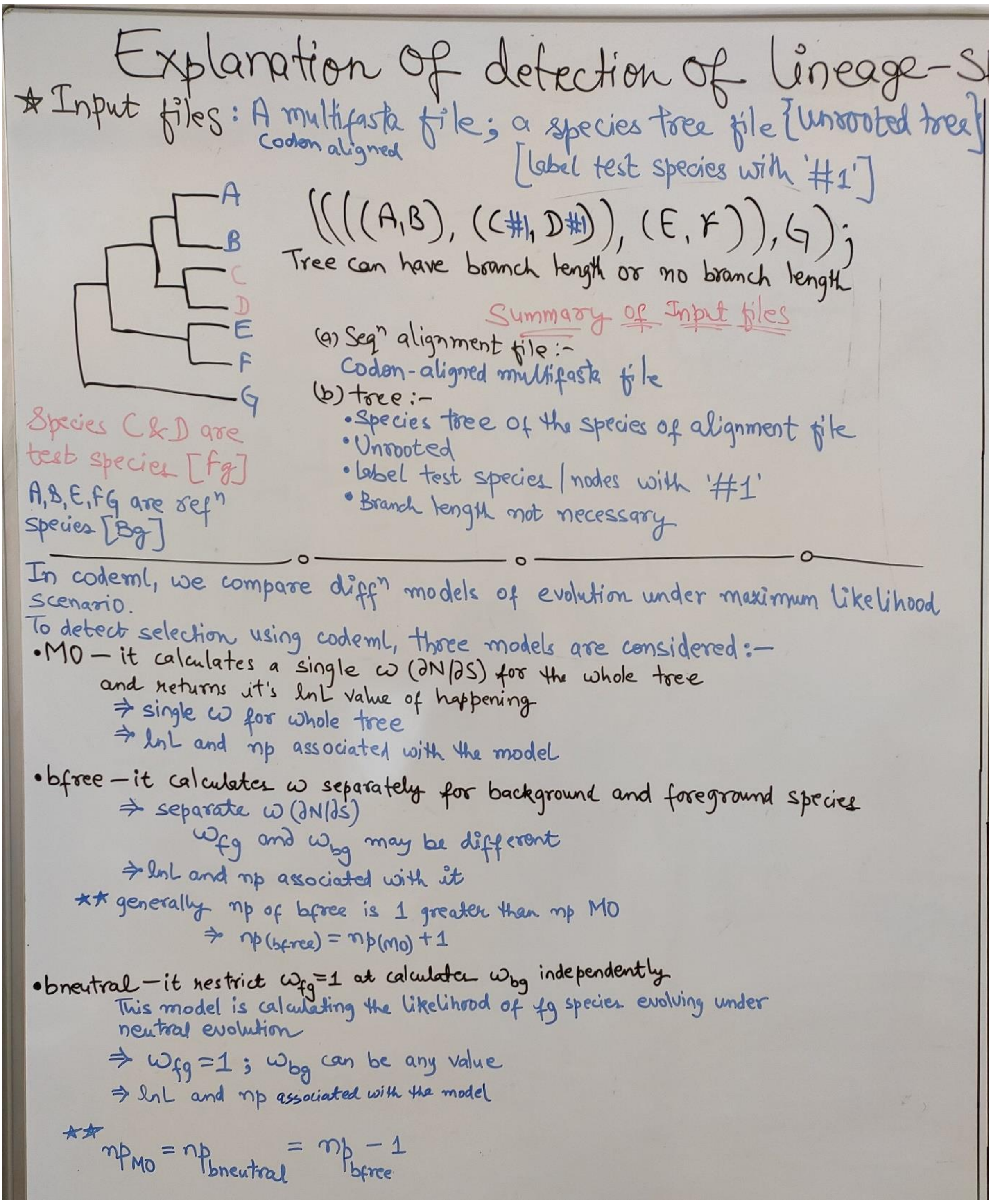

Photograph 2: Details of the three branch models used.

The codeml program is run using a config file with all the input files' details and parameter settings to be used for a particular run. We describe the settings to be used for each of the models in *Photograph 3*. The actual config files, input, and output files are available on the Github repository.

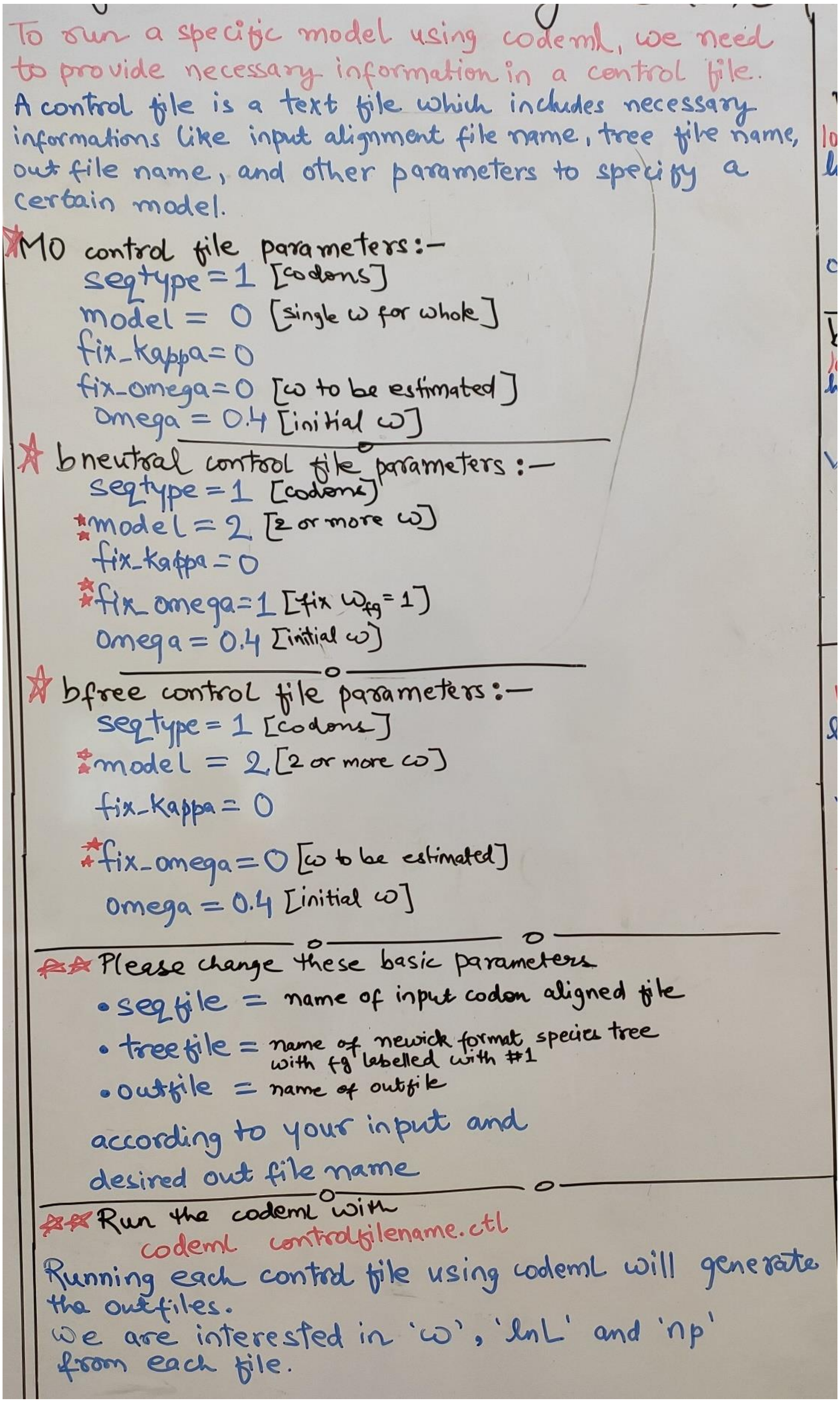

To run a specific model using codeml, we need to provide necessary information in a control file.
A control file is a text file which includes necessary informations like input alignment file name, tree file name, out file name, and other parameters to specify a certain model.

*M0 control file parameters:-
seqtype = 1 [codons]
model = 0 [single ω for whole]
fix_kappa = 0
fix_omega = 0 [ω to be estimated]
omega = 0.4 [initial ω]

* bneutral control file parameters:-
seqtype = 1 [codons]
*model = 2 [2 or more ω]
fix_kappa = 0
*fix_omega = 1 [fix ω_{fg} = 1]
omega = 0.4 [initial ω]

* bfree control file parameters:-
seqtype = 1 [codons]
*model = 2 [2 or more ω]
fix_kappa = 0
*fix_omega = 0 [ω to be estimated]
omega = 0.4 [initial ω]

** Please change these basic parameters
• seqfile = name of input codon aligned file
• treefile = name of newick format species tree with fg labelled with #1
• outfile = name of outfile
according to your input and desired out file name

** Run the codeml with
codeml controlfilename.ctl
Running each control file using codeml will generate the outfiles.
We are interested in 'ω', 'lnL' and 'np' from each file.

Photograph 3: Details of the config file settings used for each branch model.

The output files contain various details regarding the input dataset, config file settings, and output values from the run of the codeml. We require the estimates of ω and log-likelihood values from these output files for performing the likelihood ratio tests, which will determine the best fitting model. The actual lines of the output file which contain this information are

described in *Photograph 4*. The log-likelihood values are then used for calculating the p-values.

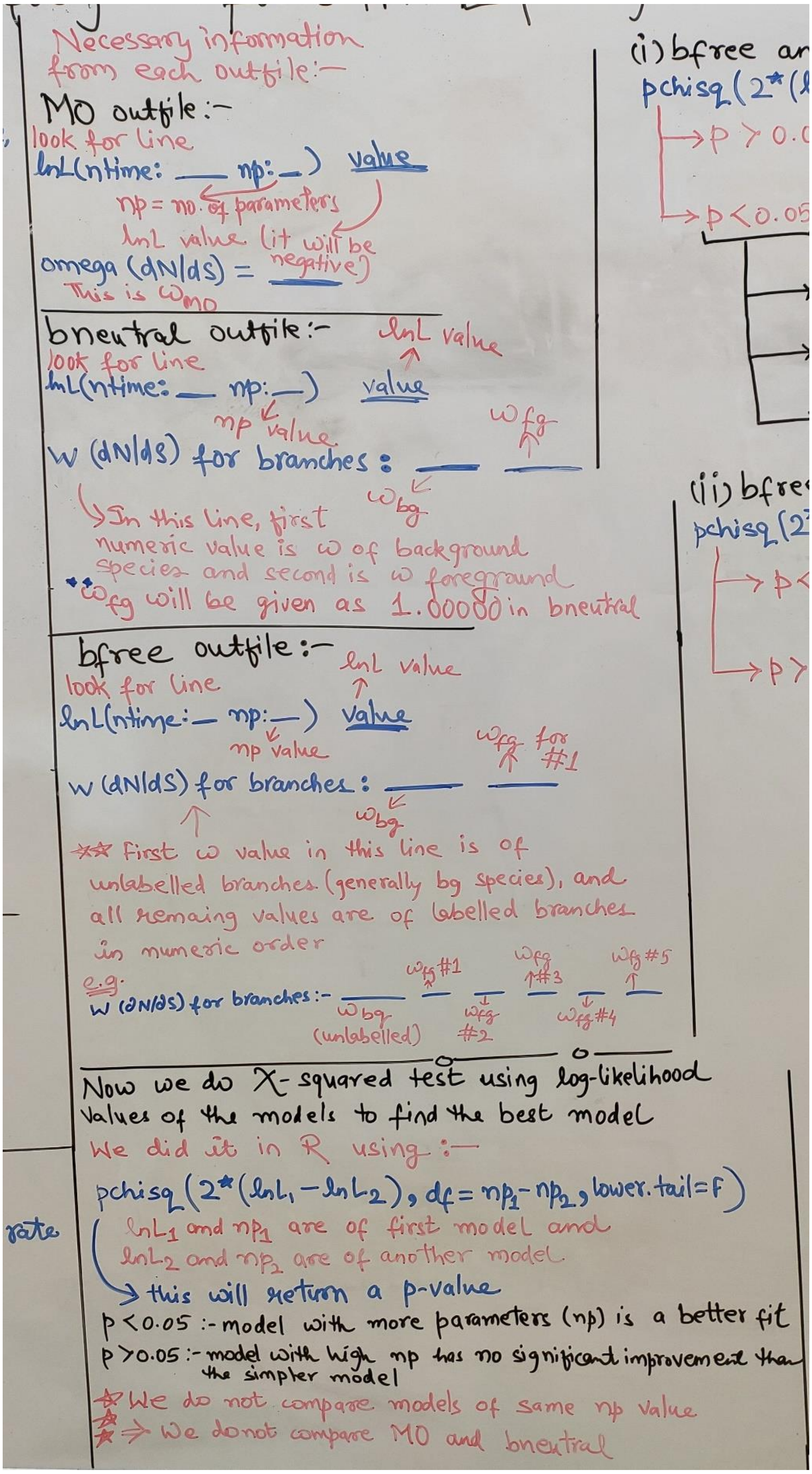

Photograph 4: Lines of the output file containing the relevant information.

The models of evolution were compared using the chi-squared test in a step-wise manner for their log-likelihood values to find the best fit model:

1. Comparison of branch-free and M0 models: A p-value less than 0.05 using the chi-squared test for their log-likelihood values implies that foreground branches are evolving significantly differently from background branches. The $\omega_{fg} < \omega_{bg}$ implies intensification of purifying selection while $\omega_{bg} < \omega_{fg} < 1$ implies relaxation of purifying selection. If $\omega_{fg} > 1$, branch-free and branch-neutral models are compared.

2. Comparison of branch-free and branch-neutral models: A p-value > 0.05 between branch-free and branch-neutral for their log-likelihood values implies that the branch-free model is not better than branch-neutral. This means the foreground species are evolving with near-neutral evolution and can be interpreted as relaxed selection. If the p-value between branch-free and branch-neutral is less than 0.05 and $\omega_{fg} > 1$, this implies that the foreground lineage is under positive selection.

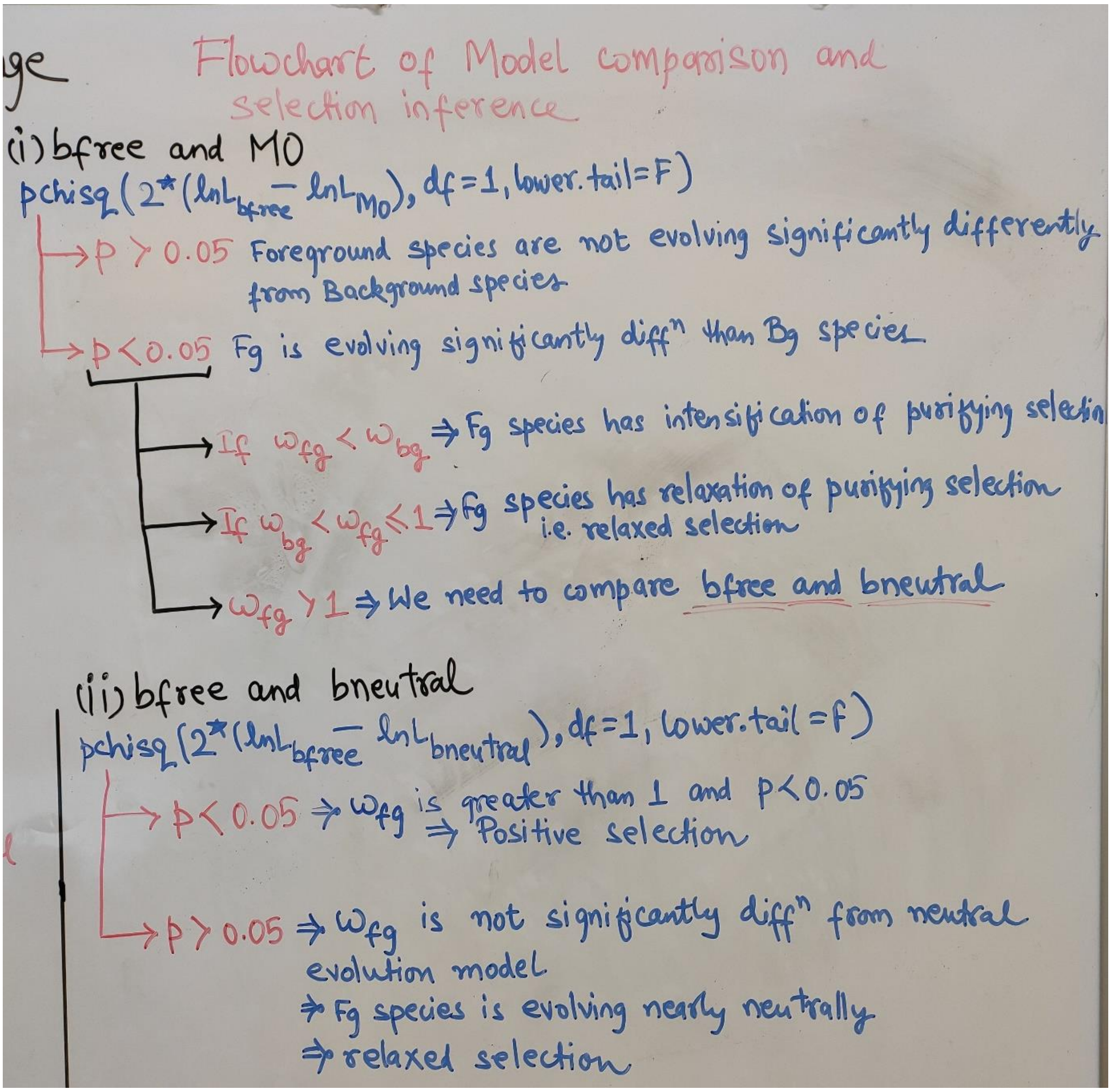

Photograph 5: Steps involved in the interpretation of the likelihood ratio test results.

For our analyses, each functional species was kept as foreground, one at a time, against all other functional species as background. In case of gene duplication (both pseudogene or one functional, one pseudogene), each copy of the gene was considered alternatively against functional copies as background (**Supplementary Table S8**).

Our results for the lineage-specific selection shows that:

- In the Afrotheria group, *Trichechus manatus* is under relaxed selection, and *Orycteropus afer* is under intensification of purifying selection.
- In the Amphibian group, *Pantherophis guttatus* and *Pelodiscus sinensis* are under intensification of purifying selection.
- In Aves group, *Anser cygnoides*, *Chrysolophus pictus*, *Coturnix japonica*, *Gallus gallus*, *Lyrurus tetrix*, *Meleagris gallopavo*, *Numida meleagris*, *Odontophorus gujanensis*, *Pavo cristatus*, *Syrmaticus mikado*, and *Tympanuchus cupido* are under relaxation, and *Picoides pubescens* and *Taeniopygia guttata* are under intensification of purifying selection. The *Phasianus colchicus* (functional *COA1*) was under significant positive selection when all the 88 functional birds sequences were kept as background, but on closer inspection, we observed its dN/dS to be 999. We suspect that it is possibly an artifact.
- In the Carnivora group, *Leptonychotes weddellii* (duplicated *COA1*) is under the intensification of purifying selection.
- In the Primates group, *Chlorocebus sabaeus* is under relaxation, while *Carlito syrichta* is under intensification of purifying selection. In primates, the species with duplicate *COA1*, *Cebus imitator*, is under relaxed selection while *Chlorocebus sabaeus* and *Gorilla gorilla* are under intensification of purifying selection.
- In the rodents (using sequences that are saturated in the analysis), *Marmota marmota* and *Urocitellus parryii* are under relaxed selection.
- In the Rodents (using only unsaturated sequences in the analysis), *Fukomyses damarensis* and *Urocitellus parryii* are under relaxed selection.

We used the codeml program from the PAML package and the RELAX program from the HyPhy package to detect lineage-specific selection. The codeml program was run using the F1x4 and F3x4 codon frequency models. While results were broadly consistent between the two models, we have considered the results of the F3x4 model. Codeml can be used to infer relaxation of purifying selection, intensification of purifying selection, and positive selection. However, RELAX is used primarily to detect relaxed selection and intensified selection. RELAX program cannot delimit between positive selection and intensification of purifying selection. We compared the results generated using both programs to check the consistency and robustness of our results for lineage-specific selection.

For most of the foreground species and background species combinations, our results are consistent between both programs. However, the inference between both programs is not the same for a few species. Below is a list provided for the same:

1. *Anser cygnoides* showed relaxed selection using PAML and non-significant results using HyPhy.
2. *Buceros rhinoceros* showed non-significant results using PAML and relaxed using HyPhy.

3. *Chlamydotis macqueenii* is non-significant results using PAML while intensified using HyPhy.
4. *Phalacrocorax carbo* is detected as intensified using PAML and non-significant results using HyPhy.
5. *Phasianus colchicus* is shown as positively selected using PAML, while HyPhy showed relaxation of selection.
6. *Picoides pubescens* species is detected as intensified using PAML and non-significant results using HyPhy.
7. *Stachyridopsis* ruficeps showed non-significant results using PAML and intensified using HyPhy.
8. *Taeniopygia guttata* is detected as intensified using PAML and non-significant results using HyPhy.
9. *Meleagris gallopavo* is detected as relaxed using PAML and intensified using HyPhy.
10. *Pavo cristatus* is detected as relaxed using PAML and intensified using HyPhy.
11. *Suricata suricatta* showed non-significant results using PAML and relaxed using HyPhy.
12. *Castor canadensis* showed non-significant results using PAML and relaxed using HyPhy.
13. *Ictidomys* tridecemlineatus showed non-significant results using PAML and relaxed using HyPhy.
14. *Oryctolagus cuniculus* showed non-significant results using PAML and intensified using HyPhy.
15. *Spermophilus dauricus* showed non-significant results using PAML and relaxed using HyPhy.
16. *Fukomys damarensis* showed relaxation using PAML and non-significant results using HyPhy.

These differences between the results from the two programs are not unexpected as the results are known to be influenced by the tree topology and set of background species used. Moreover, different versions of these programs also produce slightly different results due to differences in implementation. Codeml and RELAX use different models of sequence evolution and can have different results on the same dataset as well. Given the overall consistency of our results from both programs, the signatures of relaxed selection detected seem to be reasonably trustworthy.

Time of gene loss

The estimates of gene loss time are obtained based on the method initially proposed by Meredith[52] and later used by several papers that have identified and timed gene loss events [22,30,53–58]. Two different models called 1ds and 2ds are used to calculate the time of gene loss. The 1ds estimates are calculated assuming a single dS (i.e., synonymous substitution rate), and 2ds estimates are calculated assuming two different values of dS (one before and another after gene loss). The 2ds approach assumes that the value of the dS before gene loss is 0.7 times the value of dS after gene loss ($dS_f=0.7*dS_p$)[59].

In *Photograph 6,* we briefly explain the approach used to calculate gene loss timing and provide detailed comments for the code used for performing the calculations.

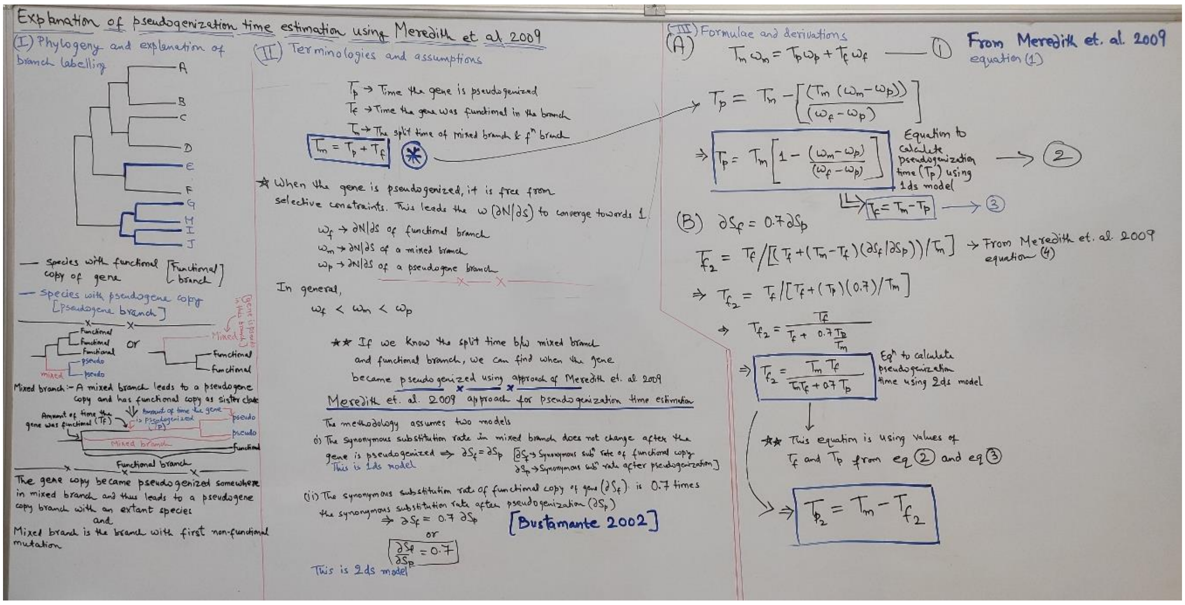

Photograph 6: Photograph of the entire whiteboard explaining the approach of Meredith et al. 2009.

In the first part of the whiteboard, we show the phylogeny and explain branch labeling. Branches of the phylogeny are labeled as either functional, pseudogenic, or mixed. Two example scenarios of what is a mixed-branch are also shown in *Photograph 7*. Please refer to the original publication by Meredith[52] for a detailed explanation.

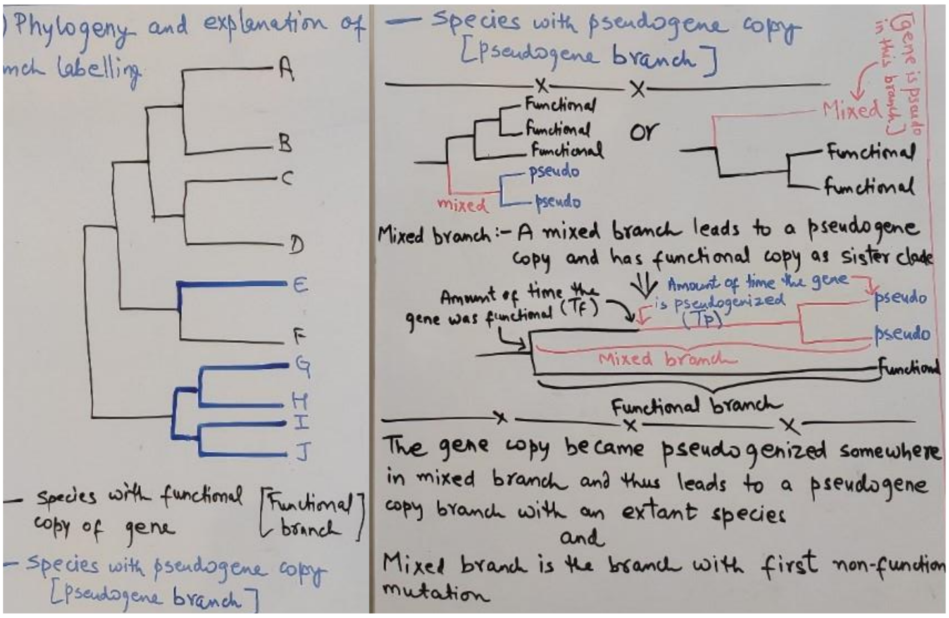

Photograph 7: Phylogeny and branch labeling as used in Meredith et al., 2009.

Having explained the process of labeling in *Photograph 7,* we next explain the terminologies used for estimating the time of gene loss. The estimates of ω are obtained using the branch-

free model (b_free with three estimates of ω) in the codeml program from the PAML package using unrooted trees described above. The actual config files used to run codeml, the multiple sequence alignments, tree files with labeling, and output files are provided in the Github repository. Two different models (**1ds** and **2ds**) are used to estimate the range of pseudogenization time.

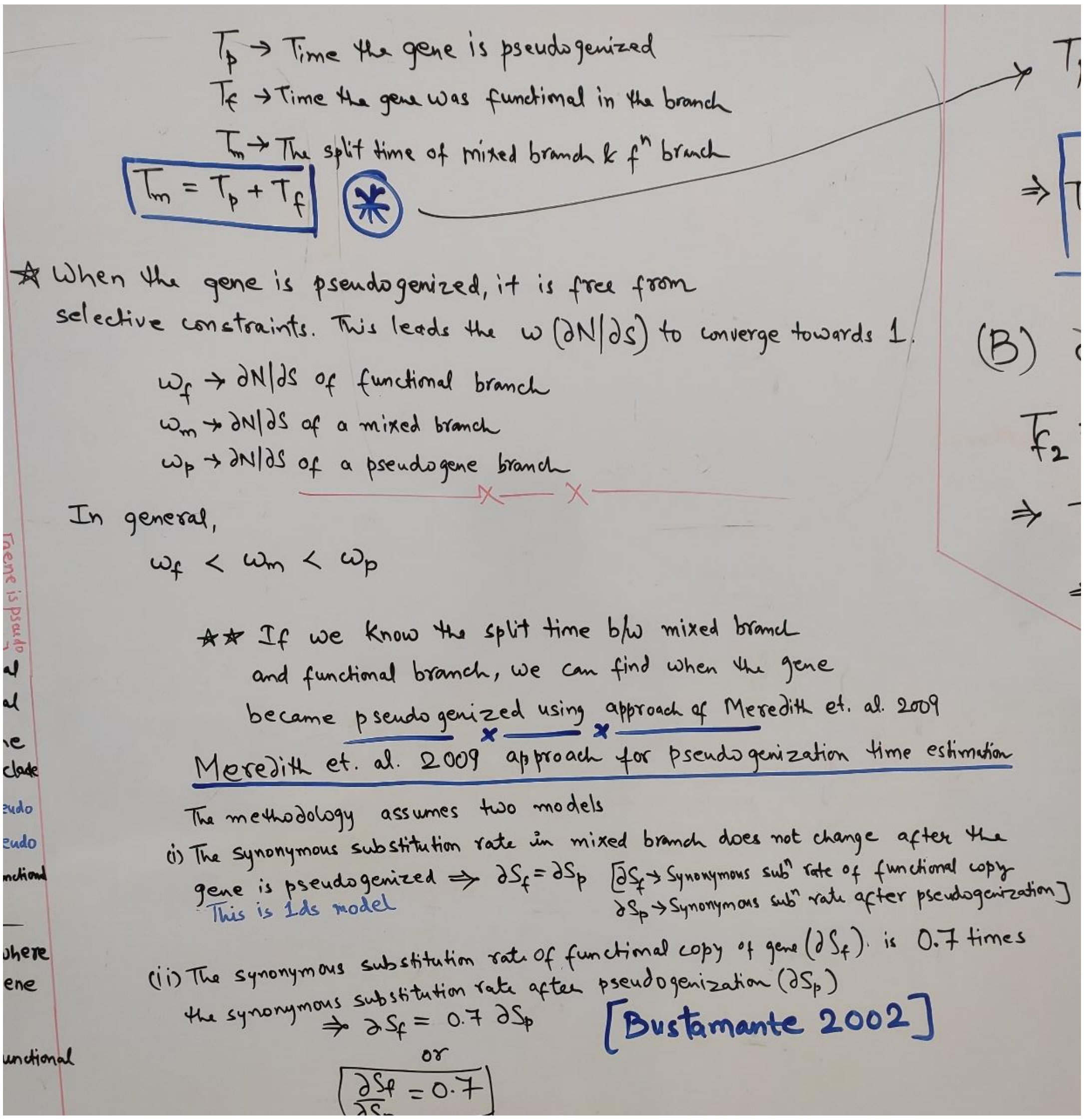

Photograph 8: Terminologies and assumptions used in pseudogenization time estimation by Meredith et al., 2009.

Having explained the terminologies in *Photograph 8*, we next use the equations from Meredith et al., 2009 to calculate the pseudogenization time for each gene loss event. Actual values of each of the estimated ω obtained using codeml, T_m obtained from the TimeTree database, and all the other parameters calculated from these values are summarised in **Supplementary Table S4**. As per the standard procedure, we assume that the ω_p (i.e., ω in the pseudogene branch) equals 1. In cases where the $\omega_m > 1$, the species split time is considered as the time of gene loss.

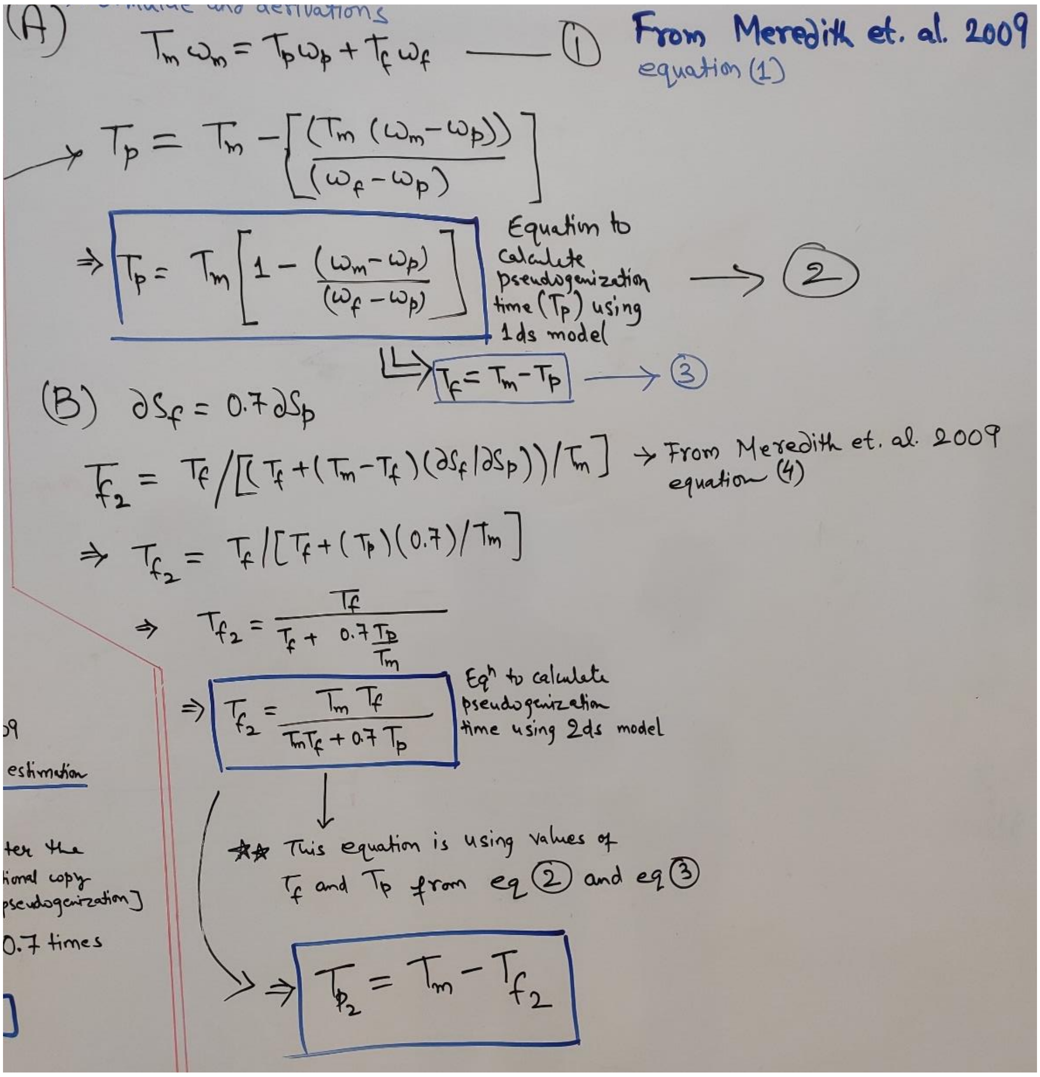

Photograph 9:Formulae and derivations from Meredith et.al., 2009.

Part-A formula in *Photograph 9* is for the **1ds** model and Part-B formula is for the **2ds** model. The following lines of code explain the shell script (wrapper_script.sh) which extracts the ω estimates from the output file of codeml and calculates the various parameters before writing it to the summary files.

#the variable bg is assigned as the list of background species used.

```
bg=`cat bg.txt|tr '\n' ','|sed 's/,$/\n/g'`
```

tm corresponds to T_m. T_m corresponding to the split time between focal species (species with gene loss) and closest species with functional copy is obtained from the split_time.txt file. The split_time.txt file is created from screening the list of all pairwise split times of the focal species with every other species with a functional copy of the gene to obtain the lowest value. See wrapper_script.sh

```
tm=`grep "$sp" split_time.txt|awk '{print $2}'`
```

#mdl corresponds to the codon frequency model used to run codeml and is being obtained from the codeml output file.

```
mdl=`grep "Codon frequency model:" $outfile|awk -F ":" '{print $2}'|awk '{print $1}'`
```

wf corresponds to ω_f and is obtained from the codeml output file.

```
wf=`grep "w (dN/dS) for branches:" $outfile|awk '{print $5}'`
```

wm corresponds to ω_m and is obtained from the codeml output file.

```
wm=`grep "w (dN/dS) for branches:" $outfile|awk '{print $6}'`
```

wp corresponds to ω_p and is obtained from the codeml output file. This is set to 1 in the calculations and not used.

```
wp=`grep "w (dN/dS) for branches:" $outfile|awk '{print $7}'`
```

#tf corresponds to T_f and is being calculated using the 1ds model.

```
tf=`echo $tm $wm $wf | awk '{print $1*(($2 - 1)/($3 - 1))}'`
```

#tp corresponds to T_p and is being calculated using the 1ds model.

```
tp=`echo $tm $tf|awk '{print $1 - $2}'`
```

#tf2 corresponds to T_{f2} and is being calculated using the 2ds model.

```
tf2=`echo $tm $tf $tp|awk '{print ($1*$2)/($2+(0.7*$3))}'`
```

#tp2 corresponds to T_{p2} and is being calculated using the 2ds model.

```
tp2=`echo $tm $tf2|awk '{print $1-$2}'`
```

Pseudogenization timing estimation using Meredith et al., 2009 methodology relies upon ω estimates (dN/dS) and its shift towards 1 in the species of interest. Codeml estimates ω in a phylogenetic framework and requires a multiple sequence alignment and corresponding species tree. However, ω estimates can be affected by several factors such as sequence alignment, background species composition, number of background species, codon frequency model, and number of species labeled. Hence, it is important to check the effect of these confounding factors on pseudogenization timing calculation.

We calculated ω for each species of interest with different combinations of background species and labeling schemes under two different codon frequency models, namely, F1x4 and F3x4. Both pseudogenization timing estimation models, i.e., 1ds and 2ds, were used for each of these estimates. The variation in the estimates of pseudogenization time using these different combinations revealed considerable variation in the estimates for the same species (see **Supplementary Fig. S955**). Increasing the sampling of species closely related to the species with gene loss provides better resolution of the timing of gene loss.

The ω values for mixed (ω_m) and functional (ω_f) branches were estimated using two different codon substitution models (F1x4 and F3x4) to ensure the robustness of the estimates (see **Supplementary Table S4**). The calculation of gene loss timing relies upon estimates of T_p

(time for which the gene has been pseudogenic) using the method Meredith et al. (2009). Based on the assumptions of 1ds and 2ds, we could get a confidence interval for the estimated time of gene loss (see **Supplementary Table S4**). Gene loss timing was estimated separately in galliforms, rodents, and Carnivores (see **Supplementary Table S4**).

GC content range and K-mer abundance

The GC content range (minimum and maximum possible values of GC% for a given amino acid sequence) was calculated (see **Supplementary Table S14**) for *COA1* and *PDX1* amino acid sequences in rodent and primate species using the window-based tool CodSeqGen[60]. The ContMap function in the R package phytools extrapolates the evolution of GC content along the phylogeny for both genes (see **Supplementary Fig. S956-S957**). The program jellyfish (v2.2.8)[61] was used to get the K-mers (count command with the flags -C -m 21 -s 1000M and -t 16) and their abundance (dump command). The seqkit fx2tab (v0.10.1)[62] option calculated the abundance of K-mers at different GC content bins and the GC content of each *COA1* exon (see **Supplementary Table S17**).

Quantification of gBGC

We calculated the gBGC for *COA1* gene sequences of each species using the program mapNH (v1.3.0) implemented in the testNH package[63]. In mapNH, we used multiple sequence alignments of the *COA1* gene and species tree as input with the flag model=K80. A single gene-wide estimate of gBGC termed GC* is obtained for each species (see **Supplementary Table S15**). These estimates of GC* (GC* > 0.9 is significant) help understand the evolution of gBGC along the phylogeny when extrapolated using the ContMap function of the phytools package in R. We also calculated the gBGC for taxonomic group-wise alignments using the phastBias and phyloFit implemented in the PHAST (v1.3) package[64,65]. In the first step, we use the phyloFit program to fit phylogenetic models to multiple sequence alignments using the specified tree topology (--tree flag with species tree as argument) and substitution model (--subst-mod flag with HKY85 model as argument). Next, the phastBias program with the –bgc flag identified gBGC tracts using the ".mod" file output from phyloFit (see **Supplementary Table S16**, see **Supplementary Fig. S189-S222**). The gBGC tracts are positions along the gene with posterior probability > 0.5.

Computational prediction of RNA binding sites

The regulation of gene expression and splicing tends to be determined by the RNA binding sites present within the exons or introns of a gene[66]. A combination of such splice enhancers and splice silencer elements work in concert to facilitate the expression of different isoforms[67]. The *COA1* gene has changed the intron-exon organization and has acquired novel splice isoforms in felid species. These changes in splicing could result from changes in the RNA binding motifs present within the exons or introns of the gene. In contrast to felids, the splicing pattern in canid species matches the ancestral state. Hence, we compared the *COA1* gene sequences of canid and felid species to identify differences in the RNA binding motifs. We used the RBPmap[68] webserver to predict the RNA binding sites in each exon and intron separately (see **Supplementary Table S25-26**).

S9 text: Comparative phylogenetic logistic regression

Based on a search of the literature, we found the following research articles that have quantified the proportion of muscle fibers in birds. The nomenclature used for muscle fiber types has changed with time as identification techniques have improved. Hence, consistently distinguishing the muscle fiber types (FG, FOG, and SO) throughout the dataset is challenging. Nonetheless, white muscle fiber is consistently fast-twitch glycolytic and is used for regression analyses. The quantitative muscle fiber data used for the regression analysis are tabulated in **Supplementary Table S27.**

1. Kaiser et al., 1973[69] describe muscles mainly in two types based on morphology and biochemical properties: a red type (type 1) which are smaller in diameter, have high mitochondrial density, high myoglobin content, neutral fat, and oxidative enzymes, and a white type (type 2) characterized mainly by lack of myoglobin and fat, low density of mitochondria and oxidative enzyme activity but high phosphorylase activity. Red muscle fiber type is adapted for aerobic metabolism metabolizing fat, while white mainly relies on anaerobic metabolism and utilizes glycogen. For the classification of muscle fiber type in pectoralis muscle of birds, the authors use histochemical localization and intensity of succinate dehydrogenase (SDH) activity. Phosphorylase activity was demonstrated using glucose-1-phosphatase, while myoglobin content was measured by fixing muscle samples in phosphate-buffered 2.5% glutaraldehyde. The authors also observed an intermediate muscle fiber type that stains intermediate between white and red.
2. Wiskus et al., 1976[70] mainly describes muscle fibers as αR (Intermediate; Fast twitch), βR (Red; Tonic), and αW (White; Fast-twitch; no glycogen staining implying anaerobic). Samples were reacted for ATPase and SDH activities and stained for glycogen (PAS) and with hematoxylin-eosin. The pectoralis exhibited 90-100% αW fibers, with remaining αR. No β fibers were detected.
3. Kiessling 1976[71] describes muscle fibers as Red (slow-twitch, narrow diameter, high NADH, alkaline ATPase, FOG), White (fast-twitch, large, low NADH, high ATPase, FG), and Intermediate (twitch fiber, in between red and white). Muscle fiber identification was based on the activity of NADH-diaphorase and myosin ATPase. In this study, the author detected only red and white twitch fiber except for quail, which had an intermediate fiber in the pectoralis muscle.
4. Rosser and George 1985[72] use terminologies of FG (fast-twitch glycolytic), FOG (fast-twitch oxidative-glycolytic), and ST (slow-tonic) to describe skeletal muscle fiber types. Myofibrillar adenosine triphosphatase (mATPase) and succinate dehydrogenase (SDH) activity were used for histochemical demonstration. SDH activity revealed that ST have moderate oxidative capacity, and fast-twitch fibers are either moderately oxidative (FOG) or weakly oxidative (FG).
5. Rosser and George 1986[15] broadly describe muscle fibers as slow tonic (alkali-labile/acid-stable mATPase activity) or fast-twitch (alkali-stable/acid-labile mATPase activity). Based on SDH activity, three types of fast-twitch fibers exist: white (low SDH), intermediate (moderate SDH), and red (high SDH). The properties of these

muscles are as follow: R (narrow, myoglobin rich, fat loaded, oxidative; aerobic; FOG); W (broad, devoid of myoglobin and fat, glycolytic, FG; anaerobic); I (in between; FOG). The percentage of fiber types in the pectoralis muscle data consists only of fast-twitch muscles (FG: W; FOG: I + R).

6. Turner and Butler, in a 1988[73] study, defined muscle fiber types based on their histochemical properties as SO (low alkaline mATPase stability/high SDH staining), FOG (high alkaline mATPase stability/high SDH staining); and FG (high alkaline mATPase stability/low SDH staining). For histochemical staining, they used SDH and mATPase.
7. Kenneth Welch and Altshuler 2009[74] described twitch fiber types as SO (slow oxidative), FG (fast glycolytic), and FOG (fast oxidative glycolytic). Muscle fibers were distinguished between slow or fast-twitch by antibody-specific reaction for MHC (myosin heavy chain) isoforms. NADH-tetrazolium reductase was used to further categorize fast-twitch muscle as FG or FOG. The authors also mention that high oxidative muscles are red and low oxidative muscles are white.
8. Geldenhuys et al. 2014[75] modified the methodology of Rosser et al. 1996 and stained samples to quantify immunofluorescence and NADH activity. They identified only two types of muscle fibers: FG and FOG.
9. Schroeder et al. 2015[76] for their study used the terminologies for muscle fiber types as Slow, FG (fast glycolytic), and FOG (fast oxidative glycolytic). MHC-specific antibody tests were done to distinguish between slow and fast muscles, and NADH-TR staining was performed for glycolytic fibers.

References

1. Simakov, O. *et al.* Deeply conserved synteny resolves early events in vertebrate evolution. *Nat. Ecol. Evol. 2020 46* **4**, 820–830 (2020).

2. Mistry, J. *et al.* Pfam: The protein families database in 2021. *Nucleic Acids Res.* **49**, D412–D419 (2021).

3. Fukasawa, Y., Oda, T., Tomii, K. & Imai, K. Origin and Evolutionary Alteration of the Mitochondrial Import System in Eukaryotic Lineages. *Mol. Biol. Evol.* **34**, 1574–1586 (2017).

4. Makarova, K. S., Wolf, Y. I., Mekhedov, S. L., Mirkin, B. G. & Koonin, E. V. Ancestral paralogs and pseudoparalogs and their role in the emergence of the eukaryotic cell. *Nucleic Acids Res.* **33**, 4626 (2005).

5. Montgomerie, S. *et al.* PROTEUS2: a web server for comprehensive protein structure prediction and structure-based annotation. *Nucleic Acids Res.* **36**, W202 (2008).

6. Gautier, R., Douguet, D., Antonny, B. & Drin, G. HELIQUEST: a web server to screen sequences with specific -helical properties. *Bioinformatics* **24**, 2101–2102 (2008).

7. Webb, B. & Sali, A. Comparative protein structure modeling using MODELLER. *Curr. Protoc. Bioinforma.* **2016**, 5.6.1-5.6.37 (2016).

8. Kelley, L. A., Mezulis, S., Yates, C. M., Wass, M. N. & Sternberg, M. J. E. The Phyre2 web portal for protein modeling, prediction and analysis. *Nat. Protoc.* **10**, 845–858 (2015).

9. Waterhouse, A. *et al.* SWISS-MODEL: Homology modelling of protein structures and complexes. *Nucleic Acids Res.* **46**, W296–W303 (2018).

10. Pettersen, E. F. *et al.* UCSF ChimeraX: Structure visualization for researchers, educators, and developers. *Protein Sci.* **30**, 70–82 (2021).

11. Van Der Lee, R., Wiel, L., Van Dam, T. J. P. & Huynen, M. A. Genome-scale detection of positive selection in nine primates predicts human-virus evolutionary conflicts. *Nucleic Acids Res.* **45**, 10634–10648 (2017).

12. Xu, J. & Zhang, J. Are Human Translated Pseudogenes Functional? *Mol. Biol. Evol.* **33**, 755–760 (2016).

13. Mittal, P., Jaiswal, S. K., Vijay, N., Saxena, R. & Sharma, V. K. Comparative analysis of corrected tiger genome provides clues to its neuronal evolution. *Sci. Rep.* **9**, (2019).

14. Pan, S. *et al.* Convergent genomic signatures of flight loss in birds suggest a switch of main fuel. *Nat. Commun. 2019 101* **10**, 1–11 (2019).

15. Rosser, B. W. C. & George, J. C. The avian pectoralis: histochemical characterization and distribution of muscle fiber types. *Can. J. Zool.* **64**, 1174–1185 (1986).

16. Zhou, X. *et al.* Beaver and Naked Mole Rat Genomes Reveal Common Paths to Longevity. *Cell Rep.* **32**, (2020).

17. Chen, Y.-C., Liu, T., Yu, C.-H., Chiang, T.-Y. & Hwang, C.-C. Effects of GC Bias in Next-Generation-Sequencing Data on De Novo Genome Assembly. *PLoS One* **8**, e62856 (2013).

18. Botero-Castro, F., Figuet, E., Tilak, M. K., Nabholz, B. & Galtier, N. Avian genomes revisited: Hidden genes uncovered and the rates versus traits paradox in birds. *Mol. Biol. Evol.* **34**, 3123–3131 (2017).

19. Hargreaves, A. D. *et al.* Genome sequence of a diabetes-prone rodent reveals a mutation hotspot around the ParaHox gene cluster. *Proc. Natl. Acad. Sci. U. S. A.* **114**, 7677–7682 (2017).

20. Xiong, Y. & Lei, F. SLC2A12 of SLC2 Gene Family in Bird Provides Functional Compensation for the Loss of SLC2A4 Gene in Other Vertebrates. *Mol. Biol. Evol.* **38**, 1276–1291 (2021).

21. Vijay, N. Loss of inner kinetochore genes is associated with the transition to an unconventional point centromere in budding yeast. *PeerJ* **8**, (2020).

22. Meredith, R. W., Zhang, G., Gilbert, M. T. P., Jarvis, E. D. & Springer, M. S. Evidence for a single loss of mineralized teeth in the common avian ancestor. *Science* **346**, 1254390 (2014).

23. Huelsmann, M. *et al.* Genes lost during the transition from land to water in cetaceans highlight genomic changes associated with aquatic adaptations. *Sci. Adv.* **5**, 6671–6696

(2019).

24. Valente, R. *et al.* Convergent Cortistatin losses parallel modifications in circadian rhythmicity and energy homeostasis in Cetacea and other mammalian lineages. *Genomics* **113**, 1064–1070 (2021).

25. Schneider, K., Adams, C. E. & Elmer, K. R. Parallel selection on ecologically relevant gene functions in the transcriptomes of highly diversifying salmonids. *BMC Genomics* **20**, 1–23 (2019).

26. Sharma, V. & Hiller, M. Loss of Enzymes in the Bile Acid Synthesis Pathway Explains Differences in Bile Composition among Mammals. *Genome Biol. Evol.* **10**, 3211–3217 (2018).

27. Barber, M. R. W., Aldridge, J. R., Webster, R. G., Magor, K. E. & Magor, K. E. Association of RIG-I with innate immunity of ducks to influenza. *Proc. Natl. Acad. Sci. U. S. A.* **107**, 5913–8 (2010).

28. Sharma, S., Shinde, S. S., Teekas, L. & Vijay, N. Evidence for the loss of plasminogen receptor KT gene in chicken. *Immunogenetics* **72**, 507–515 (2020).

29. Castro, L. F. C. *et al.* Recurrent gene loss correlates with the evolution of stomach phenotypes in gnathostome history. *Proc. R. Soc. B Biol. Sci.* **281**, 20132669–20132669 (2013).

30. Jebb, D. & Hiller, M. Recurrent loss of HMGCS2 shows that ketogenesis is not essential for the evolution of large mammalian brains. *Elife* **7**, (2018).

31. Hecker, N., Sharma, V. & Hiller, M. Convergent gene losses illuminate metabolic and physiological changes in herbivores and carnivores. *Proc. Natl. Acad. Sci. U. S. A.* **116**, 3036–3041 (2019).

32. Sharma, V. *et al.* Convergent Losses of TLR5 Suggest Altered Extracellular Flagellin Detection in Four Mammalian Lineages. *Mol. Biol. Evol.* **37**, 1847–1854 (2020).

33. Peto, H., Roe, F. J. C., Lee, P. N., Levy, L. & Clack, J. Cancer and ageing in mice and men. *Br. J. Cancer* **32**, 411–426 (1975).

34. Tollis, M., Boddy, A. M. & Maley, C. C. Peto's Paradox: How has evolution solved the problem of cancer prevention? *BMC Biology* vol. 15 60 (2017).

35. Caulin, A. F. & Maley, C. C. Peto's Paradox: Evolution's prescription for cancer prevention. *Trends in Ecology and Evolution* vol. 26 175–182 (2011).

36. Caulin, A. F., Graham, T. A., Wang, L.-S. & Maley, C. C. Solutions to Peto's paradox revealed by mathematical modelling and cross-species cancer gene analysis. *Philos. Trans. R. Soc. B Biol. Sci.* **370**, 20140222 (2015).

37. DeGregori, J. Evolved tumor suppression: Why are we so good at not getting cancer? *Cancer Research* vol. 71 3739–3744 (2011).

38. Vazquez, J. M., Sulak, M., Chigurupati, S. & Lynch, V. J. A Zombie LIF Gene in Elephants Is Upregulated by TP53 to Induce Apoptosis in Response to DNA Damage. *Cell Rep.* **24**, 1765–1776 (2018).

39. Tollis, M. *et al.* Return to the Sea, Get Huge, Beat Cancer: An Analysis of Cetacean Genomes Including an Assembly for the Humpback Whale (Megaptera novaeangliae). *Mol. Biol. Evol.* **36**, 1746–1763 (2019).

40. Møller, A. P., Erritzøe, J. & Soler, J. J. Life history, immunity, Peto's paradox and tumours in birds. *J. Evol. Biol.* **30**, 960–967 (2017).

41. Xue, Y. *et al.* Cytochrome C Oxidase Assembly Factor 1 Homolog Predicts Poor Prognosis and Promotes Cell Proliferation in Colorectal Cancer by Regulating PI3K/AKT Signaling. *Onco. Targets. Ther.* **Volume 13**, 11505–11516 (2020).

42. Fellenberg, J. *et al.* Restoration of miR-127-3p and miR-376a-3p counteracts the neoplastic phenotype of giant cell tumor of bone derived stromal cells by targeting COA1, GLE1 and PDIA6. *Cancer Lett.* **371**, 134–141 (2016).

43. Herr, I. *et al.* MiR-127 and miR-376a act as tumor suppressors by in vivo targeting of COA1 and PDIA6 in giant cell tumor of bone. *Cancer Lett.* **409**, 49–55 (2017).

44. Rhie, A. *et al.* Towards complete and error-free genome assemblies of all vertebrate species. *Nature* **592**, 737–746 (2021).

45. Osoegawa, K. *et al.* Bacterial artificial chromosome libraries for mouse sequencing and functional analysis. *Genome Res.* **10**, 116–128 (2000).

46. Obayashi, T., Kagaya, Y., Aoki, Y., Tadaka, S. & Kinoshita, K. COXPRESdb v7: A gene coexpression database for 11 animal species supported by 23 coexpression platforms for technical evaluation and evolutionary inference. *Nucleic Acids Res.* **47**, D55–D62 (2019).

47. Shinde, S. S., Teekas, L., Sharma, S. & Vijay, N. Signatures of Relaxed Selection in the CYP8B1 Gene of Birds and Mammals. *J. Mol. Evol.* **87**, 209–220 (2019).

48. Hecker, N., Sharma, V. & Hiller, M. Transition to an Aquatic Habitat Permitted the Repeated Loss of the Pleiotropic KLK8 Gene in Mammals. *Genome Biol. Evol.* **9**, 3179–3188 (2017).

49. Wertheim, J. O., Murrell, B., Smith, M. D., Kosakovsky Pond, S. L. & Scheffler, K. RELAX: Detecting Relaxed Selection in a Phylogenetic Framework. *Mol. Biol. Evol.* **32**, 820–832 (2015).

50. Yang, Z. PAML 4: Phylogenetic Analysis by Maximum Likelihood. *Mol. Biol. Evol.* **24**, 1586–1591 (2007).

51. Xia, X. DAMBE5: A Comprehensive Software Package for Data Analysis in Molecular Biology and Evolution. *Mol. Biol. Evol.* **30**, 1720–1728 (2013).

52. Meredith, R. W., Gatesy, J., Murphy, W. J., Ryder, O. A. & Springer, M. S. Molecular decay of the tooth gene enamelin (ENAM) mirrors the loss of enamel in the fossil record of placental mammals. *PLoS Genet.* **5**, (2009).

53. Gaudry, M. J. *et al.* Inactivation of thermogenic UCP1 as a historical contingency in multiple placental mammal clades. *Sci. Adv.* **3**, e1602878 (2017).

54. Meredith, R. W., Gatesy, J., Cheng, J. & Springer, M. S. Pseudogenization of the tooth

gene enamelysin (MMP20) in the common ancestor of extant baleen whales. *Proc. R. Soc. B Biol. Sci.* **278**, 993–1002 (2011).

55. Jiao, H. *et al.* Trehalase Gene as a Molecular Signature of Dietary Diversification in Mammals. *Mol. Biol. Evol.* **36**, 2171–2183 (2019).

56. Meyer, W. *et al.* Ancient convergent losses of Paraoxonase 1 yield potential risks for modern marine mammals. *Science* **361**, 591–594 (2018).

57. Janiak, M., Pinto, S., Duytschaever, G., Carrigan, M. & Melin, A. Genetic evidence of widespread variation in ethanol metabolism among mammals: revisiting the 'myth' of natural intoxication. *Biol. Lett.* **16**, (2020).

58. Emerling, C. A. Genomic regression of claw keratin, taste receptor and light-associated genes provides insights into biology and evolutionary origins of snakes. *Mol. Phylogenet. Evol.* **115**, 40–49 (2017).

59. Bustamante, C. D., Nielsen, R. & Hartl, D. L. A Maximum Likelihood Method for Analyzing Pseudogene Evolution: Implications for Silent Site Evolution in Humans and Rodents. *Mol. Biol. Evol.* **19**, 110–117 (2002).

60. Al-Ssulami, A. M., Azmi, A. M. & Hussain, M. CodSeqGen: A tool for generating synonymous coding sequences with desired GC-contents. *Genomics* **112**, 237–242 (2020).

61. Marçais, G. & Kingsford, C. A fast, lock-free approach for efficient parallel counting of occurrences of k-mers. *Bioinformatics* **27**, 764–770 (2011).

62. Shen, W., Le, S., Li, Y. & Hu, F. SeqKit: A cross-platform and ultrafast toolkit for FASTA/Q file manipulation. *PLoS One* **11**, e0163962 (2016).

63. Dutheil, J. Detecting site-specific biochemical constraints through substitution mapping. *J. Mol. Evol.* **67**, 257–265 (2008).

64. Capra, J. A., Hubisz, M. J., Kostka, D., Pollard, K. S. & Siepel, A. A Model-Based Analysis of GC-Biased Gene Conversion in the Human and Chimpanzee Genomes. *PLoS Genet.* **9**, e1003684 (2013).

65. Hubisz, M. J., Pollard, K. S. & Siepel, A. Phast and Rphast: Phylogenetic analysis with space/time models. *Brief. Bioinform.* **12**, 41–51 (2011).

66. Fu, X. D. & Ares, M. Context-dependent control of alternative splicing by RNA-binding proteins. *Nature Reviews Genetics* vol. 15 689–701 (2014).

67. Dassi, E. Handshakes and fights: The regulatory interplay of RNA-binding proteins. *Frontiers in Molecular Biosciences* vol. 4 67 (2017).

68. Paz, I., Kosti, I., Ares, M., Cline, M. & Mandel-Gutfreund, Y. RBPmap: A web server for mapping binding sites of RNA-binding proteins. *Nucleic Acids Res.* **42**, W361 (2014).

69. Kaiser, C. E. & George, J. C. Interrelationship amongst the avian orders Galliformes, Columbiformes, and Anseriformes as evinced by the fiber types in the pectoralis muscle. *Can. J. Zool.* **51**, 887–892 (1973).

70. Wiskus, K. J., Addis, P. B. & Ma, R. .-I. Distribution of βR, αR and αW Fibers in Turkey Muscles. *Poult. Sci.* **55**, 562–572 (1976).

71. Kiessling, K. Muscle structure and function in the goose, quail, pheasant, guinea hen, and chicken. *Comp. Biochem. Physiol. B.* **57**, 287–292 (1977).

72. Rosser, B. W. C. & George, J. C. Histochemical Characterization and Distribution of Fiber Types in the Pectoralis Muscle of the Ostrich (Struthio camelus) and Emu (Dromaius novaehollandiae). *Acta Zool.* **66**, 191–198 (1985).

73. Turner, D. L. & Butler, P. J. The aerobic capacity of locomotory muscles in the tufted duck, Aythya fuligula. *J. Exp. Biol.* **135**, 445–460 (1988).

74. Welch, K. C. & Altshuler, D. L. Fiber type homogeneity of the flight musculature in small birds. *Comp. Biochem. Physiol. - B Biochem. Mol. Biol.* **152**, 324–331 (2009).

75. Geldenhuys, G., Hoffman, L. C. & Muller, N. HISTOLOGICAL CHARACTERIZATION OF THE FIBER TYPES IN THE M. PECTORALIS OF EGYPTIAN GEESE: A SOUTHERN AFRICAN WILDFOWL SPECIES. 17–22 (2014).

76. Schroeder, K. L., Sylvain, N. J., Kirkpatrick, L. J. & Rosser, B. W. C. Fibre types in primary ‘flight’ muscles of the African Penguin (Spheniscus demersus). *Acta Zool.* **96**, 510–518 (2015).

Supplementary Figures

Recurrent erosion of *COA1* exemplifies conditional gene dispensability in oxidative phosphorylation

Sagar Sharad Shinde, Sandhya Sharma, Lokdeep Teekas, Ashutosh Sharma, Nagarjun Vijay

Computational Evolutionary Genomics Lab, Department of Biological Sciences, IISER Bhopal, Bhauri, Madhya Pradesh, India

*Correspondence: nagarjun@iiserb.ac.in

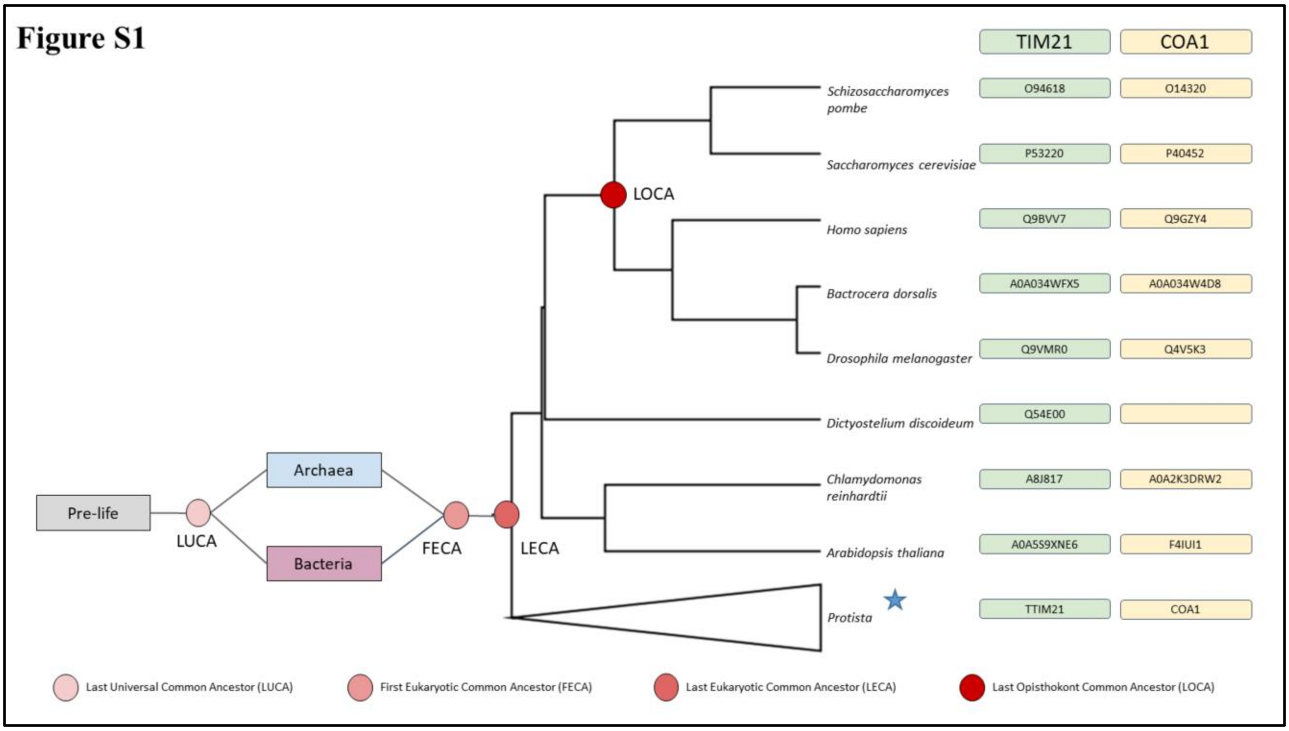

Figure S1: Presence of *TIMM21* and *COA1* gene in different clades. The blue star at Protista is representing a collapsed node of Protista. The elaborated taxanomic relationship within Protista is shown in **Supplementary Fig. S2**.

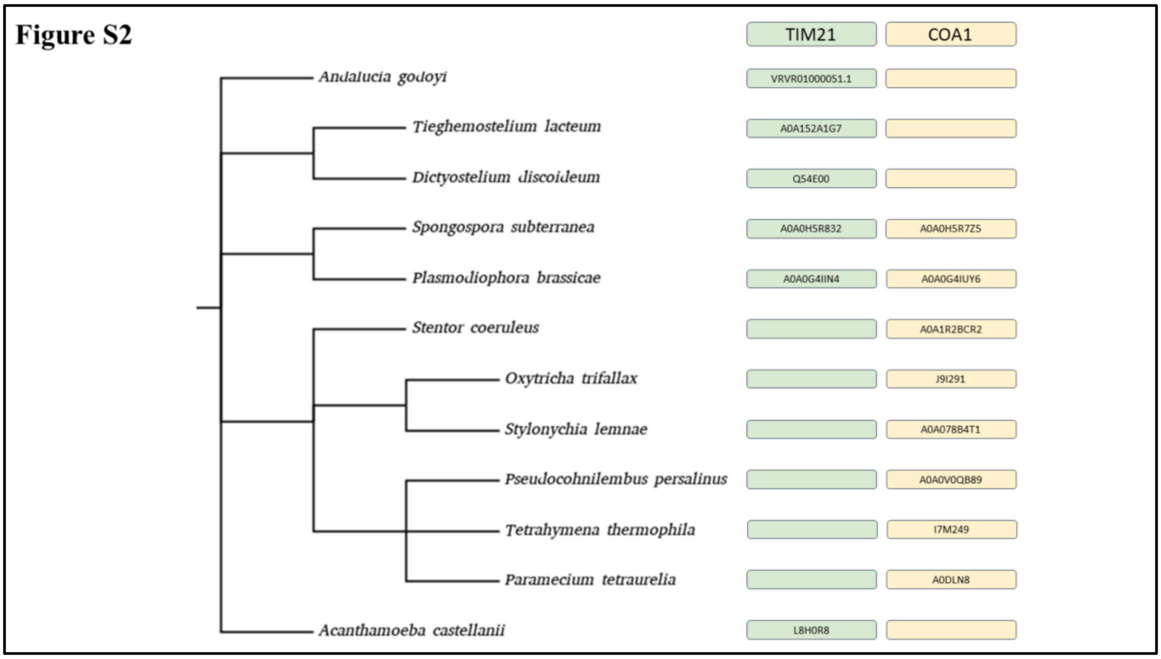

Figure S2: Taxanomic relationship of species within Protista. The tree representing taxanomic relationships is downloaded in the phylip format from NCBI taxonomy.

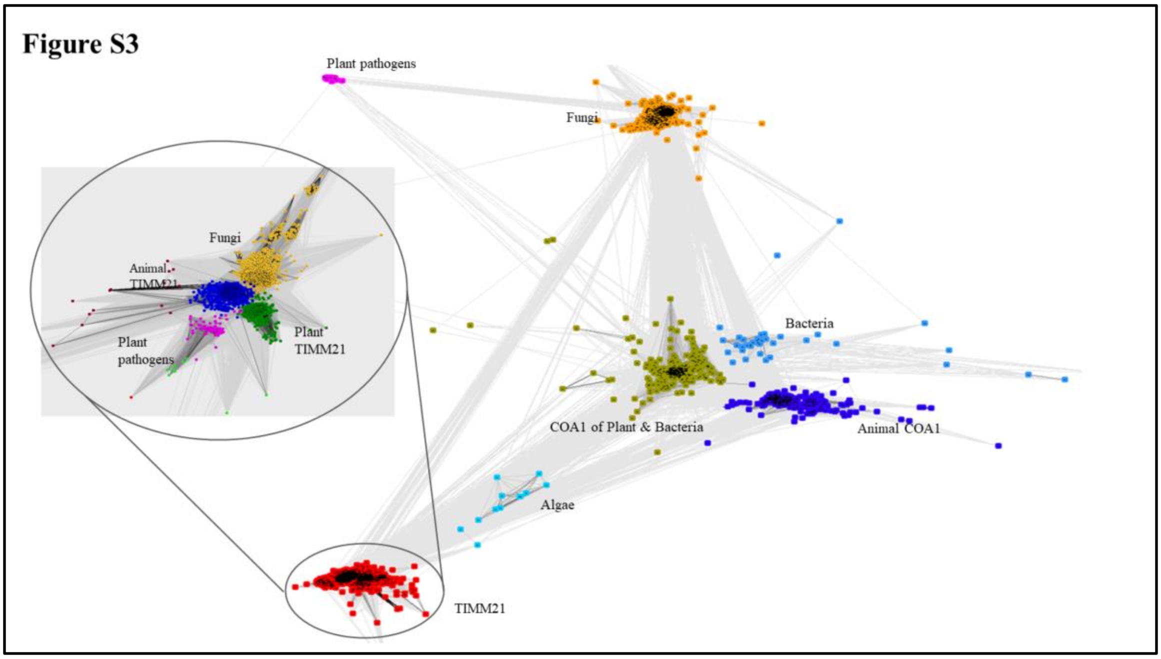

Figure S3: Cluster map of *COA1* and *TIMM21.* The amino acid sequences were downloaded from Pfam 34.0 database and used to generate the clustermap using CLANS. Different colors are used to represent distinct clusters: *COA1* is dark blue, *TIMM21* is red, homologs of *COA1* from species of fungi are in dark yellow, homologs of *COA1* from bacterial species are in the light blue cluster. *COA1* homologs in plants and bacteria are in forest green color, *COA1* from algae are in aqua blue, and homologs of *COA1* from plant pathogens are in magenta. The inset is the zoomed-in figure of *TIMM21* sequence clusters where the cluster of fungi is in yellow, animals in dark blue, plant pathogens in magenta, and the cluster of plants in dark green.

Figure S4

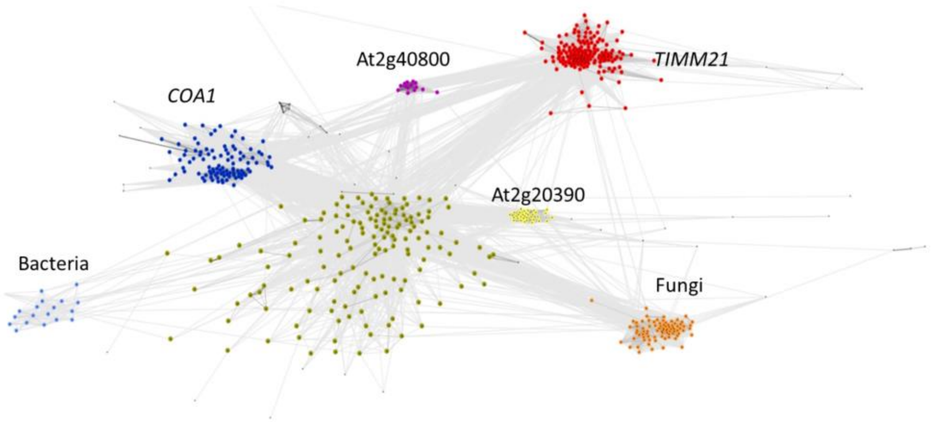

Figure S4: Cluster map of *COA1* homologs identified using profile-profile search implemented in HHblits. The cluster of *COA1*: dark blue, *TIMM21*: red, homologs of *COA1* from species of fungi: orange, homologs of *COA1* from bacterial species: light blue cluster and diffuse brown cluster, *COA1* homologs in plants, represented by *Arabidopsis thaliana* homolog At2g20390: yellow, *TIMM21*-like proteins that exist as duplicated copies in plants, represented by *Arabidopsis thaliana* homolog At2g40800: magenta.

Figure S5

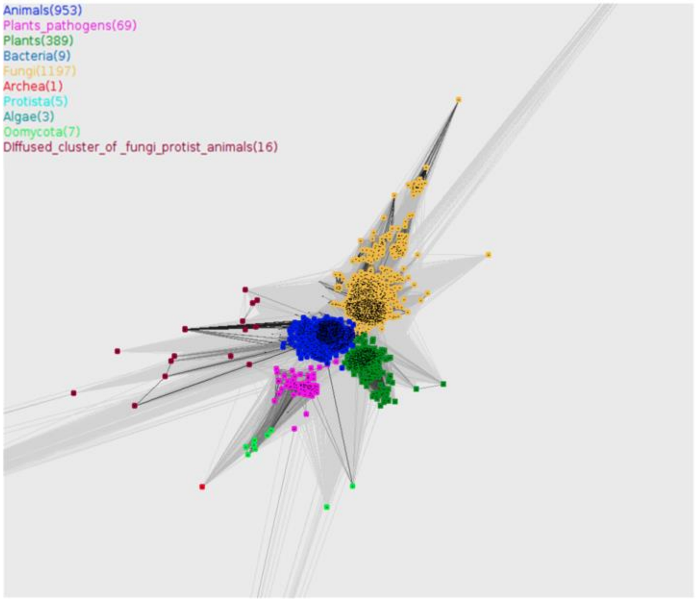

Figure S5: Cluster map of *TIMM21* amino acid sequences downloaded from Pfam 34.0 database, clusters were generated by CLANS. The clusters of fungi *TIMM21*: yellow, animal *TIMM21*: dark blue, plant pathogens cluster: magenta, plant *TIMM21* cluster: dark green, the diffused brown cluster is from fungi, protist, and animals, oomycota *TIMM21* cluster: green.

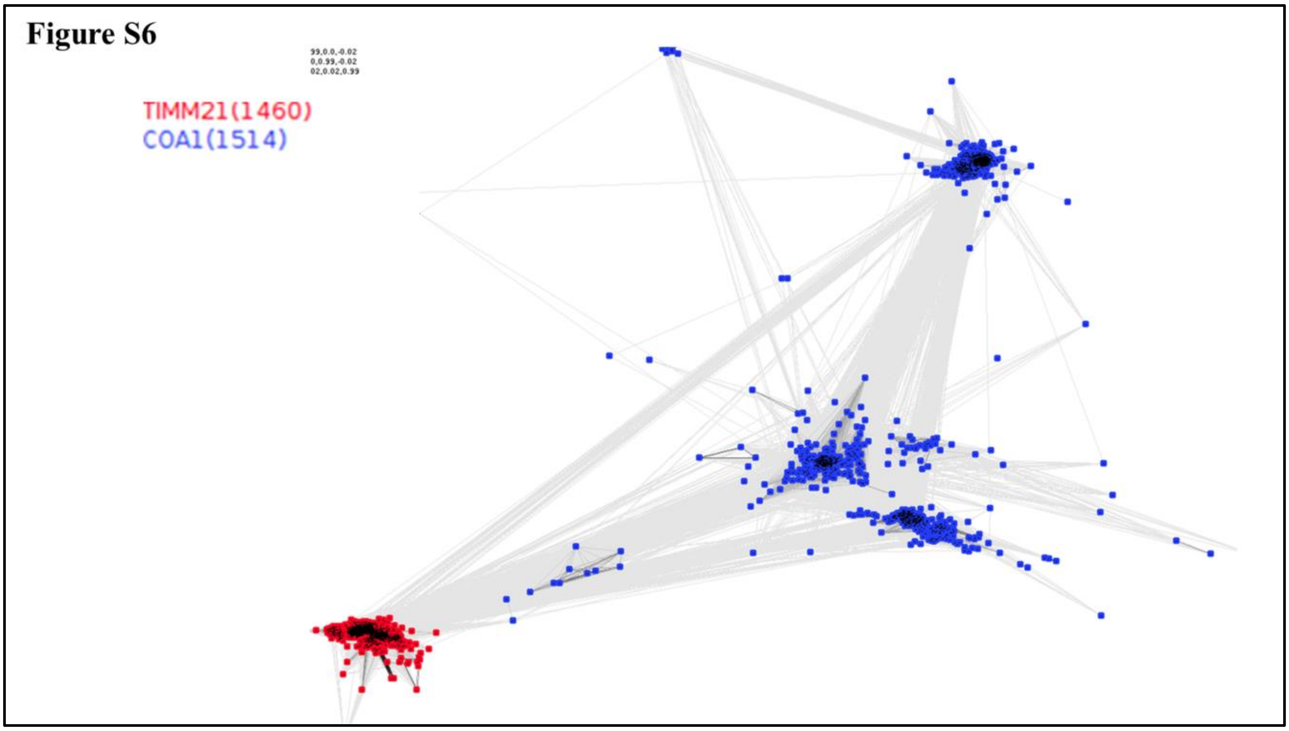

Figure S6: Cluster map of *COA1* and *TIMM21* amino acid sequences downloaded from Pfam 34.0 database, clusters were generated by CLANS. The cluster of *COA1*: dark blue, *TIMM21*: red.

Figure S7 Properties of predicted membrane helix and helix in *COA1*

COA1	Residues	Hydrophobicity	Hydrophobic moment	% polar residues
**Predicted transmembrane helix (MH) in *COA1***				
MH1	16-37	0.88818	0.05041	27.3
**Predicted helix (H)**				
H1	39-56	0.36833	0.10717	50

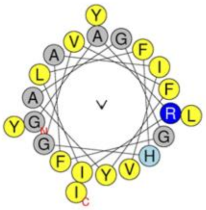

Membrane helix

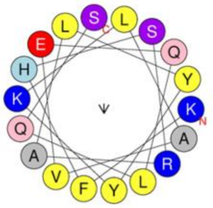

Helix

Figure S7: The HeliQuest predicted membrane properties of the membrane helix and other helix of human *COA1*.

Figure S8

Properties of predicted membrane helix and helix in *TIMM21*

TIM21	Residues	Hydrophobicity	Hydrophobic moment	% polar residues
**Predicted transmembrane helix (MH) in *TIMM21***				
MH1	75-98	0.93458	0.07898	33.3
**Predicted helix (H)**				
H1	23-35	0.43231	0.06286	69.2
H2	104-118	0.23067	0.37524	60

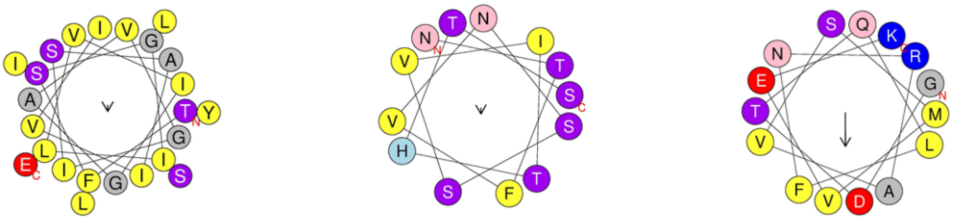

Membrane helix   Helix 1   Helix 2

Figure S8: The HeliQuest predicted membrane properties of membrane helix and helix of yeast *TIMM21*.

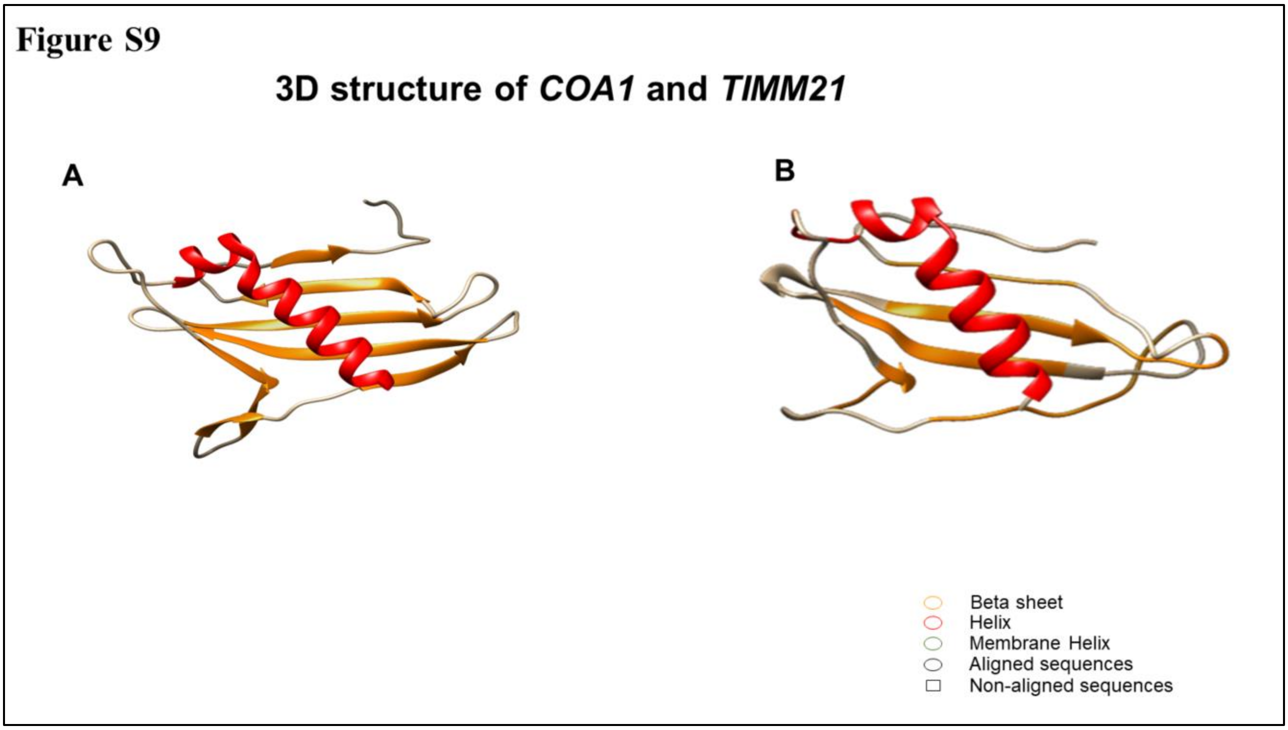

Figure S9: Structure of *COA1* and *TIMM21*. (**A**) 3D structure of *TIMM21* of *Saccharomyces cerevisiae* downloaded from the PDB database (2ciu). (**B**) The 3D structure of human *COA1* sequence homology modelled with modeller software using *TIMM21* (*Saccharomyces cerevisiae*) PDB structure.

Figure S10

HHblits output with *COA1* of DUCK

```
>UniRef100_A0A023FP12 TIM21-like protein, mitochondrial n=1 Tax=Amblyomma cajennense TaxID=34607 RepID=A0A023FP12_AMBCJ
Probab=99.79  E-value=9.6e-23  Score=155.60  Aligned_cols=121  Identities=19%  Similarity=0.336  Sum_probs=101.7
Template_Neff=3.200

Q Duck_coa1         9 WQMAIFMGVFSGGG--CVVMYNLMQ-KGFA---RTQYYQQGLEQLNNNPTALEALGAPPLKVHNIRLTDG---------S   73 (139)
Q Consensus         9 ~~~a~~~~v~~~~~--~~~~~~~v~-~~~~---~s~~y~~Al~~~~~~~~~v~~~LG~~pI~~~~~~~~~~~---------~   73 (139)
                      .|.|.|++|+++|+  .++++|.|+ +.|+   .+++|..|+++|.+||+|+++|+ ||++++....-+         +
T Consensus       165 TKTasYlGVIl~GiGVTG~mFYaIFrELFSSkSPNsVYS~ALekC~~DpRV~DaLGe-PIKgYGEeTrRGRR~HVSH~~Y  243 (339)
T UniRef100_A0A0  165 TKTASYLGVILAGIGVTGIMFYAVFRELFSSKSPNSVYSKALEKCINDPRVEDALGE-PIKGYGEETRRGRRRHVSHSEY  243 (339)
Confidence          57899999987665  667777776 4443   457999999999999999996 699886543322         3

Q Duck_coa1        74 NRVDTERAQIKLPVSGTKSAGYLYIDSEMDHSRQCWRLQDVTLKLRD--GQNISVYHSPV  131 (139)
Q Consensus        74 ~~~~~~~~~l~f~V~G~k~~G~v~v~a~r~~~~~~w~~~~l~l~~~~--~~~i~L~~~~~  131 (139)
                      ..+|..|++|+|+|+|++.+|+|++++.+++ .++|+|+++.+++|  .++|.|+|++.
T Consensus       244 eKDGvkHMRMKFYIkG~R~kgTVhLEmken~-~G~yeYRYLfVqlDdyp~~tIvIEDNR~  302 (339)
T UniRef100_A0A0  244 EKDGVKHMRMKFYIKGIRNKGTVHLEMKENE-SGNYEYRYLFVQLDDYPKETIVIEDNRE  302 (339)
Confidence          45688999999999999999999998885 799999999999998  78999999883
```

Figure S10: Alignment output of *COA1* of duck (*Anas platyrhynchos platyrhynchos)* and *TIMM21* of Cayenne tick (*Amblyomma cajennense)* from HHblits.

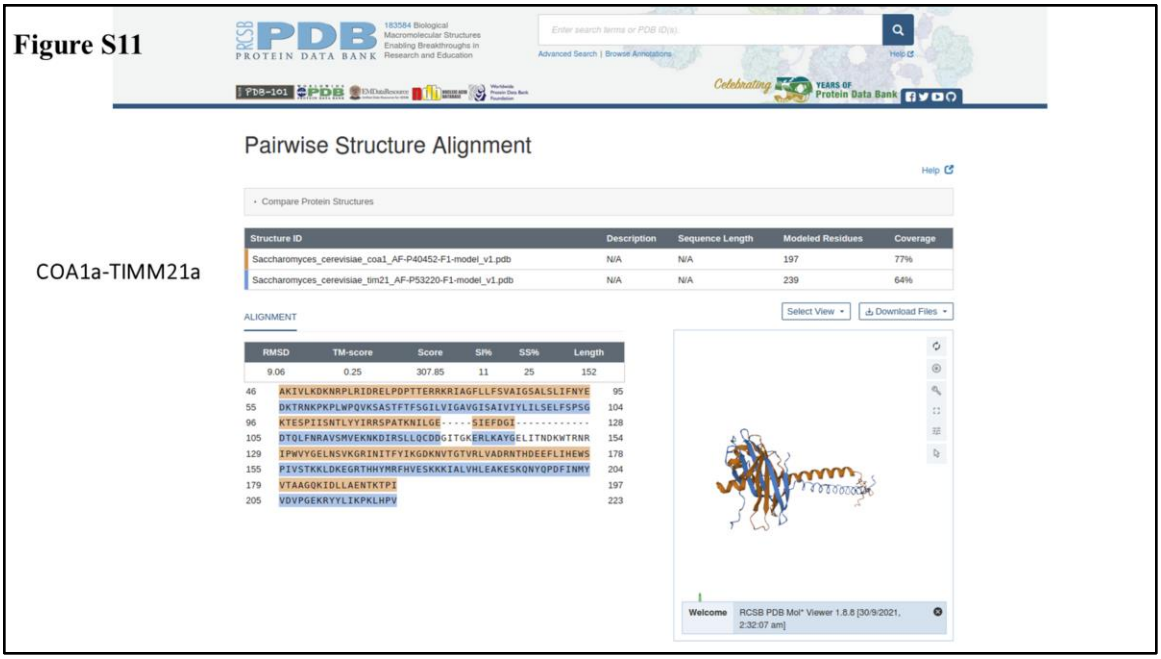

Figure S11: Pairwise comparison of predicted *COA1* (*Saccharomyces cerevisiae*) and *TIMM21* (*Saccharomyces cerevisiae*) structures downloaded from AlphaFold website.

Figure S12

COA1m-COA1a

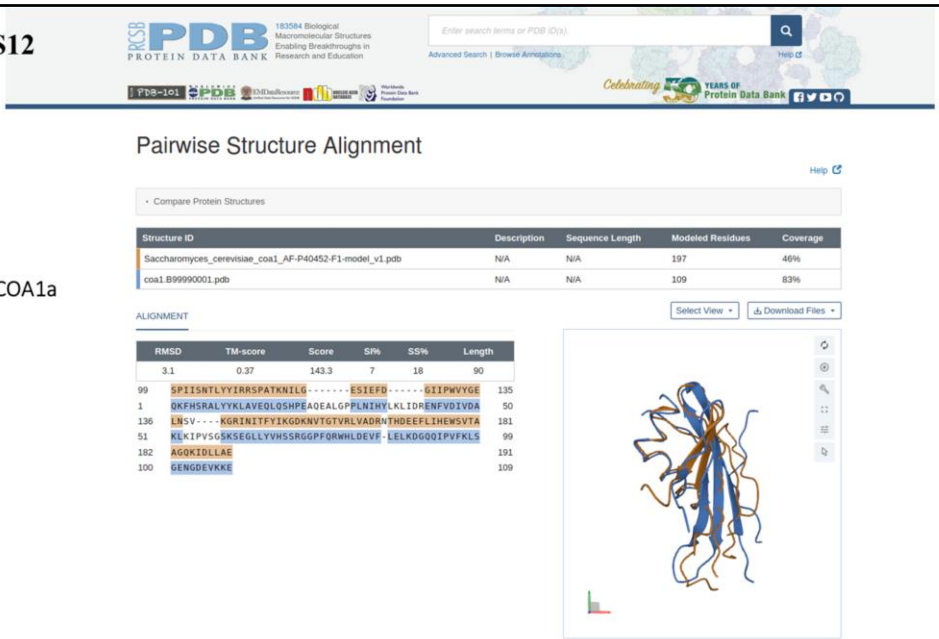

Figure S12: Pairwise alignment of human *COA1* sequence homology modelled using *TIMM21* (*Saccharomyces cerevisiae*) PDB structure and de novo predicted (*Saccharomyces cerevisiae*) *COA1* sturcture downloaded from AlphaFold website.

Figure S13

COA1m-TIMM21p

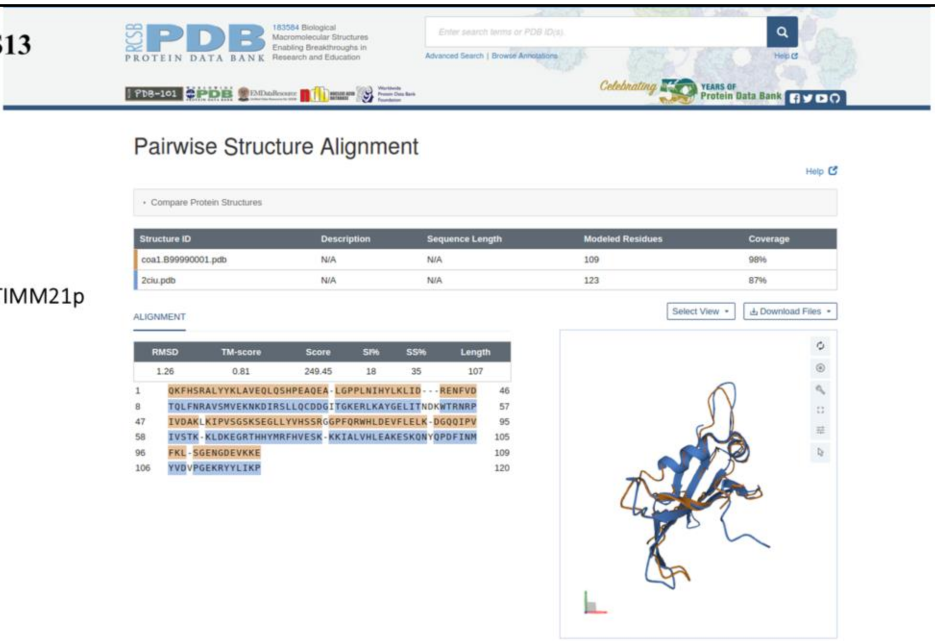

Figure S13: Pairwise alignment of human *COA1* sequence homology modelled using *TIMM21* (*Saccharomyces cerevisiae*) PDB structure and *TIMM21* structure downloaded from rcsb.org protein data bank.

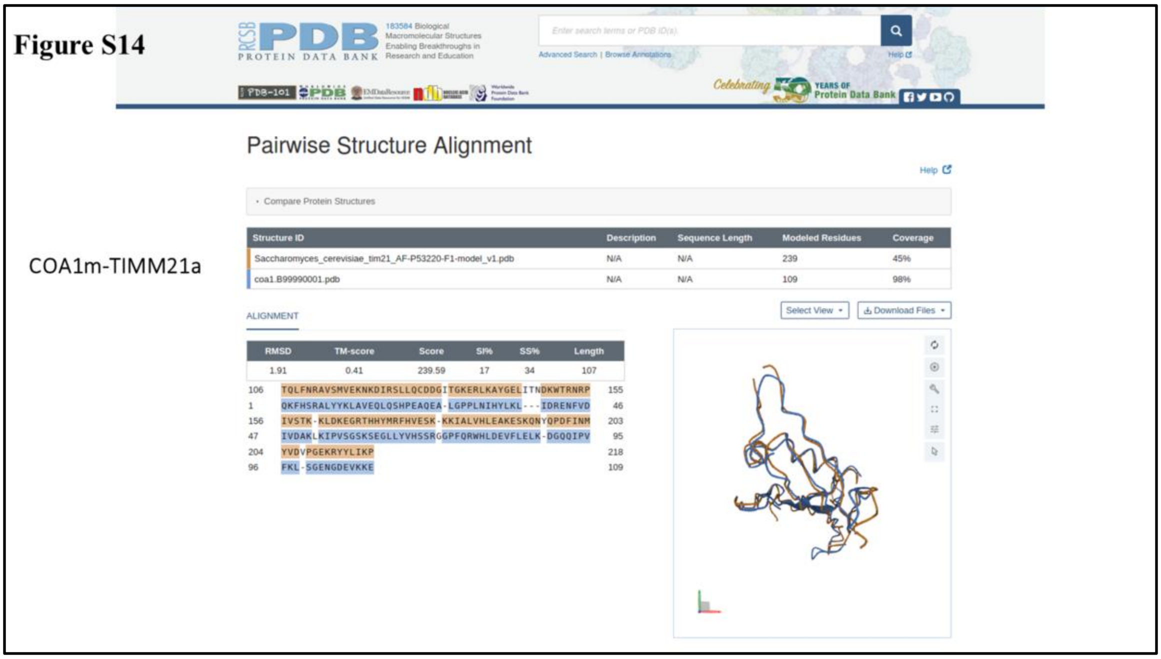

Figure S14: Pairwise alignment of human *COA1* sequence homology modelled using *TIMM21* (*Saccharomyces cerevisiae*) PDB structure and de novo predicted *TIMM21* of yeast (*Saccharomyces cerevisiae)* from AlphaFold website.

Figure S15

COA1a-TIMM21p

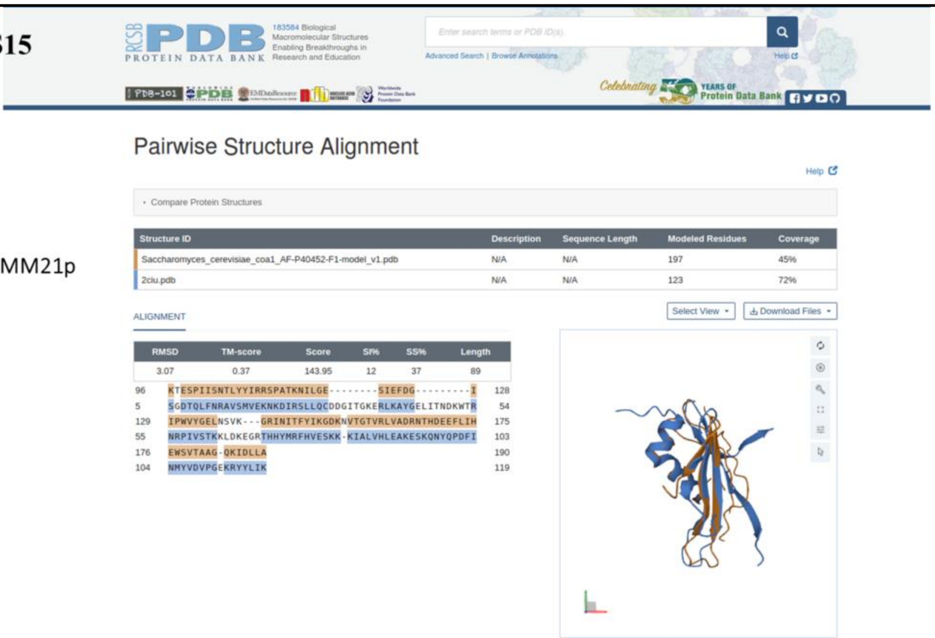

Figure S15: Pairwise structure alignmnet of de novo predicted *COA1* structure from AlphaFold website and *TIMM21* PDB structure from rcsb.org protein data bank of yeast (*Saccharomyces cerevisiae)*.

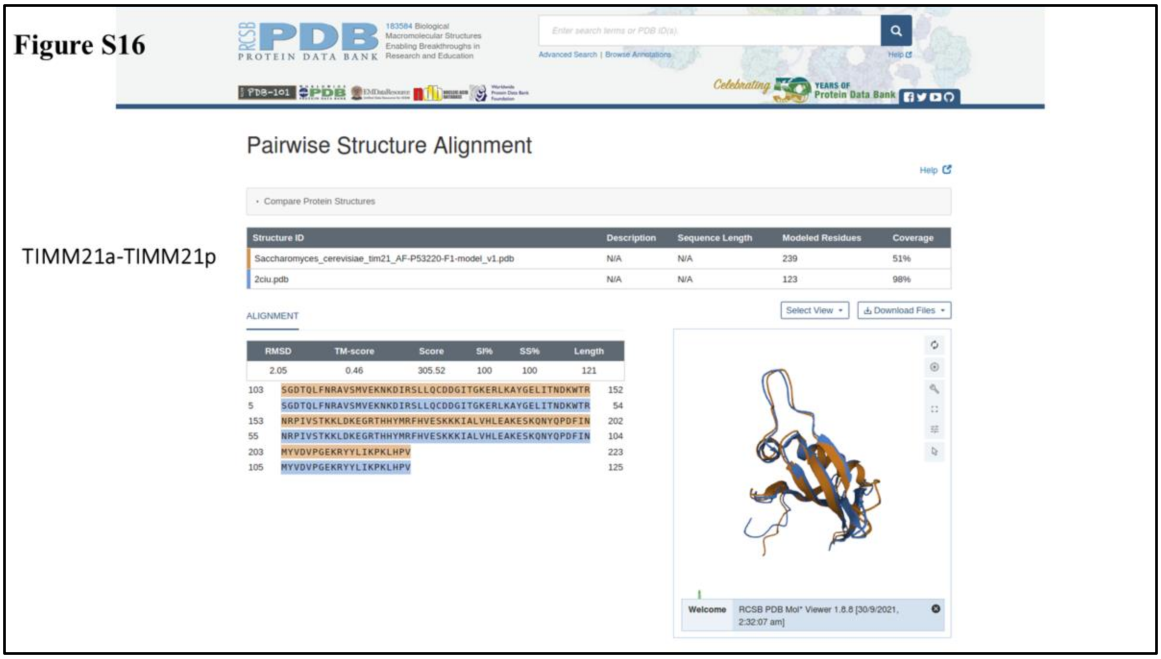

Figure S16: Pairwise structure alignment of *TIMM21* de novo predicted structure of yeast (*Saccharomyces cerevisiae)* downloaded from AlphaFold and *TIMM21* PDB structure from rcsb.org protein data bank of yeast (*Saccharomyces cerevisiae)*.

Figure S17 human Haplotype

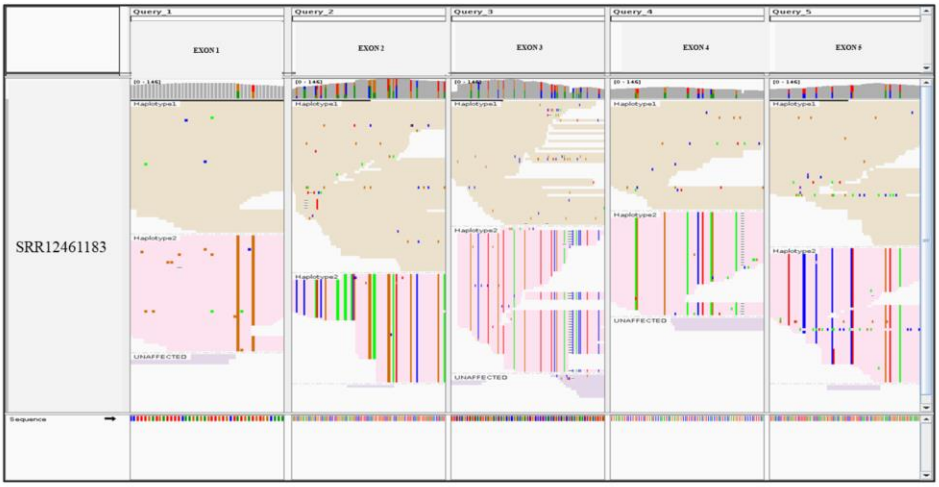

human

Figure S17: Comparison of haplotypes of *COA1* gene inferred based on sequencing reads visualized in IGV browser. Two haplotypes of *COA1* can be seen in short-read sequencing data of human (*Homo sapiens*) and correspond to the functional and pseudogene copies.

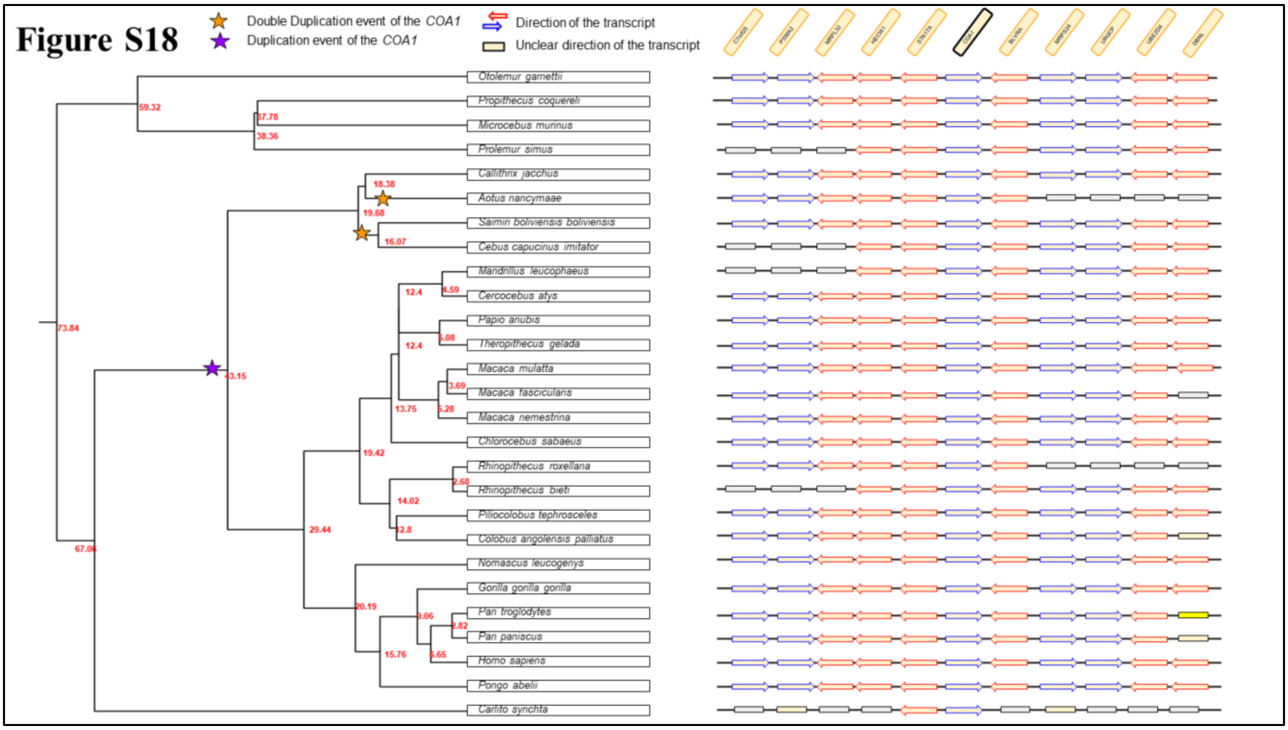

Figure S18: The duplication events of *COA1* gene in primates and their synteny with the time calibrated phylogenetic tree from TimeTree website. The first duplication occurred around 43 million year ago (MYA) and the double duplication event occurred independently at around 16 and 18 MYA as depicted. The direction of gene transcription is depicted using red and blue arrows relative to the *COA1* gene for consistency across species. Gray boxes represent the genes which are located on short scaffolds and their orientation could not be verified.

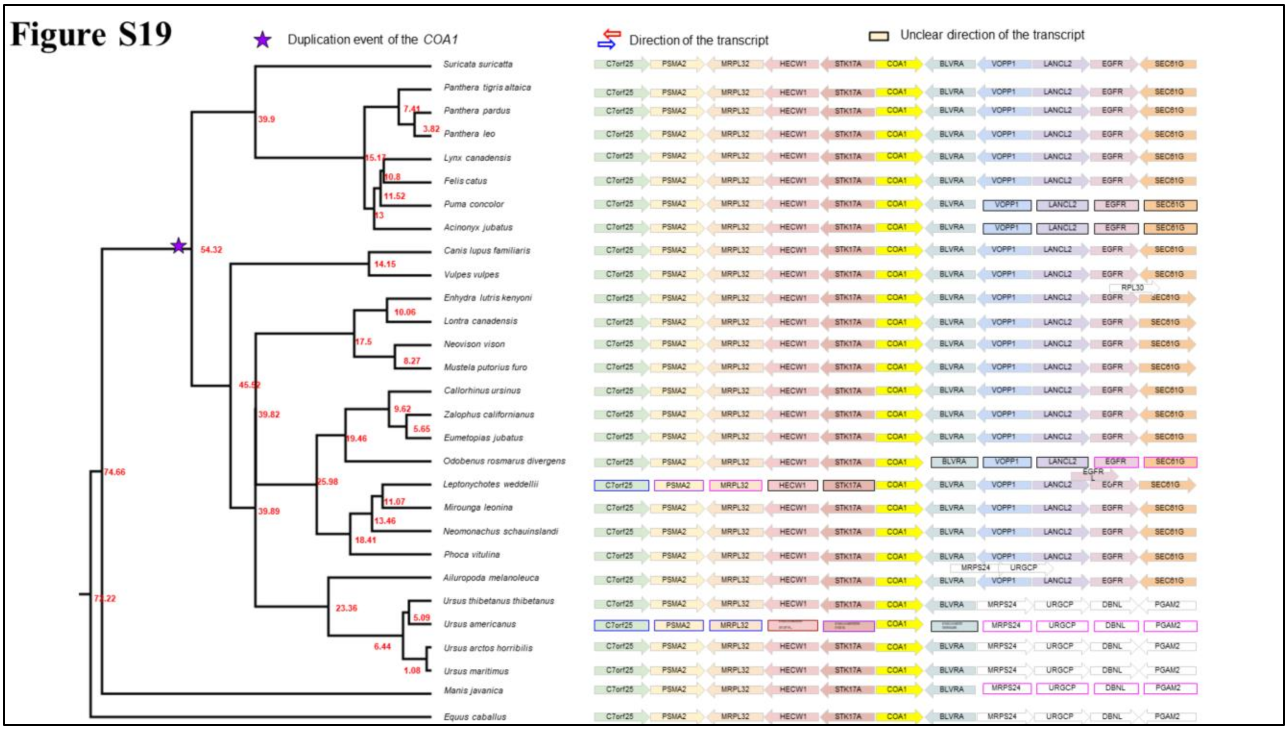

Figure S19: The independent duplication event of *COA1* gene in carnivores and their synteny with the time calibrated phylogenetic tree from TimeTree website. The duplication occurred around 54 million year ago (MYA) as depicted. The direction of gene transcription is depicted using red and blue arrows relative to the *COA1* gene for consistency across species. Gray boxes represent the genes which are located on short scaffolds and their orientation could not be verified.

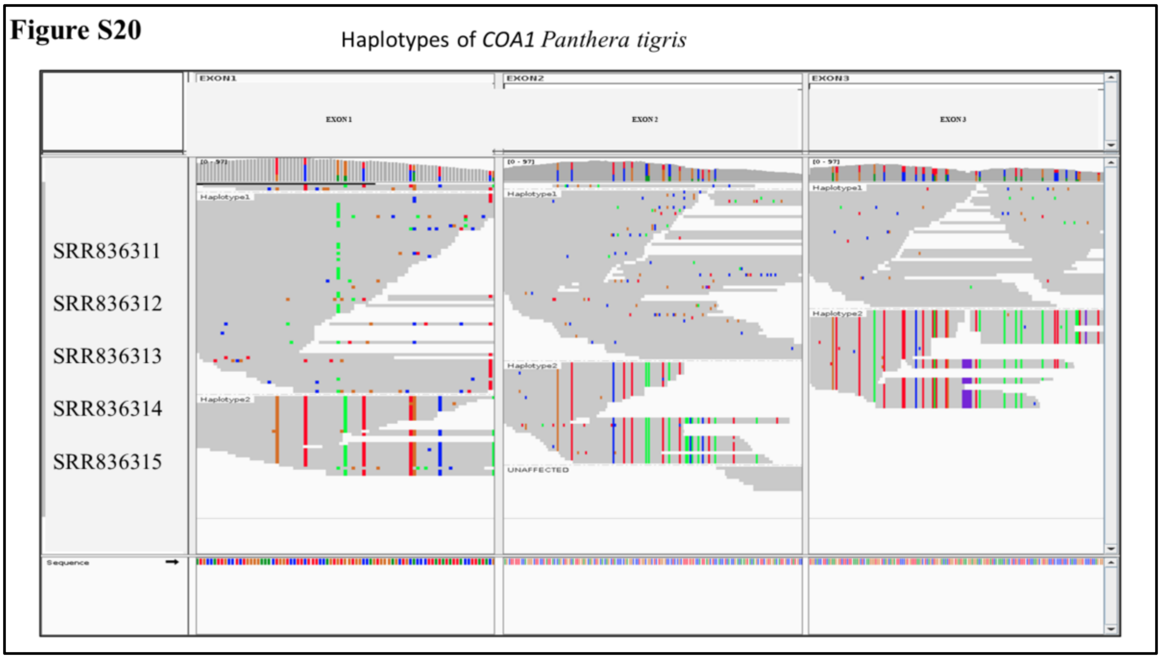

Figure S20: Two haplotypes of exon-1 to exon-4 of *COA1* in tiger (*Panthera tigris*).

Figure S21

Haplotypes of *COA1 Equus caballus*

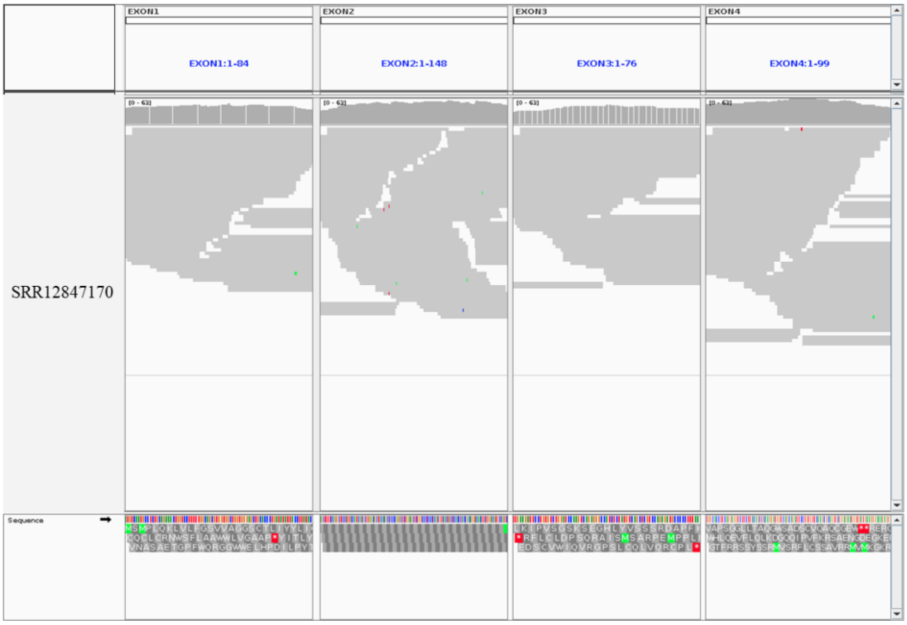

Figure S21: Single copy of *COA1* gene in horse (*Equus caballus)* :Haplotype in exon-1, exon-2, exon-3 and exon-4

Figure S22

Haplotypes of *COA1 Manis javanica*

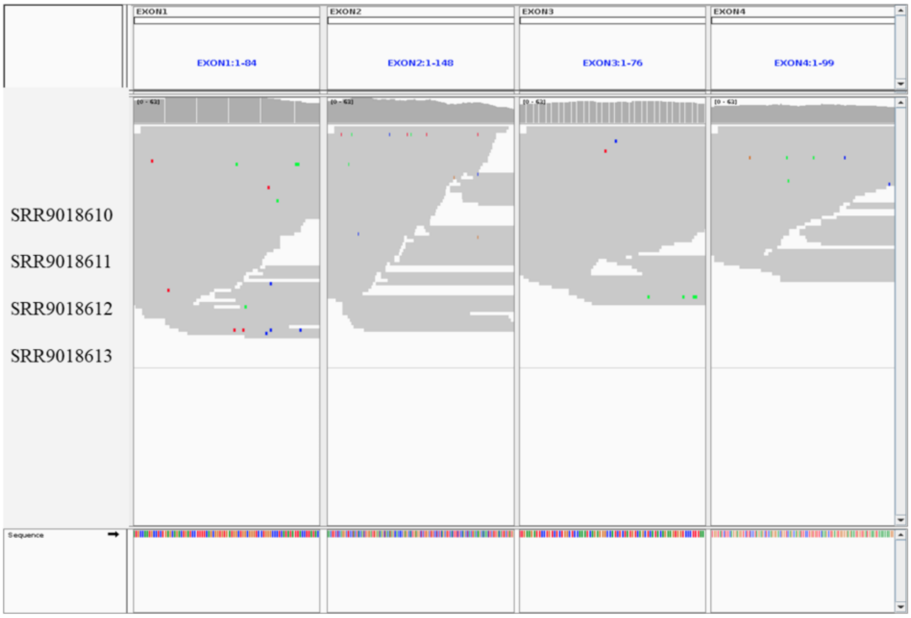

Figure S22: Single copy of *COA1* gene in pangolin (*Manis javanica)*: Haplotype in exon-1, exon-2, exon-3 and exon-4.

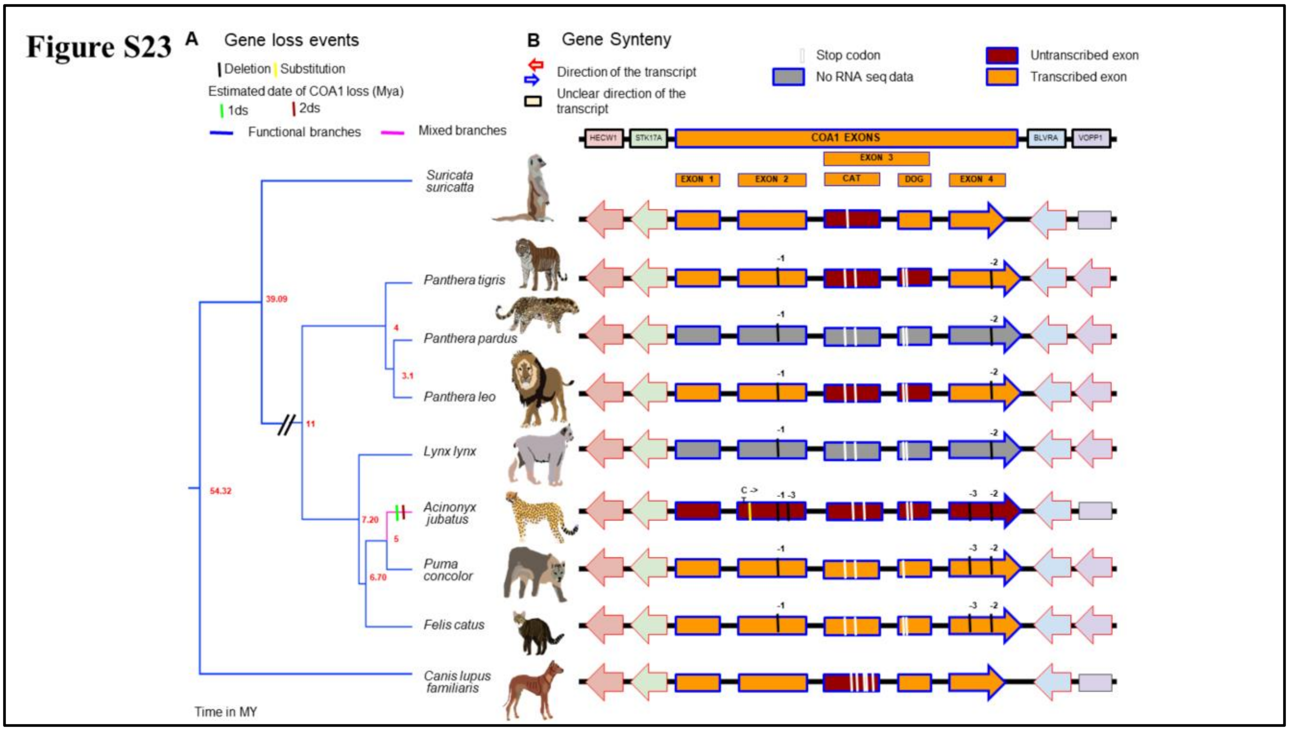

Figure S23: Loss of *COA1* gene in felids. (**A**) Gene loss event in cheetah (*Acinonyx jubatus*) besides a time-calibrated phylogenetic tree downloaded from the TimeTree website. (**B**) Gene order in the genomic region flanking the *COA1* gene and its exons in genomes. Red and blue arrows depict the direction of gene transcription relative to the *COA1* gene for consistency across species. Gray boxes represent the genes located on short scaffolds with unknown orientation.

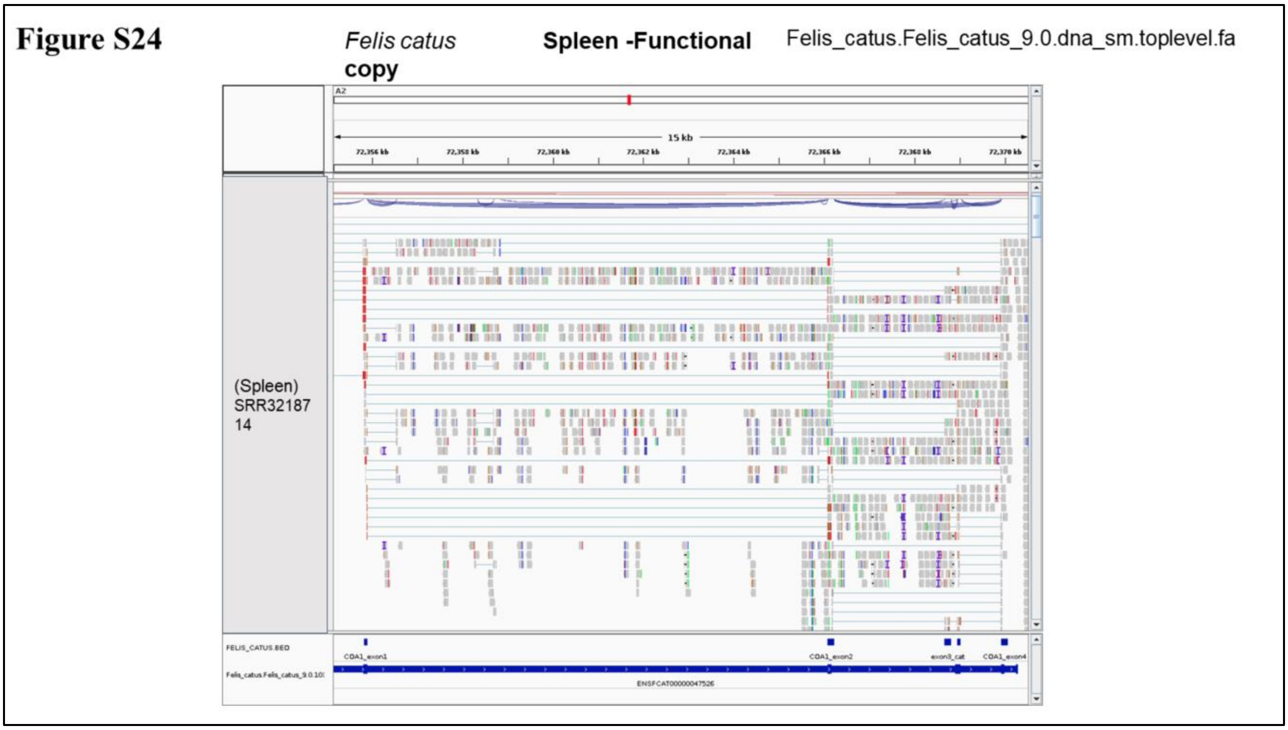

Figure S24: Screenshot of RNA-seq reads aligned to each of the putative locations of the three *COA1* exons in cat (*Felis catus*). Gray boxes represent reads aligned to the genome and thin lines connecting these boxes represent the spliced read alignments. Datasets from spleen (SRR3218714) have been mapped to the Felis_catus.Felis_catus_9.0.dna_sm.toplevel.fa. Locations of each of the exons are depicted as bed record in the bottom row.

Figure S25

Felis catus copy **Spleen -Functional** Felis_catus.Felis_catus_9.0.dna_sm.toplevel.fa

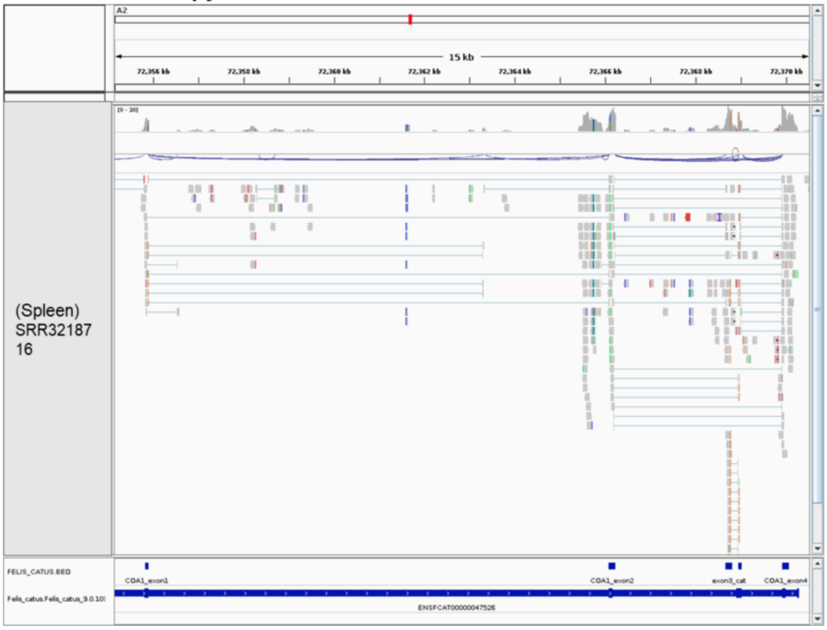

Figure S25: Screenshot of RNA-seq reads aligned to each of the putative locations of the three *COA1* exons extra 3rd exon from *Puma concolor* region in cat (*Felis catus*). Gray boxes represent reads aligned to the genome and thin lines connecting these boxes represent the spliced read alignments. Datasets from spleen (SRR3218716) have been mapped to the Felis_catus.Felis_catus_9.0.dna_sm.toplevel.fa. Locations of each of the exons are depicted as bed record in the bottom row.

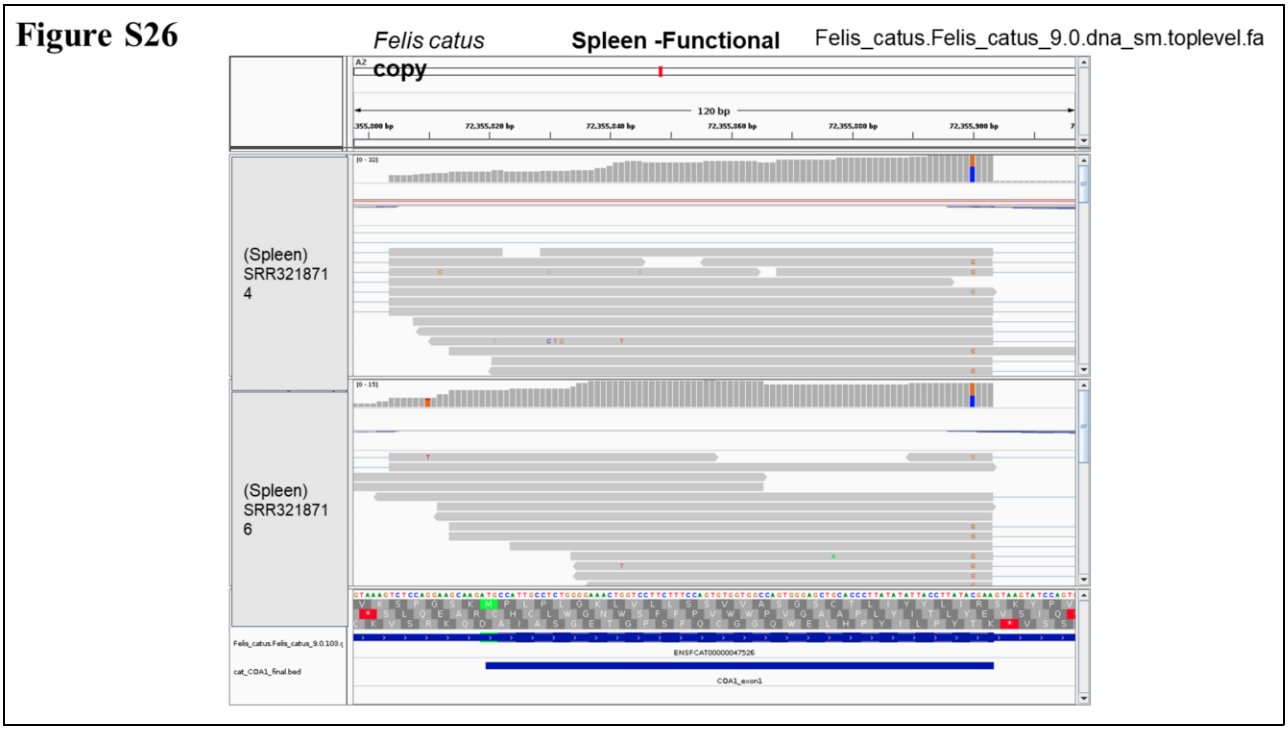

Figure S26: Zoomed view of cat *COA1* exon-1 shown in **Supplementary Fig. S26**.

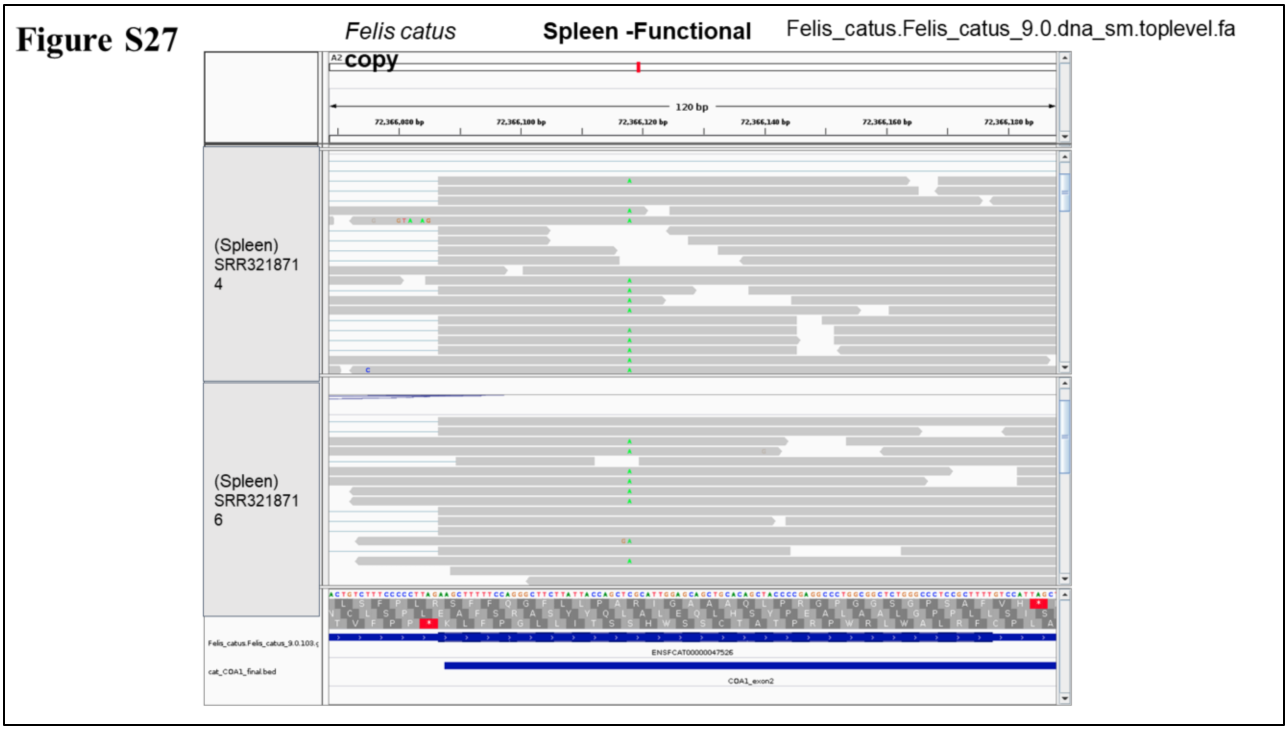

Figure S27: Zoomed view of cat *COA1* exon-2 shown in **Supplementary Fig. S26**.

.

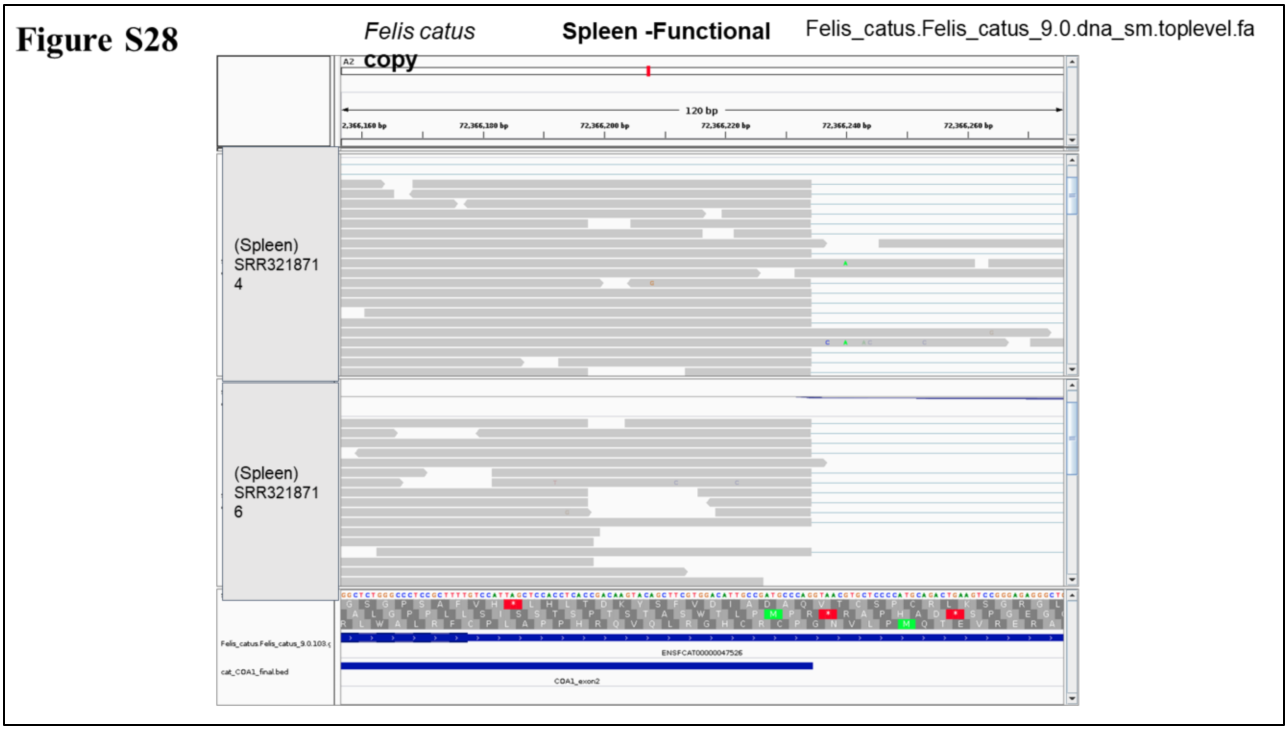

Figure S28: Zoomed view of cat *COA1* exon-2 shown in **Supplementary Fig. S26**.

.

Figure S29

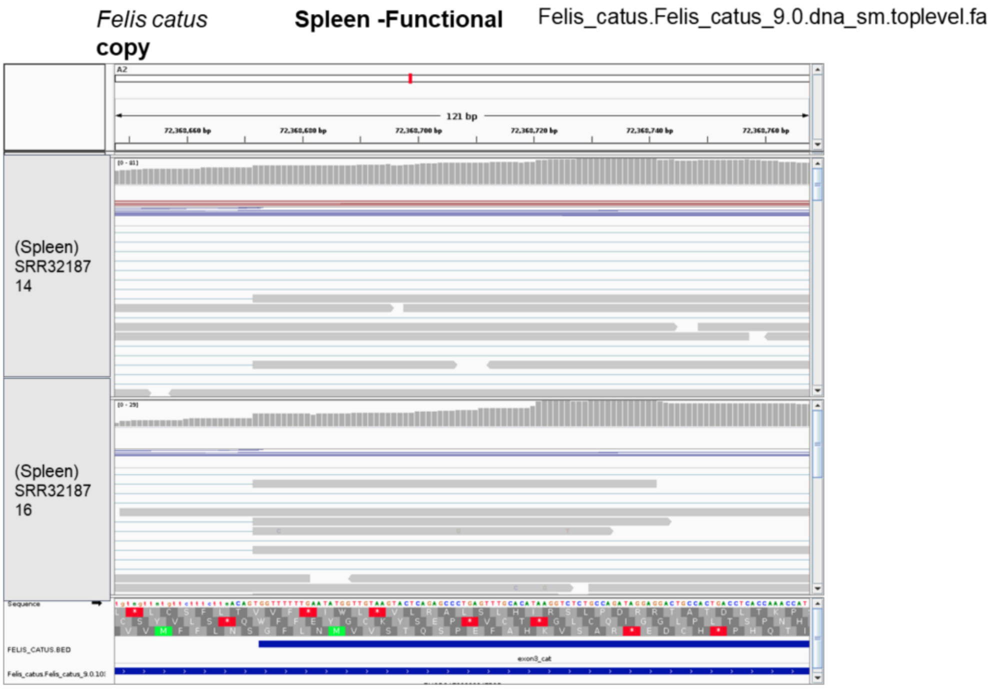

Figure S29: Zoomed view of cat *COA1* exon-3 shown in **Supplementary Fig. S26**.

.

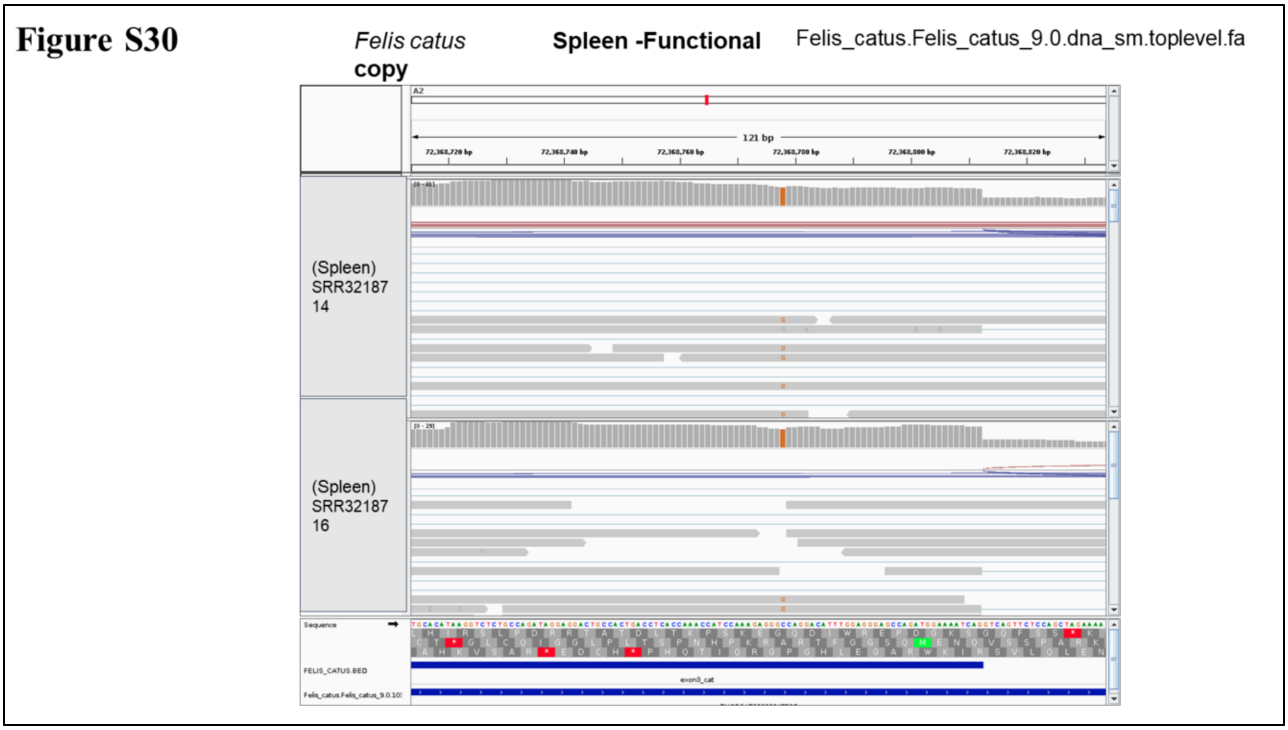

Figure S30: Zoomed view of cat *COA1* exon-3 shown in **Supplementary Fig. S26**.

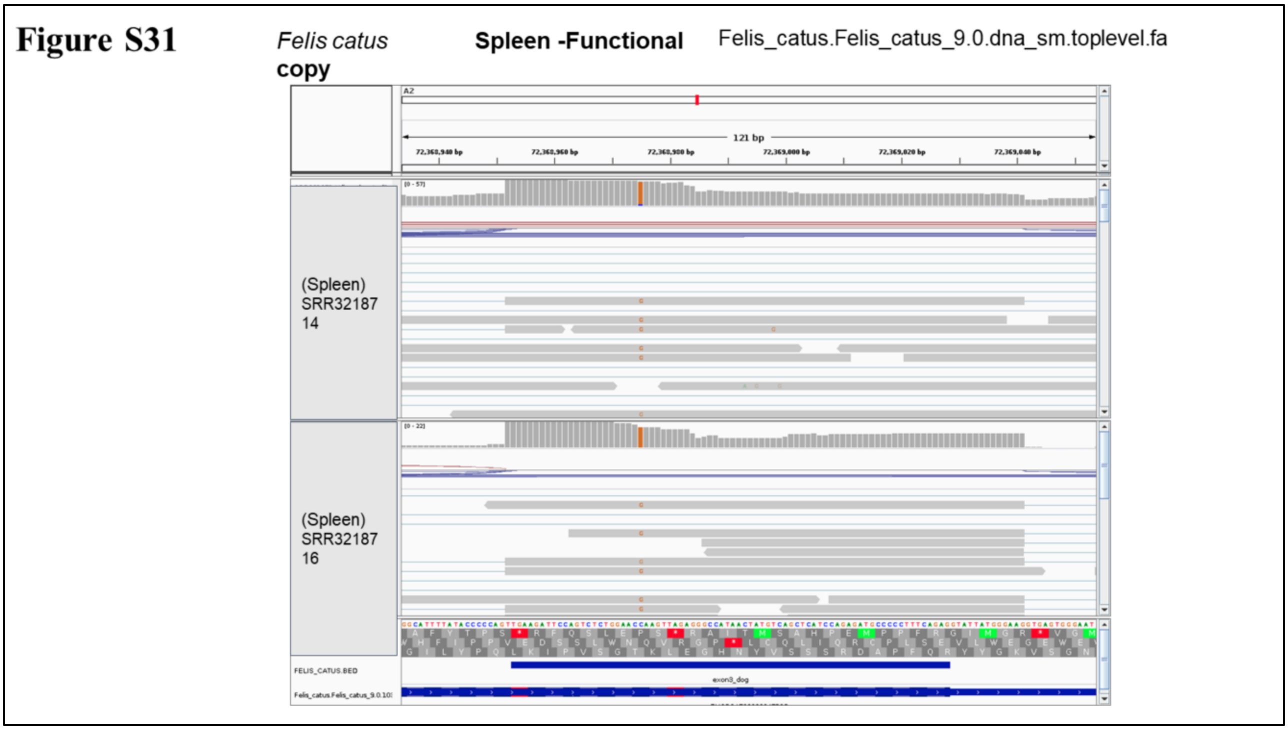

Figure S31: Zoomed view of dog *COA1* exon-3 shown in **Supplementary Fig. S26**.

Figure S32

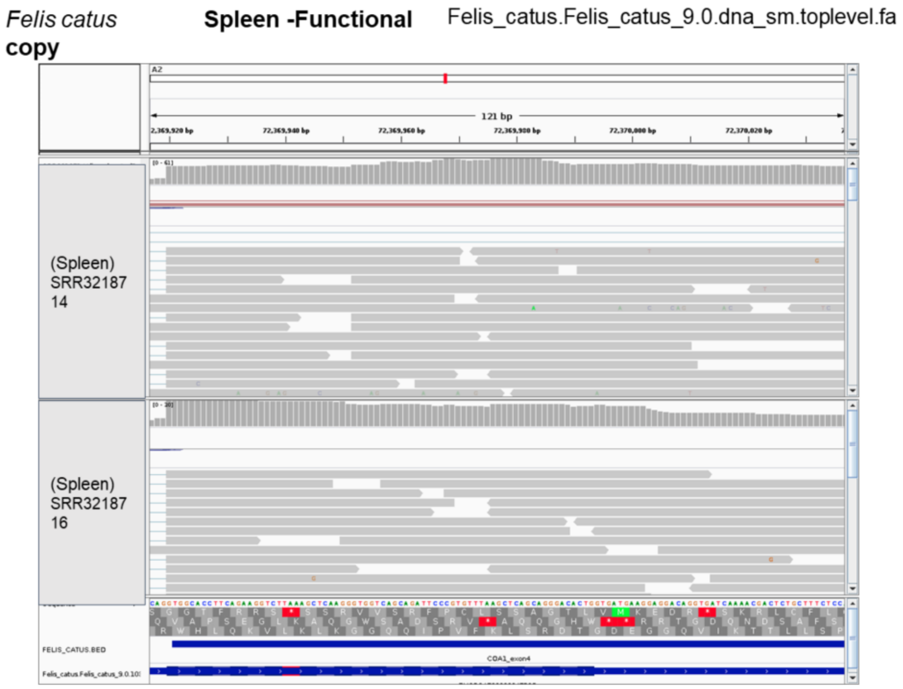

Figure S32: Zoomed view of cat *COA1* exon-4 shown in **Supplementary Fig. S26**.

Figure S33

Felis catus **copy** **Spleen -Functional** Felis_catus.Felis_catus_9.0.dna_sm.toplevel.fa

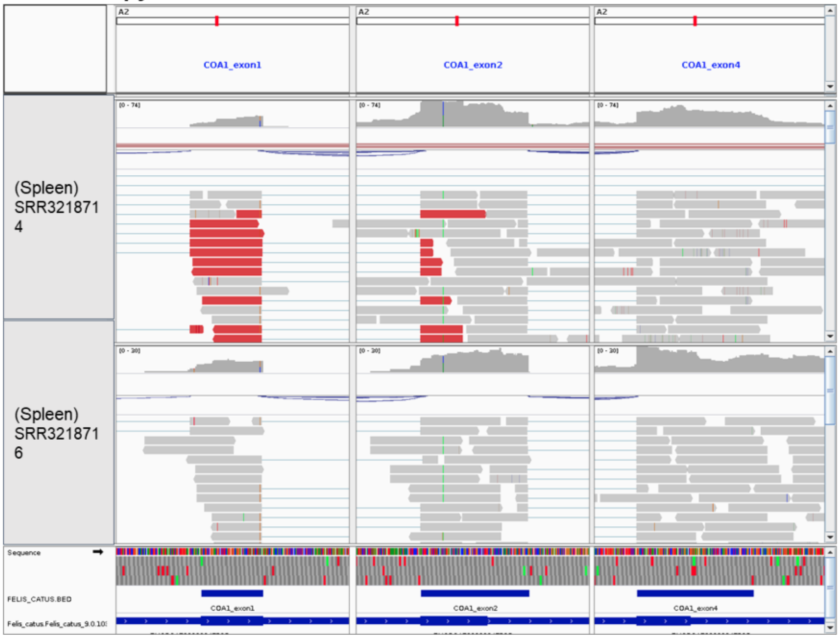

Figure S33: Zoomed view of all three cds forming exons shown in **Supplementary Fig. S26**.

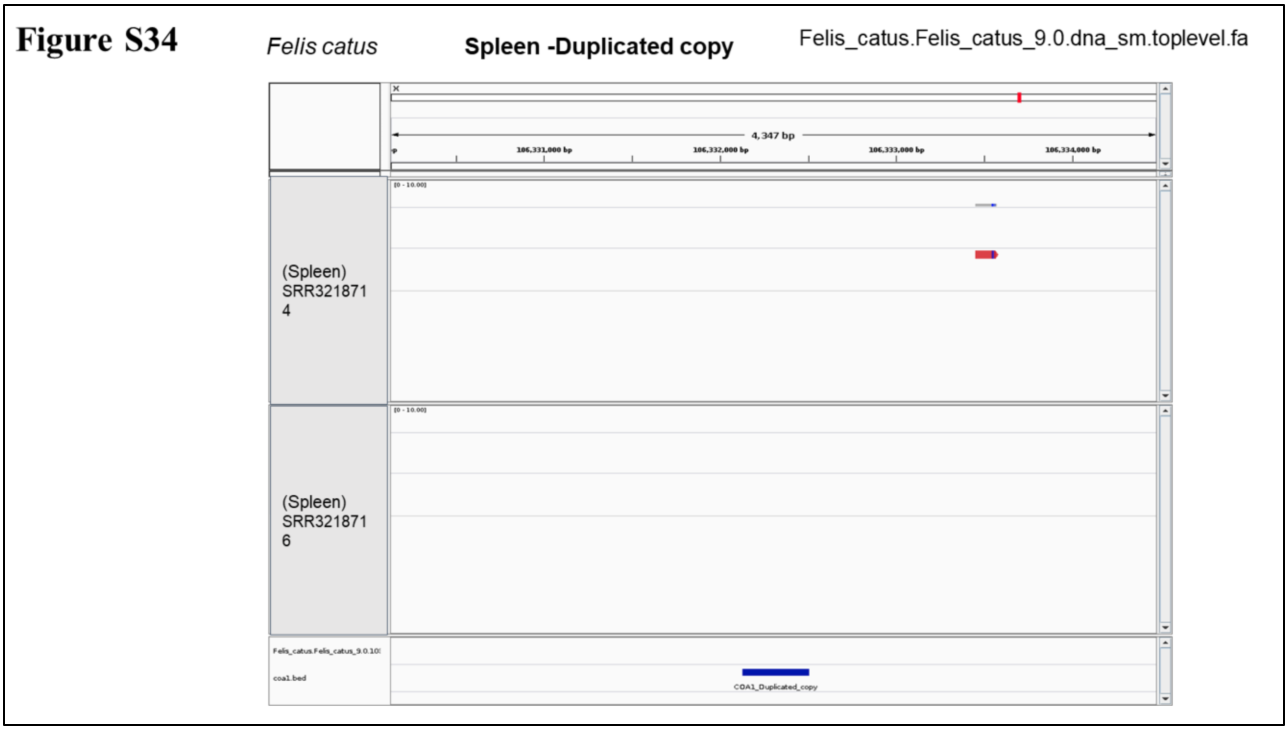

Figure S34: Screenshot of RNA-seq reads aligned to each of the putative locations of the *COA1* duplicated copy in cat (*Felis catus*). Datasets from spleen (SRR3218714 and SRR3218716) have been mapped to the Felis_catus.Felis_catus_9.0.dna_sm.toplevel.fa. Locations of of the *COA1* gene duplicated copy is depicted as bed record in the bottom row.

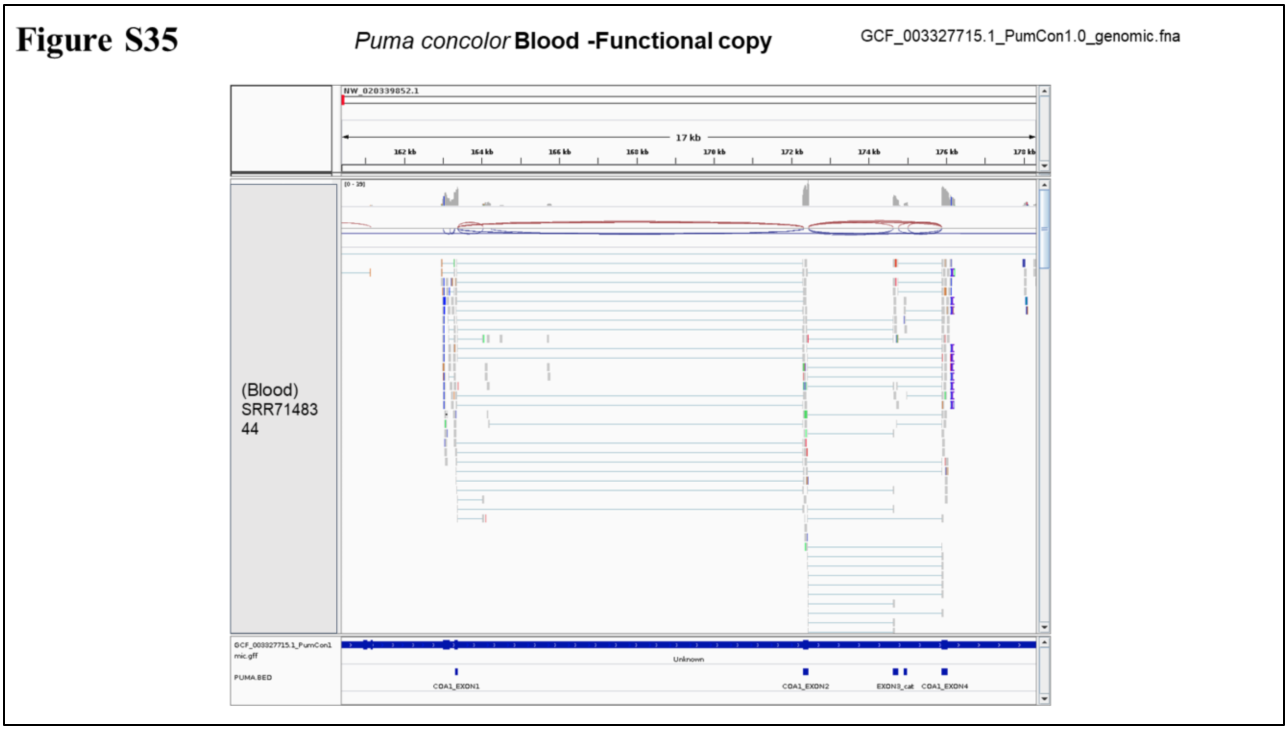

Figure S35: Screenshot of RNA-seq reads aligned to each of the putative locations of the all *COA1* exons in puma (*Puma concolor*). Gray boxes represent reads aligned to the genome and thin lines connecting these boxes represent the spliced read alignments. Datasets from blood (SRR7148344) have been mapped to the GCF_003327715.1_PumCon1.0_genomic.fna. Locations of each of the exons are depicted as bed record in the bottom row.

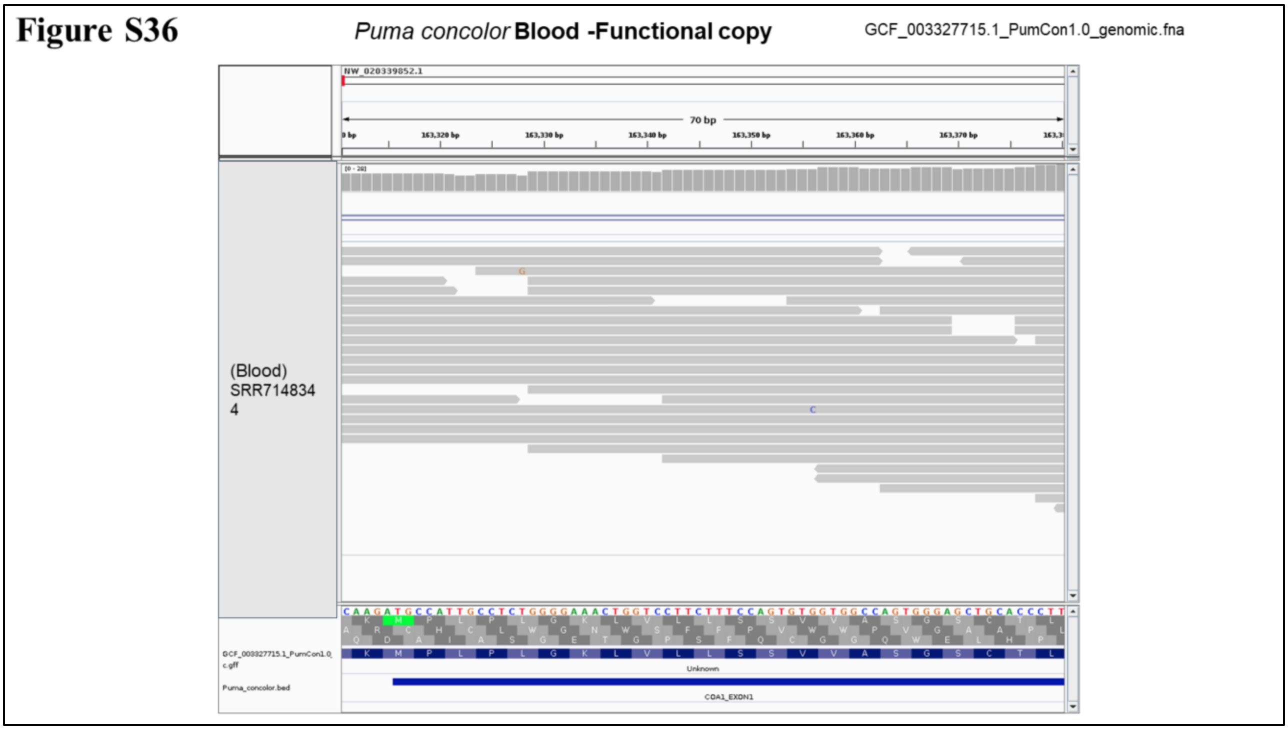

Figure S36: Zoomed view of puma *COA1* exon-1 shown in **Supplementary Fig. S35**.

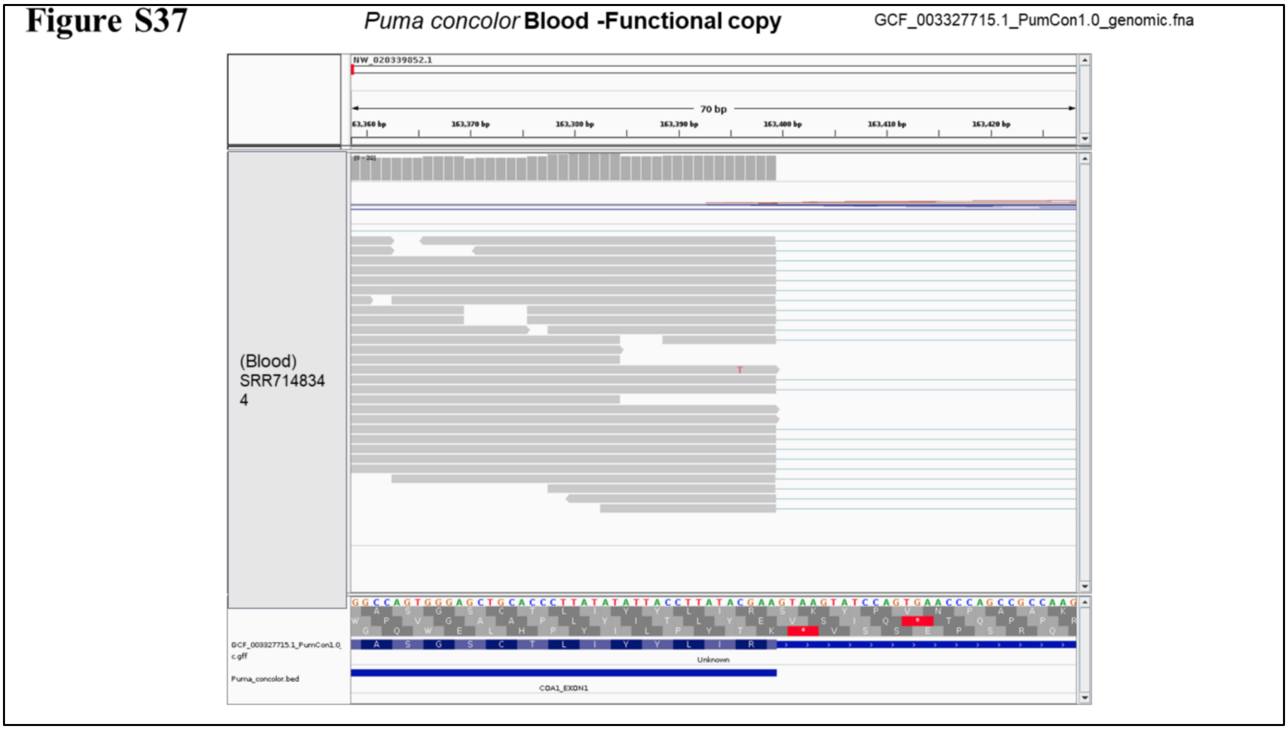

Figure S37: Zoomed view of puma *COA1* exon-1 shown in **Supplementary Fig. S35.**

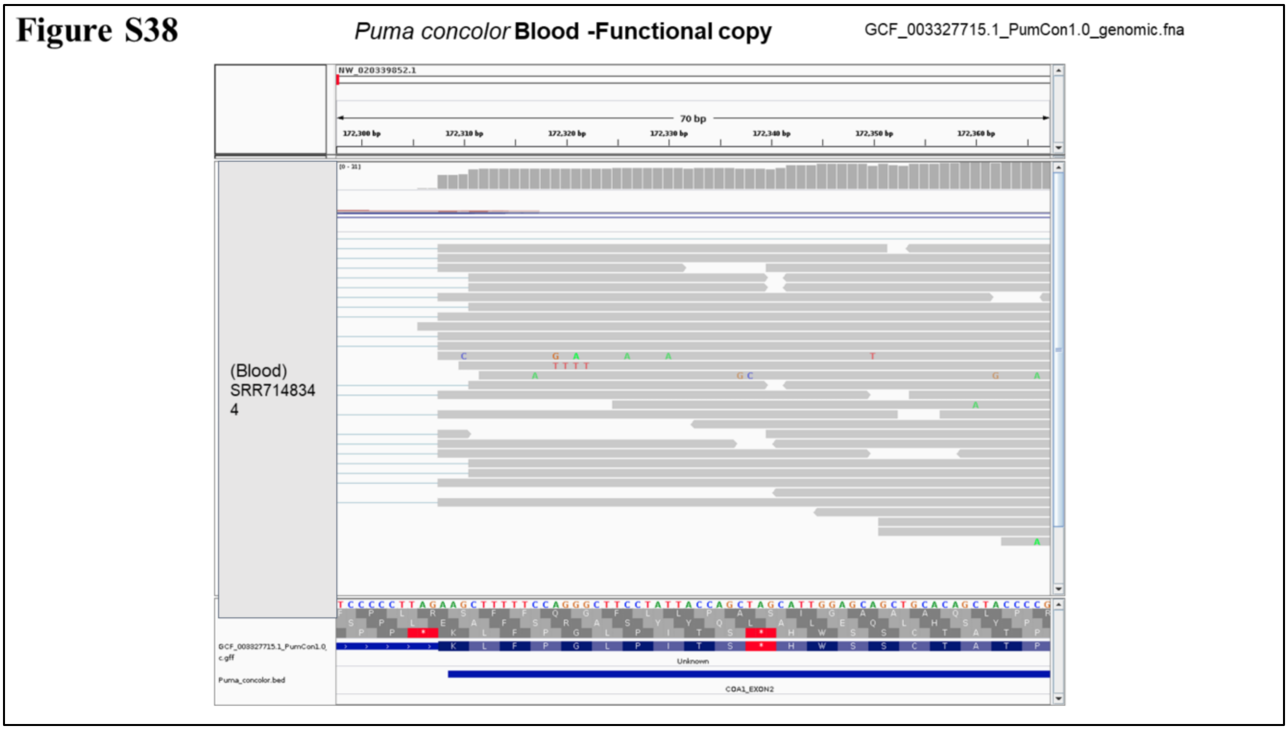

Figure S38: Zoomed view of puma *COA1* exon-2 shown in **Supplementary Fig. S35.**

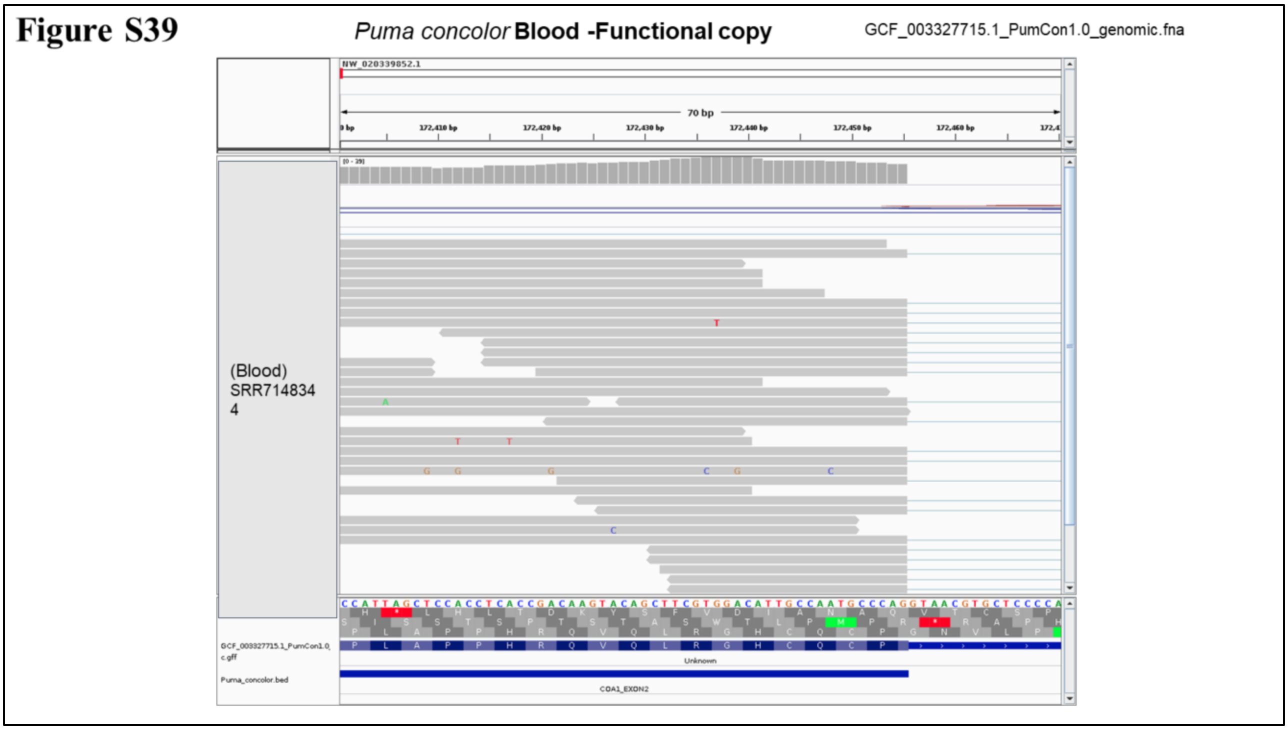

Figure S39: Zoomed view of puma *COA1* exon-2 shown in **Supplementary Fig. S35.**

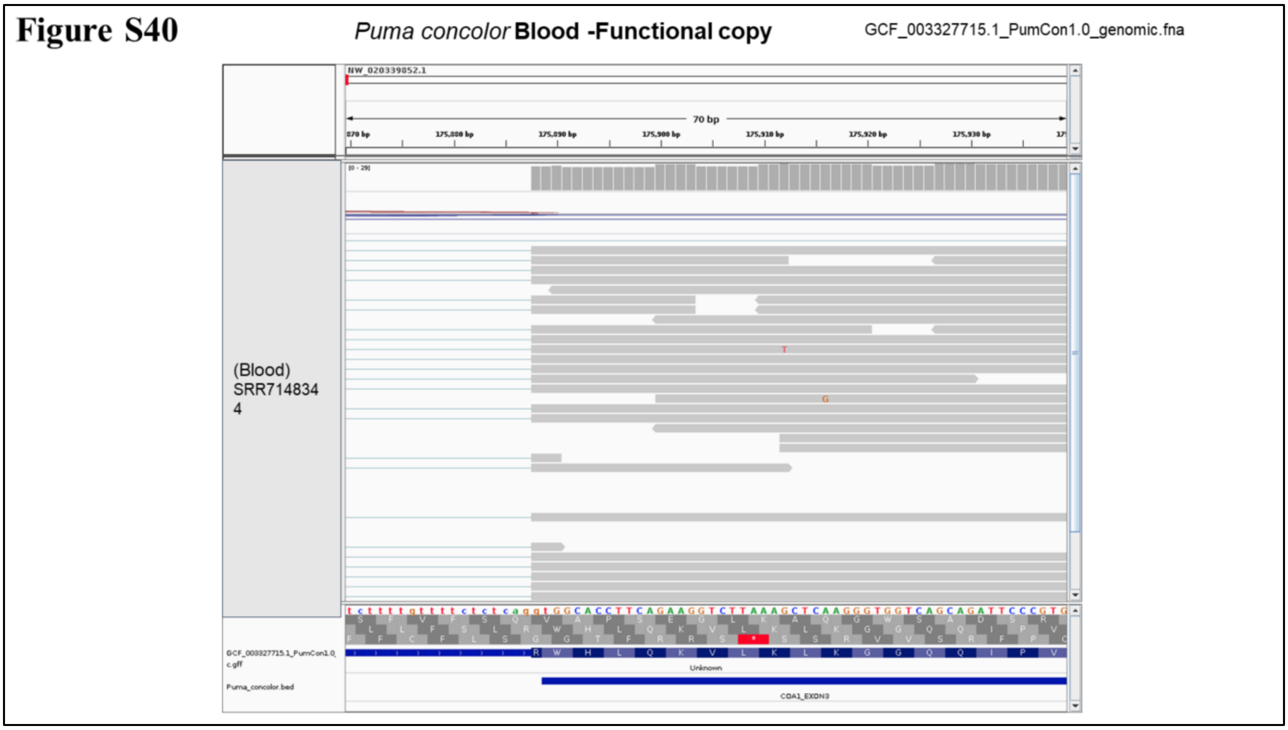

Figure S40: Zoomed view of puma *COA1* exon-3 shown in **Supplementary Fig. S35.**

Figure S41 *Puma concolor* **Blood -Functional copy** GCF_003327715.1_PumCon1.0_genomic.fna

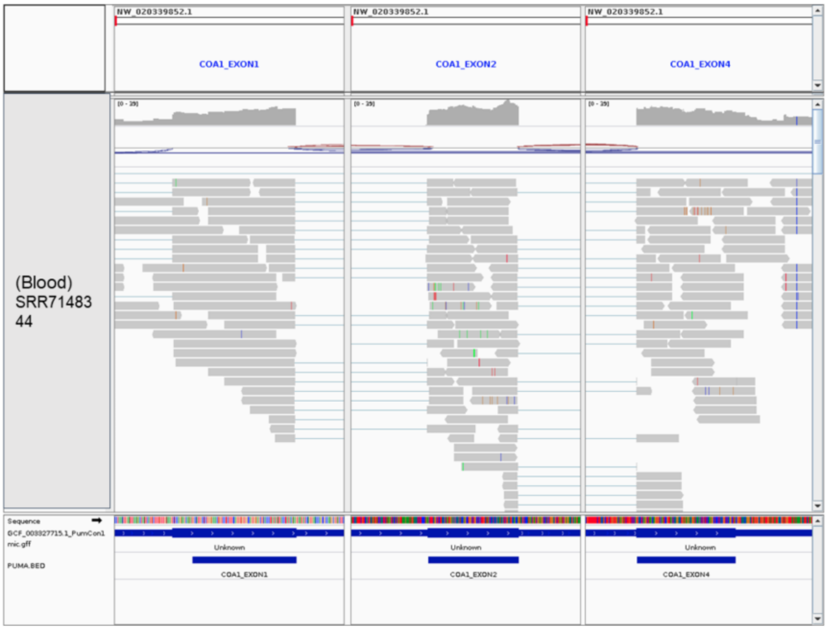

Figure S41: Zoomed view of all three cds coding exons shown in **Supplementary Fig. S35.**

Figure S42 *Puma concolor* **Blood -Duplicated copy** GCF_003327715.1_PumCon1.0_genomic.fna

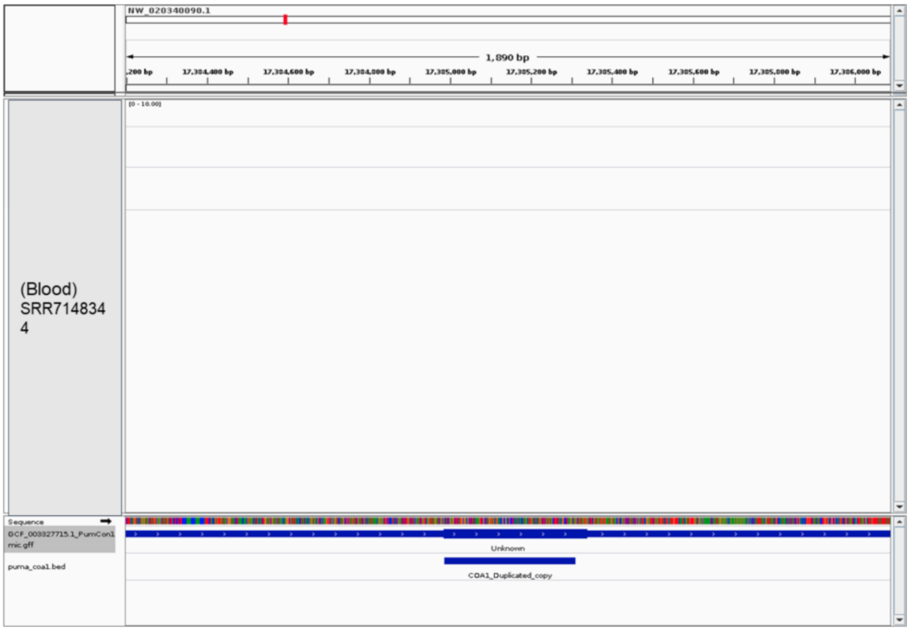

Figure S42: Screenshot of RNA-seq reads aligned to each of the putative locations of the *COA1* duplicated copy in puma (*Puma concolor*). Datasets from blood (SRR7148344) have been mapped to the GCF_003327715.1_PumCon1.0_genomic.fna. Locations of of the *COA1* gene duplicated copy is depicted as bed record in the bottom row.

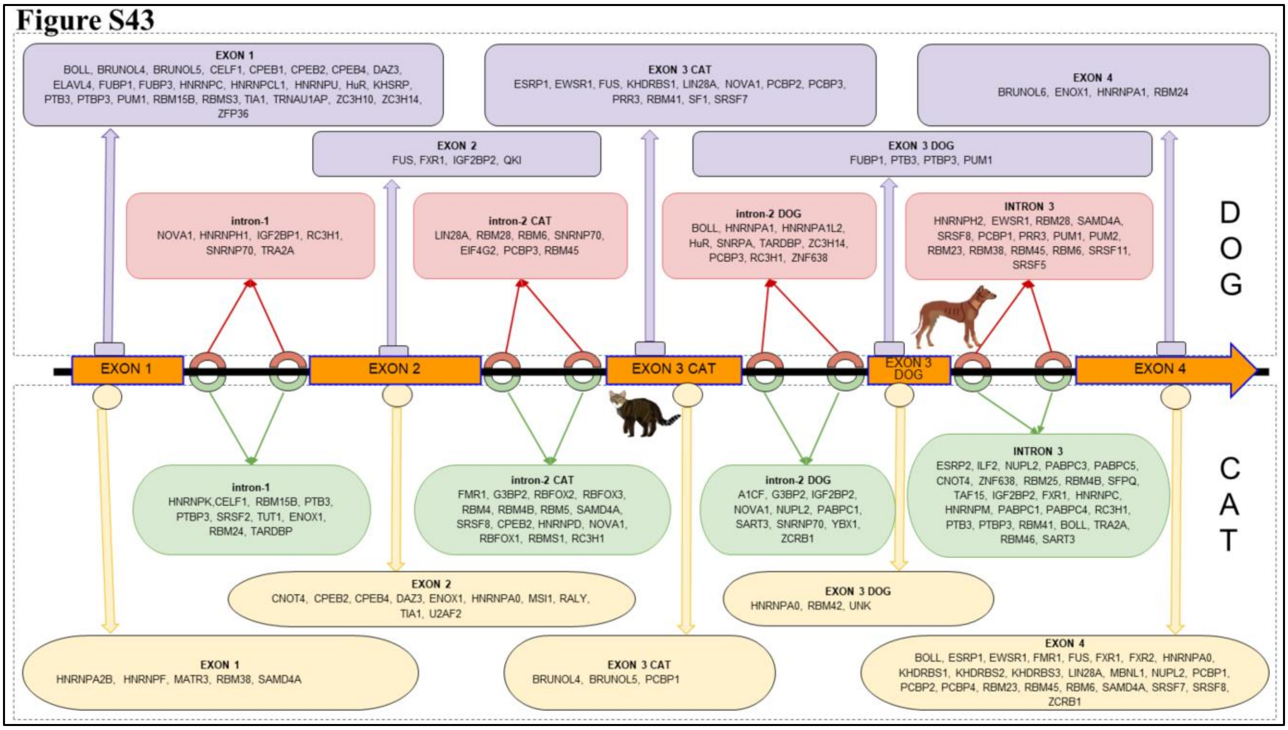

Figure S43: Different types of enhancers and silencers for different motifs of exons and introns of *COA1* gene unique in *Canis lupus familiaris* and *Felis catus* downloaded from RBPmap website (**Supplementary Table S25** and **S26**).

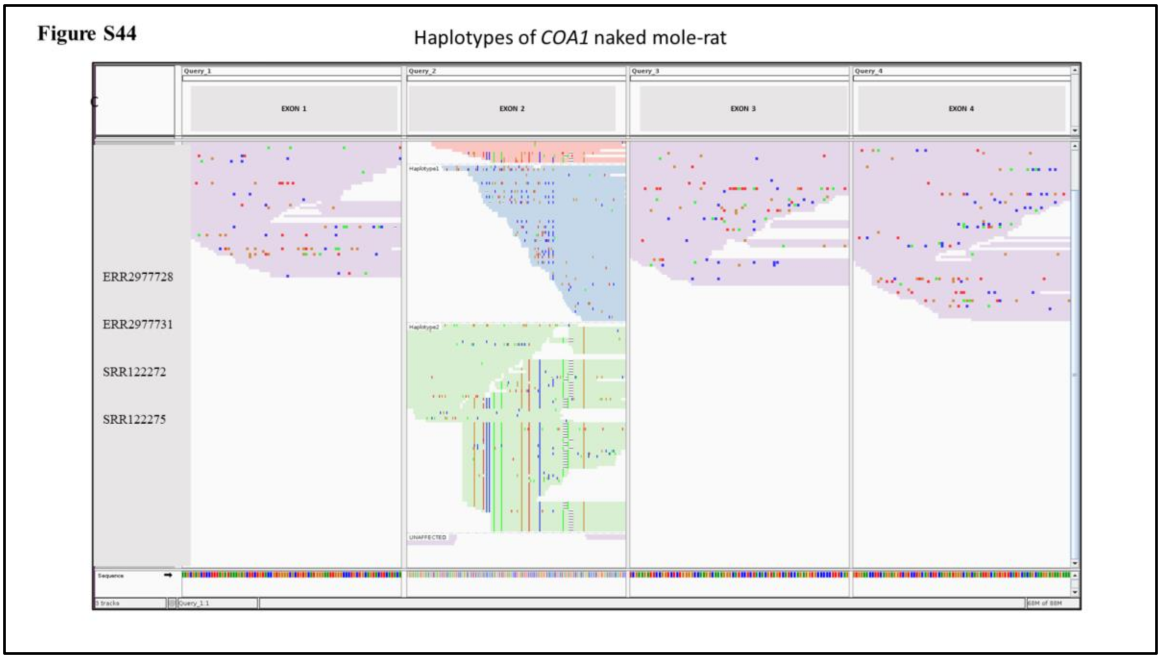

Figure S44: Two haplotypes in exon two and one haplotype of remaining exons of *COA1* in naked mole-rat (*Heterocephalus glaber*).

Figure S45

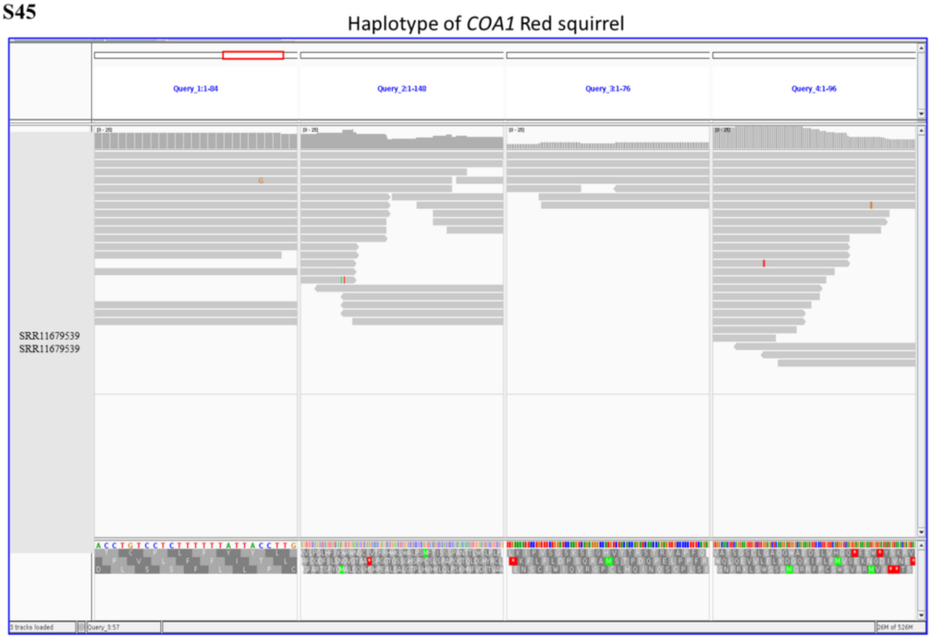

Figure S45: The single haplotype of *COA1* in red squirrel (*Sciurus vulgaris*).

Figure S46

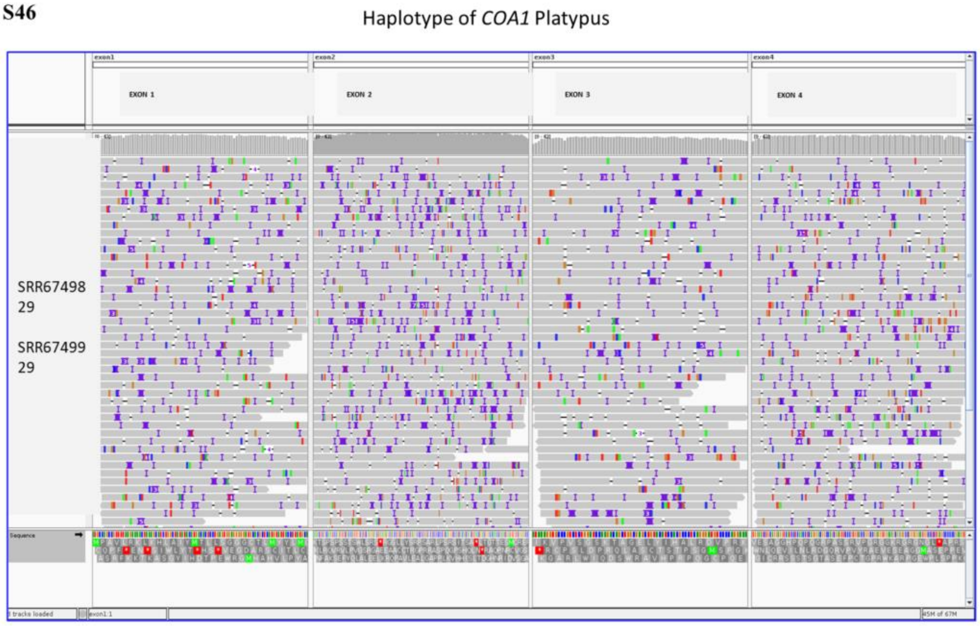

Figure S46: The single haplotype of *COA1* in the platypus (*Ornithorhynchus anatinus*).

Figure S47

Haplotype of *COA1* chicken

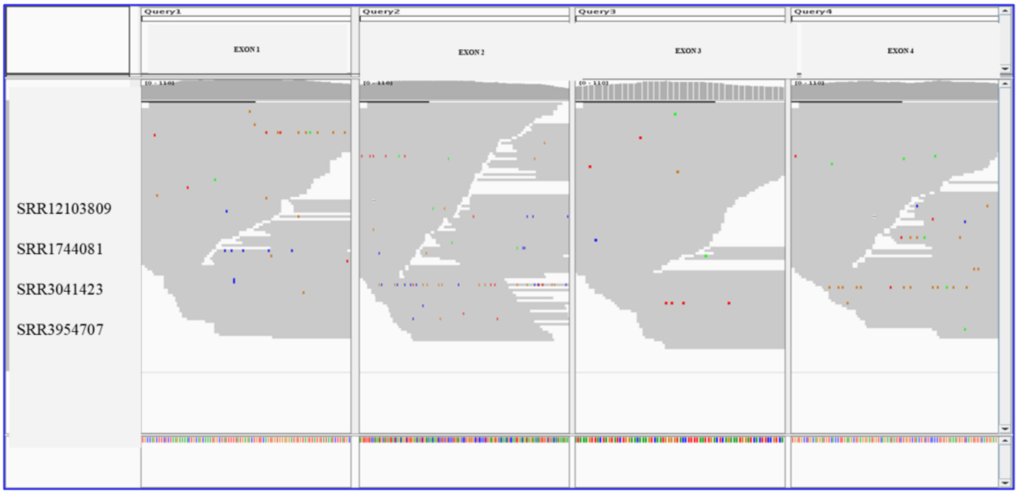

Figure S47: The single haplotype of *COA1* gene in chicken (*Gallus gallus*).

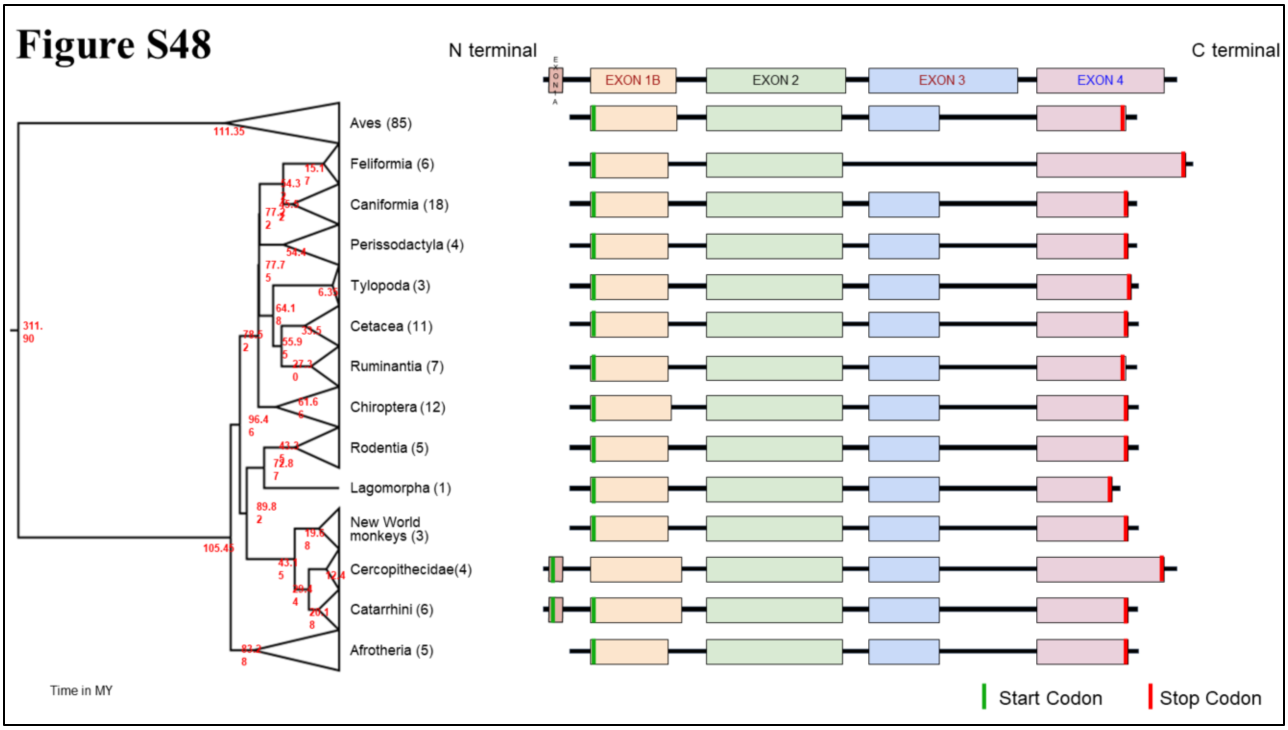

Figure S48: Overall length variation of *COA1* transcript with their start and stop codon across various groups. The numbers in brackets represents the number of species clubbed together in the time calibrated phylogenetic tree taken from TimeTree website.

Figure S49 *Acinonyx jubatus*(cheetah) *COA1* haplotype 1 figure of Illumina database

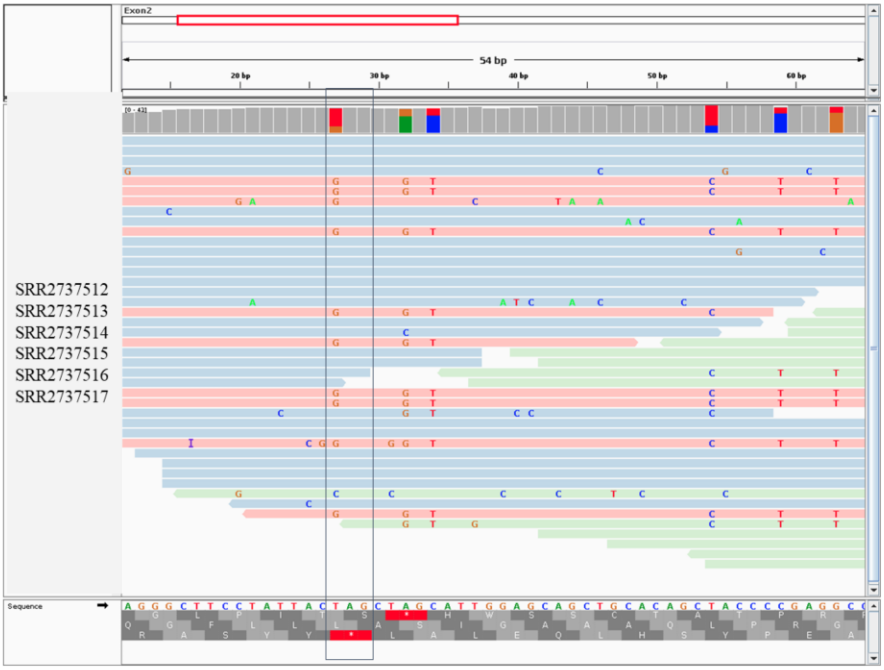

Figure S49: Zoomed view of exon-2 of *COA1*, vertical box is indicating the polymorphic site at position 27 where proportion of T is higher than G which leads a premature stop codon in haplotype 1 of *COA1* in cheetah.

Figure S50

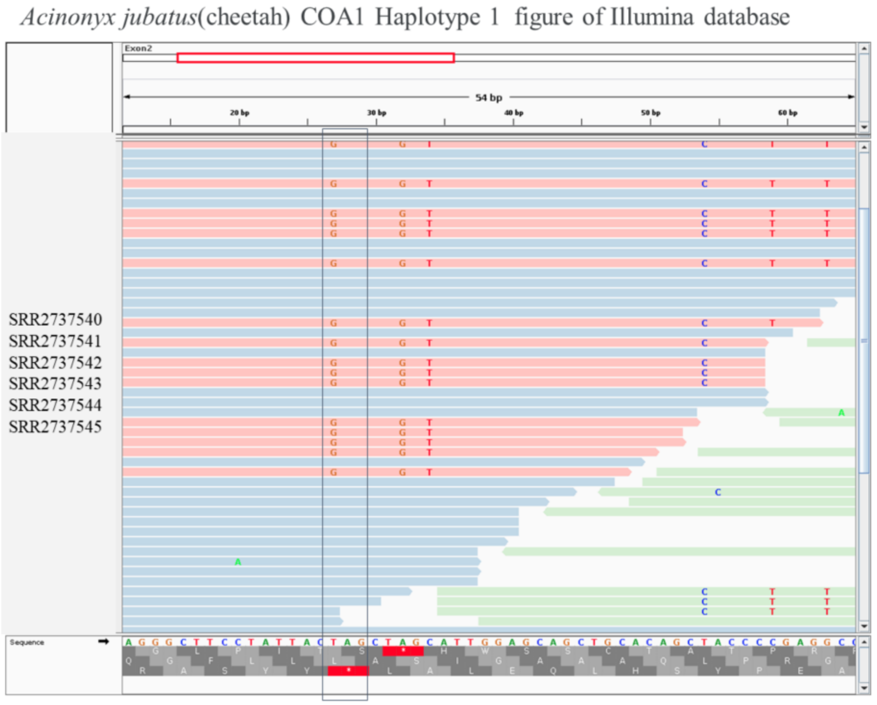

Figure S50: Zoomed view of exon-2 of *COA1*, vertical box is indicating the polymorphic site at position 27 where proportion of T is higher than G which leads a premature stop codon in cheetah.

Figure S51

Acinonyx jubatus(cheetah) *COA1* Haplotype 1 figure of Hi-C database

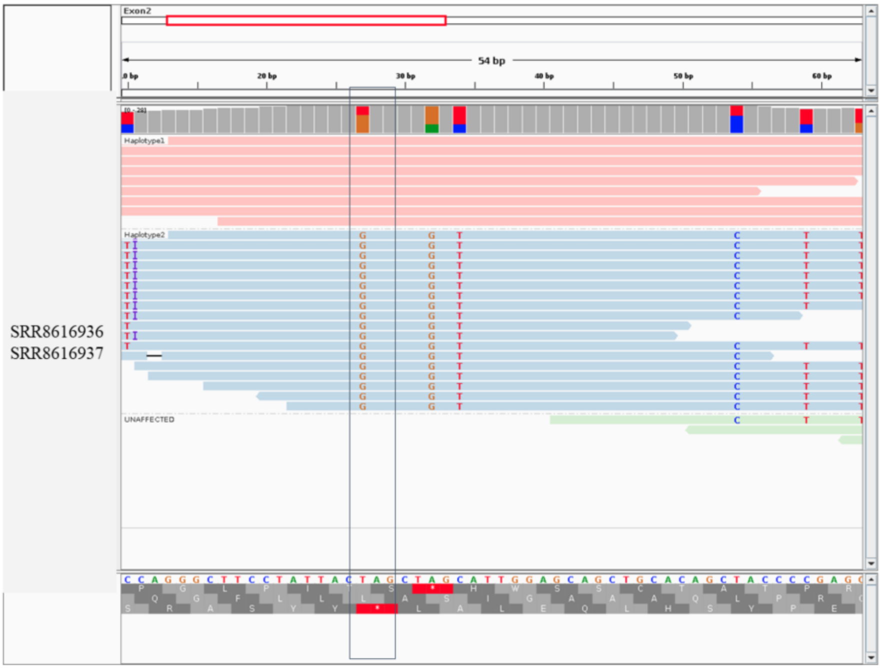

Figure S51: Zoomed view of exon-2 of *COA1*, vertical box is indicating the polymorphic site at position 27 where proportion of T is higher than G which leads a premature stop codon in cheetah.

Figure S52 *Acinonyx jubatus*(cheetah) *COA1* Haplotype 2 figure of Illumina database

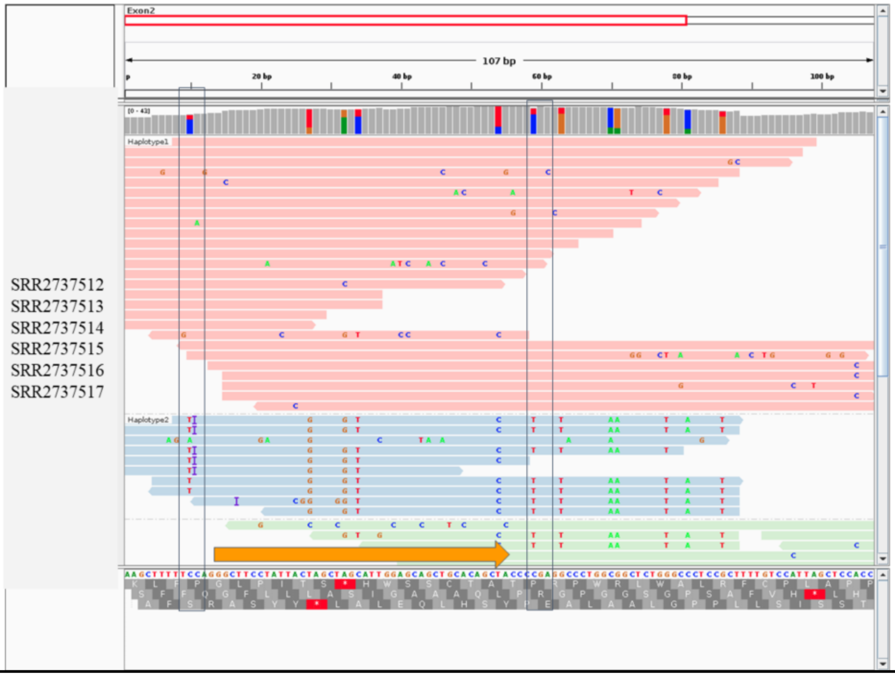

Figure S52: Zoomed view of exon-2 of *COA1*, first vertical box is indicating the insertion of T at position 10, which leads a premature stop codon at position 59 (2nd vertical box) in haplotype 2 of *COA1* in cheetah.

Figure S53

Acinonyx jubatus(cheetah) COA1 Haplotype 2 figure of Illumina database

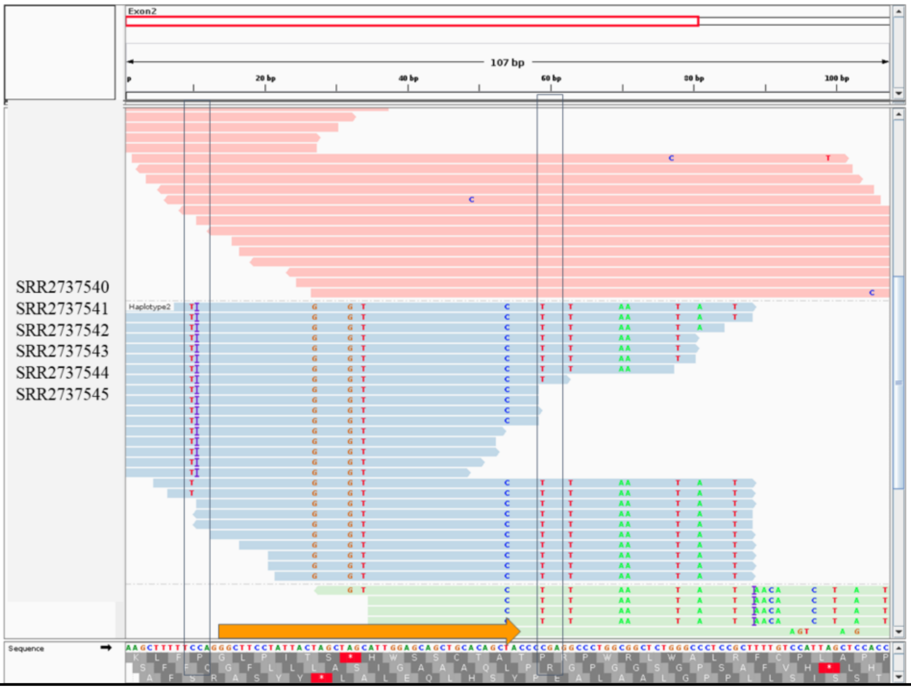

Figure S53: Zoomed view of exon-2 of *COA1*, first vertical box is indicating the insertion of T at position 10, which leads a premature stop codon at position 59 (2nd vertical box) in haplotype 2 of *COA1* in cheetah.

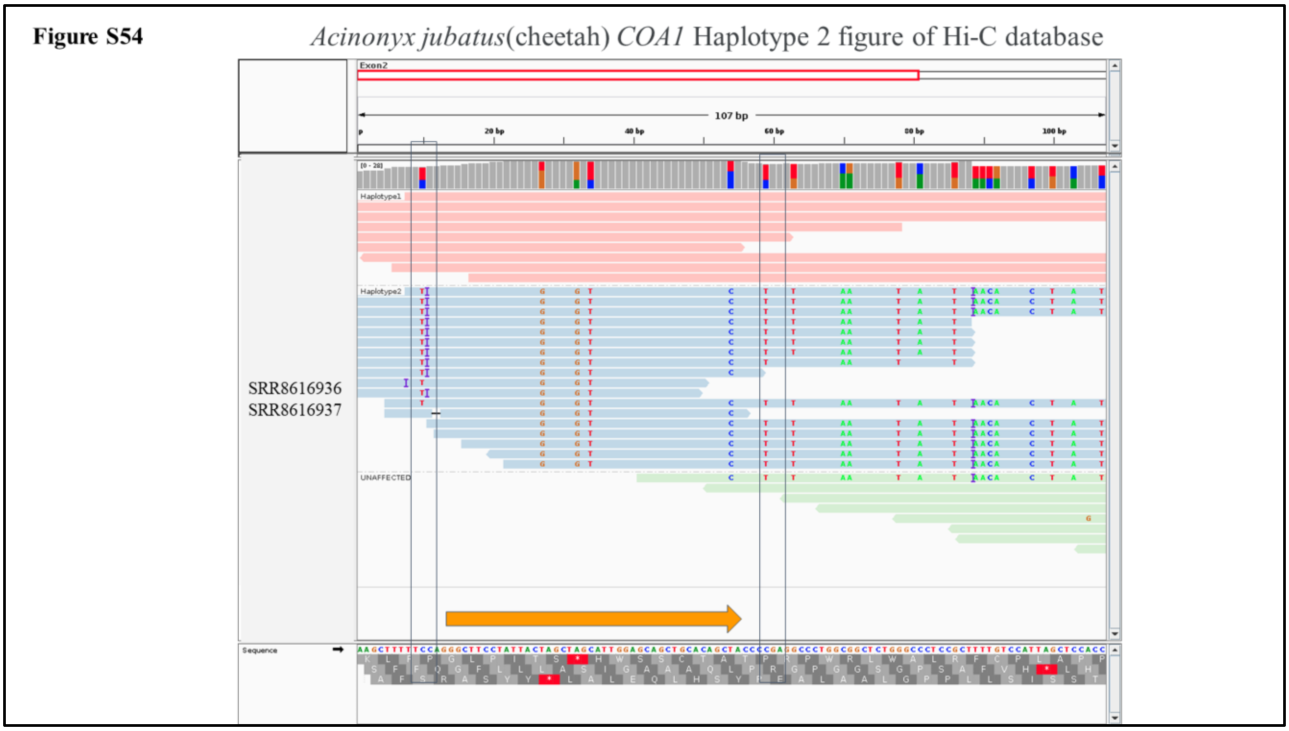

Figure S54: Zoomed view of exon-2 of *COA1*, first vertical box is indicating the insertion of T at position 10, which leads a premature stop codon at position 59 (2nd vertical box) in haplotype2 of *COA1* in cheetah.

Figure S55 *Acinonyx jubatus(cheetah)* Skin tissue GCF_003709585.1_Aci_jub_2_genomic.fna

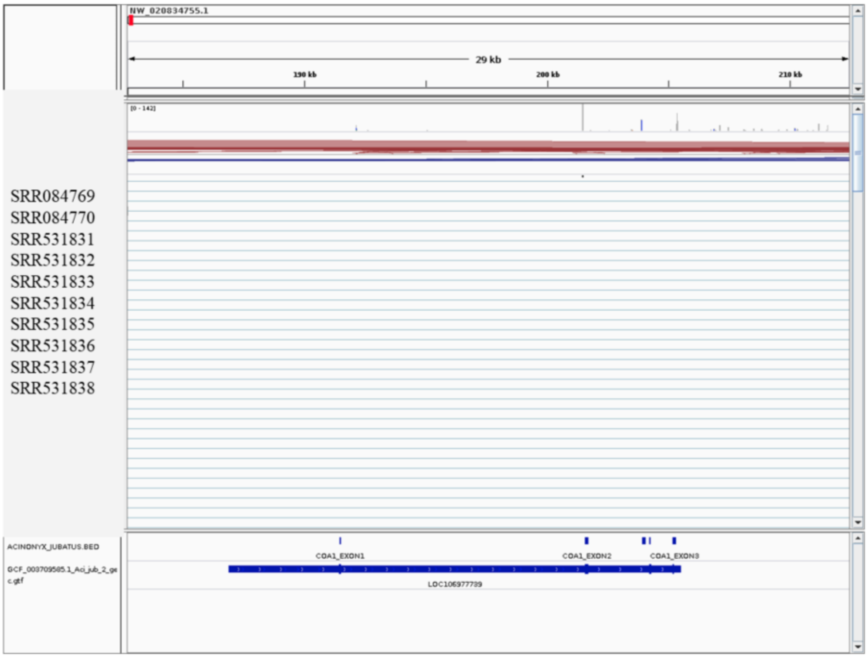

Figure S55: Screenshot of RNA-seq reads aligned to each of the putative locations of the three *COA1* exons in cheetah (*SRR11679539*). Gray boxes represent reads aligned to the genome and thin lines connecting these boxes represent the spliced read alignments. Datasets from (SRR084769 SRR084770 SRR531831 SRR531832 SRR531833 SRR531834 SRR531835 SRR531836 SRR531837 SRR531838) have been mapped to the GCF_003709585.1_Aci_jub_2_genomic.fna. Locations of each of the exons are depicted as bed record in the bottom row.

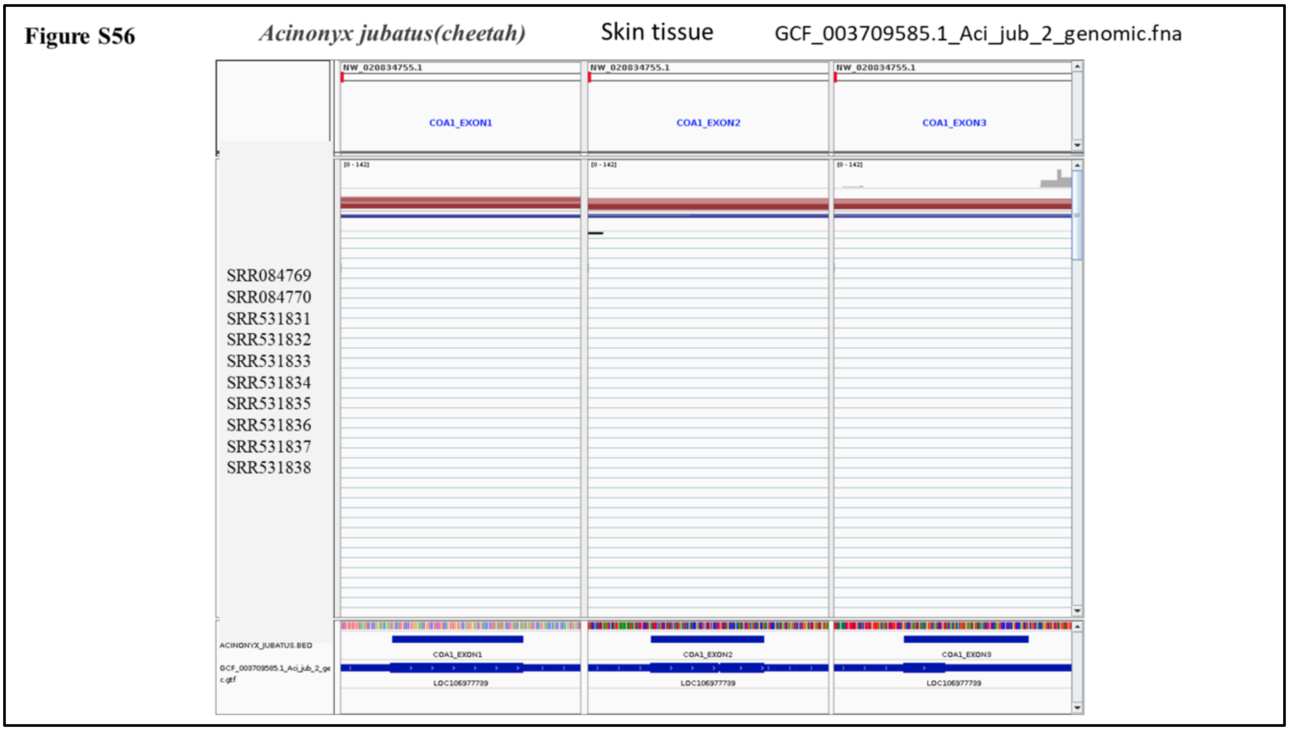

Figure S56: A zoomed in view of each of the *COA1* exons shown in **Supplementary Fig. S55**.

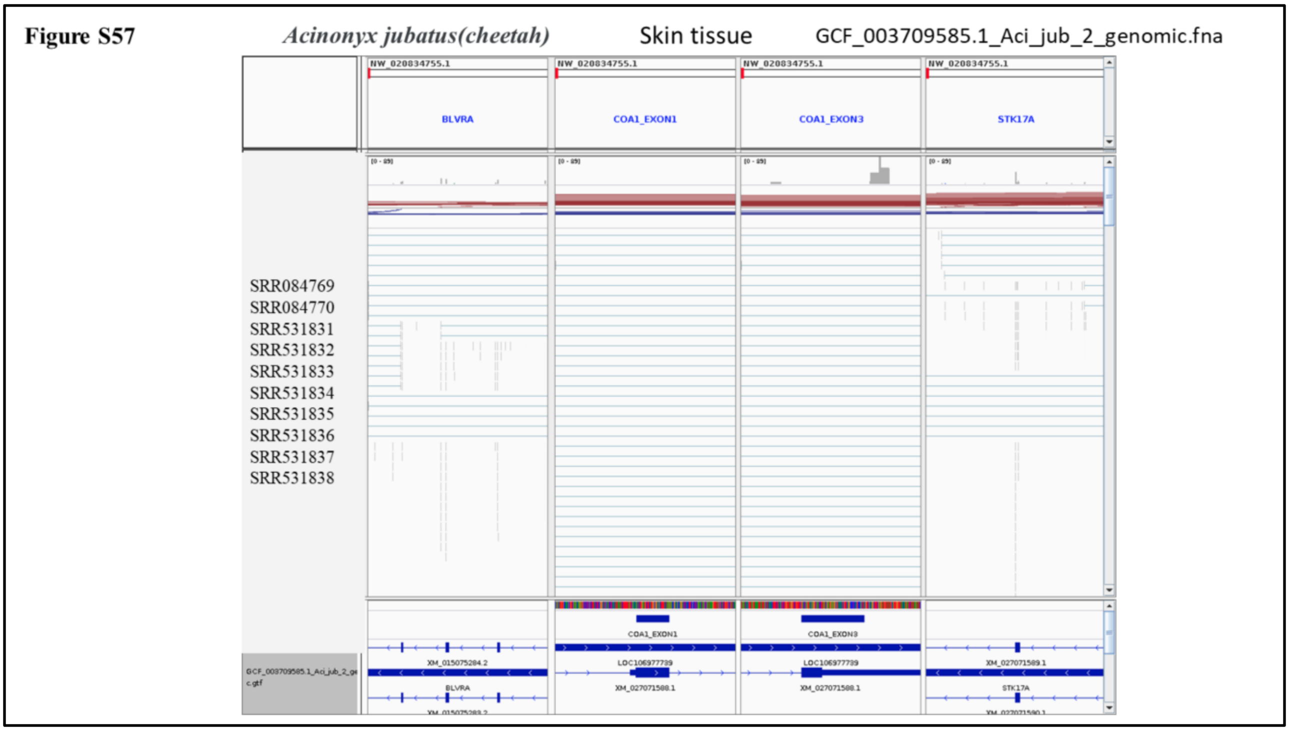

Figure S57: Screenshot of RNA-seq reads aligned to each of the putative locations of the three *COA1* exons and its nearby gene *BLVRA* and *STK17A* in cheetah (*Acinonyx jubatus*). Gray boxes represent reads aligned to the genome and thin lines connecting these boxes represent the spliced read alignments. Datasets from (SRR084769 SRR084770 SRR531831 SRR531832 SRR531833 SRR531834 SRR531835 SRR531836 SRR531837 and SRR531838) have been mapped to the GCF_003709585.1_Aci_jub_2_genomic.fna. Locations of each of the exons are depicted as bed record in the bottom row.

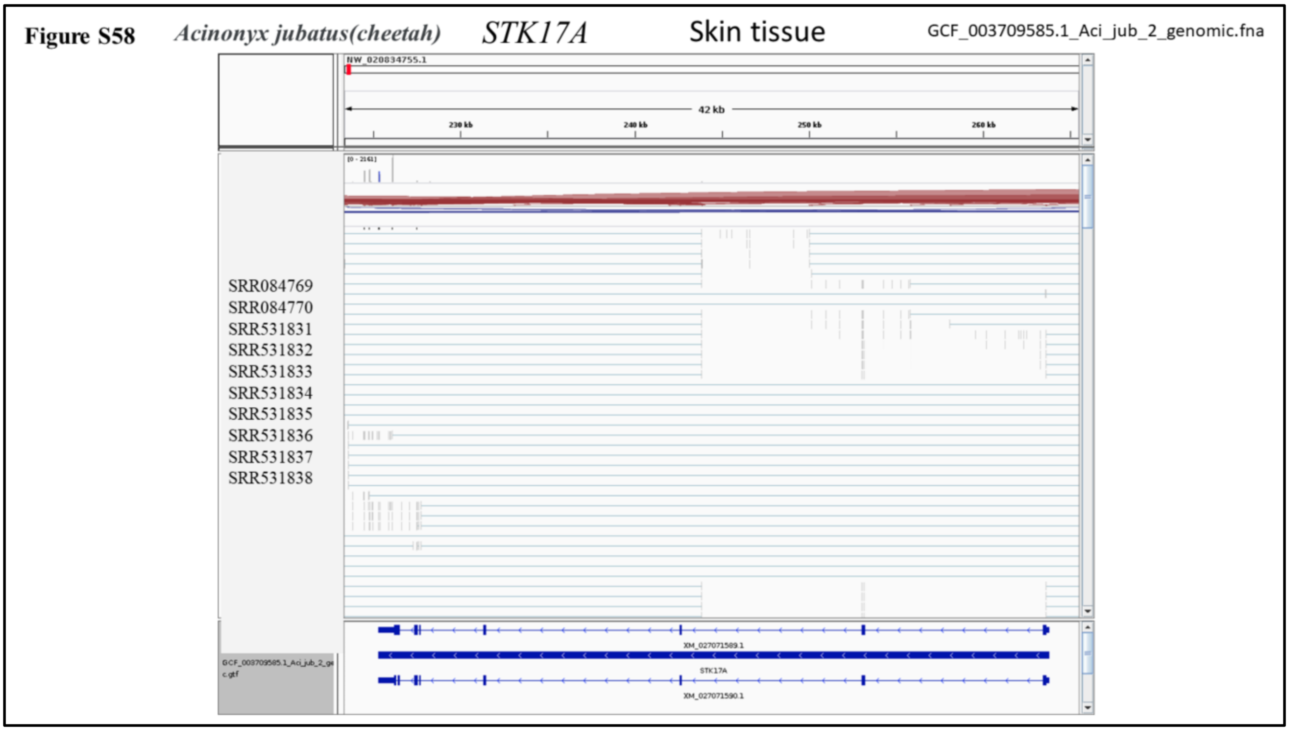

Figure S58: Screenshot of RNA-seq reads aligned to each of the putative locations of the *STK17A* in cheetah (*Acinonyx jubatus*). Gray boxes represent reads aligned to the genome and thin lines connecting these boxes represent the spliced read alignments. Datasets from (SRR084769 SRR084770 SRR531831 SRR531832 SRR531833 SRR531834 SRR531835 SRR531836 SRR531837 SRR531838) have been mapped to the GCF_003709585.1_Aci_jub_2_genomic.fna. Locations of each of the exons are depicted as bed record in the bottom row.

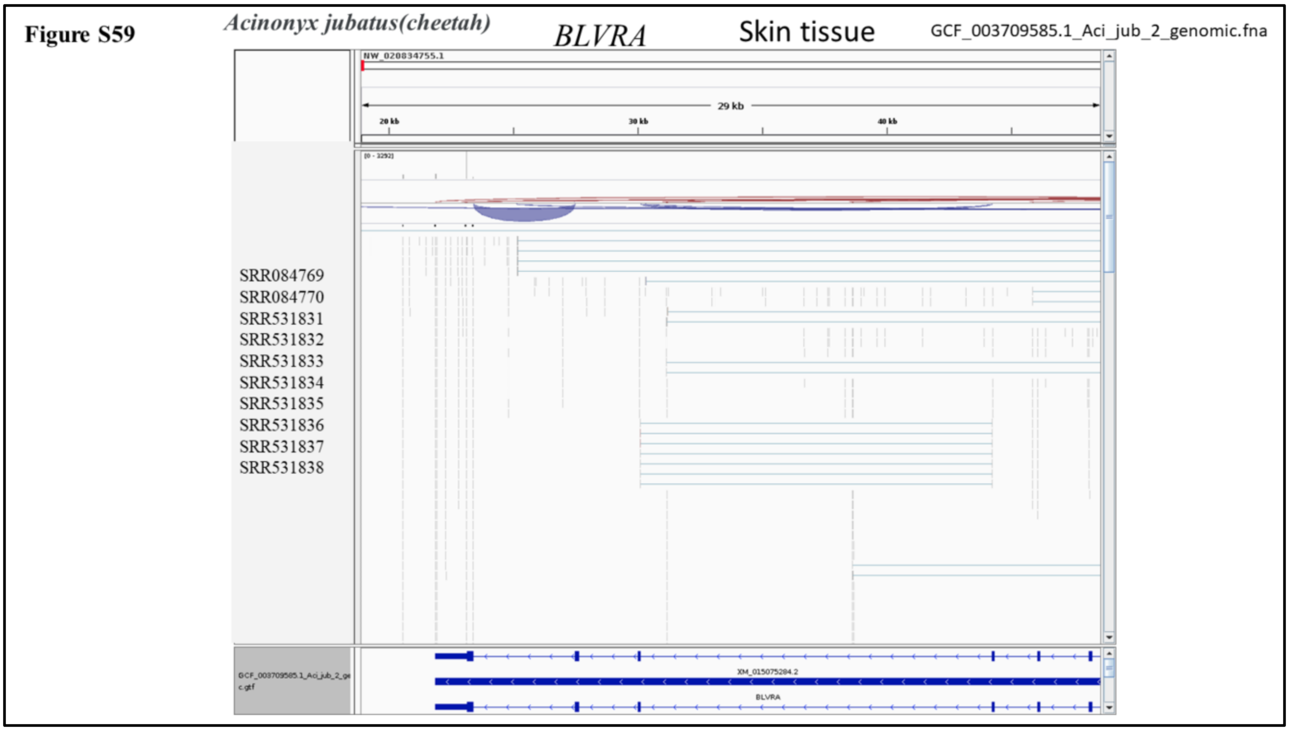

Figure S59: Screenshot of RNA-seq reads aligned to each of the putative locations of the *BLVRA* in cheetah *(Acinonyx jubatus*). Gray boxes represent reads aligned to the genome and thin lines connecting these boxes represent the spliced read alignments. Datasets from (SRR084769 SRR084770 SRR531831 SRR531832 SRR531833 SRR531834 SRR531835 SRR531836 SRR531837 SRR531838) have been mapped to the GCF_003709585.1_Aci_jub_2_genomic.fna. Locations of each of the exons are depicted as bed record in the bottom row.

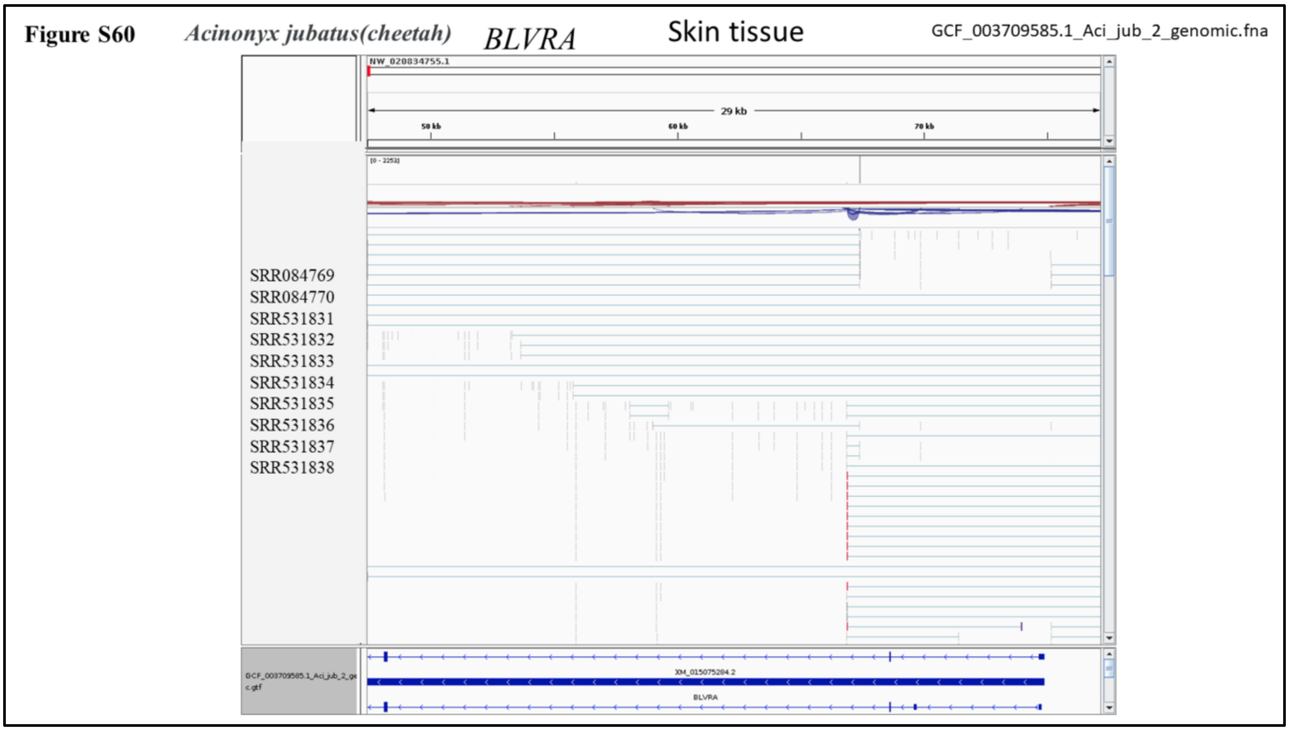

Figure S60: Screenshot of RNA-seq reads aligned to each of the putative locations of the *BLVRA* in cheetah (*Acinonyx jubatus*). Gray boxes represent reads aligned to the genome and thin lines connecting these boxes represent the spliced read alignments. Datasets from (SRR084769 SRR084770 SRR531831 SRR531832 SRR531833 SRR531834 SRR531835 SRR531836 SRR531837 SRR531838) have been mapped to the GCF_003709585.1_Aci_jub_2_genomic.fna. Locations of each of the exons are depicted as bed record in the bottom row.

Figure S61 *Felis_catus*(cat) Skin tissue Felis_catus.Felis_catus_9.0.dna_sm.toplevel.fa

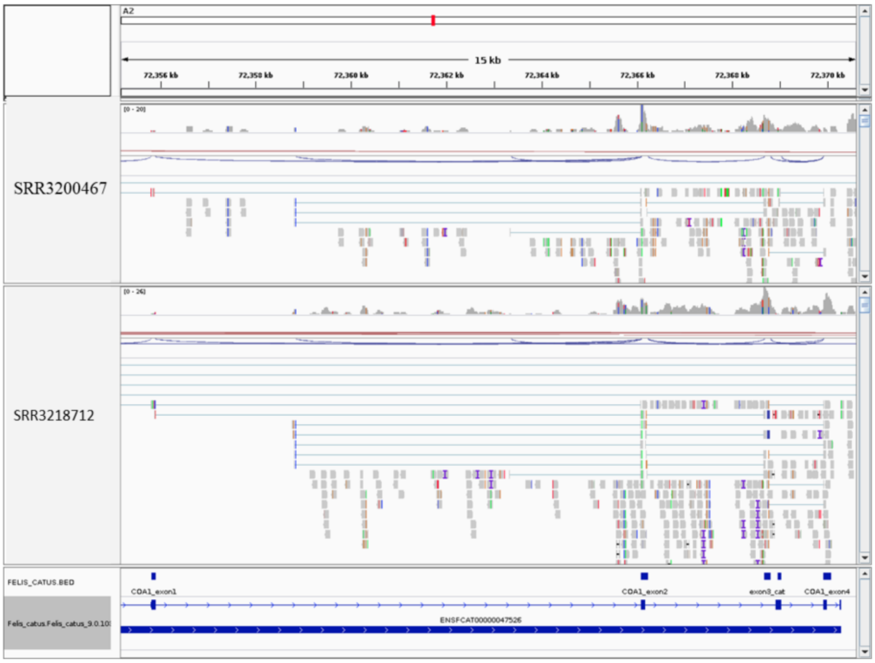

Figure S61: Screenshot of RNA-seq reads aligned to each of the putative locations of the three *COA1* exons in cat (*Felis catus*). Gray boxes represent reads aligned to the genome and thin lines connecting these boxes represent the spliced read alignments. Datasets from skin (SRR3200467 and SRR3218712) have been mapped to the Felis_catus.Felis_catus_9.0.dna_sm.toplevel.fa. Locations of each of the exons are depicted as bed record in the bottom row.

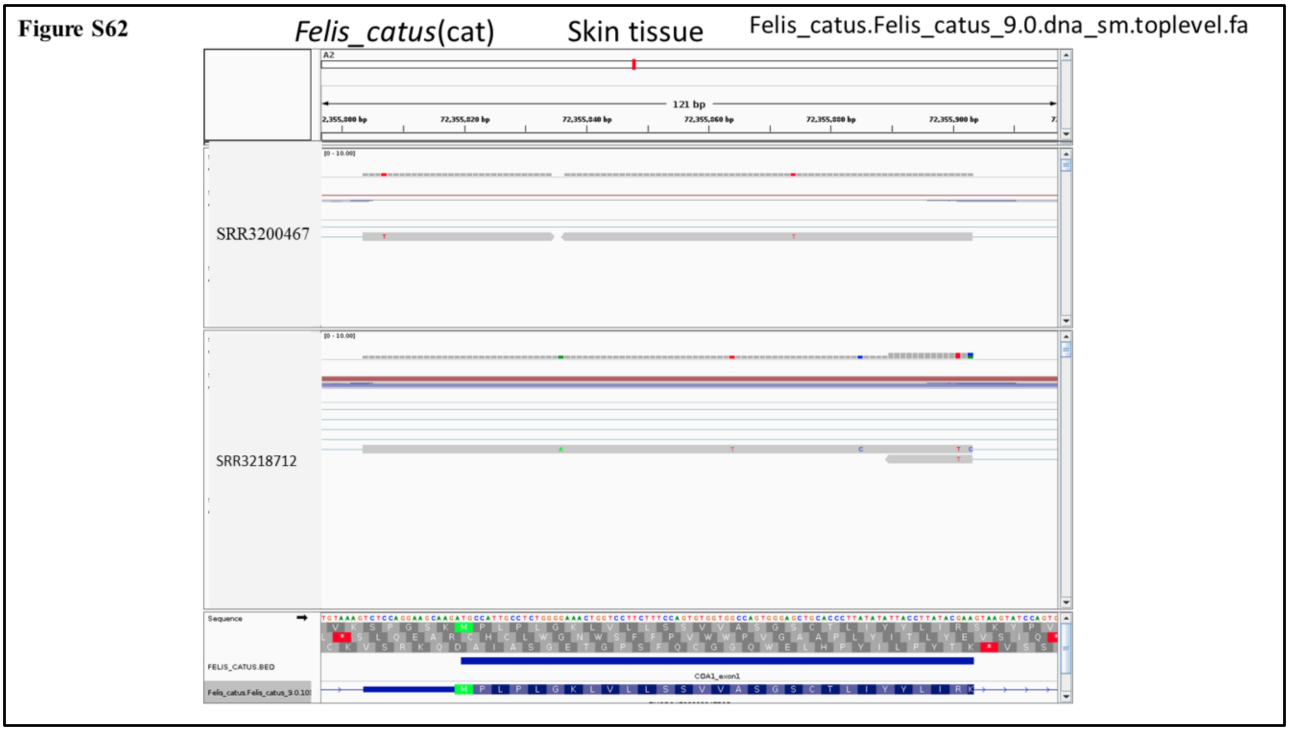

Figure S62: A zoomed in view of exon1 of the *COA1* shown in **Supplementary Fig. S61**.

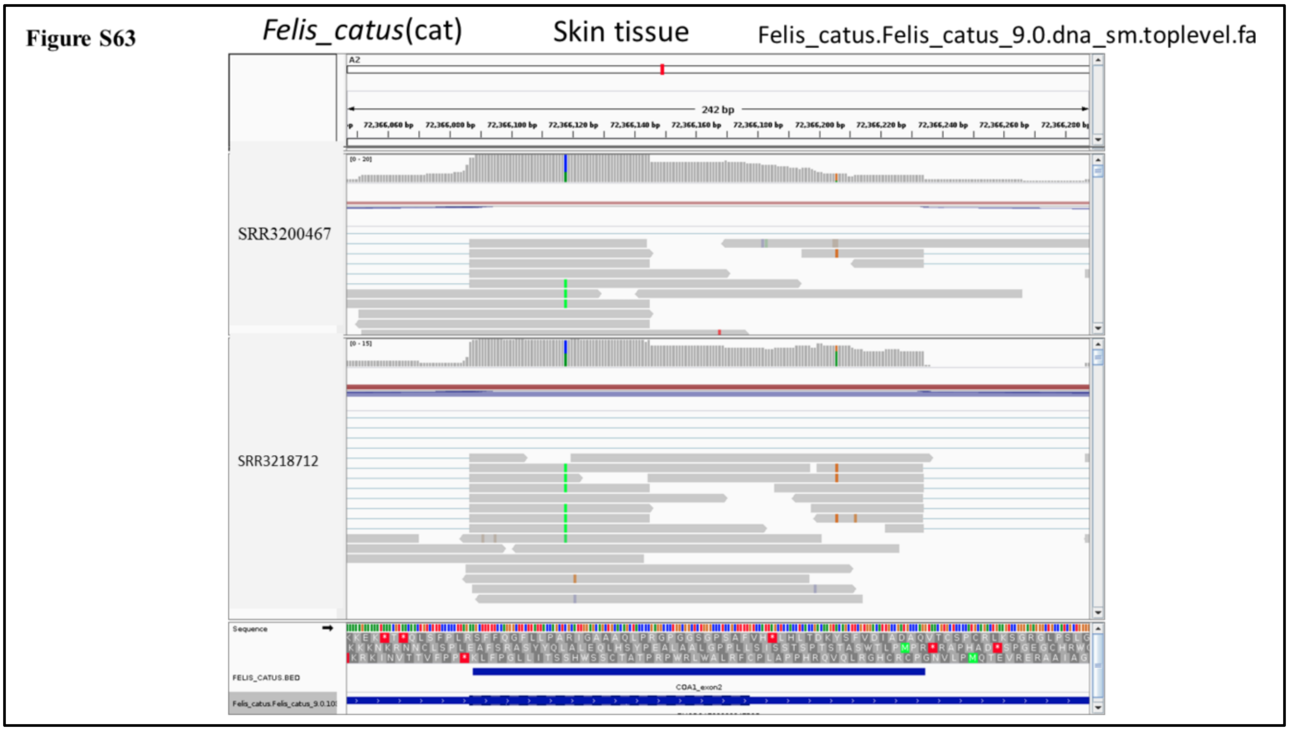

Figure S63: A zoomed in view of exon-2 of the *COA1* shown in **Supplementary Fig. S61**.

Figure S64

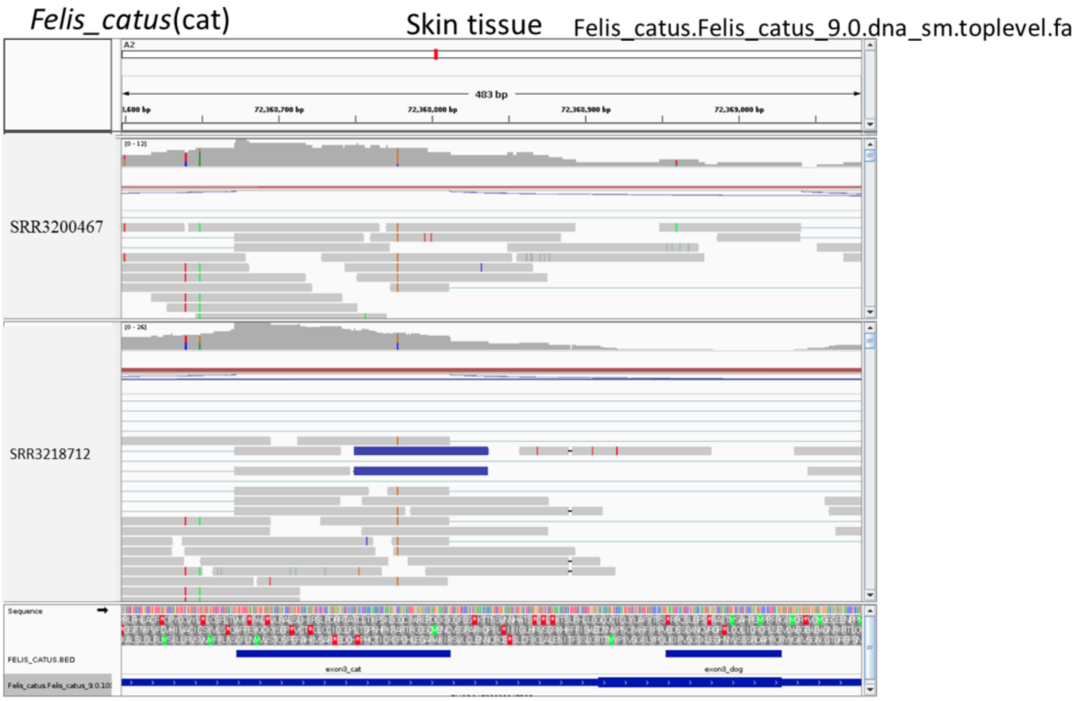

Figure S64: A zoomed in view of exon-3 of the *COA1* shown in **Supplementary Fig. S61**.

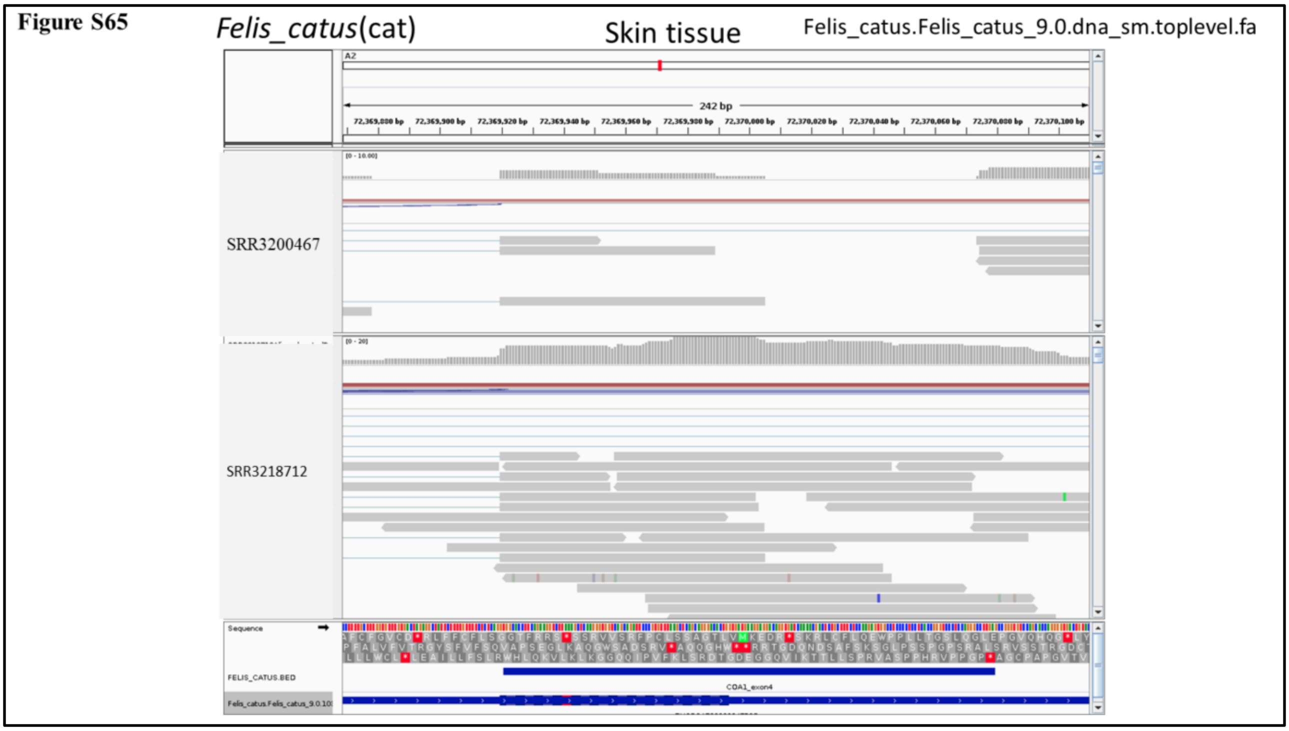

Figure S65: A zoomed in view of exon-4 of the *COA1* shown in **Supplementary Fig. S61**.

Figure S66 *Canis_lupus_familiaris*(dog) Skin tissue Canis_lupus_familiaris.CanFam3.1.dna_sm.toplevel.fa

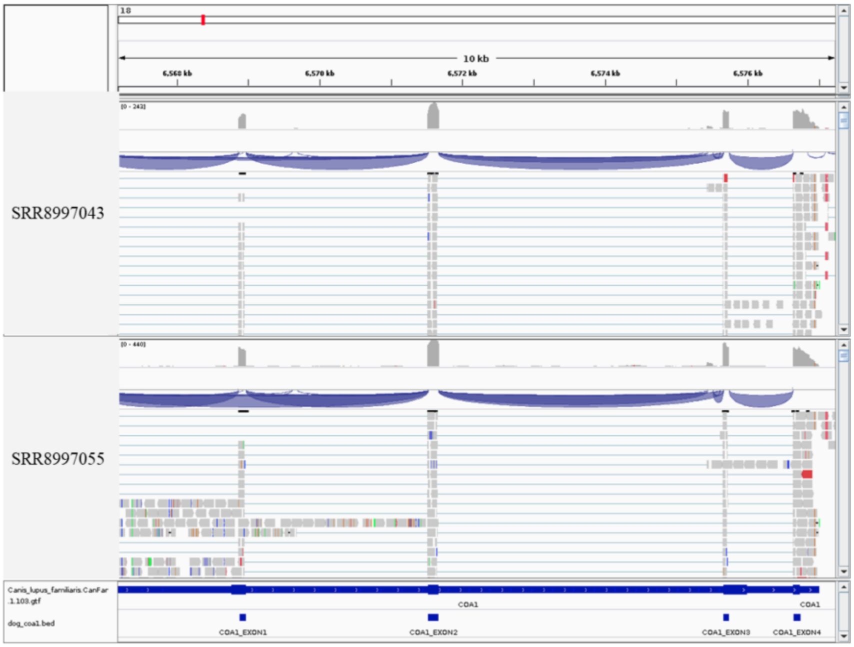

Figure S66: Screenshot of RNA-seq reads aligned to each of the putative locations of the four *COA1* exons in dog (*Canis lupus familiaris*). Gray boxes represent reads aligned to the genome and thin lines connecting these boxes represent the spliced read alignments. Datasets from skin muscle (SRR8997043 and SRR8997055) have been mapped to the Canis_lupus_familiaris.CanFam3.1.dna_sm.toplevel.fa. Locations of each of the exons are depicted as bed record in the bottom row.

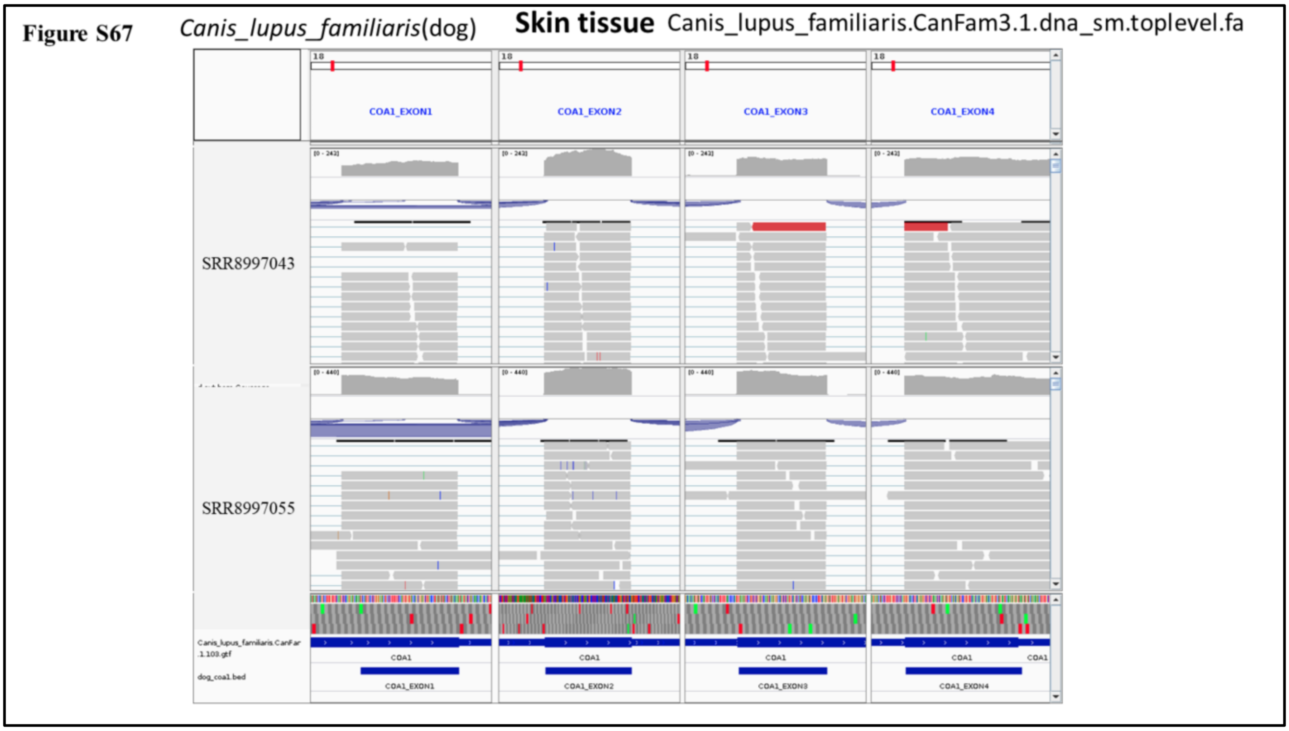

Figure S67: A zoomed in view of each of the *COA1* shown in **Supplementary Fig. S66**.

Figure S68

Acinonyx jubatus (cheetah) *COA1* haplotype figure of Illumina database

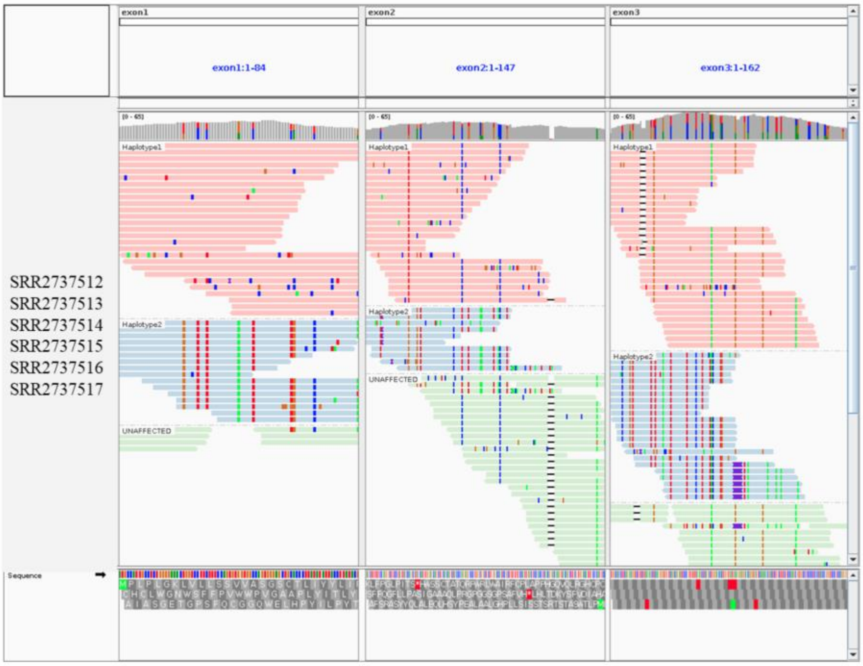

Figure S68: Comparison of haplotypes of *COA1* gene in cheetah :Two haplotypes in exon 1, 2 and single haplotype in exon-3.

Figure S69

Acinonyx jubatus(cheetah) *COA1* haplotype figure of Illumina database

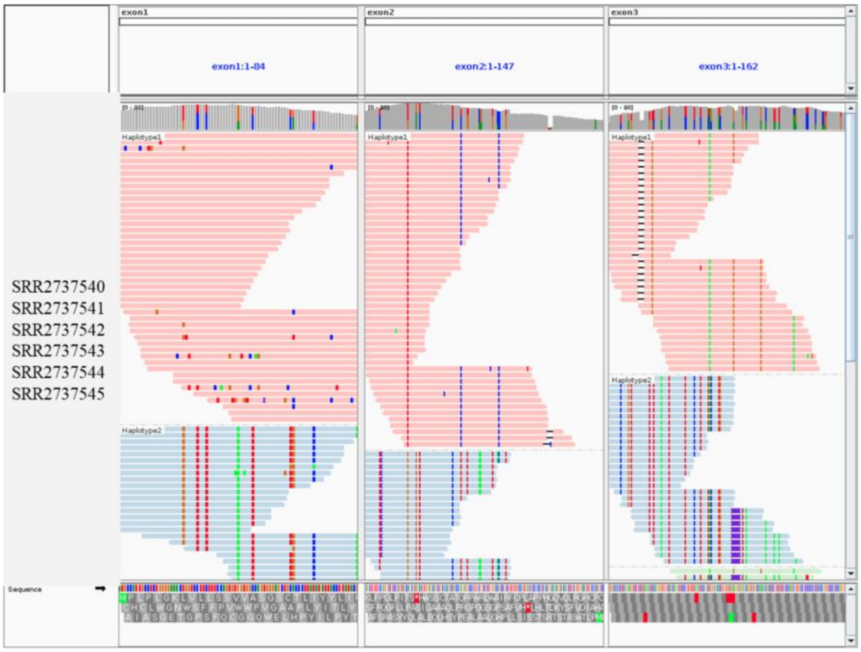

Figure S69: Comparison of haplotypes of *COA1* gene in cheetah :Two haplotypes in exon 1, 2 and single haplotype in exon-3.

Figure S70

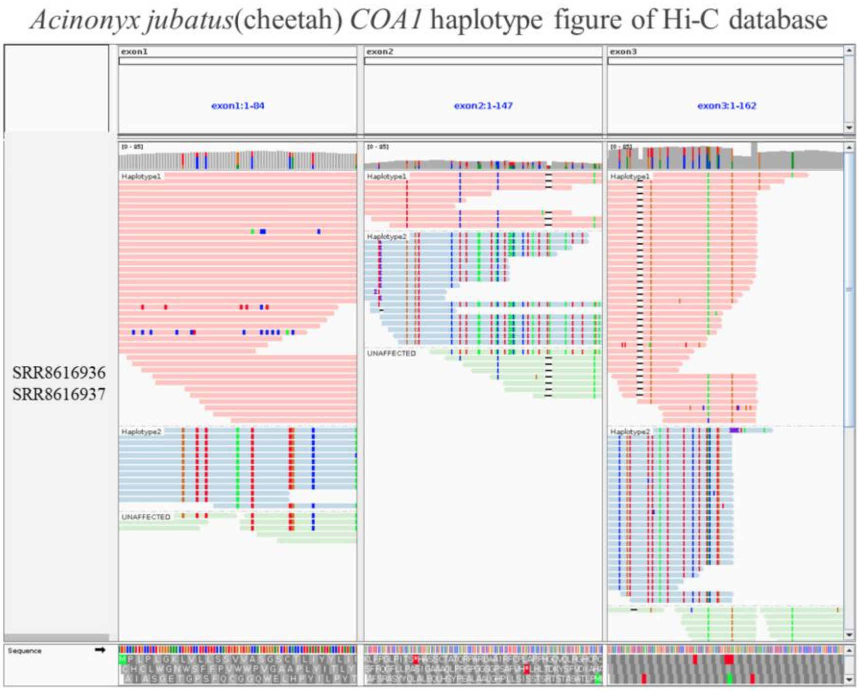

Figure S70: Comparison of haplotypes of *COA1* gene in cheetah :Two haplotypes in exon 1, 2 and single haplotype in exon-3.

Figure S71

Acinonyx jubatus(cheetah) *COA1* haplotype 2 figure of Illumina database

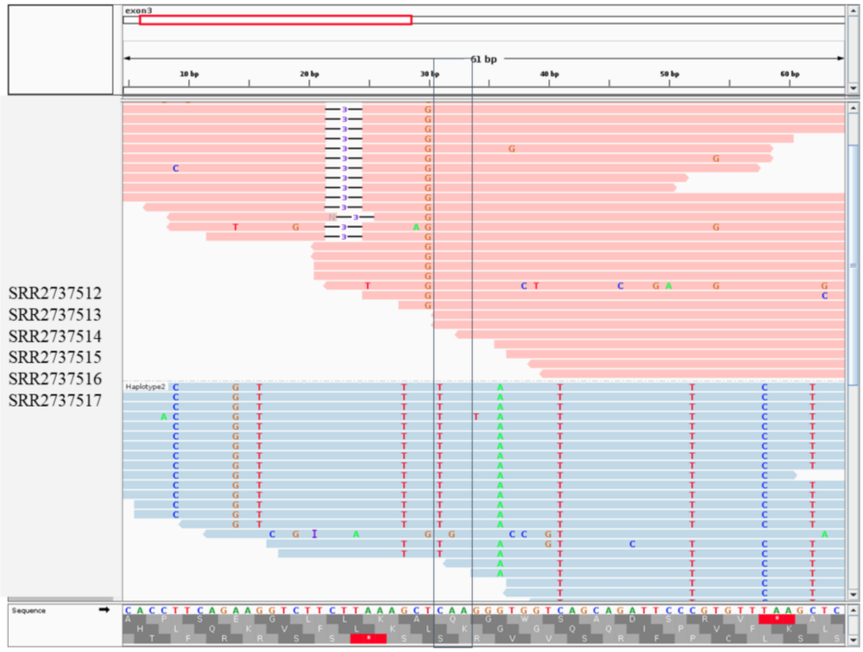

Figure S71: Zoomed view of exon-3 of *COA1* shown in **Supplementary Fig. S68**, vertical box is indicating the position 265 where T is present in haplotype 2 for those reads which don't have insertion of T at position 10 and leads to a premature stop codon in cheetah.

Figure S72

Acinonyx jubatus(cheetah) COA1 haplotype 2 figure of Illumina database

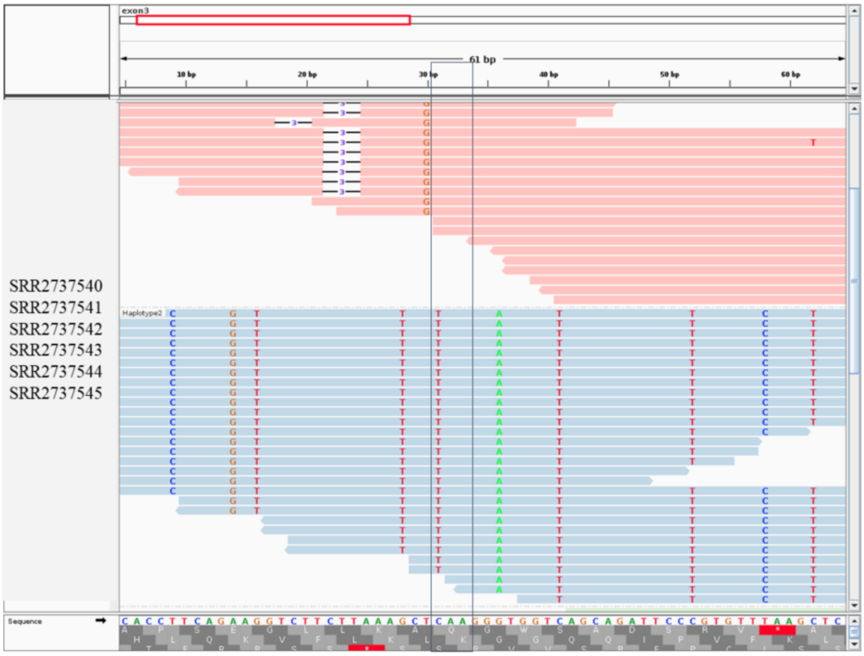

Figure S72: Zoomed view of exon-3 of *COA1* shown in **Supplementary Fig. S69**, vertical box is indicating the position 265 where T is present in haplotype 2 for those reads which don't have insertion of T at position 11 and leads to a premature stop codon in cheetah.

Figure S73 *Acinonyx jubatus*(cheetah) *COA1* haplotype 2 figure of Hi-C database

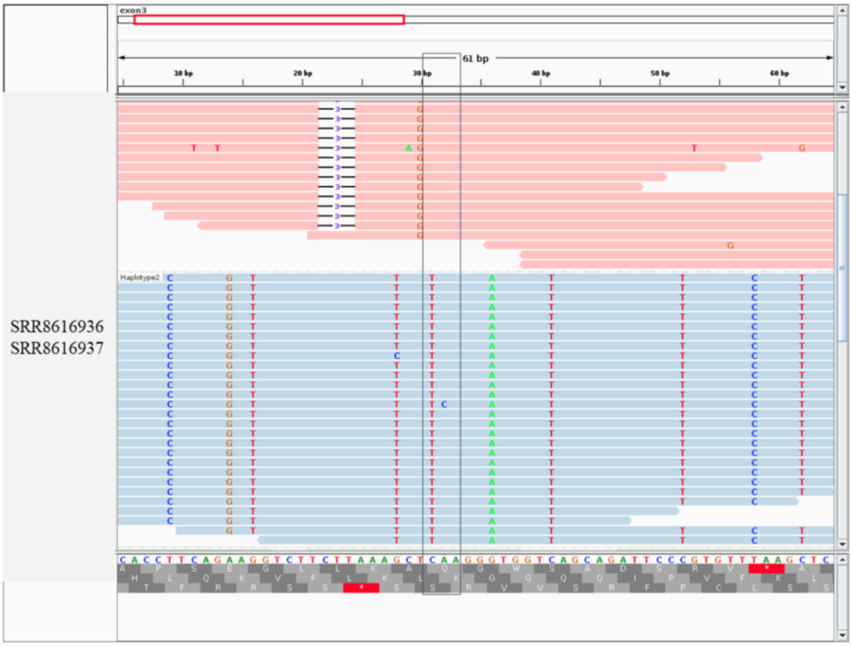

Figure S73: Zoomed view of exon-3 of *COA1* shown in **Supplementary Fig. S70**, vertical box is indicating the position 265 where T is present in haplotype 2 for those reads which don't have insertion of T at position 10 and leads to a premature stop codon in cheetah.

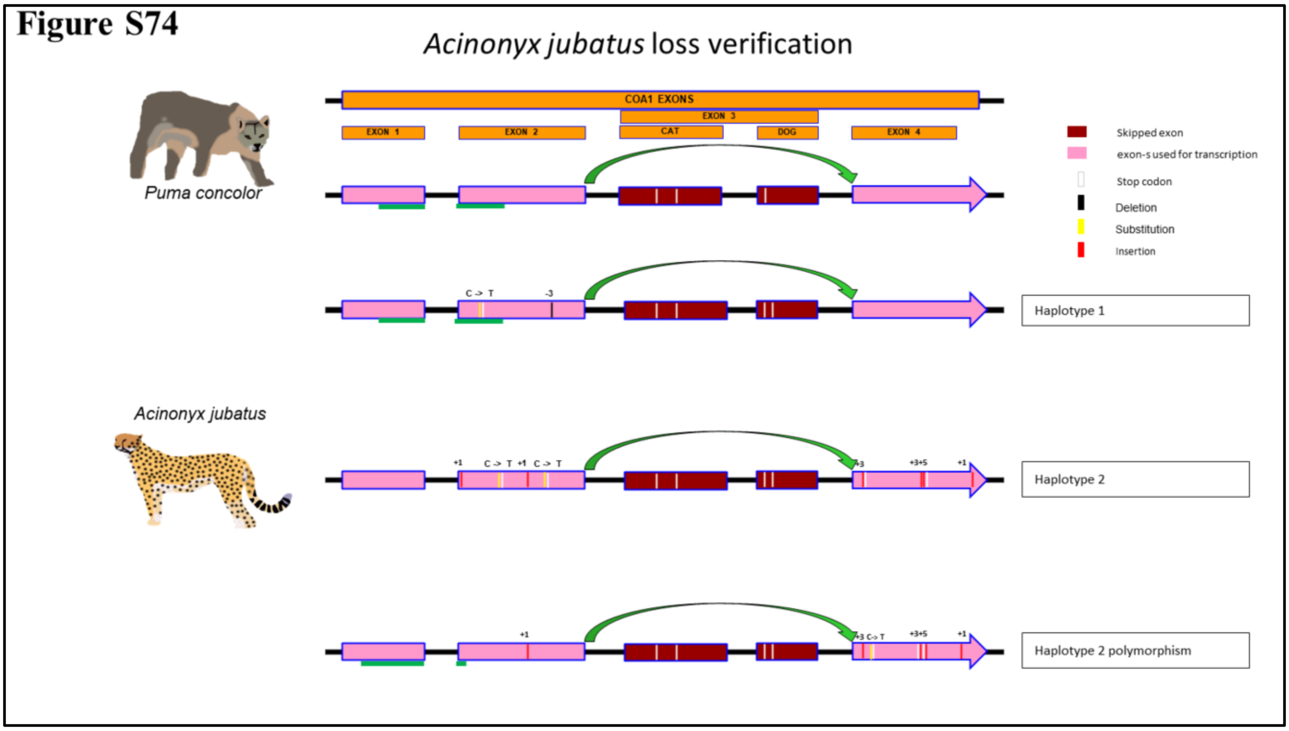

Figure S74: Verification of gene loss in two haplotypes with haplotype two polymorphism showed in **Supplementary Fig. S74**. Different events are mentioned in figure legends with different colors. Horizontal green bars represent the domain region. Puma concolor is used as reference species intact *COA1*.

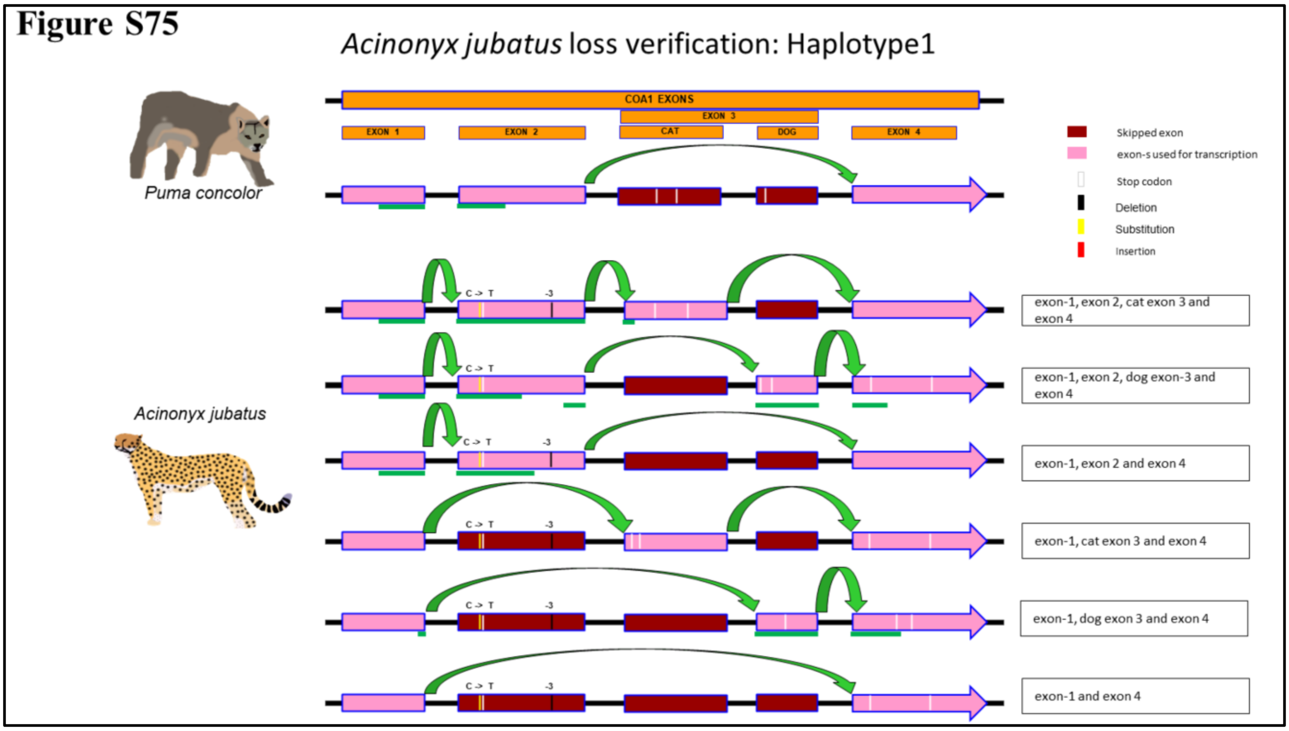

Figure S75: Different combinations of the exon with domain region in haplotype 1, green arrows represent exon splicing. More detail of colors mentioned in figure legends. *Puma concolor* is used as reference species intact *COA1*.

Figure S76

Acinonyx jubatus loss verification: Haplotype2

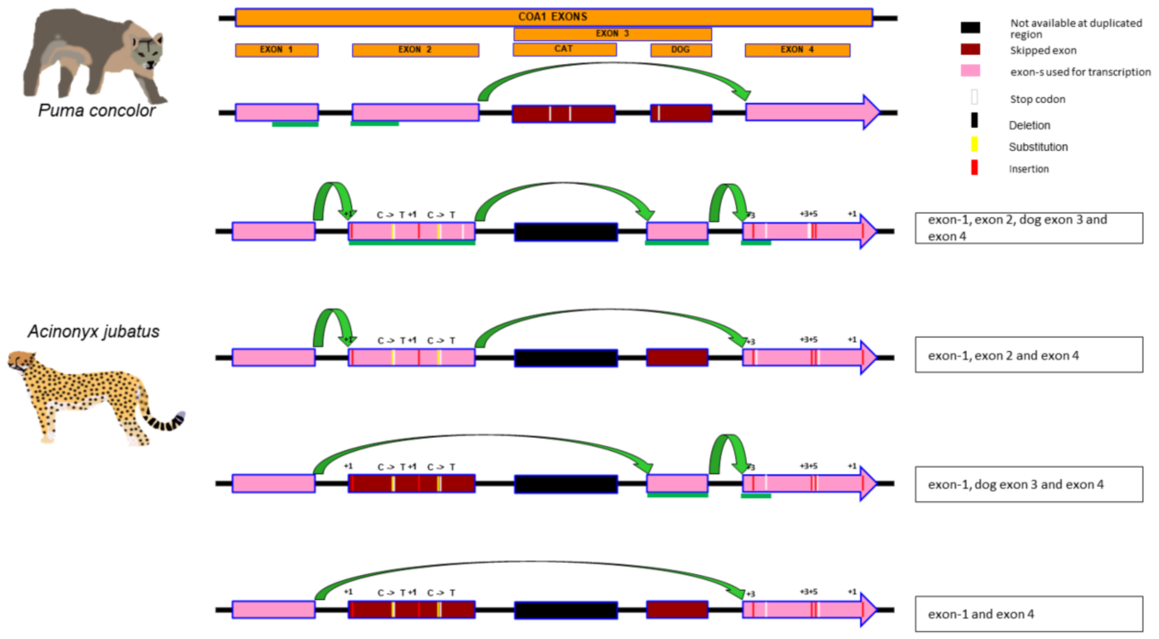

Figure S76: Different combinations of the exon with domain region in haplotype 2, green arrows represent exon splicing. More detail of colors mentioned in figure legends. *Puma concolor* is used as reference species intact *COA1*.

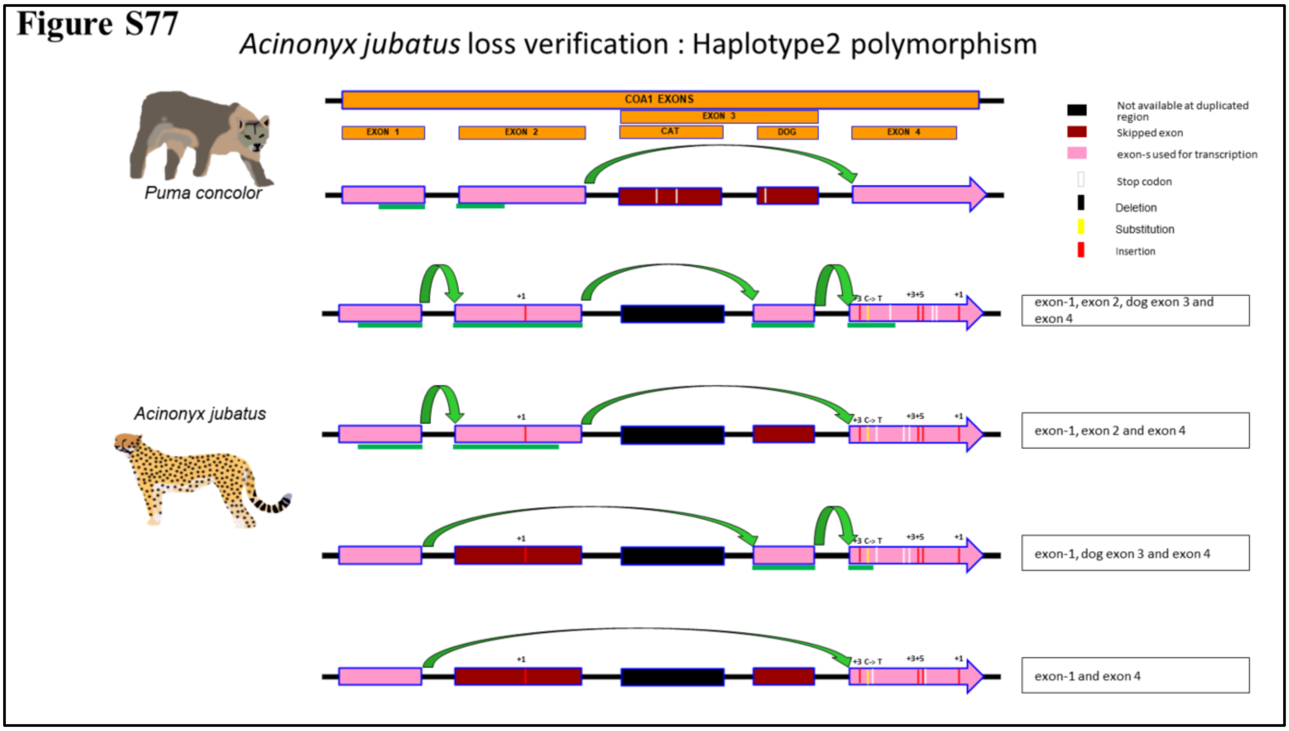

Figure S77: Different combinations of the exon with domain region in haplotype 2 polymorphism, green arrows represent exon splicing. More detail of colors mentioned in figure legends. *Puma concolor* is used as reference species intact *COA1*.

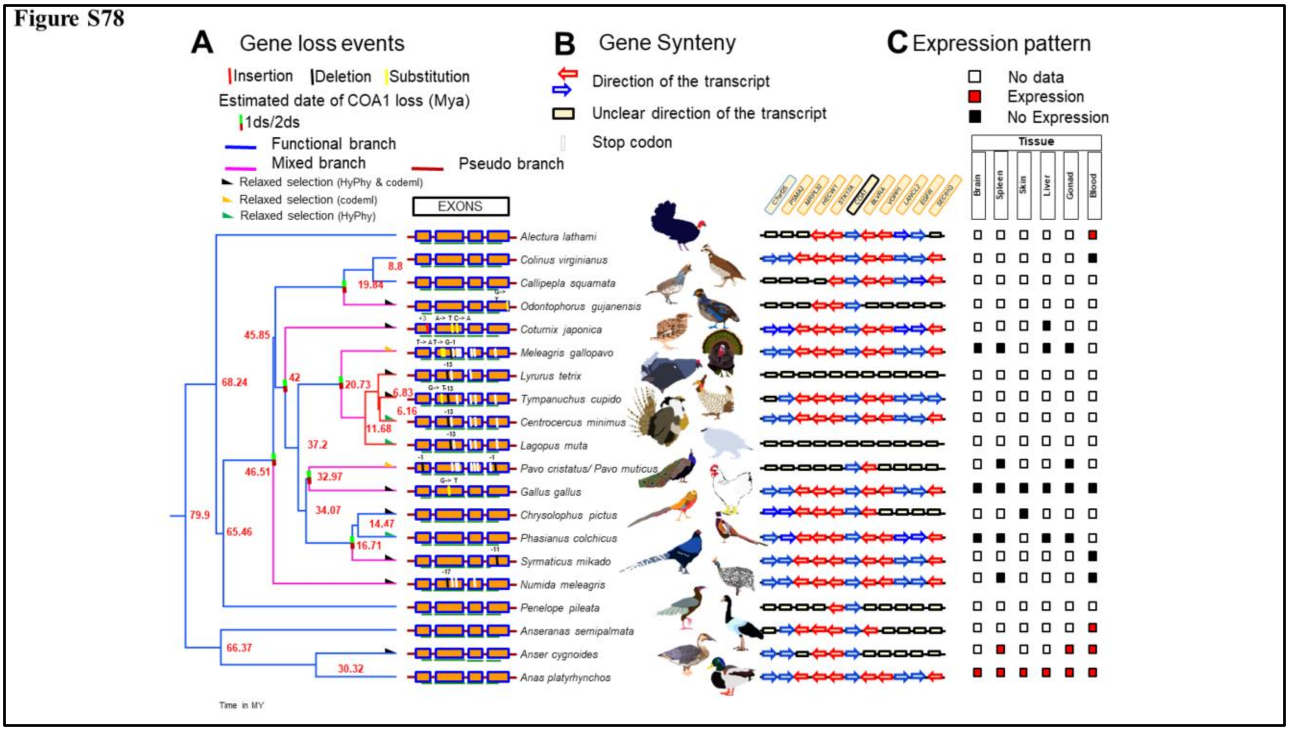

Figure S78: Recurrent loss of *COA1* gene in Galliform species. (**A**) Gene loss events in ten Galliform species besides a time-calibrated phylogenetic tree downloaded from the TimeTree website. Blue branches in the tree represent functional branches, and the pink colored branches represent mixed (functional + pseudogenic) branches and red colored branches represent pseudo branch. The method proposed by Meredith et al., 2009 was used to estimate the time of gene loss using two different substitution rates (1ds and 2ds). Short colored bars depict the locations of the gene disrupting mutations on the four exons of *COA1*. (**B**) Gene order in the

genomic region flanking the *COA1* gene in bird genomes. Red and blue arrows depict the direction of gene transcription relative to the *COA1* gene for consistency across species. Gray boxes represent the genes located on short scaffolds with unknown orientation. (**C**) The gene expression pattern of the *COA1* gene in six tissues (brain, spleen, skin, liver, gonad, and blood) was assessed by screening RNA-seq datasets. The red-colored blocks depict the robust expression of the *COA1* gene, the black-colored blocks depict a lack of *COA1* gene expression in that particular tissue, and the white-colored blocks represent a lack of data for that tissue.

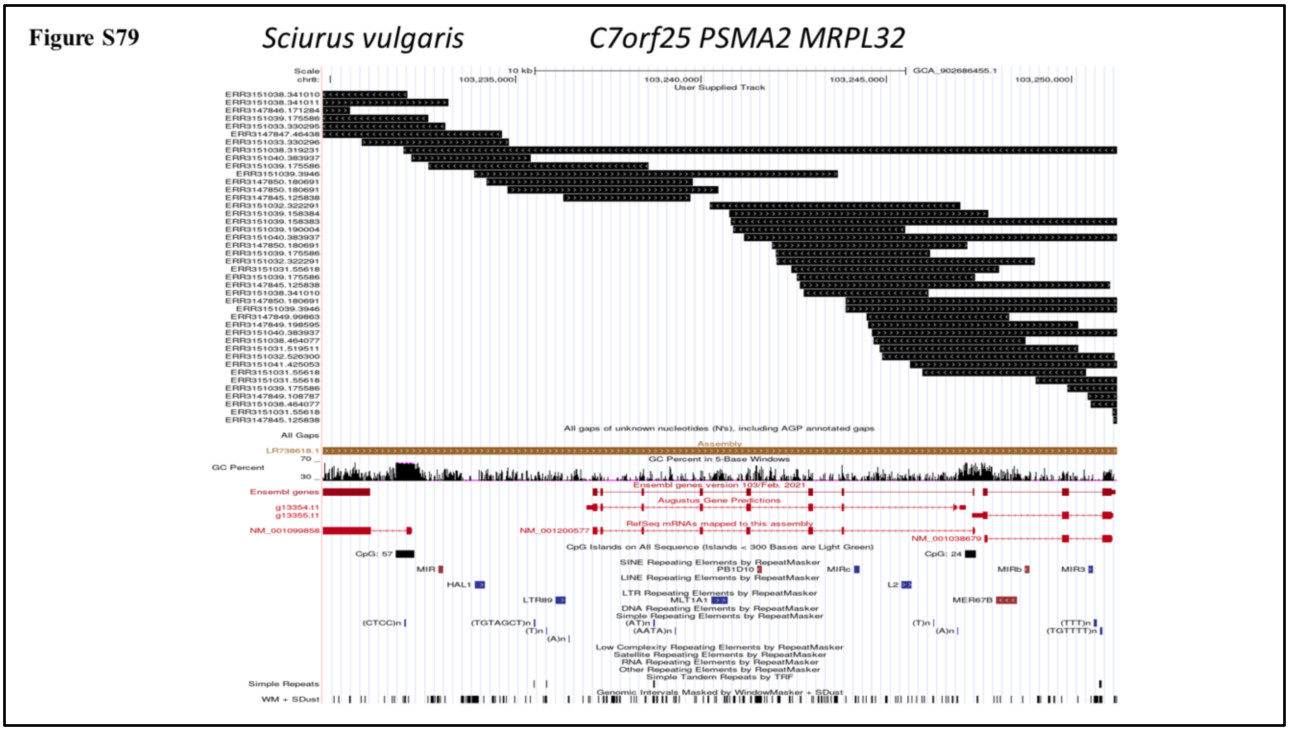

Figure S79: Screenshot of UCSC genome browser showing the alignment of > 3 kb long reads generated using PacBio technology to the Eurasian red squirrel (*Sciurus vulgaris*) **GCA_902686455.1** at the location of the *C7orf25*, *PSMA2* and *MRPL32*. Each black rectangular box represents a read that aligns to that particular position. A series of overlapping reads that span the entire region up to adjoining genes are shown. The accession numbers of each of the reads are provided beside the alignments.

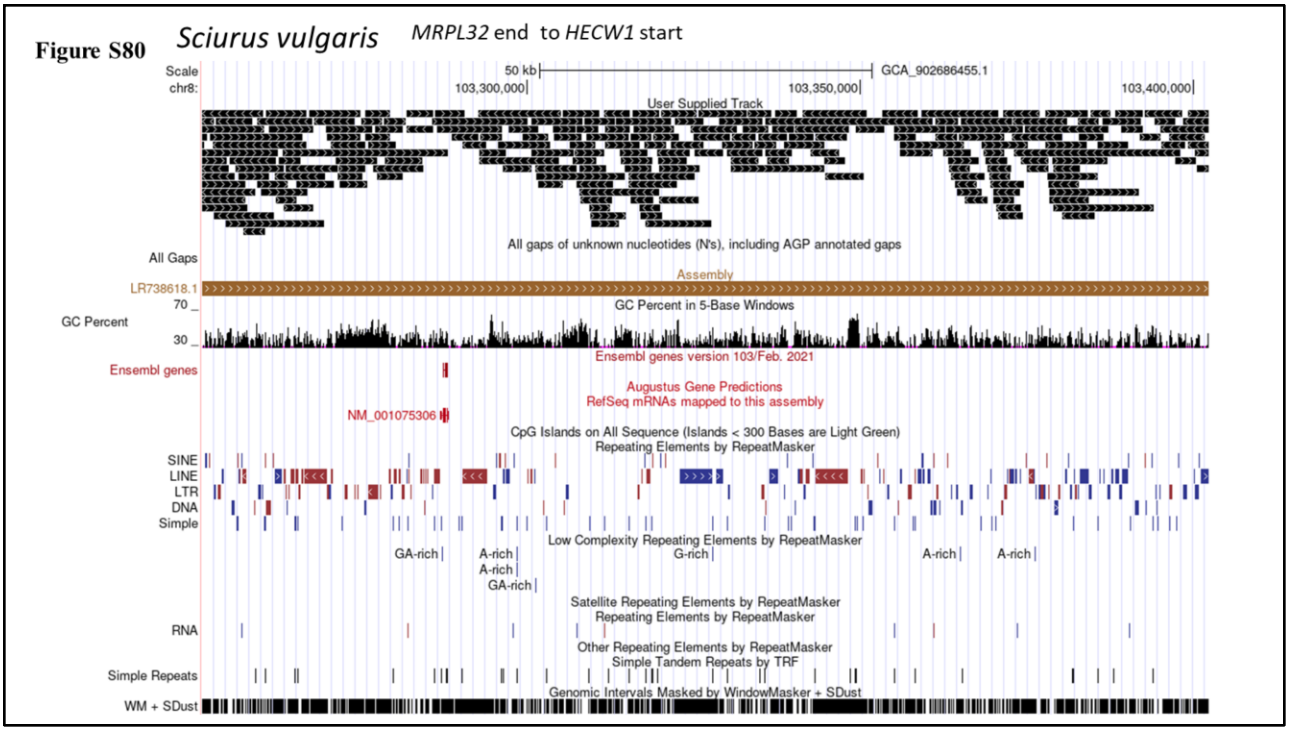

Figure S80: Screenshot of UCSC genome browser showing the alignment of > 3 kb long reads generated using PacBio technology to the Eurasian red squirrel (*Sciurus vulgaris*) **GCA_902686455.1** at the location of the *MRPL32* end to *HECW1* start. Each black rectangular box represents a read that aligns to that particular position. A series of overlapping reads that span the entire region up to adjoining genes are shown.

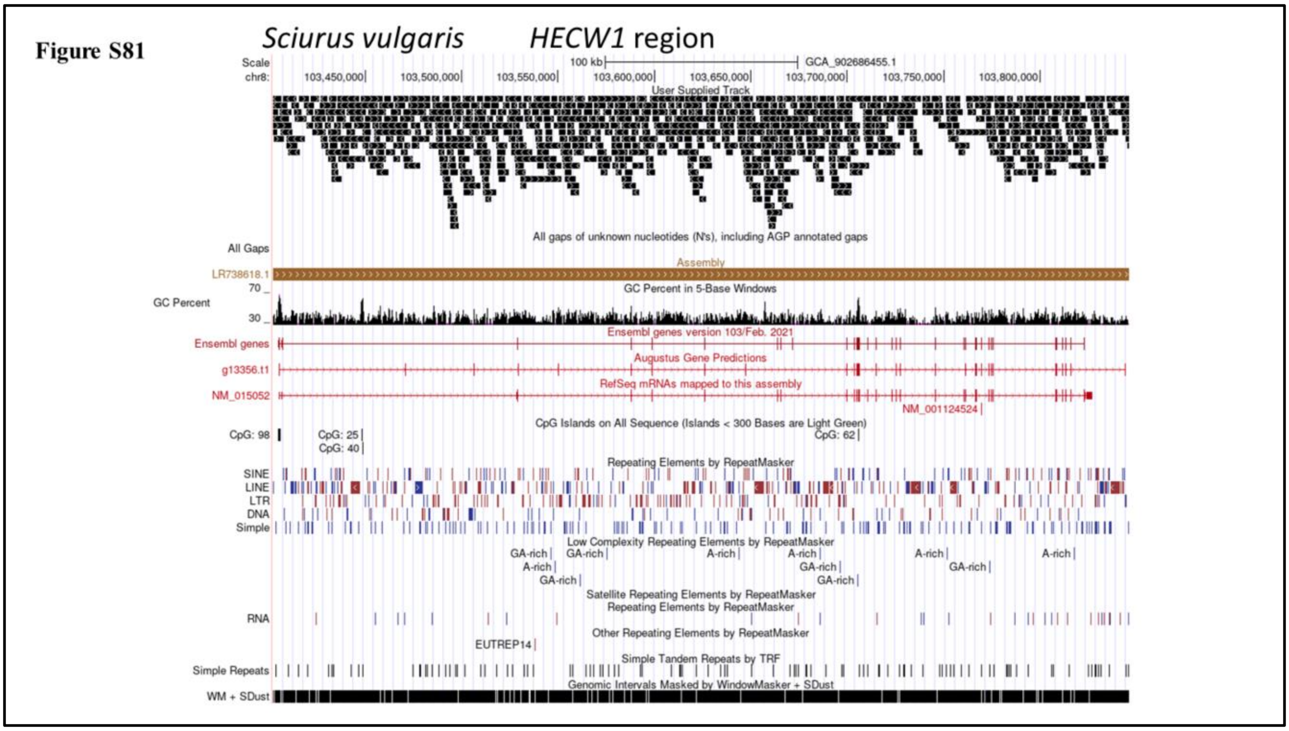

Figure S81: Screenshot of UCSC genome browser showing the alignment of > 3 kb long reads generated using PacBio technology to the Eurasian red squirrel (*Sciurus vulgaris*) **GCA_902686455.1** at the location of the *HECW1*. Each black rectangular box represents a read that aligns to that particular position. A series of overlapping reads that span the entire region up to adjoining genes are shown.

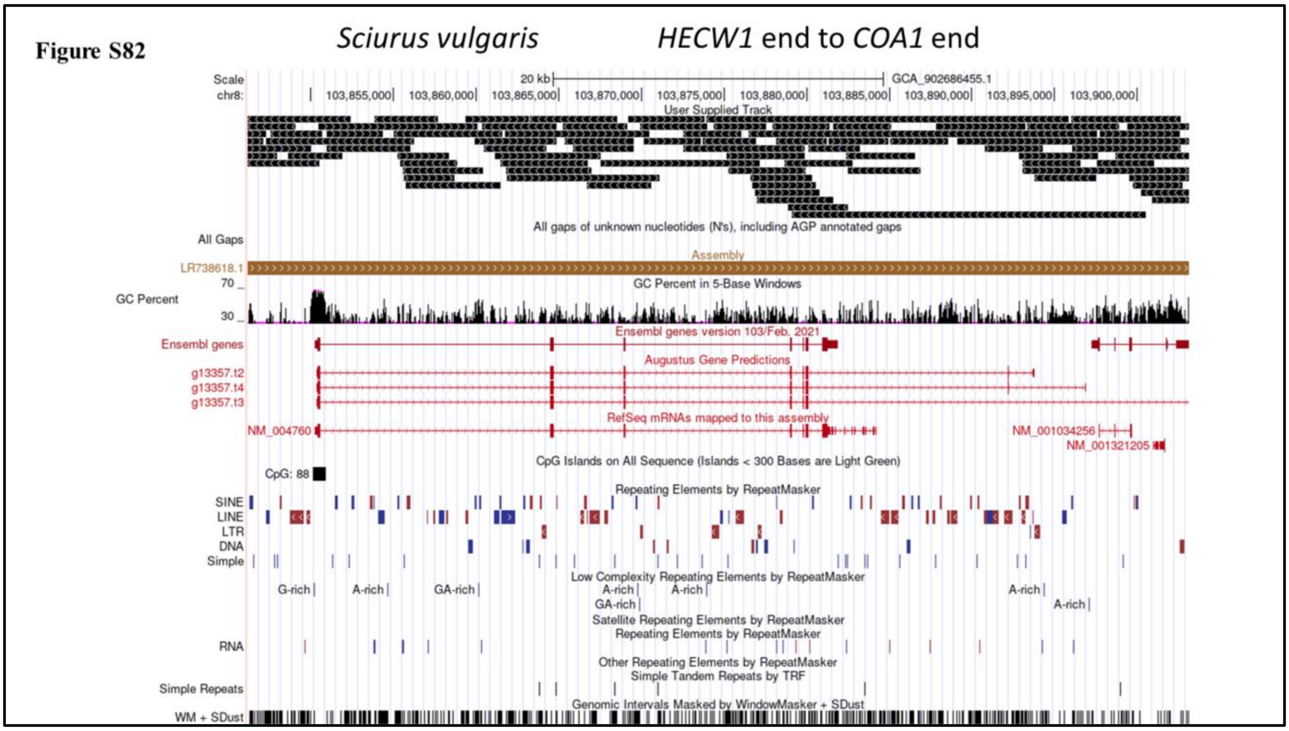

Figure S82: Screenshot of UCSC genome browser showing the alignment of > 3 kb long reads generated using PacBio technology to the Eurasian red squirrel (*Sciurus vulgaris*) **GCA_902686455.1** at the location of the *HECW1* end to *COA1* start. Each black rectangular box represents a read that aligns to that particular position. A series of overlapping reads that span the entire region up to adjoining genes are shown.

Figure S83

Sciurus vulgaris *COA1, BLVRA, UBE2D4*

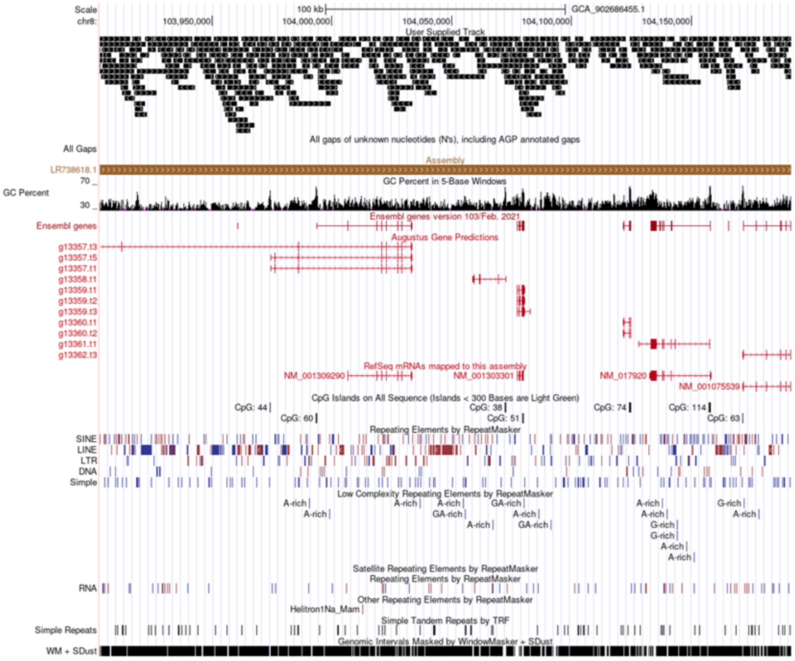

Figure S83: Screenshot of UCSC genome browser showing the alignment of > 3 kb long reads generated using PacBio technology to the Eurasian red squirrel (*Sciurus vulgaris*) **GCA_902686455.1** at the location of the *COA1* end to *UBE2D4*. Each black rectangular box represents a read that aligns to that particular position. A series of overlapping reads that span the entire region up to adjoining genes are shown.

Figure S84 *Castor canadensis* *COA1* Castor_canadensis.C.can_genome_v1.0.dna_sm.toplevel.fa

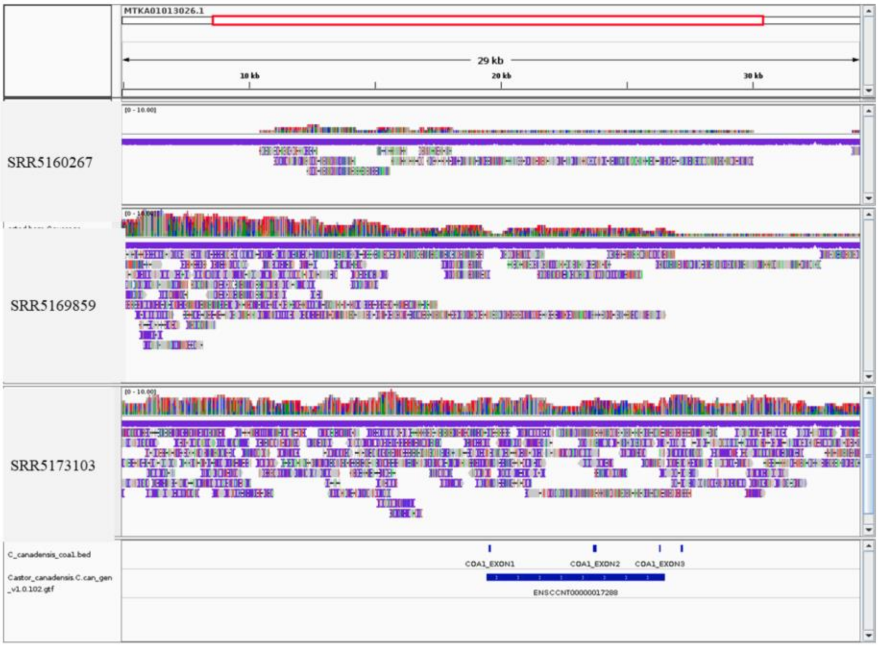

Figure S84: Screenshot of PacBio reads aligned to each of the putative locations of the *COA1* in American beaver (*Castor canadensis*). Gray boxes represent reads aligned to the genome. Raw read dataset which is mentioned above have been mapped to the Castor_canadensis.C.can_genome_v1.0.dna_sm.toplevel.fa.

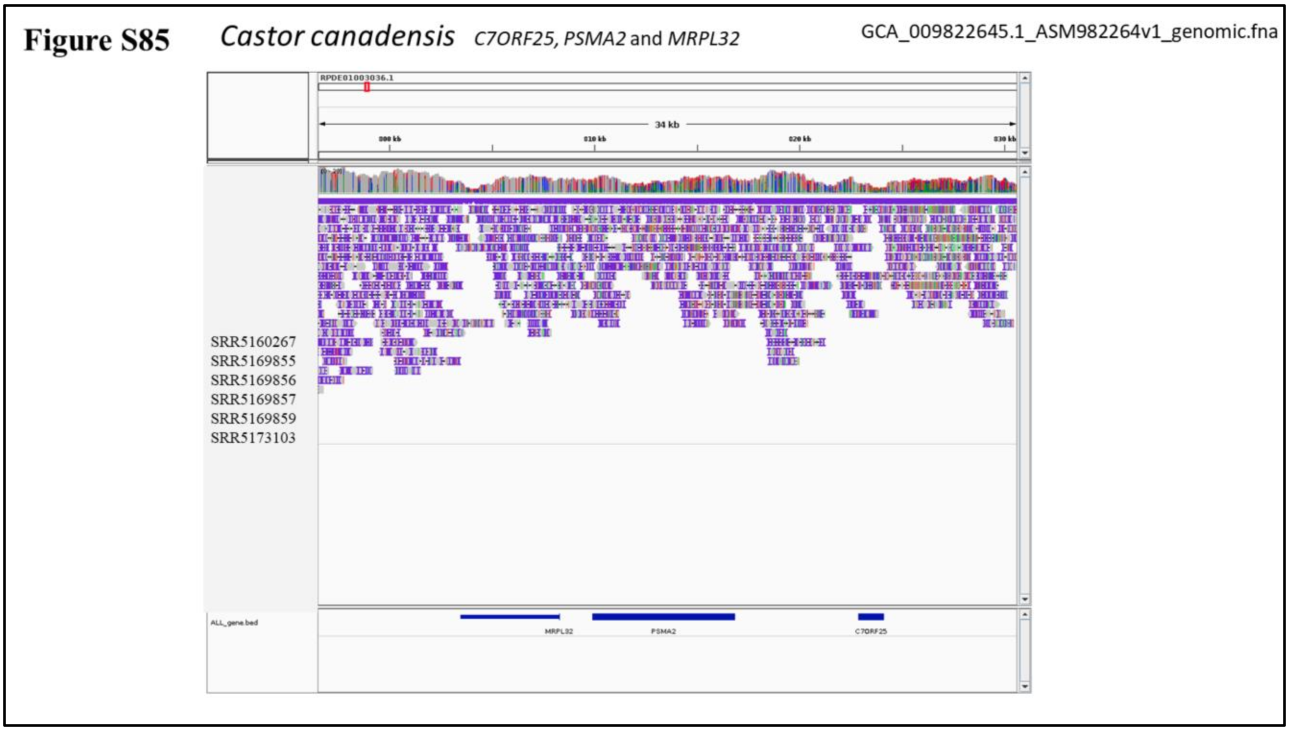

Figure S85: Screenshot of PacBio reads aligned to each of the putative locations of the *C7orf25*, *PSMA2* and *MRPL32* in American beaver (*Castor canadensis*). Gray boxes represent reads aligned to the genome. Raw read dataset which is mentioned above have been mapped to the GCA_009822645.1_ASM982264v1_genomic.fna.

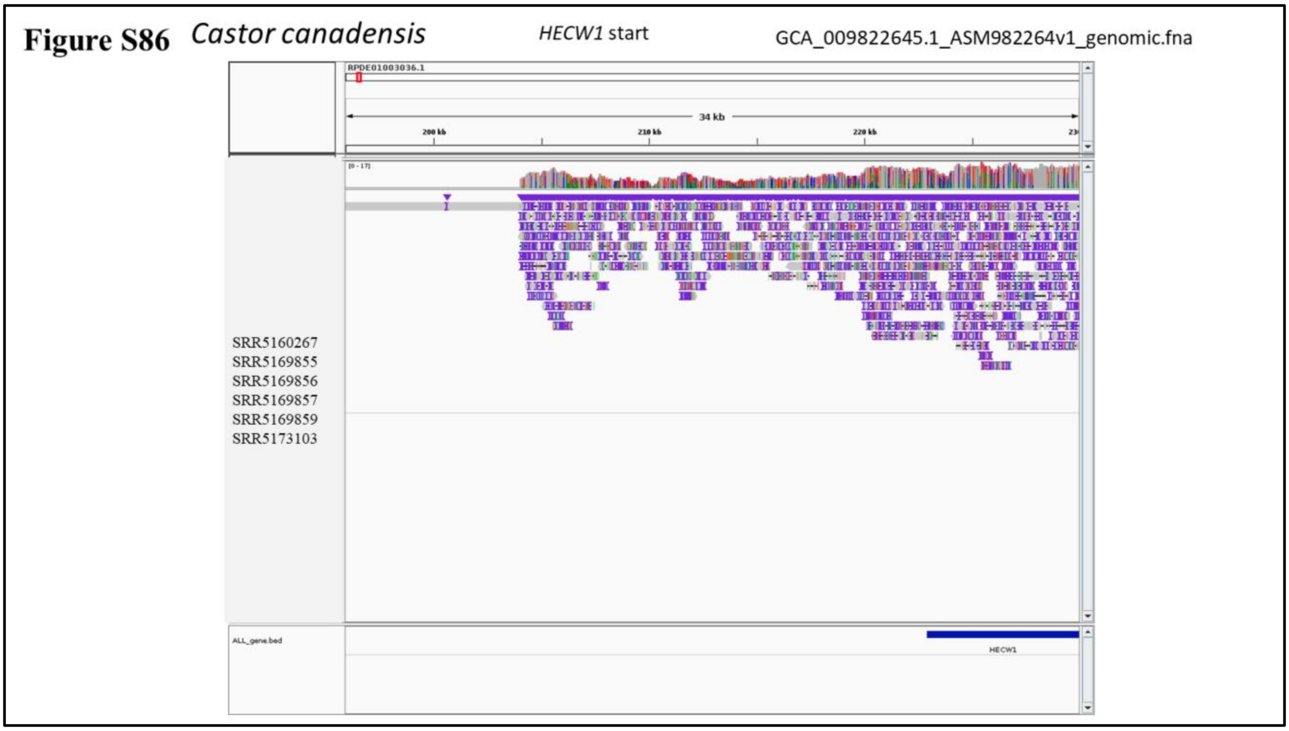

Figure S86: Screenshot of PacBio reads aligned to each of the putative locations of the *HECWI* start in American beaver (*Castor canadensis*). Gray boxes represent reads aligned to the genome. Raw read dataset which is mentioned above have been mapped to the GCA_009822645.1_ASM982264v1_genomic.fna.

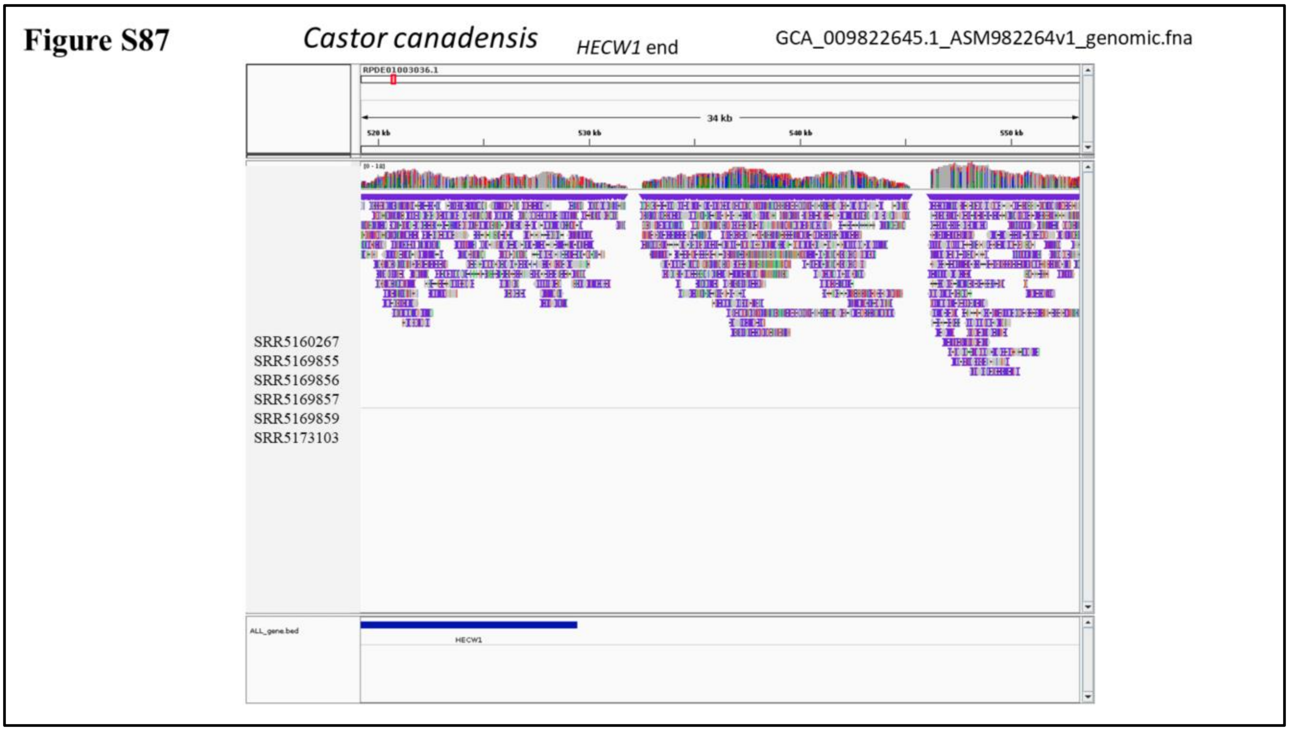

Figure S87: Screenshot of PacBio reads aligned to each of the putative locations of the *HECW1* end in American beaver (*Castor canadensis*). Gray boxes represent reads aligned to the genome. Raw read dataset which is mentioned above have been mapped to the GCA_009822645.1_ASM982264v1_genomic.fna.

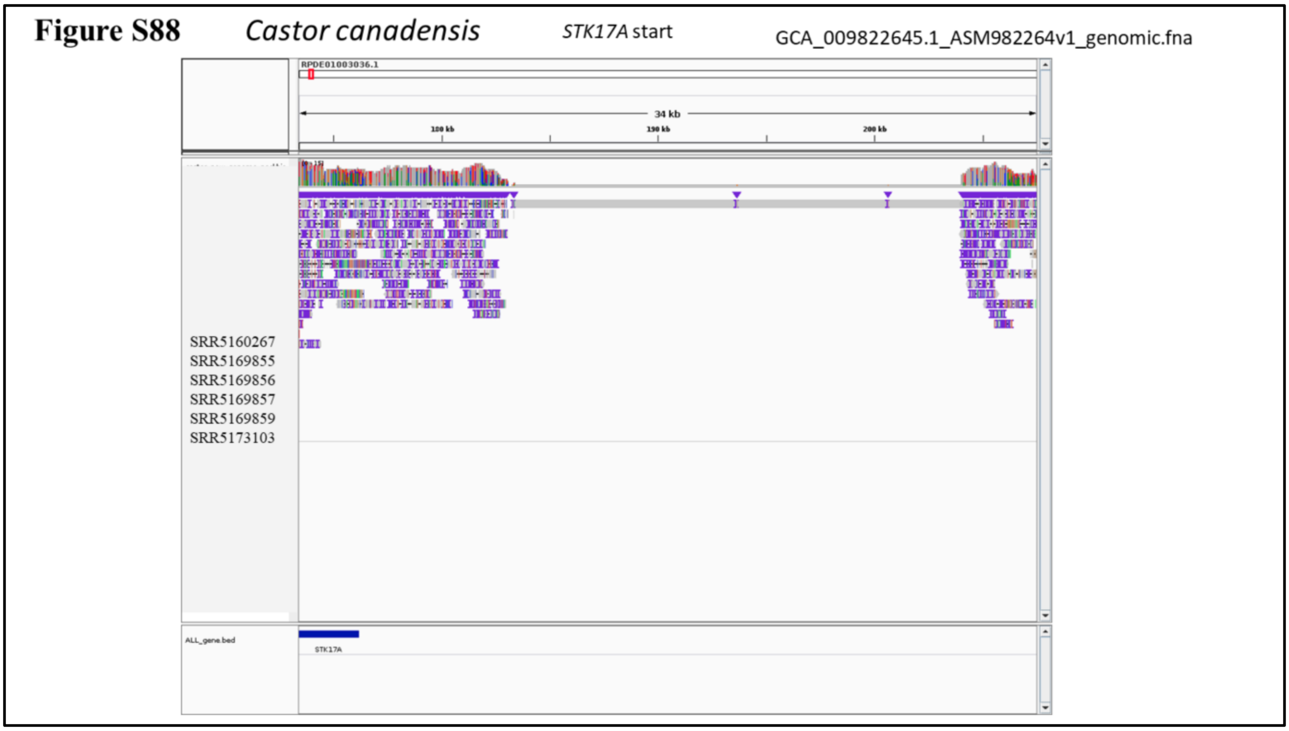

Figure S88: Screenshot of PacBio reads aligned to each of the putative locations of the *STK17A* start in American beaver (*Castor canadensis*). Gray boxes represent reads aligned to the genome. Raw read dataset which is mentioned above have been mapped to the GCA_009822645.1_ASM982264v1_genomic.fna.

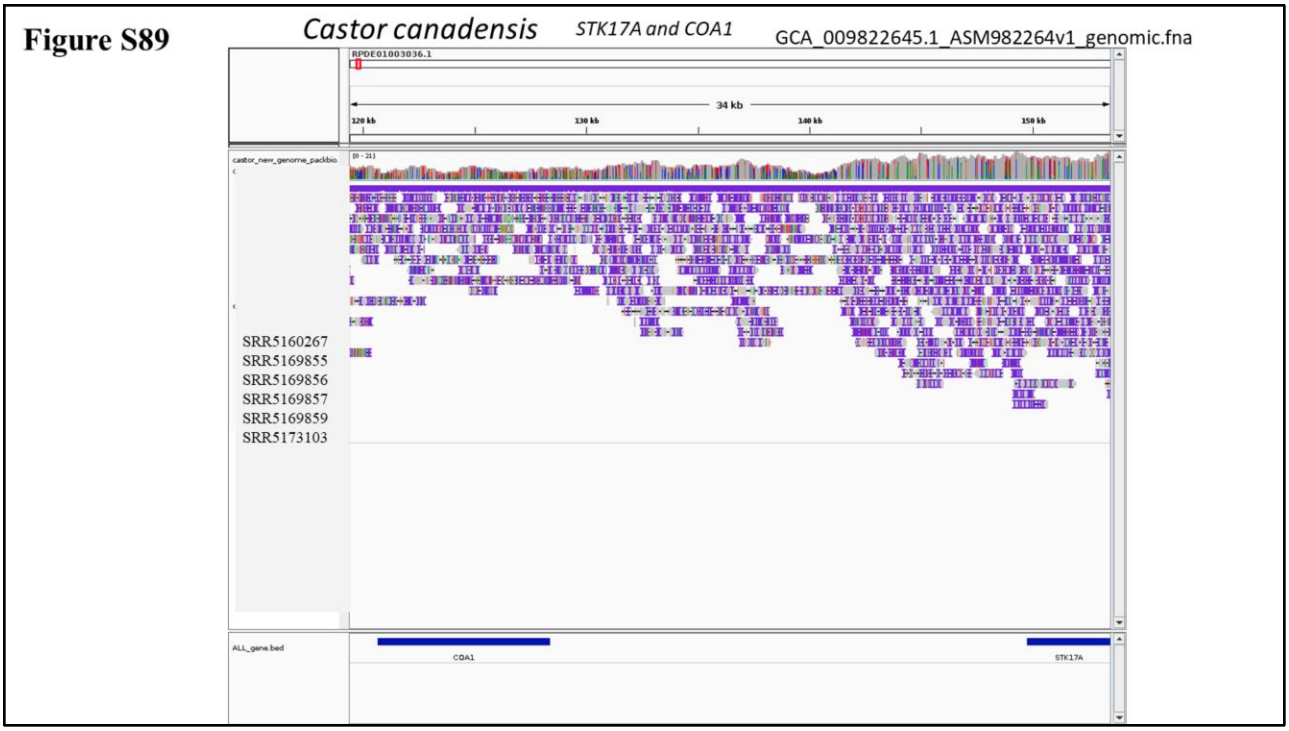

Figure S89: Screenshot of PacBio reads aligned to each of the putative locations of the *STK17A* and *COA1* in American beaver (*Castor canadensis*). Gray boxes represent reads aligned to the genome. Raw read dataset which is mentioned above have been mapped to the GCA_009822645.1_ASM982264v1_genomic.fna.

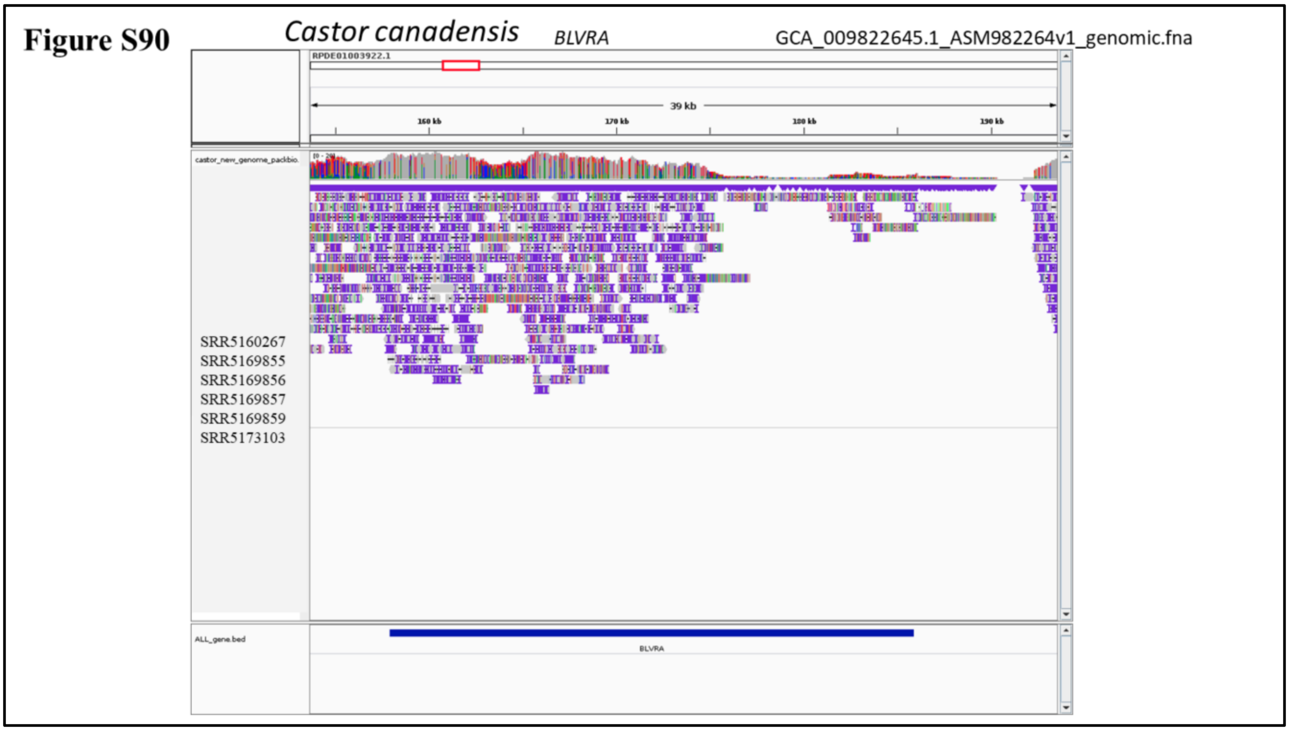

Figure S90: Screenshot of PacBio reads aligned to each of the putative locations of the *BLVRA* in American beaver (*Castor canadensis*). Gray boxes represent reads aligned to the genome. Raw read dataset which is mentioned above have been mapped to the GCA_009822645.1_ASM982264v1_genomic.fna.

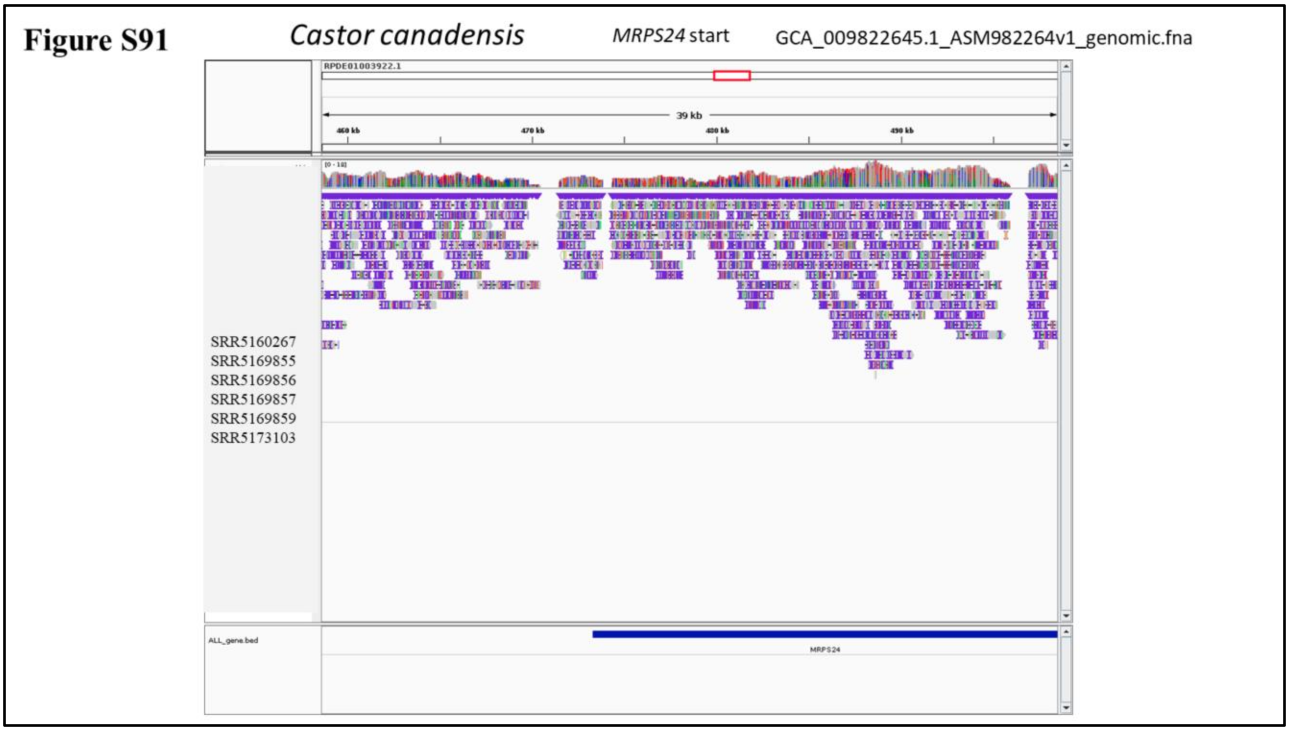

Figure S91: Screenshot of PacBio reads aligned to each of the putative locations of the *MRPS24* start in American beaver (*Castor canadensis*). Gray boxes represent reads aligned to the genome. Raw read dataset which is mentioned above have been mapped to the GCA_009822645.1_ASM982264v1_genomic.fna.

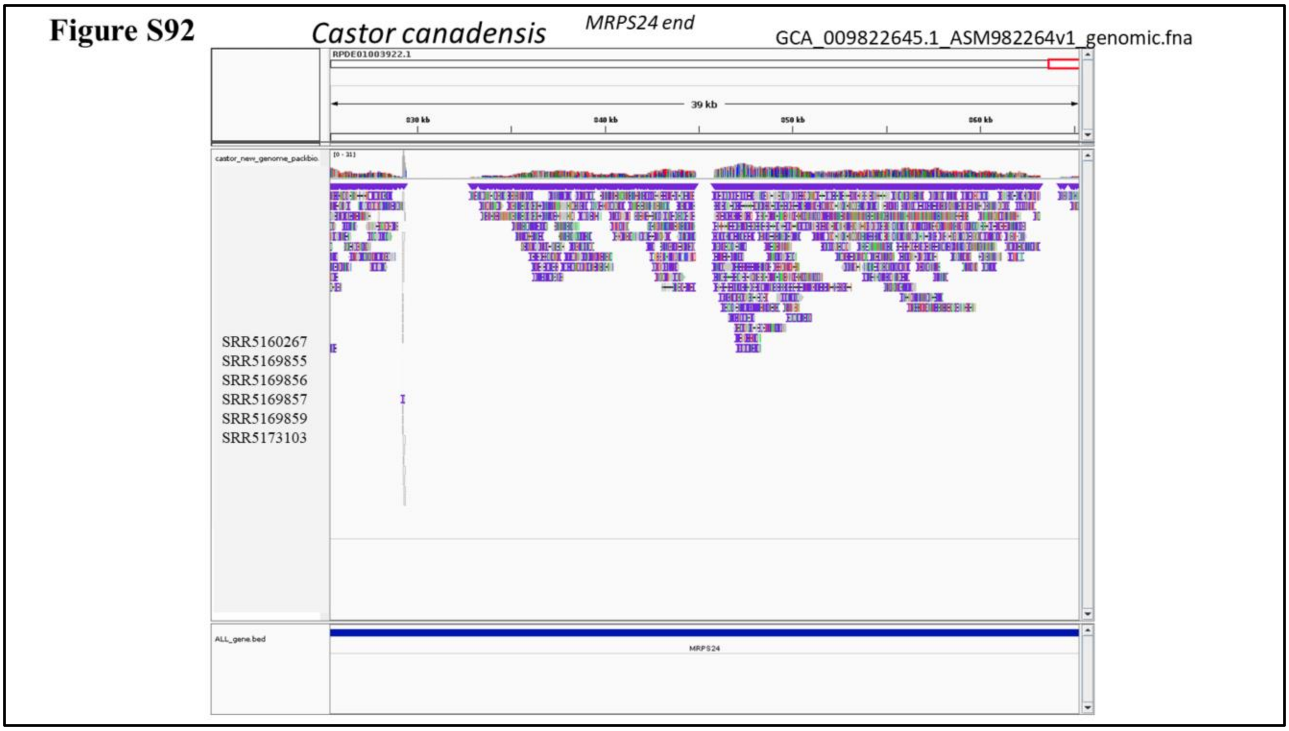

Figure S92: Screenshot of PacBio reads aligned to each of the putative locations of the *MRPS24* in American beaver (*Castor canadensis*). Gray boxes represent reads aligned to the genome. Raw read dataset which is mentioned above have been mapped to the GCA_009822645.1_ASM982264v1_genomic.fna.

Figure S93 Castor canadensis URGCP GCA_009822645.1_ASM982264v1_genomic.fna

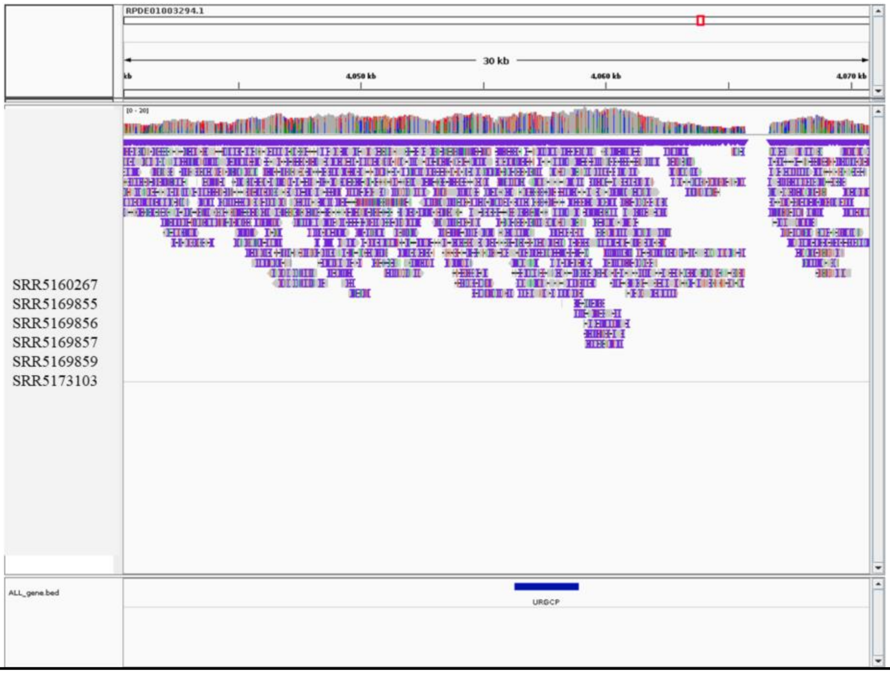

Figure S93: Screenshot of PacBio reads aligned to each of the putative locations of the *URGCP* in American beaver (*Castor canadensis*). Gray boxes represent reads aligned to the genome. Raw read dataset which is mentioned above have been mapped to the GCA_009822645.1_ASM982264v1_genomic.fna.

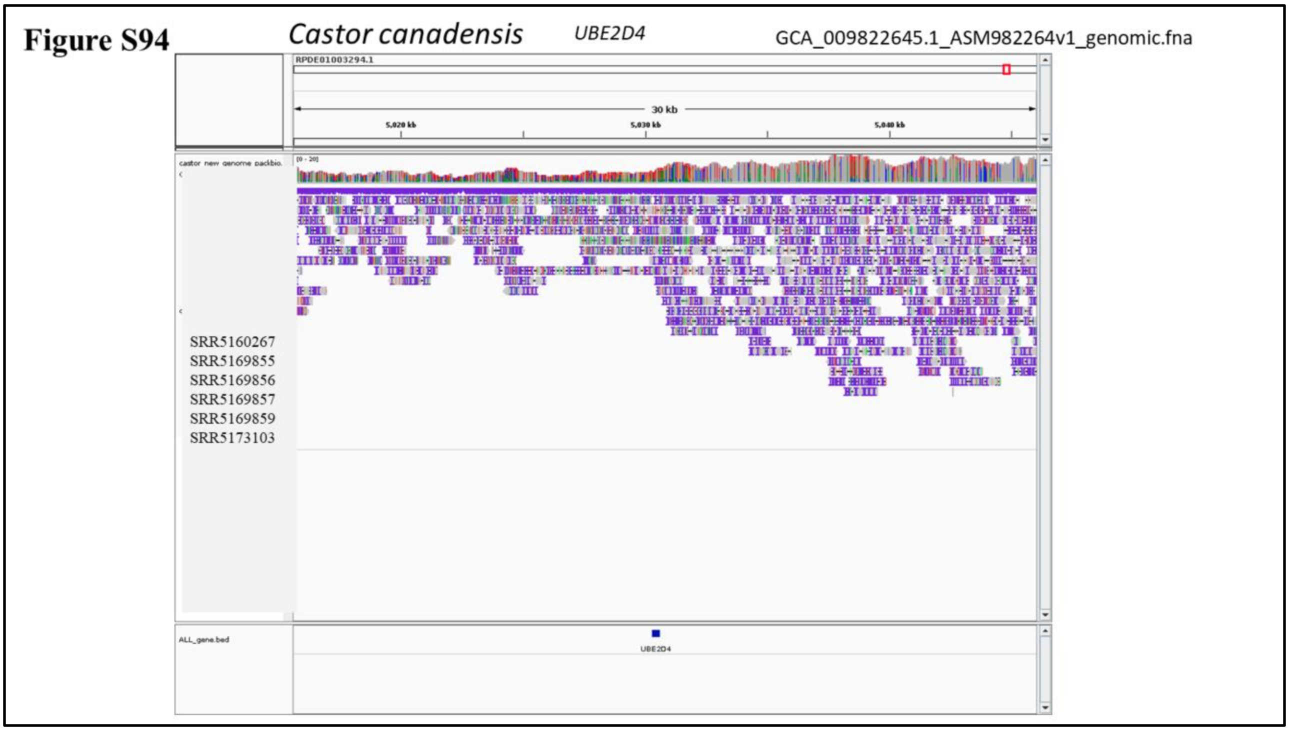

Figure S94: Screenshot of PacBio reads aligned to each of the putative locations of the *UBE2D4* in American beaver (*Castor canadensis*). Gray boxes represent reads aligned to the genome. Raw read dataset which is mentioned above have been mapped to the GCA_009822645.1_ASM982264v1_genomic.fna.

Figure S95 *Heterocephalus glaber* *AZGP1* to *C7ORF25* (*COA1* syntenic region)

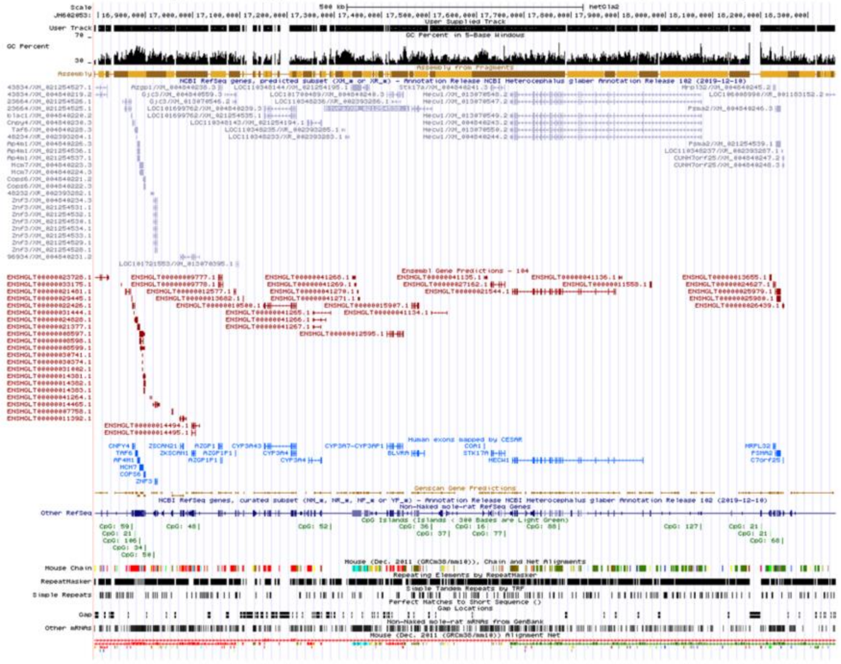

Figure S95: Screenshot of UCSC genome browser showing the alignment of > 3 kb long reads generated using PacBio technology to the naked mole-rat (*Heterocephalus glaber*) **GCA_000247695.1** at the location of the *AZGP1* to *C7orf25* (*COA1* syntenic region). Each black rectangular box represents a read that aligns to that particular position. A series of overlapping reads that span the entire region up to adjoining genes are shown.

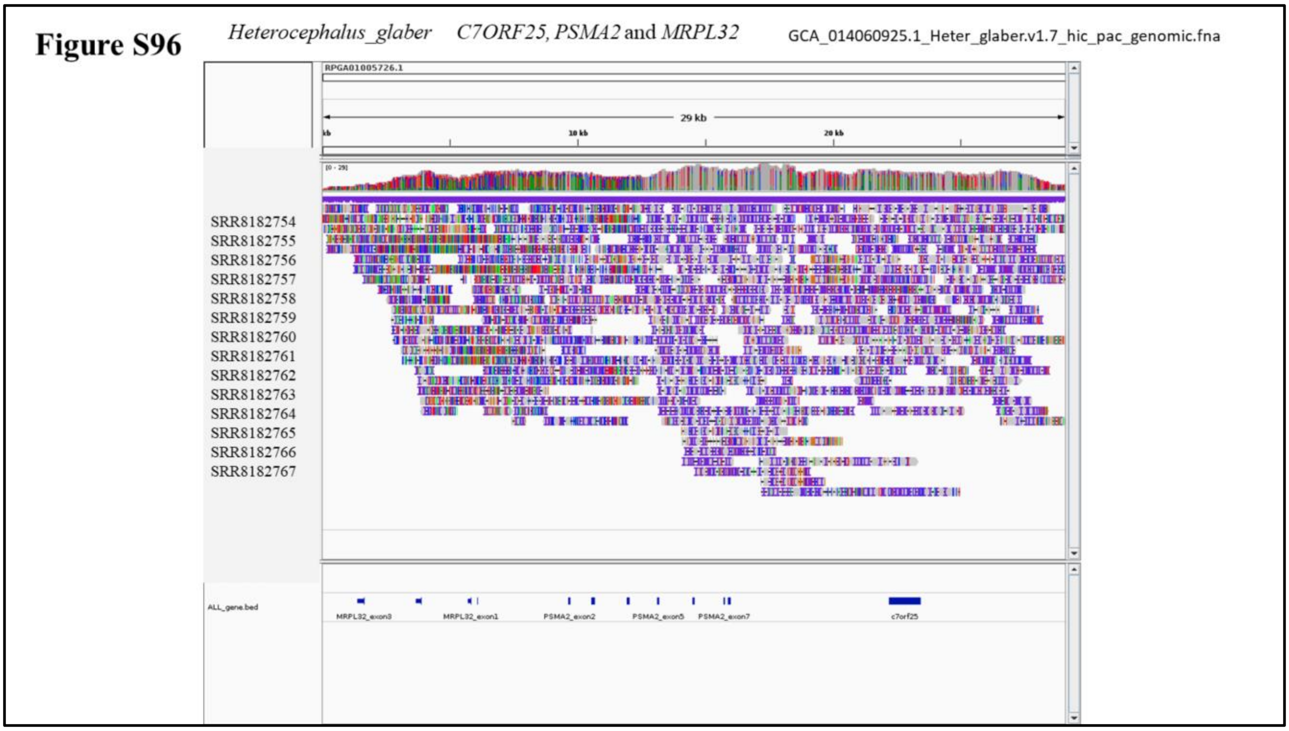

Figure S96: Screenshot of PacBio reads aligned to each of the putative locations of the *C7orf25*, *PSMA2* and *MRPL32* in naked mole-rat (*Heterocephalus glaber*). Gray boxes represent reads aligned to the genome. Raw read dataset which is mentioned above have been mapped to the GCA_014060925.1_Heter_glaber.v1.7_hic_pac_genomic.fna.

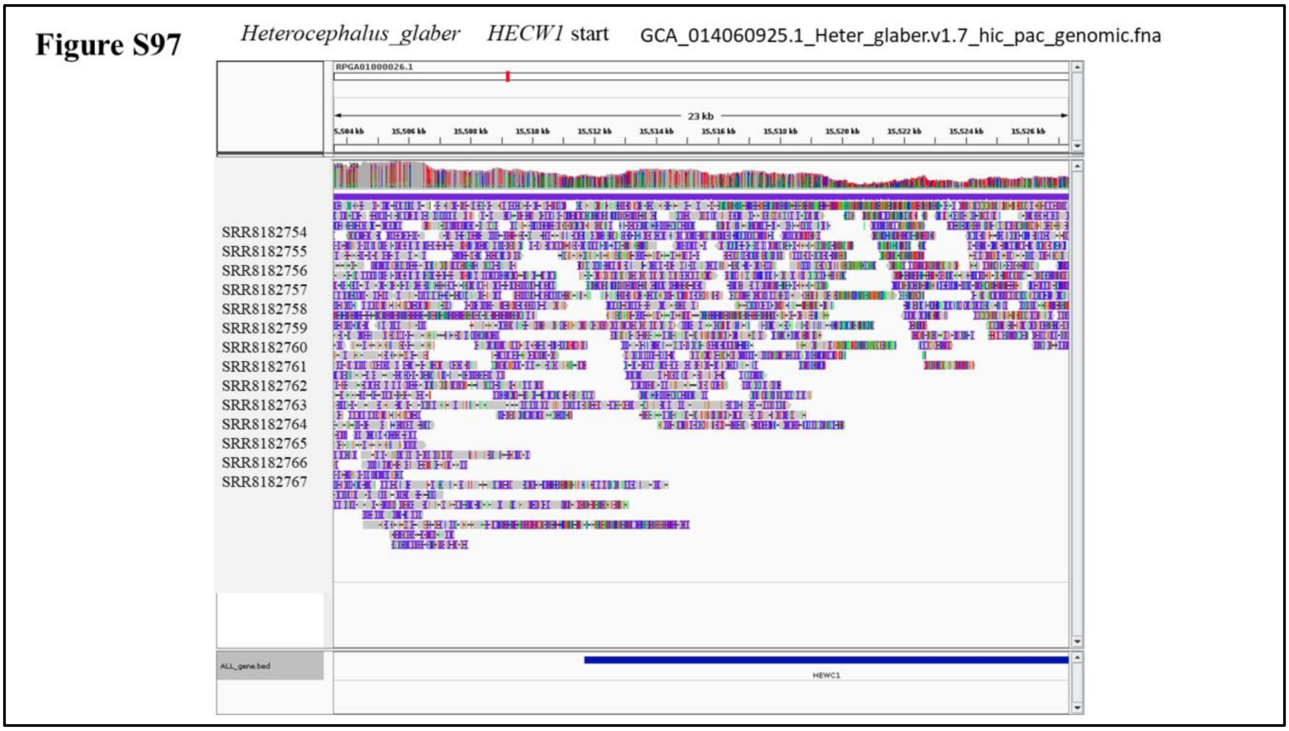

Figure S97: Screenshot of PacBio reads aligned to each of the putative locations of the *HECW1* start in naked mole-rat (*Heterocephalus glaber*). Gray boxes represent reads aligned to the genome. Raw read dataset which is mentioned above have been mapped to the GCA_014060925.1_Heter_glaber.v1.7_hic_pac_genomic.fna.

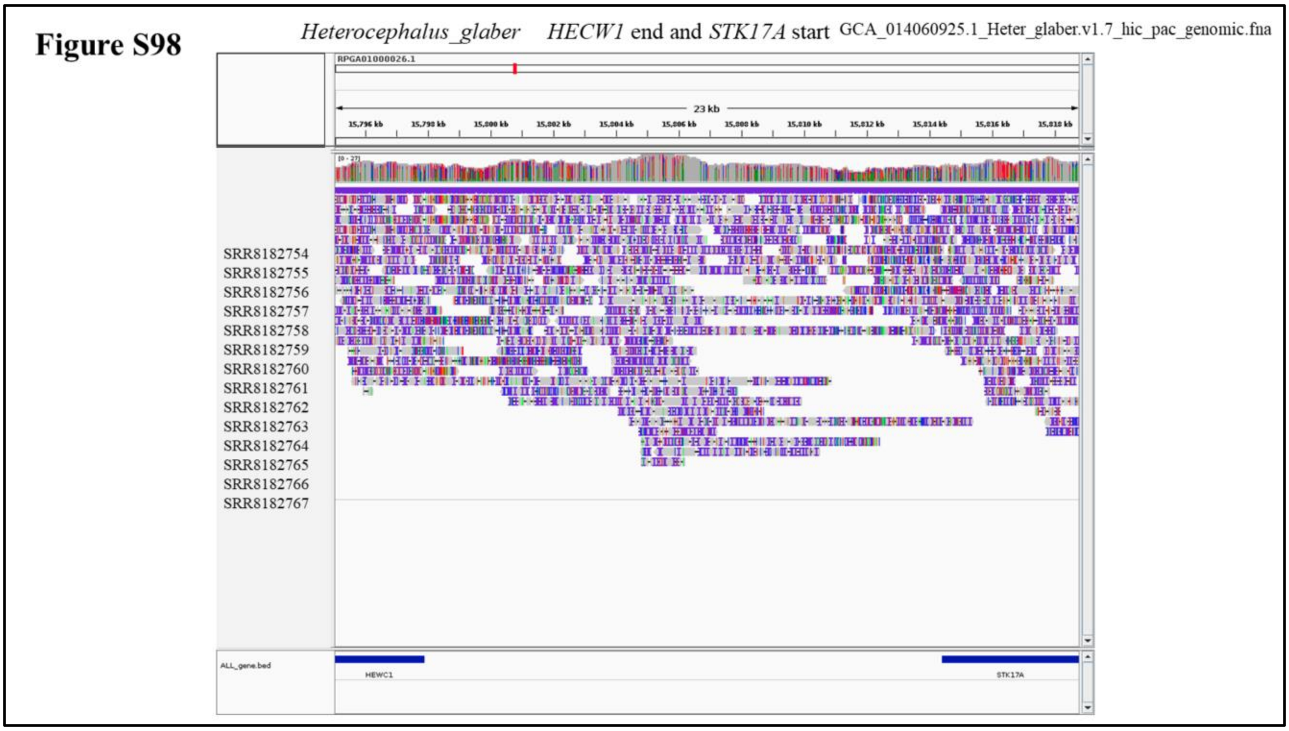

Figure S98: Screenshot of PacBio reads aligned to each of the putative locations of the *HECW1* and *STK17A* in naked mole-rat (*Heterocephalus glaber*). Gray boxes represent reads aligned to the genome. Raw read dataset which is mentioned above have been mapped to the GCA_014060925.1_Heter_glaber.v1.7_hic_pac_genomic.fna.

Figure S99 *Heterocephalus_glaber* *STK17A* and *COA1* GCA_014060925.1_Heter_glaber.v1.7_hic_pac_genomic.fna

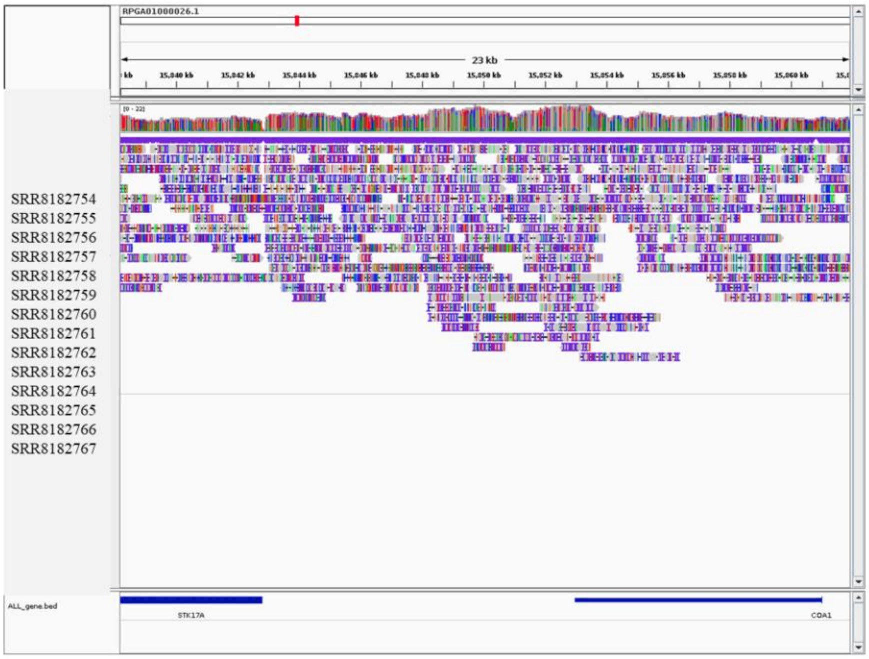

Figure S99: Screenshot of PacBio reads aligned to each of the putative locations of the *STK17A* and *COA1* in naked mole-rat (*Heterocephalus glaber*). Gray boxes represent reads aligned to the genome. Raw read dataset which is mentioned above have been mapped to the GCA_014060925.1_Heter_glaber.v1.7_hic_pac_genomic.fna.

Figure S100 *Heterocephalus_glaber* *BLVRA* GCA_014060925.1_Heter_glaber.v1.7_hic_pac_genomic.fna

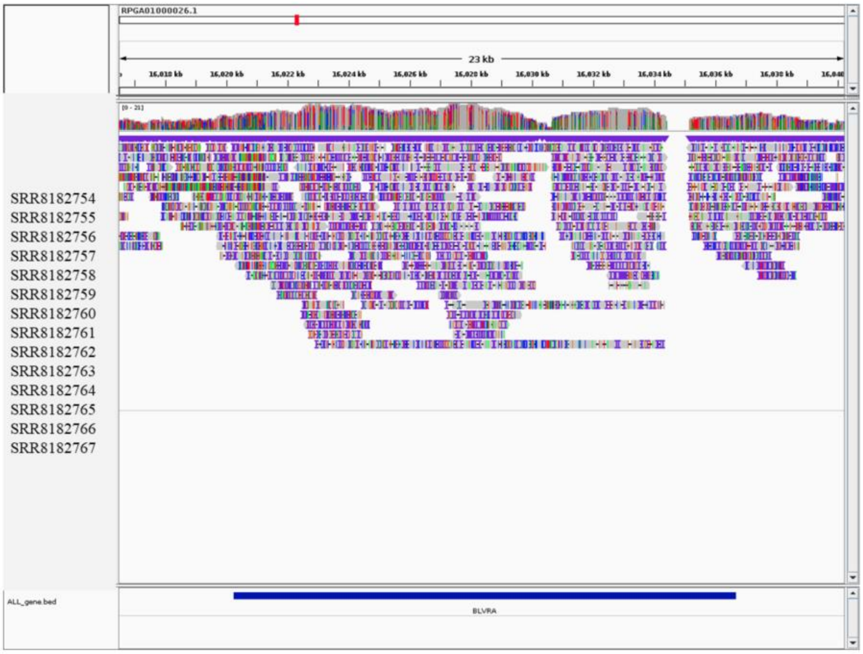

Figure S100: Screenshot of PacBio reads aligned to each of the putative locations of the *BLVRA* in naked mole-rat (*Heterocephalus glaber*). Gray boxes represent reads aligned to the genome. Raw read dataset which is mentioned above have been mapped to the GCA_014060925.1_Heter_glaber.v1.7_hic_pac_genomic.fna.

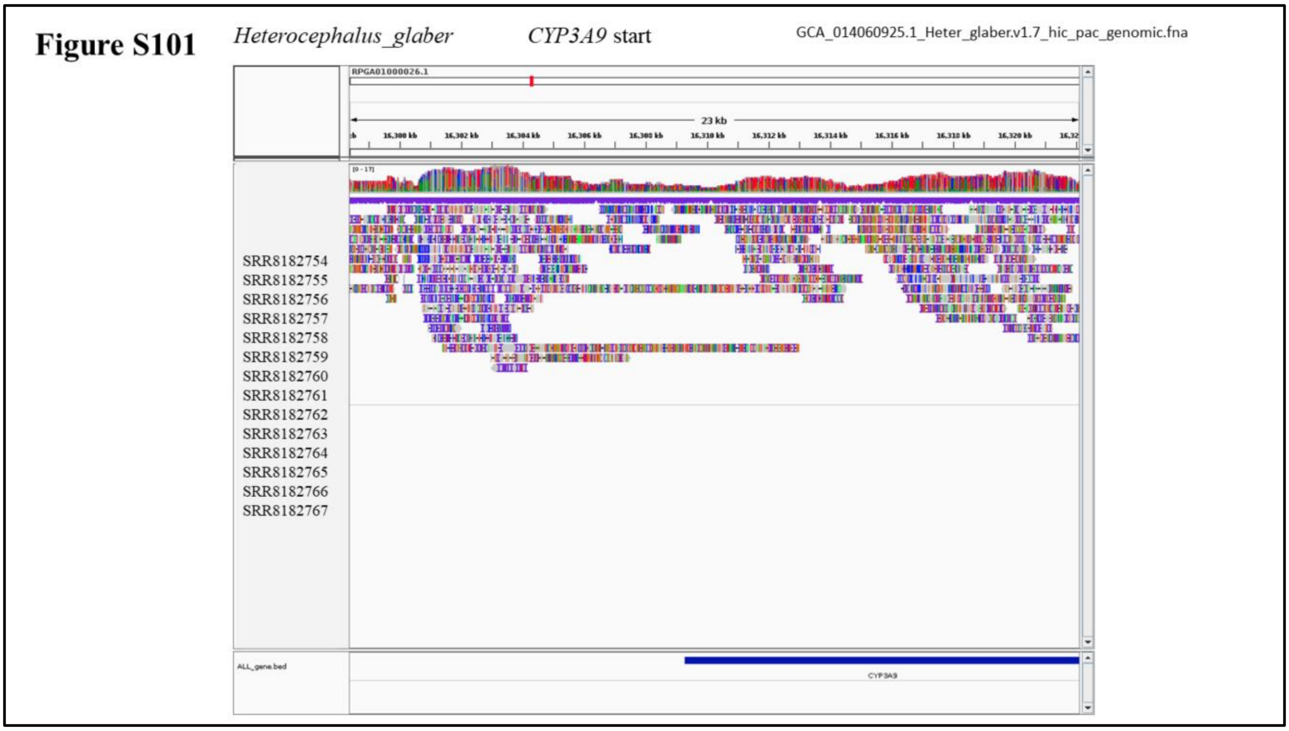

Figure S101: Screenshot of PacBio reads aligned to each of the putative locations of the *CYP2A9* start in naked mole-rat (*Heterocephalus glaber*). Gray boxes represent reads aligned to the genome. Raw read dataset which is mentioned above have been mapped to the GCA_014060925.1_Heter_glaber.v1.7_hic_pac_genomic.fna.

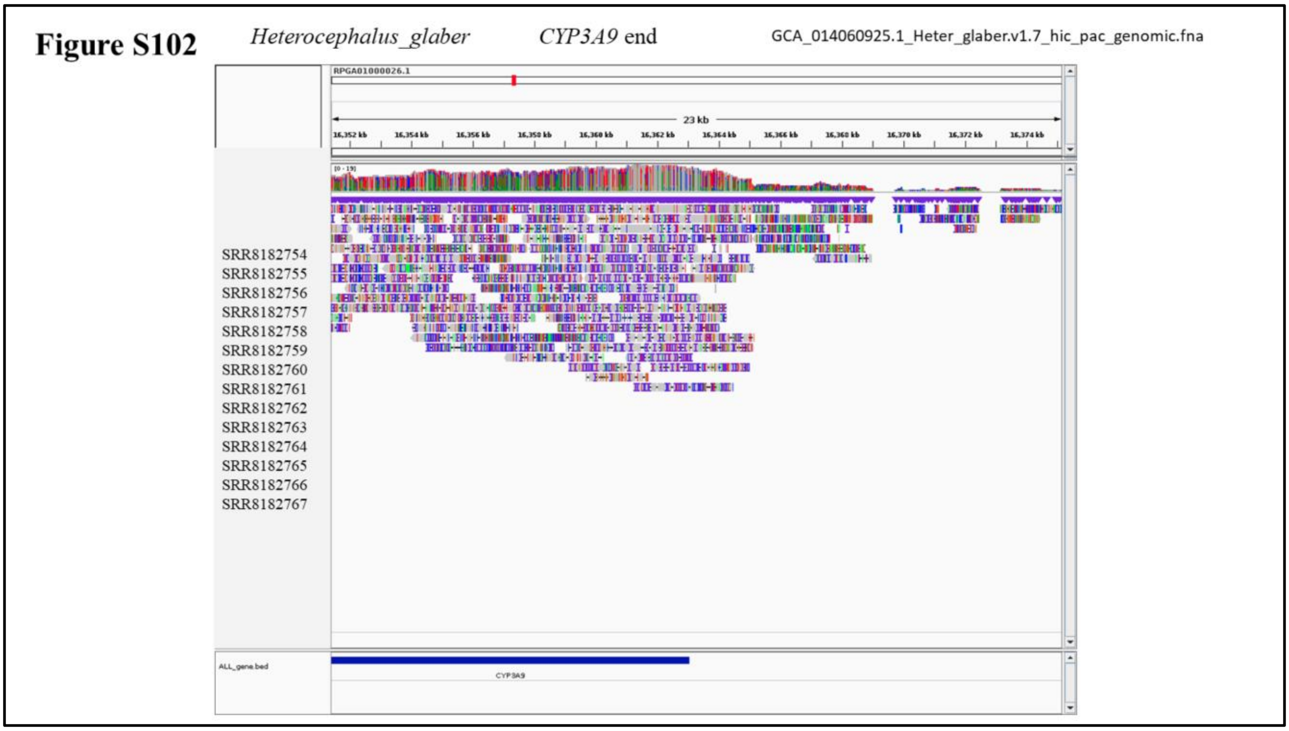

Figure S102: Screenshot of PacBio reads aligned to each of the putative locations of the *CYP3A9* end in naked mole-rat (*Heterocephalus glaber*). Gray boxes represent reads aligned to the genome. Raw read dataset which is mentioned above have been mapped to the GCA_014060925.1_Heter_glaber.v1.7_hic_pac_genomic.fna.

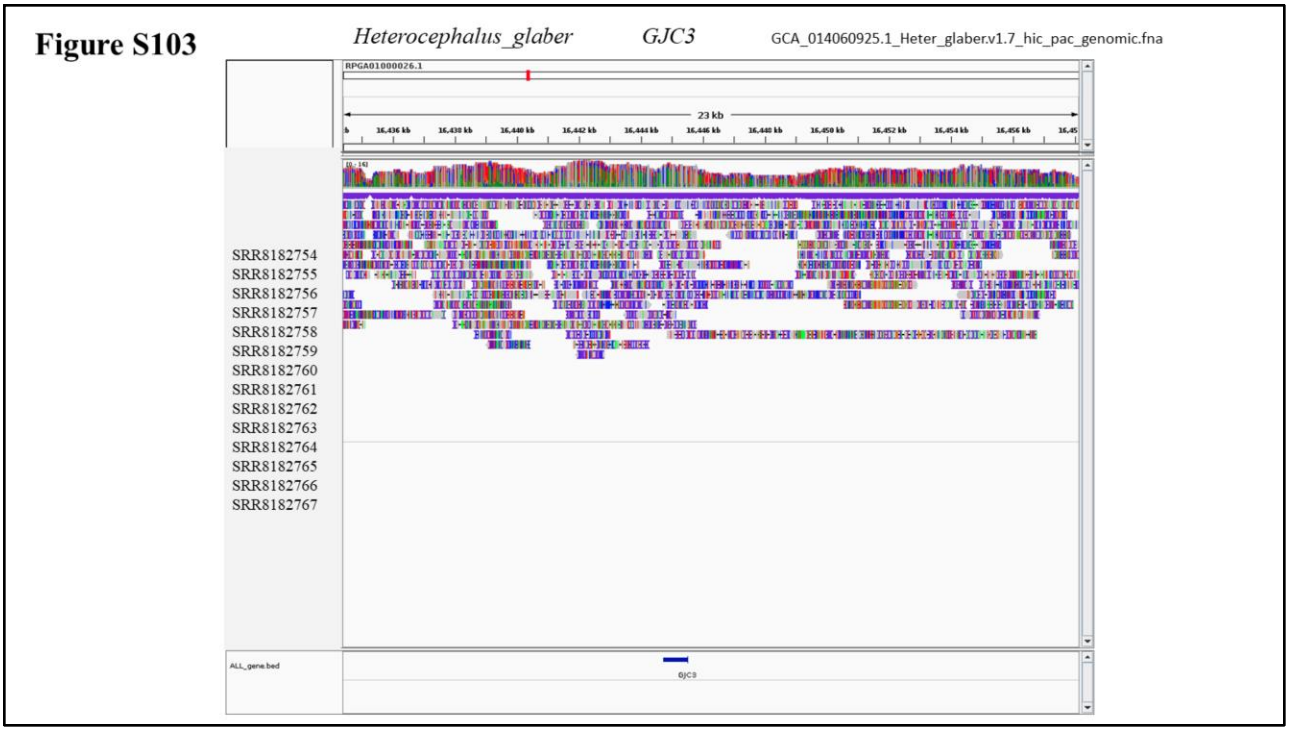

Figure S103: Screenshot of PacBio reads aligned to each of the putative locations of the *GJC3* in naked mole-rat (*Heterocephalus glaber*). Gray boxes represent reads aligned to the genome. Raw read dataset which is mentioned above have been mapped to the GCA_014060925.1_Heter_glaber.v1.7_hic_pac_genomic.fna.

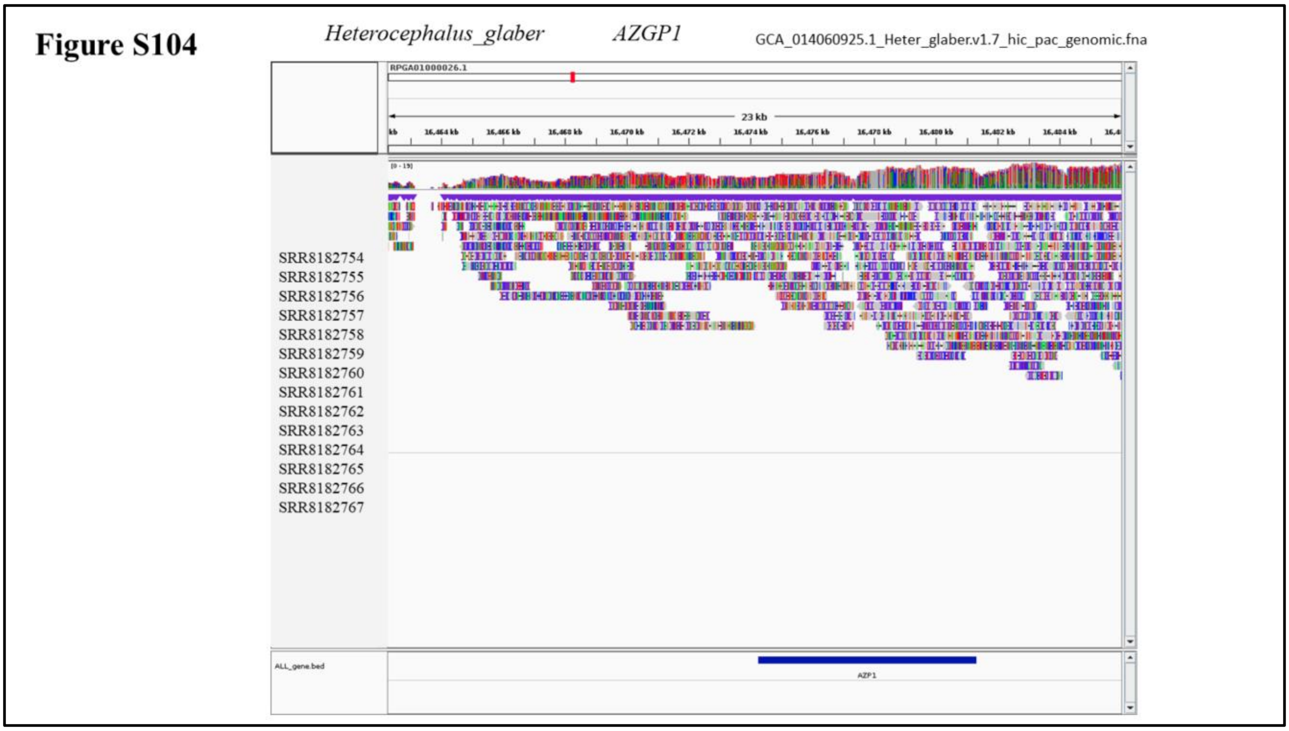

Figure S104: Screenshot of PacBio reads aligned to each of the putative locations of the *AZGP1* in naked mole-rat (*Heterocephalus glaber*). Gray boxes represent reads aligned to the genome. Raw read dataset which is mentioned above have been mapped to the GCA_014060925.1_Heter_glaber.v1.7_hic_pac_genomic.fna.

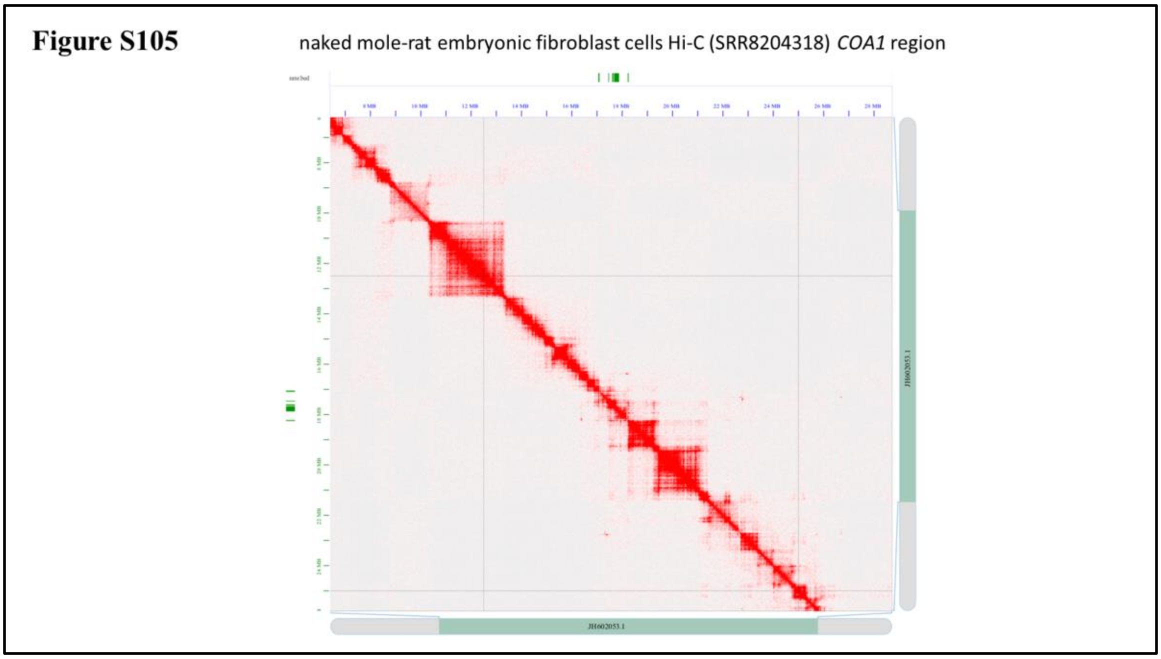

Figure S105: Hi-C figure generated using Juicebox of naked mole-rat embryonic fibroblast cells data (SRR8204318) where red dots show the genomic loci interaction between adjacent regions and gap shows there is no interaction between the nearby region. We loaded the bed file on the top and left sides of the Hi-C plot, where each line of the green color indicates the gene of interest (*COA1)* and adjacent syntenic regions. chromosome or scaffold is mentioned on the bottom and right sides of the plot.

Figure S106 naked mole-rat embryonic fibroblast cells Hi-C (SRR8204318) *COA1* duplicate region

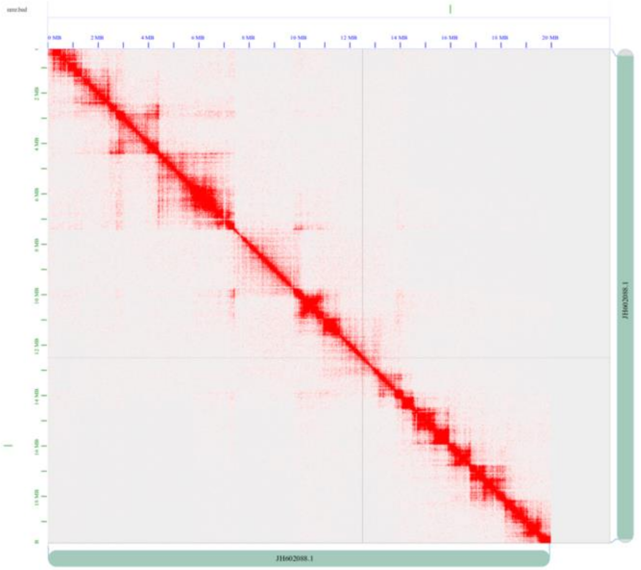

Figure S106: Hi-C figure generated using Juicebox of naked mole-rat embryonic fibroblast cells data (SRR8204318) where red dots show the genomic loci interaction between adjacent regions and gap shows there is no interaction between the nearby region. We loaded the bed file on the top and left sides of the Hi-C plot, where the green color indicates the gene of interest (*COA1* duplicate) and adjacent syntenic regions. chromosome or scaffold is mentioned on the bottom and right sides of the plot.

Figure S107

Cheetah Blood Hi-C (SRR8616936) *COA1* region

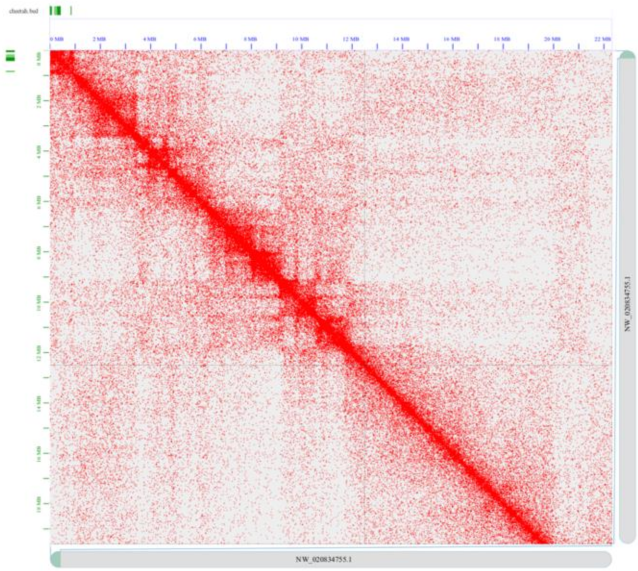

Figure S107: Hi-C figure generated using Juicebox of cheetah blood data (SRR8616936) where red dots show the genomic loci interaction between adjacent regions and gap shows there is no interaction between the nearby region. We loaded the bed file on the top and left sides of the Hi-C plot, where each line of the green color indicates the gene of interest (*COA1)* and adjacent syntenic regions. chromosome or scaffold is mentioned on the bottom and right sides of the plot.

Figure S108

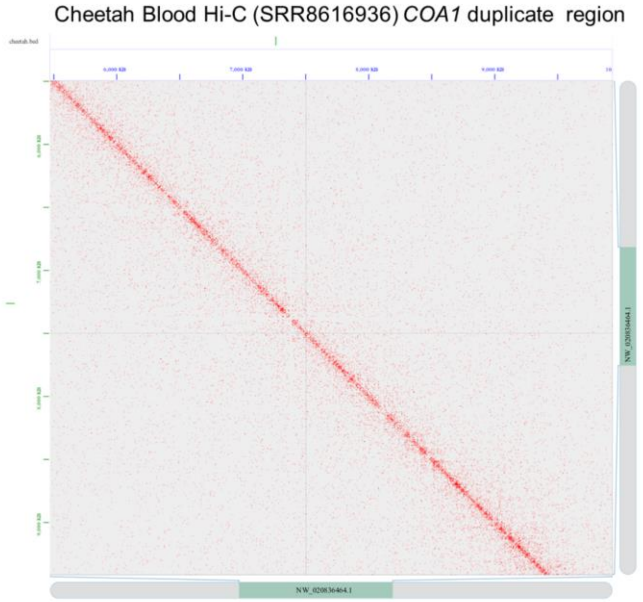

Figure S108: Hi-C figure generated using Juicebox of Cheetah blood data (SRR8616936) where red dots show the genomic loci interaction between adjacent regions and gap shows there is no interaction between the nearby region. We loaded the bed file on the top and left sides of the Hi-C plot, where the green color indicates the gene of interest (*COA1* duplicate*)* and adjacent syntenic regions. chromosome or scaffold is mentioned on the bottom and right sides of the plot.

Figure S109

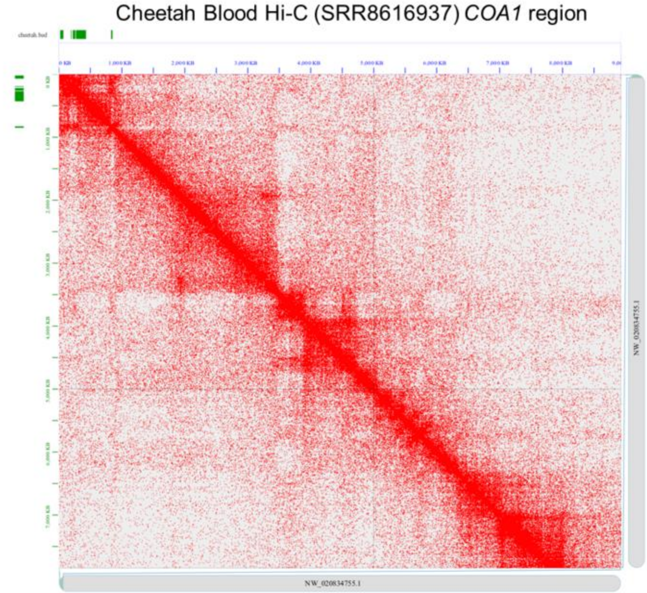

Figure S109: Hi-C figure generated using Juicebox of Cheetah blood data (SRR8616937) where red dots show the genomic loci interaction between adjacent regions and gap shows there is no interaction between the nearby region. We loaded the bed file on the top and left sides of the Hi-C plot, where each line of the green color indicates the gene of interest (*COA1)* and adjacent syntenic regions. chromosome or scaffold is mentioned on the bottom and right sides of the plot.

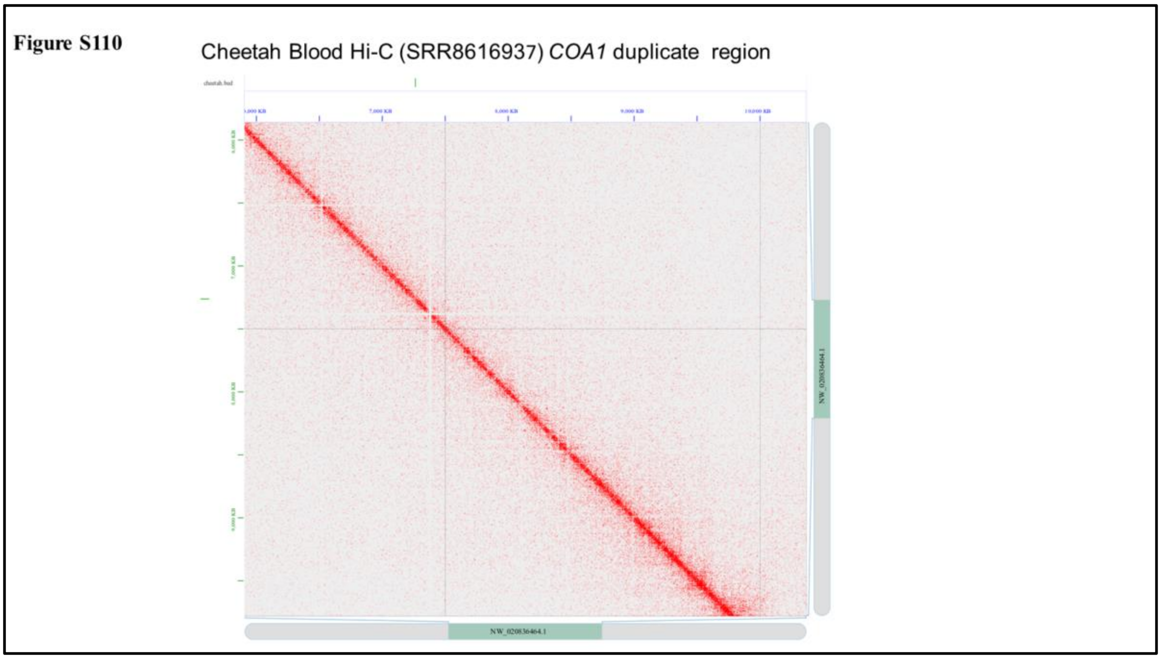

Figure S110: Hi-C figure generated using Juicebox of Cheetah blood data (SRR8616937) where red dots show the genomic loci interaction between adjacent regions and gap shows there is no interaction between the nearby region. We loaded the bed file on the top and left sides of the Hi-C plot, where the green color indicates the gene of interest (*COA1* duplicate) and adjacent syntenic regions. chromosome or scaffold is mentioned on the bottom and right sides of the plot.

Figure S111 dog Liver Hi-C (SRR1771330) *COA1* region

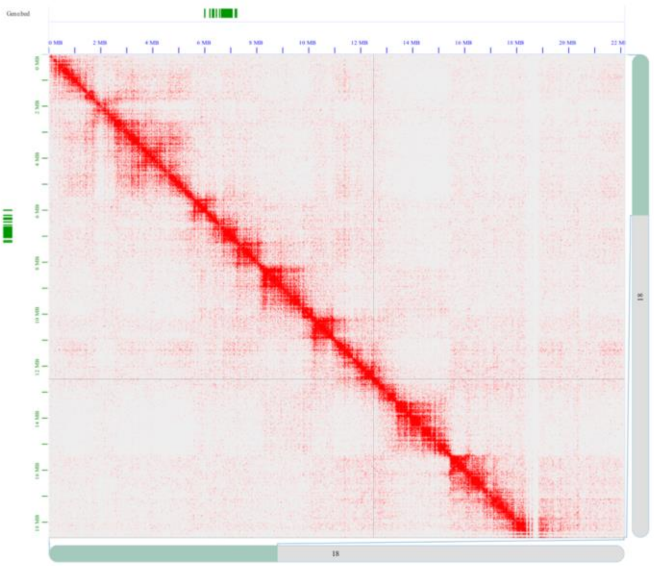

Figure S111: Hi-C figure generated using Juicebox of dog liver data (SRR1771330) where red dots show the genomic loci interaction between adjacent regions and gap shows there is no interaction between the nearby region. We loaded the bed file on the top and left sides of the Hi-C plot, where each line of the green color indicates the gene of interest (*COA1)* and adjacent syntenic regions. chromosome or scaffold is mentioned on the bottom and right sides of the plot.

Figure S112 dog Liver Hi-C (SRR1771330) *COA1* duplicate region

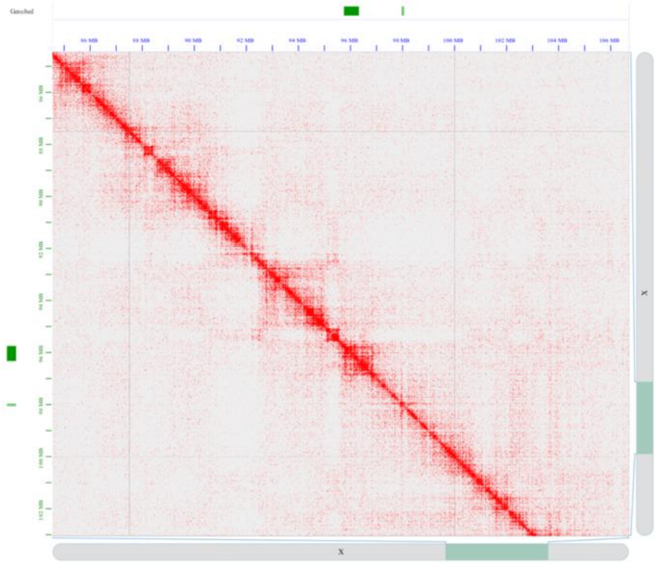

Figure S112: Hi-C figure generated using Juicebox of dog liver data (SRR1771330) where red dots show the genomic loci interaction between adjacent regions and gap shows there is no interaction between the nearby region. We loaded the bed file on the top and left sides of the Hi-C plot, where the green color indicates the gene of interest (*COA1* duplicate) and adjacent syntenic regions. chromosome or scaffold is mentioned on the bottom and right sides of the plot.

Figure S113 dog Liver Hi-C (SRR1771331) *COA1* region

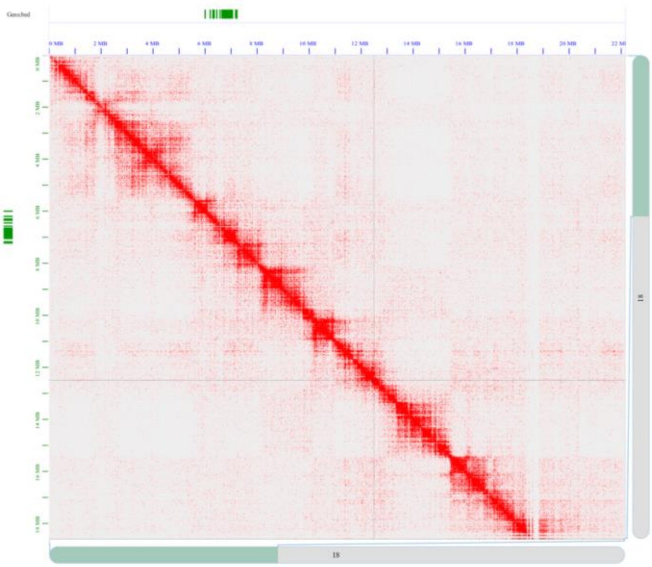

Figure S113: Hi-C figure generated using Juicebox of dog liver data (SRR1771331) where red dots show the genomic loci interaction between adjacent regions and gap shows there is no interaction between the nearby region. We loaded the bed file on the top and left sides of the Hi-C plot, where each line of the green color indicates the gene of interest (*COA1)* and adjacent syntenic regions. chromosome or scaffold is mentioned on the bottom and right sides of the plot.

Figure S114 dog Liver Hi-C (SRR1771331) *COA1* duplicate region

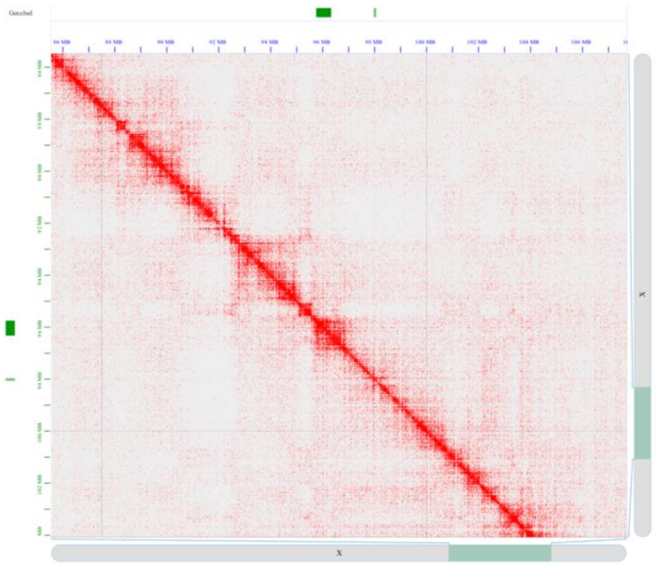

Figure S114: Hi-C figure generated using Juicebox of dog liver data (SRR1771331) where red dots show the genomic loci interaction between adjacent regions and gap shows there is no interaction between the nearby region. We loaded the bed file on the top and left sides of the Hi-C plot, where the green color indicates the gene of interest (*COA1* duplicate*)* and adjacent syntenic regions. chromosome or scaffold is mentioned on the bottom and right sides of the plot.

Figure S115 dog Liver Hi-C (SRR1771332) *COA1* region

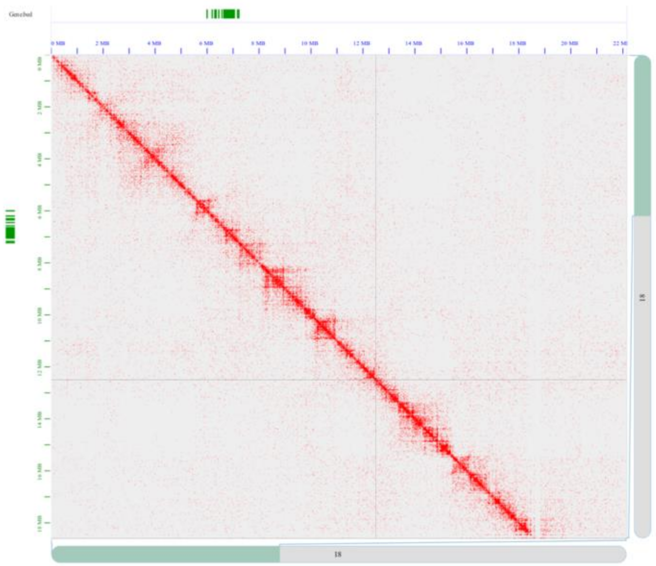

Figure 115: Hi-C figure generated using Juicebox of dog liver data (SRR1771332) where red dots show the genomic loci interaction between adjacent regions and gap shows there is no interaction between the nearby region. We loaded the bed file on the top and left sides of the Hi-C plot, where each line of the green color indicates the gene of interest (*COA1)* and adjacent syntenic regions. chromosome or scaffold is mentioned on the bottom and right sides of the plot.

Figure S116 dog Liver Hi-C (SRR1771332) *COA1* duplicate region

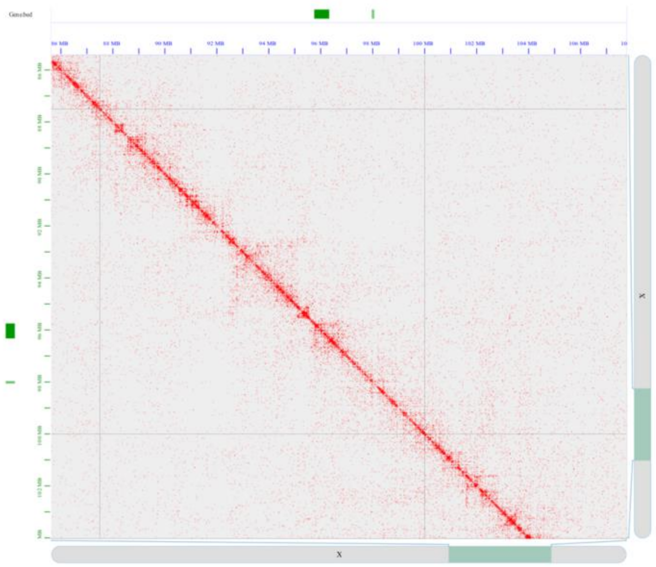

Figure S116: Hi-C figure generated using Juicebox of dog liver data (SRR1771332) where red dots show the genomic loci interaction between adjacent regions and gap shows there is no interaction between the nearby region. We loaded the bed file on the top and left sides of the Hi-C plot, where the green color indicates the gene of interest (*COA1* duplicate*)* and adjacent syntenic regions. chromosome or scaffold is mentioned on the bottom and right sides of the plot.

Figure S117 dog Liver Hi-C (SRR1771333) *COA1* region

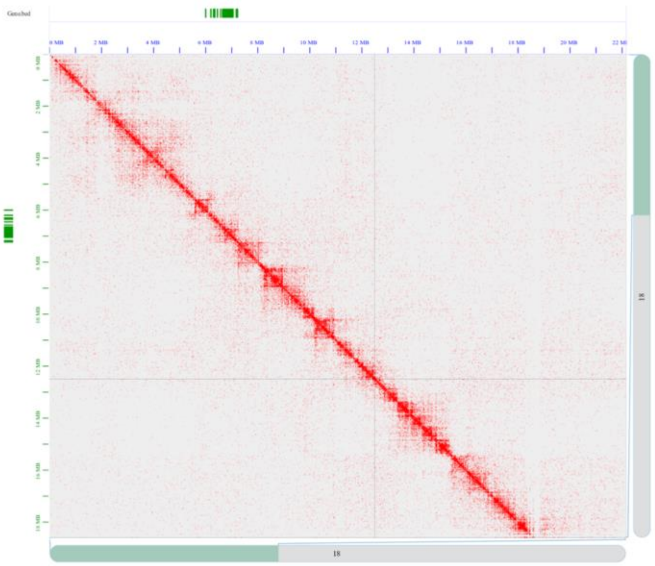

Figure 117: Hi-C figure generated using Juicebox of dog liver data (SRR1771333) where red dots show the genomic loci interaction between adjacent regions and gap shows there is no interaction between the nearby region. We loaded the bed file on the top and left sides of the Hi-C plot, where each line of the green color indicates the gene of interest (*COA1)* and adjacent syntenic regions. chromosome or scaffold is mentioned on the bottom and right sides of the plot.

Figure S118

dog Liver Hi-C (SRR1771333) *COA1* duplicate region

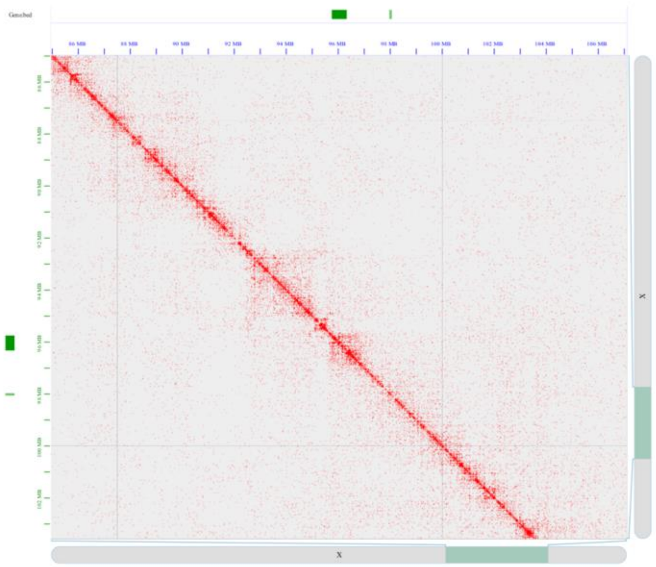

Figure S118: Hi-C figure generated using Juicebox of dog liver data (SRR1771333) where red dots show the genomic loci interaction between adjacent regions and gap shows there is no interaction between the nearby region. We loaded the bed file on the top and left sides of the Hi-C plot, where the green color indicates the gene of interest (*COA1* duplicate*)* and adjacent syntenic regions. chromosome or scaffold is mentioned on the bottom and right sides of the plot.

Figure S119 dog Liver Hi-C (SRR1771334) *COA1* region

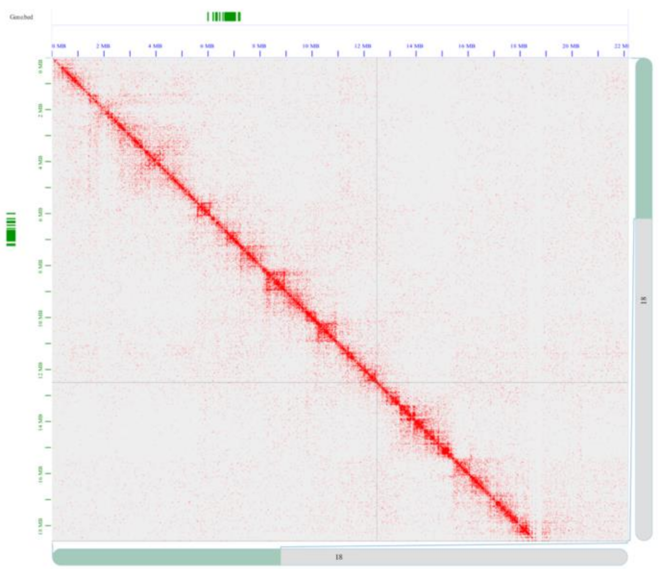

Figure S119: Hi-C figure generated using Juicebox of dog liver data (SRR1771334) where red dots show the genomic loci interaction between adjacent regions and gap shows there is no interaction between the nearby region. We loaded the bed file on the top and left sides of the Hi-C plot, where each line of the green color indicates the gene of interest (*COA1)* and adjacent syntenic regions. chromosome or scaffold is mentioned on the bottom and right sides of the plot.

Figure S120 dog Liver Hi-C (SRR1771334) *COA1* duplicate region

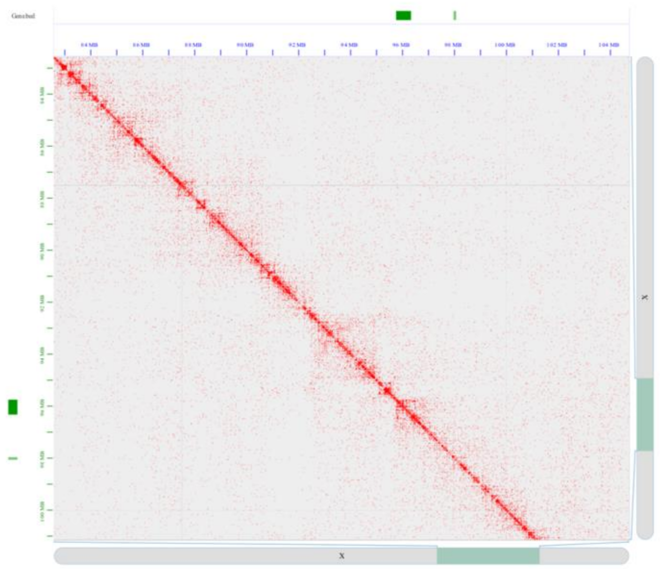

Figure S120: Hi-C figure generated using Juicebox of dog liver data (SRR1771334) where red dots show the genomic loci interaction between adjacent regions and gap shows there is no interaction between the nearby region. We loaded the bed file on the top and left sides of the Hi-C plot, where the green color indicates the gene of interest (*COA1* duplicate*)* and adjacent syntenic regions. chromosome or scaffold is mentioned on the bottom and right sides of the plot.

Figure S121 dog Liver Hi-C (SRR1771335) *COA1* region

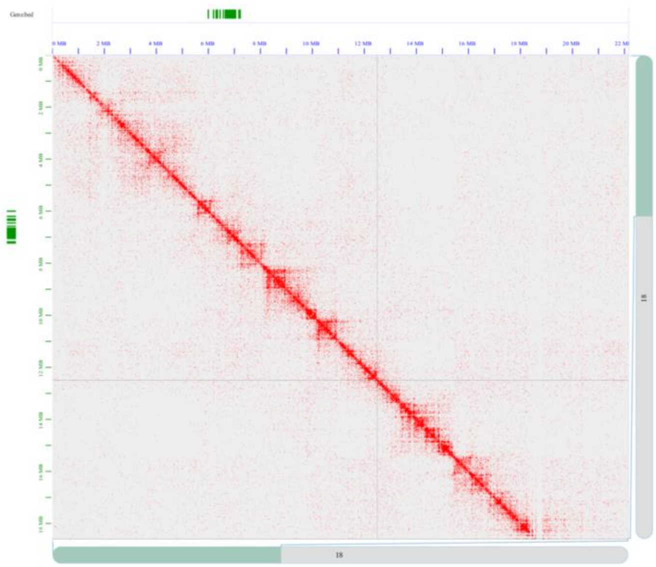

Figure S121: Hi-C figure generated using Juicebox of dog liver data (SRR1771335) where red dots show the genomic loci interaction between adjacent regions and gap shows there is no interaction between the nearby region. We loaded the bed file on the top and left sides of the Hi-C plot, where each line of the green color indicates the gene of interest (*COA1)* and adjacent syntenic regions. chromosome or scaffold is mentioned on the bottom and right sides of the plot.

Figure S122 dog Liver Hi-C (SRR1771335) *COA1* duplicate region

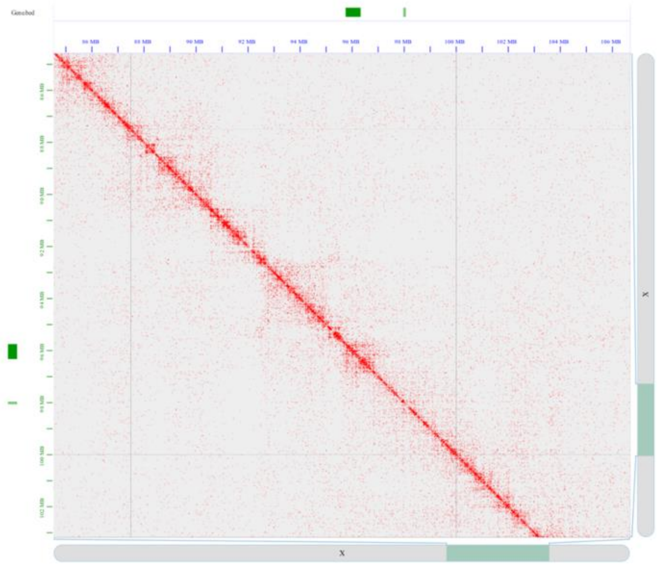

Figure S122: Hi-C figure generated using Juicebox of dog liver data (SRR1771335) where red dots show the genomic loci interaction between adjacent regions and gap shows there is no interaction between the nearby region. We loaded the bed file on the top and left sides of the Hi-C plot, where the green color indicates the gene of interest (*COA1* duplicate*)* and adjacent syntenic regions. chromosome or scaffold is mentioned on the bottom and right sides of the plot.

Figure S123 dog Liver Hi-C (SRR1771336) *COA1* region

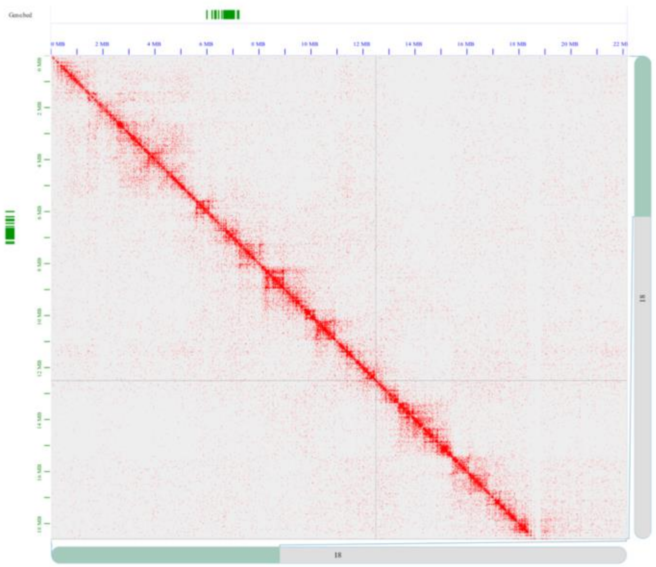

Figure S123: Hi-C figure generated using Juicebox of dog liver data (SRR1771336) where red dots show the genomic loci interaction between adjacent regions and gap shows there is no interaction between the nearby region. We loaded the bed file on the top and left sides of the Hi-C plot, where each line of the green color indicates the gene of interest (*COA1)* and adjacent syntenic regions. chromosome or scaffold is mentioned on the bottom and right sides of the plot.

Figure S124

dog Liver Hi-C (SRR1771336) *COA1* duplicate region

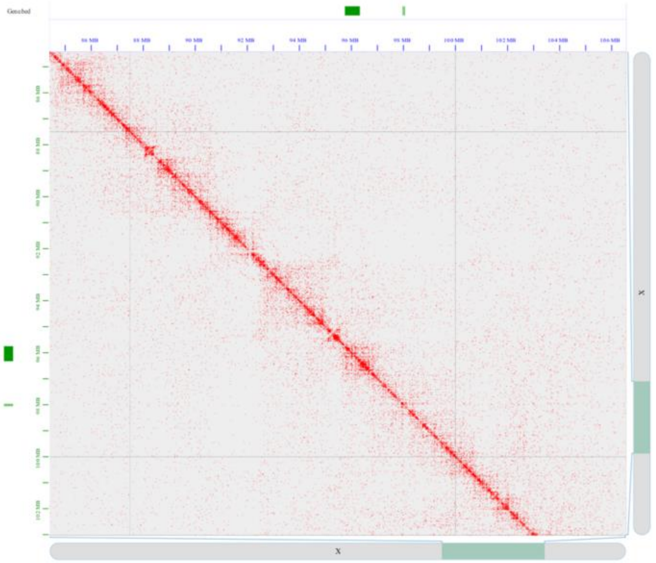

Figure S124: Hi-C figure generated using Juicebox of dog liver data (SRR1771336) where red dots show the genomic loci interaction between adjacent regions and gap shows there is no interaction between the nearby region. We loaded the bed file on the top and left sides of the Hi-C plot, where the green color indicates the gene of interest (*COA1* duplicate) and adjacent syntenic regions. chromosome or scaffold is mentioned on the bottom and right sides of the plot.

Figure S125 dog Liver Hi-C (SRR1771337) *COA1* region

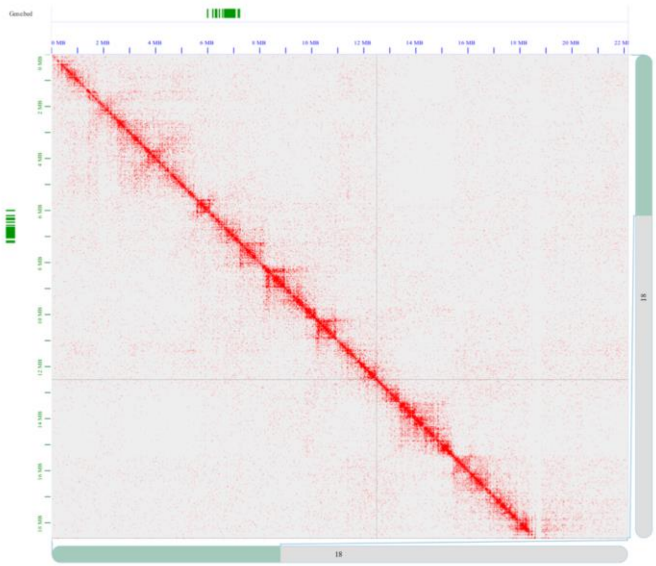

Figure S125: Hi-C figure generated using Juicebox of dog liver data (SRR1771337) where red dots show the genomic loci interaction between adjacent regions and gap shows there is no interaction between the nearby region. We loaded the bed file on the top and left sides of the Hi-C plot, where each line of the green color indicates the gene of interest (*COA1)* and adjacent syntenic regions. chromosome or scaffold is mentioned on the bottom and right sides of the plot.

Figure S126 dog Liver Hi-C (SRR1771337*) COA1* duplicate region

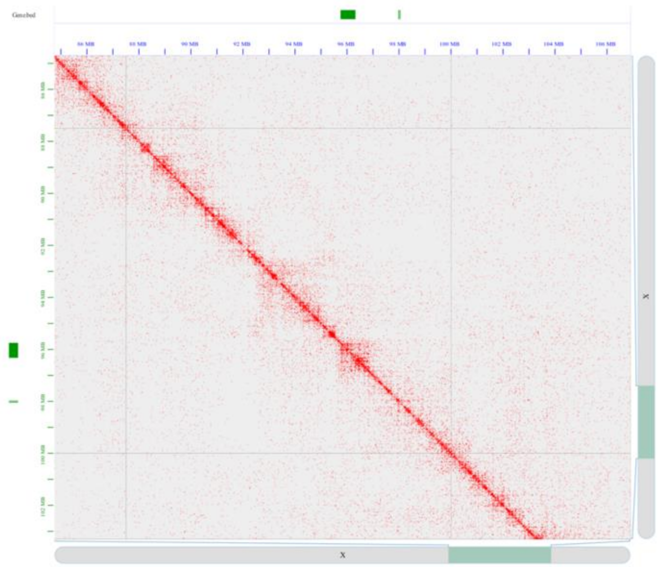

Figure S126: Hi-C figure generated using Juicebox of dog liver data (SRR1771337) where red dots show the genomic loci interaction between adjacent regions and gap shows there is no interaction between the nearby region. We loaded the bed file on the top and left sides of the Hi-C plot, where the green color indicates the gene of interest (*COA1* duplicate*)* and adjacent syntenic regions. chromosome or scaffold is mentioned on the bottom and right sides of the plot.

Figure S127

human Liver Hi-C (SRR1501147) *COA1* region

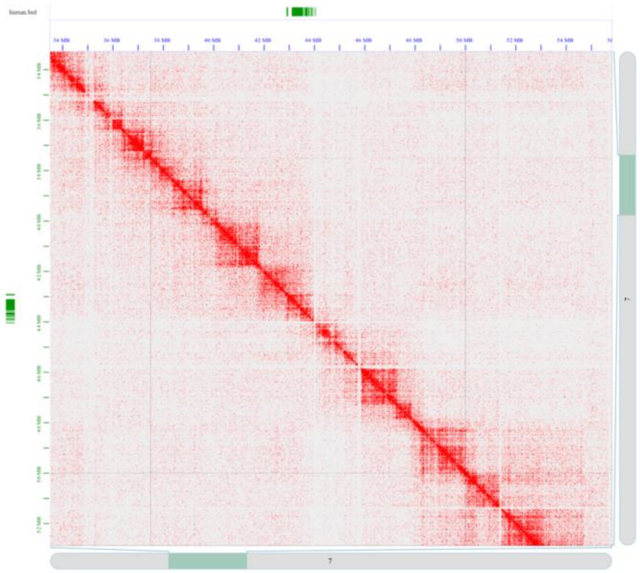

Figure S127: Hi-C figure generated using Juicebox of human liver data (SRR1501147) where red dots show the genomic loci interaction between adjacent regions and gap shows there is no interaction between the nearby region. We loaded the bed file on the top and left sides of the Hi-C plot, where the each line of green color indicates the gene of interest (*COA1*) and adjacent syntenic regions. chromosome or scaffold is mentioned on the bottom and right sides of the plot.

Figure S128 human Liver Hi-C (SRR1501147) *COA1* duplicate region

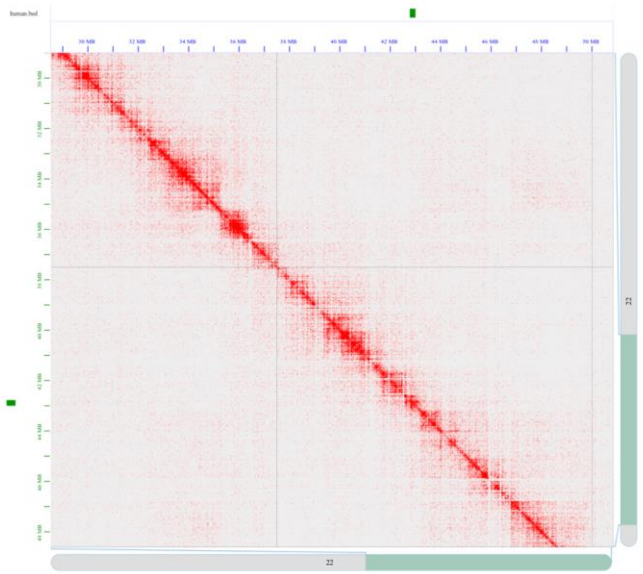

Figure S128: Hi-C figure generated using Juicebox of human liver data (SRR1501147) where red dots show the genomic loci interaction between adjacent regions and gap shows there is no interaction between the nearby region. We loaded the bed file on the top and left sides of the Hi-C plot, where the green color indicates the gene of interest (*COA1* duplicate) and adjacent syntenic regions. chromosome or scaffold is mentioned on the bottom and right sides of the plot.

Figure S129

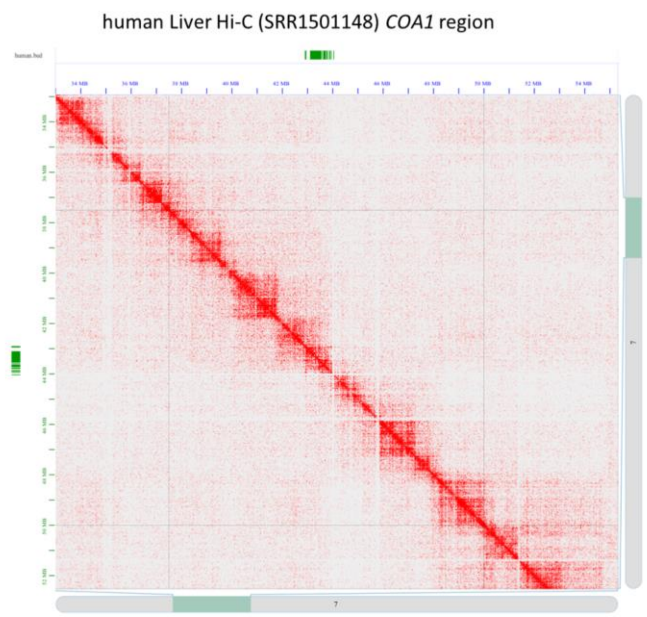

Figure S129: Hi-C figure generated using Juicebox of human liver data (SRR1501148) where red dots show the genomic loci interaction between adjacent regions and gap shows there is no interaction between the nearby region. We loaded the bed file on the top and left sides of the Hi-C plot, where the each line of green color indicates the gene of interest (*COA1*) and adjacent syntenic regions. chromosome or scaffold is mentioned on the bottom and right sides of the plot.

Figure S130

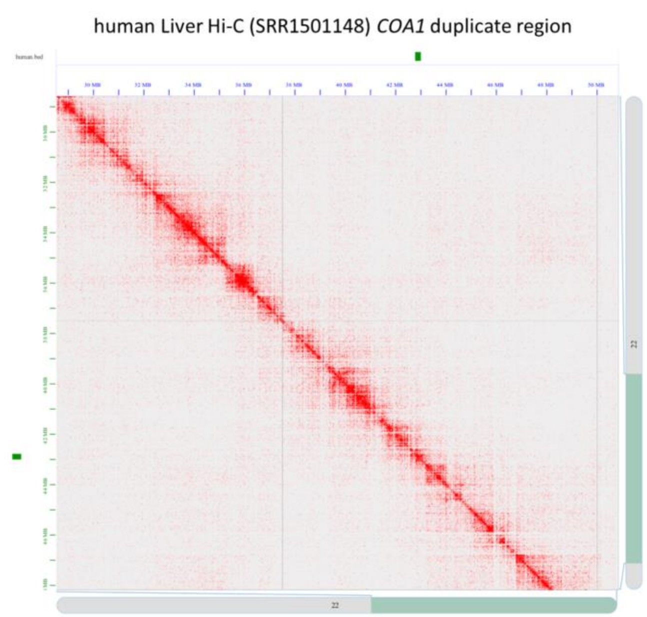

Figure S130: Hi-C figure generated using Juicebox of human liver data (SRR1501148) where red dots show the genomic loci interaction between adjacent regions and gap shows there is no interaction between the nearby region. We loaded the bed file on the top and left sides of the Hi-C plot, where the green color indicates the gene of interest (*COA1* duplicate) and adjacent syntenic regions. chromosome or scaffold is mentioned on the bottom and right sides of the plot.

Figure S131 human Liver Hi-C (SRR1501149) *COA1* region

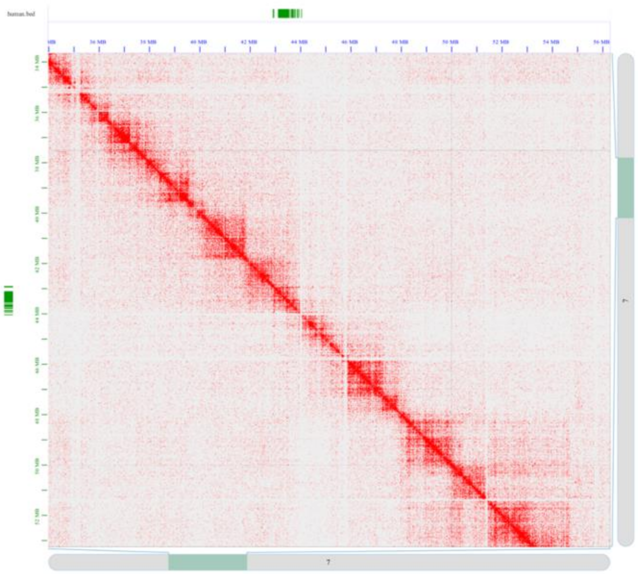

Figure S131: Hi-C figure generated using Juicebox of human liver data (SRR1501149) where red dots show the genomic loci interaction between adjacent regions and gap shows there is no interaction between the nearby region. We loaded the bed file on the top and left sides of the Hi-C plot, where the each line of green color indicates the gene of interest (*COA1*) and adjacent syntenic regions. chromosome or scaffold is mentioned on the bottom and right sides of the plot.

Figure S132

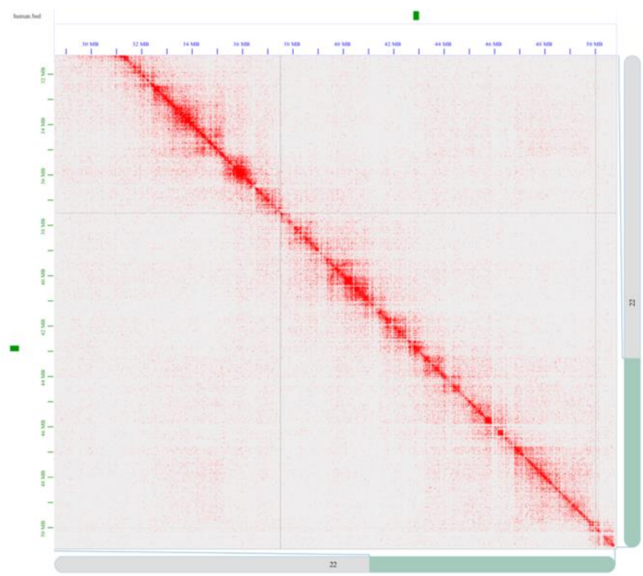

Figure S132: Hi-C figure generated using Juicebox of human liver data (SRR1501149) where red dots show the genomic loci interaction between adjacent regions and gap shows there is no interaction between the nearby region. We loaded the bed file on the top and left sides of the Hi-C plot, where the green color indicates the gene of interest (*COA1* duplicate) and adjacent syntenic regions. chromosome or scaffold is mentioned on the bottom and right sides of the plot.

Figure S133

human Liver Hi-C (SRR1501150) *COA1* region

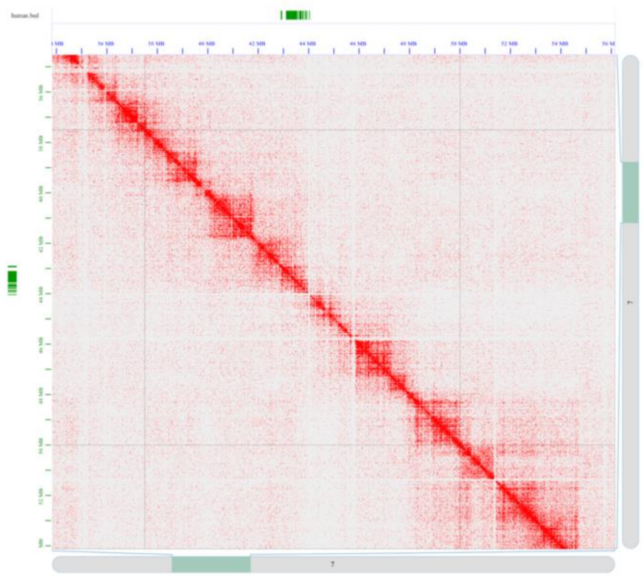

Figure S133: Hi-C figure generated using Juicebox of human liver data (SRR1501150) where red dots show the genomic loci interaction between adjacent regions and gap shows there is no interaction between the nearby region. We loaded the bed file on the top and left sides of the Hi-C plot, where the each line of green color indicates the gene of interest (*COA1*) and adjacent syntenic regions. chromosome or scaffold is mentioned on the bottom and right sides of the plot.

Figure S134 human Liver Hi-C (SRR1501150) *COA1* duplicate region

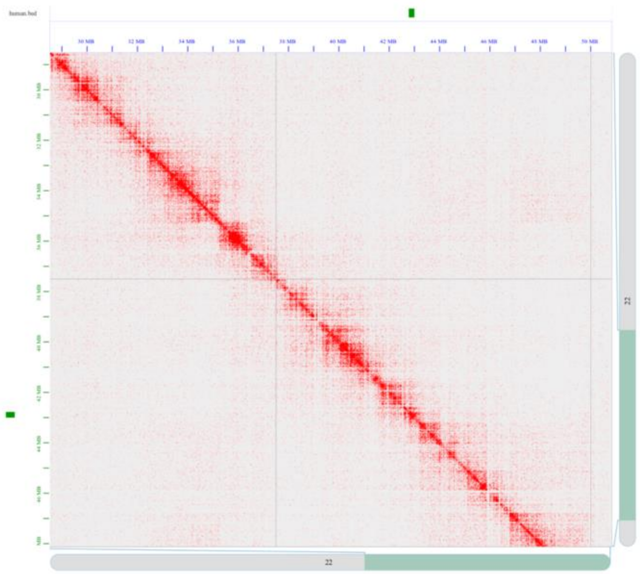

Figure S134: Hi-C figure generated using Juicebox of human liver data (SRR1501150) where red dots show the genomic loci interaction between adjacent regions and gap shows there is no interaction between the nearby region. We loaded the bed file on the top and left sides of the Hi-C plot, where the green color indicates the gene of interest (*COA1* duplicate) and adjacent syntenic regions. chromosome or scaffold is mentioned on the bottom and right sides of the plot.

Figure S135

human Liver Hi-C (SRR1501151) *COA1* region

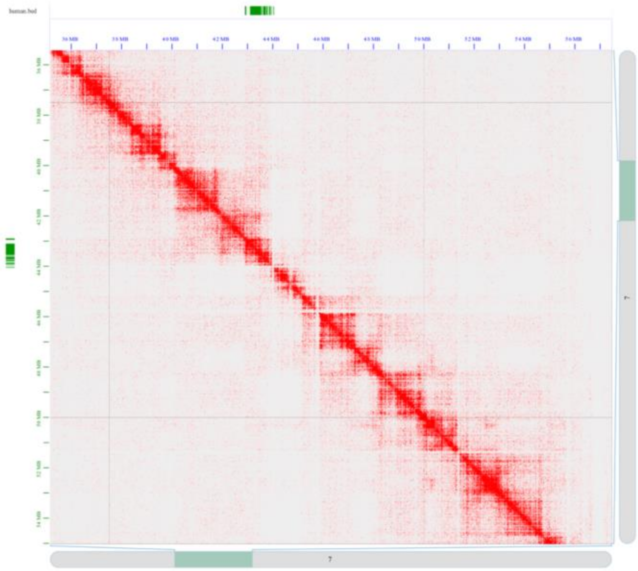

Figure S135: Hi-C figure generated using Juicebox of human liver data (SRR1501151) where red dots show the genomic loci interaction between adjacent regions and gap shows there is no interaction between the nearby region. We loaded the bed file on the top and left sides of the Hi-C plot, where the each line of green color indicates the gene of interest (*COA1*) and adjacent syntenic regions. chromosome or scaffold is mentioned on the bottom and right sides of the plot.

Figure S136 human Liver Hi-C (SRR1501151) *COA1* duplicate region

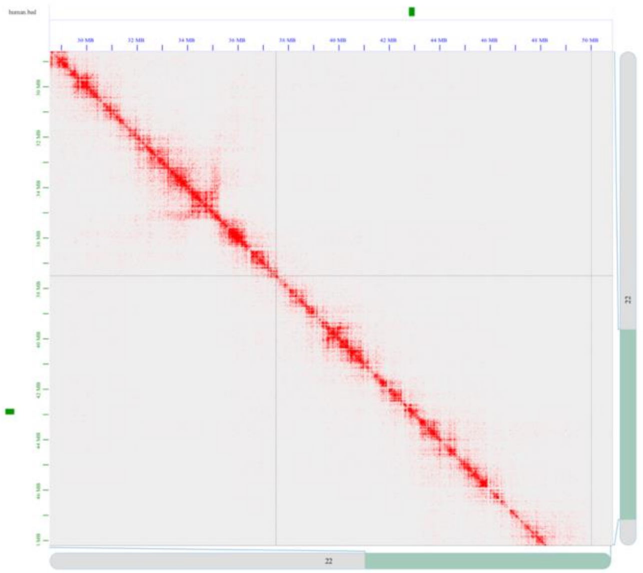

Figure S136: Hi-C figure generated using Juicebox of human liver data (SRR1501151) where red dots show the genomic loci interaction between adjacent regions and gap shows there is no interaction between the nearby region. We loaded the bed file on the top and left sides of the Hi-C plot, where the green color indicates the gene of interest (*COA1* duplicate) and adjacent syntenic regions. chromosome or scaffold is mentioned on the bottom and right sides of the plot.

Figure S137

Rhesus macaque Liver Hi-C (SRR1771328) *COA1* region

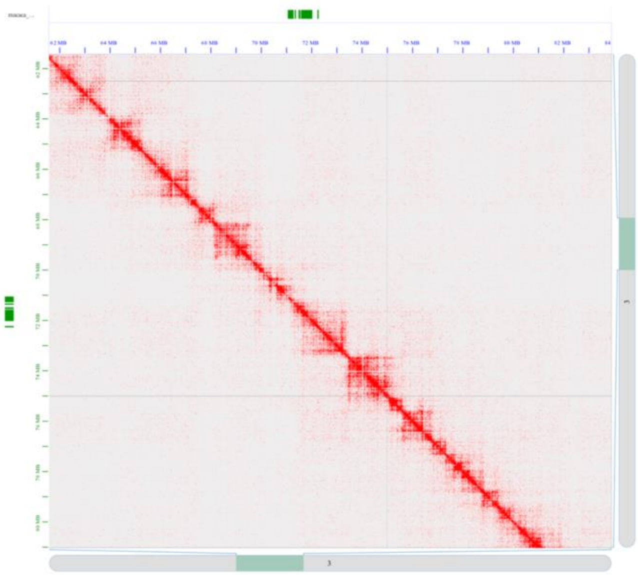

Figure S137: Hi-C figure generated using Juicebox of Rhesus macaque liver data (SRR1771328) where red dots show the genomic loci interaction between adjacent regions and gap shows there is no interaction between the nearby region. We loaded the bed file on the top and left sides of the Hi-C plot, where each line of the green color indicates the gene of interest (*COA1)* and adjacent syntenic regions. chromosome or scaffold is mentioned on the bottom and right sides of the plot.

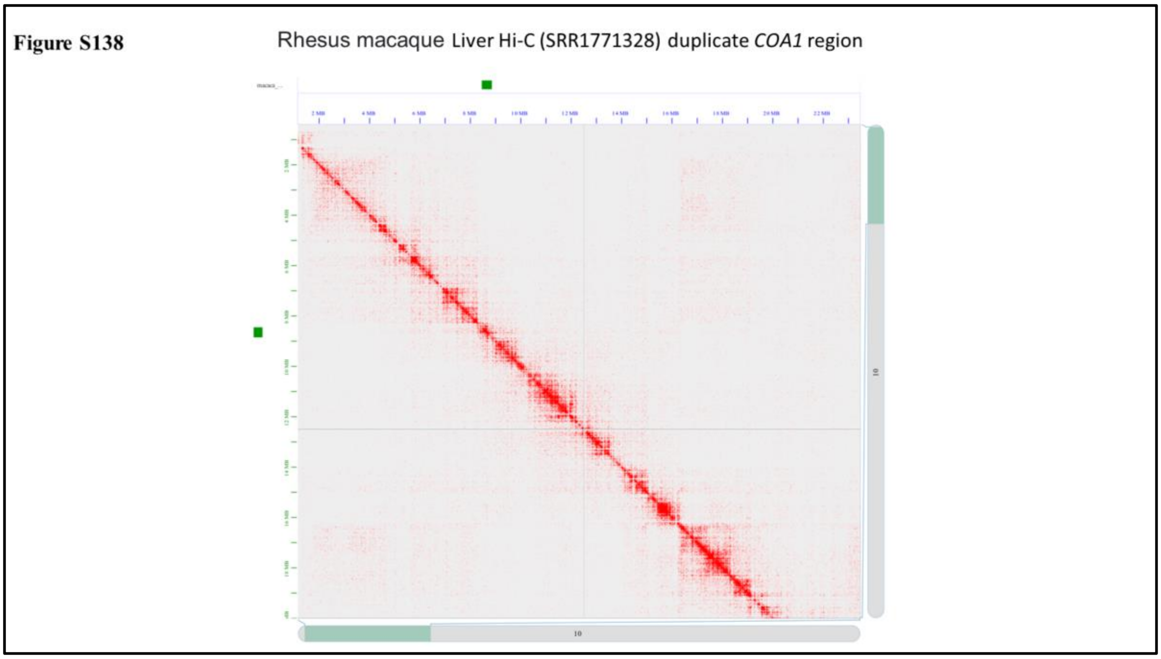

Figure S138: Hi-C figure generated using Juicebox of Rhesus macaque liver data (SRR1771328) where red dots show the genomic loci interaction between adjacent regions and gap shows there is no interaction between the nearby region. We loaded the bed file on the top and left sides of the Hi-C plot, where the green color indicates the gene of interest (*COA1* duplicate*)* and adjacent syntenic regions. chromosome or scaffold is mentioned on the bottom and right sides of the plot.

Figure S139

Rhesus macaque Liver Hi-C (SRR1771329) *COA1* region

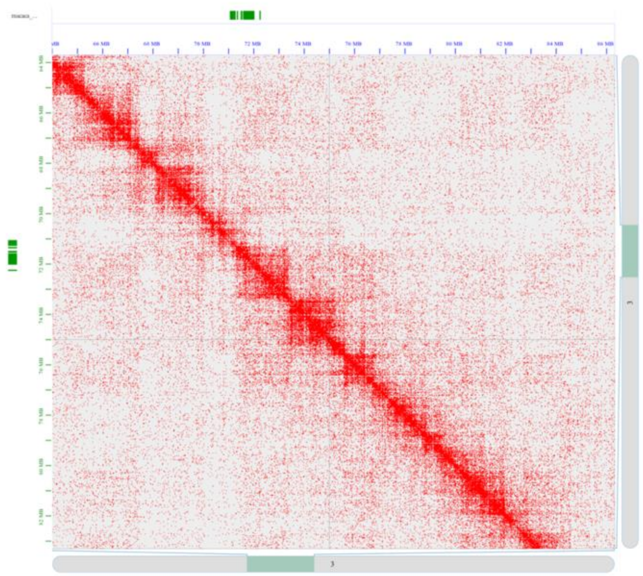

Figure S139: Hi-C figure generated using Juicebox of Rhesus macaque liver data (SRR1771329) where red dots show the genomic loci interaction between adjacent regions and gap shows there is no interaction between the nearby region. We loaded the bed file on the top and left sides of the Hi-C plot, where the each line of green color indicates the gene of interest (*COA1)* and adjacent syntenic regions. chromosome or scaffold is mentioned on the bottom and right sides of the plot.

Figure S140 Rhesus macaque Liver Hi-C (SRR1771329) duplicate *COA1* region

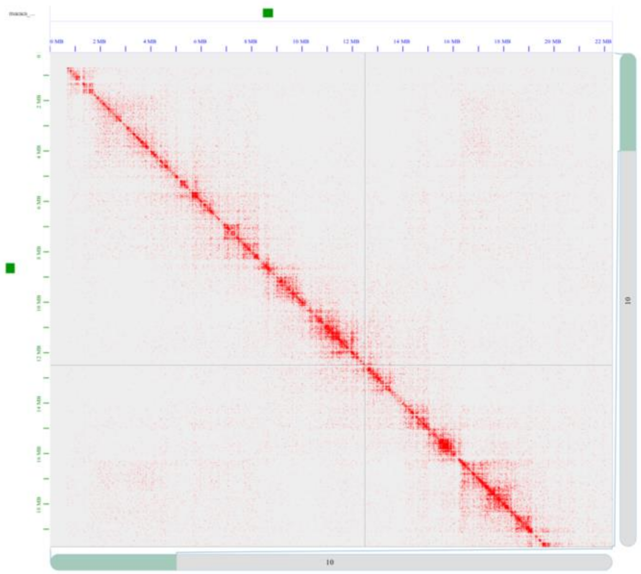

Figure S140: Hi-C figure generated using Juicebox of Rhesus macaque liver data (SRR1771329) where red dots show the genomic loci interaction between adjacent regions and gap shows there is no interaction between the nearby region. We loaded the bed file on the top and left sides of the Hi-C plot, where the green color indicates the gene of interest (*COA1* duplicate*)* and adjacent syntenic regions. chromosome or scaffold is mentioned on the bottom and right sides of the plot.

Figure S141

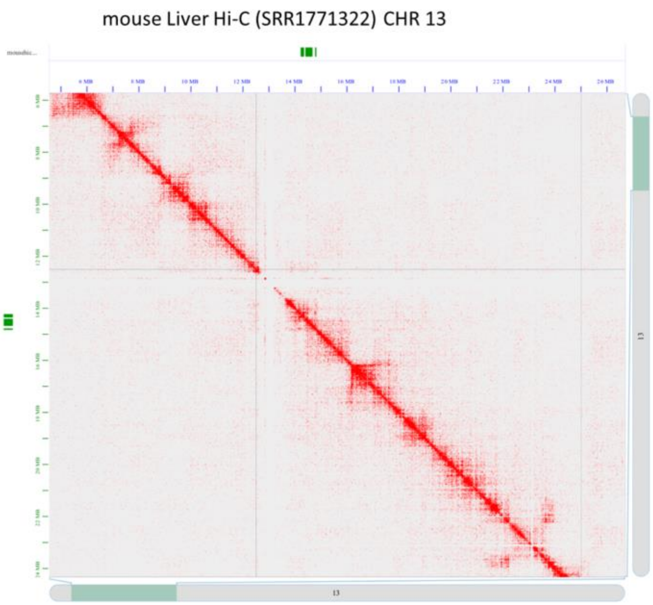

Figure S141: Hi-C figure generated using Juicebox of mouse liver data (SRR1771322) where red dots show the genomic loci interaction between adjacent regions and gap shows there is no interaction between the nearby region. We loaded the bed file on the top and left sides of the Hi-C plot, where each line of the green color indicates the gene of interest (*HECW1)* and adjacent syntenic regions. chromosome or scaffold is mentioned on the bottom and right sides of the plot.

Figure S142

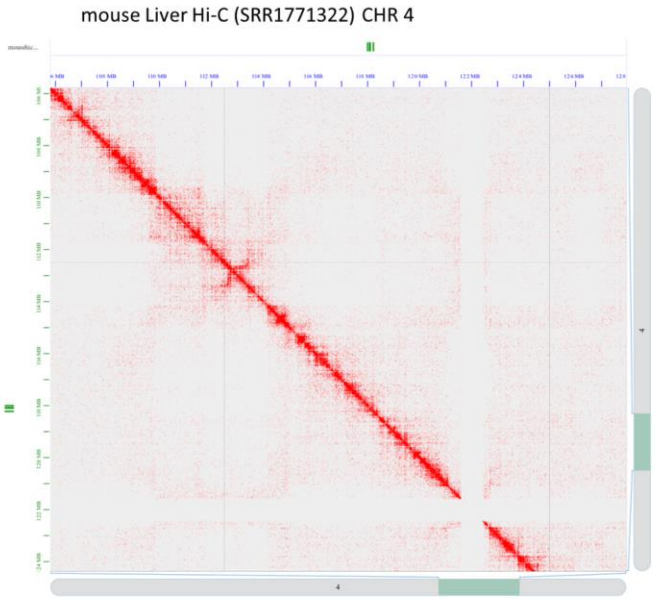

Figure S142: Hi-C figure generated using Juicebox of mouse liver data (SRR1771322) where red dots show the genomic loci interaction between adjacent regions and gap shows there is no interaction between the nearby region. We loaded the bed file on the top and left sides of the Hi-C plot, where each line of the green color indicates the gene of interest (*COA1* remnants*)* and adjacent syntenic regions. chromosome or scaffold is mentioned on the bottom and right sides of the plot.

Figure S143

mouse Liver Hi-C (SRR1771322) CHR 2

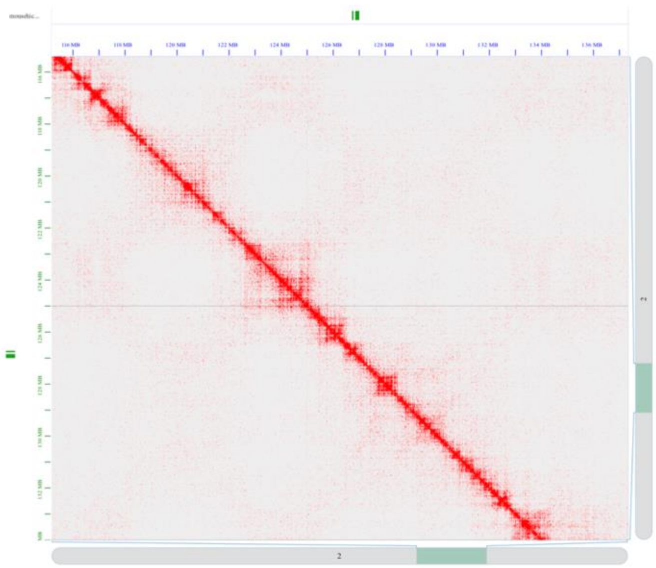

Figure S143: Hi-C figure generated using Juicebox of mouse liver data (SRR1771322) where red dots show the genomic loci interaction between adjacent regions and gap shows there is no interaction between the nearby region. We loaded the bed file on the top and left sides of the Hi-C plot, where each line of the green color indicates the gene of interest (*BLVRA)* and adjacent syntenic regions. chromosome or scaffold is mentioned on the bottom and right sides of the plot.

Figure S144

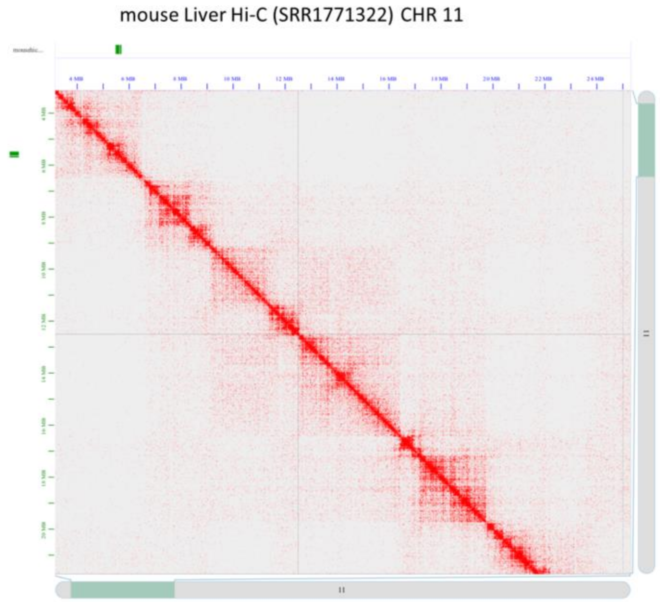

Figure S144: Hi-C figure generated using Juicebox of mouse liver data (SRR1771322) where red dots show the genomic loci interaction between adjacent regions and gap shows there is no interaction between the nearby region. We loaded the bed file on the top and left sides of the Hi-C plot, where each line of the green color indicates the gene of interest (*MRPS24)* and adjacent syntenic regions. chromosome or scaffold is mentioned on the bottom and right sides of the plot.

Figure S145

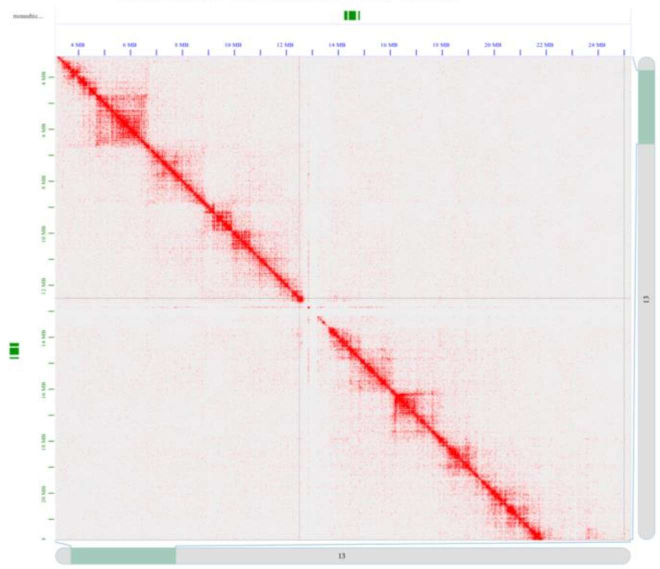

Figure S145: Hi-C figure generated using Juicebox of mouse liver data (SRR1771323) where red dots show the genomic loci interaction between adjacent regions and gap shows there is no interaction between the nearby region. We loaded the bed file on the top and left sides of the Hi-C plot, where each line of the green color indicates the gene of interest (*HECW1)* and adjacent syntenic regions. chromosome or scaffold is mentioned on the bottom and right sides of the plot.

Figure S146

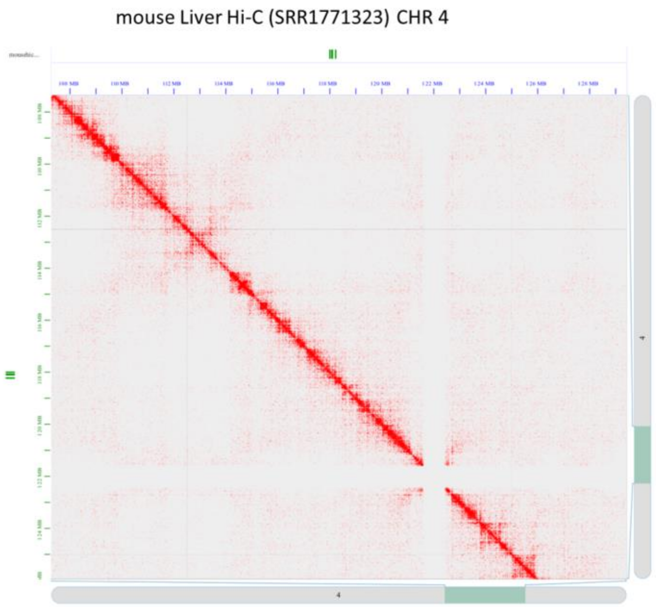

Figure S146: Hi-C figure generated using Juicebox of mouse liver data (SRR1771323) where red dots show the genomic loci interaction between adjacent regions and gap shows there is no interaction between the nearby region. We loaded the bed file on the top and left sides of the Hi-C plot, where each line of the green color indicates the gene of interest (*COA1* remnants*)* and adjacent syntenic regions. chromosome or scaffold is mentioned on the bottom and right sides of the plot.

Figure S147

mouse Liver Hi-C (SRR1771323) CHR 2

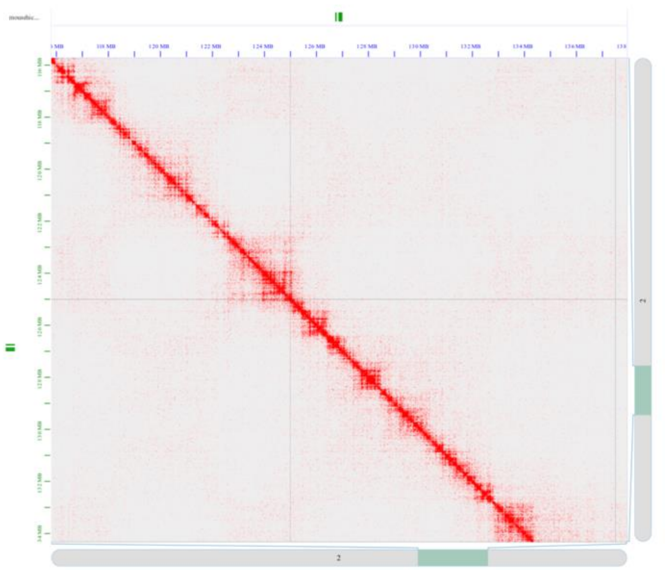

Figure S147: Hi-C figure generated using Juicebox of mouse liver data (SRR1771323) where red dots show the genomic loci interaction between adjacent regions and gap shows there is no interaction between the nearby region. We loaded the bed file on the top and left sides of the Hi-C plot, where each line of the green color indicates the gene of interest (*BLVRA)* and adjacent syntenic regions. chromosome or scaffold is mentioned on the bottom and right sides of the plot.

Figure S148

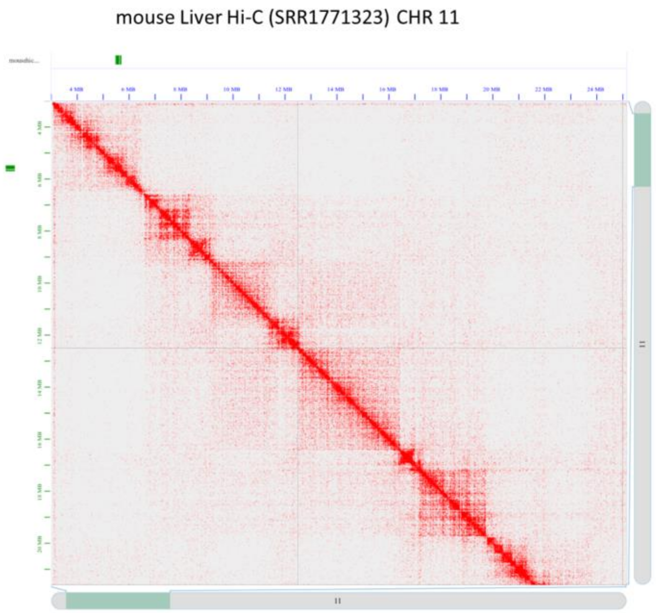

Figure S148: Hi-C figure generated using Juicebox of mouse liver data (SRR1771323) where red dots show the genomic loci interaction between adjacent regions and gap shows there is no interaction between the nearby region. We loaded the bed file on the top and left sides of the Hi-C plot, where each line of the green color indicates the gene of interest (*MRPS24)* and adjacent syntenic regions. chromosome or scaffold is mentioned on the bottom and right sides of the plot.

Figure S149

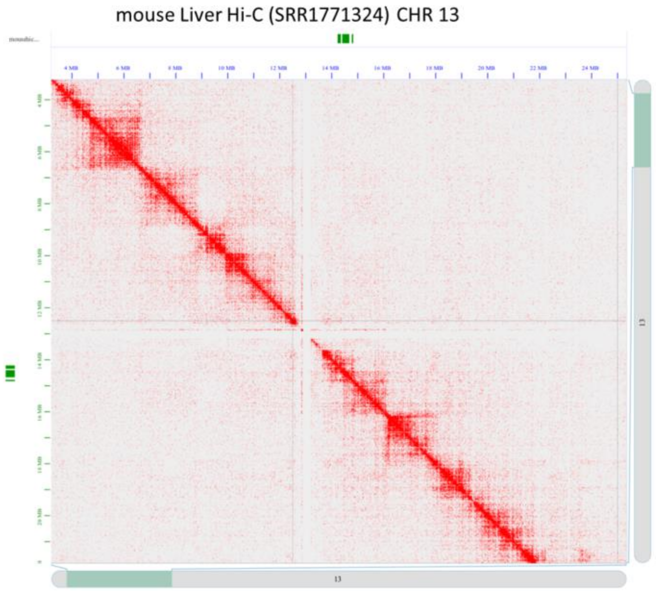

Figure S149: Hi-C figure generated using Juicebox of mouse liver data (SRR1771324) where red dots show the genomic loci interaction between adjacent regions and gap shows there is no interaction between the nearby region. We loaded the bed file on the top and left sides of the Hi-C plot, where each line of the green color indicates the gene of interest (*HECW1)* and adjacent syntenic regions. chromosome or scaffold is mentioned on the bottom and right sides of the plot.

Figure S150

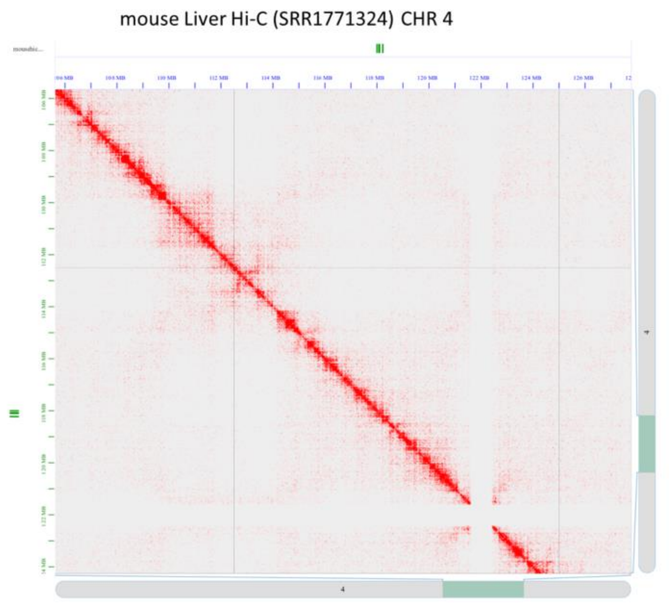

Figure S150: Hi-C figure generated using Juicebox of mouse liver data (SRR1771324) where red dots show the genomic loci interaction between adjacent regions and gap shows there is no interaction between the nearby region. We loaded the bed file on the top and left sides of the Hi-C plot, where each line of the green color indicates the gene of interest (*COA1* remnants*)* and adjacent syntenic regions. chromosome or scaffold is mentioned on the bottom and right sides of the plot.

Figure S151

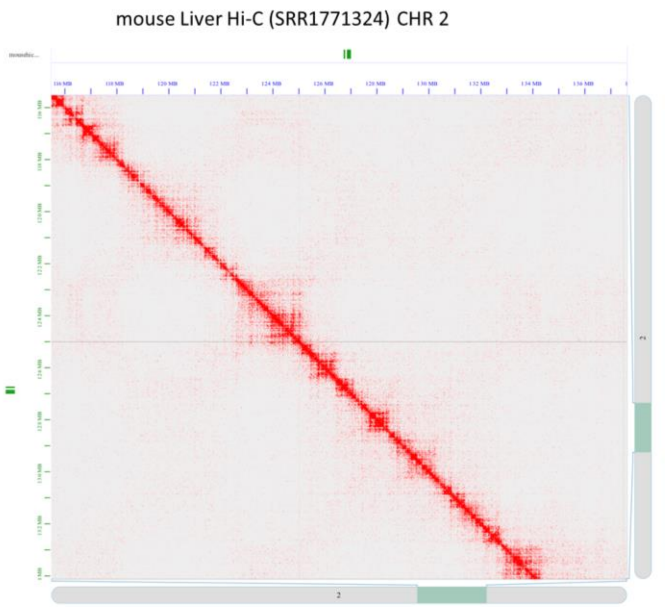

Figure S151: Hi-C figure generated using Juicebox of mouse liver data (SRR1771324) where red dots show the genomic loci interaction between adjacent regions and gap shows there is no interaction between the nearby region. We loaded the bed file on the top and left sides of the Hi-C plot, where each line of the green color indicates the gene of interest (*BLVRA)* and adjacent syntenic regions. chromosome or scaffold is mentioned on the bottom and right sides of the plot.

Figure S152

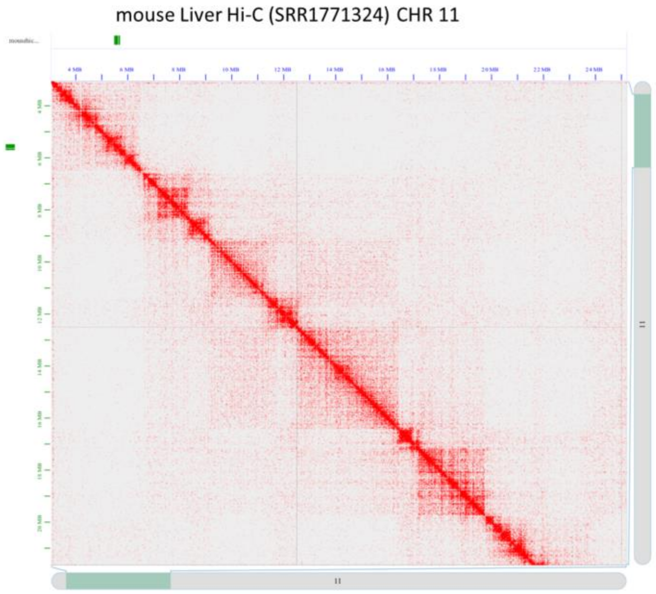

Figure S152: Hi-C figure generated using Juicebox of mouse liver data (SRR1771324) where red dots show the genomic loci interaction between adjacent regions and gap shows there is no interaction between the nearby region. We loaded the bed file on the top and left sides of the Hi-C plot, where each line of the green color indicates the gene of interest (*MRPS24)* and adjacent syntenic regions. chromosome or scaffold is mentioned on the bottom and right sides of the plot.

Figure S153

mouse Liver Hi-C (SRR1771325) CHR 13

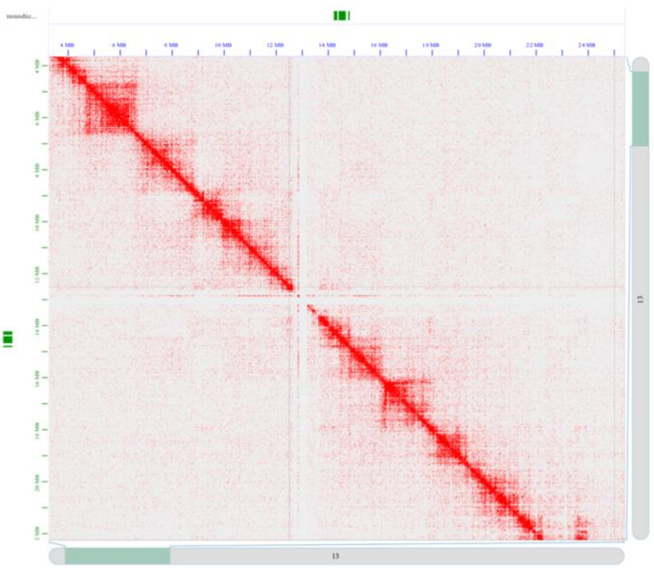

Figure S153: Hi-C figure generated using Juicebox of mouse liver data (SRR1771325) where red dots show the genomic loci interaction between adjacent regions and gap shows there is no interaction between the nearby region. We loaded the bed file on the top and left sides of the Hi-C plot, where each line of the green color indicates the gene of interest (*HECW1)* and adjacent syntenic regions. chromosome or scaffold is mentioned on the bottom and right sides of the plot.

Figure S154

mouse Liver Hi-C (SRR1771325) CHR 4

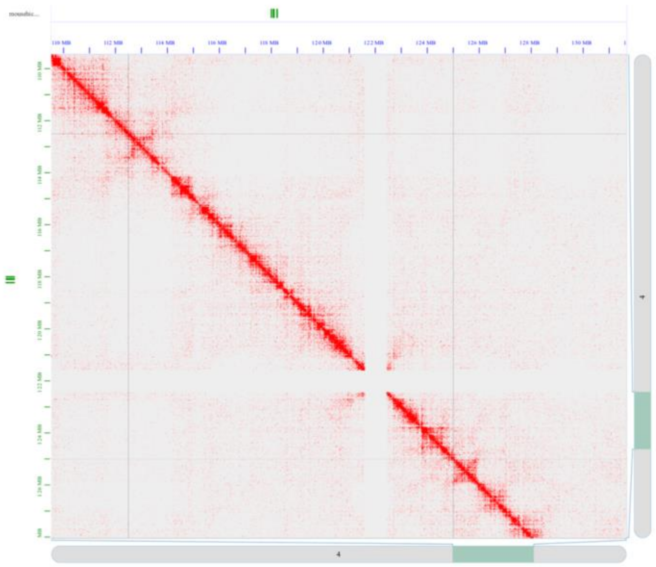

Figure S154: Hi-C figure generated using Juicebox of mouse liver data (SRR1771325) where red dots show the genomic loci interaction between adjacent regions and gap shows there is no interaction between the nearby region. We loaded the bed file on the top and left sides of the Hi-C plot, where each line of the green color indicates the gene of interest (*COA1* remnants*)* and adjacent syntenic regions. chromosome or scaffold is mentioned on the bottom and right sides of the plot.

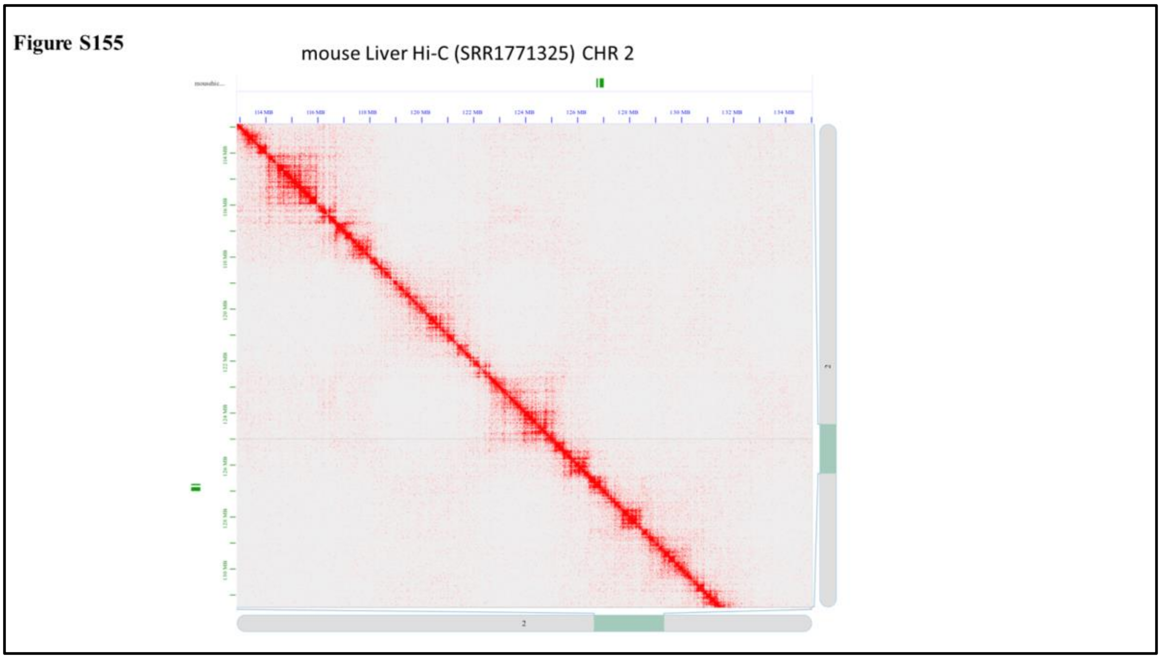

Figure S155: Hi-C figure generated using Juicebox of mouse liver data (SRR1771325) where red dots show the genomic loci interaction between adjacent regions and gap shows there is no interaction between the nearby region. We loaded the bed file on the top and left sides of the Hi-C plot, where each line of the green color indicates the gene of interest (*BLVRA)* and adjacent syntenic regions. chromosome or scaffold is mentioned on the bottom and right sides of the plot.

Figure S156

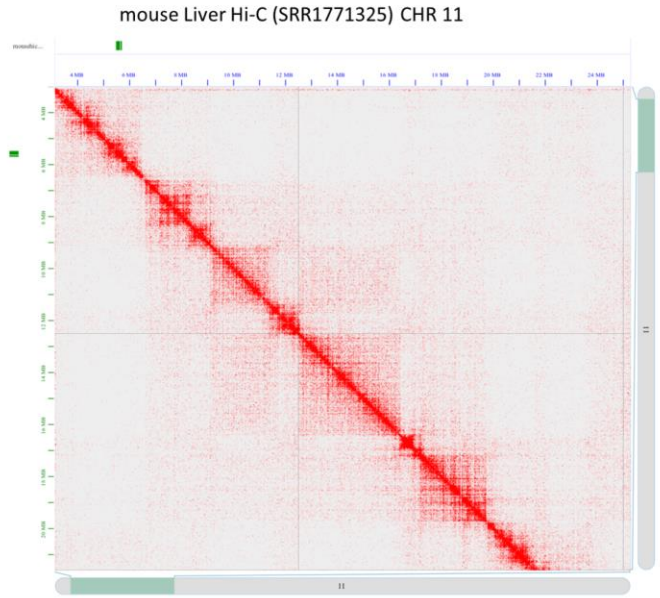

Figure S156: Hi-C figure generated using Juicebox of mouse liver data (SRR1771325) where red dots show the genomic loci interaction between adjacent regions and gap shows there is no interaction between the nearby region. We loaded the bed file on the top and left sides of the Hi-C plot, where each line of the green color indicates the gene of interest (*MRPS24)* and adjacent syntenic regions. chromosome or scaffold is mentioned on the bottom and right sides of the plot.

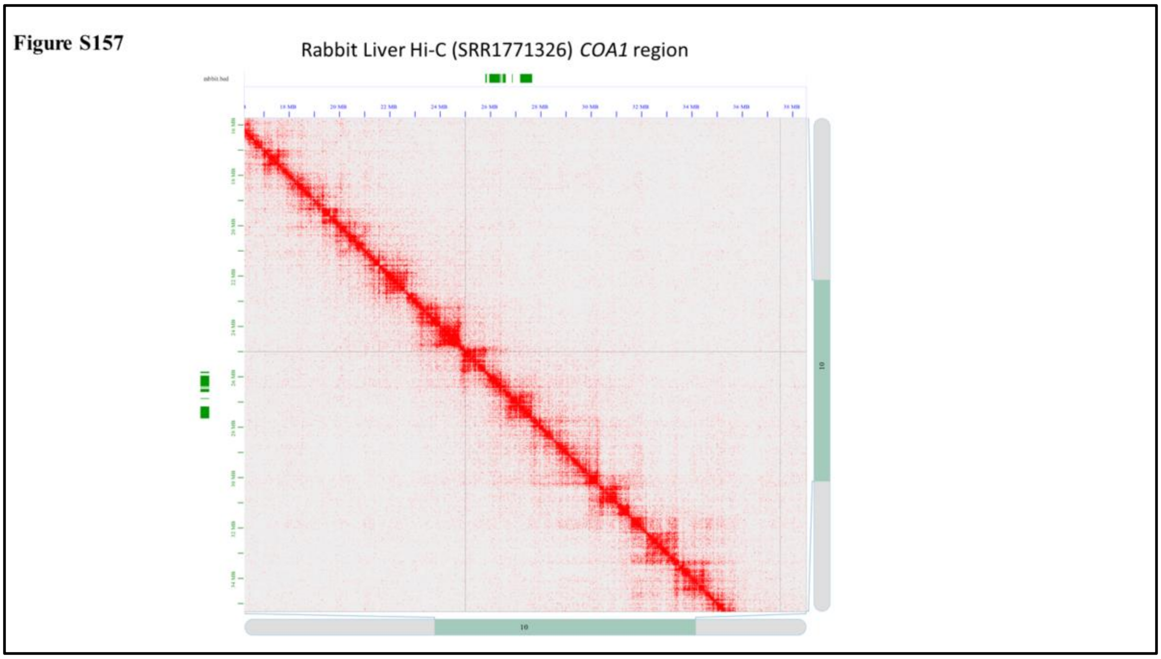

Figure S157: Hi-C figure generated using Juicebox of Rabbit liver data (SRR1771326) where red dots show the genomic loci interaction between adjacent regions and gap shows there is no interaction between the nearby region. We loaded the bed file on the top and left sides of the Hi-C plot, where each line of the green color indicates the gene of interest (*COA1)* and adjacent syntenic regions. chromosome or scaffold is mentioned on the bottom and right sides of the plot.

Figure S158

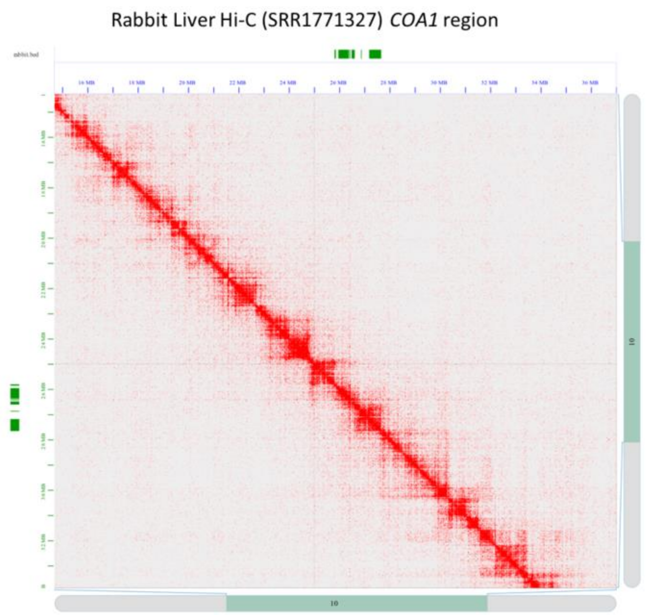

Figure S158: Hi-C figure generated using Juicebox of Rabbit liver data (SRR1771327) where red dots show the genomic loci interaction between adjacent regions and gap shows there is no interaction between the nearby region. We loaded the bed file on the top and left sides of the Hi-C plot, where each line of the green color indicates the gene of interest (*COA1)* and adjacent syntenic regions. chromosome or scaffold is mentioned on the bottom and right sides of the plot.

Figure S159

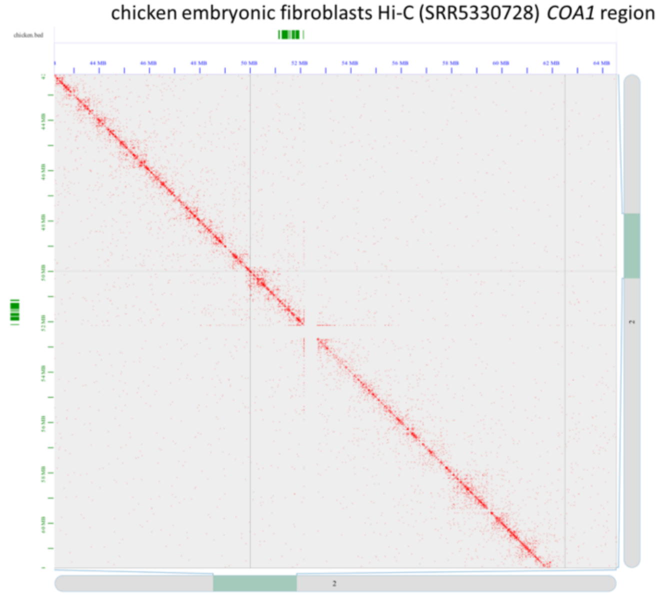

Figure S159: Hi-C figure generated using Juicebox of chicken embryonic fibroblasts (SRR5330728) where red dots show the genomic loci interaction between adjacent regions and gap shows there is no interaction between the nearby region. We loaded the bed file on the top and left sides of the Hi-C plot, where each line of the green color indicates the gene of interest (*COA1)* and adjacent syntenic regions. chromosome or scaffold is mentioned on the bottom and right sides of the plot.

Figure S160

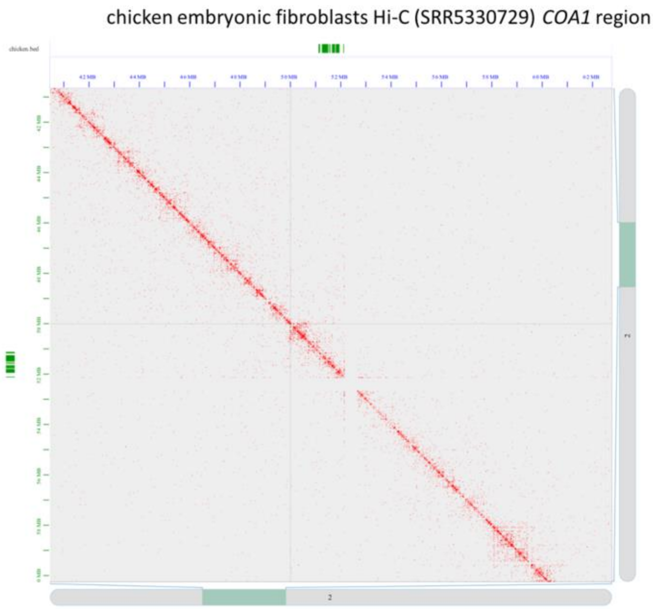

Figure S160: Hi-C figure generated using Juicebox of chicken embryonic fibroblasts (SRR5330729) where red dots show the genomic loci interaction between adjacent regions and gap shows there is no interaction between the nearby region. We loaded the bed file on the top and left sides of the Hi-C plot, where each line of the green color indicates the gene of interest (*COA1)* and adjacent syntenic regions. chromosome or scaffold is mentioned on the bottom and right sides of the plot.

Figure S161

chicken embryonic fibroblasts Hi-C (SRR5330730) *COA1* region

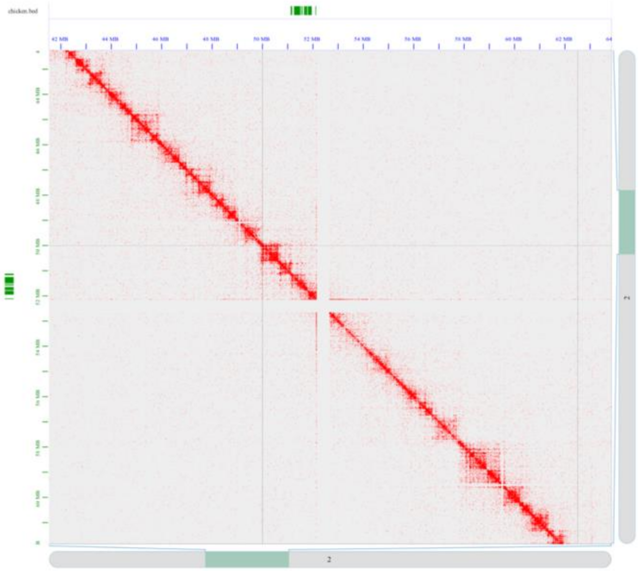

Figure S161: Hi-C figure generated using Juicebox of chicken embryonic fibroblasts (SRR5330730) where red dots show the genomic loci interaction between adjacent regions and gap shows there is no interaction between the nearby region. We loaded the bed file on the top and left sides of the Hi-C plot, where each line of the green color indicates the gene of interest (*COA1)* and adjacent syntenic regions. chromosome or scaffold is mentioned on the bottom and right sides of the plot.

Figure S162

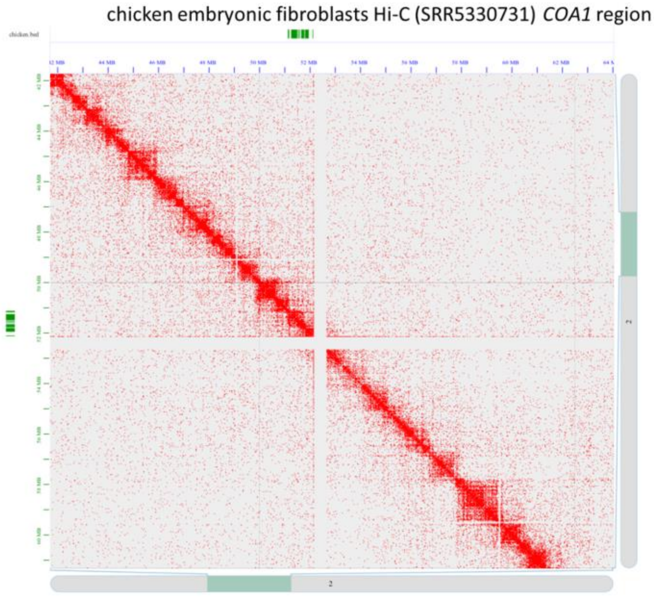

Figure S162: Hi-C figure generated using Juicebox of chicken embryonic fibroblasts (SRR5330731) where red dots show the genomic loci interaction between adjacent regions and gap shows there is no interaction between the nearby region. We loaded the bed file on the top and left sides of the Hi-C plot, where each line of the green color indicates the gene of interest (*COA1)* and adjacent syntenic regions. chromosome or scaffold is mentioned on the bottom and right sides of the plot.

Figure S163

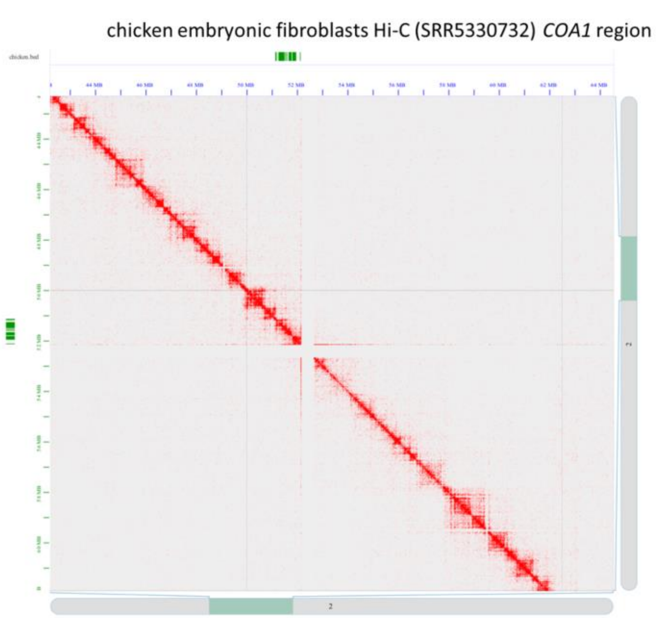

Figure S163: Hi-C figure generated using Juicebox of chicken embryonic fibroblasts (SRR5330732) where red dots show the genomic loci interaction between adjacent regions and gap shows there is no interaction between the nearby region. We loaded the bed file on the top and left sides of the Hi-C plot, where each line of the green color indicates the gene of interest (*COA1)* and adjacent syntenic regions. chromosome or scaffold is mentioned on the bottom and right sides of the plot.

Figure S164

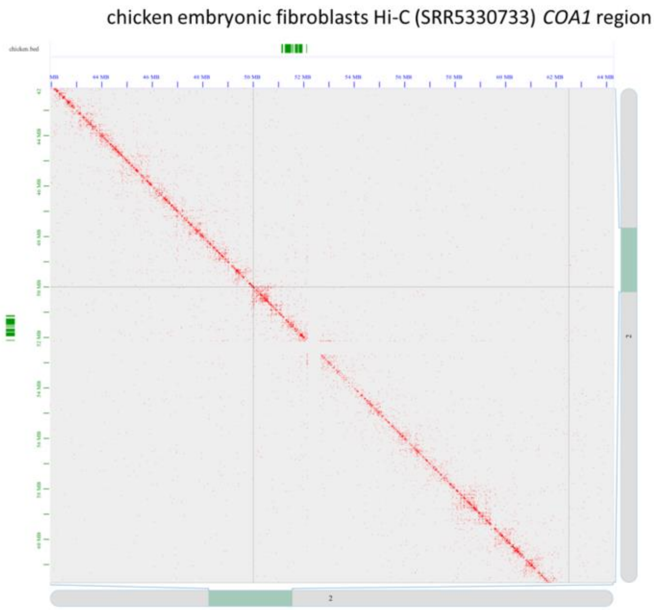

Figure S164: Hi-C figure generated using Juicebox of chicken embryonic fibroblasts (SRR5330733) where red dots show the genomic loci interaction between adjacent regions and gap shows there is no interaction between the nearby region. We loaded the bed file on the top and left sides of the Hi-C plot, where each line of the green color indicates the gene of interest (*COA1)* and adjacent syntenic regions. chromosome or scaffold is mentioned on the bottom and right sides of the plot.

Figure S165

chicken embryonic fibroblasts Hi-C (SRR5330734) *COA1* region

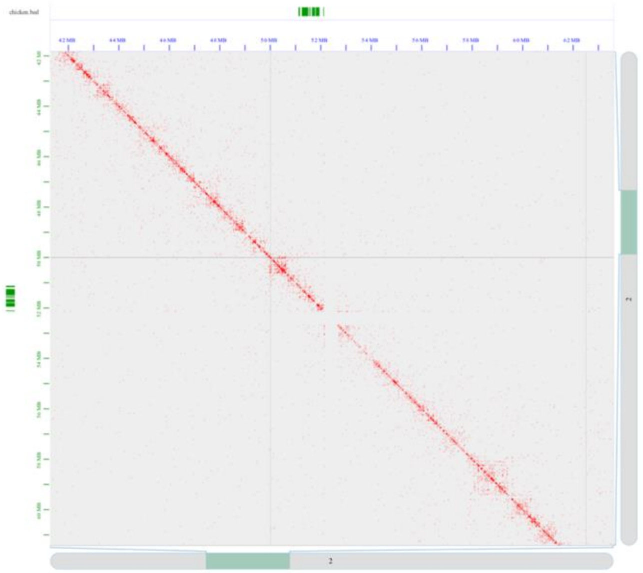

Figure S165: Hi-C figure generated using Juicebox of chicken embryonic fibroblasts (SRR5330734) where red dots show the genomic loci interaction between adjacent regions and gap shows there is no interaction between the nearby region. We loaded the bed file on the top and left sides of the Hi-C plot, where each line of the green color indicates the gene of interest (*COA1)* and adjacent syntenic regions. chromosome or scaffold is mentioned on the bottom and right sides of the plot.

Figure S166

chicken embryonic fibroblasts Hi-C (SRR5330735) *COA1* region

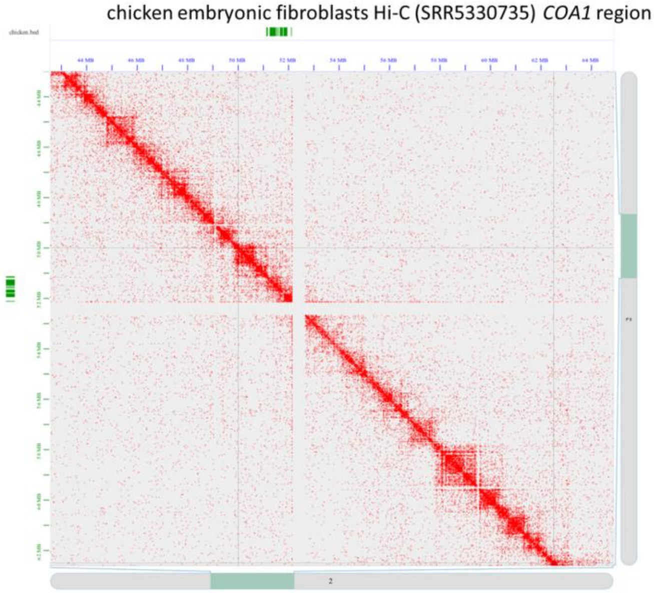

Figure S166: Hi-C figure generated using Juicebox of chicken embryonic fibroblasts (SRR5330735) where red dots show the genomic loci interaction between adjacent regions and gap shows there is no interaction between the nearby region. We loaded the bed file on the top and left sides of the Hi-C plot, where each line of the green color indicates the gene of interest (*COA1)* and adjacent syntenic regions. chromosome or scaffold is mentioned on the bottom and right sides of the plot.

Figure S167

chicken embryonic fibroblasts Hi-C (SRR5330736) *COA1* region

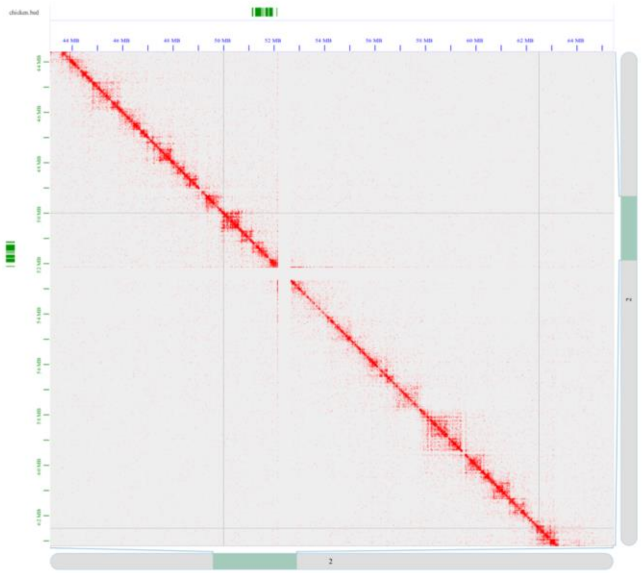

Figure S167: Hi-C figure generated using Juicebox of chicken embryonic fibroblasts (SRR5330736) where red dots show the genomic loci interaction between adjacent regions and gap shows there is no interaction between the nearby region. We loaded the bed file on the top and left sides of the Hi-C plot, where each line of the green color indicates the gene of interest (*COA1)* and adjacent syntenic regions. chromosome or scaffold is mentioned on the bottom and right sides of the plot.

Figure S168

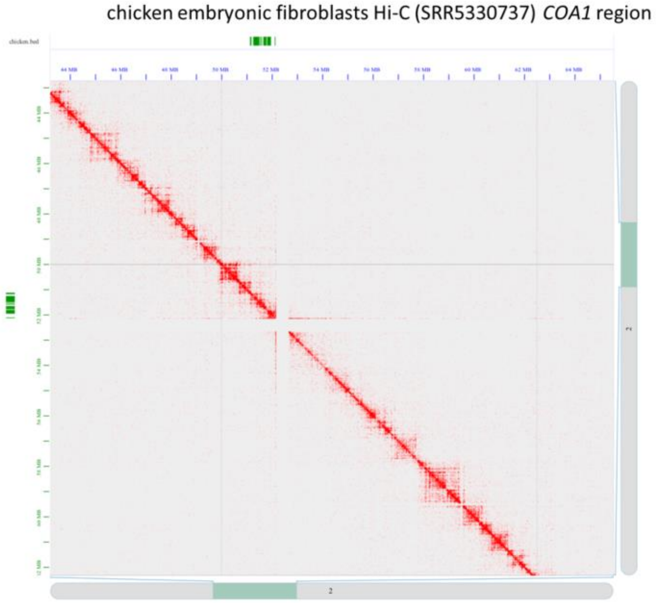

Figure S168: Hi-C figure generated using Juicebox of chicken embryonic fibroblasts (SRR5330737) where red dots show the genomic loci interaction between adjacent regions and gap shows there is no interaction between the nearby region. We loaded the bed file on the top and left sides of the Hi-C plot, where each line of the green color indicates the gene of interest (*COA1)* and adjacent syntenic regions. chromosome or scaffold is mentioned on the bottom and right sides of the plot.

Figure S169

chicken Mature erythrocytes Hi-C (SRR5330739) *COA1* region

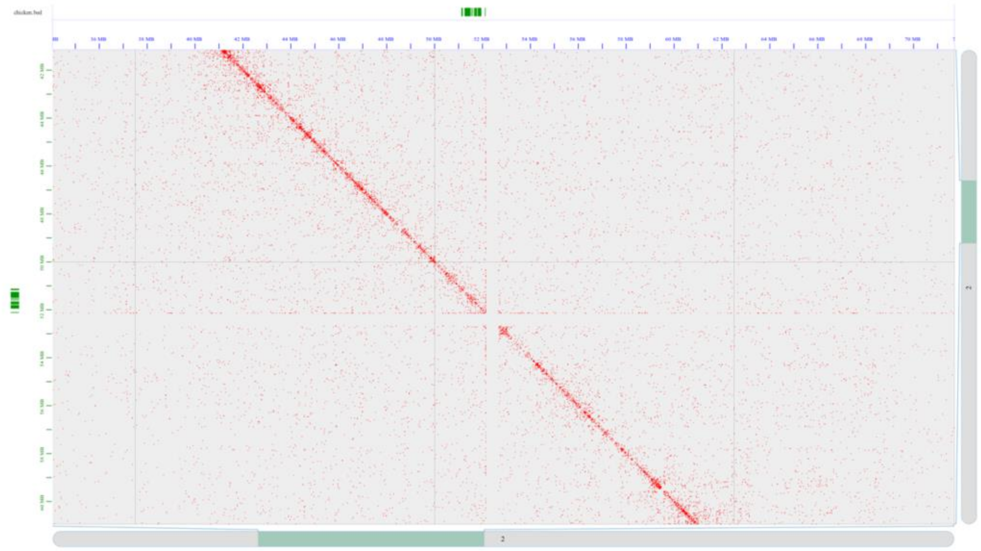

Figure S169: Hi-C figure generated using Juicebox of chicken Mature erythrocytes (SRR5330739) where red dots show the genomic loci interaction between adjacent regions and gap shows there is no interaction between the nearby region. We loaded the bed file on the top and left sides of the Hi-C plot, where each line of the green color indicates the gene of interest (*COA1)* and adjacent syntenic regions. chromosome or scaffold is mentioned on the bottom and right sides of the plot.

Figure S170

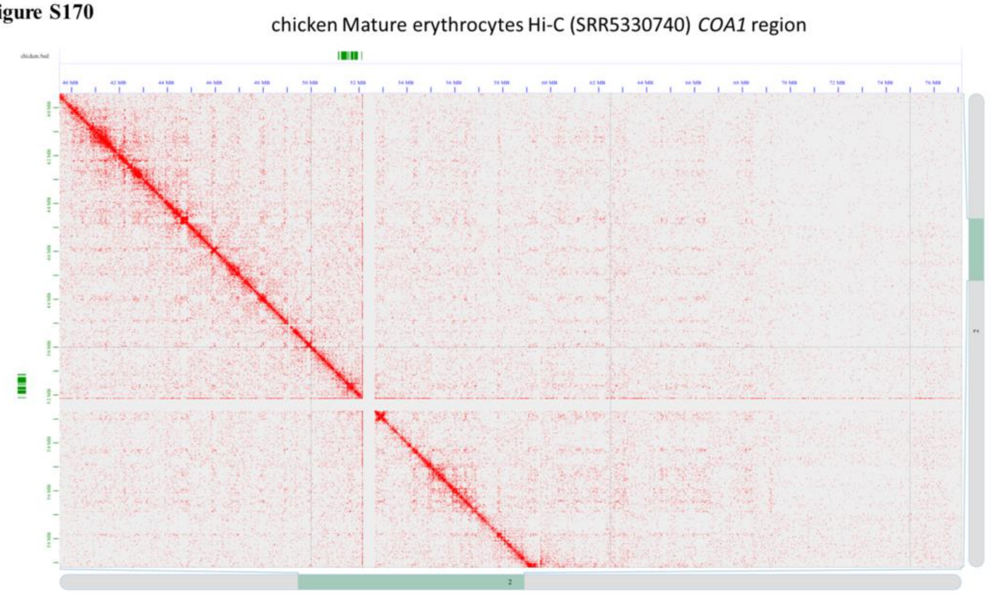

Figure S170: Hi-C figure generated using Juicebox of chicken Mature erythrocytes (SRR5330740) where red dots show the genomic loci interaction between adjacent regions and gap shows there is no interaction between the nearby region. We loaded the bed file on the top and left sides of the Hi-C plot, where each line of the green color indicates the gene of interest (*COA1)* and adjacent syntenic regions. chromosome or scaffold is mentioned on the bottom and right sides of the plot.

Figure S171

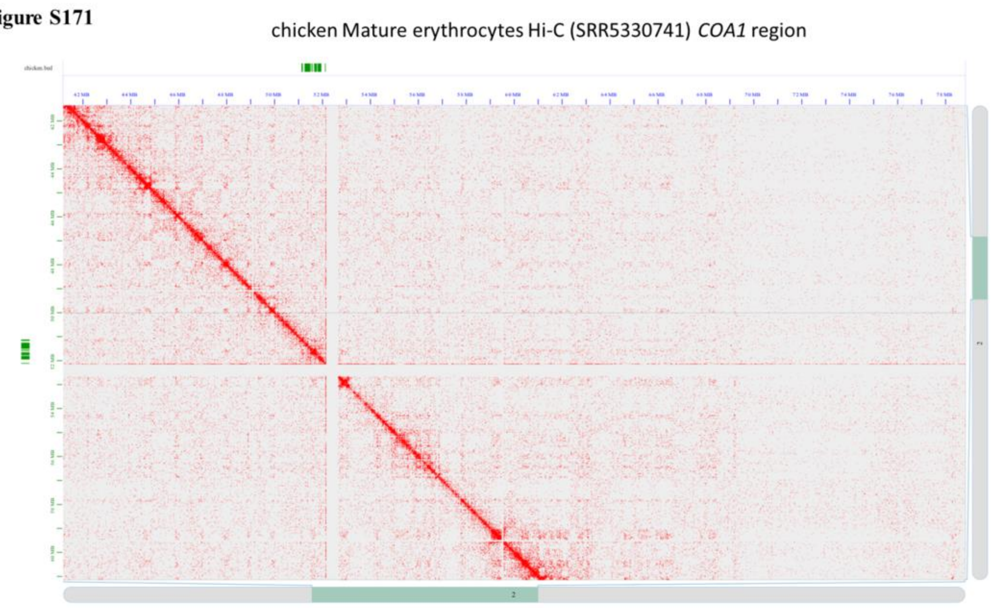

Figure S171: Hi-C figure generated using Juicebox of chicken Mature erythrocytes (SRR5330741) where red dots show the genomic loci interaction between adjacent regions and gap shows there is no interaction between the nearby region. We loaded the bed file on the top and left sides of the Hi-C plot, where each line of the green color indicates the gene of interest (*COA1)* and adjacent syntenic regions. chromosome or scaffold is mentioned on the bottom and right sides of the plot.

Figure S172

chicken Mature erythrocytes Hi-C (SRR5330742) *COA1* region

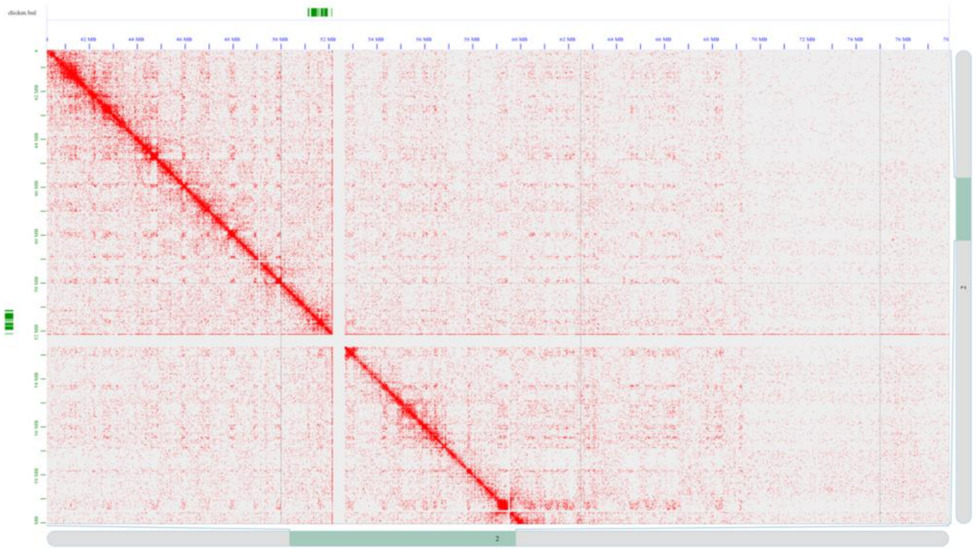

Figure S172: Hi-C figure generated using Juicebox of chicken Mature erythrocytes (SRR5330742) where red dots show the genomic loci interaction between adjacent regions and gap shows there is no interaction between the nearby region. We loaded the bed file on the top and left sides of the Hi-C plot, where each line of the green color indicates the gene of interest (*COA1)* and adjacent syntenic regions. chromosome or scaffold is mentioned on the bottom and right sides of the plot.

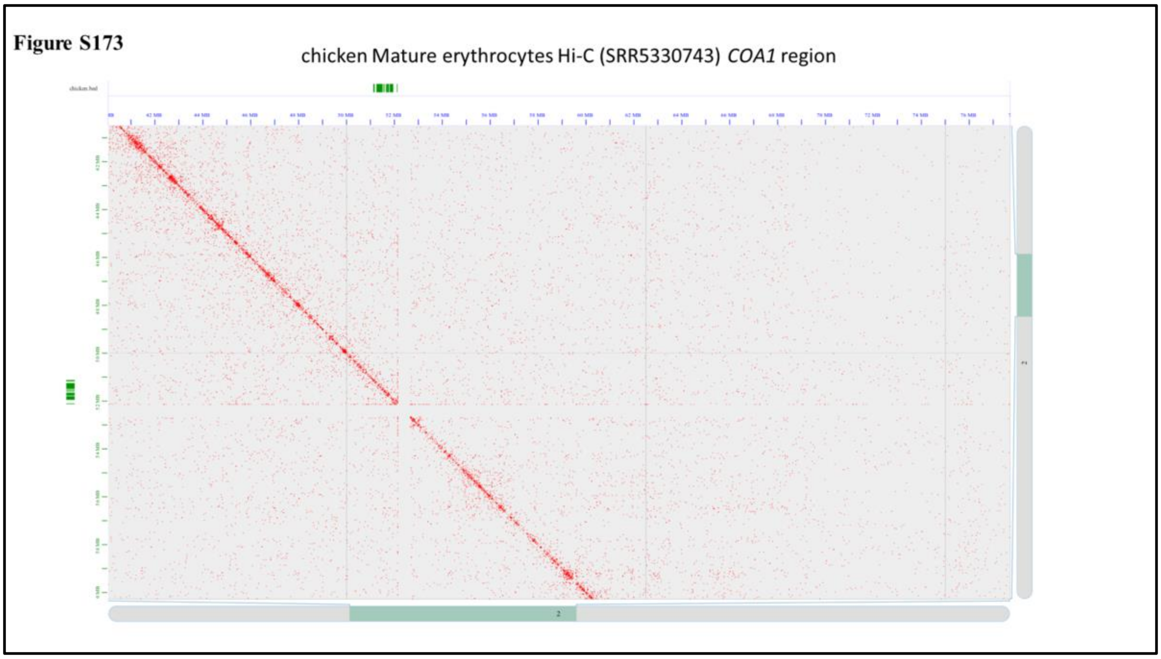

Figure S173: Hi-C figure generated using Juicebox of chicken Mature erythrocytes (SRR5330743) where red dots show the genomic loci interaction between adjacent regions and gap shows there is no interaction between the nearby region. We loaded the bed file on the top and left sides of the Hi-C plot, where each line of the green color indicates the gene of interest (*COA1)* and adjacent syntenic regions. chromosome or scaffold is mentioned on the bottom and right sides of the plot.

Figure S174

chicken Mature erythrocytes Hi-C (SRR5330744) *COA1* region

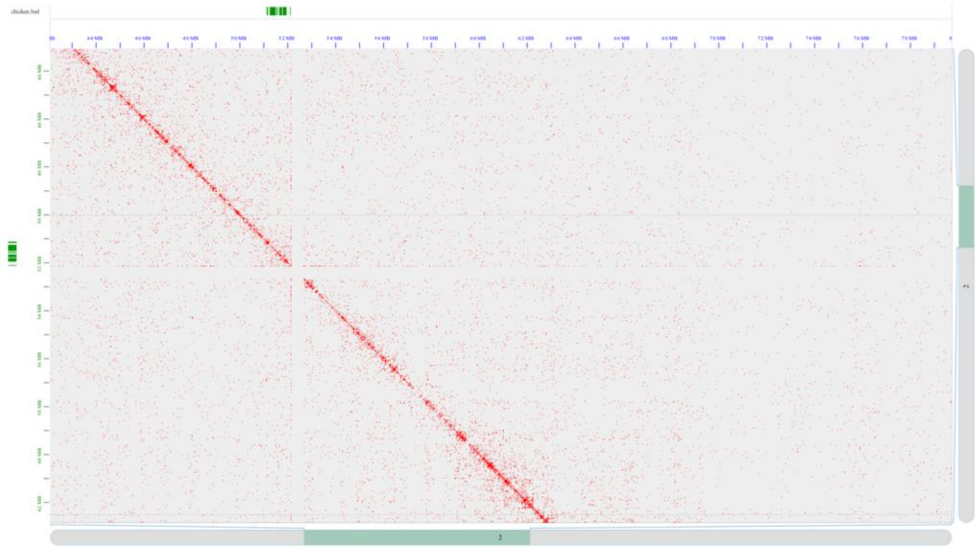

Figure S174: Hi-C figure generated using Juicebox of chicken Mature erythrocytes (SRR5330744) where red dots show the genomic loci interaction between adjacent regions and gap shows there is no interaction between the nearby region. We loaded the bed file on the top and left sides of the Hi-C plot, where each line of the green color indicates the gene of interest (*COA1)* and adjacent syntenic regions. chromosome or scaffold is mentioned on the bottom and right sides of the plot.

Figure S175

chicken Mature erythrocytes Hi-C (SRR5330745) *COA1* region

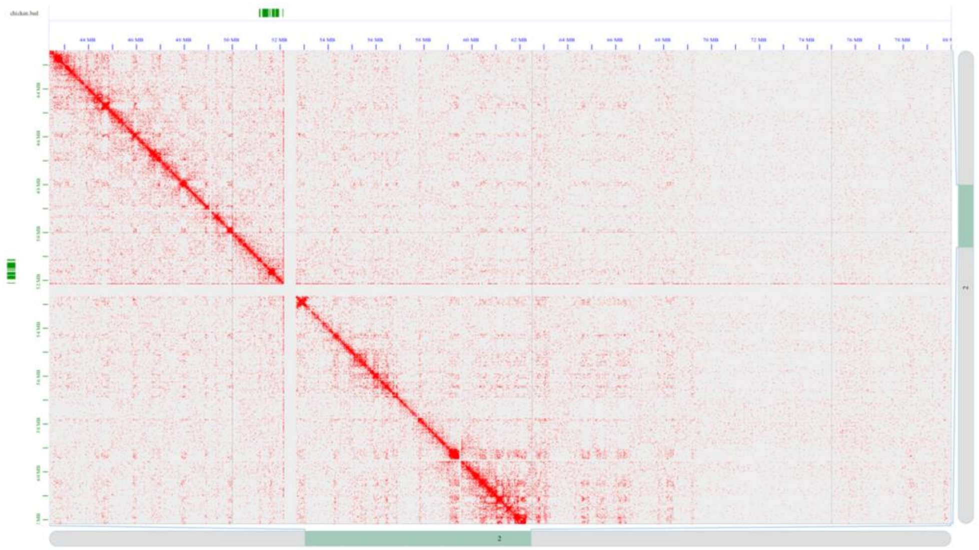

Figure S175: Hi-C figure generated using Juicebox of chicken Mature erythrocytes (SRR5330745) where red dots show the genomic loci interaction between adjacent regions and gap shows there is no interaction between the nearby region. We loaded the bed file on the top and left sides of the Hi-C plot, where each line of the green color indicates the gene of interest (*COA1)* and adjacent syntenic regions. chromosome or scaffold is mentioned on the bottom and right sides of the plot.

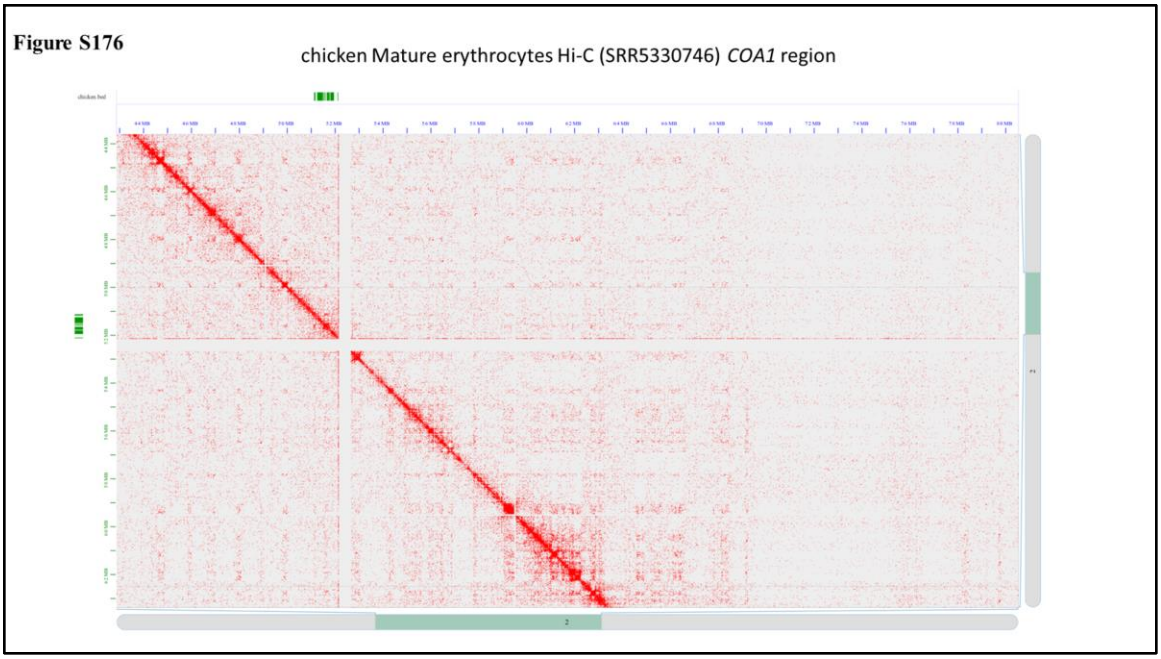

Figure S176: Hi-C figure generated using Juicebox of chicken Mature erythrocytes (SRR5330746) where red dots show the genomic loci interaction between adjacent regions and gap shows there is no interaction between the nearby region. We loaded the bed file on the top and left sides of the Hi-C plot, where each line of the green color indicates the gene of interest (*COA1)* and adjacent syntenic regions. chromosome or scaffold is mentioned on the bottom and right sides of the plot.

Figure S177

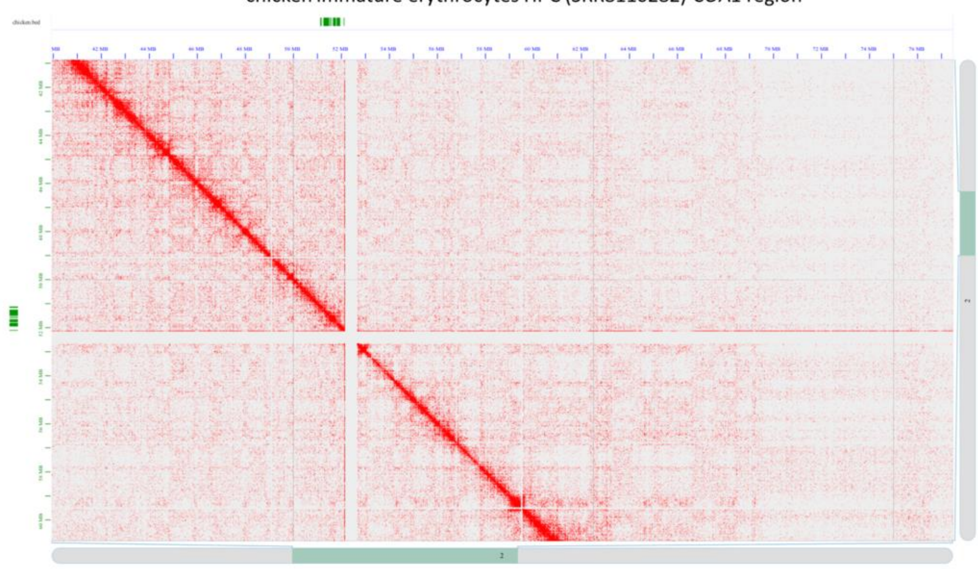

Figure S177: Hi-C figure generated using Juicebox of chicken immature erythrocytes data (SRR8110282) where red dots show the genomic loci interaction between adjacent regions and gap shows there is no interaction between the nearby region. We loaded the bed file on the top and left sides of the Hi-C plot, where each line of the green color indicates the gene of interest (*COA1)* and adjacent syntenic regions. chromosome or scaffold is mentioned on the bottom and right sides of the plot.

Figure S178

chicken immature erythrocytes Hi-C (SRR8110283) *COA1* region

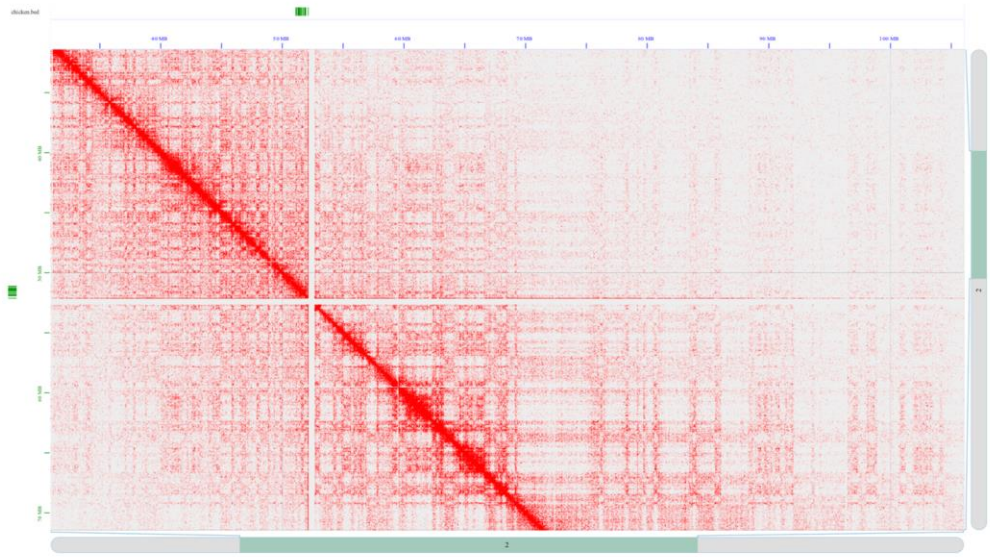

Figure S178: Hi-C figure generated using Juicebox of chicken immature erythrocytes data (SRR8110283) where red dots show the genomic loci interaction between adjacent regions and gap shows there is no interaction between the nearby region. We loaded the bed file on the top and left sides of the Hi-C plot, where each line of the green color indicates the gene of interest (*COA1)* and adjacent syntenic regions. chromosome or scaffold is mentioned on the bottom and right sides of the plot.

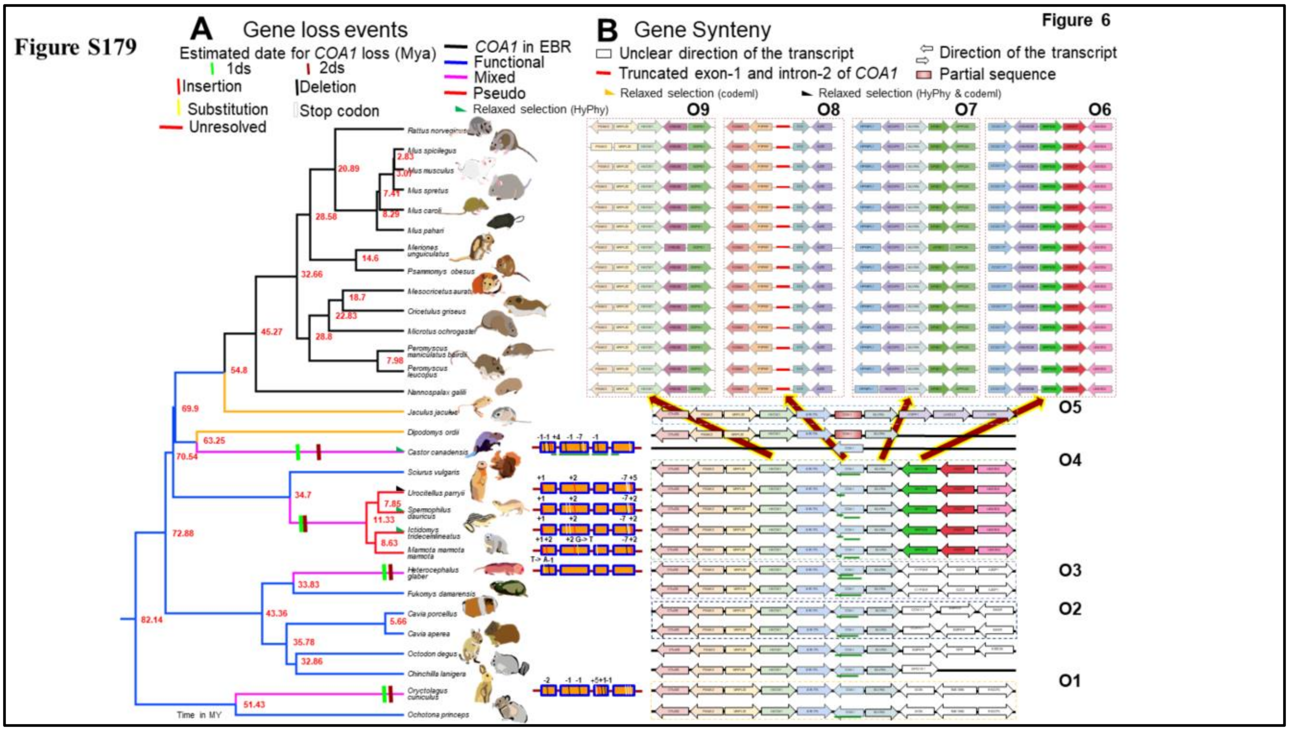

Figure S179: Recurrent loss of *COA1* gene in rodent species. (**A**) Gene loss events in seven rodent species through four events are represented exon-wise beside the time-calibrated phylogenetic tree obtained from the TimeTree website. Blue branches correspond to functional copies of *COA1*, and black branches correspond to the Evolutionary Breakpoint Region (EBR), pink banches correcsponds to mixed branches and red branches represnts pseudo branches. (**B**). Gene order in the genomic region flanking the *COA1* gene in rodent genomes. Arrows depict the direction of gene transcription relative to the *COA1* gene for consistency

across species. Boxes represent the genes located on short scaffolds with unknown orientation. Each dotted box contains one type of gene order, and the brown arrows highlighted in yellow emerging from gene order O5 depict the EBR event that leads to gene orders O6, O7, O8, and O9. Gene order O8 and O7 contain partial remains of the *COA1* gene and a functional *BLVRA* gene, respectively. A solid red line within gene order O8 depicts the partial exon one and intron-2 of *COA1* located between the *PTPRF* and *HYI* genes. The gene order O6 and O9 correspond to the regions on the left and right flanks of the region containing *STK17A*, *COA1*, and *BLVRA*.

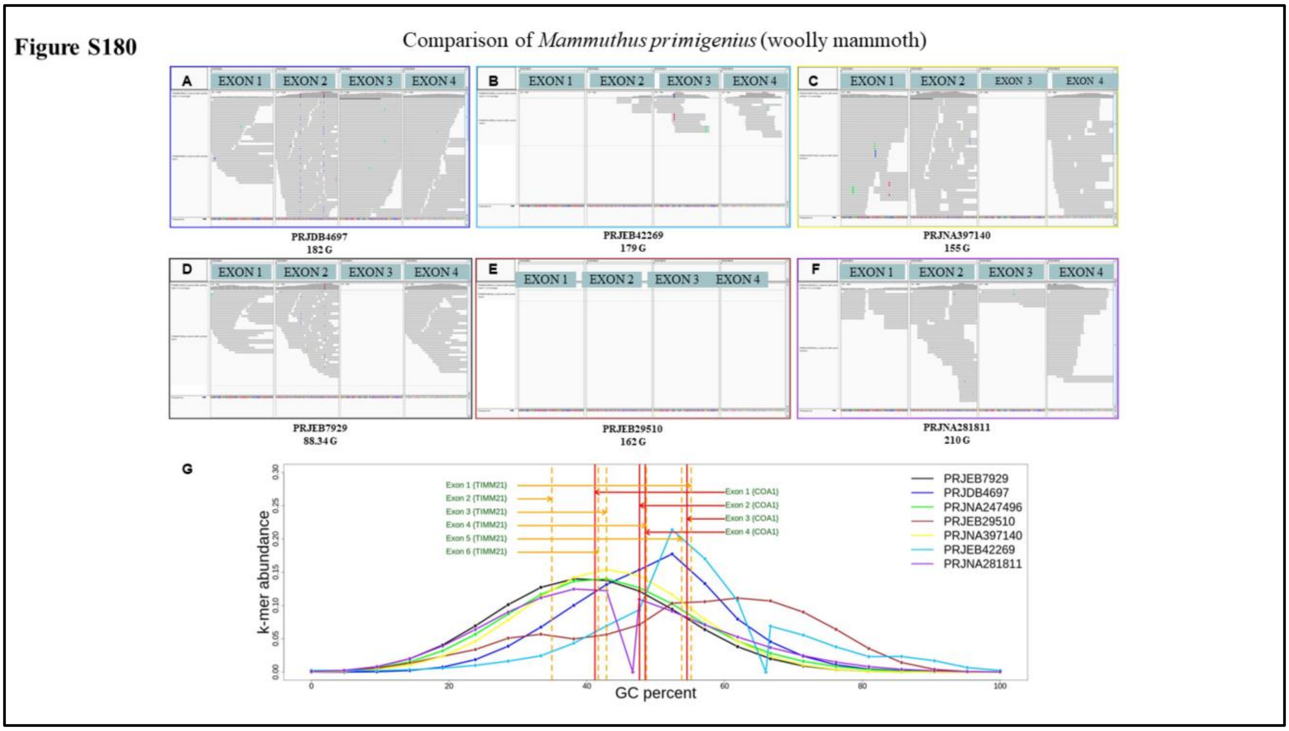

Figure S180: Comparison of different sequencing datasets of woolly mammoth (*Mammuthus primigenius*) for *COA1* gene exons. Gray rectangles show the reads mapped to each exon. Panels **A** to **F** shows the reads with sequence supporting each exon from different woolly mammoth SRA bio projects [PRJDB4697 (182 Gb), PRJEB42269 (179 Gb), PRJNA397140 (155 Gb), PRJEB7929 (88.34 Gb), PRJEB29510 (162 Gb), and PRJNA281811 (210 Gb)]. Panel **G** indicates the GC percentage vs. K-mer abundance of different project IDs mentioned in different colors. The vertical dotted lines in orange denote the GC percentage of *TIMM21* exons, and vertical solid lines in red indicate the GC percentage of *COA1* exons.

Figure S181 *TIMM21* of *Mammuthus primigenius*

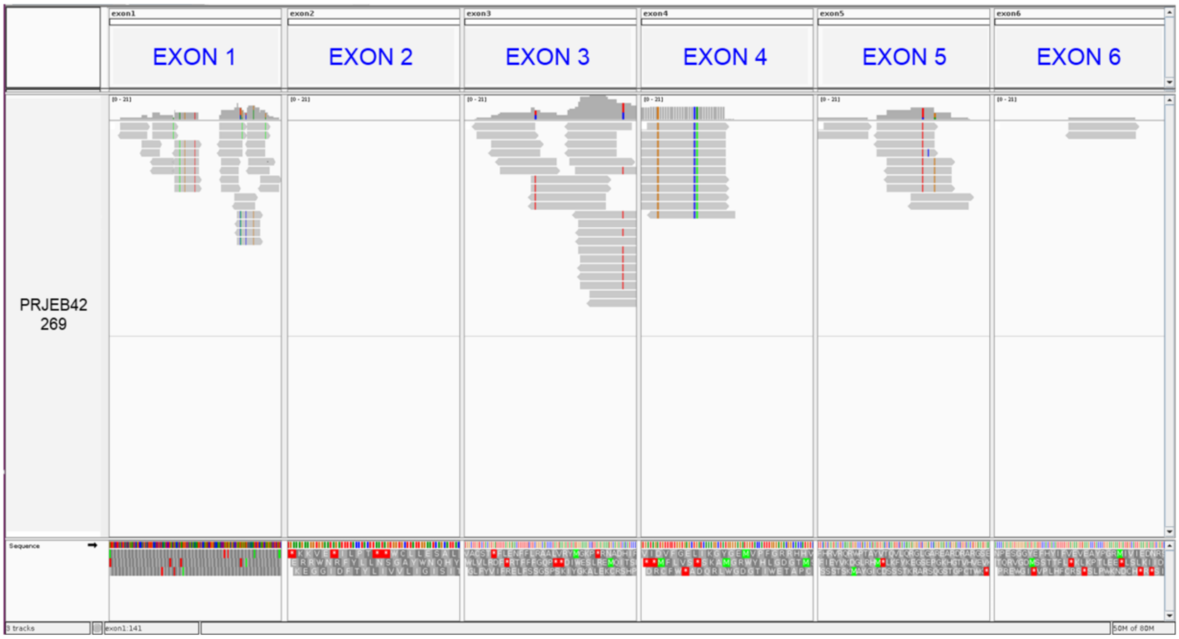

Figure S181: Exon-wise screenshot of *TIMM21* in *Mammuthus primigenius* (woolly mammoth) from Illumina dataset PRJEB42269. Gray boxes are showing reads for particular exons.

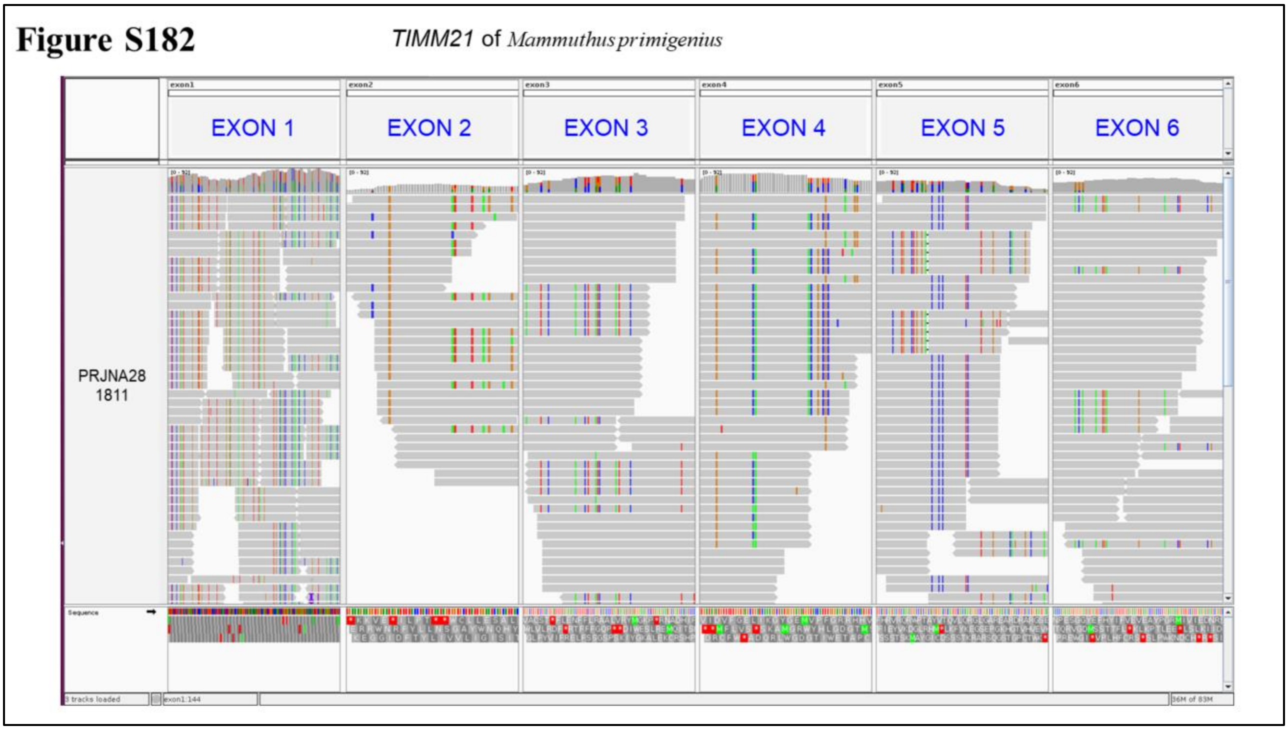

Figure S182: Exon-wise screenshot of *TIMM21* in *Mammuthus primigenius* (woolly mammoth) from Illumina dataset PRJ281811. Gray boxes are showing reads for particular exons.

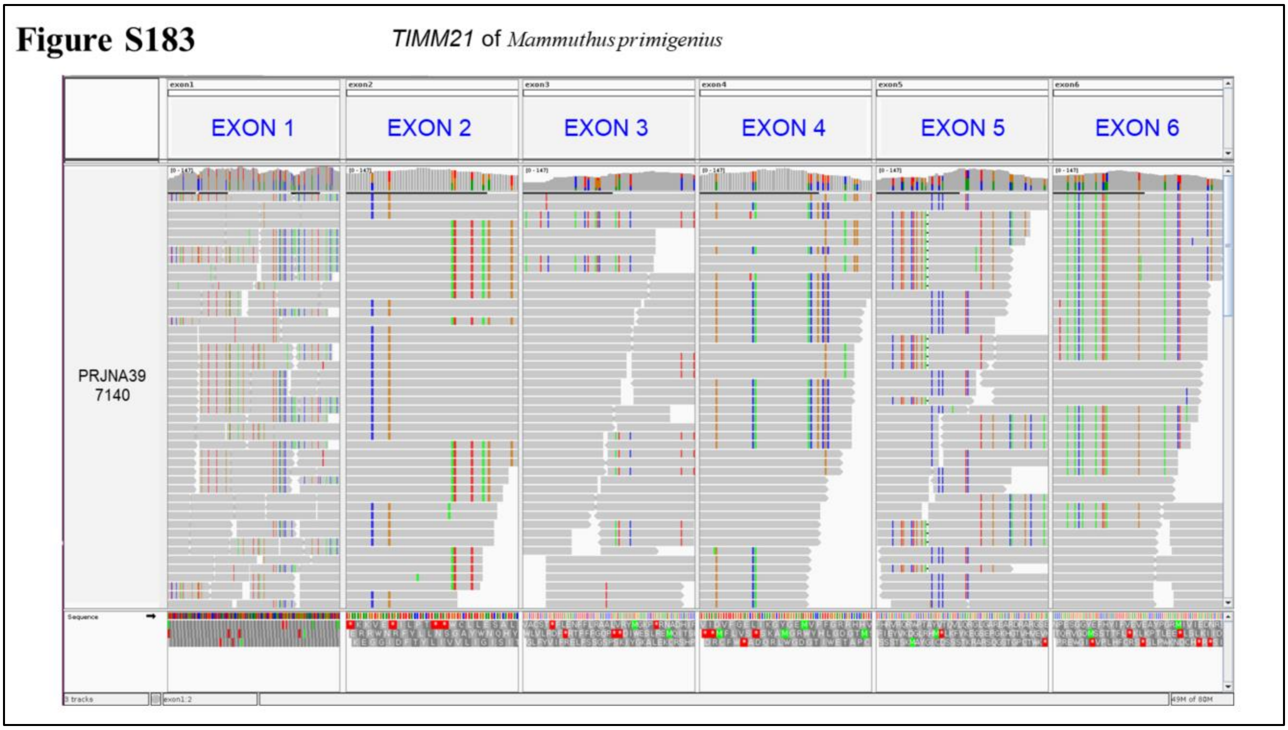

Figure S183: Exon-wise screenshot of *TIMM21* in *Mammuthus primigenius* (woolly mammoth) from Illumina dataset PRJNA397140. Gray boxes are showing reads for particular exons.

Figure S184 *TIMM21* of *Mammuthus primigenius*

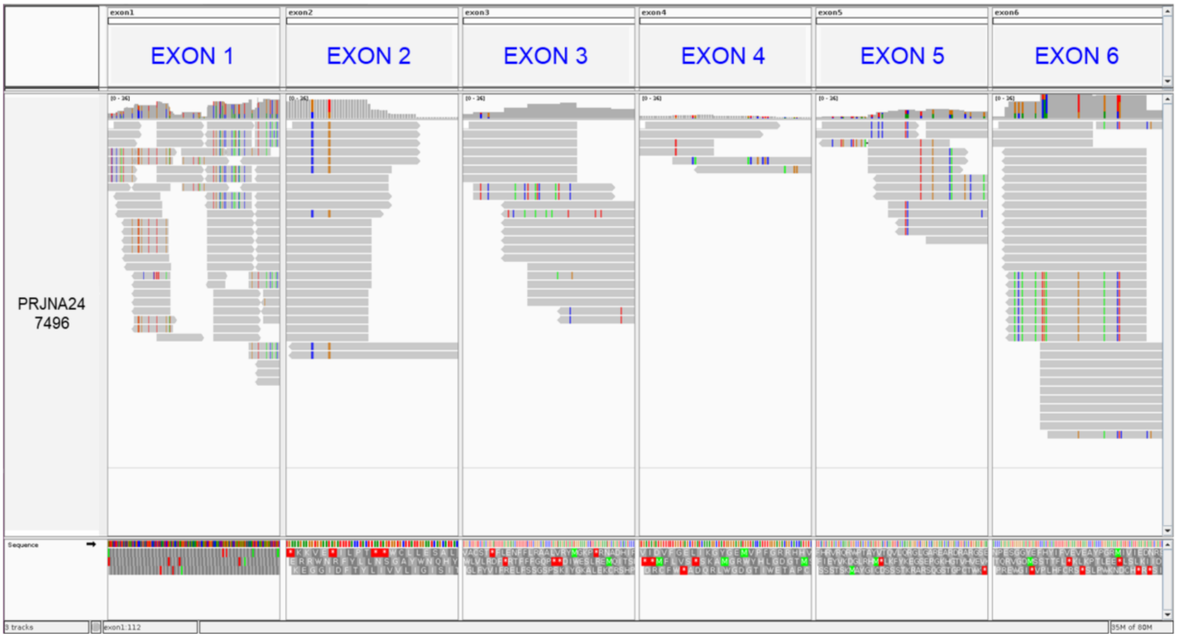

Figure S184: Exon-wise screenshot of *TIMM21* in *Mammuthus primigenius* (woolly mammoth) from Illumina dataset PRJNA247496. Gray boxes are showing reads for particular exons.

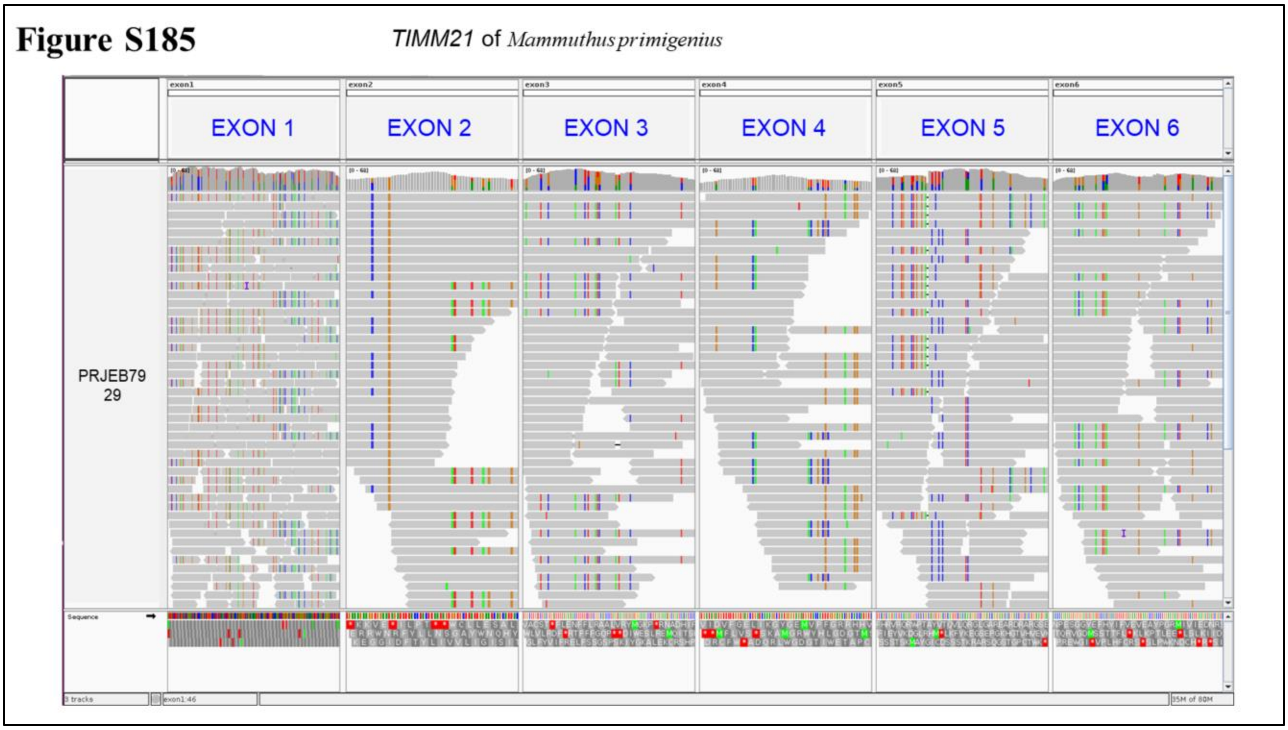

Figure S185: Exon-wise screenshot of *TIMM21* in *Mammuthus primigenius* (woolly mammoth) from Illumina dataset PRJEB7929. Gray boxes are showing reads for particular exons.

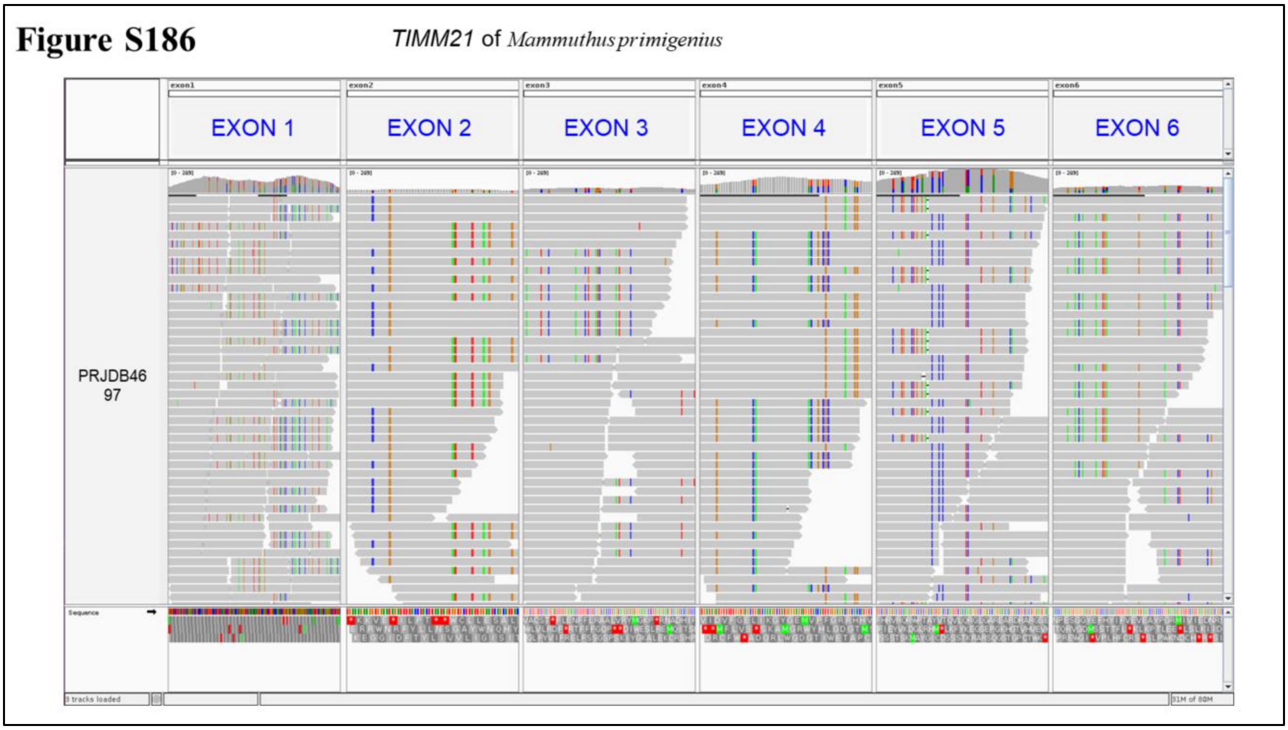

Figure S186: Exon-wise screenshot of *TIMM21* in *Mammuthus primigenius* (woolly mammoth) from Illumina dataset PRJDB4697. Gray boxes are showing reads for particular exons.

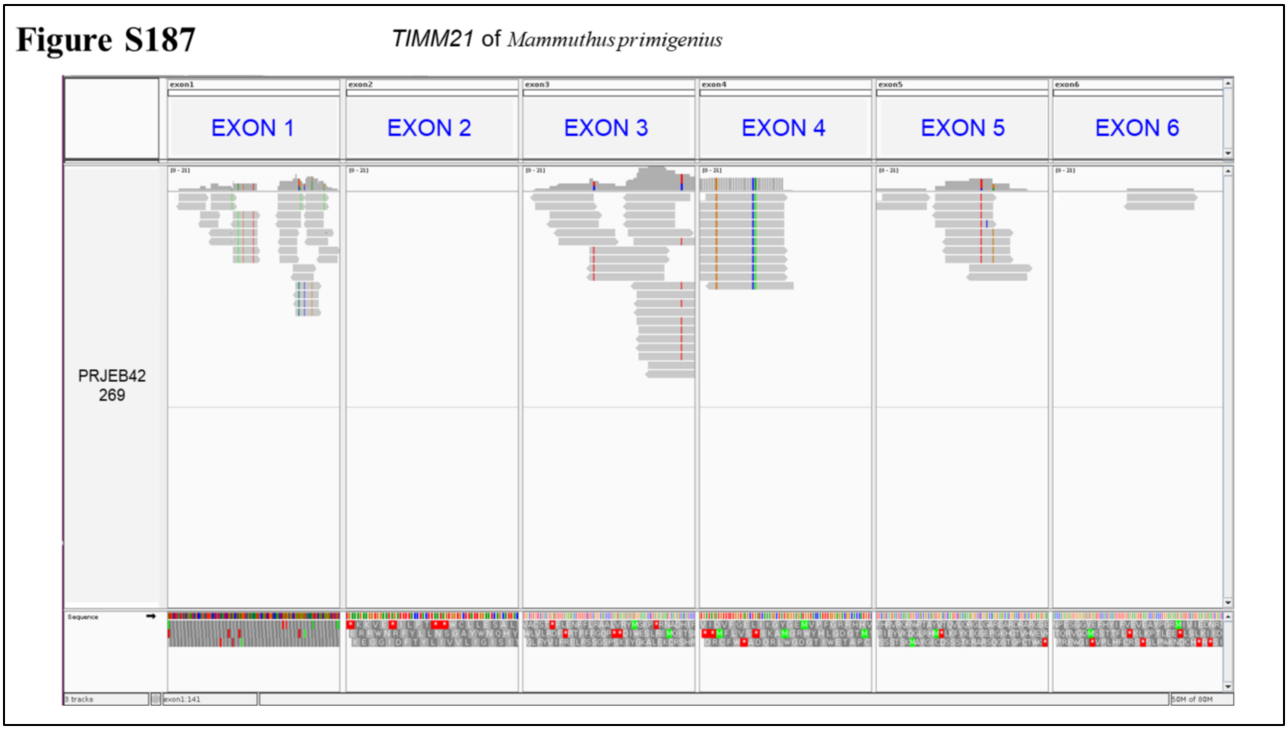

Figure S187: Exon-wise screenshot of *TIMM21* in *Mammuthus primigenius* (woolly mammoth) from Illumina dataset PRJEB42269. Gray boxes are showing reads for particular exons.

Figure S188

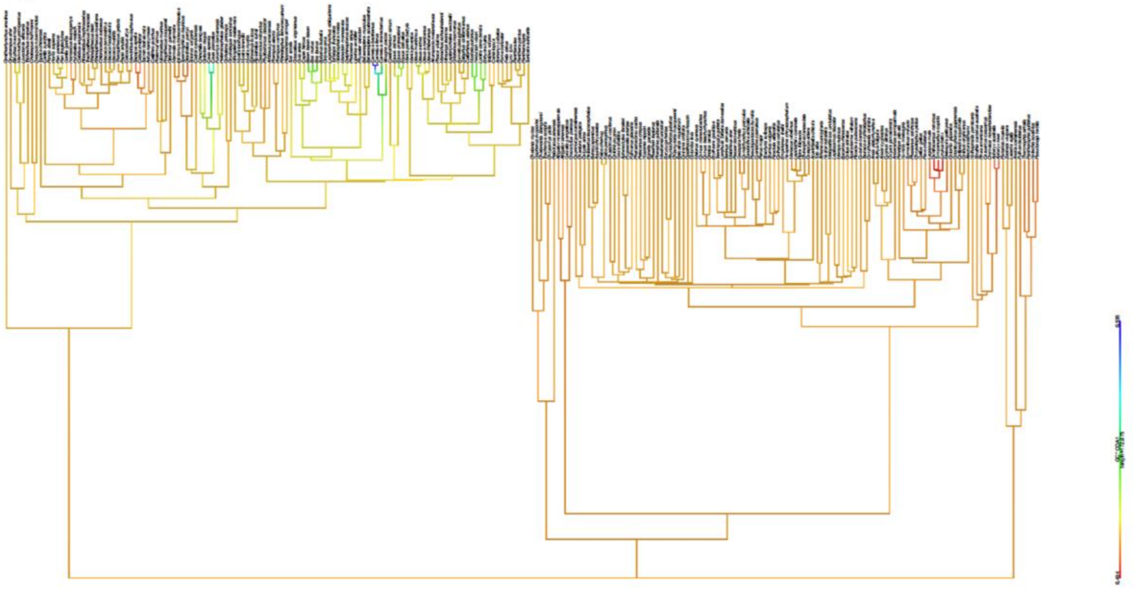

Figure S188: Ratio of GC* in *COA1* across the 240 species. The colour gradient represents the value of GC* for each species are shown with phylogenetic tree downloaded from the TimeTree website.

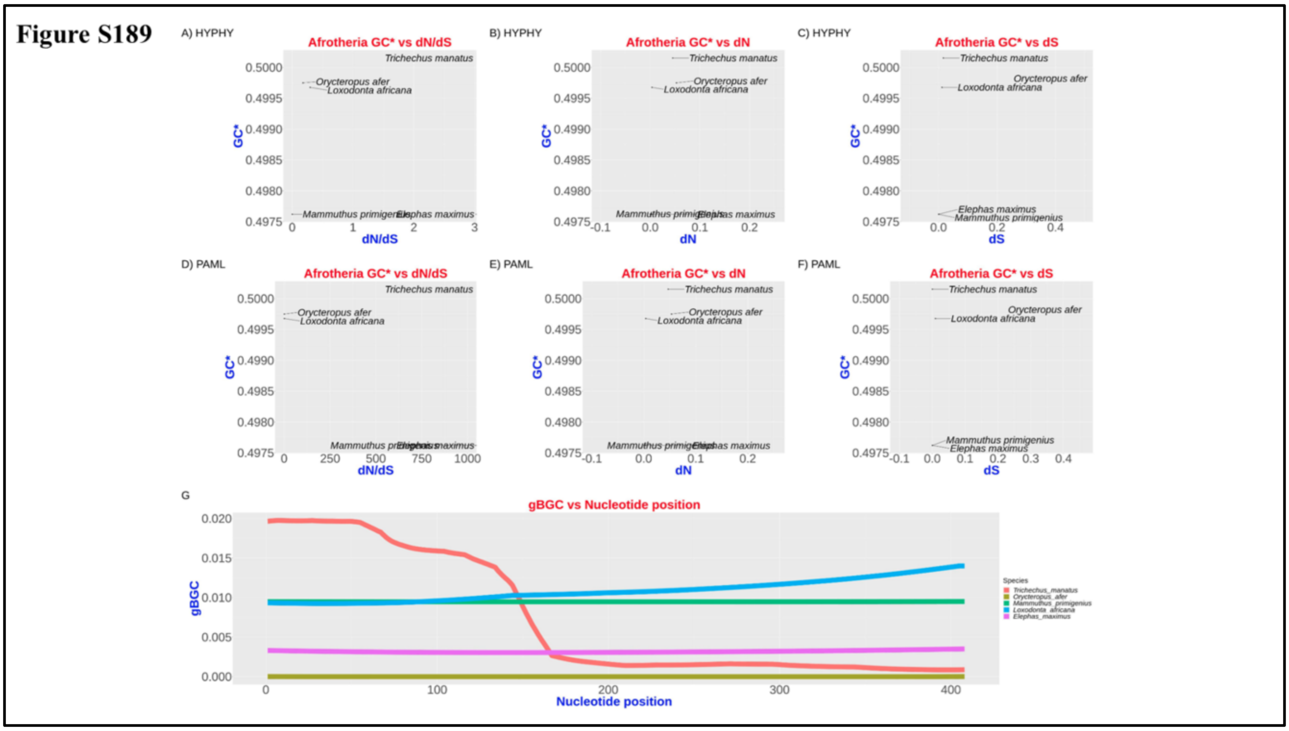

Figure S189: Values of GC* in Afrotheria are plotted with dN and dS which is calculated using mapNH. dN and dS were calculated using PAML and HyPhy both. gBGC across nucleotide position were calculated using PhastBias.

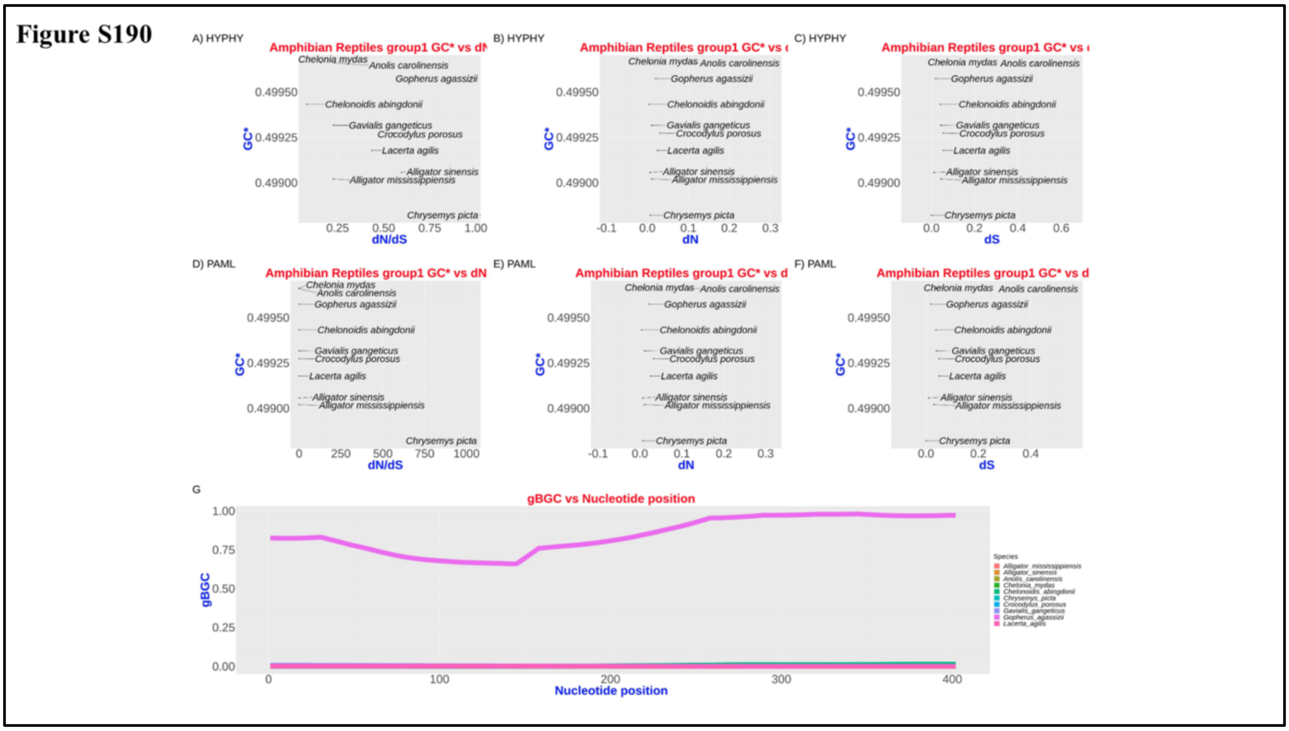

Figure S190: Values of GC* in Amphibian and Reptiles are plotted with dN and dS which is calculated using mapNH. dN and dS were calculated using PAML and HyPhy both. gBGC across nucleotide position were calculated using PhastBias. *Gopherus agassizii* has a significant gBGC across *COA1*.

Figure S191

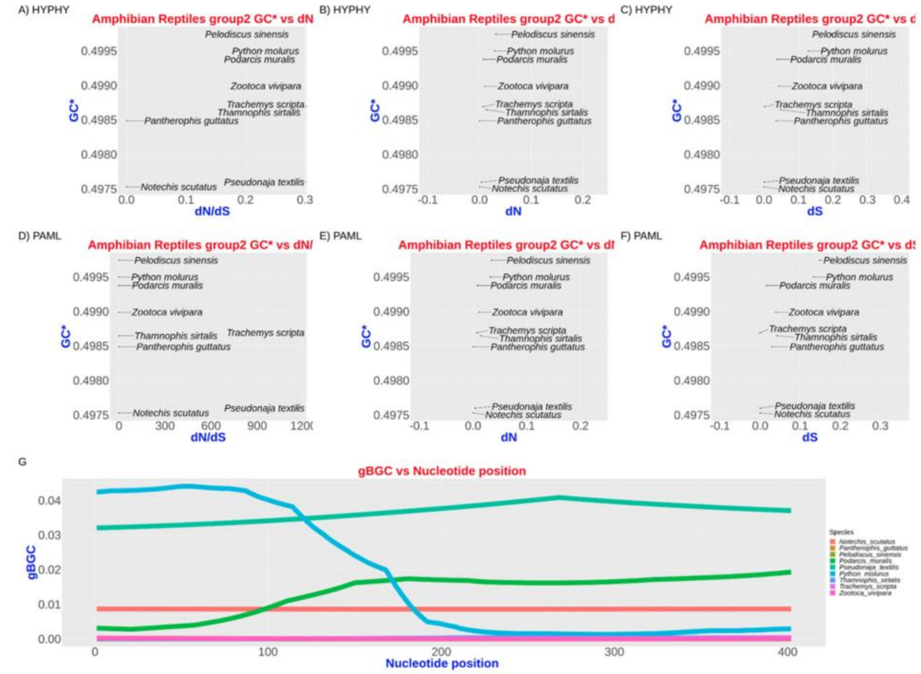

Figure S191: Values of GC* in amphibians and reptiles are plotted with dN and dS which is calculated using mapNH. dN and dS were calculated using PAML and HyPhy both. gBGC across nucleotide position were calculated using PhastBias.

Figure S192

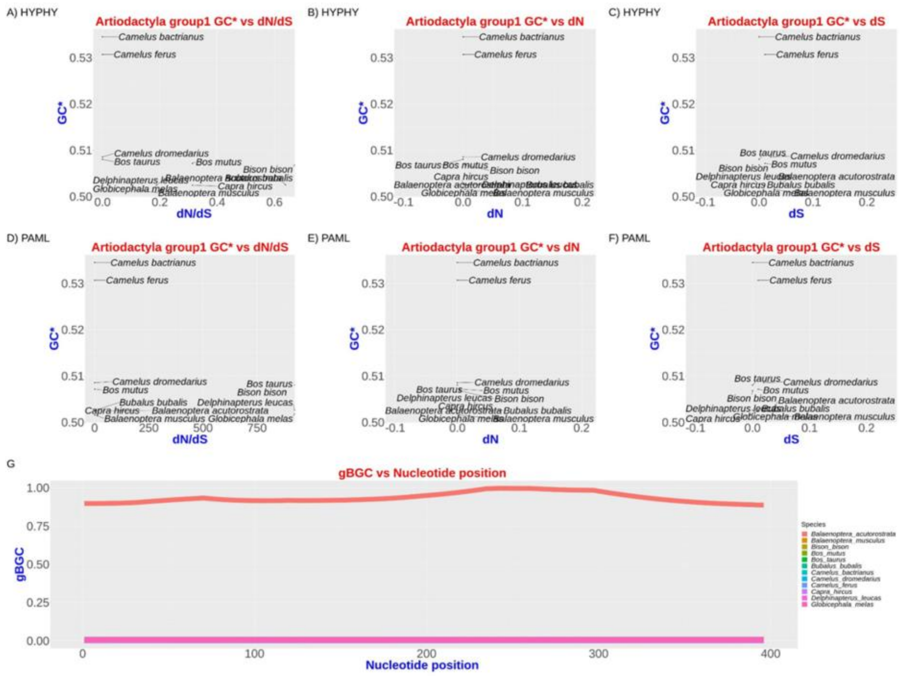

Figure S192: Values of GC* in Artiodactyla are plotted with dN and dS which is calculated using mapNH. dN and dS were calculated using PAML and HyPhy both. gBGC across nucleotide position were calculated using PhastBias. *Balaenoptera acutorostrata* has a significant gBGC across *COA1*.

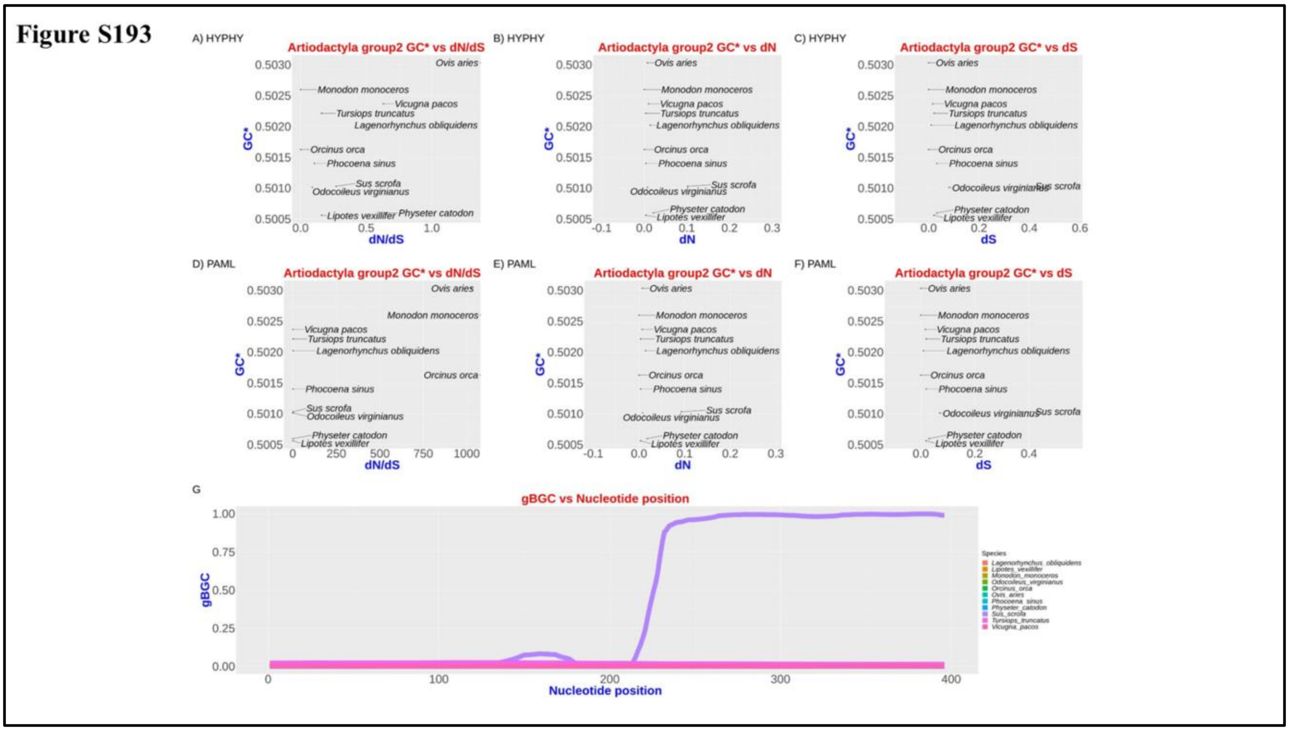

Figure S193: Values of GC* in Artiodactyla are plotted with dN and dS which is calculated using mapNH. dN and dS were calculated using PAML and HyPhy both. gBGC across nucleotide position were calculated using PhastBias. *Sus scrofa* have a significant gBGC in *COA1*.

Figure S194

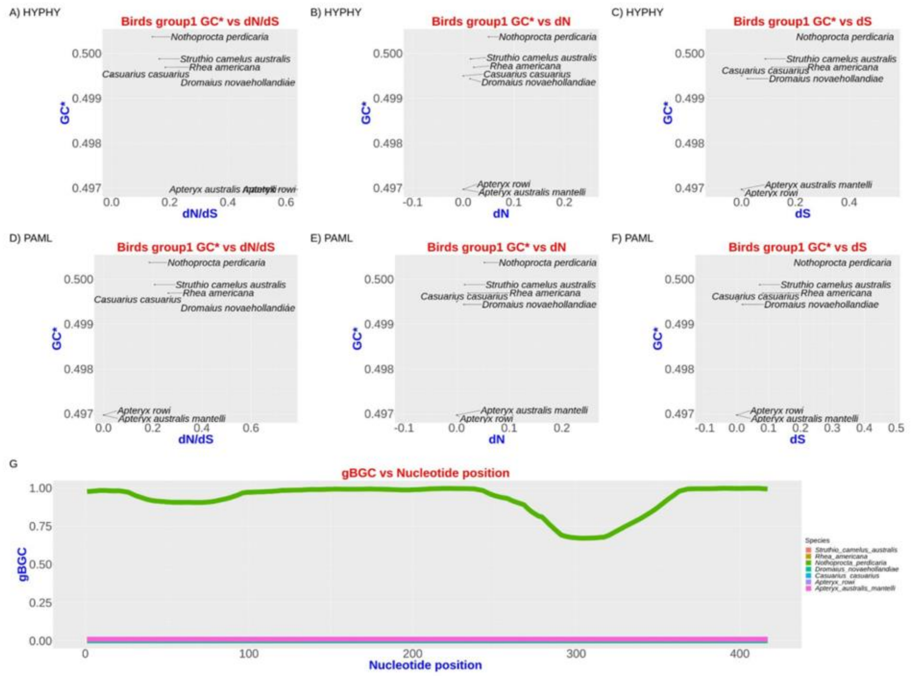

Figure S194: Values of GC* in birds are plotted with dN and dS which is calculated using mapNH. dN and dS were calculated using PAML and HyPhy both. gBGC across nucleotide position were calculated using PhastBias. *Nothoprocta perdicaria* has a significant gBGC across *COA1*.

Figure S195

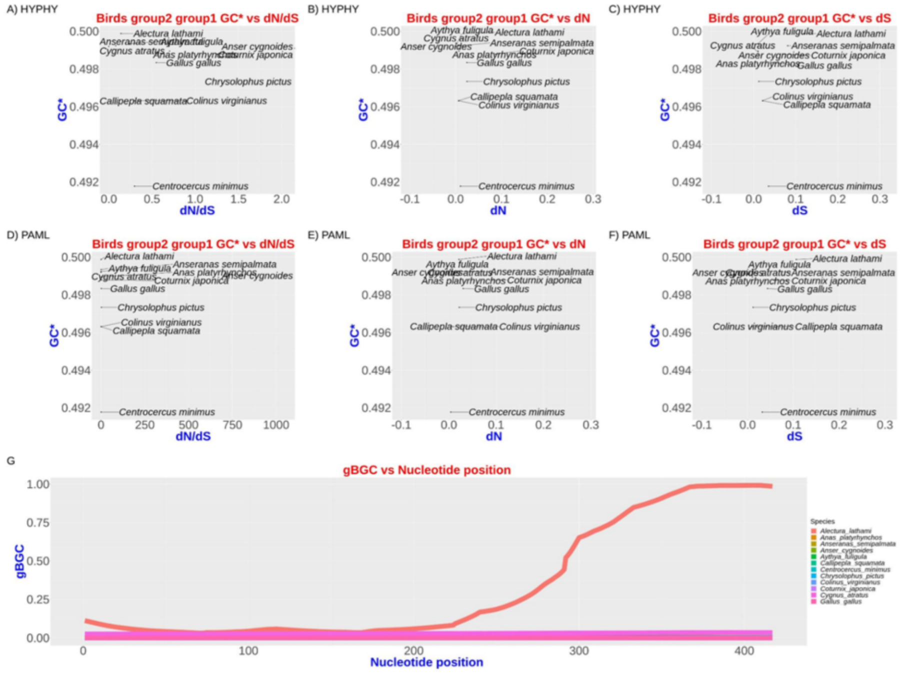

Figure S195: Values of GC* in birds are plotted with dN and dS which is calculated using mapNH. dN and dS were calculated using PAML and HyPhy both. gBGC across nucleotide position were calculated using PhastBias. *Alectura lathami* has a significant gBGC in *COA1*.

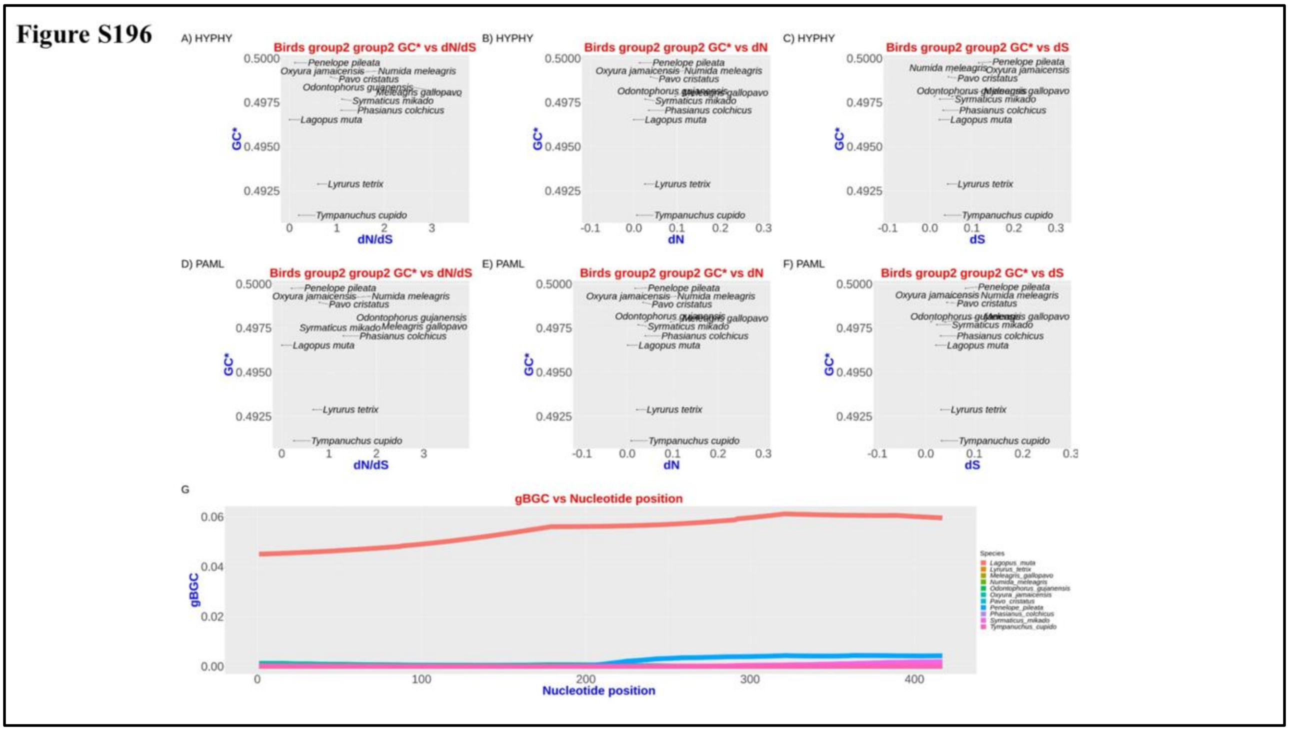

Figure S196: Values of GC* in birds are plotted with dN and dS which is calculated using mapNH. dN and dS were calculated using PAML and HyPhy both. gBGC across nucleotide position were calculated using PhastBias.

Figure S197

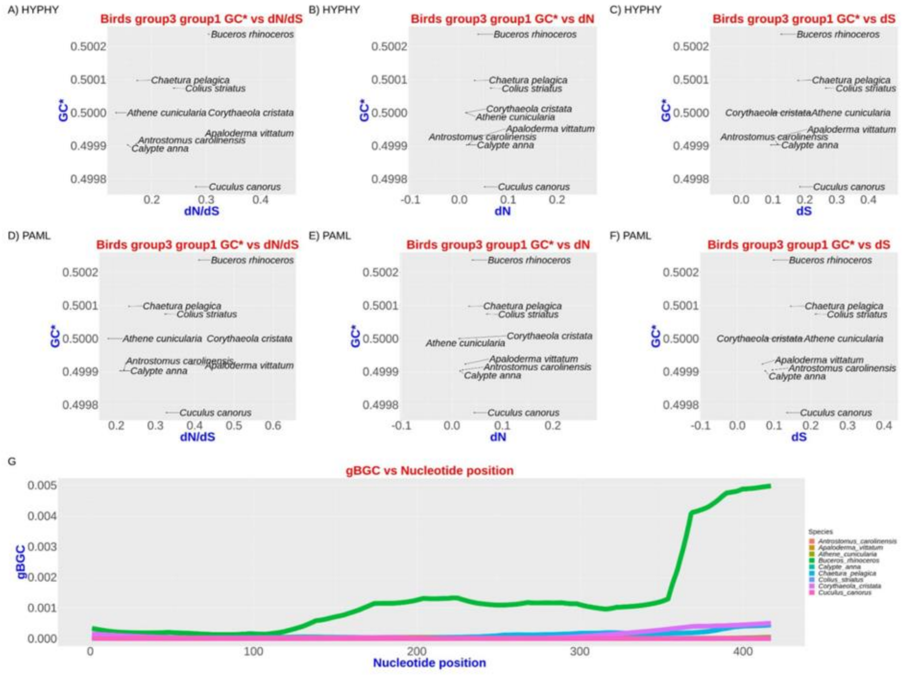

Figure S197: Values of GC* in birds are plotted with dN and dS which is calculated using mapNH. dN and dS were calculated using PAML and HyPhy both. gBGC across nucleotide position were calculated using PhastBias.

Figure S198

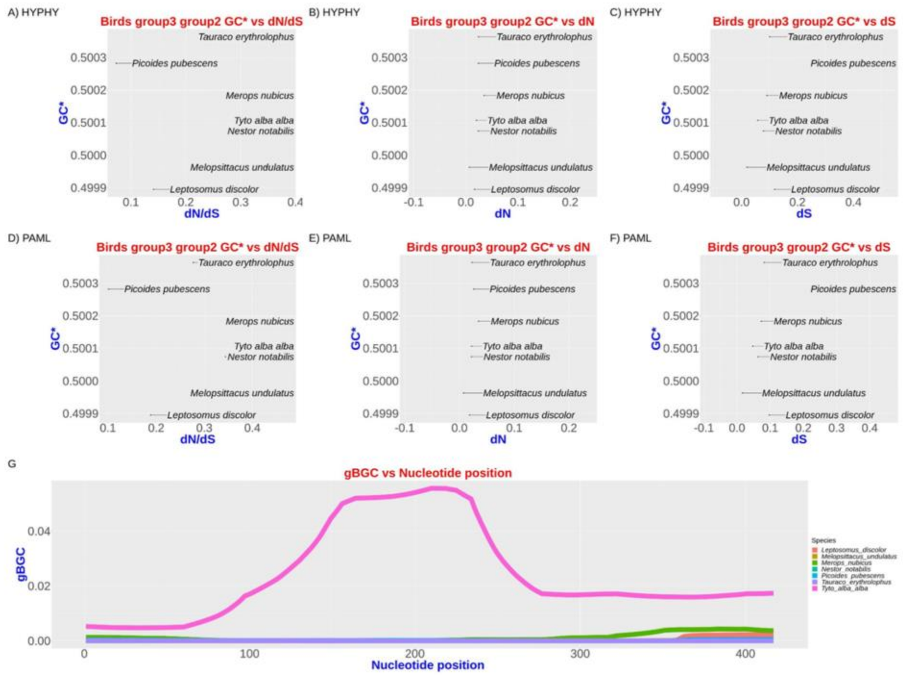

Figure S198: Values of GC* in birds are plotted with dN and dS which is calculated using mapNH. dN and dS were calculated using PAML and HyPhy both. gBGC across nucleotide position were calculated using PhastBias.

Figure S199

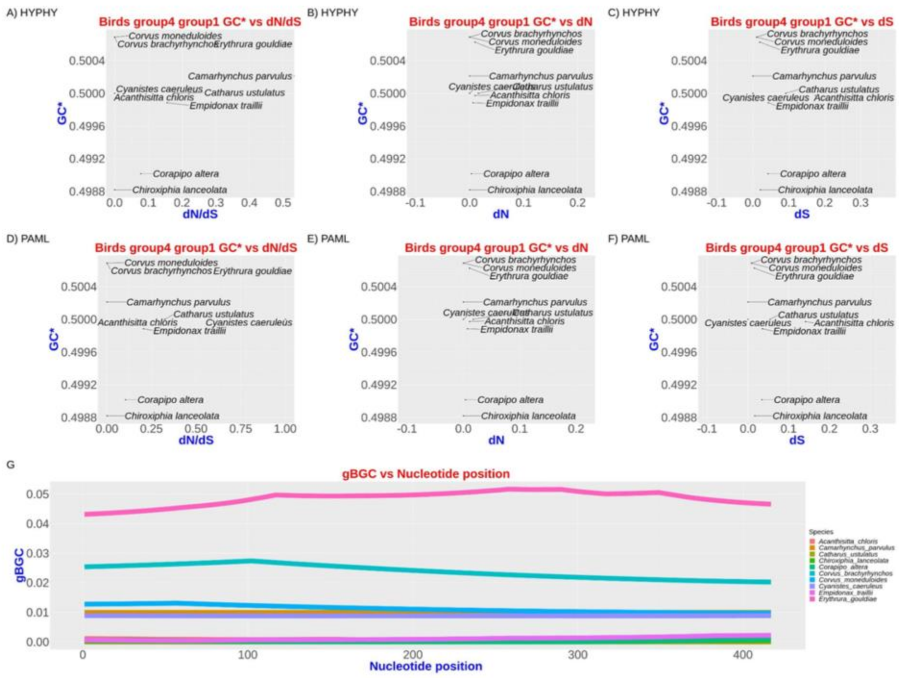

Figure S199: Values of GC* in birds are plotted with dN and dS which is calculated using mapNH. dN and dS were calculated using PAML and HyPhy both. gBGC across nucleotide position were calculated using PhastBias.

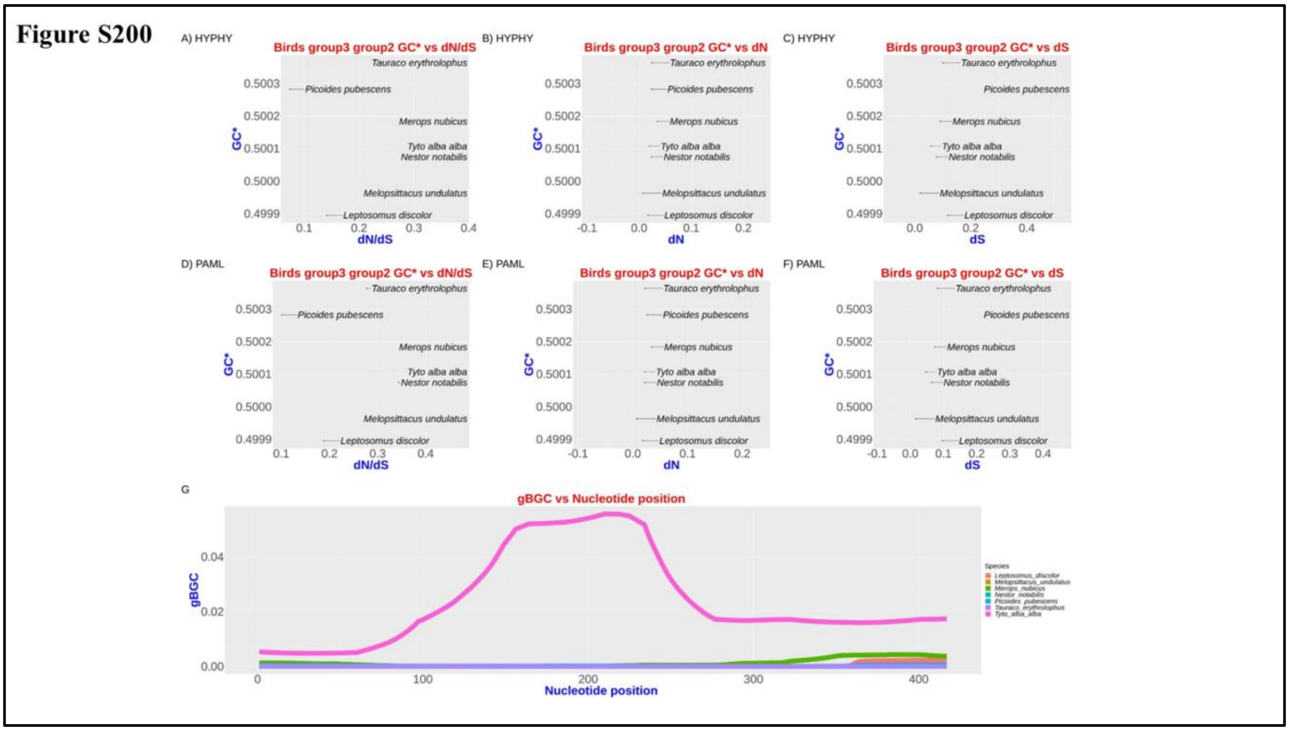

Figure S200: Values of GC* in birds are plotted with dN and dS which is calculated using mapNH. dN and dS were calculated using PAML and HyPhy both. gBGC across nucleotide position were calculated using PhastBias.

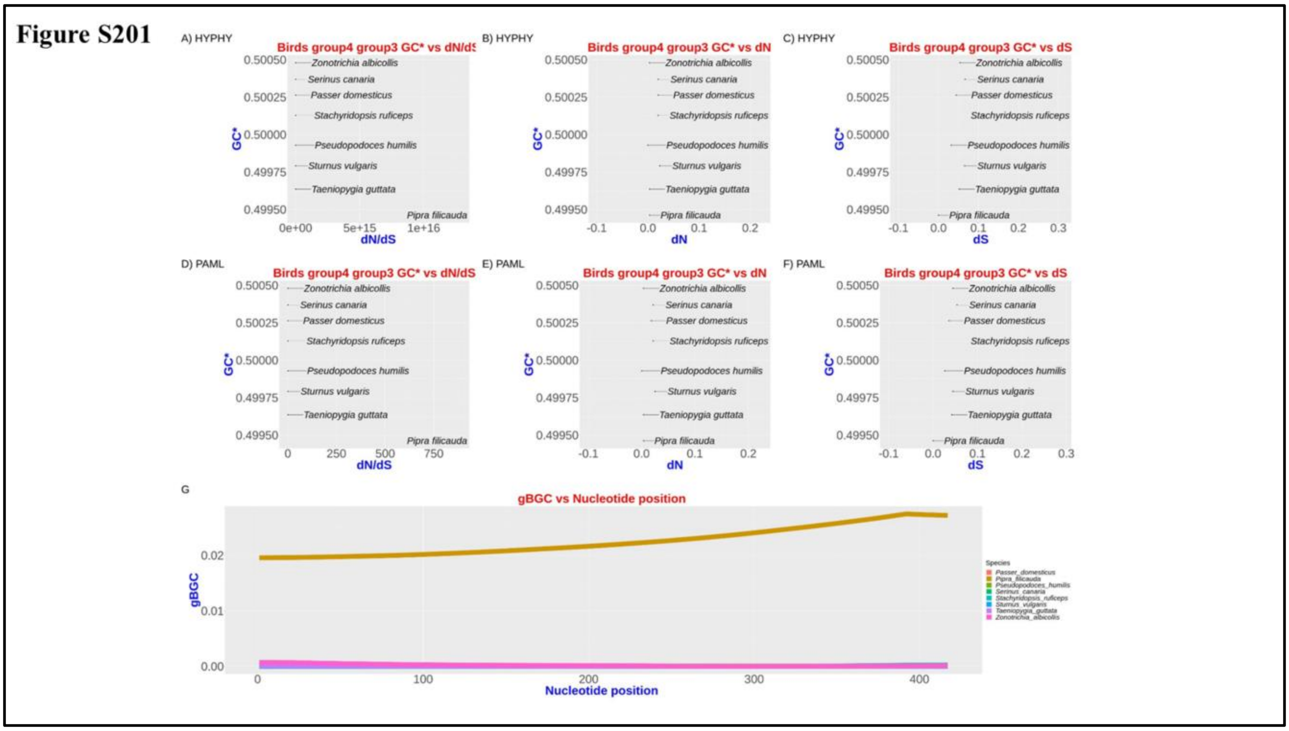

Figure S201: Values of GC* in birds are plotted with dN and dS which is calculated using mapNH. dN and dS were calculated using PAML and HyPhy both. gBGC across nucleotide position were calculated using PhastBias.

Figure S202

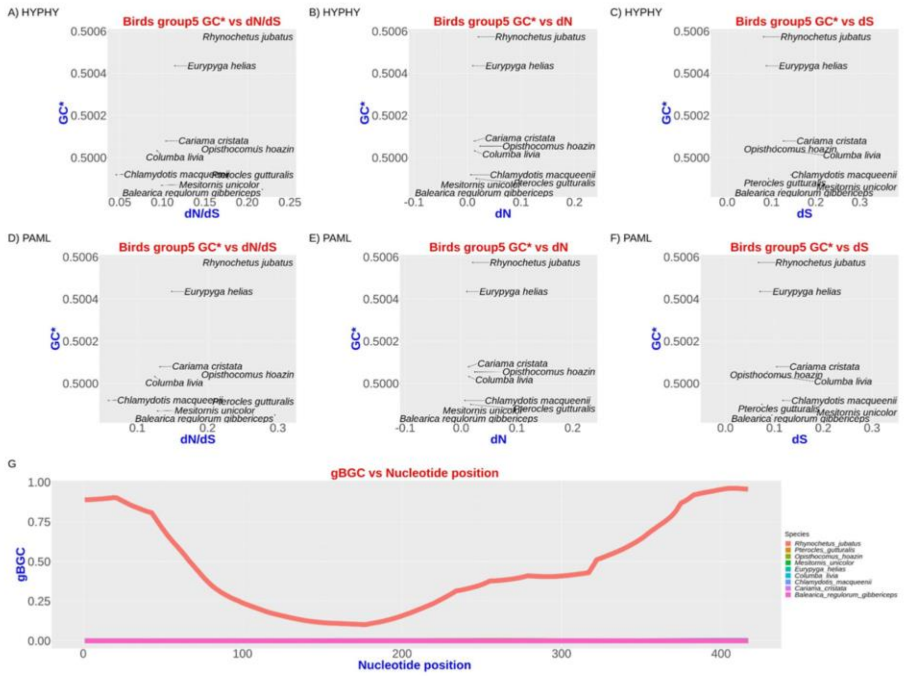

Figure S202: Values of GC* in birds are plotted with dN and dS which is calculated using mapNH. dN and dS were calculated using PAML and HyPhy both. gBGC across nucleotide position were calculated using PhastBias. *Rhynochetos jubatus* has a significant gBGC in *COA1*.

Figure S203

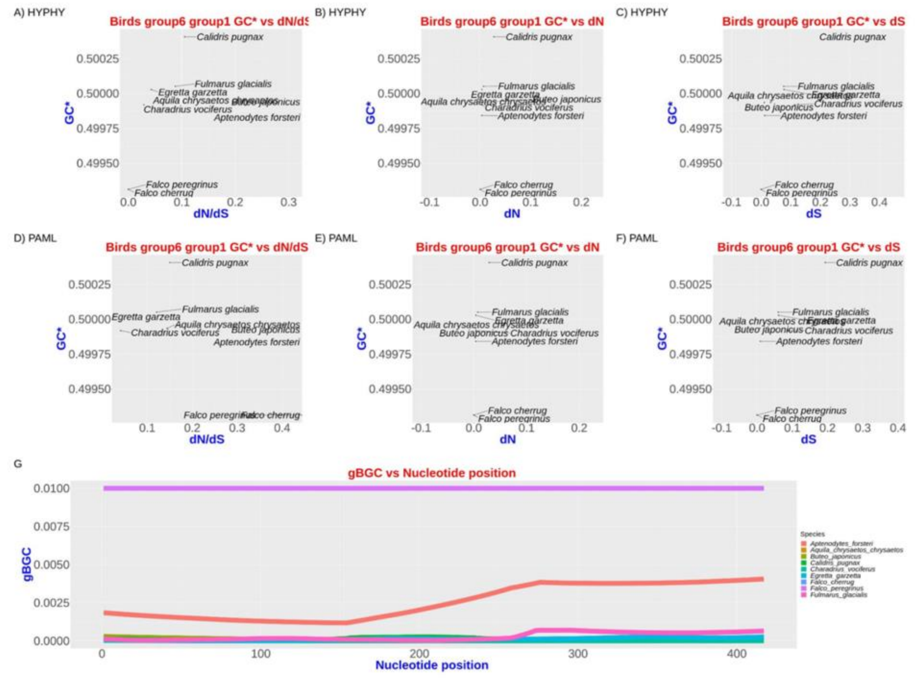

Figure S203: Values of GC* in birds are plotted with dN and dS which is calculated using mapNH. dN and dS were calculated using PAML and HyPhy both. gBGC across nucleotide position were calculated using PhastBias.

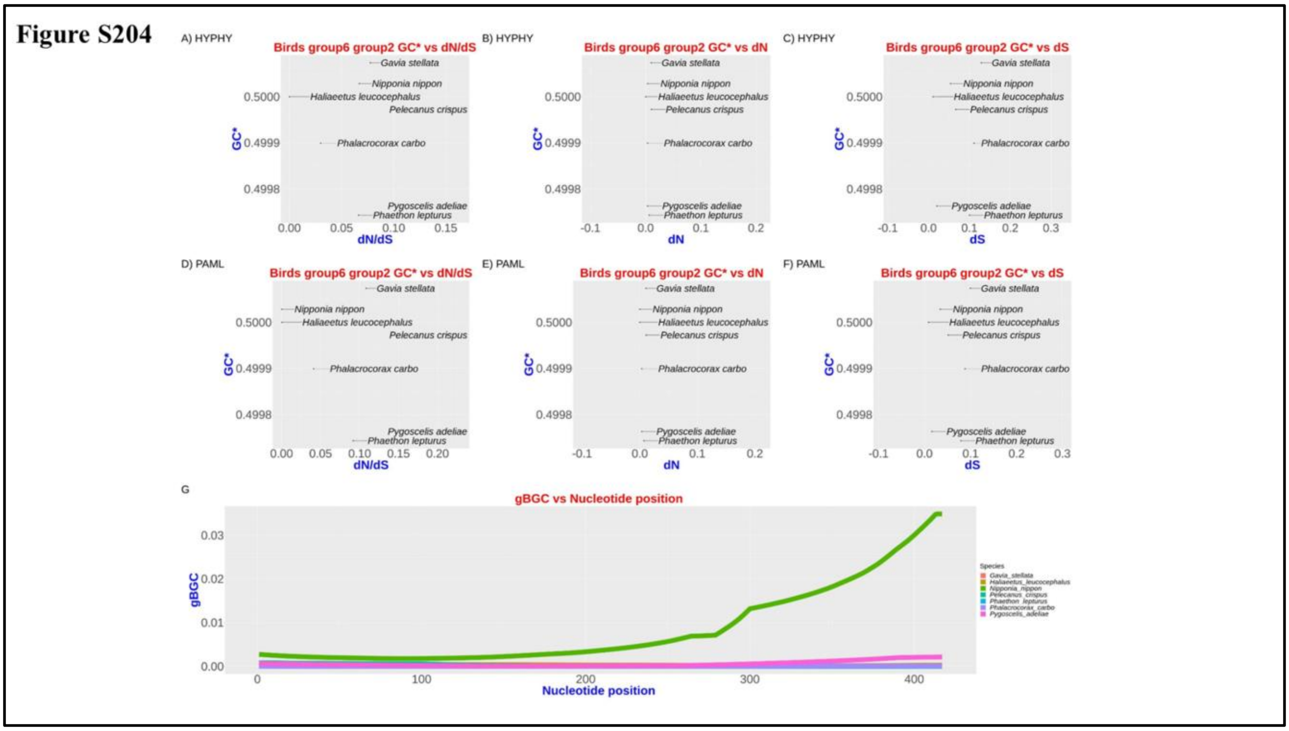

Figure S204: Values of GC* in birds are plotted with dN and dS which is calculated using mapNH. dN and dS were calculated using PAML and HyPhy both. gBGC across nucleotide position were calculated using PhastBias.

Figure S205

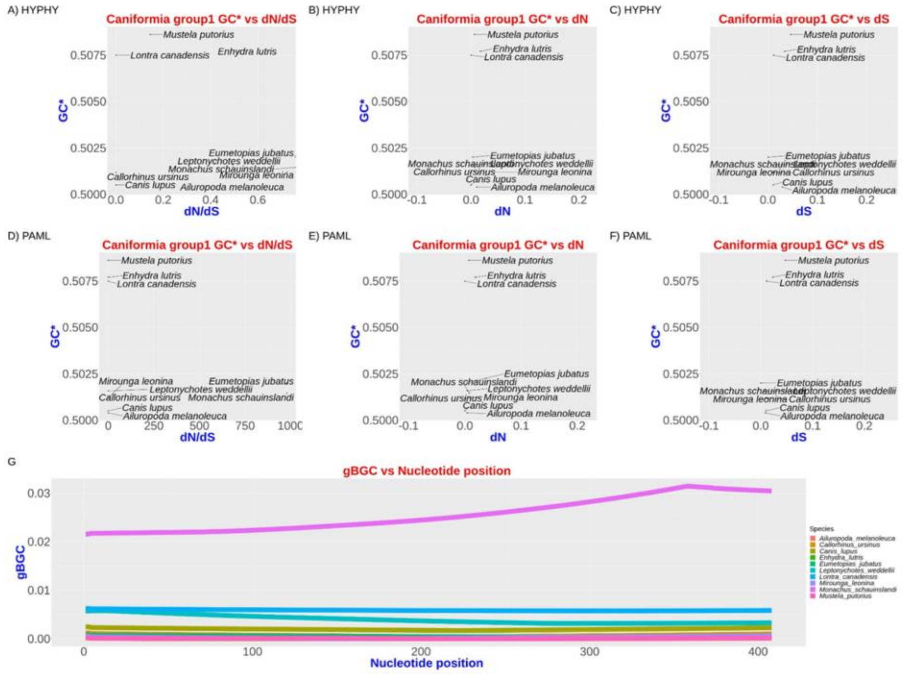

Figure S205: Values of GC* in Caniformia are plotted with dN and dS which is calculated using mapNH. dN and dS were calculated using PAML and HyPhy both. gBGC across nucleotide position were calculated using PhastBias.

Figure S206

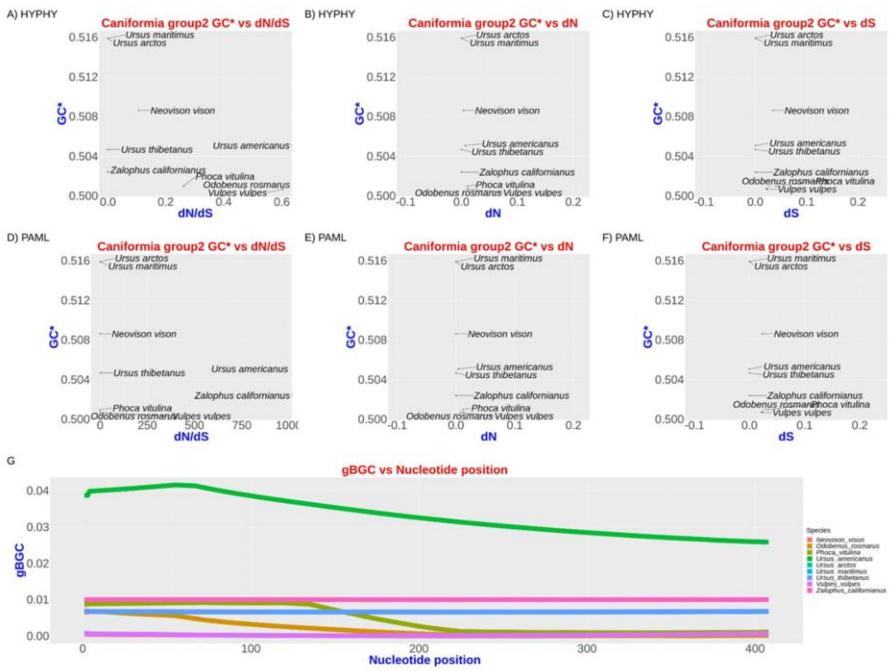

Figure S206: Values of GC* in Caniformia are plotted with dN and dS which is calculated using mapNH. dN and dS were calculated using PAML and HyPhy both. gBGC across nucleotide position were calculated using PhastBias.

Figure S207

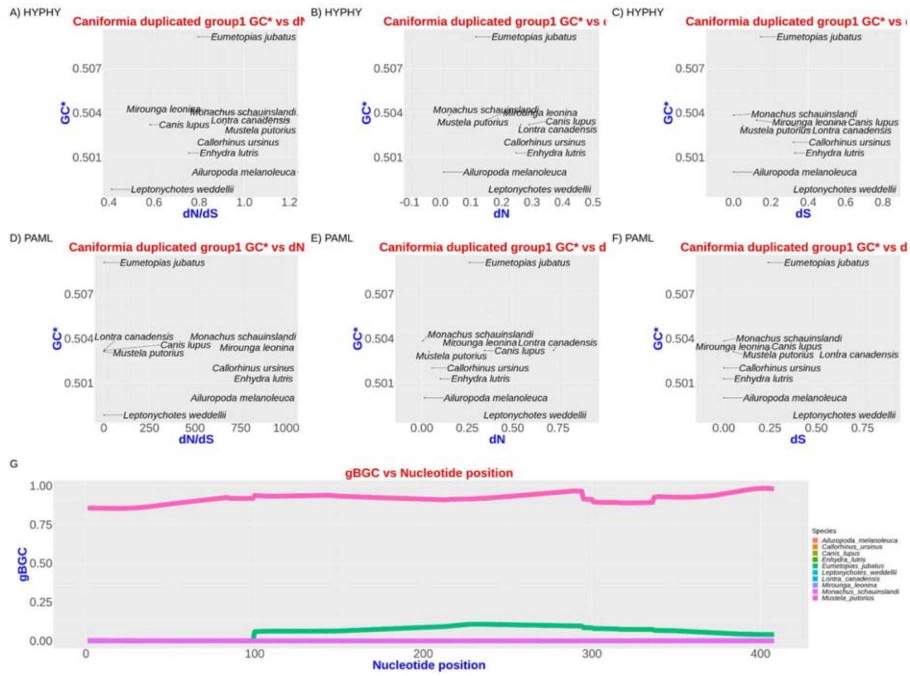

Figure S207: Values of GC* in Caniformia duplicated *COA1* are plotted with dN and dS which is calculated using mapNH. dN and dS were calculated using PAML and HyPhy both. gBGC across nucleotide position were calculated using PhastBias. *Mustela putorius* has a significant gBGC across *COA1.*

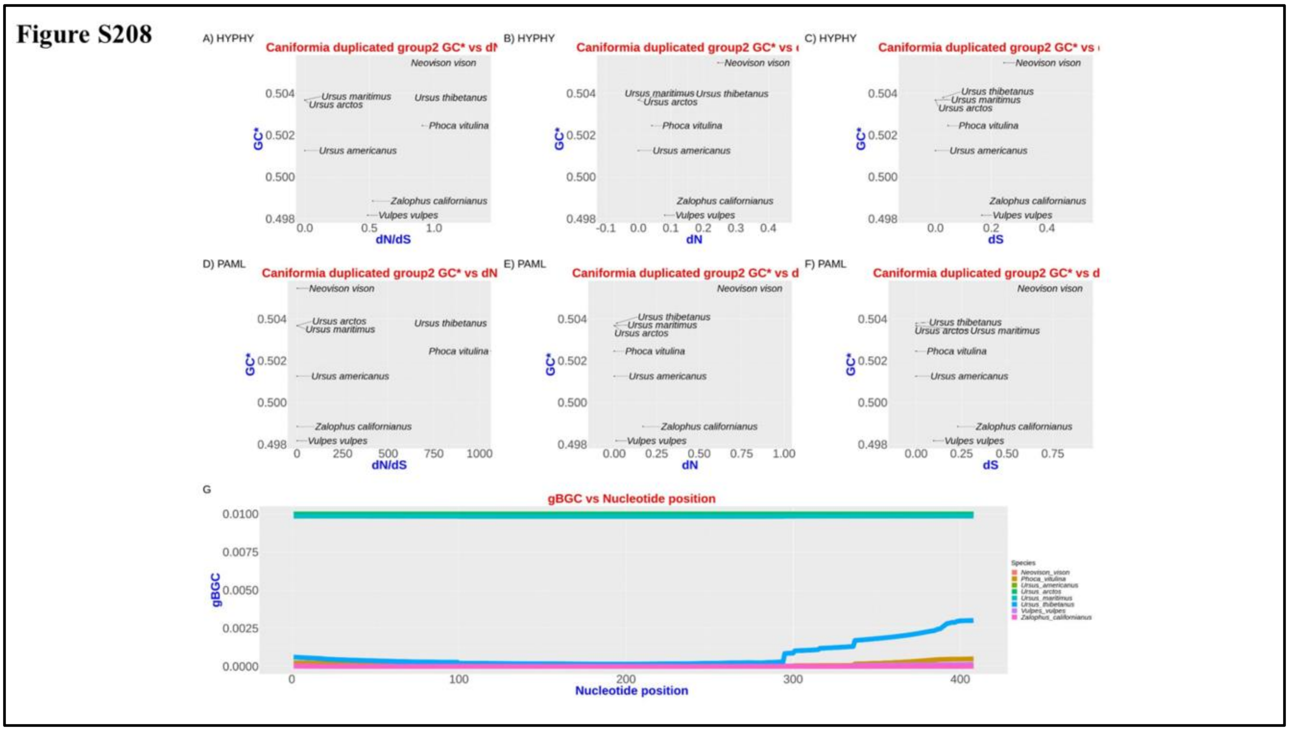

Figure S208: Values of GC* in Caniformia duplicated *COA1* are plotted with dN and dS which is calculated using mapNH. dN and dS were calculated using PAML and HyPhy both. gBGC across nucleotide position were calculated using PhastBias.

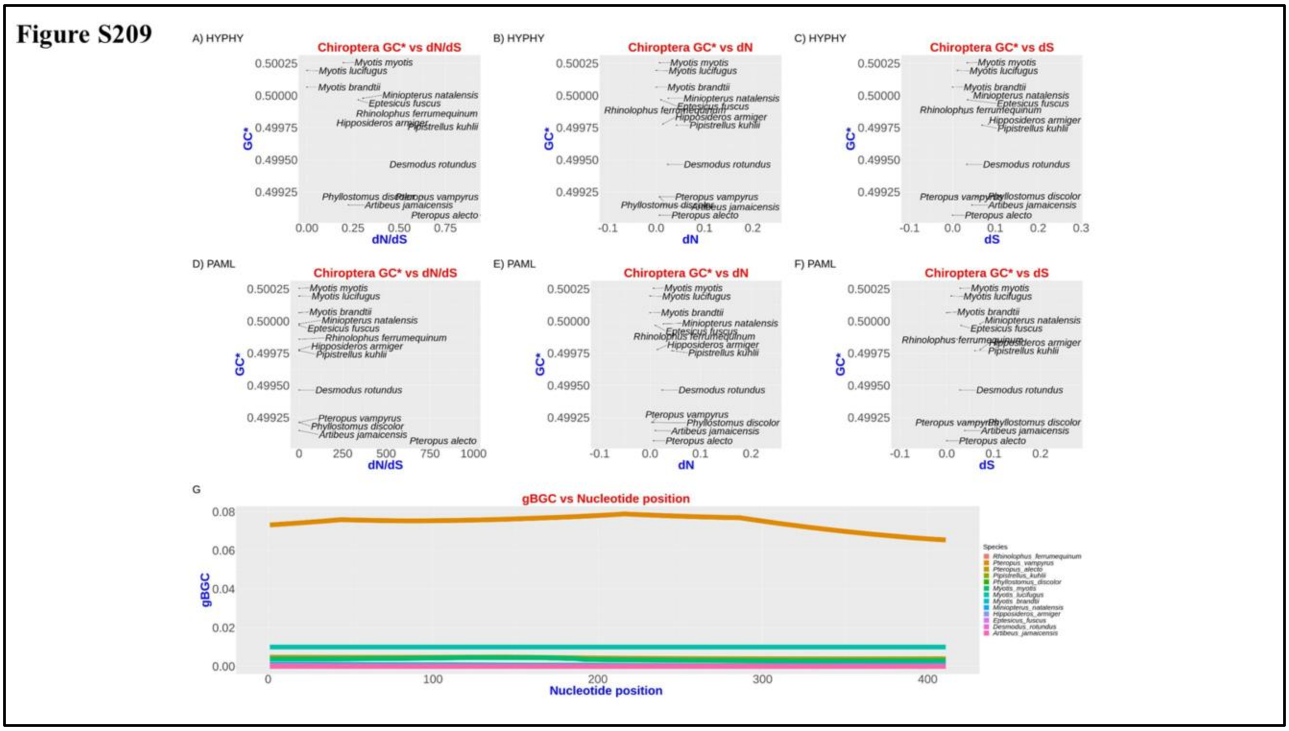

Figure S209: Values of GC* in Chiroptera are plotted with dN and dS which is calculated using mapNH. dN and dS were calculated using PAML and HyPhy both. gBGC across nucleotide position were calculated using PhastBias.

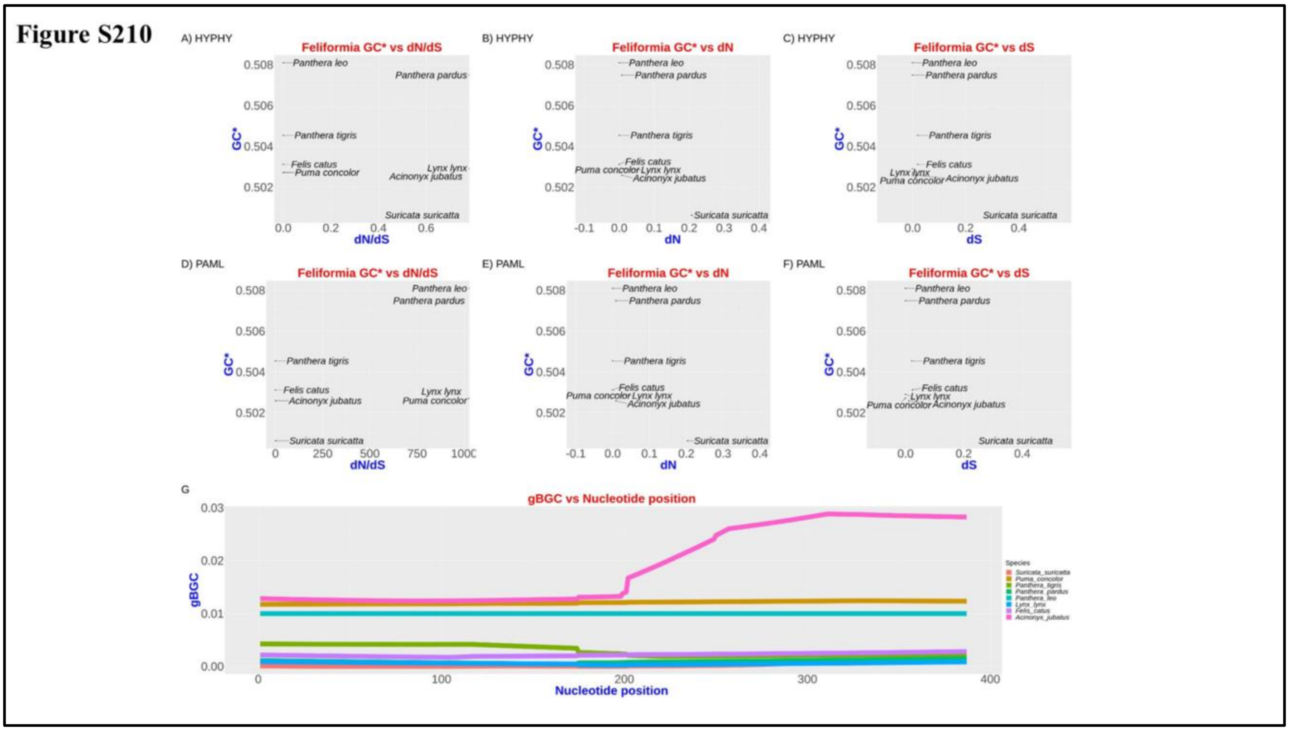

Figure S210: Values of GC* in Feliformia are plotted with dN and dS which is calculated using mapNH. dN and dS were calculated using PAML and HyPhy both. gBGC across nucleotide position were calculated using PhastBias.

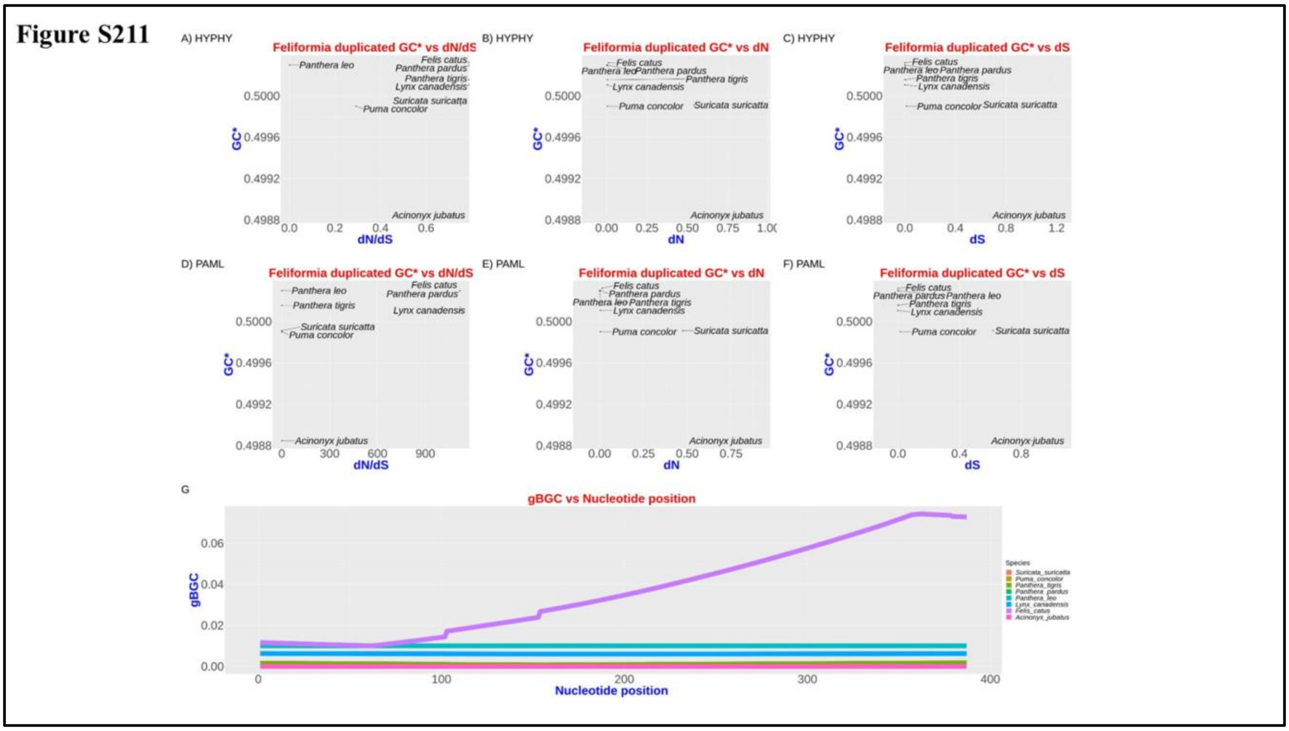

Figure S211: Values of GC* in Feliformia duplicated *COA1* are plotted with dN and dS which is calculated using mapNH. dN and dS were calculated using PAML and HyPhy both. gBGC across nucleotide position were calculated using PhastBias.

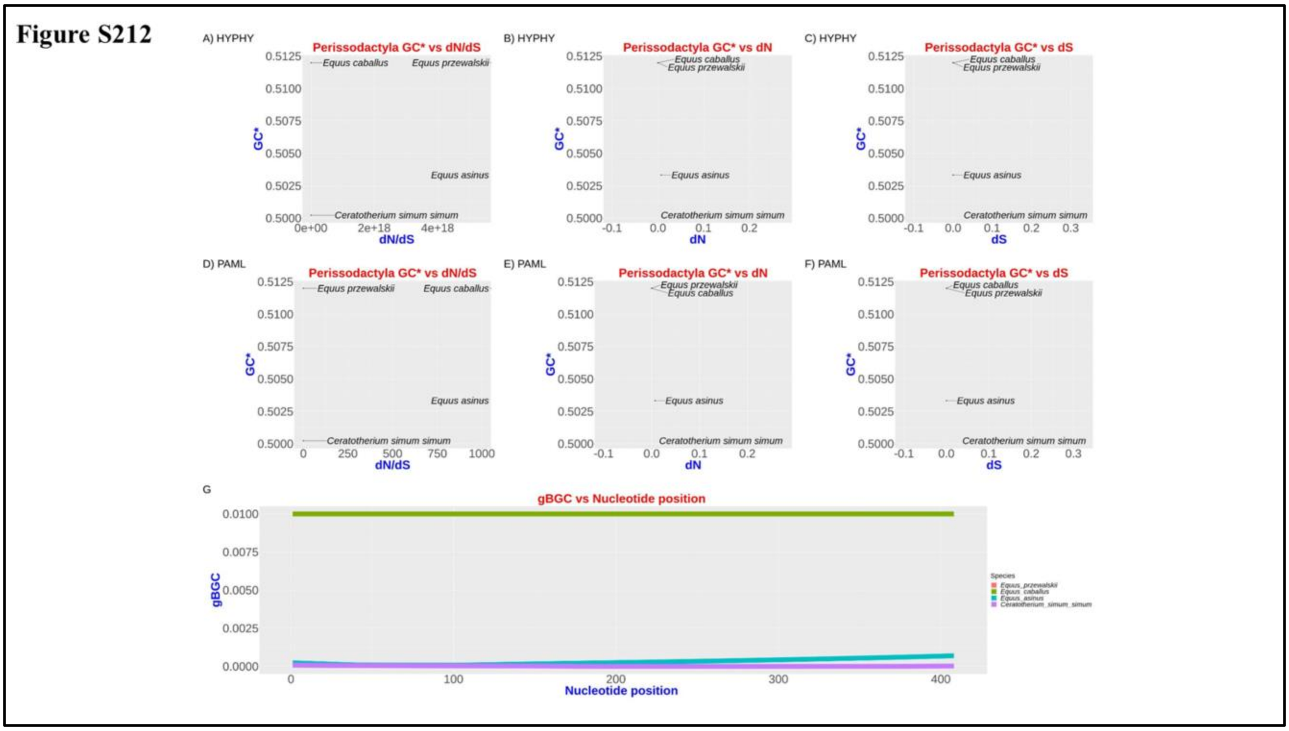

Figure S212: Values of GC* in Perissodactyla are plotted with dN and dS which is calculated using mapNH. dN and dS were calculated using PAML and HyPhy both. gBGC across nucleotide position were calculated using PhastBias.

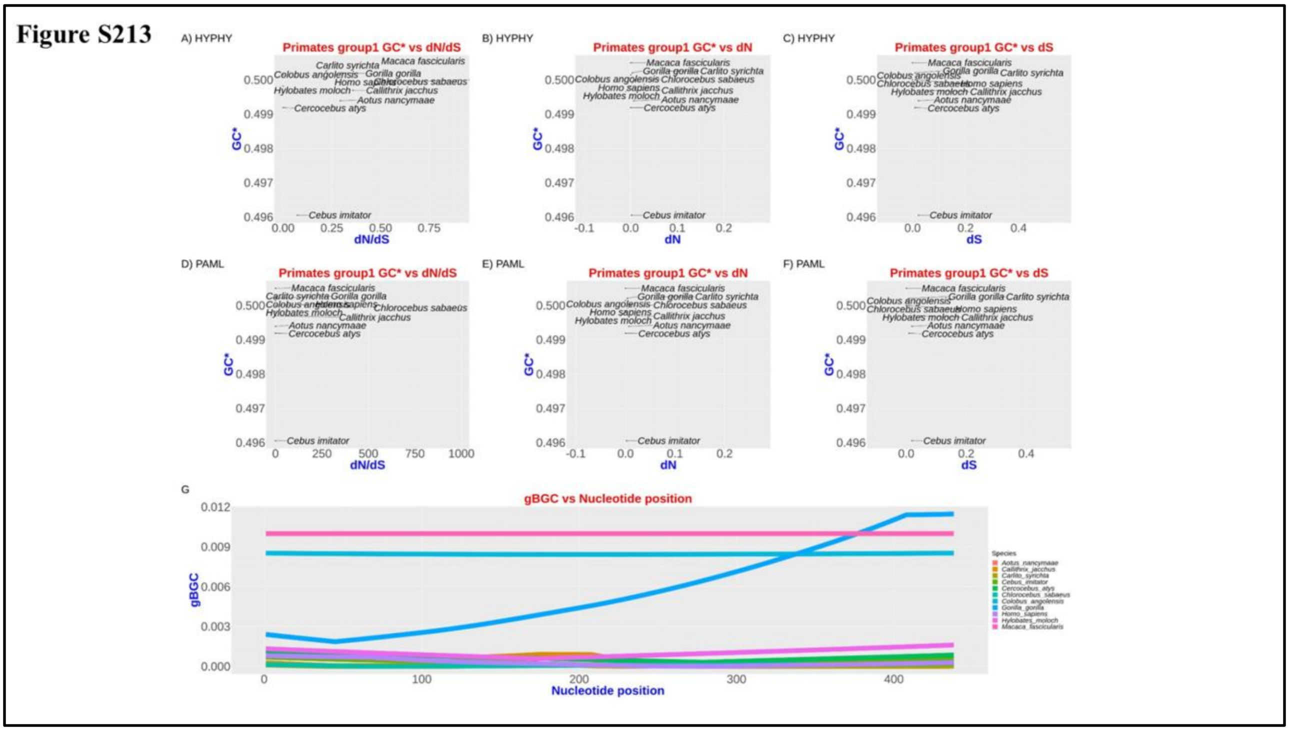

Figure S213: Values of GC* in Primates *COA1* are plotted with dN and dS which is calculated using mapNH. dN and dS were calculated using PAML and HyPhy both. gBGC across nucleotide position were calculated using PhastBias.

Figure S214

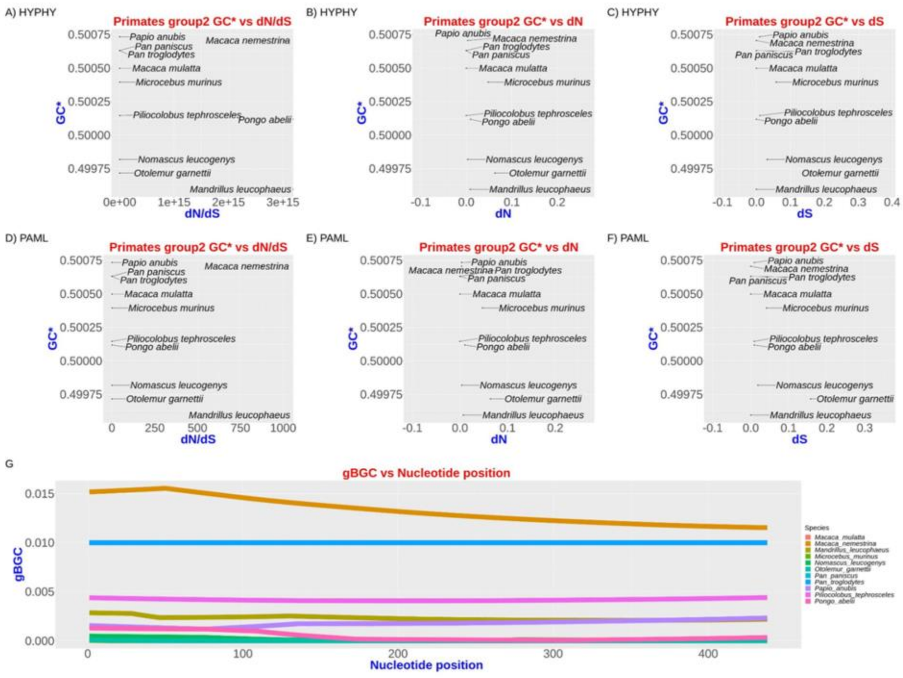

Figure S214: Values of GC* in Primates *COA1* are plotted with dN and dS which is calculated using mapNH. dN and dS were calculated using PAML and HyPhy both. gBGC across nucleotide position were calculated using PhastBias.

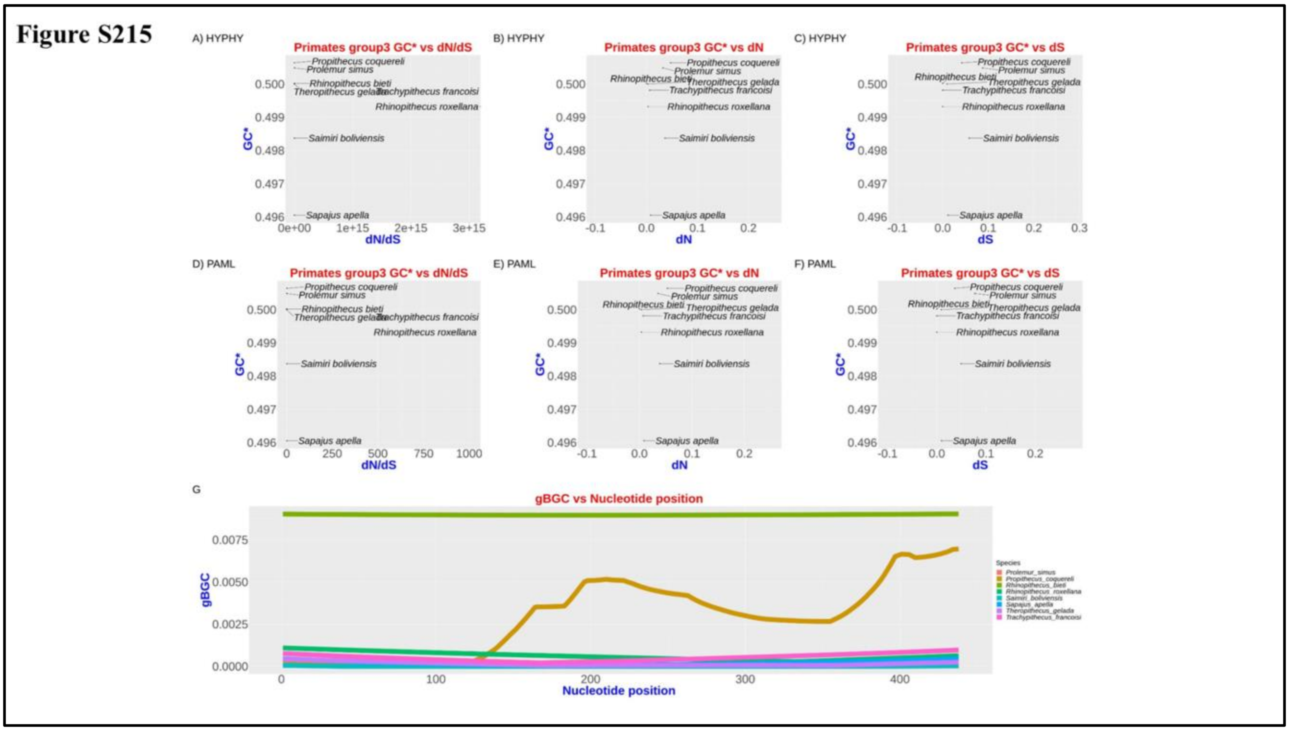

Figure S215: Values of GC* in Primates *COA1* are plotted with dN and dS which is calculated using mapNH. dN and dS were calculated using PAML and HyPhy both. gBGC across nucleotide position were calculated using PhastBias.

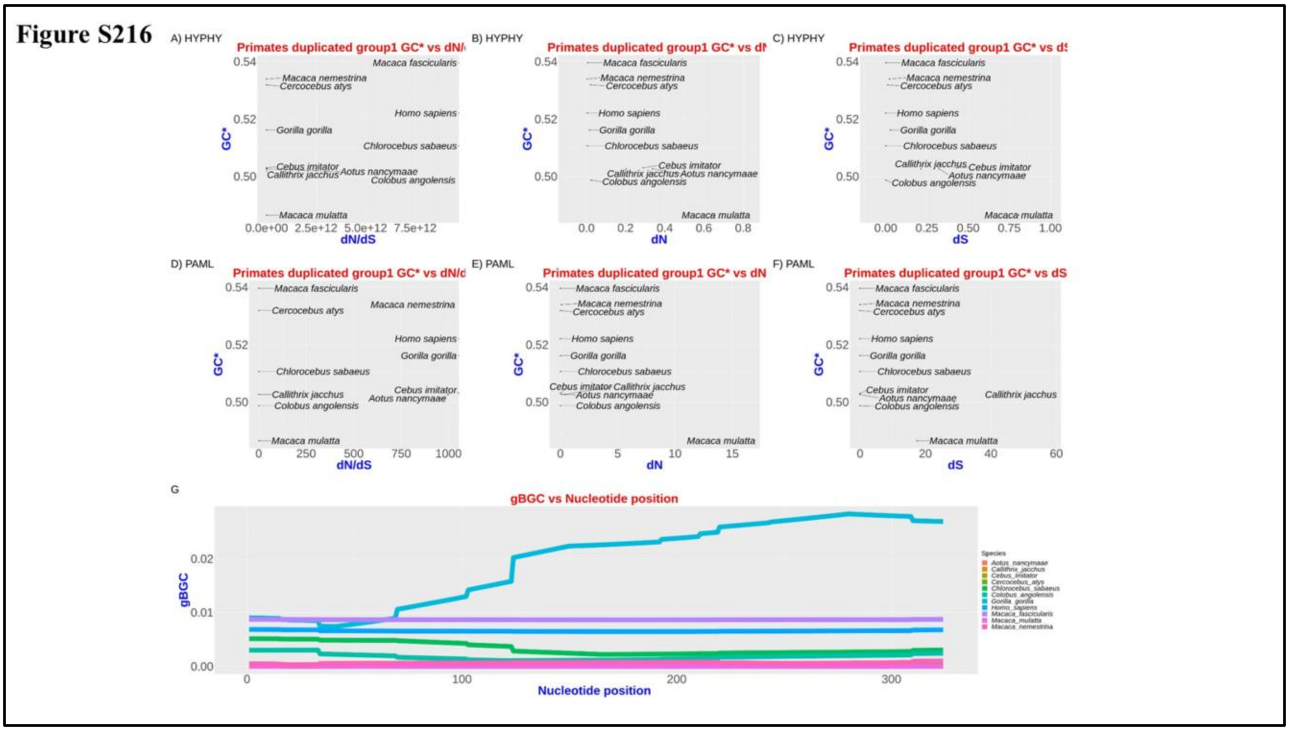

Figure S216: Values of GC* in Primates duplicated *COA1* are plotted with dN and dS which is calculated using mapNH. dN and dS were calculated using PAML and HyPhy both. gBGC across nucleotide position were calculated using PhastBias.

Figure S217

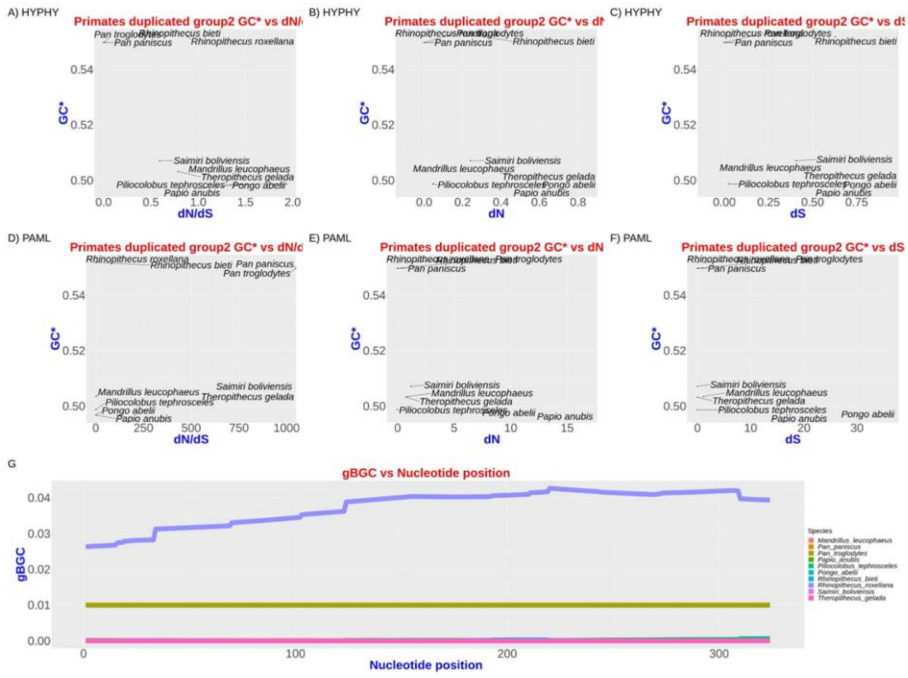

Figure S217: Values of GC* in Primates duplicated *COA1* are plotted with dN and dS which is calculated using mapNH. dN and dS were calculated using PAML and HyPhy both. gBGC across nucleotide position were calculated using PhastBias.

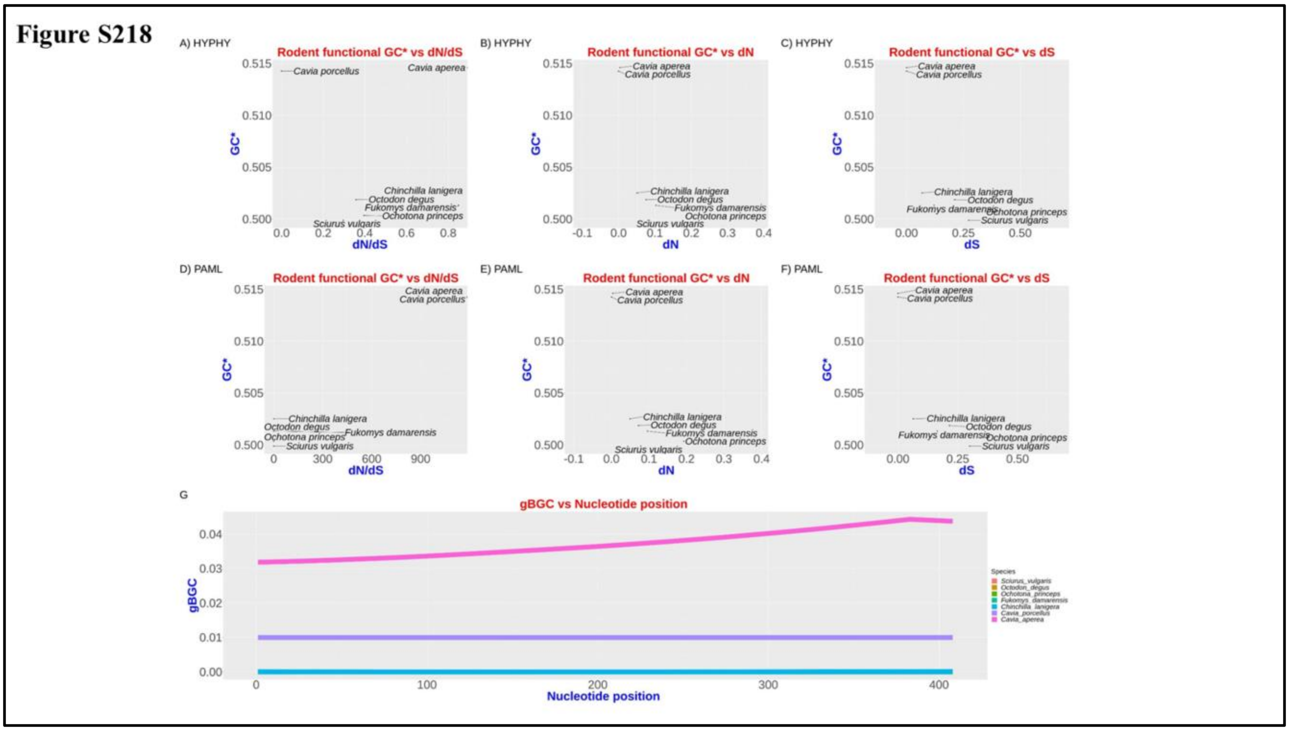

Figure S218: Values of GC* in rodents *COA1* are plotted with dN and dS which is calculated using mapNH. dN and dS were calculated using PAML and HyPhy both. gBGC across nucleotide position were calculated using PhastBias.

Figure S219

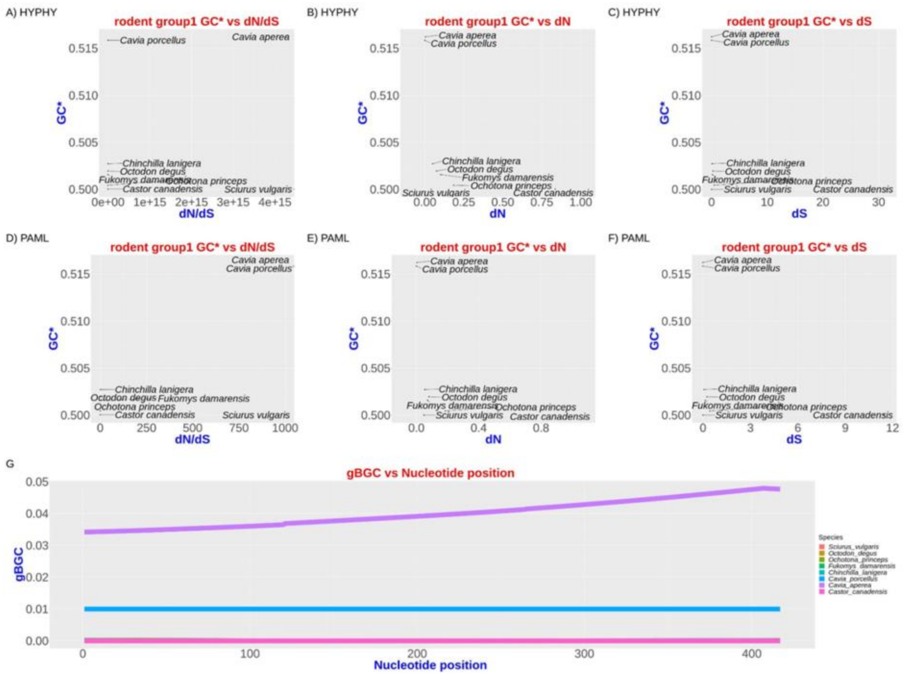

Figure S219: Values of GC* in rodents unsaturated group are plotted with dN and dS which is calculated using mapNH. dN and dS were calculated using PAML and HyPhy both. gBGC across nucleotide position were calculated using PhastBias.

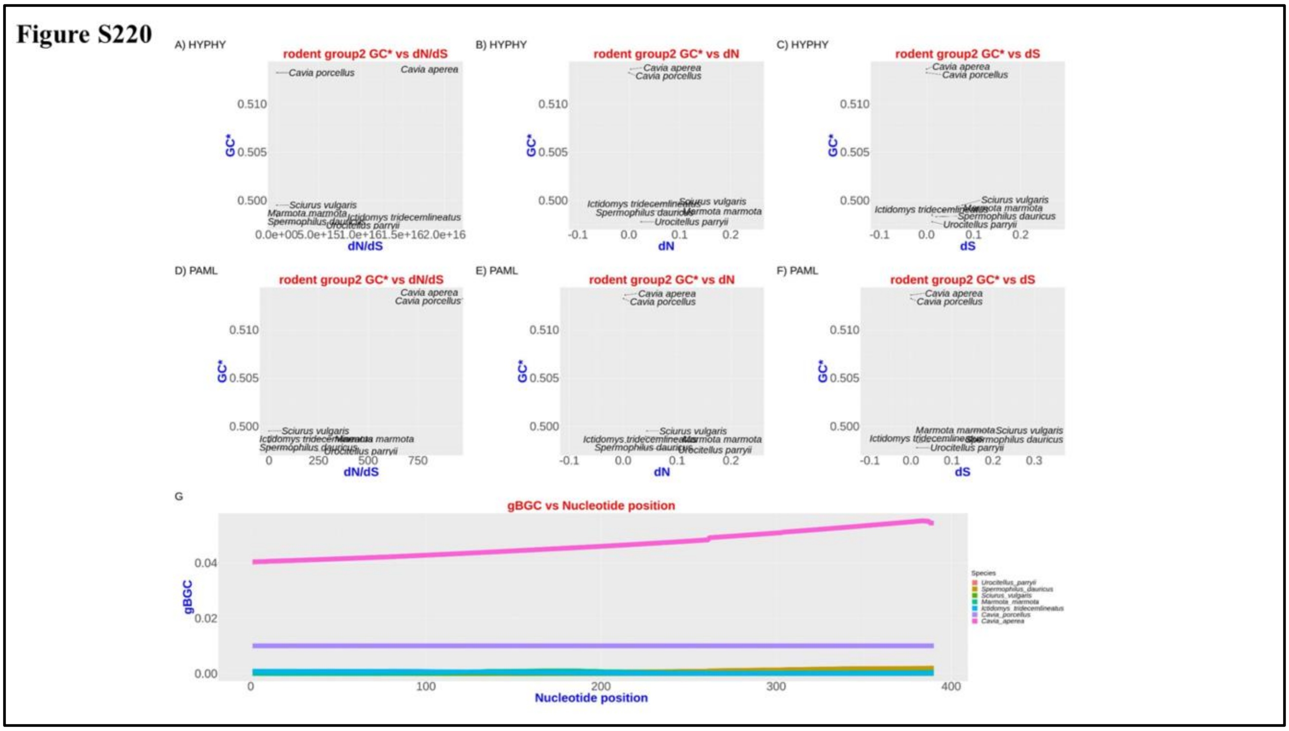

Figure S220: Values of GC* in rodents unsaturated group are plotted with dN and dS which is calculated using mapNH. dN and dS were calculated using PAML and HyPhy both. gBGC across nucleotide position were calculated using PhastBias.

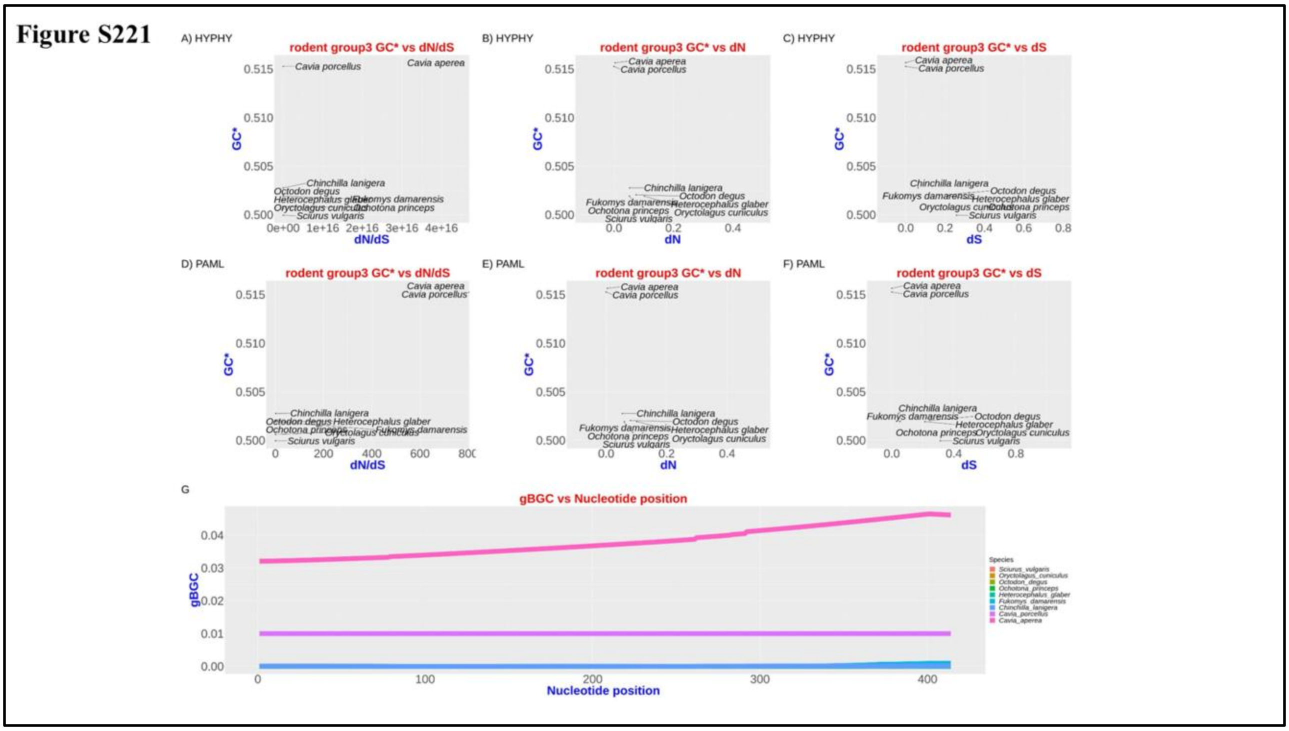

Figure S221: Values of GC* in rodents unsaturated group are plotted with dN and dS which is calculated using mapNH. dN and dS were calculated using PAML and HyPhy both. gBGC across nucleotide position were calculated using PhastBias.

Figure S222

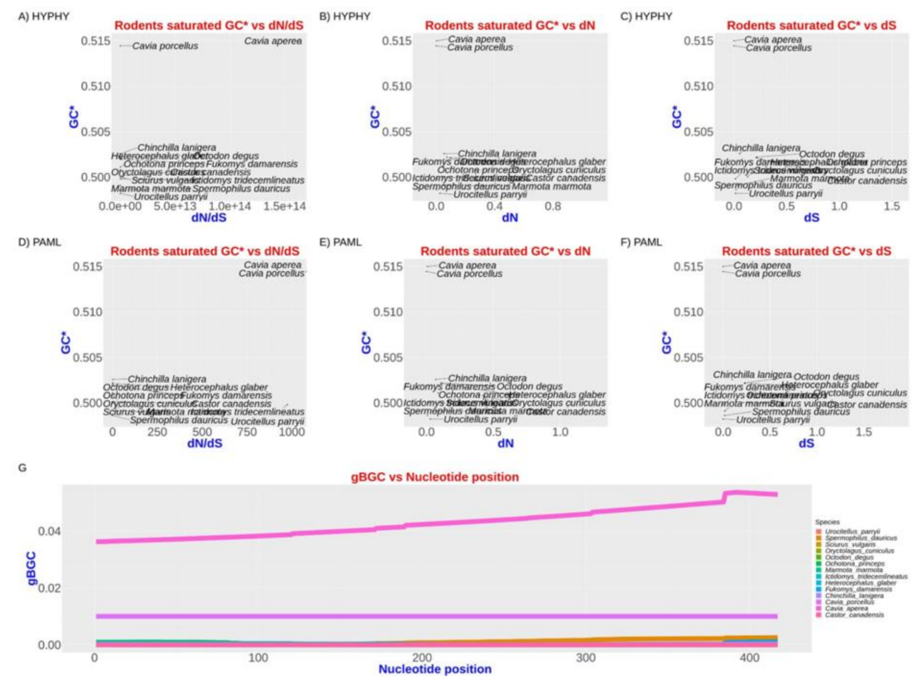

Figure S222: Values of GC* in rodents saturated group are plotted with dN and dS which is calculated using mapNH. dN and dS were calculated using PAML and HyPhy both. gBGC across nucleotide position were calculated using PhastBias.

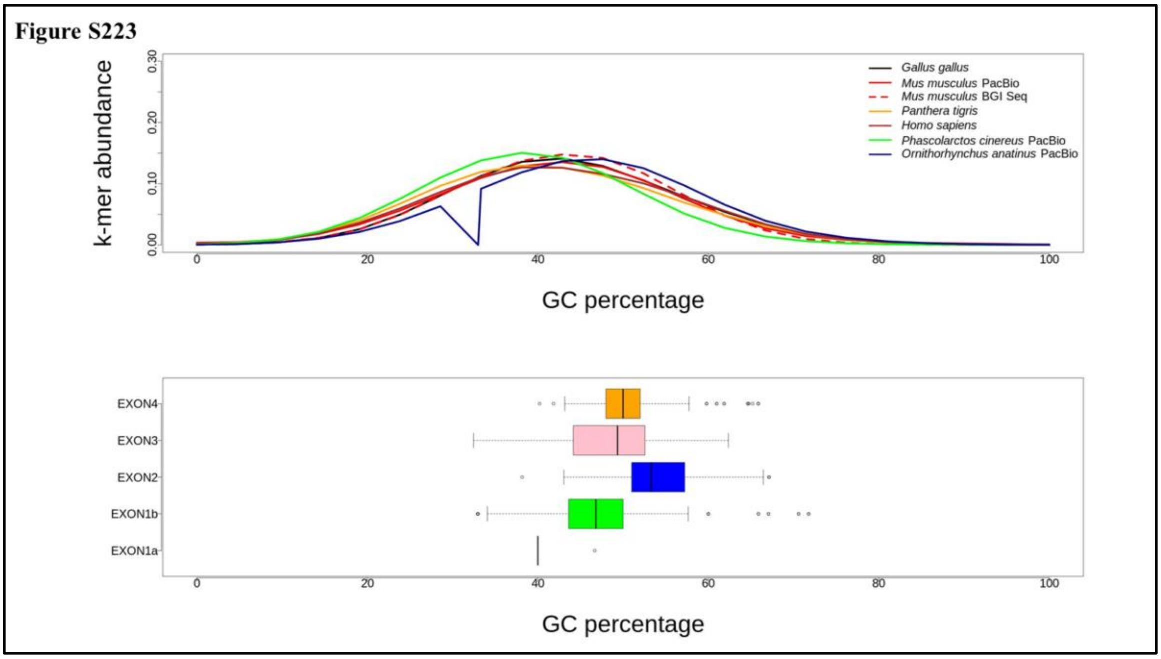

Figure S223: GC% versus K-mer plot using different data platforms like Illumina and PacBio, Each colour defines the species. Box plot of GC% of exon-1a, exon-1b, exon-2, exon-3, and exon-4 in the various group as one.

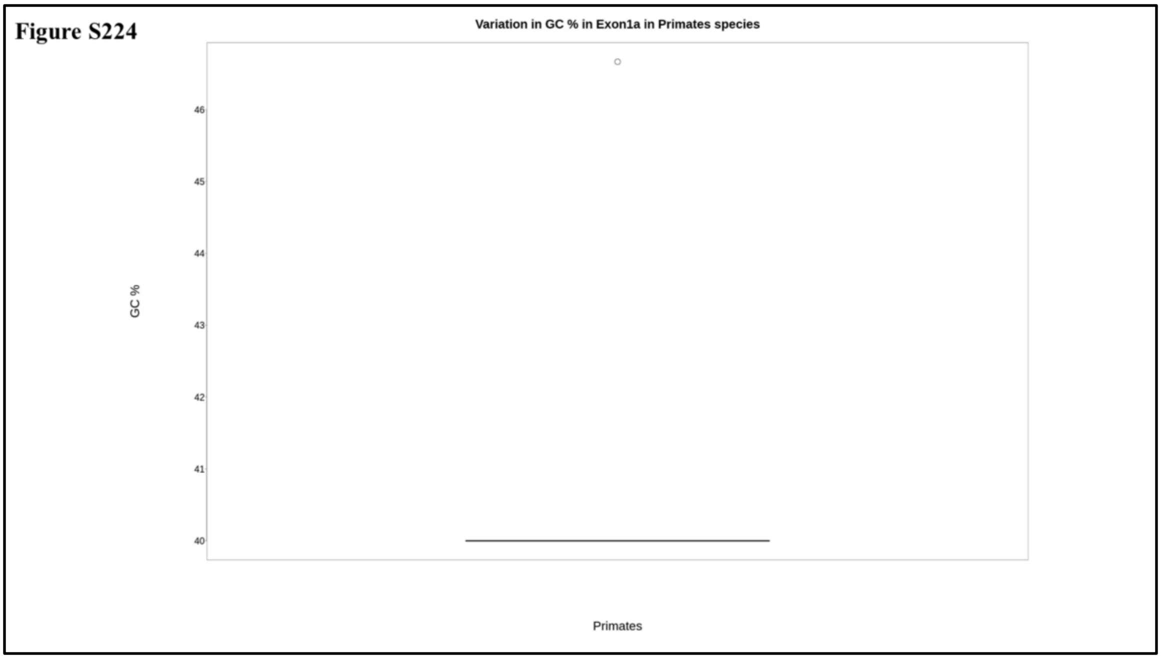

Figure S224: Box plot of GC% of exon-1a for primates group along with pairwise Wilcox test.

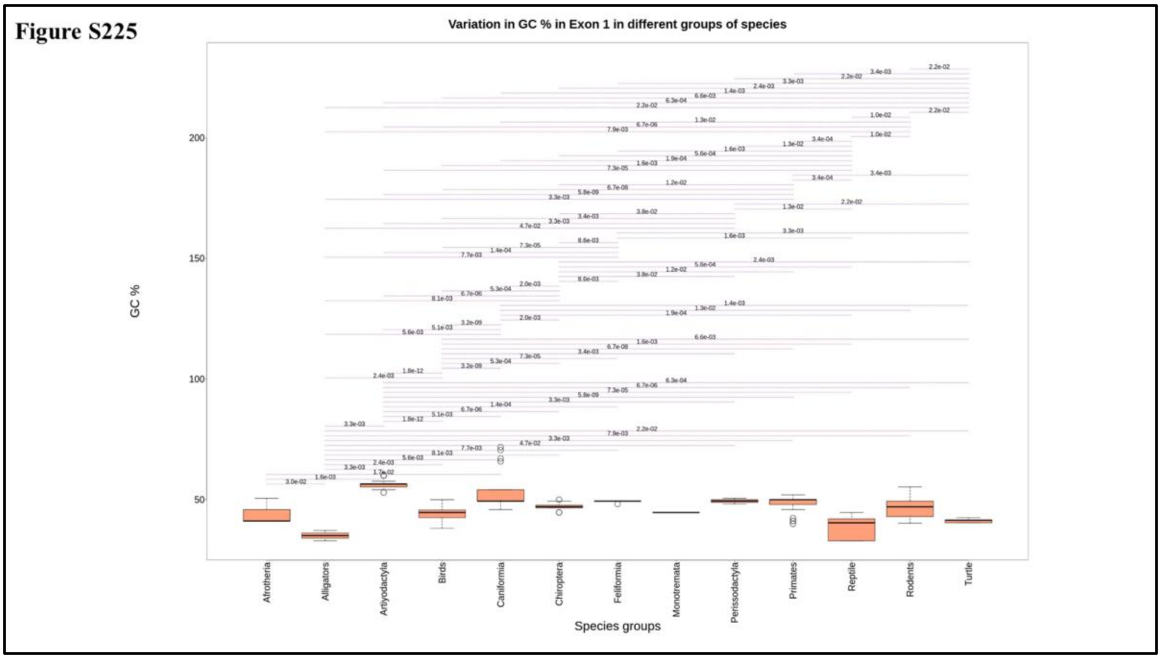

Figure S225: Box plot of GC% of exon-1 for various groups along with pairwise Wilcox test.

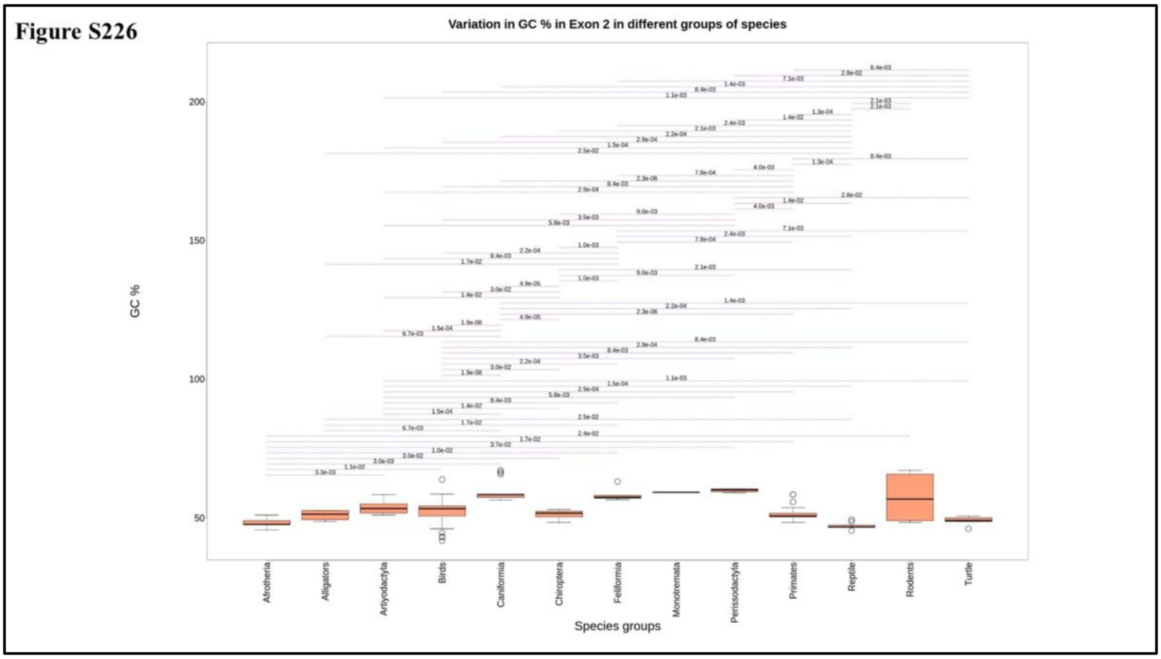

Figure S226: Box plot of GC% of exon-2 for various groups along with pairwise Wilcox test.

Figure S227

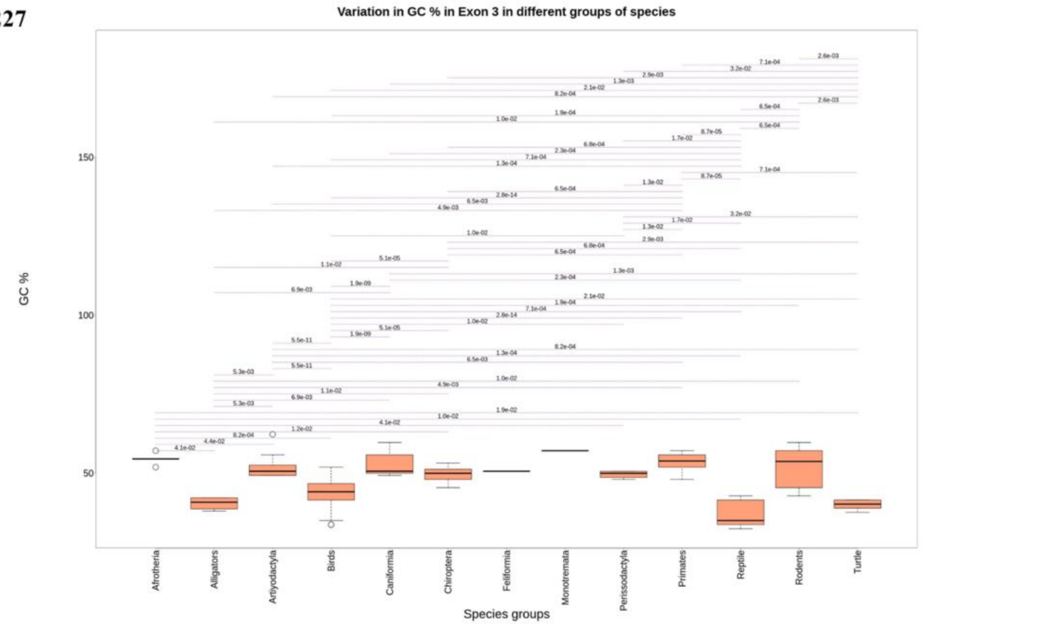

Figure S227: Box plot of GC% of exon-3 for various groups along with pairwise Wilcox test.

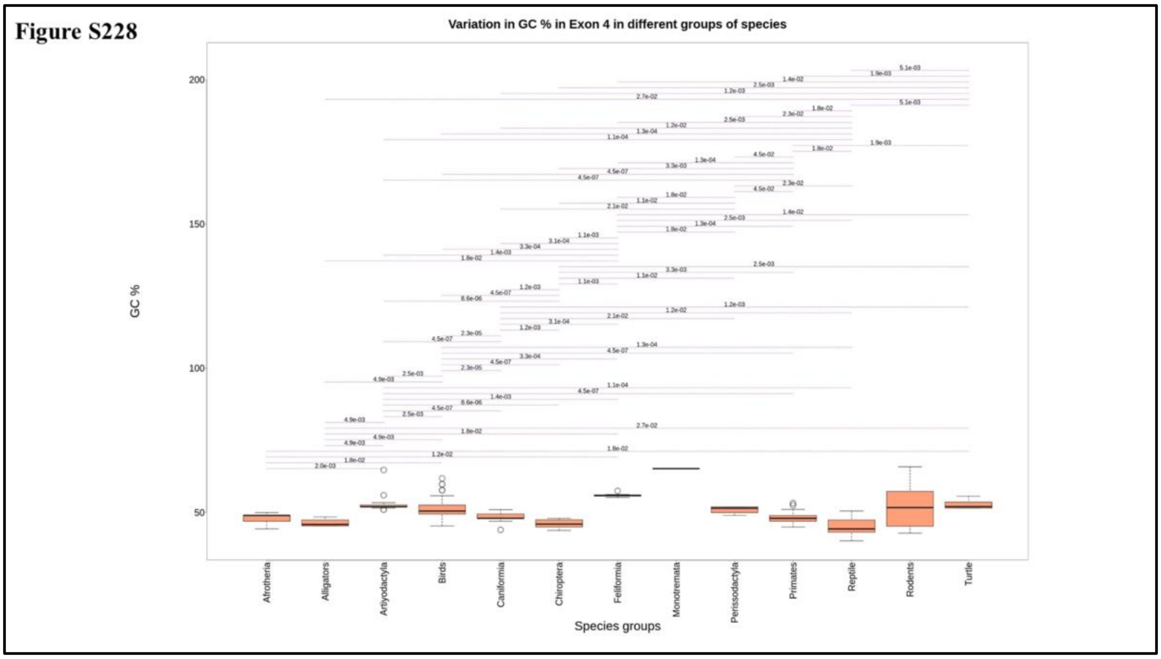

Figure S228: Box plot of GC% of exon-4 for various groups along with pairwise Wilcox test.

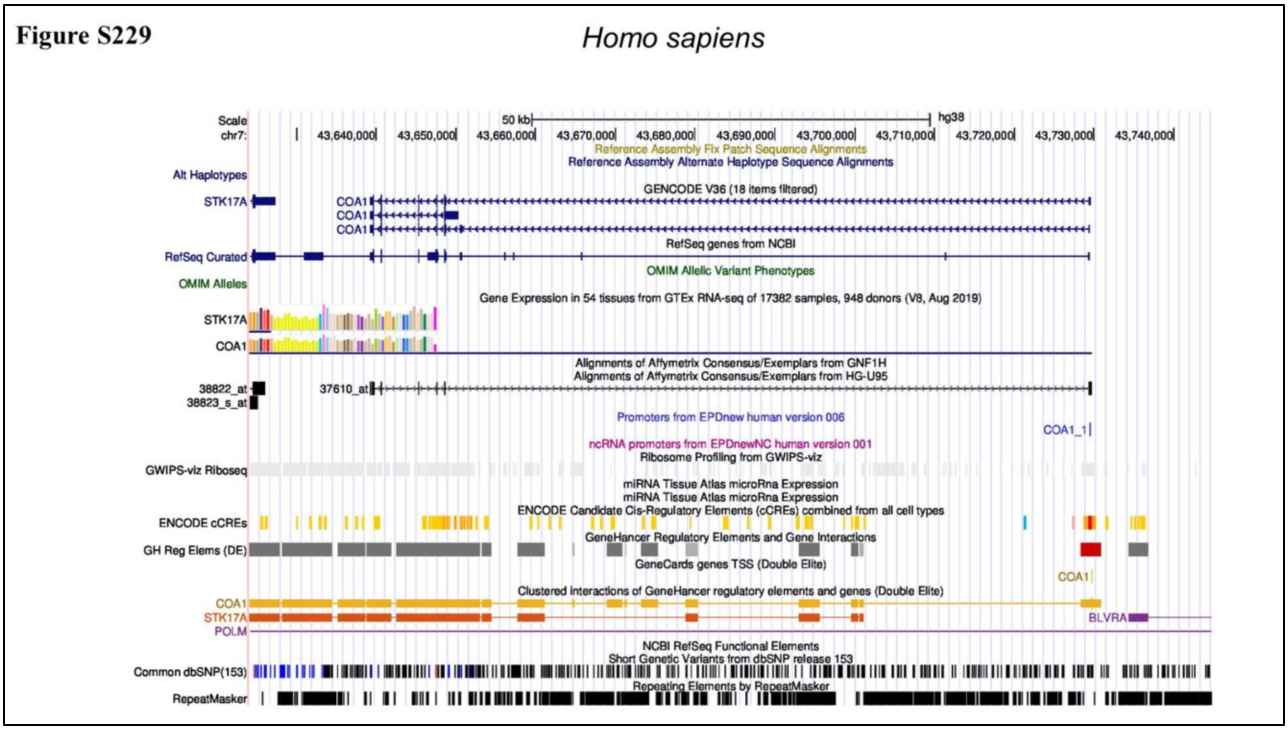

Figure S229: Screenshot of UCSC genome browser of the *COA1* in human (*Homo sapiens*).

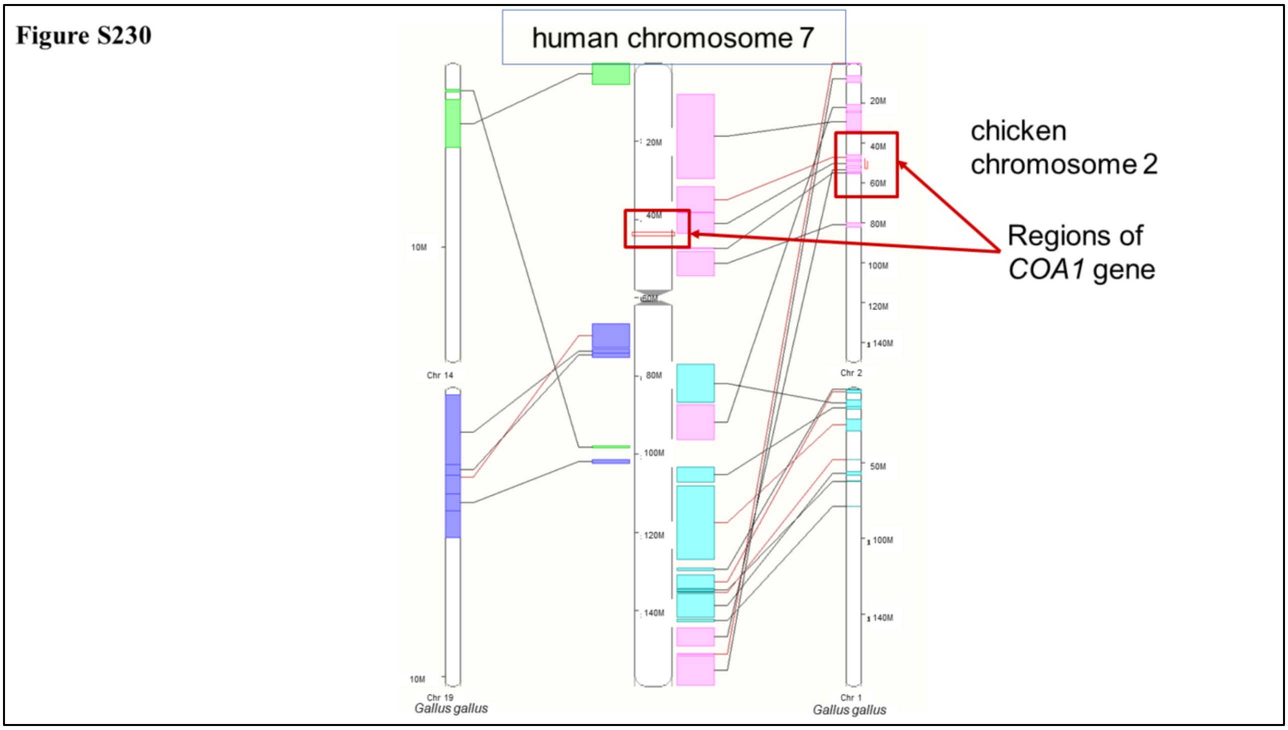

Figure S230: Conserved synteny in the *COA1* region of human chromosome 7 and chicken chromosome 2. Each of the synteny blocks is depicted by different colors and connected by lines that go from the human chromosome to chicken chromosomes. Exact location of the *COA1* gene is depicted by a red box within the chromosome representations.

Figure S231

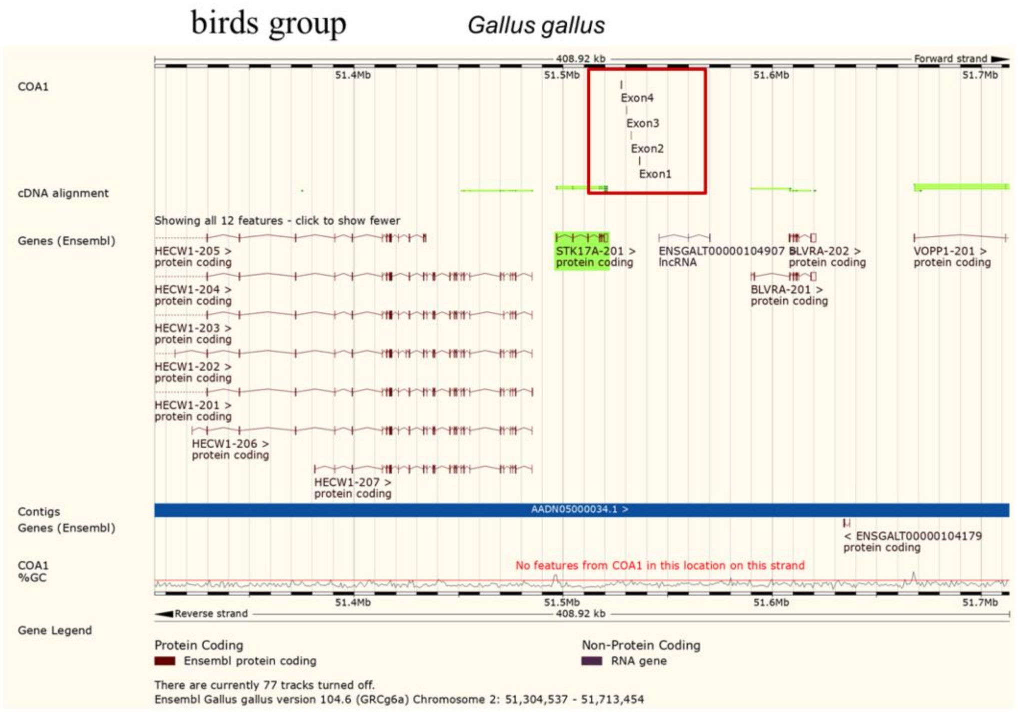

Figure S231: Gene order near *COA1* gene in the chicken (*Gallus gallus*) genome. The rectangular boxes connected by thin lines depict genes, with the boxes denoting exons and connecting lines being introns. Direction of transcription is denoted by the > or < symbols (signify positive and negative strand of transcription respectively) beside the gene names. chromosome bands and the genomic coordinates in kilobase (kb) are shown in the topmost row. Details of the contig and scaffold identity are provided in the blue bar located in the middle of the figure. Exact location of the *COA1* gene is depicted by a red box.

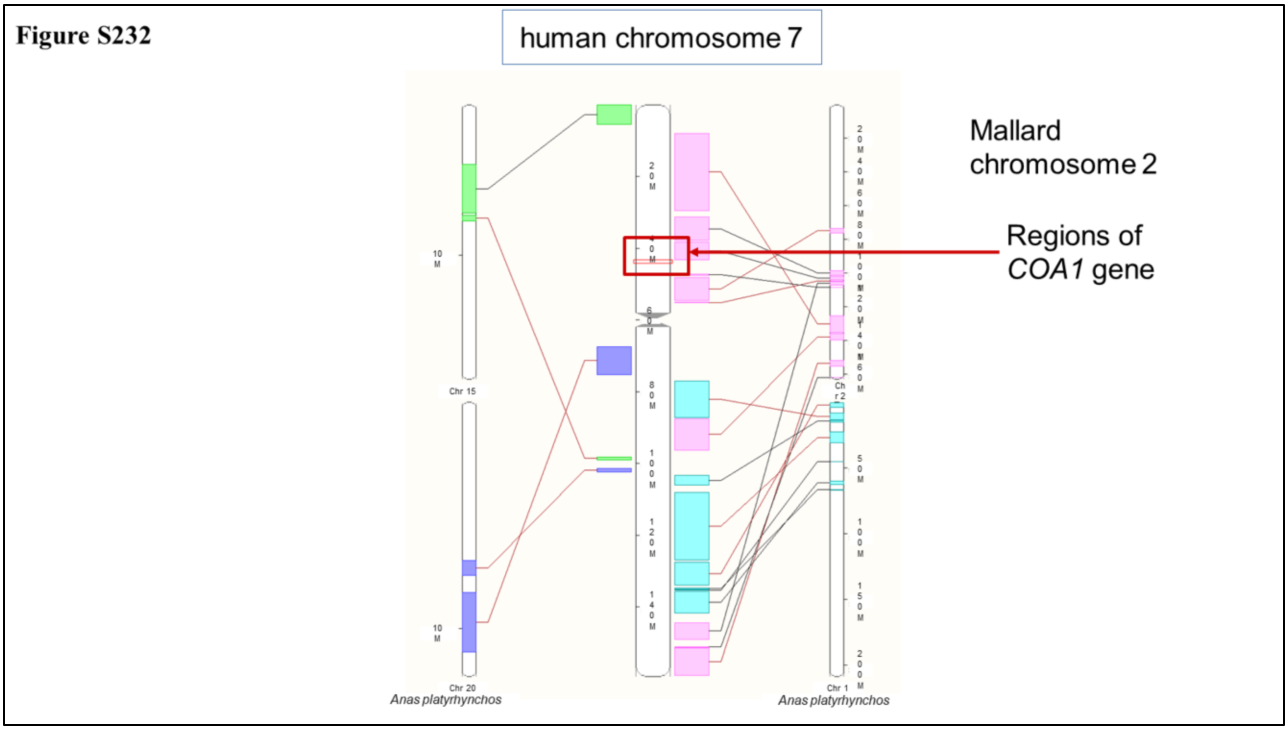

Figure S232: Conserved synteny in the *COA1* region of human chromosome 7 and mallard chromosome 2. Each of the synteny blocks is depicted by different colors and connected by lines that go from the human chromosome to mallard chromosomes. Exact location of the *COA1* gene is depicted by a red box within the chromosome representations.

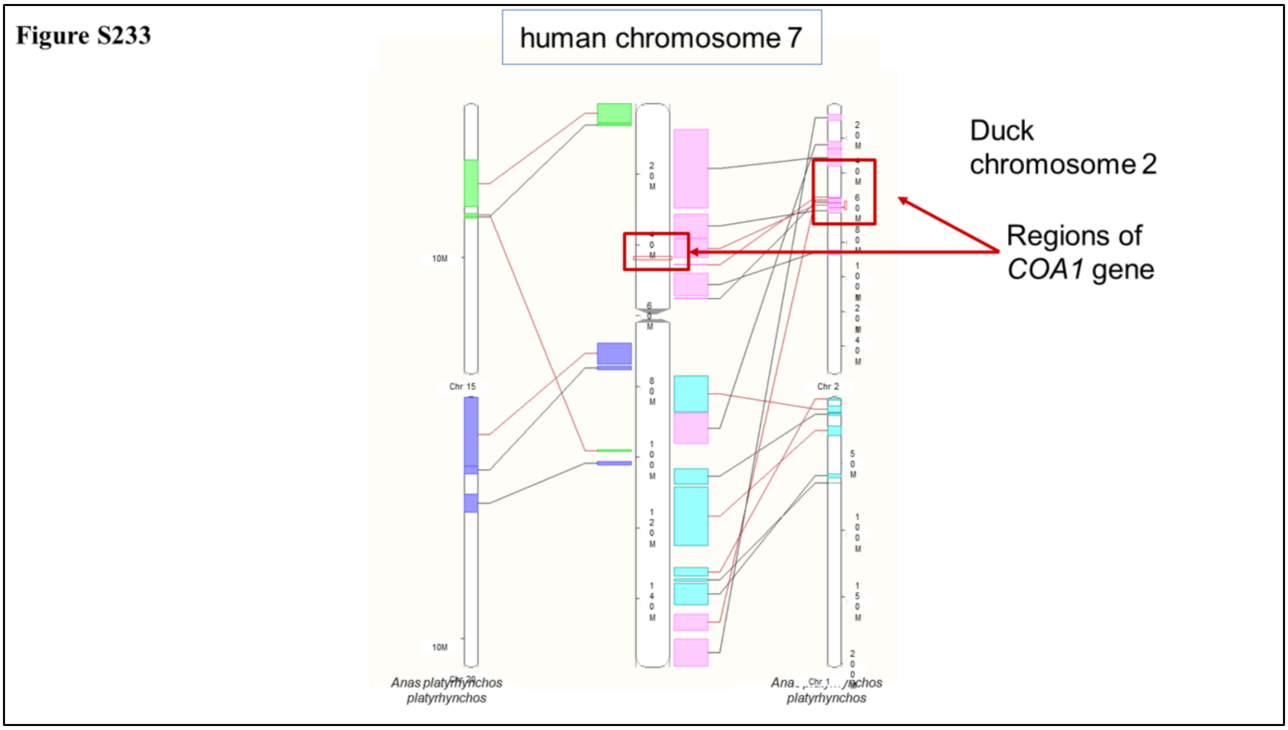

Figure S233: Conserved synteny in the *COA1* region of human chromosome 7 and duck chromosome 2. Each of the synteny blocks is depicted by different colors and connected by lines that go from the human chromosome to duck chromosomes. Exact location of the *COA1* gene is depicted by a red box within the chromosome representations.

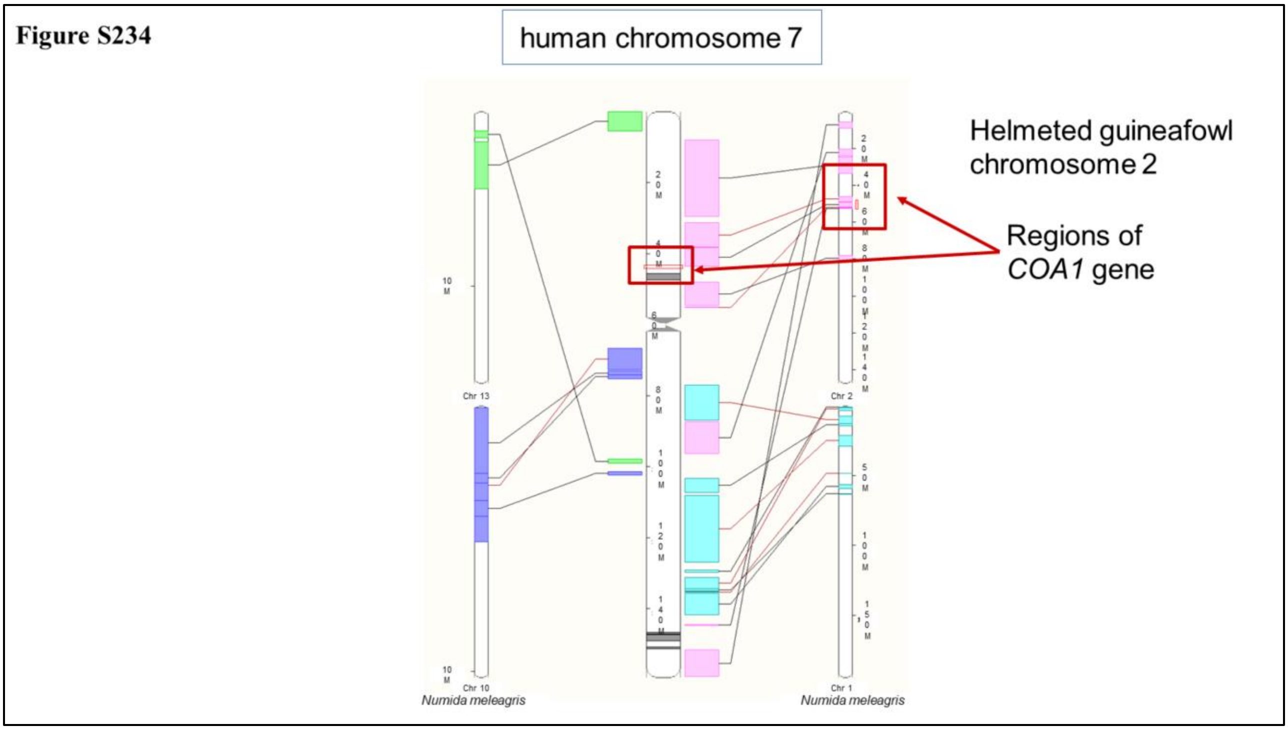

Figure S234: Conserved synteny in the *COA1* region of human chromosome 7 and helmeted guineafowl chromosome 2. Each of the synteny blocks is depicted by different colors and connected by lines that go from the human chromosome to helmeted guineafowl chromosomes. Exact location of the *COA1* gene is depicted by a red box within the chromosome representations.

Figure S235

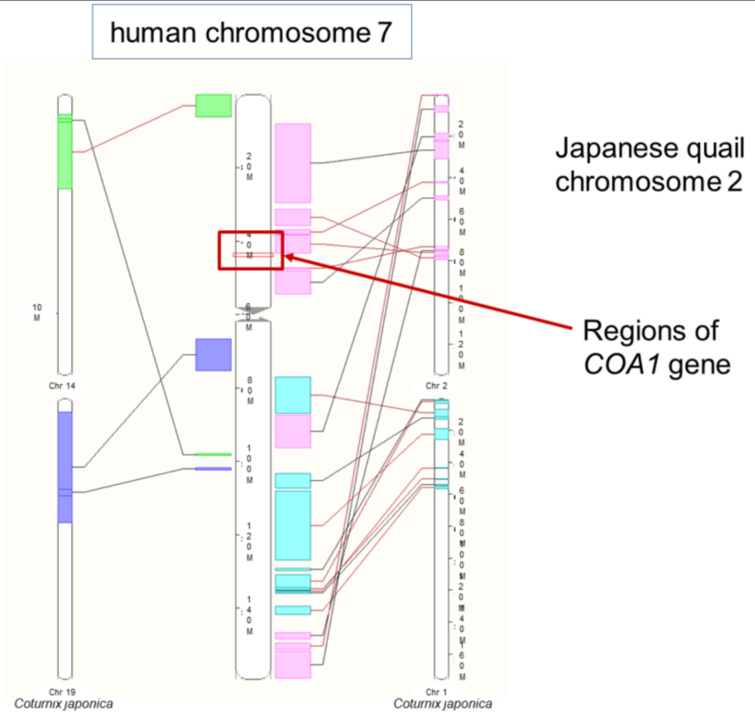

Figure S235: Conserved synteny in the *COA1* region of human chromosome 7 and Japanese quail chromosome 2. Each of the synteny blocks is depicted by different colors and connected by lines that go from the human chromosome to Japanese quail chromosomes. Exact location of the *COA1* gene is depicted by a red box within the chromosome representations.

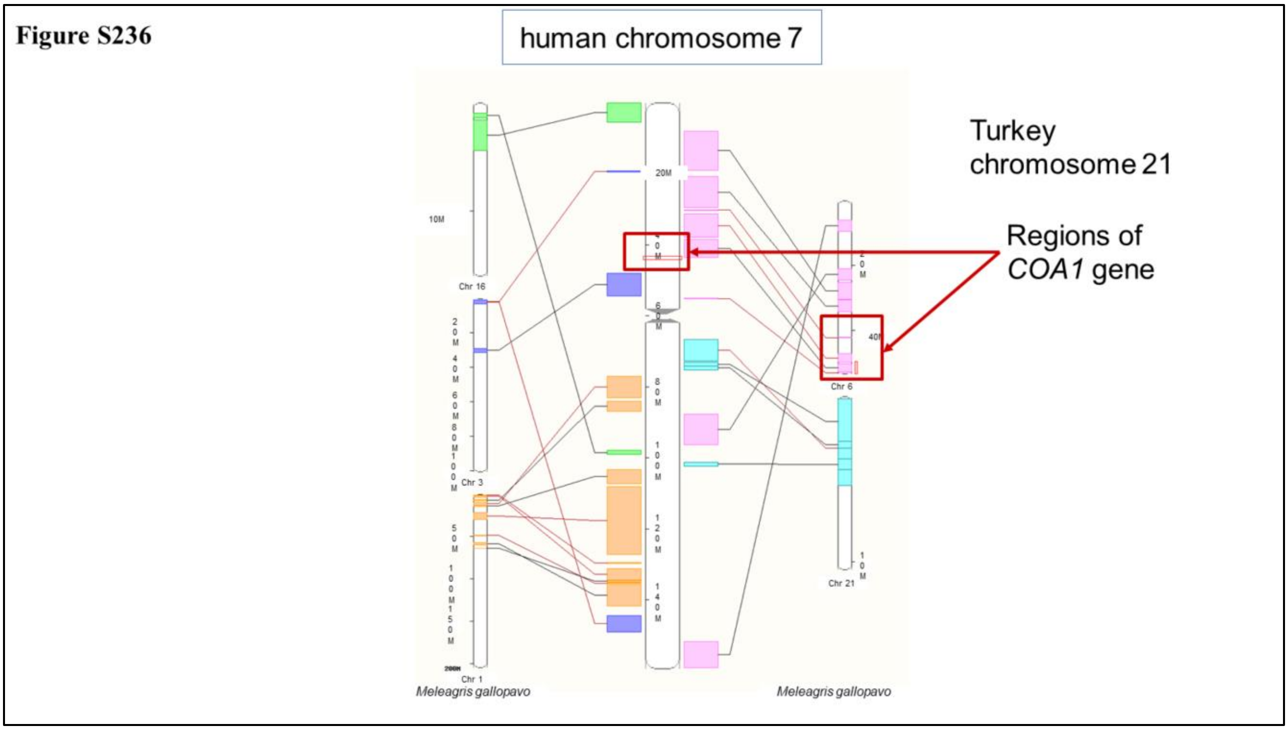

Figure S236: Conserved synteny in the *COA1* region of human chromosome 7 and turkey chromosome 21. Each of the synteny blocks is depicted by different colors and connected by lines that go from the human chromosome to turkey chromosomes. Exact location of the *COA1* gene is depicted by a red box within the chromosome representations.

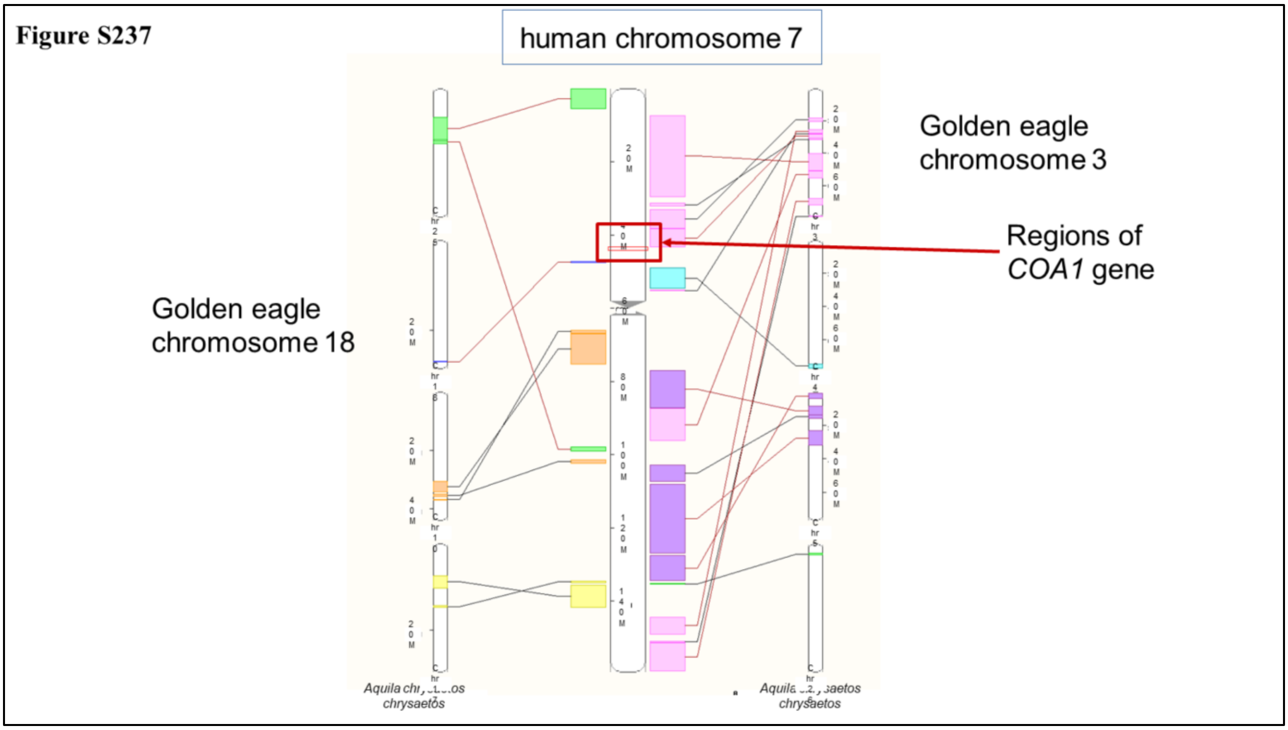

Figure S237: Conserved synteny in the *COA1* region of human chromosome 7 and golden eagle chromosome 3. Each of the synteny blocks is depicted by different colors and connected by lines that go from the human chromosome to golden eagle chromosomes. Exact location of the *COA1* gene is depicted by a red box within the chromosome representations.

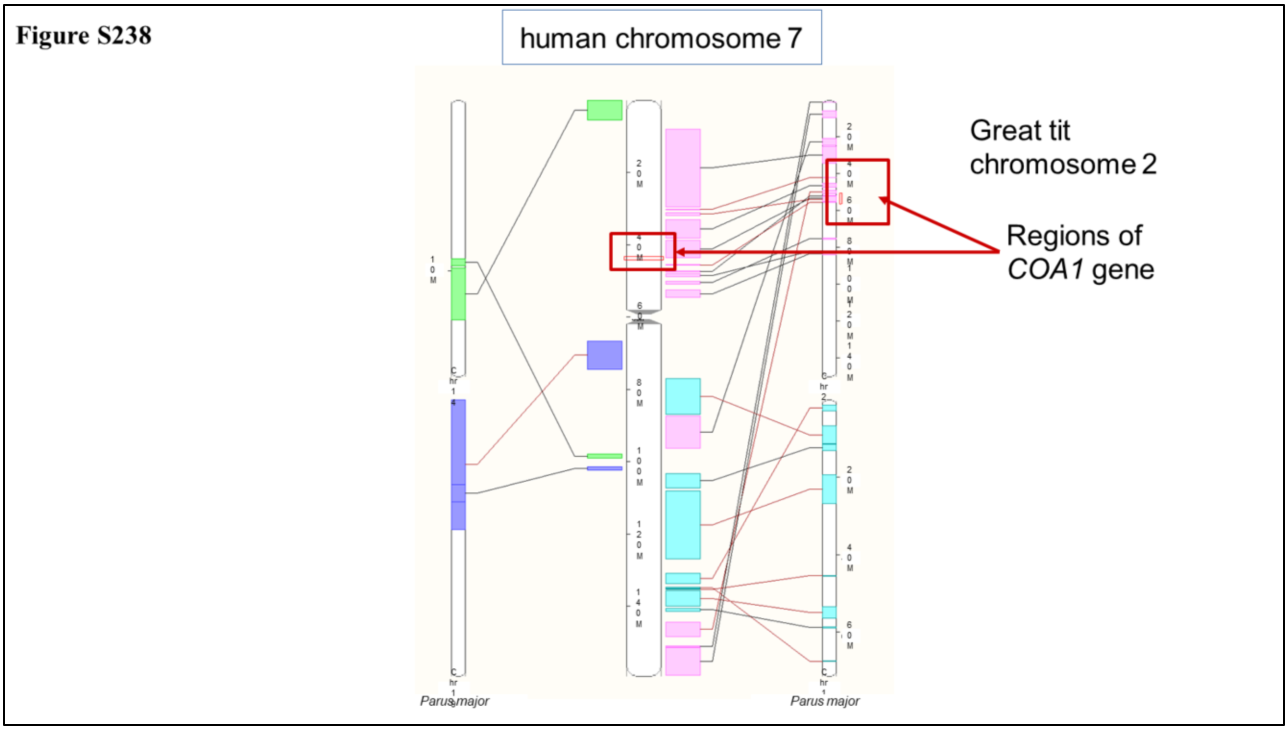

Figure S238: Conserved synteny in the *COA1* region of human chromosome 7 and great tit chromosome 2. Each of the synteny blocks is depicted by different colors and connected by lines that go from the human chromosome to great tit chromosomes. Exact location of the *COA1* gene is depicted by a red box within the chromosome representations.

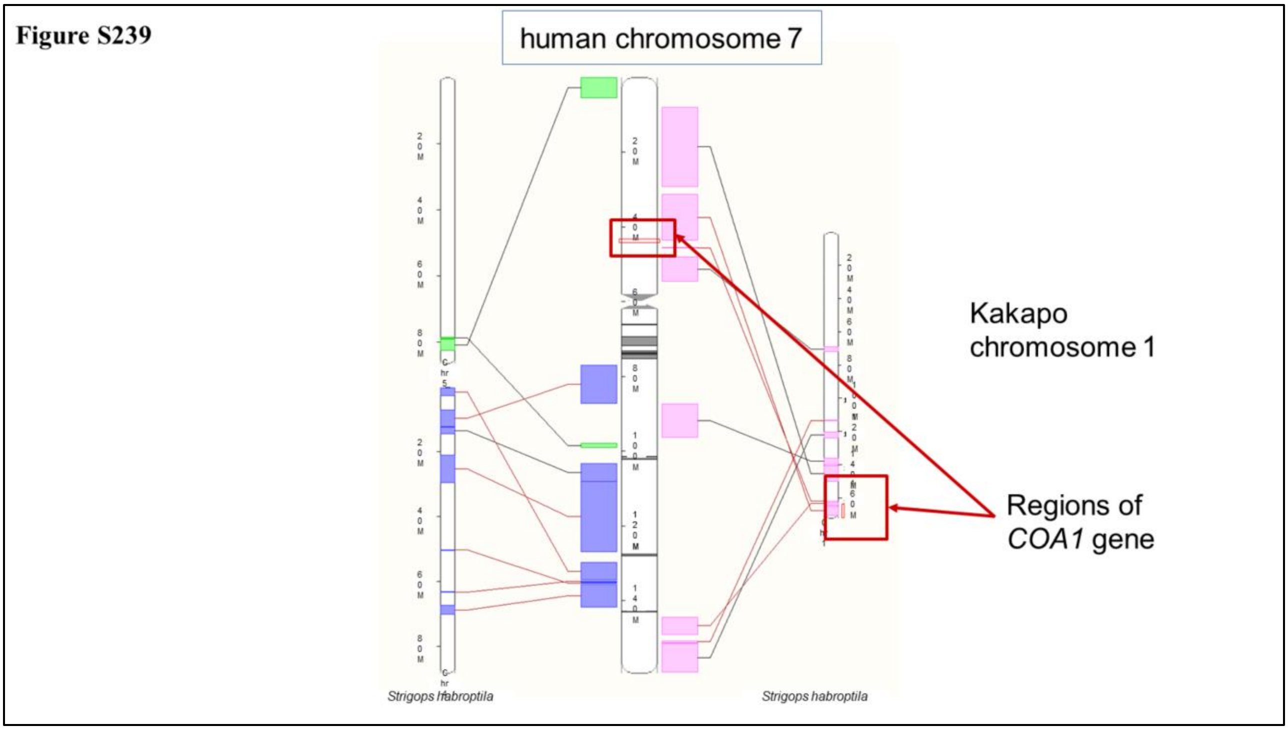

Figure S239: Conserved synteny in the *COA1* region of human chromosome 7 and kakapo chromosome 1. Each of the synteny blocks is depicted by different colors and connected by lines that go from the human chromosome to kakapo chromosomes. Exact location of the *COA1* gene is depicted by a red box within the chromosome representations.

Figure S240

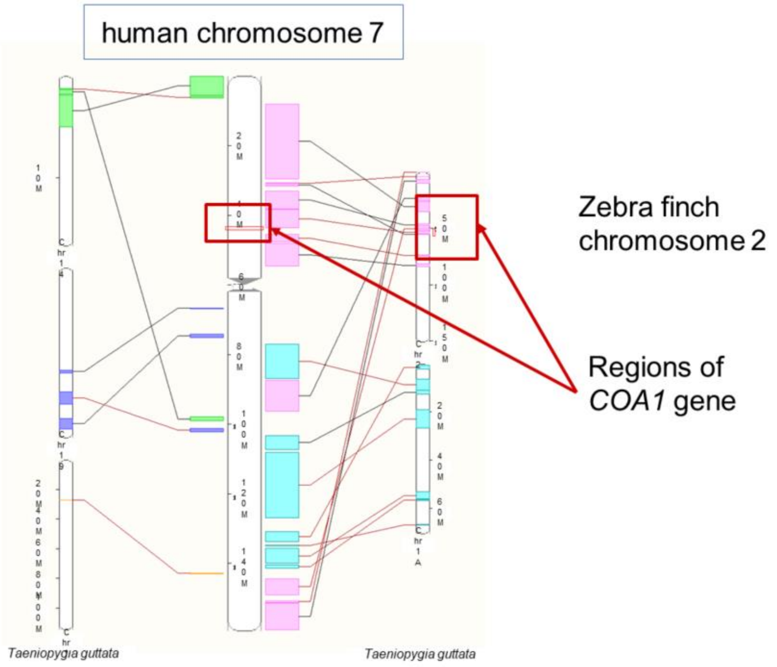

Figure S240 Conserved synteny in the *COA1* region of human chromosome 7 and zebra finch chromosome 2. Each of the synteny blocks is depicted by different colors and connected by lines that go from the human chromosome to zebra finch chromosomes. Exact location of the *COA1* gene is depicted by a red box within the chromosome representations.

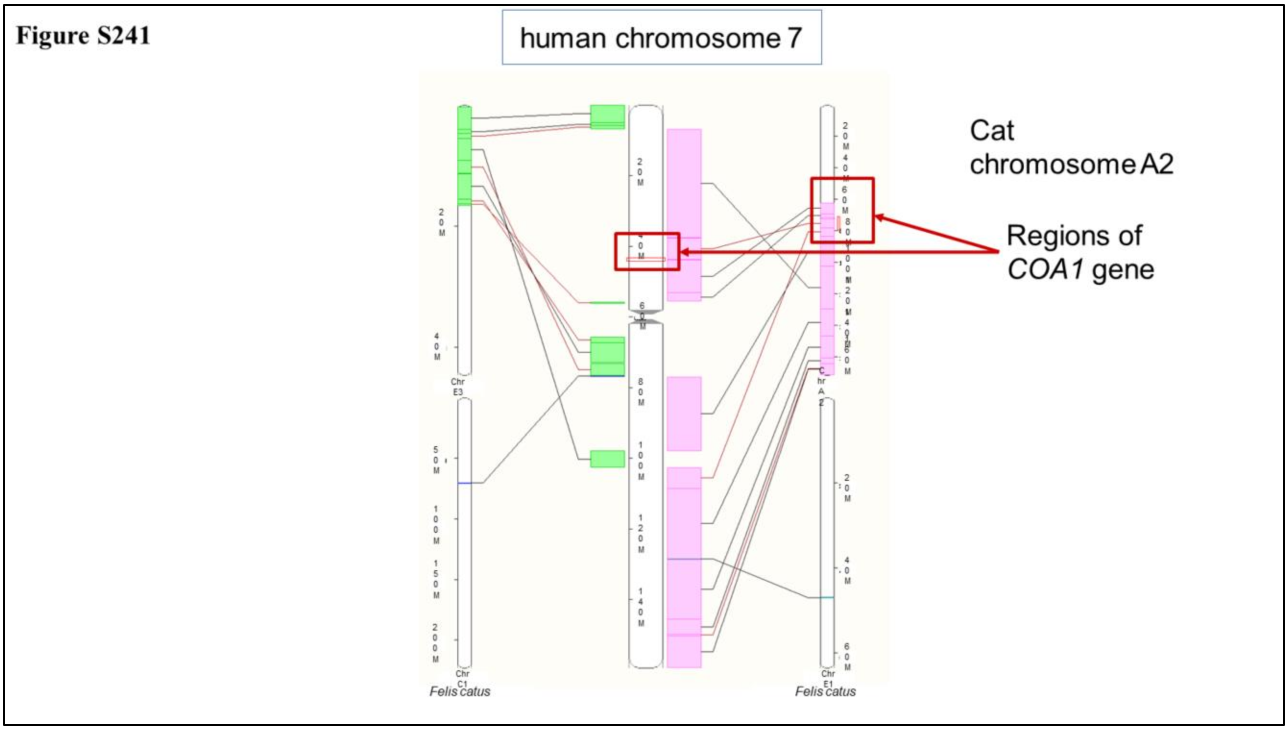

Figure S241: Conserved synteny in the *COA1* region of human chromosome 7 and cat chromosome A2. Each of the synteny blocks is depicted by different colors and connected by lines that go from the human chromosome to cat chromosomes. Exact location of the *COA1* gene is depicted by a red box within the chromosome representations.

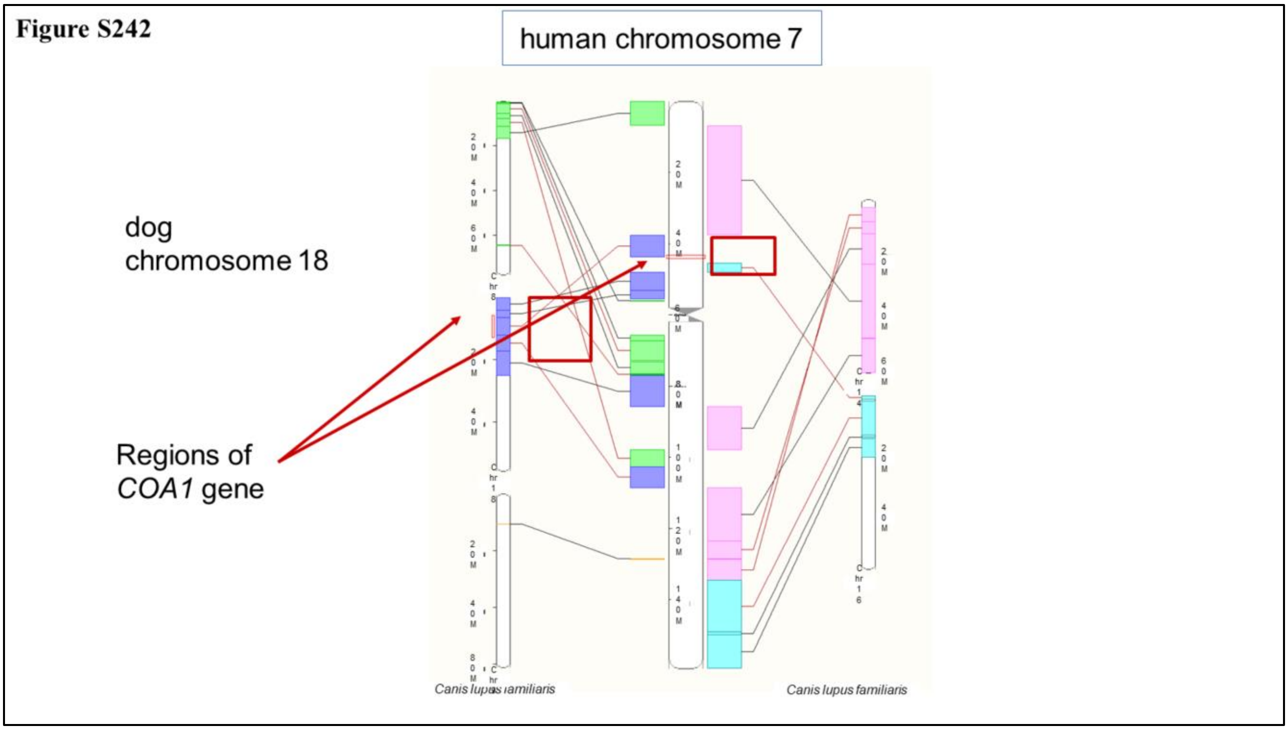

Figure S242: Conserved synteny in the *COA1* region of human chromosome 7 and dog chromosome 18. Each of the synteny blocks is depicted by different colors and connected by lines that go from the human chromosome to dog chromosomes. Exact location of the *COA1* gene is depicted by a red box within the chromosome representations.

Figure S243

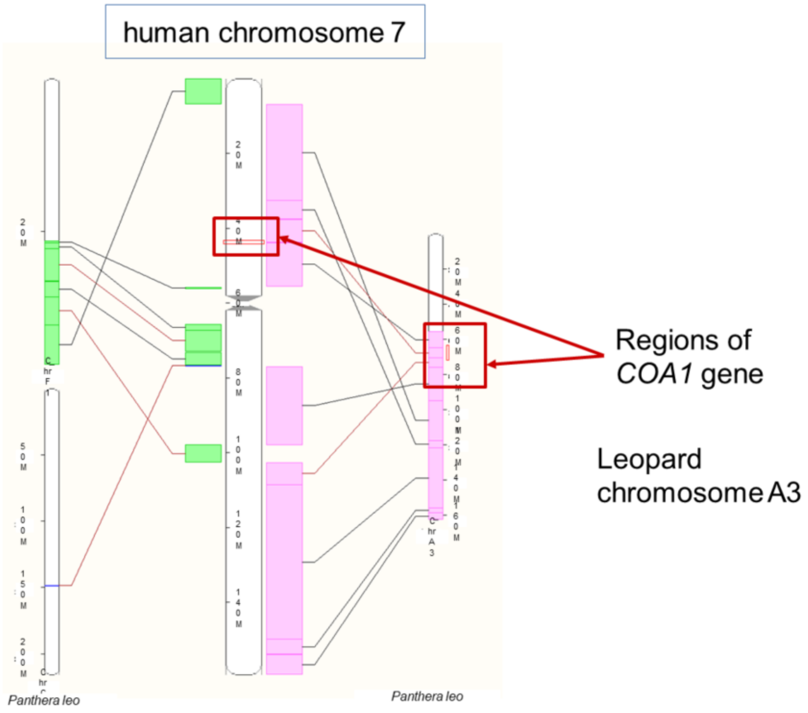

Figure S243: Conserved synteny in the *COA1* region of human chromosome 7 and leopard chromosome A3. Each of the synteny blocks is depicted by different colors and connected by lines that go from the human chromosome to leopard chromosomes. Exact location of the *COA1* gene is depicted by a red box within the chromosome representations.

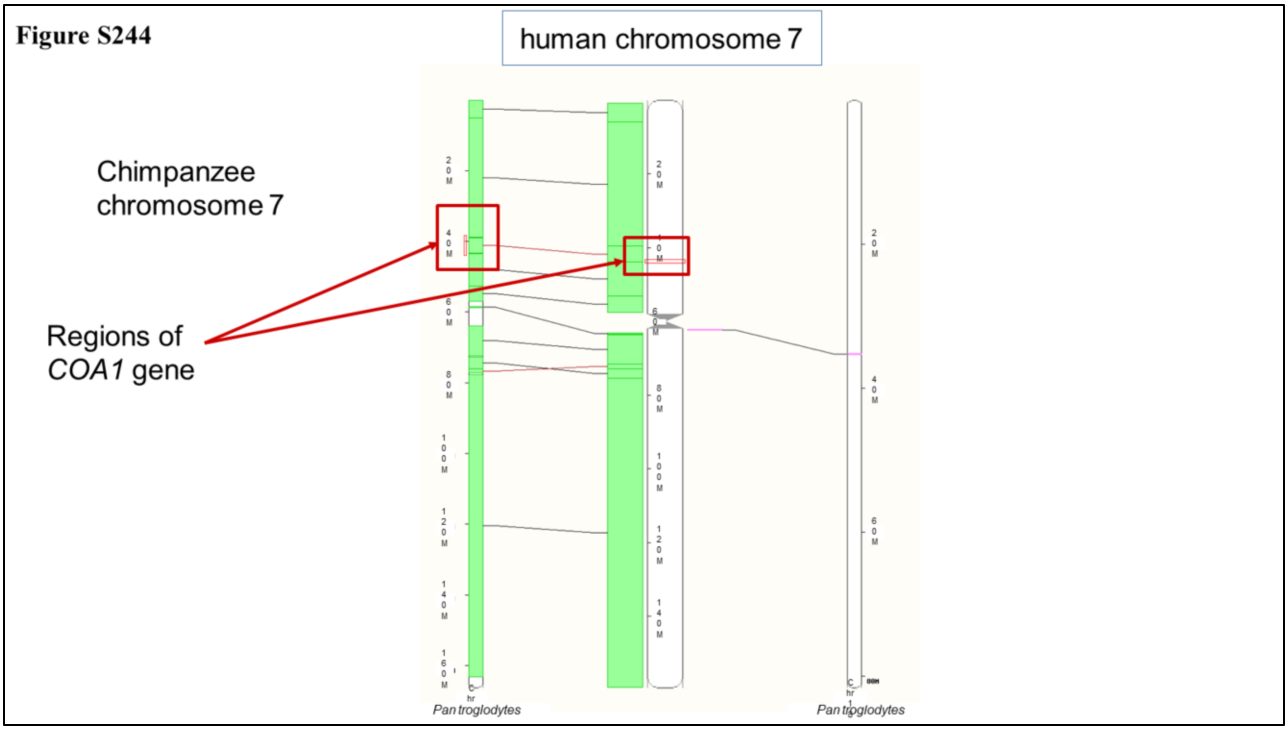

Figure S244: Conserved synteny in the *COA1* region of human chromosome 7 and chimpanzee chromosome 7. Each of the synteny blocks is depicted by different colors and connected by lines that go from the human chromosome to chimpanzee chromosomes. Exact location of the *COA1* gene is depicted by a red box within the chromosome representations.

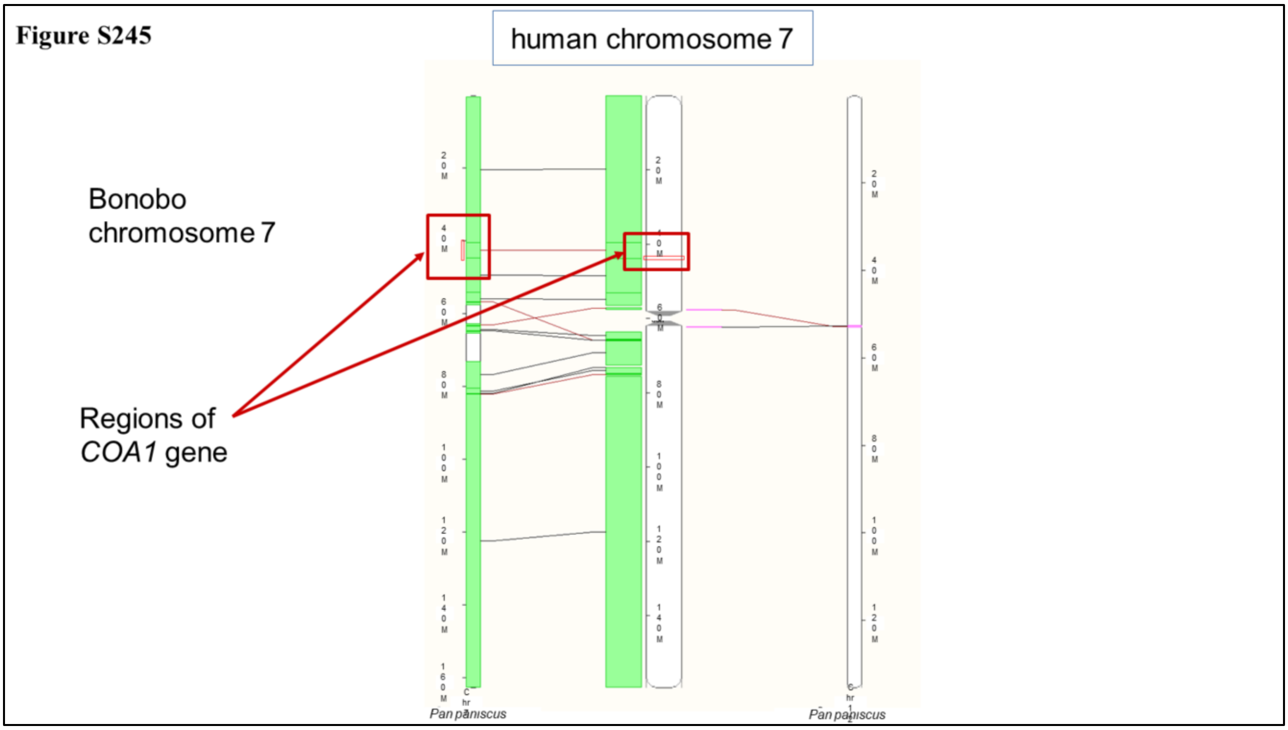

Figure S245: Conserved synteny in the *COA1* region of human chromosome 7 and bonobo chromosome 7. Each of the synteny blocks is depicted by different colors and connected by lines that go from the human chromosome to bonobo chromosomes. Exact location of the *COA1* gene is depicted by a red box within the chromosome representations.

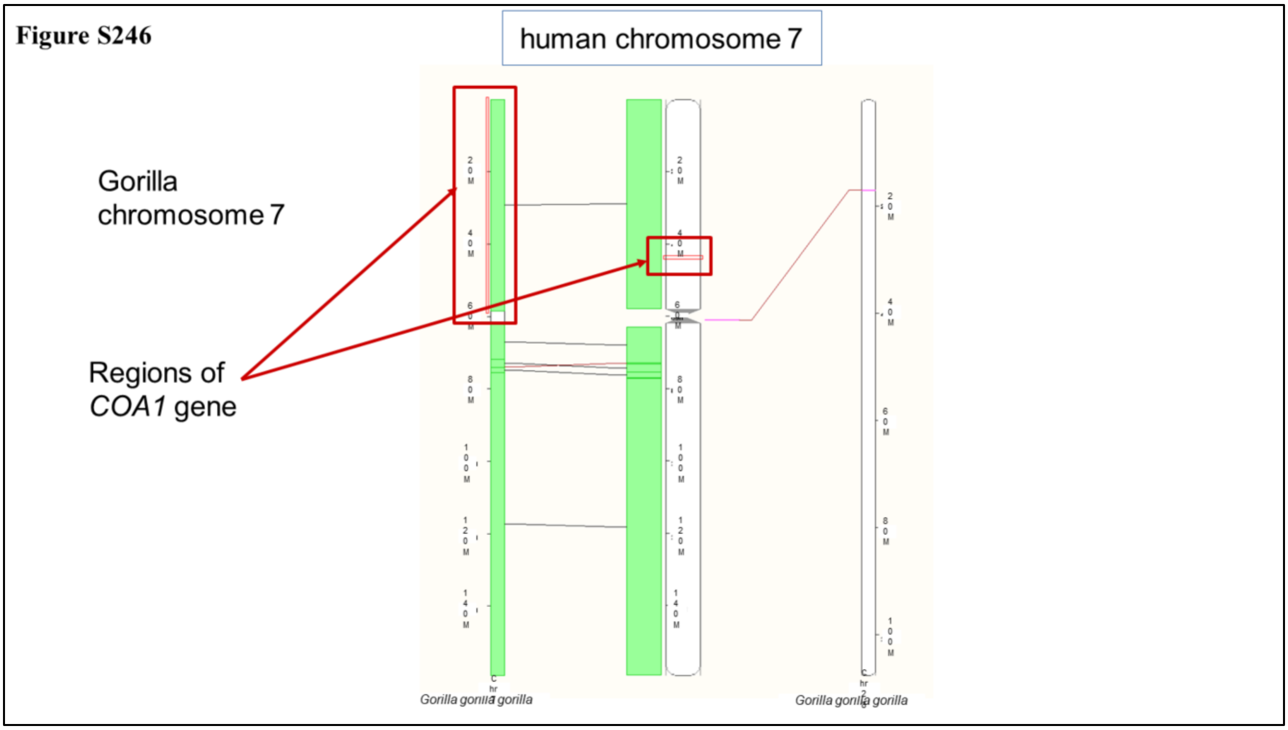

Figure S246: Conserved synteny in the *COA1* region of human chromosome 7 and gorilla chromosome 7. Each of the synteny blocks is depicted by different colors and connected by lines that go from the human chromosome to gorilla chromosomes. Exact location of the *COA1* gene is depicted by a red box within the chromosome representations.

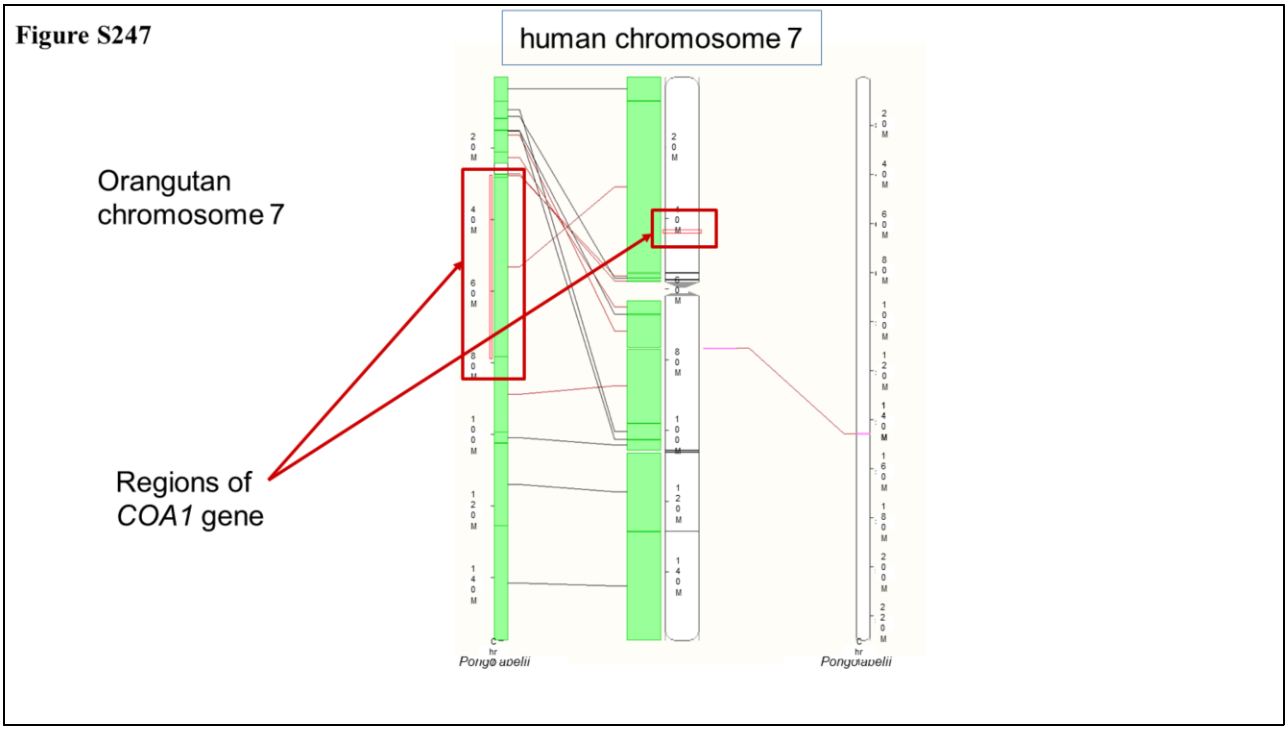

Figure S247: Conserved synteny in the *COA1* region of human chromosome 7 and orangutan chromosome 7. Each of the synteny blocks is depicted by different colors and connected by lines that go from the human chromosome to orangutan chromosomes. Exact location of the *COA1* gene is depicted by a red box within the chromosome representations.

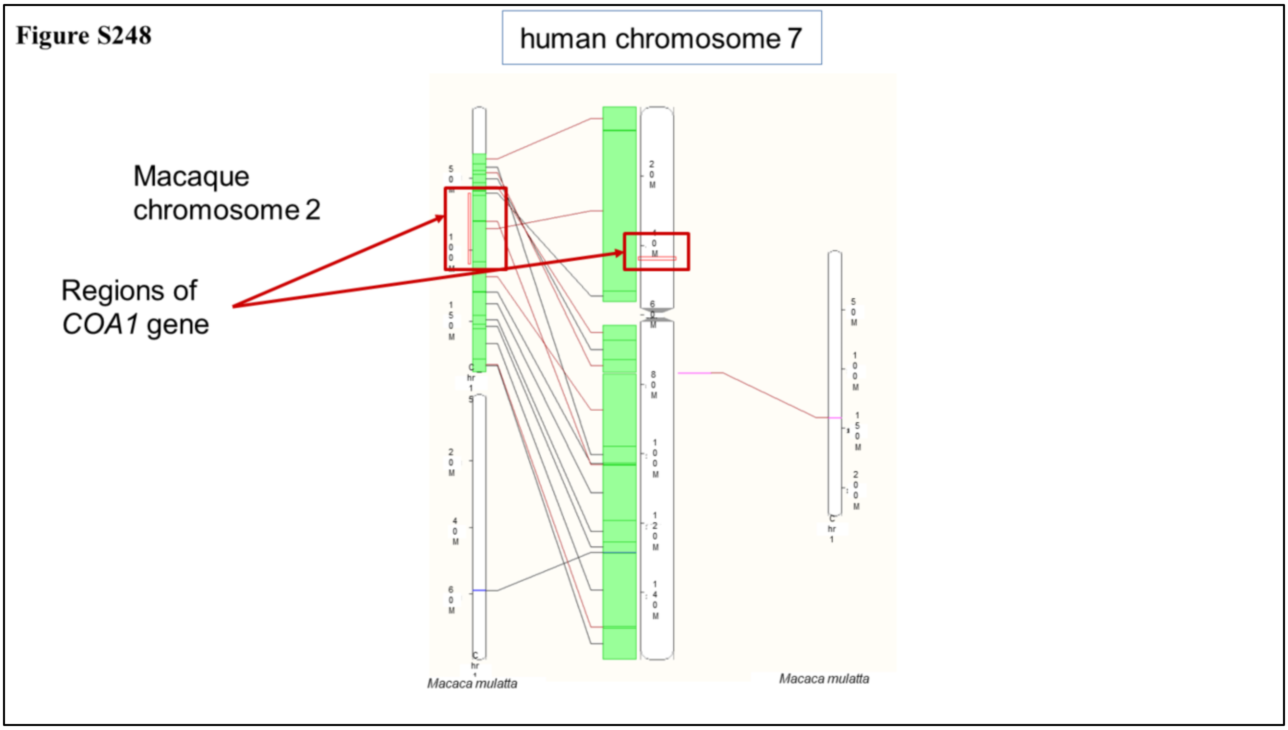

Figure S248: Conserved synteny in the *COA1* region of human chromosome 7 and macaque chromosome 2. Each of the synteny blocks is depicted by different colors and connected by lines that go from the human chromosome to macaque chromosomes. Exact location of the *COA1* gene is depicted by a red box within the chromosome representations.

Figure S249

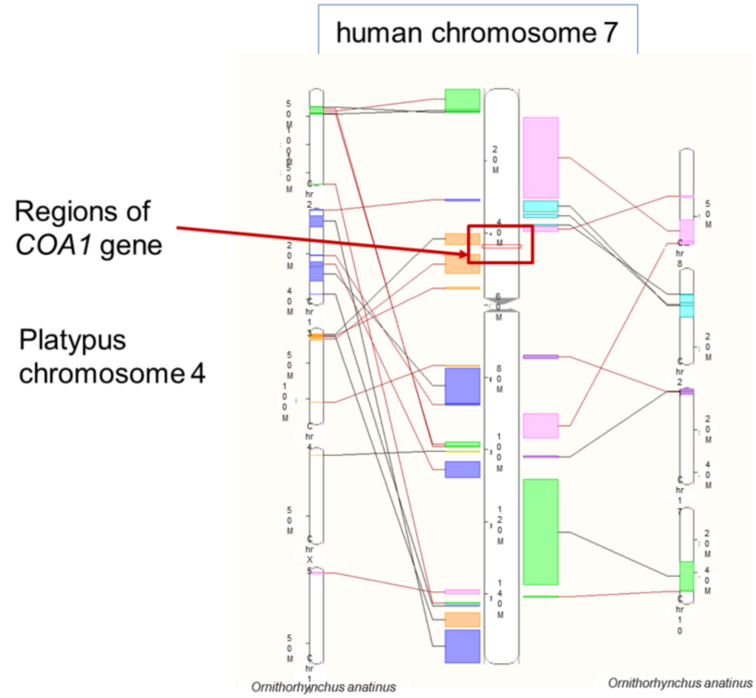

Figure S249: Conserved synteny in the *COA1* region of human chromosome 7 and platypus chromosome 4. Each of the synteny blocks is depicted by different colors and connected by lines that go from the human chromosome to platypus chromosomes. Exact location of the *COA1* gene is depicted by a red box within the chromosome representations.

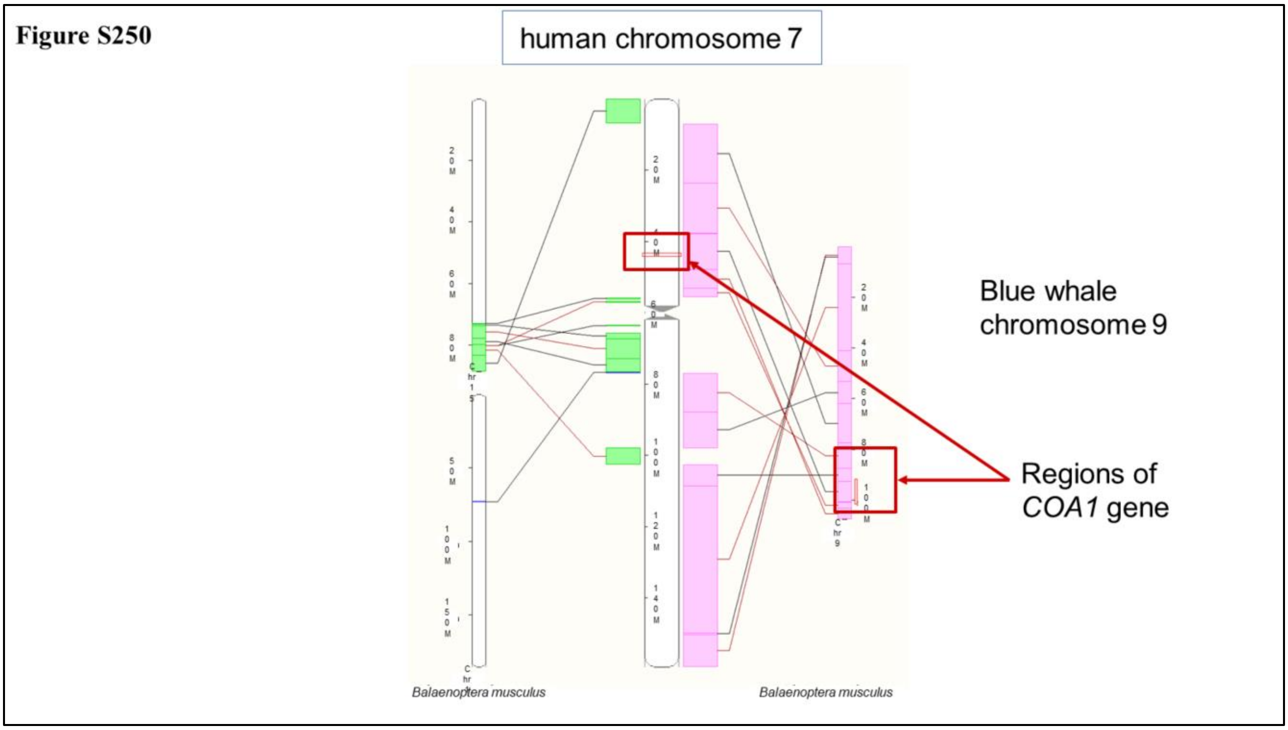

Figure S250: Conserved synteny in the *COA1* region of human chromosome 7 and blue whale chromosome 9. Each of the synteny blocks is depicted by different colors and connected by lines that go from the human chromosome to blue whale chromosomes. Exact location of the *COA1* gene is depicted by a red box within the chromosome representations.

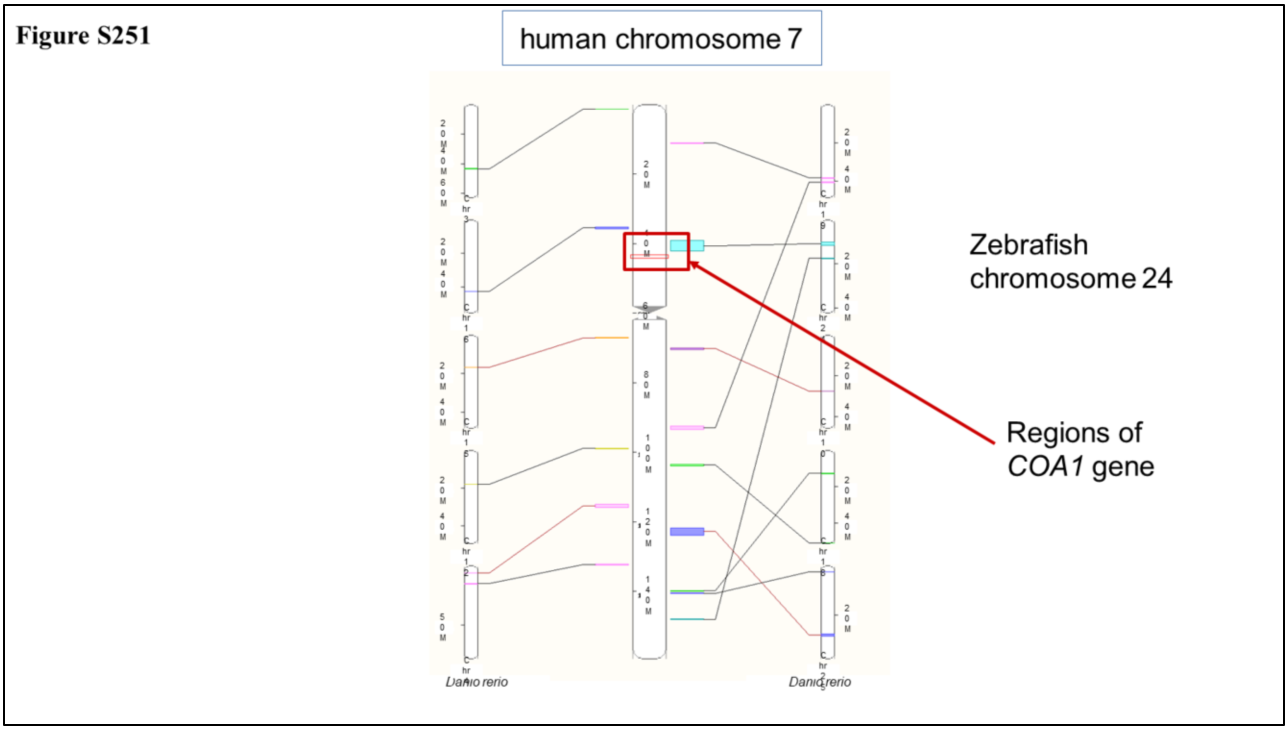

Figure S251: Conserved synteny in the *COA1* region of human chromosome 7 and zebrafish chromosome 24. Each of the synteny blocks is depicted by different colors and connected by lines that go from the human chromosome to zebrafish chromosomes. Exact location of the *COA1* gene is depicted by a red box within the chromosome representations.

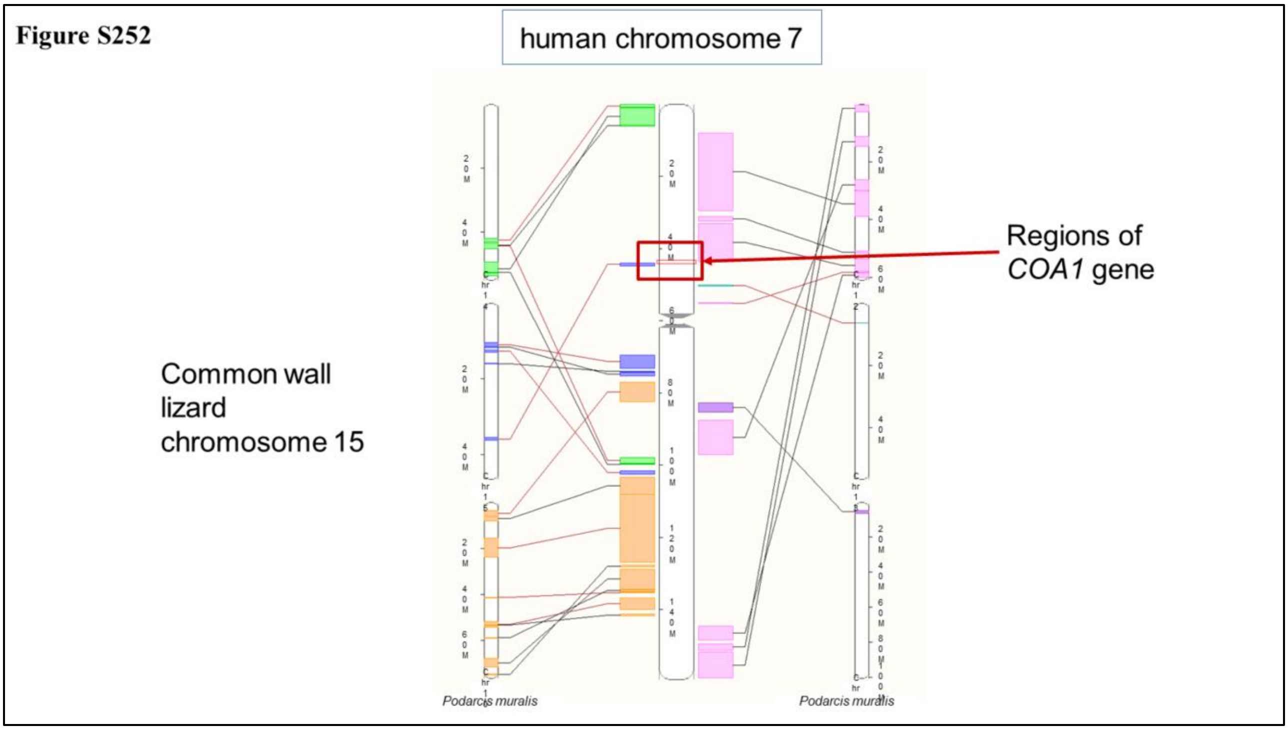

Figure S252: Conserved synteny in the *COA1* region of human chromosome 7 and common wall lizard chromosome 15. Each of the synteny blocks is depicted by different colors and connected by lines that go from the human chromosome to common wall lizard chromosomes. Exact location of the *COA1* gene is depicted by a red box within the chromosome representations.

Figure S253

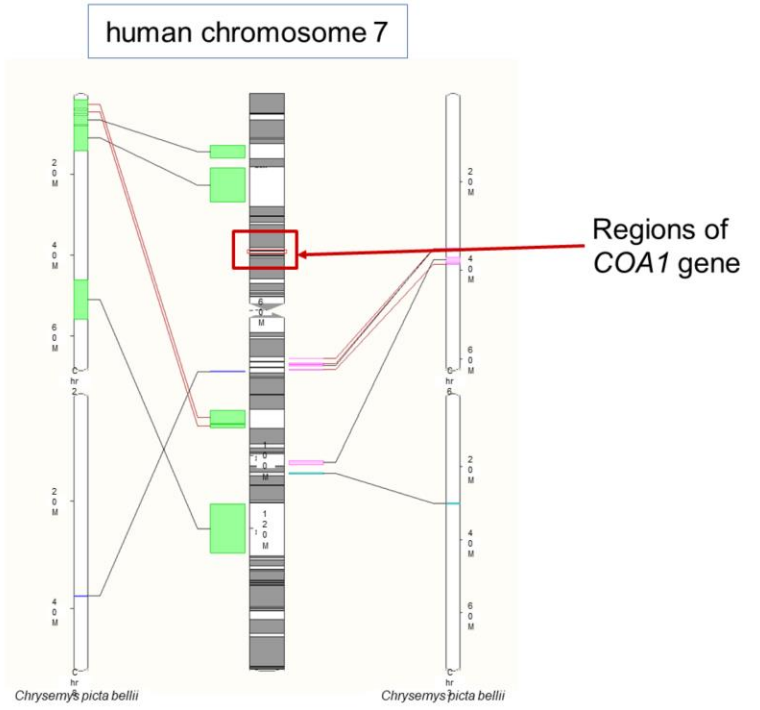

Figure S253: Conserved synteny in the *COA1* region of human chromosome 7 and painted turtle chromosome 2. Each of the synteny blocks is depicted by different colors and connected by lines that go from the human chromosome to painted turtle chromosomes. Exact location of the *COA1* gene is depicted by a red box within the chromosome representations.

Figure S254

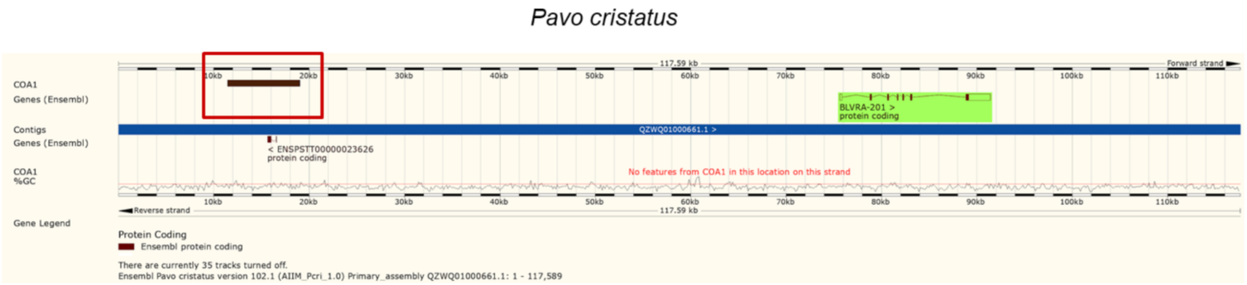

Figure S254: Gene order near *COA1* gene in the Indian peafowl genome. The rectangular boxes connected by thin lines depict genes, with the boxes denoting exons and connecting lines being introns. Direction of transcription is denoted by the > or < symbols (signify positive and negative strand of transcription respectively) beside the gene names. chromosome bands and the genomic coordinates in kilobase (kb) are shown in the topmost row. Details of the contig and scaffold identity are provided in the blue bar located in the middle of the figure. Exact location of the *COA1* gene is depicted by a red box.

Figure S255

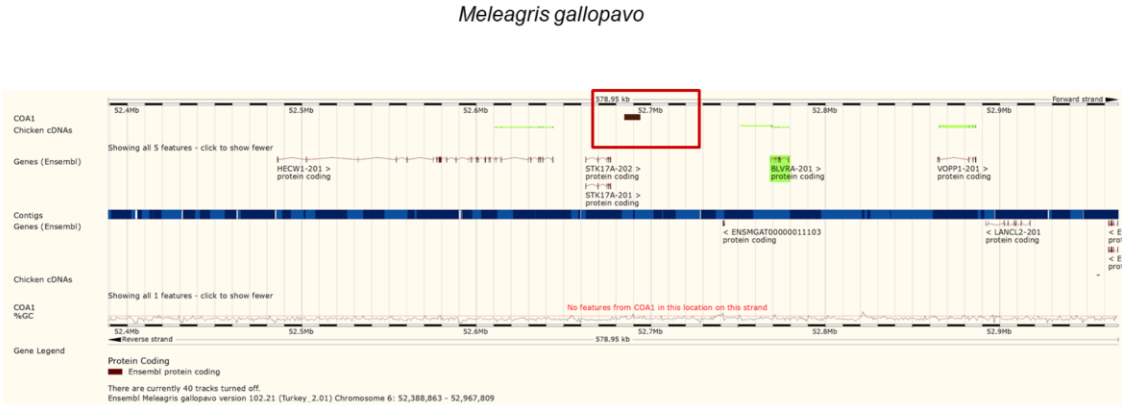

Figure S255: Gene order near *COA1* gene in the turkey genome. The rectangular boxes connected by thin lines depict genes, with the boxes denoting exons and connecting lines being introns. Direction of transcription is denoted by the > or < symbols (signify positive and negative strand of transcription respectively) beside the gene names. chromosome bands and the genomic coordinates in kilobase (kb) are shown in the topmost row. Details of the contig and scaffold identity are provided in the blue bar located in the middle of the figure. Exact location of the *COA1* gene is depicted by a red box.

Figure S256

Coturnix japonica

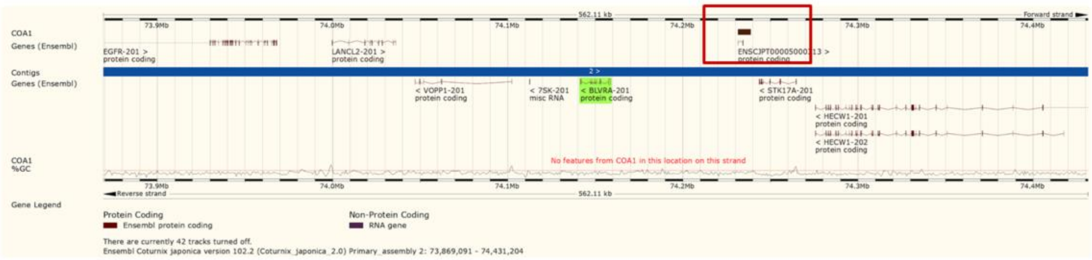

Figure S256: Gene order near *COA1* gene in the Japanese quail genome. The rectangular boxes connected by thin lines depict genes, with the boxes denoting exons and connecting lines being introns. Direction of transcription is denoted by the > or < symbols (signify positive and negative strand of transcription respectively) beside the gene names. chromosome bands and the genomic coordinates in kilobase (kb) are shown in the topmost row. Details of the contig and scaffold identity are provided in the blue bar located in the middle of the figure. Exact location of the *COA1* gene is depicted by a red box.

Figure S257

Numida meleagris

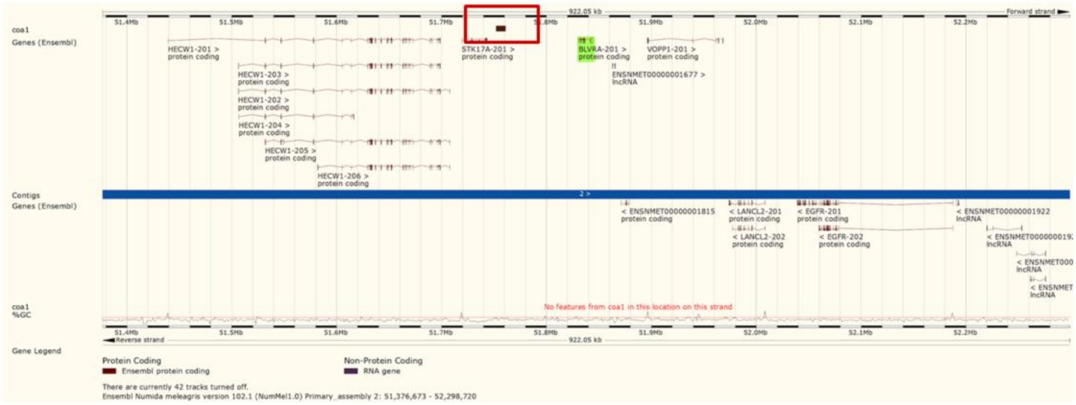

Figure S257: Gene order near *COA1* gene in the helmeted guineafowl genome. The rectangular boxes connected by thin lines depict genes, with the boxes denoting exons and connecting lines being introns. Direction of transcription is denoted by the > or < symbols (signify positive and negative strand of transcription respectively) beside the gene names. chromosome bands and the genomic coordinates in kilobase (kb) are shown in the topmost row. Details of the contig and scaffold identity are provided in the blue bar located in the middle of the figure. Exact location of the *COA1* gene is depicted by a red box.

Figure S258

Anas platyrhynchos

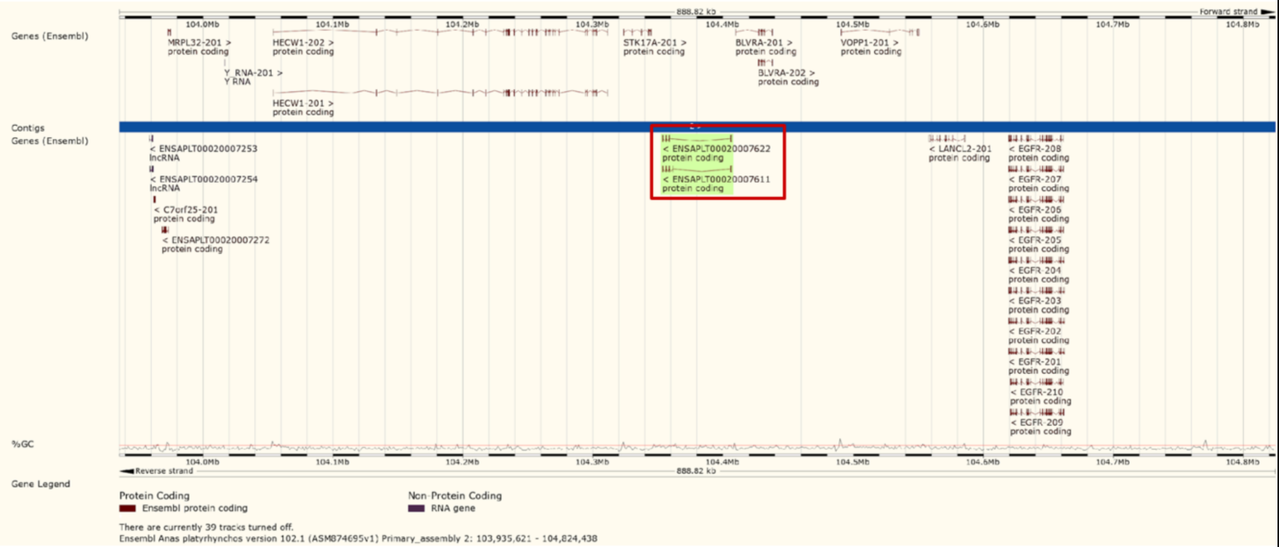

Figure S258: Gene order near *COA1* gene in the mallard genome. The rectangular boxes connected by thin lines depict genes, with the boxes denoting exons and connecting lines being introns. Direction of transcription is denoted by the > or < symbols (signify positive and negative strand of transcription respectively) beside the gene names. chromosome bands and the genomic coordinates in kilobase (kb) are shown in the topmost row. Details of the contig and scaffold identity are provided in the blue bar located in the middle of the figure. Exact location of the *COA1* gene is depicted by a red box.

Figure S259

Apteryx rowi

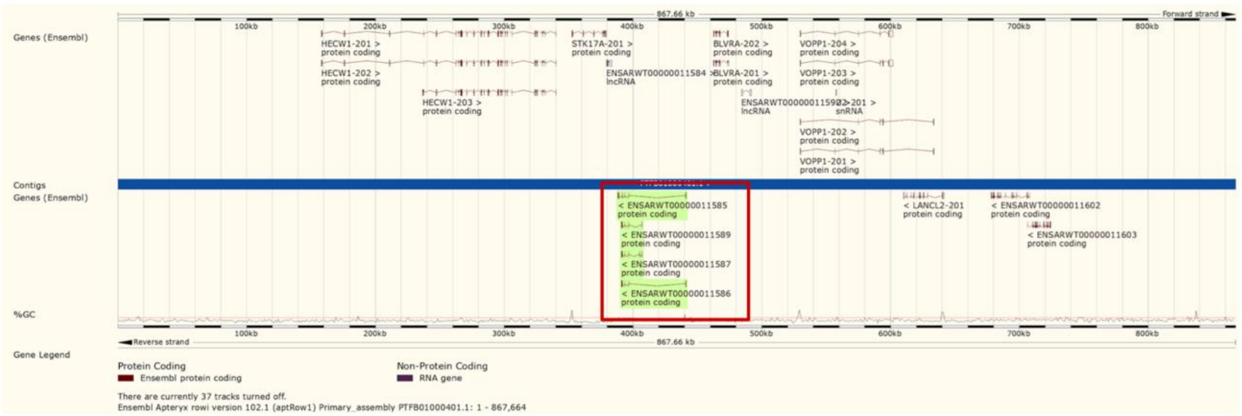

Figure S259: Gene order near *COA1* gene in the Okarito brown kiwi genome. The rectangular boxes connected by thin lines depict genes, with the boxes denoting exons and connecting lines being introns. Direction of transcription is denoted by the > or < symbols (signify positive and negative strand of transcription respectively) beside the gene names. chromosome bands and the genomic coordinates in kilobase (kb) are shown in the topmost row. Details of the contig and scaffold identity are provided in the blue bar located in the middle of the figure. Exact location of the *COA1* gene is depicted by a red box.

Figure S260

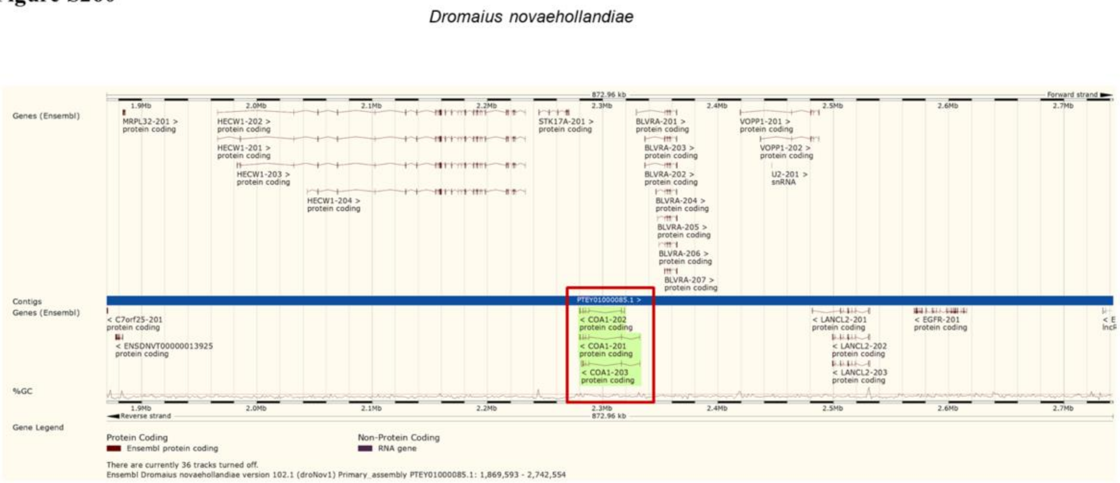

Figure S260: Gene order near *COA1* gene in the emu genome. The rectangular boxes connected by thin lines depict genes, with the boxes denoting exons and connecting lines being introns. Direction of transcription is denoted by the > or < symbols (signify positive and negative strand of transcription respectively) beside the gene names. chromosome bands and the genomic coordinates in kilobase (kb) are shown in the topmost row. Details of the contig and scaffold identity are provided in the blue bar located in the middle of the figure. Exact location of the *COA1* gene is depicted by a red box.

Figure S261

Nothoprocta perdicaria

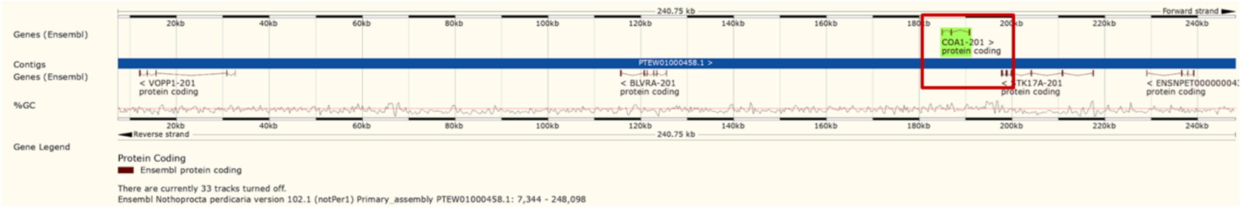

Figure S261: Gene order near *COA1* gene in the Chilean tinamou genome. The rectangular boxes connected by thin lines depict genes, with the boxes denoting exons and connecting lines being introns. Direction of transcription is denoted by the > or < symbols (signify positive and negative strand of transcription respectively) beside the gene names. chromosome bands and the genomic coordinates in kilobase (kb) are shown in the topmost row. Details of the contig and scaffold identity are provided in the blue bar located in the middle of the figure. Exact location of the *COA1* gene is depicted by a red box.

Figure S262

Leptosomus discolor

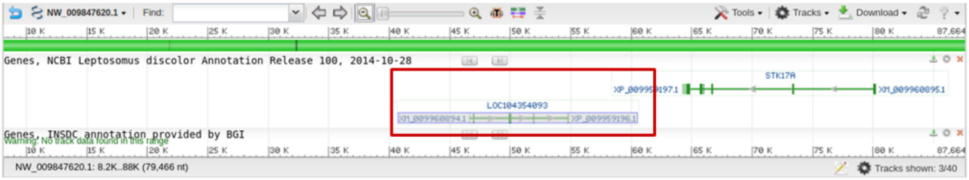

Figure S262: Gene order near *COA1* gene in the cuckoo roller genome. The rectangular boxes connected by thin lines depict genes, with the boxes denoting exons and connecting lines being introns. Direction of transcription is denoted by the > or < symbols (signify positive and negative strand of transcription respectively) beside the gene names. chromosome bands and the genomic coordinates in kilobase (kb) are shown in the topmost row. Details of the contig and scaffold identity are provided in the blue bar located in the middle of the figure. Exact location of the *COA1* gene is depicted by a red box.

Figure S263

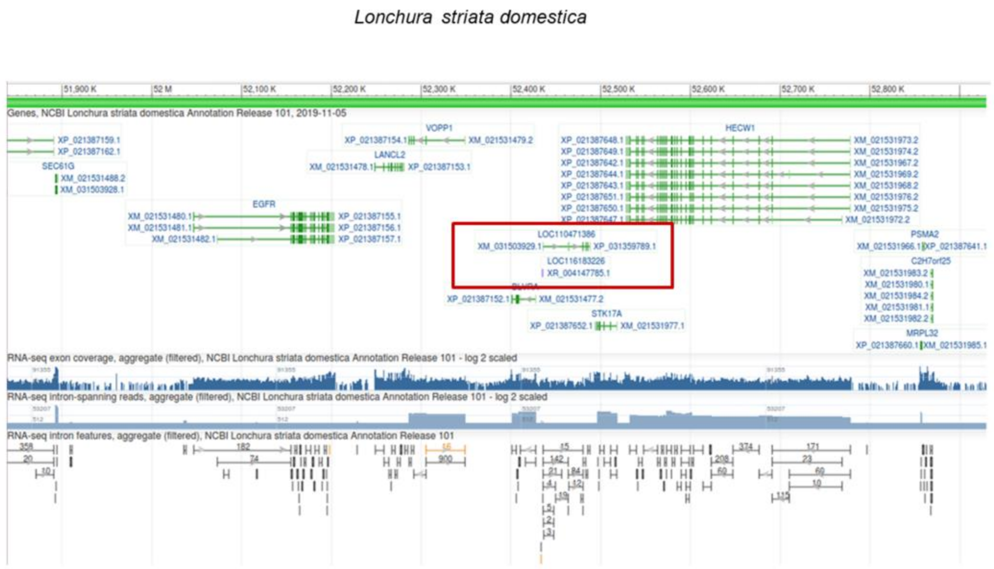

Figure S263: Gene order near *COA1* gene in the Bengalese finch genome. The rectangular boxes connected by thin lines depict genes, with the boxes denoting exons and connecting lines being introns. Direction of transcription is denoted by the > or < symbols (signify positive and negative strand of transcription respectively) beside the gene names. chromosome bands and the genomic coordinates in kilobase (kb) are shown in the topmost row. Details of the contig and scaffold identity are provided in the blue bar located in the middle of the figure. Exact location of the *COA1* gene is depicted by a red box.

Figure S264

Erythrura gouldiae

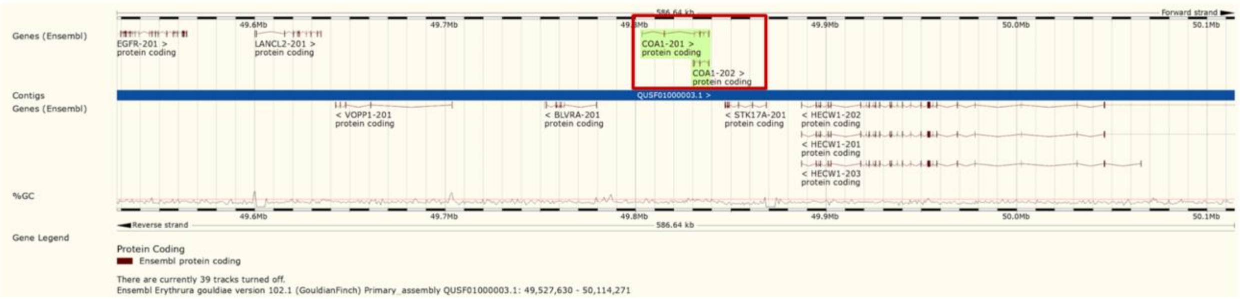

Figure S264: Gene order near *COA1* gene in the Gouldian finch genome. The rectangular boxes connected by thin lines depict genes, with the boxes denoting exons and connecting lines being introns. Direction of transcription is denoted by the > or < symbols (signify positive and negative strand of transcription respectively) beside the gene names. chromosome bands and the genomic coordinates in kilobase (kb) are shown in the topmost row. Details of the contig and scaffold identity are provided in the blue bar located in the middle of the figure. Exact location of the *COA1* gene is depicted by a red box.

Figure S265

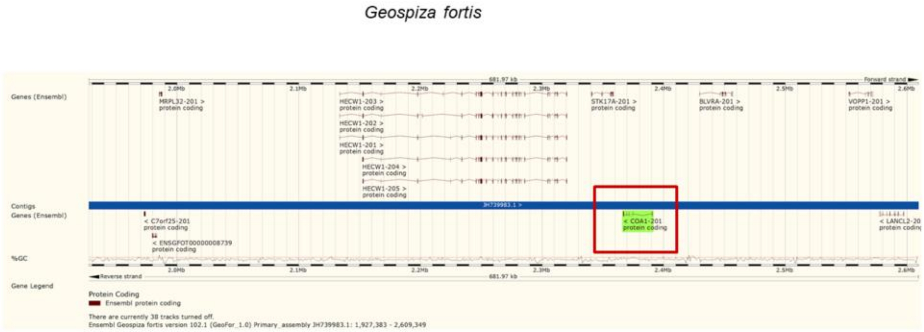

Figure S266: Gene order near *COA1* gene in the medium ground-finch genome. The rectangular boxes connected by thin lines depict genes, with the boxes denoting exons and connecting lines being introns. Direction of transcription is denoted by the > or < symbols (signify positive and negative strand of transcription respectively) beside the gene names. chromosome bands and the genomic coordinates in kilobase (kb) are shown in the topmost row. Details of the contig and scaffold identity are provided in the blue bar located in the middle of the figure. Exact location of the *COA1* gene is depicted by a red box.

Figure S266

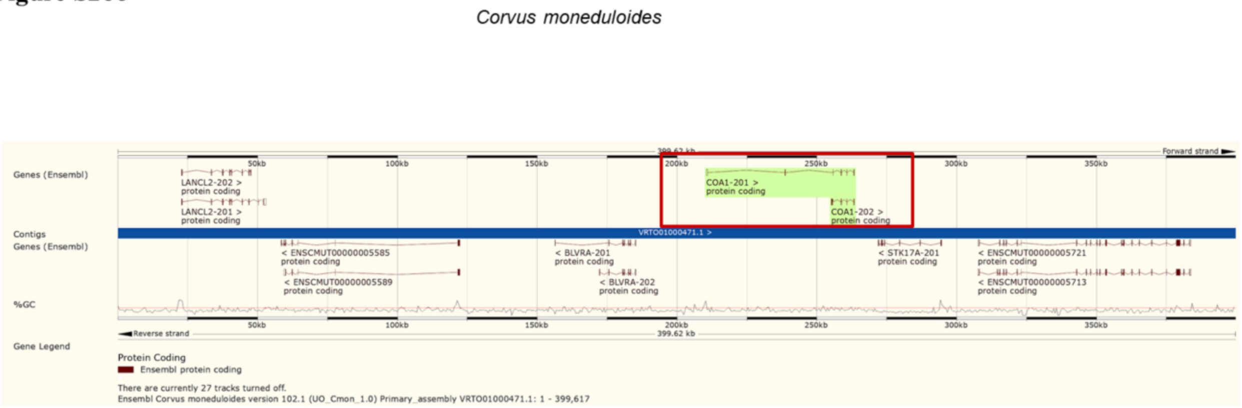

Figure S266: Gene order near *COA1* gene in the new Caledonian crow genome. The rectangular boxes connected by thin lines depict genes, with the boxes denoting exons and connecting lines being introns. Direction of transcription is denoted by the > or < symbols (signify positive and negative strand of transcription respectively) beside the gene names. chromosome bands and the genomic coordinates in kilobase (kb) are shown in the topmost row. Details of the contig and scaffold identity are provided in the blue bar located in the middle of the figure. Exact location of the *COA1* gene is depicted by a red box.

Figure S267

Manacus vitellinus

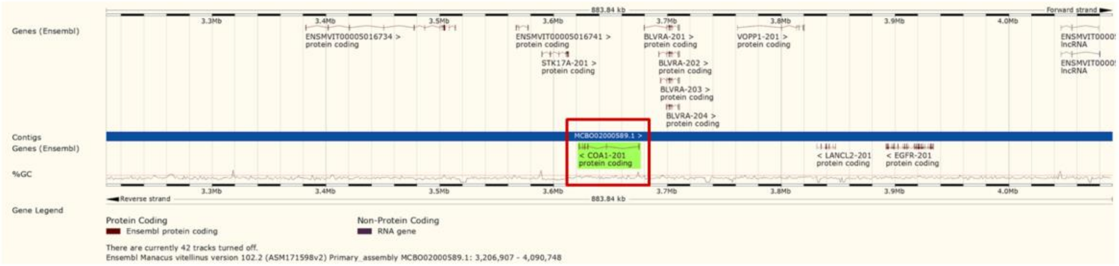

Figure S267: Gene order near *COA1* gene in the golden-collared manakin genome. The rectangular boxes connected by thin lines depict genes, with the boxes denoting exons and connecting lines being introns. Direction of transcription is denoted by the > or < symbols (signify positive and negative strand of transcription respectively) beside the gene names. chromosome bands and the genomic coordinates in kilobase (kb) are shown in the topmost row. Details of the contig and scaffold identity are provided in the blue bar located in the middle of the figure. Exact location of the *COA1* gene is depicted by a red box.

Figure S268

Zonotrichia albicollis

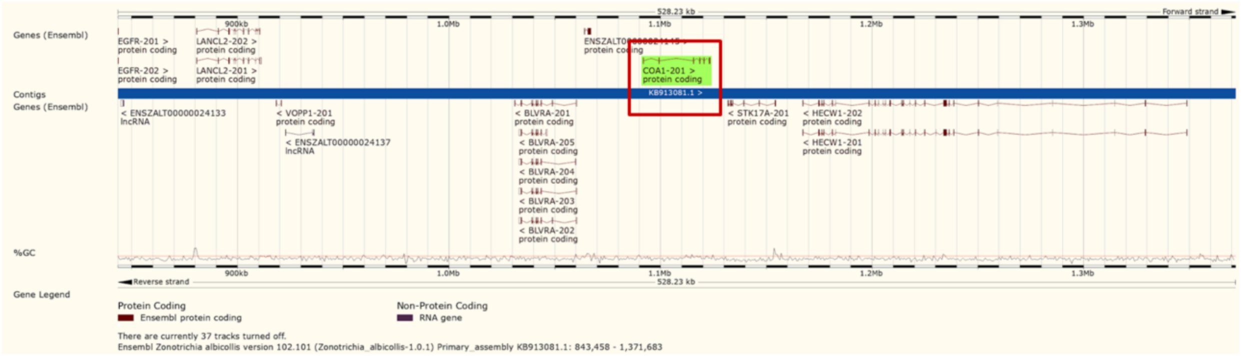

Figure S268: Gene order near *COA1* gene in the white-throated sparrow genome. The rectangular boxes connected by thin lines depict genes, with the boxes denoting exons and connecting lines being introns. Direction of transcription is denoted by the > or < symbols (signify positive and negative strand of transcription respectively) beside the gene names. chromosome bands and the genomic coordinates in kilobase (kb) are shown in the topmost row. Details of the contig and scaffold identity are provided in the blue bar located in the middle of the figure. Exact location of the *COA1* gene is depicted by a red box.

Figure S269

Cyanoderma ruficeps

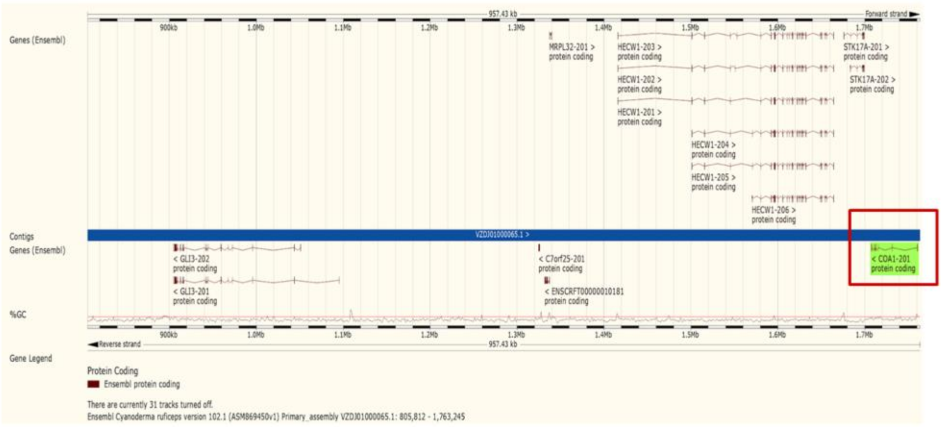

Figure S269: Gene order near *COA1* gene in the rufous-capped babbler genome. The rectangular boxes connected by thin lines depict genes, with the boxes denoting exons and connecting lines being introns. Direction of transcription is denoted by the > or < symbols (signify positive and negative strand of transcription respectively) beside the gene names. chromosome bands and the genomic coordinates in kilobase (kb) are shown in the topmost row. Details of the contig and scaffold identity are provided in the blue bar located in the middle of the figure. Exact location of the *COA1* gene is depicted by a red box.

Figure S270

Struthio camelus australis

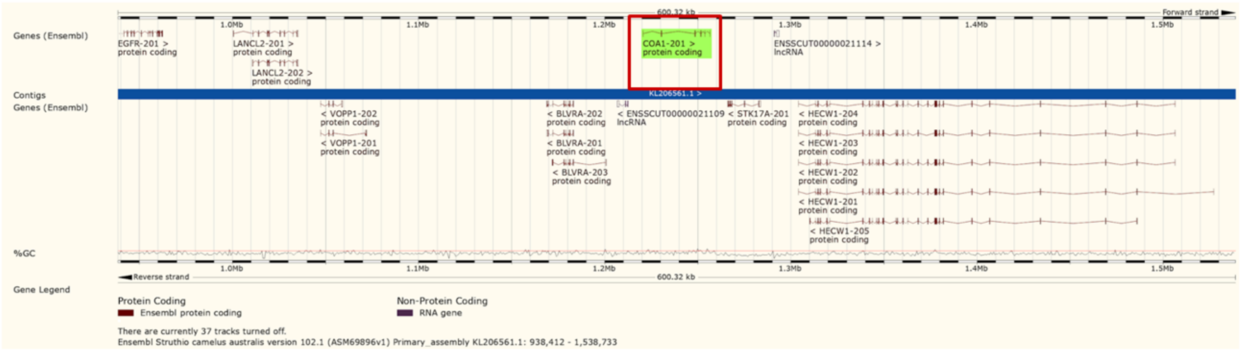

Figure S270: Gene order near *COA1* gene in the African ostrich genome. The rectangular boxes connected by thin lines depict genes, with the boxes denoting exons and connecting lines being introns. Direction of transcription is denoted by the > or < symbols (signify positive and negative strand of transcription respectively) beside the gene names. chromosome bands and the genomic coordinates in kilobase (kb) are shown in the topmost row. Details of the contig and scaffold identity are provided in the blue bar located in the middle of the figure. Exact location of the *COA1* gene is depicted by a red box.

Figure S271

Ficedula albicollis

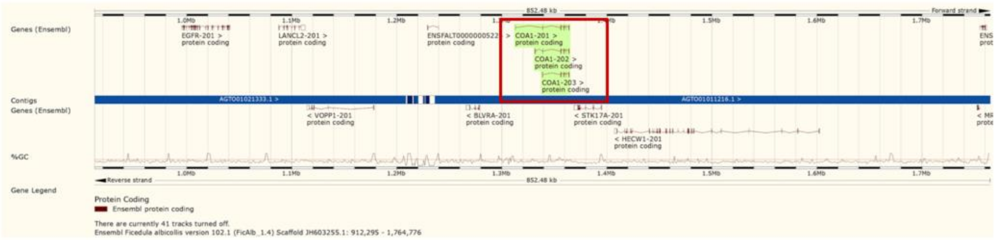

Figure S271: Gene order near *COA1* gene in the flycatcher genome. The rectangular boxes connected by thin lines depict genes, with the boxes denoting exons and connecting lines being introns. Direction of transcription is denoted by the > or < symbols (signify positive and negative strand of transcription respectively) beside the gene names. chromosome bands and the genomic coordinates in kilobase (kb) are shown in the topmost row. Details of the contig and scaffold identity are provided in the blue bar located in the middle of the figure. Exact location of the *COA1* gene is depicted by a red box.

Figure S272

Taeniopygia guttata

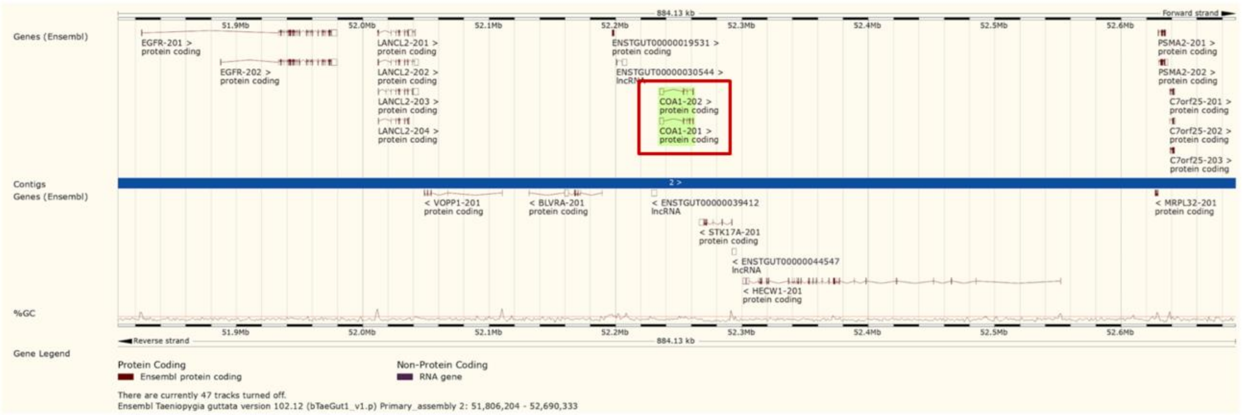

Figure S272: Gene order near *COA1* gene in the zebra finch genome. The rectangular boxes connected by thin lines depict genes, with the boxes denoting exons and connecting lines being introns. Direction of transcription is denoted by the > or < symbols (signify positive and negative strand of transcription respectively) beside the gene names. chromosome bands and the genomic coordinates in kilobase (kb) are shown in the topmost row. Details of the contig and scaffold identity are provided in the blue bar located in the middle of the figure. Exact location of the *COA1* gene is depicted by a red box.

Figure S273

Camarhynchus parvulus

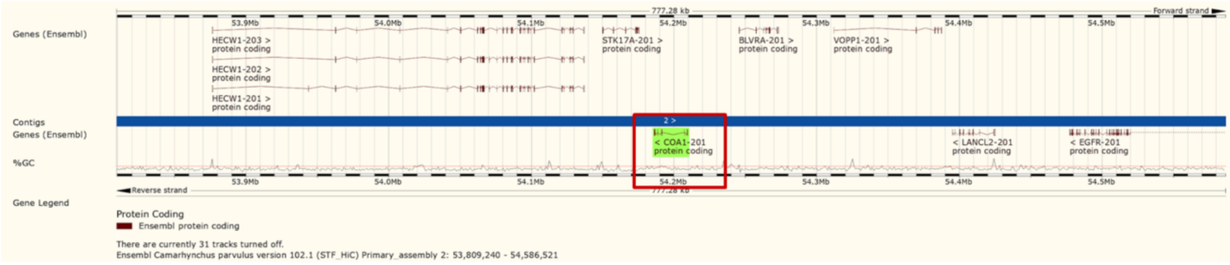

Figure S273: Gene order near *COA1* gene in the small tree finch genome. The rectangular boxes connected by thin lines depict genes, with the boxes denoting exons and connecting lines being introns. Direction of transcription is denoted by the > or < symbols (signify positive and negative strand of transcription respectively) beside the gene names. chromosome bands and the genomic coordinates in kilobase (kb) are shown in the topmost row. Details of the contig and scaffold identity are provided in the blue bar located in the middle of the figure. Exact location of the *COA1* gene is depicted by a red box.

Figure S274

Strigops habroptila

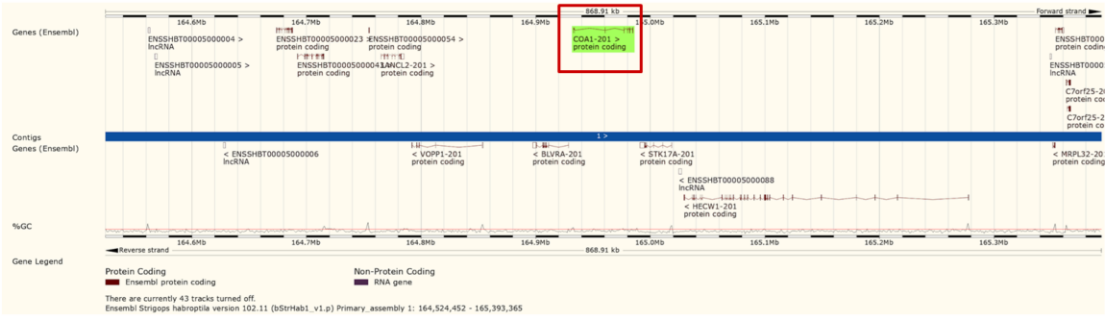

Figure S274: Gene order near *COA1* gene in the kakapo genome. The rectangular boxes connected by thin lines depict genes, with the boxes denoting exons and connecting lines being introns. Direction of transcription is denoted by the > or < symbols (signify positive and negative strand of transcription respectively) beside the gene names. chromosome bands and the genomic coordinates in kilobase (kb) are shown in the topmost row. Details of the contig and scaffold identity are provided in the blue bar located in the middle of the figure. Exact location of the *COA1* gene is depicted by a red box.

Figure S275

Parus major

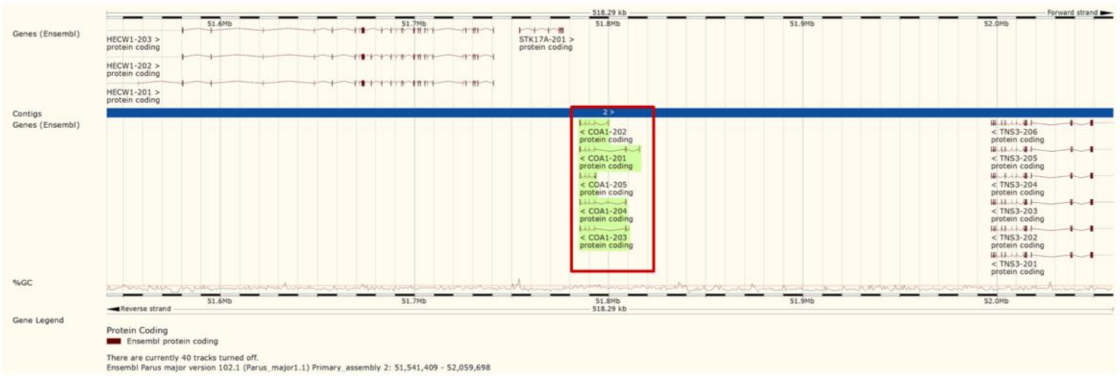

Figure S275: Gene order near *COA1* gene in the great tit genome. The rectangular boxes connected by thin lines depict genes, with the boxes denoting exons and connecting lines being introns. Direction of transcription is denoted by the > or < symbols (signify positive and negative strand of transcription respectively) beside the gene names. chromosome bands and the genomic coordinates in kilobase (kb) are shown in the topmost row. Details of the contig and scaffold identity are provided in the blue bar located in the middle of the figure. Exact location of the *COA1* gene is depicted by a red box.

Figure S276

Aquila chrysaetos chrysaetos

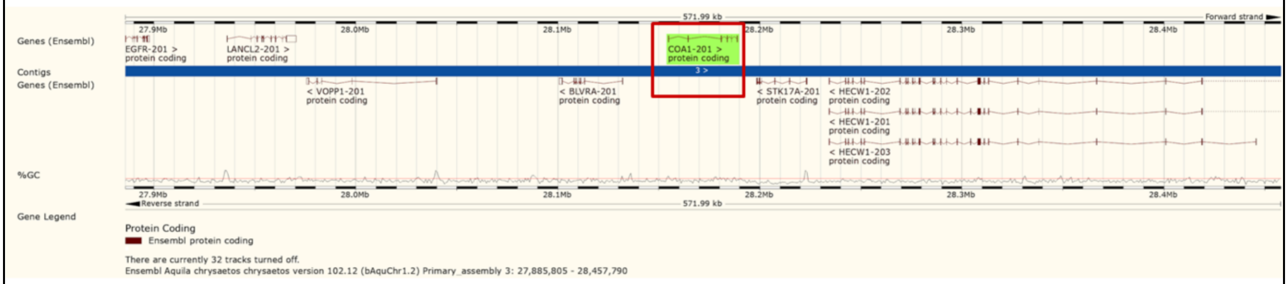

Figure S276: Gene order near *COA1* gene in the golden eagle genome. The rectangular boxes connected by thin lines depict genes, with the boxes denoting exons and connecting lines being introns. Direction of transcription is denoted by the > or < symbols (signify positive and negative strand of transcription respectively) beside the gene names. chromosome bands and the genomic coordinates in kilobase (kb) are shown in the topmost row. Details of the contig and scaffold identity are provided in the blue bar located in the middle of the figure. Exact location of the *COA1* gene is depicted by a red box.

Figure S277

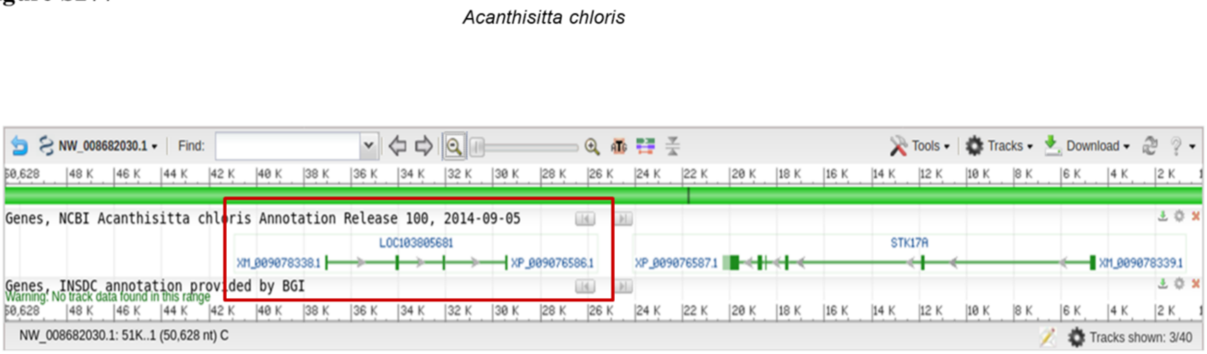

Figure S277: Gene order near *COA1* gene in the rifleman genome. The rectangular boxes connected by thin lines depict genes, with the boxes denoting exons and connecting lines being introns. Direction of transcription is denoted by the > or < symbols (signify positive and negative strand of transcription respectively) beside the gene names. chromosome bands and the genomic coordinates in kilobase (kb) are shown in the topmost row. Details of the contig and scaffold identity are provided in the blue bar located in the middle of the figure. Exact location of the *COA1* gene is depicted by a red box.

Figure S278

Anser cygnoides

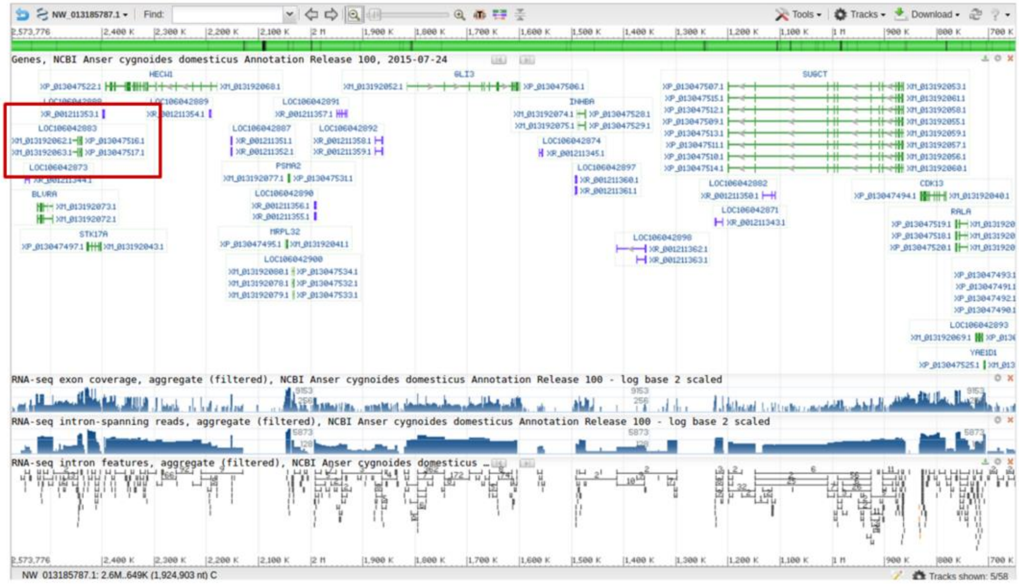

Figure S278: Gene order near *COA1* gene in the swan goose genome. The rectangular boxes connected by thin lines depict genes, with the boxes denoting exons and connecting lines being introns. Direction of transcription is denoted by the > or < symbols (signify positive and negative strand of transcription respectively) beside the gene names. chromosome bands and the genomic coordinates in kilobase (kb) are shown in the topmost row. Details of the contig and scaffold identity are provided in the blue bar located in the middle of the figure. Exact location of the *COA1* gene is depicted by a red box.

Figure S279

Calidris pugnax

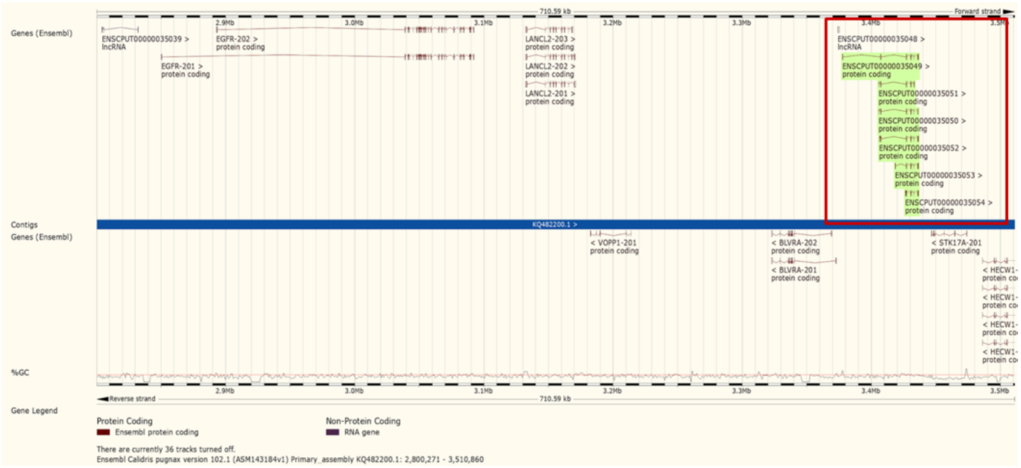

Figure S279: Gene order near *COA1* gene in the ruff genome. The rectangular boxes connected by thin lines depict genes, with the boxes denoting exons and connecting lines being introns. Direction of transcription is denoted by the > or < symbols (signify positive and negative strand of transcription respectively) beside the gene names. chromosome bands and the genomic coordinates in kilobase (kb) are shown in the topmost row. Details of the contig and scaffold identity are provided in the blue bar located in the middle of the figure. Exact location of the *COA1* gene is depicted by a red box.

Figure S280

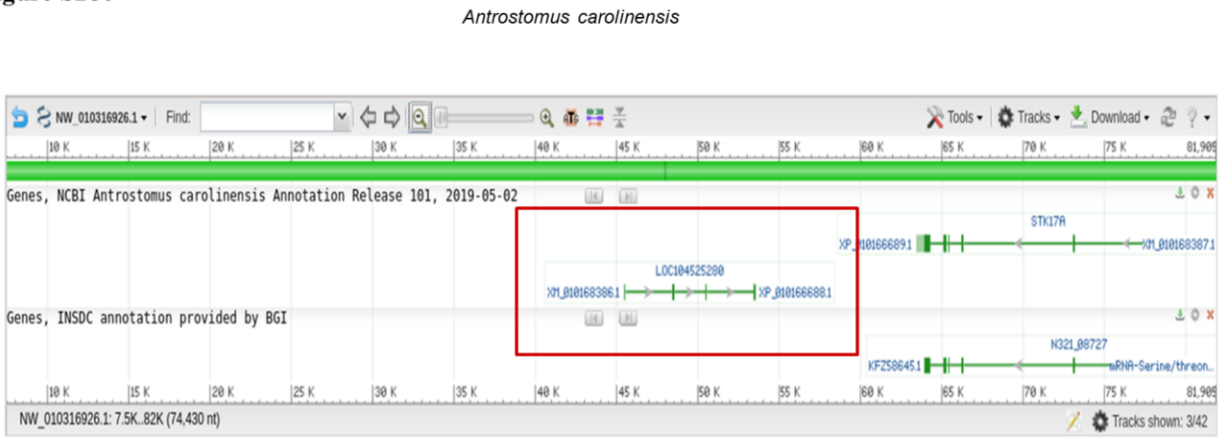

Figure S280: Gene order near *COA1* gene in the chuck-will-widow genome. The rectangular boxes connected by thin lines depict genes, with the boxes denoting exons and connecting lines being introns. Direction of transcription is denoted by the > or < symbols (signify positive and negative strand of transcription respectively) beside the gene names. chromosome bands and the genomic coordinates in kilobase (kb) are shown in the topmost row. Details of the contig and scaffold identity are provided in the blue bar located in the middle of the figure. Exact location of the *COA1* gene is depicted by a red box.

Figure S281

Apaloderma vittatum

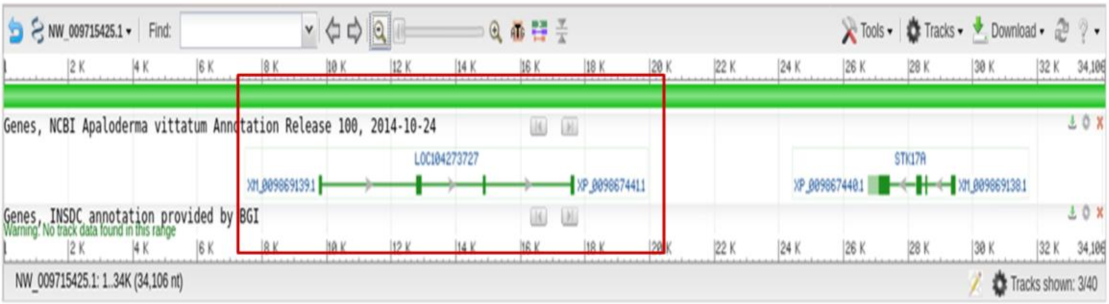

Figure S281: Gene order near *COA1* gene in the bar-tailed trogon genome. The rectangular boxes connected by thin lines depict genes, with the boxes denoting exons and connecting lines being introns. Direction of transcription is denoted by the > or < symbols (signify positive and negative strand of transcription respectively) beside the gene names. chromosome bands and the genomic coordinates in kilobase (kb) are shown in the topmost row. Details of the contig and scaffold identity are provided in the blue bar located in the middle of the figure. Exact location of the *COA1* gene is depicted by a red box.

Figure S282

Aptenodytes forsteri

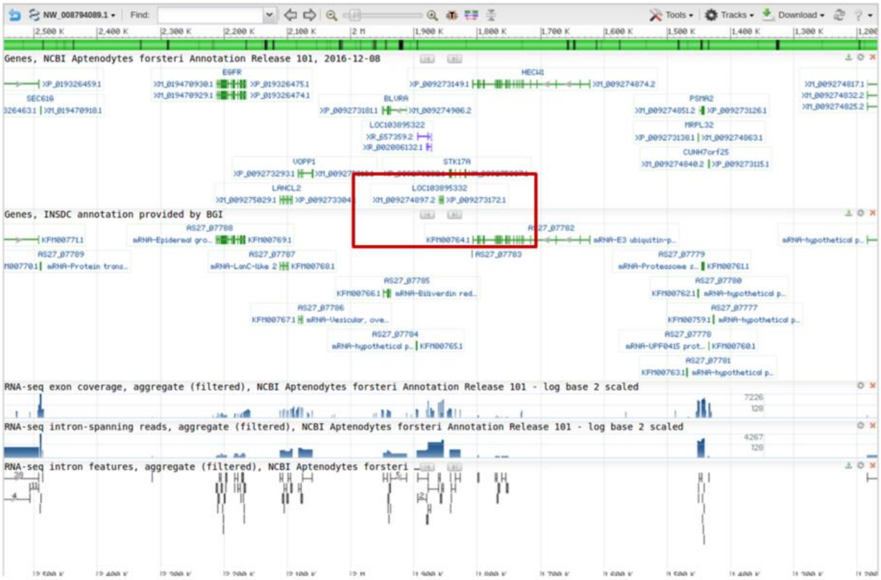

Figure S282: Gene order near *COA1* gene in the emperor penguin genome. The rectangular boxes connected by thin lines depict genes, with the boxes denoting exons and connecting lines being introns. Direction of transcription is denoted by the > or < symbols (signify positive and negative strand of transcription respectively) beside the gene names. chromosome bands and the genomic coordinates in kilobase (kb) are shown in the topmost row. Details of the contig and scaffold identity are provided in the blue bar located in the middle of the figure. Exact location of the *COA1* gene is depicted by a red box.

Figure S283

Apteryx australis mantelli

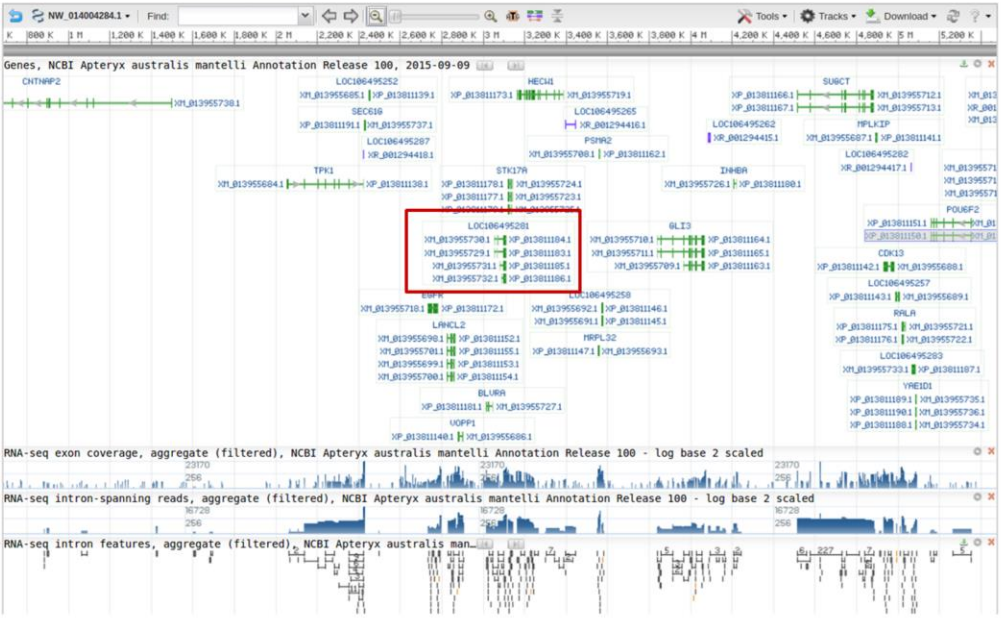

Figure S283: Gene order near *COA1* gene in the North Island brown kiwi genome. The rectangular boxes connected by thin lines depict genes, with the boxes denoting exons and connecting lines being introns. Direction of transcription is denoted by the > or < symbols (signify positive and negative strand of transcription respectively) beside the gene names. chromosome bands and the genomic coordinates in kilobase (kb) are shown in the topmost row. Details of the contig and scaffold identity are provided in the blue bar located in the middle of the figure. Exact location of the *COA1* gene is depicted by a red box.

Figure S284

Aythya fuligula

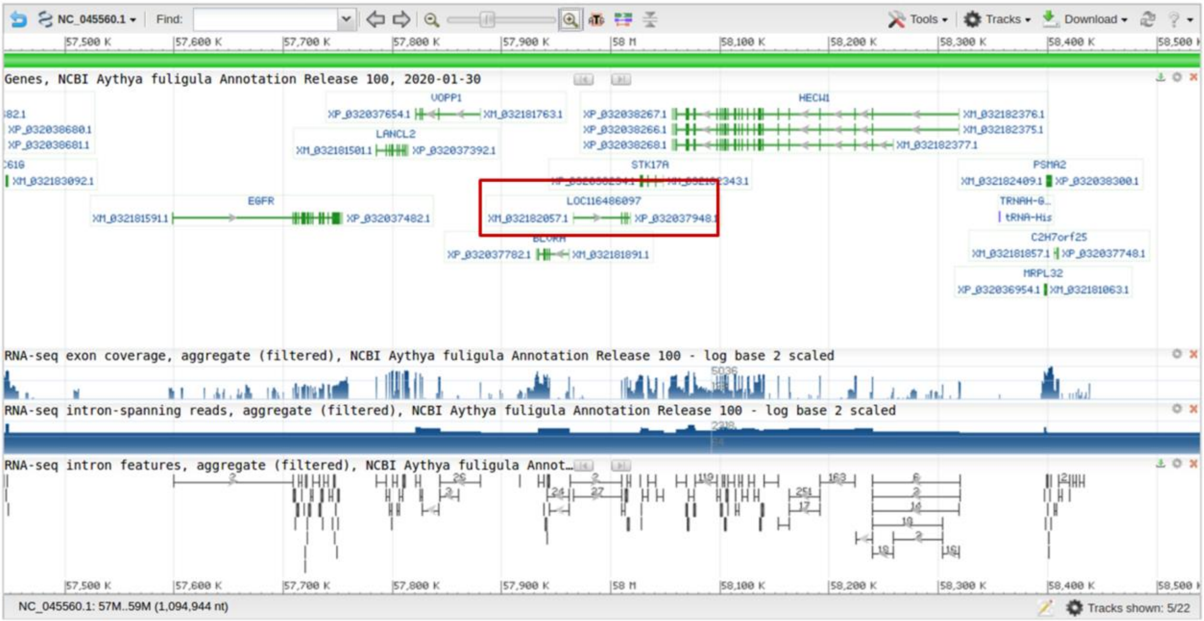

Figure S284: Gene order near *COA1* gene in tufted duck genome. The rectangular boxes connected by thin lines depict genes, with the boxes denoting exons and connecting lines being introns. Direction of transcription is denoted by the > or < symbols (signify positive and negative strand of transcription respectively) beside the gene names. chromosome bands and the genomic coordinates in kilobase (kb) are shown in the topmost row. Details of the contig and scaffold identity are provided in the blue bar located in the middle of the figure. Exact location of the *COA1* gene is depicted by a red box.

Figure S285

Balearica regulorum gibbericeps

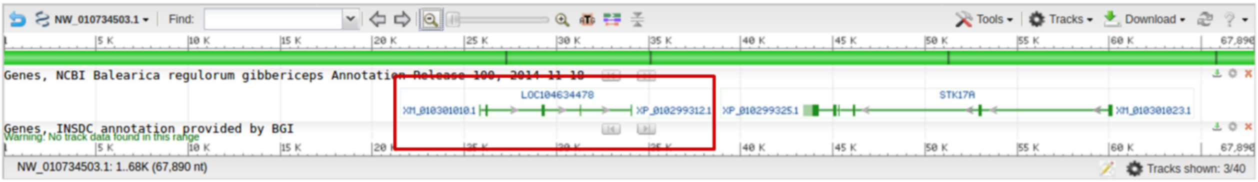

Figure S285: Gene order near *COA1* gene in the East African gray crowned-crane genome. The rectangular boxes connected by thin lines depict genes, with the boxes denoting exons and connecting lines being introns. Direction of transcription is denoted by the > or < symbols (signify positive and negative strand of transcription respectively) beside the gene names. chromosome bands and the genomic coordinates in kilobase (kb) are shown in the topmost row. Details of the contig and scaffold identity are provided in the blue bar located in the middle of the figure. Exact location of the *COA1* gene is depicted by a red box.

Figure S286

Cyanistes caeruleus

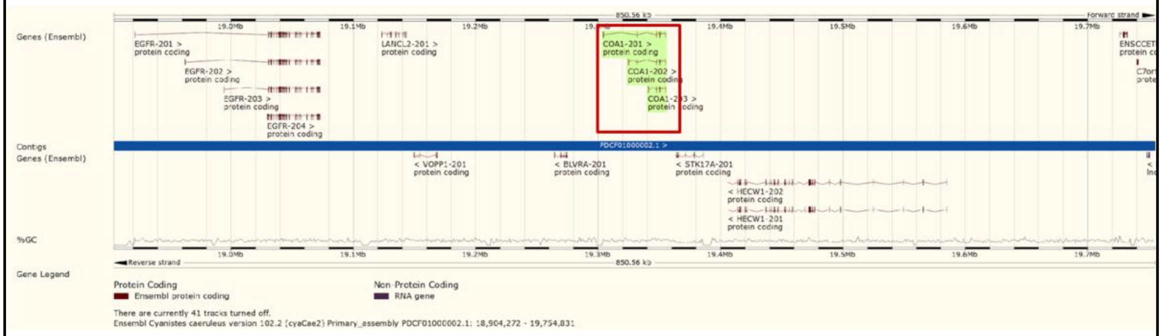

Figure S286: Gene order near *COA1* gene in the blue tit genome. The rectangular boxes connected by thin lines depict genes, with the boxes denoting exons and connecting lines being introns. Direction of transcription is denoted by the > or < symbols (signify positive and negative strand of transcription respectively) beside the gene names. chromosome bands and the genomic coordinates in kilobase (kb) are shown in the topmost row. Details of the contig and scaffold identity are provided in the blue bar located in the middle of the figure. Exact location of the *COA1* gene is depicted by a red box.

Figure S287

Lepidothrix coronata

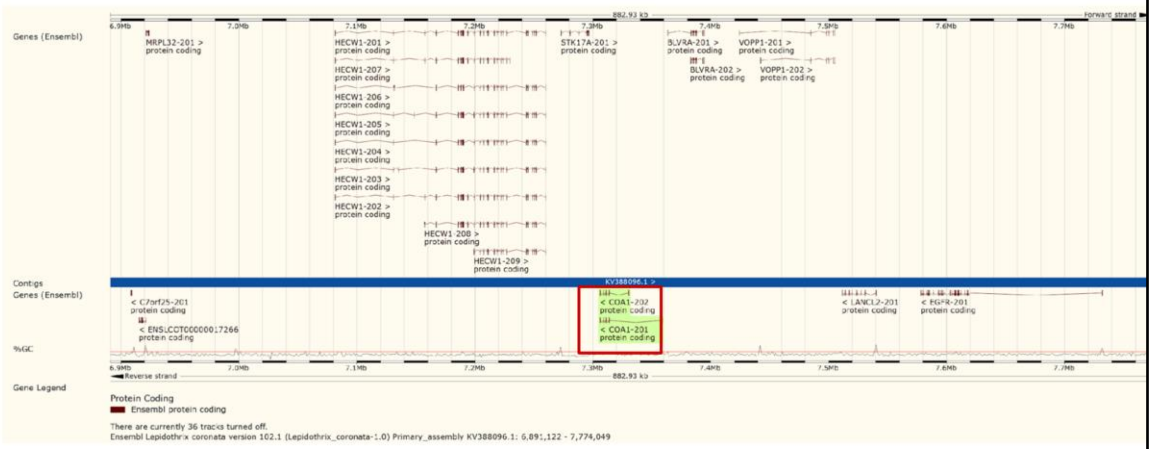

Figure S287: Gene order near *COA1* gene in the blue-crowned manakin genome. The rectangular boxes connected by thin lines depict genes, with the boxes denoting exons and connecting lines being introns. Direction of transcription is denoted by the > or < symbols (signify positive and negative strand of transcription respectively) beside the gene names. chromosome bands and the genomic coordinates in kilobase (kb) are shown in the topmost row. Details of the contig and scaffold identity are provided in the blue bar located in the middle of the figure. Exact location of the *COA1* gene is depicted by a red box.

Figure S288

Buceros rhinoceros silvestris

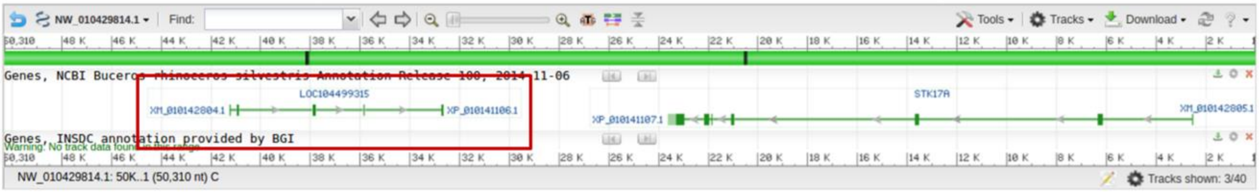

Figure S288: Gene order near *COA1* gene in the rhinoceros hornbill genome. The rectangular boxes connected by thin lines depict genes, with the boxes denoting exons and connecting lines being introns. Direction of transcription is denoted by the > or < symbols (signify positive and negative strand of transcription respectively) beside the gene names. chromosome bands and the genomic coordinates in kilobase (kb) are shown in the topmost row. Details of the contig and scaffold identity are provided in the blue bar located in the middle of the figure. Exact location of the *COA1* gene is depicted by a red box.

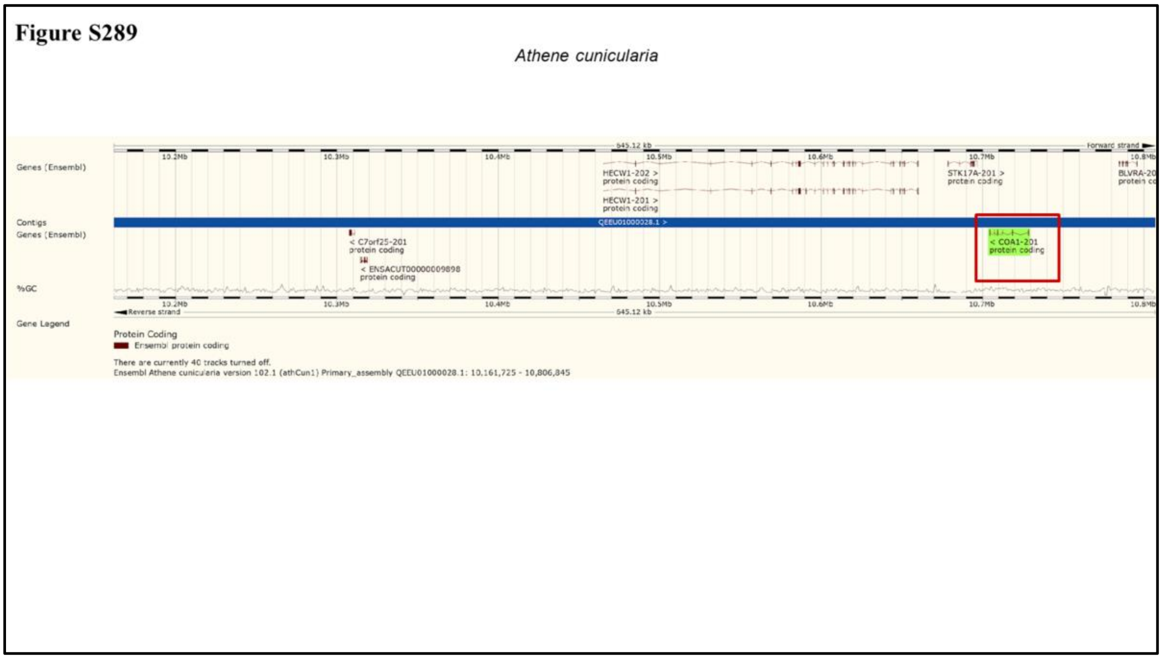

Figure S289: Gene order near *COA1* gene in the burrowing owl genome. The rectangular boxes connected by thin lines depict genes, with the boxes denoting exons and connecting lines being introns. Direction of transcription is denoted by the > or < symbols (signify positive and negative strand of transcription respectively) beside the gene names. chromosome bands and the genomic coordinates in kilobase (kb) are shown in the topmost row. Details of the contig and scaffold identity are provided in the blue bar located in the middle of the figure. Exact location of the *COA1* gene is depicted by a red box.

Figure S290

Calypte anna

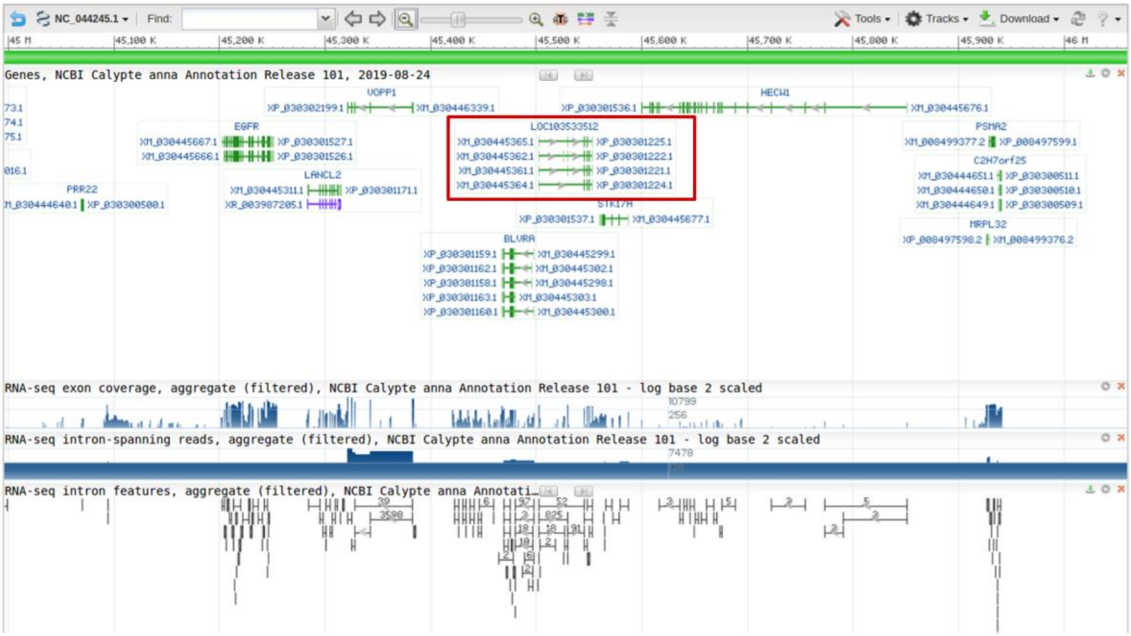

Figure S290: Gene order near *COA1* gene in the Anna's hummingbird genome. The rectangular boxes connected by thin lines depict genes, with the boxes denoting exons and connecting lines being introns. Direction of transcription is denoted by the > or < symbols (signify positive and negative strand of transcription respectively) beside the gene names. chromosome bands and the genomic coordinates in kilobase (kb) are shown in the topmost row. Details of the contig and scaffold identity are provided in the blue bar located in the middle of the figure. Exact location of the *COA1* gene is depicted by a red box.

Figure S291

Cariama cristata

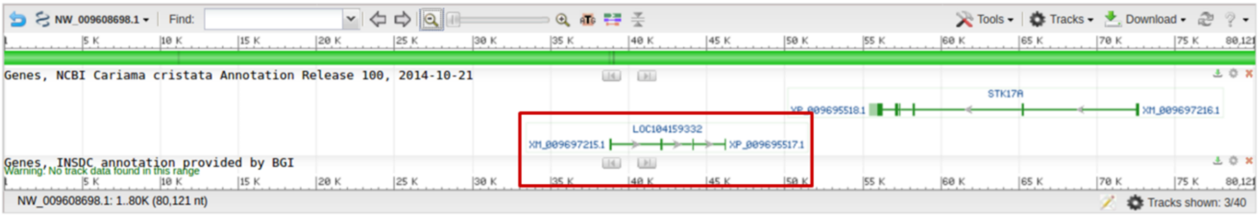

Figure S291: Gene order near *COA1* gene in the red-legged seriema genome. The rectangular boxes connected by thin lines depict genes, with the boxes denoting exons and connecting lines being introns. Direction of transcription is denoted by the > or < symbols (signify positive and negative strand of transcription respectively) beside the gene names. chromosome bands and the genomic coordinates in kilobase (kb) are shown in the topmost row. Details of the contig and scaffold identity are provided in the blue bar located in the middle of the figure. Exact location of the *COA1* gene is depicted by a red box.

Figure S292

Chaetura pelagica

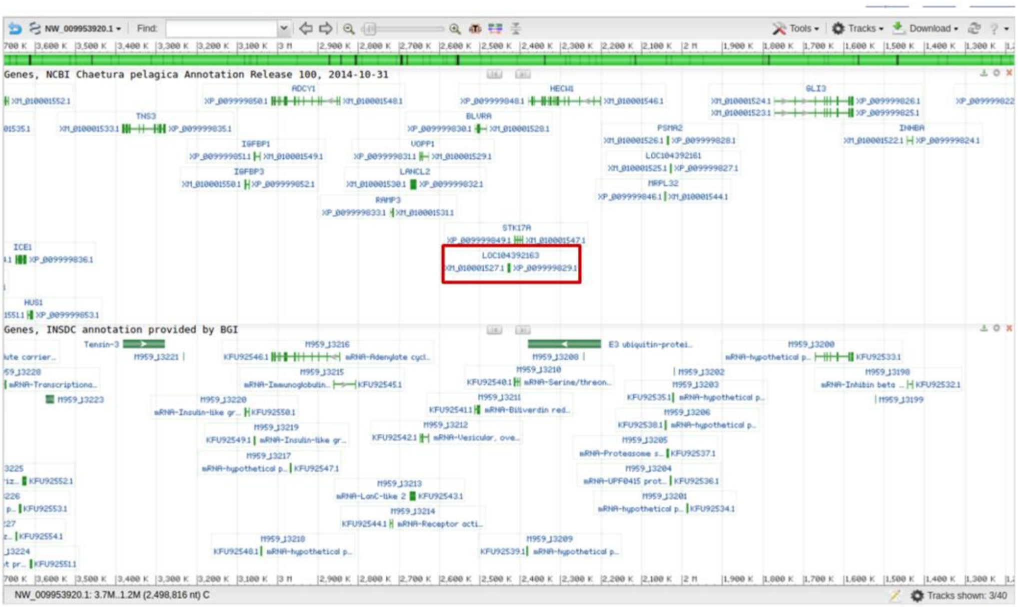

Figure S292: Gene order near *COA1* gene in the chimney swift genome. The rectangular boxes connected by thin lines depict genes, with the boxes denoting exons and connecting lines being introns. Direction of transcription is denoted by the > or < symbols (signify positive and negative strand of transcription respectively) beside the gene names. chromosome bands and the genomic coordinates in kilobase (kb) are shown in the topmost row. Details of the contig and scaffold identity are provided in the blue bar located in the middle of the figure. Exact location of the *COA1* gene is depicted by a red box.

Figure S293

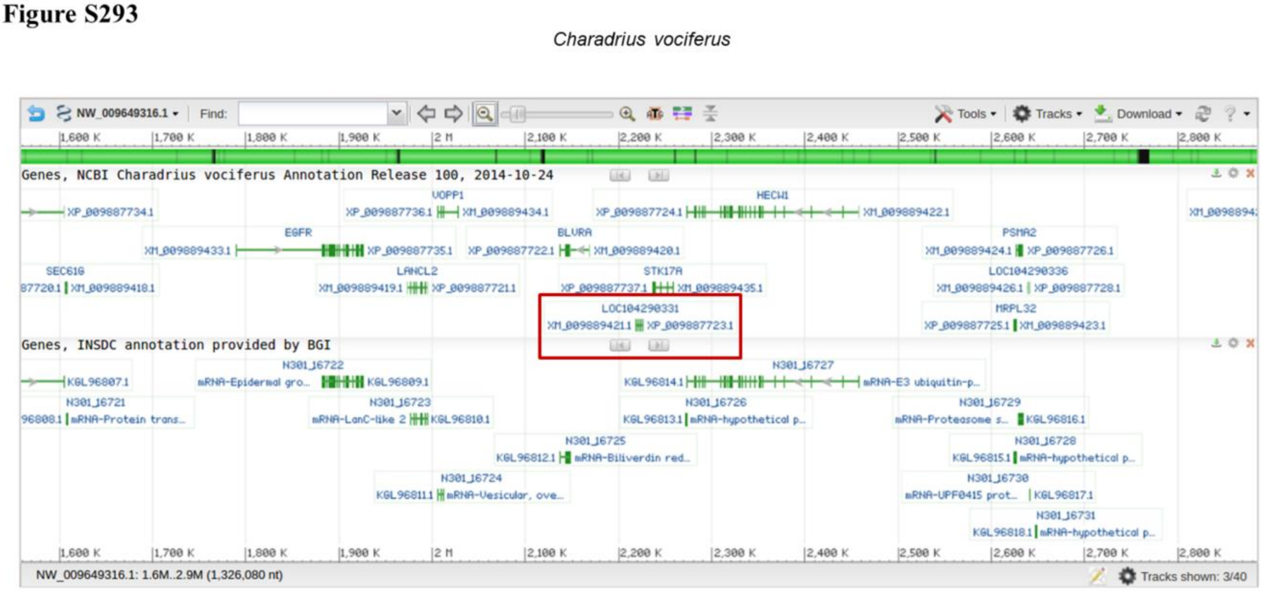

Figure S293: Gene order near *COA1* gene in the killdeer genome. The rectangular boxes connected by thin lines depict genes, with the boxes denoting exons and connecting lines being introns. Direction of transcription is denoted by the > or < symbols (signify positive and negative strand of transcription respectively) beside the gene names. chromosome bands and the genomic coordinates in kilobase (kb) are shown in the topmost row. Details of the contig and scaffold identity are provided in the blue bar located in the middle of the figure. Exact location of the *COA1* gene is depicted by a red box.

Figure S294

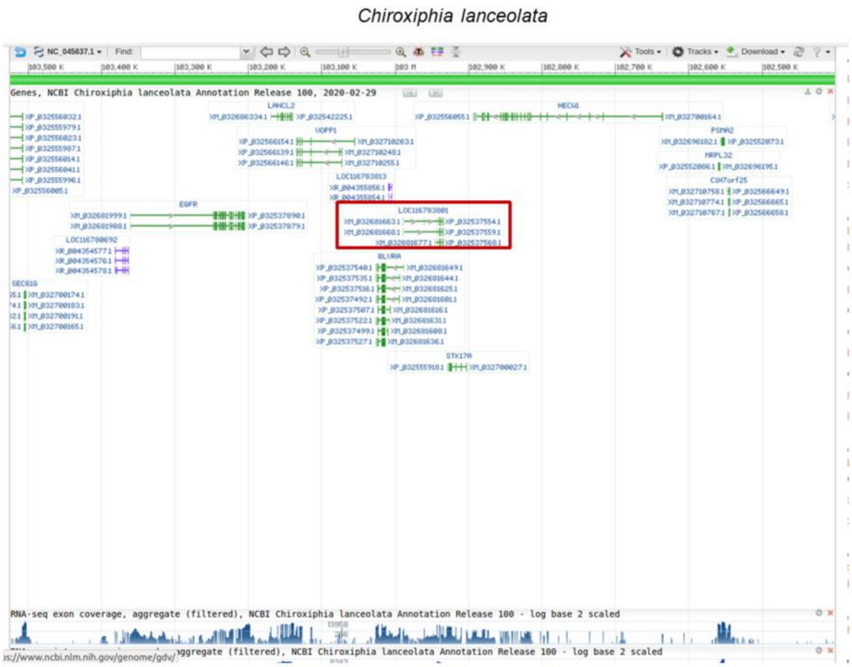

Figure S294: Gene order near *COA1* gene in the lance-tailed manakin genome. The rectangular boxes connected by thin lines depict genes, with the boxes denoting exons and connecting lines being introns. Direction of transcription is denoted by the > or < symbols (signify positive and negative strand of transcription respectively) beside the gene names. chromosome bands and the genomic coordinates in kilobase (kb) are shown in the topmost row. Details of the contig and scaffold identity are provided in the blue bar located in the middle of the figure. Exact location of the *COA1* gene is depicted by a red box.

Figure S295

Chlamydotis macqueenii

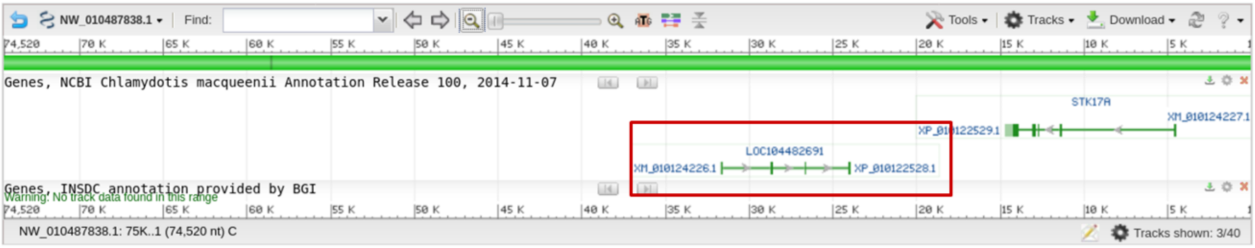

Figure S295: Gene order near *COA1* gene in the MacQueen's bustard genome. The rectangular boxes connected by thin lines depict genes, with the boxes denoting exons and connecting lines being introns. Direction of transcription is denoted by the > or < symbols (signify positive and negative strand of transcription respectively) beside the gene names. chromosome bands and the genomic coordinates in kilobase (kb) are shown in the topmost row. Details of the contig and scaffold identity are provided in the blue bar located in the middle of the figure. Exact location of the *COA1* gene is depicted by a red box.

Figure S296

Colius striatus

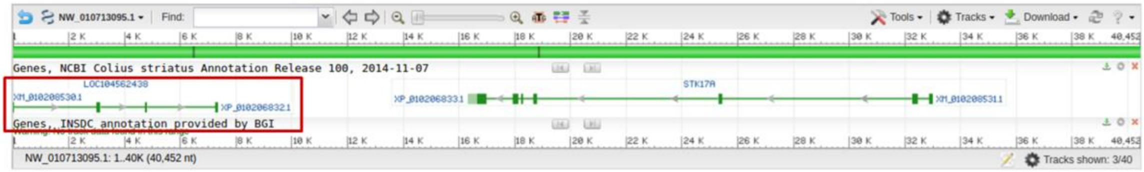

Figure S296: Gene order near *COA1* gene in the speckled mousebird genome. The rectangular boxes connected by thin lines depict genes, with the boxes denoting exons and connecting lines being introns. Direction of transcription is denoted by the > or < symbols (signify positive and negative strand of transcription respectively) beside the gene names. chromosome bands and the genomic coordinates in kilobase (kb) are shown in the topmost row. Details of the contig and scaffold identity are provided in the blue bar located in the middle of the figure. Exact location of the *COA1* gene is depicted by a red box.

Figure S297

Columba livia

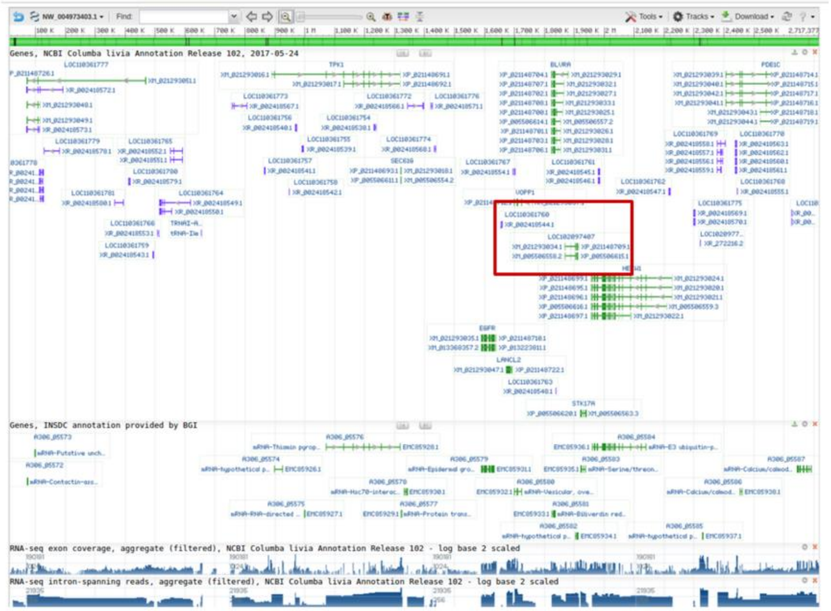

Figure S297: Gene order near *COA1* gene in the rock pigeon genome. The rectangular boxes connected by thin lines depict genes, with the boxes denoting exons and connecting lines being introns. Direction of transcription is denoted by the > or < symbols (signify positive and negative strand of transcription respectively) beside the gene names. chromosome bands and the genomic coordinates in kilobase (kb) are shown in the topmost row. Details of the contig and scaffold identity are provided in the blue bar located in the middle of the figure. Exact location of the *COA1* gene is depicted by a red box.

Figure S298

Serinus canaria

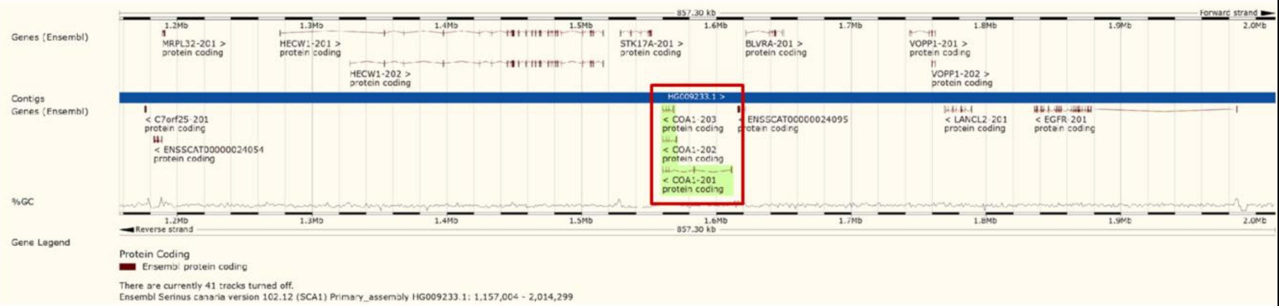

Figure S298: Gene order near *COA1* gene in the common canary genome. The rectangular boxes connected by thin lines depict genes, with the boxes denoting exons and connecting lines being introns. Direction of transcription is denoted by the > or < symbols (signify positive and negative strand of transcription respectively) beside the gene names. chromosome bands and the genomic coordinates in kilobase (kb) are shown in the topmost row. Details of the contig and scaffold identity are provided in the blue bar located in the middle of the figure. Exact location of the *COA1* gene is depicted by a red box.

Figure S299

Falco tinnunculus

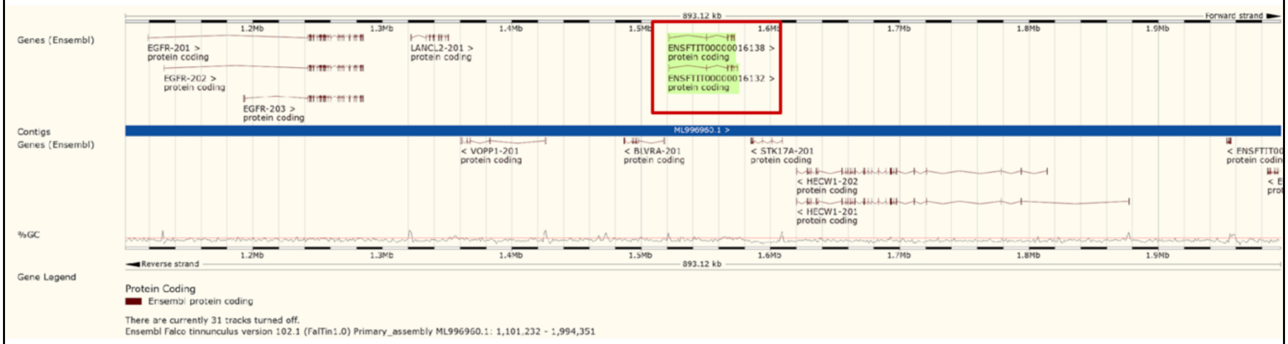

Figure S299: Gene order near *COA1* gene in the common kestrel genome. The rectangular boxes connected by thin lines depict genes, with the boxes denoting exons and connecting lines being introns. Direction of transcription is denoted by the > or < symbols (signify positive and negative strand of transcription respectively) beside the gene names. chromosome bands and the genomic coordinates in kilobase (kb) are shown in the topmost row. Details of the contig and scaffold identity are provided in the blue bar located in the middle of the figure. Exact location of the *COA1* gene is depicted by a red box.

Figure S300

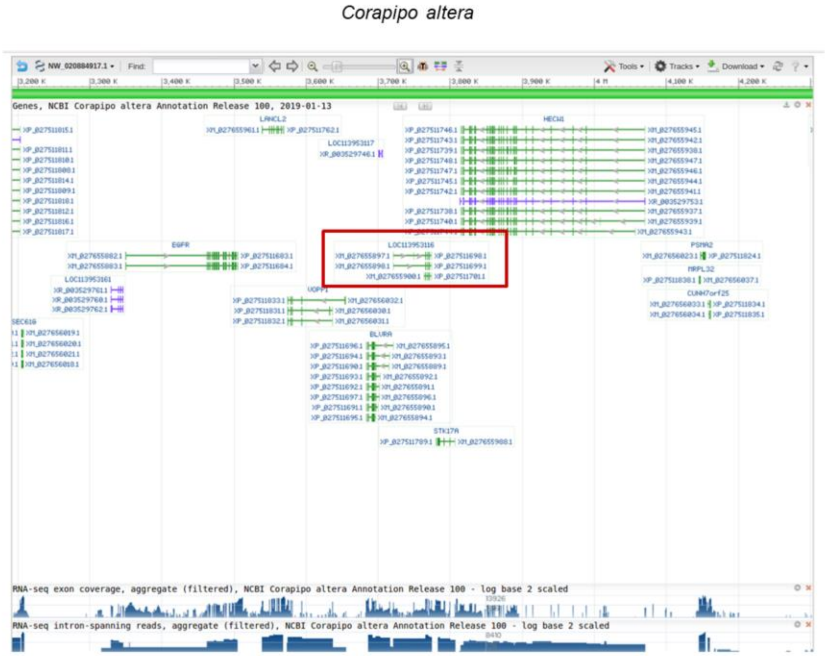

Figure S300: Gene order near *COA1* gene in the white-ruffed manakin genome. The rectangular boxes connected by thin lines depict genes, with the boxes denoting exons and connecting lines being introns. Direction of transcription is denoted by the > or < symbols (signify positive and negative strand of transcription respectively) beside the gene names. chromosome bands and the genomic coordinates in kilobase (kb) are shown in the topmost row. Details of the contig and scaffold identity are provided in the blue bar located in the middle of the figure. Exact location of the *COA1* gene is depicted by a red box.

Figure S301

Corvus brachyrhynchos

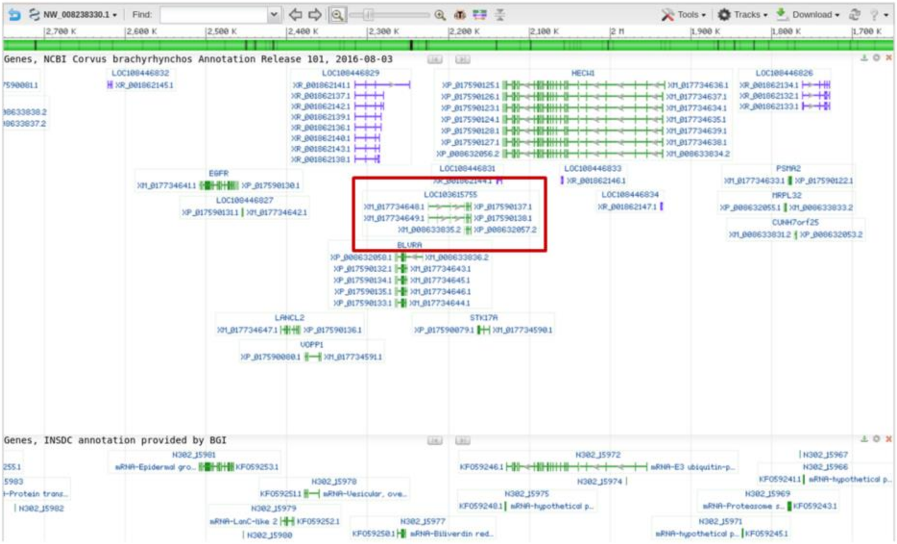

Figure S301: Gene order near *COA1* gene in the American crow genome. The rectangular boxes connected by thin lines depict genes, with the boxes denoting exons and connecting lines being introns. Direction of transcription is denoted by the > or < symbols (signify positive and negative strand of transcription respectively) beside the gene names. chromosome bands and the genomic coordinates in kilobase (kb) are shown in the topmost row. Details of the contig and scaffold identity are provided in the blue bar located in the middle of the figure. Exact location of the *COA1* gene is depicted by a red box.

Figure S302

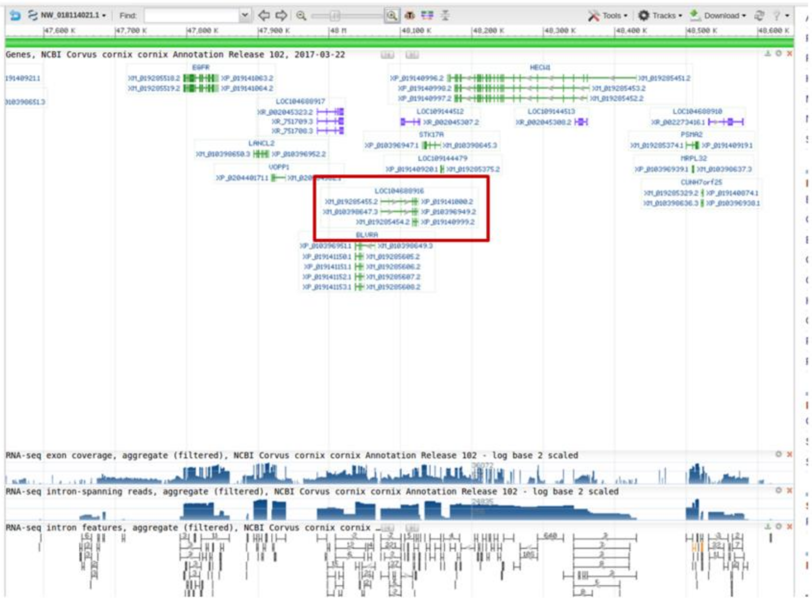

Figure S302: Gene order near *COA1* gene in the hooded crow genome. The rectangular boxes connected by thin lines depict genes, with the boxes denoting exons and connecting lines being introns. Direction of transcription is denoted by the > or < symbols (signify positive and negative strand of transcription respectively) beside the gene names. chromosome bands and the genomic coordinates in kilobase (kb) are shown in the topmost row. Details of the contig and scaffold identity are provided in the blue bar located in the middle of the figure. Exact location of the *COA1* gene is depicted by a red box.

Figure S303

Cuculus canorus

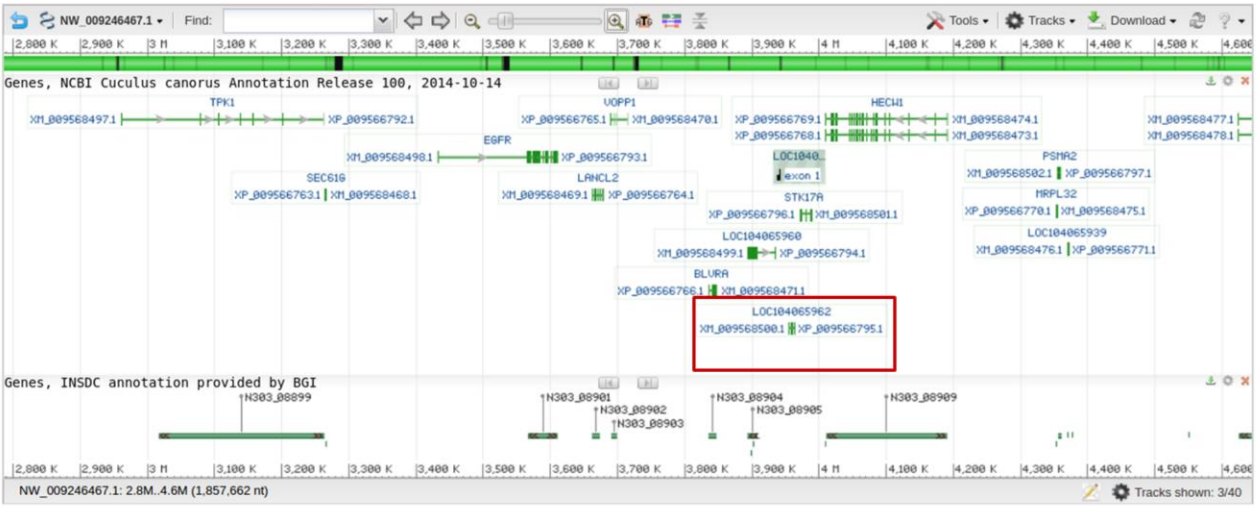

Figure S303: Gene order near *COA1* gene in the common cuckoo genome. The rectangular boxes connected by thin lines depict genes, with the boxes denoting exons and connecting lines being introns. Direction of transcription is denoted by the > or < symbols (signify positive and negative strand of transcription respectively) beside the gene names. chromosome bands and the genomic coordinates in kilobase (kb) are shown in the topmost row. Details of the contig and scaffold identity are provided in the blue bar located in the middle of the figure. Exact location of the *COA1* gene is depicted by a red box.

Figure S304

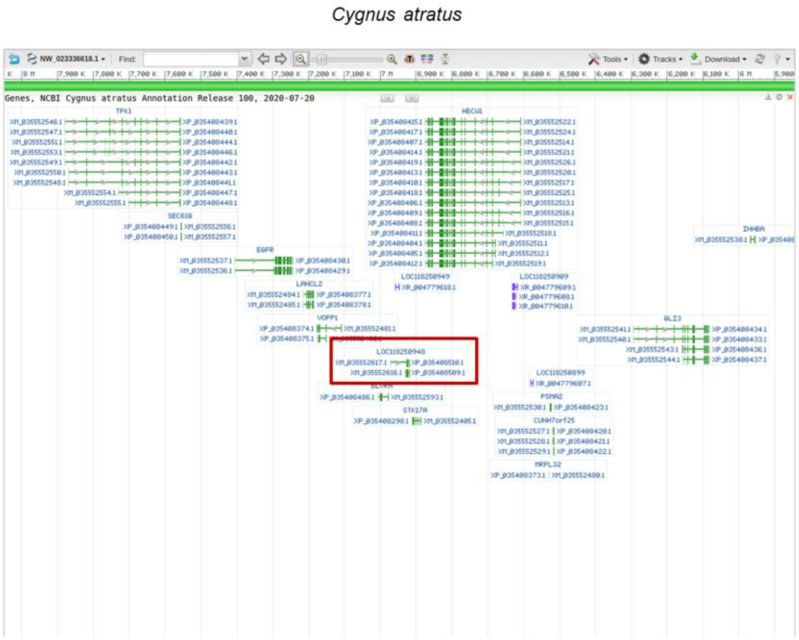

Figure S304: Gene order near *COA1* gene in the black swan genome. The rectangular boxes connected by thin lines depict genes, with the boxes denoting exons and connecting lines being introns. Direction of transcription is denoted by the > or < symbols (signify positive and negative strand of transcription respectively) beside the gene names. chromosome bands and the genomic coordinates in kilobase (kb) are shown in the topmost row. Details of the contig and scaffold identity are provided in the blue bar located in the middle of the figure. Exact location of the *COA1* gene is depicted by a red box.

Figure S305

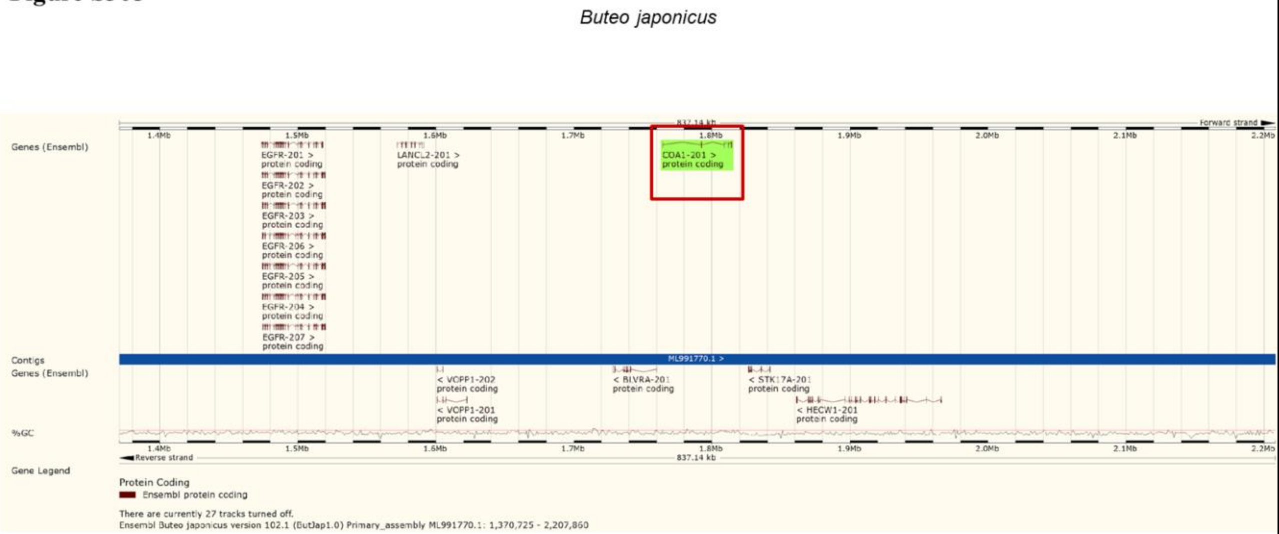

Figure S305: Gene order near *COA1* gene in the eastern buzzard genome. The rectangular boxes connected by thin lines depict genes, with the boxes denoting exons and connecting lines being introns. Direction of transcription is denoted by the > or < symbols (signify positive and negative strand of transcription respectively) beside the gene names. chromosome bands and the genomic coordinates in kilobase (kb) are shown in the topmost row. Details of the contig and scaffold identity are provided in the blue bar located in the middle of the figure. Exact location of the *COA1* gene is depicted by a red box.

Figure S306

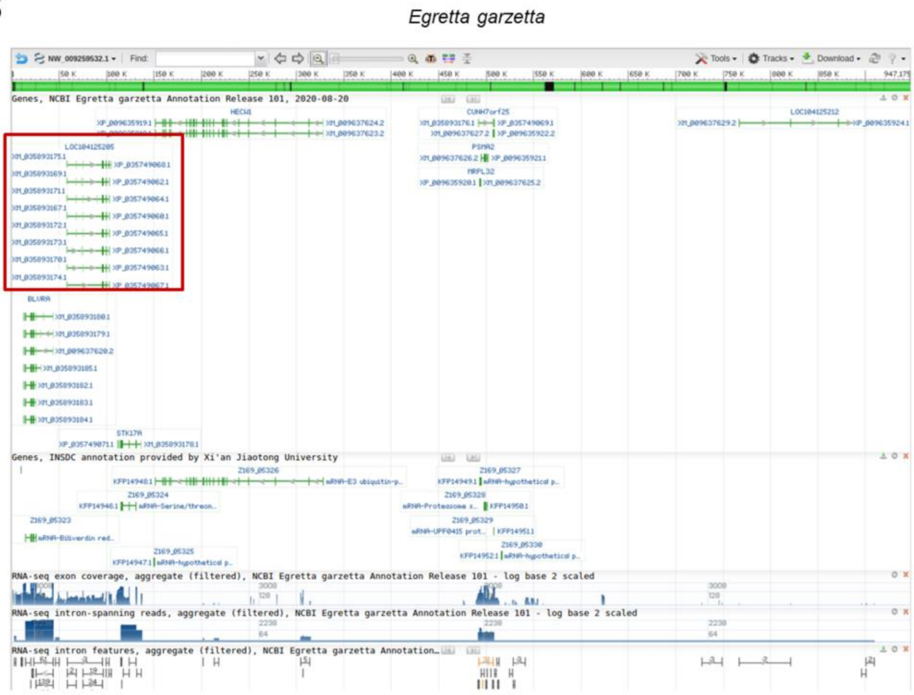

Figure S306: Gene order near *COA1* gene in the little egret genome. The rectangular boxes connected by thin lines depict genes, with the boxes denoting exons and connecting lines being introns. Direction of transcription is denoted by the > or < symbols (signify positive and negative strand of transcription respectively) beside the gene names. chromosome bands and the genomic coordinates in kilobase (kb) are shown in the topmost row. Details of the contig and scaffold identity are provided in the blue bar located in the middle of the figure. Exact location of the *COA1* gene is depicted by a red box.

Figure S307

Empidonax traillii

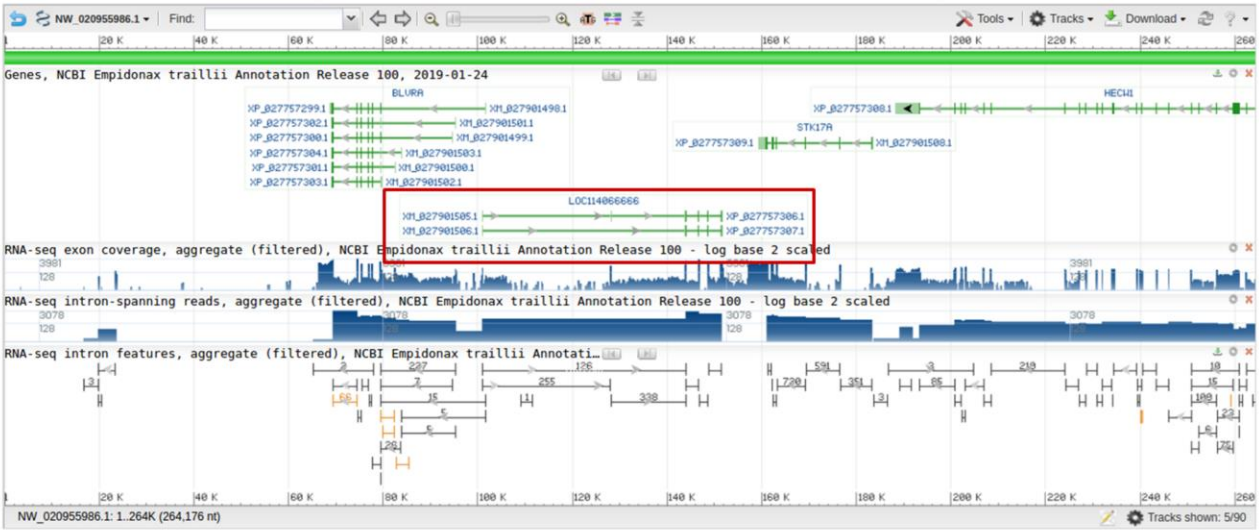

Figure S307: Gene order near *COA1* gene in the willow flycatcher genome. The rectangular boxes connected by thin lines depict genes, with the boxes denoting exons and connecting lines being introns. Direction of transcription is denoted by the > or < symbols (signify positive and negative strand of transcription respectively) beside the gene names. chromosome bands and the genomic coordinates in kilobase (kb) are shown in the topmost row. Details of the contig and scaffold identity are provided in the blue bar located in the middle of the figure. Exact location of the *COA1* gene is depicted by a red box.

Figure S308

Eurypyga helias

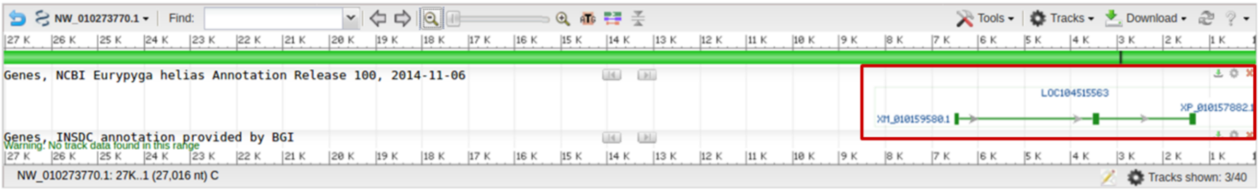

Figure S308: Gene order near *COA1* gene in the sunbittern genome. The rectangular boxes connected by thin lines depict genes, with the boxes denoting exons and connecting lines being introns. Direction of transcription is denoted by the > or < symbols (signify positive and negative strand of transcription respectively) beside the gene names. chromosome bands and the genomic coordinates in kilobase (kb) are shown in the topmost row. Details of the contig and scaffold identity are provided in the blue bar located in the middle of the figure. Exact location of the *COA1* gene is depicted by a red box.

Figure S309

Falco cherrug

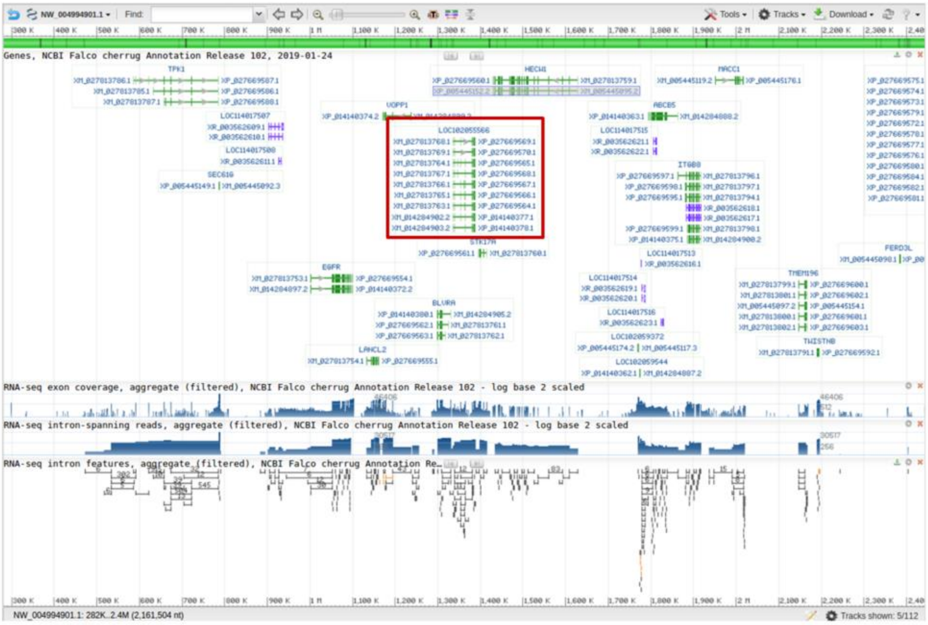

Figure S309: Gene order near *COA1* gene in the saker falcon genome. The rectangular boxes connected by thin lines depict genes, with the boxes denoting exons and connecting lines being introns. Direction of transcription is denoted by the > or < symbols (signify positive and negative strand of transcription respectively) beside the gene names. chromosome bands and the genomic coordinates in kilobase (kb) are shown in the topmost row. Details of the contig and scaffold identity are provided in the blue bar located in the middle of the figure. Exact location of the *COA1* gene is depicted by a red box.

Figure S310

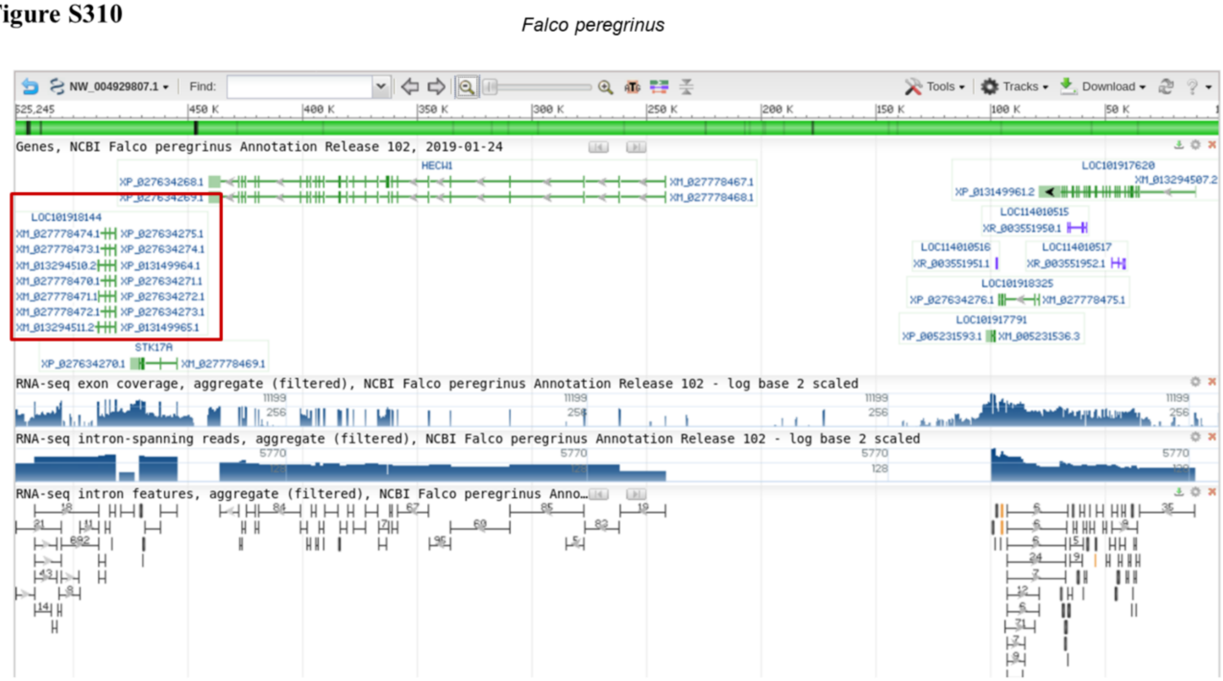

Figure S310: Gene order near *COA1* gene in the peregrine falcon genome. The rectangular boxes connected by thin lines depict genes, with the boxes denoting exons and connecting lines being introns. Direction of transcription is denoted by the > or < symbols (signify positive and negative strand of transcription respectively) beside the gene names. chromosome bands and the genomic coordinates in kilobase (kb) are shown in the topmost row. Details of the contig and scaffold identity are provided in the blue bar located in the middle of the figure. Exact location of the *COA1* gene is depicted by a red box.

Figure S311

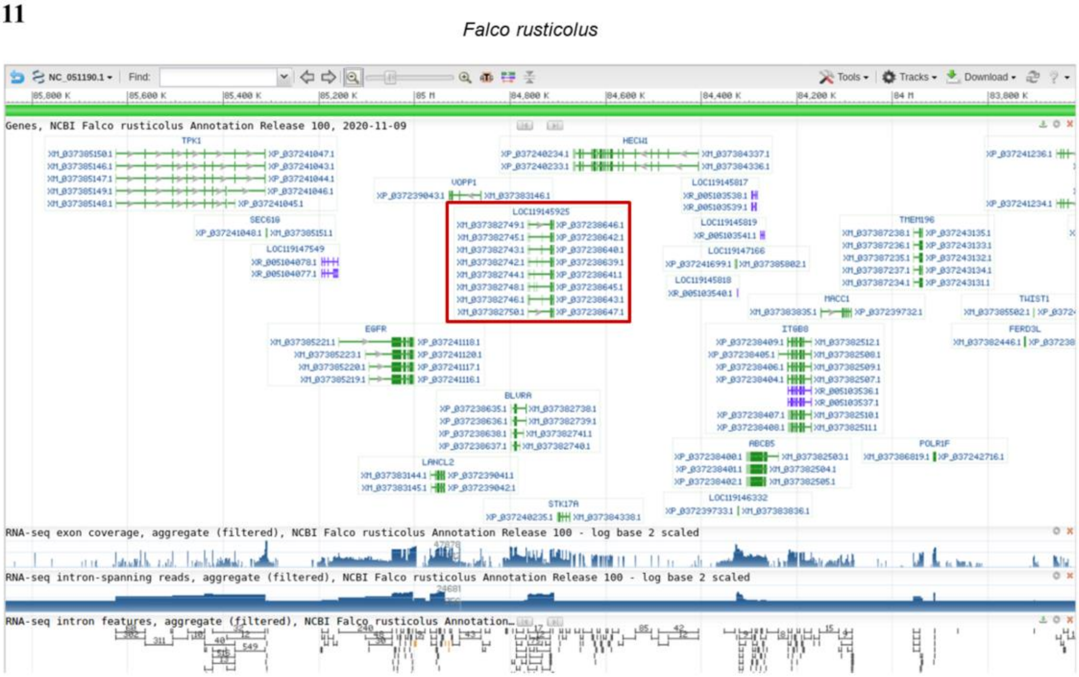

Figure S311: Gene order near *COA1* gene in the gyrfalcon genome. The rectangular boxes connected by thin lines depict genes, with the boxes denoting exons and connecting lines being introns. Direction of transcription is denoted by the > or < symbols (signify positive and negative strand of transcription respectively) beside the gene names. chromosome bands and the genomic coordinates in kilobase (kb) are shown in the topmost row. Details of the contig and scaffold identity are provided in the blue bar located in the middle of the figure. Exact location of the *COA1* gene is depicted by a red box.

Figure S312

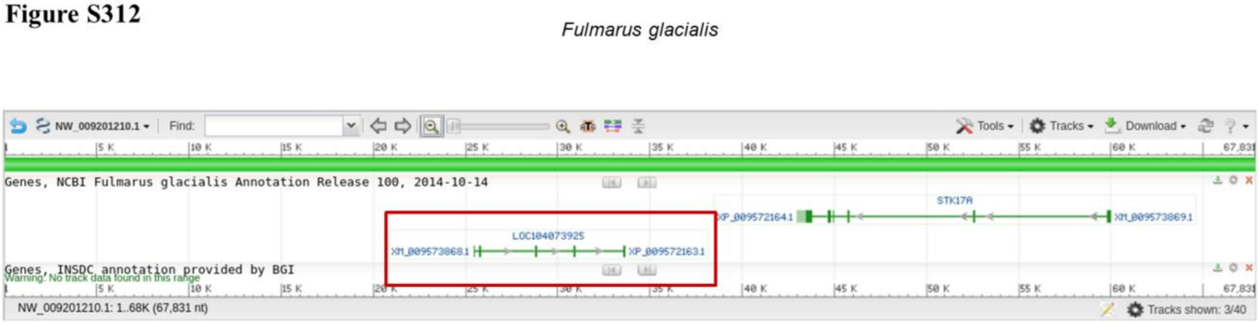

Figure S312: Gene order near *COA1* gene in the Northern fulmar genome. The rectangular boxes connected by thin lines depict genes, with the boxes denoting exons and connecting lines being introns. Direction of transcription is denoted by the > or < symbols (signify positive and negative strand of transcription respectively) beside the gene names. chromosome bands and the genomic coordinates in kilobase (kb) are shown in the topmost row. Details of the contig and scaffold identity are provided in the blue bar located in the middle of the figure. Exact location of the *COA1* gene is depicted by a red box.

Figure S313

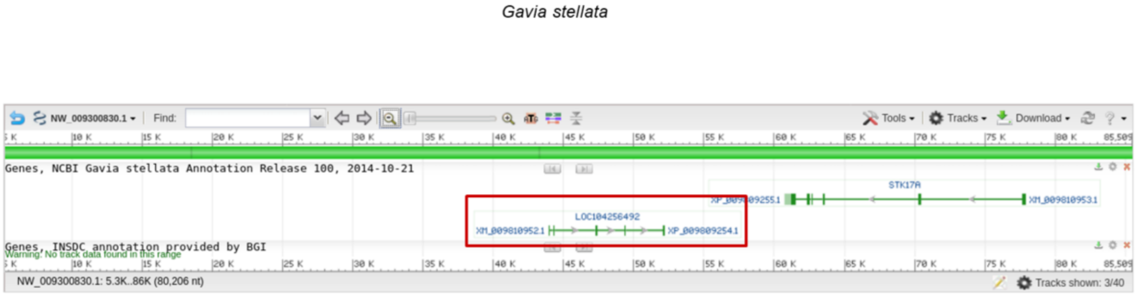

Figure S313: Gene order near *COA1* gene in the red-throated loon genome. The rectangular boxes connected by thin lines depict genes, with the boxes denoting exons and connecting lines being introns. Direction of transcription is denoted by the > or < symbols (signify positive and negative strand of transcription respectively) beside the gene names. chromosome bands and the genomic coordinates in kilobase (kb) are shown in the topmost row. Details of the contig and scaffold identity are provided in the blue bar located in the middle of the figure. Exact location of the *COA1* gene is depicted by a red box.

Figure S314

Haliaeetus albicilla

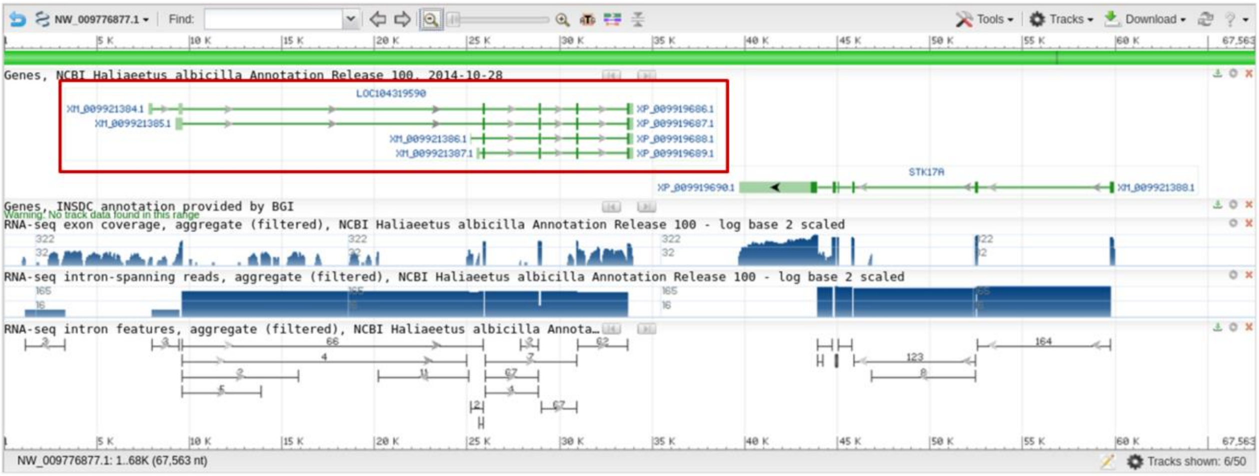

Figure S314: Gene order near *COA1* gene in the white-tailed eagle genome. The rectangular boxes connected by thin lines depict genes, with the boxes denoting exons and connecting lines being introns. Direction of transcription is denoted by the > or < symbols (signify positive and negative strand of transcription respectively) beside the gene names. chromosome bands and the genomic coordinates in kilobase (kb) are shown in the topmost row. Details of the contig and scaffold identity are provided in the blue bar located in the middle of the figure. Exact location of the *COA1* gene is depicted by a red box.

Figure S315

Haliaeetus leucocephalus

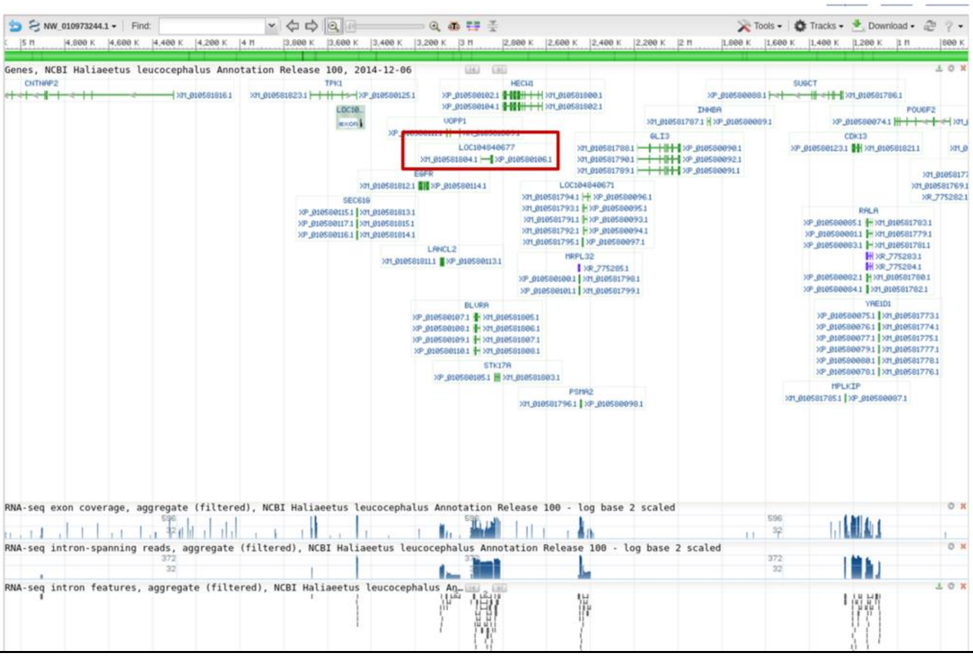

Figure S315: Gene order near *COA1* gene in the bald eagle genome. The rectangular boxes connected by thin lines depict genes, with the boxes denoting exons and connecting lines being introns. Direction of transcription is denoted by the > or < symbols (signify positive and negative strand of transcription respectively) beside the gene names. chromosome bands and the genomic coordinates in kilobase (kb) are shown in the topmost row. Details of the contig and scaffold identity are provided in the blue bar located in the middle of the figure. Exact location of the *COA1* gene is depicted by a red box.

Figure S316

Melopsittacus undulatus

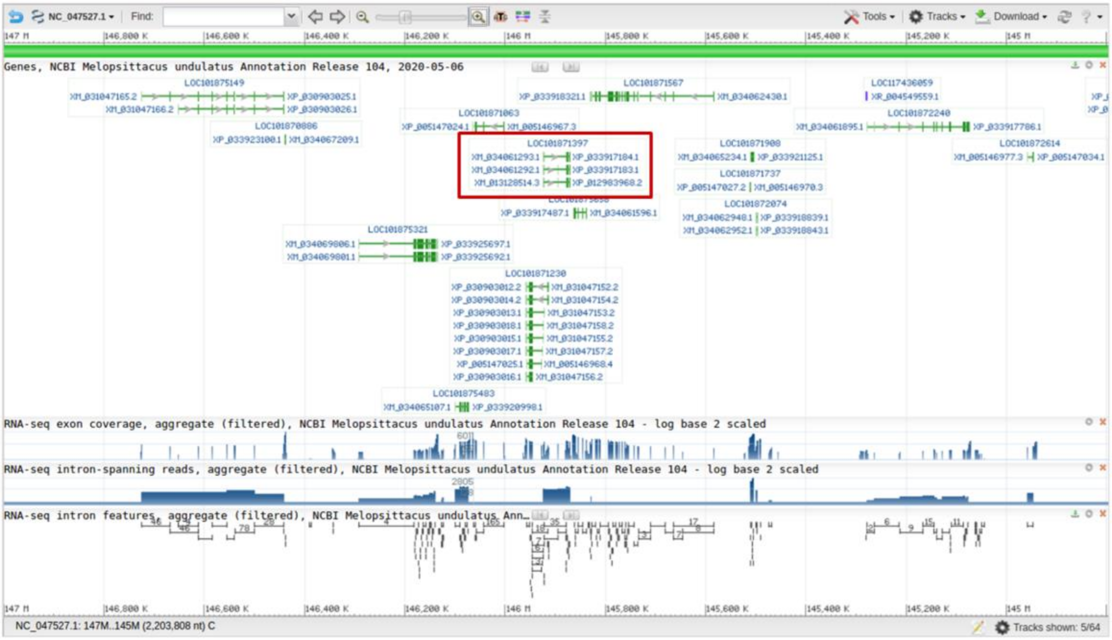

Figure S316: Gene order near *COA1* gene in the budgerigar genome. The rectangular boxes connected by thin lines depict genes, with the boxes denoting exons and connecting lines being introns. Direction of transcription is denoted by the > or < symbols (signify positive and negative strand of transcription respectively) beside the gene names. chromosome bands and the genomic coordinates in kilobase (kb) are shown in the topmost row. Details of the contig and scaffold identity are provided in the blue bar located in the middle of the figure. Exact location of the *COA1* gene is depicted by a red box.

Figure S317

Merops nubicus

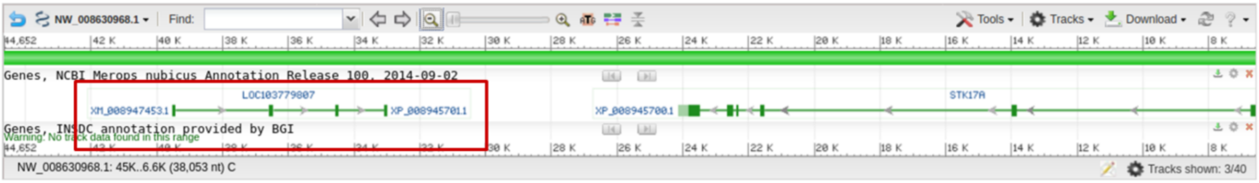

Figure S317: Gene order near *COA1* gene in the carmine bee-eater genome. The rectangular boxes connected by thin lines depict genes, with the boxes denoting exons and connecting lines being introns. Direction of transcription is denoted by the > or < symbols (signify positive and negative strand of transcription respectively) beside the gene names. chromosome bands and the genomic coordinates in kilobase (kb) are shown in the topmost row. Details of the contig and scaffold identity are provided in the blue bar located in the middle of the figure. Exact location of the *COA1* gene is depicted by a red box.

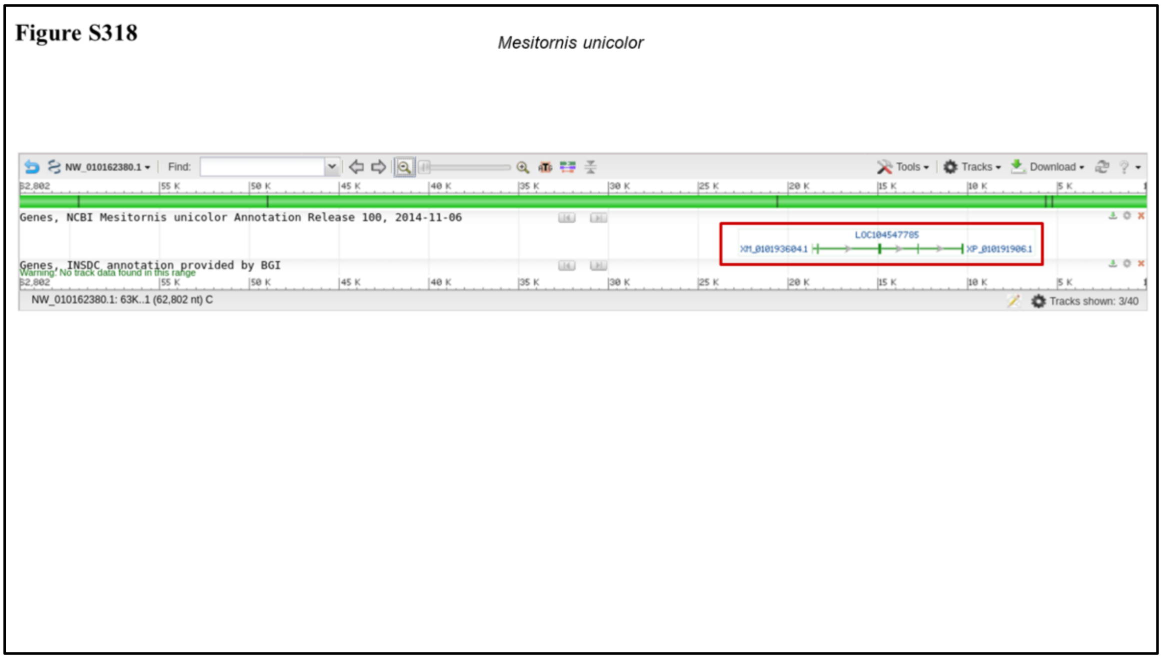

Figure S318: Gene order near *COA1* gene in the brown roatelo genome. The rectangular boxes connected by thin lines depict genes, with the boxes denoting exons and connecting lines being introns. Direction of transcription is denoted by the > or < symbols (signify positive and negative strand of transcription respectively) beside the gene names. chromosome bands and the genomic coordinates in kilobase (kb) are shown in the topmost row. Details of the contig and scaffold identity are provided in the blue bar located in the middle of the figure. Exact location of the *COA1* gene is depicted by a red box.

Figure S319

Molothrus ater

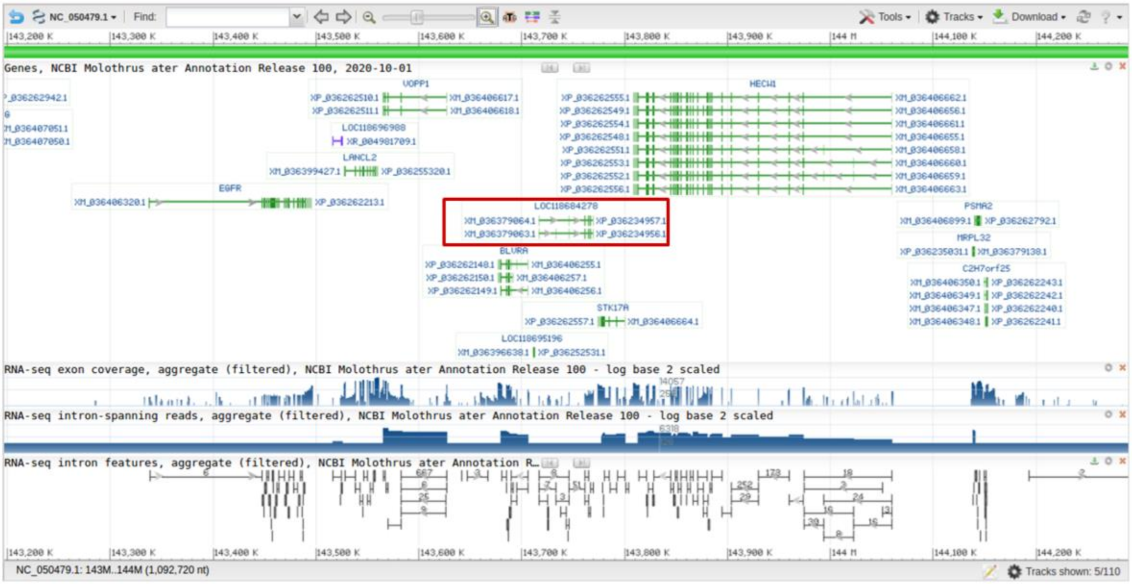

Figure S319: Gene order near *COA1* gene in the brown-headed cowbird genome. The rectangular boxes connected by thin lines depict genes, with the boxes denoting exons and connecting lines being introns. Direction of transcription is denoted by the > or < symbols (signify positive and negative strand of transcription respectively) beside the gene names. chromosome bands and the genomic coordinates in kilobase (kb) are shown in the topmost row. Details of the contig and scaffold identity are provided in the blue bar located in the middle of the figure. Exact location of the *COA1* gene is depicted by a red box.

Figure S320

Nestor notabilis

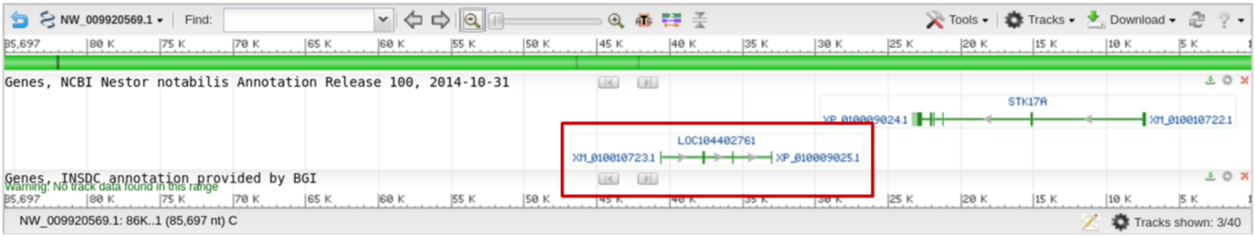

Figure S320: Gene order near *COA1* gene in the kea genome. The rectangular boxes connected by thin lines depict genes, with the boxes denoting exons and connecting lines being introns. Direction of transcription is denoted by the > or < symbols (signify positive and negative strand of transcription respectively) beside the gene names. chromosome bands and the genomic coordinates in kilobase (kb) are shown in the topmost row. Details of the contig and scaffold identity are provided in the blue bar located in the middle of the figure. Exact location of the *COA1* gene is depicted by a red box.

Figure S321

Nipponia nippon

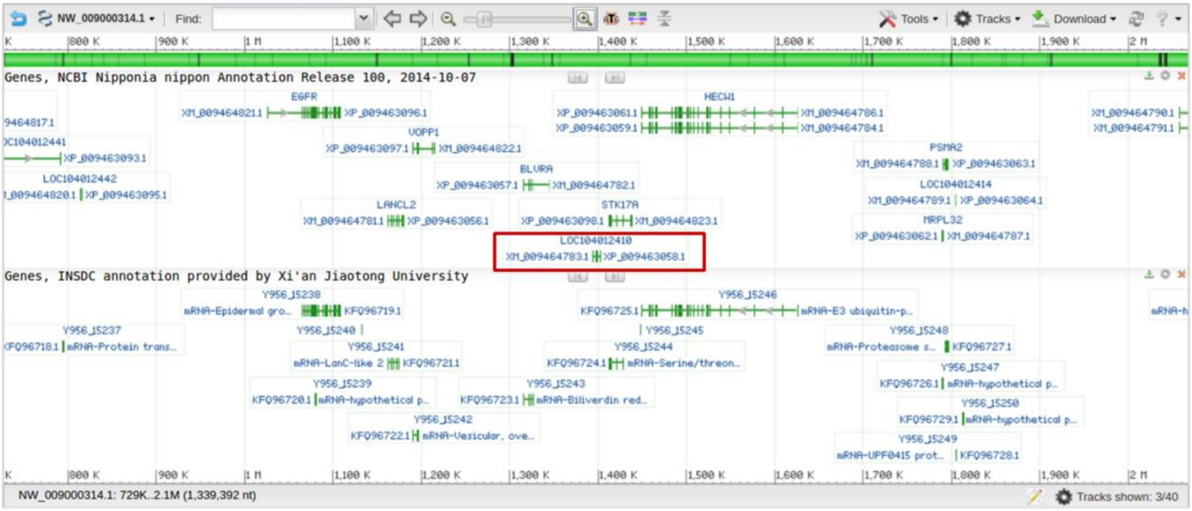

Figure S321: Gene order near *COA1* gene in the crested ibis genome. The rectangular boxes connected by thin lines depict genes, with the boxes denoting exons and connecting lines being introns. Direction of transcription is denoted by the > or < symbols (signify positive and negative strand of transcription respectively) beside the gene names. chromosome bands and the genomic coordinates in kilobase (kb) are shown in the topmost row. Details of the contig and scaffold identity are provided in the blue bar located in the middle of the figure. Exact location of the *COA1* gene is depicted by a red box.

Figure S322

Opisthocomus hoazin

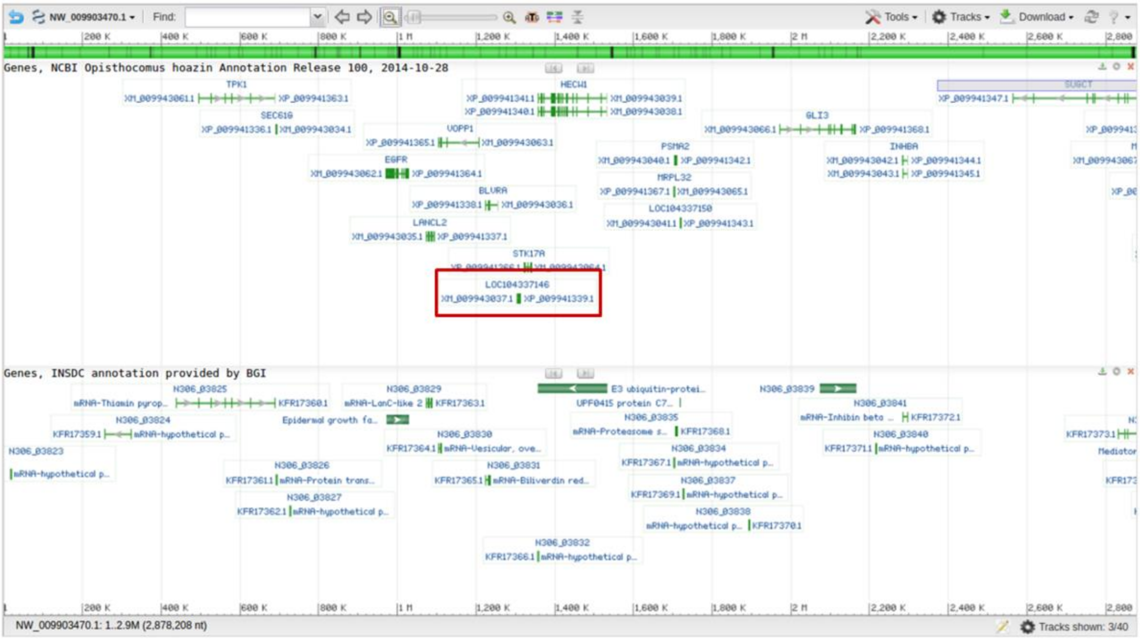

Figure S322: Gene order near *COA1* gene in the hoatzin genome. The rectangular boxes connected by thin lines depict genes, with the boxes denoting exons and connecting lines being introns. Direction of transcription is denoted by the > or < symbols (signify positive and negative strand of transcription respectively) beside the gene names. chromosome bands and the genomic coordinates in kilobase (kb) are shown in the topmost row. Details of the contig and scaffold identity are provided in the blue bar located in the middle of the figure. Exact location of the *COA1* gene is depicted by a red box.

Figure S323

Phaethon lepturus

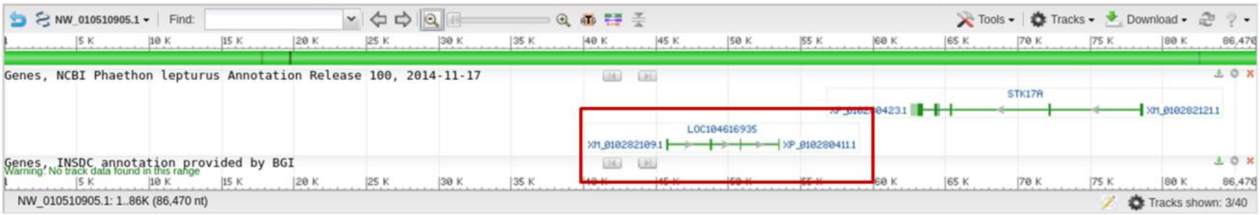

Figure S323: Gene order near *COA1* gene in the white-tailed tropicbird genome. The rectangular boxes connected by thin lines depict genes, with the boxes denoting exons and connecting lines being introns. Direction of transcription is denoted by the > or < symbols (signify positive and negative strand of transcription respectively) beside the gene names. chromosome bands and the genomic coordinates in kilobase (kb) are shown in the topmost row. Details of the contig and scaffold identity are provided in the blue bar located in the middle of the figure. Exact location of the *COA1* gene is depicted by a red box.

Figure S324

Phalacrocorax carbo

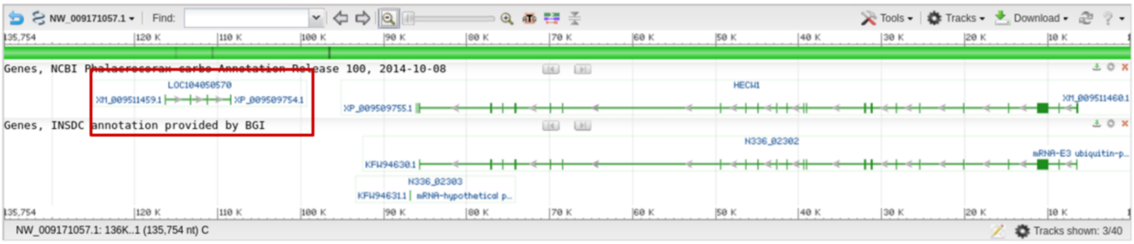

Figure S324: Gene order near *COA1* gene in the great cormorant genome. The rectangular boxes connected by thin lines depict genes, with the boxes denoting exons and connecting lines being introns. Direction of transcription is denoted by the > or < symbols (signify positive and negative strand of transcription respectively) beside the gene names. chromosome bands and the genomic coordinates in kilobase (kb) are shown in the topmost row. Details of the contig and scaffold identity are provided in the blue bar located in the middle of the figure. Exact location of the *COA1* gene is depicted by a red box.

Figure S325

Picoides pubescens

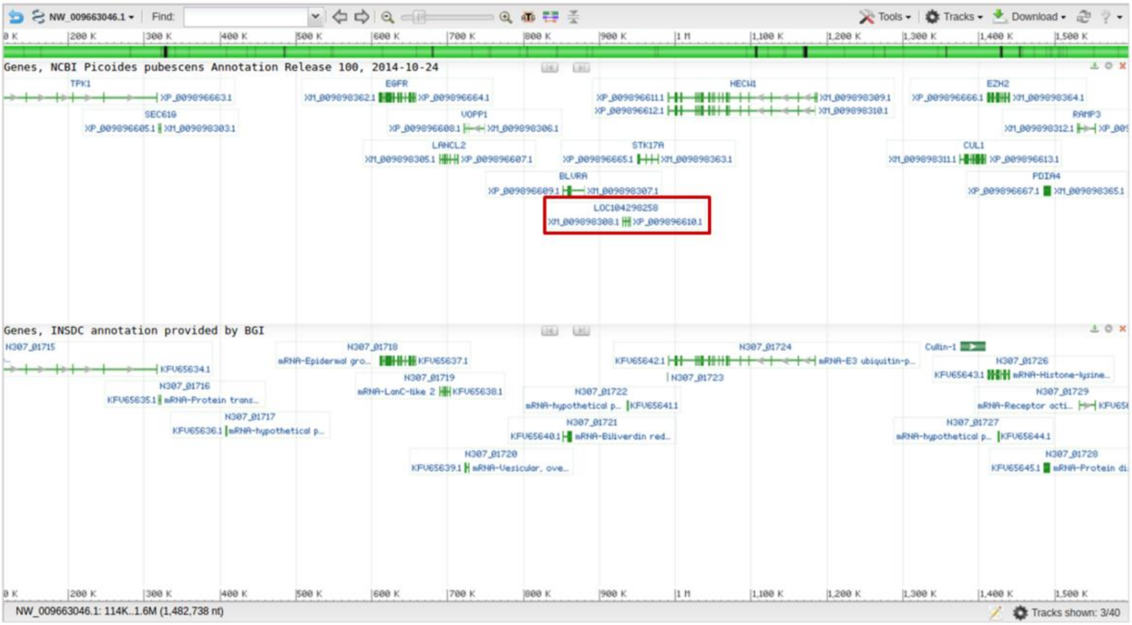

Figure S325: Gene order near *COA1* gene in the downy woodpeeker genome. The rectangular boxes connected by thin lines depict genes, with the boxes denoting exons and connecting lines being introns. Direction of transcription is denoted by the > or < symbols (signify positive and negative strand of transcription respectively) beside the gene names. chromosome bands and the genomic coordinates in kilobase (kb) are shown in the topmost row. Details of the contig and scaffold identity are provided in the blue bar located in the middle of the figure. Exact location of the *COA1* gene is depicted by a red box.

Figure S326

Pterocles gutturalis

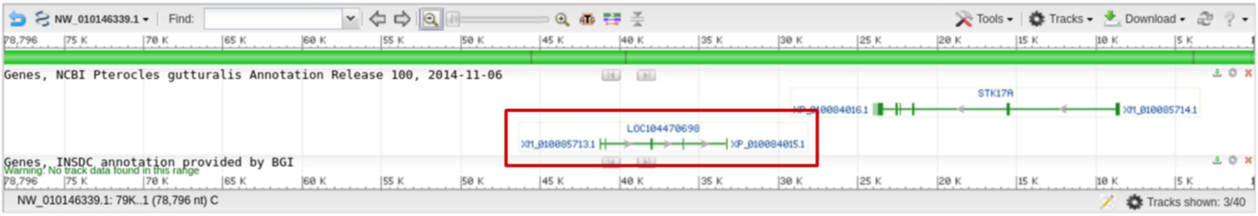

Figure S326: Gene order near *COA1* gene in the yellow-throated sandgrouse genome. The rectangular boxes connected by thin lines depict genes, with the boxes denoting exons and connecting lines being introns. Direction of transcription is denoted by the > or < symbols (signify positive and negative strand of transcription respectively) beside the gene names. chromosome bands and the genomic coordinates in kilobase (kb) are shown in the topmost row. Details of the contig and scaffold identity are provided in the blue bar located in the middle of the figure. Exact location of the *COA1* gene is depicted by a red box.

Figure S327

Pygoscelis adeliae

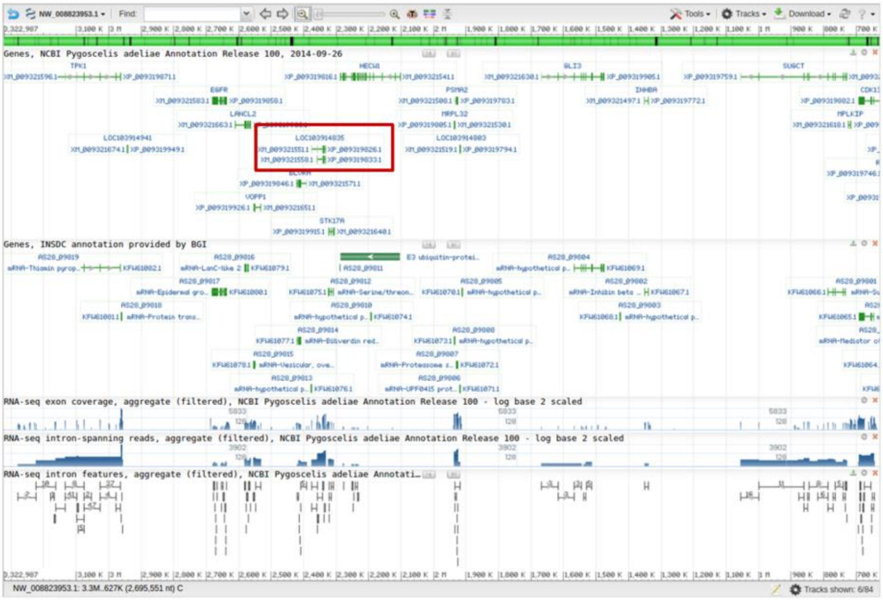

Figure S327: Gene order near *COA1* gene in the Adelie penguin genome. The rectangular boxes connected by thin lines depict genes, with the boxes denoting exons and connecting lines being introns. Direction of transcription is denoted by the > or < symbols (signify positive and negative strand of transcription respectively) beside the gene names. chromosome bands and the genomic coordinates in kilobase (kb) are shown in the topmost row. Details of the contig and scaffold identity are provided in the blue bar located in the middle of the figure. Exact location of the *COA1* gene is depicted by a red box.

Figure S328

Malurus cyaneus samueli

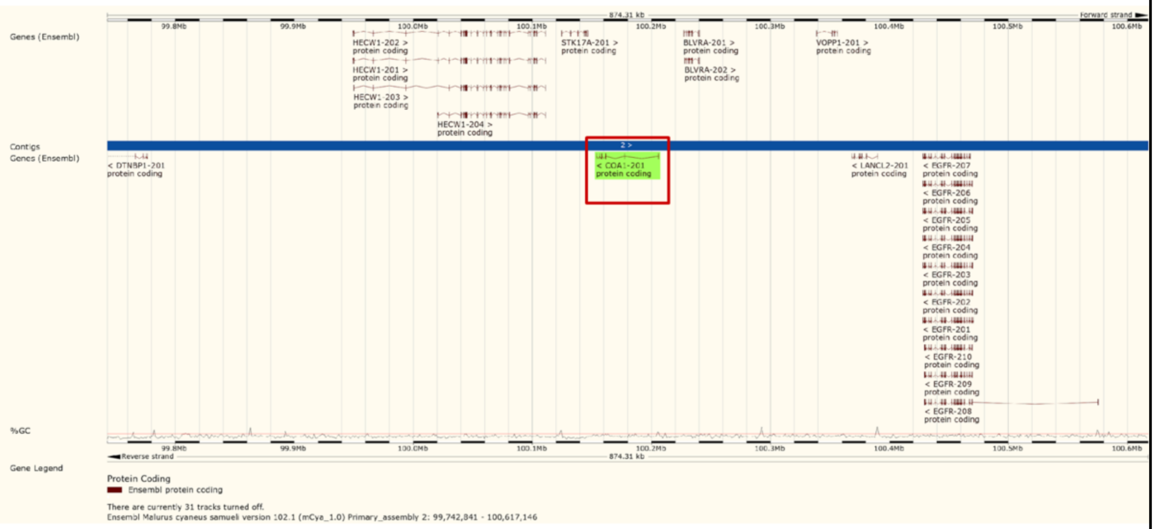

Figure S328: Gene order near *COA1* gene in the superb fairywren genome. The rectangular boxes connected by thin lines depict genes, with the boxes denoting exons and connecting lines being introns. Direction of transcription is denoted by the > or < symbols (signify positive and negative strand of transcription respectively) beside the gene names. chromosome bands and the genomic coordinates in kilobase (kb) are shown in the topmost row. Details of the contig and scaffold identity are provided in the blue bar located in the middle of the figure. Exact location of the *COA1* gene is depicted by a red box.

Figure S329

Catharus ustulatus

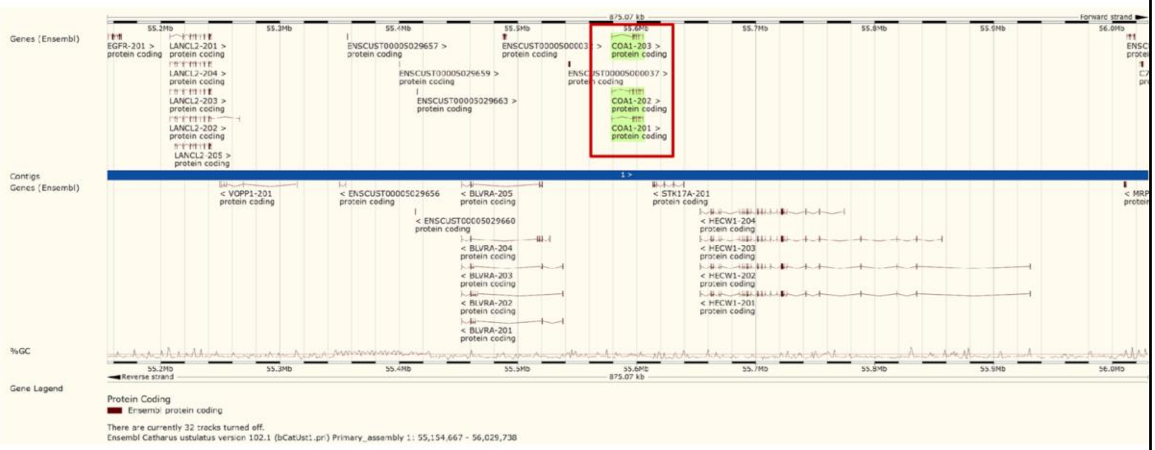

Figure S329: Gene order near *COA1* gene in the Swainson's thrush genome. The rectangular boxes connected by thin lines depict genes, with the boxes denoting exons and connecting lines being introns. Direction of transcription is denoted by the > or < symbols (signify positive and negative strand of transcription respectively) beside the gene names. chromosome bands and the genomic coordinates in kilobase (kb) are shown in the topmost row. Details of the contig and scaffold identity are provided in the blue bar located in the middle of the figure. Exact location of the *COA1* gene is depicted by a red box.

Figure S330

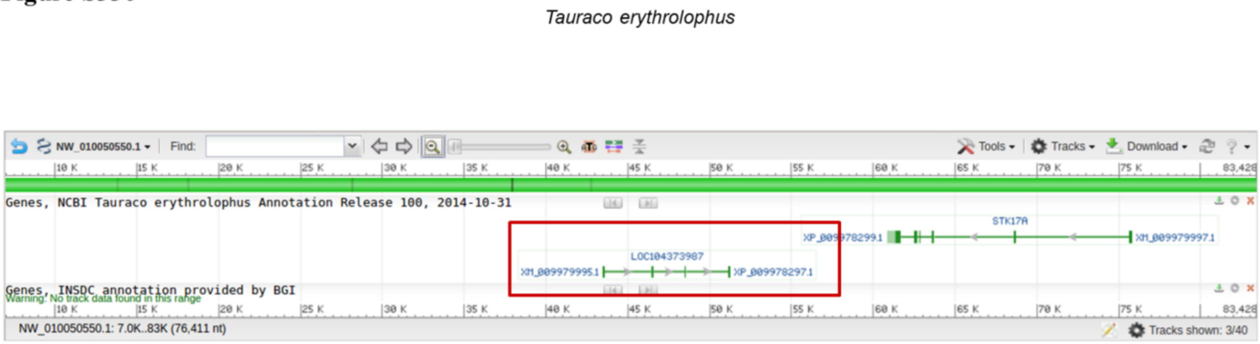

Figure S330: Gene order near *COA1* gene in the red-crested turaco genome. The rectangular boxes connected by thin lines depict genes, with the boxes denoting exons and connecting lines being introns. Direction of transcription is denoted by the > or < symbols (signify positive and negative strand of transcription respectively) beside the gene names. chromosome bands and the genomic coordinates in kilobase (kb) are shown in the topmost row. Details of the contig and scaffold identity are provided in the blue bar located in the middle of the figure. Exact location of the *COA1* gene is depicted by a red box.

Figure S331

Tinamus guttatus

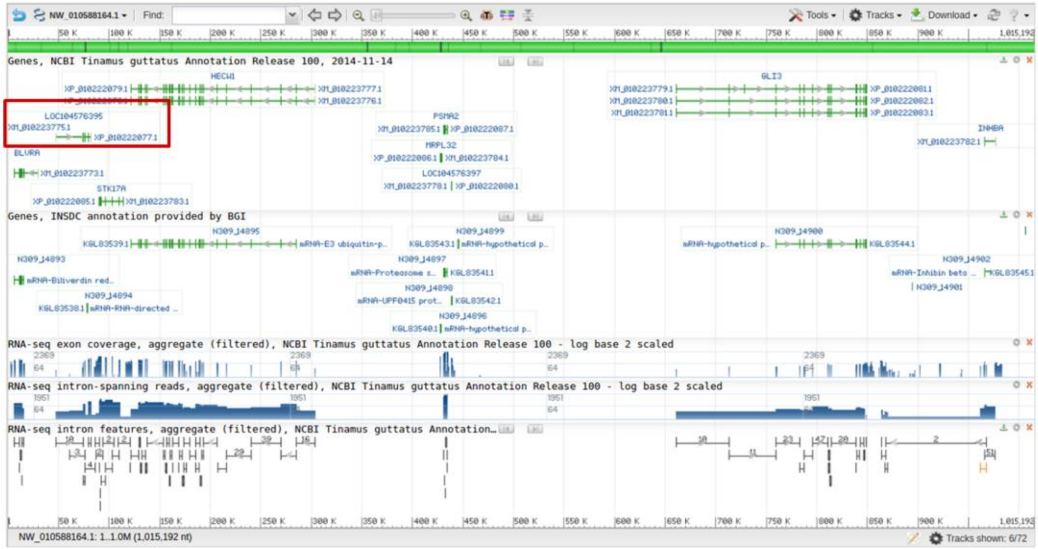

Figure S331: Gene order near *COA1* gene in the white-throated tinamou genome. The rectangular boxes connected by thin lines depict genes, with the boxes denoting exons and connecting lines being introns. Direction of transcription is denoted by the > or < symbols (signify positive and negative strand of transcription respectively) beside the gene names. chromosome bands and the genomic coordinates in kilobase (kb) are shown in the topmost row. Details of the contig and scaffold identity are provided in the blue bar located in the middle of the figure. Exact location of the *COA1* gene is depicted by a red box.

Figure S332

Tyto alba

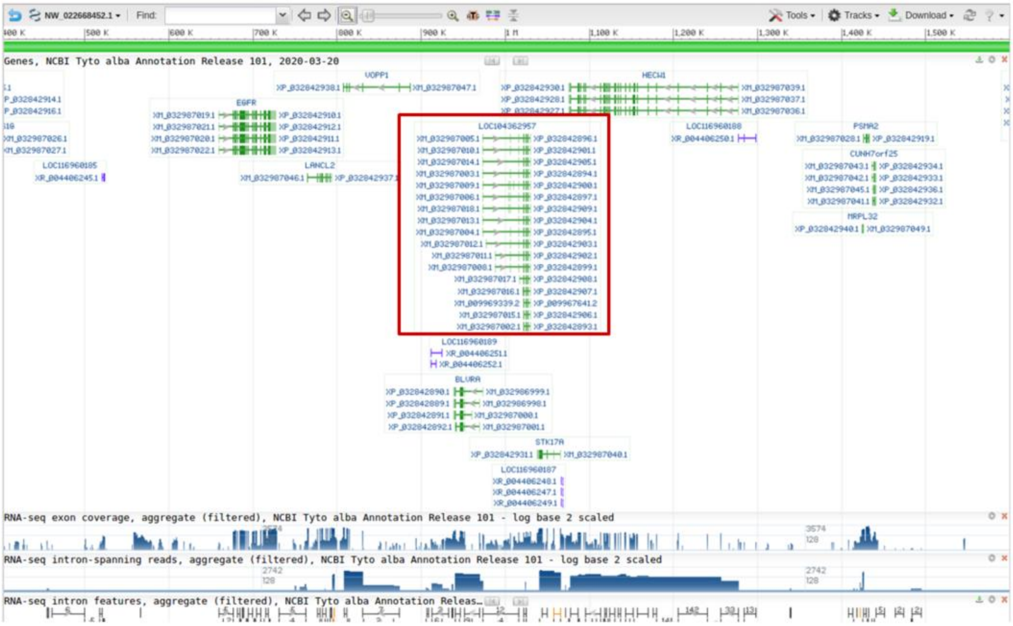

Figure S332: Gene order near *COA1* gene in the barn owl genome. The rectangular boxes connected by thin lines depict genes, with the boxes denoting exons and connecting lines being introns. Direction of transcription is denoted by the > or < symbols (signify positive and negative strand of transcription respectively) beside the gene names. chromosome bands and the genomic coordinates in kilobase (kb) are shown in the topmost row. Details of the contig and scaffold identity are provided in the blue bar located in the middle of the figure. Exact location of the *COA1* gene is depicted by a red box.

Figure S333

Oxyura jamaicensis

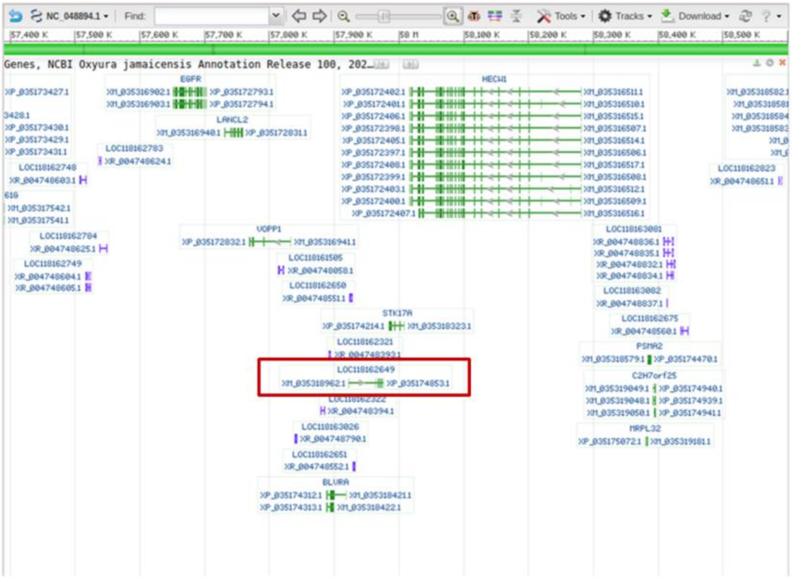

Figure S333: Gene order near *COA1* gene in the ruddy duck genome. The rectangular boxes connected by thin lines depict genes, with the boxes denoting exons and connecting lines being introns. Direction of transcription is denoted by the > or < symbols (signify positive and negative strand of transcription respectively) beside the gene names. chromosome bands and the genomic coordinates in kilobase (kb) are shown in the topmost row. Details of the contig and scaffold identity are provided in the blue bar located in the middle of the figure. Exact location of the *COA1* gene is depicted by a red box.

Figure S334

Pipra filicauda

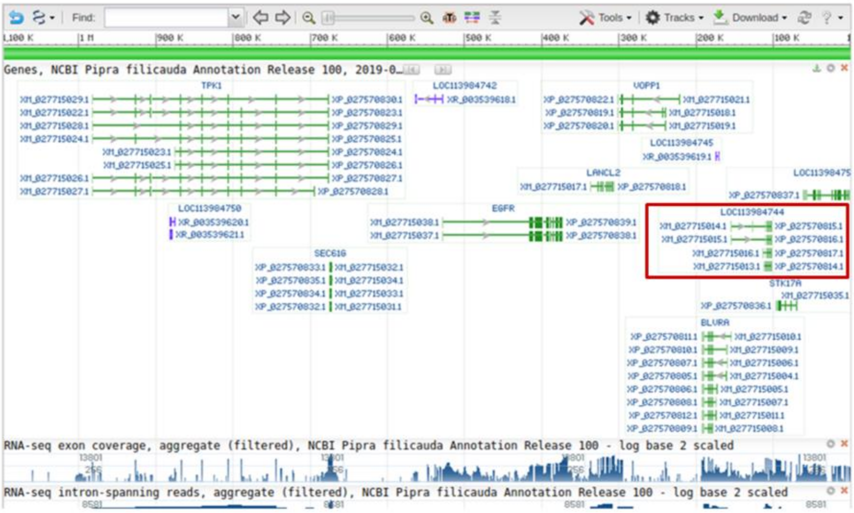

Figure S334: Gene order near *COA1* gene in the wire-tailed manakin genome. The rectangular boxes connected by thin lines depict genes, with the boxes denoting exons and connecting lines being introns. Direction of transcription is denoted by the > or < symbols (signify positive and negative strand of transcription respectively) beside the gene names. chromosome bands and the genomic coordinates in kilobase (kb) are shown in the topmost row. Details of the contig and scaffold identity are provided in the blue bar located in the middle of the figure. Exact location of the *COA1* gene is depicted by a red box.

Figure S335

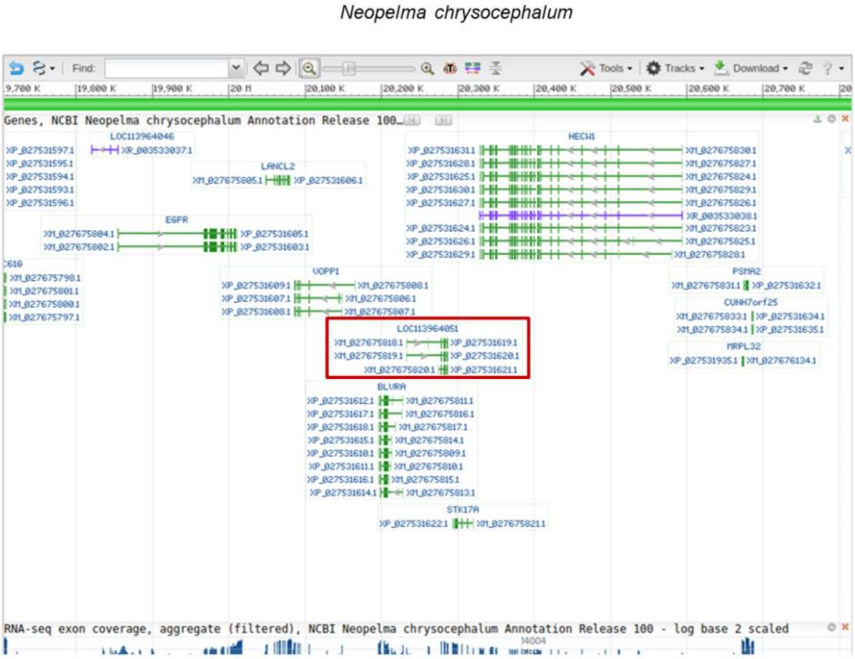

Figure S335: Gene order near *COA1* gene in the saffron-crested tyrant-manakin genome. The rectangular boxes connected by thin lines depict genes, with the boxes denoting exons and connecting lines being introns. Direction of transcription is denoted by the > or < symbols (signify positive and negative strand of transcription respectively) beside the gene names. chromosome bands and the genomic coordinates in kilobase (kb) are shown in the topmost row. Details of the contig and scaffold identity are provided in the blue bar located in the middle of the figure. Exact location of the *COA1* gene is depicted by a red box.

Figure S336

Sturnus vulgaris

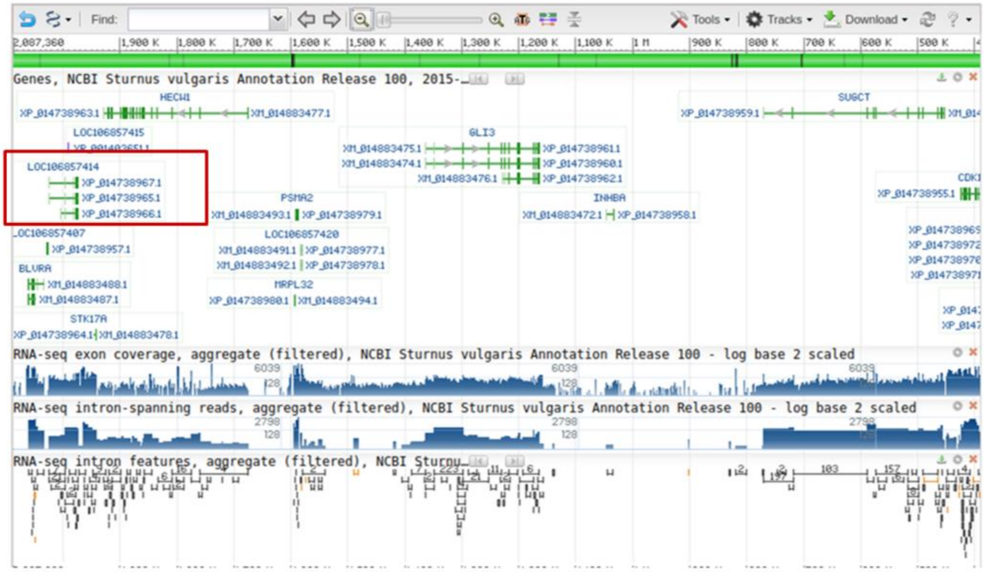

Figure S336: Gene order near *COA1* gene in the common starling genome. The rectangular boxes connected by thin lines depict genes, with the boxes denoting exons and connecting lines being introns. Direction of transcription is denoted by the > or < symbols (signify positive and negative strand of transcription respectively) beside the gene names. chromosome bands and the genomic coordinates in kilobase (kb) are shown in the topmost row. Details of the contig and scaffold identity are provided in the blue bar located in the middle of the figure. Exact location of the *COA1* gene is depicted by a red box.

Figure S337

Pseudopodoces humilis

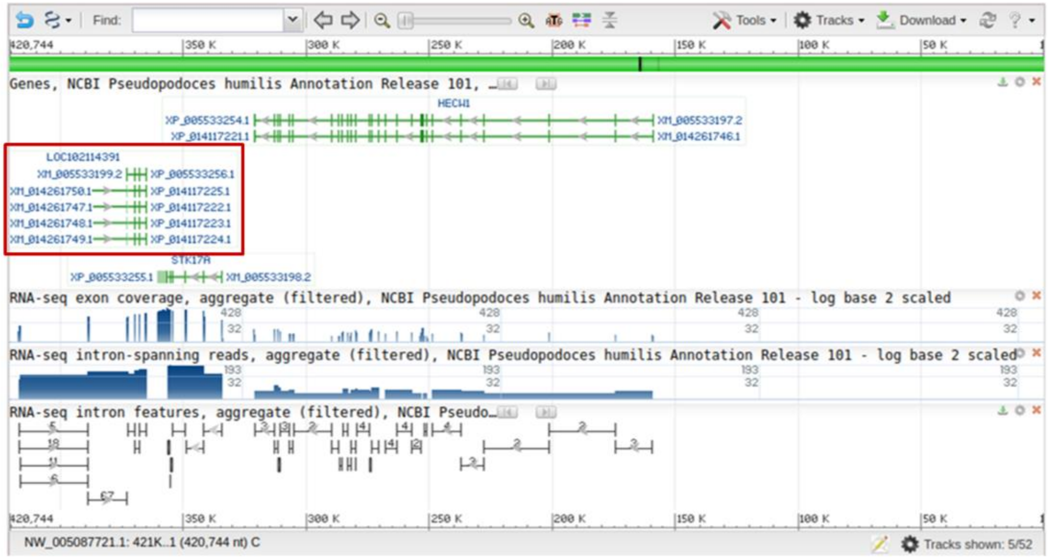

Figure S337: Gene order near *COA1* gene in the Tibetan ground-tit genome. The rectangular boxes connected by thin lines depict genes, with the boxes denoting exons and connecting lines being introns. Direction of transcription is denoted by the > or < symbols (signify positive and negative strand of transcription respectively) beside the gene names. chromosome bands and the genomic coordinates in kilobase (kb) are shown in the topmost row. Details of the contig and scaffold identity are provided in the blue bar located in the middle of the figure. Exact location of the *COA1* gene is depicted by a red box.

Figure S338

Pelecanus crispus

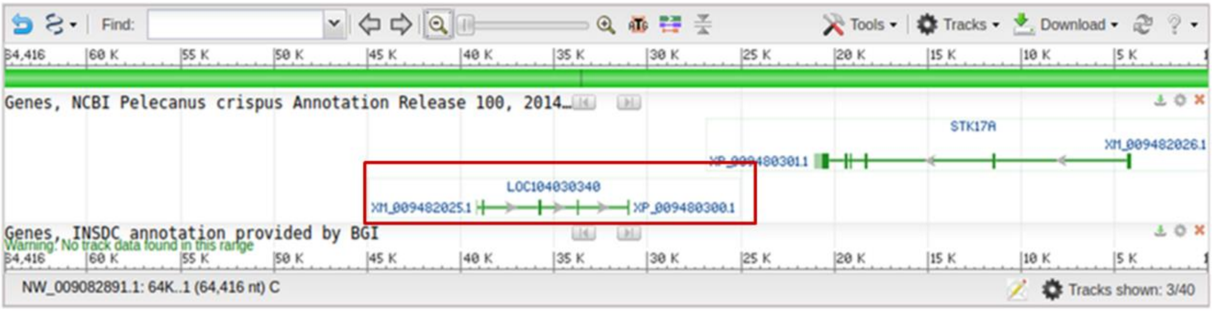

Figure S338: Gene order near *COA1* gene in the Dalmatian pelican genome. The rectangular boxes connected by thin lines depict genes, with the boxes denoting exons and connecting lines being introns. Direction of transcription is denoted by the > or < symbols (signify positive and negative strand of transcription respectively) beside the gene names. chromosome bands and the genomic coordinates in kilobase (kb) are shown in the topmost row. Details of the contig and scaffold identity are provided in the blue bar located in the middle of the figure. Exact location of the *COA1* gene is depicted by a red box.

Figure S339

Rodents group

Ochotona princeps

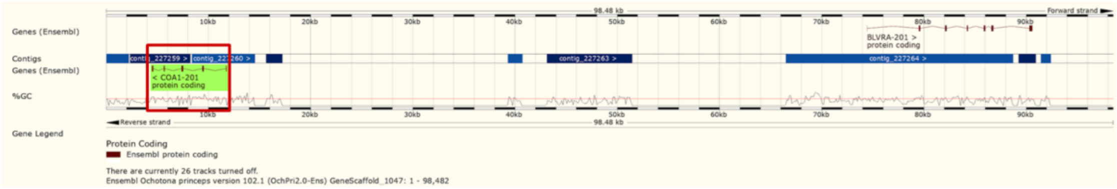

Figure S339: Gene order near *COA1* gene in the American pika (*Ochotona princeps)* genome. The rectangular boxes connected by thin lines depict genes, with the boxes denoting exons and connecting lines being introns. Direction of transcription is denoted by the > or < symbols (signify positive and negative strand of transcription respectively) beside the gene names. chromosome bands and the genomic coordinates in kilobase (kb) are shown in the topmost row. Details of the contig and scaffold identity are provided in the blue bar located in the middle of the figure. Exact location of the *COA1* gene is depicted by a red box.

Figure S340

Fukomys damarensis

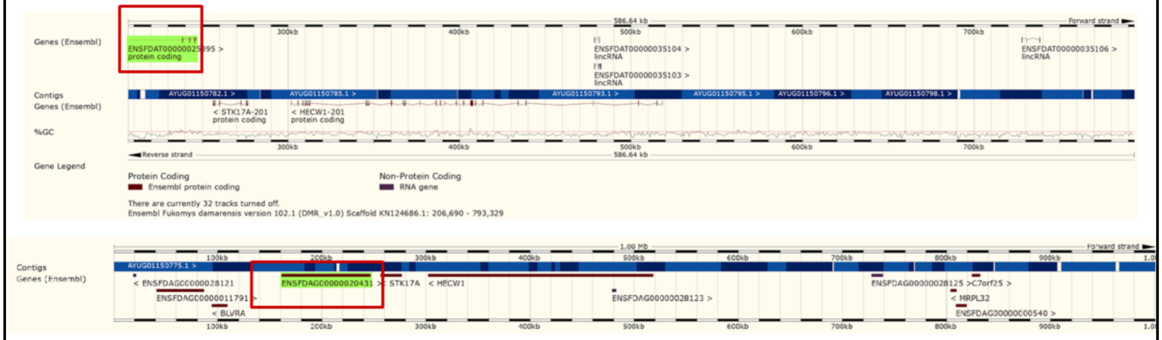

Figure S340: Gene order near *COA1* gene in the damara mole-rat genome. The rectangular boxes connected by thin lines depict genes, with the boxes denoting exons and connecting lines being introns. Direction of transcription is denoted by the > or < symbols (signify positive and negative strand of transcription respectively) beside the gene names. chromosome bands and the genomic coordinates in kilobase (kb) are shown in the topmost row. Details of the contig and scaffold identity are provided in the blue bar located in the middle of the figure. Exact location of the *COA1* gene is depicted by a red box.

Figure S341

Cavia porcellus

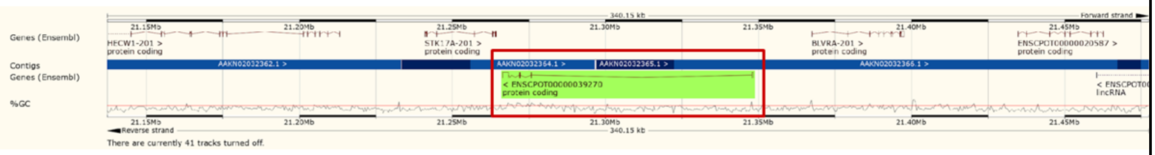

Figure S341: Gene order near *COA1* gene in the domestic guinea pig genome. The rectangular boxes connected by thin lines depict genes, with the boxes denoting exons and connecting lines being introns. Direction of transcription is denoted by the > or < symbols (signify positive and negative strand of transcription respectively) beside the gene names. chromosome bands and the genomic coordinates in kilobase (kb) are shown in the topmost row. Details of the contig and scaffold identity are provided in the blue bar located in the middle of the figure. Exact location of the *COA1* gene is depicted by a red box.

Figure S342

Sciurus vulgaris

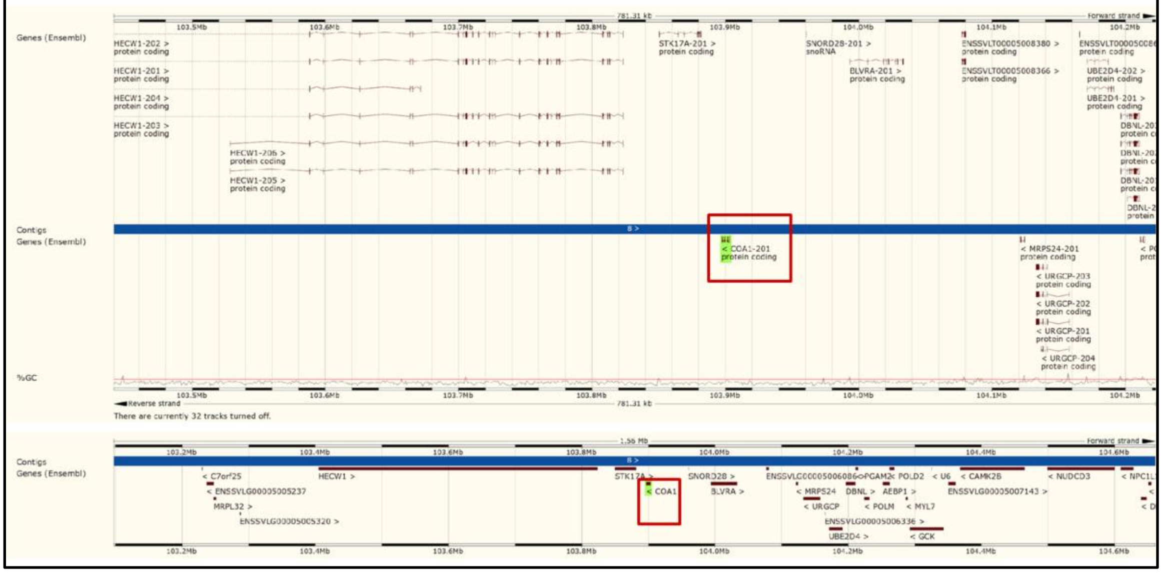

Figure S342: Gene order near *COA1* gene in the Eurasian red squirrel genome. The rectangular boxes connected by thin lines depict genes, with the boxes denoting exons and connecting lines being introns. Direction of transcription is denoted by the > or < symbols (signify positive and negative strand of transcription respectively) beside the gene names. chromosome bands and the genomic coordinates in kilobase (kb) are shown in the topmost row. Details of the contig and scaffold identity are provided in the blue bar located in the middle of the figure. Exact location of the *COA1* gene is depicted by a red box.

Figure S343

Chinchilla lanigera

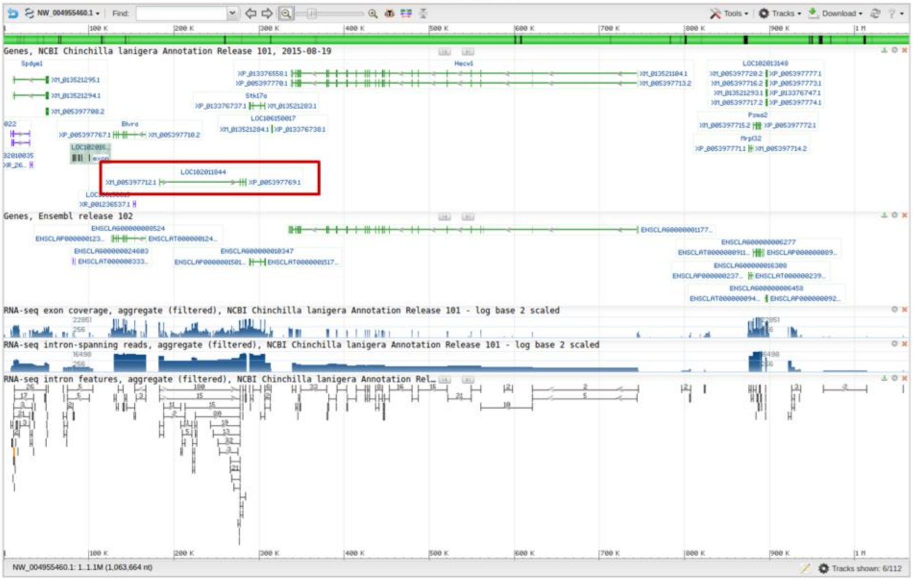

Figure S343: Gene order near *COA1* gene in the long-tailed chinchilla genome. The rectangular boxes connected by thin lines depict genes, with the boxes denoting exons and connecting lines being introns. Direction of transcription is denoted by the > or < symbols (signify positive and negative strand of transcription respectively) beside the gene names. chromosome bands and the genomic coordinates in kilobase (kb) are shown in the topmost row. Details of the contig and scaffold identity are provided in the blue bar located in the middle of the figure. Exact location of the *COA1* gene is depicted by a red box.

Figure S344

Octodon degus

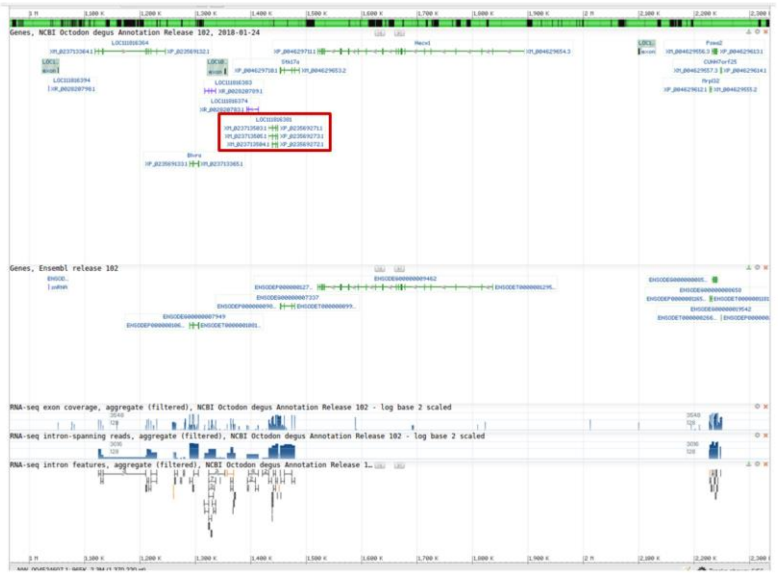

Figure S344: Gene order near *COA1* gene in the degu genome. The rectangular boxes connected by thin lines depict genes, with the boxes denoting exons and connecting lines being introns. Direction of transcription is denoted by the > or < symbols (signify positive and negative strand of transcription respectively) beside the gene names. chromosome bands and the genomic coordinates in kilobase (kb) are shown in the topmost row. Details of the contig and scaffold identity are provided in the blue bar located in the middle of the figure. Exact location of the *COA1* gene is depicted by a red box.

Figure S345

Heterocephalus glaber

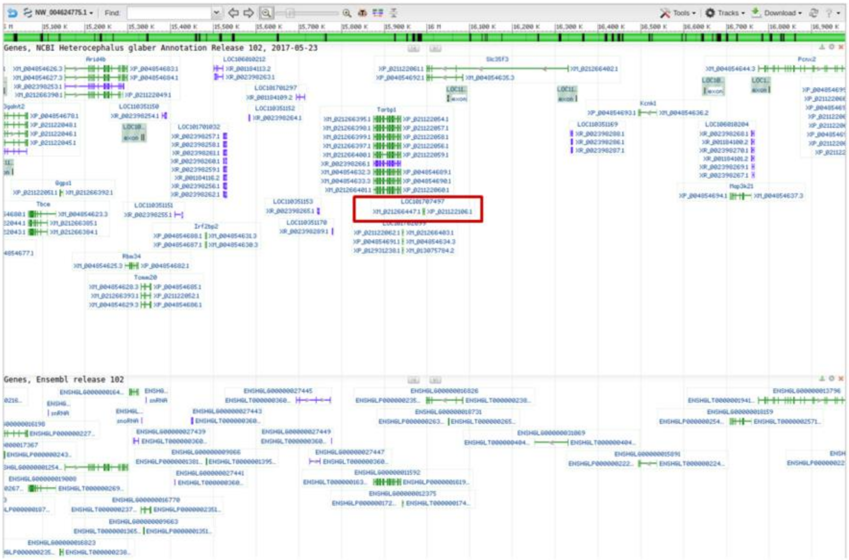

Figure S345: Gene order near *COA1* gene in the naked mole-rat genome. The rectangular boxes connected by thin lines depict genes, with the boxes denoting exons and connecting lines being introns. Direction of transcription is denoted by the > or < symbols (signify positive and negative strand of transcription respectively) beside the gene names. chromosome bands and the genomic coordinates in kilobase (kb) are shown in the topmost row. Details of the contig and scaffold identity are provided in the blue bar located in the middle of the figure. Exact location of the *COA1* gene is depicted by a red box.

Figure S346

Placental mammals group

Homo sapiens

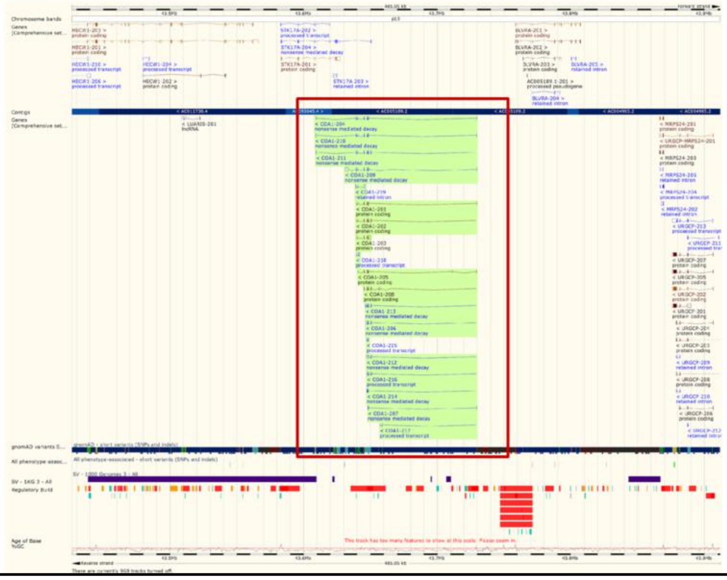

Figure S346: Gene order near *COA1* gene in the human genome. The rectangular boxes connected by thin lines depict genes, with the boxes denoting exons and connecting lines being introns. Direction of transcription is denoted by the > or < symbols (signify positive and negative strand of transcription respectively) beside the gene names. chromosome bands and the genomic coordinates in kilobase (kb) are shown in the topmost row. Details of the contig and scaffold identity are provided in the blue bar located in the middle of the figure. Exact location of the *COA1* gene is depicted by a red box.

Figure S347

Macaca mulatta

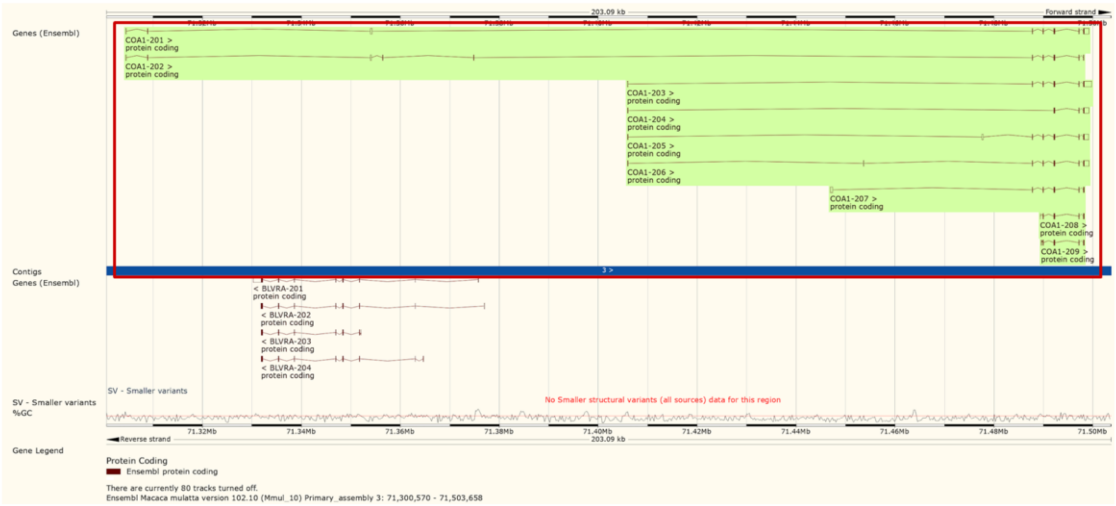

Figure S347: Gene order near *COA1* gene in the macaque genome. The rectangular boxes connected by thin lines depict genes, with the boxes denoting exons and connecting lines being introns. Direction of transcription is denoted by the > or < symbols (signify positive and negative strand of transcription respectively) beside the gene names. chromosome bands and the genomic coordinates in kilobase (kb) are shown in the topmost row. Details of the contig and scaffold identity are provided in the blue bar located in the middle of the figure. Exact location of the *COA1* gene is depicted by a red box.

Figure S348

Chlorocebus sabaeus

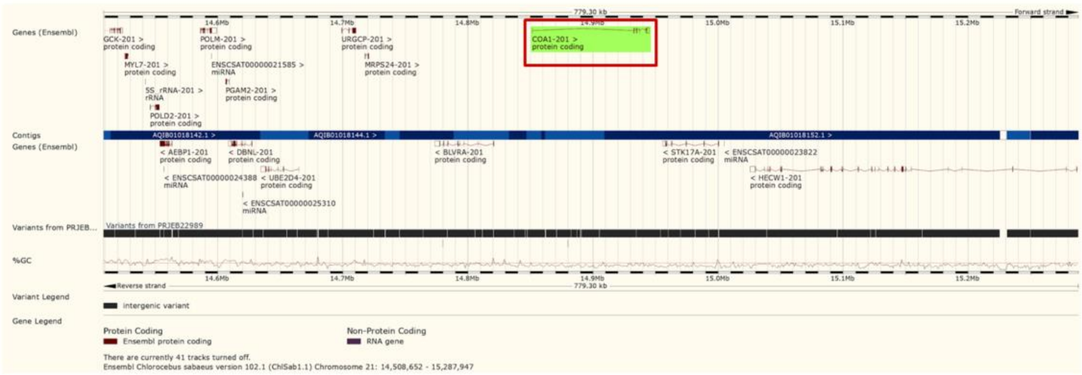

Figure S348: Gene order near *COA1* gene in the vervet-AGM genome. The rectangular boxes connected by thin lines depict genes, with the boxes denoting exons and connecting lines being introns. Direction of transcription is denoted by the > or < symbols (signify positive and negative strand of transcription respectively) beside the gene names. chromosome bands and the genomic coordinates in kilobase (kb) are shown in the topmost row. Details of the contig and scaffold identity are provided in the blue bar located in the middle of the figure. Exact location of the *COA1* gene is depicted by a red box.

Figure S349

Nomascus leucogenys

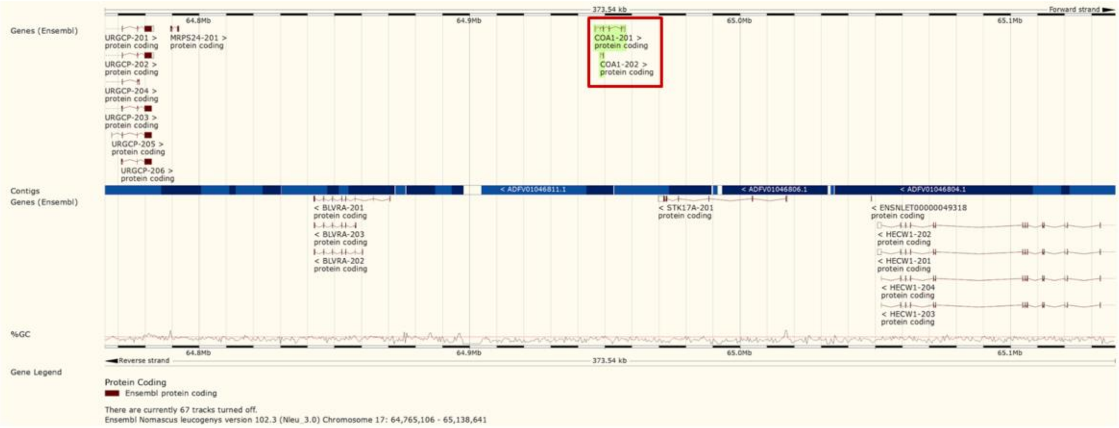

Figure S349: Gene order near *COA1* gene in the gibbon genome. The rectangular boxes connected by thin lines depict genes, with the boxes denoting exons and connecting lines being introns. Direction of transcription is denoted by the > or < symbols (signify positive and negative strand of transcription respectively) beside the gene names. chromosome bands and the genomic coordinates in kilobase (kb) are shown in the topmost row. Details of the contig and scaffold identity are provided in the blue bar located in the middle of the figure. Exact location of the *COA1* gene is depicted by a red box.

Figure S350

Pan troglodytes

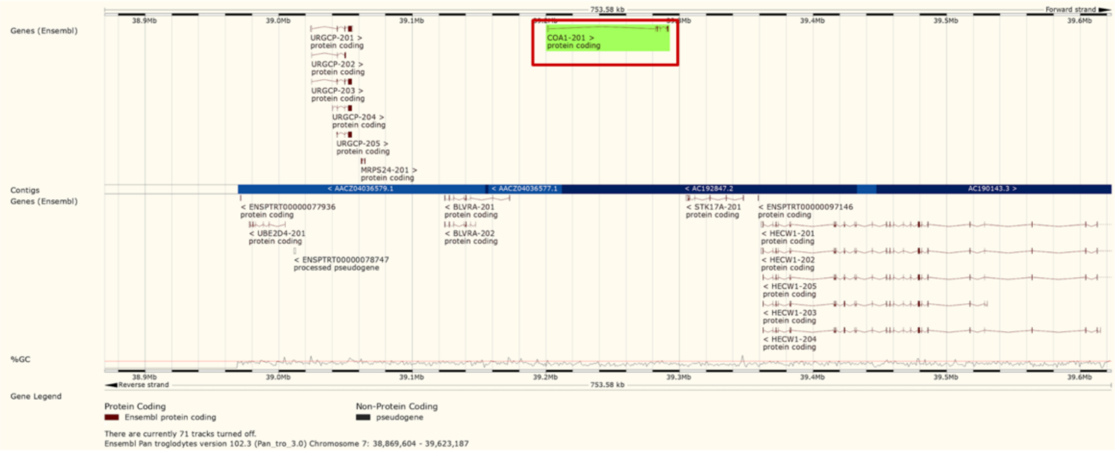

Figure S350: Gene order near *COA1* gene in the chimpanzee genome. The rectangular boxes connected by thin lines depict genes, with the boxes denoting exons and connecting lines being introns. Direction of transcription is denoted by the > or < symbols (signify positive and negative strand of transcription respectively) beside the gene names. chromosome bands and the genomic coordinates in kilobase (kb) are shown in the topmost row. Details of the contig and scaffold identity are provided in the blue bar located in the middle of the figure. Exact location of the *COA1* gene is depicted by a red box.

Figure S351

Colobus angolensis palliatus

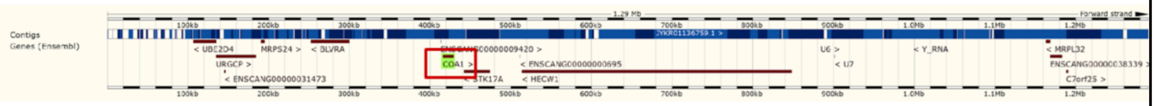

Figure S351: Gene order near *COA1* gene in the Angola colobus genome. The rectangular boxes connected by thin lines depict genes, with the boxes denoting exons and connecting lines being introns. Direction of transcription is denoted by the > or < symbols (signify positive and negative strand of transcription respectively) beside the gene names. chromosome bands and the genomic coordinates in kilobase (kb) are shown in the topmost row. Details of the contig and scaffold identity are provided in the blue bar located in the middle of the figure. Exact location of the *COA1* gene is depicted by a red box.

Figure S352

Rhinopithecus bieti

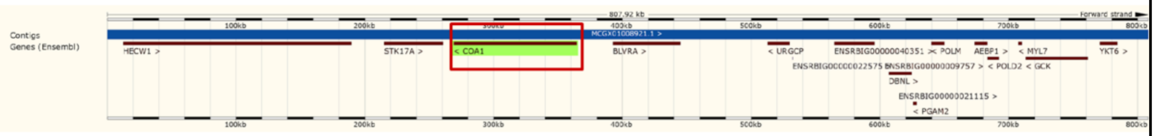

Figure S352: Gene order near *COA1* gene in the black snub-nosed monkey genome. The rectangular boxes connected by thin lines depict genes, with the boxes denoting exons and connecting lines being introns. Direction of transcription is denoted by the > or < symbols (signify positive and negative strand of transcription respectively) beside the gene names. chromosome bands and the genomic coordinates in kilobase (kb) are shown in the topmost row. Details of the contig and scaffold identity are provided in the blue bar located in the middle of the figure. Exact location of the *COA1* gene is depicted by a red box.

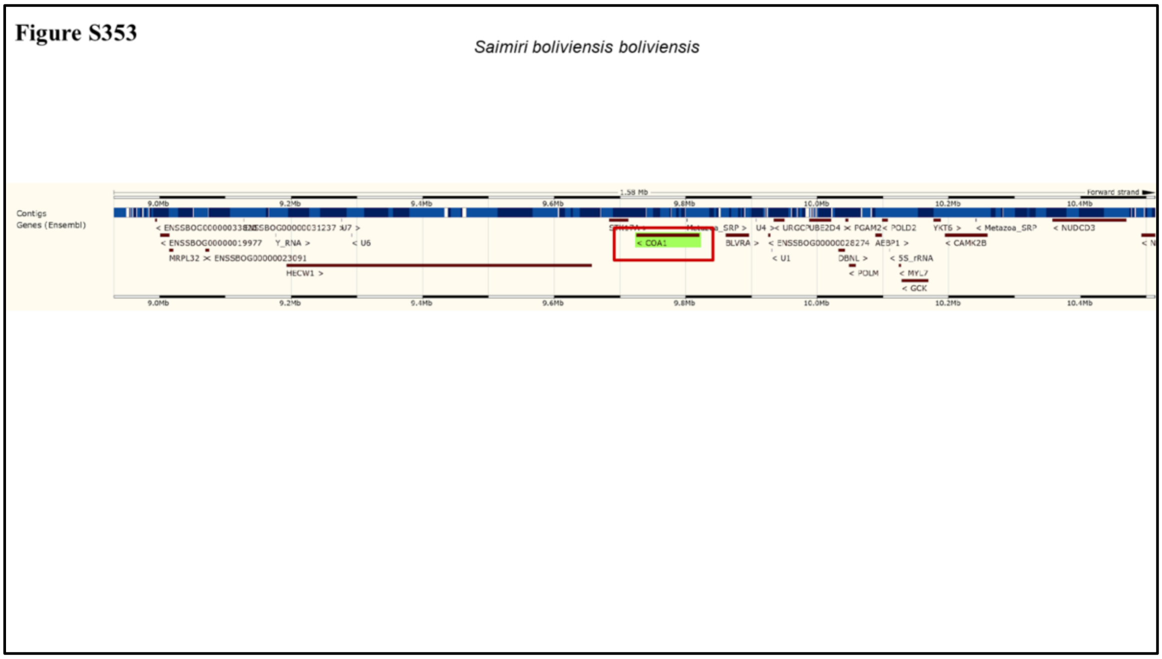

Figure S353: Gene order near *COA1* gene in the Bolivian squirrel monkey genome. The rectangular boxes connected by thin lines depict genes, with the boxes denoting exons and connecting lines being introns. Direction of transcription is denoted by the > or < symbols (signify positive and negative strand of transcription respectively) beside the gene names. chromosome bands and the genomic coordinates in kilobase (kb) are shown in the topmost row. Details of the contig and scaffold identity are provided in the blue bar located in the middle of the figure. Exact location of the *COA1* gene is depicted by a red box.

Figure S354

Otolemur garnettii

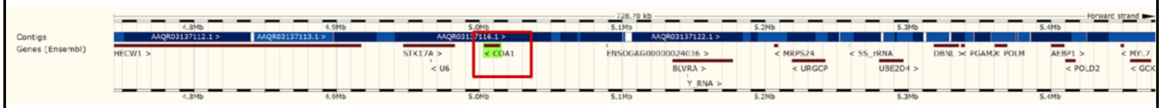

Figure S354: Gene order near *COA1* gene in the bushbaby genome. The rectangular boxes connected by thin lines depict genes, with the boxes denoting exons and connecting lines being introns. Direction of transcription is denoted by the > or < symbols (signify positive and negative strand of transcription respectively) beside the gene names. chromosome bands and the genomic coordinates in kilobase (kb) are shown in the topmost row. Details of the contig and scaffold identity are provided in the blue bar located in the middle of the figure. Exact location of the *COA1* gene is depicted by a red box.

Figure S355

Pongo abelii

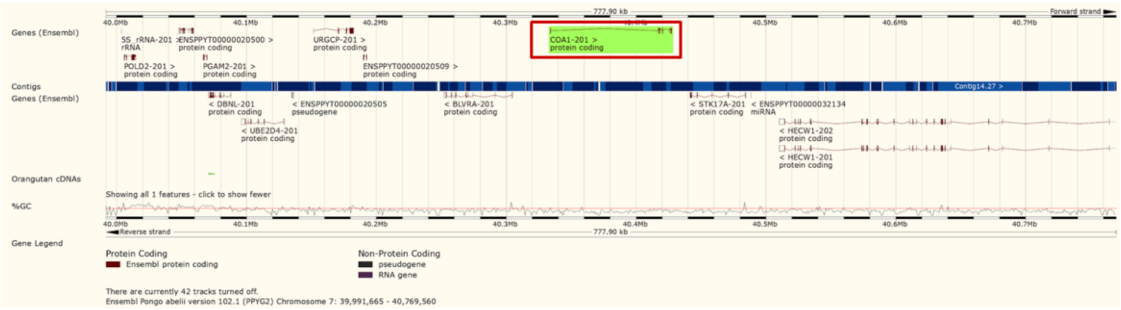

Figure S355: Gene order near *COA1* gene in the orangutan genome. The rectangular boxes connected by thin lines depict genes, with the boxes denoting exons and connecting lines being introns. Direction of transcription is denoted by the > or < symbols (signify positive and negative strand of transcription respectively) beside the gene names. chromosome bands and the genomic coordinates in kilobase (kb) are shown in the topmost row. Details of the contig and scaffold identity are provided in the blue bar located in the middle of the figure. Exact location of the *COA1* gene is depicted by a red box.

Figure S356

Cebus capucinus imitator

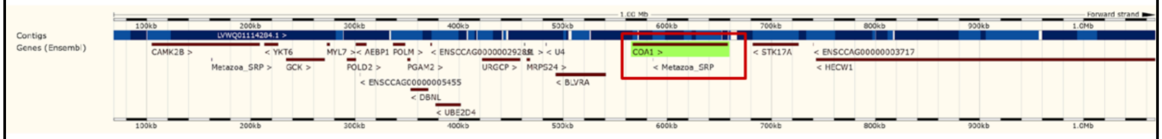

Figure S356: Gene order near *COA1* gene in the capuchin genome. The rectangular boxes connected by thin lines depict genes, with the boxes denoting exons and connecting lines being introns. Direction of transcription is denoted by the > or < symbols (signify positive and negative strand of transcription respectively) beside the gene names. chromosome bands and the genomic coordinates in kilobase (kb) are shown in the topmost row. Details of the contig and scaffold identity are provided in the blue bar located in the middle of the figure. Exact location of the *COA1* gene is depicted by a red box.

Figure S357

Propithecus coquereli

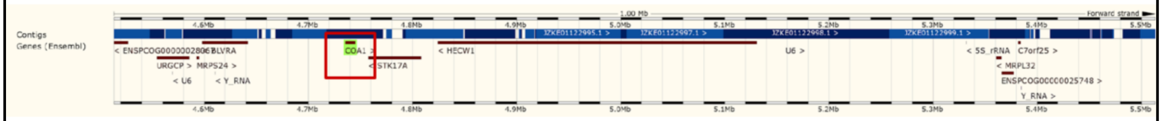

Figure S357: Gene order near *COA1* gene in the Coquerel's sifaka genome. The rectangular boxes connected by thin lines depict genes, with the boxes denoting exons and connecting lines being introns. Direction of transcription is denoted by the > or < symbols (signify positive and negative strand of transcription respectively) beside the gene names. chromosome bands and the genomic coordinates in kilobase (kb) are shown in the topmost row. Details of the contig and scaffold identity are provided in the blue bar located in the middle of the figure. Exact location of the *COA1* gene is depicted by a red box.

Figure S358

Macaca fascicularis

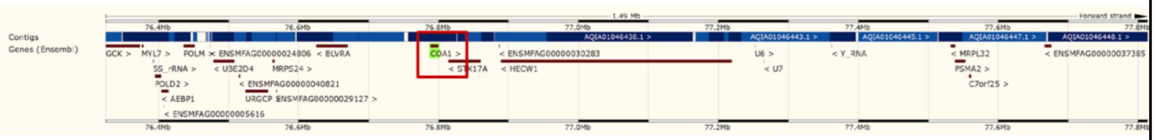

Figure S358: Gene order near *COA1* gene in the crab-eating macaque genome. The rectangular boxes connected by thin lines depict genes, with the boxes denoting exons and connecting lines being introns. Direction of transcription is denoted by the > or < symbols (signify positive and negative strand of transcription respectively) beside the gene names. chromosome bands and the genomic coordinates in kilobase (kb) are shown in the topmost row. Details of the contig and scaffold identity are provided in the blue bar located in the middle of the figure. Exact location of the *COA1* gene is depicted by a red box.

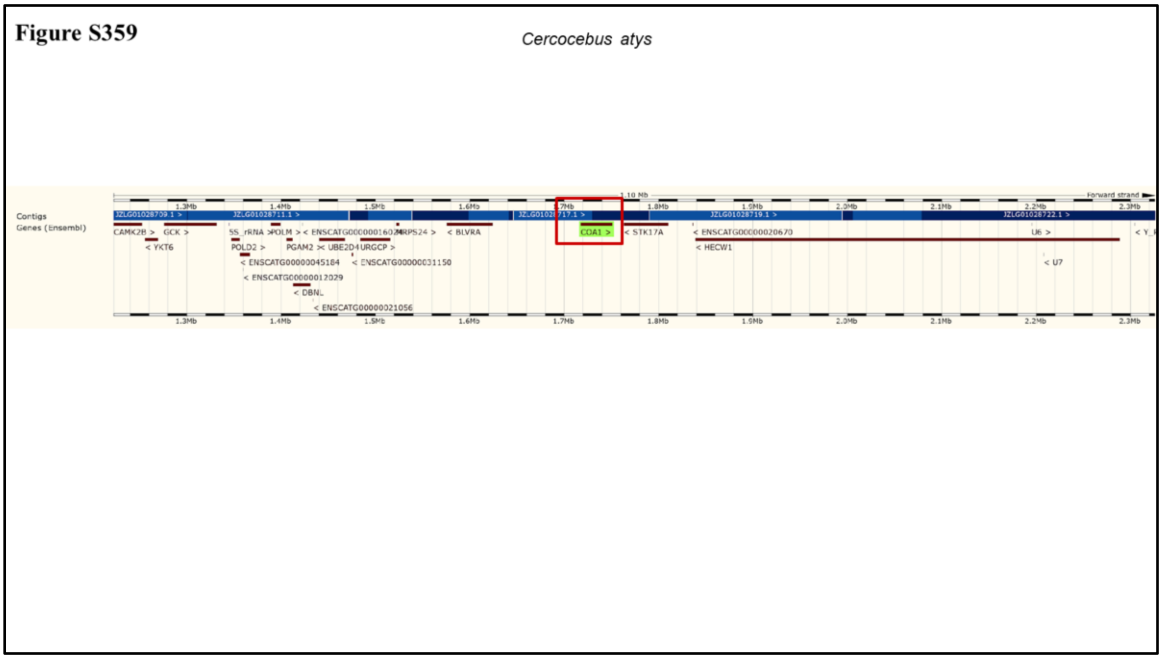

Figure S359: Gene order near *COA1* gene in the sooty mangabey genome. The rectangular boxes connected by thin lines depict genes, with the boxes denoting exons and connecting lines being introns. Direction of transcription is denoted by the > or < symbols (signify positive and negative strand of transcription respectively) beside the gene names. chromosome bands and the genomic coordinates in kilobase (kb) are shown in the topmost row. Details of the contig and scaffold identity are provided in the blue bar located in the middle of the figure. Exact location of the *COA1* gene is depicted by a red box.

Figure S360

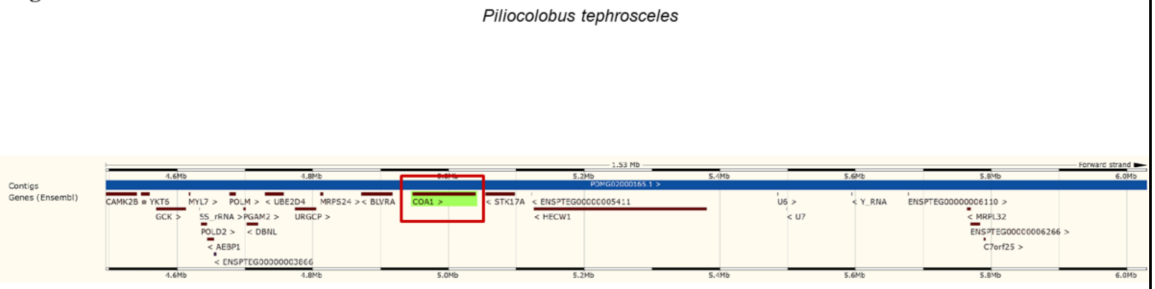

Figure S360: Gene order near *COA1* gene in the Ugandan red colobus genome. The rectangular boxes connected by thin lines depict genes, with the boxes denoting exons and connecting lines being introns. Direction of transcription is denoted by the > or < symbols (signify positive and negative strand of transcription respectively) beside the gene names. chromosome bands and the genomic coordinates in kilobase (kb) are shown in the topmost row. Details of the contig and scaffold identity are provided in the blue bar located in the middle of the figure. Exact location of the *COA1* gene is depicted by a red box.

Figure S361

Mandrillus leucophaeus

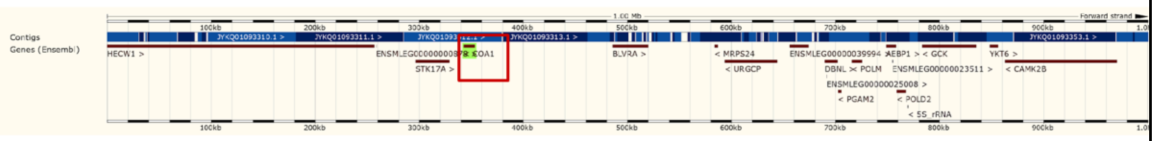

Figure S361: Gene order near *COA1* gene in the drill genome. The rectangular boxes connected by thin lines depict genes, with the boxes denoting exons and connecting lines being introns. Direction of transcription is denoted by the > or < symbols (signify positive and negative strand of transcription respectively) beside the gene names. chromosome bands and the genomic coordinates in kilobase (kb) are shown in the topmost row. Details of the contig and scaffold identity are provided in the blue bar located in the middle of the figure. Exact location of the *COA1* gene is depicted by a red box.

Figure S362

Theropithecus gelada

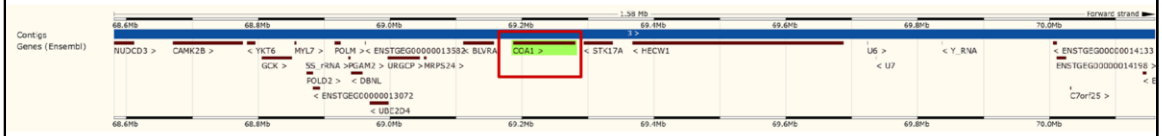

Figure S362: Gene order near *COA1* gene in the gelada genome. The rectangular boxes connected by thin lines depict genes, with the boxes denoting exons and connecting lines being introns. Direction of transcription is denoted by the > or < symbols (signify positive and negative strand of transcription respectively) beside the gene names. chromosome bands and the genomic coordinates in kilobase (kb) are shown in the topmost row. Details of the contig and scaffold identity are provided in the blue bar located in the middle of the figure. Exact location of the *COA1* gene is depicted by a red box.

Figure S363

Gorilla gorilla gorilla

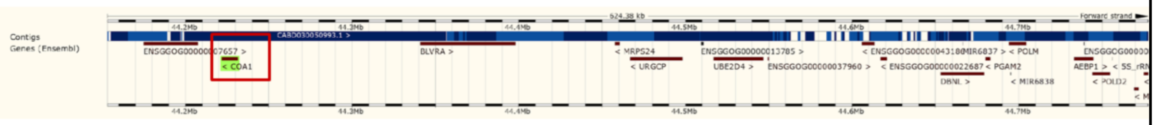

Figure S363: Gene order near *COA1* gene in the gorilla genome. The rectangular boxes connected by thin lines depict genes, with the boxes denoting exons and connecting lines being introns. Direction of transcription is denoted by the > or < symbols (signify positive and negative strand of transcription respectively) beside the gene names. chromosome bands and the genomic coordinates in kilobase (kb) are shown in the topmost row. Details of the contig and scaffold identity are provided in the blue bar located in the middle of the figure. Exact location of the *COA1* gene is depicted by a red box.

Figure S364

Prolemur simus

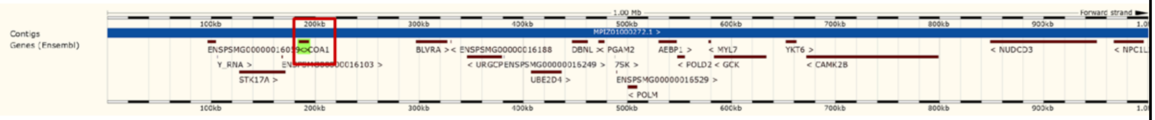

Figure S364: Gene order near *COA1* gene in the greater bamboo lemur genome. The rectangular boxes connected by thin lines depict genes, with the boxes denoting exons and connecting lines being introns. Direction of transcription is denoted by the > or < symbols (signify positive and negative strand of transcription respectively) beside the gene names. chromosome bands and the genomic coordinates in kilobase (kb) are shown in the topmost row. Details of the contig and scaffold identity are provided in the blue bar located in the middle of the figure. Exact location of the *COA1* gene is depicted by a red box.

Figure S365

Callithrix jacchus

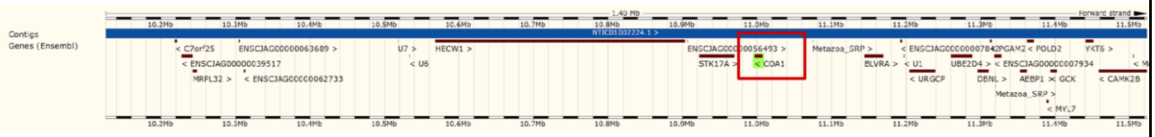

Figure S365: Gene order near *COA1* gene in the marmoset genome. The rectangular boxes connected by thin lines depict genes, with the boxes denoting exons and connecting lines being introns. Direction of transcription is denoted by the > or < symbols (signify positive and negative strand of transcription respectively) beside the gene names. chromosome bands and the genomic coordinates in kilobase (kb) are shown in the topmost row. Details of the contig and scaffold identity are provided in the blue bar located in the middle of the figure. Exact location of the *COA1* gene is depicted by a red box.

Figure S366

Aotus nancymaae

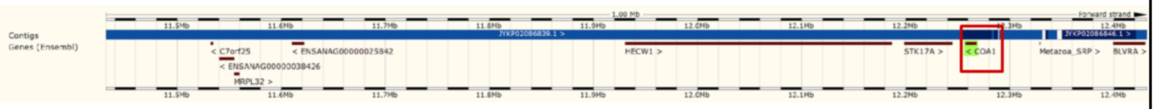

Figure S366: Gene order near *COA1* gene in the Ma's night monkey genome. The rectangular boxes connected by thin lines depict genes, with the boxes denoting exons and connecting lines being introns. Direction of transcription is denoted by the > or < symbols (signify positive and negative strand of transcription respectively) beside the gene names. chromosome bands and the genomic coordinates in kilobase (kb) are shown in the topmost row. Details of the contig and scaffold identity are provided in the blue bar located in the middle of the figure. Exact location of the *COA1* gene is depicted by a red box.

Figure S367

Microcebus murinus

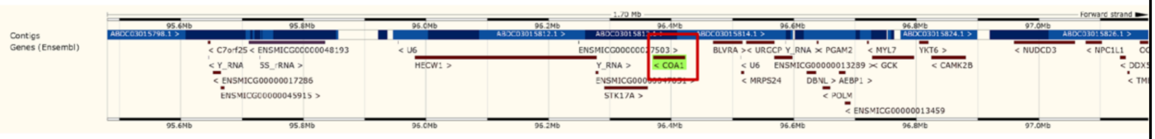

Figure S367: Gene order near *COA1* gene in the mouse lemur genome. The rectangular boxes connected by thin lines depict genes, with the boxes denoting exons and connecting lines being introns. Direction of transcription is denoted by the > or < symbols (signify positive and negative strand of transcription respectively) beside the gene names. chromosome bands and the genomic coordinates in kilobase (kb) are shown in the topmost row. Details of the contig and scaffold identity are provided in the blue bar located in the middle of the figure. Exact location of the *COA1* gene is depicted by a red box.

Figure S368

Papio anubis

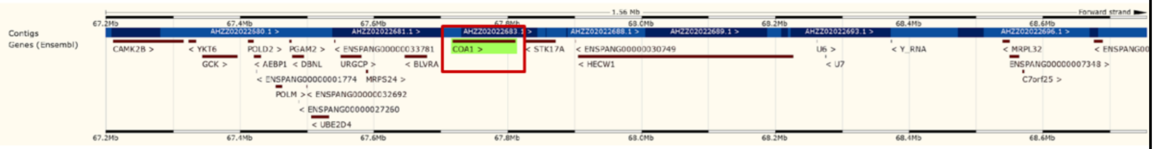

Figure S368: Gene order near *COA1* gene in the olive baboon genome. The rectangular boxes connected by thin lines depict genes, with the boxes denoting exons and connecting lines being introns. Direction of transcription is denoted by the > or < symbols (signify positive and negative strand of transcription respectively) beside the gene names. chromosome bands and the genomic coordinates in kilobase (kb) are shown in the topmost row. Details of the contig and scaffold identity are provided in the blue bar located in the middle of the figure. Exact location of the *COA1* gene is depicted by a red box.

Figure S369

Macaca nemestrina

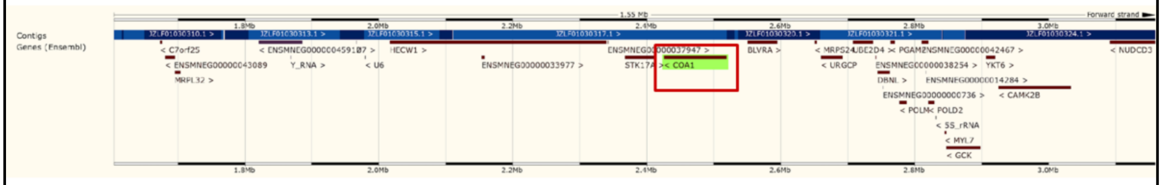

Figure S369: Gene order near *COA1* gene in the pig-tailed macaque genome. The rectangular boxes connected by thin lines depict genes, with the boxes denoting exons and connecting lines being introns. Direction of transcription is denoted by the > or < symbols (signify positive and negative strand of transcription respectively) beside the gene names. chromosome bands and the genomic coordinates in kilobase (kb) are shown in the topmost row. Details of the contig and scaffold identity are provided in the blue bar located in the middle of the figure. Exact location of the *COA1* gene is depicted by a red box.

Figure S370

Primates group duplication of *COA1* gene

Callithrix jacchus

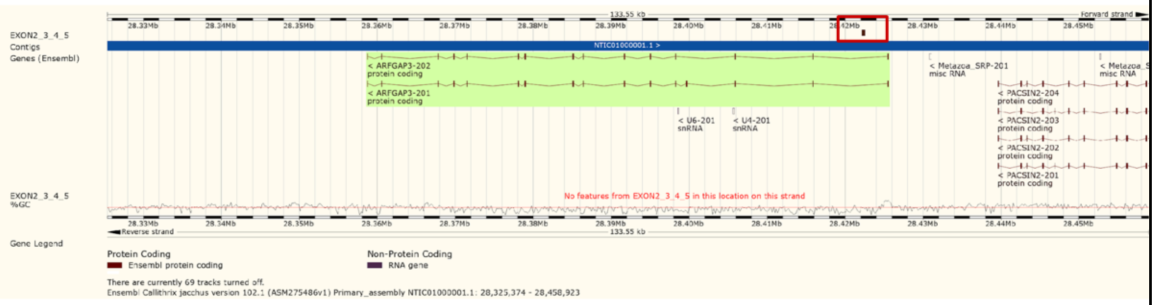

Figure S370: Gene order near *COA1* gene in the marmoset genome. The rectangular boxes connected by thin lines depict genes, with the boxes denoting exons and connecting lines being introns. Direction of transcription is denoted by the > or < symbols (signify positive and negative strand of transcription respectively) beside the gene names. chromosome bands and the genomic coordinates in kilobase (kb) are shown in the topmost row. Details of the contig and scaffold identity are provided in the blue bar located in the middle of the figure. Exact location of the *COA1* gene is depicted by a red box.

Figure S371

Aotus nancymaae

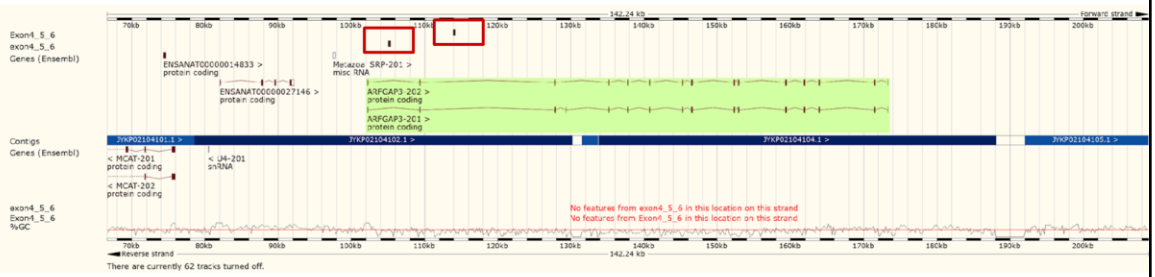

Figure S371: Gene order near *COA1* gene in the Ma's night monkey genome. The rectangular boxes connected by thin lines depict genes, with the boxes denoting exons and connecting lines being introns. Direction of transcription is denoted by the > or < symbols (signify positive and negative strand of transcription respectively) beside the gene names. chromosome bands and the genomic coordinates in kilobase (kb) are shown in the topmost row. Details of the contig and scaffold identity are provided in the blue bar located in the middle of the figure. Exact location of the *COA1* gene is depicted by a red box.

Figure S372

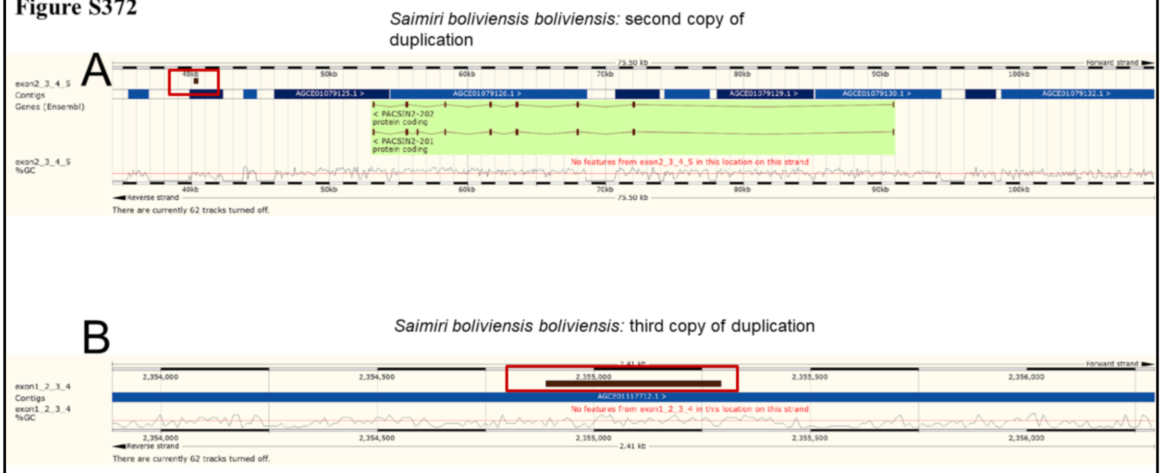

Figure S372: Gene order near *COA1* gene in the Bolivian squirrel monkey genome. The rectangular boxes connected by thin lines depict genes, with the boxes denoting exons and connecting lines being introns. Direction of transcription is denoted by the > or < symbols (signify positive and negative strand of transcription respectively) beside the gene names. chromosome bands and the genomic coordinates in kilobase (kb) are shown in the topmost row. Details of the contig and scaffold identity are provided in the blue bar located in the middle of the figure. Exact location of the *COA1* gene is depicted by a red box.

Figure S373

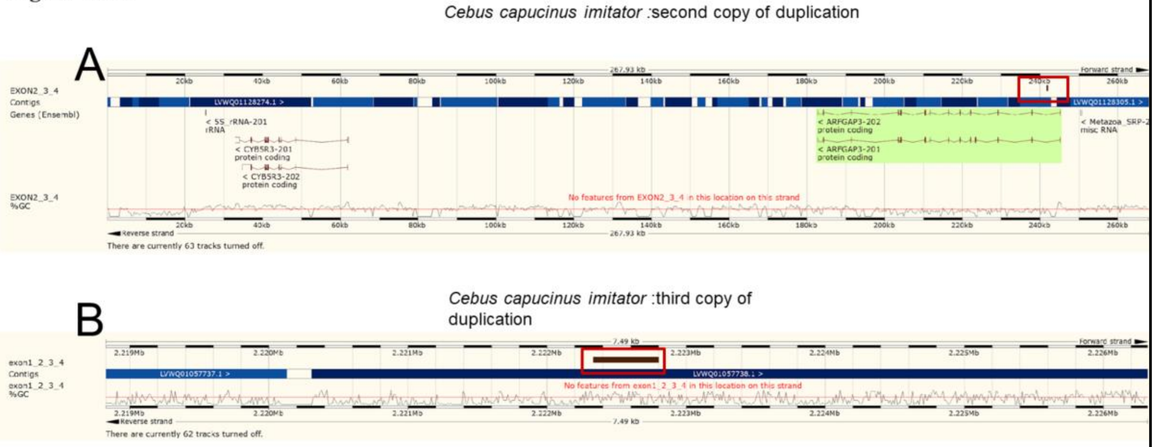

Figure S373: Gene order near *COA1* gene in the capuchin genome. The rectangular boxes connected by thin lines depict genes, with the boxes denoting exons and connecting lines being introns. Direction of transcription is denoted by the > or < symbols (signify positive and negative strand of transcription respectively) beside the gene names. chromosome bands and the genomic coordinates in kilobase (kb) are shown in the topmost row. Details of the contig and scaffold identity are provided in the blue bar located in the middle of the figure. Exact location of the *COA1* gene is depicted by a red box.

Figure S374

Mandrillus leucophaeus

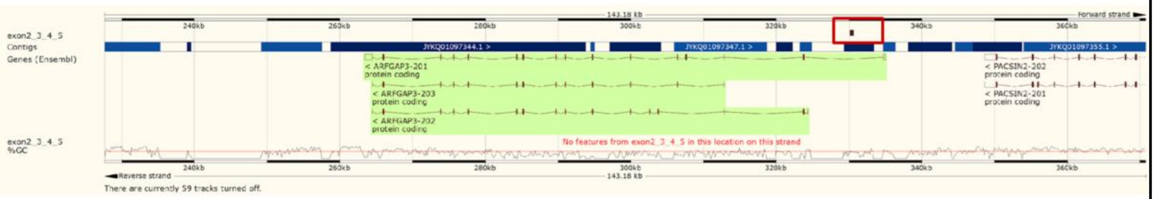

Figure S374: Gene order near *COA1* gene in the drill genome. The rectangular boxes connected by thin lines depict genes, with the boxes denoting exons and connecting lines being introns. Direction of transcription is denoted by the > or < symbols (signify positive and negative strand of transcription respectively) beside the gene names. chromosome bands and the genomic coordinates in kilobase (kb) are shown in the topmost row. Details of the contig and scaffold identity are provided in the blue bar located in the middle of the figure. Exact location of the *COA1* gene is depicted by a red box.

Figure S375

Cercocebus atys

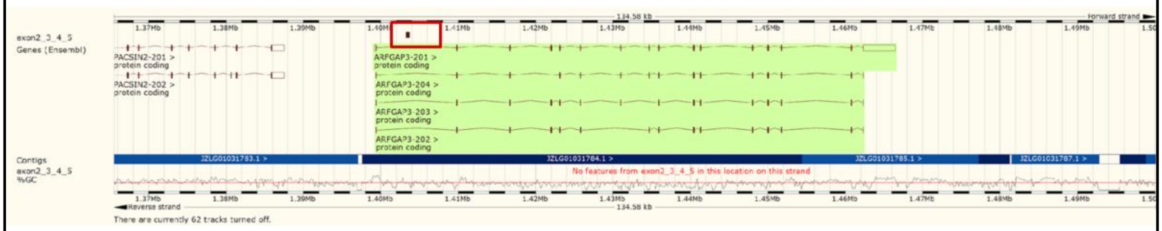

Figure S375: Gene order near *COA1* gene in the sooty mangabey genome. The rectangular boxes connected by thin lines depict genes, with the boxes denoting exons and connecting lines being introns. Direction of transcription is denoted by the > or < symbols (signify positive and negative strand of transcription respectively) beside the gene names. chromosome bands and the genomic coordinates in kilobase (kb) are shown in the topmost row. Details of the contig and scaffold identity are provided in the blue bar located in the middle of the figure. Exact location of the *COA1* gene is depicted by a red box.

Figure S376

Papio anubis

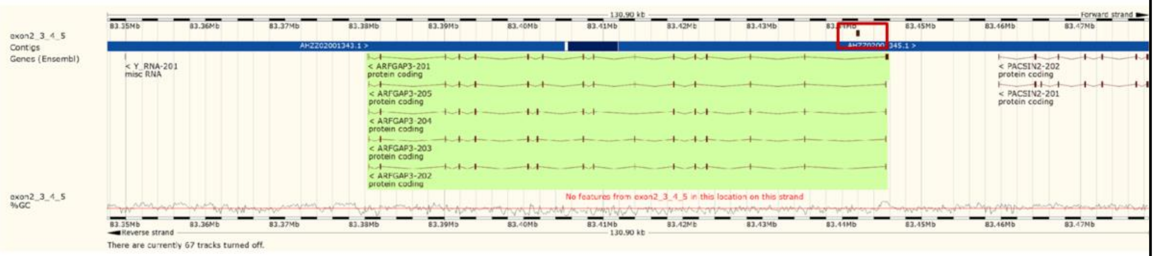

Figure S376: Gene order near *COA1* gene in the olive baboon genome. The rectangular boxes connected by thin lines depict genes, with the boxes denoting exons and connecting lines being introns. Direction of transcription is denoted by the > or < symbols (signify positive and negative strand of transcription respectively) beside the gene names. chromosome bands and the genomic coordinates in kilobase (kb) are shown in the topmost row. Details of the contig and scaffold identity are provided in the blue bar located in the middle of the figure. Exact location of the *COA1* gene is depicted by a red box.

Figure S377

Theropithecus gelada

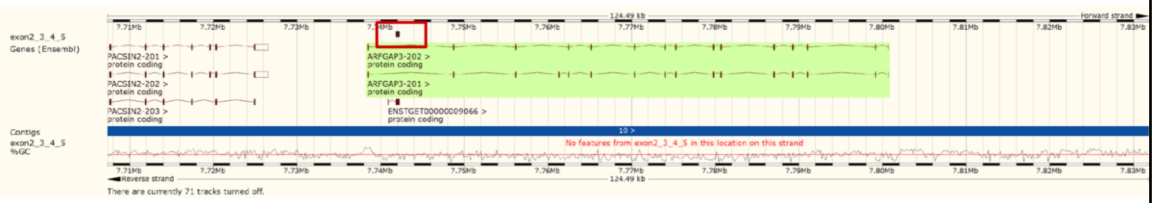

Figure S377: Gene order near *COA1* gene in the gelada genome. The rectangular boxes connected by thin lines depict genes, with the boxes denoting exons and connecting lines being introns. Direction of transcription is denoted by the > or < symbols (signify positive and negative strand of transcription respectively) beside the gene names. chromosome bands and the genomic coordinates in kilobase (kb) are shown in the topmost row. Details of the contig and scaffold identity are provided in the blue bar located in the middle of the figure. Exact location of the *COA1* gene is depicted by a red box.

Figure S378

Macaca fascicularis

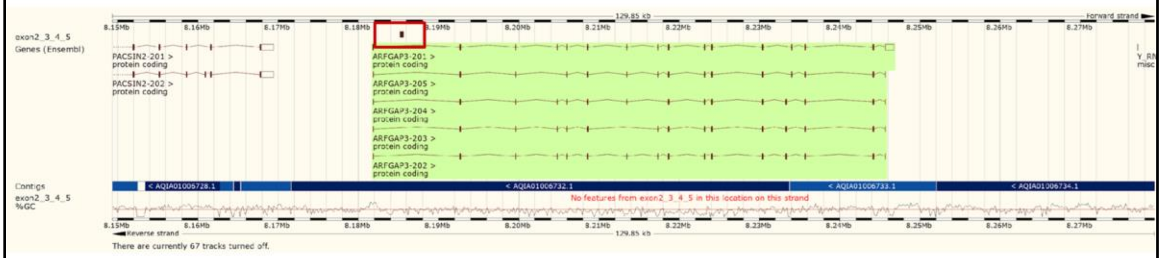

Figure S378: Gene order near *COA1* gene in the crab-eating macaque genome. The rectangular boxes connected by thin lines depict genes, with the boxes denoting exons and connecting lines being introns. Direction of transcription is denoted by the > or < symbols (signify positive and negative strand of transcription respectively) beside the gene names. chromosome bands and the genomic coordinates in kilobase (kb) are shown in the topmost row. Details of the contig and scaffold identity are provided in the blue bar located in the middle of the figure. Exact location of the *COA1* gene is depicted by a red box.

Figure S379

Macaca mulatta

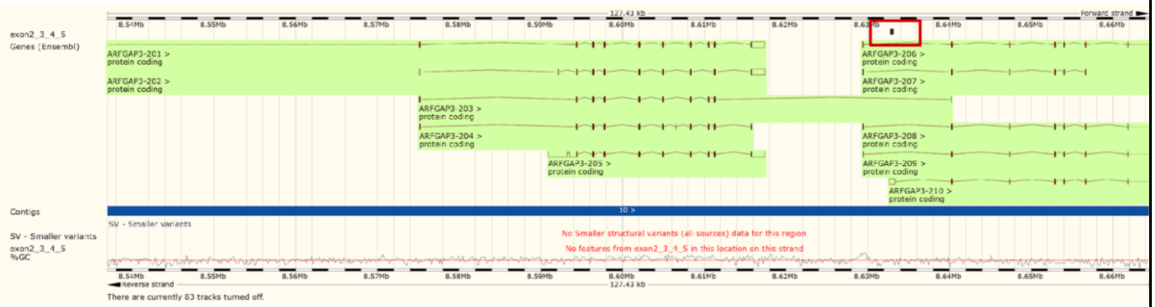

Figure S379: Gene order near *COA1* gene in the macaque genome. The rectangular boxes connected by thin lines depict genes, with the boxes denoting exons and connecting lines being introns. Direction of transcription is denoted by the > or < symbols (signify positive and negative strand of transcription respectively) beside the gene names. chromosome bands and the genomic coordinates in kilobase (kb) are shown in the topmost row. Details of the contig and scaffold identity are provided in the blue bar located in the middle of the figure. Exact location of the *COA1* gene is depicted by a red box.

Figure S380

Macaca nemestrina

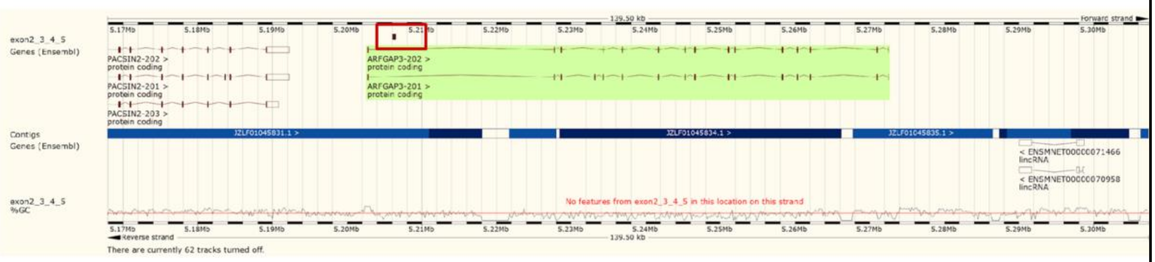

Figure S380: Gene order near *COA1* gene in the pig-tailed macaque genome. The rectangular boxes connected by thin lines depict genes, with the boxes denoting exons and connecting lines being introns. Direction of transcription is denoted by the > or < symbols (signify positive and negative strand of transcription respectively) beside the gene names. chromosome bands and the genomic coordinates in kilobase (kb) are shown in the topmost row. Details of the contig and scaffold identity are provided in the blue bar located in the middle of the figure. Exact location of the *COA1* gene is depicted by a red box.

Figure S381

Chlorocebus sabaeus

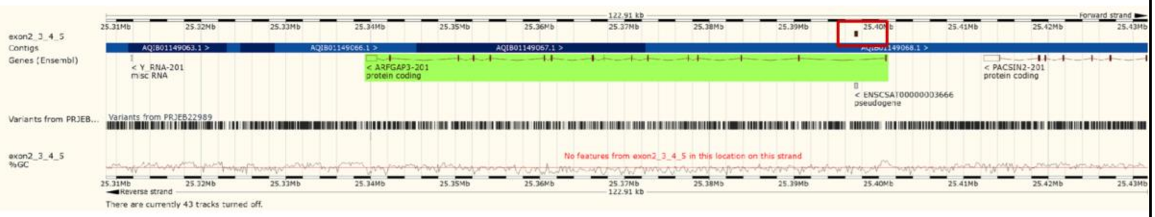

Figure S381: Gene order near *COA1* gene in the vervet-AGM genome. The rectangular boxes connected by thin lines depict genes, with the boxes denoting exons and connecting lines being introns. Direction of transcription is denoted by the > or < symbols (signify positive and negative strand of transcription respectively) beside the gene names. chromosome bands and the genomic coordinates in kilobase (kb) are shown in the topmost row. Details of the contig and scaffold identity are provided in the blue bar located in the middle of the figure. Exact location of the *COA1* gene is depicted by a red box.

Figure S382

Rhinopithecus bieti

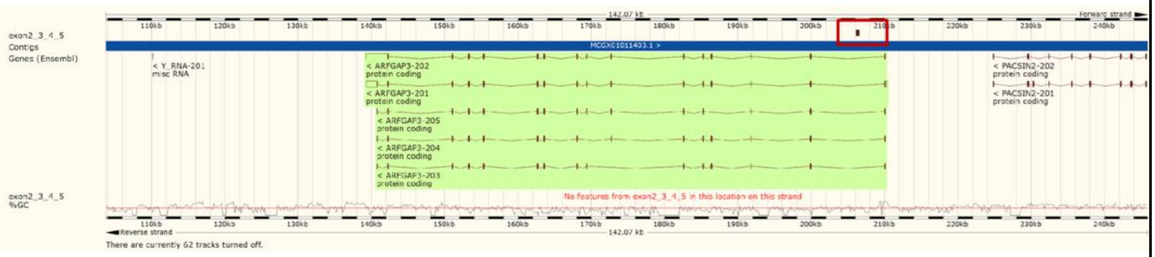

Figure S382: Gene order near *COA1* gene in the black snub-nosed monkey genome. The rectangular boxes connected by thin lines depict genes, with the boxes denoting exons and connecting lines being introns. Direction of transcription is denoted by the > or < symbols (signify positive and negative strand of transcription respectively) beside the gene names. chromosome bands and the genomic coordinates in kilobase (kb) are shown in the topmost row. Details of the contig and scaffold identity are provided in the blue bar located in the middle of the figure. Exact location of the *COA1* gene is depicted by a red box.

Figure S383

Rhinopithecus roxellana

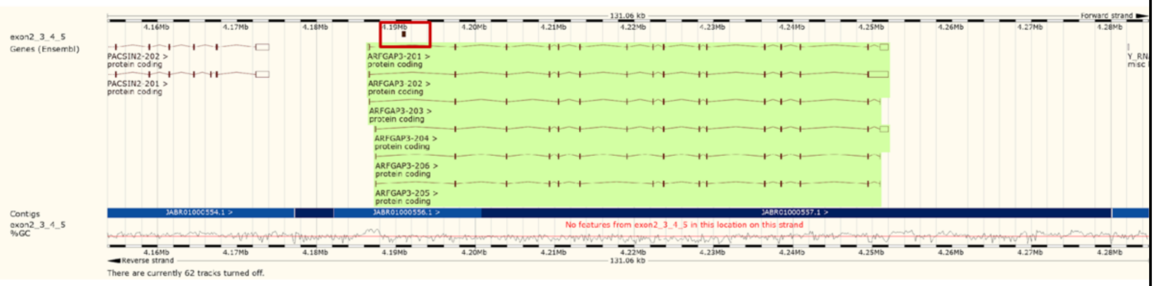

Figure S383: Gene order near *COA1* gene in the golden snub-nosed monkey genome. The rectangular boxes connected by thin lines depict genes, with the boxes denoting exons and connecting lines being introns. Direction of transcription is denoted by the > or < symbols (signify positive and negative strand of transcription respectively) beside the gene names. chromosome bands and the genomic coordinates in kilobase (kb) are shown in the topmost row. Details of the contig and scaffold identity are provided in the blue bar located in the middle of the figure. Exact location of the *COA1* gene is depicted by a red box.

Figure S384

Piliocolobus tephrosceles

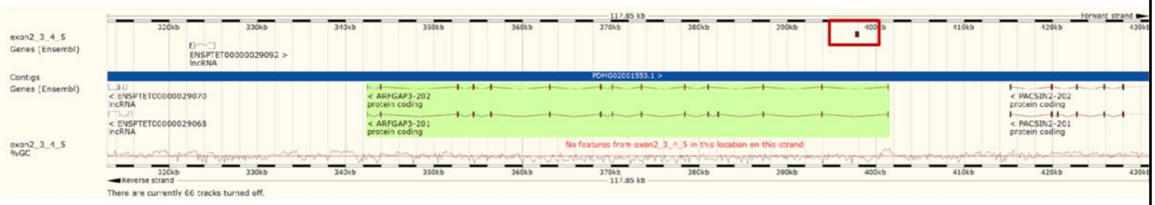

Figure S384: Gene order near *COA1* gene in the Ugandan red colobus genome. The rectangular boxes connected by thin lines depict genes, with the boxes denoting exons and connecting lines being introns. Direction of transcription is denoted by the > or < symbols (signify positive and negative strand of transcription respectively) beside the gene names. chromosome bands and the genomic coordinates in kilobase (kb) are shown in the topmost row. Details of the contig and scaffold identity are provided in the blue bar located in the middle of the figure. Exact location of the *COA1* gene is depicted by a red box.

Figure S385

Colobus angolensis palliatus

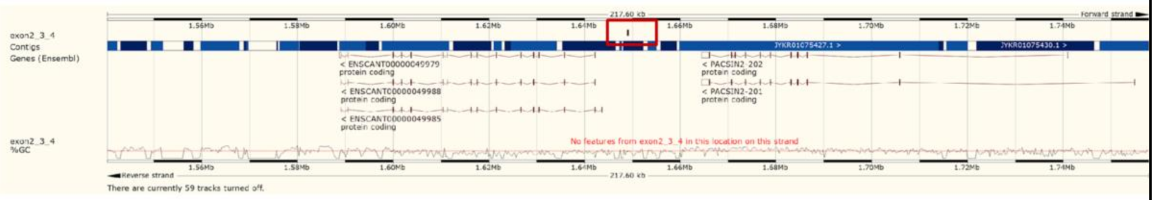

Figure S385: Gene order near *COA1* gene in the Angola colobus genome. The rectangular boxes connected by thin lines depict genes, with the boxes denoting exons and connecting lines being introns. Direction of transcription is denoted by the > or < symbols (signify positive and negative strand of transcription respectively) beside the gene names. chromosome bands and the genomic coordinates in kilobase (kb) are shown in the topmost row. Details of the contig and scaffold identity are provided in the blue bar located in the middle of the figure. Exact location of the *COA1* gene is depicted by a red box.

Figure S386

Nomascus leucogenys

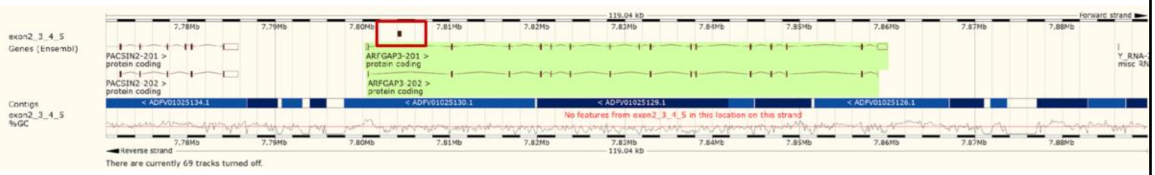

Figure S386: Gene order near *COA1* gene in the gibbon genome. The rectangular boxes connected by thin lines depict genes, with the boxes denoting exons and connecting lines being introns. Direction of transcription is denoted by the > or < symbols (signify positive and negative strand of transcription respectively) beside the gene names. chromosome bands and the genomic coordinates in kilobase (kb) are shown in the topmost row. Details of the contig and scaffold identity are provided in the blue bar located in the middle of the figure. Exact location of the *COA1* gene is depicted by a red box.

Figure S387

Gorilla gorilla gorilla

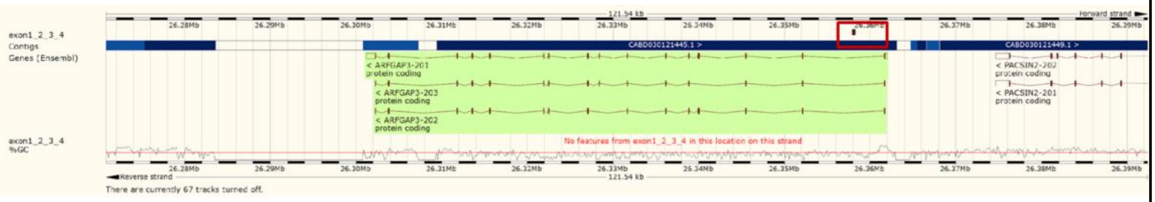

Figure S387: Gene order near *COA1* gene in the gorilla genome. The rectangular boxes connected by thin lines depict genes, with the boxes denoting exons and connecting lines being introns. Direction of transcription is denoted by the > or < symbols (signify positive and negative strand of transcription respectively) beside the gene names. chromosome bands and the genomic coordinates in kilobase (kb) are shown in the topmost row. Details of the contig and scaffold identity are provided in the blue bar located in the middle of the figure. Exact location of the *COA1* gene is depicted by a red box.

Figure S388

Pan troglodytes

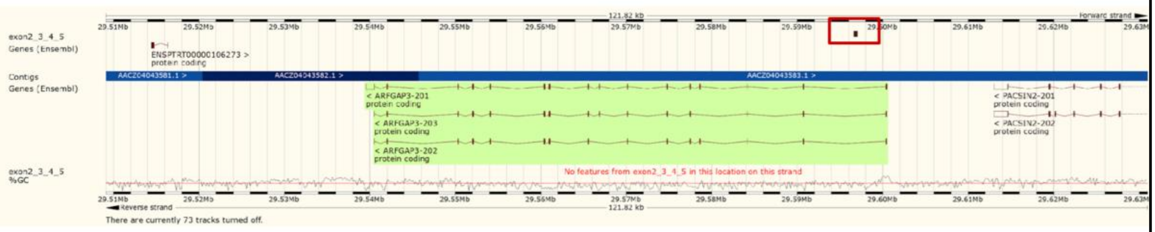

Figure S388: Gene order near *COA1* gene in the chimpanzee genome. The rectangular boxes connected by thin lines depict genes, with the boxes denoting exons and connecting lines being introns. Direction of transcription is denoted by the > or < symbols (signify positive and negative strand of transcription respectively) beside the gene names. chromosome bands and the genomic coordinates in kilobase (kb) are shown in the topmost row. Details of the contig and scaffold identity are provided in the blue bar located in the middle of the figure. Exact location of the *COA1* gene is depicted by a red box.

Figure S389

Pan paniscus

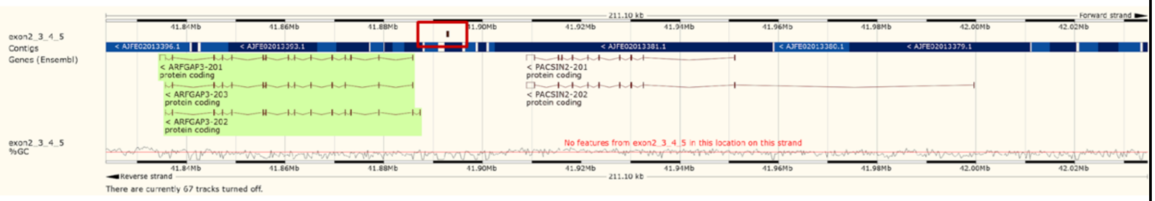

Figure S389: Gene order near *COA1* gene in the bonobo genome. The rectangular boxes connected by thin lines depict genes, with the boxes denoting exons and connecting lines being introns. Direction of transcription is denoted by the > or < symbols (signify positive and negative strand of transcription respectively) beside the gene names. chromosome bands and the genomic coordinates in kilobase (kb) are shown in the topmost row. Details of the contig and scaffold identity are provided in the blue bar located in the middle of the figure. Exact location of the *COA1* gene is depicted by a red box.

Figure S390

Homo sapiens

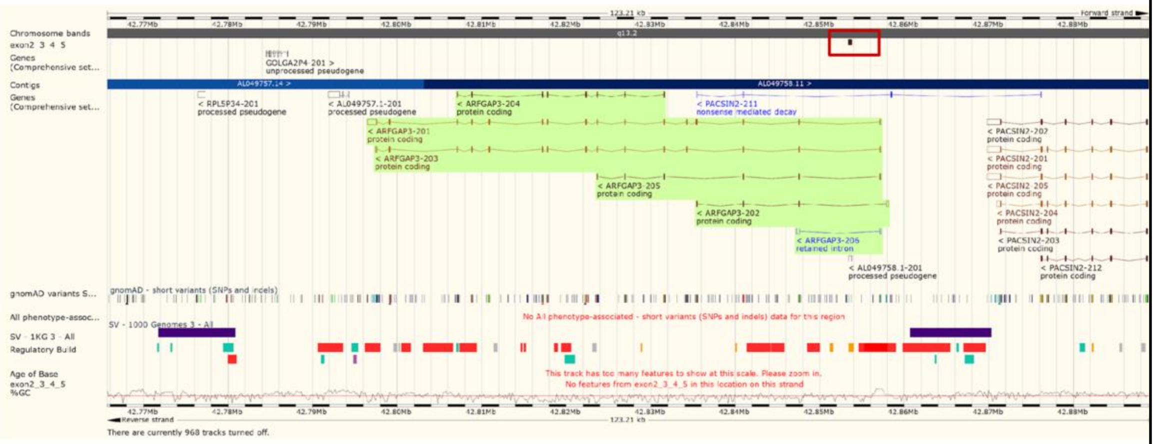

Figure S390: Gene order near *COA1* gene in the human genome. The rectangular boxes connected by thin lines depict genes, with the boxes denoting exons and connecting lines being introns. Direction of transcription is denoted by the > or < symbols (signify positive and negative strand of transcription respectively) beside the gene names. chromosome bands and the genomic coordinates in kilobase (kb) are shown in the topmost row. Details of the contig and scaffold identity are provided in the blue bar located in the middle of the figure. Exact location of the *COA1* gene is depicted by a red box.

Figure S391

Pongo abelii

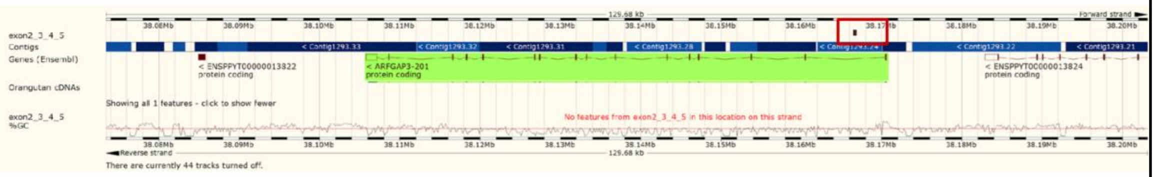

Figure S391: Gene order near *COA1* gene in the orangutan genome. The rectangular boxes connected by thin lines depict genes, with the boxes denoting exons and connecting lines being introns. Direction of transcription is denoted by the > or < symbols (signify positive and negative strand of transcription respectively) beside the gene names. chromosome bands and the genomic coordinates in kilobase (kb) are shown in the topmost row. Details of the contig and scaffold identity are provided in the blue bar located in the middle of the figure. Exact location of the *COA1* gene is depicted by a red box.

Figure S392

Tupaia belangeri

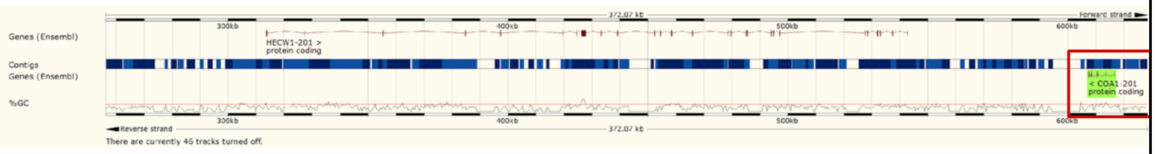

Figure S392: Gene order near *COA1* gene in the tree shrew genome. The rectangular boxes connected by thin lines depict genes, with the boxes denoting exons and connecting lines being introns. Direction of transcription is denoted by the > or < symbols (signify positive and negative strand of transcription respectively) beside the gene names. chromosome bands and the genomic coordinates in kilobase (kb) are shown in the topmost row. Details of the contig and scaffold identity are provided in the blue bar located in the middle of the figure. Exact location of the *COA1* gene is depicted by a red box.

Figure S393

Ursus thibetanus thibetanus

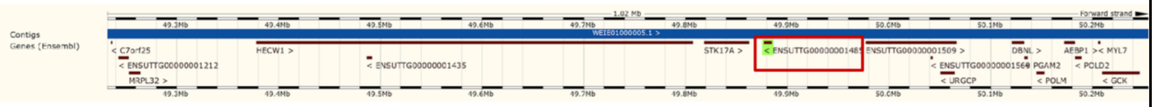

Figure S393: Gene order near *COA1* gene in the American black bear genome. The rectangular boxes connected by thin lines depict genes, with the boxes denoting exons and connecting lines being introns. Direction of transcription is denoted by the > or < symbols (signify positive and negative strand of transcription respectively) beside the gene names. chromosome bands and the genomic coordinates in kilobase (kb) are shown in the topmost row. Details of the contig and scaffold identity are provided in the blue bar located in the middle of the figure. Exact location of the *COA1* gene is depicted by a red box.

Figure S394

Canis lupus dingo

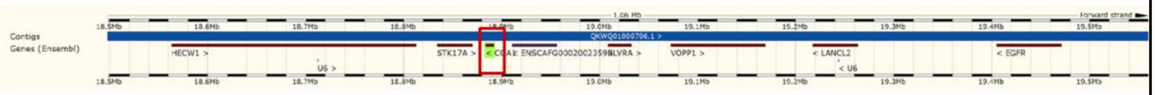

Figure S394: Gene order near *COA1* gene in the dingo genome. The rectangular boxes connected by thin lines depict genes, with the boxes denoting exons and connecting lines being introns. Direction of transcription is denoted by the > or < symbols (signify positive and negative strand of transcription respectively) beside the gene names. chromosome bands and the genomic coordinates in kilobase (kb) are shown in the topmost row. Details of the contig and scaffold identity are provided in the blue bar located in the middle of the figure. Exact location of the *COA1* gene is depicted by a red box.

Figure S395

Mustela putorius furo

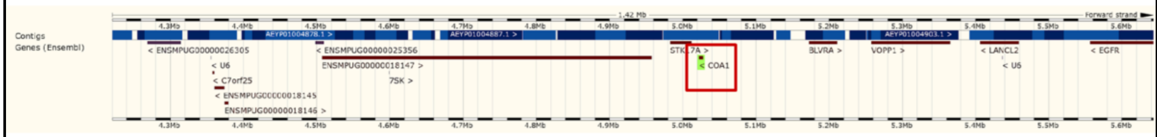

Figure S395: Gene order near *COA1* gene in the ferret genome. The rectangular boxes connected by thin lines depict genes, with the boxes denoting exons and connecting lines being introns. Direction of transcription is denoted by the > or < symbols (signify positive and negative strand of transcription respectively) beside the gene names. chromosome bands and the genomic coordinates in kilobase (kb) are shown in the topmost row. Details of the contig and scaffold identity are provided in the blue bar located in the middle of the figure. Exact location of the *COA1* gene is depicted by a red box.

Figure S396

Ursus maritimus

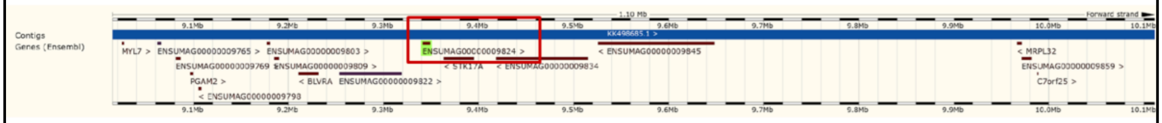

Figure S396: Gene order near *COA1* gene in the polar bear genome. The rectangular boxes connected by thin lines depict genes, with the boxes denoting exons and connecting lines being introns. Direction of transcription is denoted by the > or < symbols (signify positive and negative strand of transcription respectively) beside the gene names. chromosome bands and the genomic coordinates in kilobase (kb) are shown in the topmost row. Details of the contig and scaffold identity are provided in the blue bar located in the middle of the figure. Exact location of the *COA1* gene is depicted by a red box.

Figure S397

Vulpes vulpes

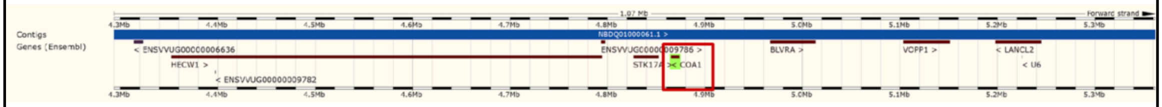

Figure S397: Gene order near *COA1* gene in the red fox genome. The rectangular boxes connected by thin lines depict genes, with the boxes denoting exons and connecting lines being introns. Direction of transcription is denoted by the > or < symbols (signify positive and negative strand of transcription respectively) beside the gene names. chromosome bands and the genomic coordinates in kilobase (kb) are shown in the topmost row. Details of the contig and scaffold identity are provided in the blue bar located in the middle of the figure. Exact location of the *COA1* gene is depicted by a red box.

Figure S398

Camelus dromedarius

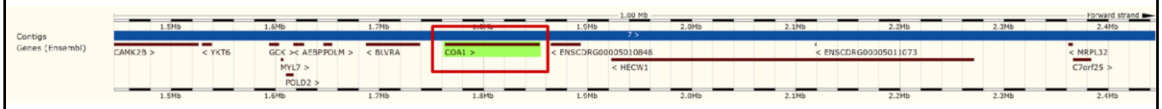

Figure S398: Gene order near *COA1* gene in the Arabian camel genome. The rectangular boxes connected by thin lines depict genes, with the boxes denoting exons and connecting lines being introns. Direction of transcription is denoted by the > or < symbols (signify positive and negative strand of transcription respectively) beside the gene names. chromosome bands and the genomic coordinates in kilobase (kb) are shown in the topmost row. Details of the contig and scaffold identity are provided in the blue bar located in the middle of the figure. Exact location of the *COA1* gene is depicted by a red box.

Figure S399

Dasypus novemcinctus

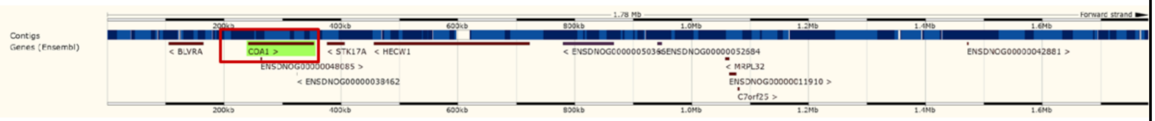

Figure S399: Gene order near *COA1* gene in the armadillo genome. The rectangular boxes connected by thin lines depict genes, with the boxes denoting exons and connecting lines being introns. Direction of transcription is denoted by the > or < symbols (signify positive and negative strand of transcription respectively) beside the gene names. chromosome bands and the genomic coordinates in kilobase (kb) are shown in the topmost row. Details of the contig and scaffold identity are provided in the blue bar located in the middle of the figure. Exact location of the *COA1* gene is depicted by a red box.

Figure S400

Delphinapterus leucas

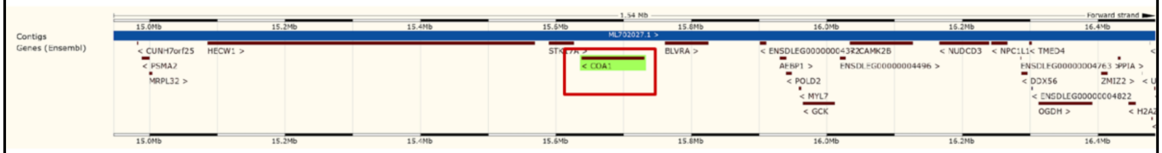

Figure S400: Gene order near *COA1* gene in the Beluga whale genome. The rectangular boxes connected by thin lines depict genes, with the boxes denoting exons and connecting lines being introns. Direction of transcription is denoted by the > or < symbols (signify positive and negative strand of transcription respectively) beside the gene names. chromosome bands and the genomic coordinates in kilobase (kb) are shown in the topmost row. Details of the contig and scaffold identity are provided in the blue bar located in the middle of the figure. Exact location of the *COA1* gene is depicted by a red box.

Figure S401

Balaenoptera musculus

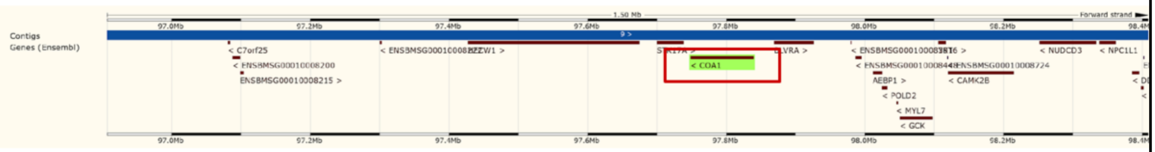

Figure S401: Gene order near *COA1* gene in the blue whale genome. The rectangular boxes connected by thin lines depict genes, with the boxes denoting exons and connecting lines being introns. Direction of transcription is denoted by the > or < symbols (signify positive and negative strand of transcription respectively) beside the gene names. chromosome bands and the genomic coordinates in kilobase (kb) are shown in the topmost row. Details of the contig and scaffold identity are provided in the blue bar located in the middle of the figure. Exact location of the *COA1* gene is depicted by a red box.

Figure S402

Catagonus wagneri

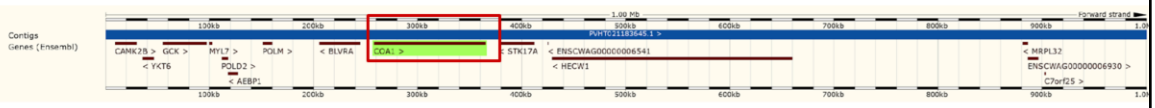

Figure S402: Gene order near *COA1* gene in the chacoan peccary genome. The rectangular boxes connected by thin lines depict genes, with the boxes denoting exons and connecting lines being introns. Direction of transcription is denoted by the > or < symbols (signify positive and negative strand of transcription respectively) beside the gene names. chromosome bands and the genomic coordinates in kilobase (kb) are shown in the topmost row. Details of the contig and scaffold identity are provided in the blue bar located in the middle of the figure. Exact location of the *COA1* gene is depicted by a red box.

Figure S403

Tursiops truncatus

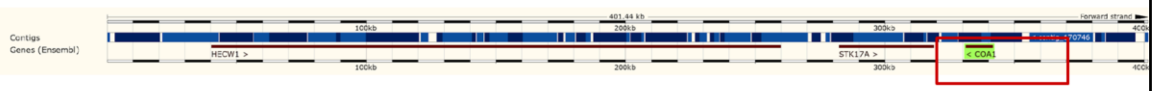

Figure S403: Gene order near *COA1* gene in the dolphin genome. The rectangular boxes connected by thin lines depict genes, with the boxes denoting exons and connecting lines being introns. Direction of transcription is denoted by the > or < symbols (signify positive and negative strand of transcription respectively) beside the gene names. chromosome bands and the genomic coordinates in kilobase (kb) are shown in the topmost row. Details of the contig and scaffold identity are provided in the blue bar located in the middle of the figure. Exact location of the *COA1* gene is depicted by a red box.

Figure S404

Bos grunniens

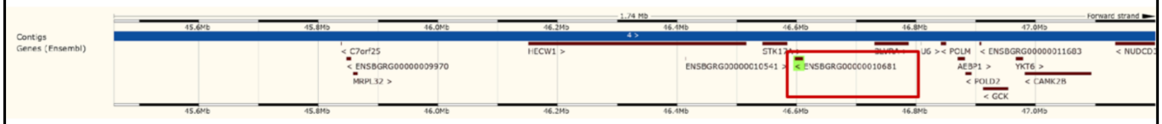

Figure S404: Gene order near *COA1* gene in the domestic yalk genome. The rectangular boxes connected by thin lines depict genes, with the boxes denoting exons and connecting lines being introns. Direction of transcription is denoted by the > or < symbols (signify positive and negative strand of transcription respectively) beside the gene names. chromosome bands and the genomic coordinates in kilobase (kb) are shown in the topmost row. Details of the contig and scaffold identity are provided in the blue bar located in the middle of the figure. Exact location of the *COA1* gene is depicted by a red box.

Figure S405

Equus asinus asinus

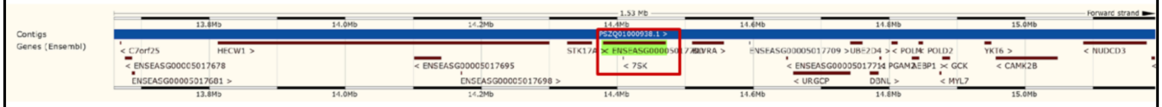

Figure S405: Gene order near *COA1* gene in the donkey genome. The rectangular boxes connected by thin lines depict genes, with the boxes denoting exons and connecting lines being introns. Direction of transcription is denoted by the > or < symbols (signify positive and negative strand of transcription respectively) beside the gene names. chromosome bands and the genomic coordinates in kilobase (kb) are shown in the topmost row. Details of the contig and scaffold identity are provided in the blue bar located in the middle of the figure. Exact location of the *COA1* gene is depicted by a red box.

Figure S406

Capra hircus

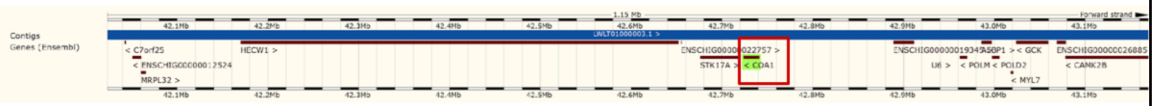

Figure S406: Gene order near *COA1* gene in the goat genome. The rectangular boxes connected by thin lines depict genes, with the boxes denoting exons and connecting lines being introns. Direction of transcription is denoted by the > or < symbols (signify positive and negative strand of transcription respectively) beside the gene names. chromosome bands and the genomic coordinates in kilobase (kb) are shown in the topmost row. Details of the contig and scaffold identity are provided in the blue bar located in the middle of the figure. Exact location of the *COA1* gene is depicted by a red box.

Figure S407

Rhinolophus ferrumequinum

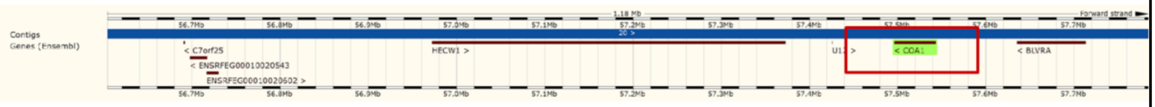

Figure S408: Gene order near *COA1* gene in the greater horseshoe bat genome. The rectangular boxes connected by thin lines depict genes, with the boxes denoting exons and connecting lines being introns. Direction of transcription is denoted by the > or < symbols (signify positive and negative strand of transcription respectively) beside the gene names. chromosome bands and the genomic coordinates in kilobase (kb) are shown in the topmost row. Details of the contig and scaffold identity are provided in the blue bar located in the middle of the figure. Exact location of the *COA1* gene is depicted by a red box.

Figure S408

Erinaceus europaeus

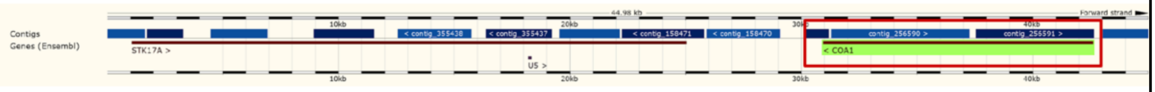

Figure S408: Gene order near *COA1* gene in the hedgehog genome. The rectangular boxes connected by thin lines depict genes, with the boxes denoting exons and connecting lines being introns. Direction of transcription is denoted by the > or < symbols (signify positive and negative strand of transcription respectively) beside the gene names. chromosome bands and the genomic coordinates in kilobase (kb) are shown in the topmost row. Details of the contig and scaffold identity are provided in the blue bar located in the middle of the figure. Exact location of the *COA1* gene is depicted by a red box.

Figure S409 *Equus caballus*

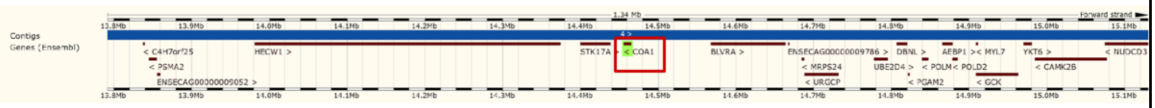

Figure S409: Gene order near *COA1* gene in the horse genome. The rectangular boxes connected by thin lines depict genes, with the boxes denoting exons and connecting lines being introns. Direction of transcription is denoted by the > or < symbols (signify positive and negative strand of transcription respectively) beside the gene names. chromosome bands and the genomic coordinates in kilobase (kb) are shown in the topmost row. Details of the contig and scaffold identity are provided in the blue bar located in the middle of the figure. Exact location of the *COA1* gene is depicted by a red box.

Figure S410

Myotis lucifugus

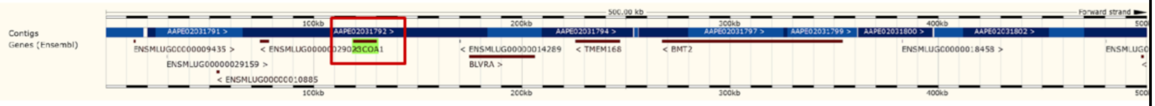

Figure S410: Gene order near *COA1* gene in the microbat genome. The rectangular boxes connected by thin lines depict genes, with the boxes denoting exons and connecting lines being introns. Direction of transcription is denoted by the > or < symbols (signify positive and negative strand of transcription respectively) beside the gene names. chromosome bands and the genomic coordinates in kilobase (kb) are shown in the topmost row. Details of the contig and scaffold identity are provided in the blue bar located in the middle of the figure. Exact location of the *COA1* gene is depicted by a red box.

Figure S411

Monodon monoceros

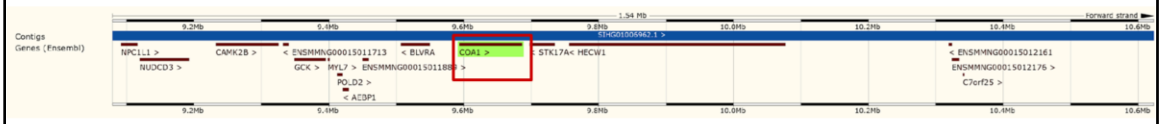

Figure S411: Gene order near *COA1* gene in the narwhal genome. The rectangular boxes connected by thin lines depict genes, with the boxes denoting exons and connecting lines being introns. Direction of transcription is denoted by the > or < symbols (signify positive and negative strand of transcription respectively) beside the gene names. chromosome bands and the genomic coordinates in kilobase (kb) are shown in the topmost row. Details of the contig and scaffold identity are provided in the blue bar located in the middle of the figure. Exact location of the *COA1* gene is depicted by a red box.

Figure S412

Sus scrofa

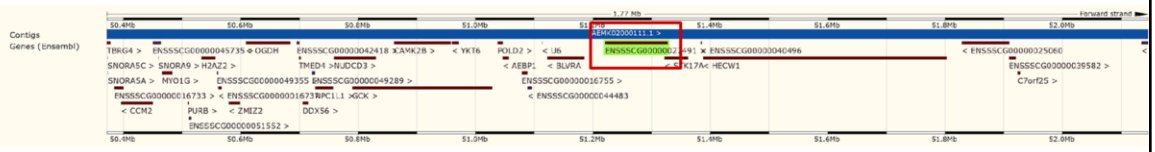

Figure S412: Gene order near *COA1* gene in the pig genome. The rectangular boxes connected by thin lines depict genes, with the boxes denoting exons and connecting lines being introns. Direction of transcription is denoted by the > or < symbols (signify positive and negative strand of transcription respectively) beside the gene names. chromosome bands and the genomic coordinates in kilobase (kb) are shown in the topmost row. Details of the contig and scaffold identity are provided in the blue bar located in the middle of the figure. Exact location of the *COA1* gene is depicted by a red box.

Figure S413

Ovis aries

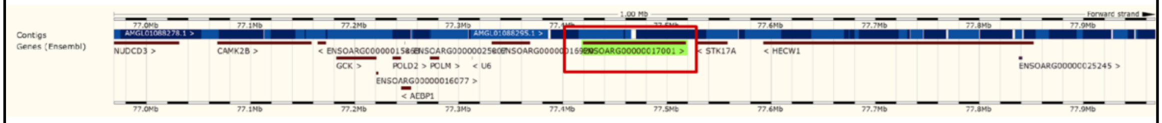

Figure S413: Gene order near *COA1* gene in the sheep genome. The rectangular boxes connected by thin lines depict genes, with the boxes denoting exons and connecting lines being introns. Direction of transcription is denoted by the > or < symbols (signify positive and negative strand of transcription respectively) beside the gene names. chromosome bands and the genomic coordinates in kilobase (kb) are shown in the topmost row. Details of the contig and scaffold identity are provided in the blue bar located in the middle of the figure. Exact location of the *COA1* gene is depicted by a red box.

Figure S414

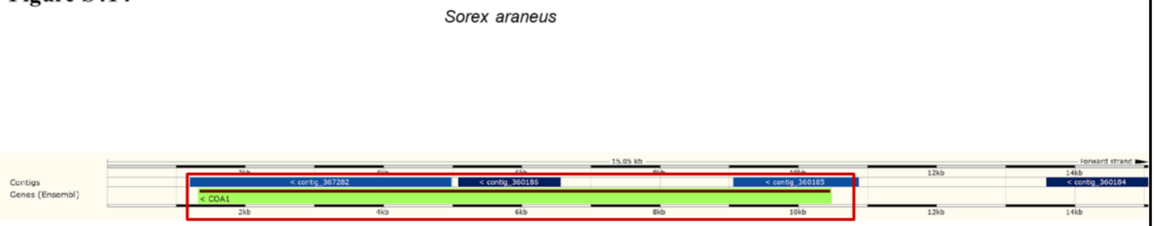

Figure S414: Gene order near *COA1* gene in the shrew genome. The rectangular boxes connected by thin lines depict genes, with the boxes denoting exons and connecting lines being introns. Direction of transcription is denoted by the > or < symbols (signify positive and negative strand of transcription respectively) beside the gene names. chromosome bands and the genomic coordinates in kilobase (kb) are shown in the topmost row. Details of the contig and scaffold identity are provided in the blue bar located in the middle of the figure. Exact location of the *COA1* gene is depicted by a red box.

Figure S415

Moschus moschiferus

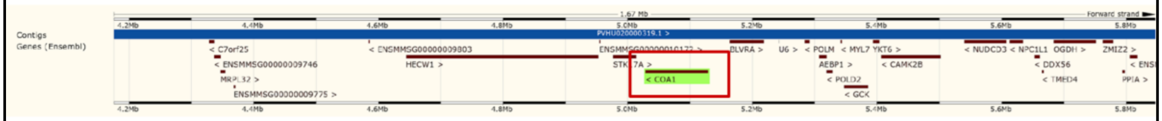

Figure S415: Gene order near *COA1* gene in the Siberian musk deer genome. The rectangular boxes connected by thin lines depict genes, with the boxes denoting exons and connecting lines being introns. Direction of transcription is denoted by the > or < symbols (signify positive and negative strand of transcription respectively) beside the gene names. chromosome bands and the genomic coordinates in kilobase (kb) are shown in the topmost row. Details of the contig and scaffold identity are provided in the blue bar located in the middle of the figure. Exact location of the *COA1* gene is depicted by a red box.

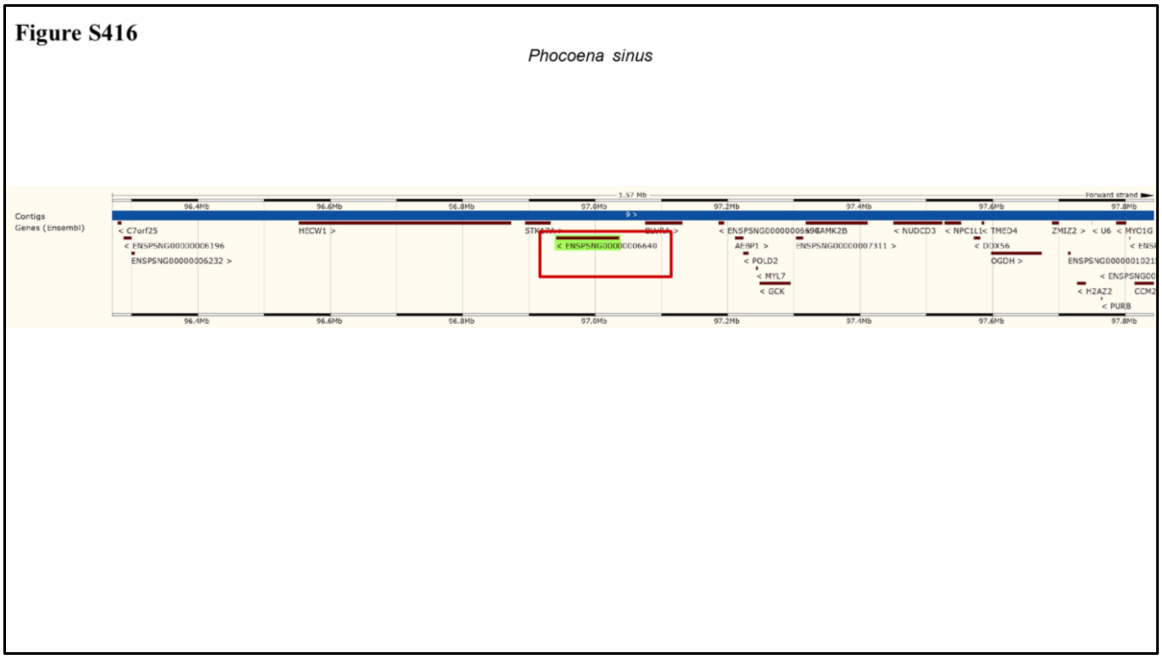

Figure S416: Gene order near *COA1* gene in the Vaquita genome. The rectangular boxes connected by thin lines depict genes, with the boxes denoting exons and connecting lines being introns. Direction of transcription is denoted by the > or < symbols (signify positive and negative strand of transcription respectively) beside the gene names. chromosome bands and the genomic coordinates in kilobase (kb) are shown in the topmost row. Details of the contig and scaffold identity are provided in the blue bar located in the middle of the figure. Exact location of the *COA1* gene is depicted by a red box.

Figure S417

Bos mutus

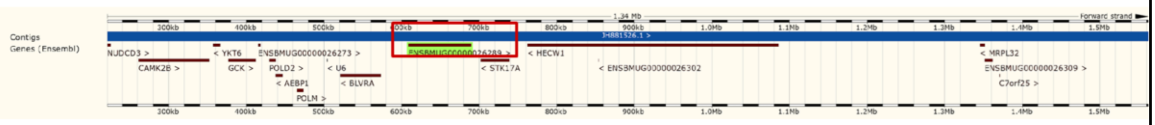

Figure S417: Gene order near *COA1* gene in the wild yak genome. The rectangular boxes connected by thin lines depict genes, with the boxes denoting exons and connecting lines being introns. Direction of transcription is denoted by the > or < symbols (signify positive and negative strand of transcription respectively) beside the gene names. chromosome bands and the genomic coordinates in kilobase (kb) are shown in the topmost row. Details of the contig and scaffold identity are provided in the blue bar located in the middle of the figure. Exact location of the *COA1* gene is depicted by a red box.

Figure S418

Cervus hanglu yarkandensis

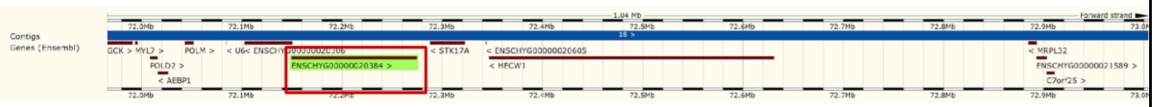

Figure S418: Gene order near *COA1* gene in the Yarkand deer genome. The rectangular boxes connected by thin lines depict genes, with the boxes denoting exons and connecting lines being introns. Direction of transcription is denoted by the > or < symbols (signify positive and negative strand of transcription respectively) beside the gene names. chromosome bands and the genomic coordinates in kilobase (kb) are shown in the topmost row. Details of the contig and scaffold identity are provided in the blue bar located in the middle of the figure. Exact location of the *COA1* gene is depicted by a red box.

Figure S419 Reptiles group *Salvator merianae*

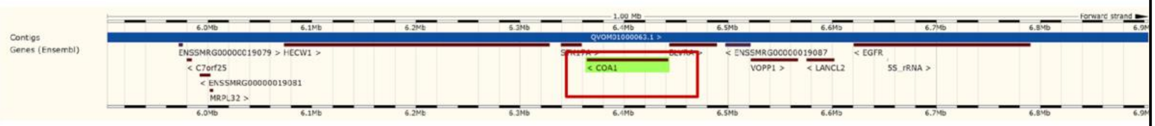

Figure S419: Gene order near *COA1* gene in the Argentine black and white tegu genome. The rectangular boxes connected by thin lines depict genes, with the boxes denoting exons and connecting lines being introns. Direction of transcription is denoted by the > or < symbols (signify positive and negative strand of transcription respectively) beside the gene names. chromosome bands and the genomic coordinates in kilobase (kb) are shown in the topmost row. Details of the contig and scaffold identity are provided in the blue bar located in the middle of the figure. Exact location of the *COA1* gene is depicted by a red box.

Figure S420

Pelodiscus sinensis

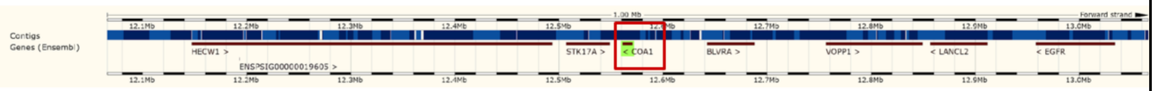

Figure S420: Gene order near *COA1* gene in the Chinese softshell turtle genome. The rectangular boxes connected by thin lines depict genes, with the boxes denoting exons and connecting lines being introns. Direction of transcription is denoted by the > or < symbols (signify positive and negative strand of transcription respectively) beside the gene names. chromosome bands and the genomic coordinates in kilobase (kb) are shown in the topmost row. Details of the contig and scaffold identity are provided in the blue bar located in the middle of the figure. Exact location of the *COA1* gene is depicted by a red box.

Figure S421

Chelydra serpentina

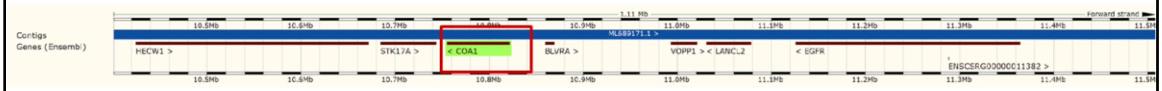

Figure S421: Gene order near *COA1* gene in the common snapping turtle genome. The rectangular boxes connected by thin lines depict genes, with the boxes denoting exons and connecting lines being introns. Direction of transcription is denoted by the > or < symbols (signify positive and negative strand of transcription respectively) beside the gene names. chromosome bands and the genomic coordinates in kilobase (kb) are shown in the topmost row. Details of the contig and scaffold identity are provided in the blue bar located in the middle of the figure. Exact location of the *COA1* gene is depicted by a red box.

Figure S422

Pseudonaja textilis

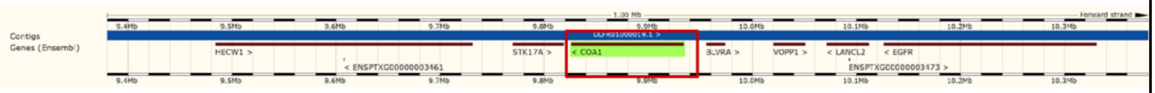

Figure S422: Gene order near *COA1* gene in the Eastern brown snake genome. The rectangular boxes connected by thin lines depict genes, with the boxes denoting exons and connecting lines being introns. Direction of transcription is denoted by the > or < symbols (signify positive and negative strand of transcription respectively) beside the gene names. chromosome bands and the genomic coordinates in kilobase (kb) are shown in the topmost row. Details of the contig and scaffold identity are provided in the blue bar located in the middle of the figure. Exact location of the *COA1* gene is depicted by a red box.

Figure S423

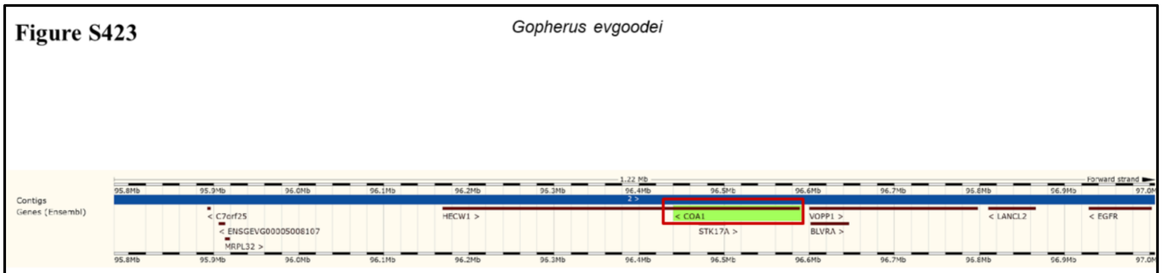

Figure S423: Gene order near *COA1* gene in the Goodes thornscrub tortoise genome. The rectangular boxes connected by thin lines depict genes, with the boxes denoting exons and connecting lines being introns. Direction of transcription is denoted by the > or < symbols (signify positive and negative strand of transcription respectively) beside the gene names. chromosome bands and the genomic coordinates in kilobase (kb) are shown in the topmost row. Details of the contig and scaffold identity are provided in the blue bar located in the middle of the figure. Exact location of the *COA1* gene is depicted by a red box.

Figure S424

Varanus komodoensis

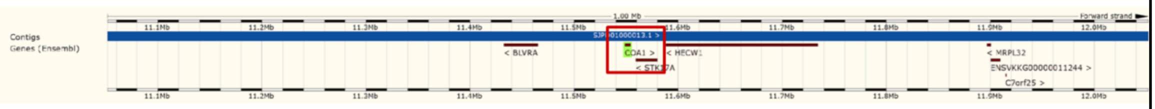

Figure S424: Gene order near *COA1* gene in the komodo dragon genome. The rectangular boxes connected by thin lines depict genes, with the boxes denoting exons and connecting lines being introns. Direction of transcription is denoted by the > or < symbols (signify positive and negative strand of transcription respectively) beside the gene names. chromosome bands and the genomic coordinates in kilobase (kb) are shown in the topmost row. Details of the contig and scaffold identity are provided in the blue bar located in the middle of the figure. Exact location of the *COA1* gene is depicted by a red box.

Figure S425

Notechis scutatus

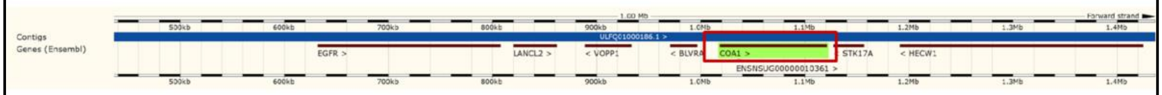

Figure S425: Gene order near *COA1* gene in the mainland tiger snake genome. The rectangular boxes connected by thin lines depict genes, with the boxes denoting exons and connecting lines being introns. Direction of transcription is denoted by the > or < symbols (signify positive and negative strand of transcription respectively) beside the gene names. chromosome bands and the genomic coordinates in kilobase (kb) are shown in the topmost row. Details of the contig and scaffold identity are provided in the blue bar located in the middle of the figure. Exact location of the *COA1* gene is depicted by a red box.

Figure S426

Pelusios castaneus

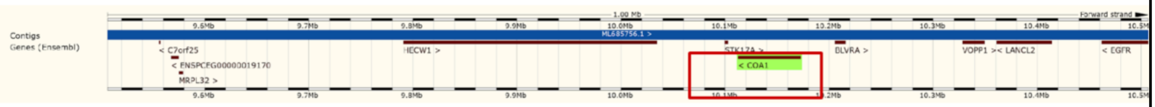

Figure S426: Gene order near *COA1* gene in the West African mud turtle genome. The rectangular boxes connected by thin lines depict genes, with the boxes denoting exons and connecting lines being introns. Direction of transcription is denoted by the > or < symbols (signify positive and negative strand of transcription respectively) beside the gene names. chromosome bands and the genomic coordinates in kilobase (kb) are shown in the topmost row. Details of the contig and scaffold identity are provided in the blue bar located in the middle of the figure. Exact location of the *COA1* gene is depicted by a red box.

Figure S427

Crocodylus porosus

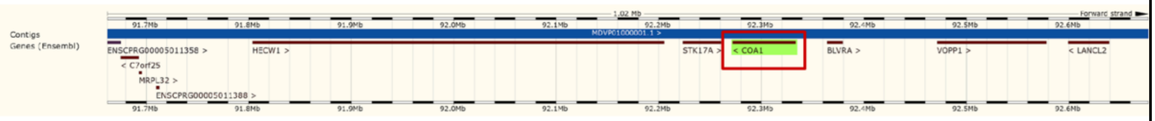

Figure S427: Gene order near *COA1* gene in the Australian saltwater crocodile genome. The rectangular boxes connected by thin lines depict genes, with the boxes denoting exons and connecting lines being introns. Direction of transcription is denoted by the > or < symbols (signify positive and negative strand of transcription respectively) beside the gene names. chromosome bands and the genomic coordinates in kilobase (kb) are shown in the topmost row. Details of the contig and scaffold identity are provided in the blue bar located in the middle of the figure. Exact location of the *COA1* gene is depicted by a red box.

Figure S428

human - Flycatcher

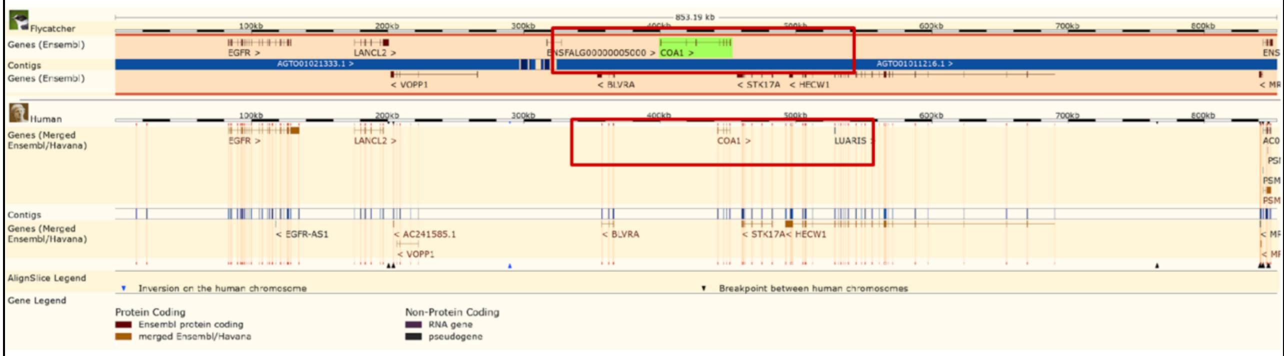

Figure S428: Gene order near *COA1* gene in the flycatcher genome. The rectangular boxes connected by thin lines depict genes, with the boxes denoting exons and connecting lines being introns. Direction of transcription is denoted by the > or < symbols (signify positive and negative strand of transcription respectively) beside the gene names. chromosome bands and the genomic coordinates in kilobase (kb) are shown in the topmost row. Details of the contig and scaffold identity are provided in the blue bar located in the middle of the figure. Exact location of the *COA1* gene is depicted by a red box.

Figure S429 human – Chilean tinamou

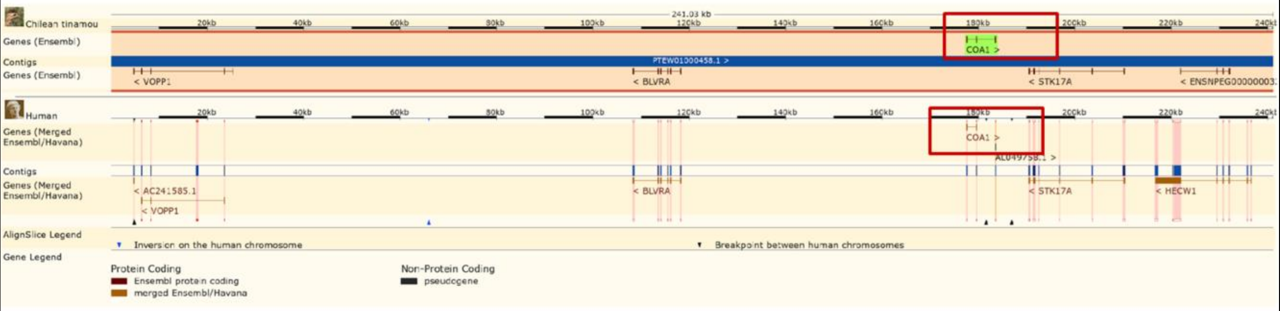

Figure S429: Gene order near *COA1* gene in the Chilean tinamou genome. The rectangular boxes connected by thin lines depict genes, with the boxes denoting exons and connecting lines being introns. Direction of transcription is denoted by the > or < symbols (signify positive and negative strand of transcription respectively) beside the gene names. chromosome bands and the genomic coordinates in kilobase (kb) are shown in the topmost row. Details of the contig and scaffold identity are provided in the blue bar located in the middle of the figure. Exact location of the *COA1* gene is depicted by a red box.

Figure S430

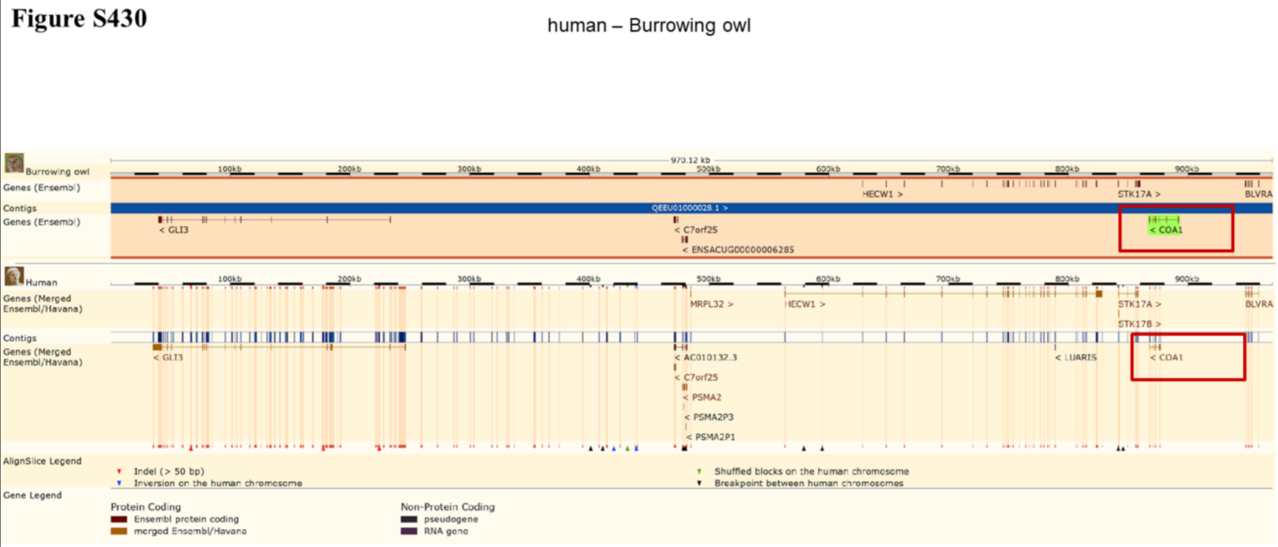

Figure S430: Gene order near *COA1* gene in the burrowing owl genome. The rectangular boxes connected by thin lines depict genes, with the boxes denoting exons and connecting lines being introns. Direction of transcription is denoted by the > or < symbols (signify positive and negative strand of transcription respectively) beside the gene names. chromosome bands and the genomic coordinates in kilobase (kb) are shown in the topmost row. Details of the contig and scaffold identity are provided in the blue bar located in the middle of the figure. Exact location of the *COA1* gene is depicted by a red box.

Figure S431

human – Blue-crowned manakin

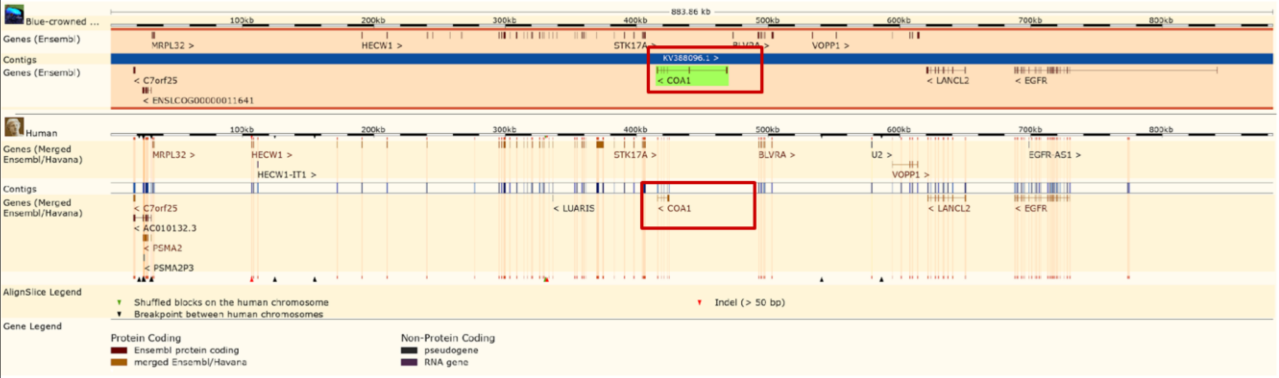

Figure S431: Gene order near *COA1* gene in the blue-crowned manakin genome. The rectangular boxes connected by thin lines depict genes, with the boxes denoting exons and connecting lines being introns. Direction of transcription is denoted by the > or < symbols (signify positive and negative strand of transcription respectively) beside the gene names. chromosome bands and the genomic coordinates in kilobase (kb) are shown in the topmost row. Details of the contig and scaffold identity are provided in the blue bar located in the middle of the figure. Exact location of the *COA1* gene is depicted by a red box.

Figure S432

human – Damara mole-rat

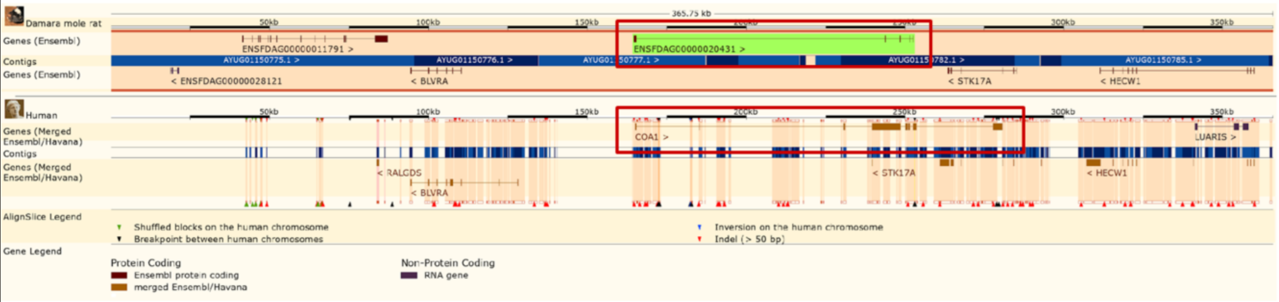

Figure S432: Gene order near *COA1* gene in the damara mole-rat genome. The rectangular boxes connected by thin lines depict genes, with the boxes denoting exons and connecting lines being introns. Direction of transcription is denoted by the > or < symbols (signify positive and negative strand of transcription respectively) beside the gene names. chromosome bands and the genomic coordinates in kilobase (kb) are shown in the topmost row. Details of the contig and scaffold identity are provided in the blue bar located in the middle of the figure. Exact location of the *COA1* gene is depicted by a red box.

Figure S433

human – Pika

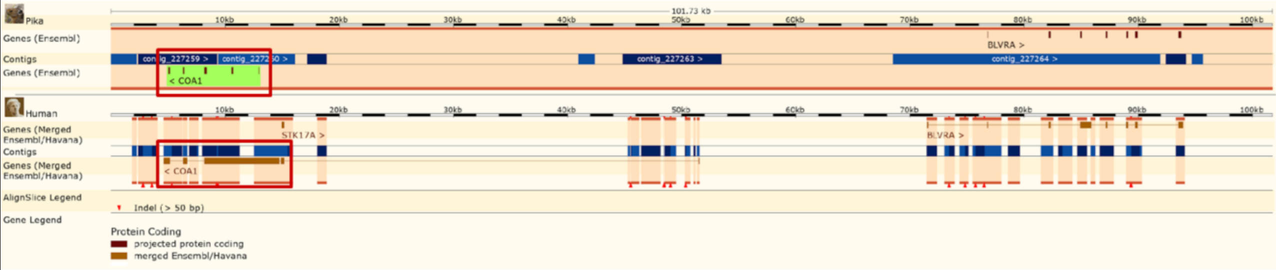

Figure S433: Gene order near *COA1* gene in the golden eagle genome. The rectangular boxes connected by thin lines depict genes, with the boxes denoting exons and connecting lines being introns. Direction of transcription is denoted by the > or < symbols (signify positive and negative strand of transcription respectively) beside the gene names. chromosome bands and the genomic coordinates in kilobase (kb) are shown in the topmost row. Details of the contig and scaffold identity are provided in the blue bar located in the middle of the figure. Exact location of the *COA1* gene is depicted by a red box.

Figure S434

human – Guinea pig

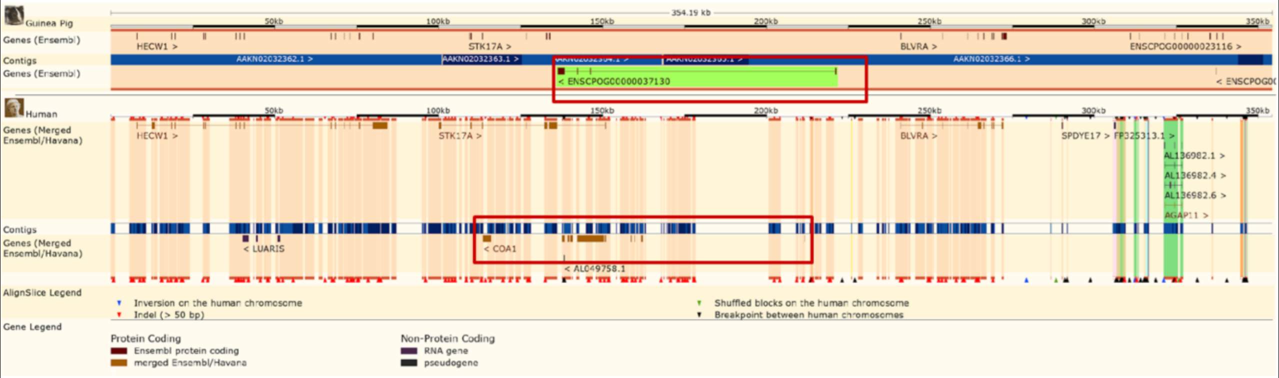

Figure S434: Gene order near *COA1* gene in the guinea pig genome. The rectangular boxes connected by thin lines depict genes, with the boxes denoting exons and connecting lines being introns. Direction of transcription is denoted by the > or < symbols (signify positive and negative strand of transcription respectively) beside the gene names. chromosome bands and the genomic coordinates in kilobase (kb) are shown in the topmost row. Details of the contig and scaffold identity are provided in the blue bar located in the middle of the figure. Exact location of the *COA1* gene is depicted by a red box.

Figure S435

human – Eurasian red squirrel

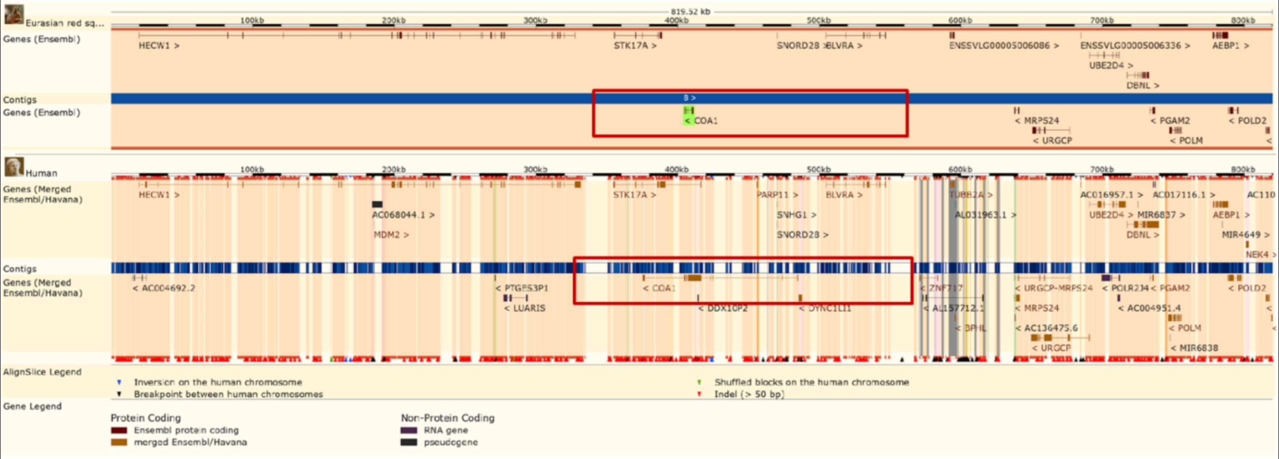

Figure S435: Gene order near *COA1* gene in the Eurasian red squirrel genome. The rectangular boxes connected by thin lines depict genes, with the boxes denoting exons and connecting lines being introns. Direction of transcription is denoted by the > or < symbols (signify positive and negative strand of transcription respectively) beside the gene names. chromosome bands and the genomic coordinates in kilobase (kb) are shown in the topmost row. Details of the contig and scaffold identity are provided in the blue bar located in the middle of the figure. Exact location of the *COA1* gene is depicted by a red box.

Figure S436

human - Kakapo

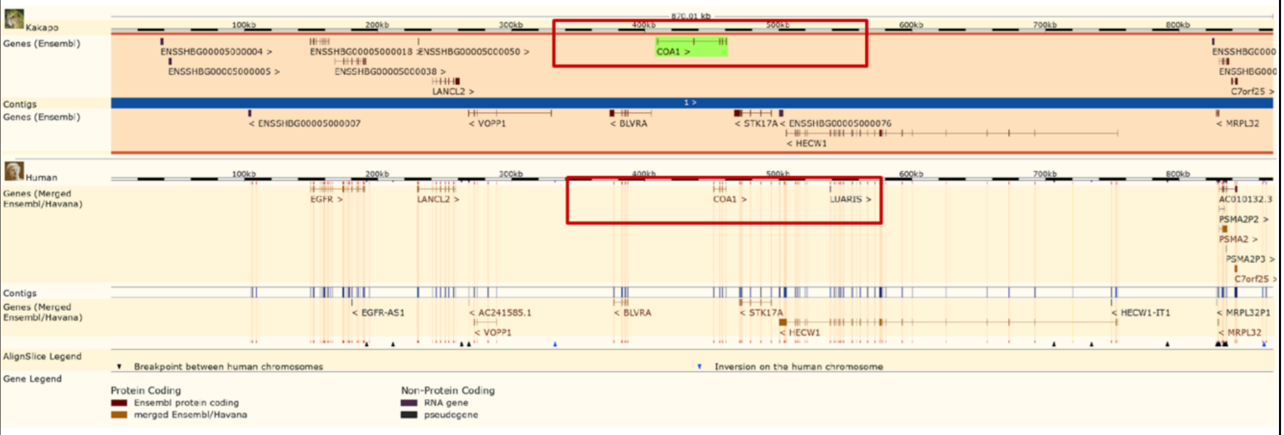

Figure S436: Gene order near *COA1* gene in the kakapo genome. The rectangular boxes connected by thin lines depict genes, with the boxes denoting exons and connecting lines being introns. Direction of transcription is denoted by the > or < symbols (signify positive and negative strand of transcription respectively) beside the gene names. chromosome bands and the genomic coordinates in kilobase (kb) are shown in the topmost row. Details of the contig and scaffold identity are provided in the blue bar located in the middle of the figure. Exact location of the *COA1* gene is depicted by a red box.

Figure S437

human - Zebra finch

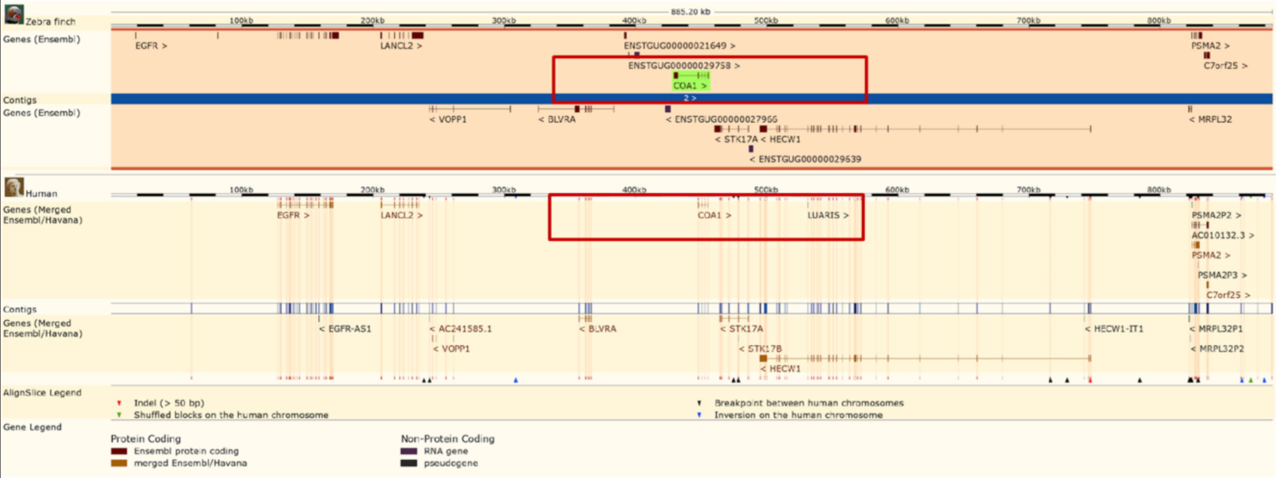

Figure S437: Gene order near *COA1* gene in the zebra finch genome. The rectangular boxes connected by thin lines depict genes, with the boxes denoting exons and connecting lines being introns. Direction of transcription is denoted by the > or < symbols (signify positive and negative strand of transcription respectively) beside the gene names. chromosome bands and the genomic coordinates in kilobase (kb) are shown in the topmost row. Details of the contig and scaffold identity are provided in the blue bar located in the middle of the figure. Exact location of the *COA1* gene is depicted by a red box.

Figure S438

human – Angola colobus

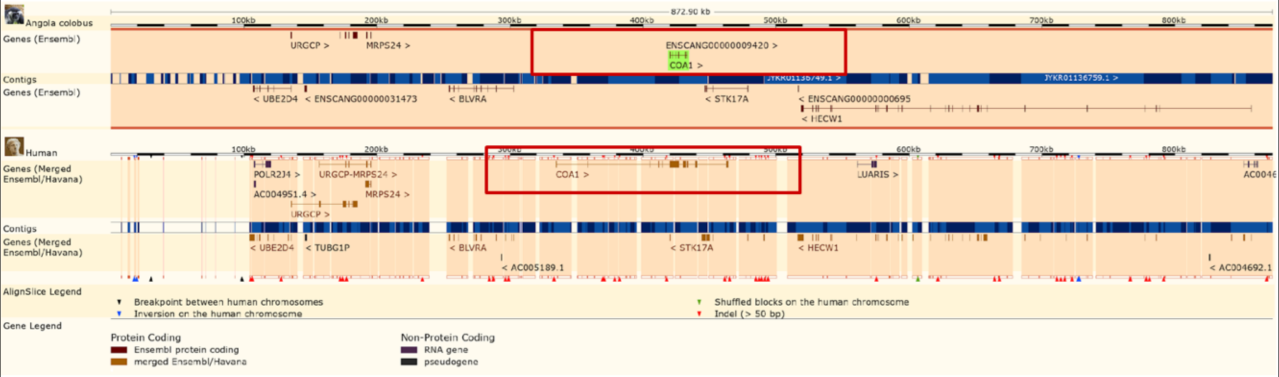

Figure S438: Gene order near *COA1* gene in the Angola colobus genome. The rectangular boxes connected by thin lines depict genes, with the boxes denoting exons and connecting lines being introns. Direction of transcription is denoted by the > or < symbols (signify positive and negative strand of transcription respectively) beside the gene names. chromosome bands and the genomic coordinates in kilobase (kb) are shown in the topmost row. Details of the contig and scaffold identity are provided in the blue bar located in the middle of the figure. Exact location of the *COA1* gene is depicted by a red box.

Figure S439

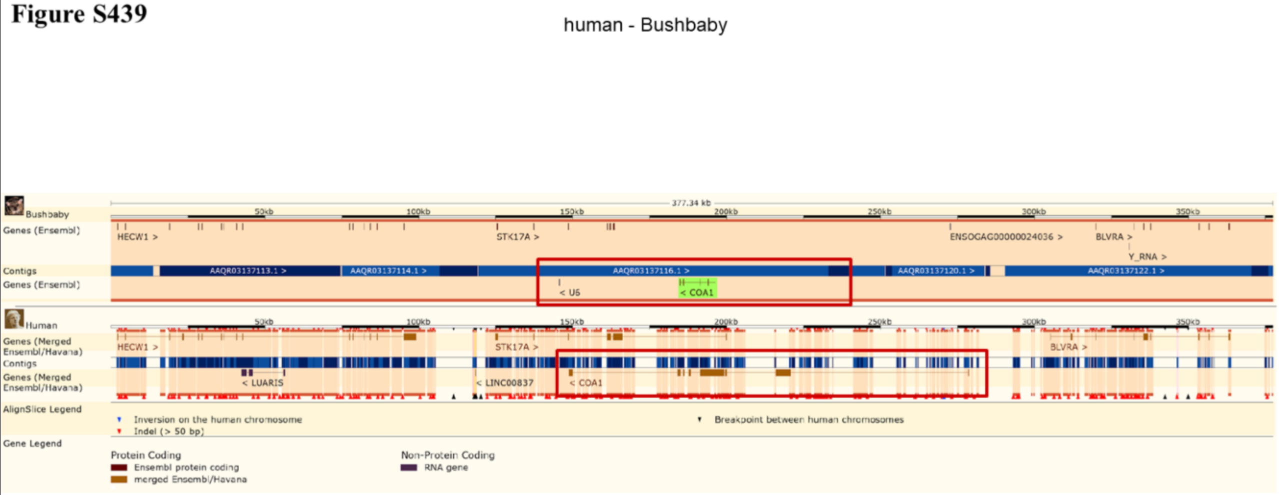

Figure S439: Gene order near *COA1* gene in the bushbaby genome. The rectangular boxes connected by thin lines depict genes, with the boxes denoting exons and connecting lines being introns. Direction of transcription is denoted by the > or < symbols (signify positive and negative strand of transcription respectively) beside the gene names. chromosome bands and the genomic coordinates in kilobase (kb) are shown in the topmost row. Details of the contig and scaffold identity are provided in the blue bar located in the middle of the figure. Exact location of the *COA1* gene is depicted by a red box.

Figure S440

human - Gibbon

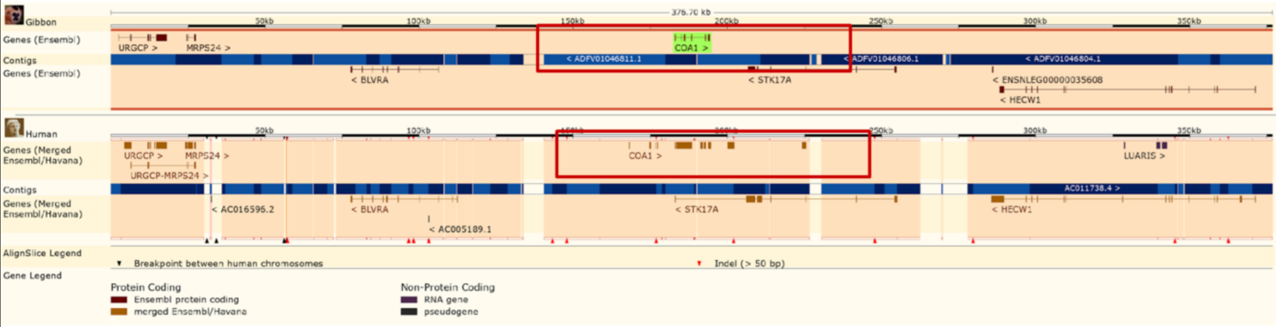

Figure S440: Gene order near *COA1* gene in the gibbon genome. The rectangular boxes connected by thin lines depict genes, with the boxes denoting exons and connecting lines being introns. Direction of transcription is denoted by the > or < symbols (signify positive and negative strand of transcription respectively) beside the gene names. chromosome bands and the genomic coordinates in kilobase (kb) are shown in the topmost row. Details of the contig and scaffold identity are provided in the blue bar located in the middle of the figure. Exact location of the *COA1* gene is depicted by a red box.

Figure S441

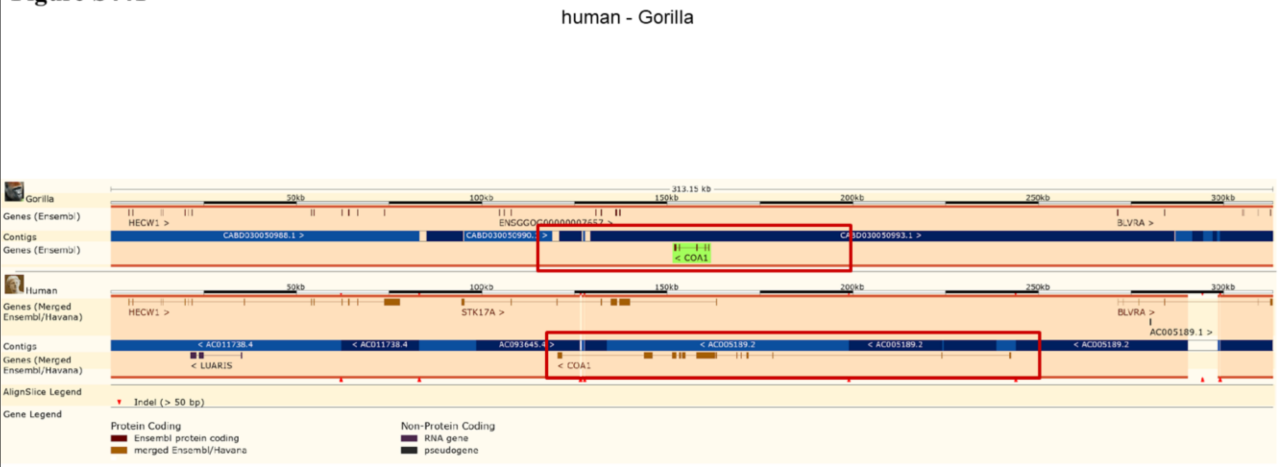

Figure S441: Gene order near *COA1* gene in the gorilla genome. The rectangular boxes connected by thin lines depict genes, with the boxes denoting exons and connecting lines being introns. Direction of transcription is denoted by the > or < symbols (signify positive and negative strand of transcription respectively) beside the gene names. chromosome bands and the genomic coordinates in kilobase (kb) are shown in the topmost row. Details of the contig and scaffold identity are provided in the blue bar located in the middle of the figure. Exact location of the *COA1* gene is depicted by a red box.

Figure S442

human – Ma's night monkey

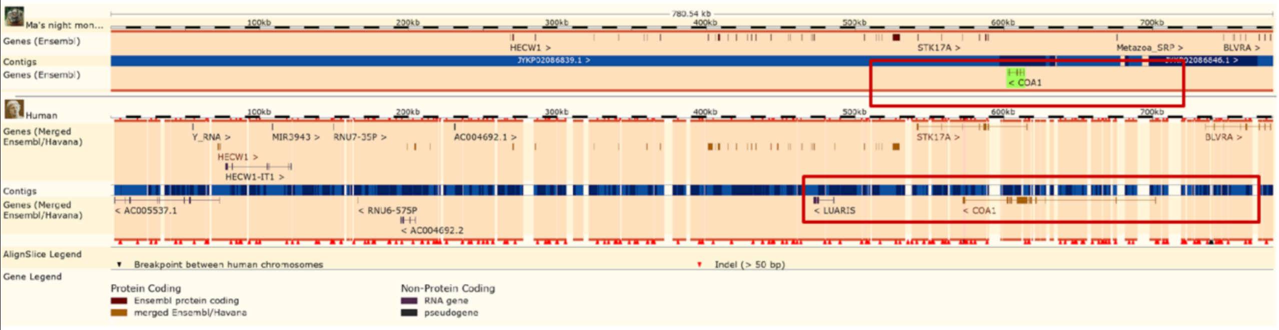

Figure S442: Gene order near *COA1* gene in the Ma's night monkey genome. The rectangular boxes connected by thin lines depict genes, with the boxes denoting exons and connecting lines being introns. Direction of transcription is denoted by the > or < symbols (signify positive and negative strand of transcription respectively) beside the gene names. chromosome bands and the genomic coordinates in kilobase (kb) are shown in the topmost row. Details of the contig and scaffold identity are provided in the blue bar located in the middle of the figure. Exact location of the *COA1* gene is depicted by a red box.

Figure S443

human - Marmoset

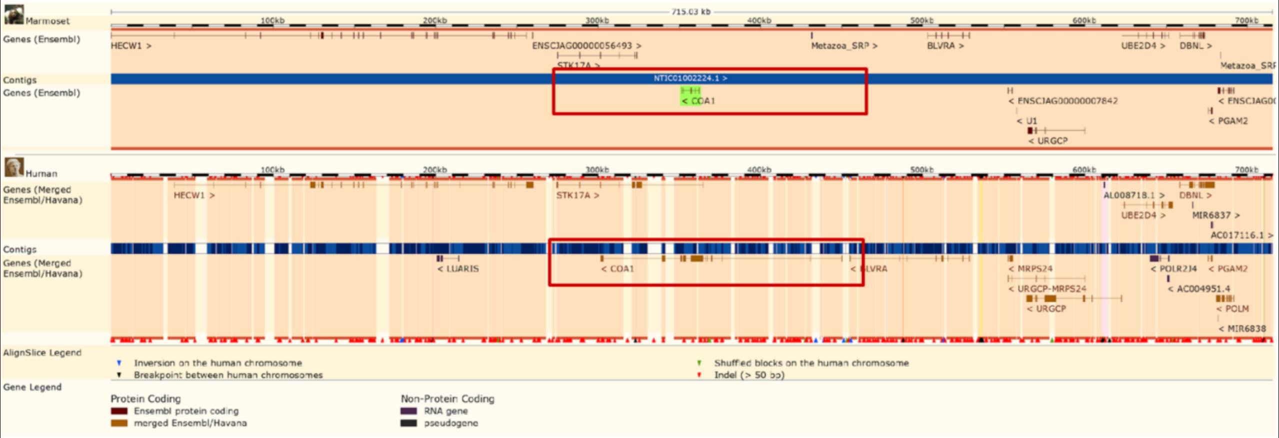

Figure S443: Gene order near *COA1* gene in the marmoset genome. The rectangular boxes connected by thin lines depict genes, with the boxes denoting exons and connecting lines being introns. Direction of transcription is denoted by the > or < symbols (signify positive and negative strand of transcription respectively) beside the gene names. chromosome bands and the genomic coordinates in kilobase (kb) are shown in the topmost row. Details of the contig and scaffold identity are provided in the blue bar located in the middle of the figure. Exact location of the *COA1* gene is depicted by a red box.

Figure S444

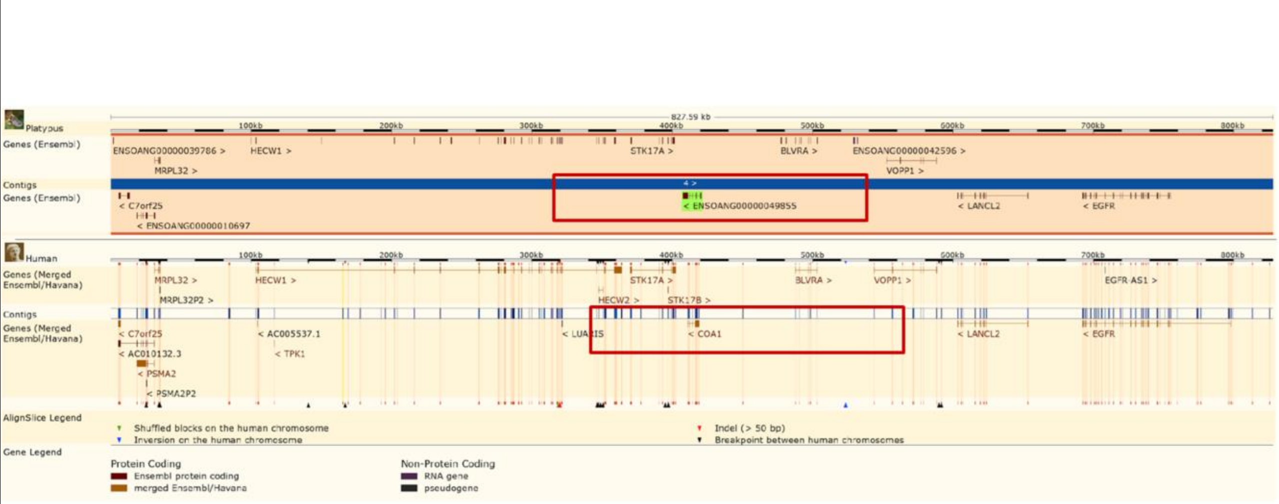

Figure S444: Gene order near *COA1* gene in the platypus genome. The rectangular boxes connected by thin lines depict genes, with the boxes denoting exons and connecting lines being introns. Direction of transcription is denoted by the > or < symbols (signify positive and negative strand of transcription respectively) beside the gene names. chromosome bands and the genomic coordinates in kilobase (kb) are shown in the topmost row. Details of the contig and scaffold identity are provided in the blue bar located in the middle of the figure. Exact location of the *COA1* gene is depicted by a red box.

Figure S445

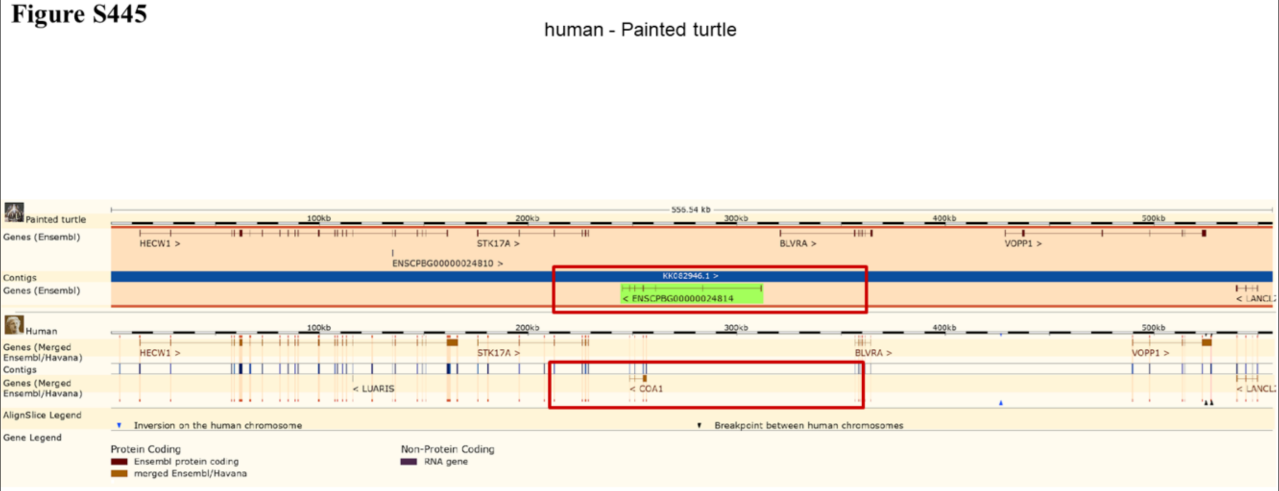

Figure S445: Gene order near *COA1* gene in the painted turtle genome. The rectangular boxes connected by thin lines depict genes, with the boxes denoting exons and connecting lines being introns. Direction of transcription is denoted by the > or < symbols (signify positive and negative strand of transcription respectively) beside the gene names. chromosome bands and the genomic coordinates in kilobase (kb) are shown in the topmost row. Details of the contig and scaffold identity are provided in the blue bar located in the middle of the figure. Exact location of the *COA1* gene is depicted by a red box.

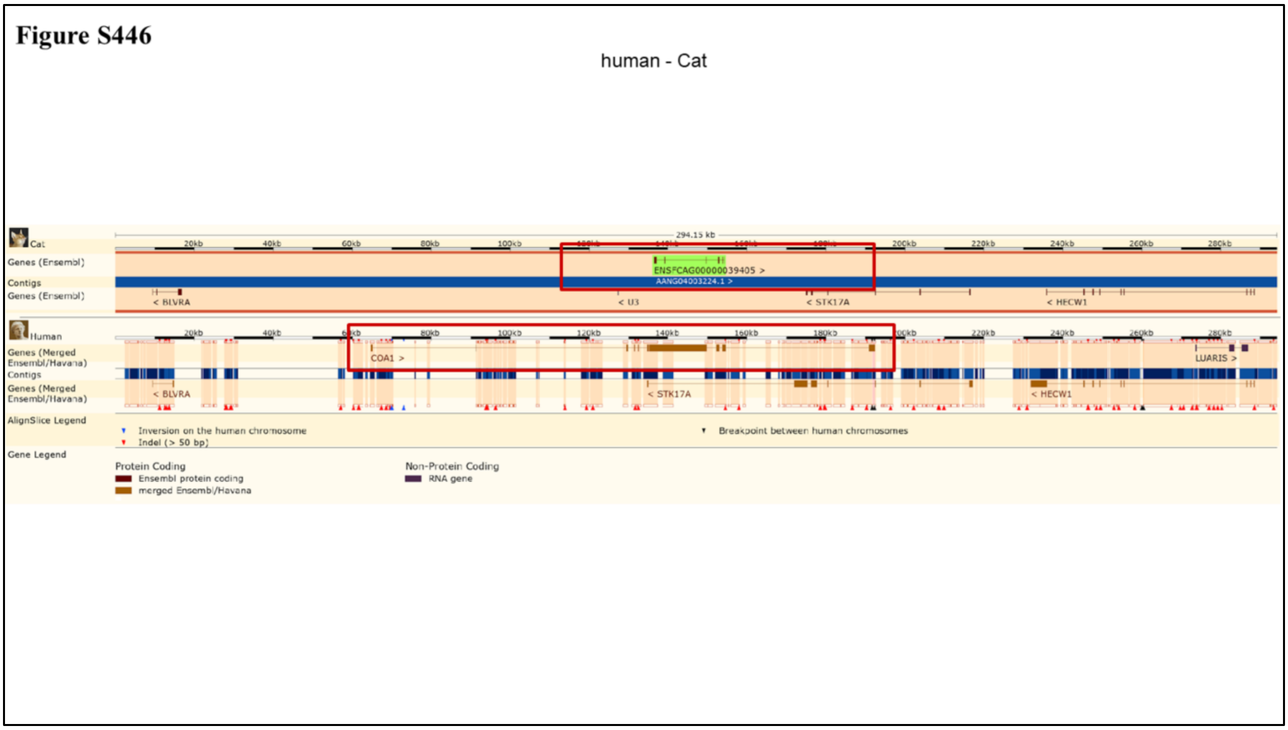

Figure S446: Gene order near *COA1* gene in the cat genome. The rectangular boxes connected by thin lines depict genes, with the boxes denoting exons and connecting lines being introns. Direction of transcription is denoted by the > or < symbols (signify positive and negative strand of transcription respectively) beside the gene names. chromosome bands and the genomic coordinates in kilobase (kb) are shown in the topmost row. Details of the contig and scaffold identity are provided in the blue bar located in the middle of the figure. Exact location of the *COA1* gene is depicted by a red box.

Figure S447

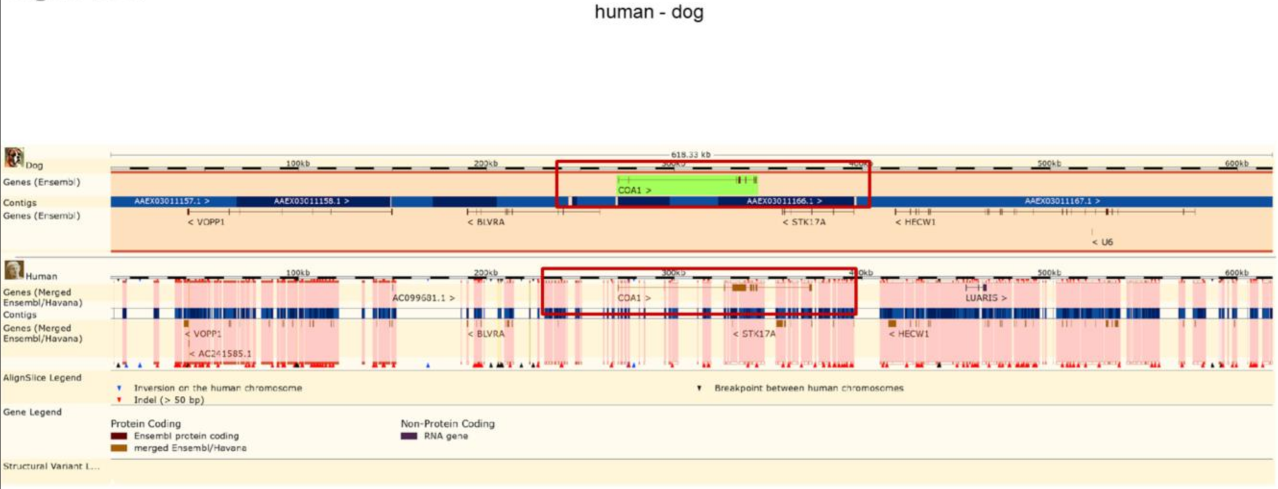

Figure S447: Gene order near *COA1* gene in the dog genome. The rectangular boxes connected by thin lines depict genes, with the boxes denoting exons and connecting lines being introns. Direction of transcription is denoted by the > or < symbols (signify positive and negative strand of transcription respectively) beside the gene names. chromosome bands and the genomic coordinates in kilobase (kb) are shown in the topmost row. Details of the contig and scaffold identity are provided in the blue bar located in the middle of the figure. Exact location of the *COA1* gene is depicted by a red box.

Figure S448

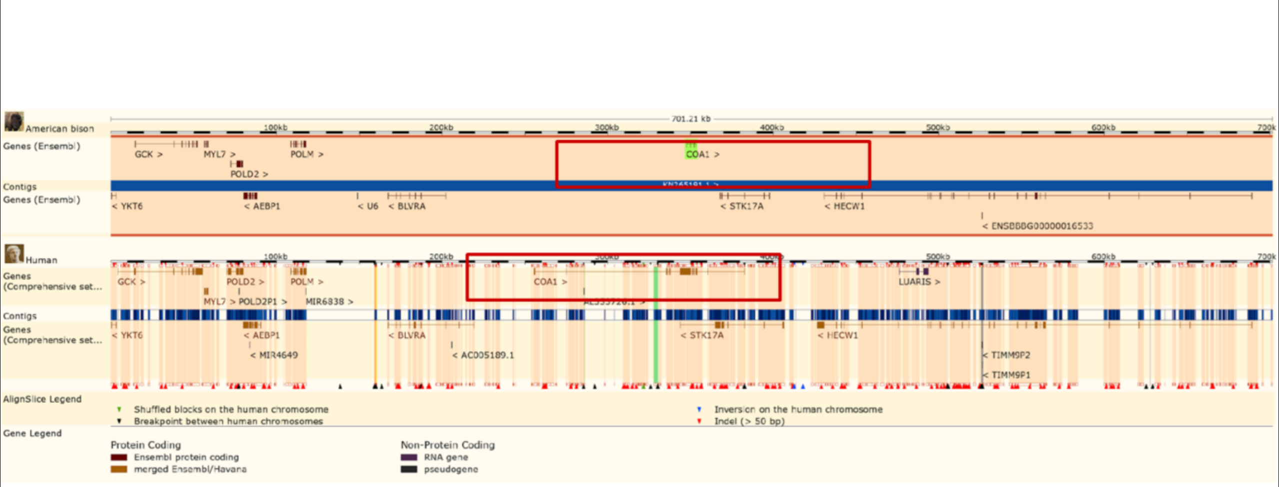

Figure S448: Gene order near *COA1* gene in the American bison genome. The rectangular boxes connected by thin lines depict genes, with the boxes denoting exons and connecting lines being introns. Direction of transcription is denoted by the > or < symbols (signify positive and negative strand of transcription respectively) beside the gene names. chromosome bands and the genomic coordinates in kilobase (kb) are shown in the topmost row. Details of the contig and scaffold identity are provided in the blue bar located in the middle of the figure. Exact location of the *COA1* gene is depicted by a red box.

Figure S449

human - Armadillo

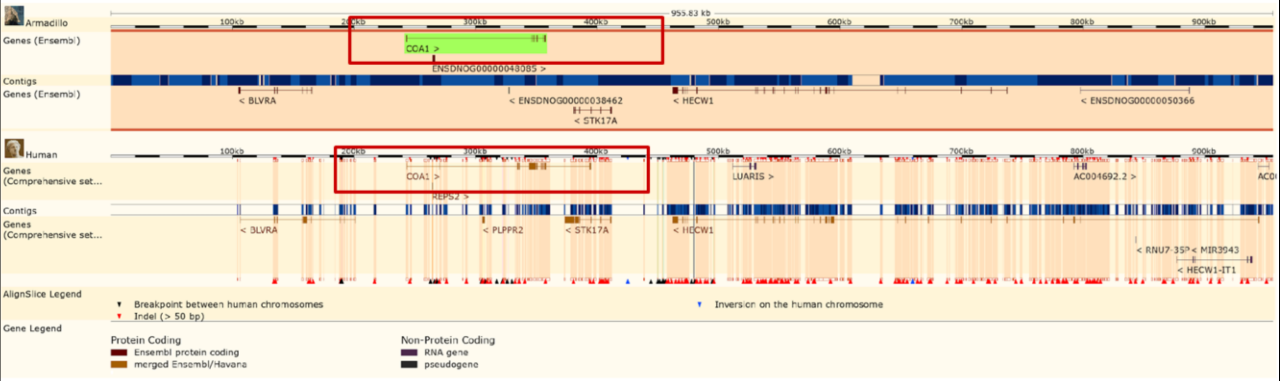

Figure S449: Gene order near *COA1* gene in the armadillo genome. The rectangular boxes connected by thin lines depict genes, with the boxes denoting exons and connecting lines being introns. Direction of transcription is denoted by the > or < symbols (signify positive and negative strand of transcription respectively) beside the gene names. chromosome bands and the genomic coordinates in kilobase (kb) are shown in the topmost row. Details of the contig and scaffold identity are provided in the blue bar located in the middle of the figure. Exact location of the *COA1* gene is depicted by a red box.

Figure S450

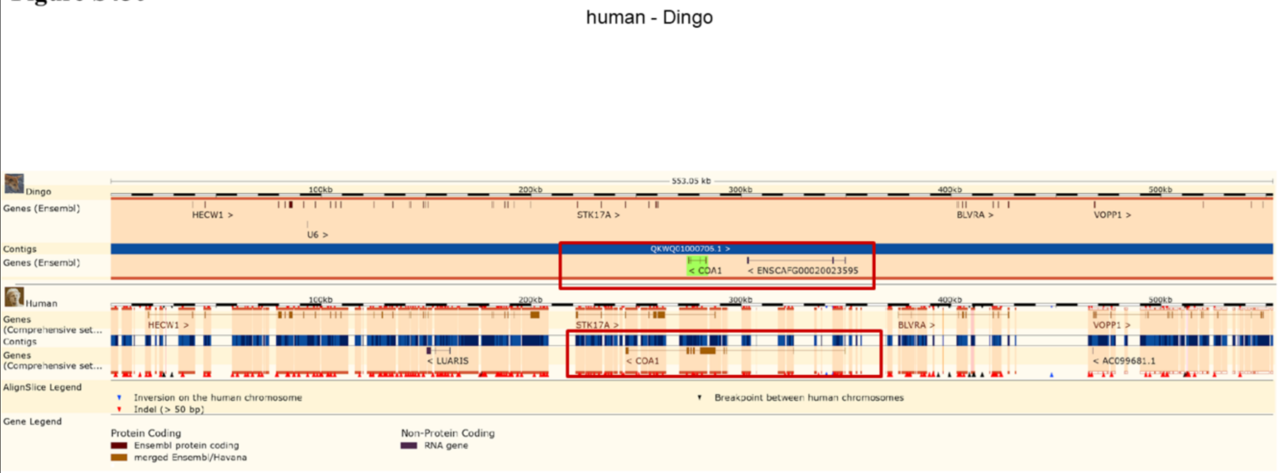

Figure S450: Gene order near *COA1* gene in the dingo genome. The rectangular boxes connected by thin lines depict genes, with the boxes denoting exons and connecting lines being introns. Direction of transcription is denoted by the > or < symbols (signify positive and negative strand of transcription respectively) beside the gene names. chromosome bands and the genomic coordinates in kilobase (kb) are shown in the topmost row. Details of the contig and scaffold identity are provided in the blue bar located in the middle of the figure. Exact location of the *COA1* gene is depicted by a red box.

Figure S451

human - Cow

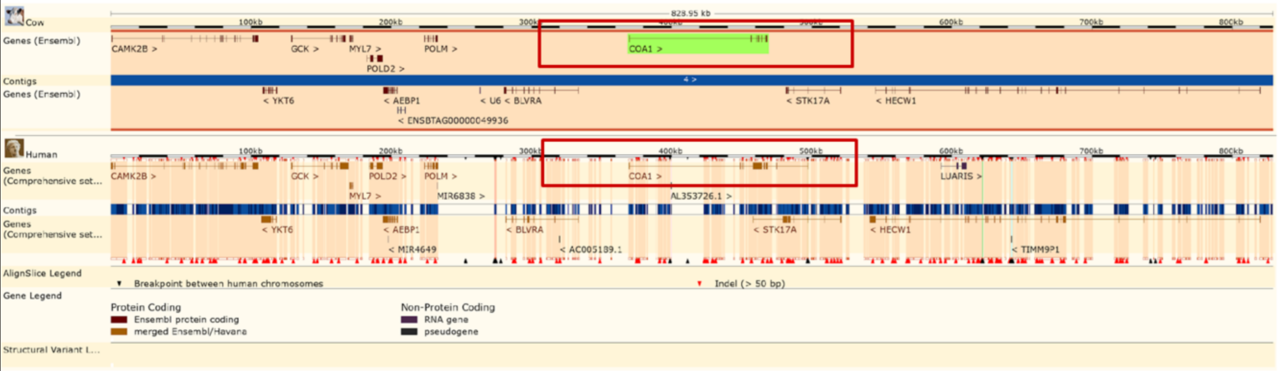

Figure S451: Gene order near *COA1* gene in the cow genome. The rectangular boxes connected by thin lines depict genes, with the boxes denoting exons and connecting lines being introns. Direction of transcription is denoted by the > or < symbols (signify positive and negative strand of transcription respectively) beside the gene names. chromosome bands and the genomic coordinates in kilobase (kb) are shown in the topmost row. Details of the contig and scaffold identity are provided in the blue bar located in the middle of the figure. Exact location of the *COA1* gene is depicted by a red box.

Figure S452

human - Blue whale

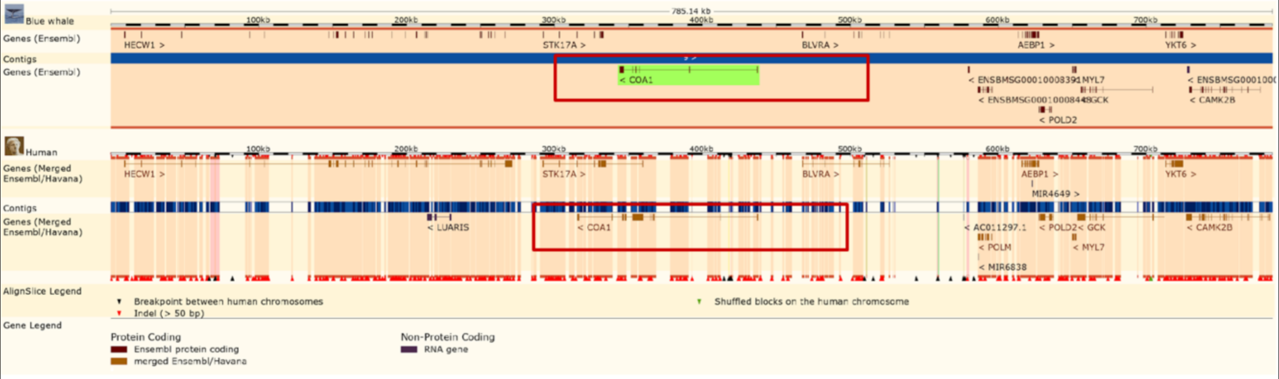

Figure S452: Gene order near *COA1* gene in the blue whale genome. The rectangular boxes connected by thin lines depict genes, with the boxes denoting exons and connecting lines being introns. Direction of transcription is denoted by the > or < symbols (signify positive and negative strand of transcription respectively) beside the gene names. chromosome bands and the genomic coordinates in kilobase (kb) are shown in the topmost row. Details of the contig and scaffold identity are provided in the blue bar located in the middle of the figure. Exact location of the *COA1* gene is depicted by a red box.

Figure S453

human - Bonobo

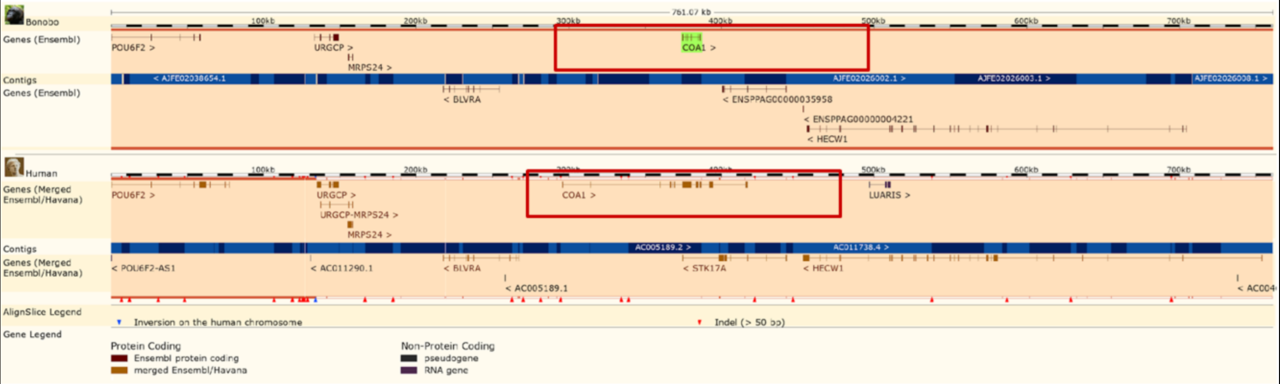

Figure S453: Gene order near *COA1* gene in the bonobo genome. The rectangular boxes connected by thin lines depict genes, with the boxes denoting exons and connecting lines being introns. Direction of transcription is denoted by the > or < symbols (signify positive and negative strand of transcription respectively) beside the gene names. chromosome bands and the genomic coordinates in kilobase (kb) are shown in the topmost row. Details of the contig and scaffold identity are provided in the blue bar located in the middle of the figure. Exact location of the *COA1* gene is depicted by a red box.

Figure S454

human - Chimpanzee

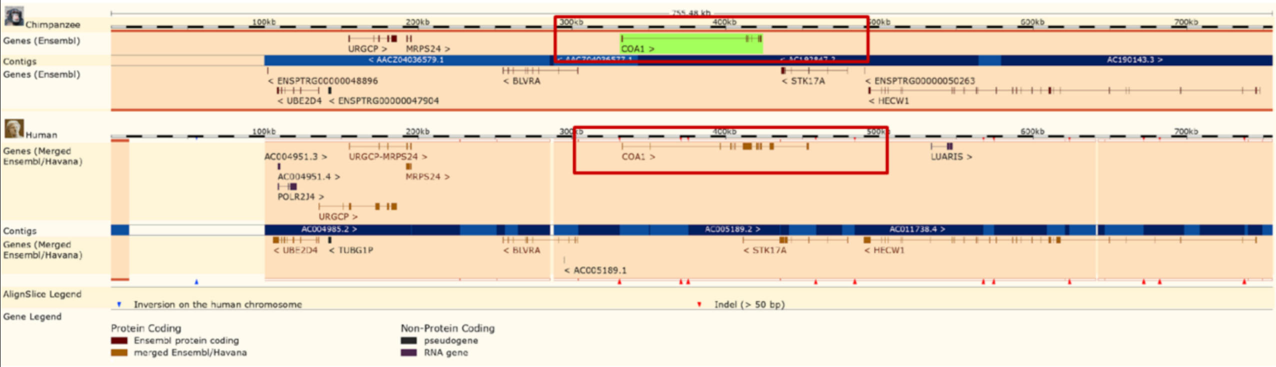

Figure S454: Gene order near *COA1* gene in the chimpanzee genome. The rectangular boxes connected by thin lines depict genes, with the boxes denoting exons and connecting lines being introns. Direction of transcription is denoted by the > or < symbols (signify positive and negative strand of transcription respectively) beside the gene names. chromosome bands and the genomic coordinates in kilobase (kb) are shown in the topmost row. Details of the contig and scaffold identity are provided in the blue bar located in the middle of the figure. Exact location of the *COA1* gene is depicted by a red box.

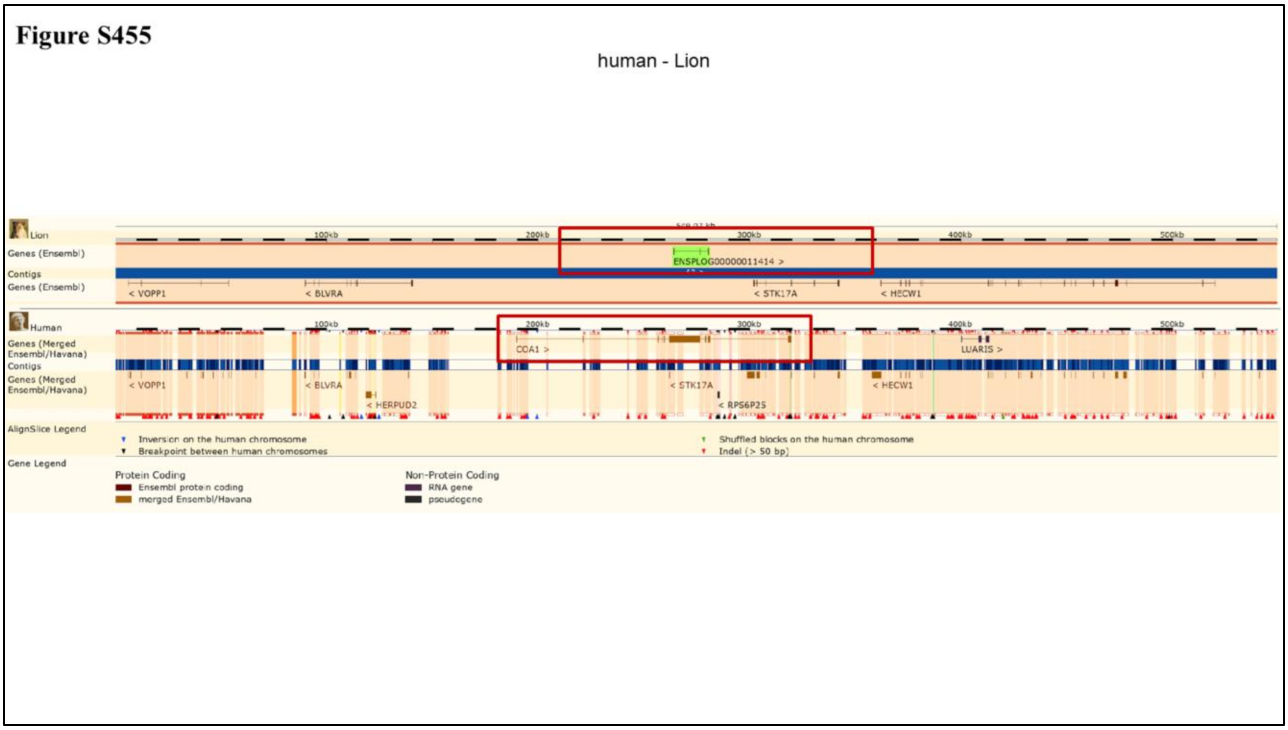

Figure S455: Gene order near *COA1* gene in the lion genome. The rectangular boxes connected by thin lines depict genes, with the boxes denoting exons and connecting lines being introns. Direction of transcription is denoted by the > or < symbols (signify positive and negative strand of transcription respectively) beside the gene names. chromosome bands and the genomic coordinates in kilobase (kb) are shown in the topmost row. Details of the contig and scaffold identity are provided in the blue bar located in the middle of the figure. Exact location of the *COA1* gene is depicted by a red box.

Figure S456

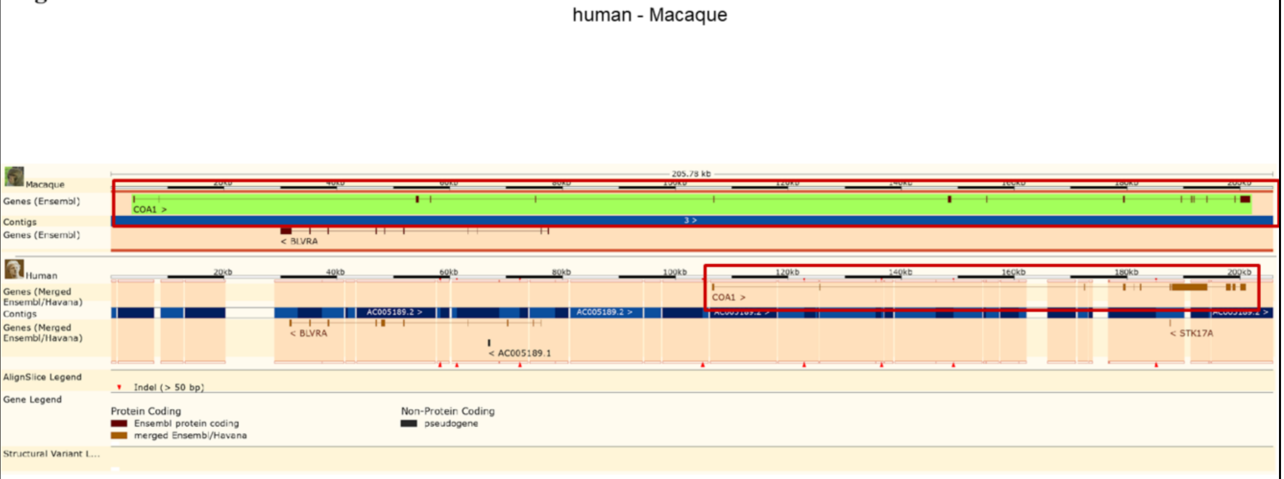

Figure S456: Gene order near *COA1* gene in the macaque genome. The rectangular boxes connected by thin lines depict genes, with the boxes denoting exons and connecting lines being introns. Direction of transcription is denoted by the > or < symbols (signify positive and negative strand of transcription respectively) beside the gene names. chromosome bands and the genomic coordinates in kilobase (kb) are shown in the topmost row. Details of the contig and scaffold identity are provided in the blue bar located in the middle of the figure. Exact location of the *COA1* gene is depicted by a red box.

Figure S457

human - Orangutan

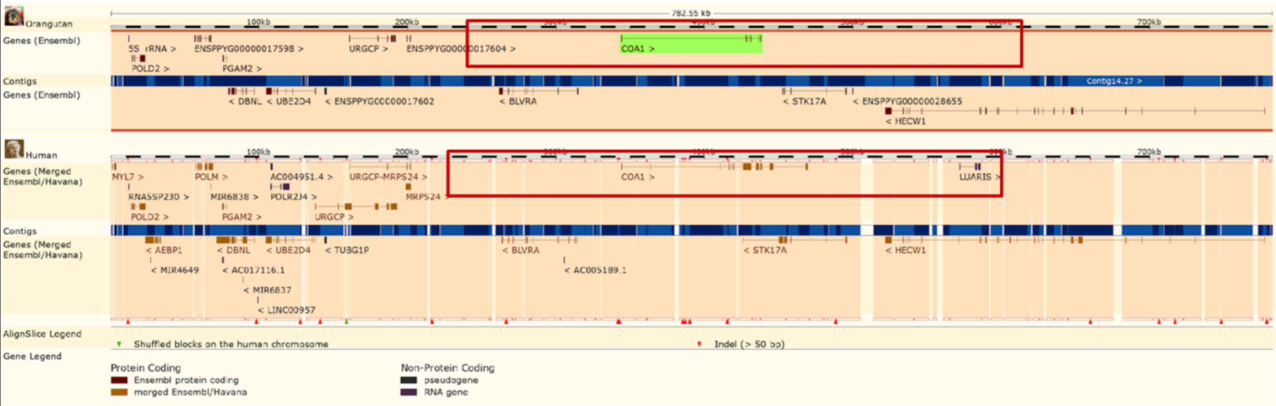

Figure S457: Gene order near *COA1* gene in the orangutan genome. The rectangular boxes connected by thin lines depict genes, with the boxes denoting exons and connecting lines being introns. Direction of transcription is denoted by the > or < symbols (signify positive and negative strand of transcription respectively) beside the gene names. chromosome bands and the genomic coordinates in kilobase (kb) are shown in the topmost row. Details of the contig and scaffold identity are provided in the blue bar located in the middle of the figure. Exact location of the *COA1* gene is depicted by a red box.

Figure S458

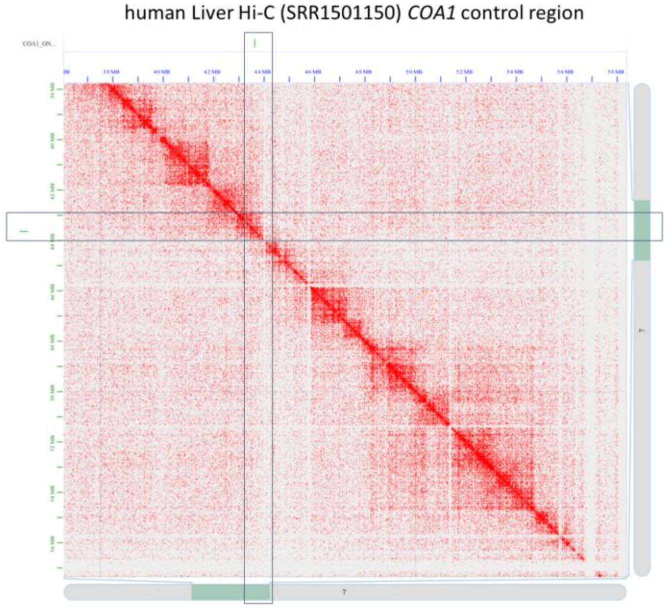

Figure S458: Hi-C figure generated using Juicebox of human liver data (SRR1501150) where red dots show the genomic loci interaction between adjacent regions and gap shows there is no interaction between the nearby region. We loaded the bed file on the top and left sides of the Hi-C plot, where the green color indicates the region of interest (*COA1*) removed the reads using bedtools subtract for control. chromosome or scaffold is mentioned on the bottom and right sides of the plot.

Figure S459

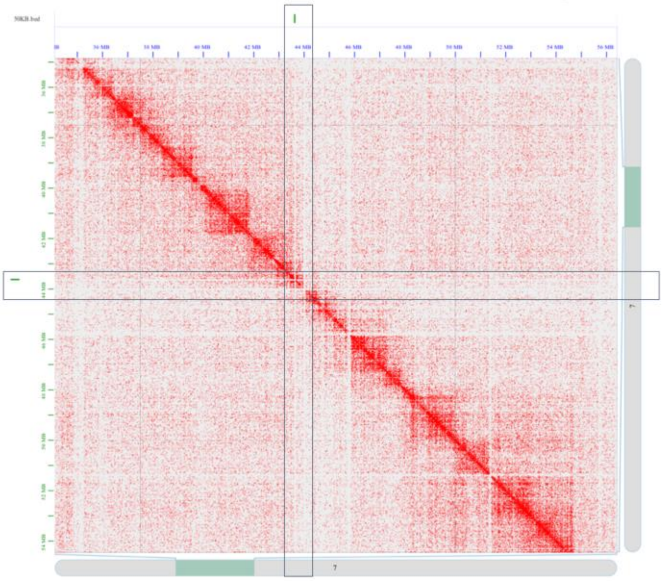

Figure S459: Hi-C figure generated using Juicebox of human liver data (SRR1501150) where red dots show the genomic loci interaction between adjacent regions and gap shows there is no interaction between the nearby region. We loaded the bed file on the top and left sides of the Hi-C plot, where the green color indicates the region of interest (with *COA1*) removed 50 kb region reads using bedtools subtract for control. chromosome or scaffold is mentioned on the bottom and right sides of the plot.

Figure S460

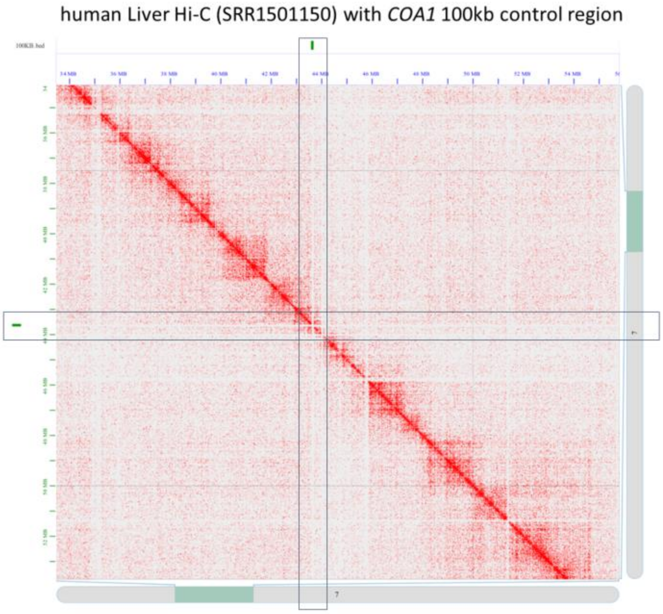

Figure S460: Hi-C figure generated using Juicebox of human liver data (SRR1501150) where red dots show the genomic loci interaction between adjacent regions and gap shows no interaction between the nearby region. We loaded the bed file on the top and left sides of the Hi-C plot, where the green color indicates the region of interest (with *COA1*) removed 100 kb region reads using bedtools subtract for control. chromosome or scaffold is mentioned on the bottom and right sides of the plot.

Figure S461

human Liver Hi-C (SRR1501150) with *COA1* 500kb control region

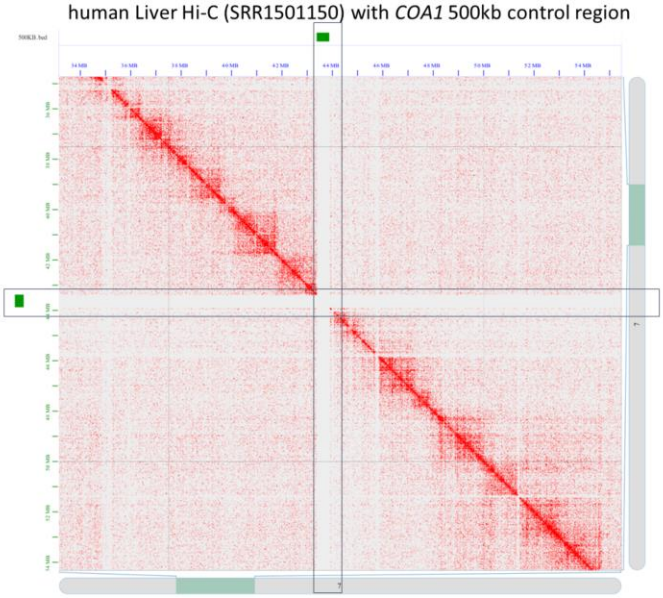

Figure S461: Hi-C figure generated using Juicebox of human liver data (SRR1501150) where red dots show the genomic loci interaction between adjacent regions and gap shows no interaction between the nearby region. We loaded the bed file on the top and left sides of the Hi-C plot, where the green color indicates the region of interest (with *COA1*) removed 500 kb region reads using bedtools subtract for control. chromosome or scaffold is mentioned on the bottom and right sides of the plot.

Figure S462

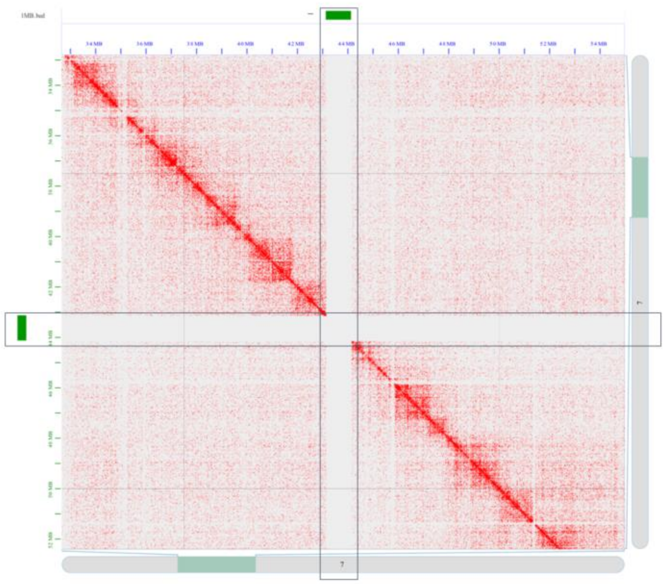

Figure S462: Hi-C figure generated using Juicebox of human liver data (SRR1501150) where red dots show the genomic loci interaction between adjacent regions and gap shows no interaction between the nearby region. We loaded the bed file on the top and left sides of the Hi-C plot, where the green color indicates the region of interest (with *COA1*) removed 100 mb region reads using bedtools subtract for control. chromosome or scaffold is mentioned on the bottom and right sides of the plot.

Figure S463 *Gallus gallus* Gallus_gallus.GRCg6a.dna_sm.toplevel.fa

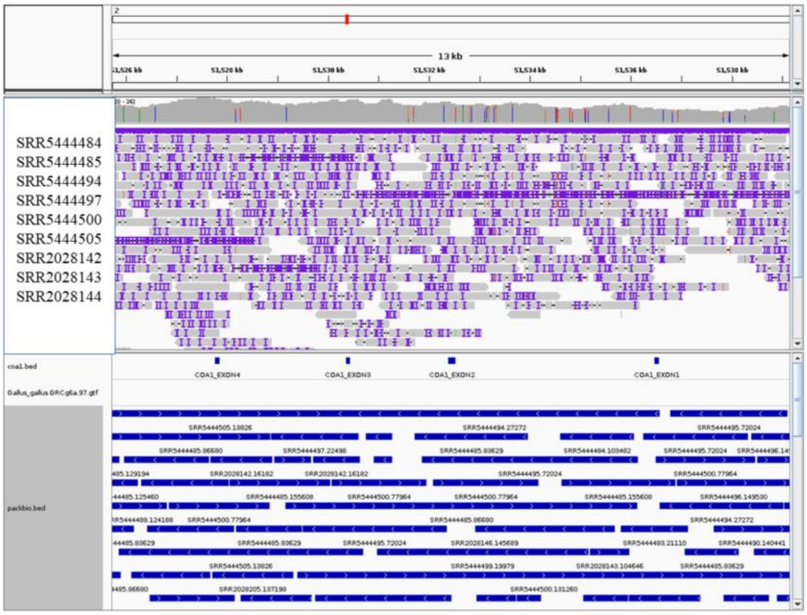

Figure S463: Screenshot of PacBio reads aligned to each of the putative locations of the four *COA1* exons in chicken (*Gallus gallus*). Gray boxes represent reads aligned to the genome. Raw read datasets which is mentioned above have been mapped to the Gallus_gallus.GRCg6a.dna_sm.toplevel.fa genome. Locations of each of the exons are depicted as bed record in the bottom row.

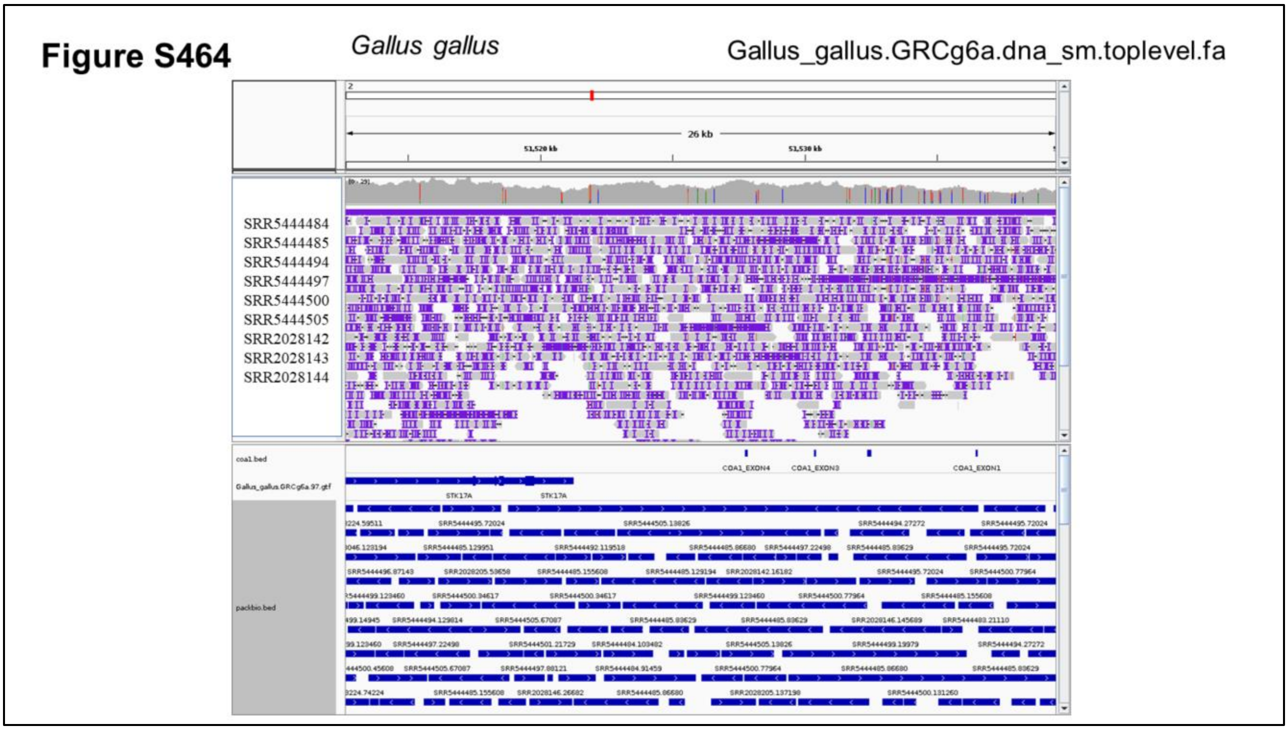

Figure S464: Screenshot of PacBio reads aligned to each of the putative locations of the four *COA1* exons and its nearby gene *STK17A* in chicken (*Gallus gallus*). Gray boxes represent reads aligned to the genome. Raw read datasets which is mentioned above have been mapped to the Gallus_gallus.GRCg6a.dna_sm.toplevel.fa genome. Locations of each of the exons are depicted as bed record in the bottom row.

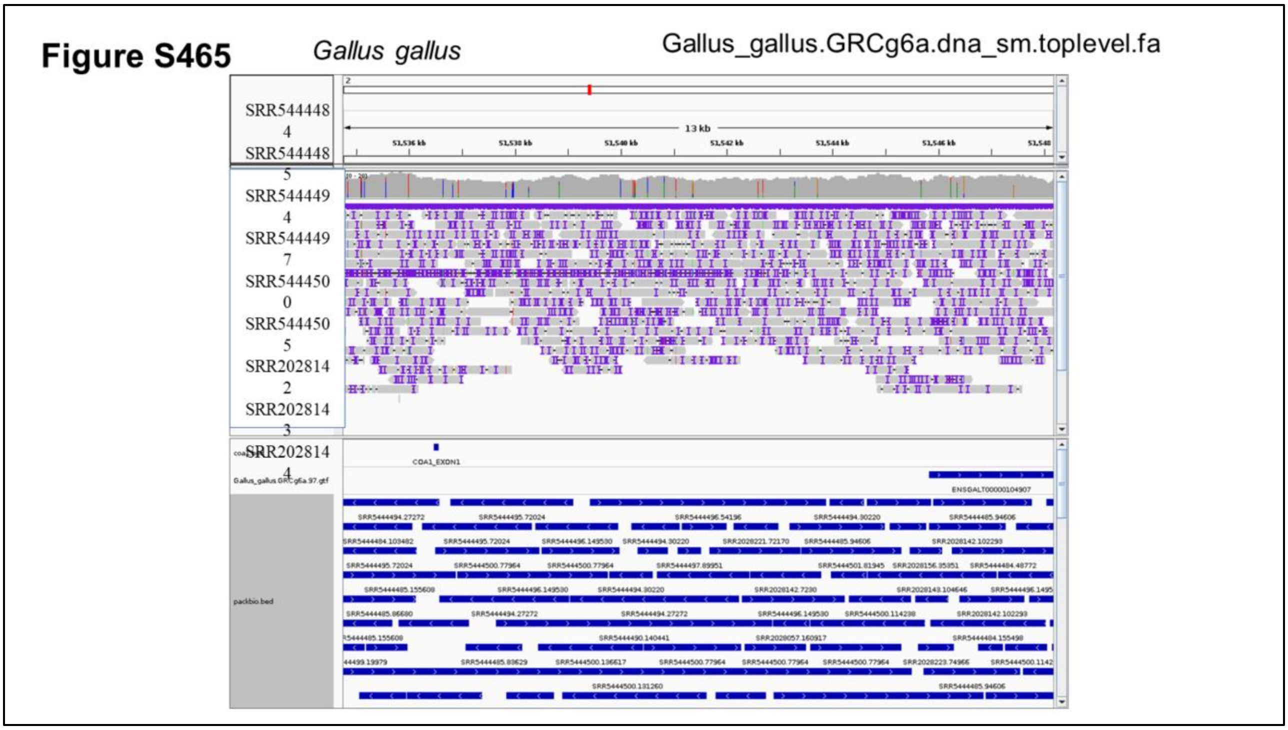

Figure S465: Screenshot of PacBio reads aligned to each of the putative locations of the four *COA1* exons and its nearby gene *BLVRA* in chicken (*Gallus gallus*). Gray boxes represent reads aligned to the genome. Raw read datasets which is mentioned above have been mapped to the Gallus_gallus.GRCg6a.dna_sm.toplevel.fa genome. Locations of each of the exons are depicted as bed record in the bottom row.

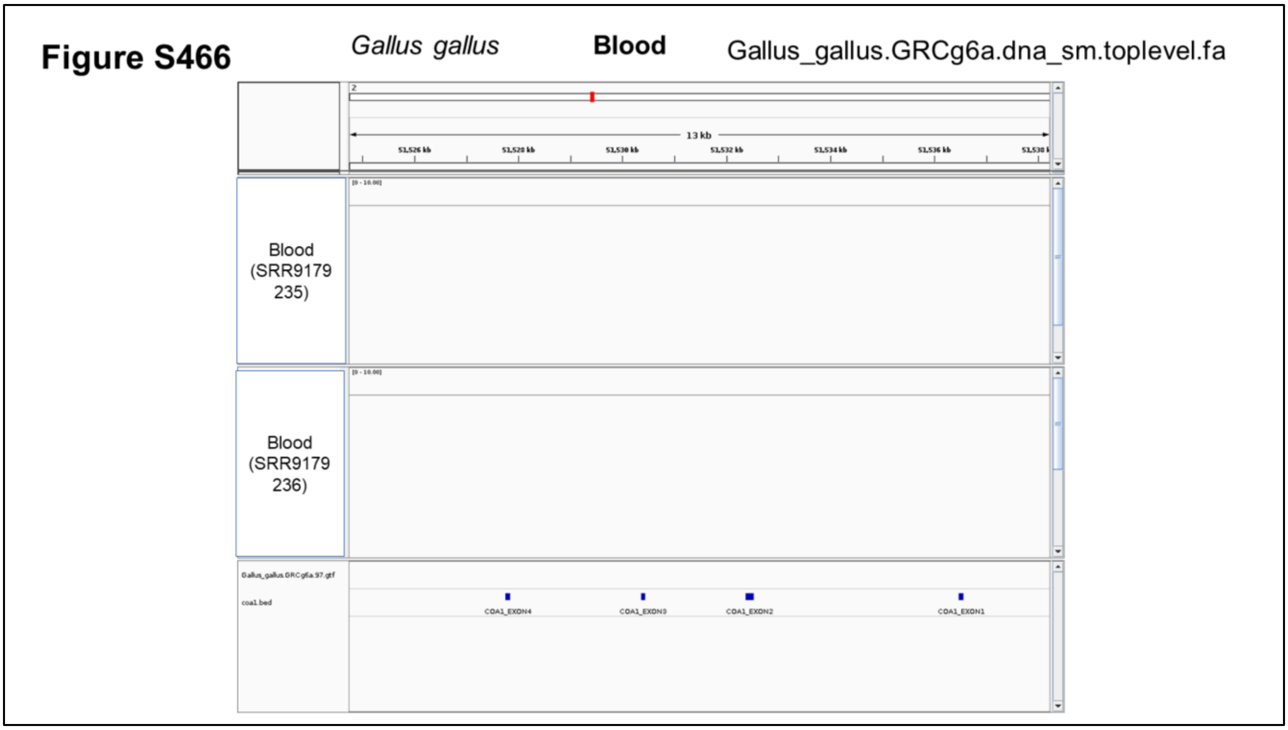

Figure S466: Screenshot of RNA-seq reads aligned to each of the putative locations of the four *COA1* exons in chicken (*Gallus gallus*). Gray boxes represent reads aligned to the genome and thin lines connecting these boxes represent the spliced read alignments. Datasets from blood (SRR9179235) and (SRR9179236) have been mapped to the Gallus_gallus.GRCg6a.dna_sm.toplevel.fa genome. Locations of each of the exons are depicted as bed record in the bottom row.

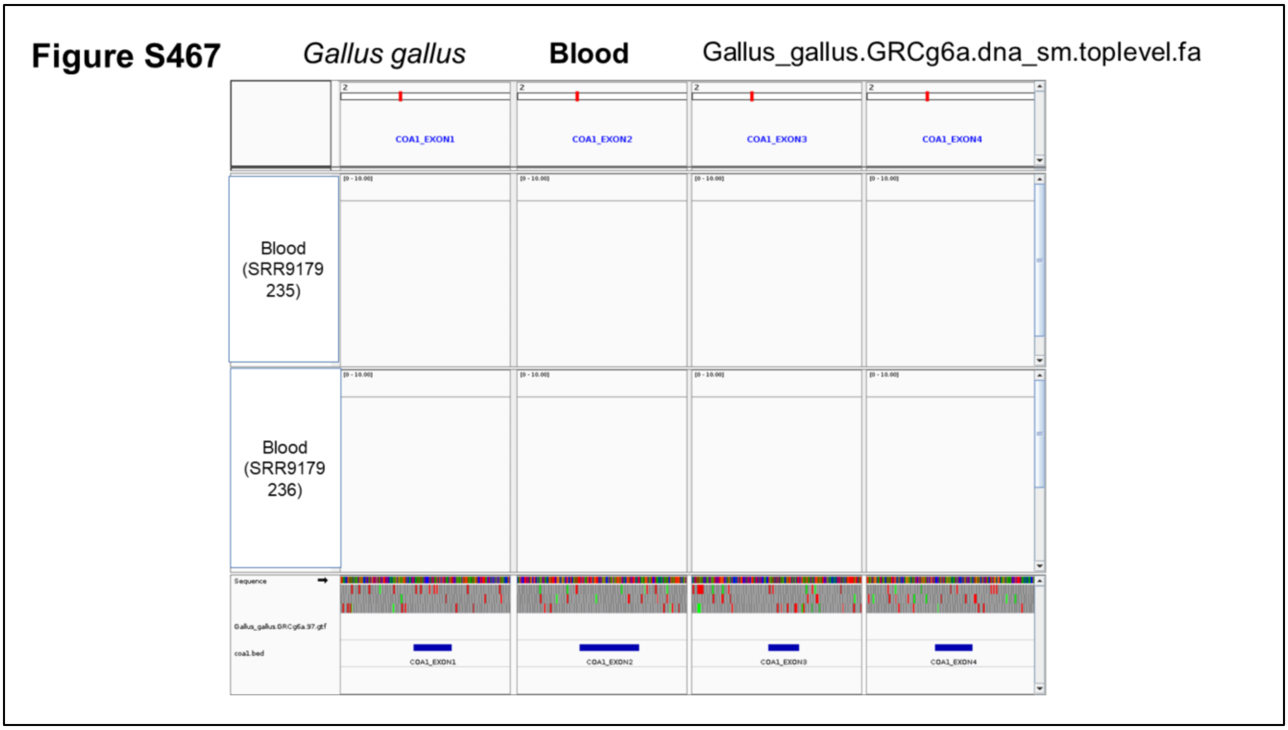

Figure S467: A zoomed in view of each of the *COA1* exons shown in **Supplementary Fig. S466**.

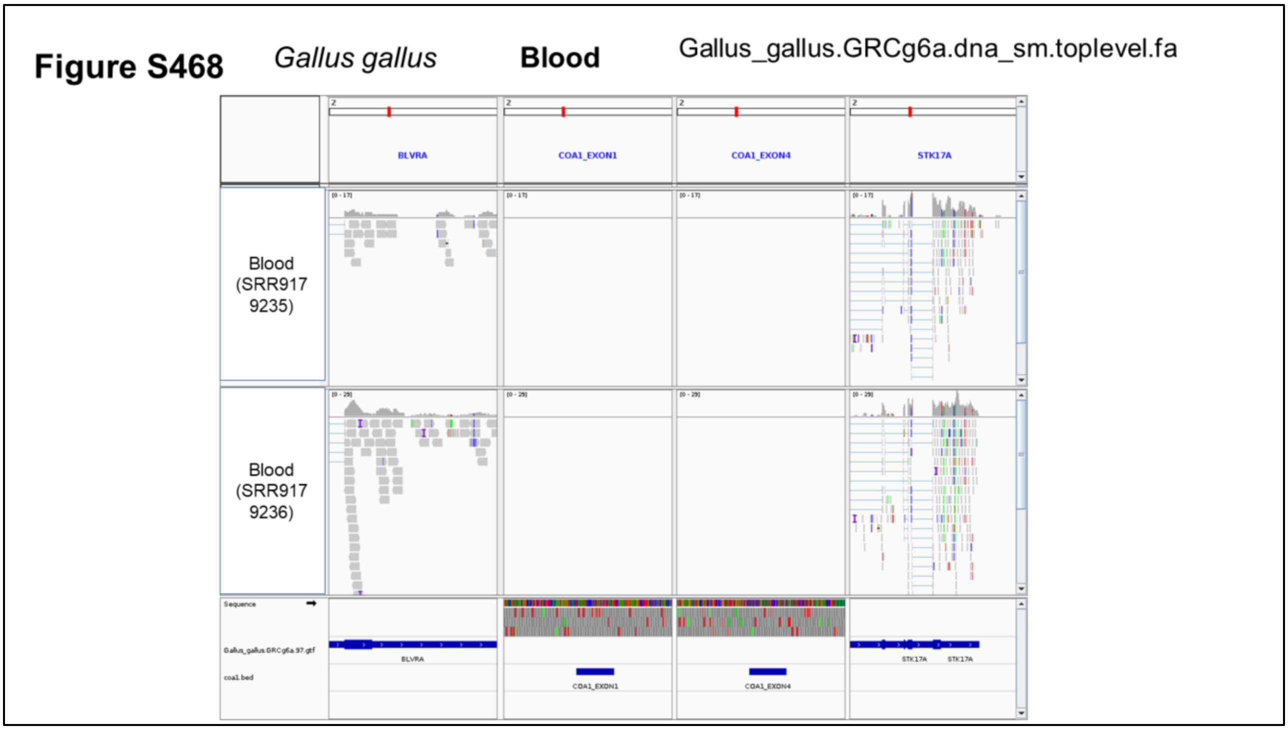

Figure S468: Screenshot of RNA-seq reads aligned to *BLVRA* and *STK17A* to *COA1* exon 1 and 4 respectively in chicken (*Gallus gallus*). Gray boxes represent reads aligned to the genome and thin lines connecting these boxes represent the spliced read alignments. Datasets from blood (SRR9179235) and (SRR9179236) have been mapped to the Gallus_gallus.GRCg6a.dna_sm.toplevel.fa genome. Locations of each of the exons are depicted as bed record in the bottom row.

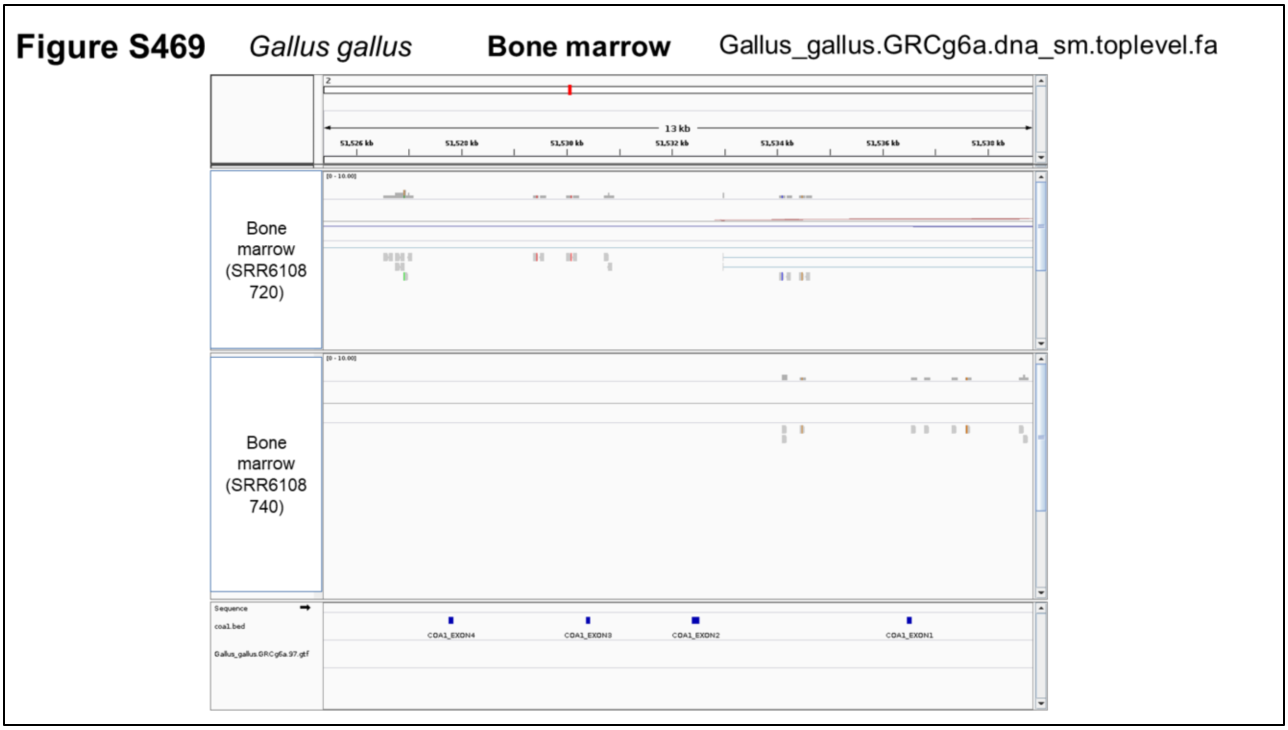

Figure S469: Screenshot of RNA-seq reads aligned to each of the putative locations of the four *COA1* exons in chicken (*Gallus gallus*). Gray boxes represent reads aligned to the genome and thin lines connecting these boxes represent the spliced read alignments. Datasets from bone marrow (SRR6108720 and SRR6108740) have been mapped to the Gallus_gallus.GRCg6a.dna_sm.toplevel.fa genome. Locations of each of the exons are depicted as bed record in the bottom row.

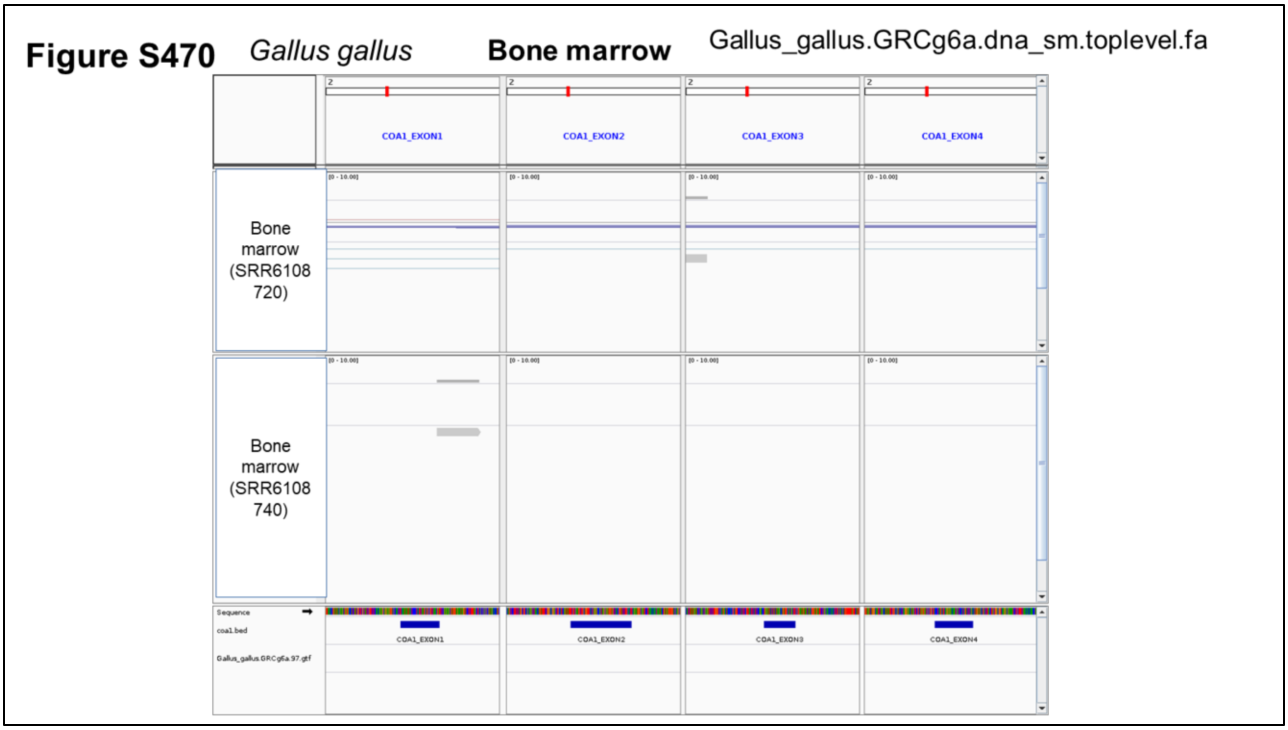

Figure S470: A zoomed in view of each of the *COA1* exons shown in **Supplementary Fig. S469**.

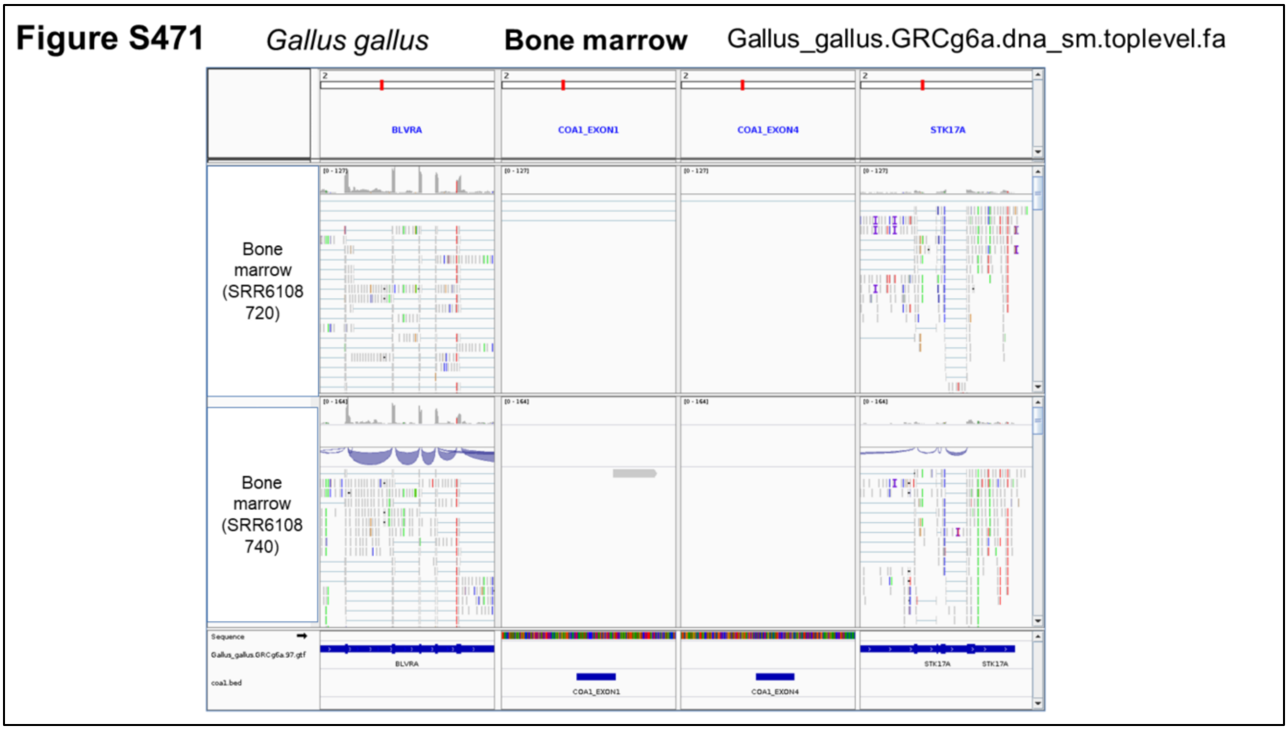

Figure S471: Screenshot of RNA-seq reads aligned to *BLVRA* and *STK17A* to *COA1* exon 1 and 4 respectively in chicken (*Gallus gallus*). Gray boxes represent reads aligned to the genome and thin lines connecting these boxes represent the spliced read alignments. Datasets from bone marrow (SRR6108720 and SRR6108740) have been mapped to the Gallus_gallus.GRCg6a.dna_sm.toplevel.fa genome. Locations of each of the exons are depicted as bed record in the bottom row.

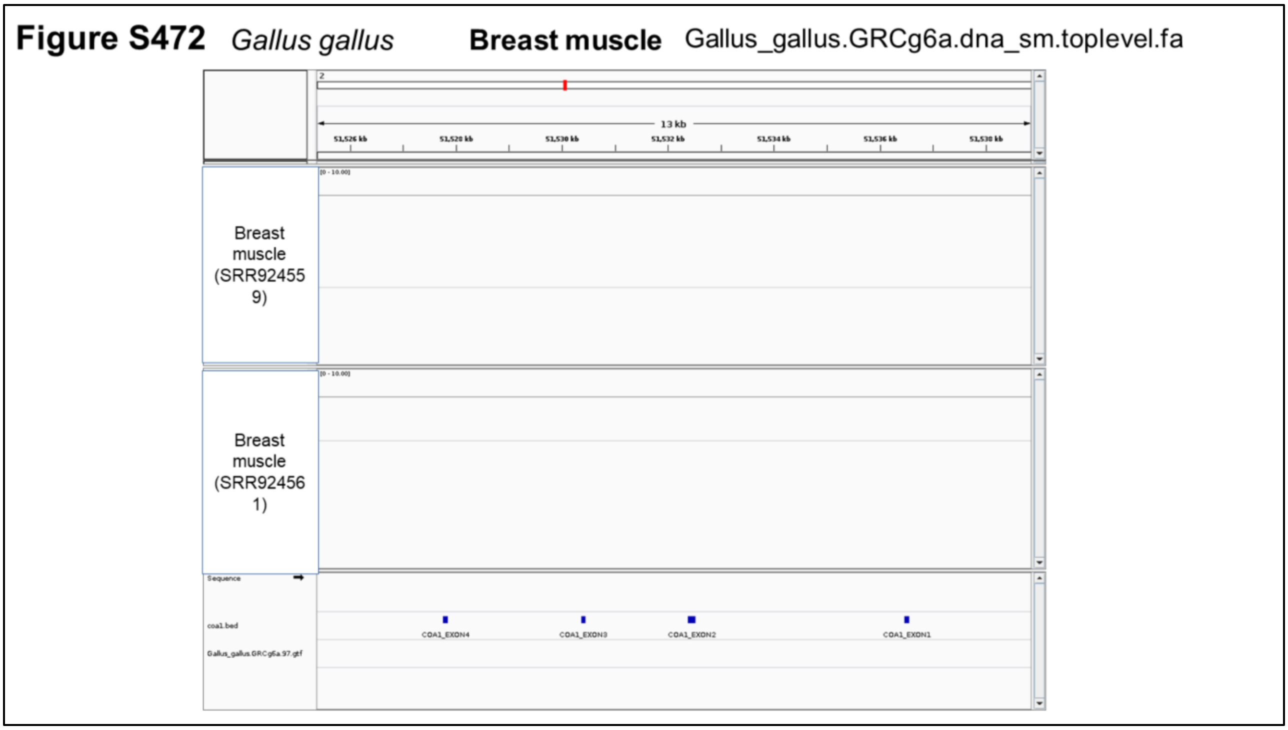

Figure S472: Screenshot of RNA-seq reads aligned to each of the putative locations of the four *COA1* exons in chicken (*Gallus gallus*). Gray boxes represent reads aligned to the genome and thin lines connecting these boxes represent the spliced read alignments. Datasets from breast muscle (SRR924559 and SRR924561) have been mapped to the Gallus_gallus.GRCg6a.dna_sm.toplevel.fa genome. Locations of each of the exons are depicted as bed record in the bottom row.

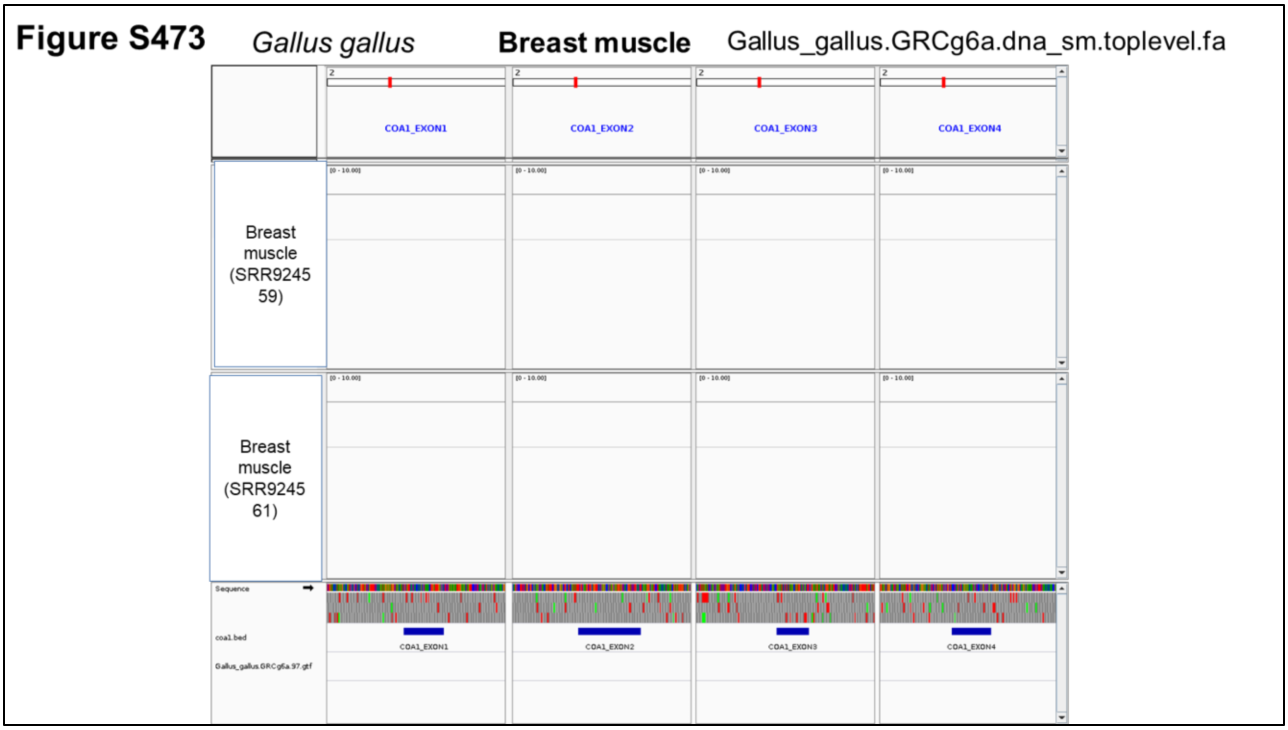

Figure S473: A zoomed in view of each of the *COA1* exons shown **Supplementary Fig. S472**.

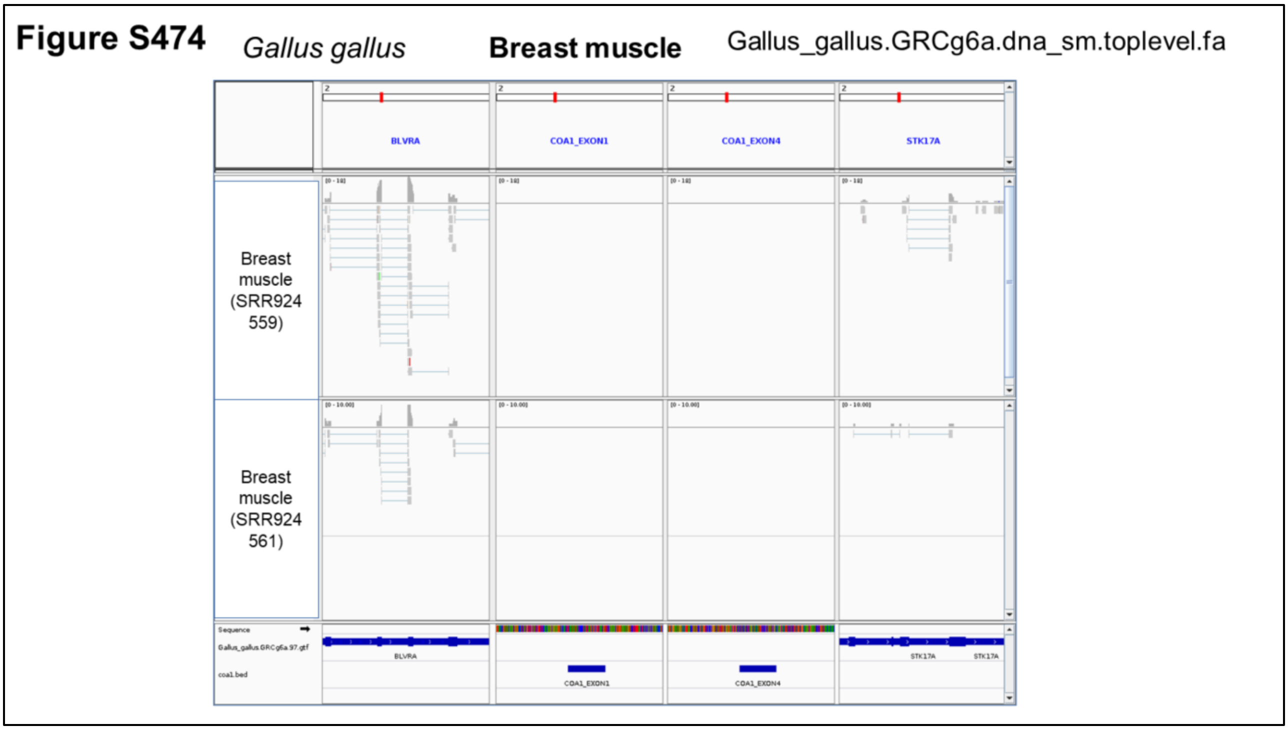

Figure S474: Screenshot of RNA-seq reads aligned to *BLVRA* and *STK17A* to *COA1* exon 1 and 4 respectively in chicken (*Gallus gallus*). Gray boxes represent reads aligned to the genome and thin lines connecting these boxes represent the spliced read alignments. Datasets from breast muscle (SRR924559 and SRR924561) have been mapped to the Gallus_gallus.GRCg6a.dna_sm.toplevel.fa genome. Locations of each of the exons are depicted as bed record in the bottom row.

Figure S475

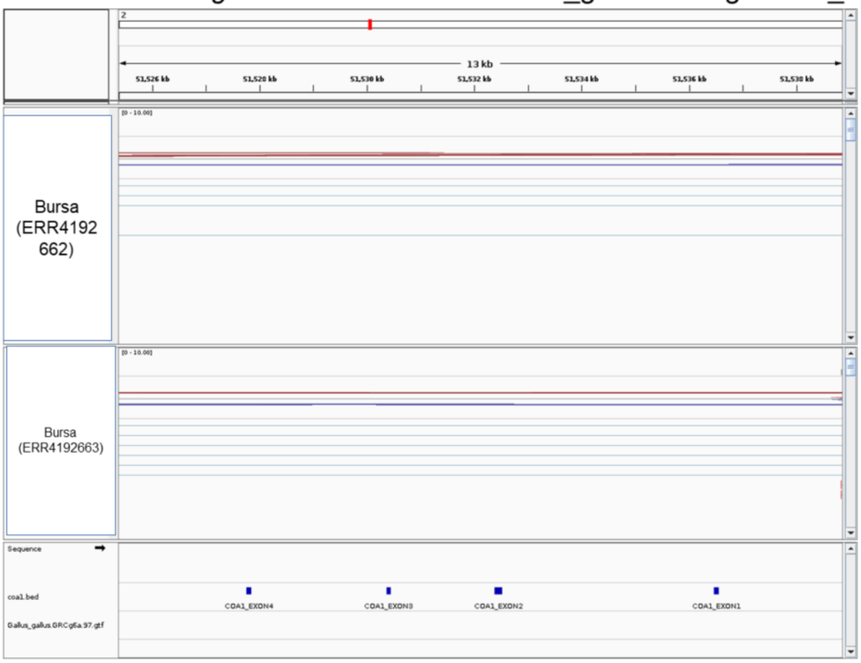

Figure S475: Screenshot of RNA-seq reads aligned to each of the putative locations of the four *COA1* exons in chicken (*Gallus gallus*). Gray boxes represent reads aligned to the genome and thin lines connecting these boxes represent the spliced read alignments. Datasets from bursa (ERR4192662 and ERR4192663) have been mapped to the Gallus_gallus.GRCg6a.dna_sm.toplevel.fa genome. Locations of each of the exons are depicted as bed record in the bottom row.

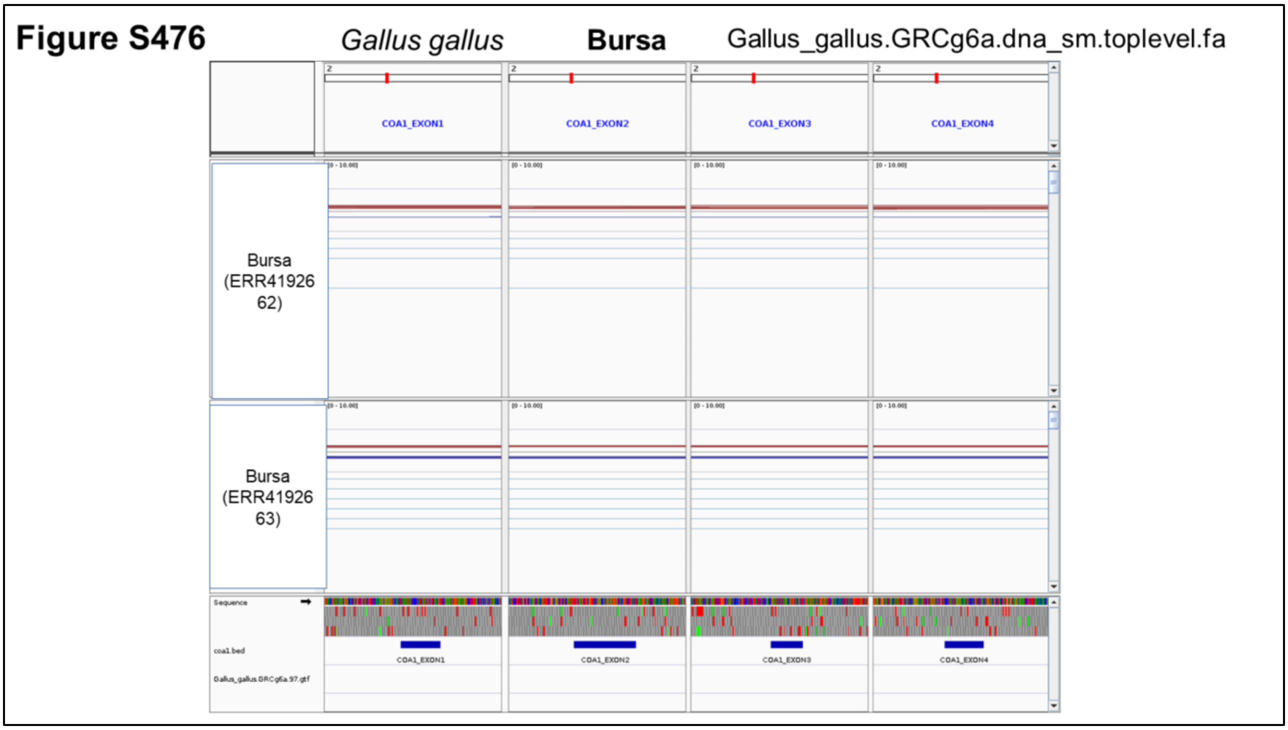

Figure S476: A zoomed in view of each of the *COA1* exons shown in **Supplementary Fig. S475**.

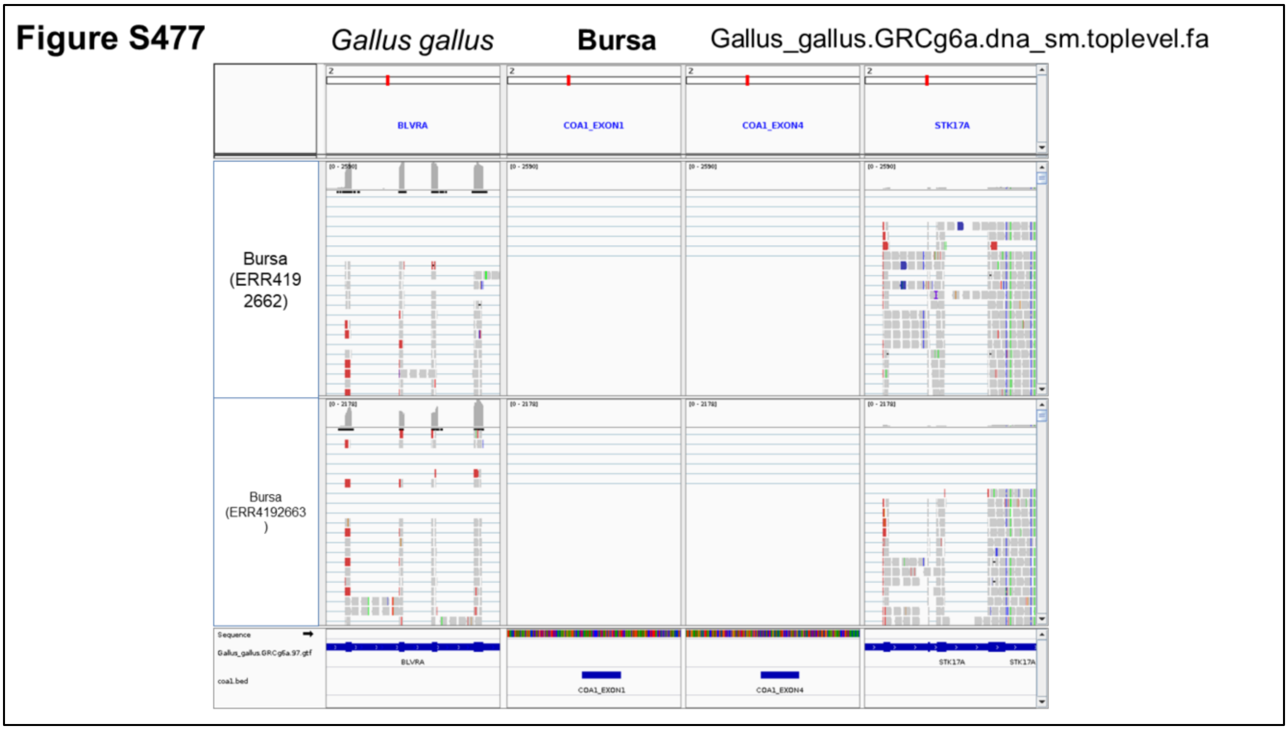

Figure. S477: Screenshot of RNA-seq reads aligned to *BLVRA* and *STK17A* to *COA1* exon 1 and 4 respectively in chicken (*Gallus gallus*). Gray boxes represent reads aligned to the genome and thin lines connecting these boxes represent the spliced read alignments. Datasets from bursa (ERR4192662 and ERR4192663) have been mapped to the Gallus_gallus.GRCg6a.dna_sm.toplevel.fa genome. Locations of each of the exons are depicted as bed record in the bottom row.

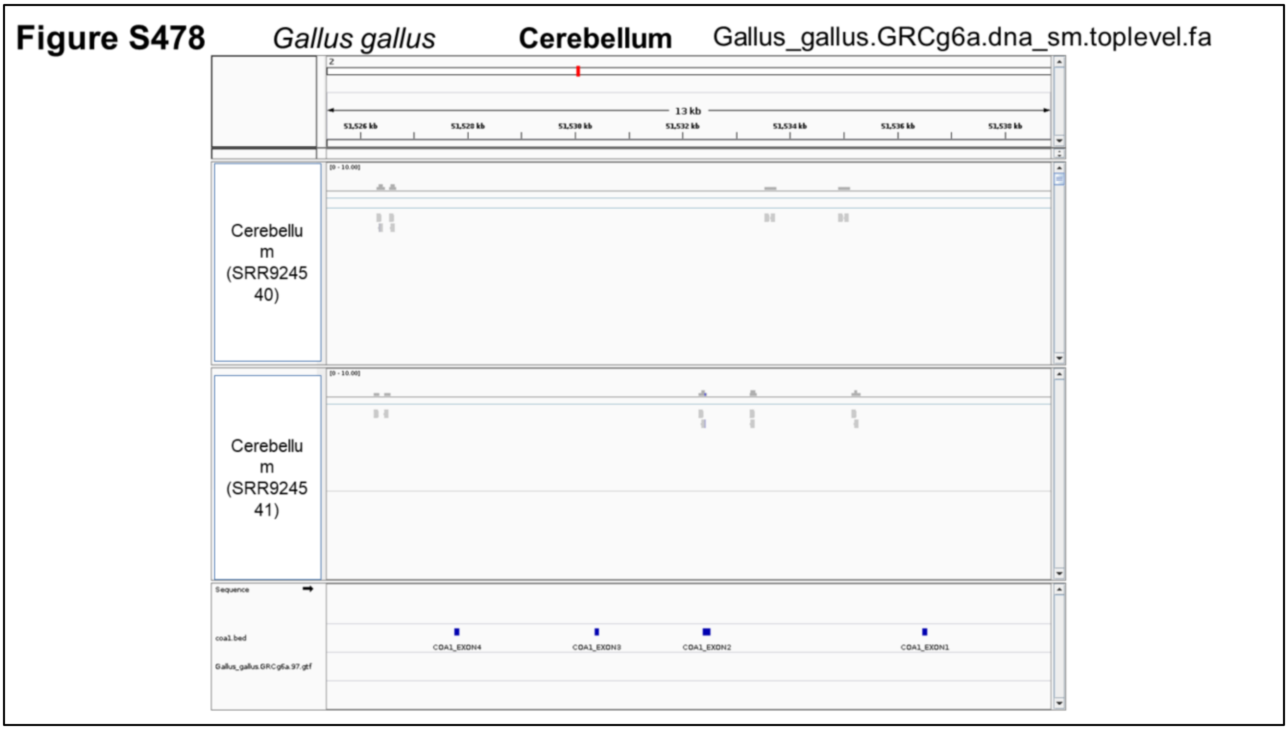

Figure S478: Screenshot of RNA-seq reads aligned to each of the putative locations of the four *COA1* exons in chicken (*Gallus gallus*). Gray boxes represent reads aligned to the genome and thin lines connecting these boxes represent the spliced read alignments. Datasets from Cerebellum (SRR924540 and SRR924541) have been mapped to the Gallus_gallus.GRCg6a.dna_sm.toplevel.fa genome. Locations of each of the exons are depicted as bed record in the bottom row.

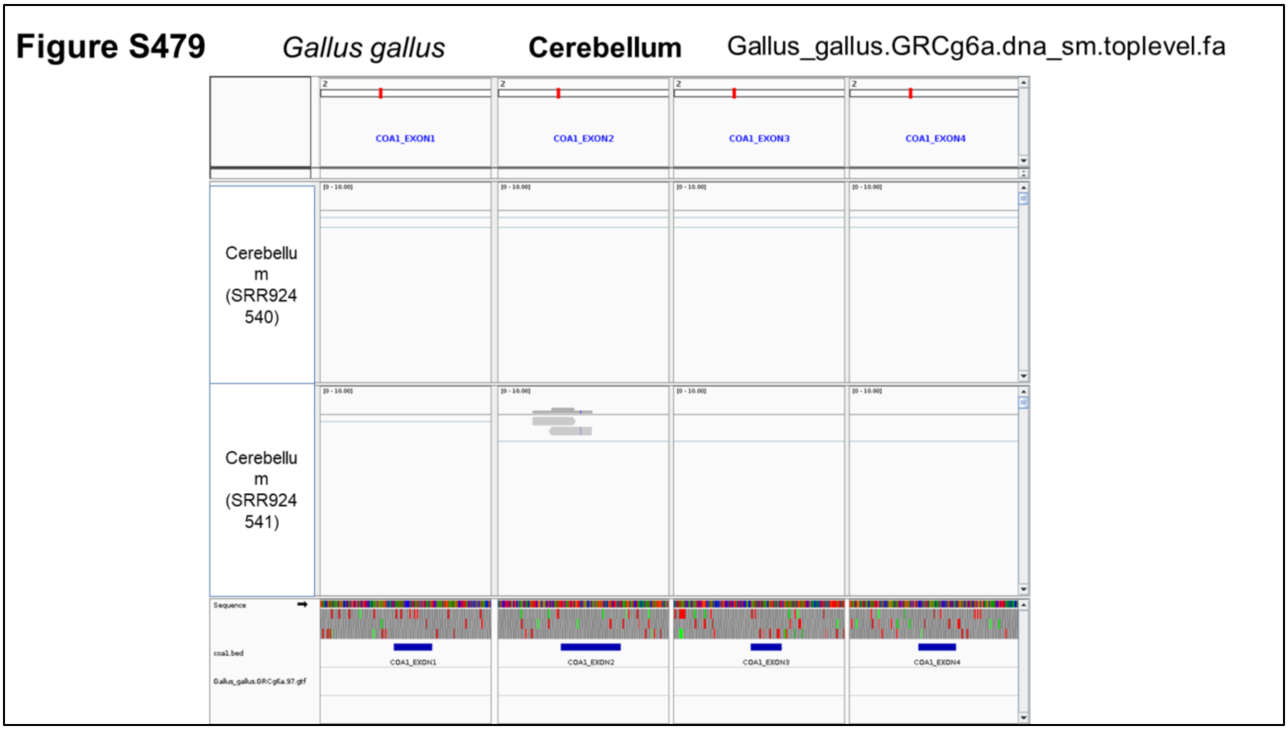

Figure S479: A zoomed in view of each of the *COA1* exons shown in **Supplementary Fig. S478**.

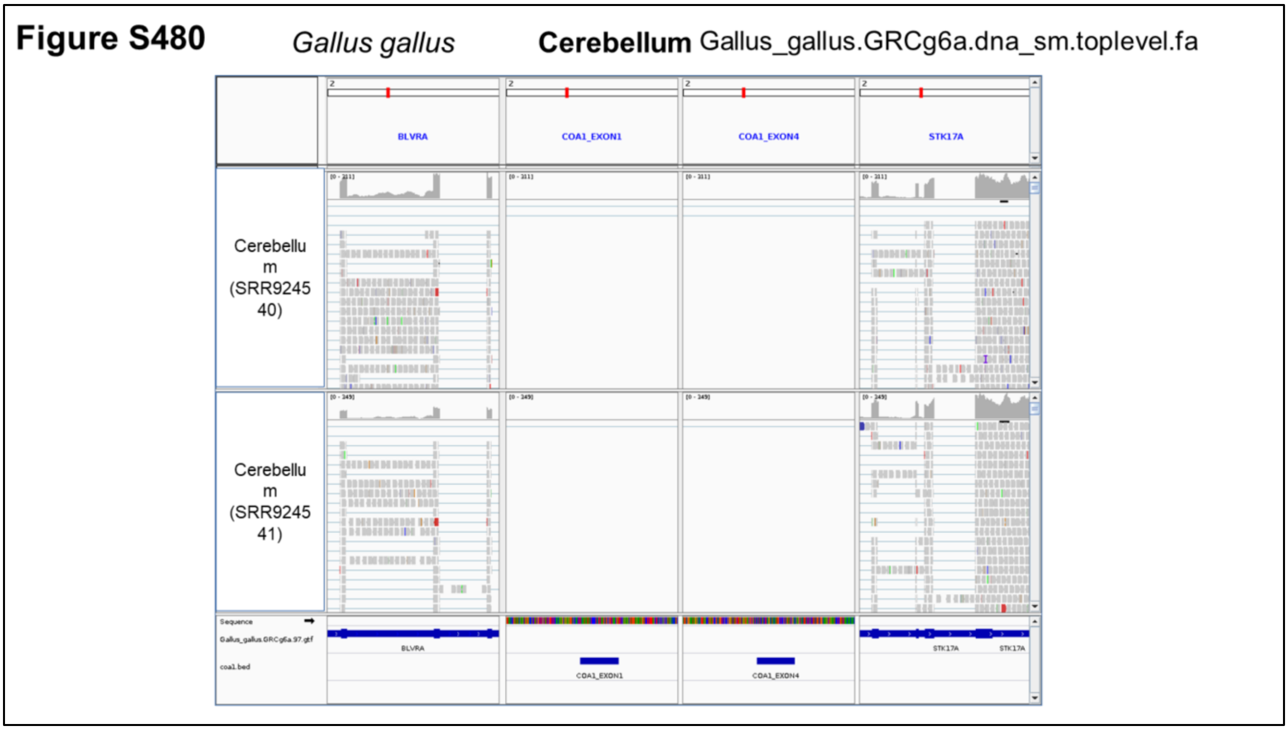

Figure S480: Screenshot of RNA-seq reads aligned to *BLVRA* and *STK17A* to *COA1* exon 1 and 4 respectively in chicken (*Gallus gallus*). Gray boxes represent reads aligned to the genome and thin lines connecting these boxes represent the spliced read alignments. Datasets from cerebellum (SRR924540 and SRR924541) have been mapped to the Gallus_gallus.GRCg6a.dna_sm.toplevel.fa genome. Locations of each of the exons are depicted as bed record in the bottom row.

Figure S481 *Gallus gallus* **Cerebrum** Gallus_gallus.GRCg6a.dna_sm.toplevel.fa

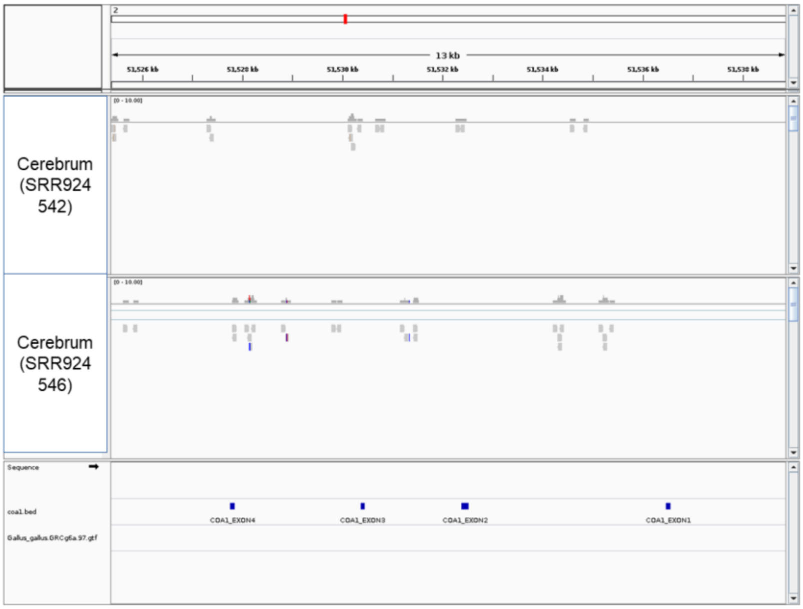

Figure S481: Screenshot of RNA-seq reads aligned to each of the putative locations of the four *COA1* exons in chicken (*Gallus gallus*). Gray boxes represent reads aligned to the genome and thin lines connecting these boxes represent the spliced read alignments. Datasets from cerebrum (SRR924542 and SRR924546) have been mapped to the Gallus_gallus.GRCg6a.dna_sm.toplevel.fa genome. Locations of each of the exons are depicted as bed record in the bottom row.

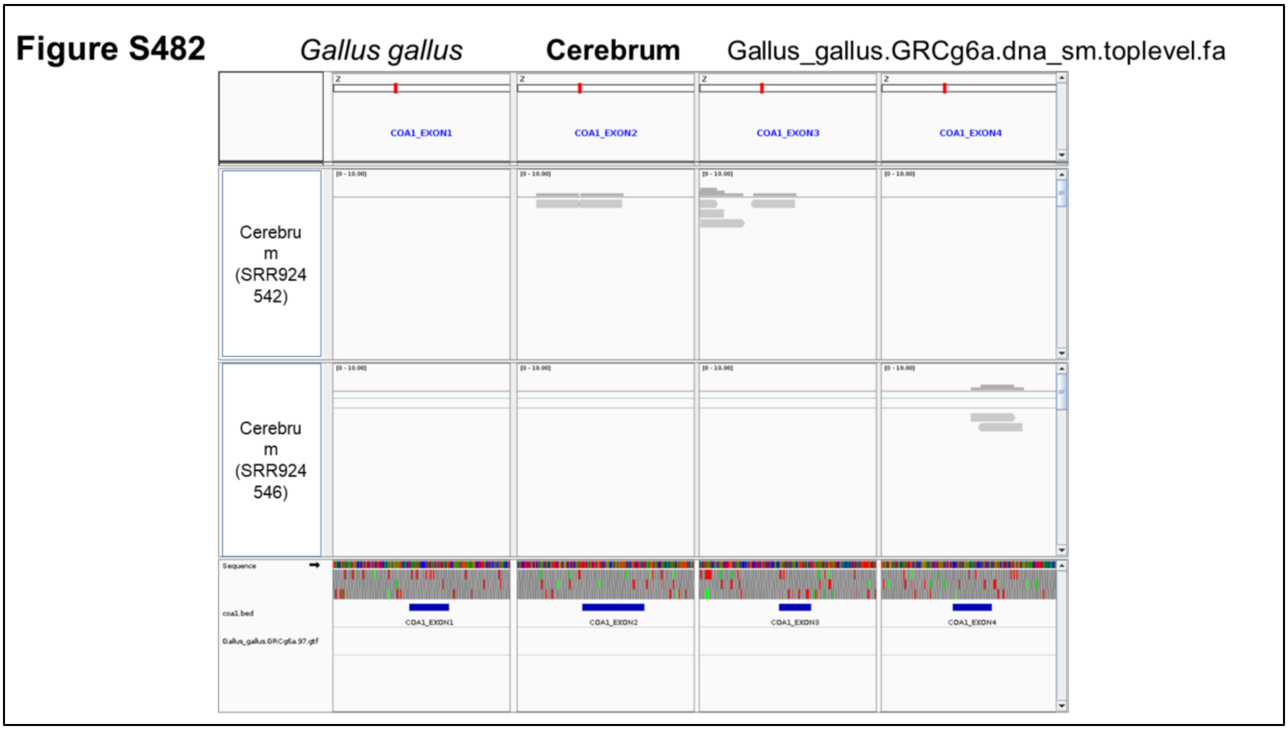

Figure S482: A zoomed in view of each of the *COA1* exons shown in **Supplementary Fig. S481**.

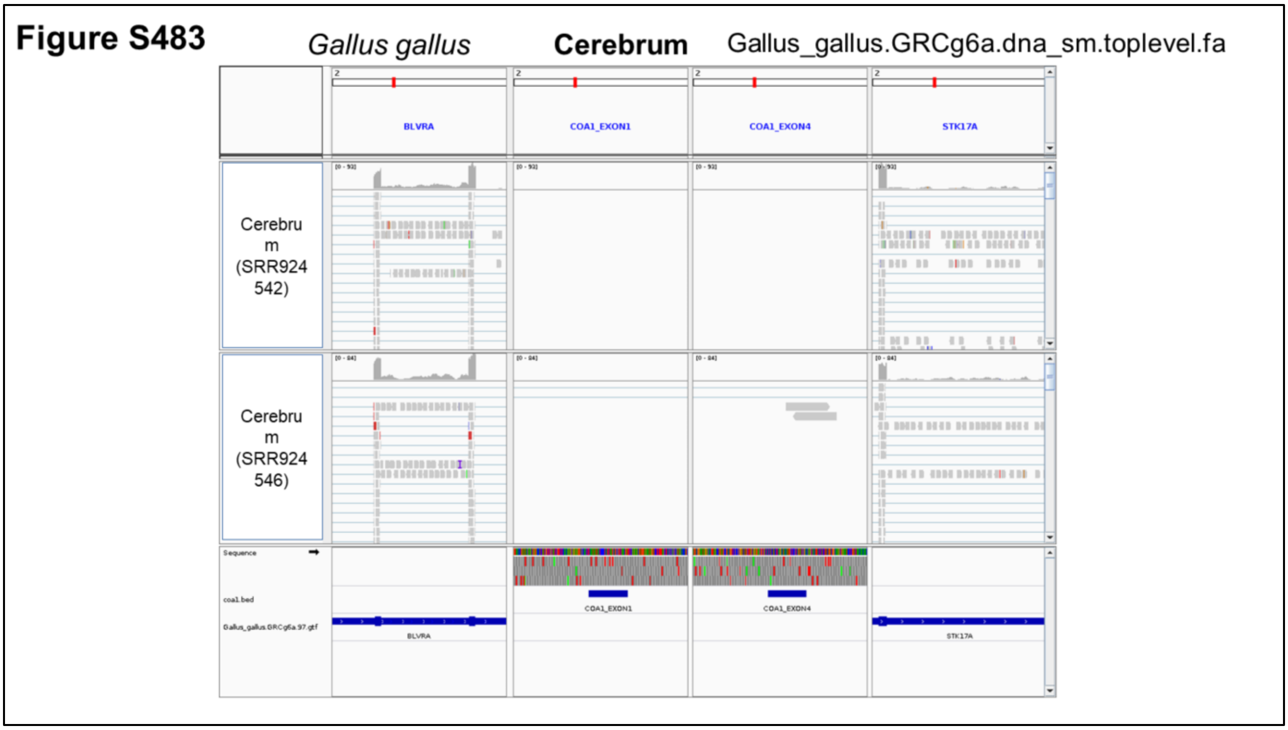

Figure S483: Screenshot of RNA-seq reads aligned to *BLVRA* and *STK17A* to *COA1* exon 1 and 4 respectively in chicken (*Gallus gallus*). Gray boxes represent reads aligned to the genome and thin lines connecting these boxes represent the spliced read alignments. Datasets from cerebrum (SRR924542 and SRR924546) have been mapped to the Gallus_gallus.GRCg6a.dna_sm.toplevel.fa genome. Locations of each of the exons are depicted as bed record in the bottom row.

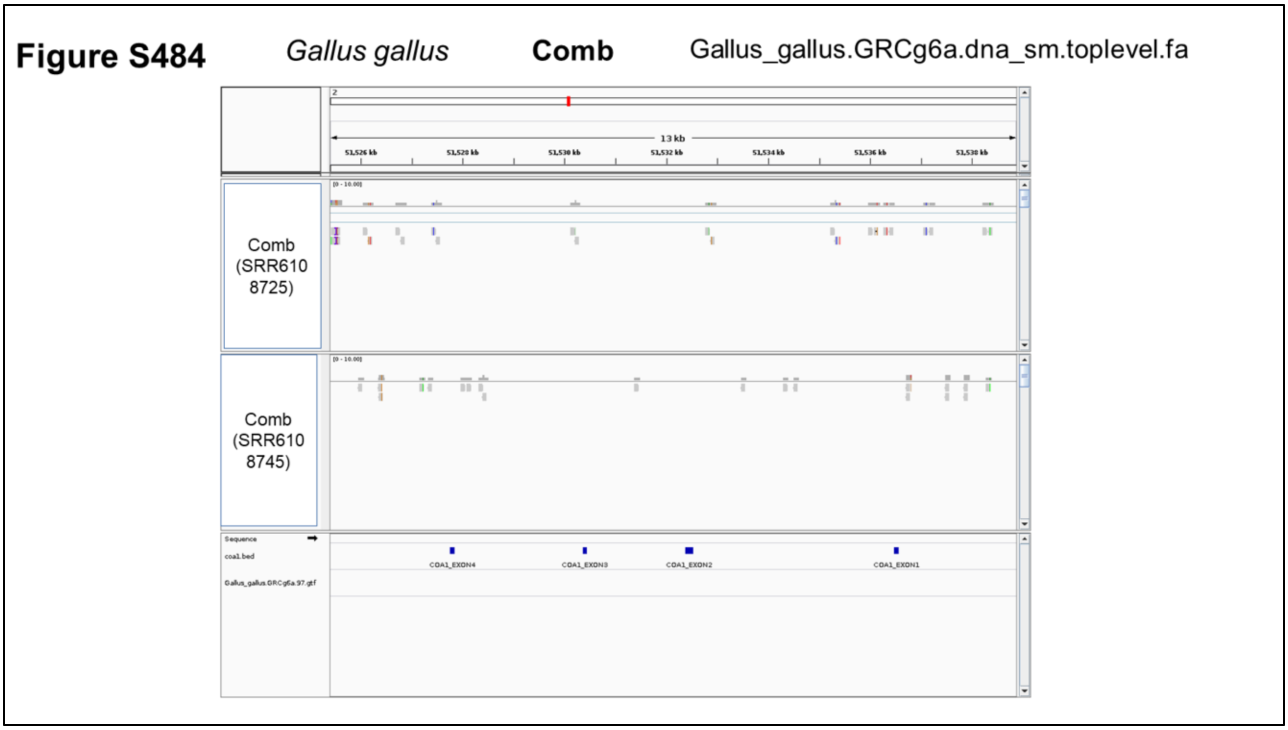

Figure S484: Screenshot of RNA-seq reads aligned to each of the putative locations of the four *COA1* exons in chicken (*Gallus gallus*). Gray boxes represent reads aligned to the genome and thin lines connecting these boxes represent the spliced read alignments. Datasets from comb (SRR6108725) and (SRR6108745) have been mapped to the Gallus_gallus.GRCg6a.dna_sm.toplevel.fa genome. Locations of each of the exons are depicted as bed record in the bottom row.

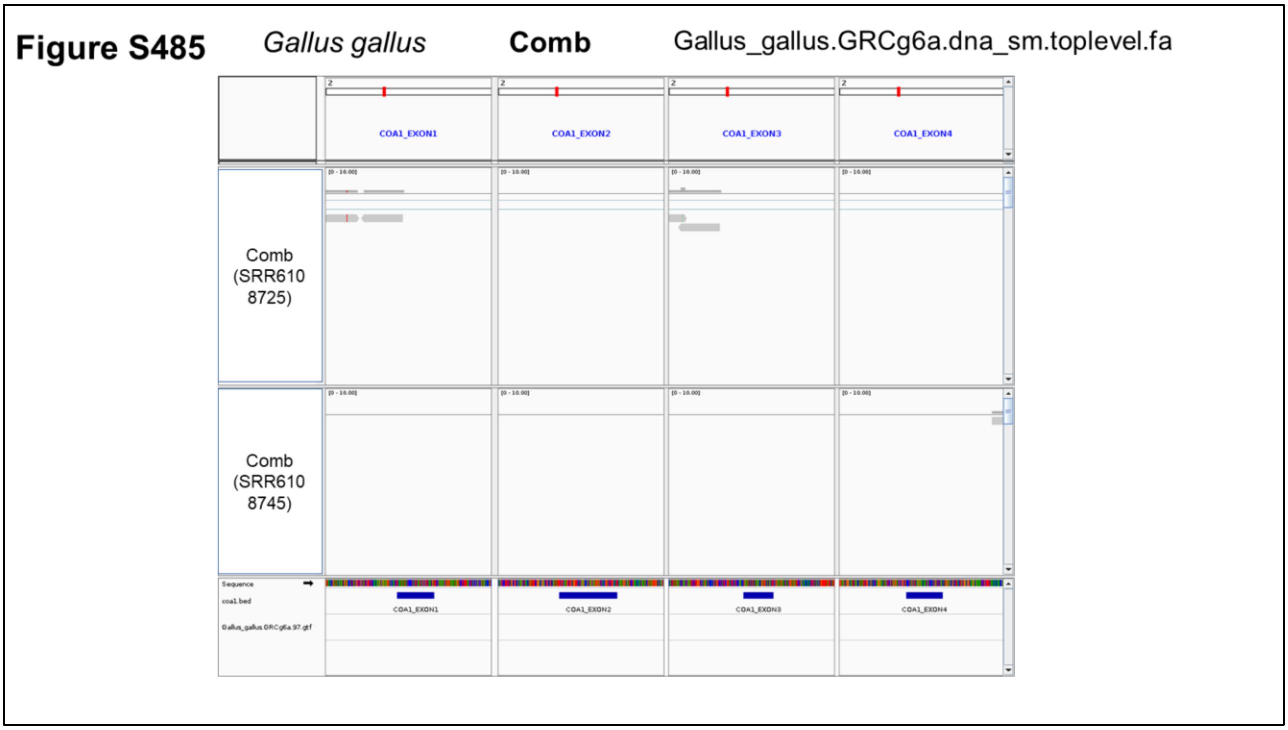

Figure S485 A zoomed in view of each of the *COA1* exons shown in **Supplementary Fig. S484**.

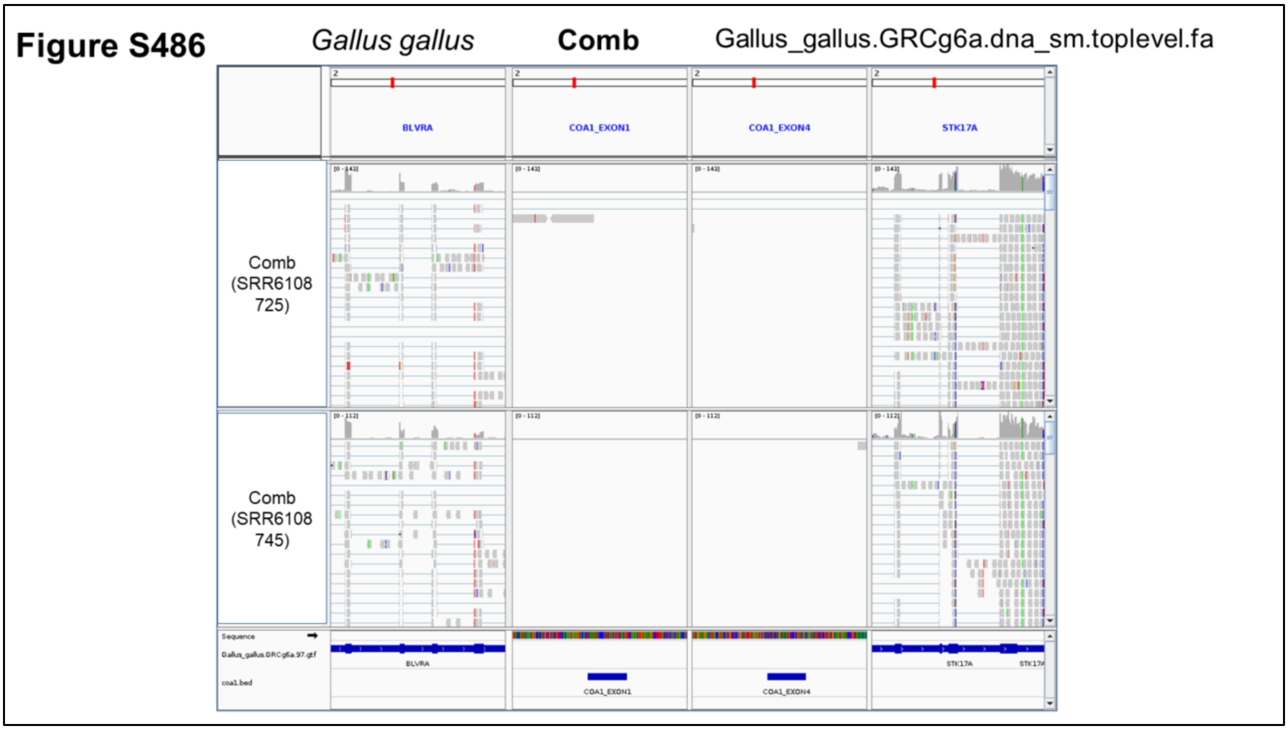

Figure S486: Screenshot of RNA-seq reads aligned to *BLVRA* and *STK17A* to *COA1* exon 1 and 4 respectively in chicken (*Gallus gallus*). Gray boxes represent reads aligned to the genome and thin lines connecting these boxes represent the spliced read alignments. Datasets from comb (SRR6108725) and (SRR6108745) have been mapped to the Gallus_gallus.GRCg6a.dna_sm.toplevel.fa genome. Locations of each of the exons are depicted as bed record in the bottom row.

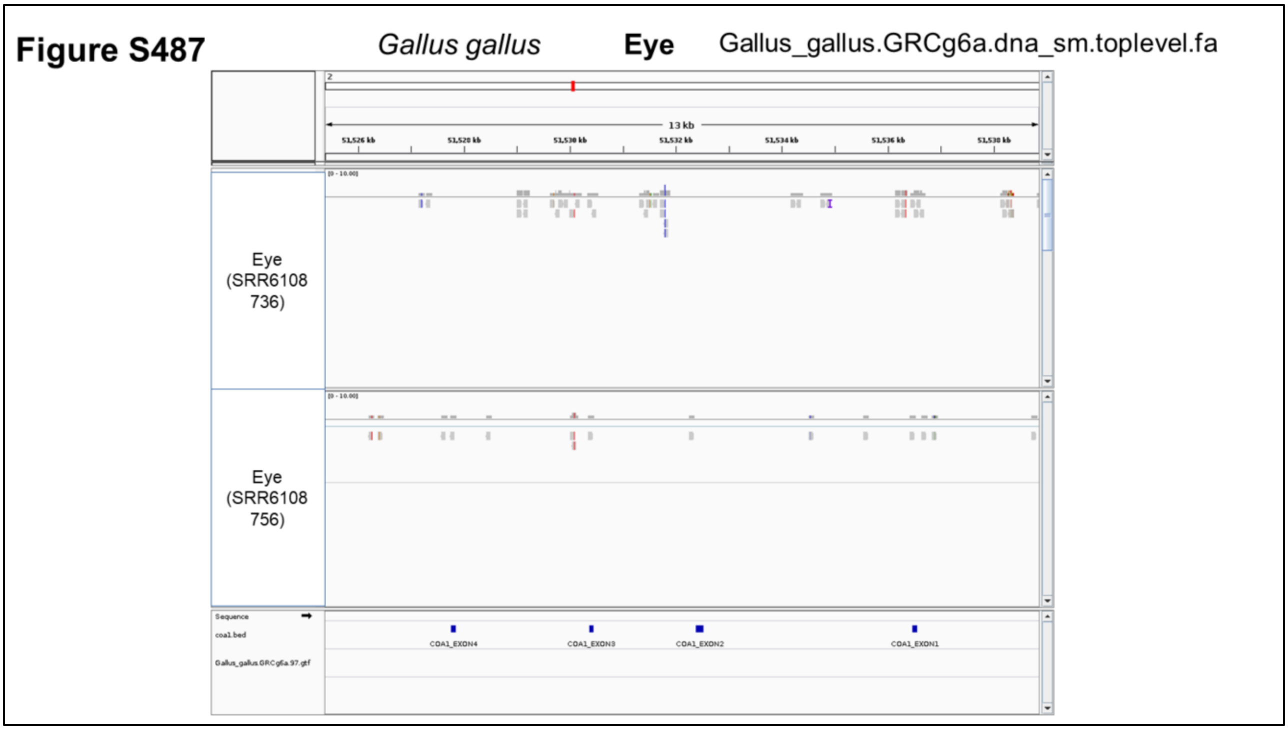

Figure S487: Screenshot of RNA-seq reads aligned to each of the putative locations of the four *COA1* exons in chicken (*Gallus gallus*). Gray boxes represent reads aligned to the genome and thin lines connecting these boxes represent the spliced read alignments. Datasets from eye (SRR6108736) and (SRR6108756) have been mapped to the Gallus_gallus.GRCg6a.dna_sm.toplevel.fa genome. Locations of each of the exons are depicted as bed record in the bottom row.

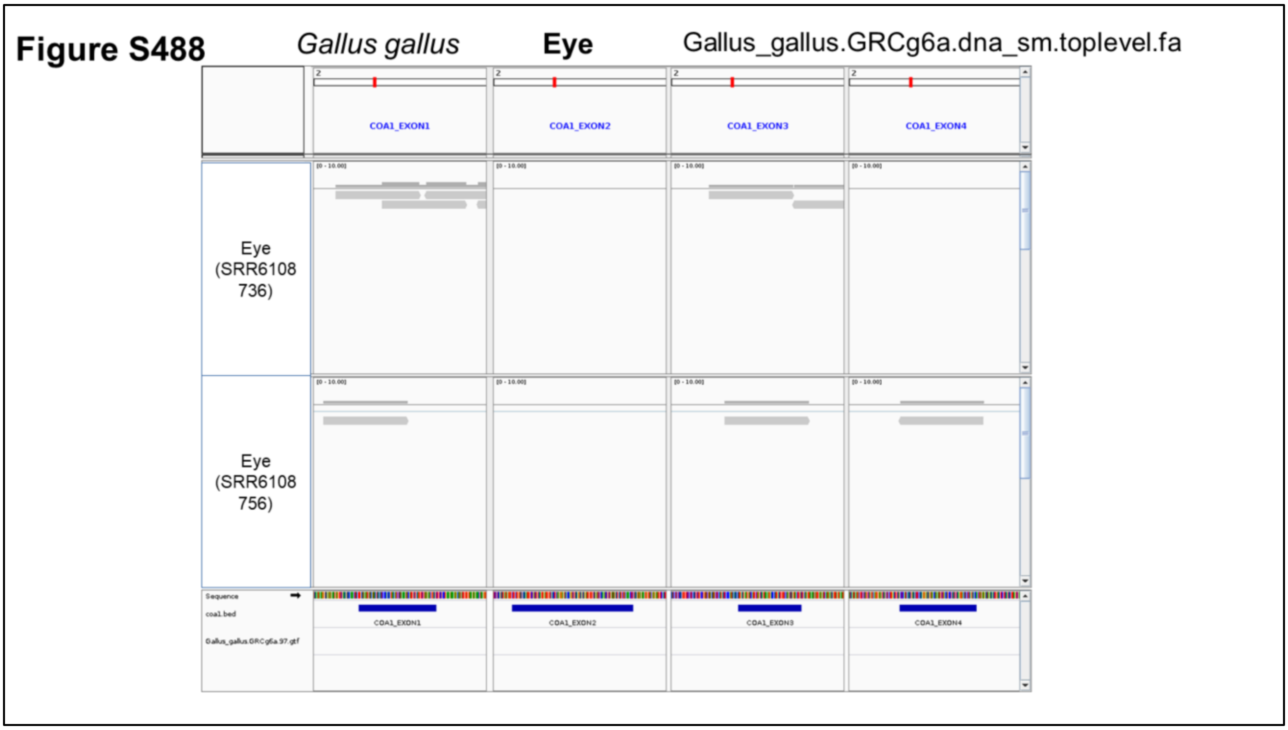

Figure S488: A zoomed in view of each of the *COA1* exons shown in **Supplementary Fig. S487**.

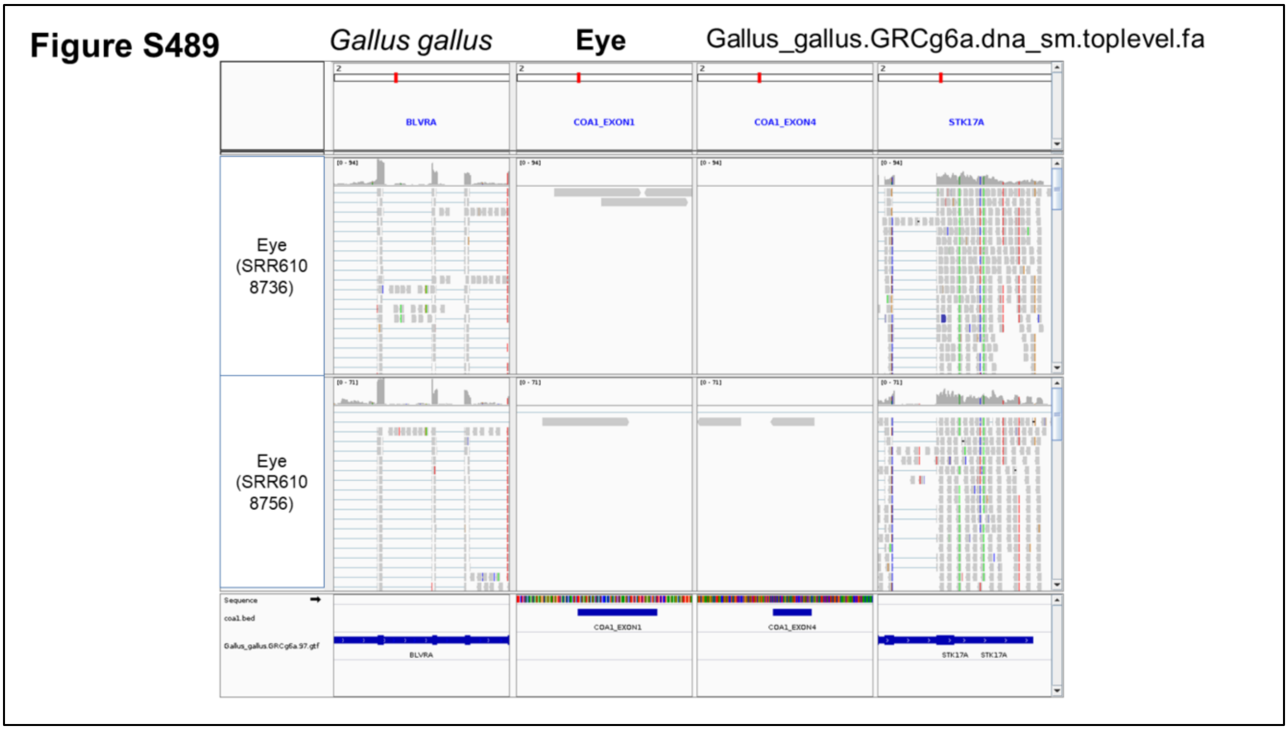

Figure S489: Screenshot of RNA-seq reads aligned to *BLVRA* and *STK17A* to *COA1* exon 1 and 4 respectively in chicken (*Gallus gallus*). Gray boxes represent reads aligned to the genome and thin lines connecting these boxes represent the spliced read alignments. Datasets from eye (SRR6108736) and (SRR6108756) have been mapped to the Gallus_gallus.GRCg6a.dna_sm.toplevel.fa genome. Locations of each of the exons are depicted as bed record in the bottom row.

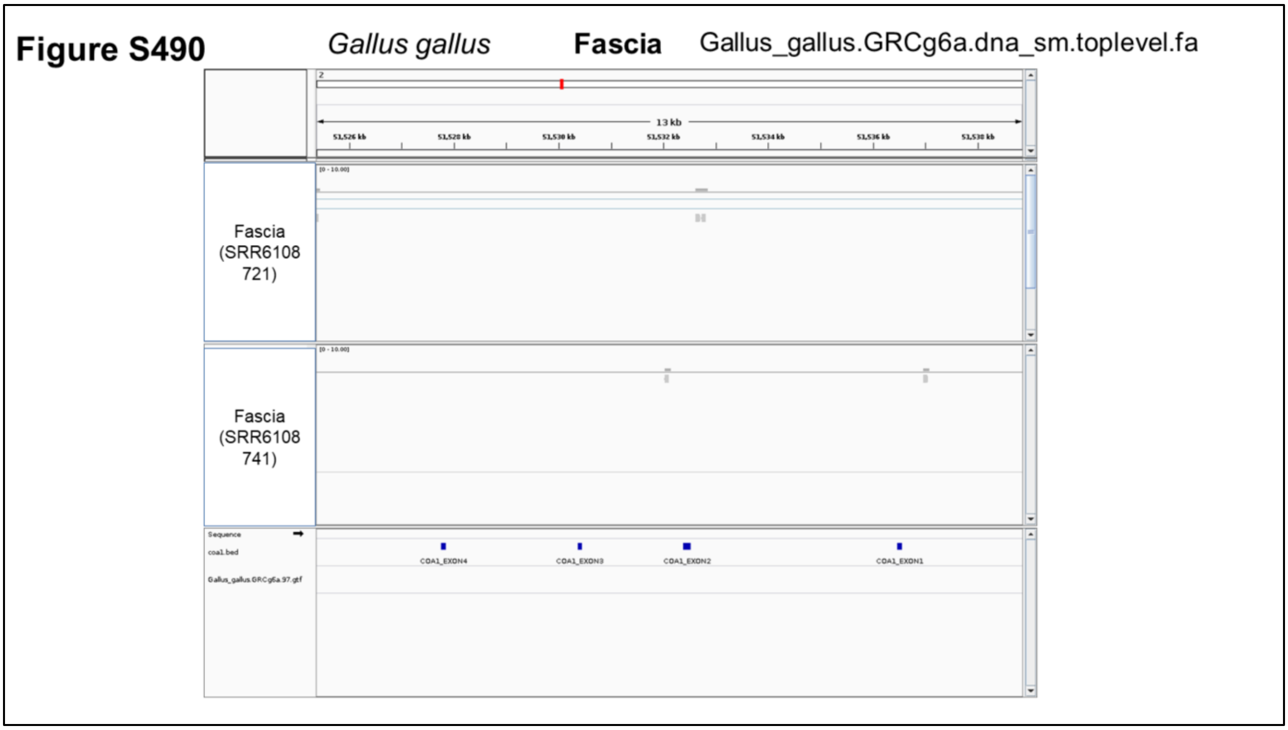

Figure S490: Screenshot of RNA-seq reads aligned to each of the putative locations of the four *COA1* exons in chicken (*Gallus gallus*). Gray boxes represent reads aligned to the genome and thin lines connecting these boxes represent the spliced read alignments. Datasets from fascia (SRR6108721) and (SRR6108741) have been mapped to the Gallus_gallus.GRCg6a.dna_sm.toplevel.fa genome. Locations of each of the exons are depicted as bed record in the bottom row.

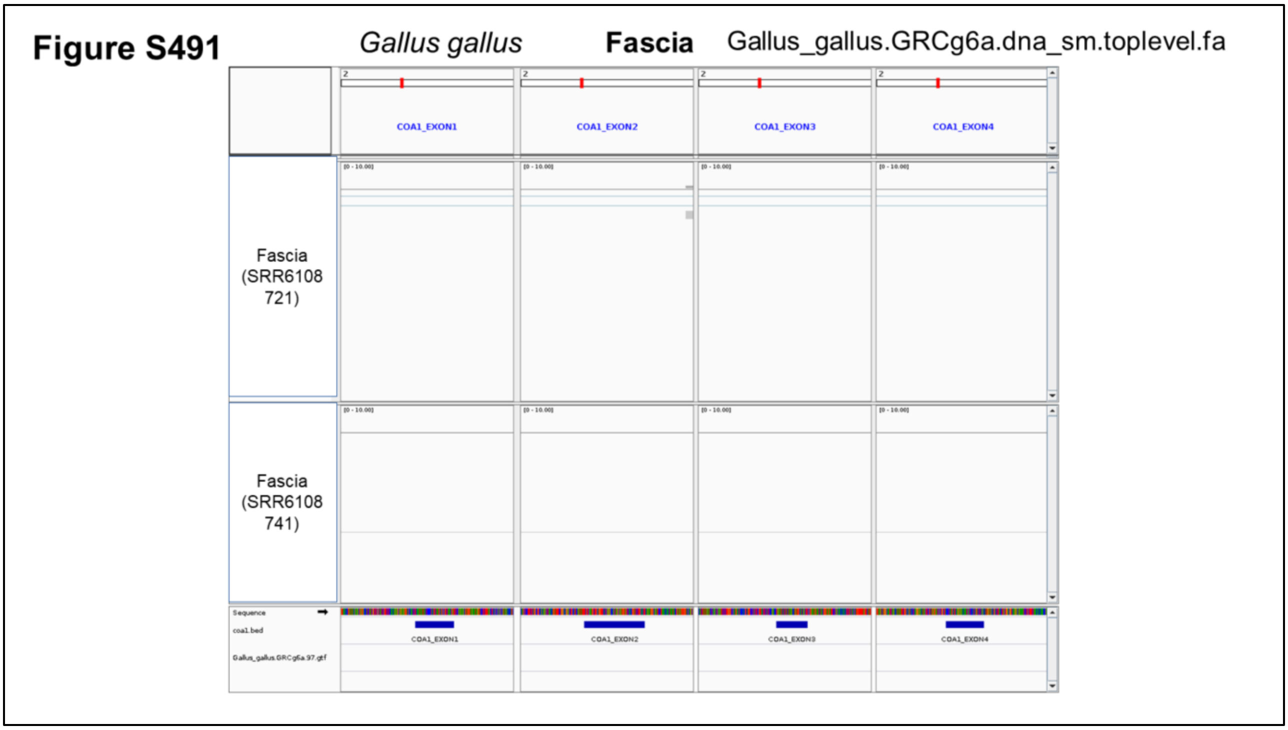

Figure S491: A zoomed in view of each of the *COA1* exons shown in **Supplementary Fig. S490**.

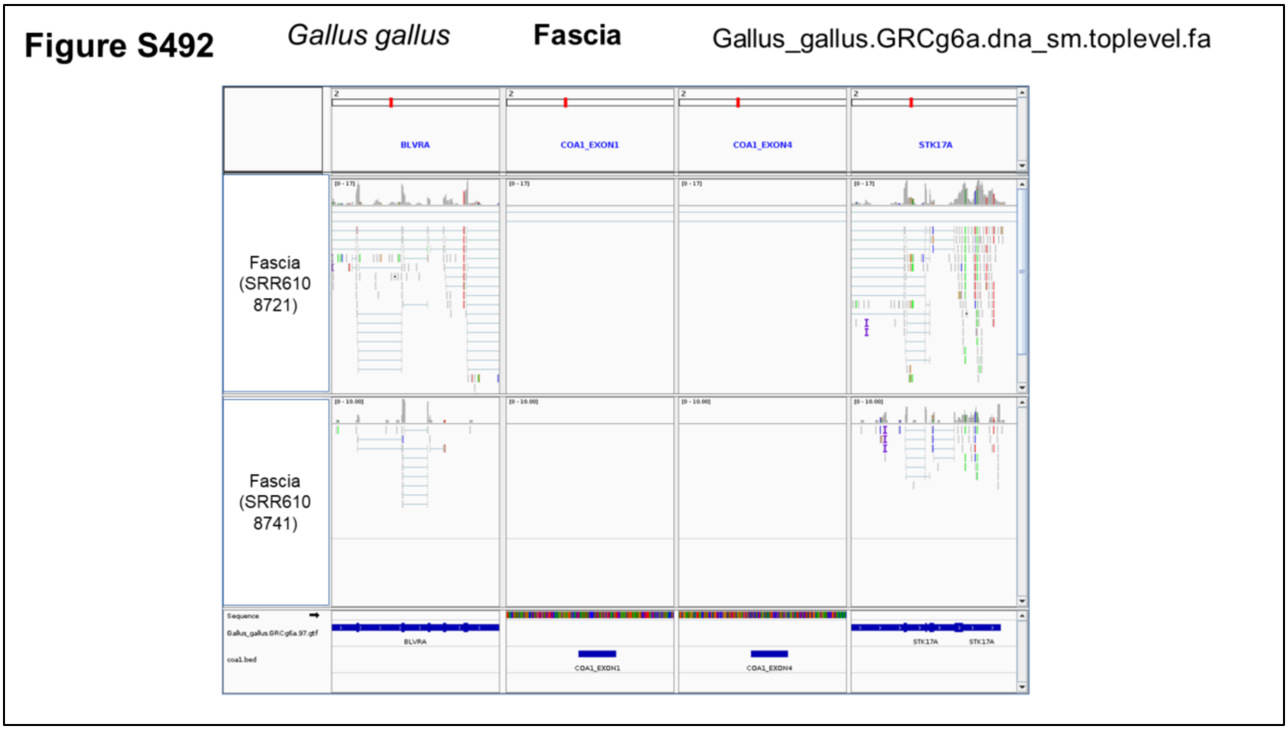

Figure S492: Screenshot of RNA-seq reads aligned to *BLVRA* and *STK17A* to *COA1* exon 1 and 4 respectively in chicken (*Gallus gallus*). Gray boxes represent reads aligned to the genome and thin lines connecting these boxes represent the spliced read alignments. Datasets from fascia (SRR6108721) and (SRR6108741) have been mapped to the Gallus_gallus.GRCg6a.dna_sm.toplevel.fa genome. Locations of each of the exons are depicted as bed record in the bottom row.

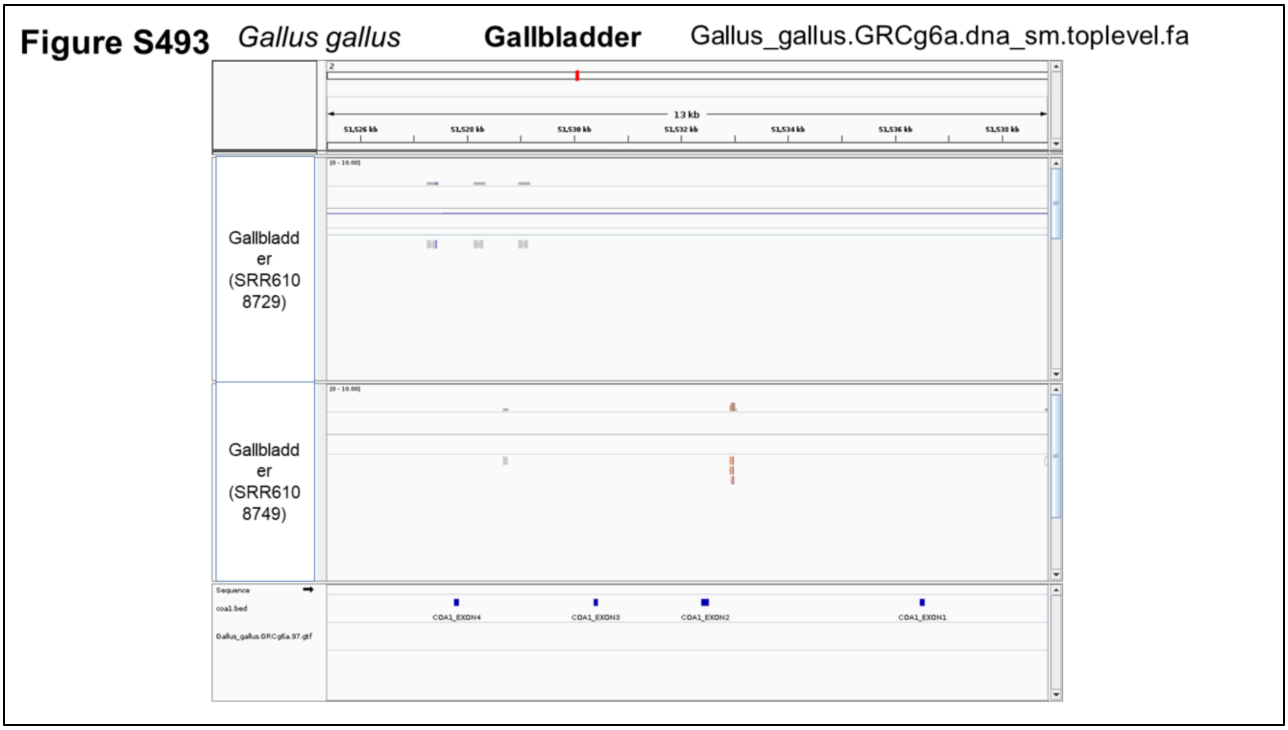

Figure S493: Screenshot of RNA-seq reads aligned to each of the putative locations of the four *COA1* exons in chicken (*Gallus gallus*). Gray boxes represent reads aligned to the genome and thin lines connecting these boxes represent the spliced read alignments. Datasets from gallbladder (SRR6108729) and (SRR6108749) have been mapped to the Gallus_gallus.GRCg6a.dna_sm.toplevel.fa genome. Locations of each of the exons are depicted as bed record in the bottom row.

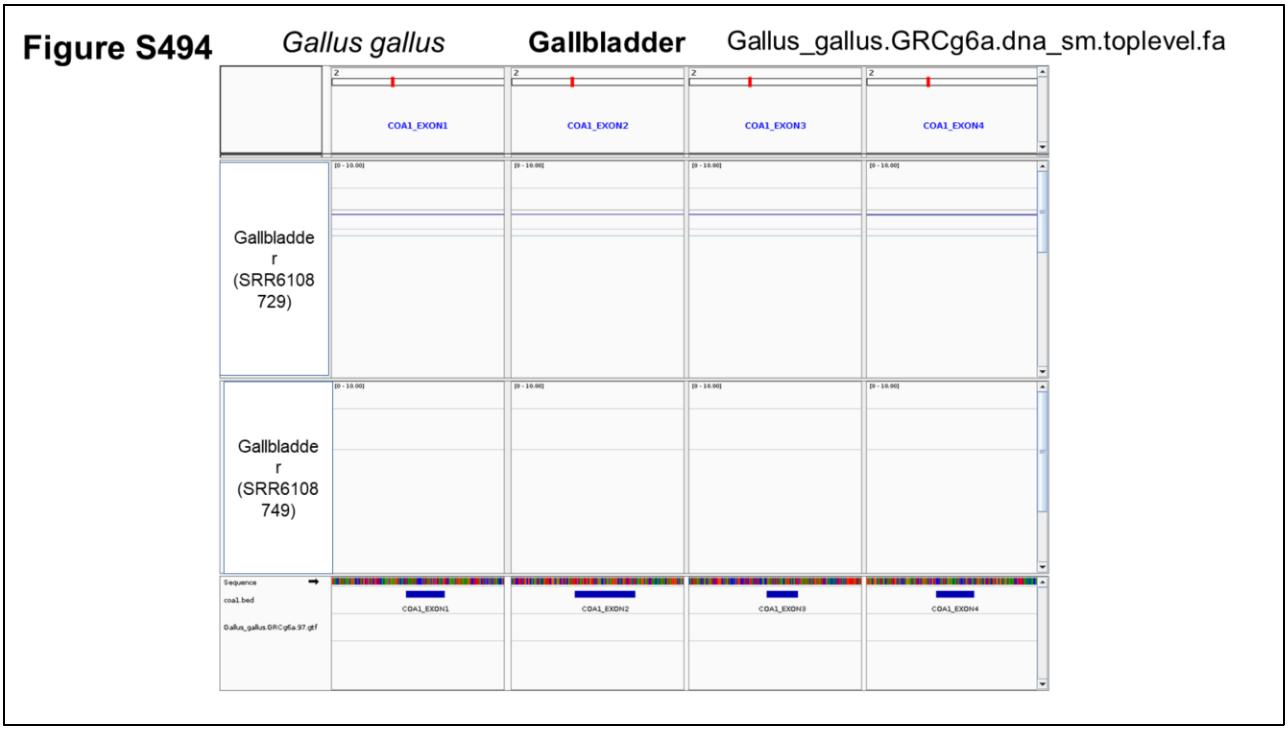

Figure S494: A zoomed in view of each of the *COA1* exons shown in **Supplementary Fig. S493**.

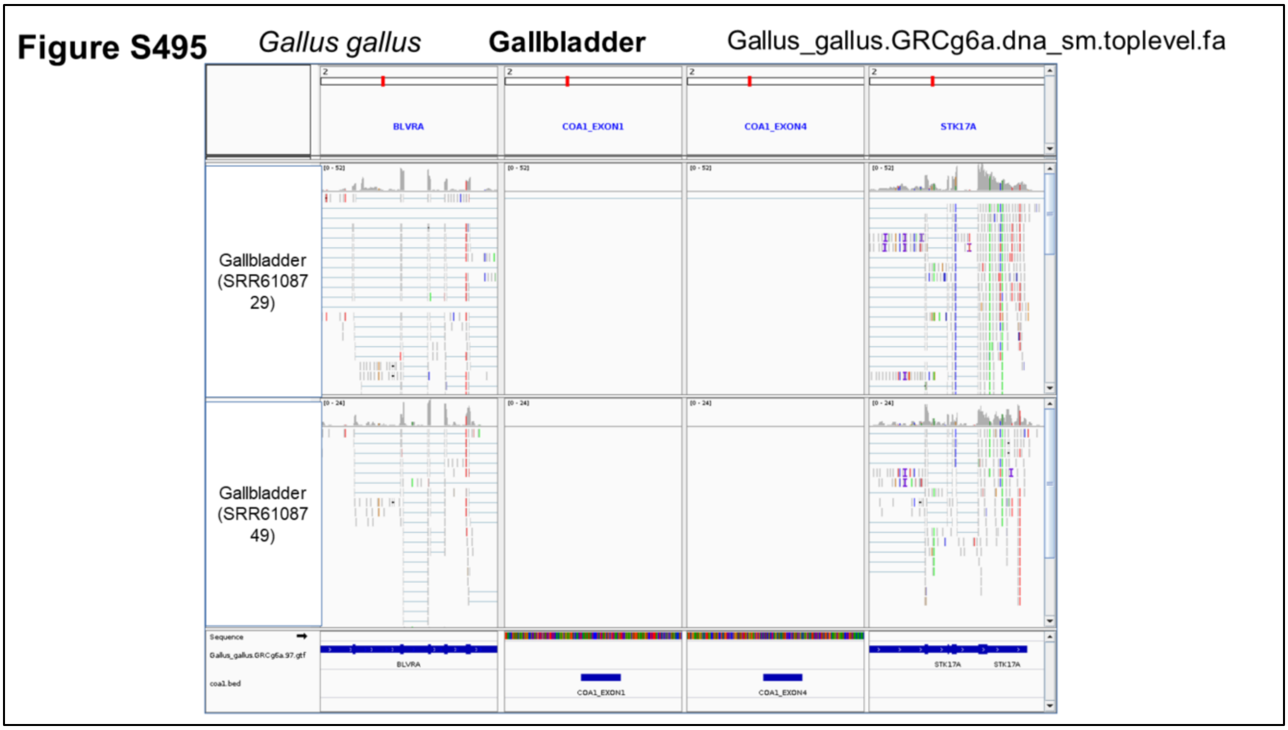

Figure S495: Screenshot of RNA-seq reads aligned to *BLVRA* and *STK17A* to *COA1* exon 1 and 4 respectively in chicken (*Gallus gallus*). Gray boxes represent reads aligned to the genome and thin lines connecting these boxes represent the spliced read alignments. Datasets from gallbladder (SRR6108729) and (SRR6108749) have been mapped to the Gallus_gallus.GRCg6a.dna_sm.toplevel.fa genome. Locations of each of the exons are depicted as bed record in the bottom row.

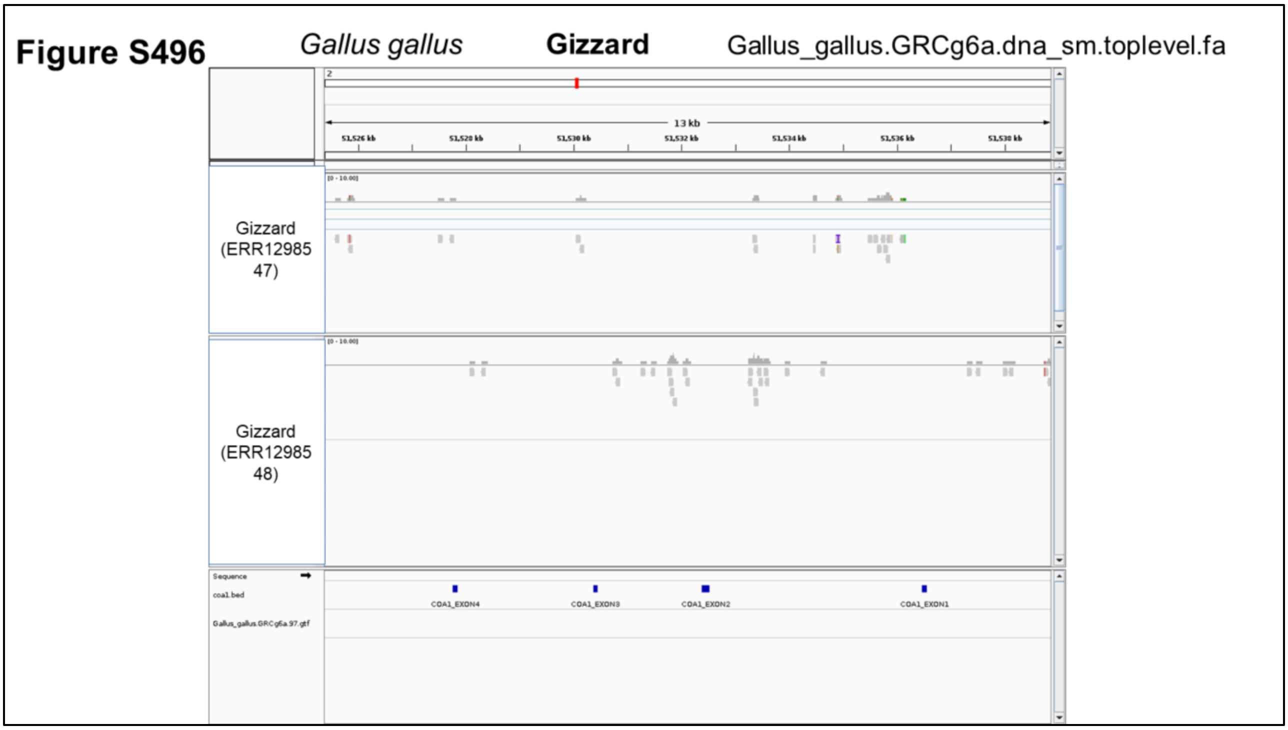

Figure S496: Screenshot of RNA-seq reads aligned to each of the putative locations of the four *COA1* exons in chicken (*Gallus gallus*). Gray boxes represent reads aligned to the genome and thin lines connecting these boxes represent the spliced read alignments. Datasets from gizzard (ERR1298547) and (ERR1298548) have been mapped to the Gallus_gallus.GRCg6a.dna_sm.toplevel.fa genome. Locations of each of the exons are depicted as bed record in the bottom row.

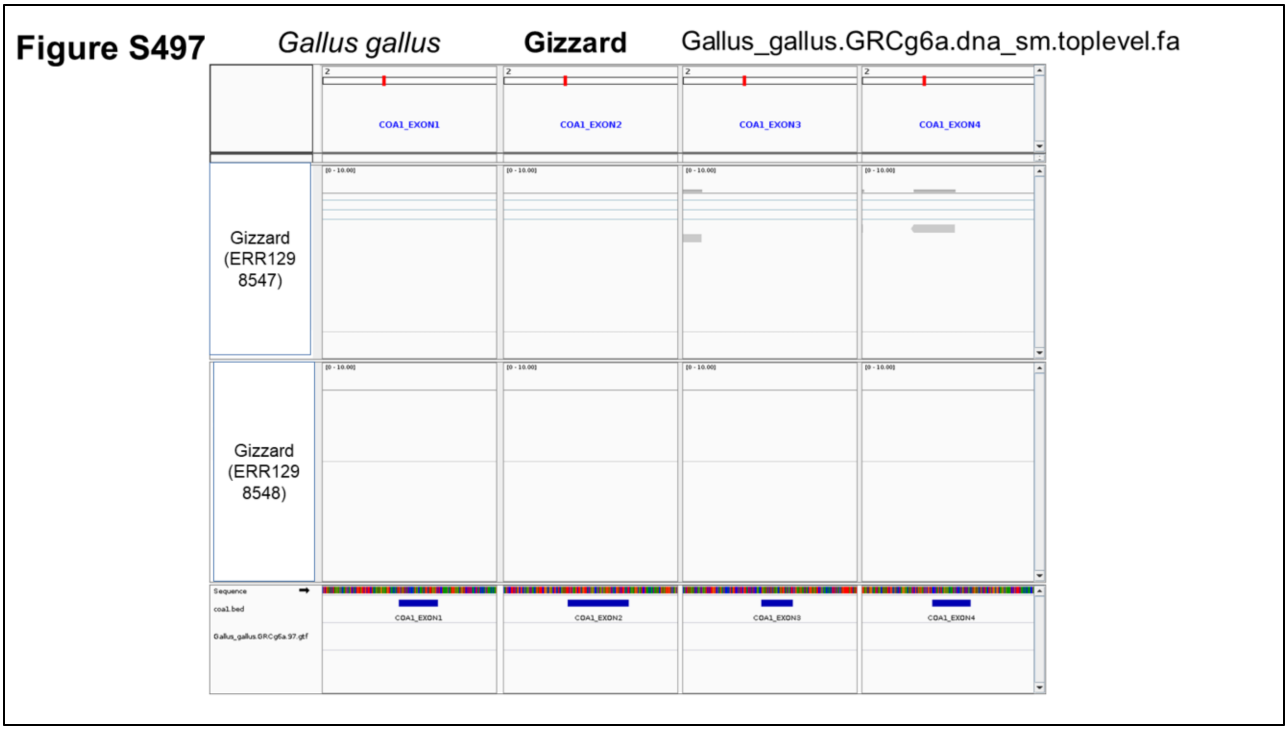

Figure S497: A zoomed in view of each of the *COA1* exons shown in **Supplementary Fig. S496**.

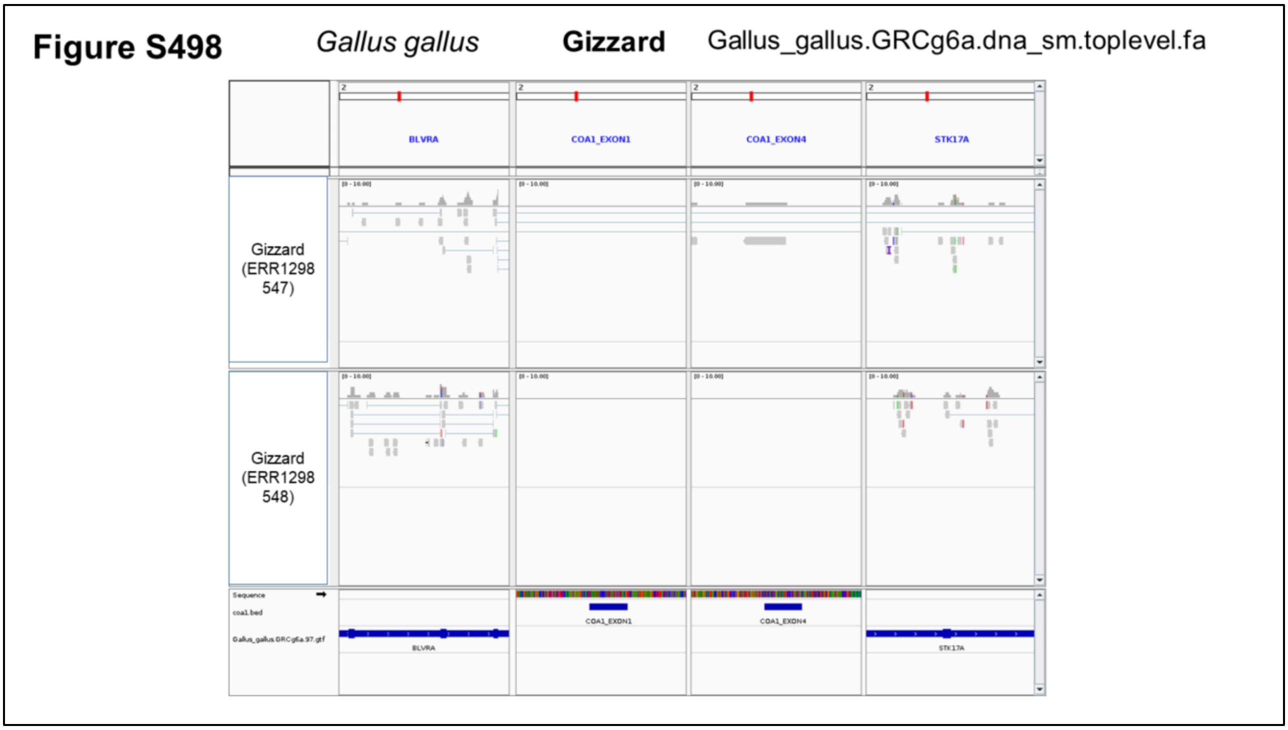

Figure S498: Screenshot of RNA-seq reads aligned to *BLVRA* and *STK17A* to *COA1* exon 1 and 4 respectively in chicken (*Gallus gallus*). Gray boxes represent reads aligned to the genome and thin lines connecting these boxes represent the spliced read alignments. Datasets from gizzard (ERR1298547) and (ERR1298548) have been mapped to the Gallus_gallus.GRCg6a.dna_sm.toplevel.fa genome. Locations of each of the exons are depicted as bed record in the bottom row.

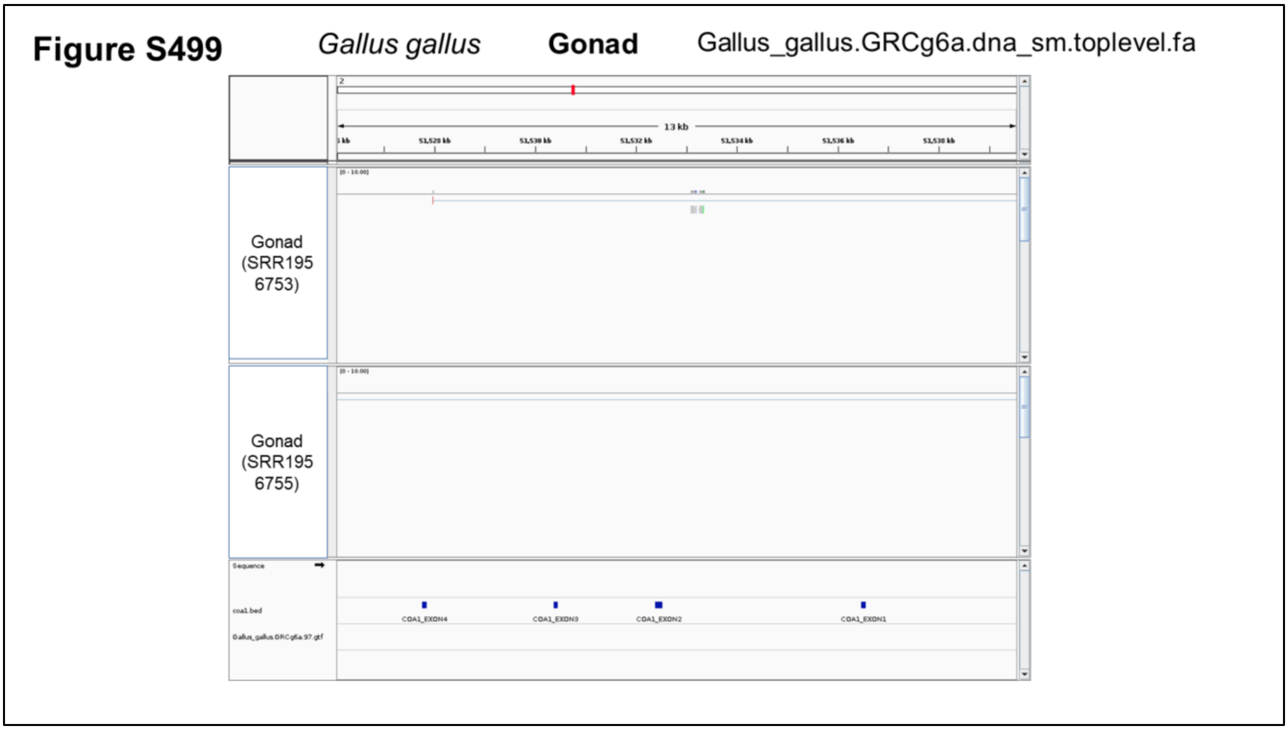

Figure S499: Screenshot of RNA-seq reads aligned to each of the putative locations of the four *COA1* exons in chicken (*Gallus gallus*). Gray boxes represent reads aligned to the genome and thin lines connecting these boxes represent the spliced read alignments. Datasets from gonad (SRR1956753) and (SRR1956755) have been mapped to the Gallus_gallus.GRCg6a.dna_sm.toplevel.fa genome. Locations of each of the exons are depicted as bed record in the bottom row.

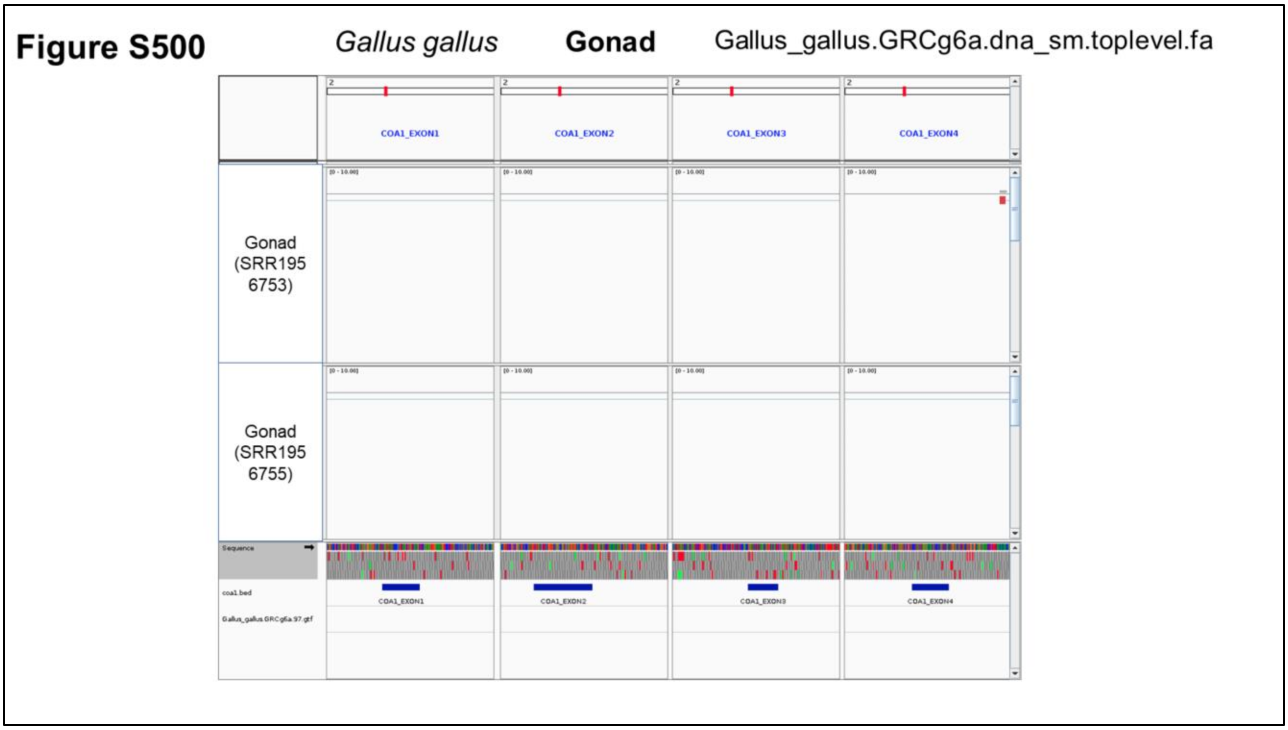

Figure S500: A zoomed in view of each of the *COA1* exons shown in **Supplementary Fig. S499**.

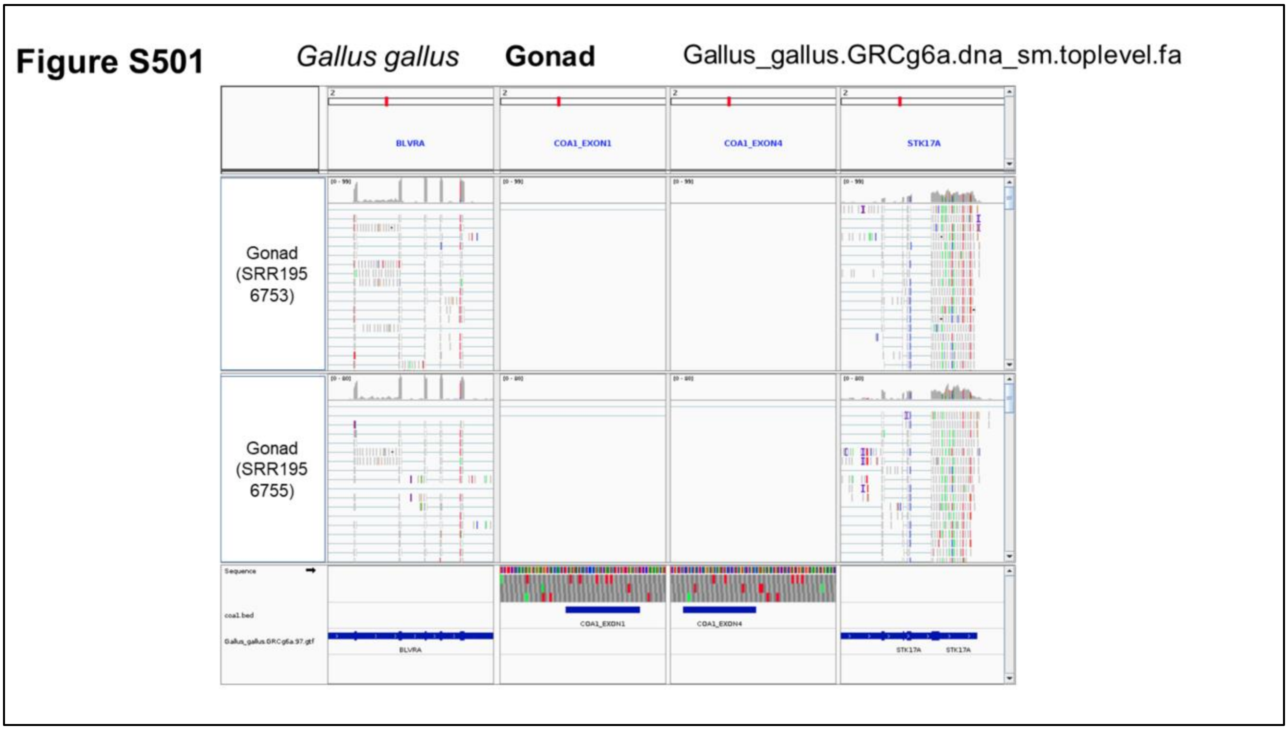

Figure S501: Screenshot of RNA-seq reads aligned to *BLVRA* and *STK17A* to *COA1* exon 1 and 4 respectively in chicken (*Gallus gallus*). Gray boxes represent reads aligned to the genome and thin lines connecting these boxes represent the spliced read alignments. Datasets from gonad (SRR1956753) and (SRR1956755) have been mapped to the Gallus_gallus.GRCg6a.dna_sm.toplevel.fa genome. Locations of each of the exons are depicted as bed record in the bottom row.

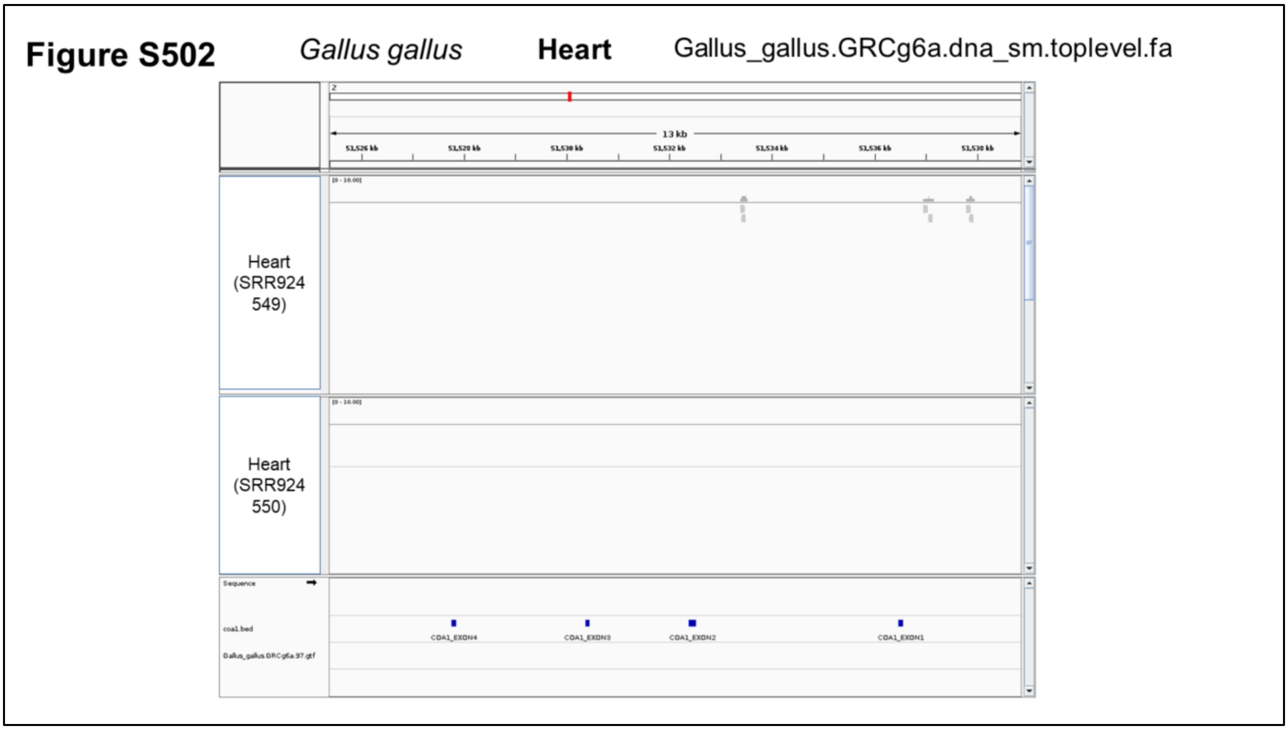

Figure S502: Screenshot of RNA-seq reads aligned to each of the putative locations of the four *COA1* exons in chicken (*Gallus gallus*). Gray boxes represent reads aligned to the genome and thin lines connecting these boxes represent the spliced read alignments. Datasets from heart (SRR924549) and (SRR924550) have been mapped to the Gallus_gallus.GRCg6a.dna_sm.toplevel.fa genome. Locations of each of the exons are depicted as bed record in the bottom row.

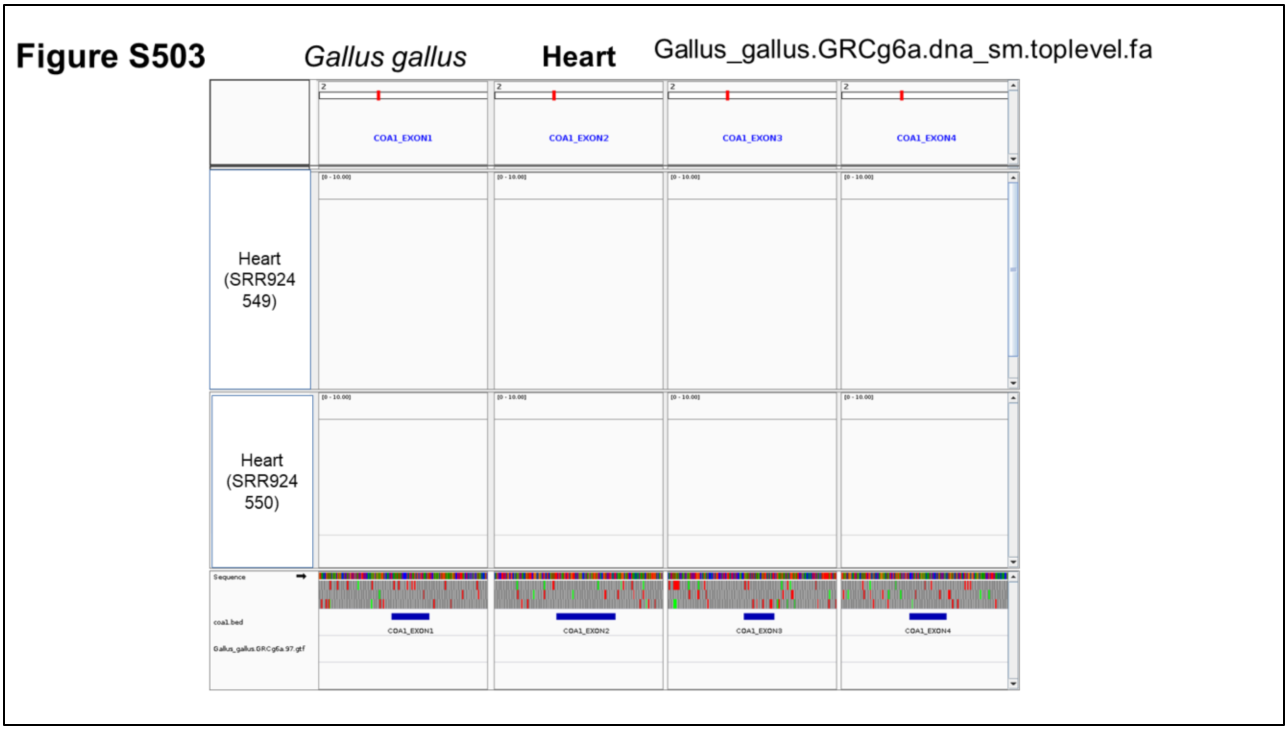

Figure S503: A zoomed in view of each of the *COA1* exons shown in **Supplementary Fig. S502**.

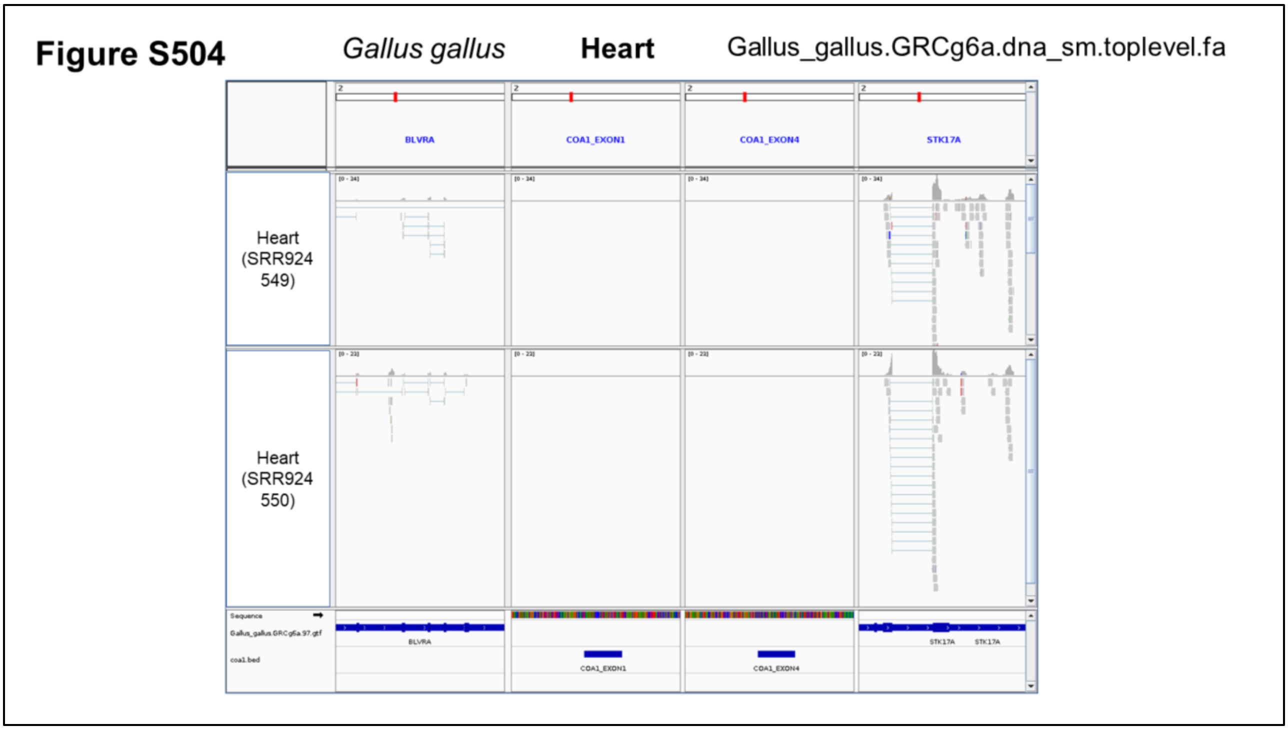

Figure S504: Screenshot of RNA-seq reads aligned to *BLVRA* and *STK17A* to *COA1* exon 1 and 4 respectively in chicken (*Gallus gallus*). Gray boxes represent reads aligned to the genome and thin lines connecting these boxes represent the spliced read alignments. Datasets from heart (SRR924549) and (SRR924550) have been mapped to the Gallus_gallus.GRCg6a.dna_sm.toplevel.fa genome. Locations of each of the exons are depicted as bed record in the bottom row.

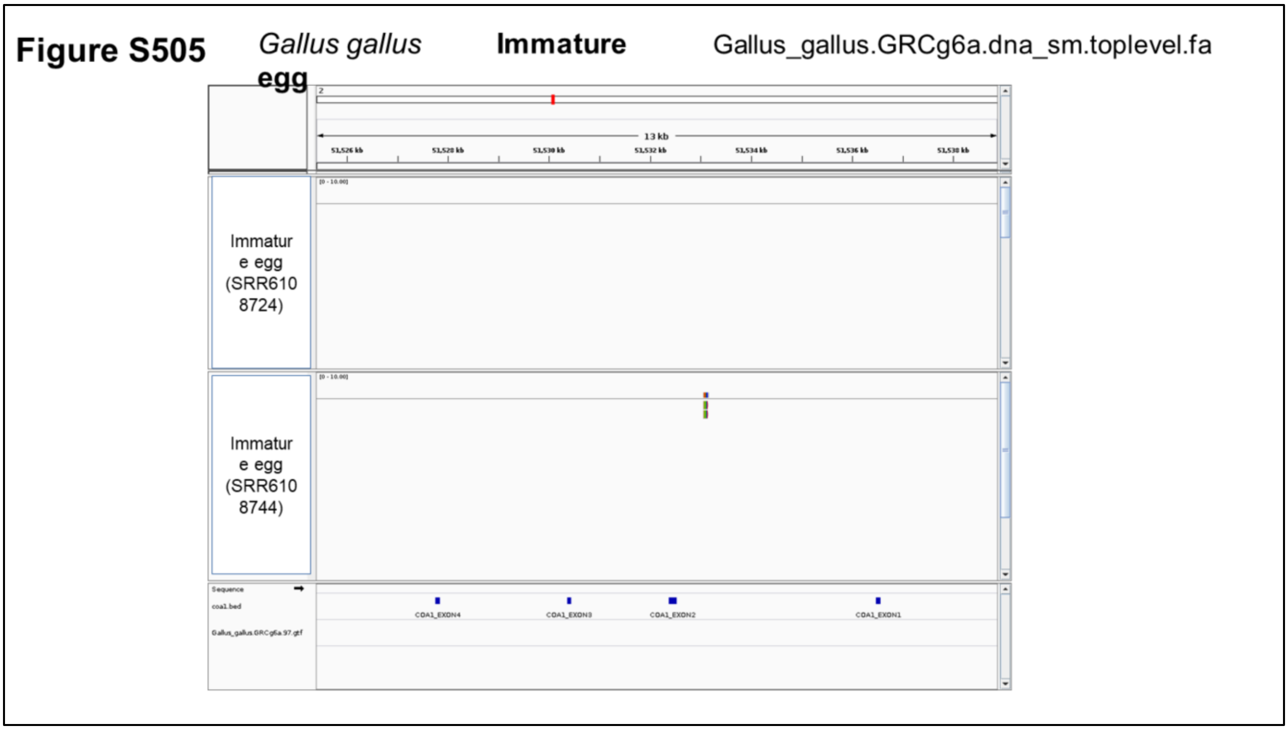

Figure S505: Screenshot of RNA-seq reads aligned to each of the putative locations of the four *COA1* exons in chicken (*Gallus gallus*). Gray boxes represent reads aligned to the genome and thin lines connecting these boxes represent the spliced read alignments. Datasets from immature egg (SRR5108724) and (SRR5108744) have been mapped to the Gallus_gallus.GRCg6a.dna_sm.toplevel.fa genome. Locations of each of the exons are depicted as bed record in the bottom row.

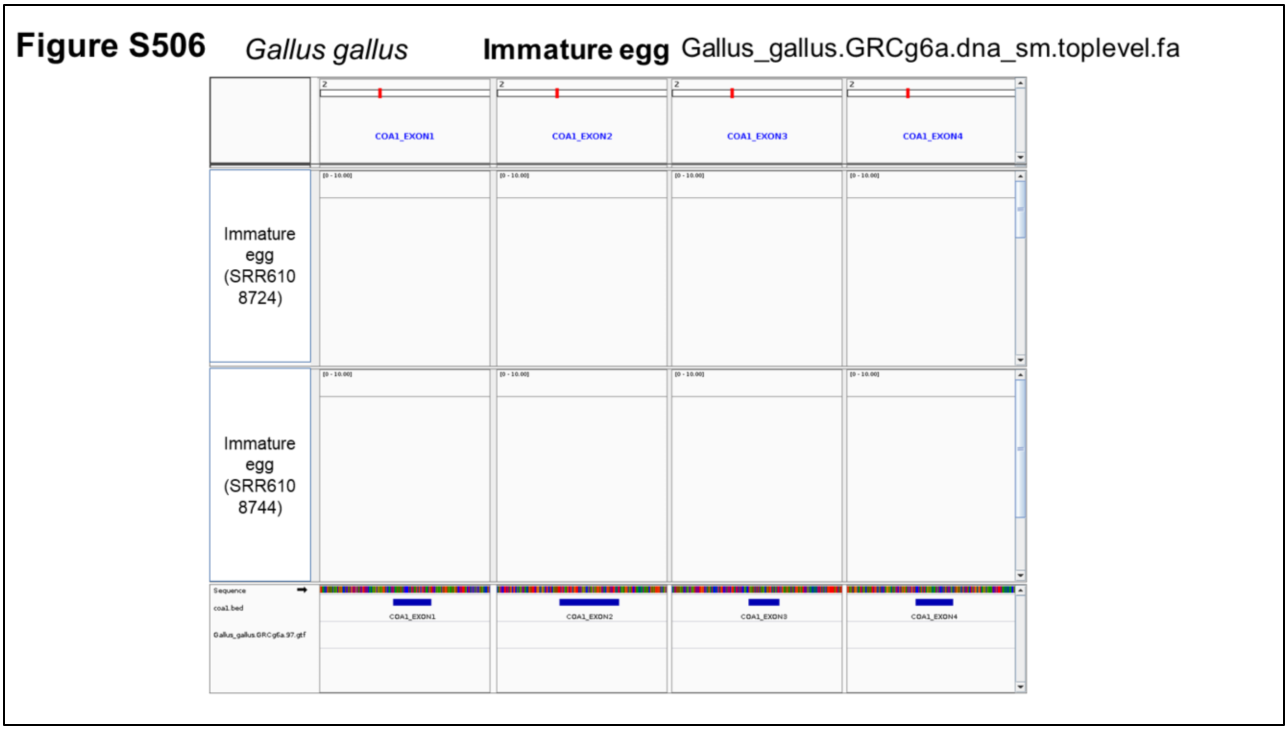

Figure S506: A zoomed in view of each of the *COA1* exons shown in shown in **Supplementary Fig. S505**.

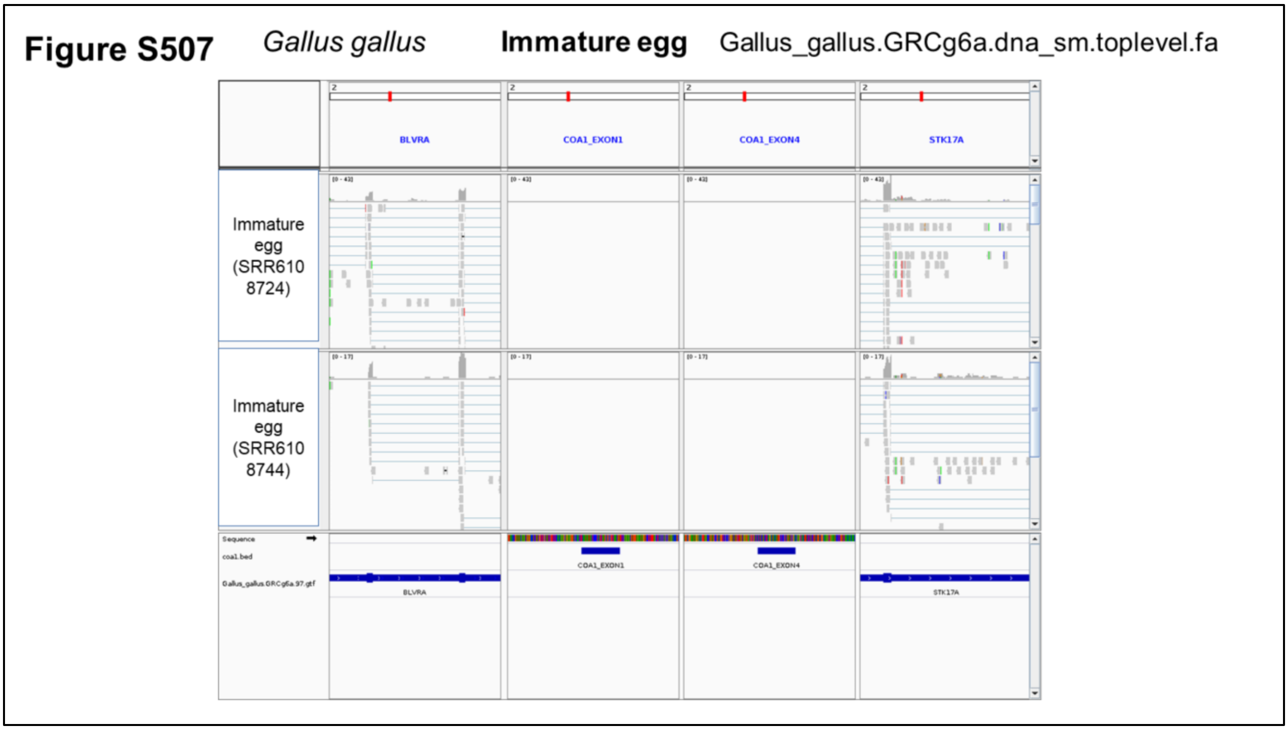

Figure S507: Screenshot of RNA-seq reads aligned to *BLVRA* and *STK17A* to *COA1* exon 1 and 4 respectively in chicken (*Gallus gallus*). Gray boxes represent reads aligned to the genome and thin lines connecting these boxes represent the spliced read alignments. Datasets from immature egg (SRR5108724) and (SRR5108744) have been mapped to the Gallus_gallus.GRCg6a.dna_sm.toplevel.fa genome. Locations of each of the exons are depicted as bed record in the bottom row.

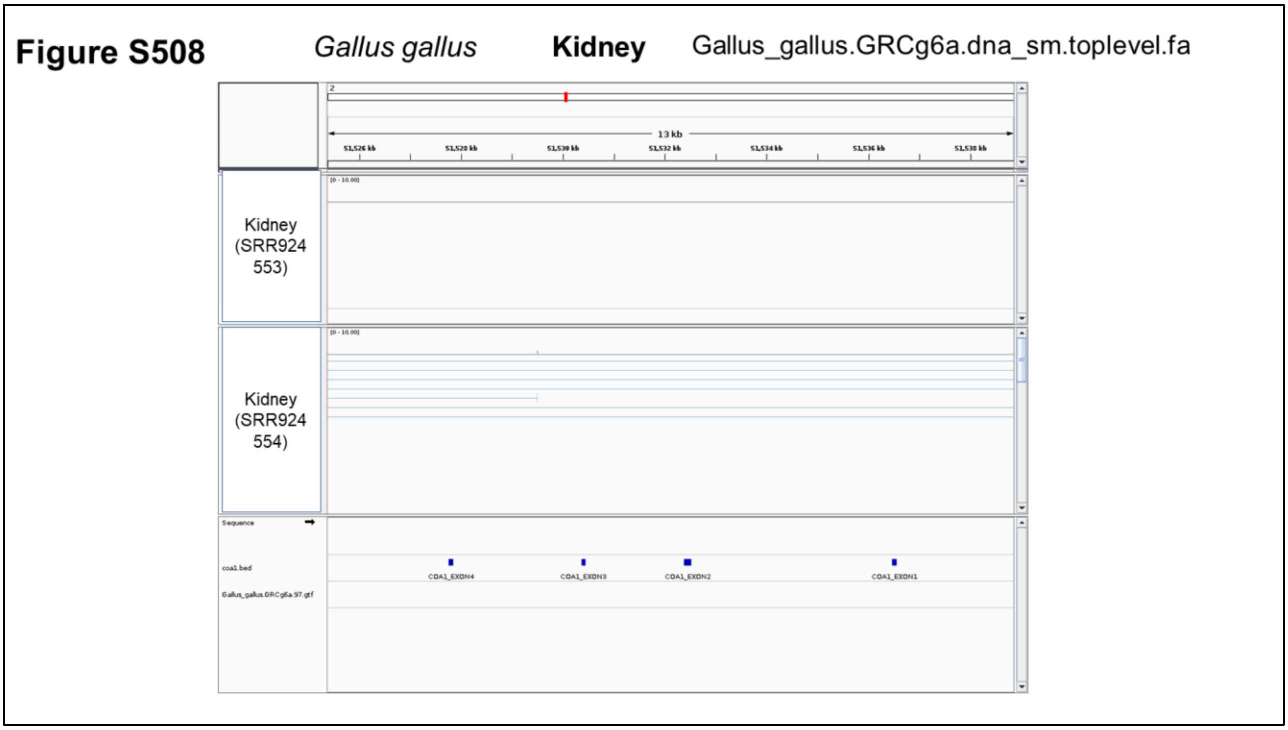

Figure S508: Screenshot of RNA-seq reads aligned to each of the putative locations of the four *COA1* exons in chicken (*Gallus gallus*). Gray boxes represent reads aligned to the genome and thin lines connecting these boxes represent the spliced read alignments. Datasets from kidney (SRR924553) and (SRR924554) have been mapped to the Gallus_gallus.GRCg6a.dna_sm.toplevel.fa genome. Locations of each of the exons are depicted as bed record in the bottom row.

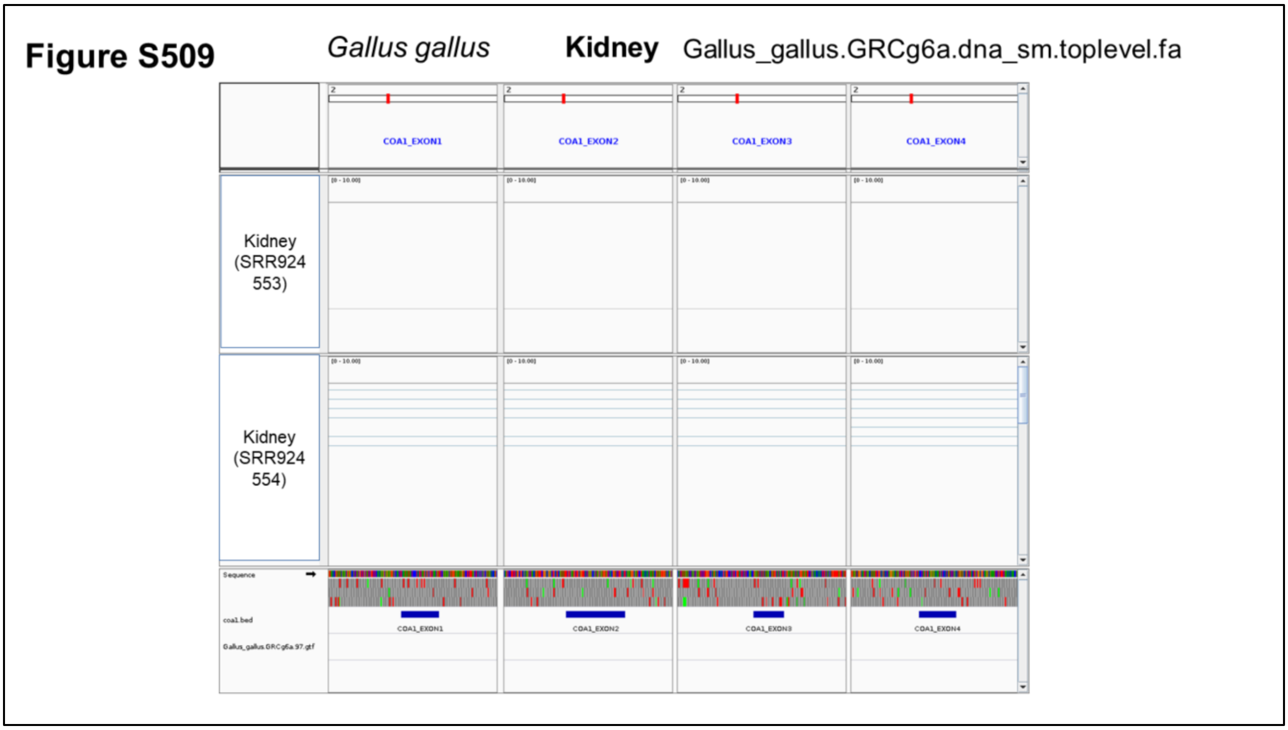

Figure S509: A zoomed in view of each of the *COA1* exons shown in **Supplementary Fig. S508**.

Figure S510 *Gallus gallus* **Kidney** Gallus_gallus.GRCg6a.dna_sm.toplevel.fa

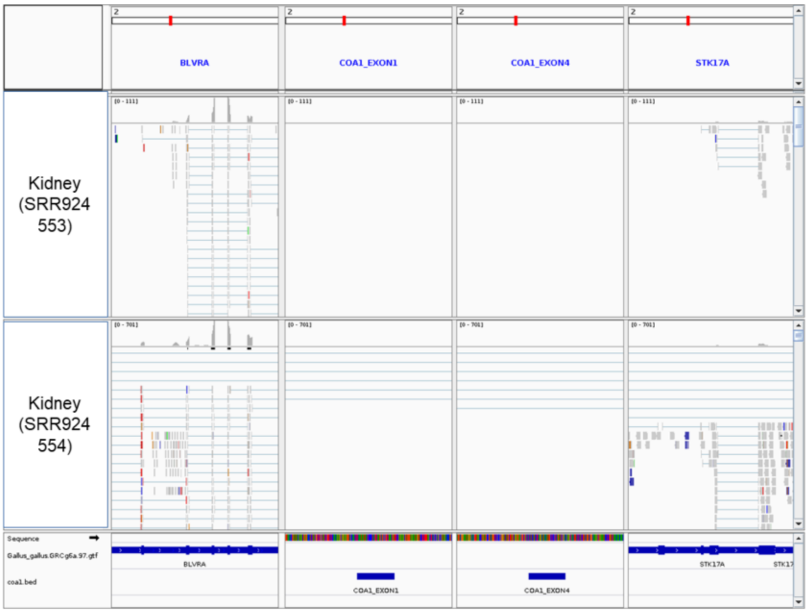

Figure S510: Screenshot of RNA-seq reads aligned to *BLVRA* and *STK17A* to *COA1* exon 1 and 4 respectively in chicken (*Gallus gallus*). Gray boxes represent reads aligned to the genome and thin lines connecting these boxes represent the spliced read alignments. Datasets from kidney (SRR924553) and (SRR924554) have been mapped to the Gallus_gallus.GRCg6a.dna_sm.toplevel.fa genome. Locations of each of the exons are depicted as bed record in the bottom row.

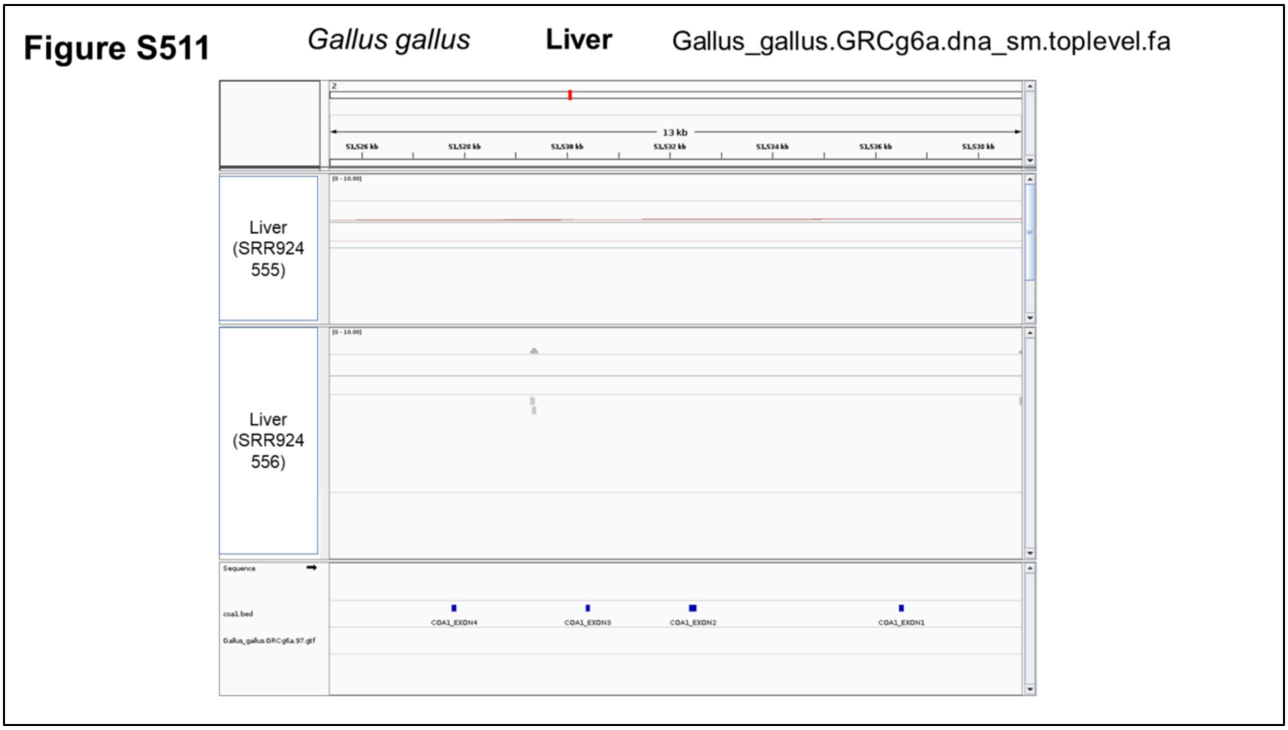

Figure S511: Screenshot of RNA-seq reads aligned to each of the putative locations of the four *COA1* exons in chicken (*Gallus gallus*). Gray boxes represent reads aligned to the genome and thin lines connecting these boxes represent the spliced read alignments. Datasets from liver (SRR924555) and (SRR924556) have been mapped to the Gallus_gallus.GRCg6a.dna_sm.toplevel.fa genome. Locations of each of the exons are depicted as bed record in the bottom row.

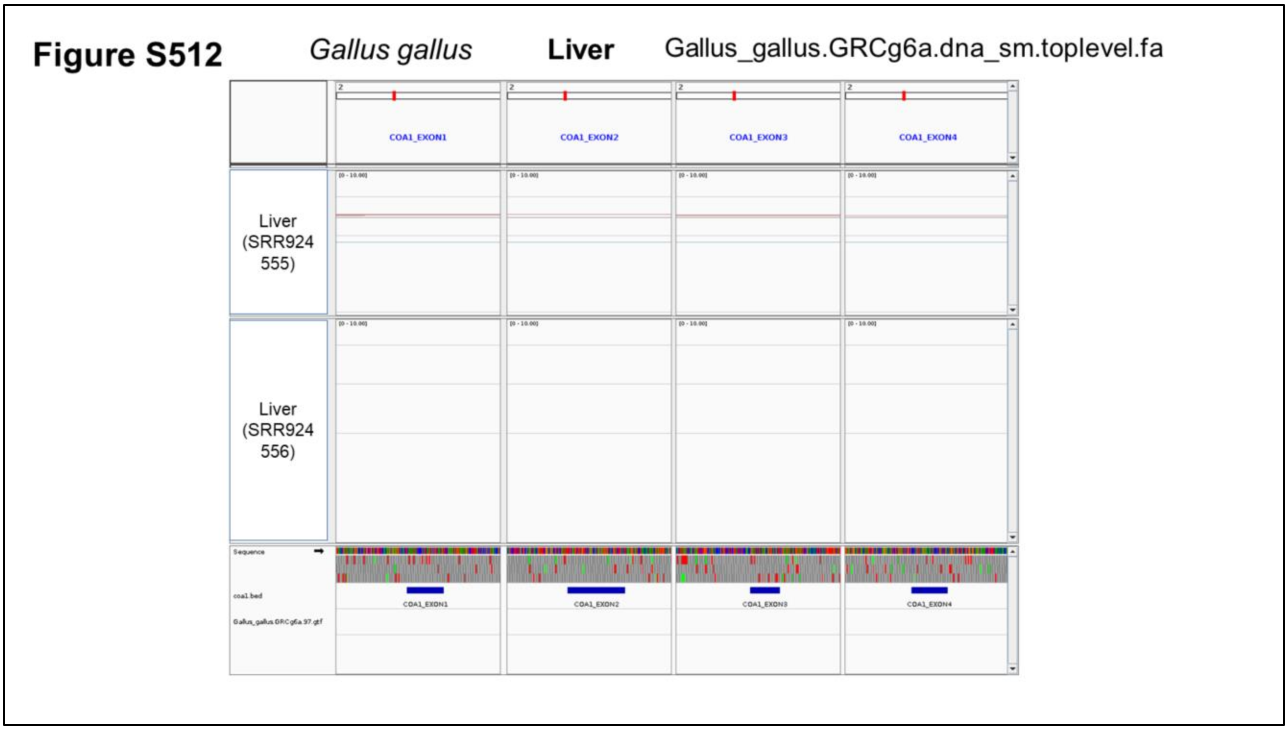

Figure S512: A zoomed in view of each of the *COA1* exons shown in **Supplementary Fig. S511**.

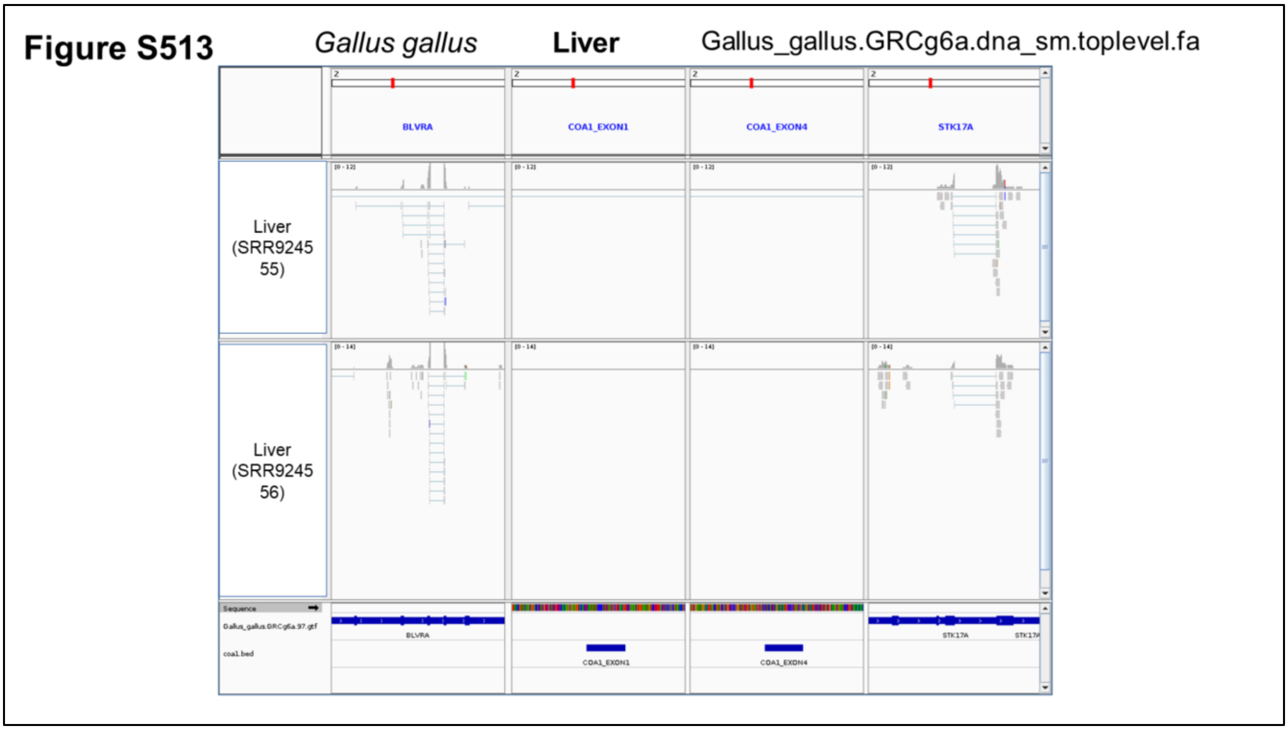

Figure S513: Screenshot of RNA-seq reads aligned to *BLVRA* and *STK17A* to *COA1* exon 1 and 4 respectively in chicken (*Gallus gallus*). Gray boxes represent reads aligned to the genome and thin lines connecting these boxes represent the spliced read alignments. Datasets from liver (SRR924555) and (SRR924556) have been mapped to the Gallus_gallus.GRCg6a.dna_sm.toplevel.fa genome. Locations of each of the exons are depicted as bed record in the bottom row.

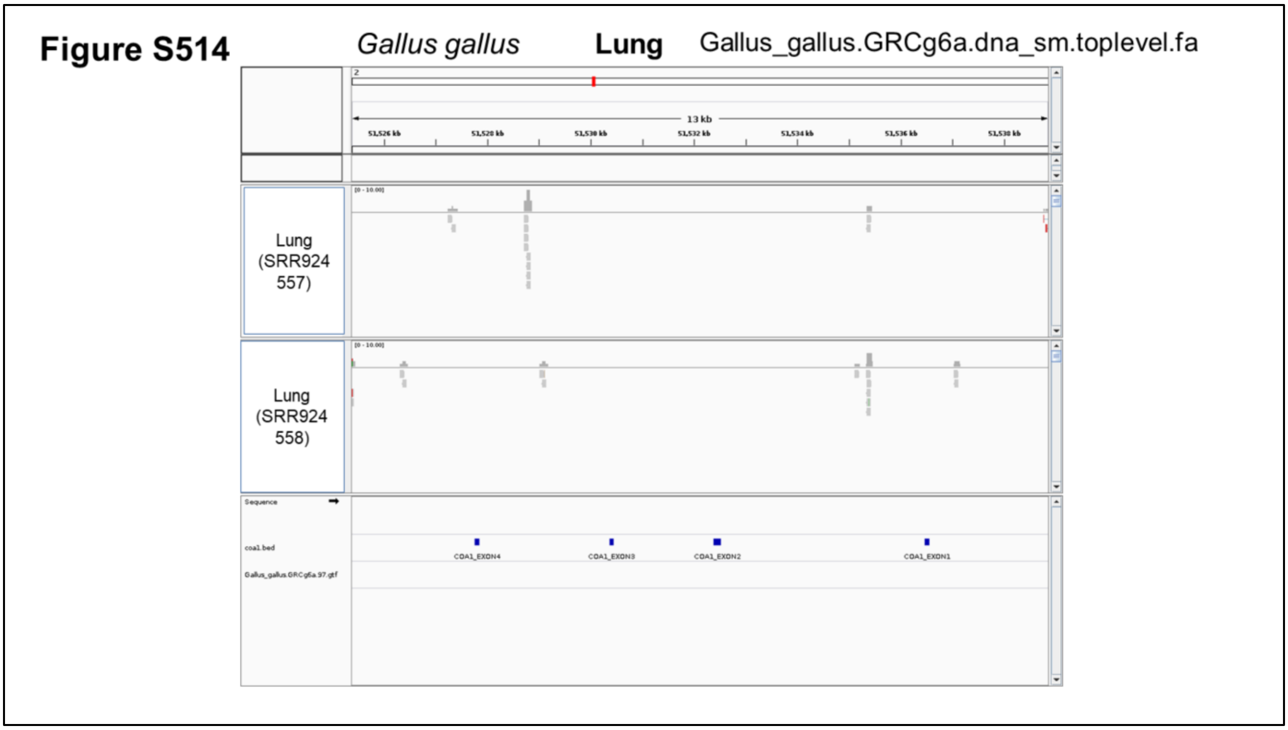

Figure S514: Screenshot of RNA-seq reads aligned to each of the putative locations of the four *COA1* exons in chicken (*Gallus gallus*). Gray boxes represent reads aligned to the genome and thin lines connecting these boxes represent the spliced read alignments. Datasets from lung (SRR924557) and (SRR924558) have been mapped to the Gallus_gallus.GRCg6a.dna_sm.toplevel.fa genome. Locations of each of the exons are depicted as bed record in the bottom row.

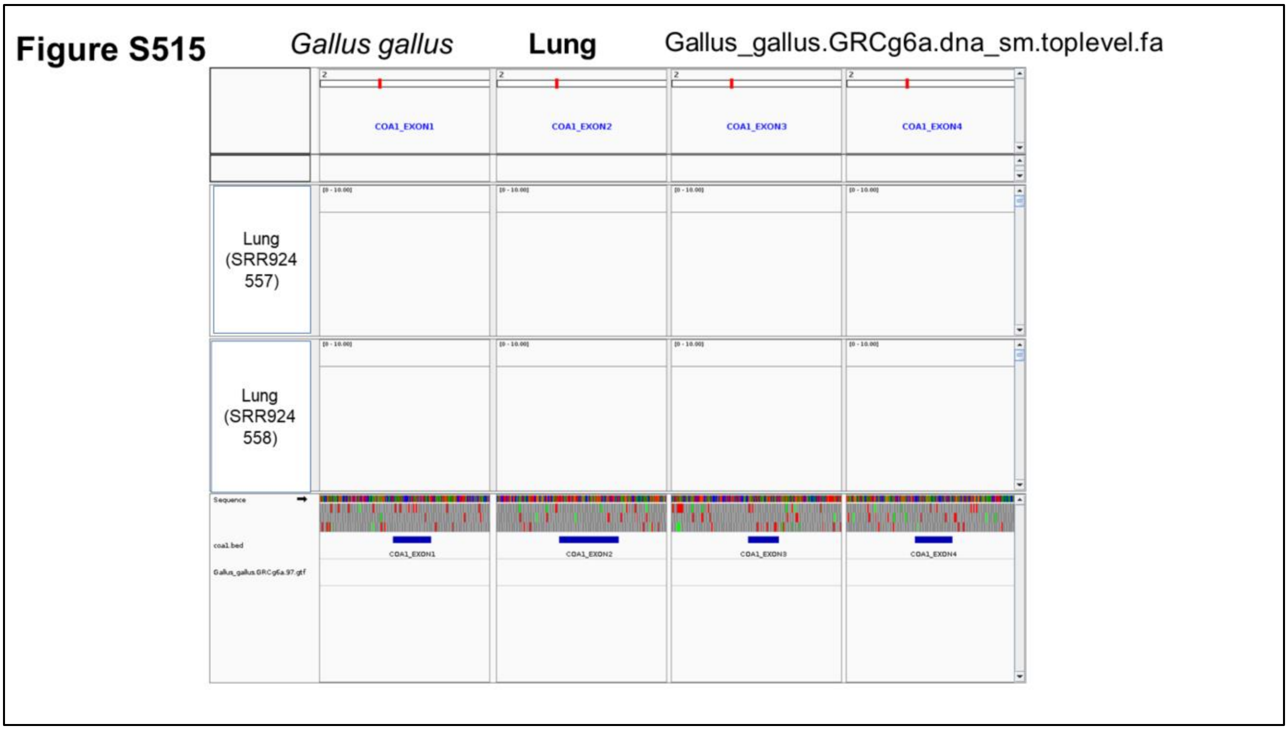

Figure S515: A zoomed in view of each of the *COA1* exons shown in **Supplementary Fig. S514**.

Figure S516 *Gallus gallus* **Lung** Gallus_gallus.GRCg6a.dna_sm.toplevel.fa

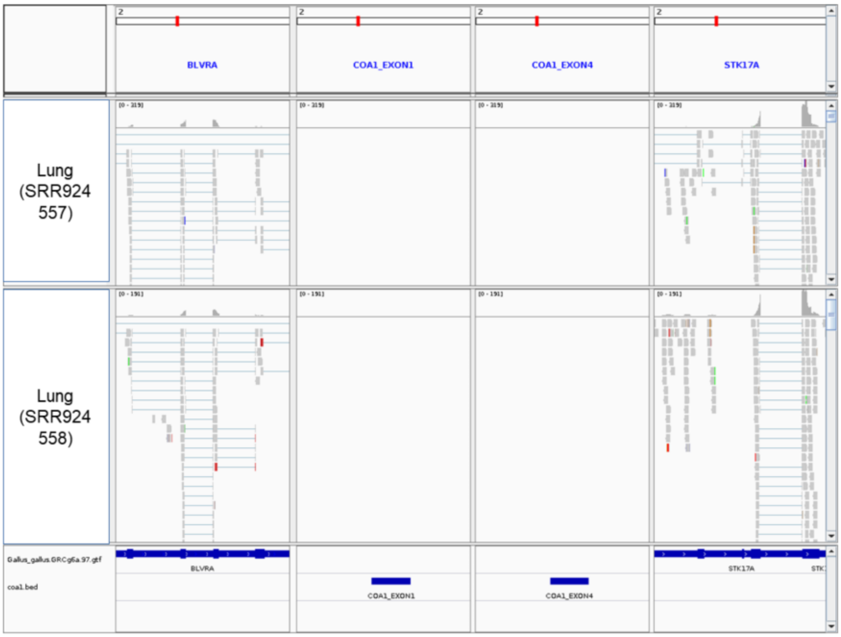

Figure S516: Screenshot of RNA-seq reads aligned to *BLVRA* and *STK17A* to *COA1* exon 1 and 4 respectively in chicken (*Gallus gallus*). Gray boxes represent reads aligned to the genome and thin lines connecting these boxes represent the spliced read alignments. Datasets from lung (SRR924557) and (SRR924558) have been mapped to the Gallus_gallus.GRCg6a.dna_sm.toplevel.fa genome. Locations of each of the exons are depicted as bed record in the bottom row.

Figure S517 *Gallus gallus* **Mature egg** Gallus_gallus.GRCg6a.dna_sm.toplevel.fa

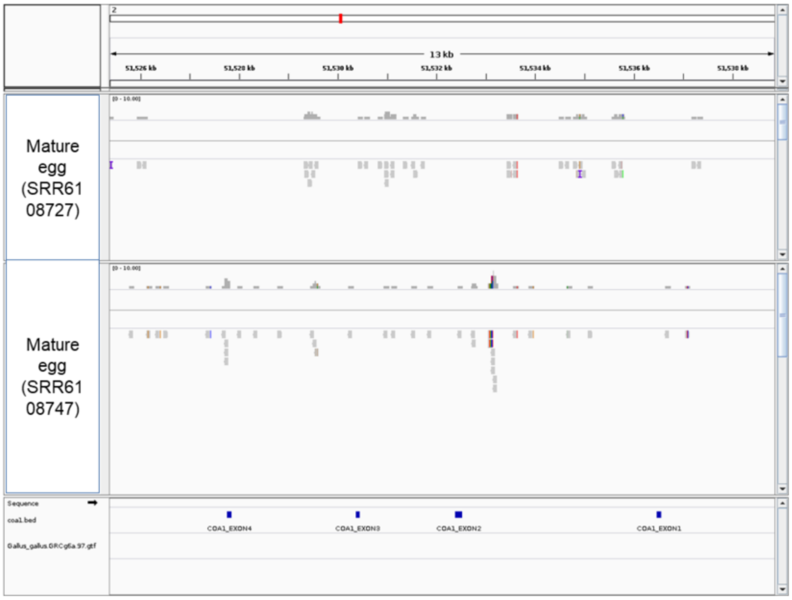

Figure S517: Screenshot of RNA-seq reads aligned to each of the putative locations of the four *COA1* exons in chicken (*Gallus gallus*). Gray boxes represent reads aligned to the genome and thin lines connecting these boxes represent the spliced read alignments. Datasets from mature egg (SRR6108727) and (SRR6108747) have been mapped to the Gallus_gallus.GRCg6a.dna_sm.toplevel.fa genome. Locations of each of the exons are depicted as bed record in the bottom row.

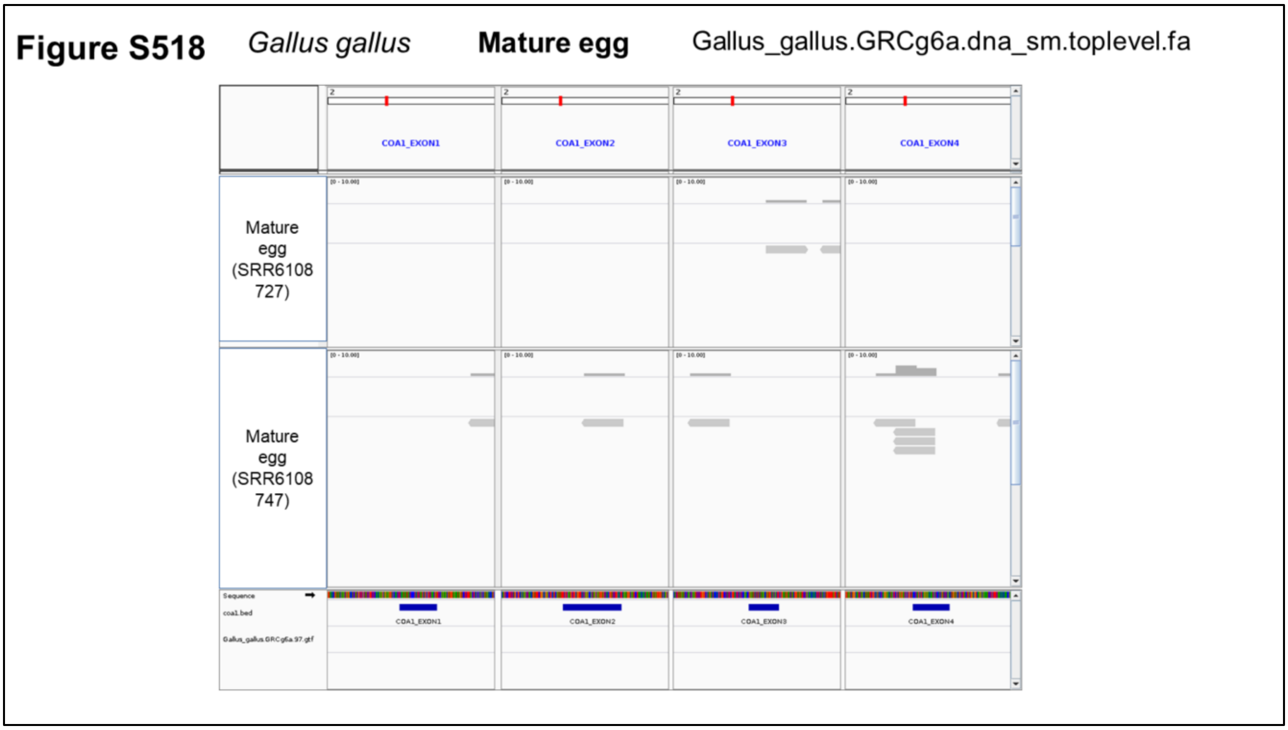

Figure S518: A zoomed in view of each of the *COA1* exons shown in **Supplementary Fig. S517**.

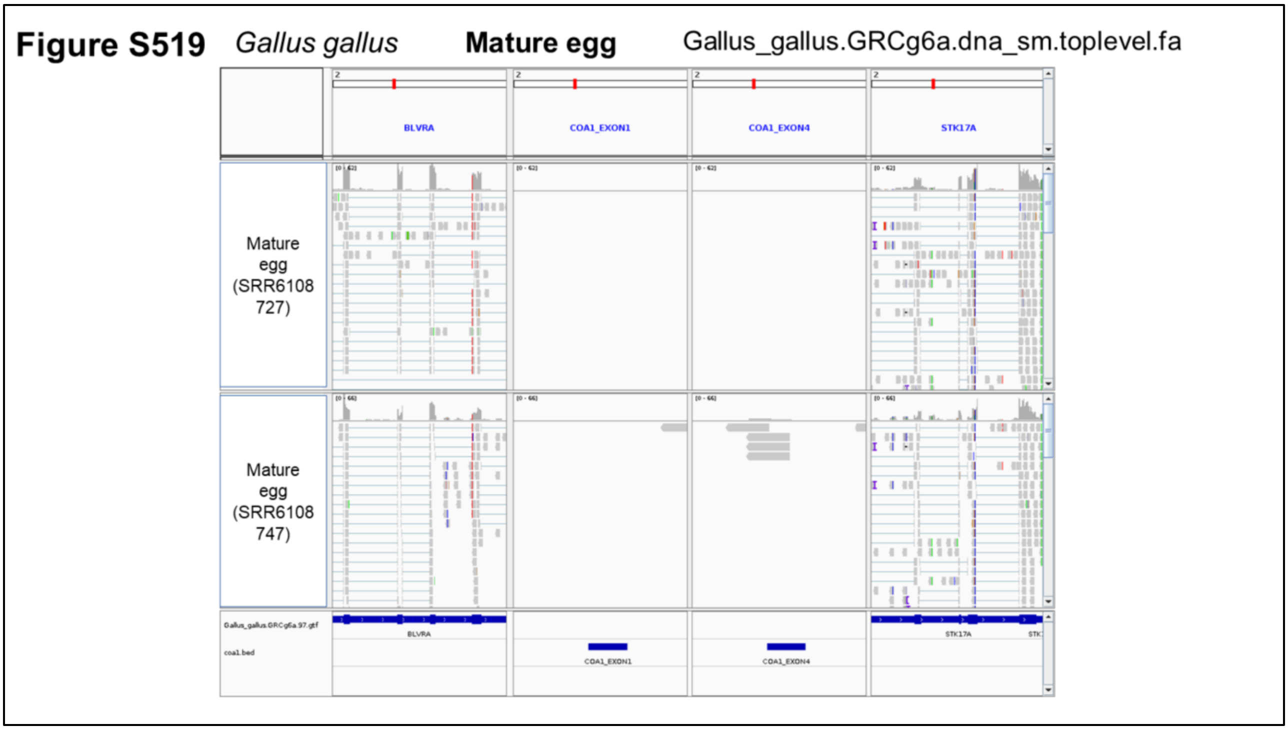

Figure S519: Screenshot of RNA-seq reads aligned to *BLVRA* and *STK17A* to *COA1* exon 1 and 4 respectively in chicken (*Gallus gallus*). Gray boxes represent reads aligned to the genome and thin lines connecting these boxes represent the spliced read alignments. Datasets from mature egg (SRR6108727) and (SRR6108747) have been mapped to the Gallus_gallus.GRCg6a.dna_sm.toplevel.fa genome. Locations of each of the exons are depicted as bed record in the bottom row.

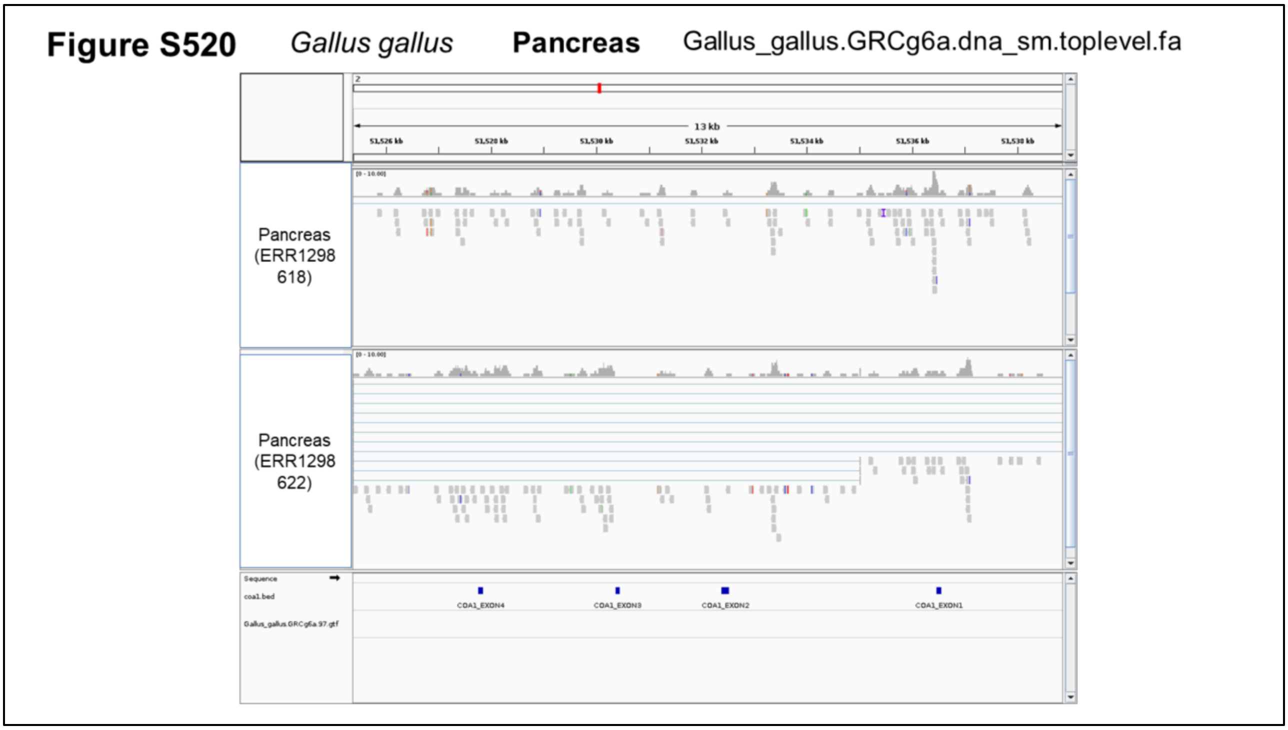

Figure S520: Screenshot of RNA-seq reads aligned to each of the putative locations of the four *COA1* exons in chicken (*Gallus gallus*). Gray boxes represent reads aligned to the genome and thin lines connecting these boxes represent the spliced read alignments. Datasets from pancreas (ERR1298618) and (ERR1298622) have been mapped to the Gallus_gallus.GRCg6a.dna_sm.toplevel.fa genome. Locations of each of the exons are depicted as bed record in the bottom row.

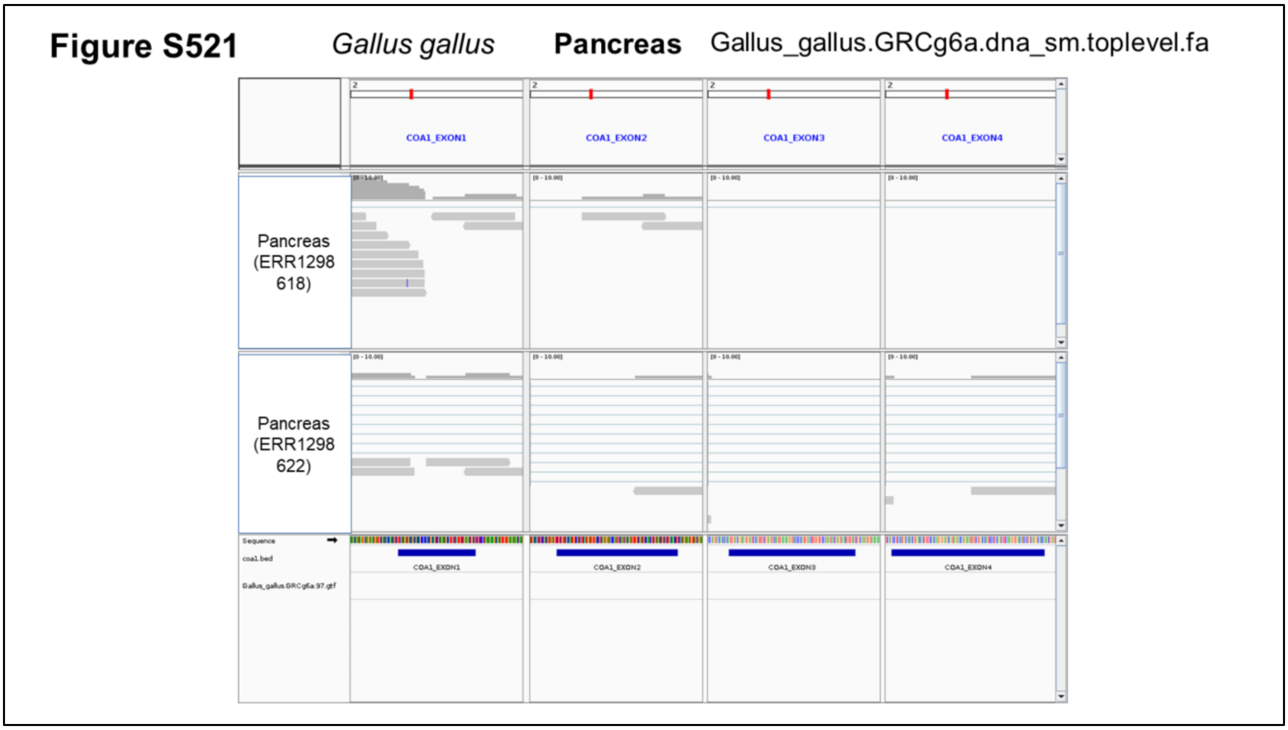

Figure S521: A zoomed in view of each of the *COA1* exons shown in **Supplementary Fig. S520**.

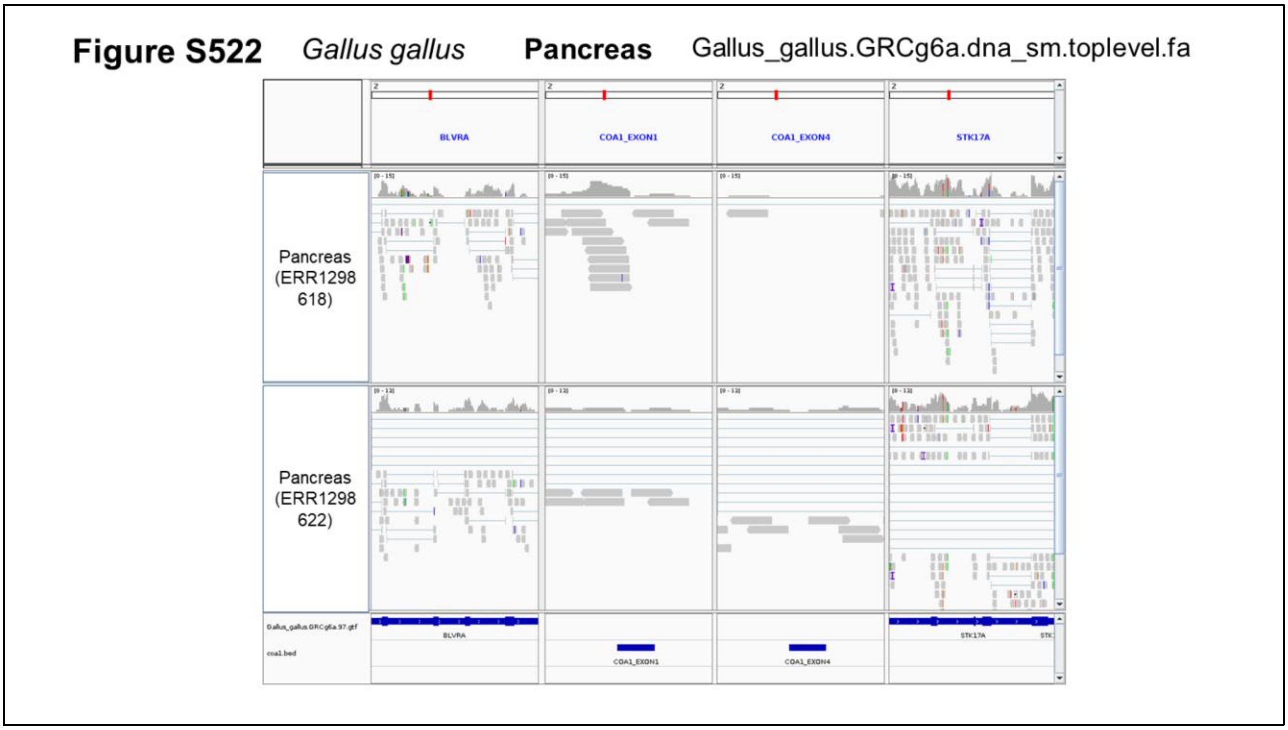

Figure S522: Screenshot of RNA-seq reads aligned to *BLVRA* and *STK17A* to *COA1* exon 1 and 4 respectively in chicken (*Gallus gallus*). Gray boxes represent reads aligned to the genome and thin lines connecting these boxes represent the spliced read alignments. Datasets from pancreas (ERR1298618) and (ERR1298622) have been mapped to the Gallus_gallus.GRCg6a.dna_sm.toplevel.fa genome. Locations of each of the exons are depicted as bed record in the bottom row.

Figure S523 *Gallus gallus* **Shank** Gallus_gallus.GRCg6a.dna_sm.toplevel.fa

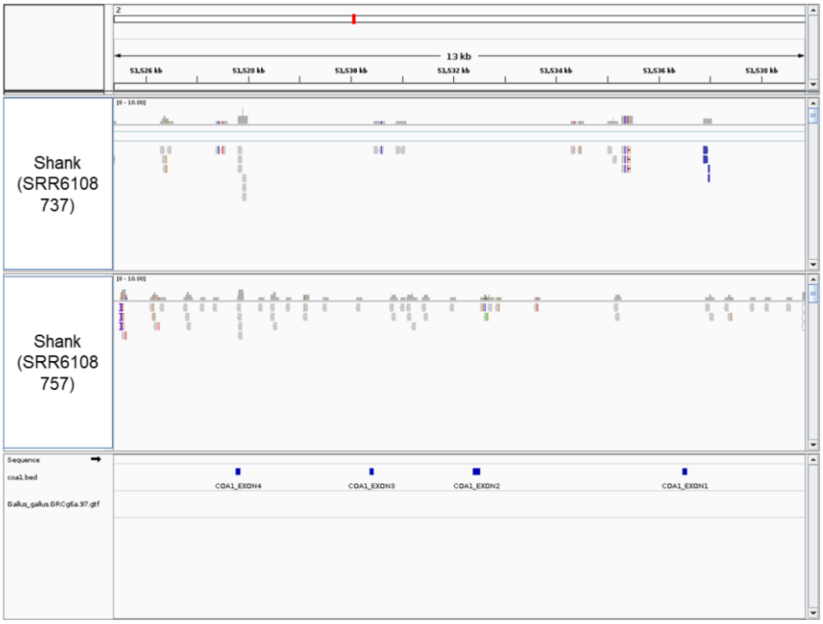

Figure S523: Screenshot of RNA-seq reads aligned to each of the putative locations of the four *COA1* exons in chicken (*Gallus gallus*). Gray boxes represent reads aligned to the genome and thin lines connecting these boxes represent the spliced read alignments. Datasets from shank (SRR6108737) and (SRR6108757) have been mapped to the Gallus_gallus.GRCg6a.dna_sm.toplevel.fa genome. Locations of each of the exons are depicted as bed record in the bottom row.

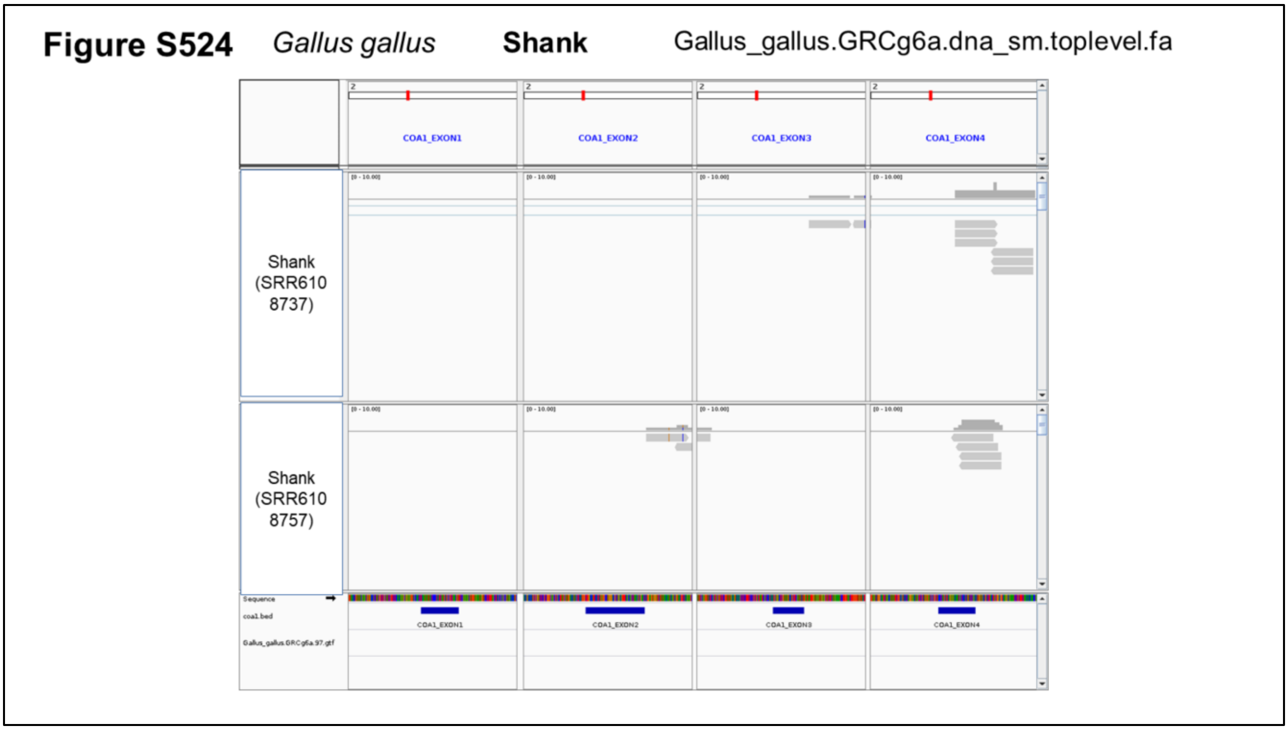

Figure S524: A zoomed in view of each of the *COA1* exons shown in **Supplementary Fig. S523**

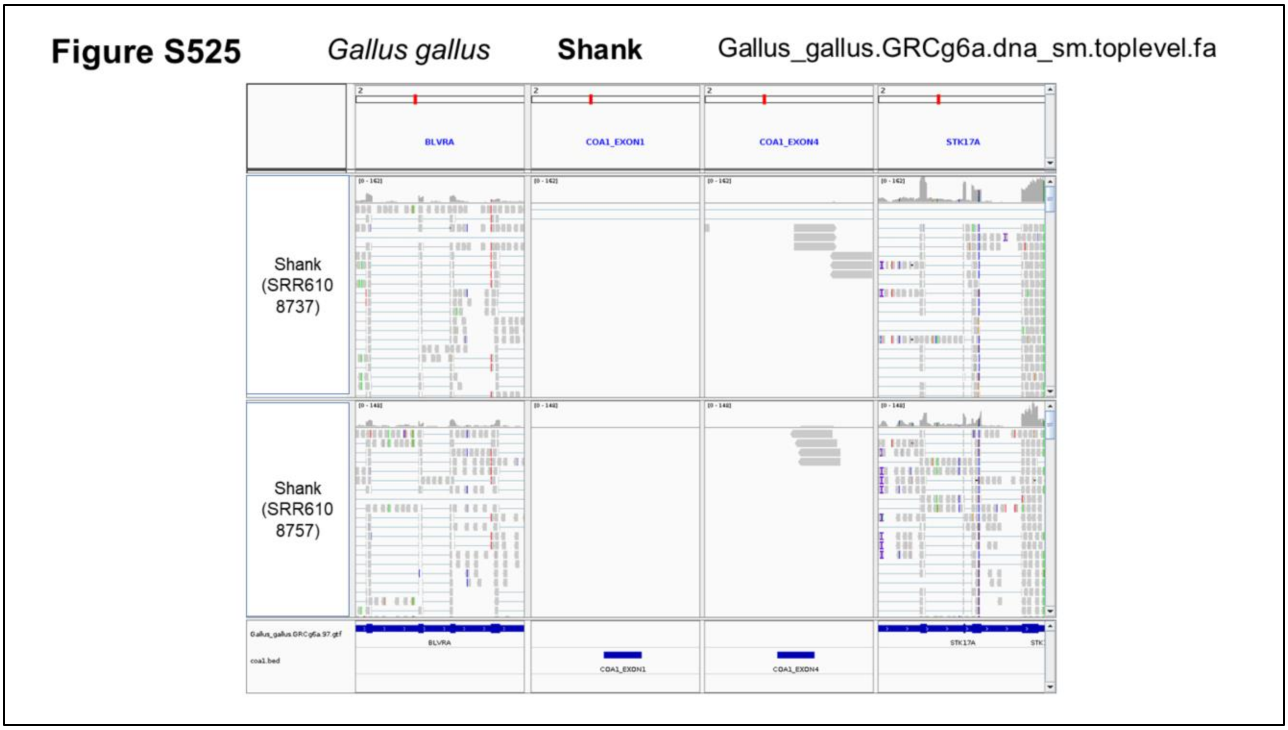

Figure S525: Screenshot of RNA-seq reads aligned to *BLVRA* and *STK17A* to *COA1* exon 1 and 4 respectively in chicken (*Gallus gallus*). Gray boxes represent reads aligned to the genome and thin lines connecting these boxes represent the spliced read alignments. Datasets from shank (SRR6108737) and (SRR6108757) have been mapped to the Gallus_gallus.GRCg6a.dna_sm.toplevel.fa genome. Locations of each of the exons are depicted as bed record in the bottom row.

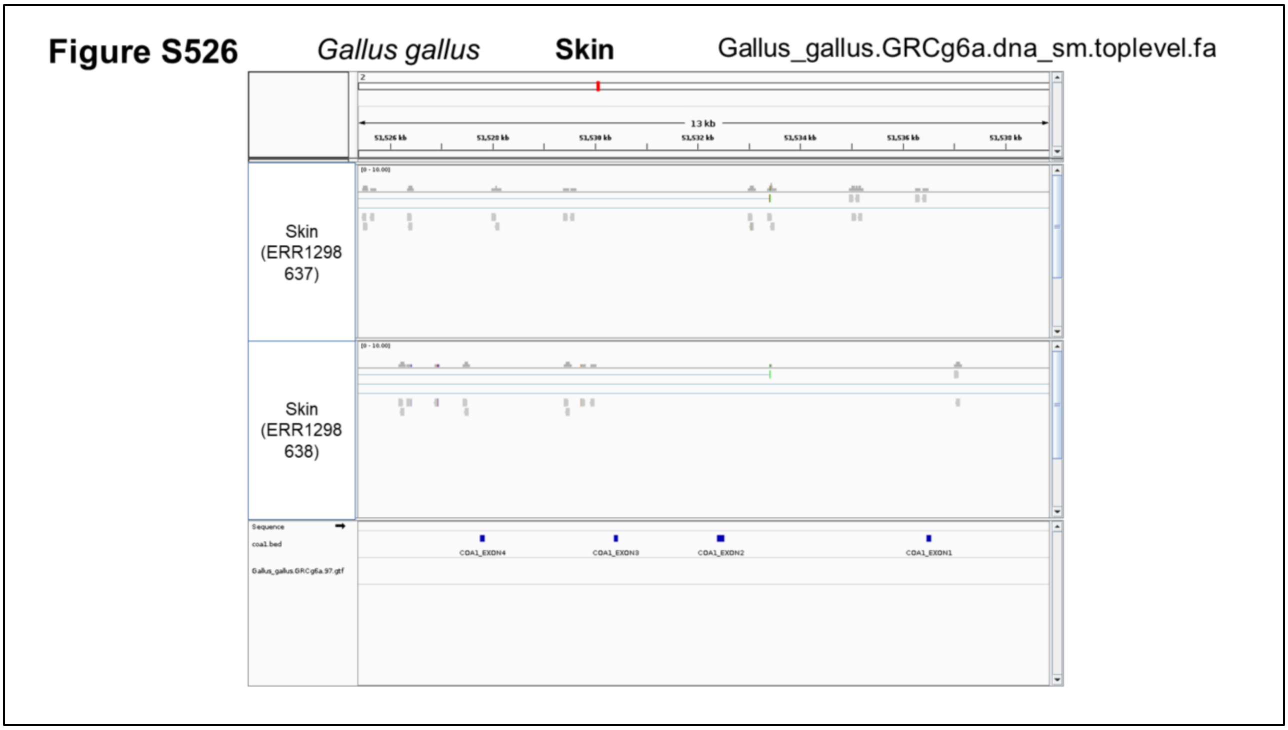

Figure S526: Screenshot of RNA-seq reads aligned to each of the putative locations of the four *COA1* exons in chicken (*Gallus gallus*). Gray boxes represent reads aligned to the genome and thin lines connecting these boxes represent the spliced read alignments. Datasets from skin (ERR1298637) and (ERR1298638) have been mapped to the Gallus_gallus.GRCg6a.dna_sm.toplevel.fa genome. Locations of each of the exons are depicted as bed record in the bottom row.

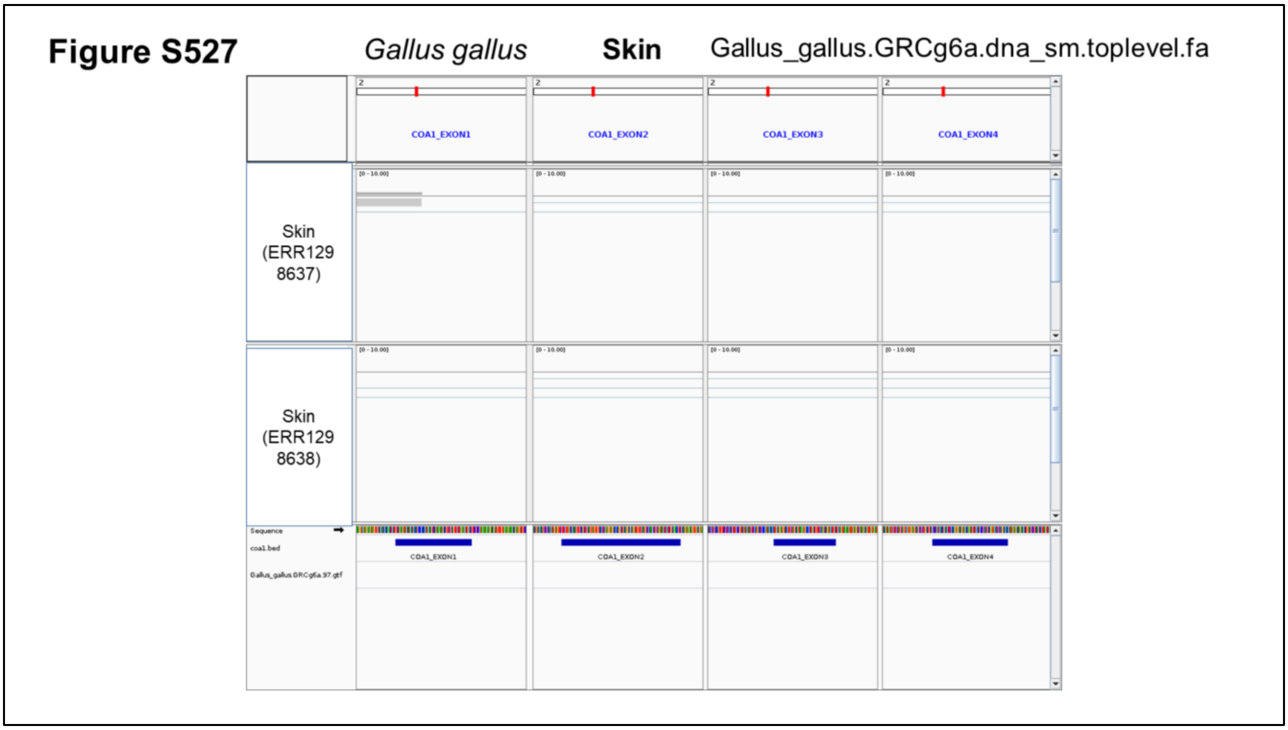

Figure S527: A zoomed in view of each of the *COA1* exons shown in **Supplementary Fig. S526**.

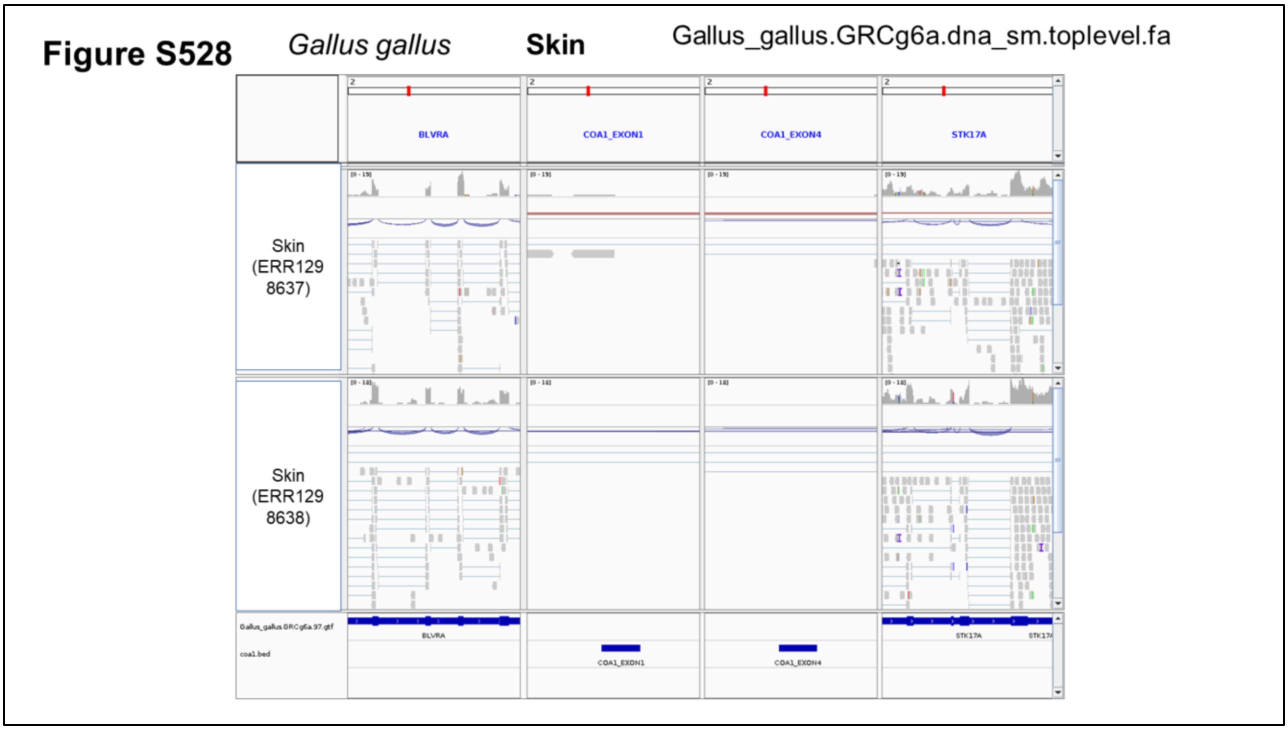

Figure S528: Screenshot of RNA-seq reads aligned to *BLVRA* and *STK17A* to *COA1* exon 1 and 4 respectively in chicken (*Gallus gallus*). Gray boxes represent reads aligned to the genome and thin lines connecting these boxes represent the spliced read alignments. Datasets from skin (ERR1298637) and (ERR1298638) have been mapped to the Gallus_gallus.GRCg6a.dna_sm.toplevel.fa genome. Locations of each of the exons are depicted as bed record in the bottom row.

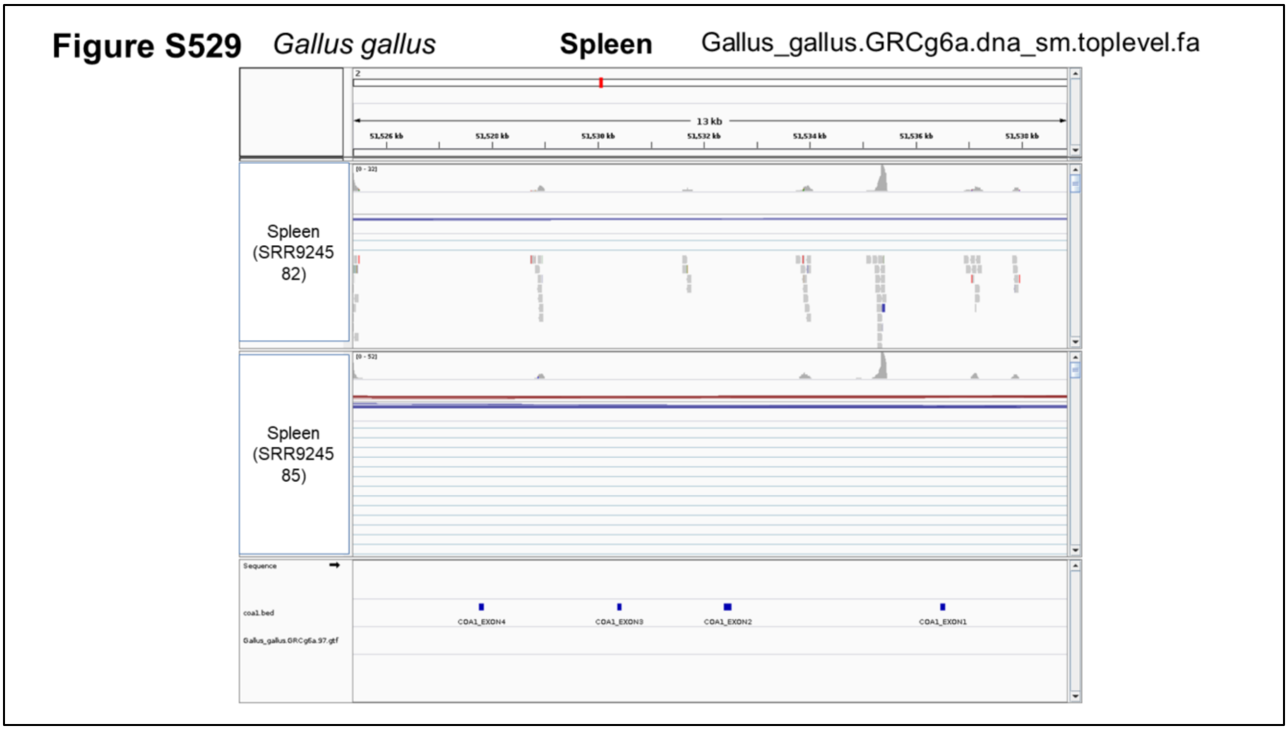

Figure S529: Screenshot of RNA-seq reads aligned to each of the putative locations of the four *COA1* exons in chicken (*Gallus gallus*). Gray boxes represent reads aligned to the genome and thin lines connecting these boxes represent the spliced read alignments. Datasets from spleen (SRR924582) and (SRR924585) have been mapped to the Gallus_gallus.GRCg6a.dna_sm.toplevel.fa genome. Locations of each of the exons are depicted as bed record in the bottom row.

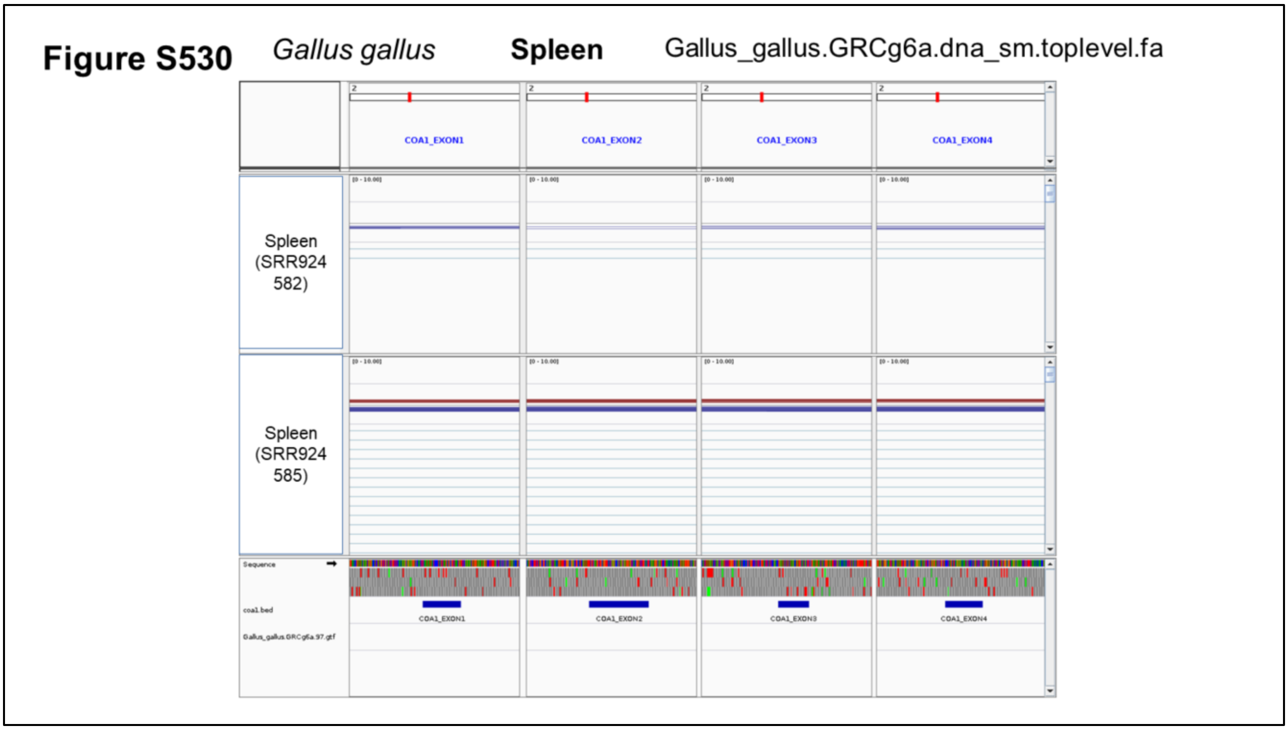

Figure S530: A zoomed in view of each of the *COA1* exons shown in **Supplementary Fig. S529**.

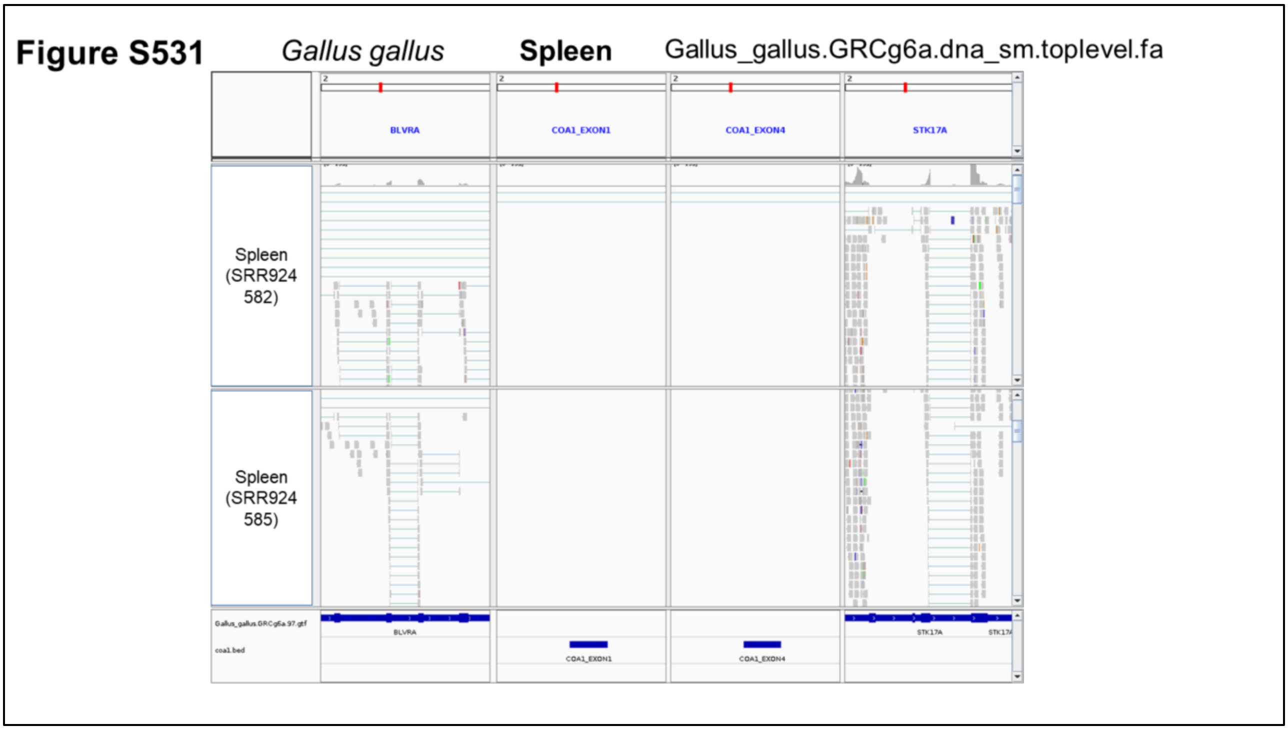

Figure S531: Screenshot of RNA-seq reads aligned to *BLVRA* and *STK17A* to *COA1* exon 1 and 4 respectively in chicken (*Gallus gallus*). Gray boxes represent reads aligned to the genome and thin lines connecting these boxes represent the spliced read alignments. Datasets from spleen (SRR924582) and (SRR924585) have been mapped to the Gallus_gallus.GRCg6a.dna_sm.toplevel.fa genome. Locations of each of the exons are depicted as bed record in the bottom row.

Figure S532 Gallus gallus **Uterus** Gallus_gallus.GRCg6a.dna_sm.toplevel.fa

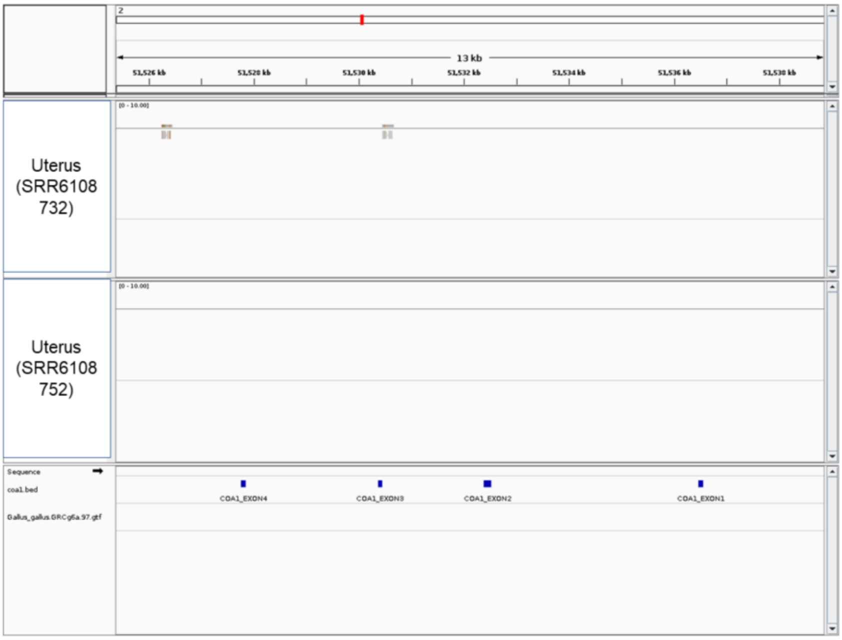

Figure S532: Screenshot of RNA-seq reads aligned to each of the putative locations of the four *COA1* exons in chicken (*Gallus gallus*). Gray boxes represent reads aligned to the genome and thin lines connecting these boxes represent the spliced read alignments. Datasets from uterus (SRR6108732) and (SRR6108752) have been mapped to the Gallus_gallus.GRCg6a.dna_sm.toplevel.fa genome. Locations of each of the exons are depicted as bed record in the bottom row.

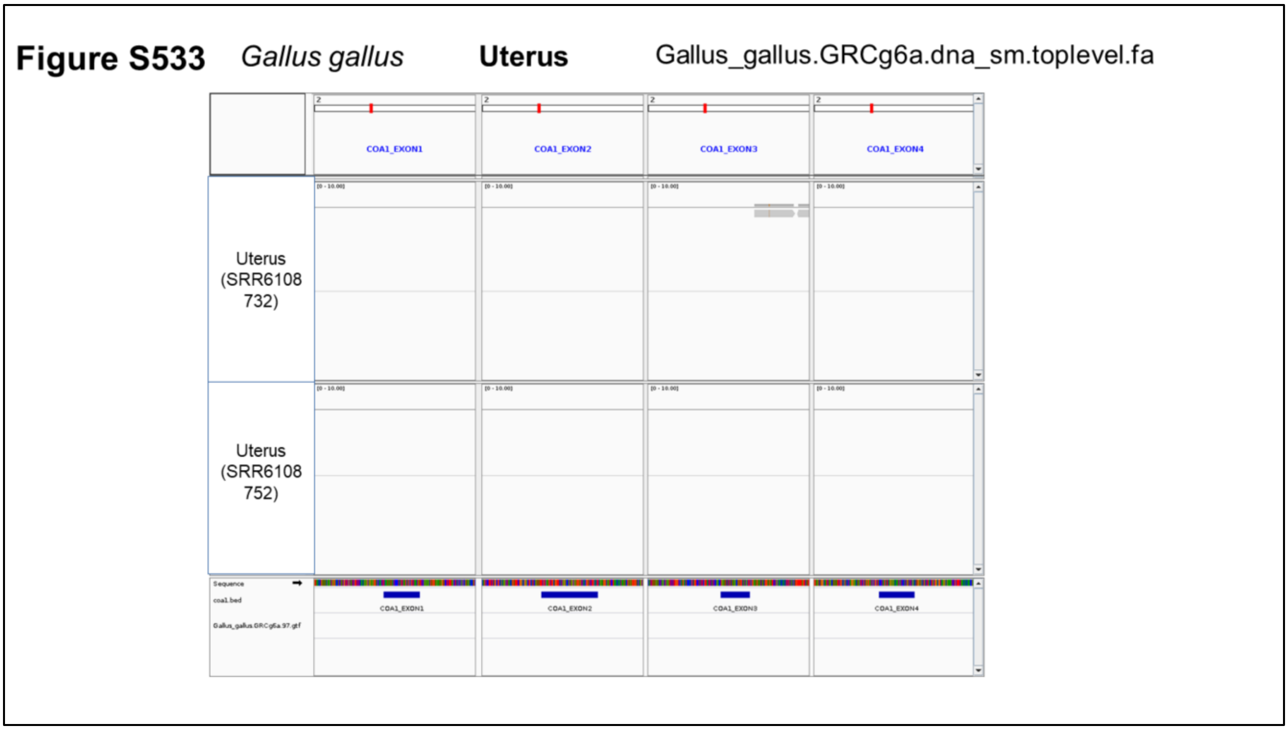

Figure S533: A zoomed in view of each of the *COA1* exons shown in **Supplementary Fig. S532**.

Figure S534 *Gallus gallus* **Uterus** Gallus_gallus.GRCg6a.dna_sm.toplevel.fa

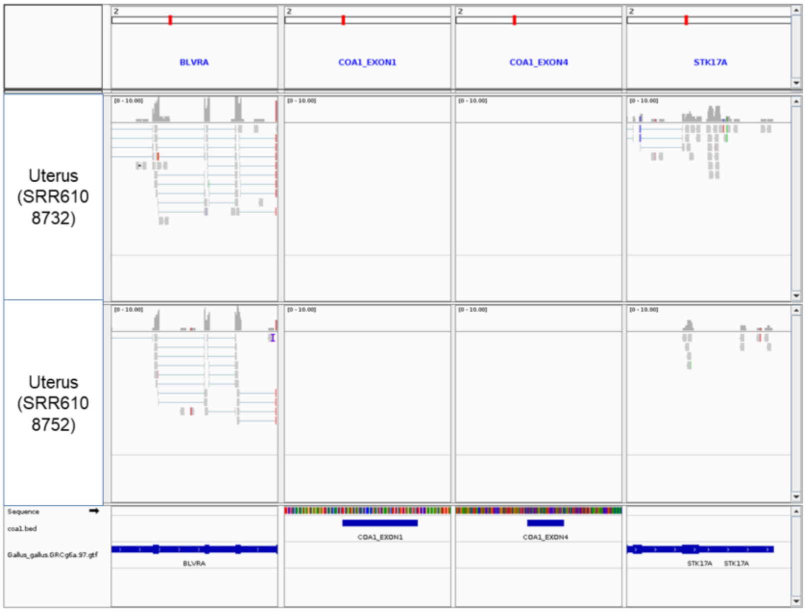

Figure S534: Screenshot of RNA-seq reads aligned to *BLVRA* and *STK17A* to *COA1* exon 1 and 4 respectively in chicken (*Gallus gallus*). Gray boxes represent reads aligned to the genome and thin lines connecting these boxes represent the spliced read alignments. Datasets from uterus (SRR6108732) and (SRR6108752) have been mapped to the Gallus_gallus.GRCg6a.dna_sm.toplevel.fa genome. Locations of each of the exons are depicted as bed record in the bottom row.

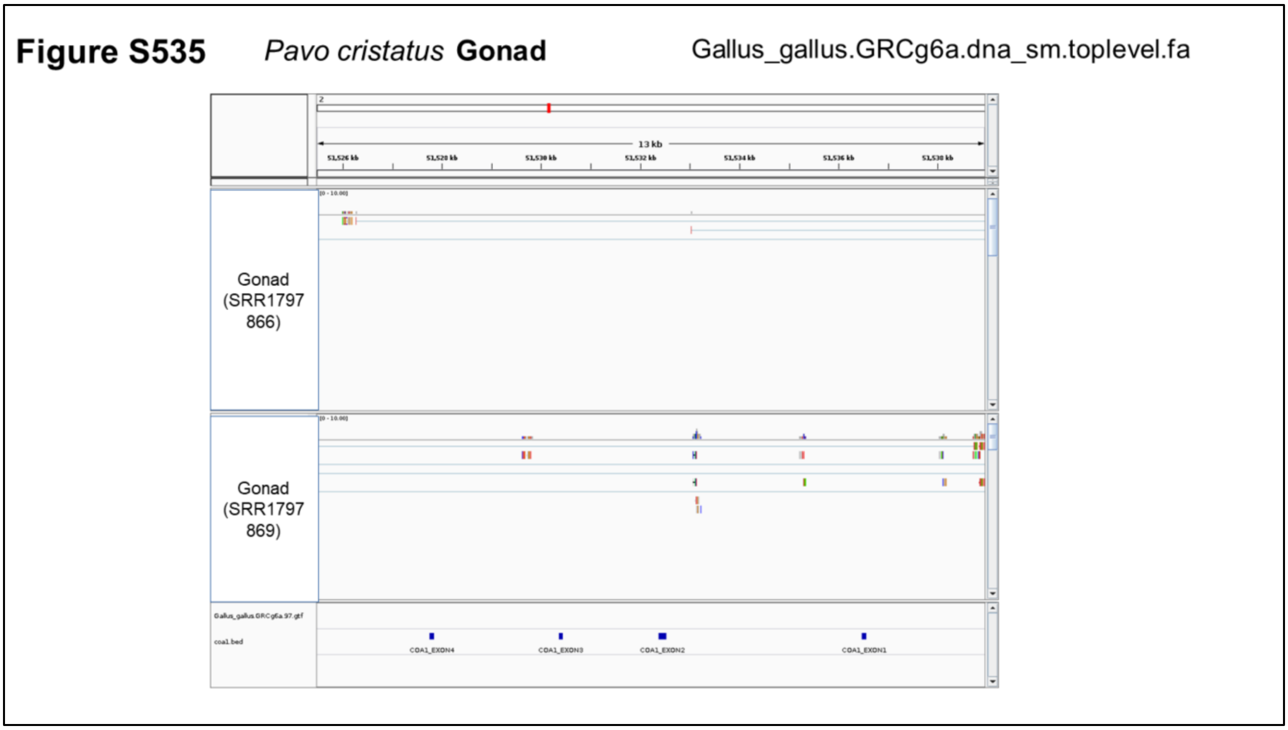

Figure S535: Screenshot of RNA-seq reads aligned to each of the putative locations of the four *COA1* exons in Peacock (*Pavo cristatus*). Gray boxes represent reads aligned to the genome and thin lines connecting these boxes represent the spliced read alignments. Datasets from gonad (SRR1797866) and (SRR1797869) have been mapped to the Gallus_gallus.GRCg6a.dna_sm.toplevel.fa genome. Locations of each of the exons are depicted as bed record in the bottom row.

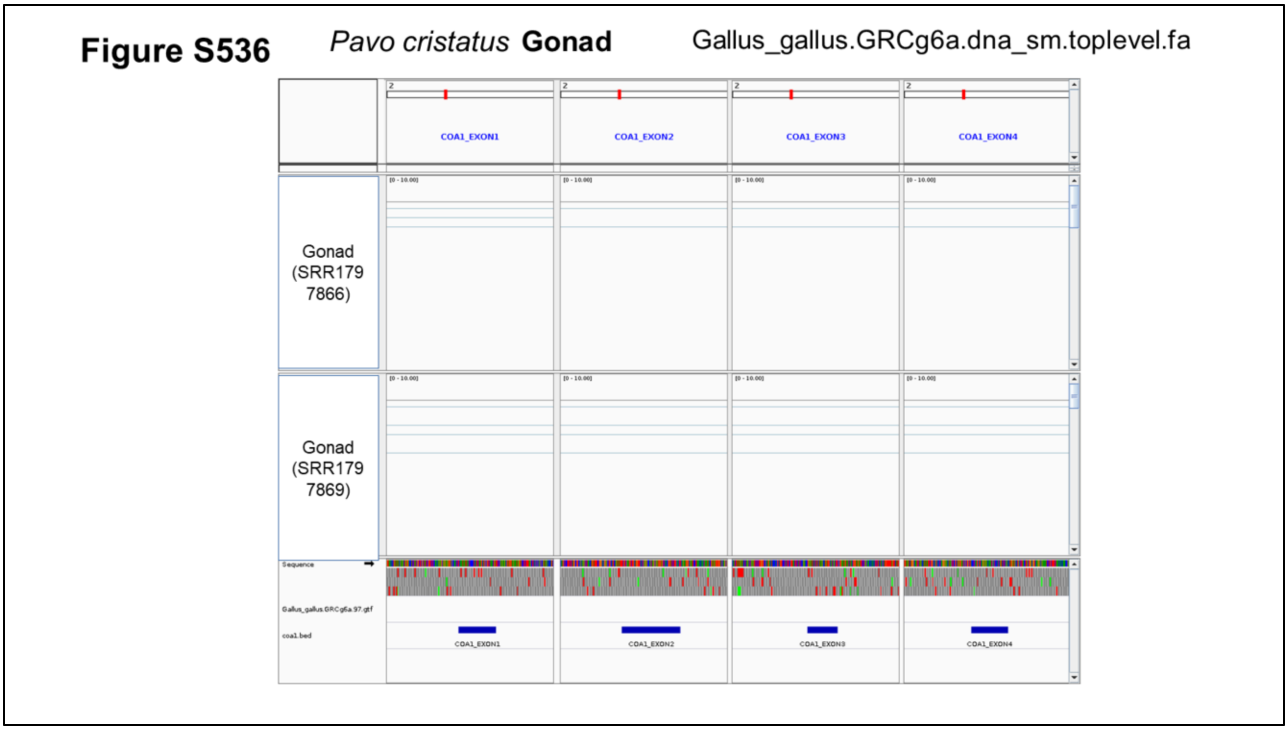

Figure S536: A zoomed in view of each of the *COA1* exons shown in **Supplementary Fig. S535**.

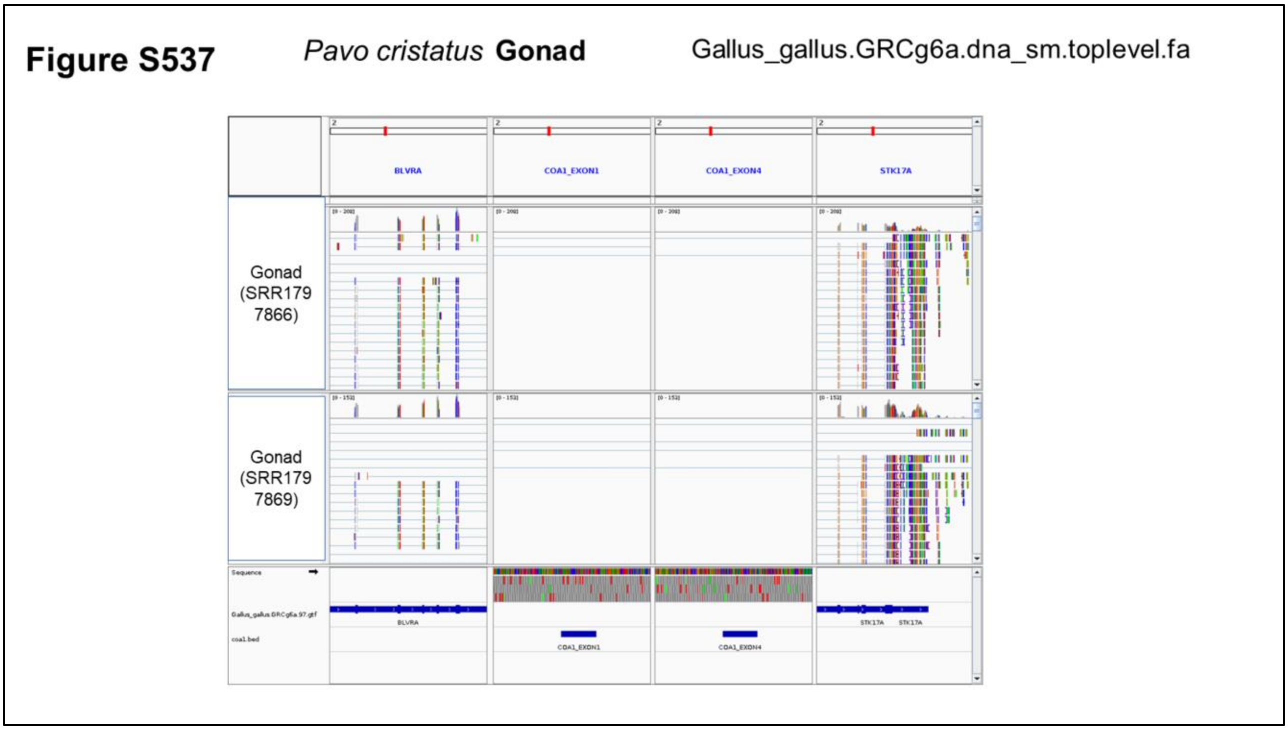

Figure S537: Screenshot of RNA-seq reads aligned to *BLVRA* and *STK17A* to *COA1* exon 1 and 4 respectively in Peacock (*Pavo cristatus*). Gray boxes represent reads aligned to the genome and thin lines connecting these boxes represent the spliced read alignments. Datasets from gonad (SRR1797866) and (SRR1797869) have been mapped to the Gallus_gallus.GRCg6a.dna_sm.toplevel.fa genome. Locations of each of the exons are depicted as bed record in the bottom row.

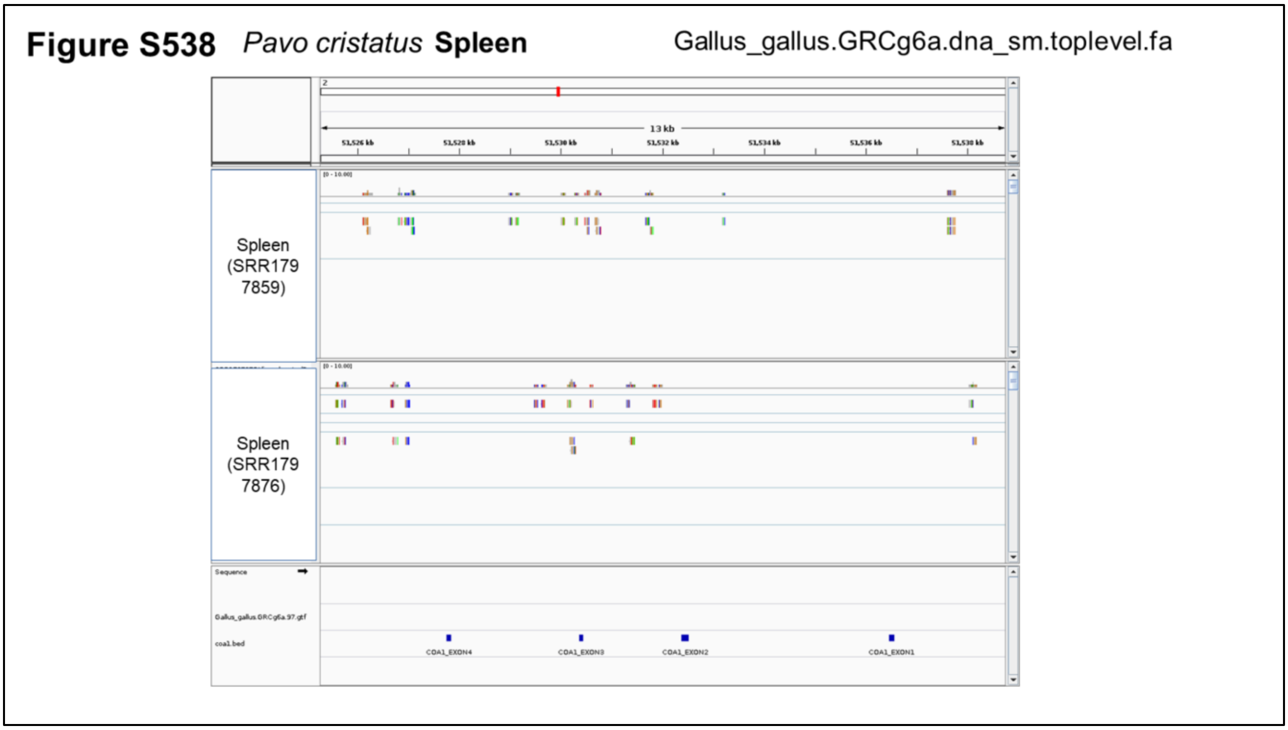

Figure S538: Screenshot of RNA-seq reads aligned to each of the putative locations of the four *COA1* exons in Peacock (*Pavo cristatus*). Gray boxes represent reads aligned to the genome and thin lines connecting these boxes represent the spliced read alignments. Datasets from spleen (SRR1797859) and (SRR1797876) have been mapped to the Gallus_gallus.GRCg6a.dna_sm.toplevel.fa genome. Locations of each of the exons are depicted as bed record in the bottom row.

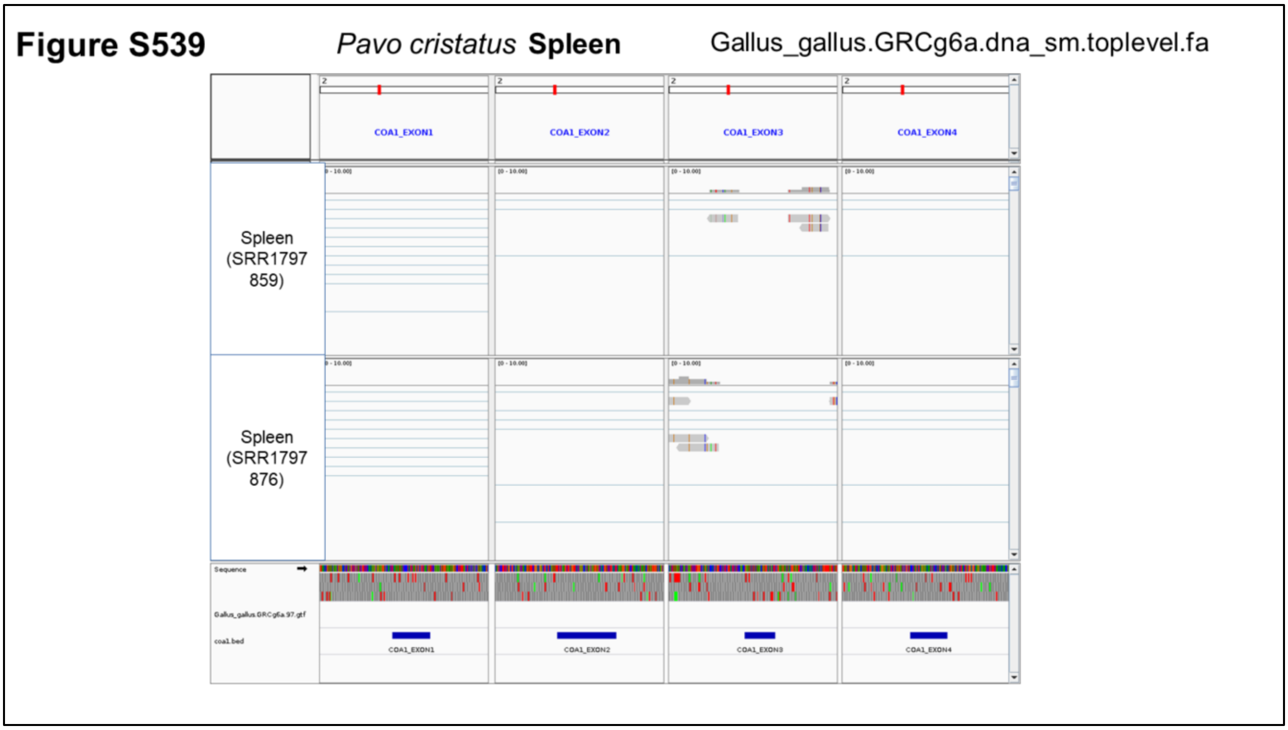

Figure S539: A zoomed in view of each of the *COA1* exons shown in **Supplementary Fig. S538**.

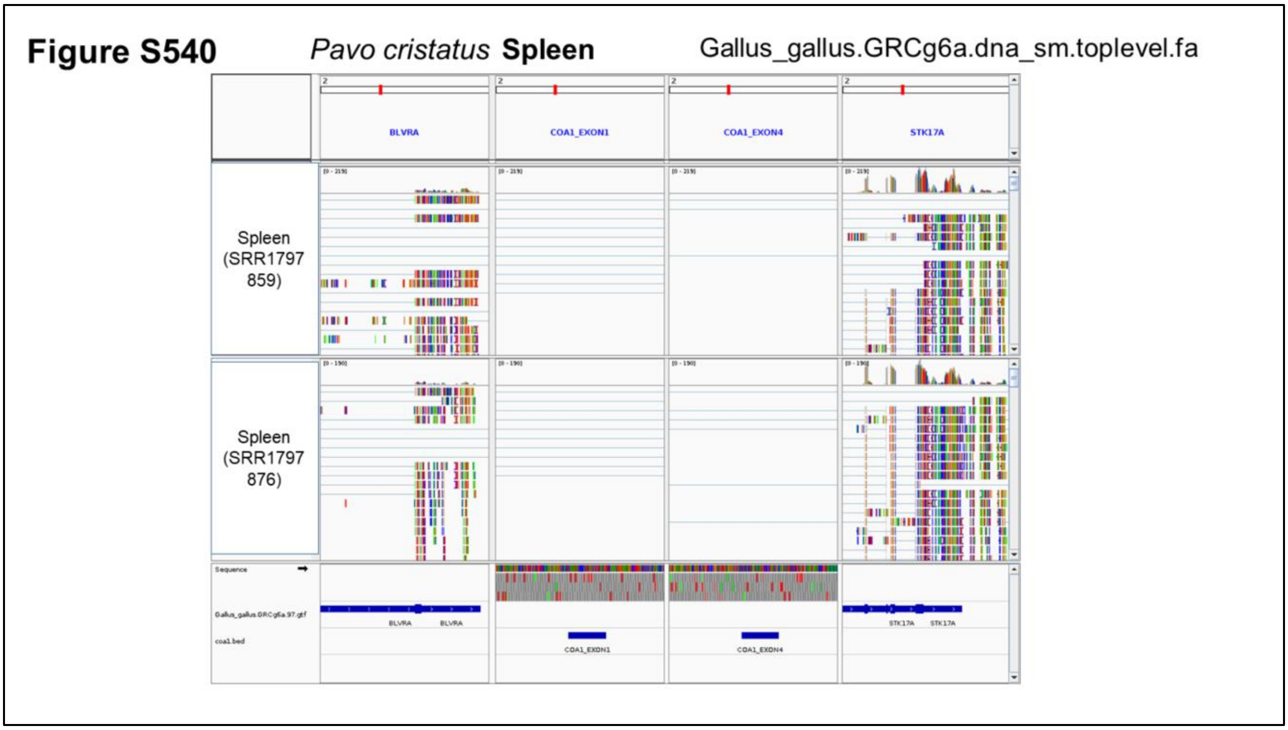

Figure S540: Screenshot of RNA-seq reads aligned to *BLVRA* and *STK17A* to *COA1* exon 1 and 4 respectively in Peacock (*Pavo cristatus*). Gray boxes represent reads aligned to the genome and thin lines connecting these boxes represent the spliced read alignments. Datasets from spleen (SRR1797859) and (SRR1797876) have been mapped to the Gallus_gallus.GRCg6a.dna_sm.toplevel.fa genome. Locations of each of the exons are depicted as bed record in the bottom row.

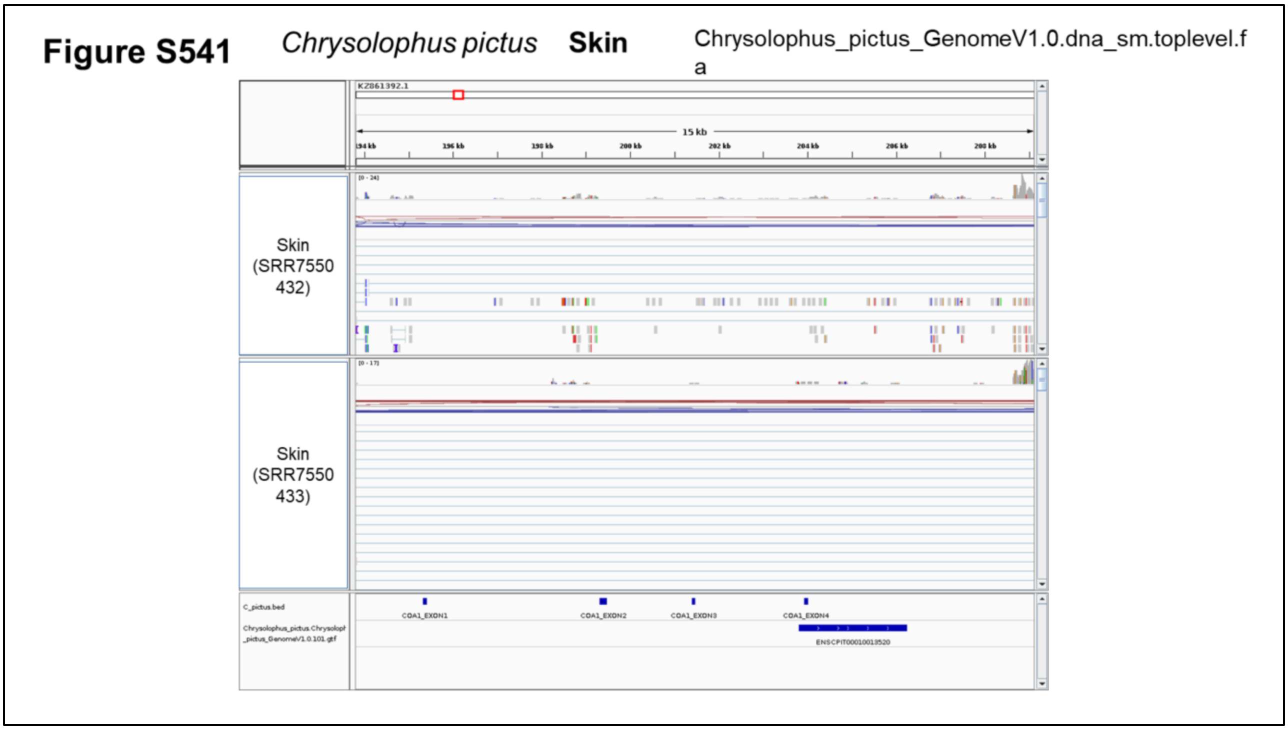

Figure S541: Screenshot of RNA-seq reads aligned to each of the putative locations of the four *COA1* exons in golden pheasant (*Chrysolophus pictus*). Gray boxes represent reads aligned to the genome and thin lines connecting these boxes represent the spliced read alignments. Datasets from skin (SRR7550432) and (SRR7550433) have been mapped to the Chrysolophus_pictus_GenomeV1.0.dna_sm.toplevel.fa genome. Locations of each of the exons are depicted as bed record in the bottom row.

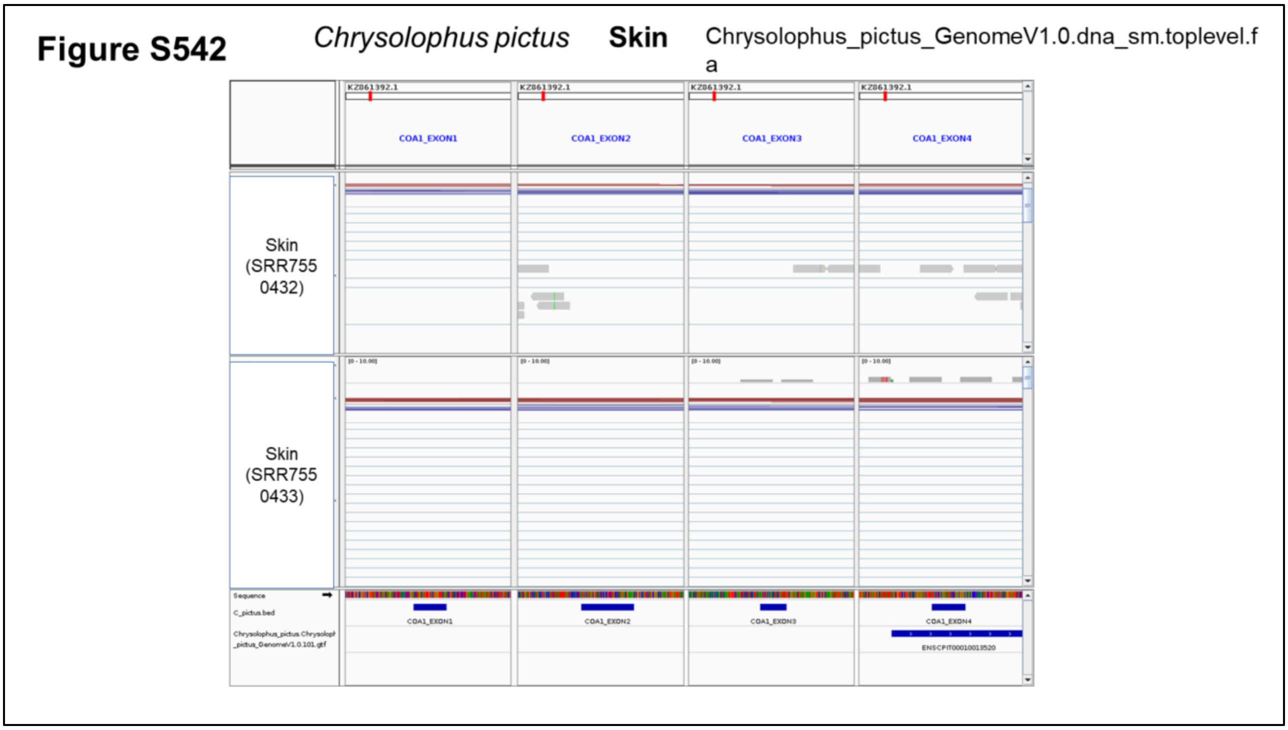

Figure S542: A zoomed in view of each of the *COA1* exons shown in **Supplementary Fig. S541**.

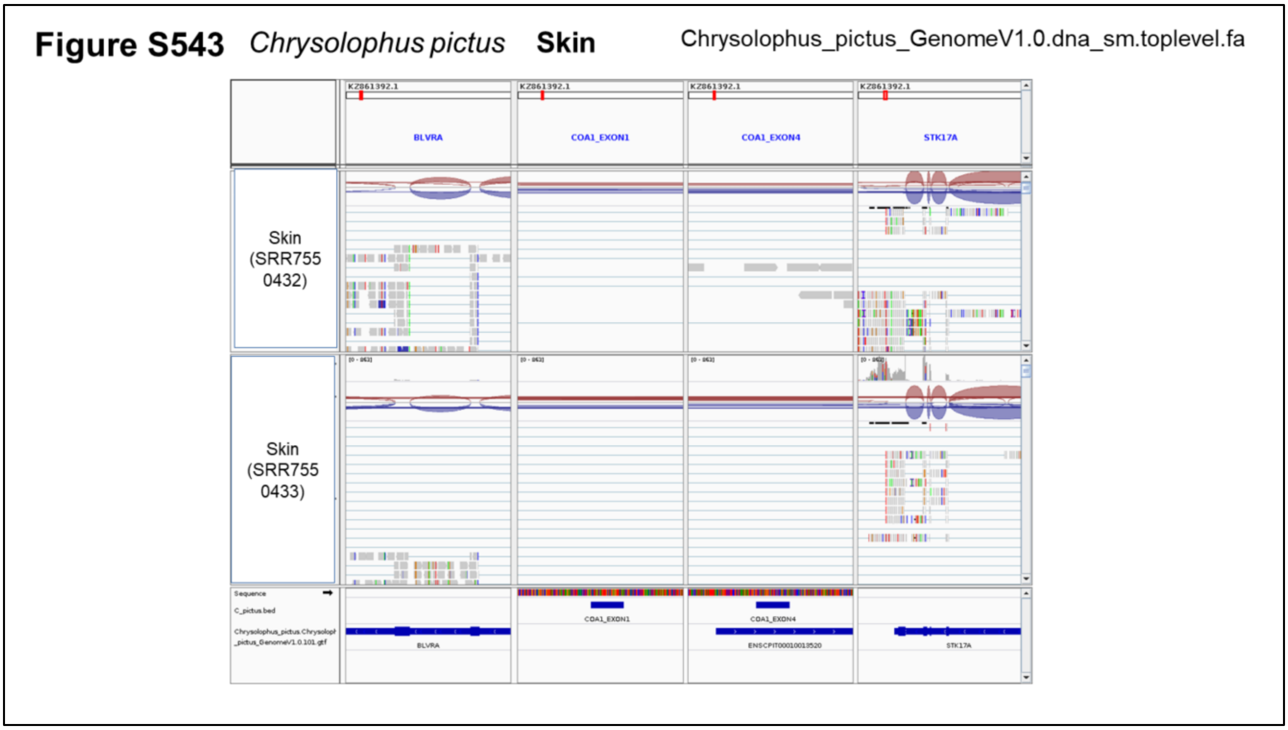

Figure S543: Screenshot of RNA-seq reads aligned to *BLVRA* and *STK17A* to *COA1* exon 1 and 4 respectively in golden pheasant (*Chrysolophus pictus*). Gray boxes represent reads aligned to the genome and thin lines connecting these boxes represent the spliced read alignments. Datasets from skin (SRR7550432) and (SRR7550433) have been mapped to the Chrysolophus_pictus_GenomeV1.0.dna_sm.toplevel.fa. Locations of each of the exons are depicted as bed record in the bottom row.

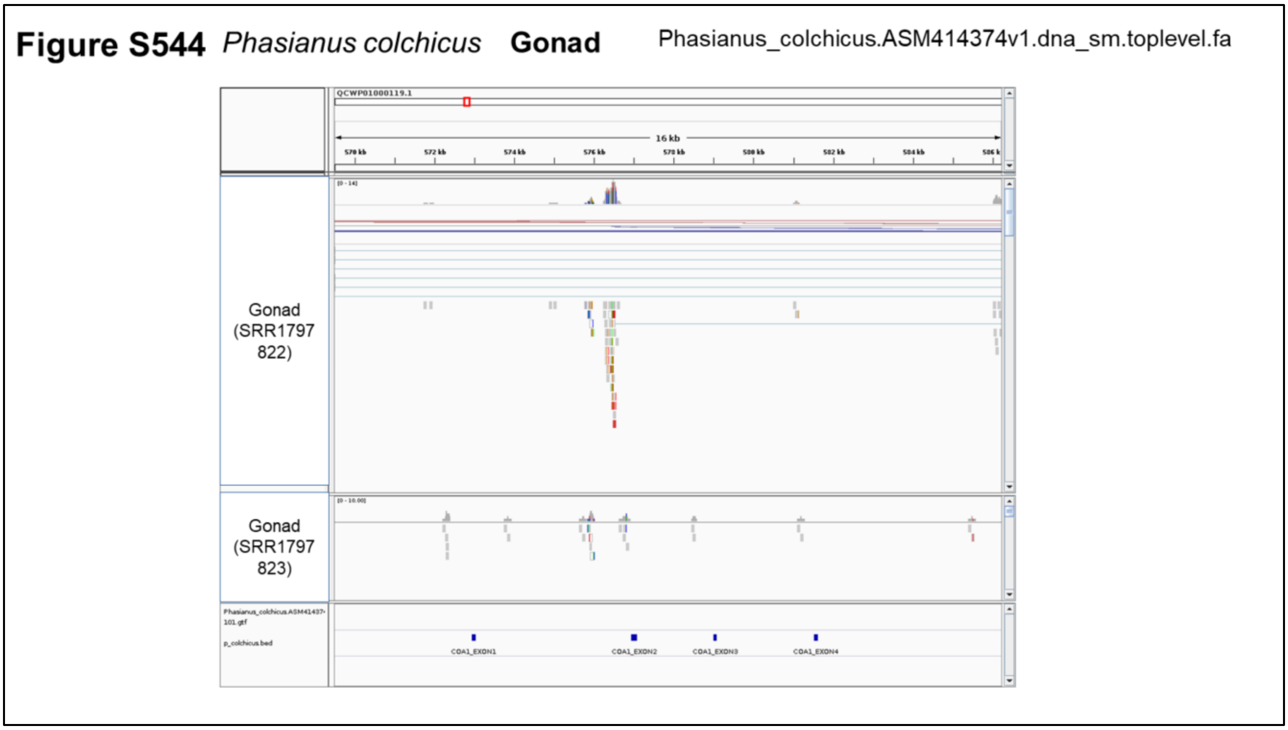

Figure S554: Screenshot of RNA-seq reads aligned to each of the putative locations of the four *COA1* exons in common pheasant (*Phasianus colchicus*). Gray boxes represent reads aligned to the genome and thin lines connecting these boxes represent the spliced read alignments. Datasets from gonad (SRR1797822) and (SRR1797823) have been mapped to the Phasianus_colchicus.ASM414374v1.dna_sm.toplevel.fa genome. Locations of each of the exons are depicted as bed record in the bottom row.

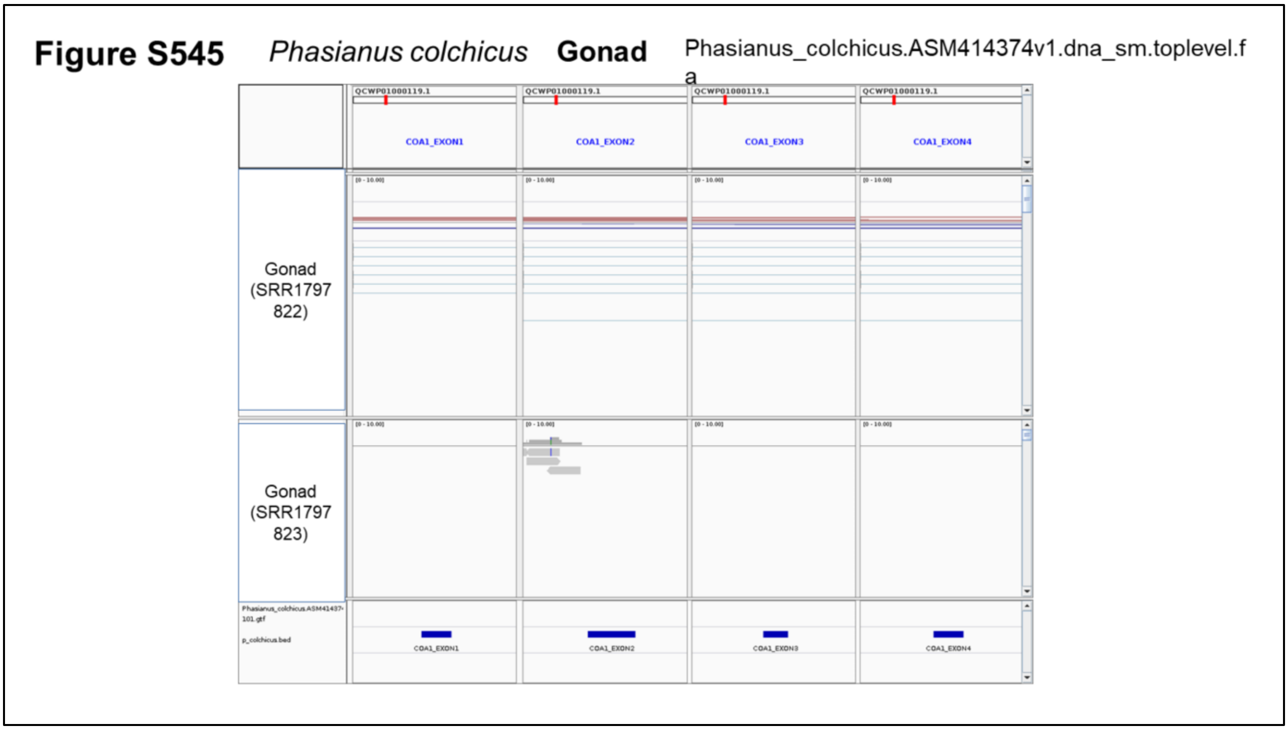

Figure S545: A zoomed in view of each of the *COA1* exons shown in **Supplementary Fig. S544**.

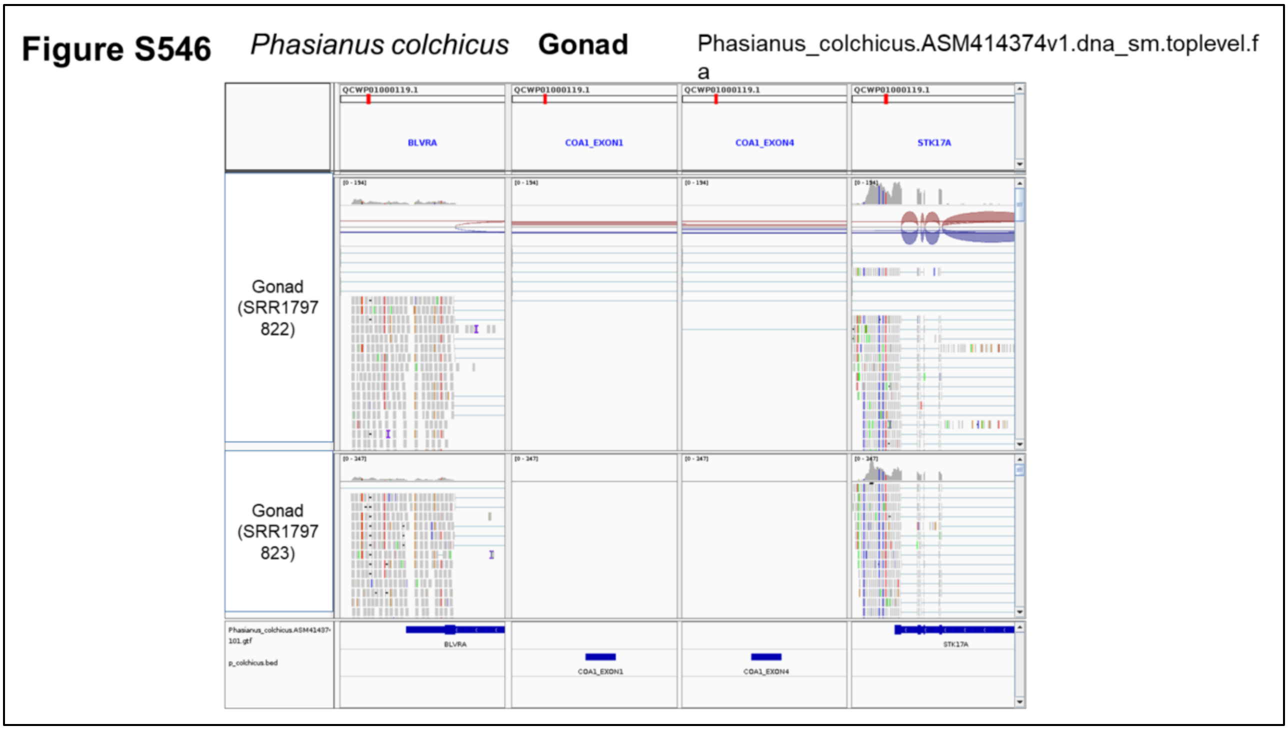

Figure S546: Screenshot of RNA-seq reads aligned to *BLVRA* and *STK17A* to *COA1* exon 1 and 4 respectively in common pheasant (*Phasianus colchicus*). Gray boxes represent reads aligned to the genome and thin lines connecting these boxes represent the spliced read alignments. Datasets from gonad (SRR1797822) and (SRR1797823) have been mapped to the Phasianus_colchicus.ASM414374v1.dna_sm.toplevel.fa genome. Locations of each of the exons are depicted as bed record in the bottom row.

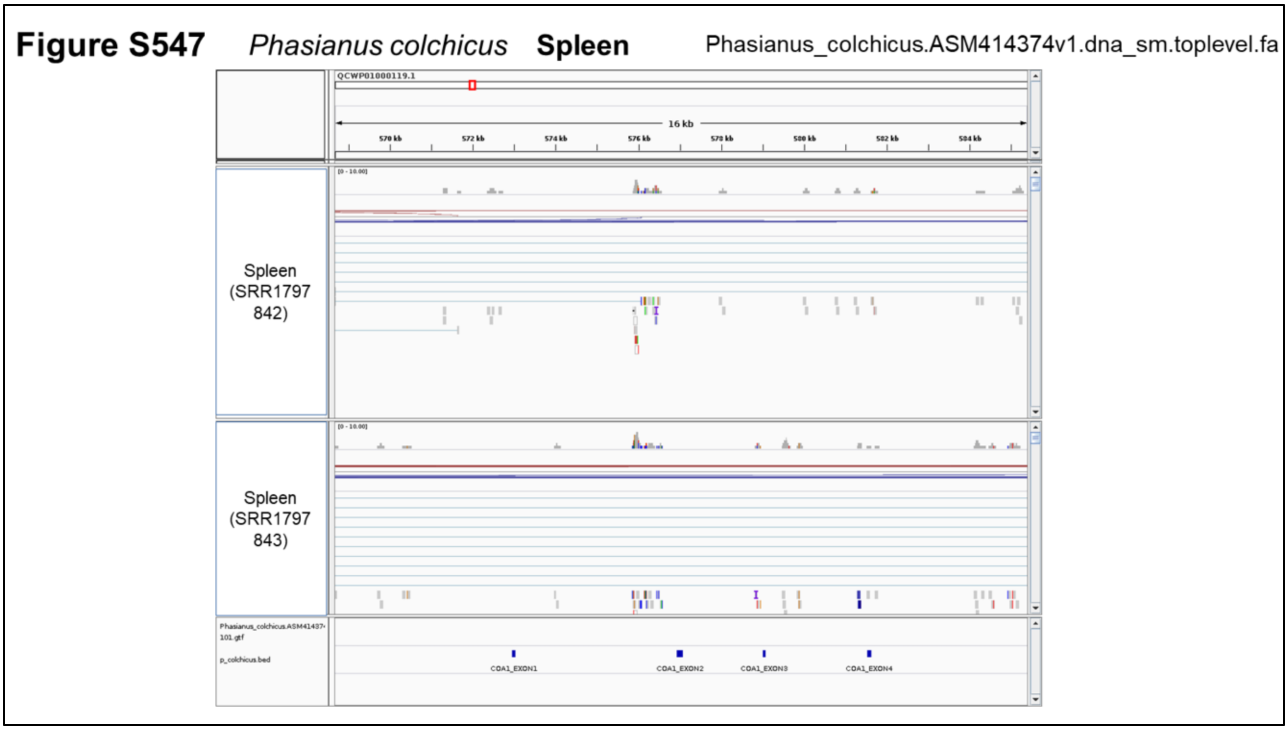

Figure S547: Screenshot of RNA-seq reads aligned to each of the putative locations of the four *COA1* exons in common pheasant (*Phasianus colchicus*). Gray boxes represent reads aligned to the genome and thin lines connecting these boxes represent the spliced read alignments. Datasets from spleen (SRR1797842) and (SRR1797843) have been mapped to the Phasianus_colchicus.ASM414374v1.dna_sm.toplevel.fa genome. Locations of each of the exons are depicted as bed record in the bottom row.

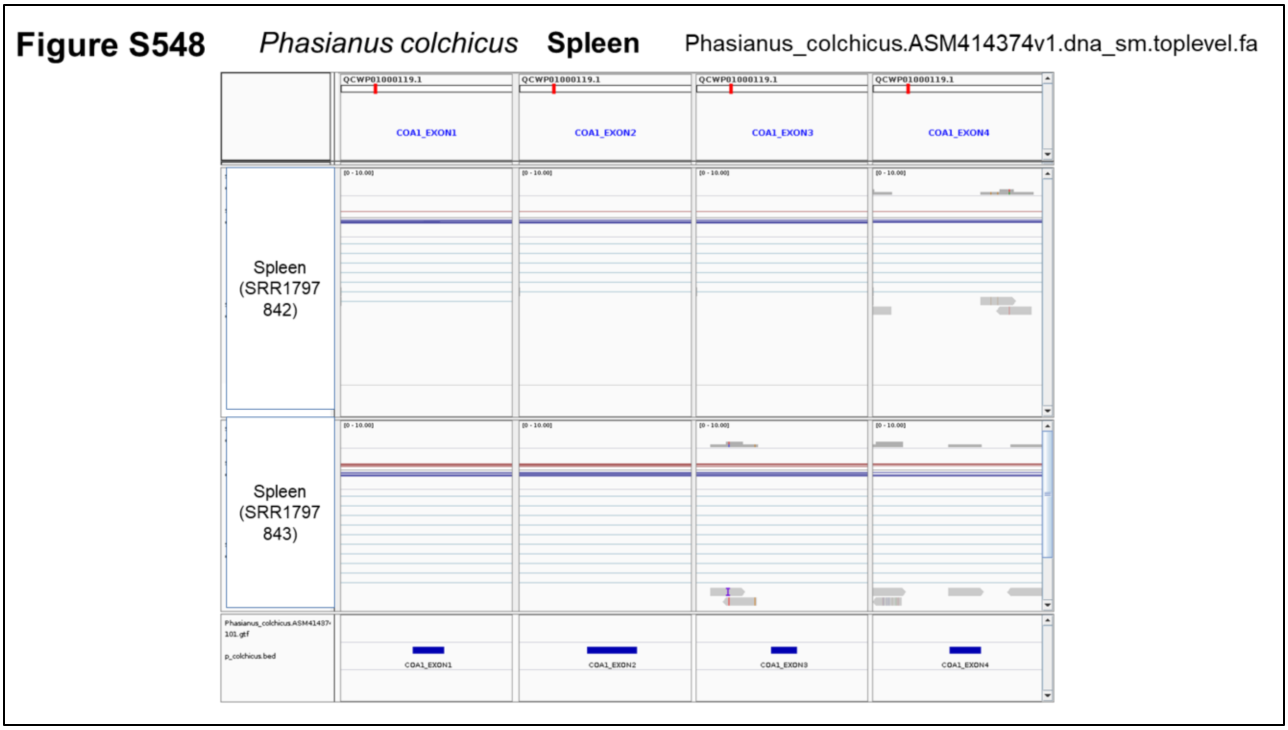

Figure S548: A zoomed in view of each of the *COA1* exons shown in **Supplementary Fig. S547**.

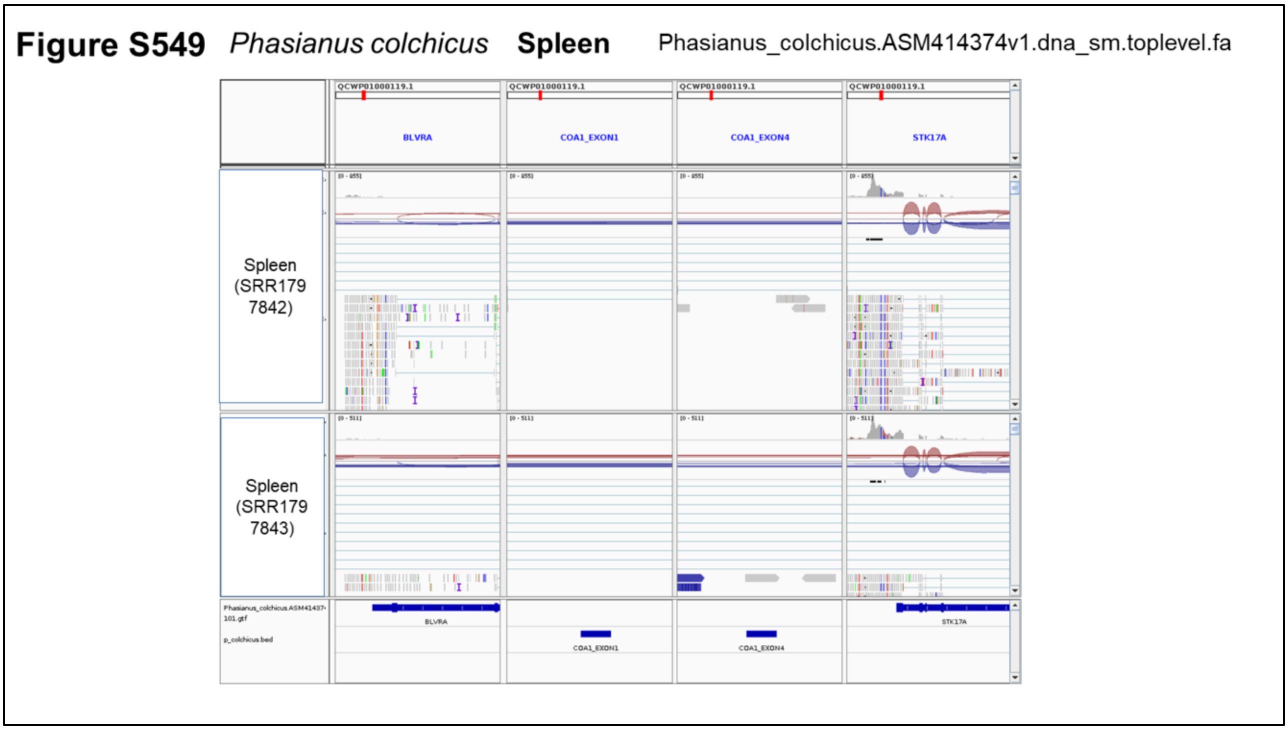

Figure S549: Screenshot of RNA-seq reads aligned to *BLVRA* and *STK17A* to *COA1* exon 1 and 4 respectively in common pheasant (*Phasianus colchicus*). Gray boxes represent reads aligned to the genome and thin lines connecting these boxes represent the spliced read alignments. Datasets from spleen (SRR1797842) and (SRR1797843) have been mapped to the Phasianus_colchicus.ASM414374v1.dna_sm.toplevel.fa genome. Locations of each of the exons are depicted as bed record in the bottom row.

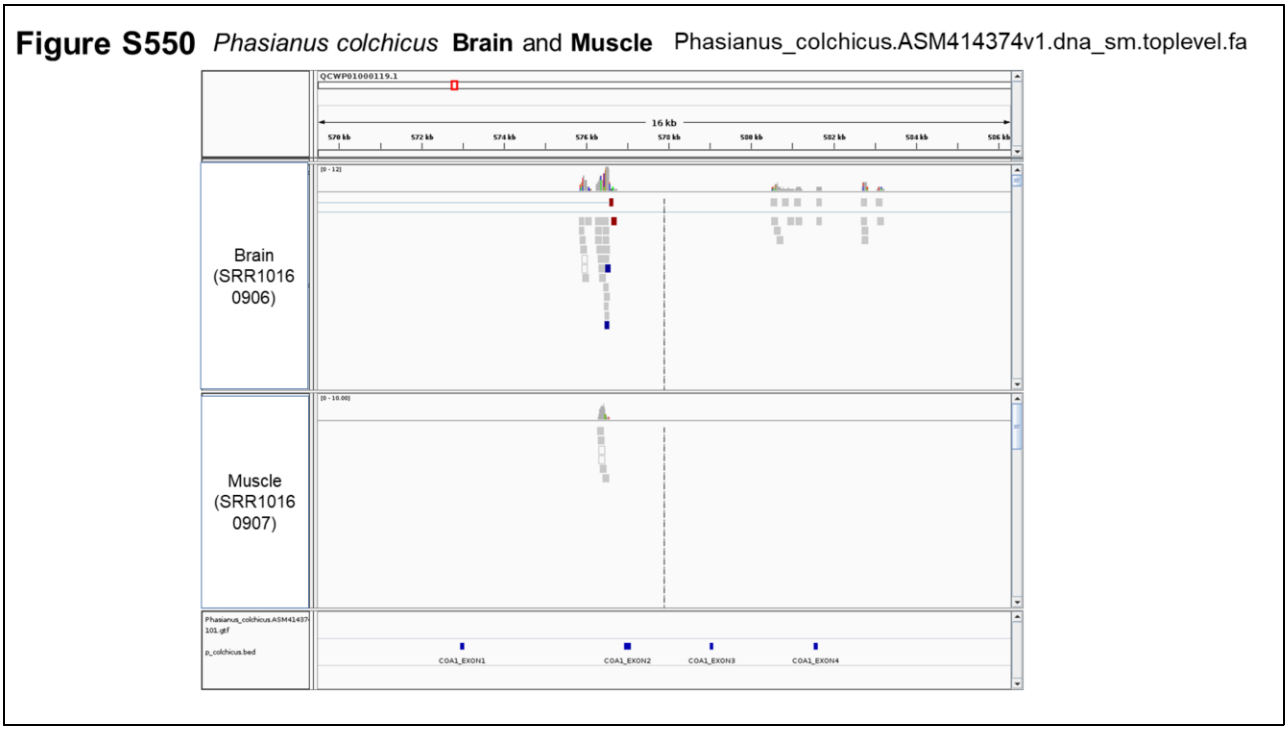

Figure S550: Screenshot of RNA-seq reads aligned to each of the putative locations of the four *COA1* exons in common pheasant (*Phasianus colchicus*). Gray boxes represent reads aligned to the genome and thin lines connecting these boxes represent the spliced read alignments. Datasets from Brain (SRR10160906) and Muscle (SRR10160907) have been mapped to the Phasianus_colchicus.ASM414374v1.dna_sm.toplevel.fa genome. Locations of each of the exons are depicted as bed record in the bottom row.

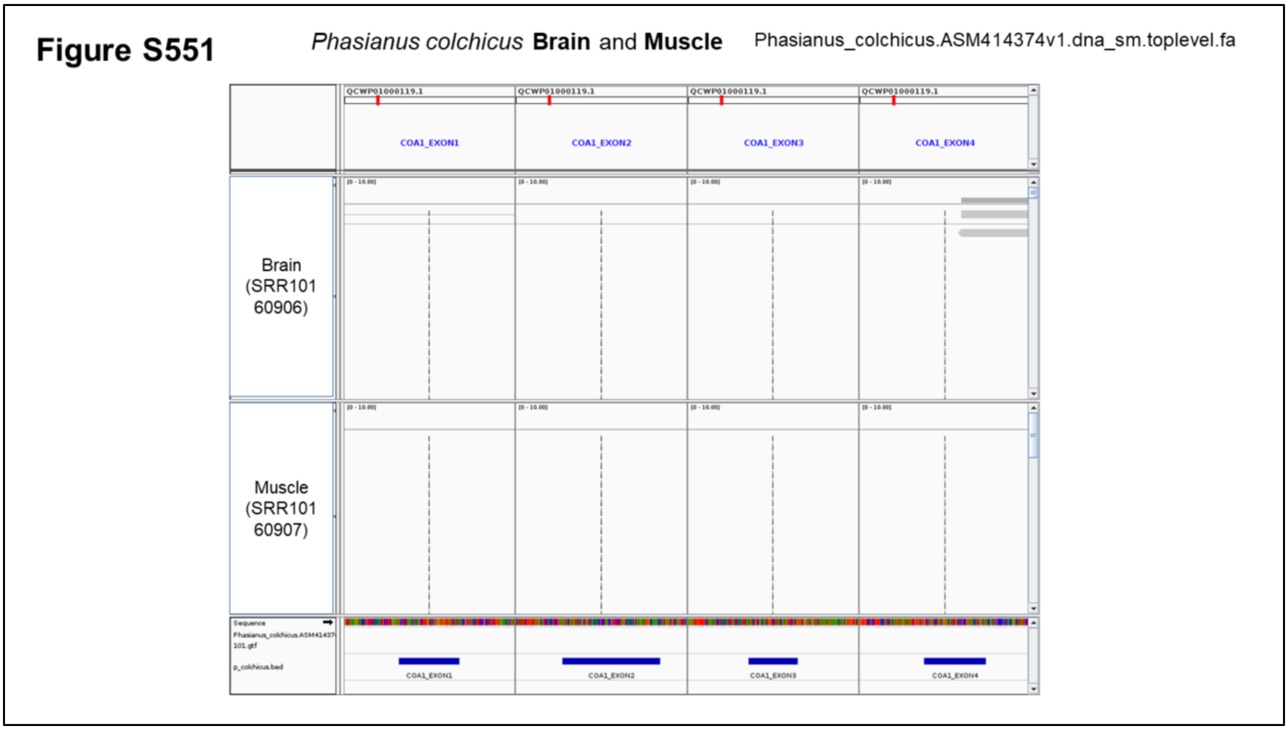

Figure S551: A zoomed in view of each of the *COA1* exons shown in **Supplementary Fig. S550**.

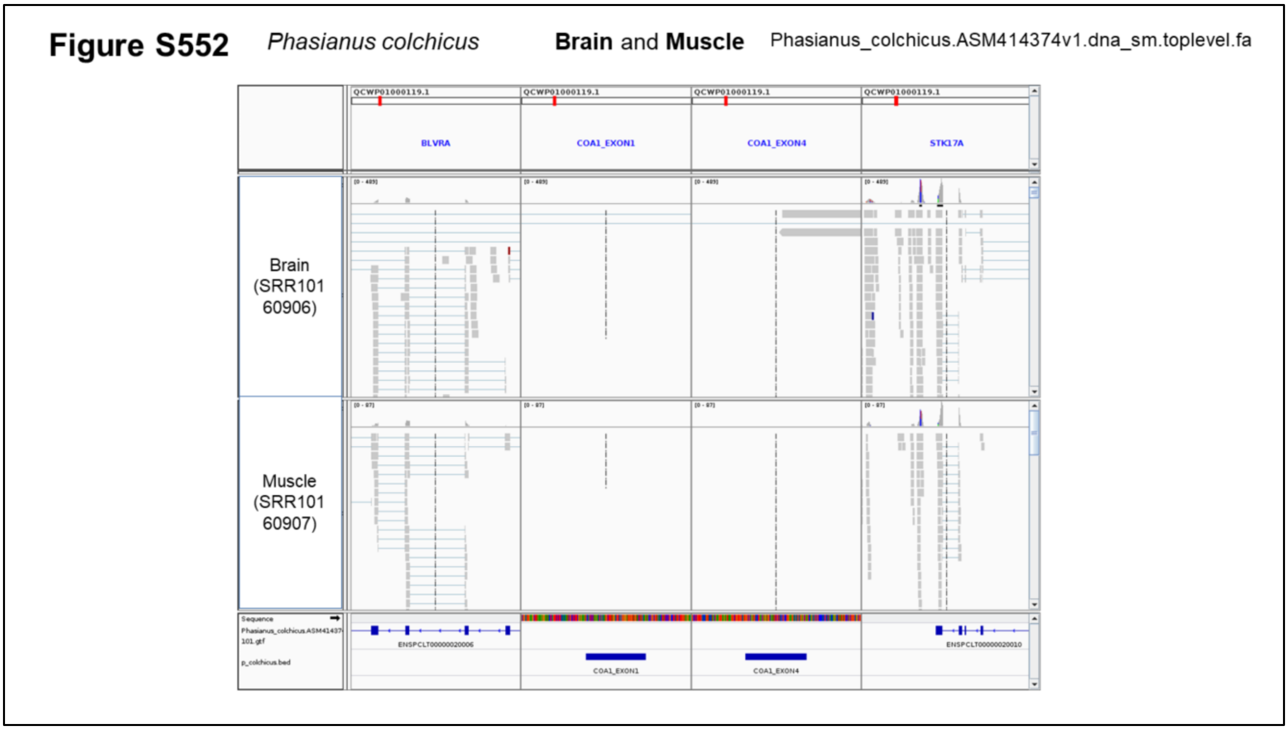

Figure S552: Screenshot of RNA-seq reads aligned to *BLVRA* and *STK17A* to *COA1* exon 1 and 4 respectively in common pheasant (*Phasianus colchicus*). Gray boxes represent reads aligned to the genome and thin lines connecting these boxes represent the spliced read alignments. Datasets from brain (SRR10160906) and muscle (SRR10160907) have been mapped to the Phasianus_colchicus.ASM414374v1.dna_sm.toplevel.fa genome. Locations of each of the exons are depicted as bed record in the bottom row.

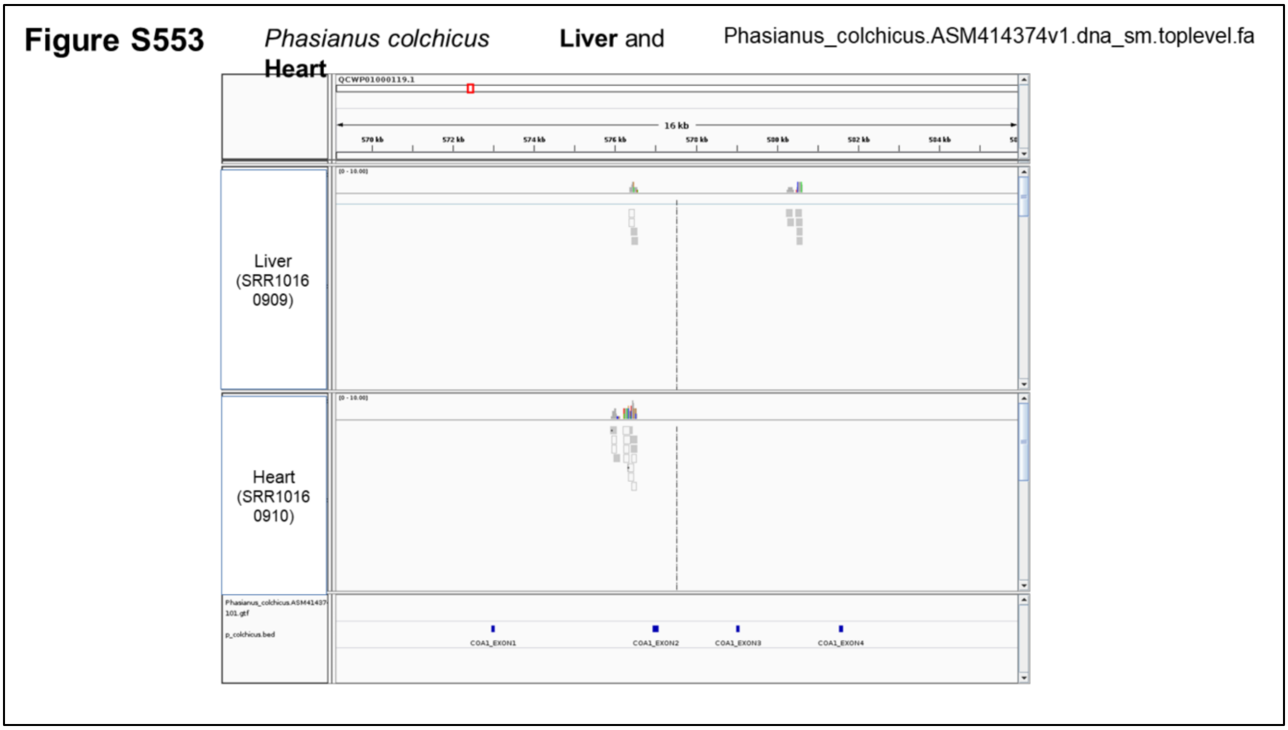

Figure S553: Screenshot of RNA-seq reads aligned to each of the putative locations of the four *COA1* exons in common pheasant (*Phasianus colchicus*). Gray boxes represent reads aligned to the genome and thin lines connecting these boxes represent the spliced read alignments. Datasets from liver (SRR10160909) and heart (SRR10160910) have been mapped to the Phasianus_colchicus.ASM414374v1.dna_sm.toplevel.fa genome. Locations of each of the exons are depicted as bed record in the bottom row.

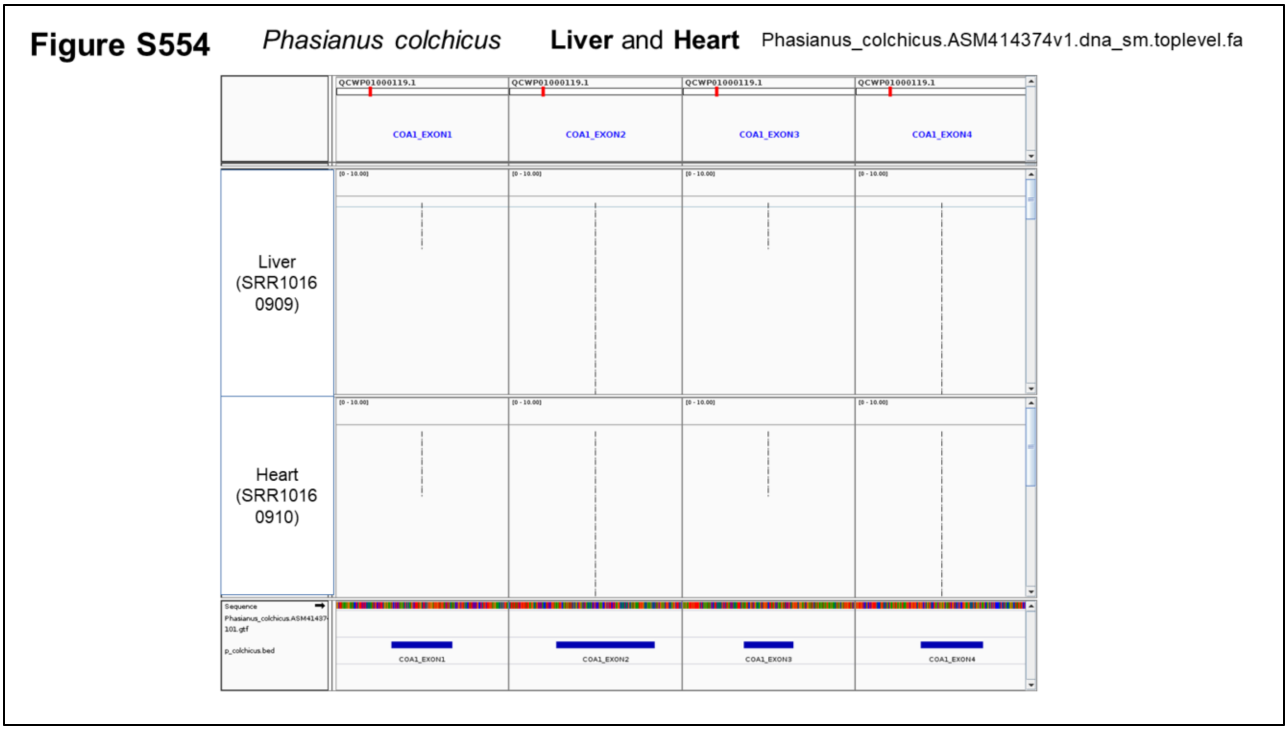

Figure S554: A zoomed in view of each of the *COA1* exons shown in **Supplementary Fig. S553**.

Figure S555 *Phasianus colchicus* **Liver** and **Heart** Phasianus_colchicus.ASM414374v1.dna_sm.toplevel.fa

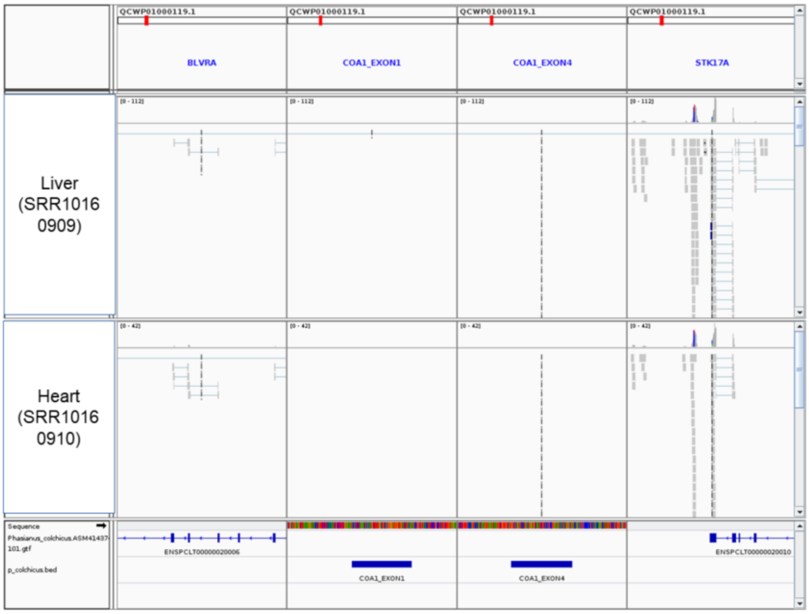

Figure S555: Screenshot of RNA-seq reads aligned to *BLVRA* and *STK17A* to *COA1* exon 1 and 4 respectively in common pheasant (*Phasianus colchicus*). Gray boxes represent reads aligned to the genome and thin lines connecting these boxes represent the spliced read alignments. Datasets from liver (SRR10160909) and heart (SRR10160910) have been mapped to the Phasianus_colchicus.ASM414374v1.dna_sm.toplevel.fa genome. Locations of each of the exons are depicted as bed record in the bottom row.

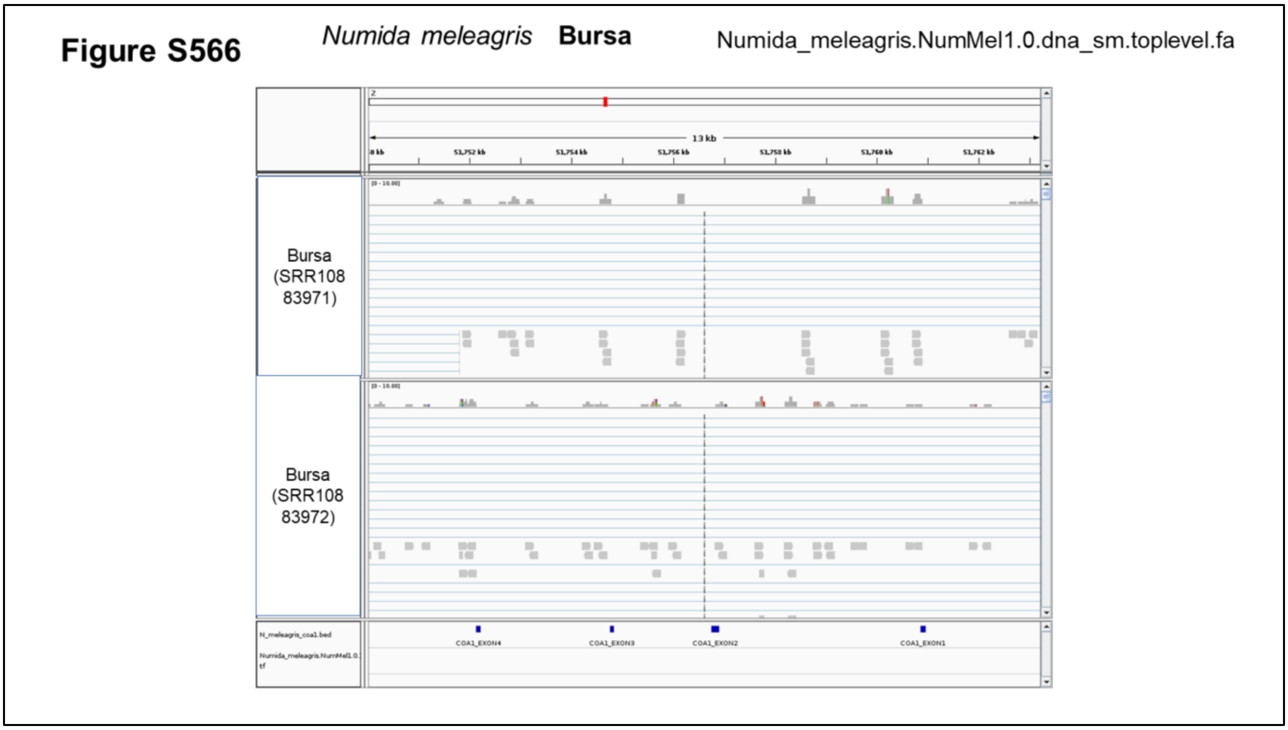

Figure S556: Screenshot of RNA-seq reads aligned to each of the putative locations of the four *COA1* exons in helmeted guineafowl (*Numida meleagris*). Gray boxes represent reads aligned to the genome and thin lines connecting these boxes represent the spliced read alignments. Datasets from bursa (SRR10883971) and (SRR10883972) have been mapped to the Numida_meleagris.NumMel1.0.dna_sm.toplevel.fa genome. Locations of each of the exons are depicted as bed record in the bottom row.

Figure S557 *Numida meleagris* **Bursa** Numida_meleagris.NumMel1.0.dna_sm.toplevel.fa

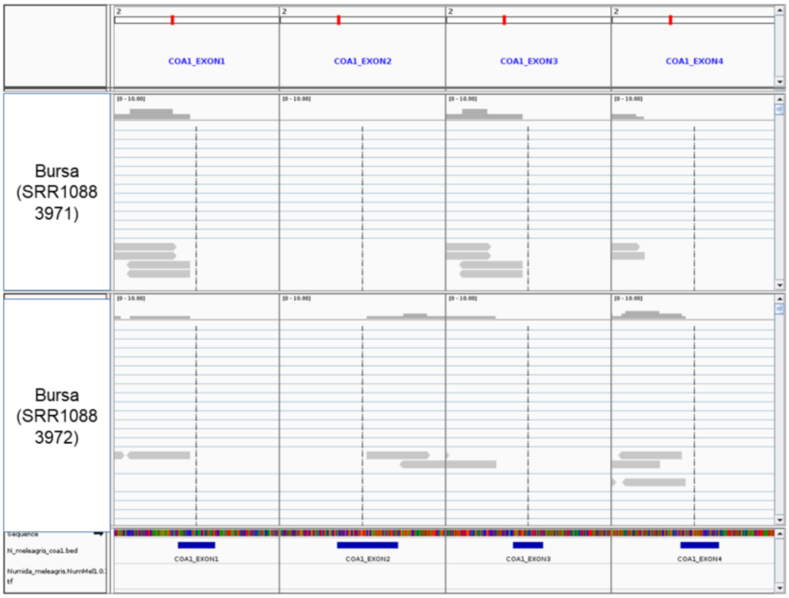

Figure S557: A zoomed in view of each of the *COA1* exons shown in **Supplementary Fig. S556**.

Figure S558 *Numida meleagris* **Bursa** Numida_meleagris.NumMel1.0.dna_sm.toplevel.fa

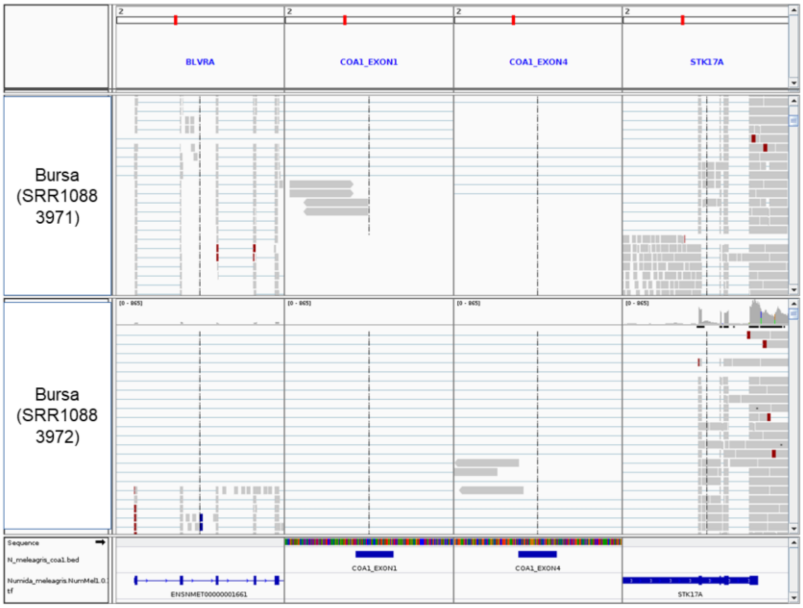

Figure S558: Screenshot of RNA-seq reads aligned to *BLVRA* and *STK17A* to *COA1* exon 1 and 4 respectively in helmeted guineafowl (*Numida meleagris*). Gray boxes represent reads aligned to the genome and thin lines connecting these boxes represent the spliced read alignments. Datasets from bursa (SRR10883971) and (SRR10883972) have been mapped to the Numida_meleagris.NumMel1.0.dna_sm.toplevel.fa genome. Locations of each of the exons are depicted as bed record in the bottom row.

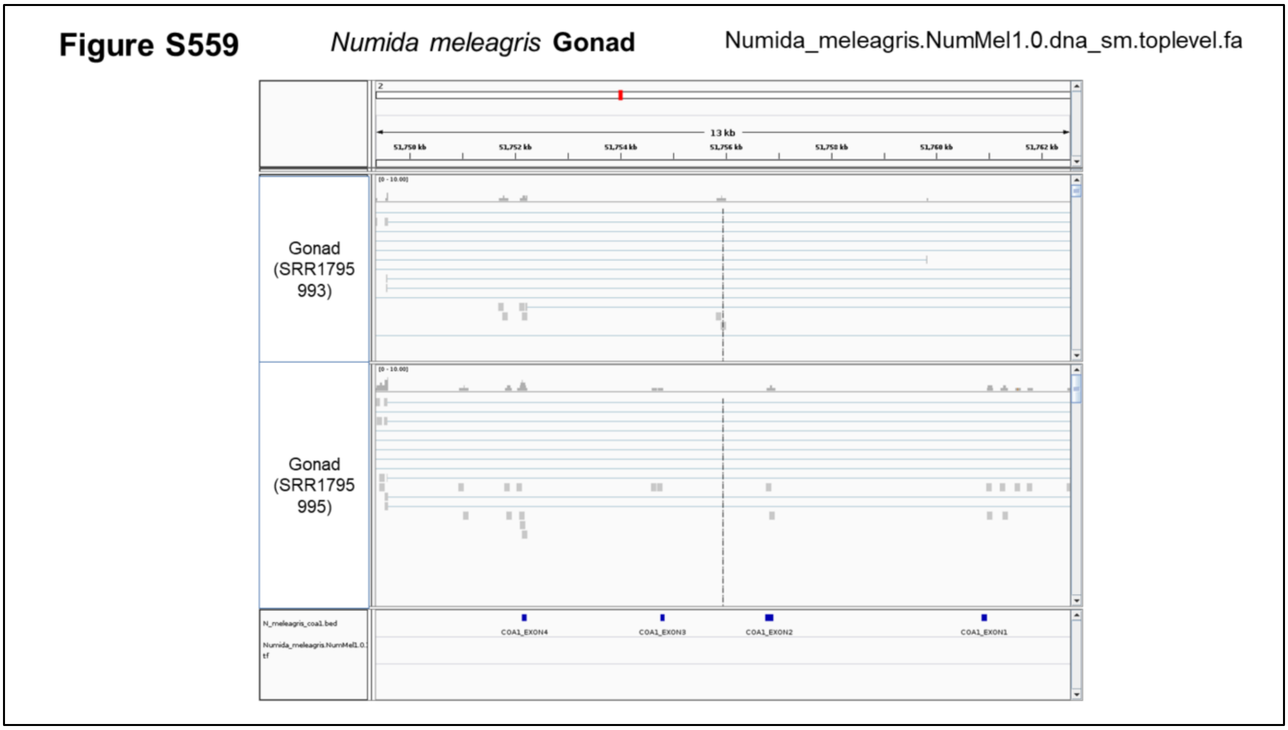

Figure S559: Screenshot of RNA-seq reads aligned to each of the putative locations of the four *COA1* exons in helmeted guineafowl (*Numida meleagris*). Gray boxes represent reads aligned to the genome and thin lines connecting these boxes represent the spliced read alignments. Datasets from gonad (SRR1795993) and (SRR1795995) have been mapped to the Numida_meleagris.NumMel1.0.dna_sm.toplevel.fa genome. Locations of each of the exons are depicted as bed record in the bottom row.

Figure S560 *Numida meleagris* **Gonad** Numida_meleagris.NumMel1.0.dna_sm.toplevel.fa

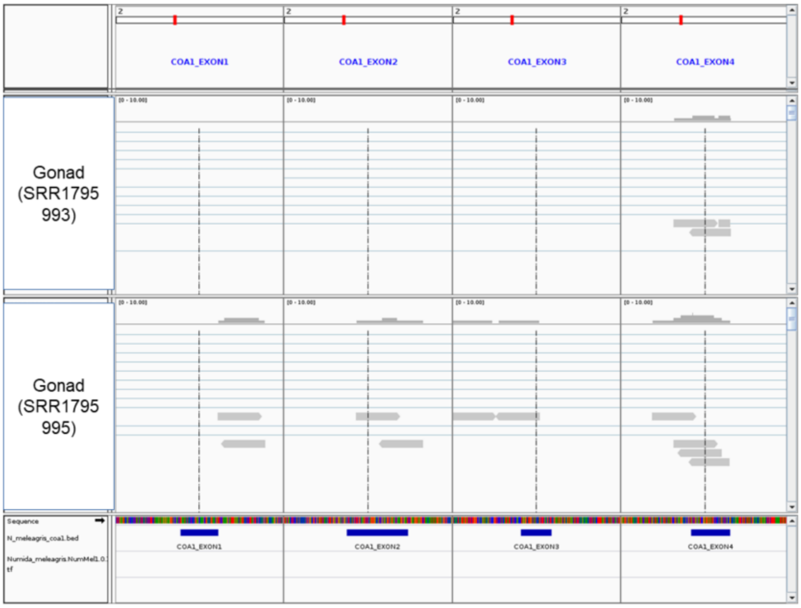

Figure S560: A zoomed in view of each of the *COA1* exons **Supplementary Fig. S559**.

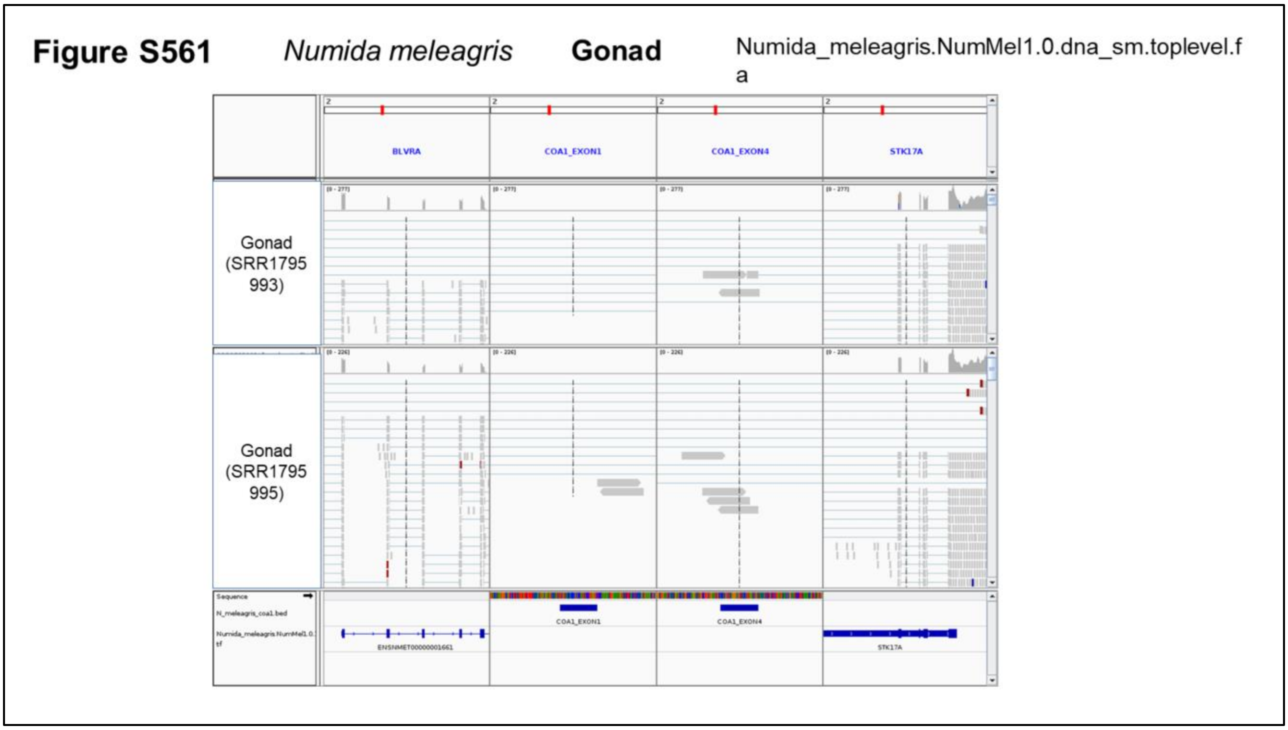

Figure S561: Screenshot of RNA-seq reads aligned to *BLVRA* and *STK17A* to *COA1* exon 1 and 4 respectively in helmeted guineafowl (*Numida meleagris*). Gray boxes represent reads aligned to the genome and thin lines connecting these boxes represent the spliced read alignments. Datasets from gonad (SRR1795993) and (SRR1795995) have been mapped to the Numida_meleagris.NumMel1.0.dna_sm.toplevel.fa genome. Locations of each of the exons are depicted as bed record in the bottom row.

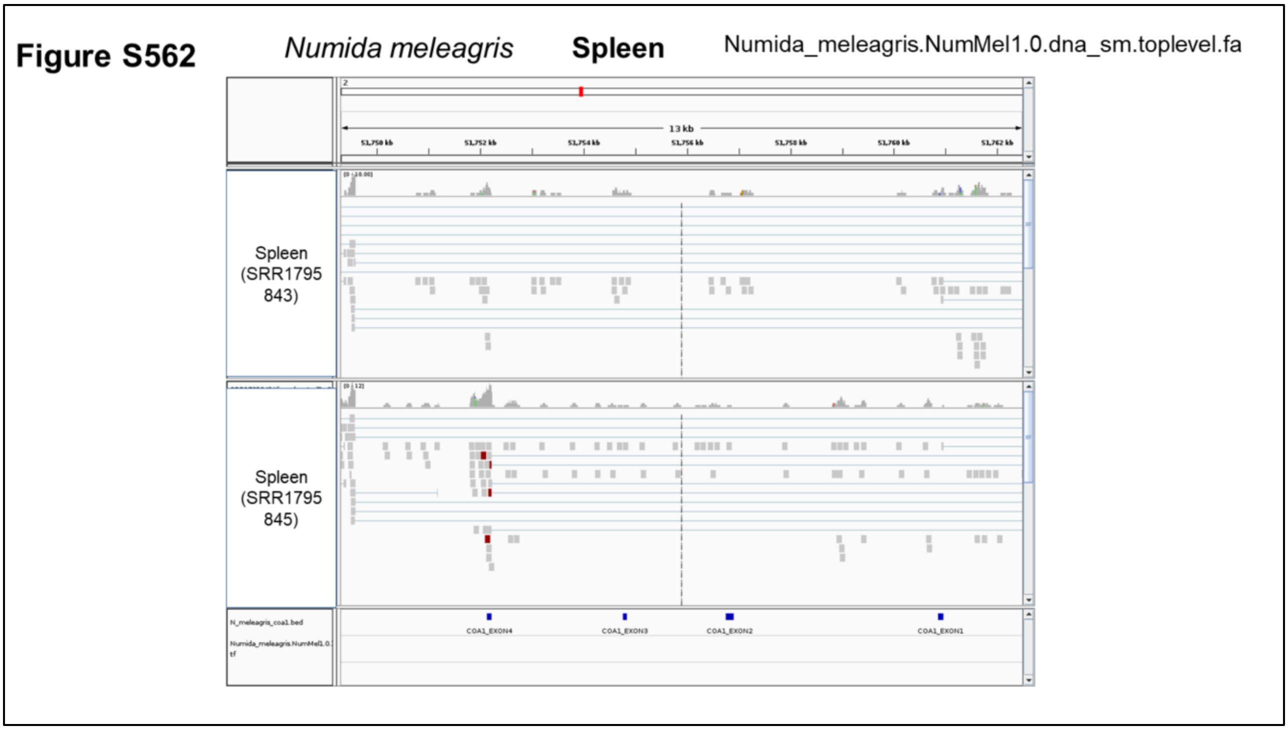

Figure S562: Screenshot of RNA-seq reads aligned to each of the putative locations of the four *COA1* exons in helmeted guineafowl (*Numida meleagris*). Gray boxes represent reads aligned to the genome and thin lines connecting these boxes represent the spliced read alignments. Datasets from spleen (SRR1795843) and (SRR1795845) have been mapped to the Numida_meleagris.NumMel1.0.dna_sm.toplevel.fa genome. Locations of each of the exons are depicted as bed record in the bottom row.

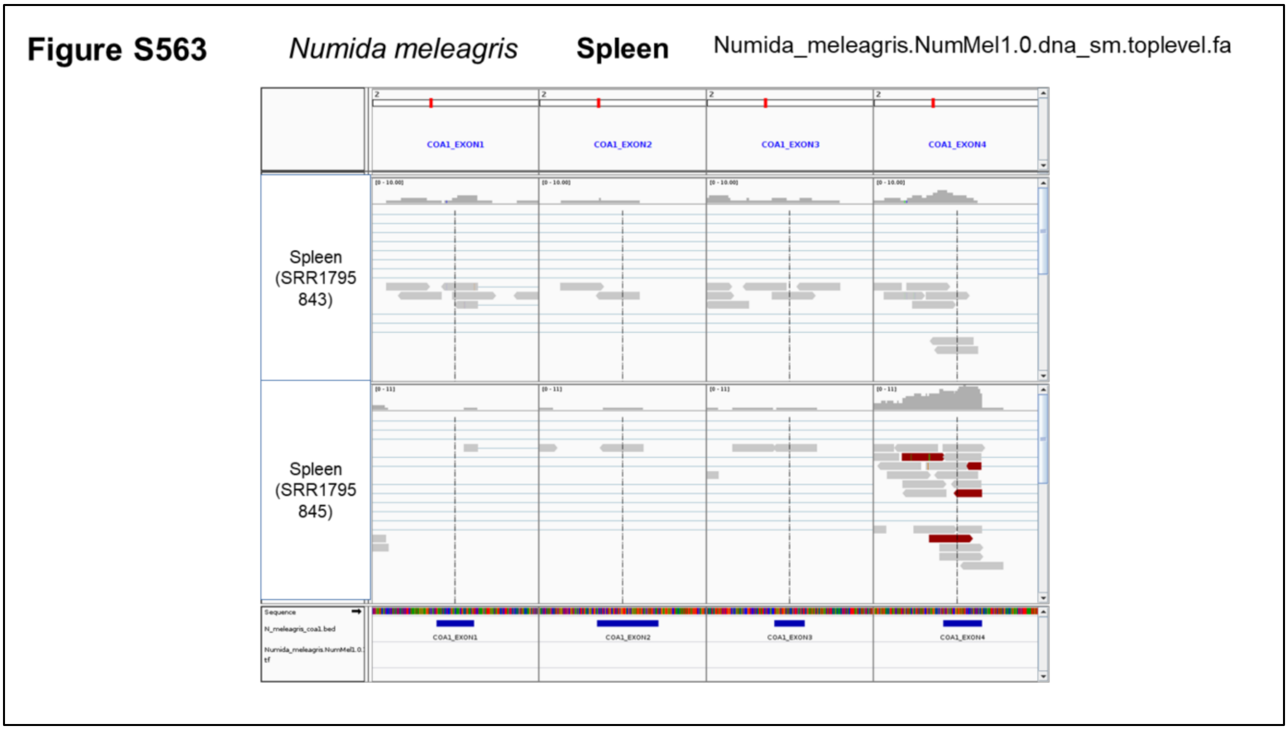

Figure S563: A zoomed in view of each of the *COA1* exons shown in **Supplementary Fig. S562**.

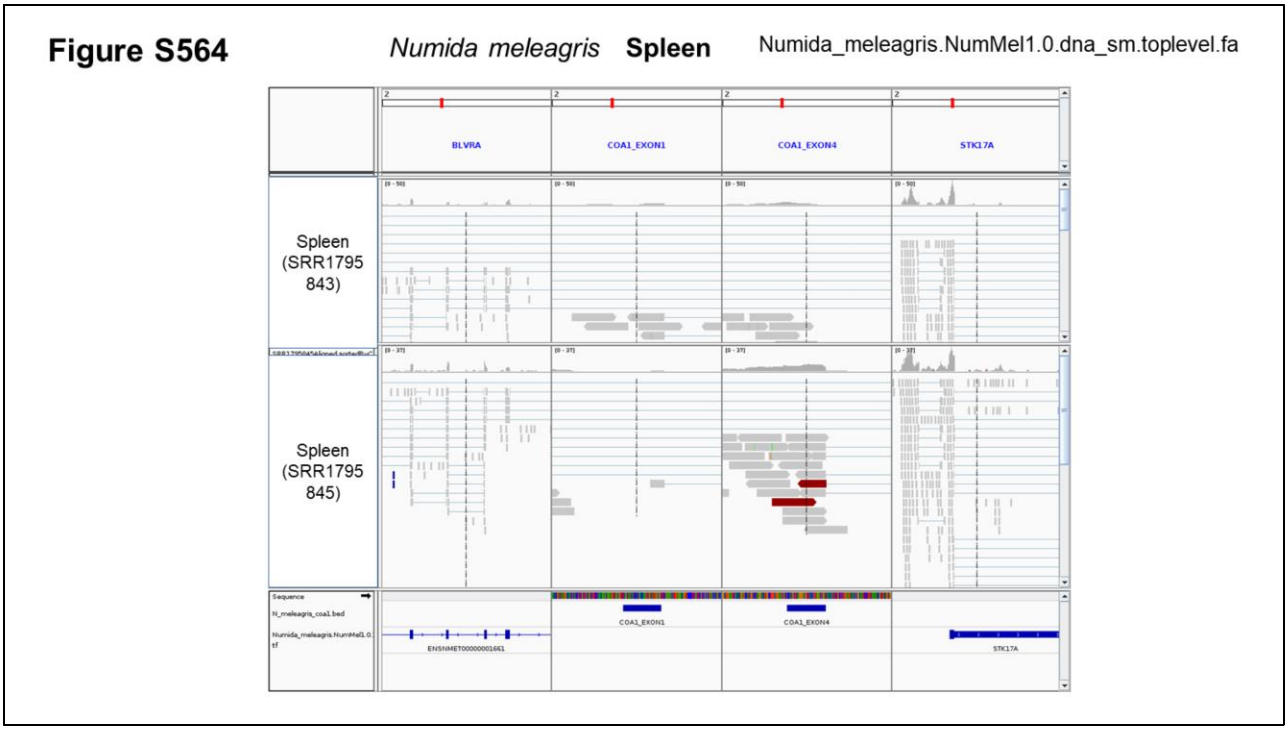

Figure S564: Screenshot of RNA-seq reads aligned to *BLVRA* and *STK17A* to *COA1* exon 1 and 4 respectively in helmeted guineafowl (*Numida meleagris*). Gray boxes represent reads aligned to the genome and thin lines connecting these boxes represent the spliced read alignments. Datasets from spleen (SRR1795843) and (SRR1795845) have been mapped to the Numida_meleagris.NumMel1.0.dna_sm.toplevel.fa genome. Locations of each of the exons are depicted as bed record in the bottom row.

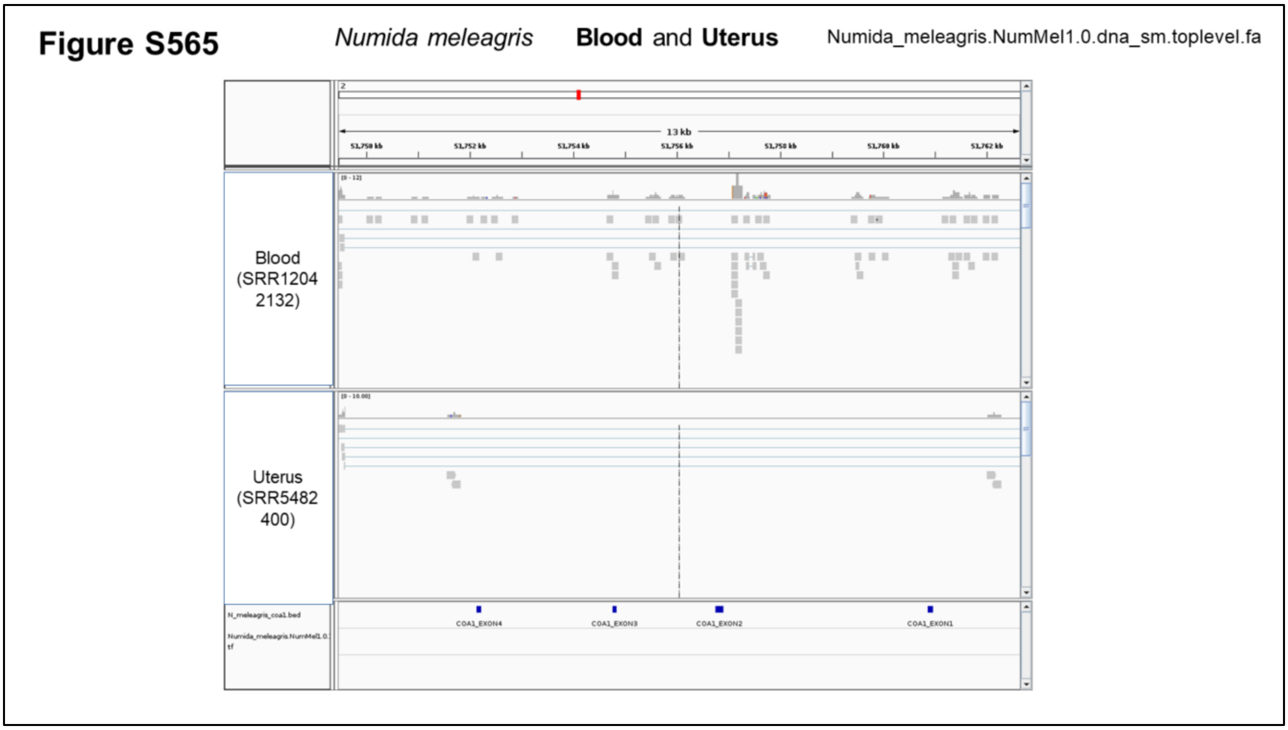

Figure S565: Screenshot of RNA-seq reads aligned to each of the putative locations of the four *COA1* exons in helmeted guineafowl (*Numida meleagris*). Gray boxes represent reads aligned to the genome and thin lines connecting these boxes represent the spliced read alignments. Datasets from blood (SRR12042132) and uterus (SRR5482400) have been mapped to the Numida_meleagris.NumMel1.0.dna_sm.toplevel.fa genome. Locations of each of the exons are depicted as bed record in the bottom row.

Figure S566 *Numida meleagris* **Blood** and **Uterus** Numida_meleagris.NumMel1.0.dna_sm.toplevel.fa

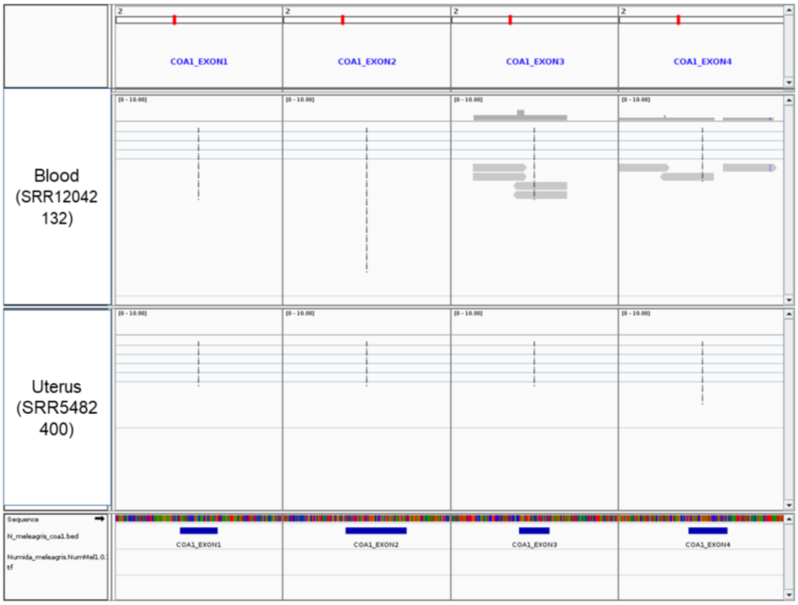

Figure S566: A zoomed in view of each of the *COA1* exons shown in **Supplementary Fig. S565**.

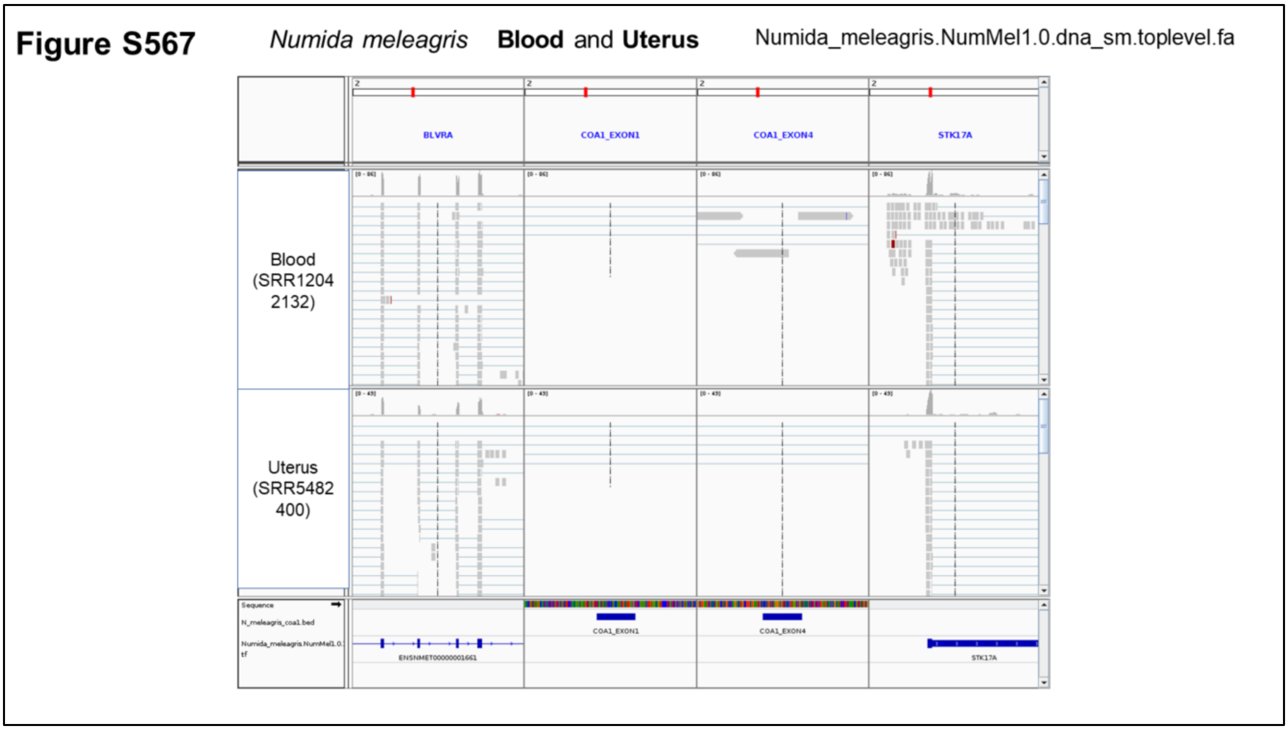

Figure S567: Screenshot of RNA-seq reads aligned to *BLVRA* and *STK17A* to *COA1* exon 1 and 4 respectively in helmeted guineafowl (*Numida meleagris*). Gray boxes represent reads aligned to the genome and thin lines connecting these boxes represent the spliced read alignments. Datasets from blood (SRR12042132) and uterus (SRR5482400) have been mapped to the Numida_meleagris.NumMel1.0.dna_sm.toplevel.fa genome. Locations of each of the exons are depicted as bed record in the bottom row.

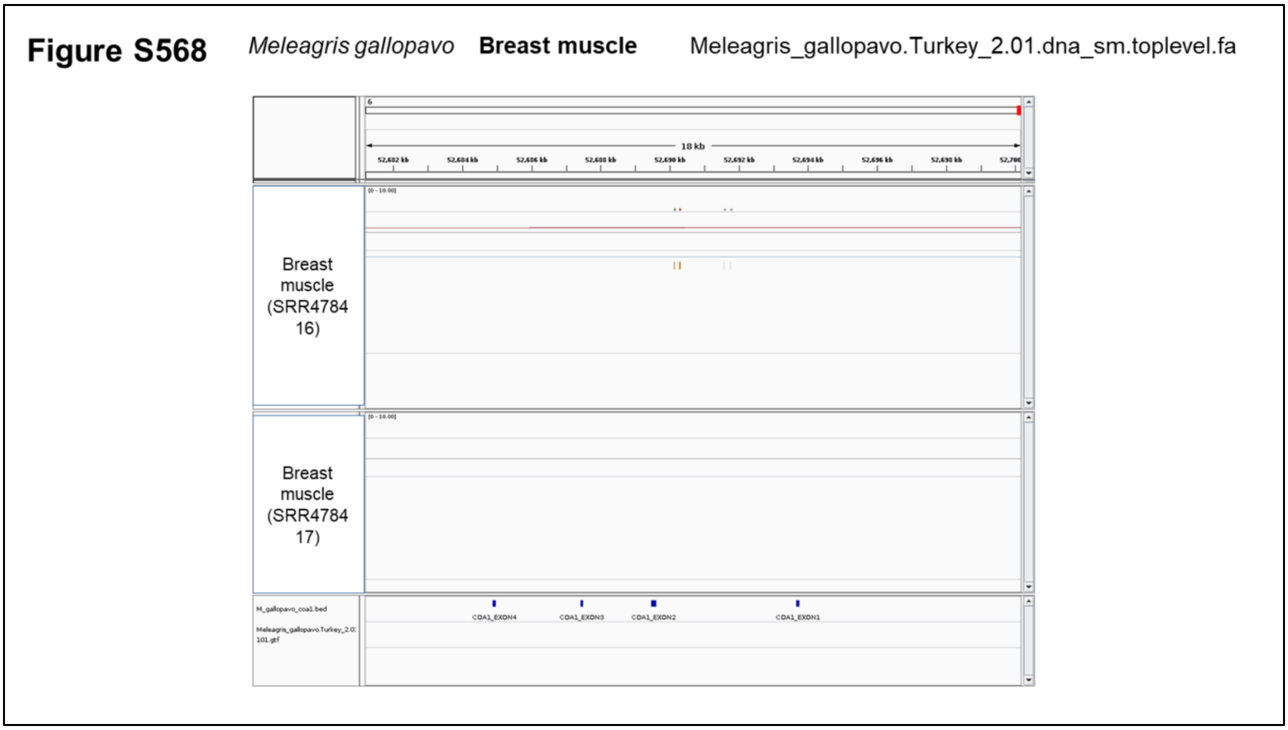

Figure S568: Screenshot of RNA-seq reads aligned to each of the putative locations of the four *COA1* exons in turkey (*Meleagris gallopavo*). Gray boxes represent reads aligned to the genome and thin lines connecting these boxes represent the spliced read alignments. Datasets from breast muscle (SRR478416) and (SRR478417) have been mapped to the Meleagris_gallopavo.Turkey_2.01.dna_sm.toplevel.fa genome. Locations of each of the exons are depicted as bed record in the bottom row.

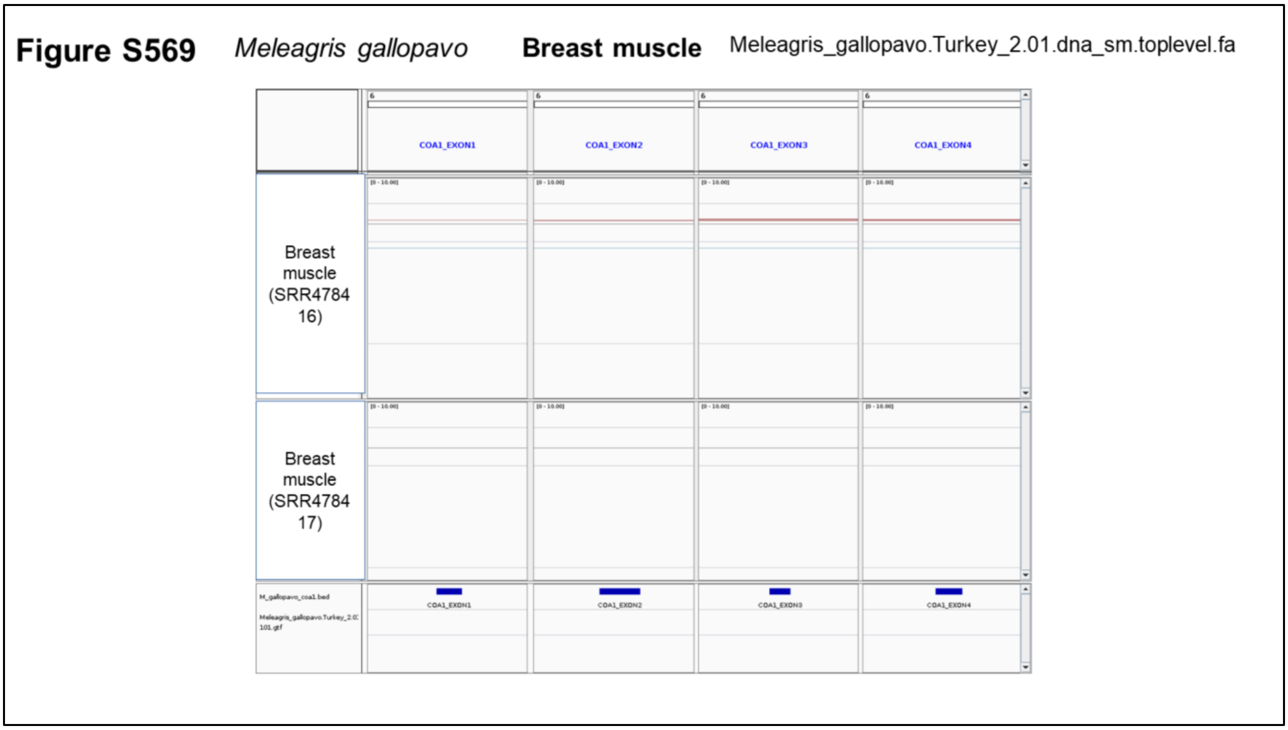

Figure S569: A zoomed in view of each of the *COA1* exons shown in **Supplementary Fig. S568**.

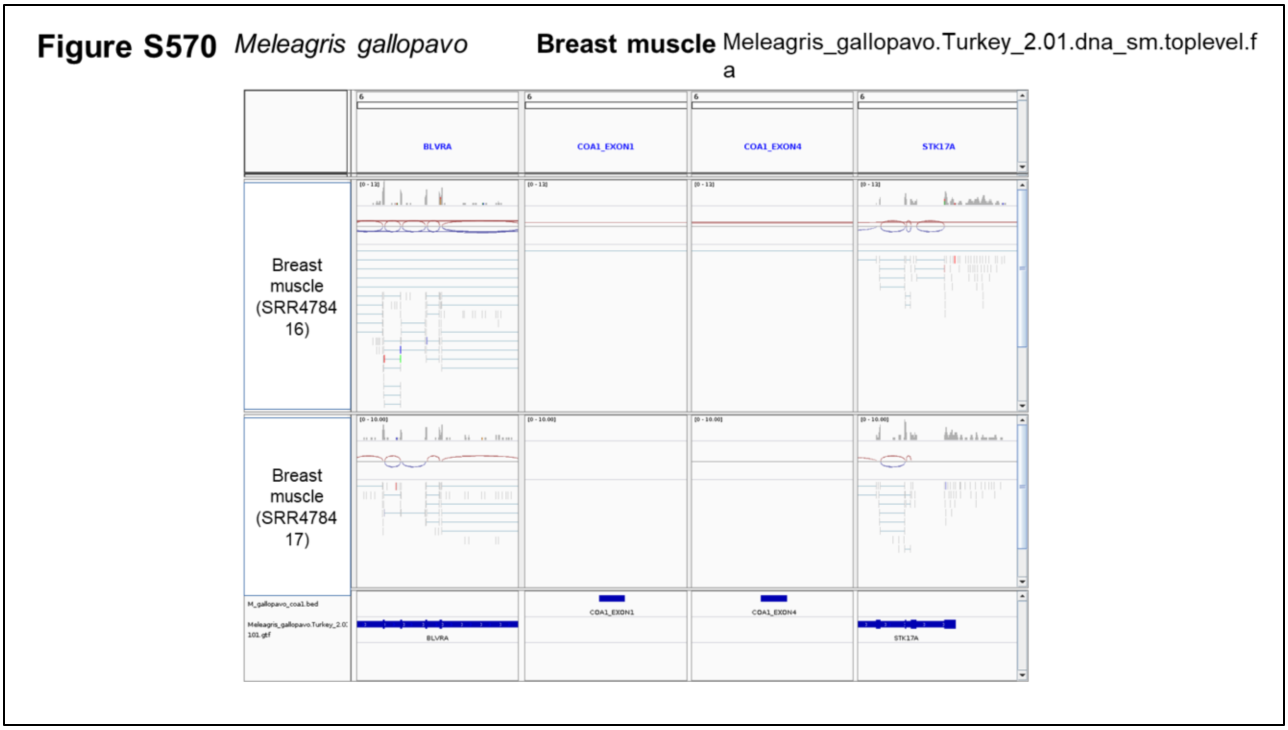

Figure S570: Screenshot of RNA-seq reads aligned to *BLVRA* and *STK17A* to *COA1* exon 1 and 4 respectively in turkey (*Meleagris gallopavo*). Gray boxes represent reads aligned to the genome and thin lines connecting these boxes represent the spliced read alignments. Datasets from breast muscle (SRR478416) and (SRR478417) have been mapped to the Meleagris_gallopavo.Turkey_2.01.dna_sm.toplevel.fa genome. Locations of each of the exons are depicted as bed record in the bottom row.

Figure S571 *Meleagris gallopavo* **Gonad** Meleagris_gallopavo.Turkey_2.01.dna_sm.toplevel.fa

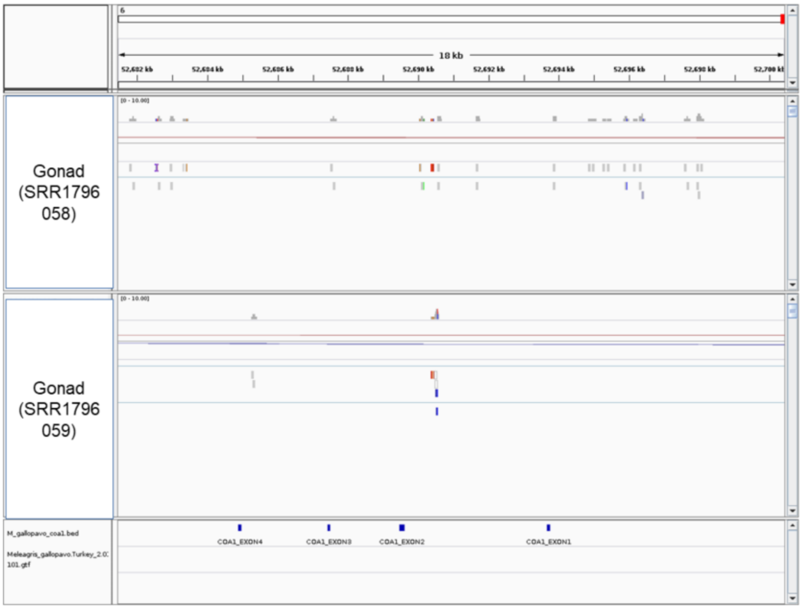

Figure S571: Screenshot of RNA-seq reads aligned to each of the putative locations of the four *COA1* exons in turkey (*Meleagris gallopavo*). Gray boxes represent reads aligned to the genome and thin lines connecting these boxes represent the spliced read alignments. Datasets from gonad (SRR1796058) and (SRR1796059) have been mapped to the Meleagris_gallopavo.Turkey_2.01.dna_sm.toplevel.fa genome. Locations of each of the exons are depicted as bed record in the bottom row.

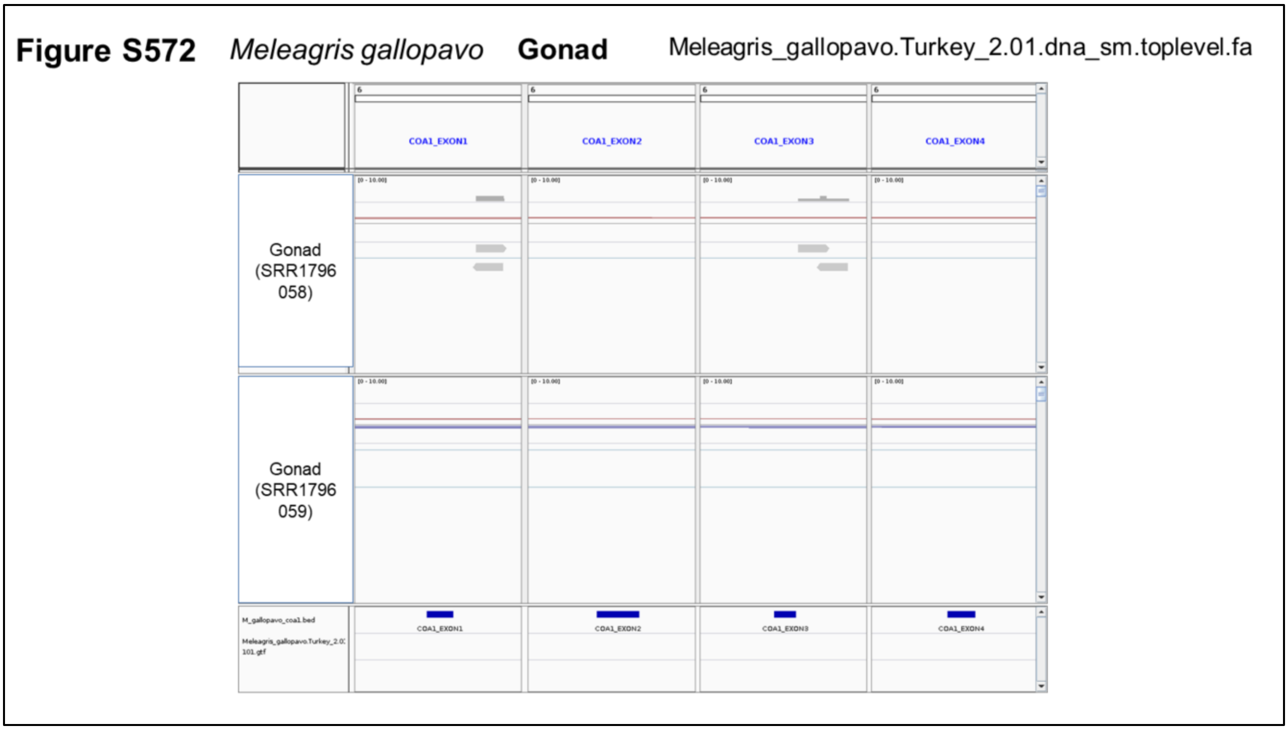

Figure S572: A zoomed in view of each of the *COA1* exons shown in **Supplementary Fig. S571**.

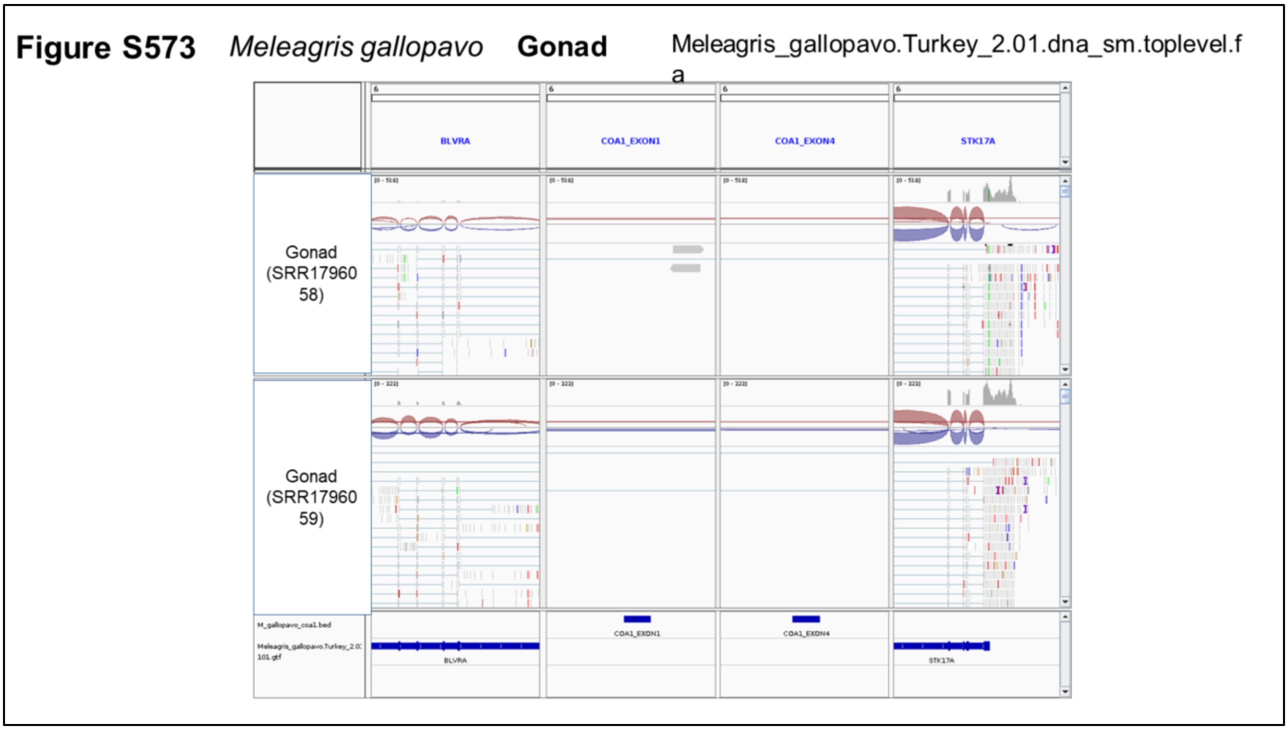

Figure S573: Screenshot of RNA-seq reads aligned to *BLVRA* and *STK17A* to *COA1* exon 1 and 4 respectively in turkey (*Meleagris gallopavo*). Gray boxes represent reads aligned to the genome and thin lines connecting these boxes represent the spliced read alignments. Datasets from gonad (SRR1796058) and (SRR1796059) have been mapped to the Meleagris_gallopavo.Turkey_2.01.dna_sm.toplevel.fa genome. Locations of each of the exons are depicted as bed record in the bottom row.

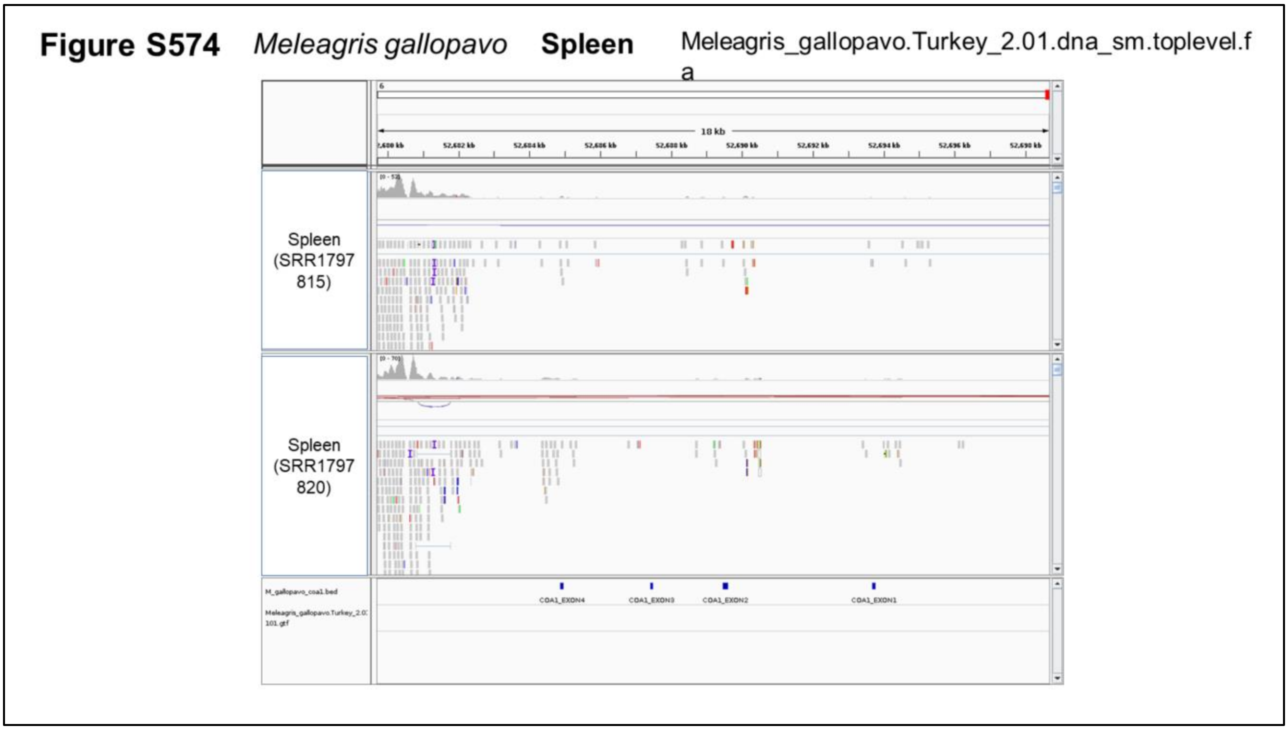

Figure S574: Screenshot of RNA-seq reads aligned to each of the putative locations of the four *COA1* exons in turkey (*Meleagris gallopavo*). Gray boxes represent reads aligned to the genome and thin lines connecting these boxes represent the spliced read alignments. Datasets from spleen (SRR1797815) and (SRR1797820) have been mapped to the Meleagris_gallopavo.Turkey_2.01.dna_sm.toplevel.fa genome. Locations of each of the exons are depicted as bed record in the bottom row.

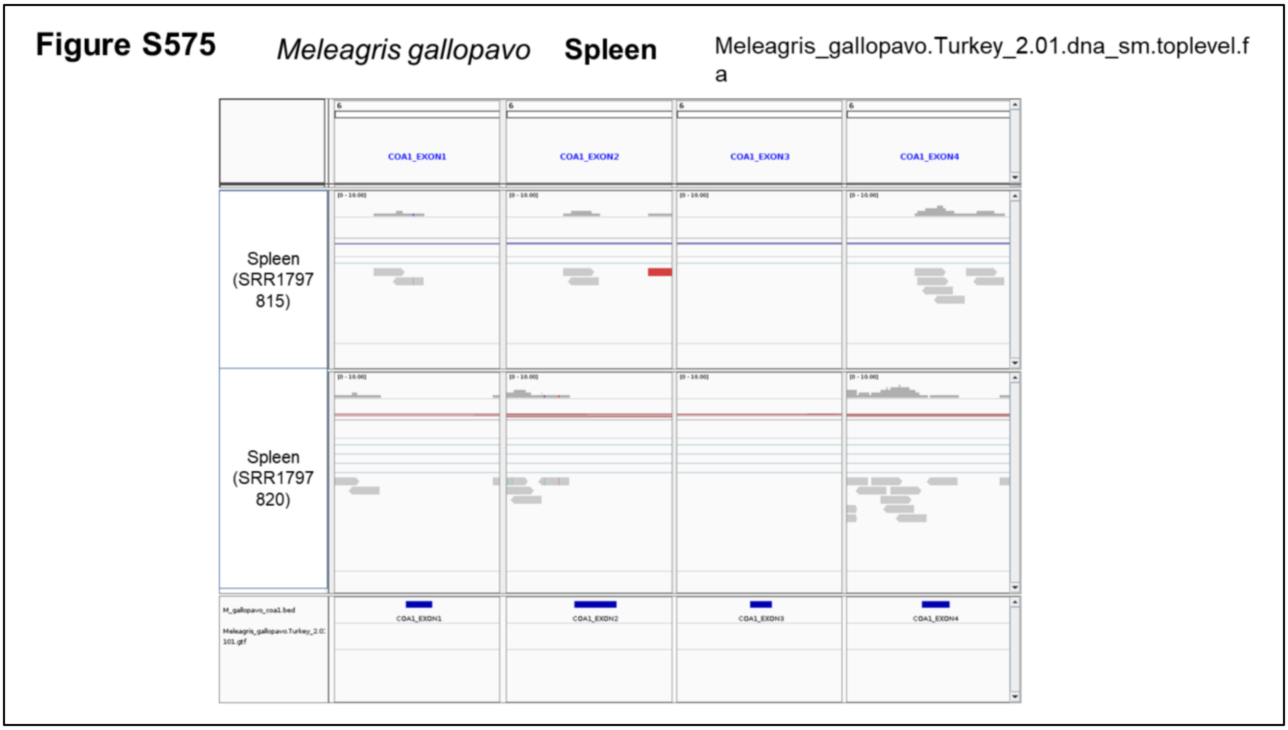

Figure S575: A zoomed in view of each of the *COA1* exons shown in **Supplementary Fig. S574**.

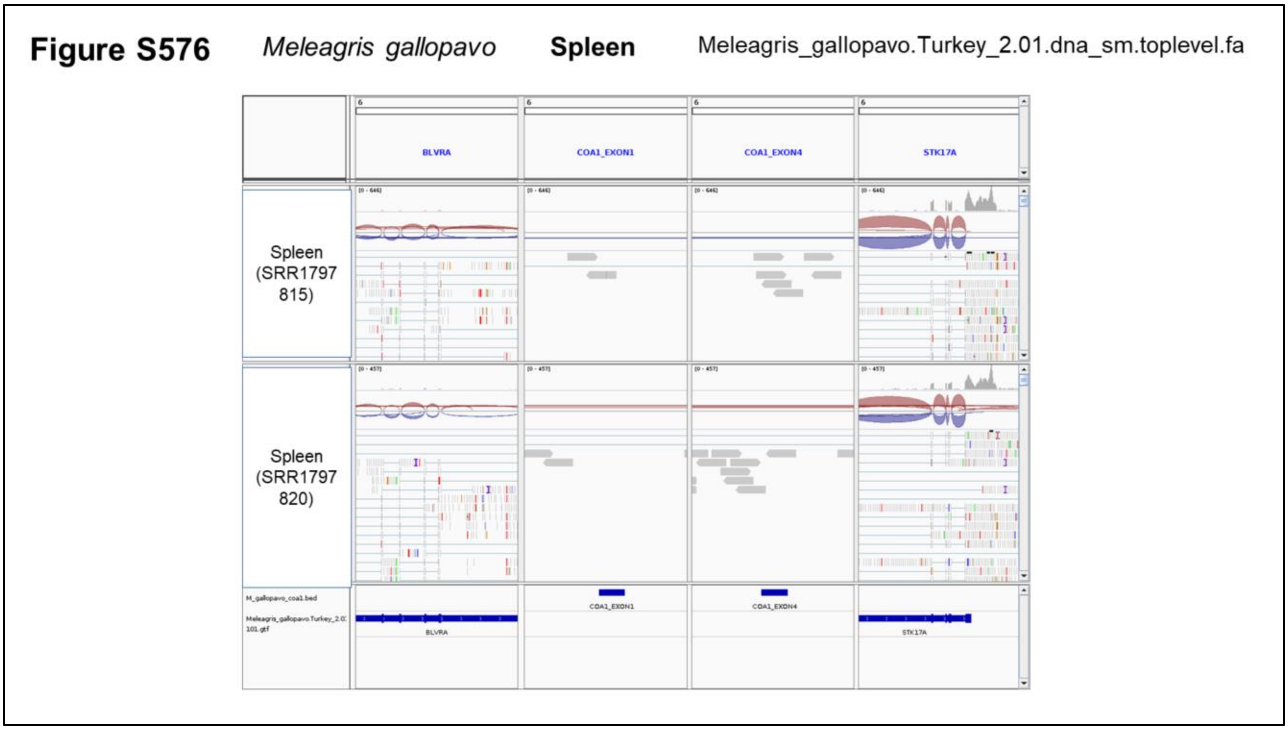

Figure S576: Screenshot of RNA-seq reads aligned to *BLVRA* and *STK17A* to *COA1* exon 1 and 4 respectively in turkey (*Meleagris gallopavo*). Gray boxes represent reads aligned to the genome and thin lines connecting these boxes represent the spliced read alignments. Datasets from spleen (SRR1797815) and (SRR1797820) have been mapped to the Meleagris_gallopavo.Turkey_2.01.dna_sm.toplevel.fa genome. Locations of each of the exons are depicted as bed record in the bottom row.

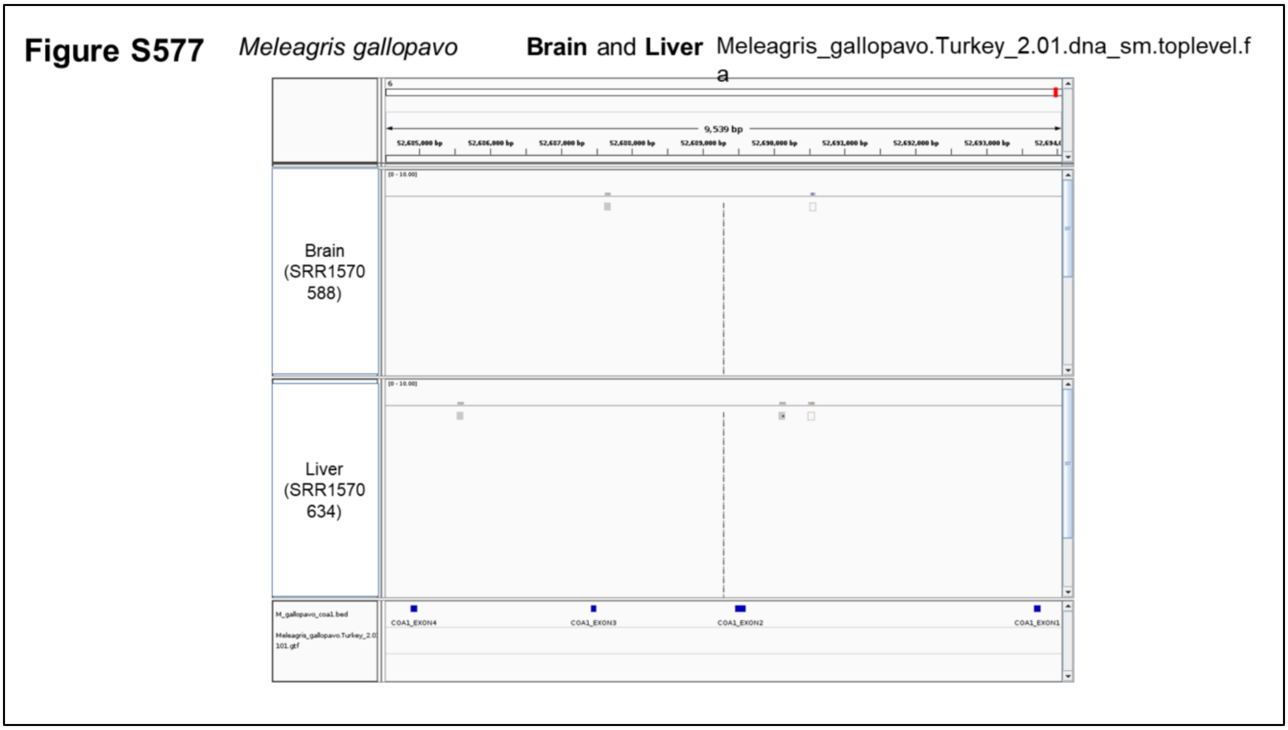

Figure S577: Screenshot of RNA-seq reads aligned to each of the putative locations of the four *COA1* exons in turkey (*Meleagris gallopavo*). Gray boxes represent reads aligned to the genome and thin lines connecting these boxes represent the spliced read alignments. Datasets from Brain (SRR1570588) and liver (SRR1570634) have been mapped to the Meleagris_gallopavo.Turkey_2.01.dna_sm.toplevel.fa genome. Locations of each of the exons are depicted as bed record in the bottom row.

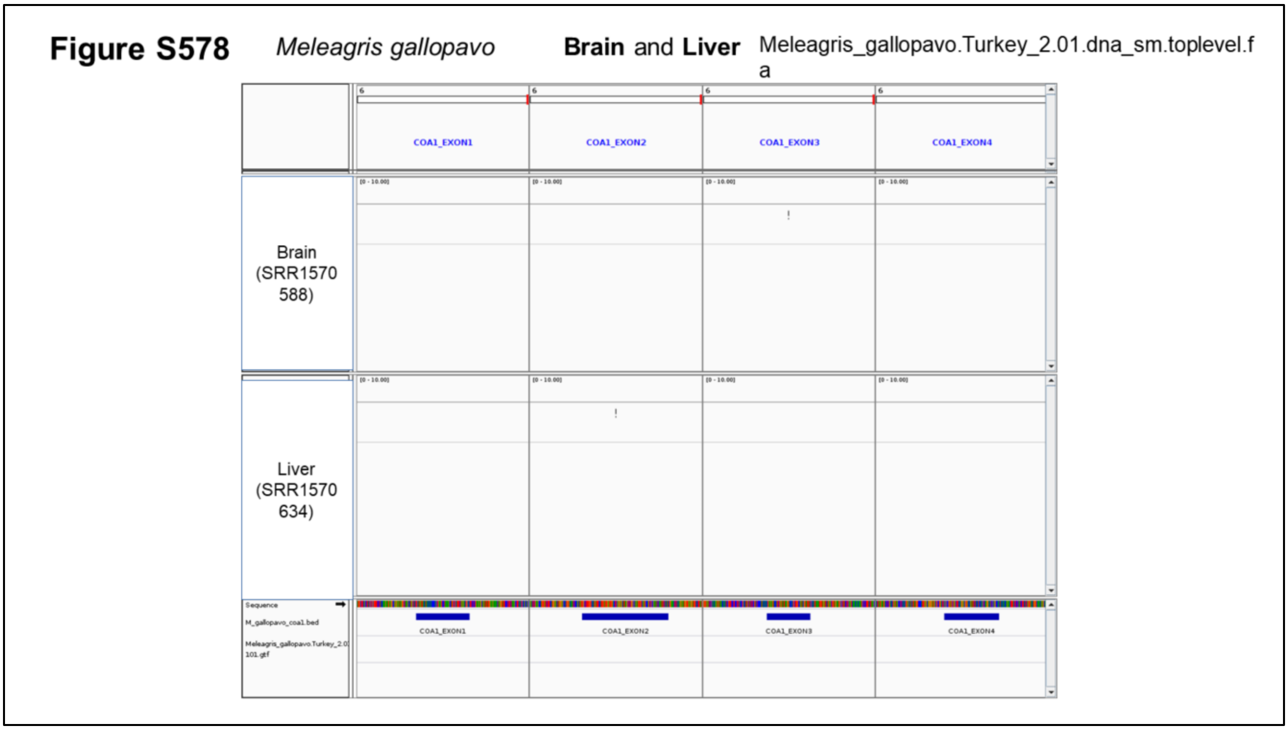

Figure S578: A zoomed in view of each of the *COA1* exons shown in **Supplementary Fig. S577**.

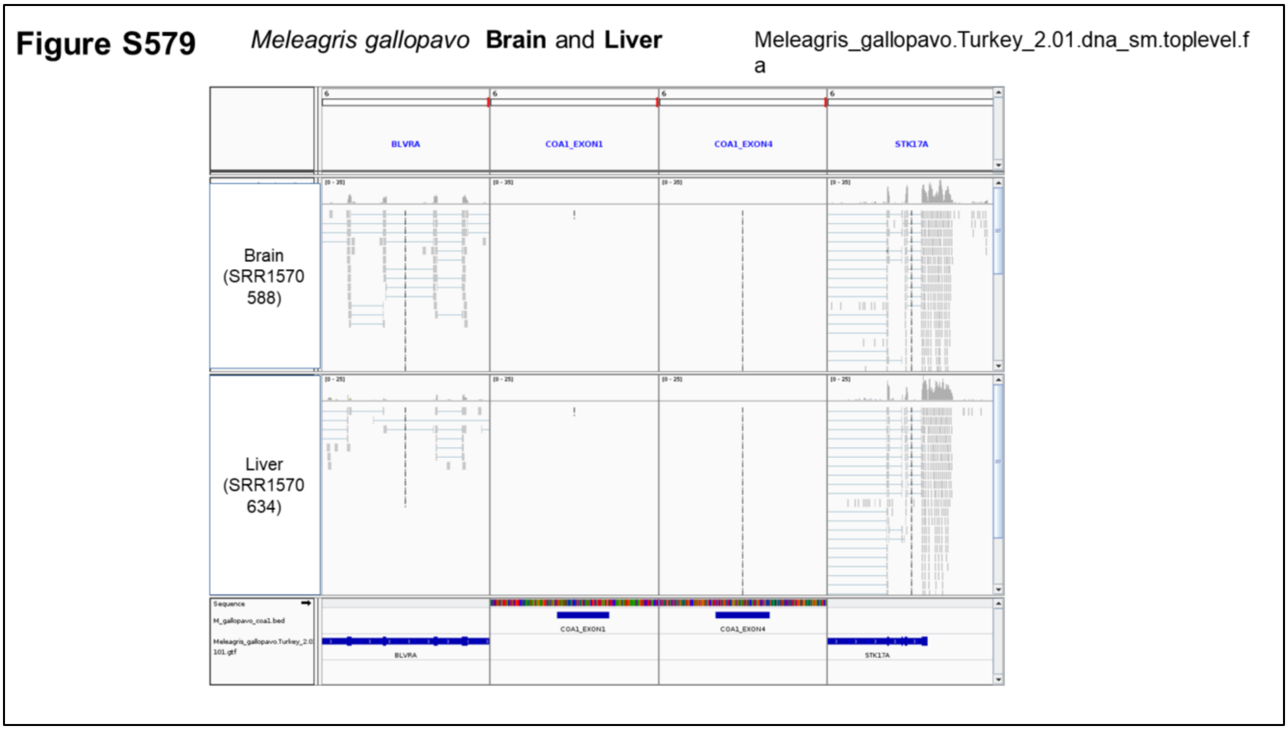

Figure S579: Screenshot of RNA-seq reads aligned to *BLVRA* and *STK17A* to *COA1* exon 1 and 4 respectively in turkey (*Meleagris gallopavo*). Gray boxes represent reads aligned to the genome and thin lines connecting these boxes represent the spliced read alignments. Datasets from Brain (SRR1570588) and liver (SRR1570634) have been mapped to the Meleagris_gallopavo.Turkey_2.01.dna_sm.toplevel.fa genome. Locations of each of the exons are depicted as bed record in the bottom row.

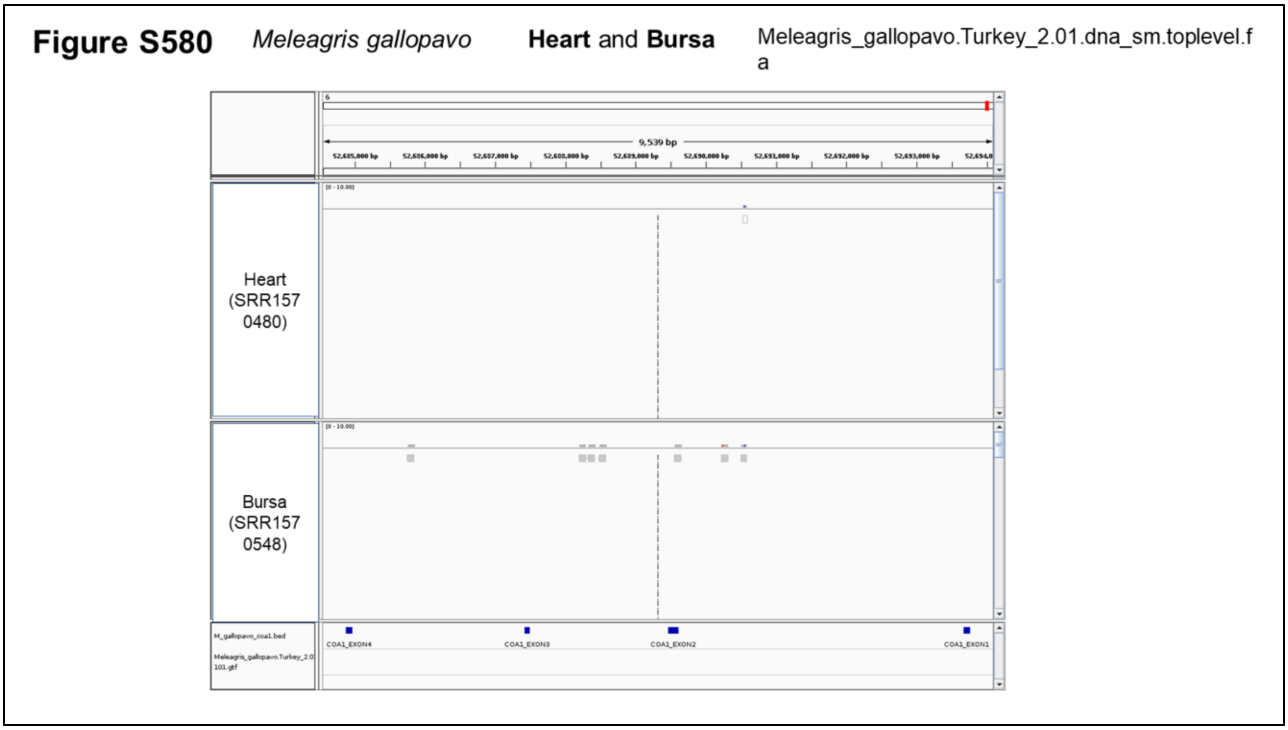

Figure S580: Screenshot of RNA-seq reads aligned to each of the putative locations of the four *COA1* exons in turkey (*Meleagris gallopavo*). Gray boxes represent reads aligned to the genome and thin lines connecting these boxes represent the spliced read alignments. Datasets from heart (SRR1570480) and bursa (SRR1570548) have been mapped to the Meleagris_gallopavo.Turkey_2.01.dna_sm.toplevel.fa genome. Locations of each of the exons are depicted as bed record in the bottom row.

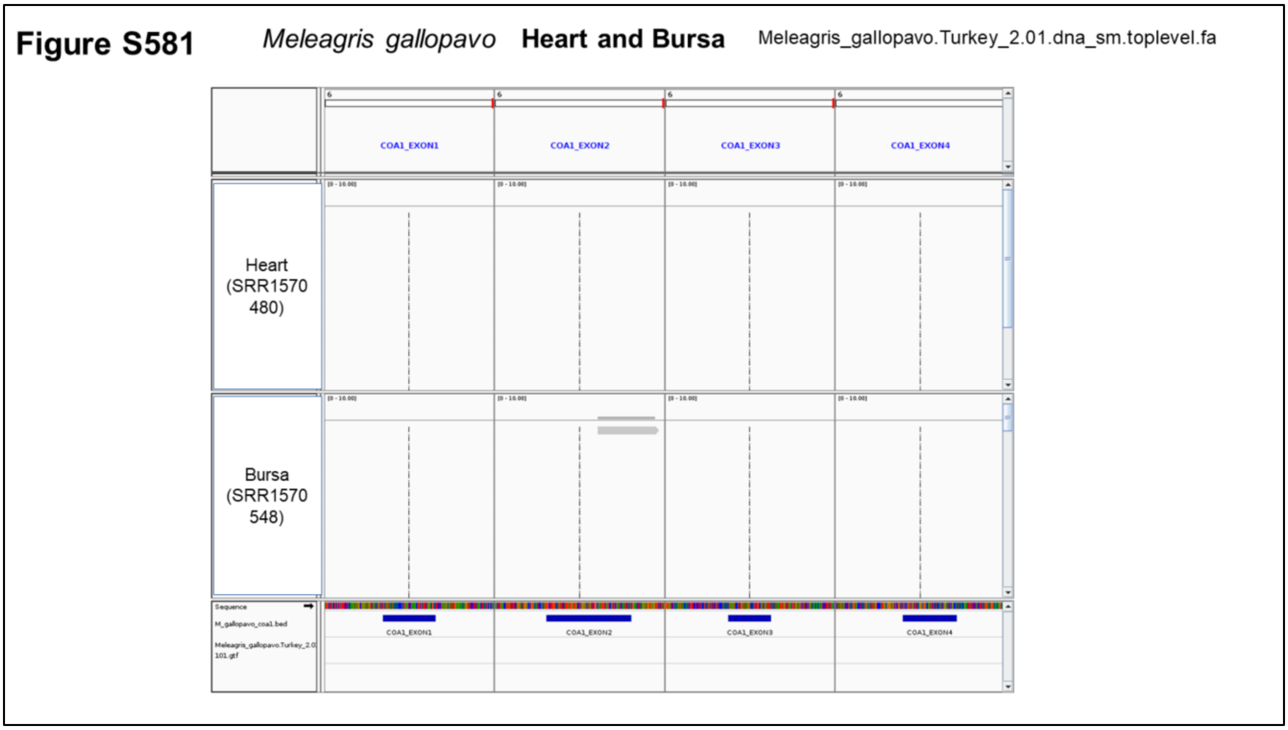

Figure S581: A zoomed in view of each of the *COA1* exons shown in **Supplementary Fig. S580**.

Figure S582 *Meleagris gallopavo* **Heart** and **Bursa** Meleagris_gallopavo.Turkey_2.01.dna_sm.toplevel.fa

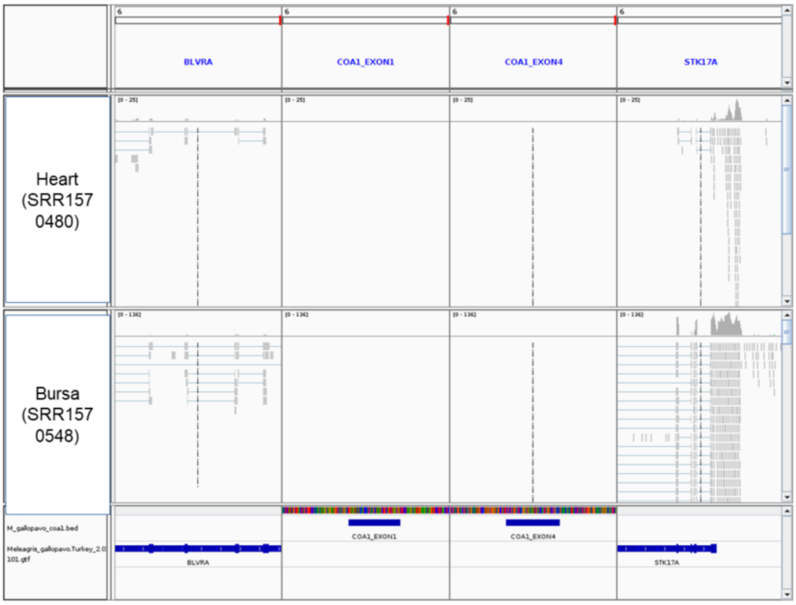

Figure S582: Screenshot of RNA-seq reads aligned to *BLVRA* and *STK17A* to *COA1* exon 1 and 4 respectively in turkey (*Meleagris gallopavo*). Gray boxes represent reads aligned to the genome and thin lines connecting these boxes represent the spliced read alignments. Datasets from heart (SRR1570480) and bursa (SRR1570548) have been mapped to the Meleagris_gallopavo.Turkey_2.01.dna_sm.toplevel.fa genome. Locations of each of the exons are depicted as bed record in the bottom row.

Figure S583 *Coturnix japonica* **Kidney** Coturnix_japonica_2.0.dna_sm.toplevel.fa

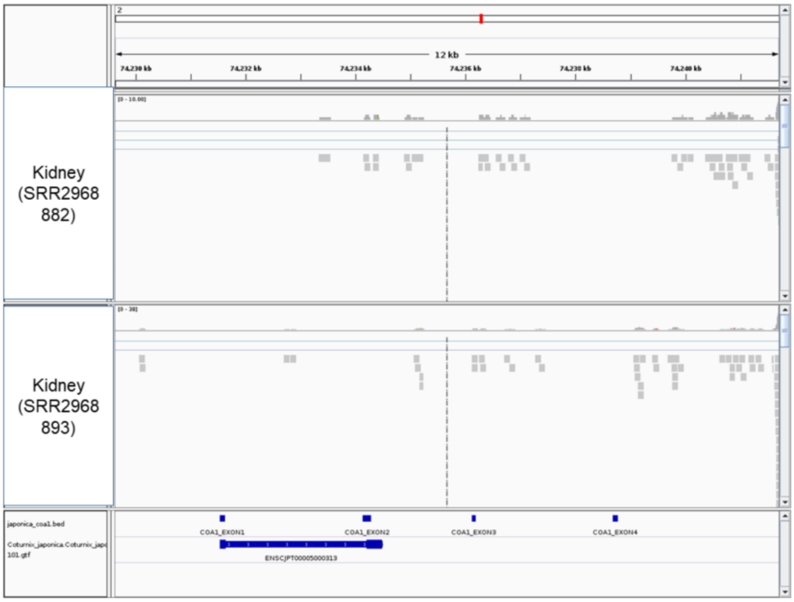

Figure S583: Screenshot of RNA-seq reads aligned to each of the putative locations of the four *COA1* exons in Japanese quail (*Coturnix japonica*). Gray boxes represent reads aligned to the genome and thin lines connecting these boxes represent the spliced read alignments. Datasets from kidney (SRR1570480) and (SRR1570548) have been mapped to the Coturnix_japonica_2.0.dna_sm.toplevel.fa genome. Locations of each of the exons are depicted as bed record in the bottom row.

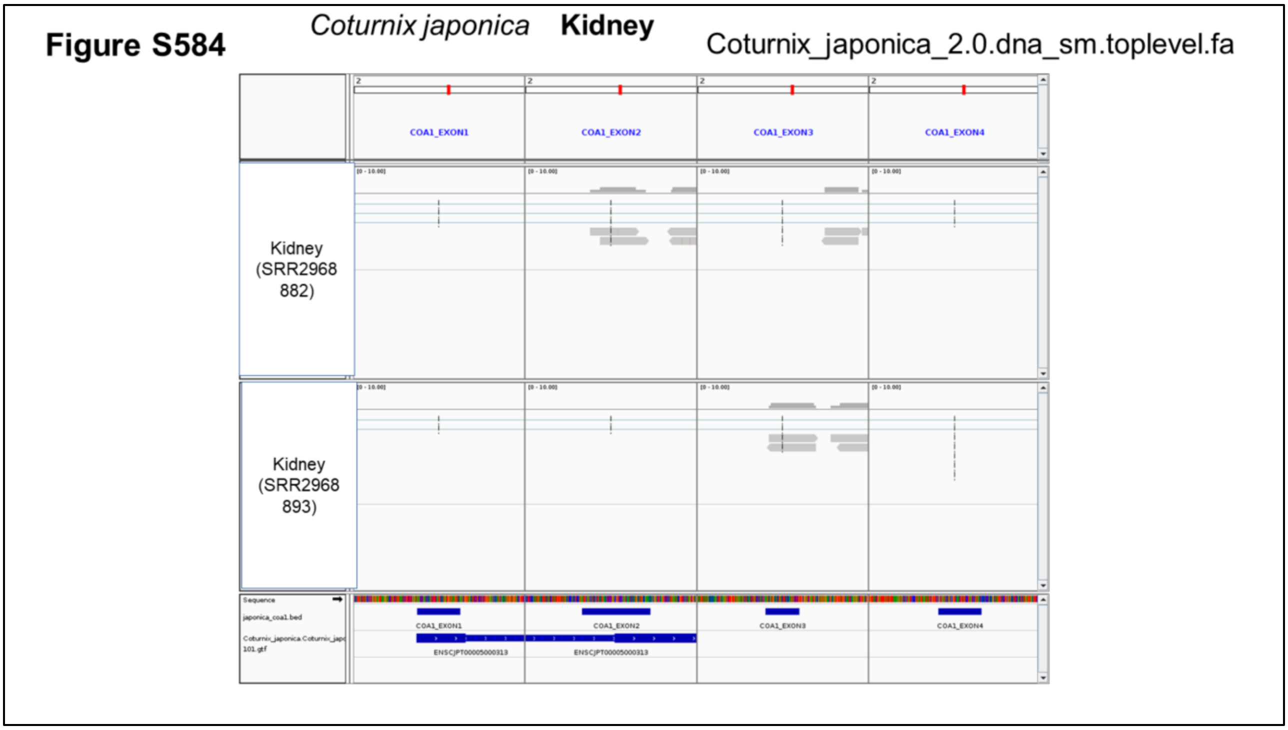

Figure S584: A zoomed in view of each of the *COA1* exons shown in **Supplementary Fig. S583**.

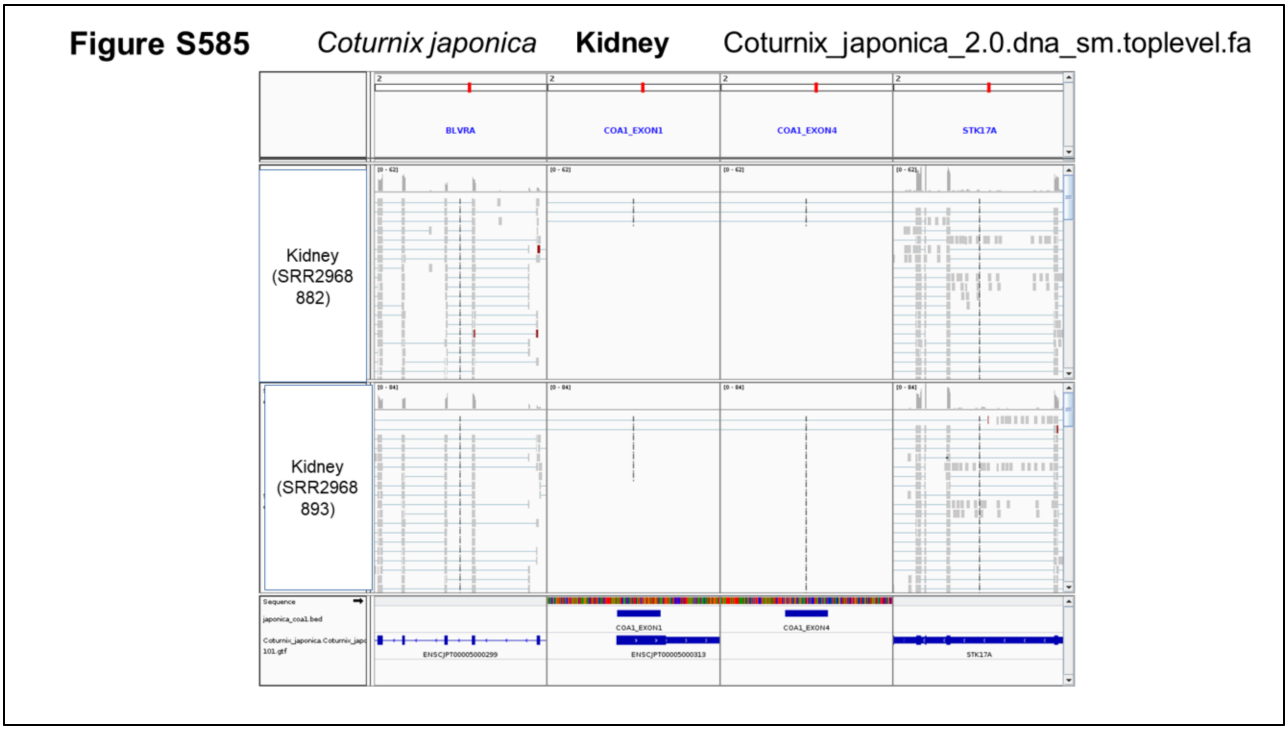

Figure S585: Screenshot of RNA-seq reads aligned to *BLVRA* and *STK17A* to *COA1* exon 1 and 4 respectively in Japanese quail (*Coturnix japonica*). Gray boxes represent reads aligned to the genome and thin lines connecting these boxes represent the spliced read alignments. Datasets from kidney (SRR1570480) and (SRR1570548) have been mapped to the Coturnix_japonica_2.0.dna_sm.toplevel.fa genome. Locations of each of the exons are depicted as bed record in the bottom row.

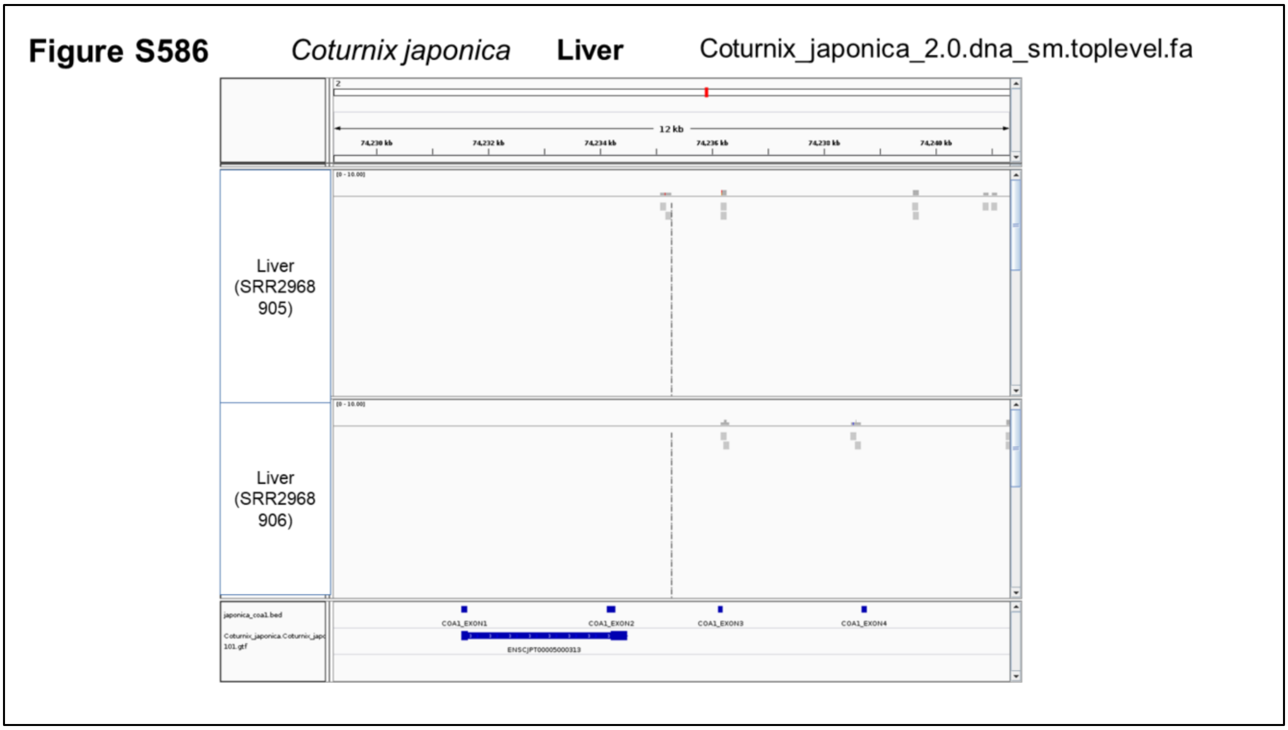

Figure S586: Screenshot of RNA-seq reads aligned to each of the putative locations of the four *COA1* exons in Japanese quail (*Coturnix japonica*). Gray boxes represent reads aligned to the genome and thin lines connecting these boxes represent the spliced read alignments. Datasets from liver (SRR2968905) and (SRR2968906) have been mapped to the Coturnix_japonica_2.0.dna_sm.toplevel.fa genome. Locations of each of the exons are depicted as bed record in the bottom row.

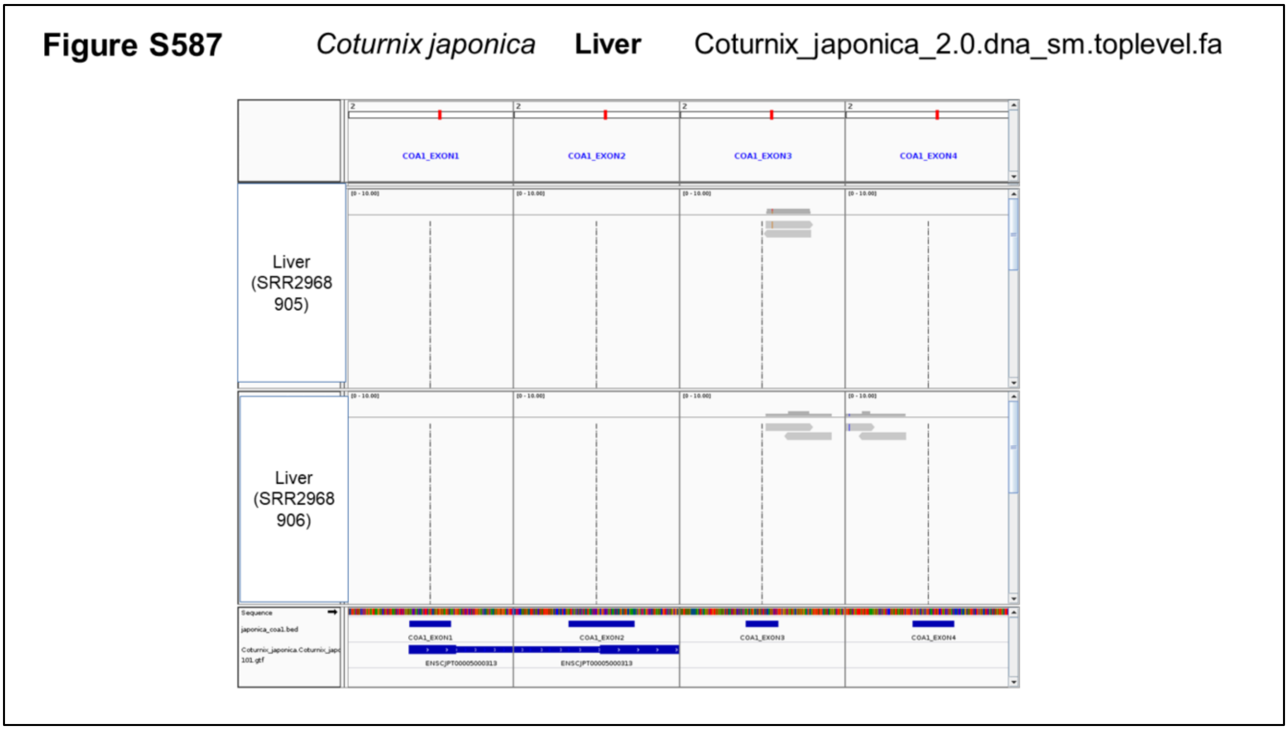

Figure S587: A zoomed in view of each of the *COA1* exons shown in **Supplementary Fig. S586**.

Figure S588 *Coturnix japonica* **Liver** Coturnix_japonica_2.0.dna_sm.toplevel.fa

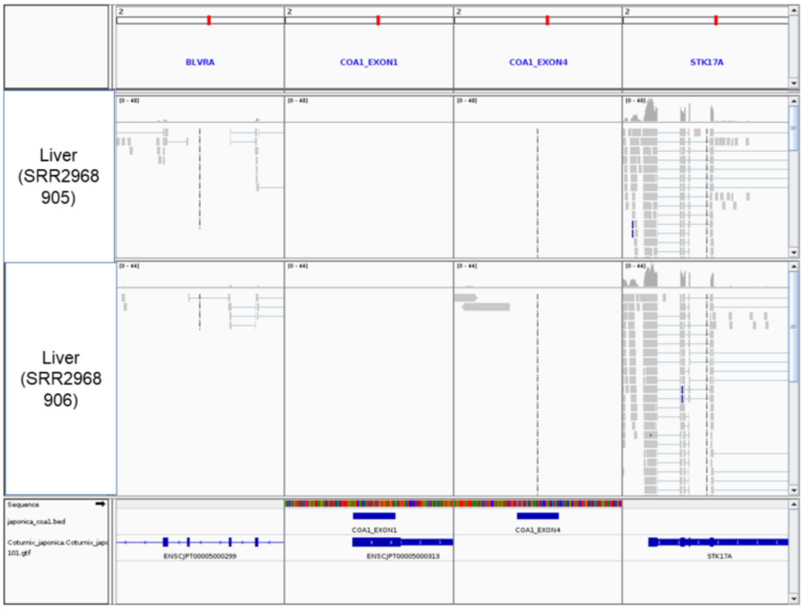

Figure S588: Screenshot of RNA-seq reads aligned to *BLVRA* and *STK17A* to *COA1* exon 1 and 4 respectively in Japanese quail (*Coturnix japonica*). Gray boxes represent reads aligned to the genome and thin lines connecting these boxes represent the spliced read alignments. Datasets from liver (SRR2968905) and (SRR2968906) have been mapped to the Coturnix_japonica_2.0.dna_sm.toplevel.fa genome. Locations of each of the exons are depicted as bed record in the bottom row.

Figure S589 *Coturnix japonica* **Muscle** Coturnix_japonica_2.0.dna_sm.toplevel.fa

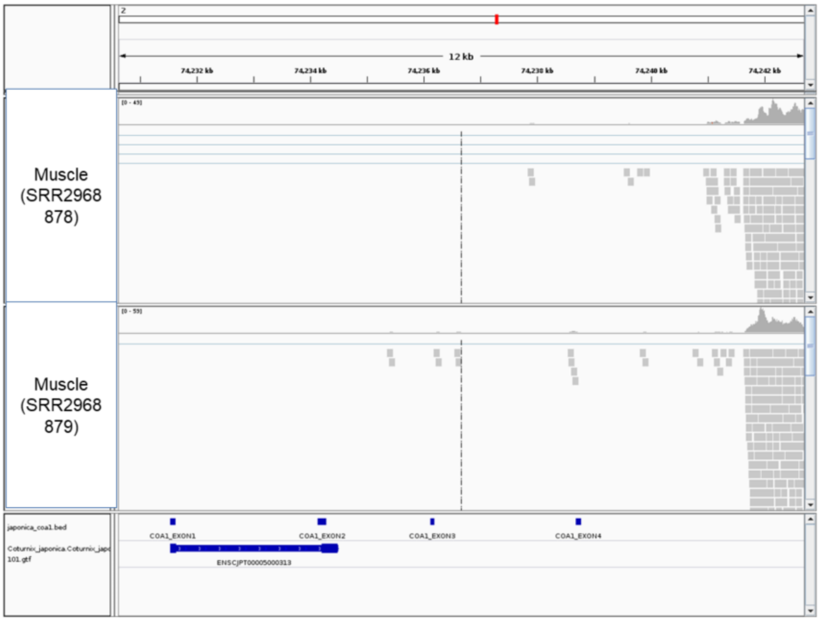

Figure S589: Screenshot of RNA-seq reads aligned to each of the putative locations of the four *COA1* exons in Japanese quail (*Coturnix japonica*). Gray boxes represent reads aligned to the genome and thin lines connecting these boxes represent the spliced read alignments. Datasets from muscle (SRR2968878) and (SRR2968879) have been mapped to the Coturnix_japonica_2.0.dna_sm.toplevel.fa genome. Locations of each of the exons are depicted as bed record in the bottom row.

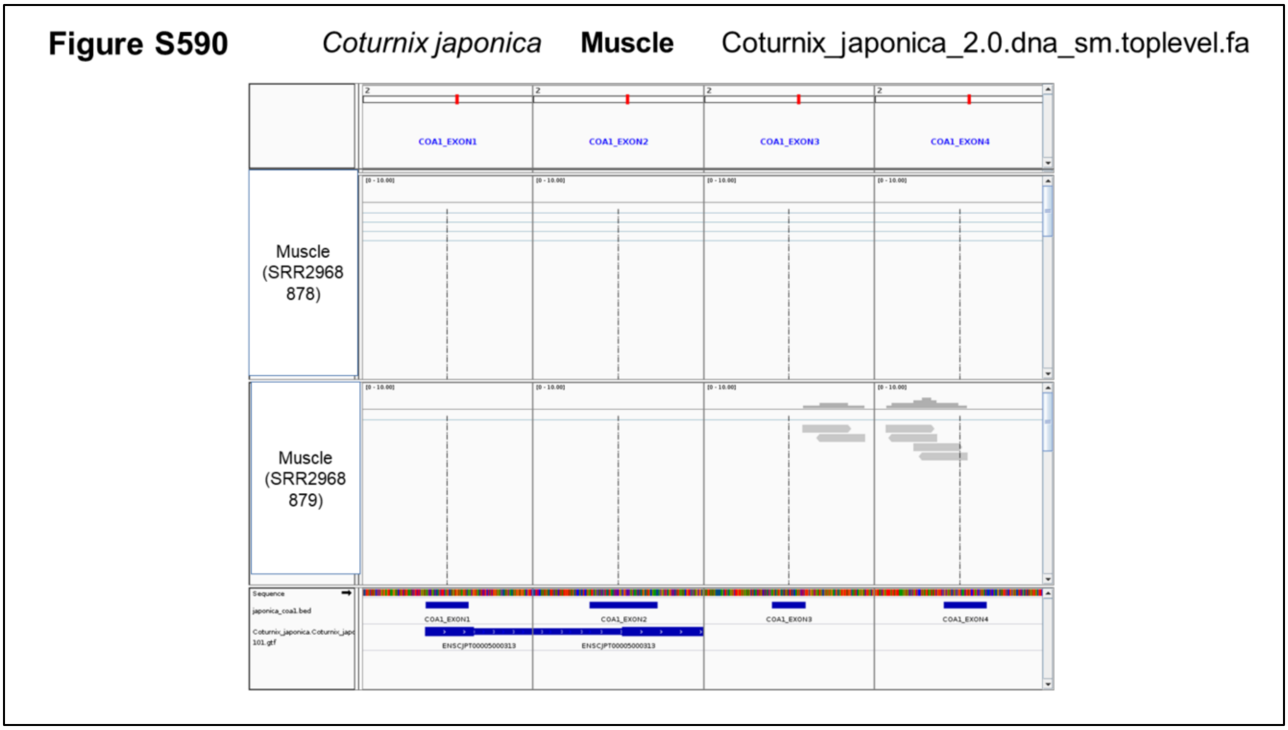

Figure S590: A zoomed in view of each of the *COA1* exons shown in **Supplementary Fig. S589**.

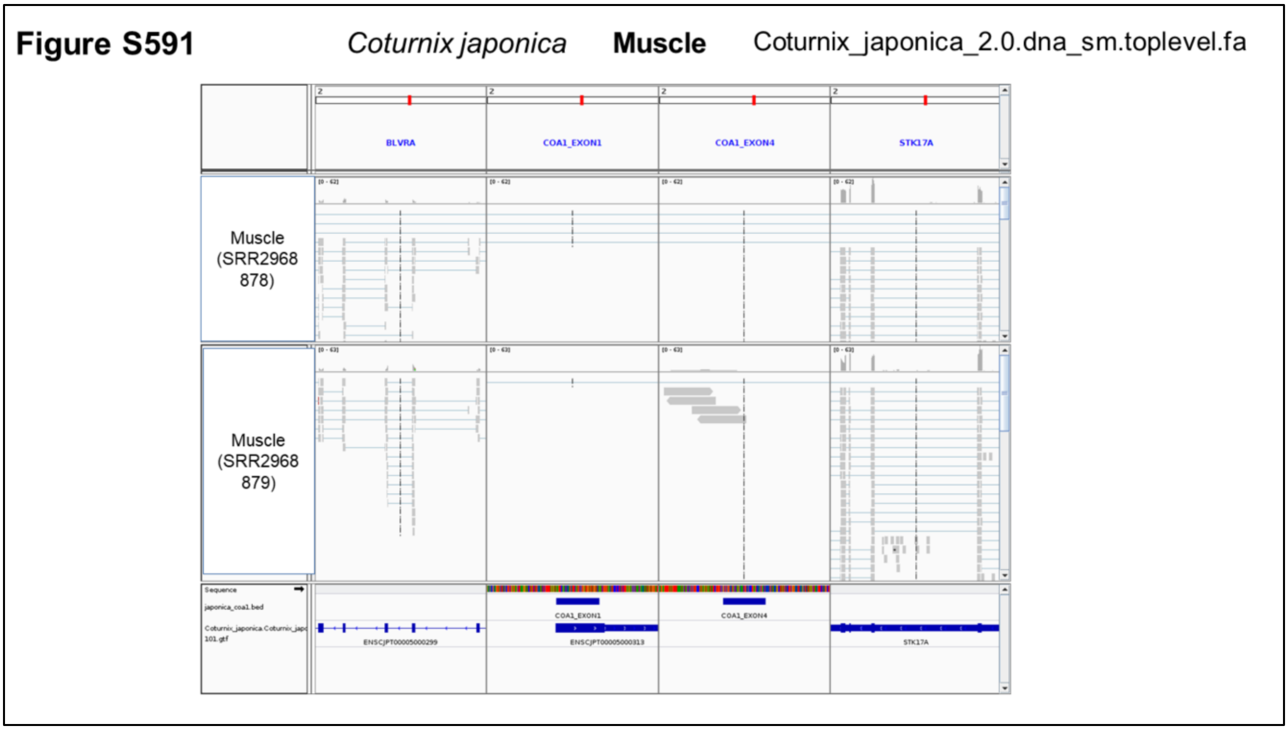

Figure S591: Screenshot of RNA-seq reads aligned to *BLVRA* and *STK17A* to *COA1* exon 1 and 4 respectively in Japanese quail (*Coturnix japonica*). Gray boxes represent reads aligned to the genome and thin lines connecting these boxes represent the spliced read alignments. Datasets from muscle (SRR2968878) and (SRR2968879) have been mapped to the Coturnix_japonica_2.0.dna_sm.toplevel.fa genome. Locations of each of the exons are depicted as bed record in the bottom row.

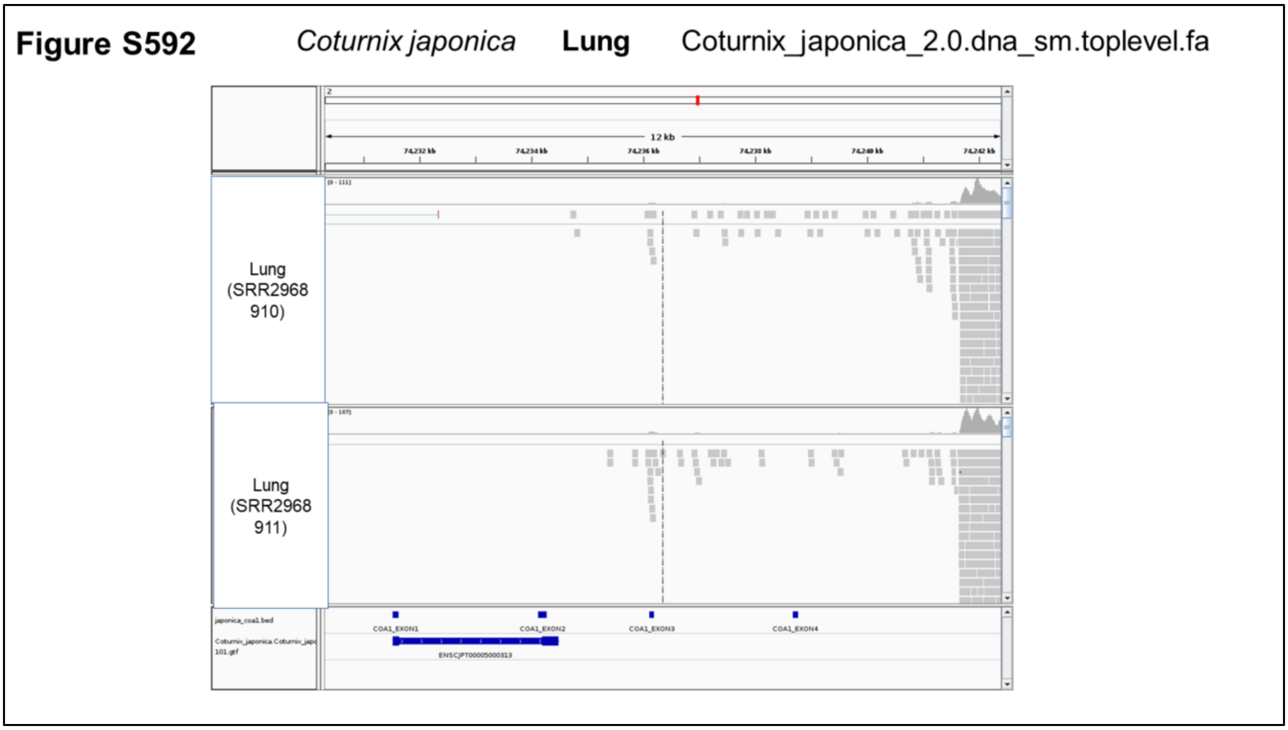

Figure S592: Screenshot of RNA-seq reads aligned to each of the putative locations of the four *COA1* exons in Japanese quail (*Coturnix japonica*). Gray boxes represent reads aligned to the genome and thin lines connecting these boxes represent the spliced read alignments. Datasets from lung (SRR2968910) and (SRR2968911) have been mapped to the Coturnix_japonica_2.0.dna_sm.toplevel.fa genome. Locations of each of the exons are depicted as bed record in the bottom row.

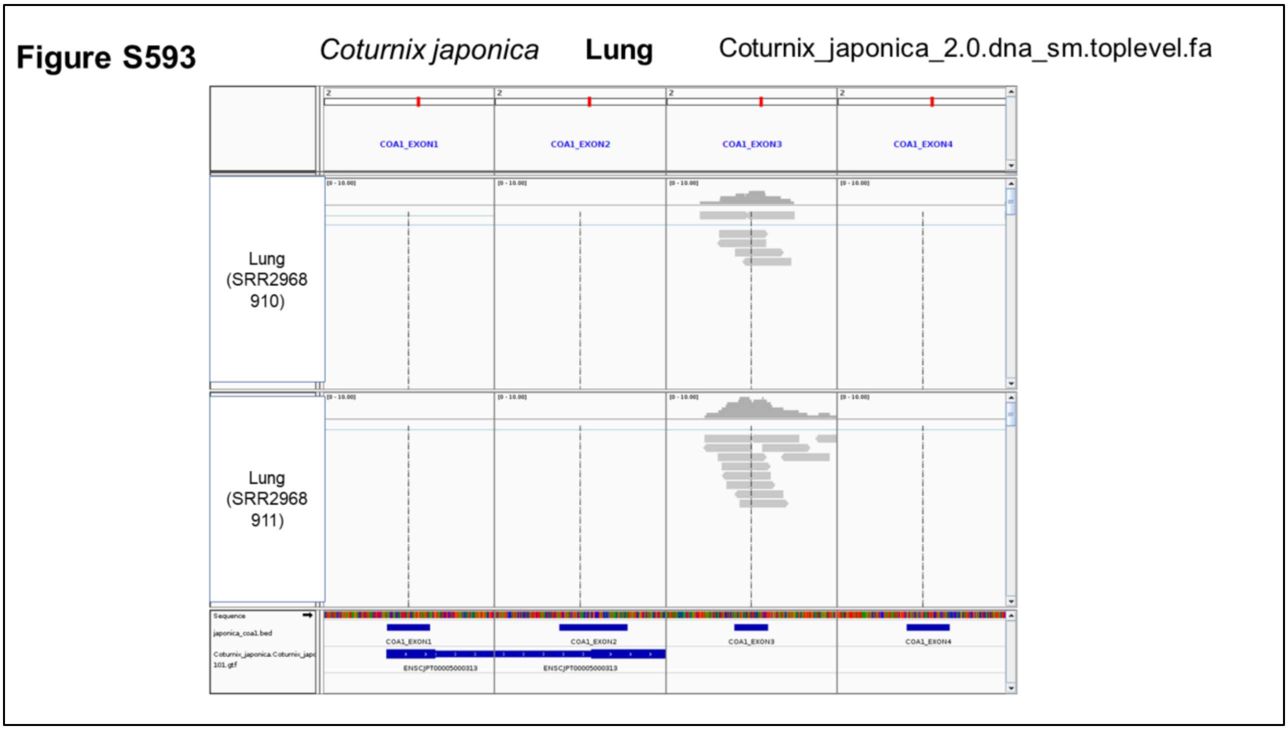

Figure S593: A zoomed in view of each of the *COA1* exons shown in **Supplementary Fig. S592**.

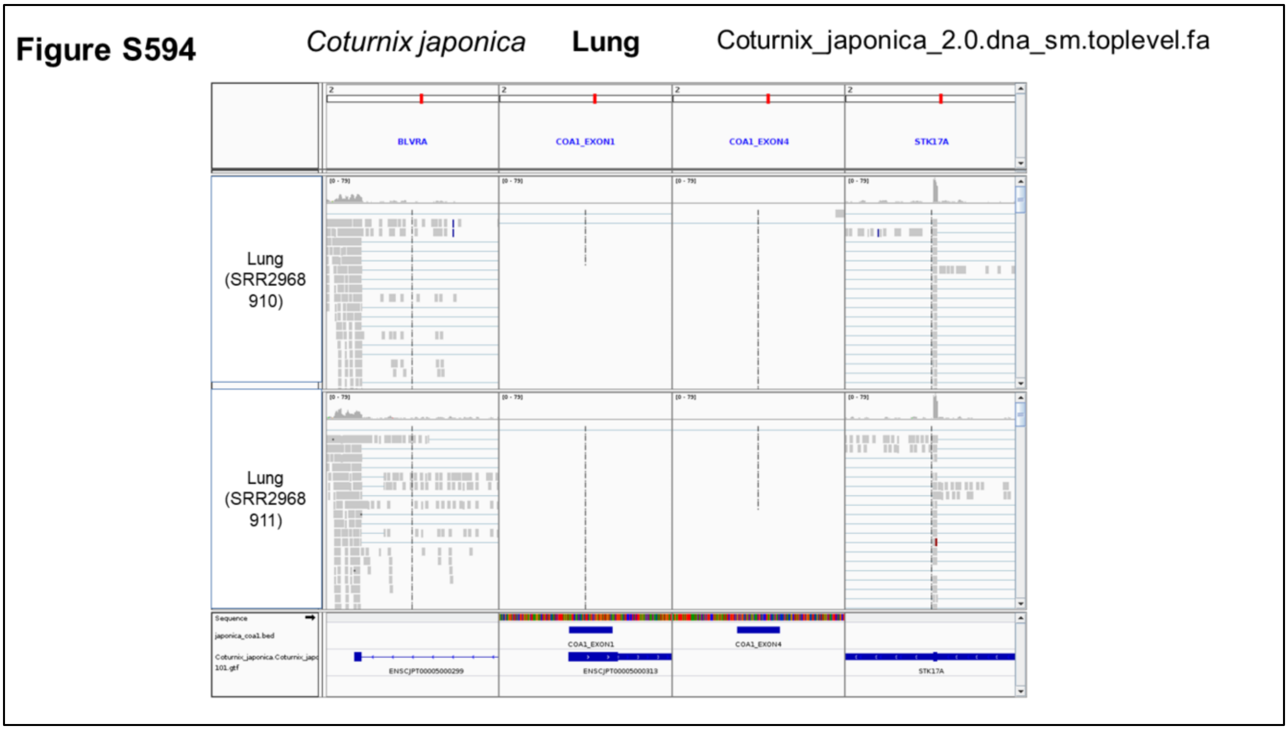

Figure S594: Screenshot of RNA-seq reads aligned to *BLVRA* and *STK17A* to *COA1* exon 1 and 4 respectively in Japanese quail (*Coturnix japonica*). Gray boxes represent reads aligned to the genome and thin lines connecting these boxes represent the spliced read alignments. Datasets from lung (SRR2968910) and (SRR2968911) have been mapped to the Coturnix_japonica_2.0.dna_sm.toplevel.fa genome. Locations of each of the exons are depicted as bed record in the bottom row.

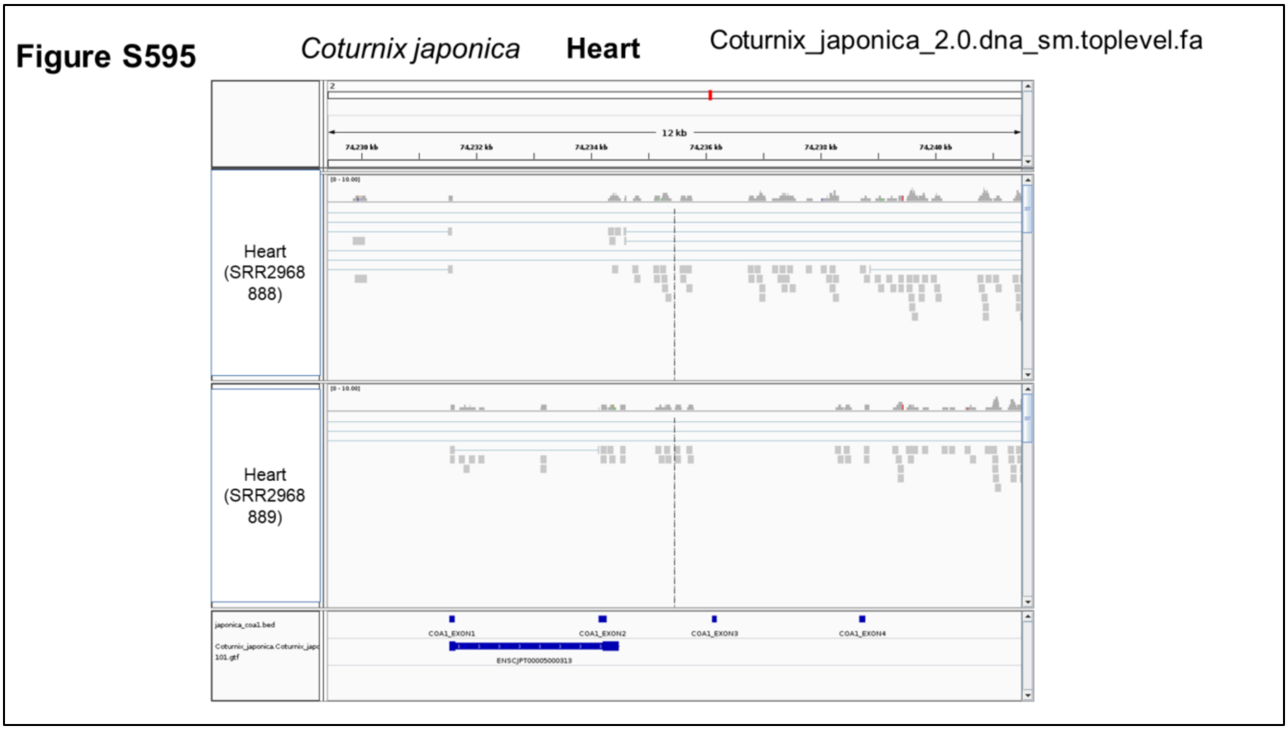

Figure S595: Screenshot of RNA-seq reads aligned to each of the putative locations of the four *COA1* exons in Japanese quail (*Coturnix japonica*). Gray boxes represent reads aligned to the genome and thin lines connecting these boxes represent the spliced read alignments. Datasets from heart (SRR2968888) and (SRR2968889) have been mapped to the Coturnix_japonica_2.0.dna_sm.toplevel.fa genome. Locations of each of the exons are depicted as bed record in the bottom row.

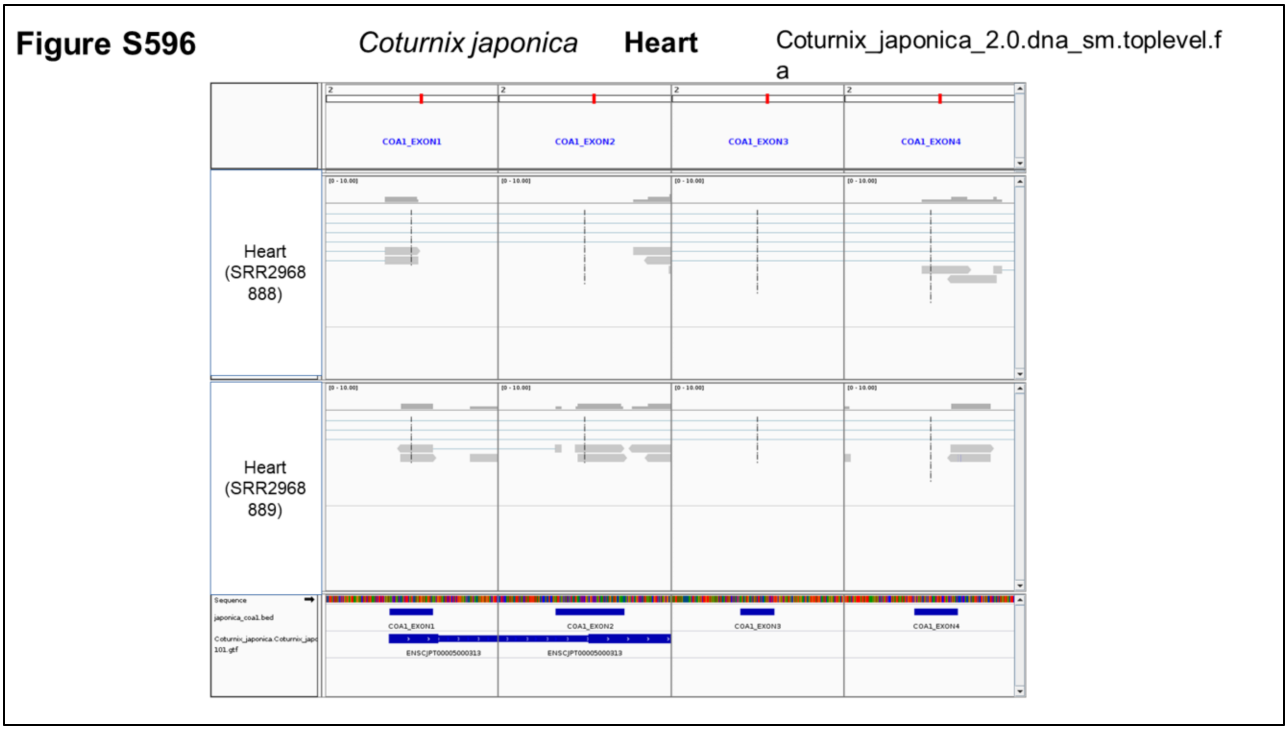

Figure S596: A zoomed in view of each of the *COA1* exons shown in **Supplementary Fig. S595**.

Figure S597 *Coturnix japonica* **Heart** Coturnix_japonica_2.0.dna_sm.toplevel.fa

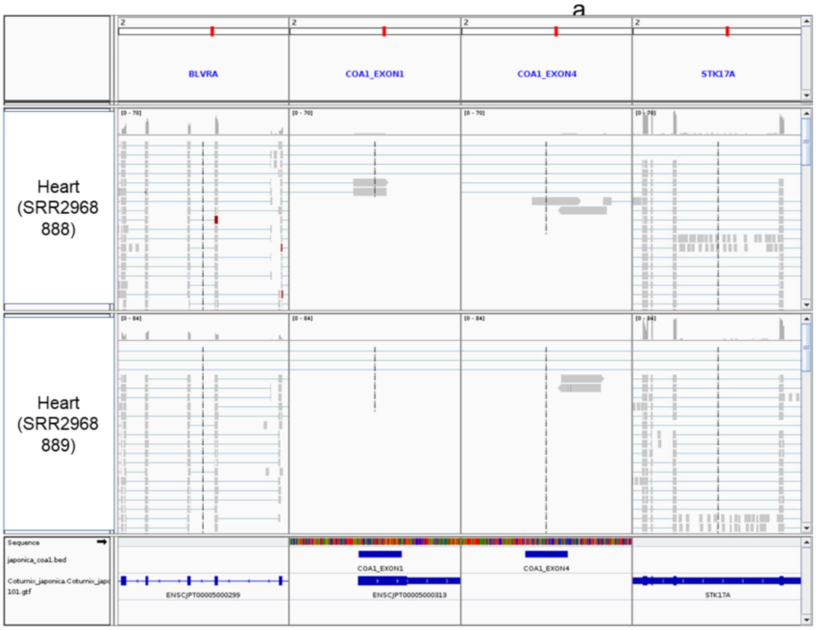

Figure S597: Screenshot of RNA-seq reads aligned to *BLVRA* and *STK17A* to *COA1* exon 1 and 4 respectively in Japanese quail (*Coturnix japonica*). Gray boxes represent reads aligned to the genome and thin lines connecting these boxes represent the spliced read alignments. Datasets from heart (SRR2968888) and (SRR2968889) have been mapped to the Coturnix_japonica_2.0.dna_sm.toplevel.fa genome. Locations of each of the exons are depicted as bed record in the bottom row.

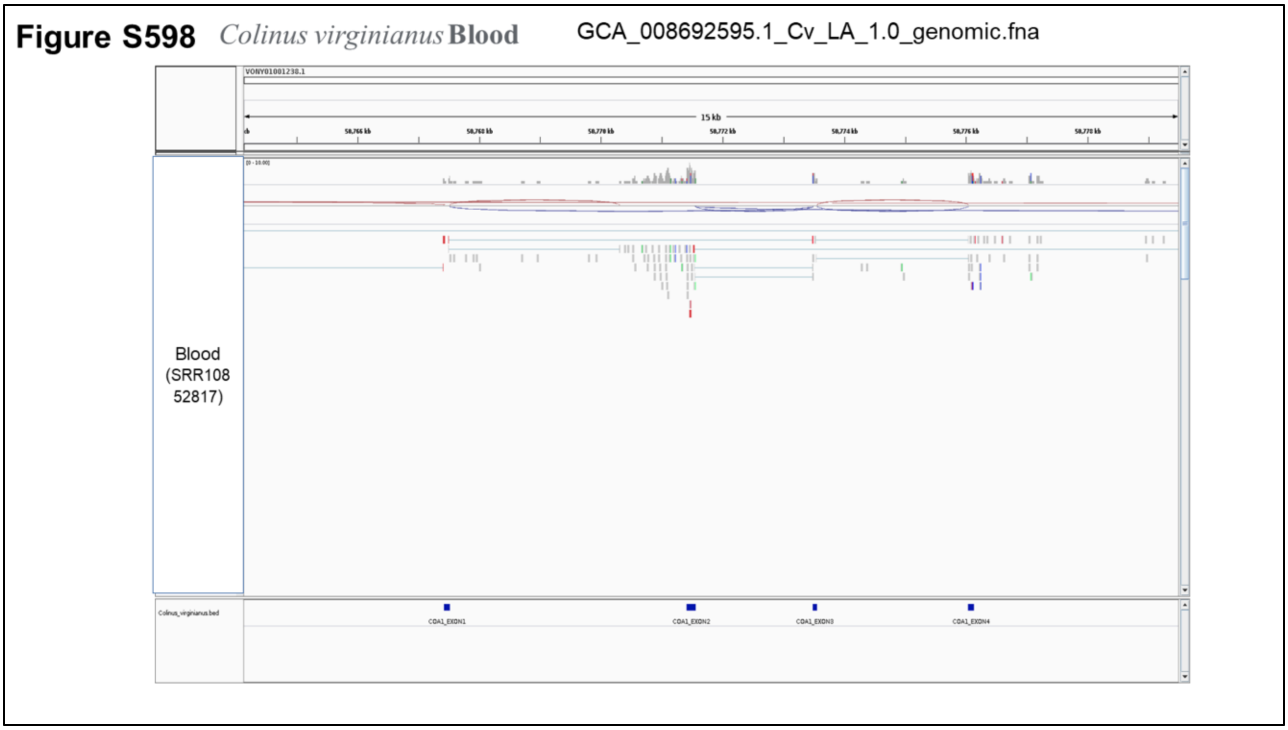

Figure S598: Screenshot of RNA-seq reads aligned to each of the putative locations of the four *COA1* exons in northern bobwhite (*Colinus virginianus*). Gray boxes represent reads aligned to the genome and thin lines connecting these boxes represent the spliced read alignments. Datasets from blood (SRR10852817) have been mapped to the GCA_008692595.1_Cv_LA_1.0_genomic.fna genome. Locations of each of the exons are depicted as bed record in the bottom row.

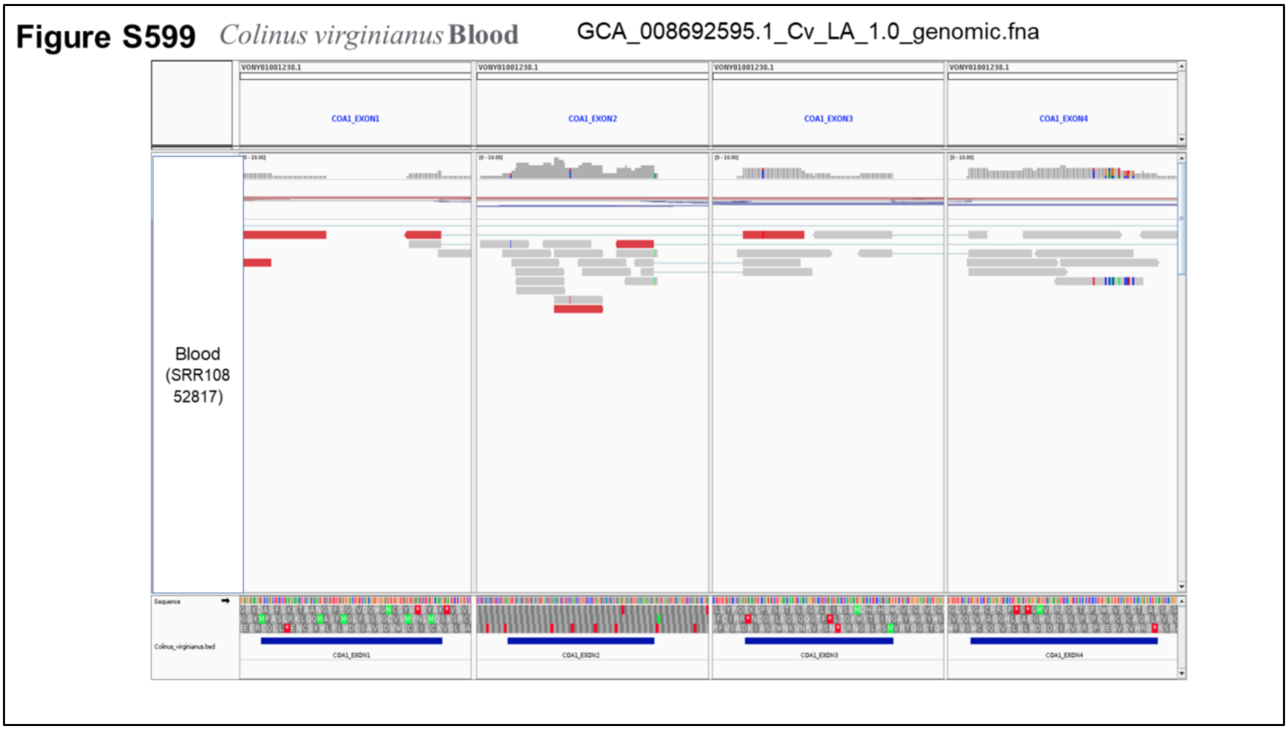

Figure S599: A zoomed in view of each of the *COA1* exons shown in **Supplementary Fig. S598**.

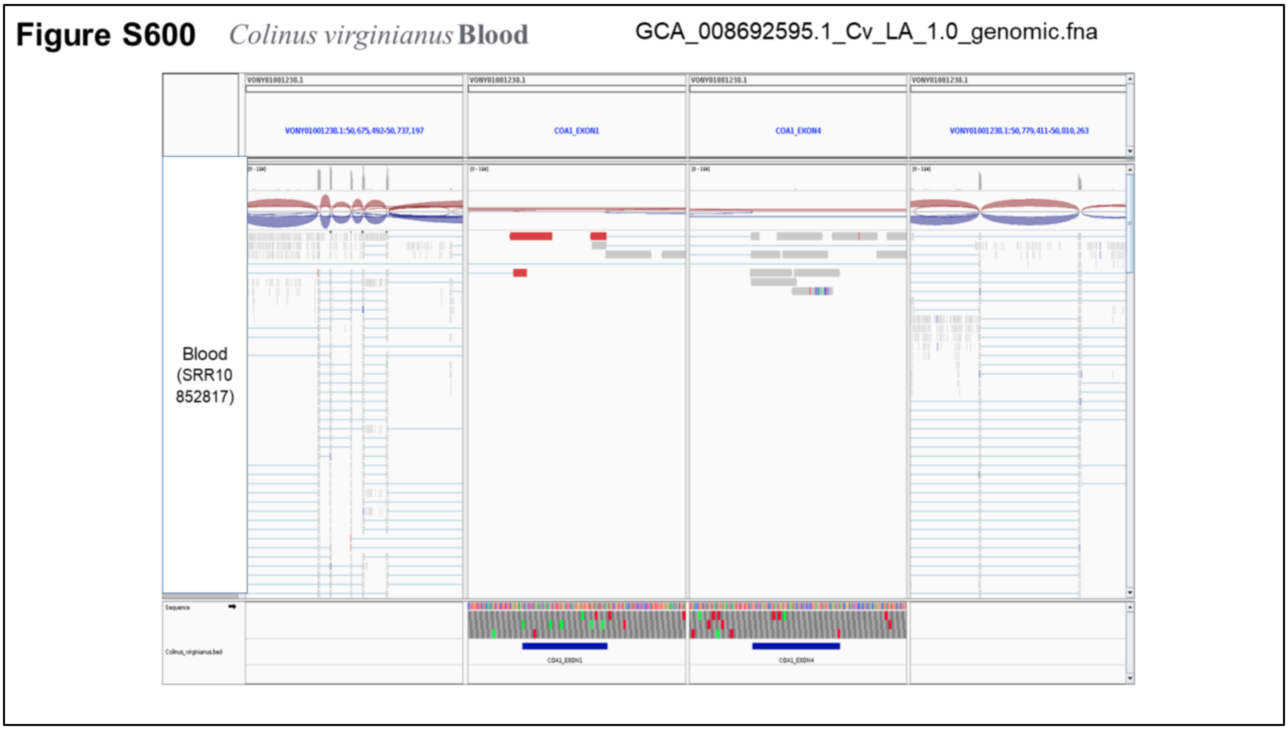

Figure S600: Screenshot of RNA-seq reads aligned to *BLVRA* and *STK17A* to *COA1* exon 1 and 4 respectively in northern bobwhite (*Colinus virginianus*). Gray boxes represent reads aligned to the genome and thin lines connecting these boxes represent the spliced read alignments. Datasets from blood (SRR10852817) have been mapped to the GCA_008692595.1_Cv_LA_1.0_genomic.fna genome. Locations of each of the exons are depicted as bed record in the bottom row.

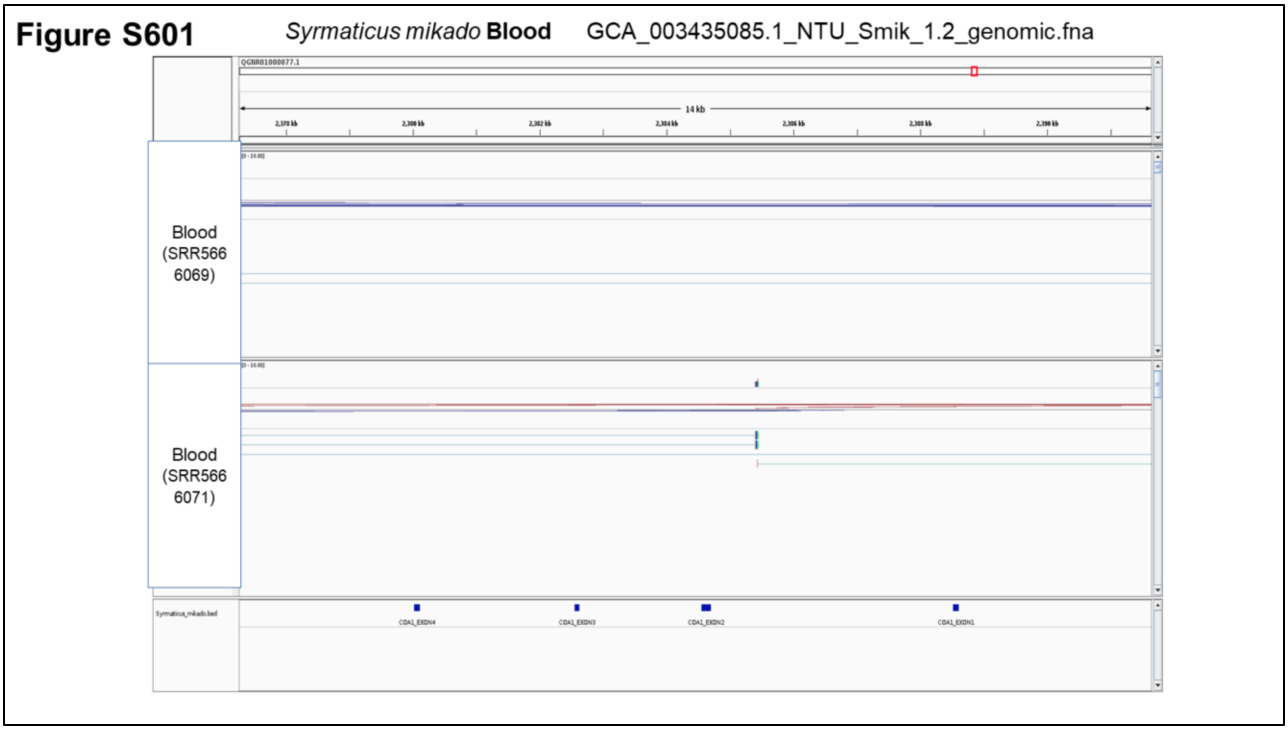

Figure S601: Screenshot of RNA-seq reads aligned to each of the putative locations of the four *COA1* exons in Mikado pheasant (*Syrmaticus mikado*). Gray boxes represent reads aligned to the genome and thin lines connecting these boxes represent the spliced read alignments. Datasets from blood (SRR5666069 and SRR5666071) have been mapped to the GCA_003435085.1_NTU_Smik_1.2_genomic.fna genome. Locations of each of the exons are depicted as bed record in the bottom row.

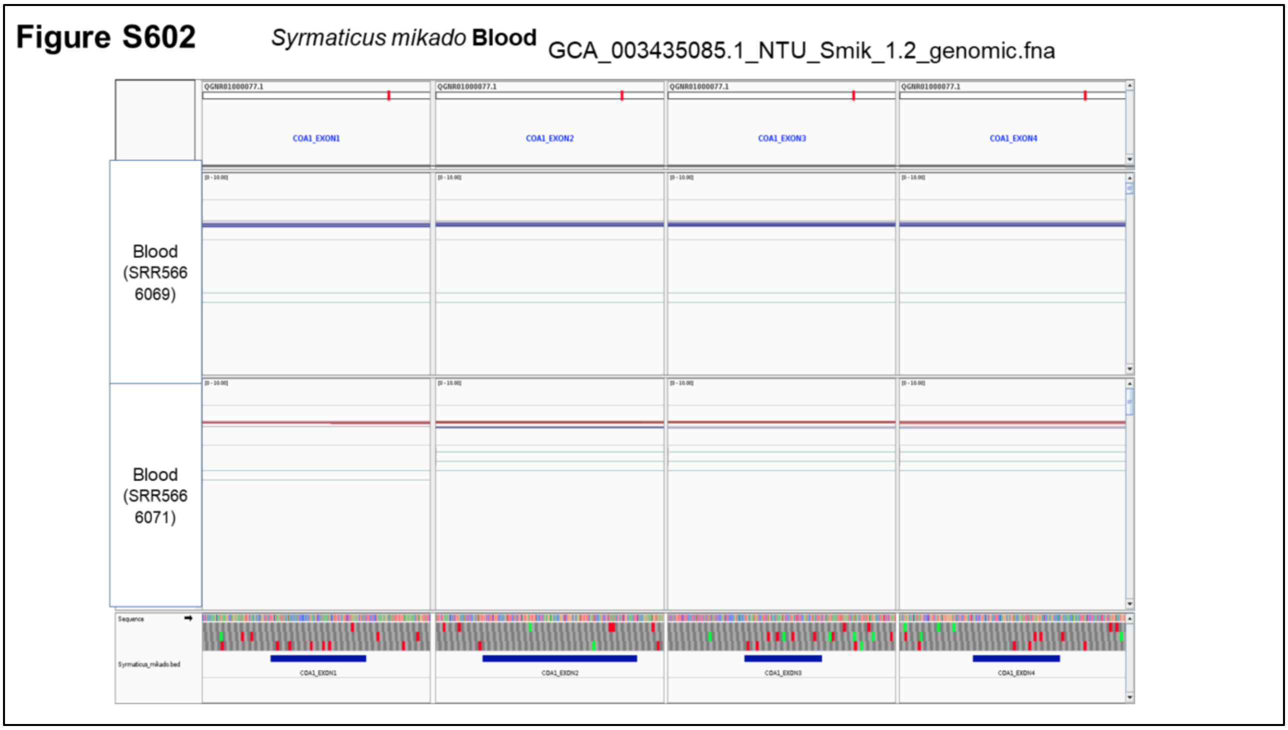

Figure S602: A zoomed in view of each of the *COA1* exons shown in **Supplementary Fig. S601**.

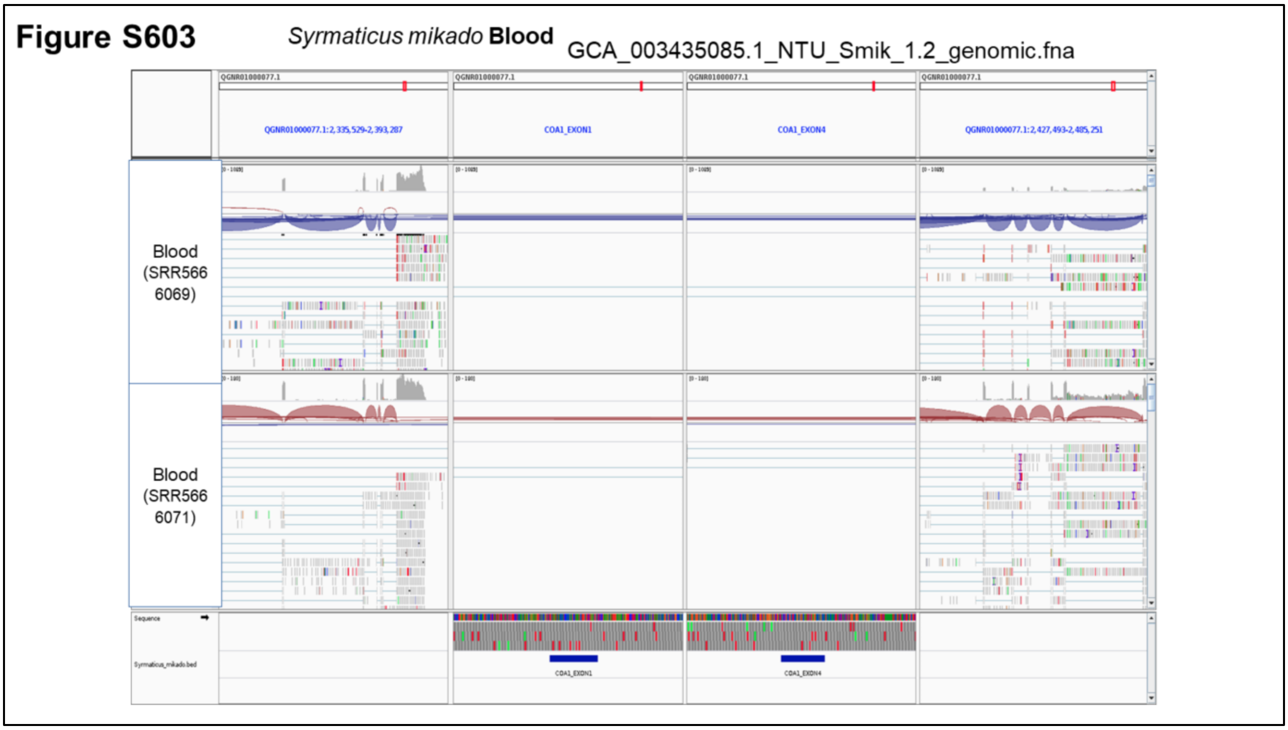

Figure S603: Screenshot of RNA-seq reads aligned to *BLVRA* and *STK17A* to *COA1* exon 1 and 4 respectively in Mikado pheasant (*Syrmaticus mikado*). Gray boxes represent reads aligned to the genome and thin lines connecting these boxes represent the spliced read alignments. Datasets from blood (SRR5666069 and SRR5666071) have been mapped to the GCA_003435085.1_NTU_Smik_1.2_genomic.fna genome. Locations of each of the exons are depicted as bed record in the bottom row.

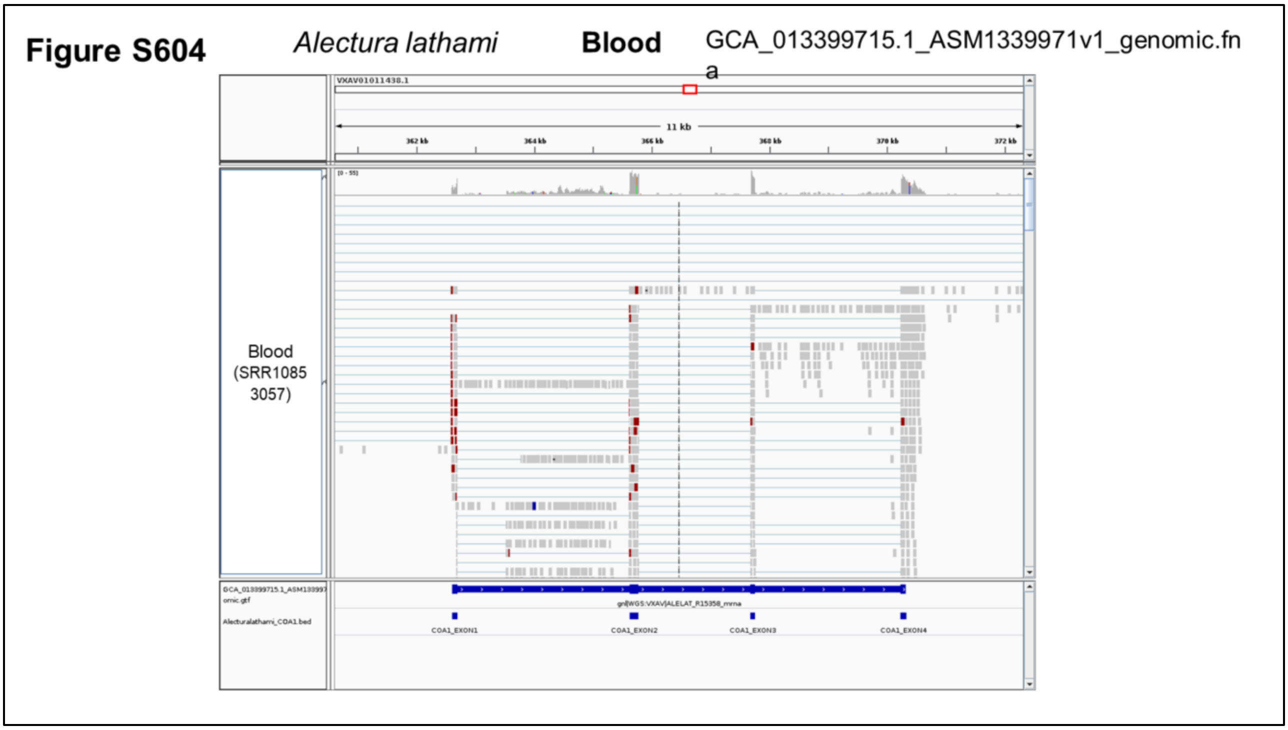

Figure S604: Screenshot of RNA-seq reads aligned to each of the putative locations of the four *COA1* exons in Australian brush turkey (*Alectura lathami*). Gray boxes represent reads aligned to the genome and thin lines connecting these boxes represent the spliced read alignments. Datasets from blood (SRR10853057) have been mapped to the GCA_013399715.1_ASM1339971v1_genomic.fna genome. Locations of each of the exons are depicted as bed record in the bottom row.

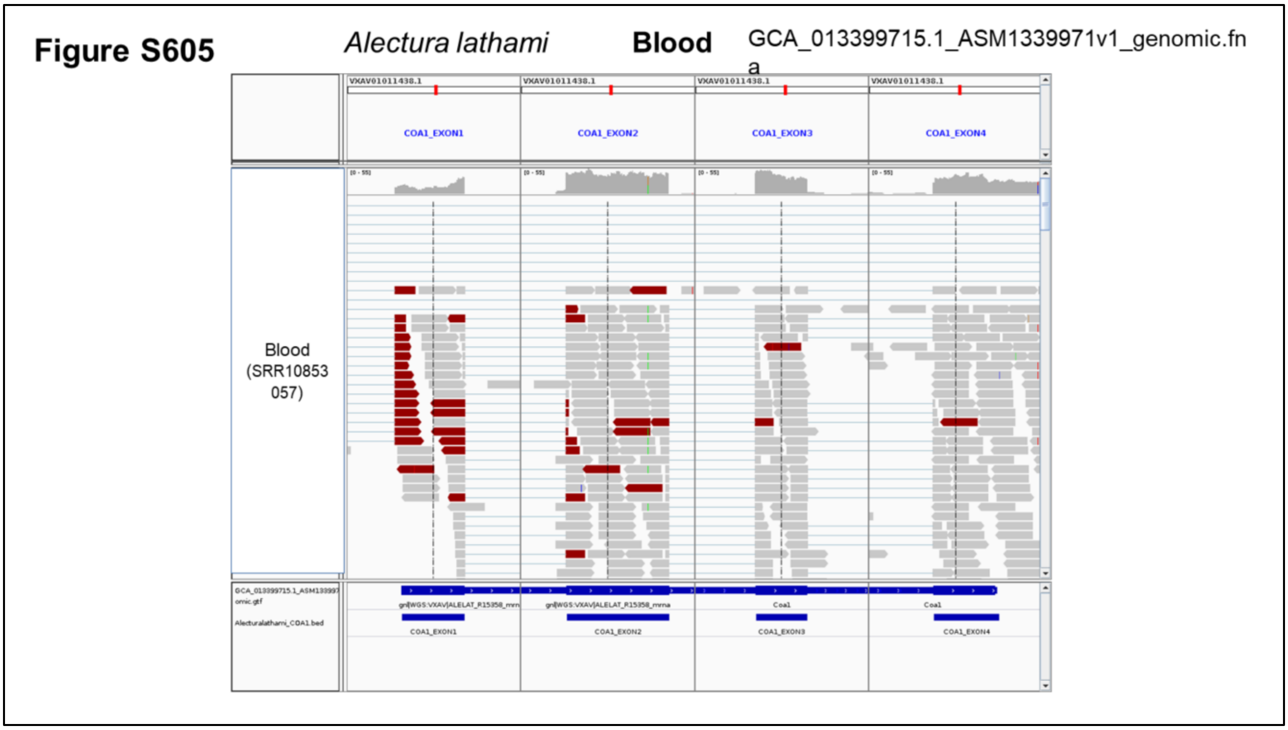

Figure S605: A zoomed in view of each of the *COA1* exons shown in **Supplementary Fig. S604**.

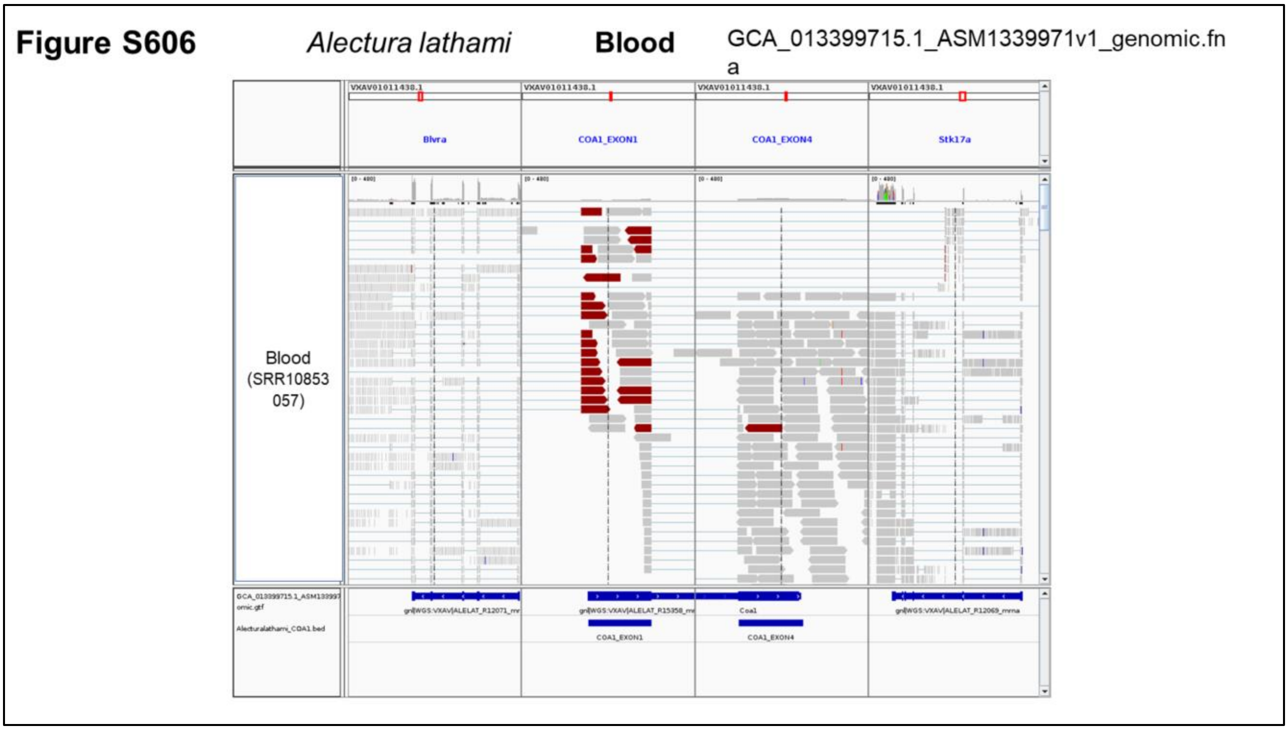

Figure S606: Screenshot of RNA-seq reads aligned to *BLVRA* and *STK17A* to *COA1* exon 1 and 4 respectively in Australian brush turkey (*Alectura lathami*). Gray boxes represent reads aligned to the genome and thin lines connecting these boxes represent the spliced read alignments. Datasets from blood (SRR10853057) have been mapped to the GCA_013399715.1_ASM1339971v1_genomic.fna genome. Locations of each of the exons are depicted as bed record in the bottom row.

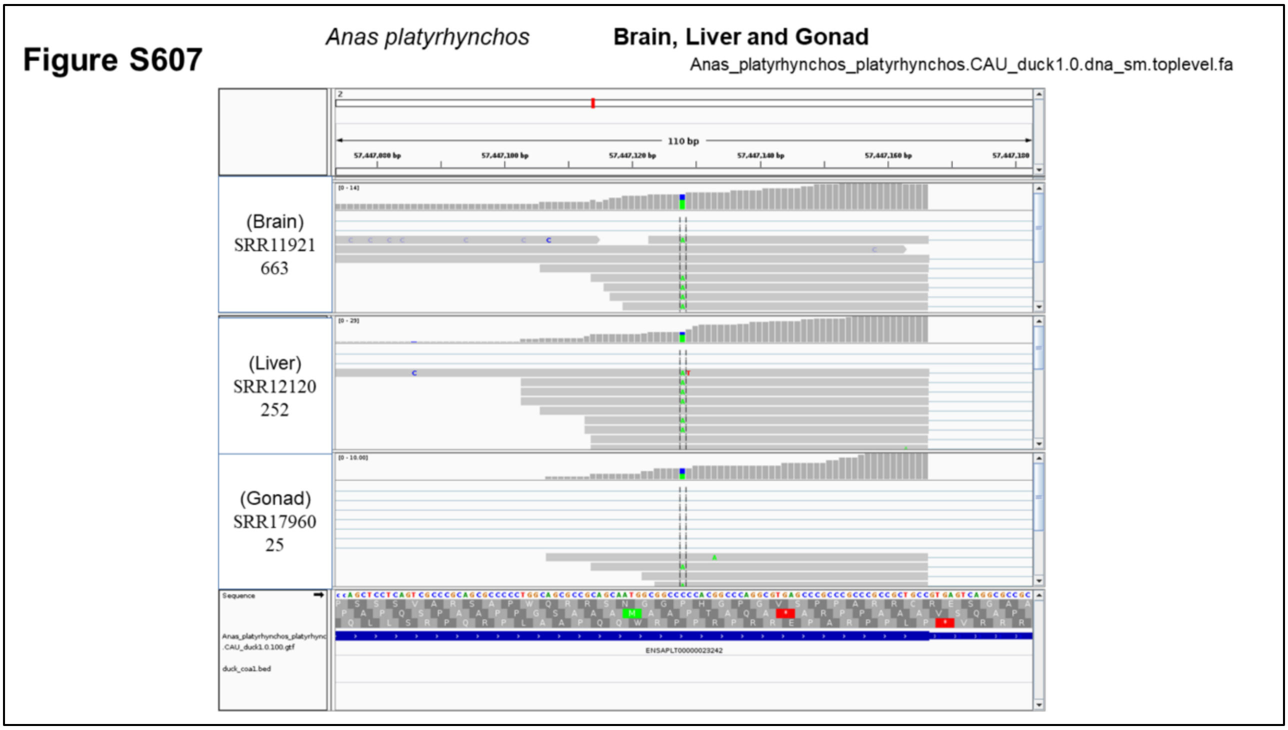

Figure S607: A premature stop codon in 1st exon of *COA1* of mallard duck (*Anas platyrhynchos).*

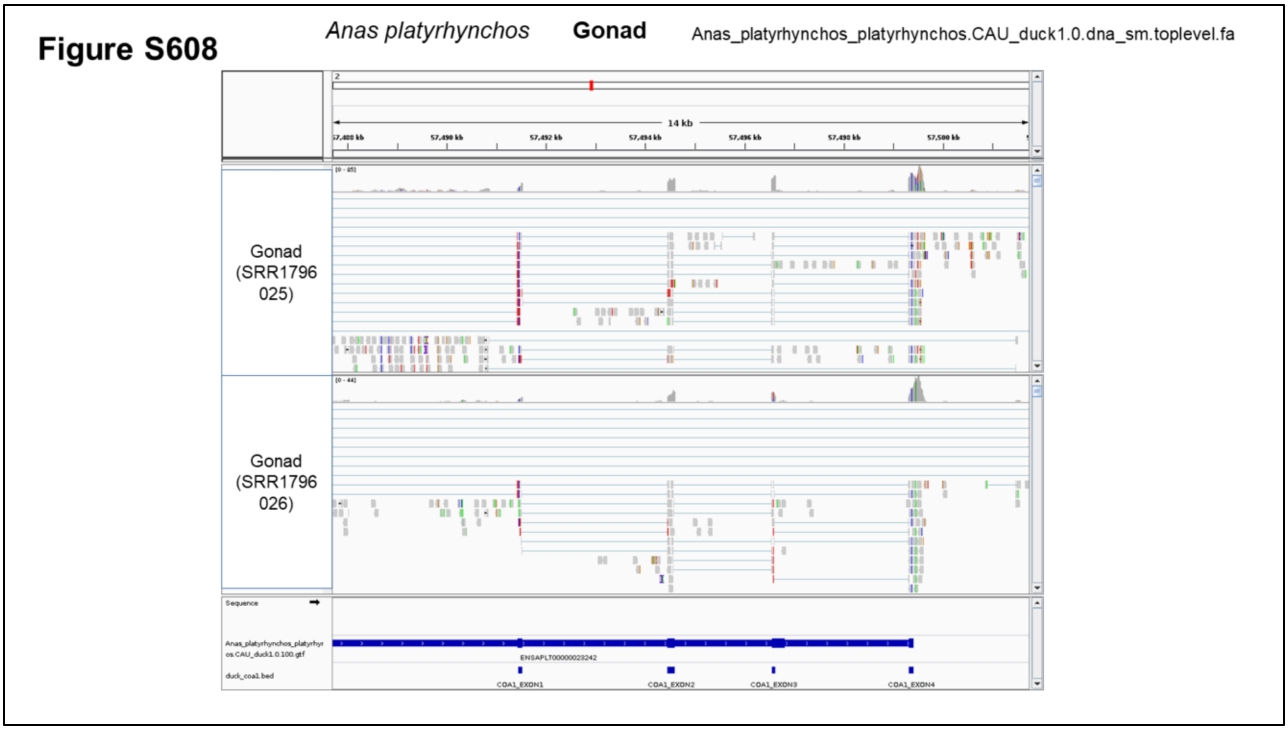

Figure S608: Screenshot of RNA-seq reads aligned to each of the putative locations of the four *COA1* exons in mallard duck (*Anas platyrhynchos*). Gray boxes represent reads aligned to the genome and thin lines connecting these boxes represent the spliced read alignments. Datasets from gonad (SRR1796025) and (SRR1796026) have been mapped to the Anas_platyrhynchos_platyrhynchos.CAU_duck1.0.dna_sm.toplevel.fa genome. Locations of each of the exons are depicted as bed record in the bottom row.

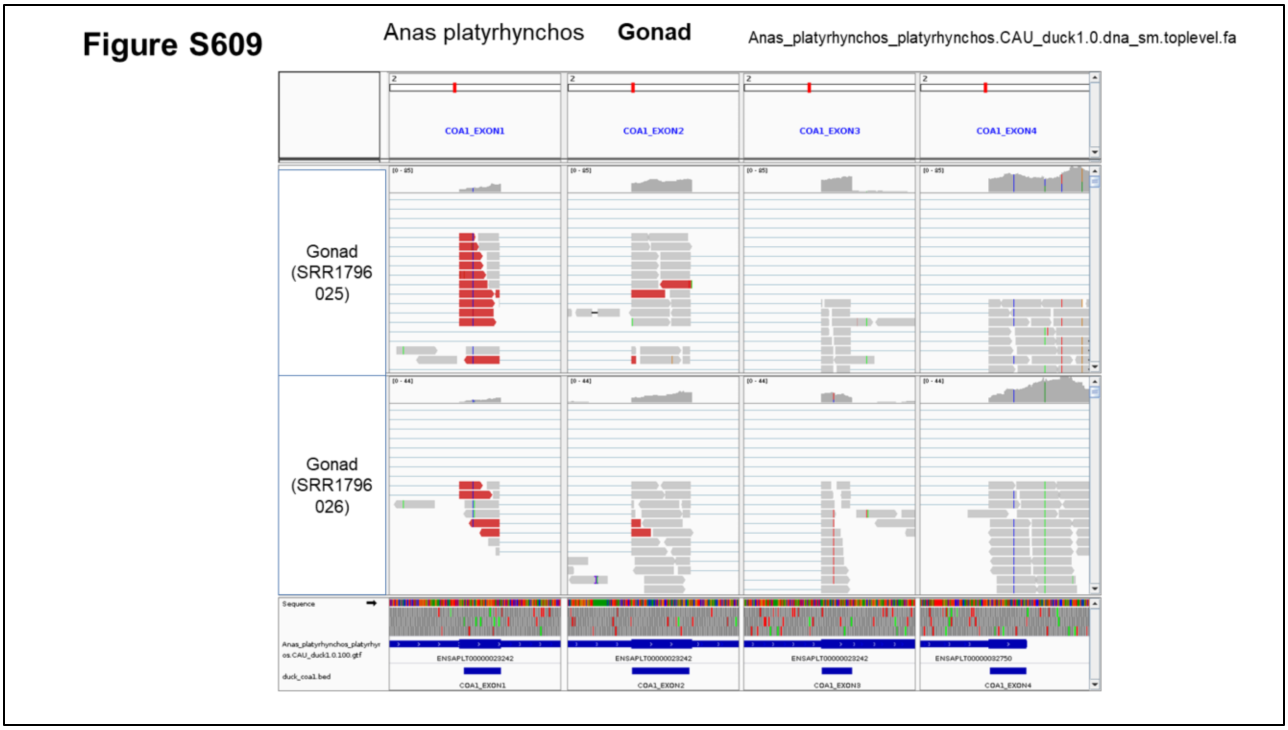

Figure S609: A zoomed in view of each of the *COA1* exons shown in **Supplementary Fig. S608**.

Figure S610 *Anas platyrhynchos* **Spleen** Anas_platyrhynchos_platyrhynchos.CAU_duck1.0.dna_sm.toplevel.fa

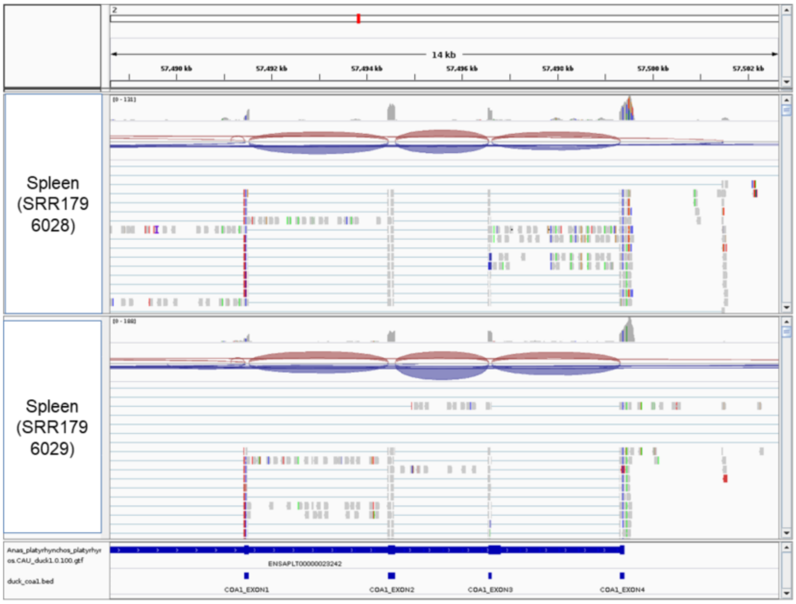

Figure S610: Screenshot of RNA-seq reads aligned to each of the putative locations of the four *COA1* exons in mallard duck (*Anas platyrhynchos*). Gray boxes represent reads aligned to the genome and thin lines connecting these boxes represent the spliced read alignments. Datasets from spleen (SRR1796028) and (SRR1796029) have been mapped to the Anas_platyrhynchos_platyrhynchos.CAU_duck1.0.dna_sm.toplevel.fa genome. Locations of each of the exons are depicted as bed record in the bottom row.

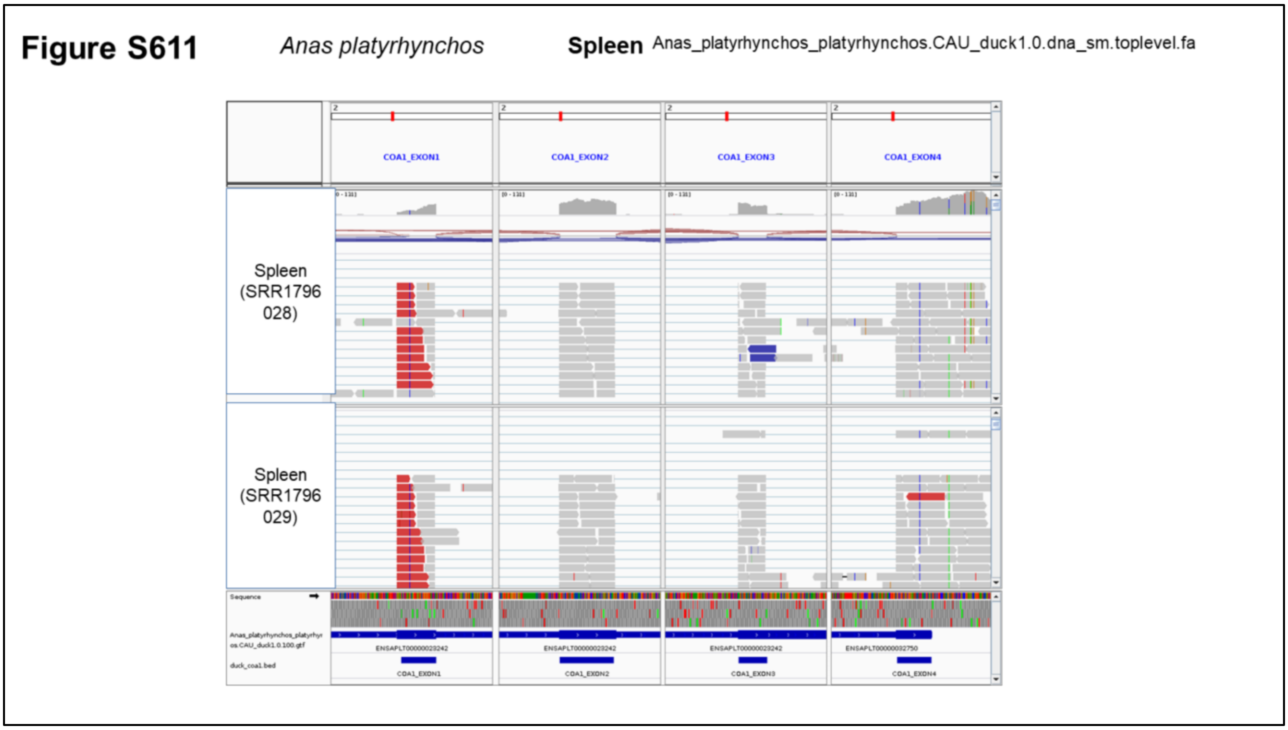

Figure S611: A zoomed in view of each of the *COA1* exons shown in **Supplementary Fig. S610**.

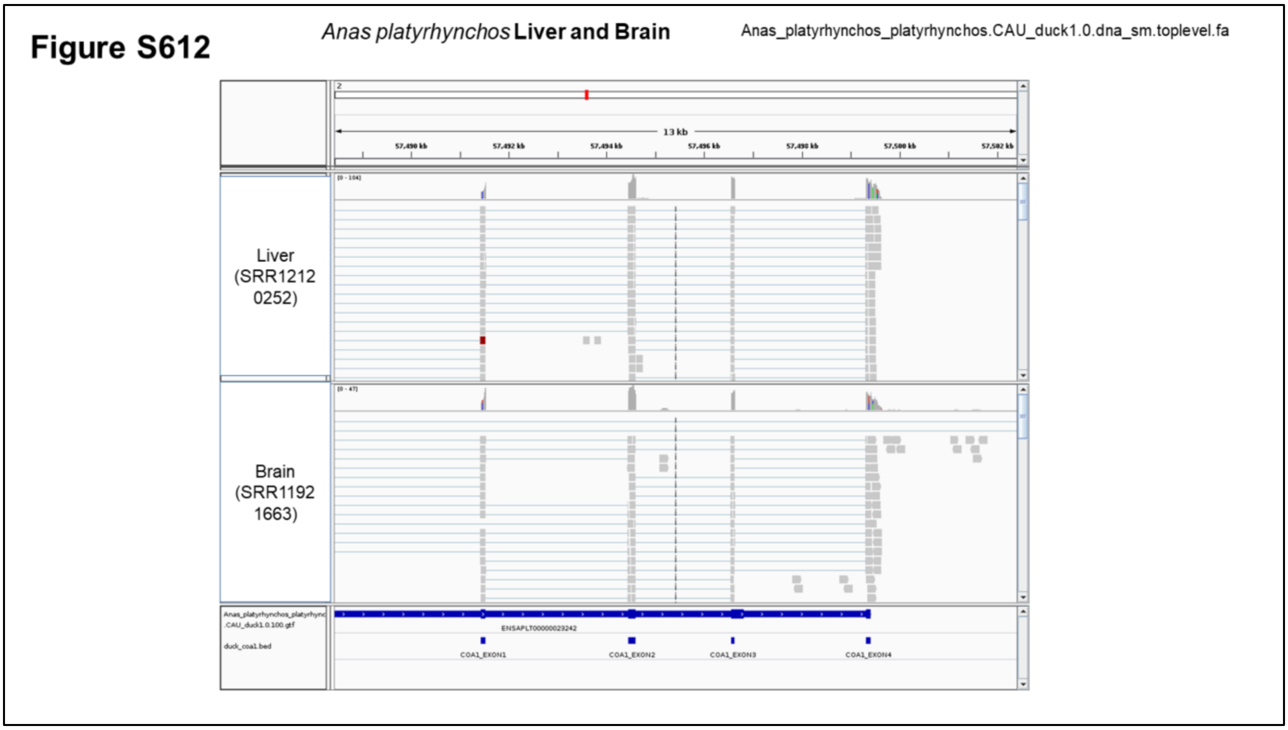

Figure S612: Screenshot of RNA-seq reads aligned to each of the putative locations of the four *COA1* exons in mallard duck (*Anas platyrhynchos*). Gray boxes represent reads aligned to the genome and thin lines connecting these boxes represent the spliced read alignments. Datasets from liver (SRR12120252) and brain (SRR11921663) have been mapped to the Anas_platyrhynchos_platyrhynchos.CAU_duck1.0.dna_sm.toplevel.fa genome. Locations of each of the exons are depicted as bed record in the bottom row.

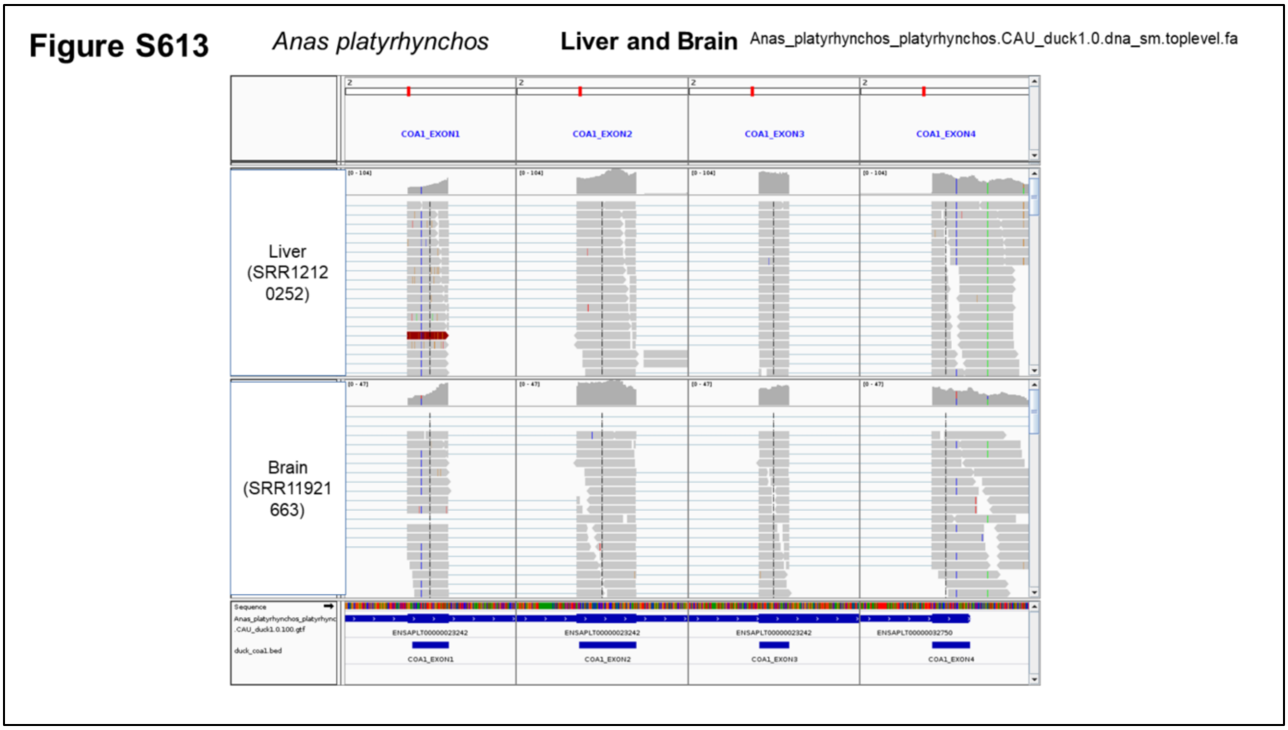

Figure S613: A zoomed in view of each of the *COA1* exons shown in **Supplementary Fig. S612**.

Figure S614 *Anas platyrhynchos* **Skin** Anas_platyrhynchos_platyrhynchos.CAU_duck1.0.dna_sm.toplevel.fa

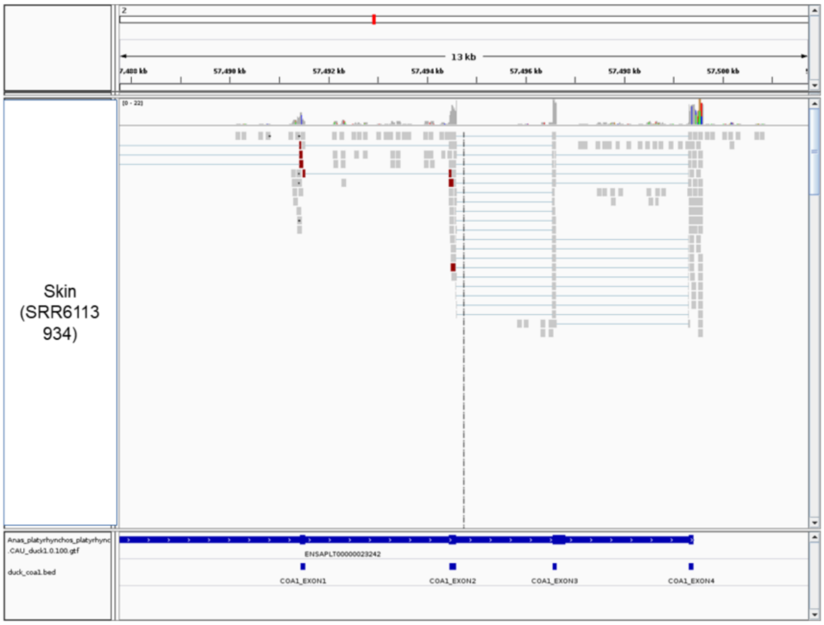

Figure S614: Screenshot of RNA-seq reads aligned to each of the putative locations of the four *COA1* exons in mallard duck (*Anas platyrhynchos*). Gray boxes represent reads aligned to the genome and thin lines connecting these boxes represent the spliced read alignments. Datasets from Skin (SRR6113934) have been mapped to the Anas_platyrhynchos_platyrhynchos.CAU_duck1.0.dna_sm.toplevel.fa genome. Locations of each of the exons are depicted as bed record in the bottom row.

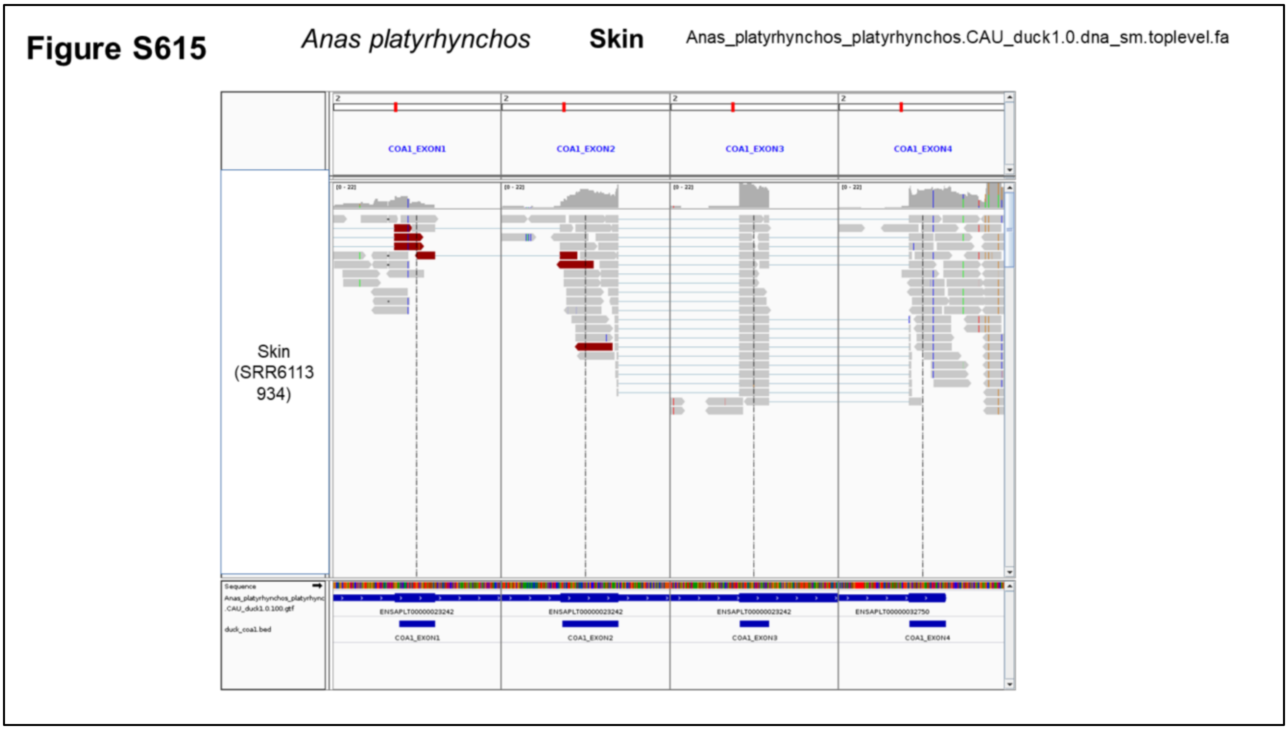

Figure S615: A zoomed in view of each of the *COA1* exons shown in **Supplementary Fig. S614**.

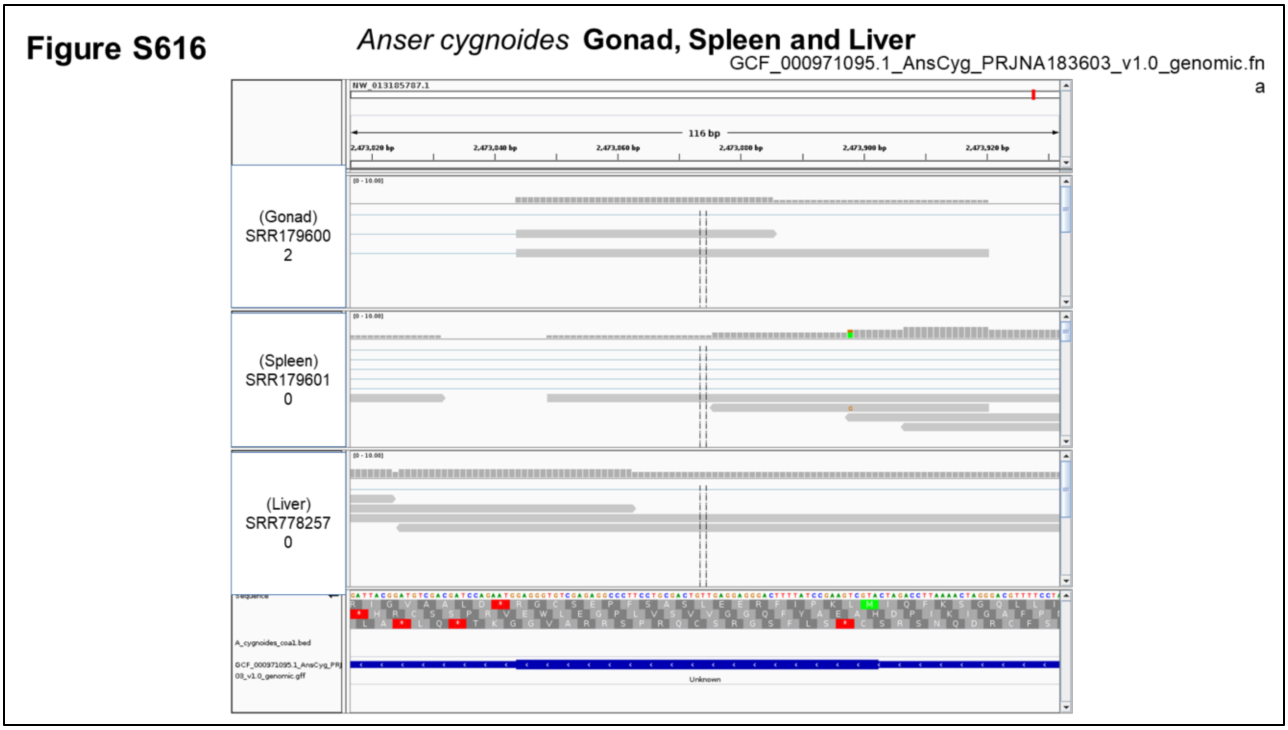

Figure S616: Less expression of first exon of 167 aa length transcript in swan goose (*Anser cygnoides*).

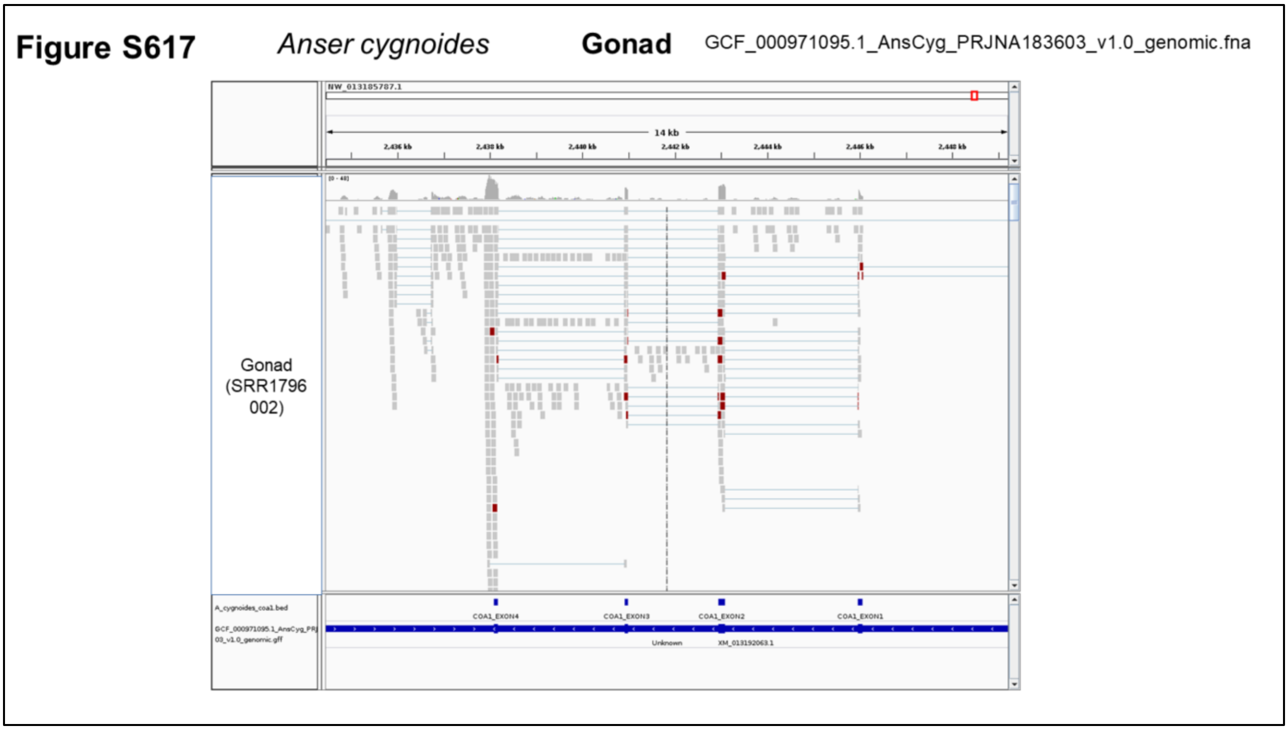

Figure S617: Screenshot of RNA-seq reads aligned to each of the putative locations of the four *COA1* exons in swan goose (*Anser cygnoides*). Gray boxes represent reads aligned to the genome and thin lines connecting these boxes represent the spliced read alignments. Datasets from gonad (SRR1796002) have been mapped to the GCF_000971095.1_AnsCyg_PRJNA183603_v1.0_genomic.fna genome. Locations of each of the exons are depicted as bed record in the bottom row.

Figure S618 *Anser cygnoides* **Gonad** GCF_000971095.1_AnsCyg_PRJNA183603_v1.0_genomic.fna

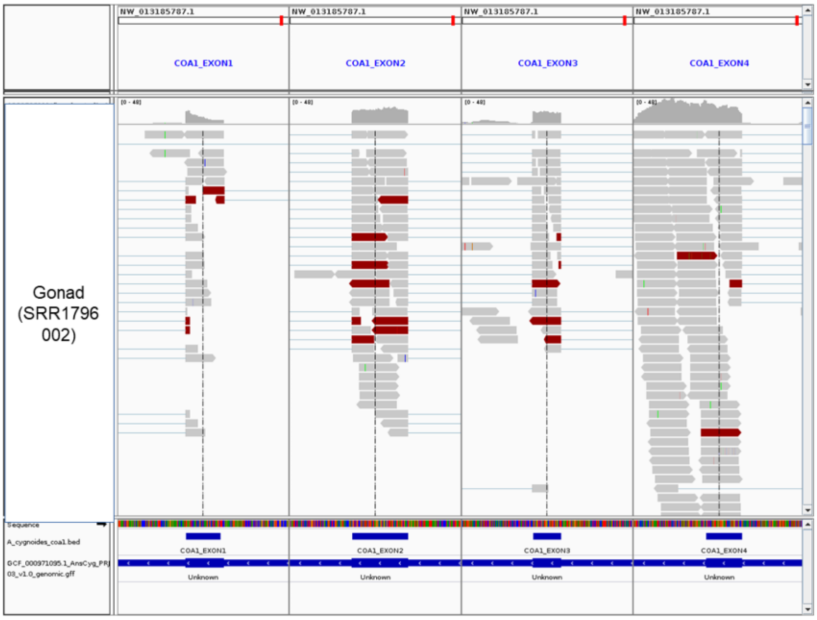

Figure S618: A zoomed in view of each of the *COA1* exons shown in **Supplementary Fig. S617**.

Figure S619 *Anser cygnoides* **Liver** GCF_000971095.1_AnsCyg_PRJNA183603_v1.0_genomic.fna

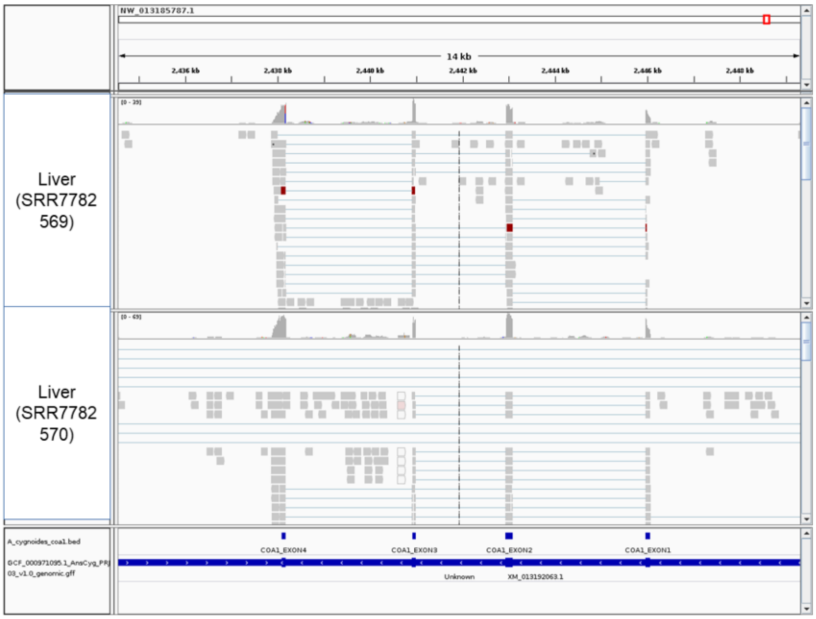

Figure S619: Screenshot of RNA-seq reads aligned to each of the putative locations of the four *COA1* exons in swan goose (*Anser cygnoides*). Gray boxes represent reads aligned to the genome and thin lines connecting these boxes represent the spliced read alignments. Datasets from liver (SRR7782569) and (SRR7782570) have been mapped to the GCF_000971095.1_AnsCyg_PRJNA183603_v1.0_genomic.fna genome. Locations of each of the exons are depicted as bed record in the bottom row.

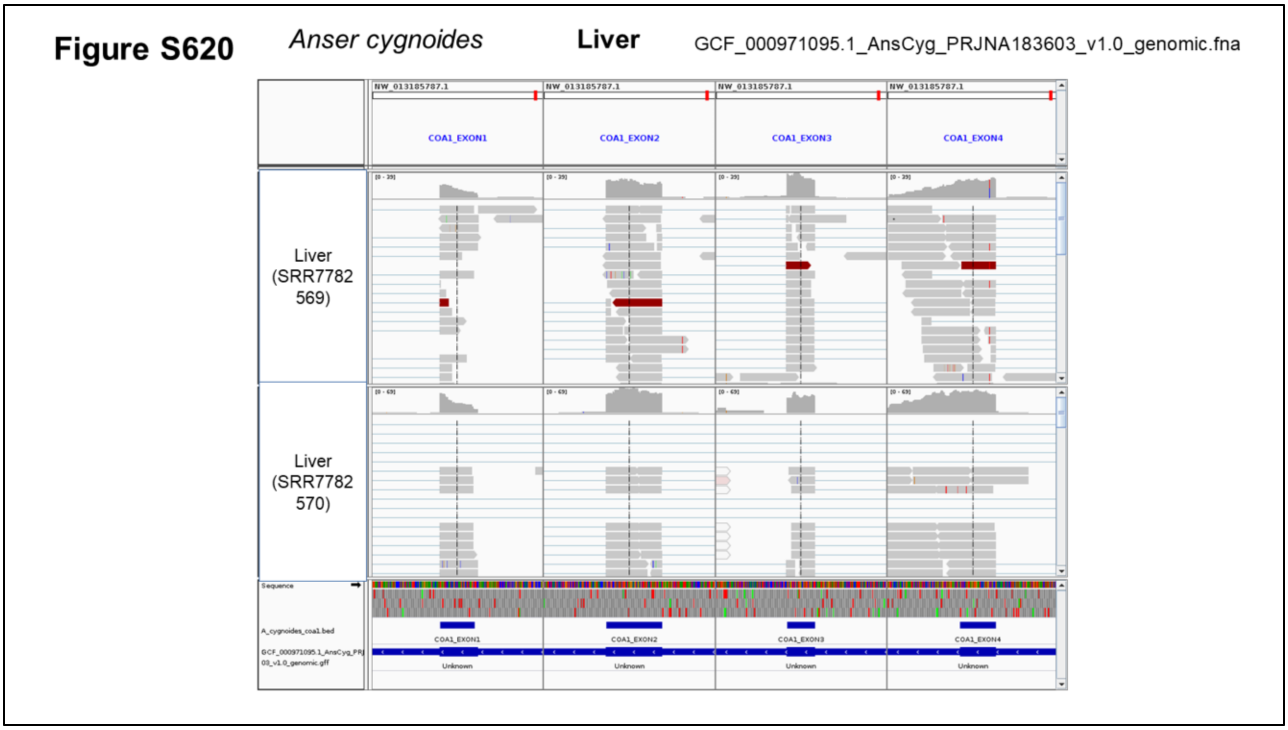

Figure S620: A zoomed in view of each of the *COA1* exons shown in **Supplementary Fig. S619**.

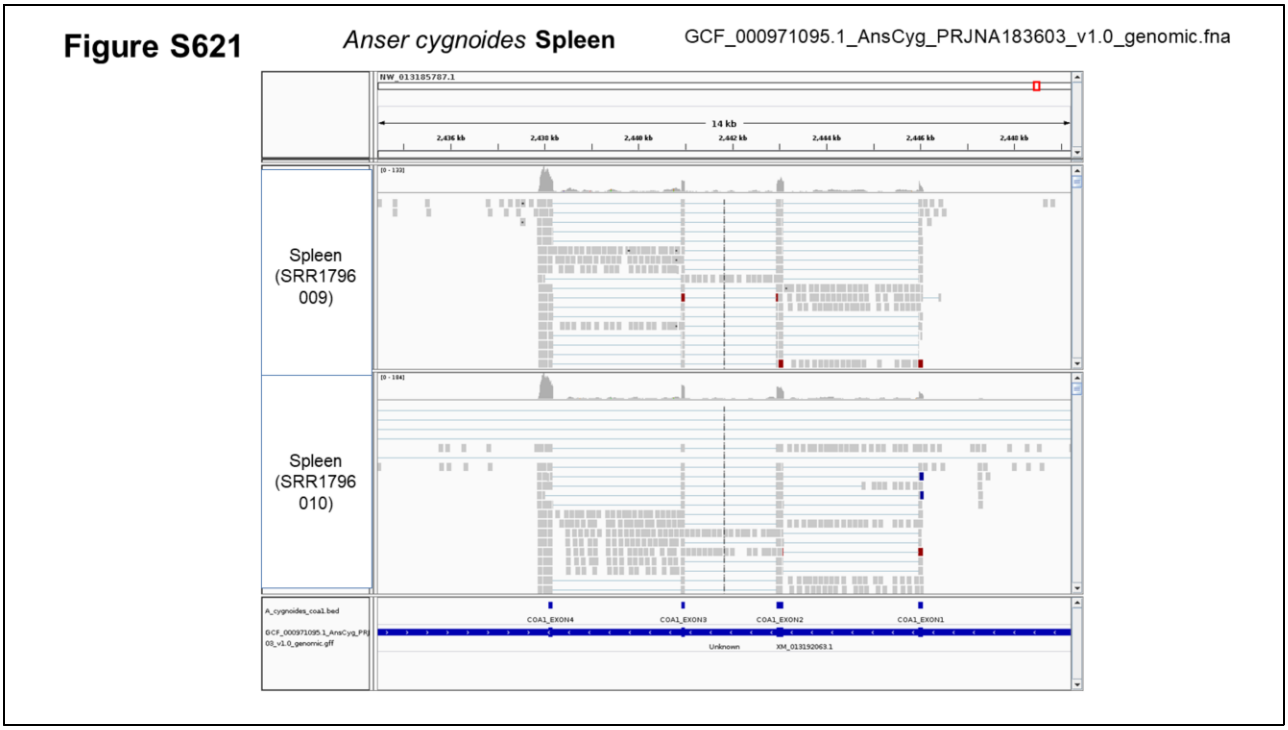

Figure S621: Screenshot of RNA-seq reads aligned to each of the putative locations of the four *COA1* exons in swan goose (*Anser cygnoides*). Gray boxes represent reads aligned to the genome and thin lines connecting these boxes represent the spliced read alignments. Datasets from spleen (SRR1796009) and (SRR1796010) have been mapped to the GCF_000971095.1_AnsCyg_PRJNA183603_v1.0_genomic.fna genome. Locations of each of the exons are depicted as bed record in the bottom row.

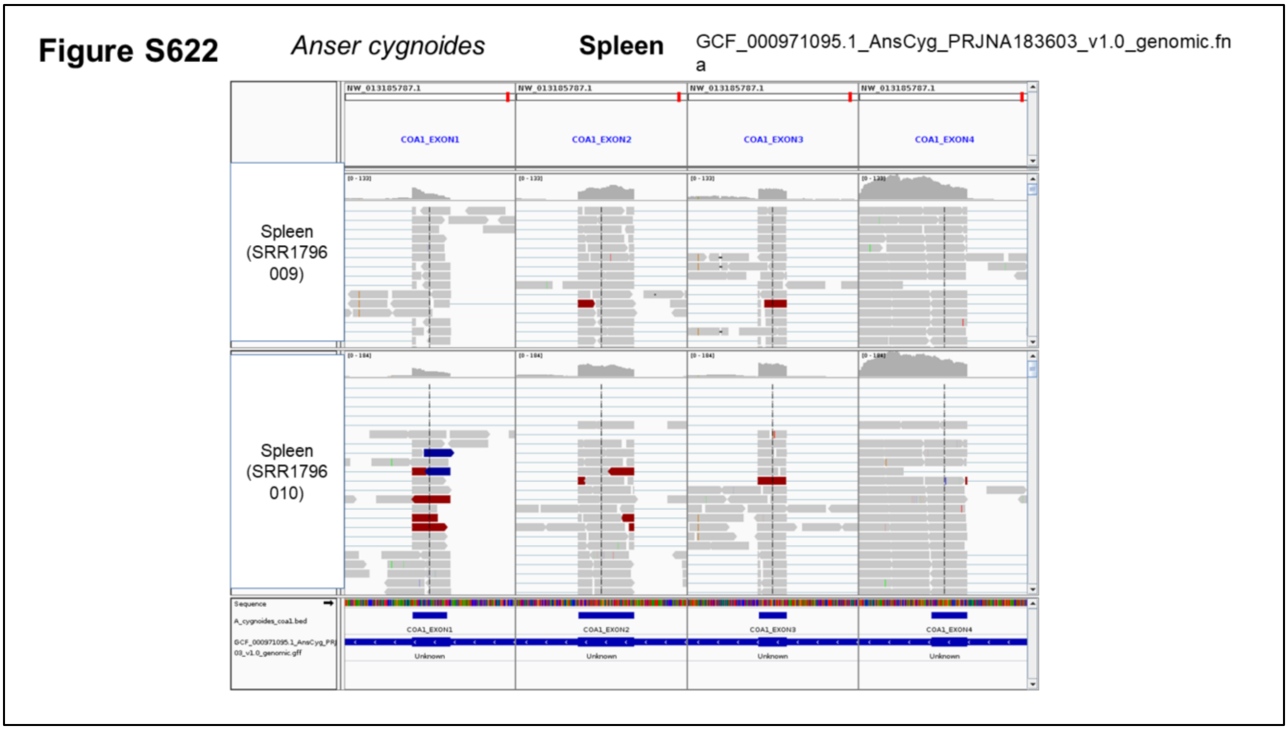

Figure S622: A zoomed in view of each of the *COA1* exons shown in **Supplementary Fig. S621**.

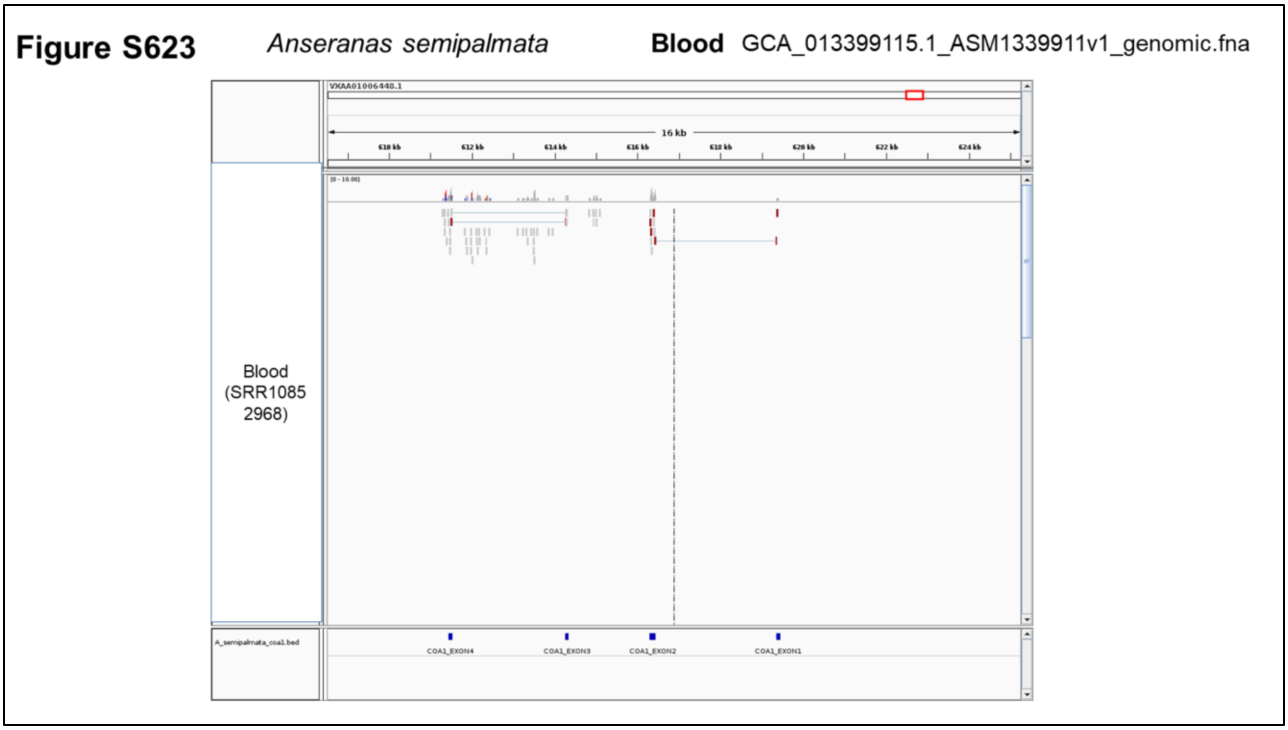

Figure S623: Screenshot of RNA-seq reads aligned to each of the putative locations of the four *COA1* exons in magpie goose (*Anseranas semipalmata*). Gray boxes represent reads aligned to the genome and thin lines connecting these boxes represent the spliced read alignments. Datasets from blood (SRR10852968) have been mapped to the GCA_013399115.1_ASM1339911v1_genomic.fna genome. Locations of each of the exons are depicted as bed record in the bottom row.

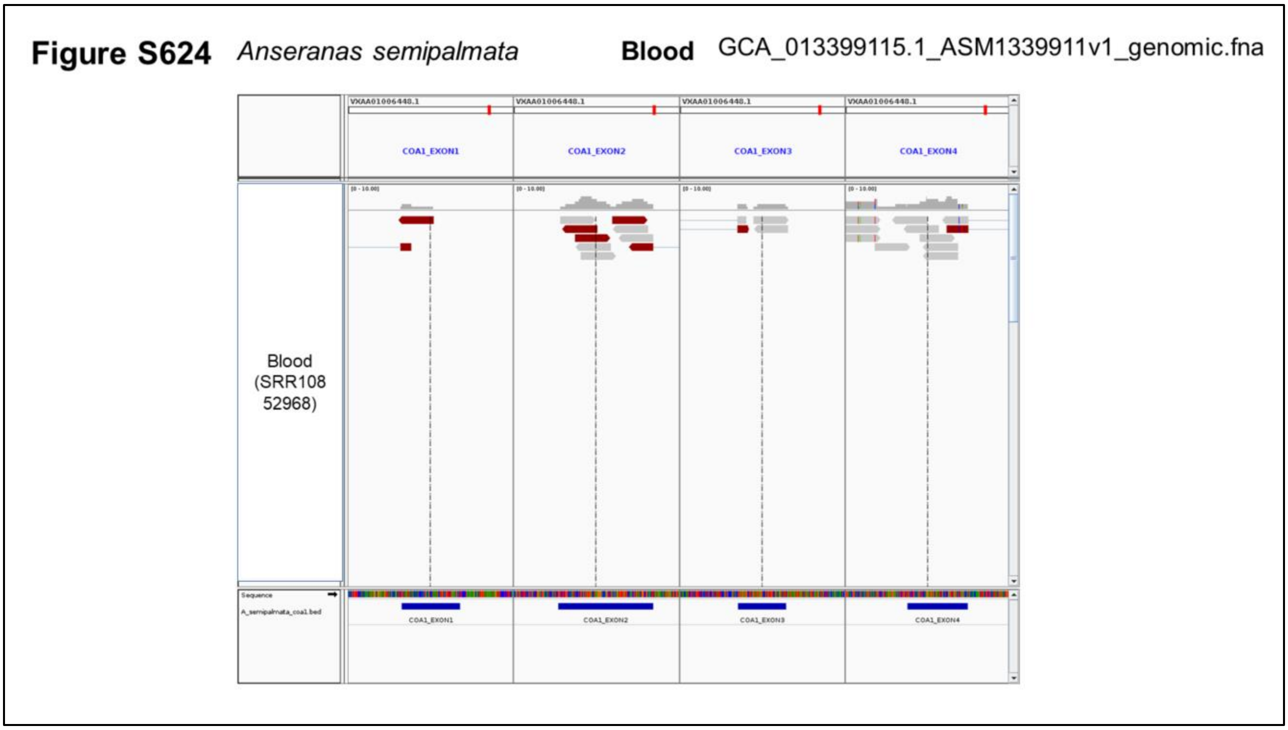

Figure S624: A zoomed in view of each of the *COA1* exons shown in **Supplementary Fig. S623**.

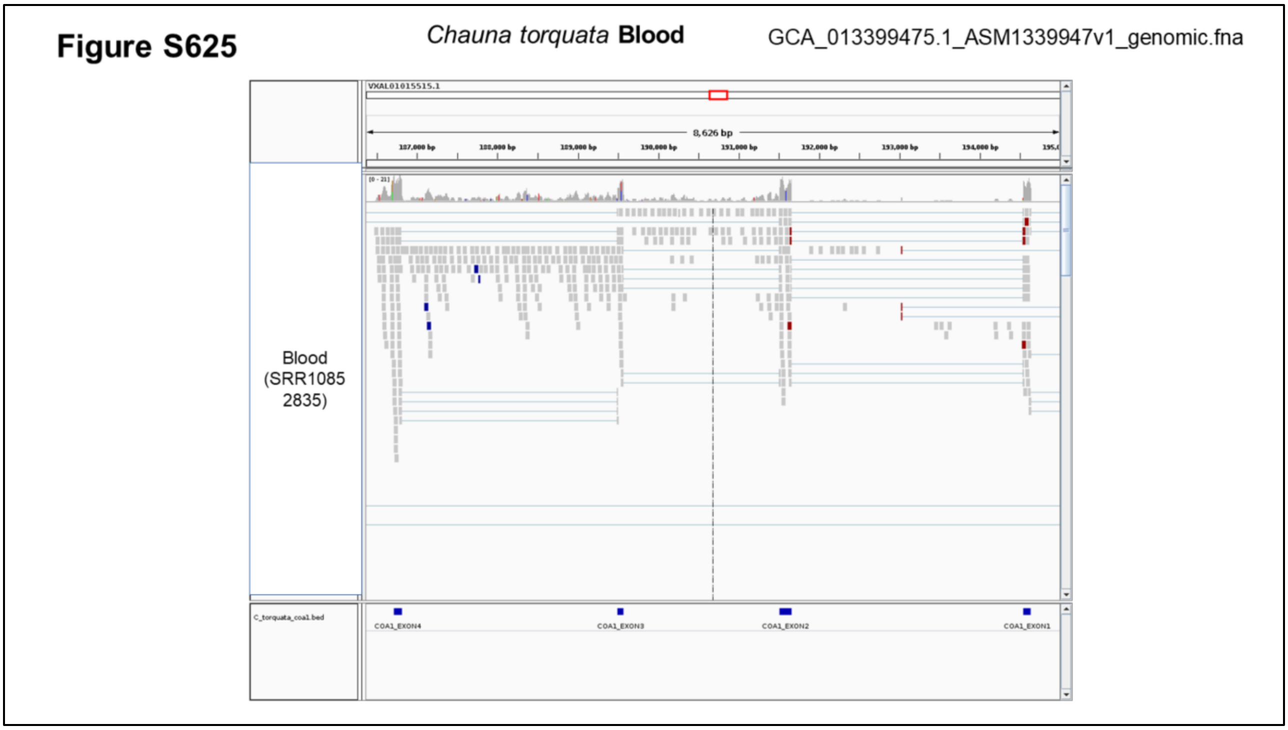

Figure S625: Screenshot of RNA-seq reads aligned to each of the putative locations of the four *COA1* exons in southern screamer (*Chauna torquata*). Gray boxes represent reads aligned to the genome and thin lines connecting these boxes represent the spliced read alignments. Datasets from blood (SRR10852835) have been mapped to the GCA_013399475.1_ASM1339947v1_genomic.fna genome. Locations of each of the exons are depicted as bed record in the bottom row.

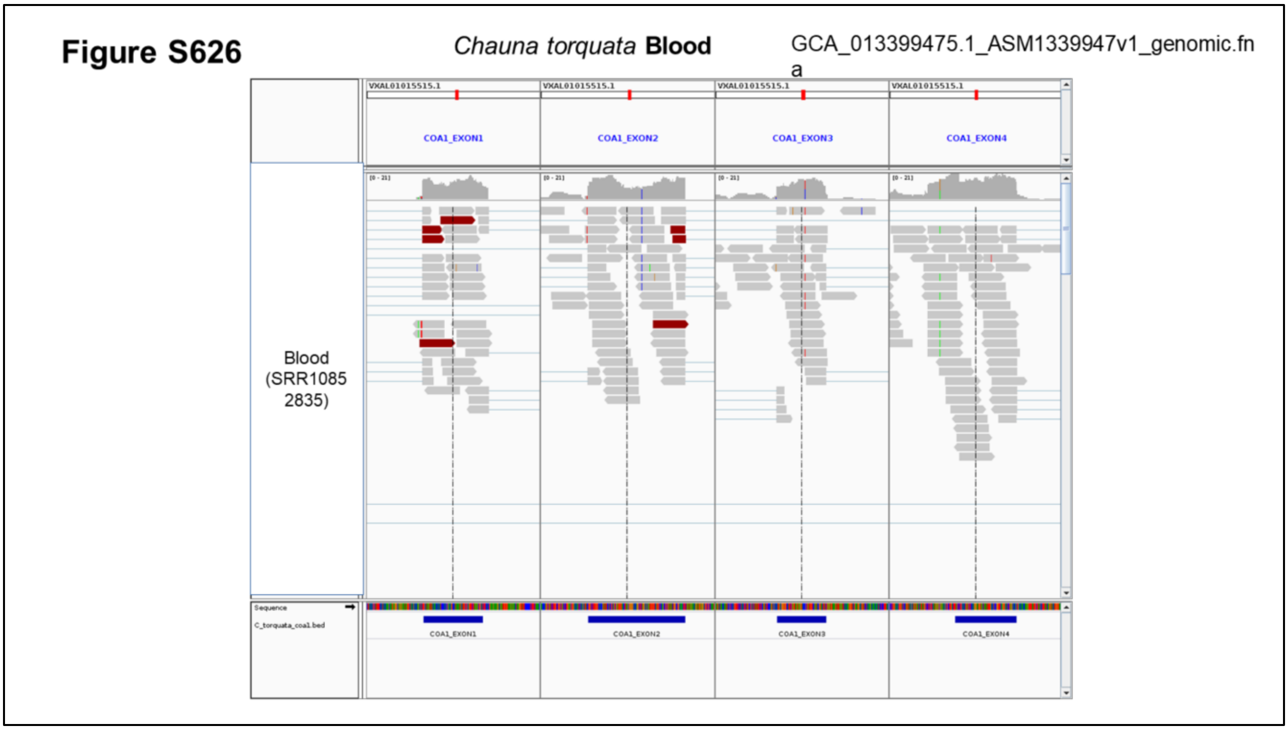

Figure S626: A zoomed in view of each of the *COA1* exons shown in **Supplementary Fig. S625**.

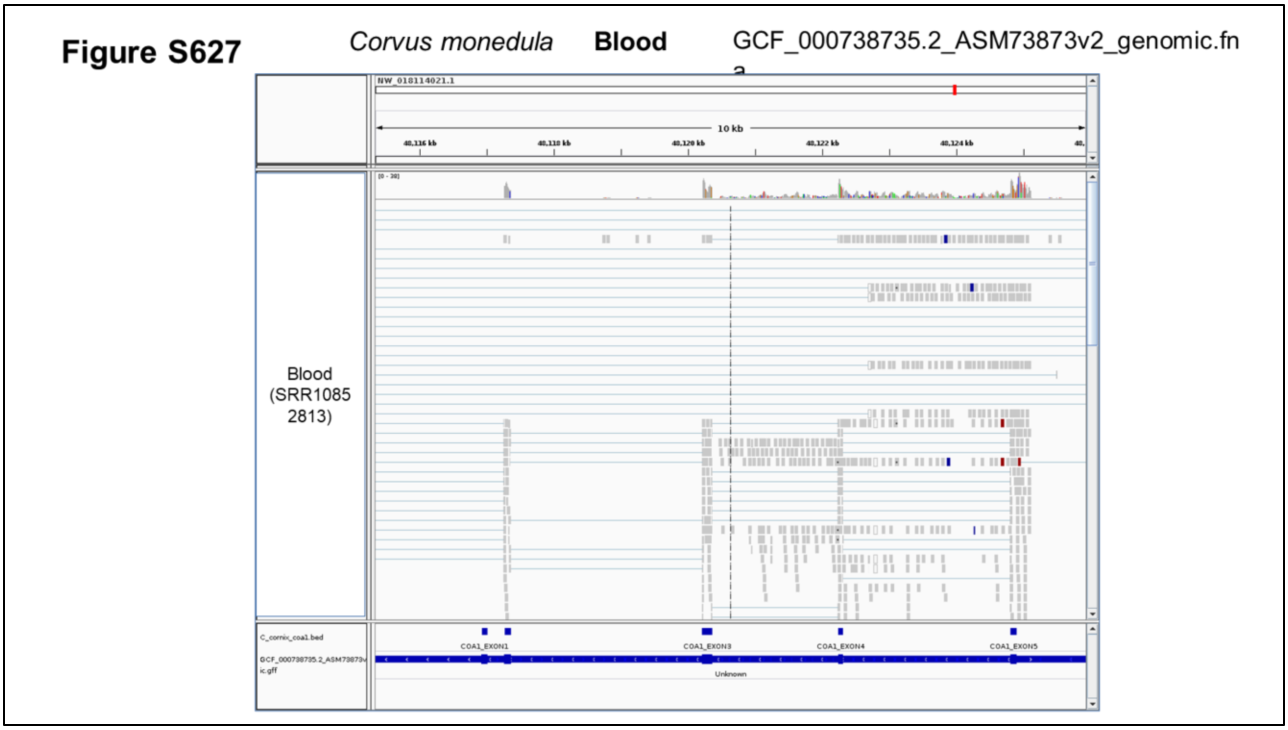

Figure S627: Screenshot of RNA-seq reads aligned to each of the putative locations of the five *COA1* exons in western Jackdaw (*Corvus monedula*). Gray boxes represent reads aligned to the genome and thin lines connecting these boxes represent the spliced read alignments. Datasets from blood (SRR10852813) have been mapped to the GCF_000738735.2_ASM73873v2_genomic.fna genome. Locations of each of the exons are depicted as bed record in the bottom row.

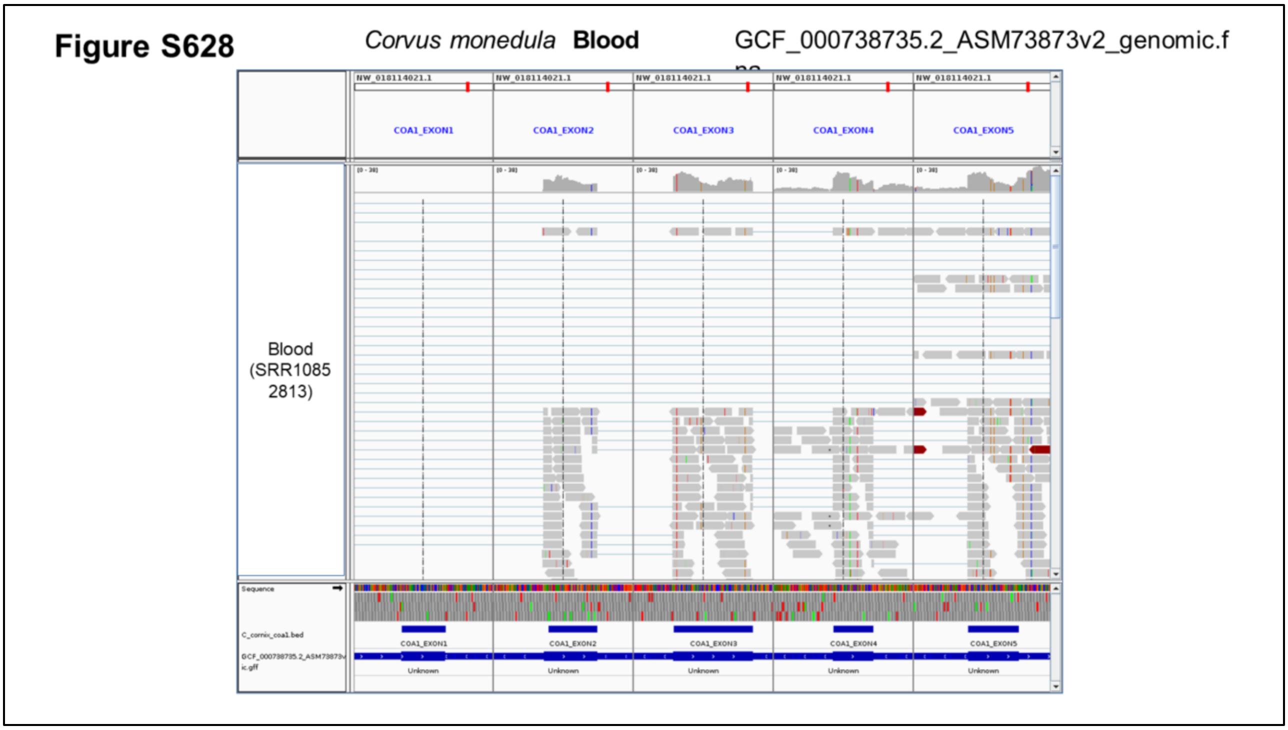

Figure S628: A zoomed in view of each of the *COA1* exons shown in **Supplementary Fig. S627**.

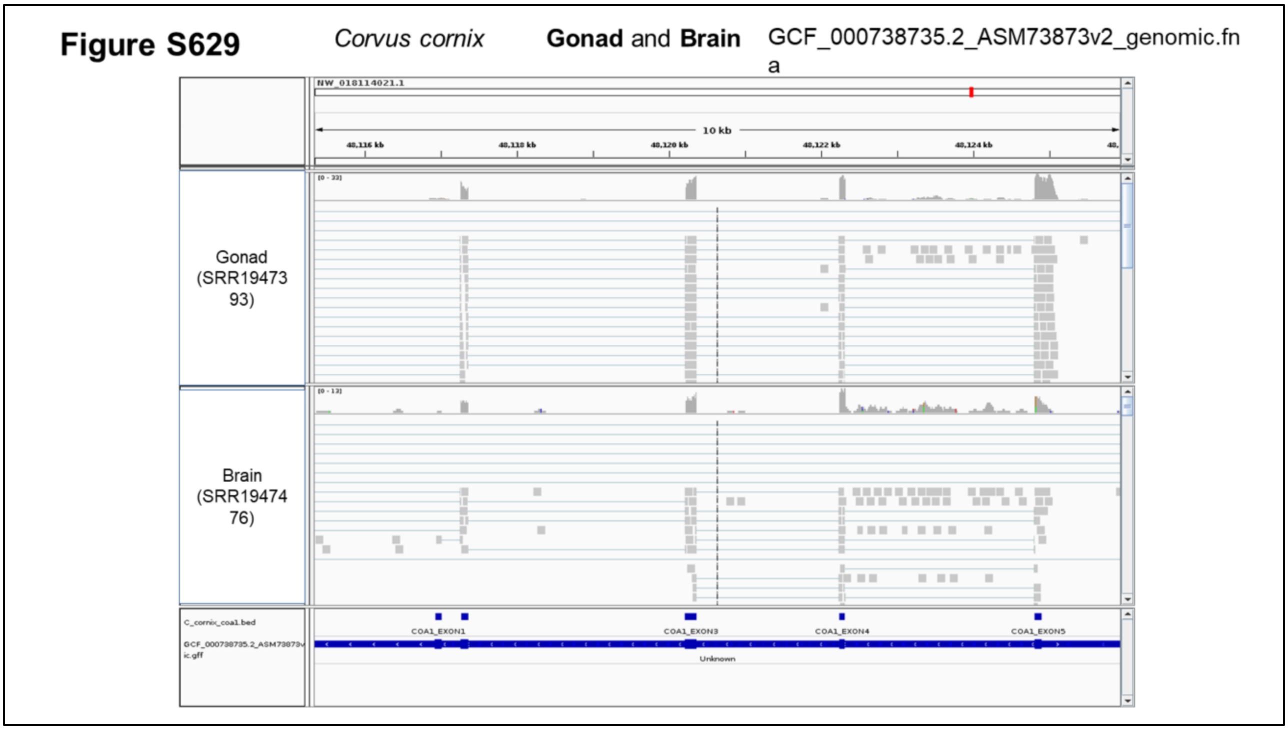

Figure S629: Screenshot of RNA-seq reads aligned to each of the putative locations of the four *COA1* exons in hooded crow (*Corvus cornix*). Gray boxes represent reads aligned to the genome and thin lines connecting these boxes represent the spliced read alignments. Datasets from gonad (SRR1947393) and brain (SRR1947476) have been mapped to the GCF_000738735.2_ASM73873v2_genomic.fna genome. Locations of each of the exons are depicted as bed record in the bottom row.

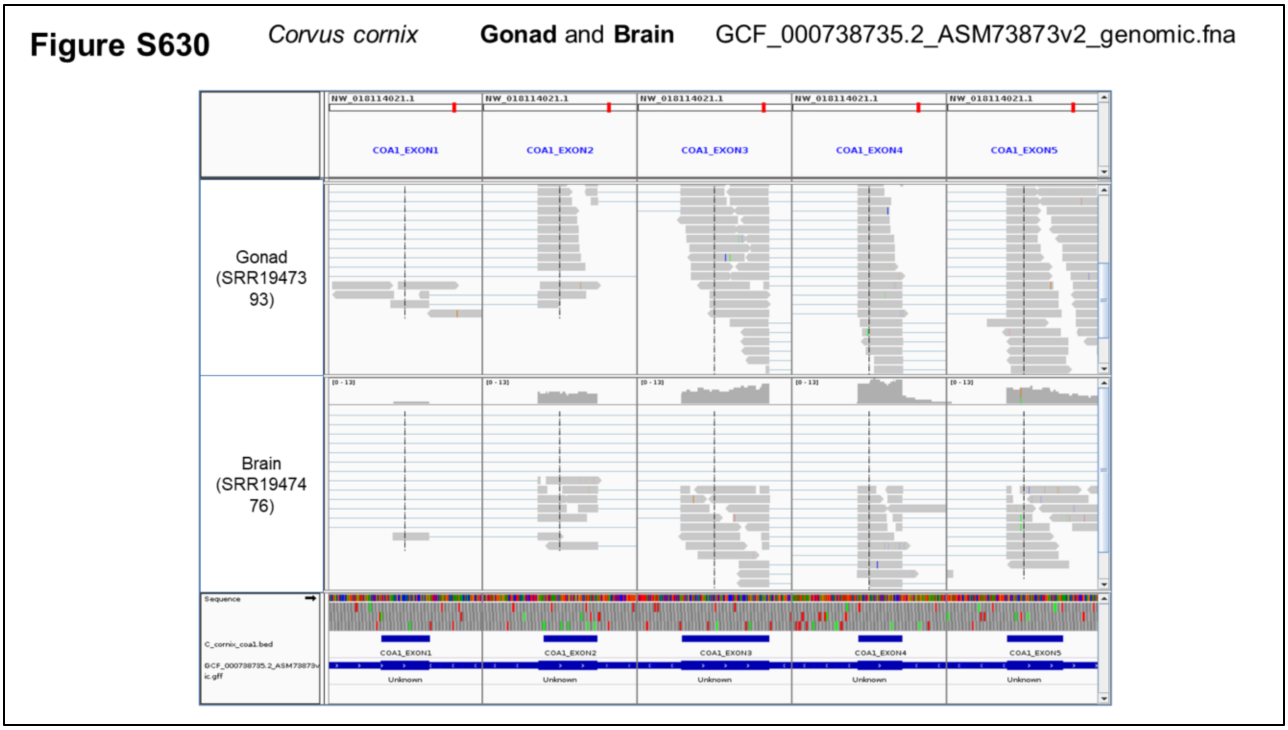

Figure S630: A zoomed in view of each of the *COA1* exons shown in **Supplementary Fig. S629**.

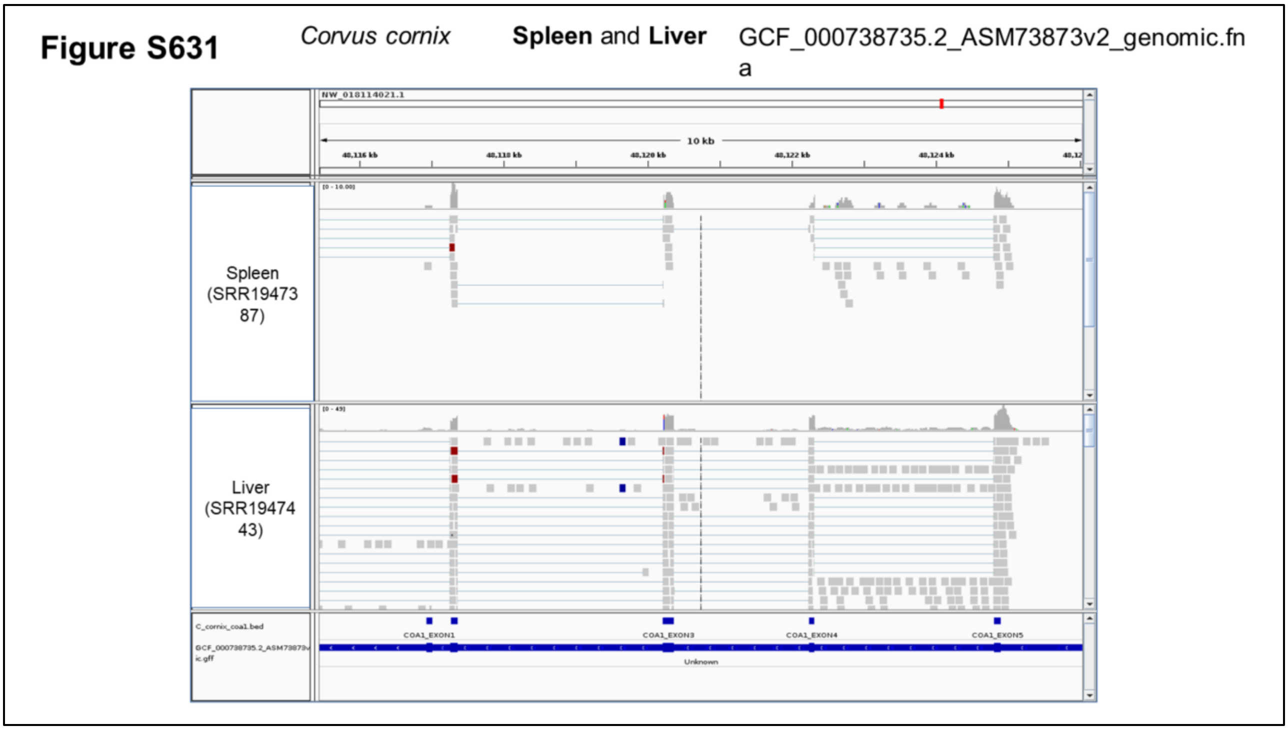

Figure S631: Screenshot of RNA-seq reads aligned to each of the putative locations of the four *COA1* exons in hooded crow (*Corvus cornix*). Gray boxes represent reads aligned to the genome and thin lines connecting these boxes represent the spliced read alignments. Datasets from spleen (SRR1947387) and liver (SRR1947443) have been mapped to the GCF_000738735.2_ASM73873v2_genomic.fna genome. Locations of each of the exons are depicted as bed record in the bottom row.

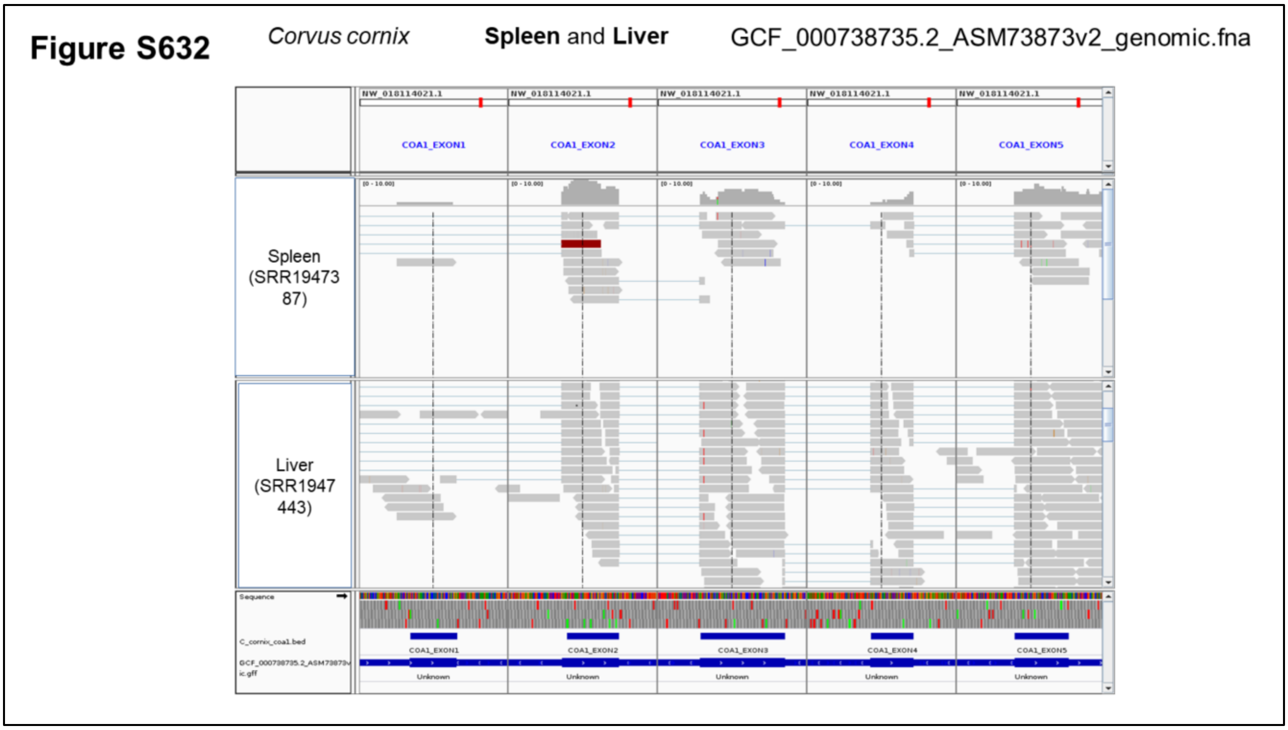

Figure S632: A zoomed in view of each of the *COA1* exons shown in **Supplementary Fig. S631**.

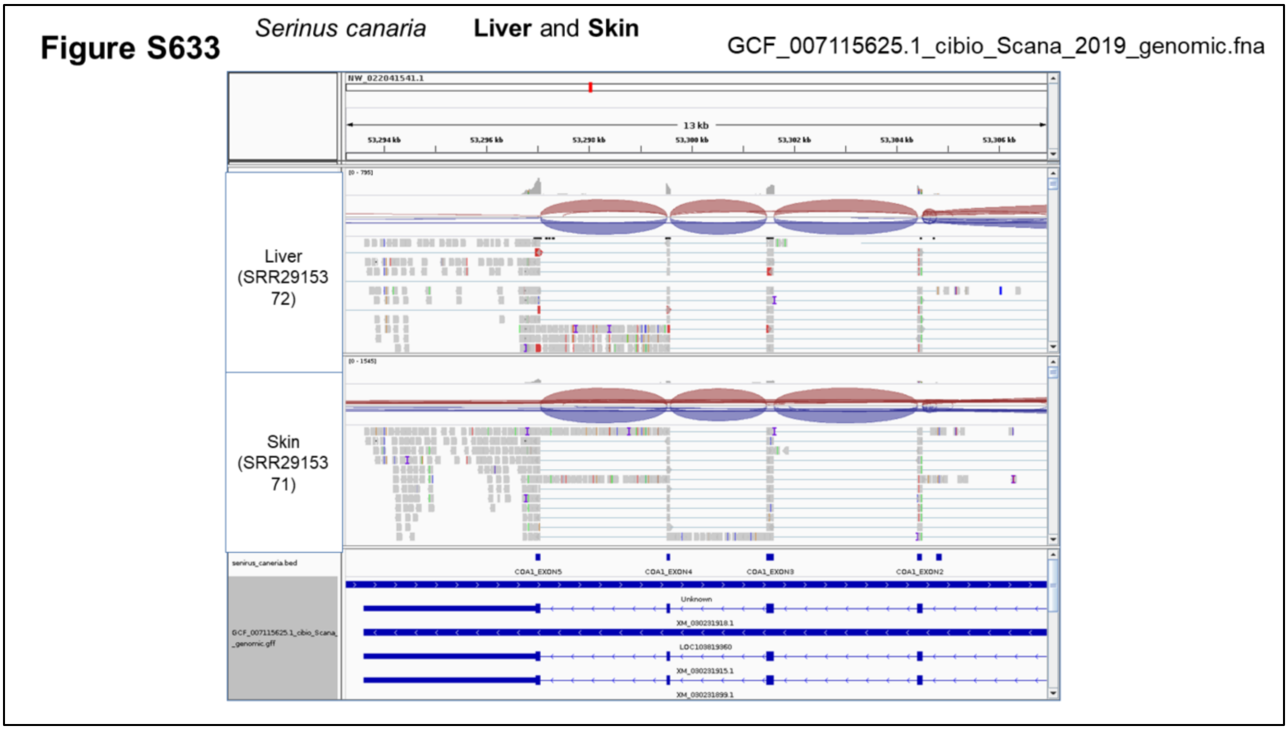

Figure S633: Screenshot of RNA-seq reads aligned to each of the putative locations of the four *COA1* exons in common canary (*Serinus canaria*). Gray boxes represent reads aligned to the genome and thin lines connecting these boxes represent the spliced read alignments. Datasets from liver (SRR152971) and skin (SRR152972) have been mapped to the GCF_007115625.1_cibio_Scana_2019_genomic.fna genome. Locations of each of the exons are depicted as bed record in the bottom row.

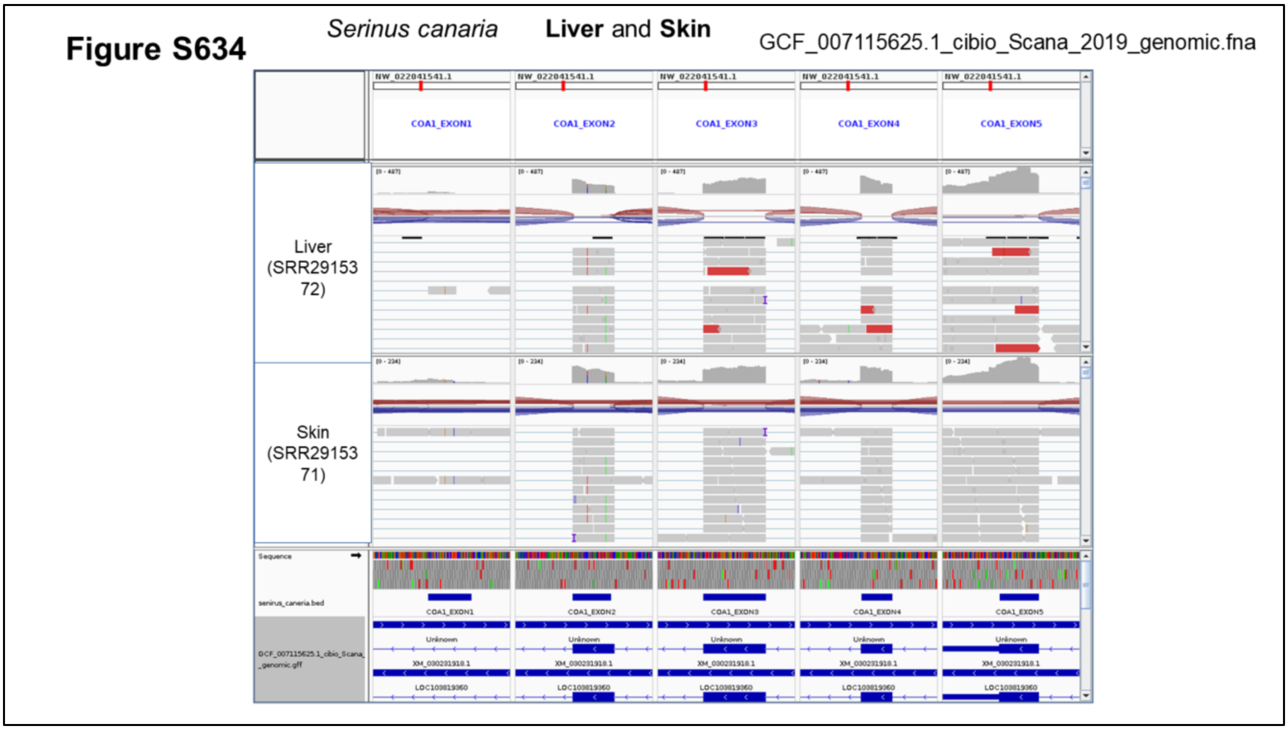

Figure S634: A zoomed in view of each of the *COA1* exons shown in **Supplementary Fig. S633**.

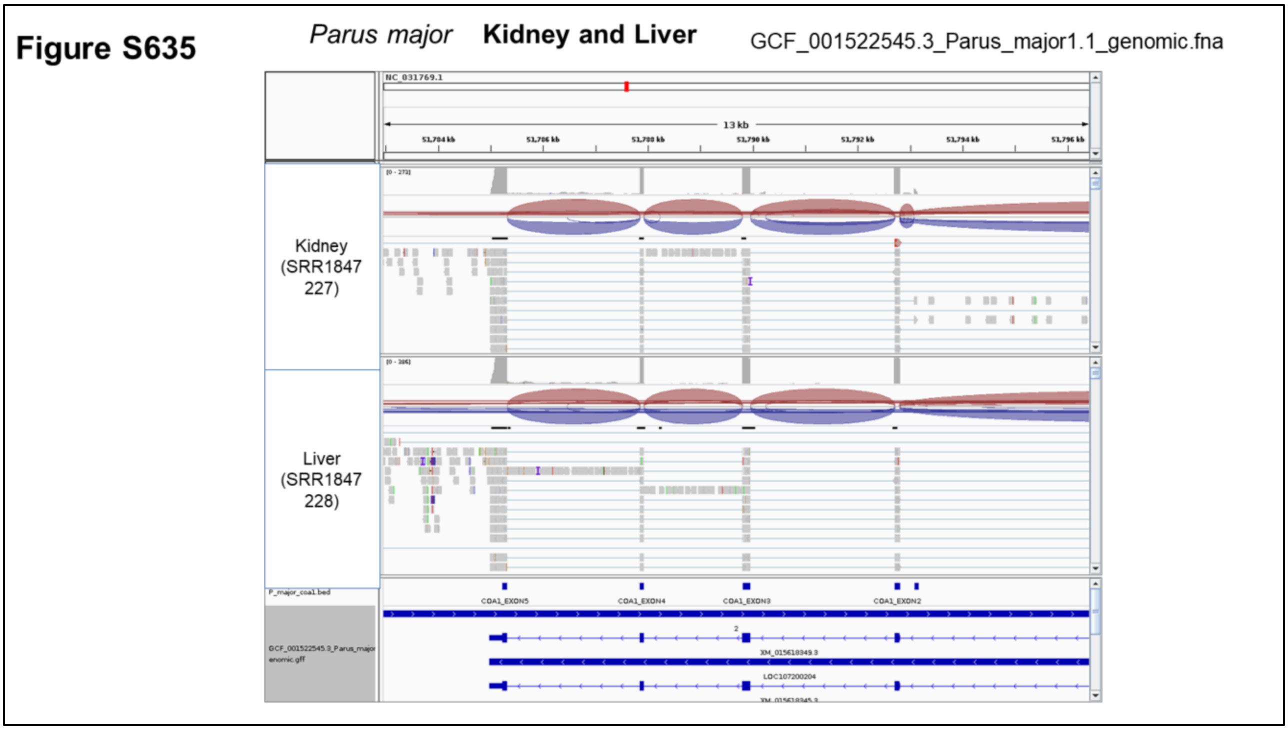

Figure S635: Screenshot of RNA-seq reads aligned to each of the putative locations of the four *COA1* exons in great tit (*Parus major*). Gray boxes represent reads aligned to the genome and thin lines connecting these boxes represent the spliced read alignments. Datasets from kidney (SRR1847227) and liver (SRR1847228) have been mapped to the GCF_001522545.3_Parus_major1.1_genomic.fna genome. Locations of each of the exons are depicted as bed record in the bottom row.

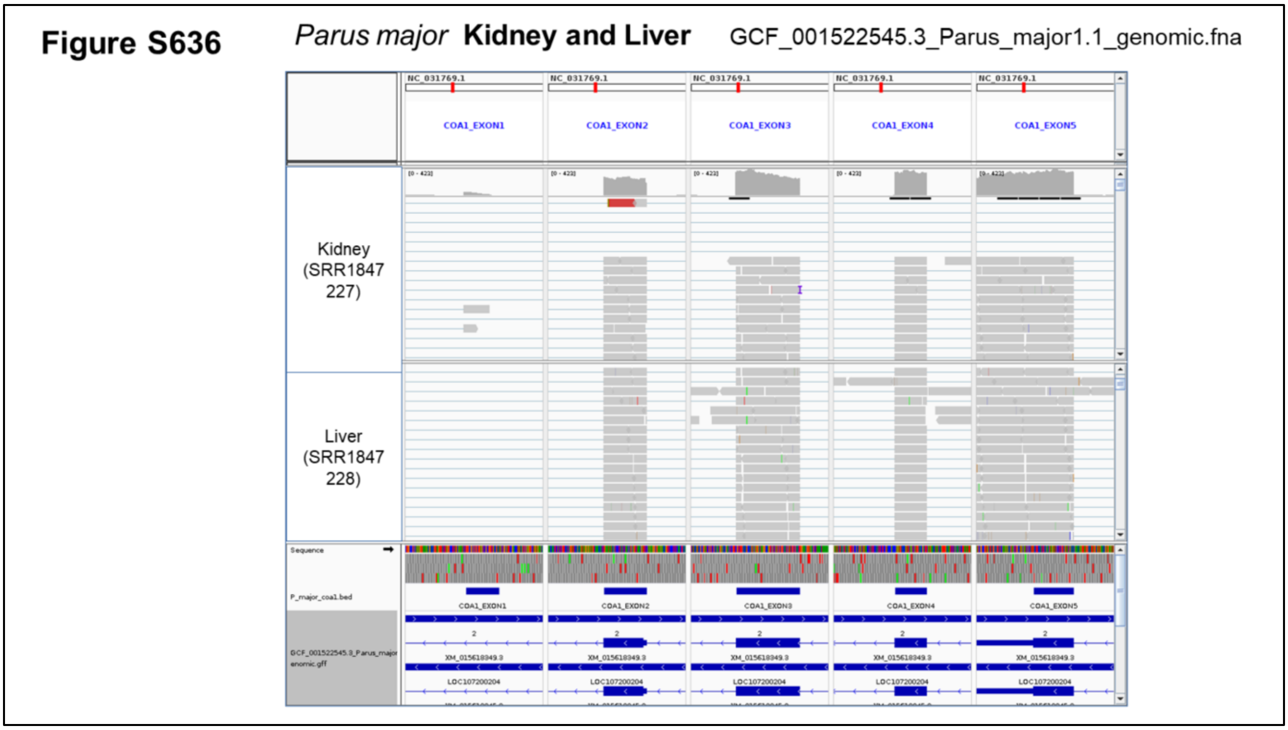

Figure S636: A zoomed in view of each of the *COA1* exons shown in **Supplementary Fig. S635**.

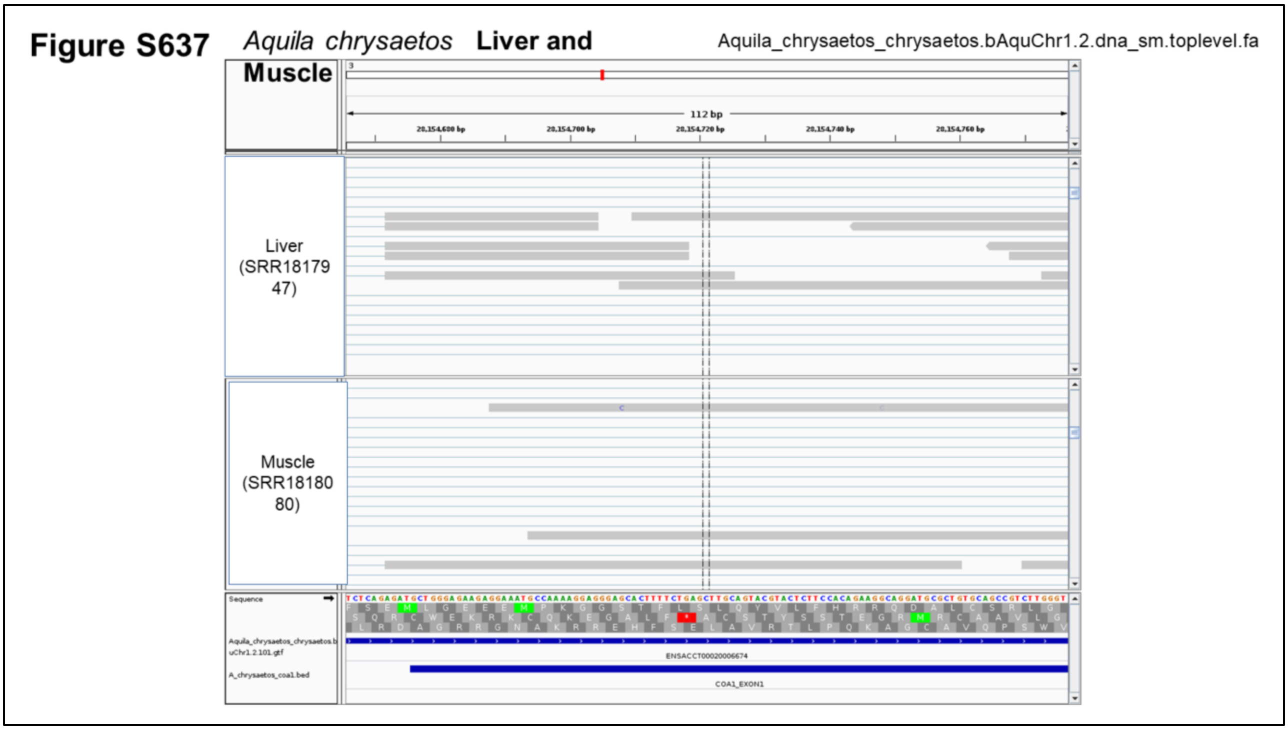

Figure S637: Less expression and in-frame stop codon in exon 1 of the six exon annotation of *COA1* of golden eagle (*Aquila chrysaetos*).

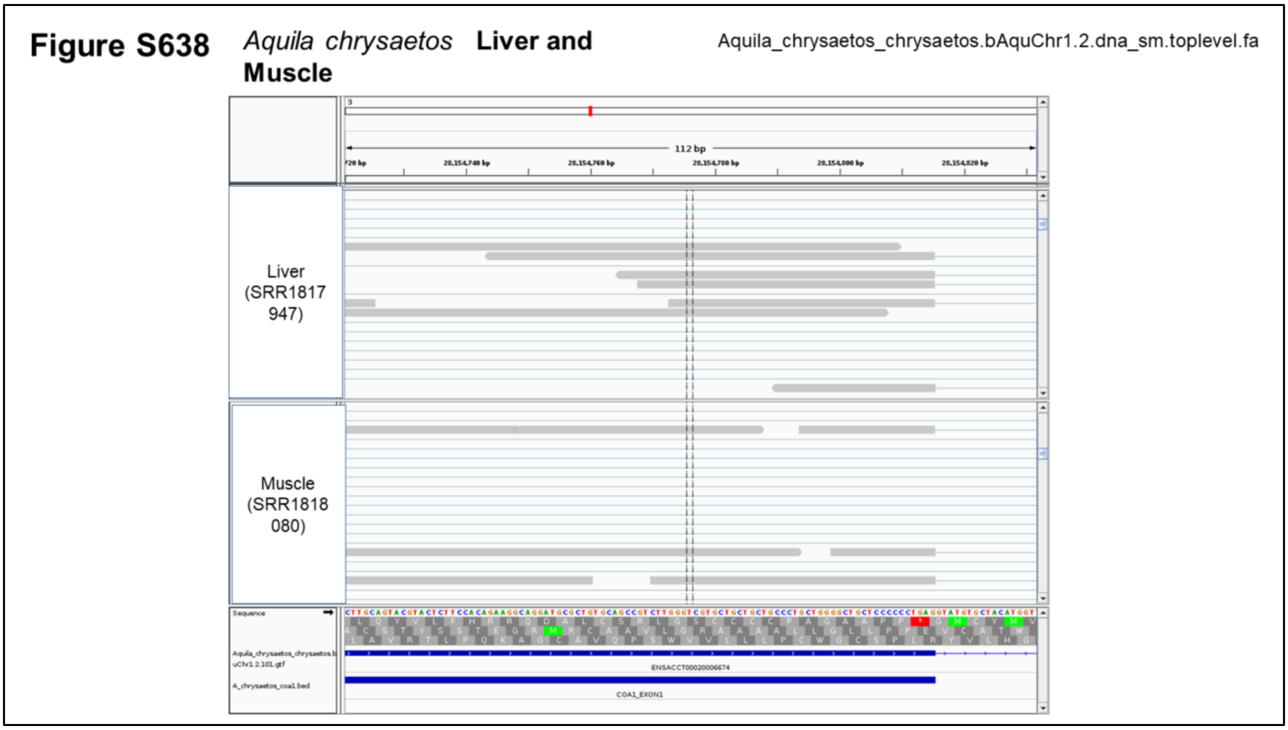

Figure S638: Continuation of **Supplementary Fig. S637** showing low expression in exon 1 of the six exon annotation of *COA1* of golden eagle (*Aquila chrysaetos*).

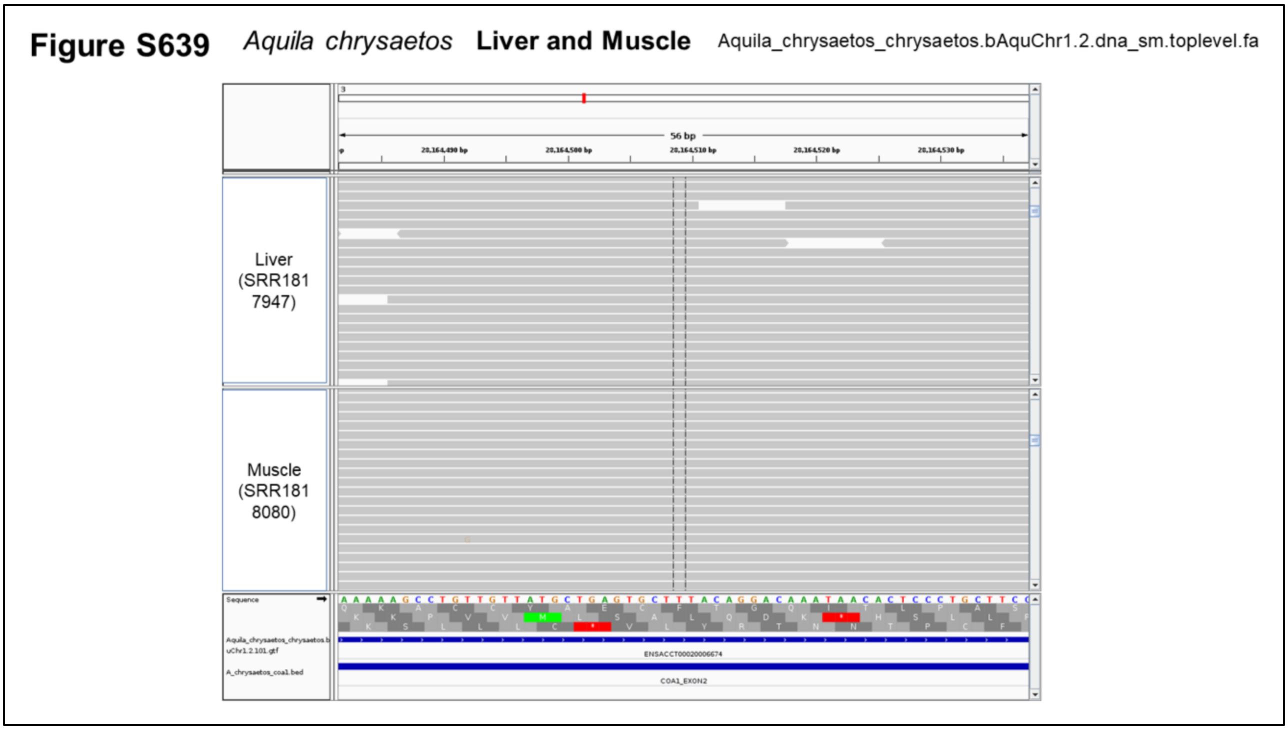

Figure S639: In-frame stop codon in exon 2 of the six exon annotation of *COA1* of golden eagle (*Aquila chrysaetos*).

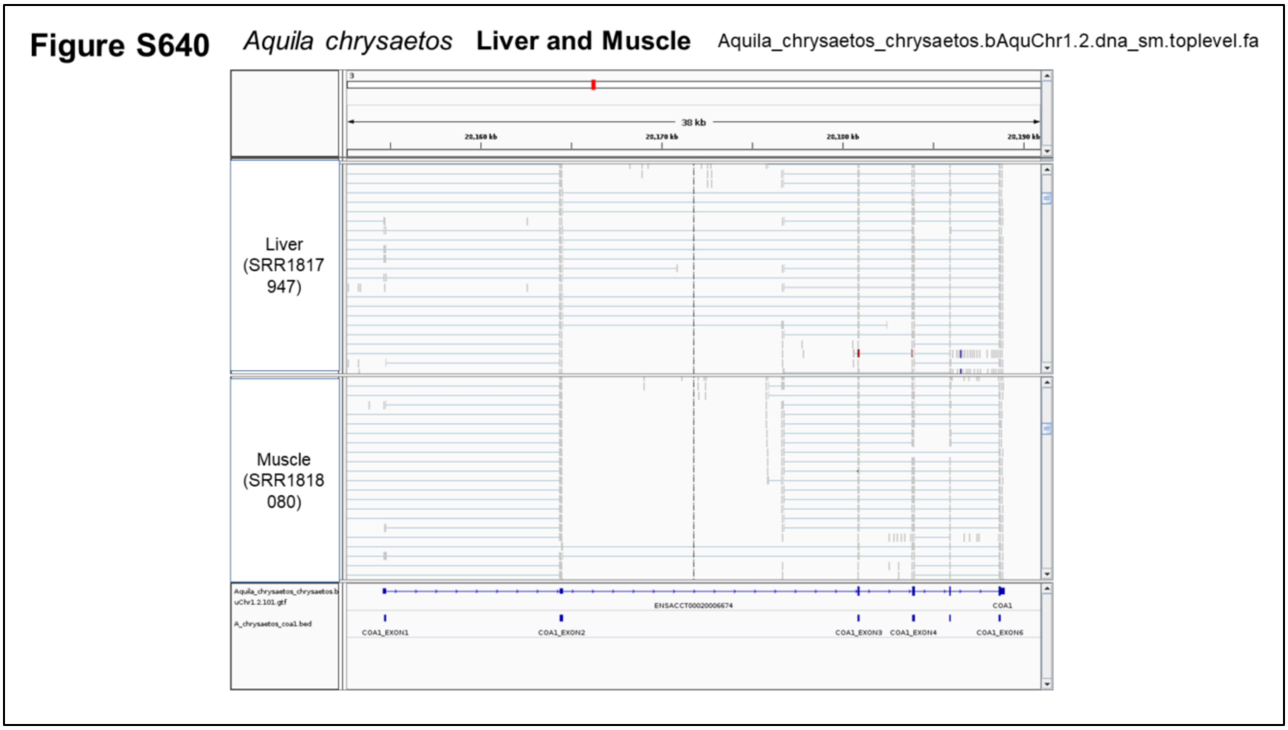

Figure S640: Screenshot of RNA-seq reads aligned to each of the putative locations of the six *COA1* exons in golden eagle (*Aquila chrysaetos*). Gray boxes represent reads aligned to the genome and thin lines connecting these boxes represent the spliced read alignments. Datasets from liver (SRR1817947) and muscle (SRR1818080) have been mapped to the Aquila_chrysaetos_chrysaetos.bAquChr1.2.dna_sm.toplevel.fa genome. Locations of each of the exons are depicted as bed record in the bottom row.

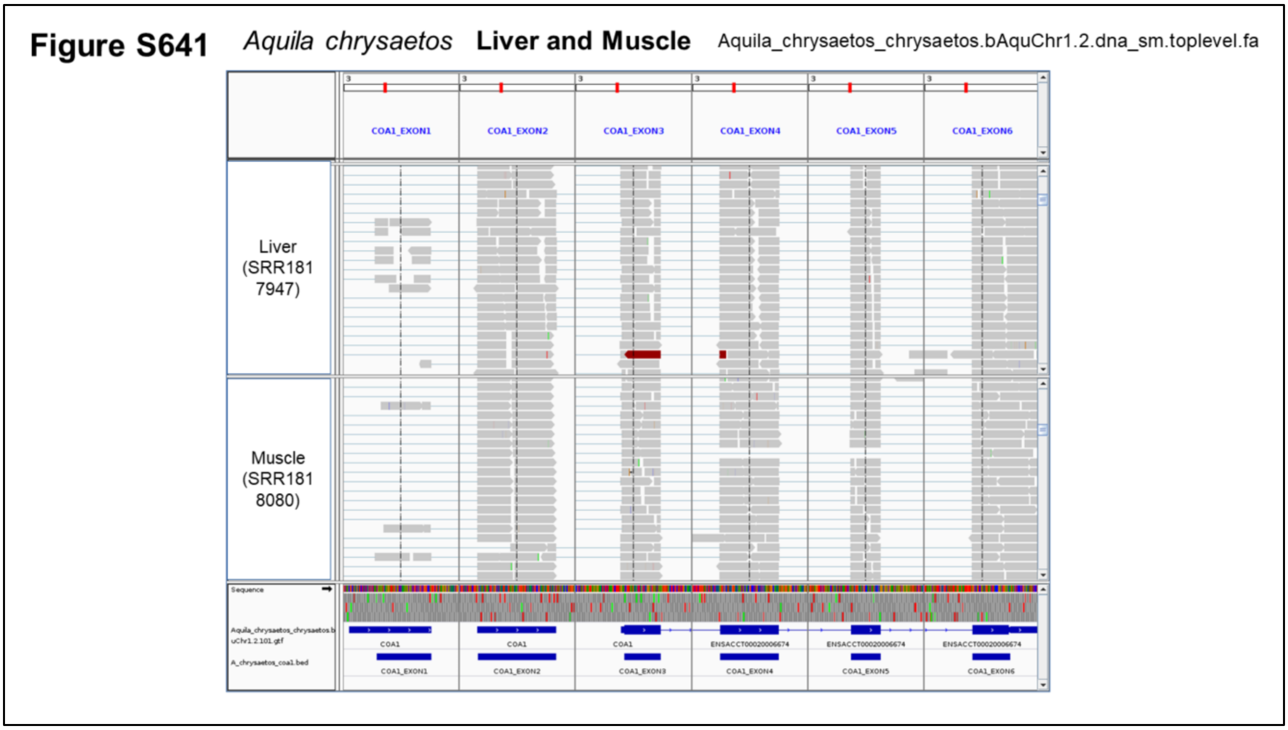

Figure S641: A zoomed in view of each of the *COA1* exons shown in **Supplementary Fig. S640**.

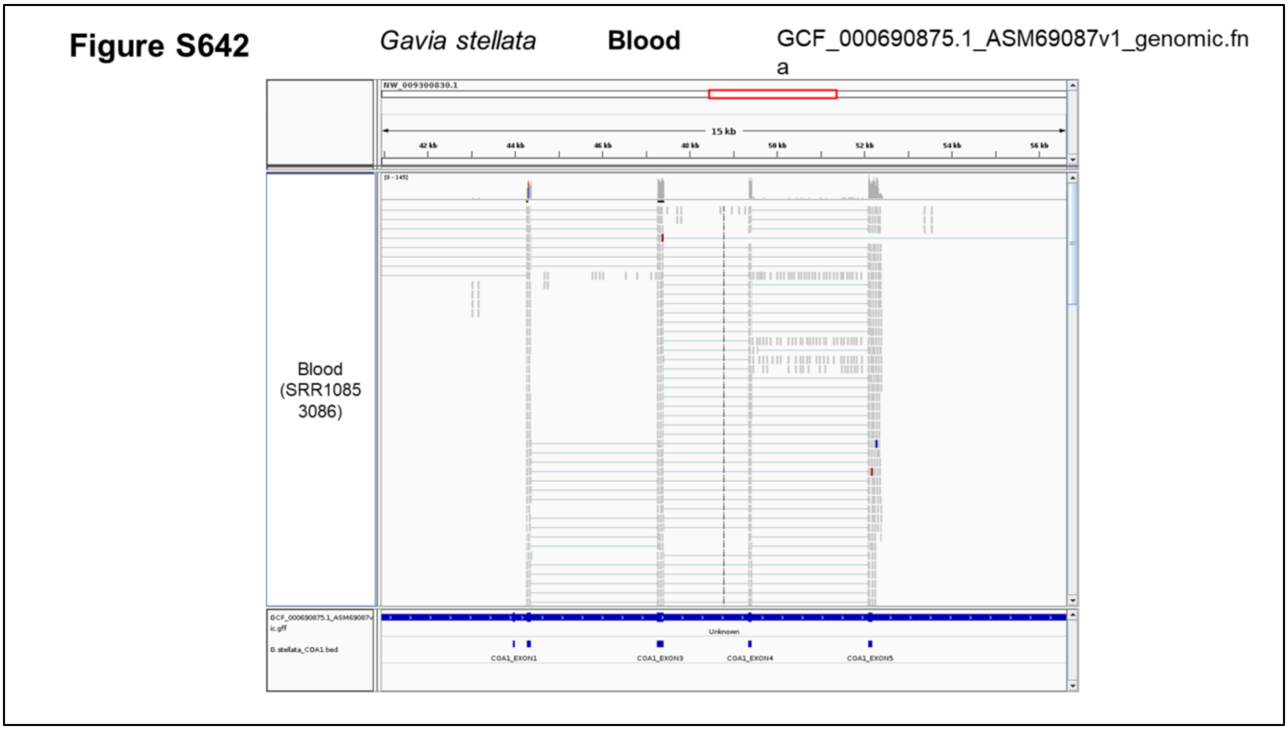

Figure S642: Screenshot of RNA-seq reads aligned to each of the putative locations of the four *COA1* exons in red-throated loon (*Gavia stellata*). Gray boxes represent reads aligned to the genome and thin lines connecting these boxes represent the spliced read alignments. Datasets from blood tissue (SRR10853086) have been mapped to the GCF_000690875.1_ASM69087v1_genomic.fna genome. Locations of each of the exons are depicted as bed record in the bottom row.

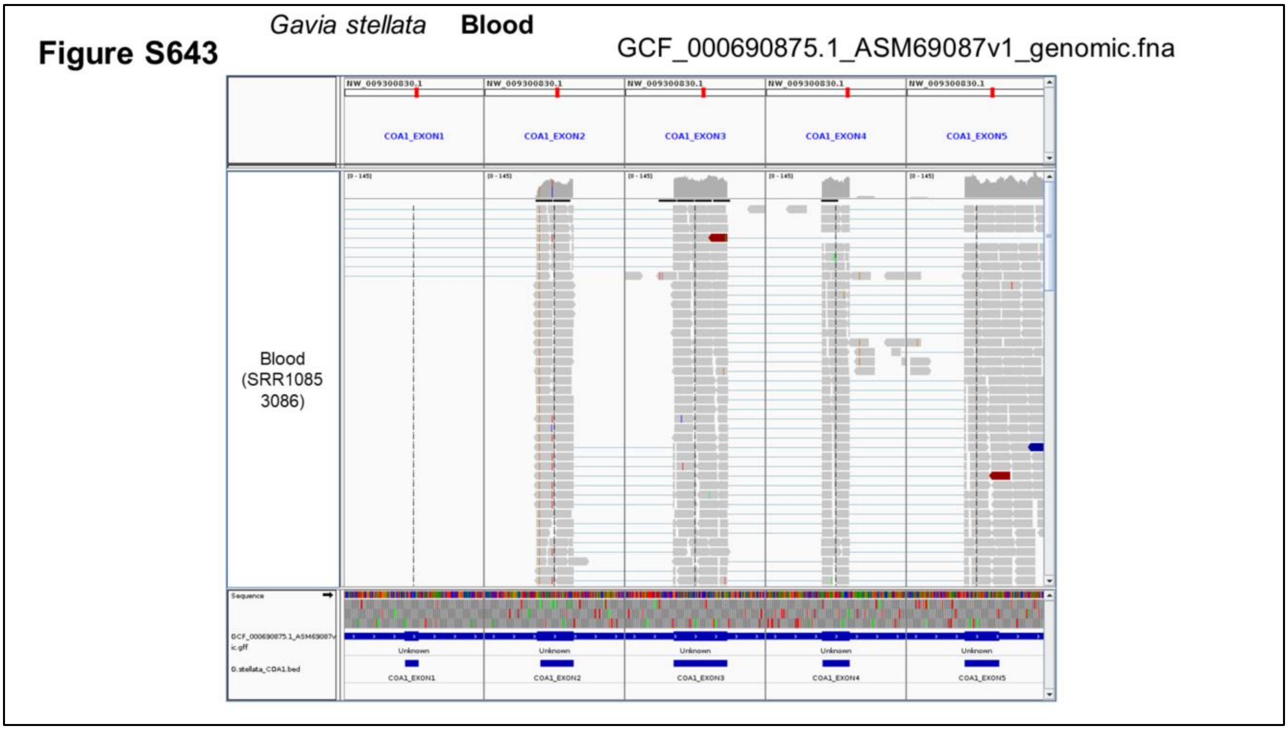

Figure S643: A zoomed in view of each of the *COA1* exons shown in **Supplementary Fig. S642**.

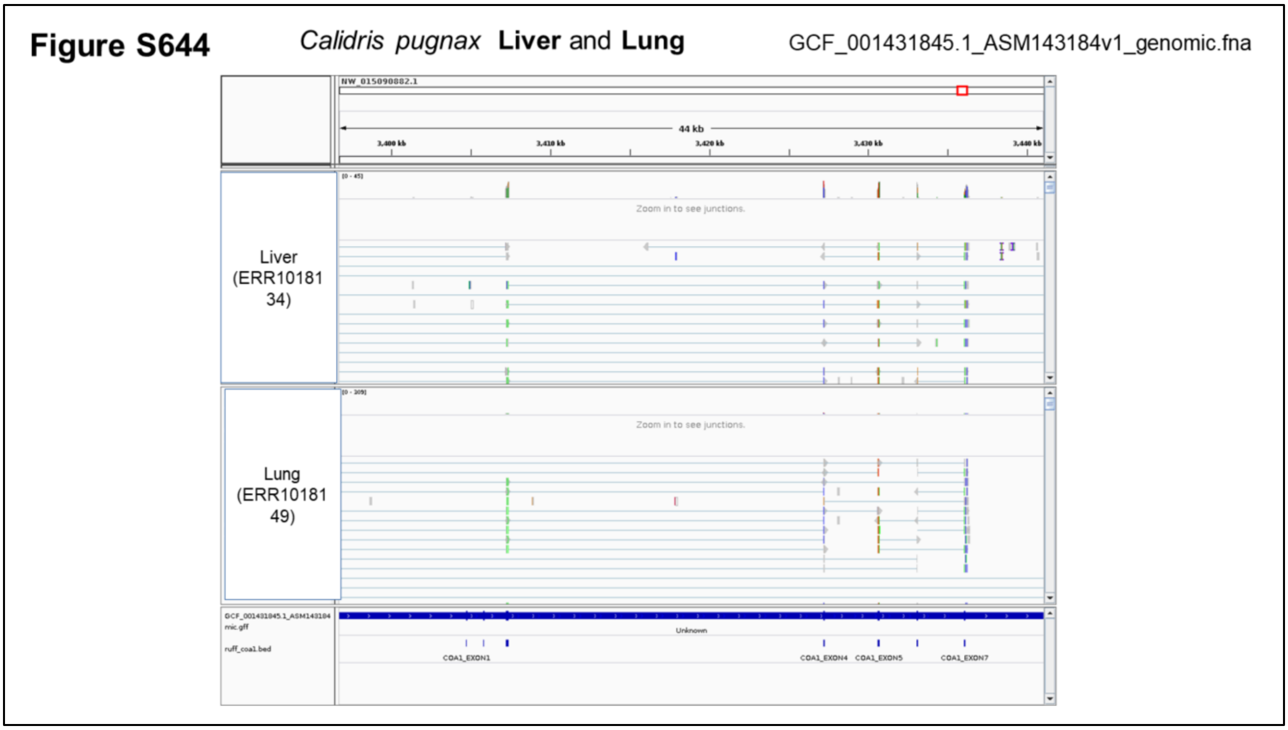

Figure S644: Screenshot of RNA-seq reads aligned to each of the putative locations of the seven annotated *COA1* exons in ruff (*Calidris pugnax*). Gray boxes represent reads aligned to the genome and thin lines connecting these boxes represent the spliced read alignments. Datasets from liver (ERR1018134) and lung (ERR1018149) have been mapped to the GCF_001431845.1_ASM143184v1_genomic.fna genome. Locations of each of the exons are depicted as bed record in the bottom row.

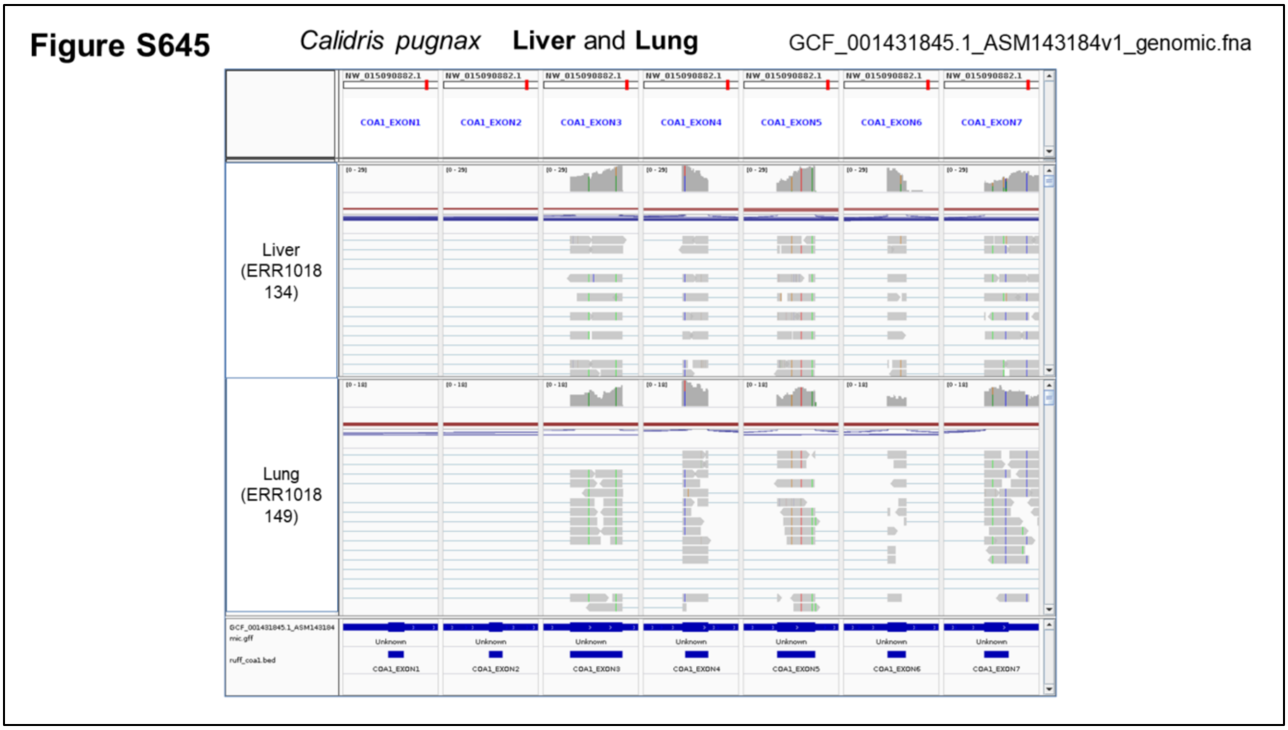

Figure S645: A zoomed in view of each of the *COA1* exons shown in **Supplementary Fig. S644**.

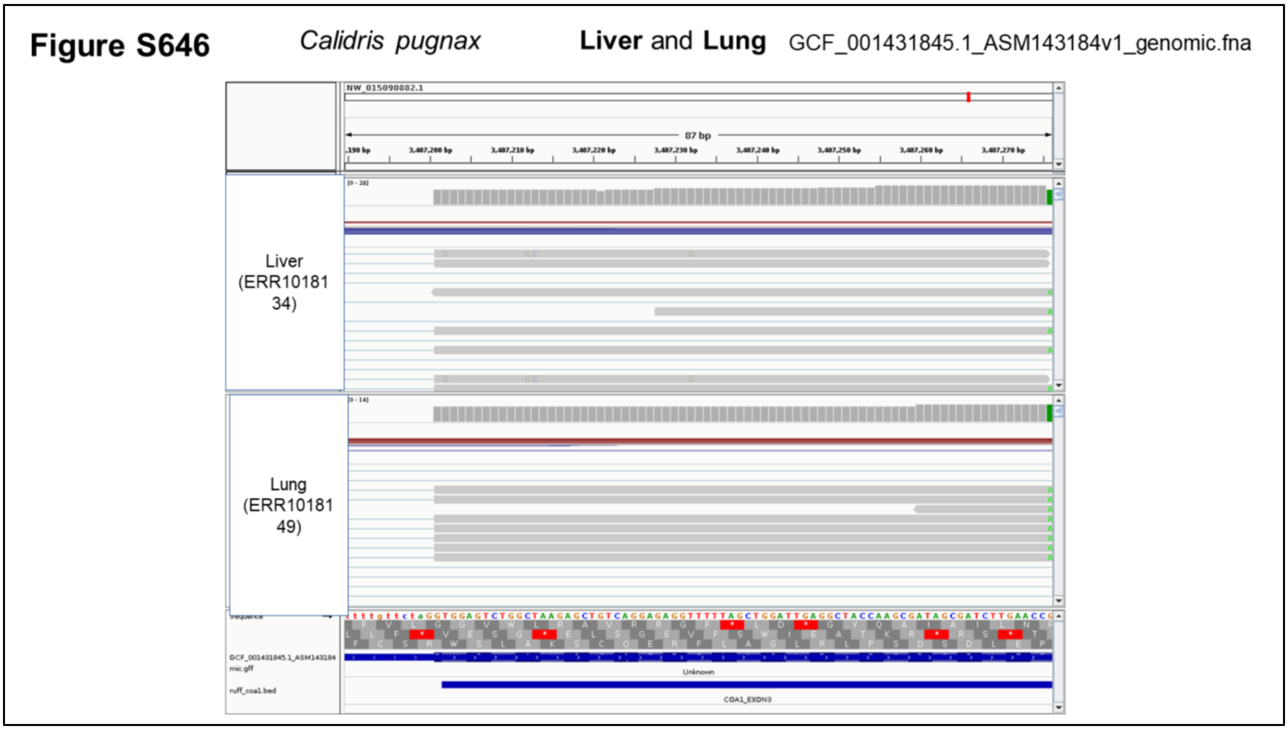

Figure S646: Exon-3 of the seven exon annotation of *COA1* in ruff (*Calidris pugnax*) did not have any start codon.

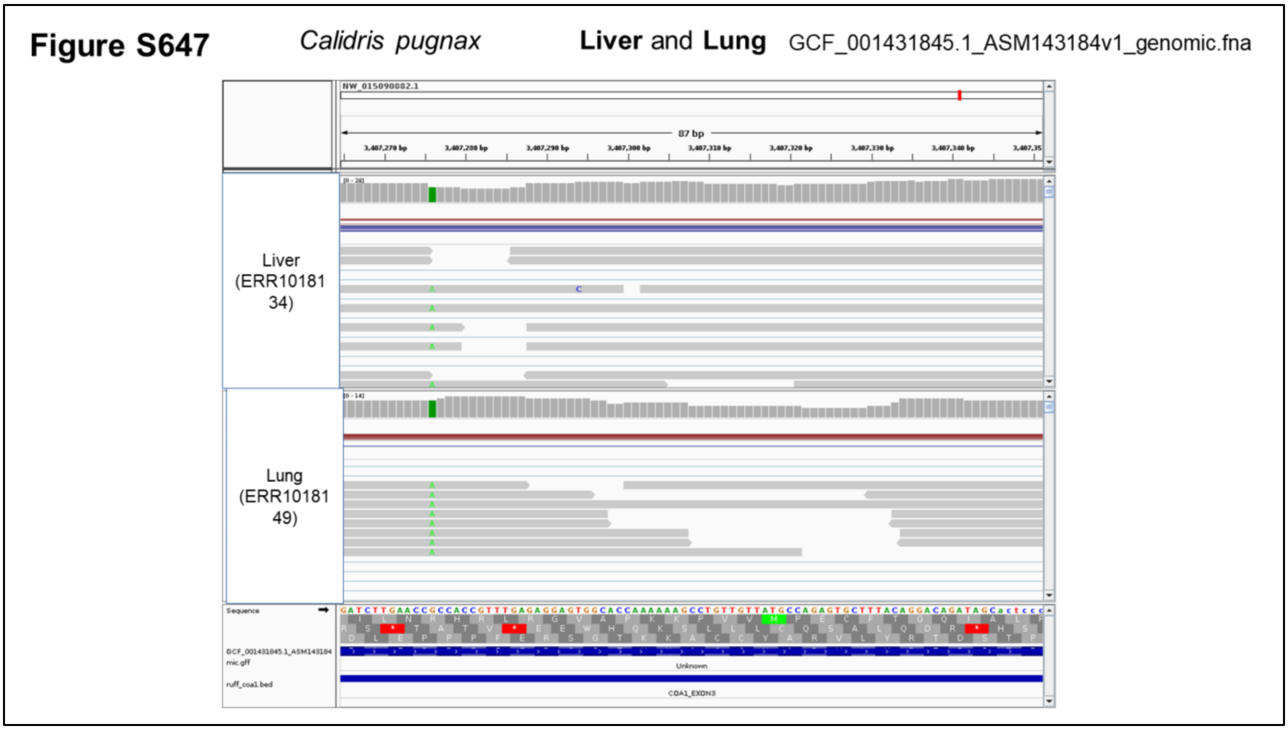

Figure S647: Continuation of **Supplementary Fig. S646**.

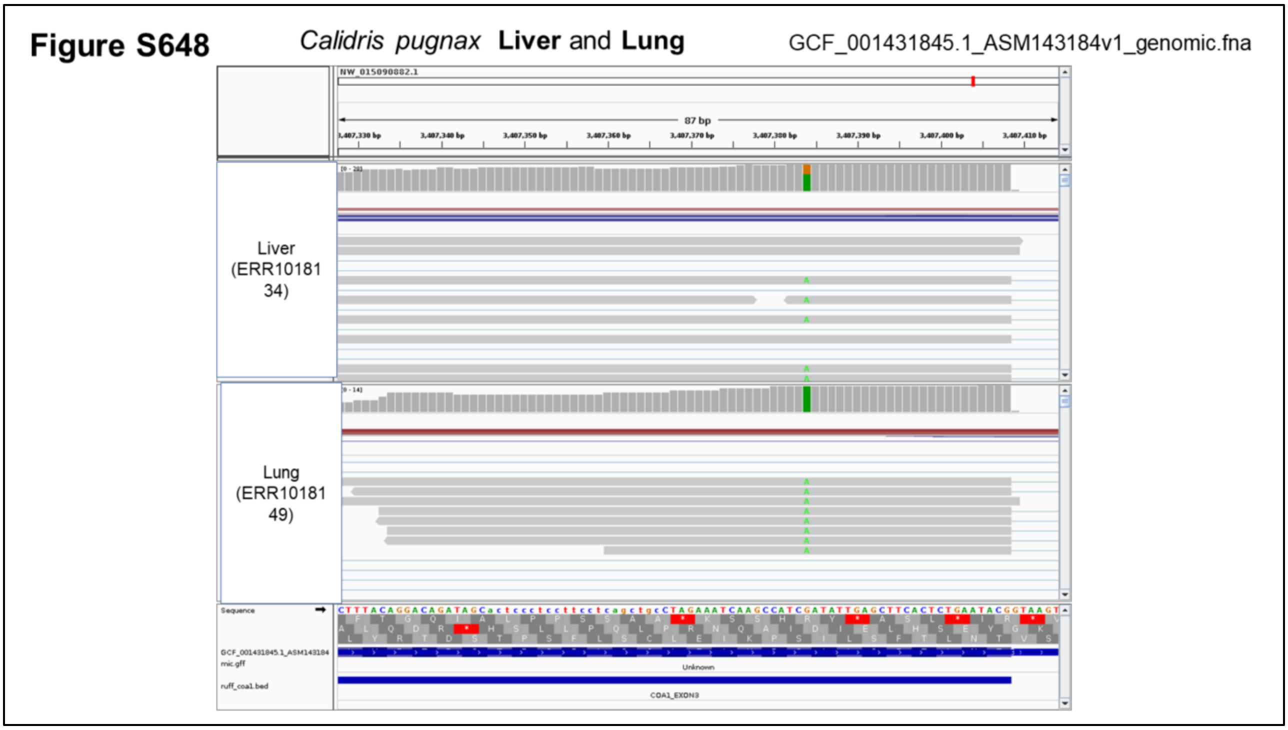

Figure S648: Continuation of **Supplementary Fig. S647**.

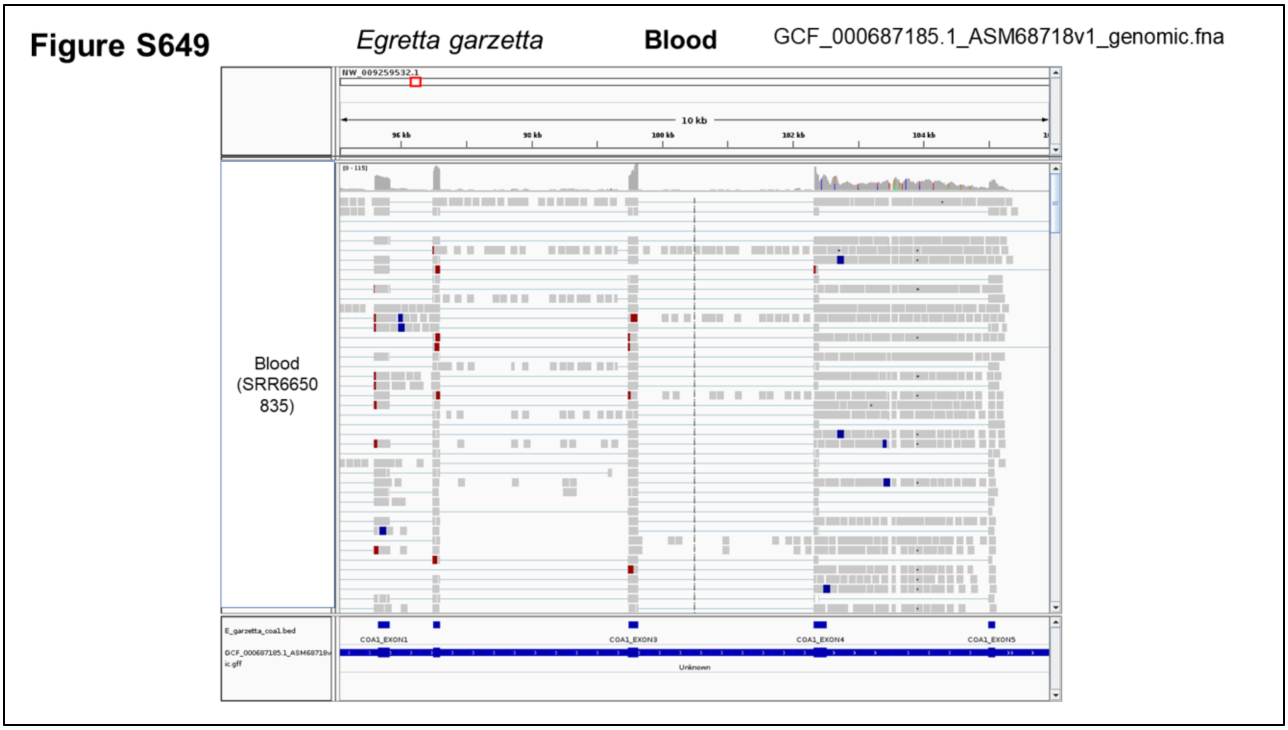

Figure S649: Screenshot of RNA-seq reads aligned to each of the putative locations of the five *COA1* exons in little egret (*Egretta garzetta*). Gray boxes represent reads aligned to the genome and thin lines connecting these boxes represent the spliced read alignments. Datasets from blood (SRR6650835) have been mapped to the GCF_000687185.1_ASM68718v1_genomic.fna genome. Locations of each of the exons are depicted as bed record in the bottom row.

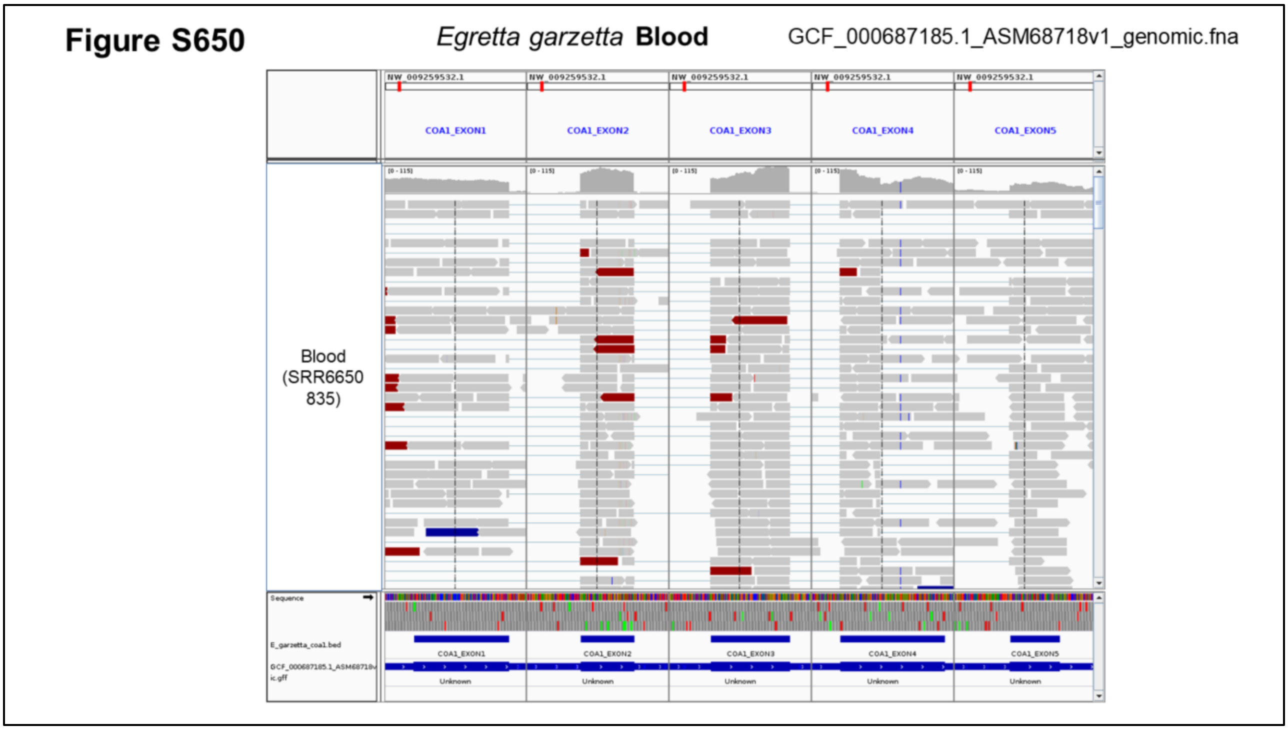

Figure S650: A zoomed in view of each of the *COA1* exons shown in **Supplementary Fig. S649**.

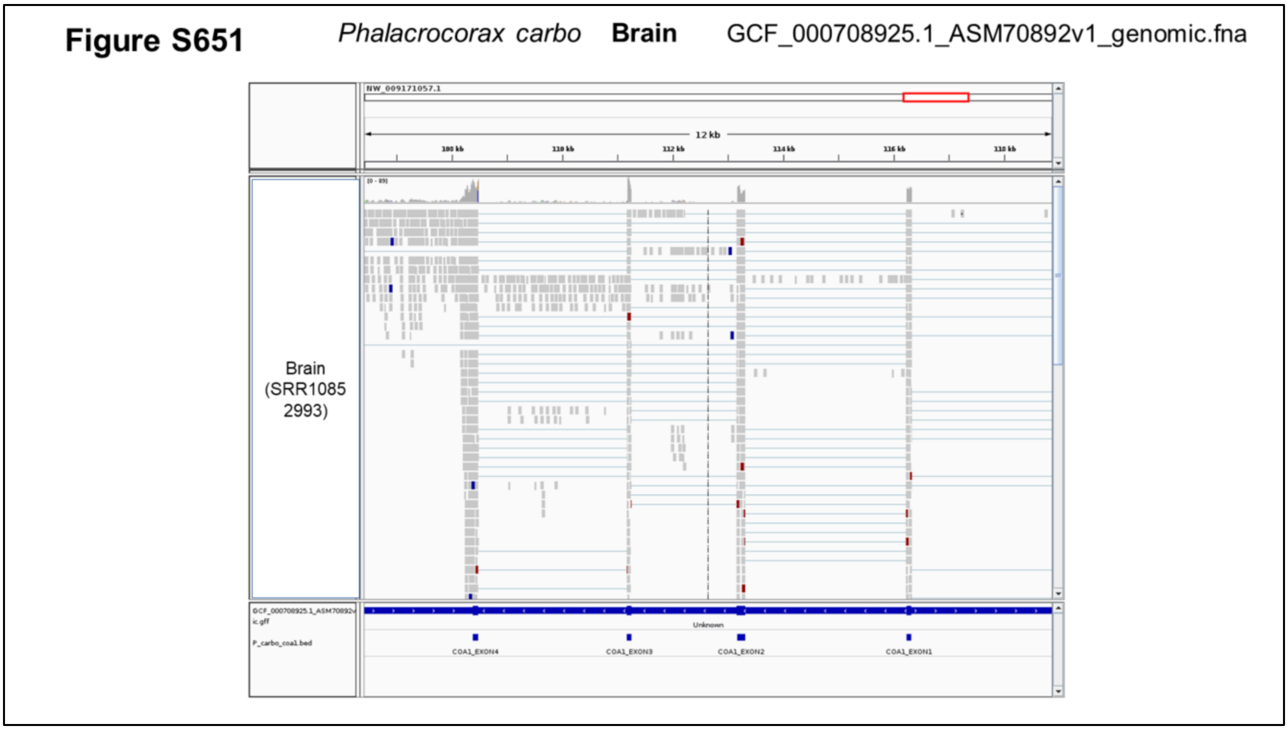

Figure S651: Screenshot of RNA-seq reads aligned to each of the putative locations of the four *COA1* exons in great cormorant (*Phalacrocorax carbo*). Gray boxes represent reads aligned to the genome and thin lines connecting these boxes represent the spliced read alignments. Datasets from brain (SRR10852993) have been mapped to the GCF_000708925.1_ASM70892v1_genomic.fna genome. Locations of each of the exons are depicted as bed record in the bottom row.

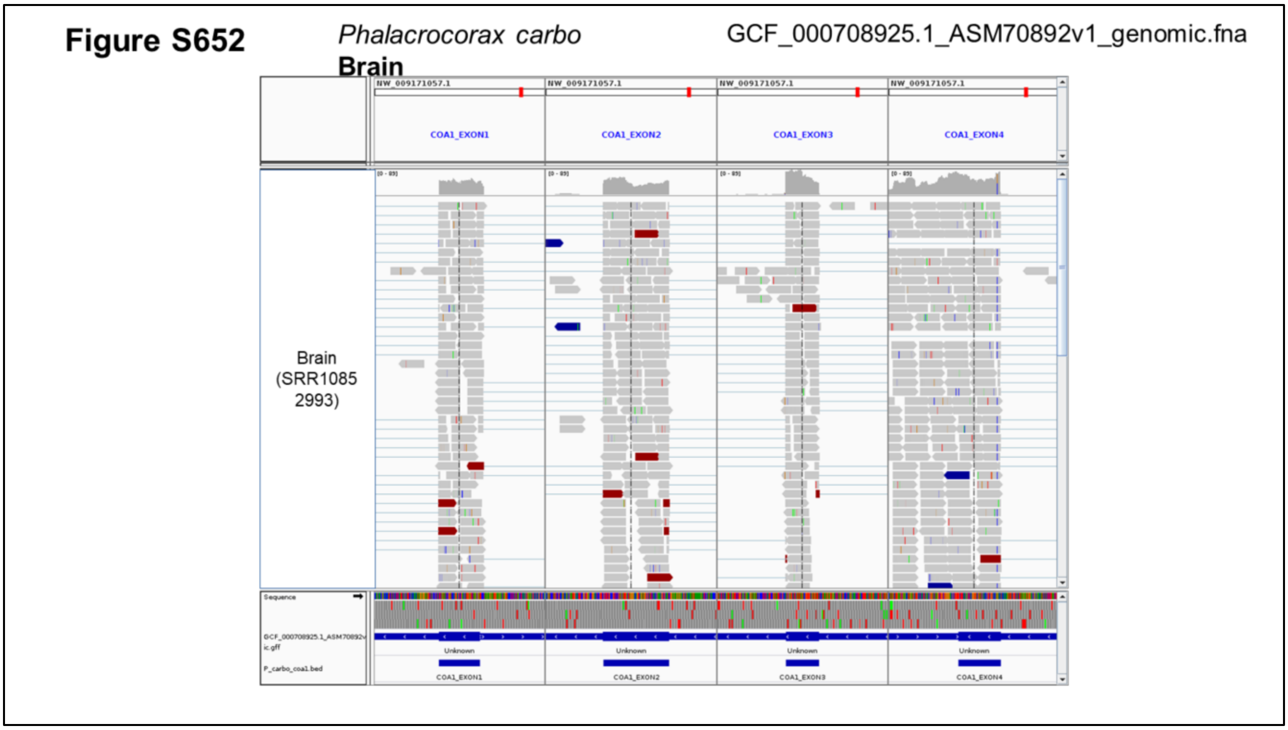

Figure S652: A zoomed in view of each of the *COA1* exons shown in **Supplementary Fig. S651**.

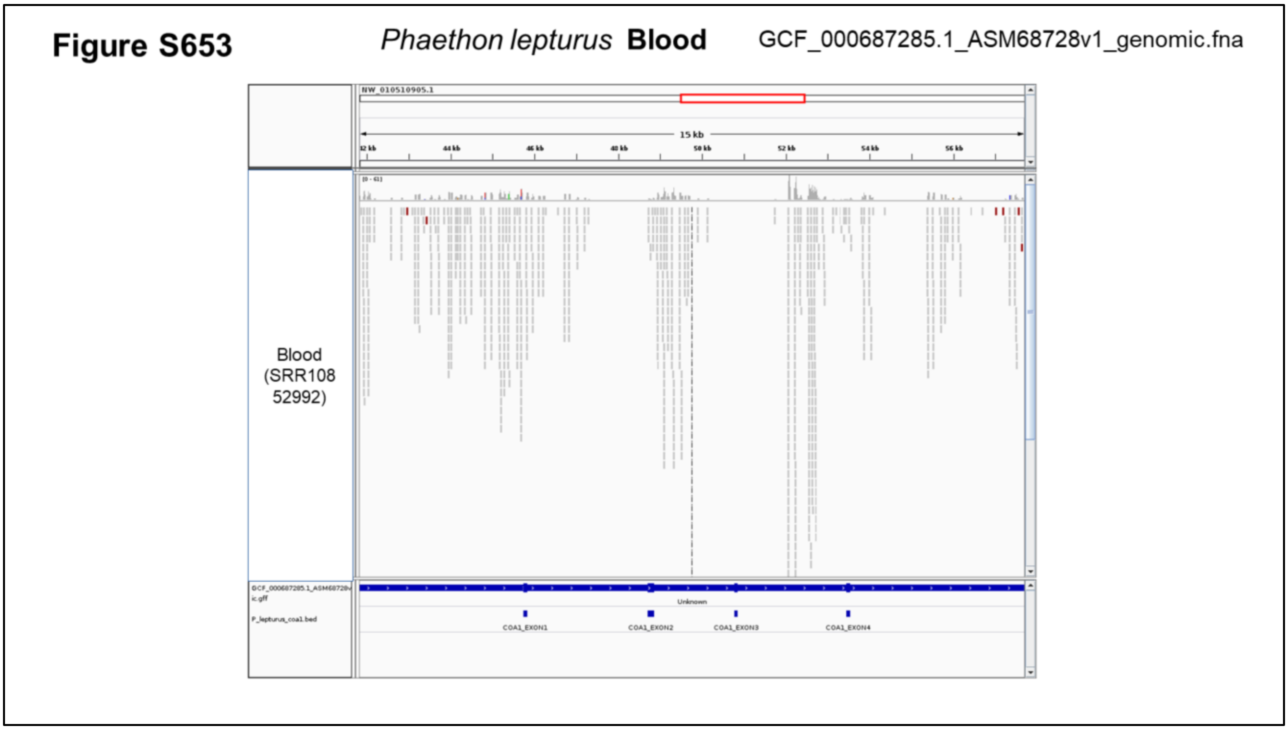

Figure S653: Screenshot of RNA-seq reads aligned to each of the putative locations of the four *COA1* exons in white-tailed tropicbird (*Phaethon lepturus*). Gray boxes represent reads aligned to the genome and thin lines connecting these boxes represent the spliced read alignments. Datasets from blood (SRR10852992) have been mapped to the GCF_000687285.1_ASM68728v1_genomic.fna genome. Locations of each of the exons are depicted as bed record in the bottom row.

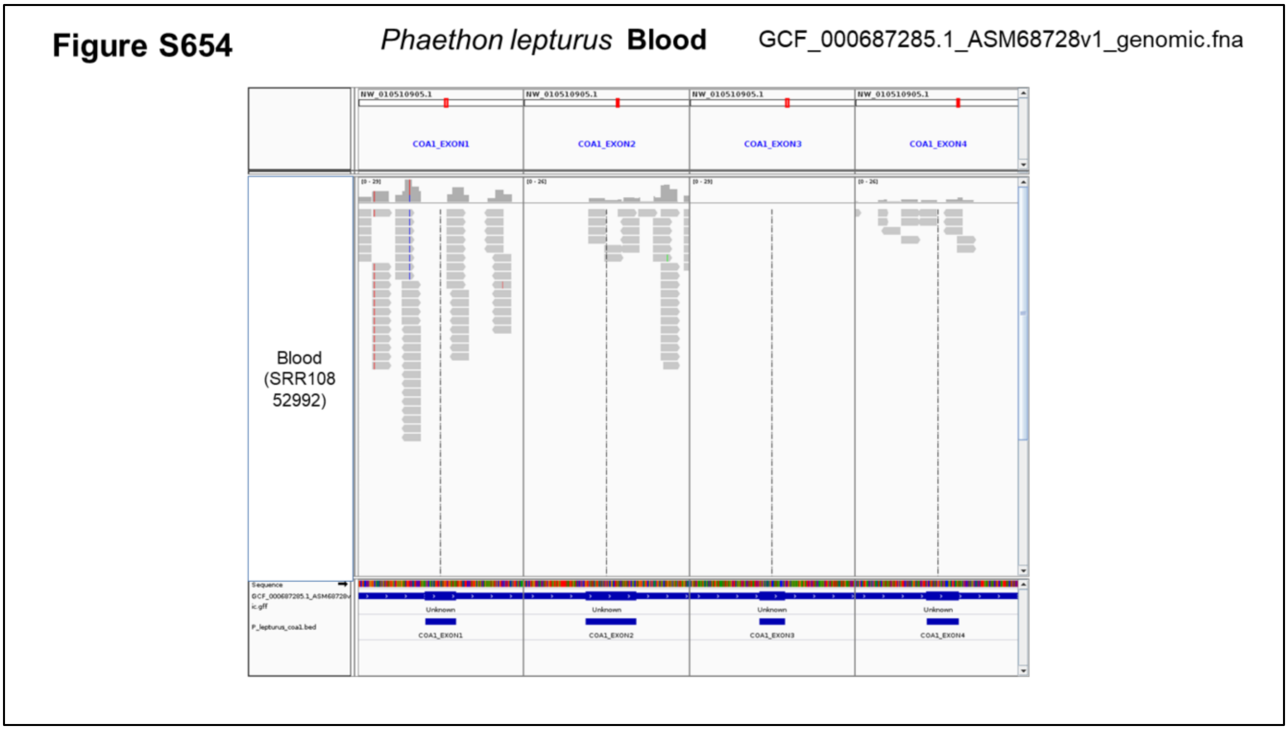

Figure S654: A zoomed in view of each of the *COA1* exons shown in **Supplementary Fig. S653**.

Figure S655 *Opisthocomus hoazin* **Muscle** GCF_000692075.1_ASM69207v1_genomic.fna

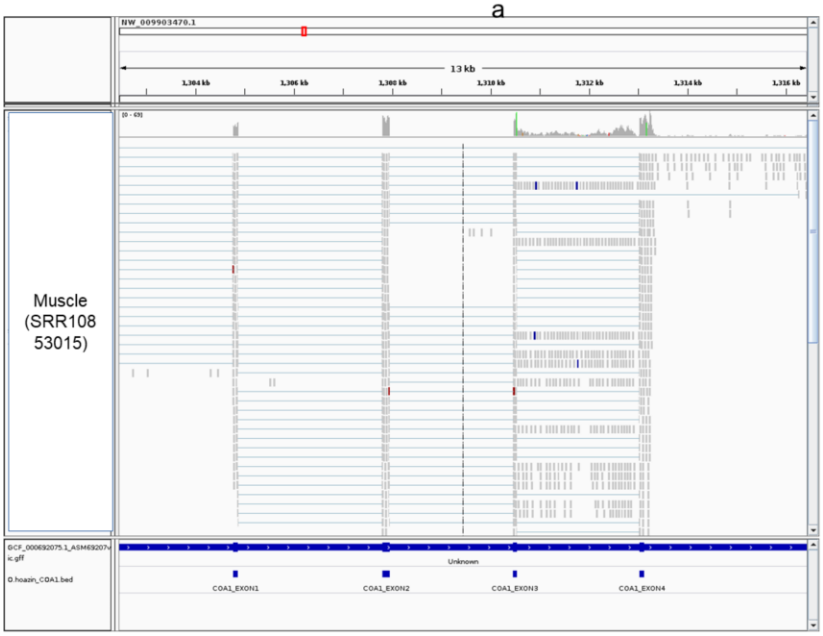

Figure S655: Screenshot of RNA-seq reads aligned to each of the putative locations of the four *COA1* exons in hoatzin (*Opisthocomus hoazin*). Gray boxes represent reads aligned to the genome and thin lines connecting these boxes represent the spliced read alignments. Datasets from muscle (SRR10853015) have been mapped to the GCF_000692075.1_ASM69207v1_genomic.fna genome. Locations of each of the exons are depicted as bed record in the bottom row.

Figure S656 *Opisthocomus hoazin* **Muscle** GCF_000692075.1_ASM69207v1_genomic.fna

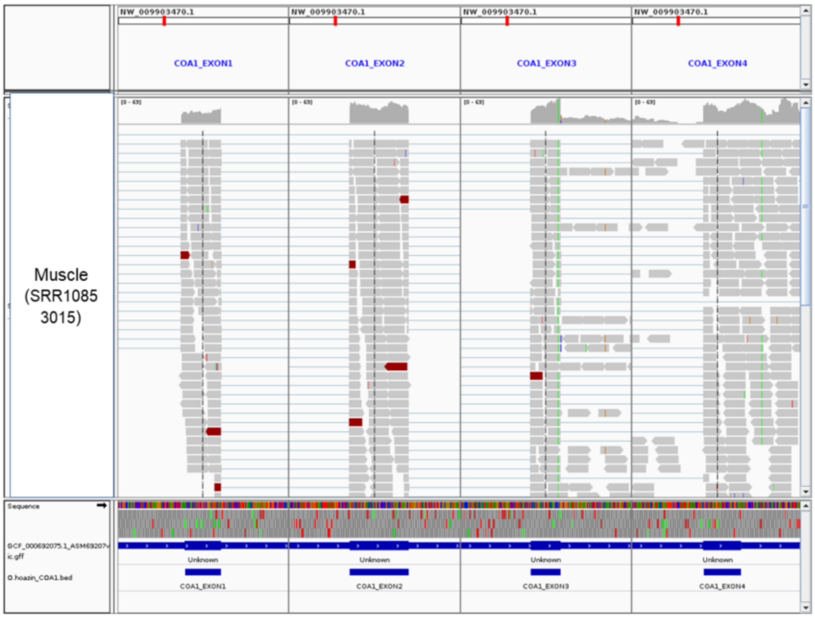

Figure S656: A zoomed in view of each of the *COA1* exons shown in **Supplementary Fig. S655**.

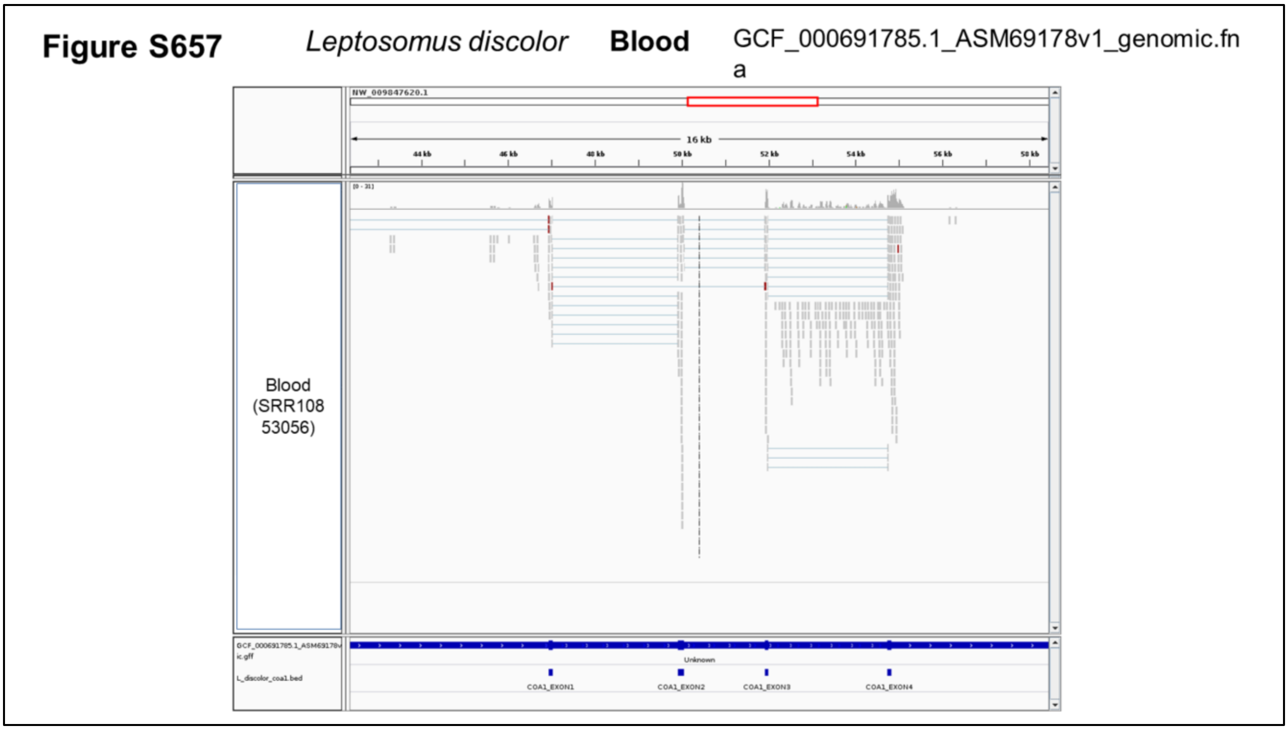

Figure S657: Screenshot of RNA-seq reads aligned to each of the putative locations of the four *COA1* exons in cuckoo roller (*Leptosomus discolor*). Gray boxes represent reads aligned to the genome and thin lines connecting these boxes represent the spliced read alignments. Datasets from blood (SRR10853056) have been mapped to the GCF_000691785.1_ASM69178v1_genomic.fna genome. Locations of each of the exons are depicted as bed record in the bottom row.

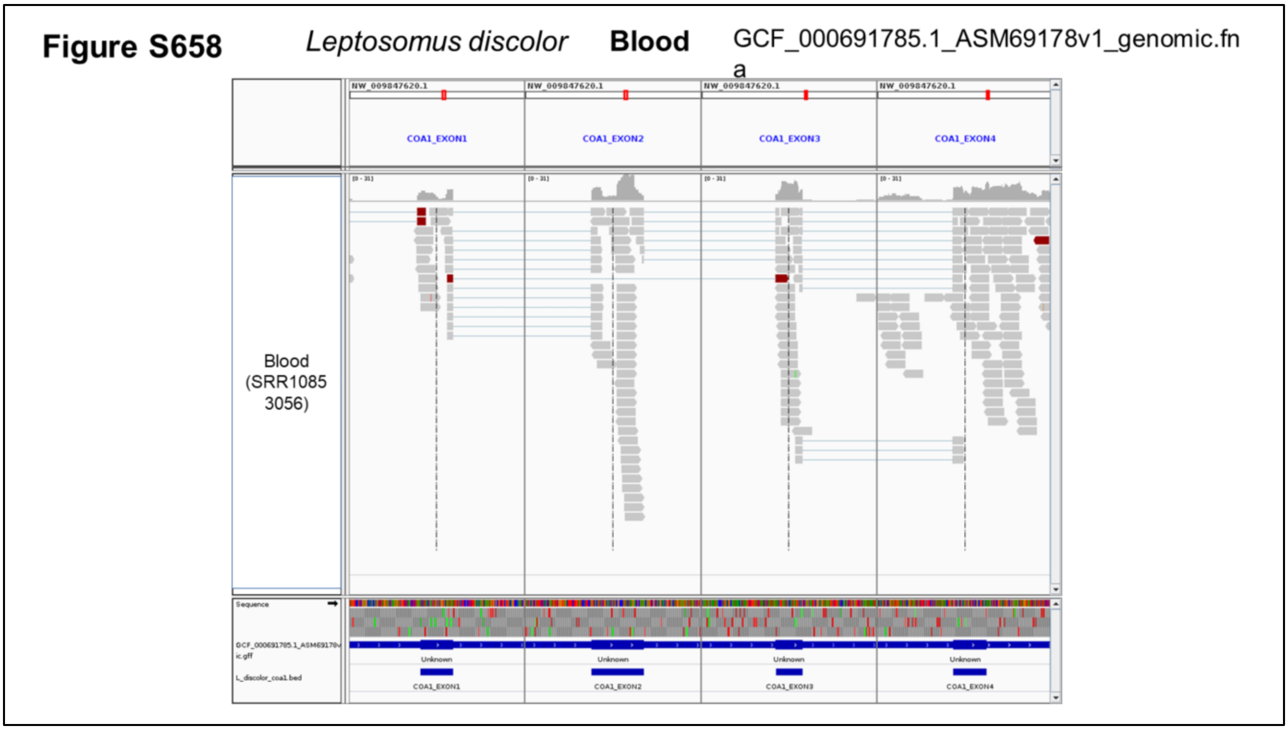

Figure S658: A zoomed in view of each of the *COA1* exons shown in **Supplementary Fig. S657**.

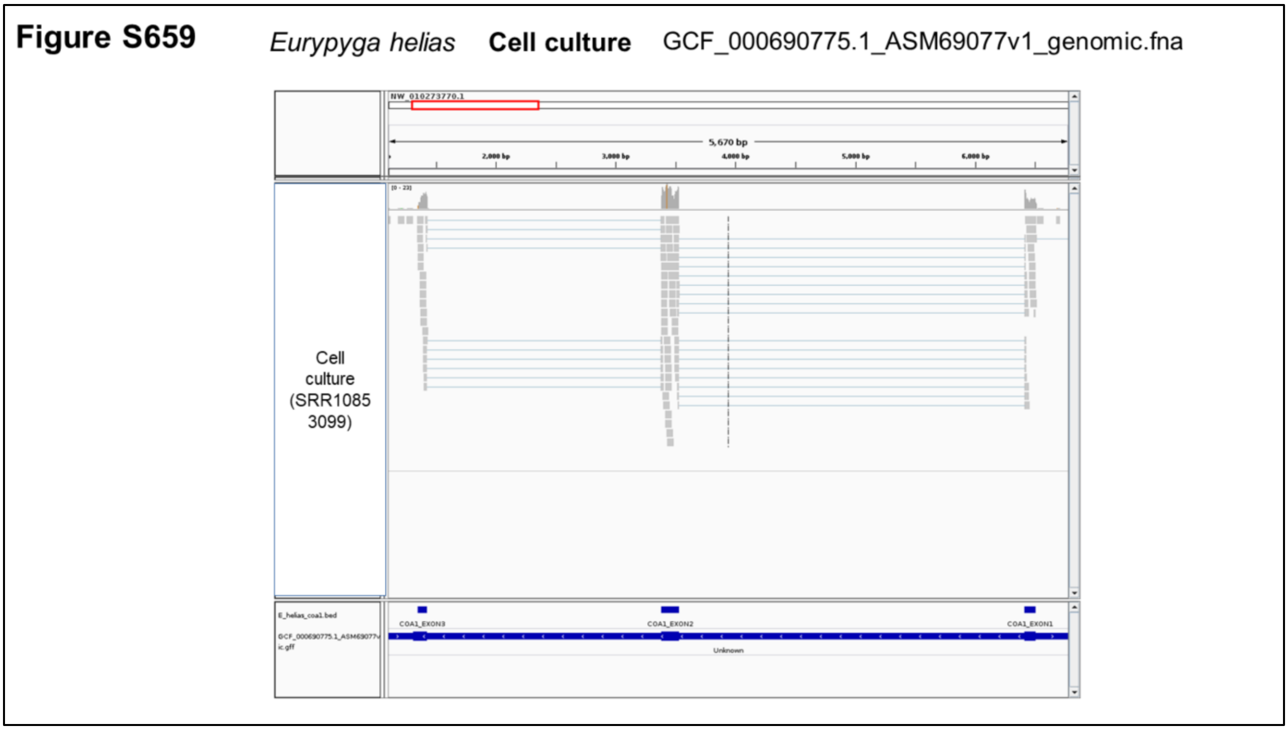

Figure S659: Screenshot of RNA-seq reads aligned to each of the putative locations of the *COA1* exon 1-3 in sunbittern (*Eurypyga helias*). Gray boxes represent reads aligned to the genome and thin lines connecting these boxes represent the spliced read alignments. Datasets from cell culture (SRR10853099) have been mapped to the GCF_000690775.1_ASM69077v1_genomic.fna genome. Locations of each of the exons are depicted as bed record in the bottom row.

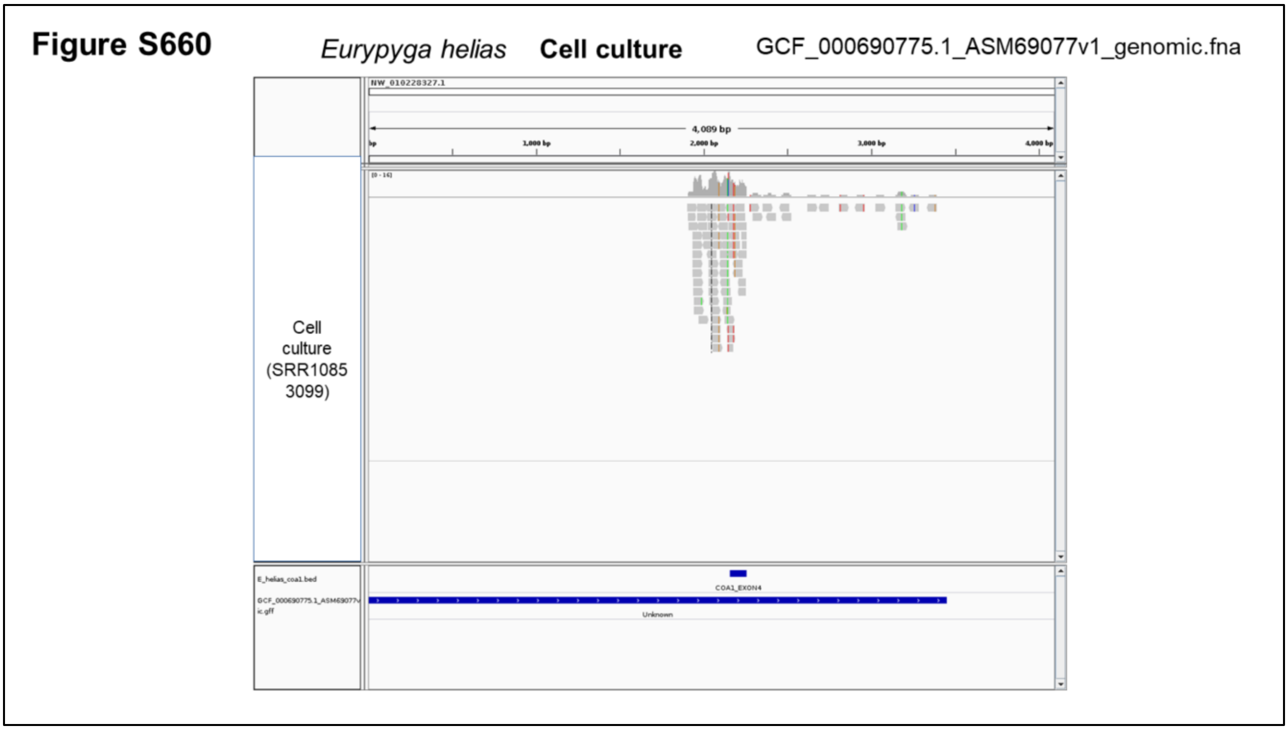

Figure S660: Screenshot of RNA-seq reads aligned to the putative locations of the *COA1* exon 4 in sunbittern (*Eurypyga helias*). Gray boxes represent reads aligned to the genome and thin lines connecting these boxes represent the spliced read alignments. Datasets from cell culture (SRR10853099) have been mapped to the GCF_000690775.1_ASM69077v1_genomic.fna genome. Locations of each of the exons are depicted as bed record in the bottom row.

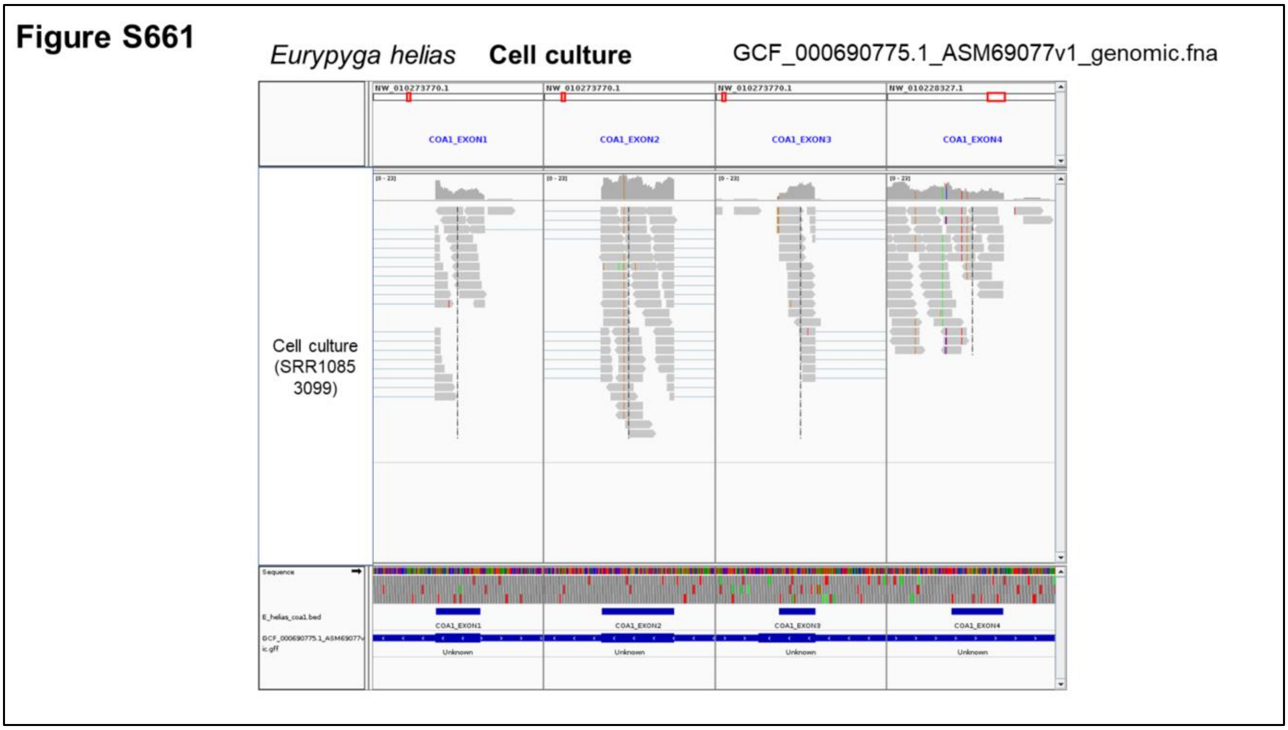

Figure S661: A zoomed in view of each of the *COA1* exons shown in **Supplementary Fig. S660**.

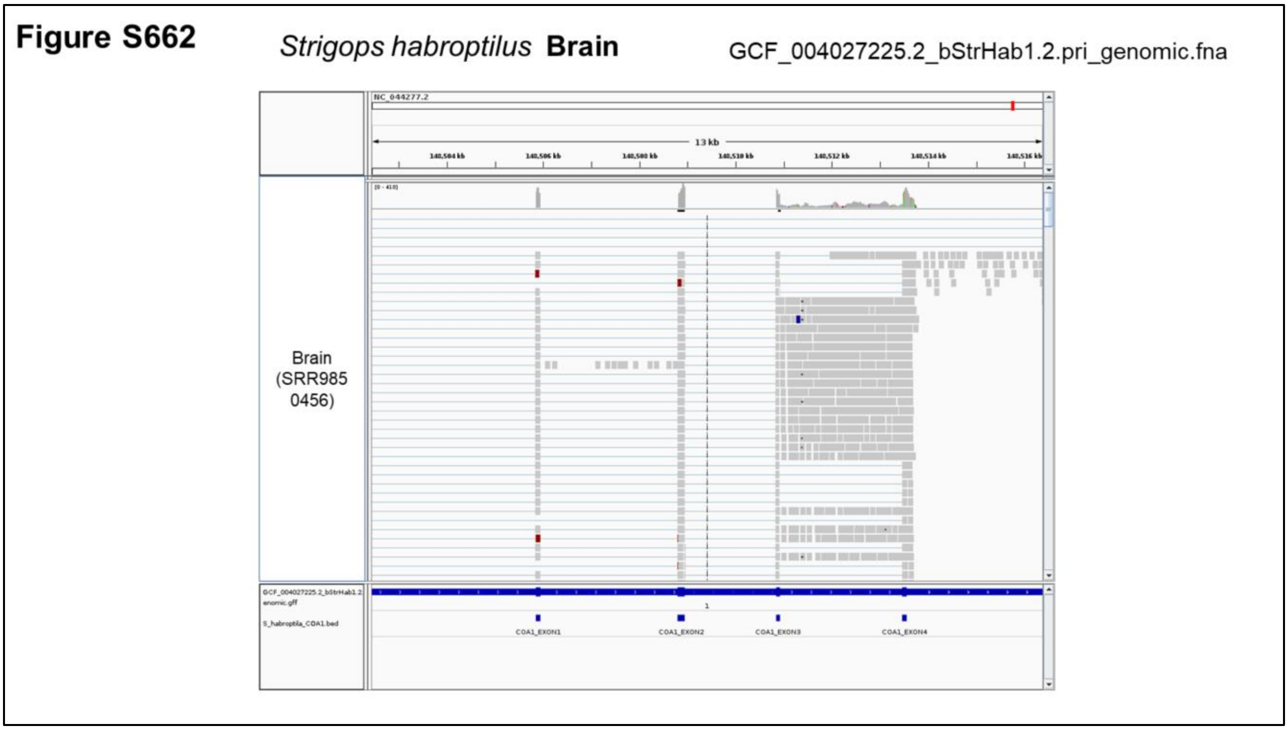

Figure S662: Screenshot of RNA-seq reads aligned to each of the putative locations of the four *COA1* exons in kakapo (*Strigops habroptilus*). Gray boxes represent reads aligned to the genome and thin lines connecting these boxes represent the spliced read alignments. Datasets from brain (SRR9850456) have been mapped to the GCF_004027225.2_bStrHab1.2.pri_genomic.fna genome. Locations of each of the exons are depicted as bed record in the bottom row.

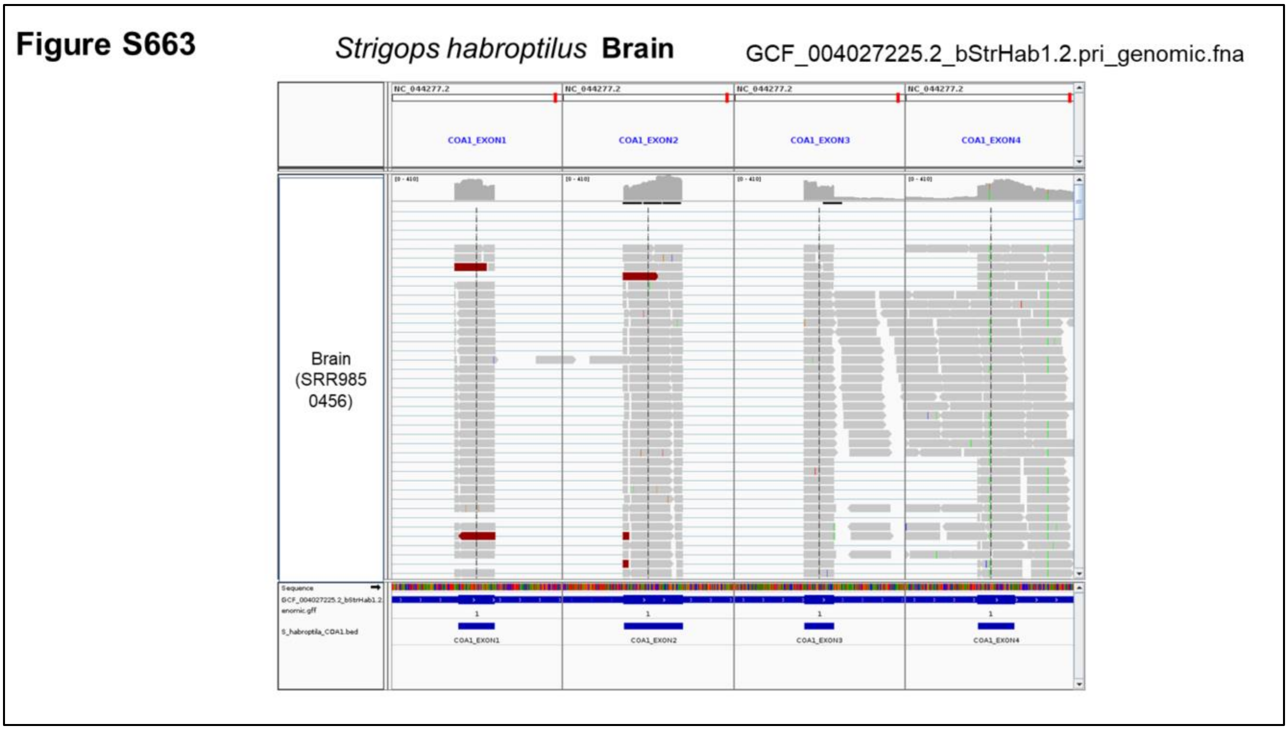

Figure S663: A zoomed in view of each of the *COA1* exons shown in **Supplementary Fig. S662**.

Figure S664 *Zonotrichia albicollis* **Blood** GCF_000385455.1_Zonotrichia_albicollis-1.0.1_genomic.fna

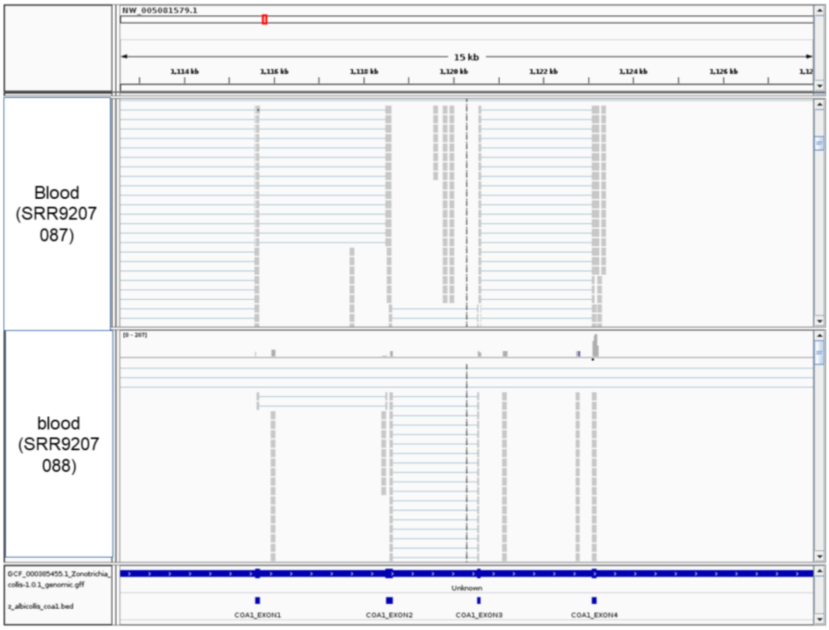

Figure S664: Screenshot of RNA-seq reads aligned to each of the putative locations of the four *COA1* exons in white-throated sparrow (*Zonotrichia albicollis*). Gray boxes represent reads aligned to the genome and thin lines connecting these boxes represent the spliced read alignments. Datasets from blood (SRR9207087) and (SRR9207088) have been mapped to the GCF_000385455.1_Zonotrichia_albicollis-1.0.1_genomic.fna genome. Locations of each of the exons are depicted as bed record in the bottom row.

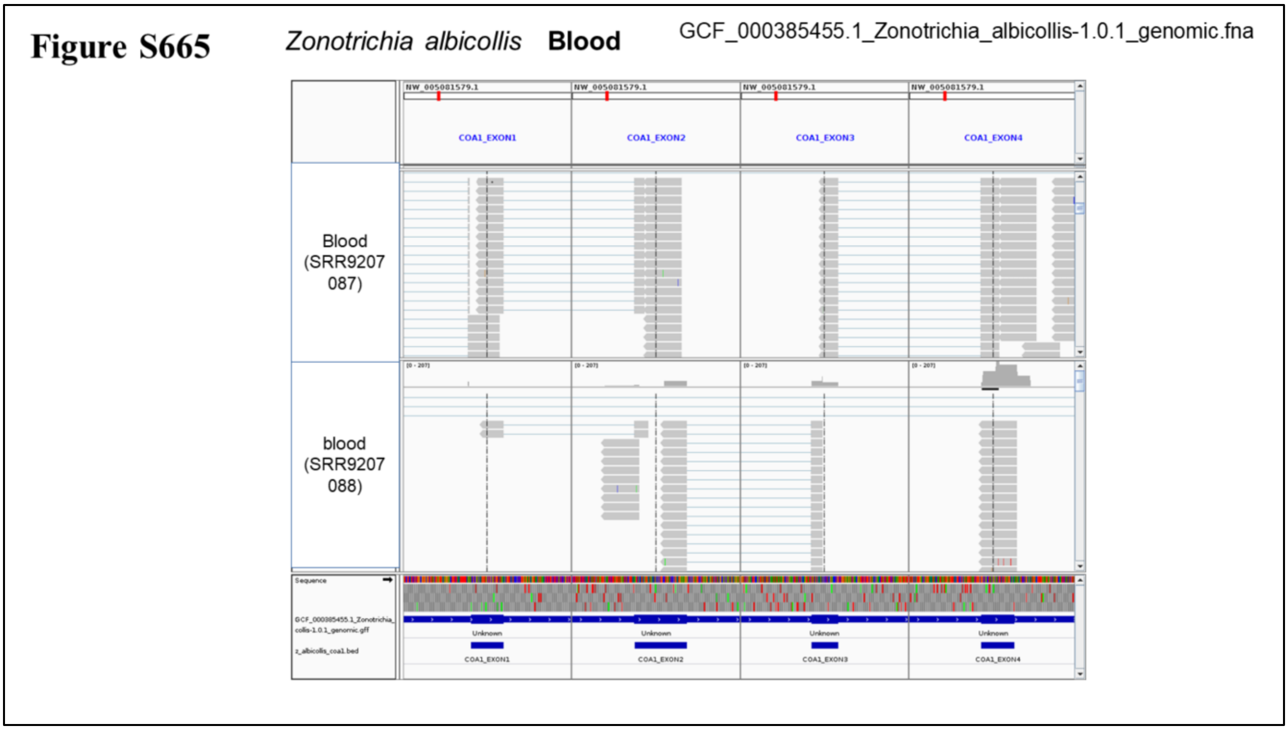

Figure S665: A zoomed in view of each of the *COA1* exons shown in **Supplementary Fig. S664**.

Figure S666

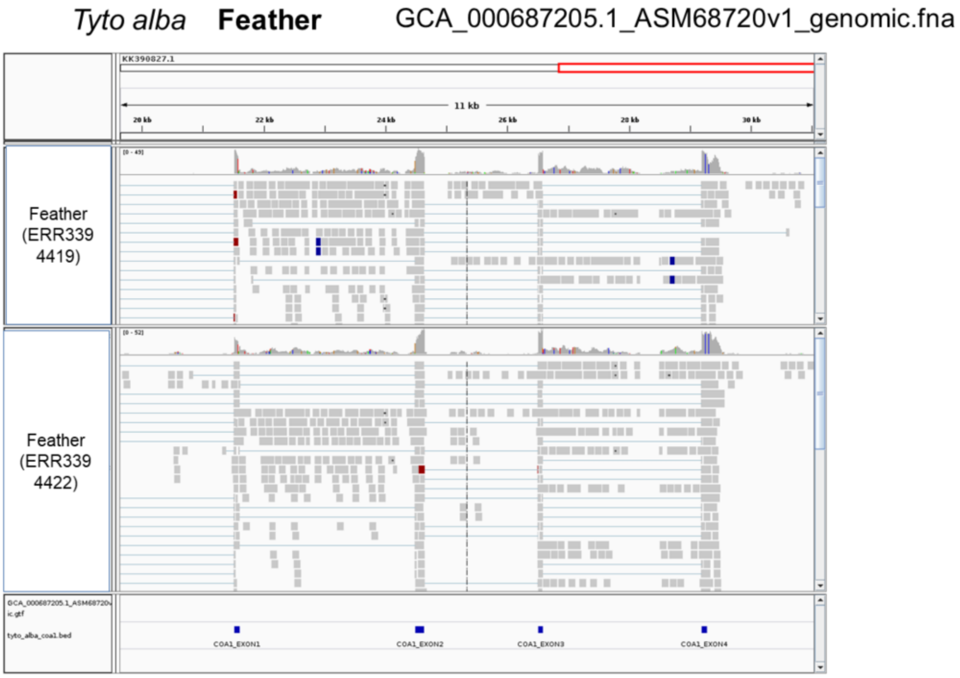

Figure S666: Screenshot of RNA-seq reads aligned to each of the putative locations of the four *COA1* exons in barn owl (*Tyto alba*). Gray boxes represent reads aligned to the genome and thin lines connecting these boxes represent the spliced read alignments. Datasets from feather (ERR3394419) and (ERR3394422) have been mapped to the GCA_000687205.1_ASM68720v1_genomic.fna genome. Locations of each of the exons are depicted as bed record in the bottom row.

Figure S667 *Tyto alba* **Feather** GCA_000687205.1_ASM68720v1_genomic.fna

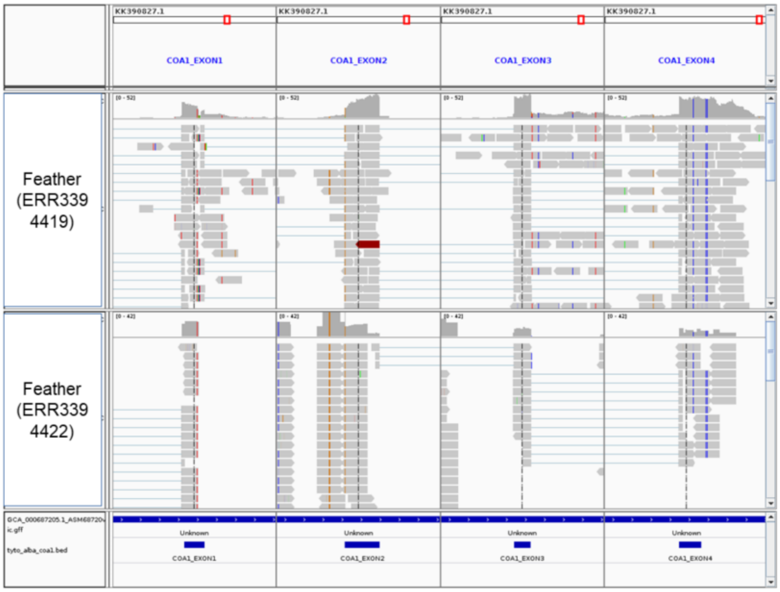

Figure S667: A zoomed in view of each of the *COA1* exons shown in **Supplementary Fig. S666**.

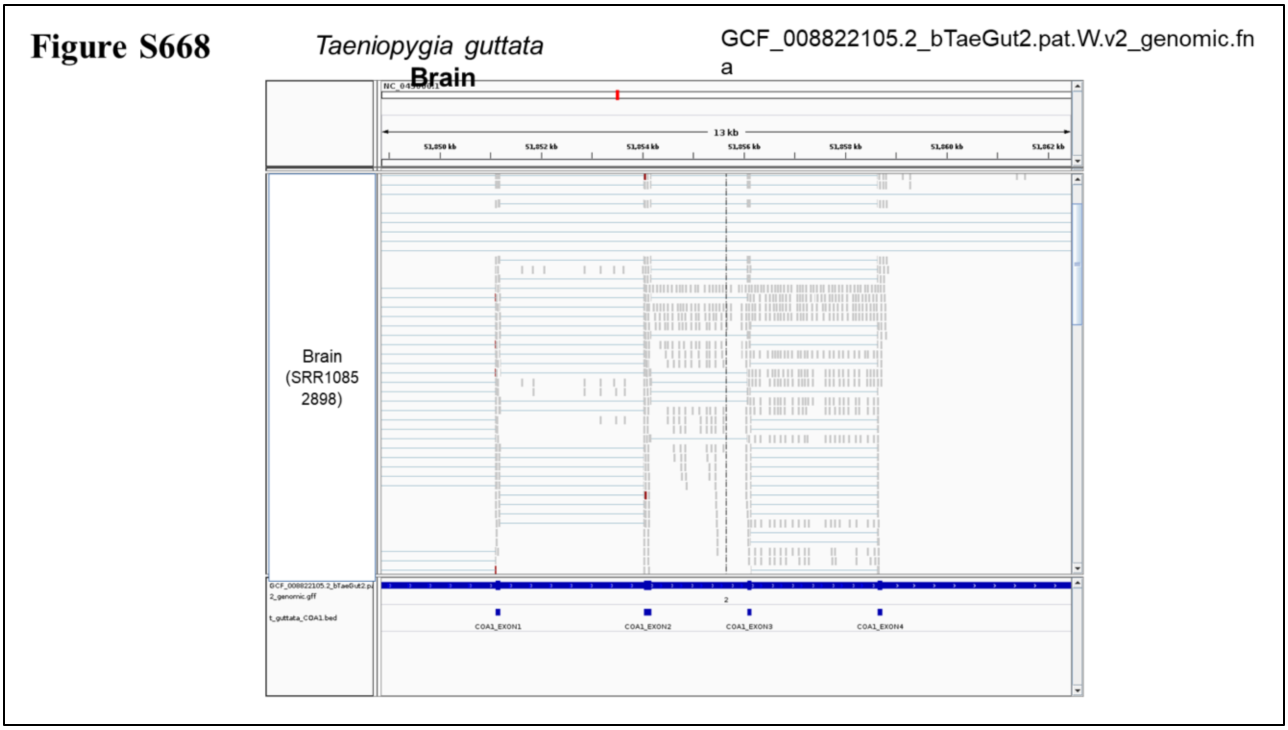

Figure S668: Screenshot of RNA-seq reads aligned to each of the putative locations of the four *COA1* exons in zebra finch (*Taeniopygia guttata*). Gray boxes represent reads aligned to the genome and thin lines connecting these boxes represent the spliced read alignments. Datasets from brain (SRR10852898) have been mapped to the GCF_008822105.2_bTaeGut2.pat.W.v2_genomic.fna genome. Locations of each of the exons are depicted as bed record in the bottom row.

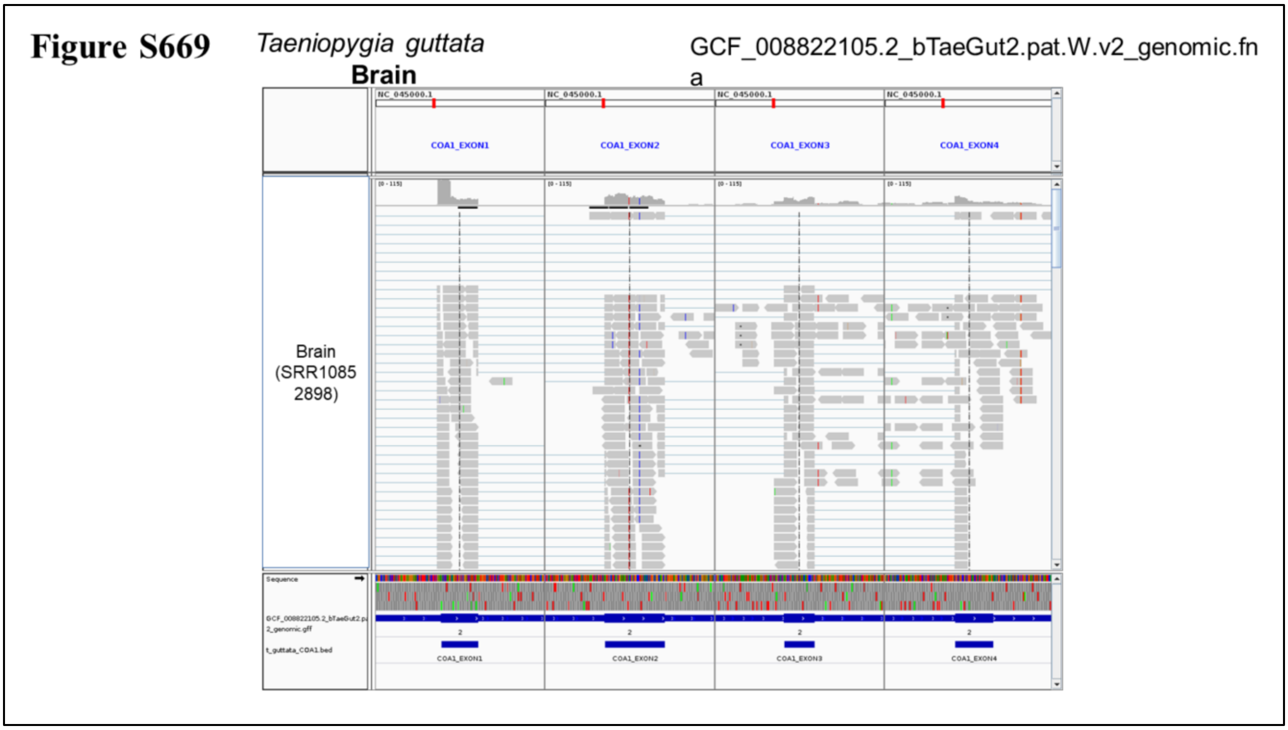

Figure S669: A zoomed in view of each of the *COA1* exons shown in **Supplementary Fig. S668**.

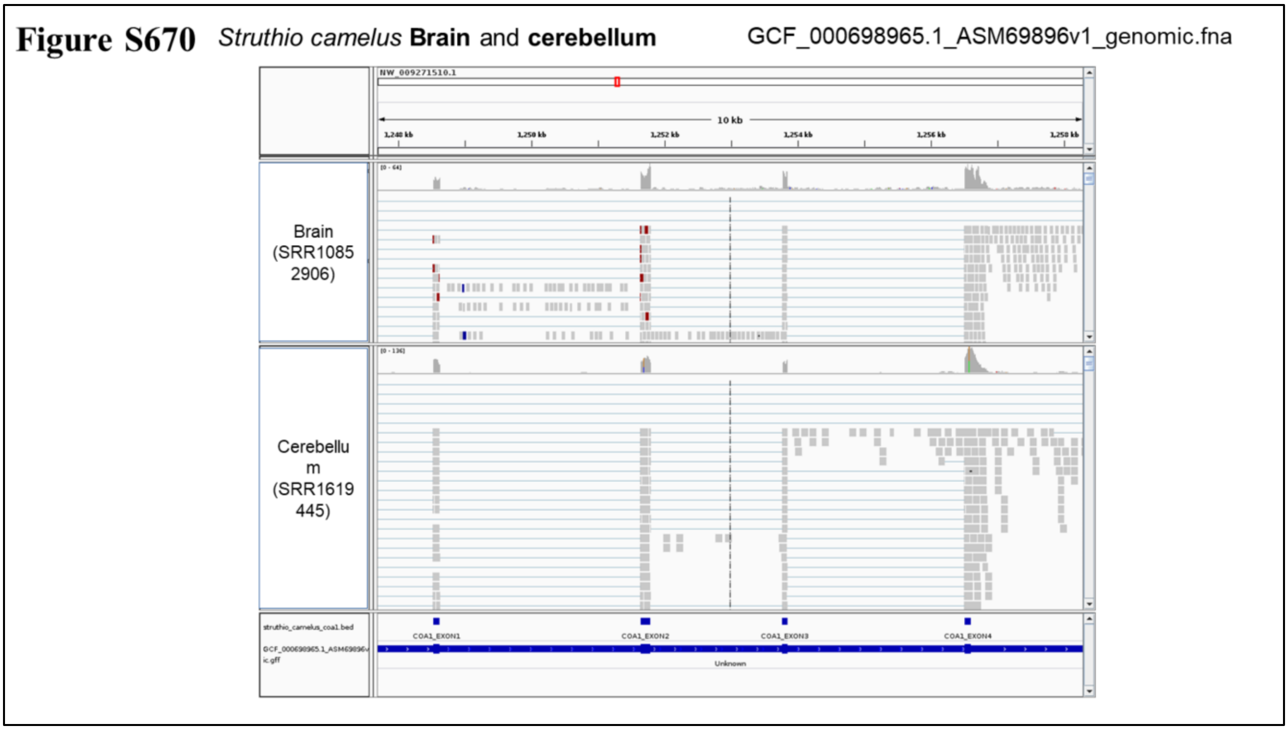

Figure S670: Screenshot of RNA-seq reads aligned to each of the putative locations of the four *COA1* exons in ostrich (*Struthio camelus*). Gray boxes represent reads aligned to the genome and thin lines connecting these boxes represent the spliced read alignments. Datasets from brain (SRR10852906) and cerebellum (SRR1619445) have been mapped to the GCF_000698965.1_ASM69896v1_genomic.fna genome. Locations of each of the exons are depicted as bed record in the bottom row.

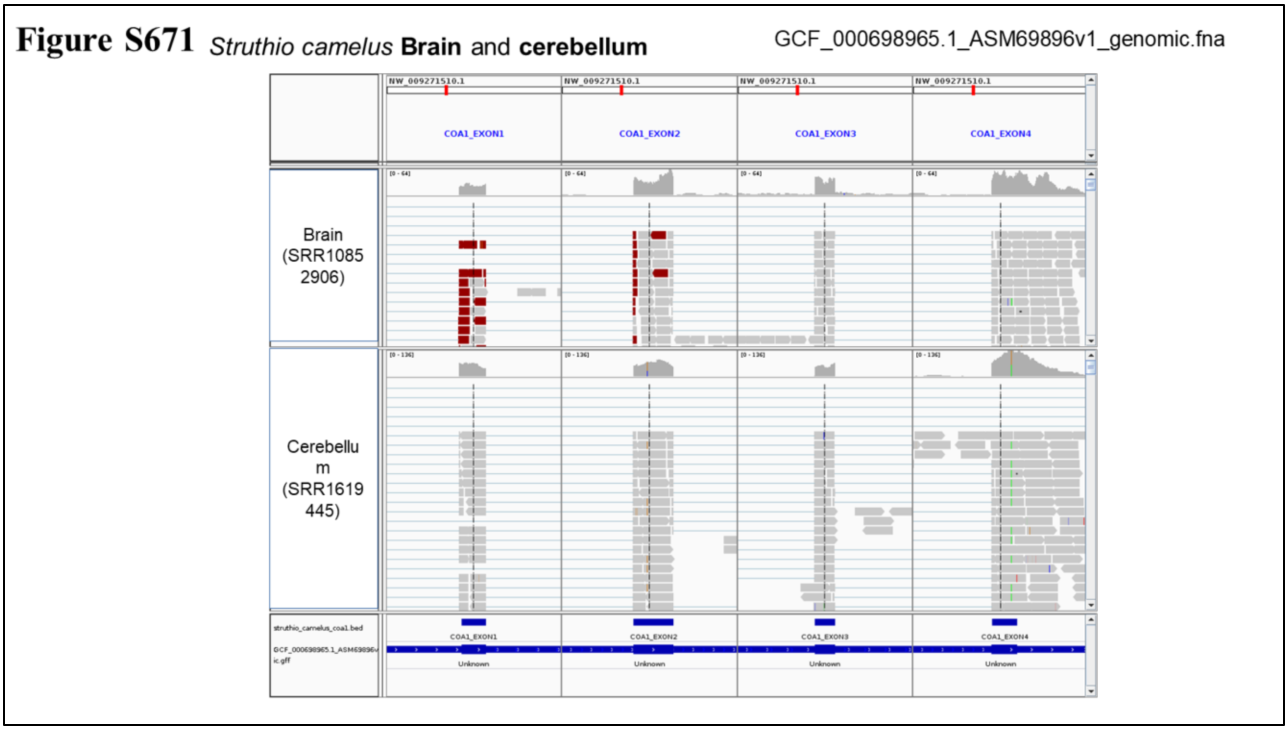

Figure S671: A zoomed in view of each of the *COA1* exons shown in **Supplementary Fig. S670**.

Figure S672 *Pygoscelis adeliae* **Cloacal swab and Muscle** GCF_000699105.1_ASM69910v1_genomic.fna

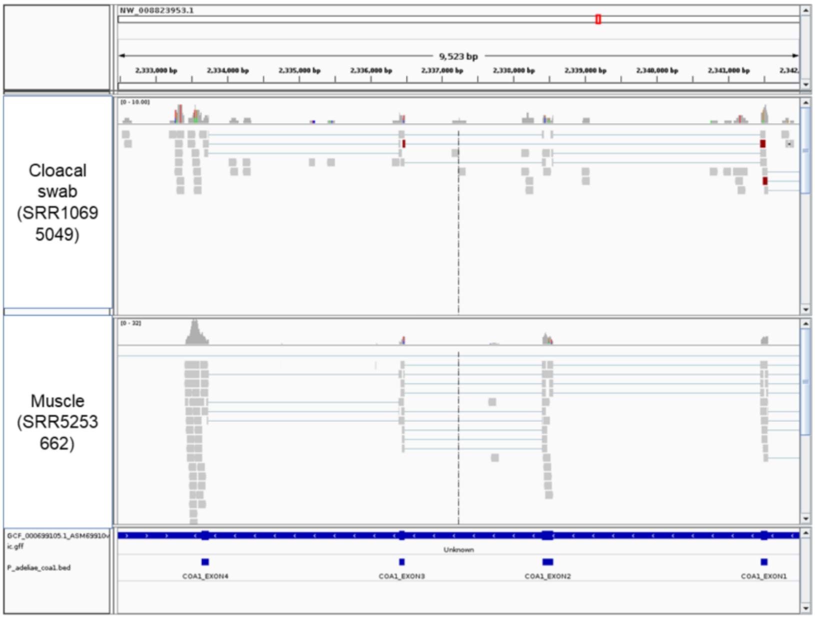

Figure S672: Screenshot of RNA-seq reads aligned to each of the putative locations of the four *COA1* exons in Adélie penguin (*Pygoscelis adeliae*). Gray boxes represent reads aligned to the genome and thin lines connecting these boxes represent the spliced read alignments. Datasets from cloacal swab (SRR10695049) and muscle (SRR5253662) have been mapped to the GCF_000699105.1_ASM69910v1_genomic.fna genome. Locations of each of the exons are depicted as bed record in the bottom row.

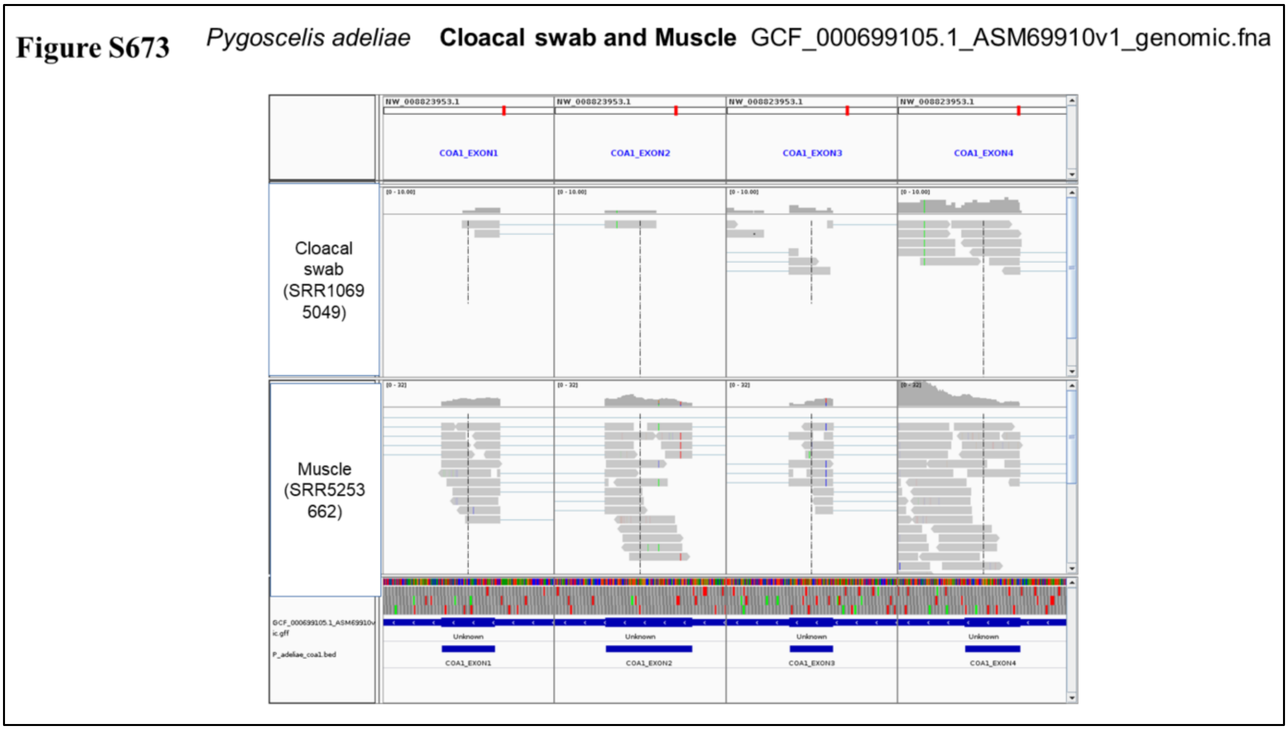

Figure S673: A zoomed in view of each of the *COA1* exons shown in **Supplementary Fig. S672**.

Figure S674 *Passer domesticus* **Blood** GCA_001700915.1_Passer_domesticus-1.0_genomic.fna

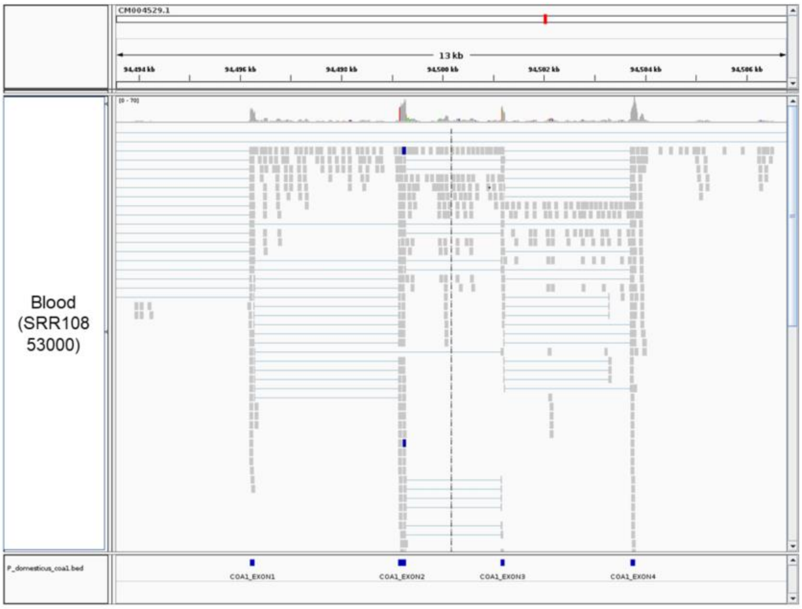

Figure S674: Screenshot of RNA-seq reads aligned to each of the putative locations of the four *COA1* exons in house sparrow (*Passer domesticus*). Gray boxes represent reads aligned to the genome and thin lines connecting these boxes represent the spliced read alignments. Datasets from blood (SRR10853000) have been mapped to the GCA_001700915.1_Passer_domesticus-1.0_genomic.fna genome. Locations of each of the exons are depicted as bed record in the bottom row.

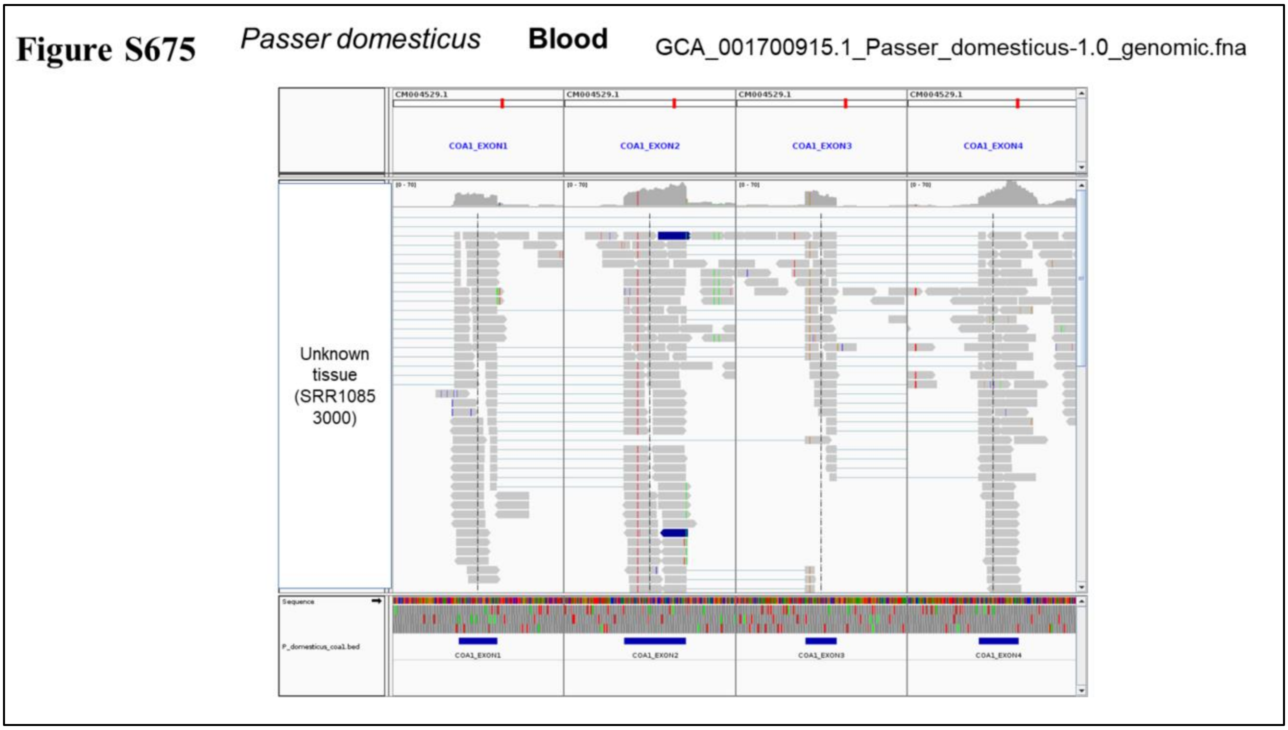

Figure S675: A zoomed in view of each of the *COA1* exons shown in **Supplementary Fig. S674**.

Figure S676 *Melopsittacus undulatus* GCF_012275295.1_bMelUnd1.mat.Z_genomic.fna

Feather

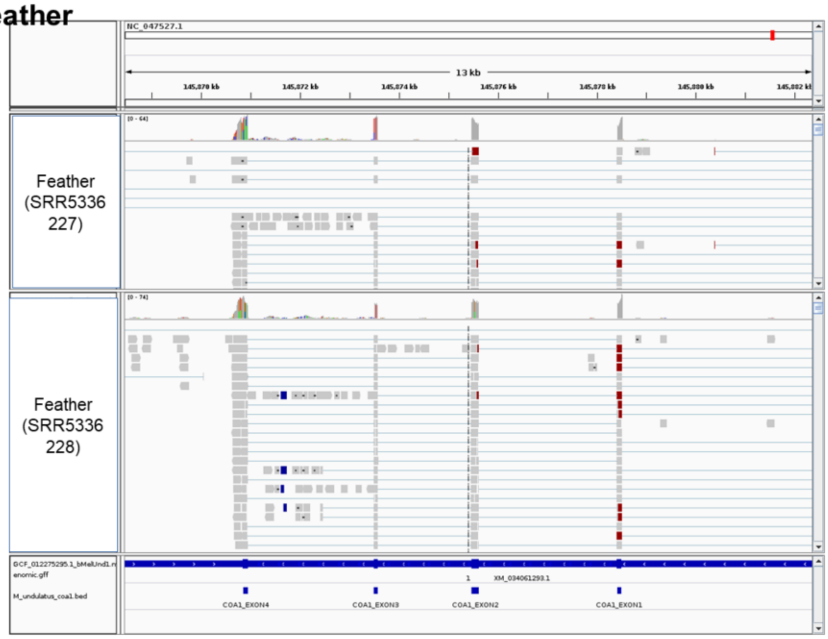

Figure S676: Screenshot of RNA-seq reads aligned to each of the putative locations of the four *COA1* exons in budgerigar (*Melopsittacus undulatus*). Gray boxes represent reads aligned to the genome and thin lines connecting these boxes represent the spliced read alignments. Datasets from feather (SRR5336227) and (SRR5336228) have been mapped to the GCF_012275295.1_bMelUnd1.mat.Z_genomic.fna genome. Locations of each of the exons are depicted as bed record in the bottom row.

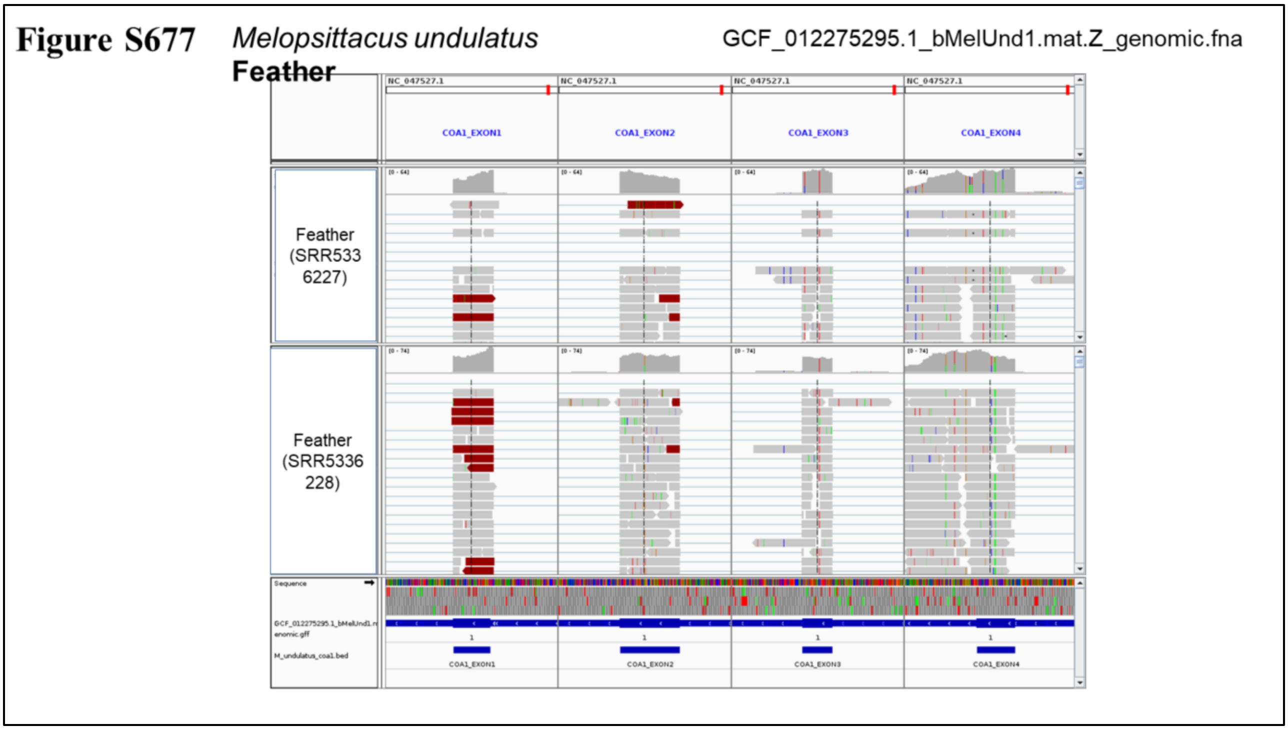

Figure S677: A zoomed in view of each of the *COA1* exons shown in **Supplementary Fig. S676**.

Figure S678 *Erythrura gouldiae* **Skin** GCA_003676055.1_GouldianFinch_genomic.fna

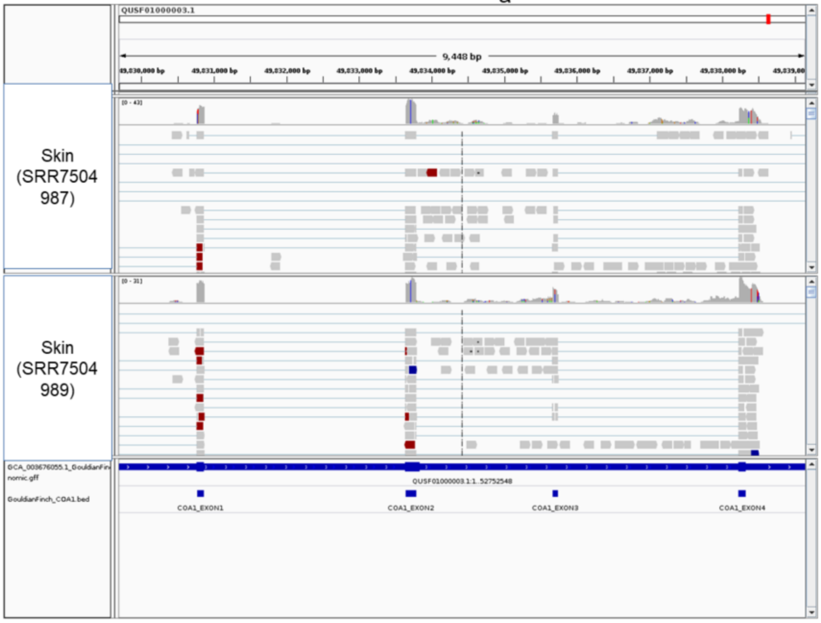

Figure S678: Screenshot of RNA-seq reads aligned to each of the putative locations of the four *COA1* exons in gouldian finch (*Erythrura gouldiae*). Gray boxes represent reads aligned to the genome and thin lines connecting these boxes represent the spliced read alignments. Datasets from skin (SRR7504987) and (SRR7504989) have been mapped to the GCA_003676055.1_GouldianFinch_genomic.fna genome. Locations of each of the exons are depicted as bed record in the bottom row.

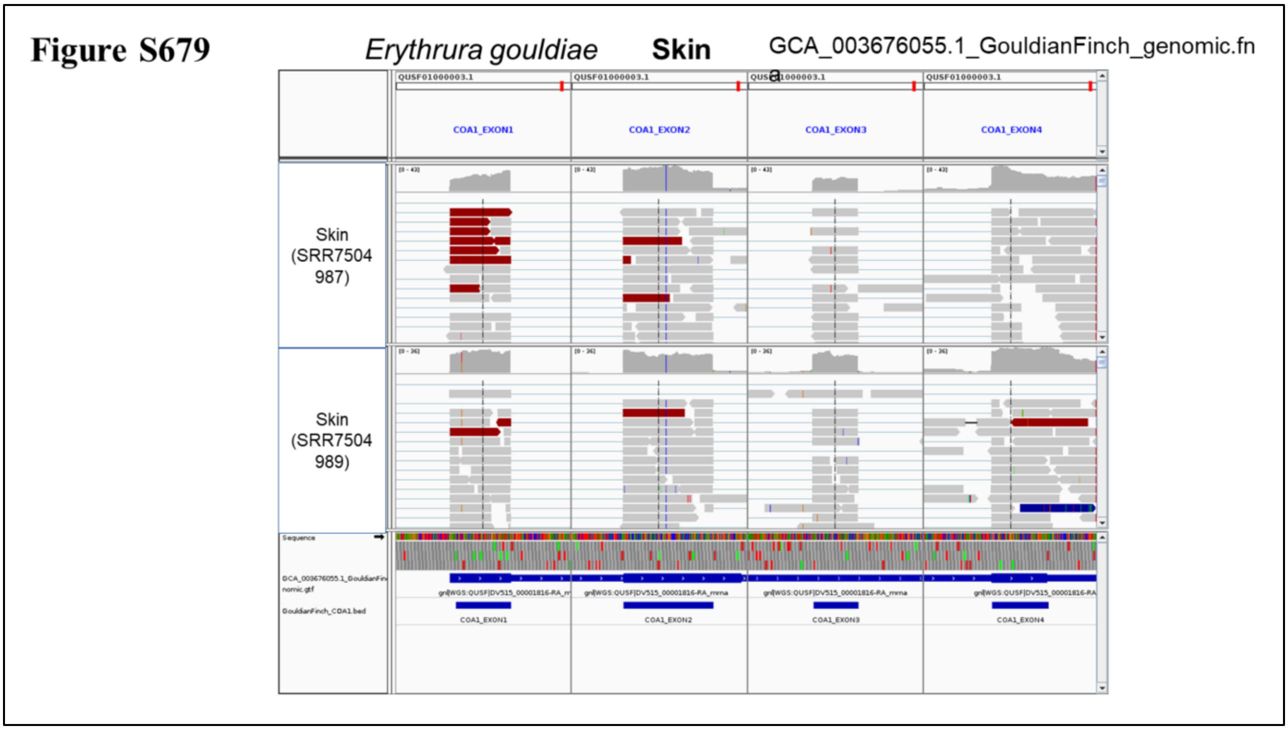

Figure S679: A zoomed in view of each of the *COA1* exons shown in **Supplementary Fig. S678**.

Figure S680

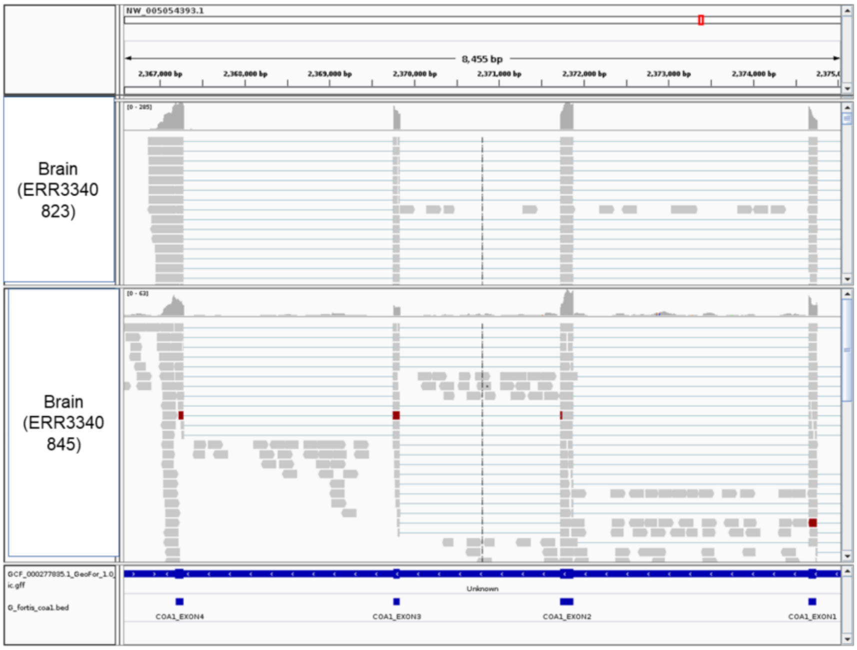

Figure S680: Screenshot of RNA-seq reads aligned to each of the putative locations of the four *COA1* exons in medium ground finch (*Geospiza fortis*). Gray boxes represent reads aligned to the genome and thin lines connecting these boxes represent the spliced read alignments. Datasets from brain (ERR3340823) and (ERR3340845) have been mapped to the GCF_000277835.1_GeoFor_1.0_genomic.fna genome. Locations of each of the exons are depicted as bed record in the bottom row.

Figure S681

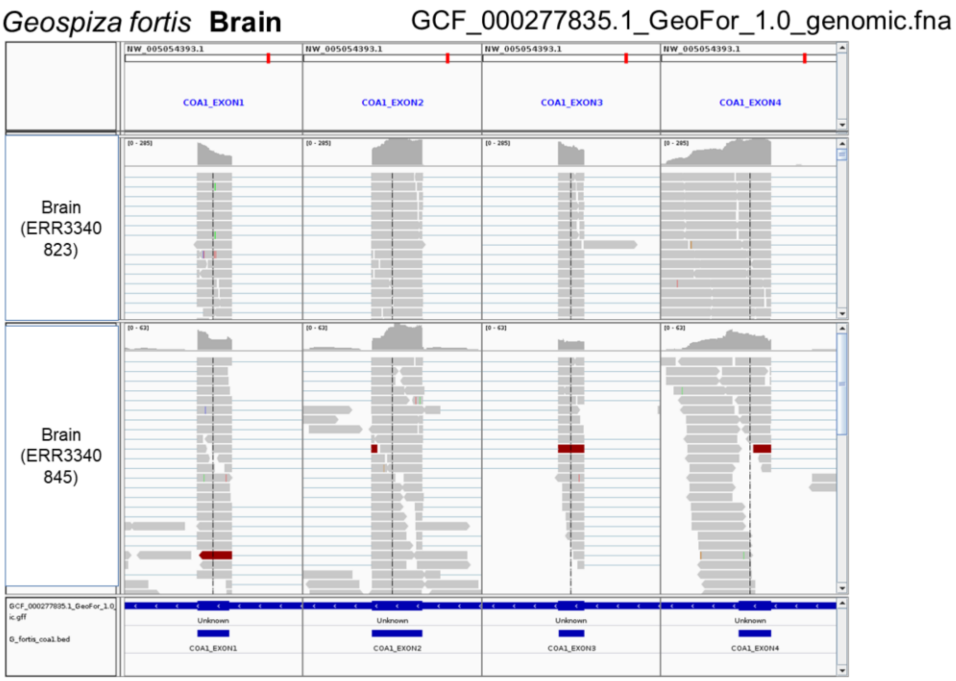

Figure S681: A zoomed in view of each of the *COA1* exons shown in **Supplementary Fig. S680**.

Figure S682 *Ficedula albicollis* **Heart and Kidney** Ficedula_albicollis.FicAlb_1.4.dna_sm.toplevel.fa

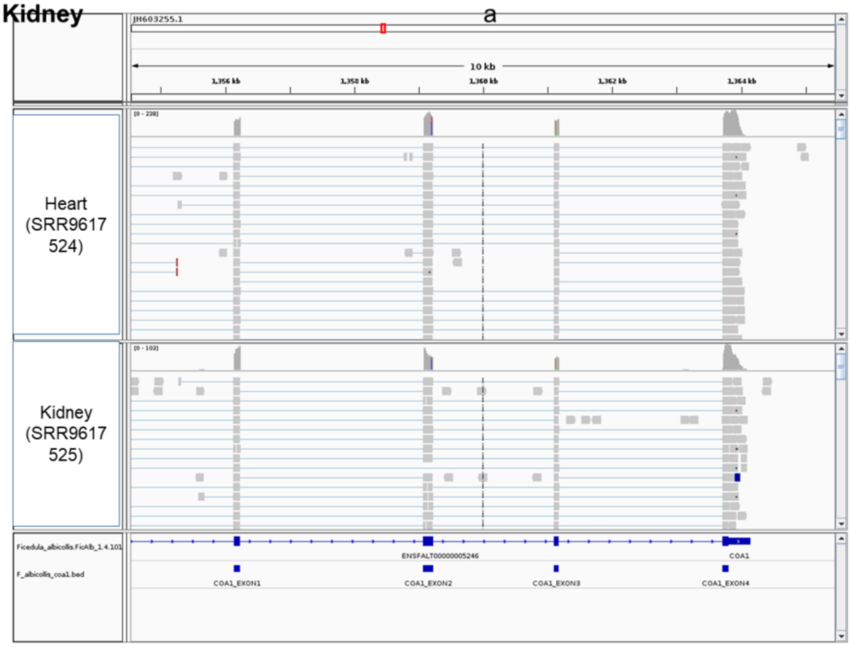

Figure S682: Screenshot of RNA-seq reads aligned to each of the putative locations of the four *COA1* exons in collared flycatcher (*Ficedula albicollis*). Gray boxes represent reads aligned to the genome and thin lines connecting these boxes represent the spliced read alignments. Datasets from heart (SRR9617524) and kidney (SRR9617525) have been mapped to the Ficedula_albicollis.FicAlb_1.4.dna_sm.toplevel.fa genome. Locations of each of the exons are depicted as bed record in the bottom row.

Figure S683

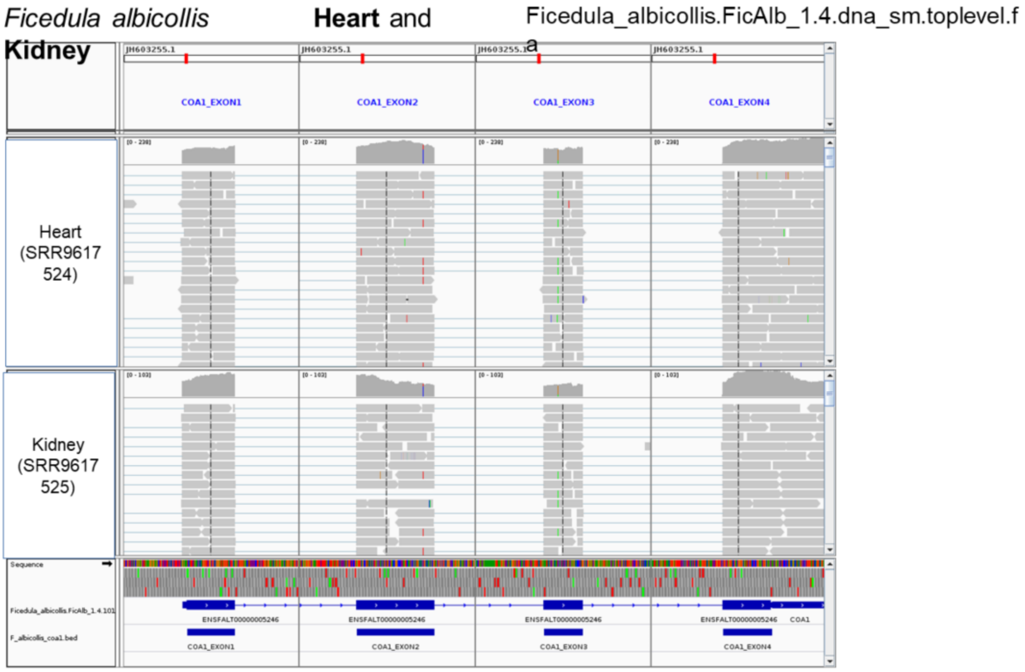

Figure S683: A zoomed in view of each of the *COA1* exons shown in **Supplementary Fig. S682**.

Figure S684 *Falco tinnunculus* **Cochlea** and **Blood** GCA_010332995.1_FalTin1.0_genomic.fna

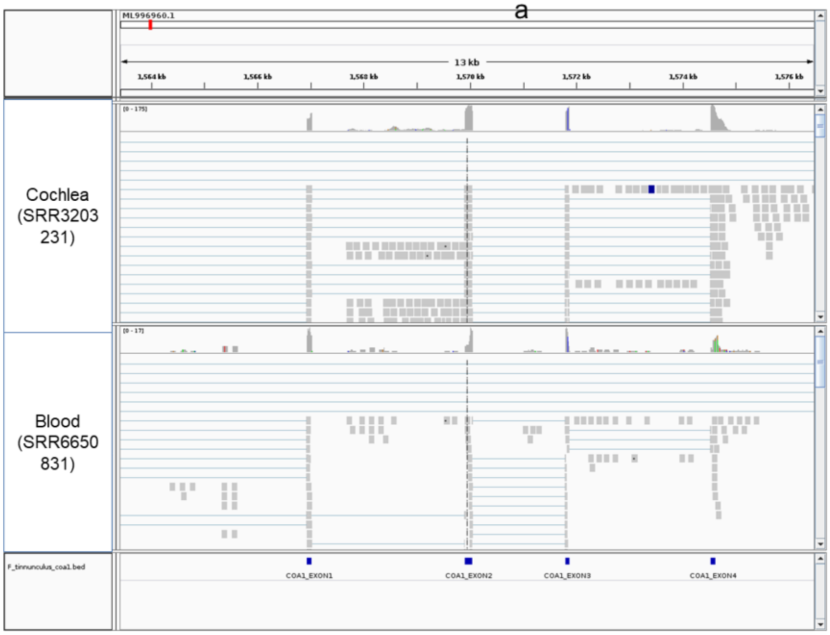

Figure S684: Screenshot of RNA-seq reads aligned to each of the putative locations of the four *COA1* exons in common kestrel (*Falco tinnunculus*). Gray boxes represent reads aligned to the genome and thin lines connecting these boxes represent the spliced read alignments. Datasets from cochlea (SRR3203231) and blood (SRR6650831) have been mapped to the GCA_010332995.1_FalTin1.0_genomic.fna genome. Locations of each of the exons are depicted as bed record in the bottom row.

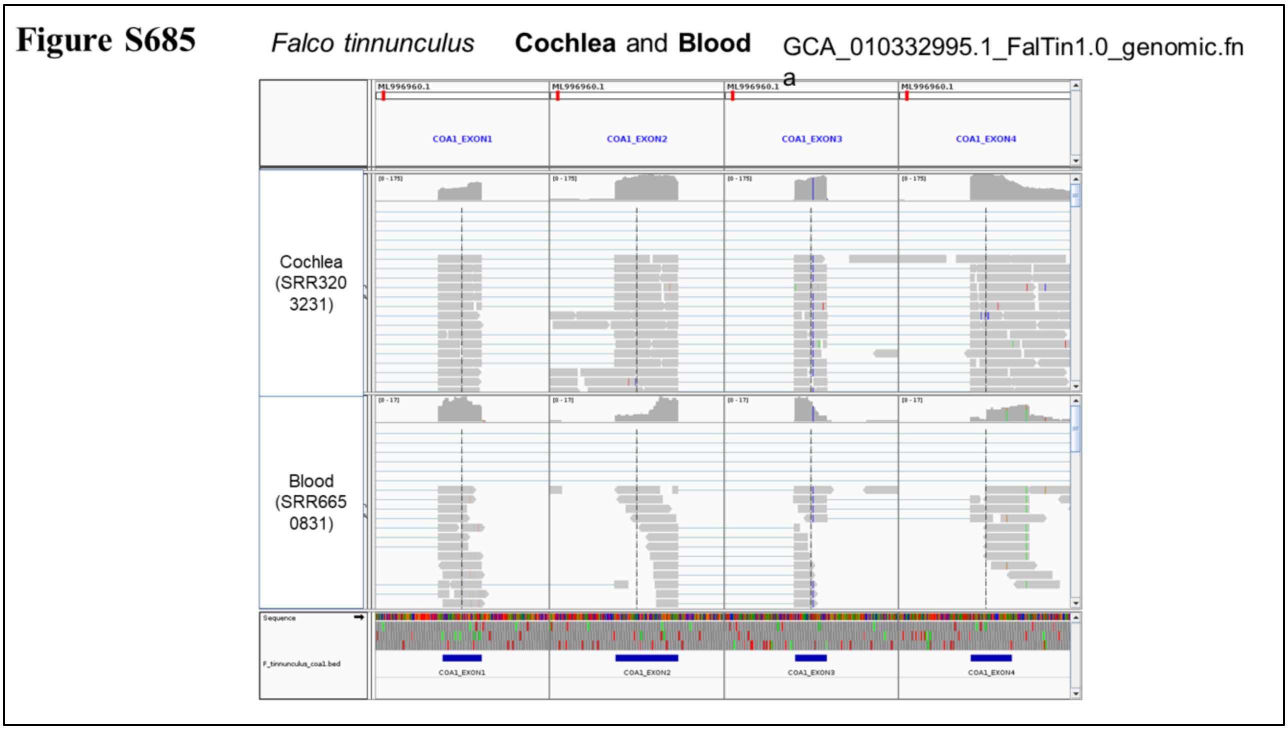

Figure S685: A zoomed in view of each of the *COA1* exons shown in **Supplementary Fig. S684**.

Figure S686 *Falco subbuteo* **Cochlea** GCF_000337955.1_F_peregrinus_v1.0_genomic.fna

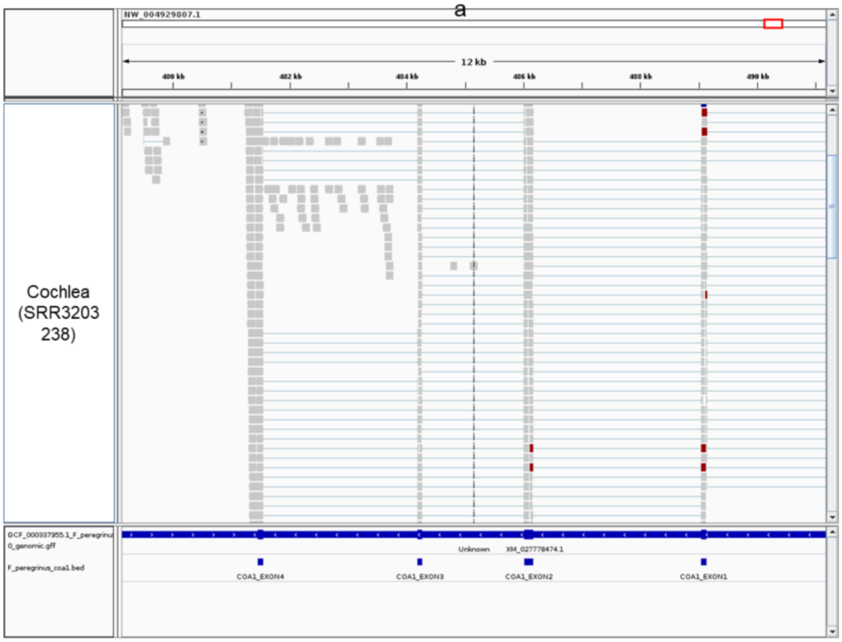

Figure S686: Screenshot of RNA-seq reads aligned to each of the putative locations of the four *COA1* exons in Eurasian hobby (*Falco subbuteo*). Gray boxes represent reads aligned to the genome and thin lines connecting these boxes represent the spliced read alignments. Datasets from cochlea (SRR3203238) have been mapped to the GCF_000337955.1_F_peregrinus_v1.0_genomic.fna genome. Locations of each of the exons are depicted as bed record in the bottom row.

Figure S687

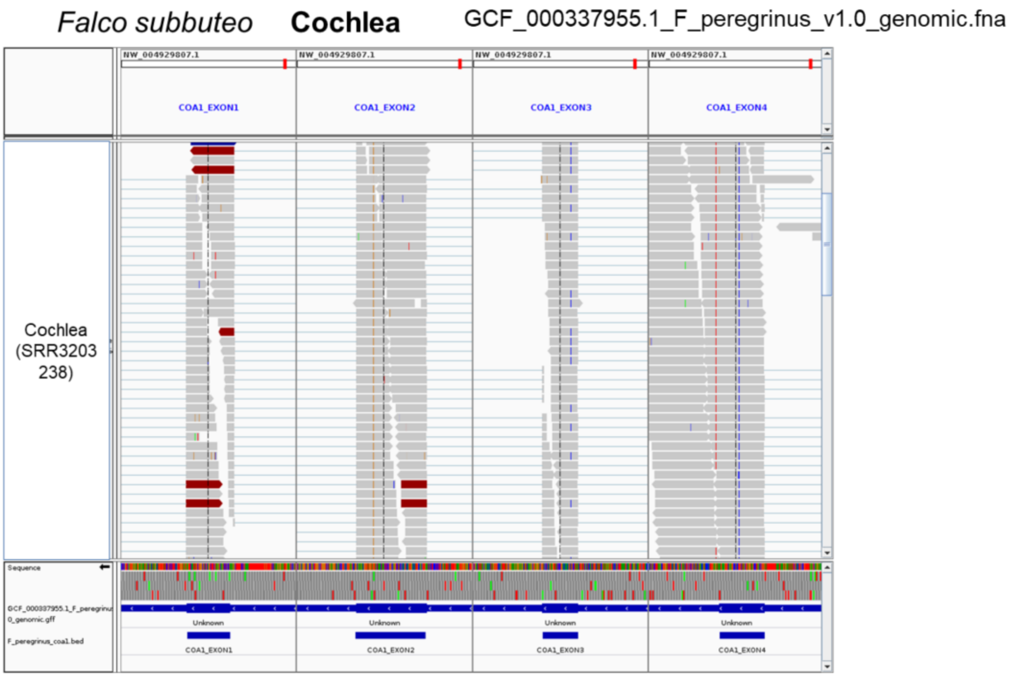

Figure S687: A zoomed in view of each of the *COA1* exons shown in **Supplementary Fig. S686**.

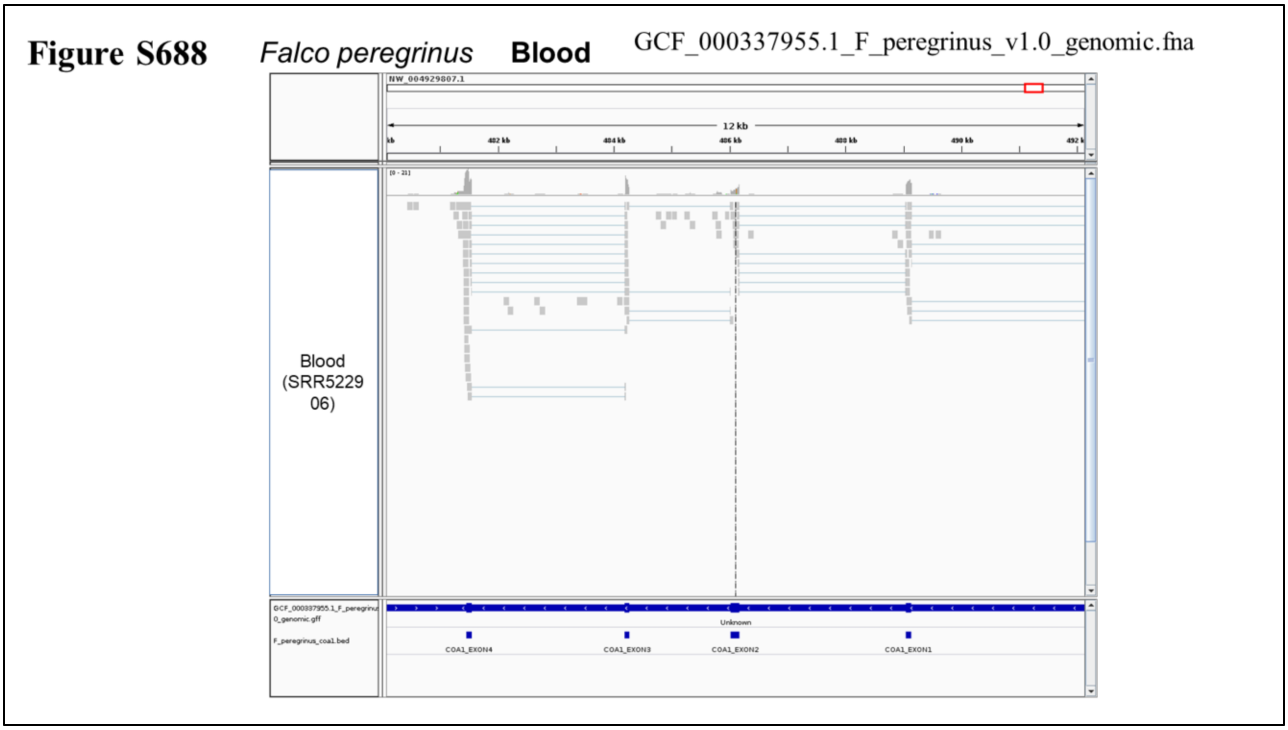

Figure S688: Screenshot of RNA-seq reads aligned to each of the putative locations of the four *COA1* exons in peregrine falcon (*Falco peregrinus*). Gray boxes represent reads aligned to the genome and thin lines connecting these boxes represent the spliced read alignments. Datasets from blood (SRR522906) have been mapped to the GCF_000337955.1_F_peregrinus_v1.0_genomic.fna genome. Locations of each of the exons are depicted as bed record in the bottom row.

Figure S689

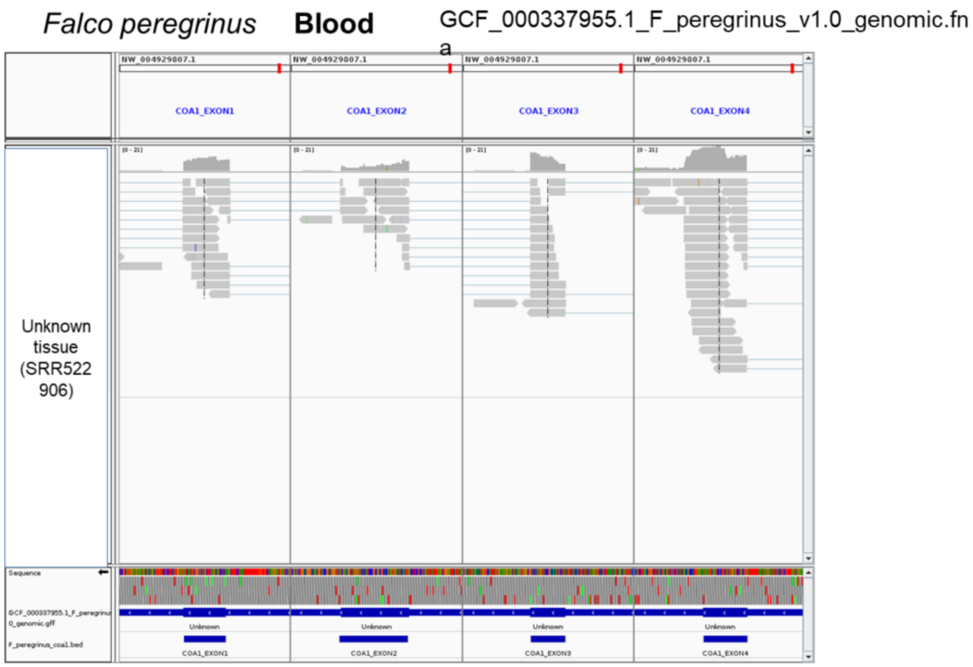

Figure S689: A zoomed in view of each of the *COA1* exons shown in **Supplementary Fig. S688**.

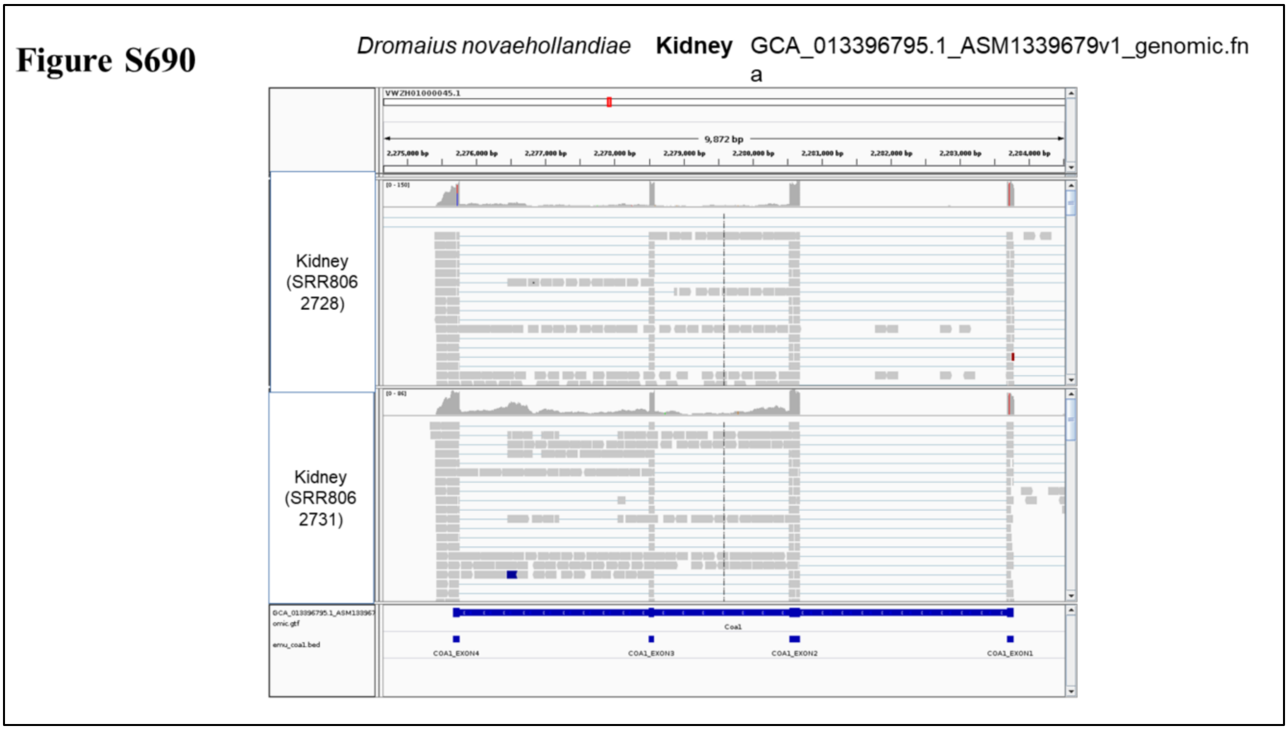

Figure S690: Screenshot of RNA-seq reads aligned to each of the putative locations of the four *COA1* exons in emu (*Dromaius novaehollandiae*). Gray boxes represent reads aligned to the genome and thin lines connecting these boxes represent the spliced read alignments. Datasets from kidney (SRR8062728) and (SRR8062731) have been mapped to the GCA_013396795.1_ASM1339679v1_genomic.fna genome. Locations of each of the exons are depicted as bed record in the bottom row.

Figure S691 *Dromaius novaehollandiae* **Kidney** GCA_013396795.1_ASM1339679v1_genomic.fna

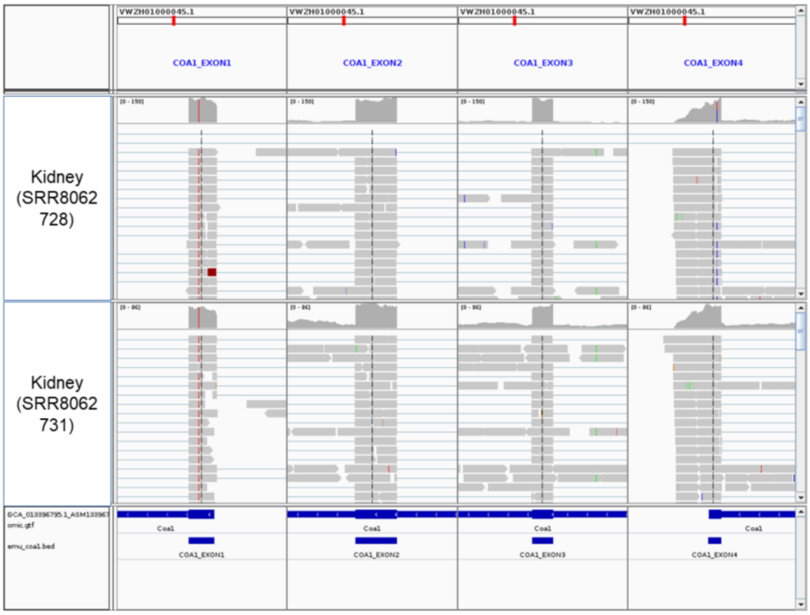

Figure S691: A zoomed in view of each of the *COA1* exons shown in **Supplementary Fig. S690**.

Figure S692

Camarhynchus parvulus **Lower beak** and **Heart** GCF_901933205.1_STF_HiC_genomic.fna

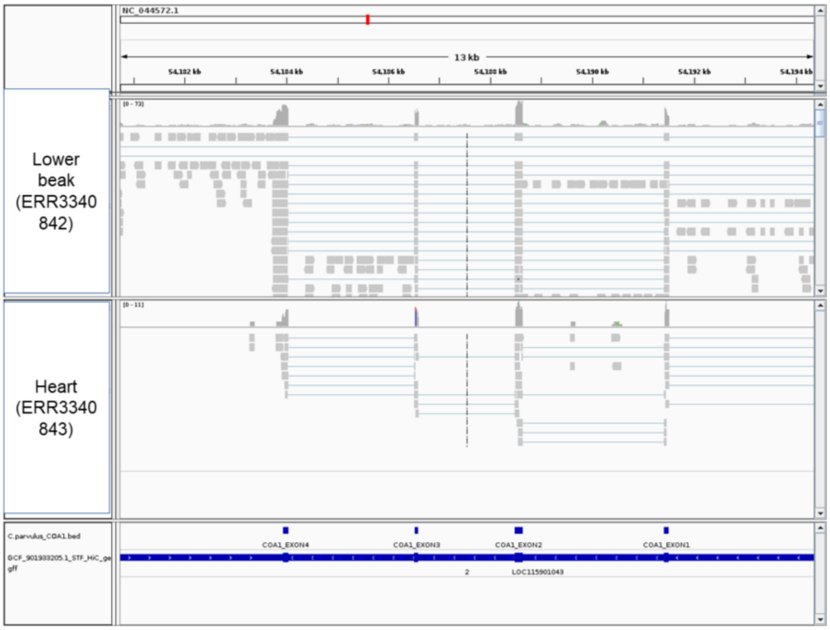

Figure S692: Screenshot of RNA-seq reads aligned to each of the putative locations of the four *COA1* exons in small tree finch (*Camarhynchus parvulus*). Gray boxes represent reads aligned to the genome and thin lines connecting these boxes represent the spliced read alignments. Datasets from lower beak (ERR3340842) and heart (ERR3340843) have been mapped to the GCF_901933205.1_STF_HiC_genomic.fna genome. Locations of each of the exons are depicted as bed record in the bottom row.

Figure S693 *Camarhynchus parvulus* **Lower beak and Heart** GCF_901933205.1_STF_HiC_genomic.fna

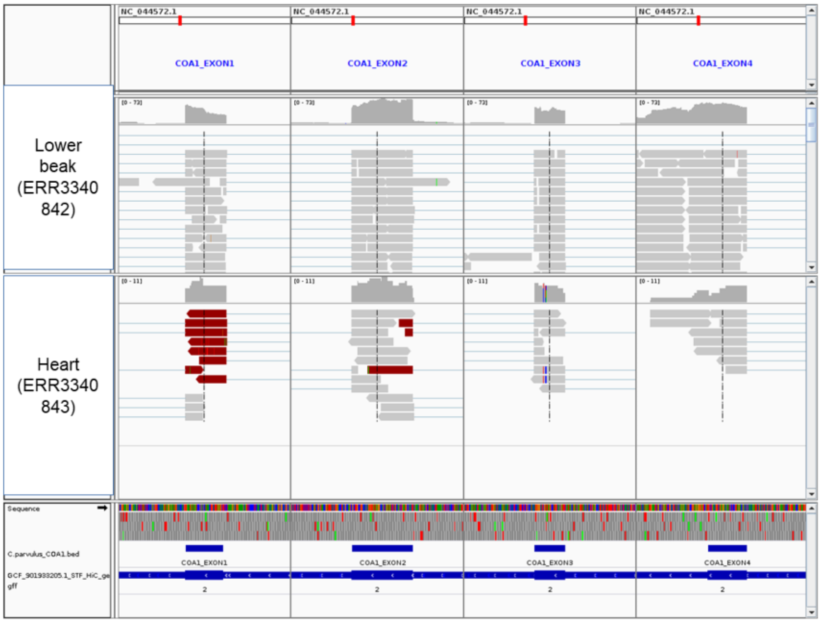

Figure S693: A zoomed in view of each of the *COA1* exons shown in **Supplementary Fig. S692**.

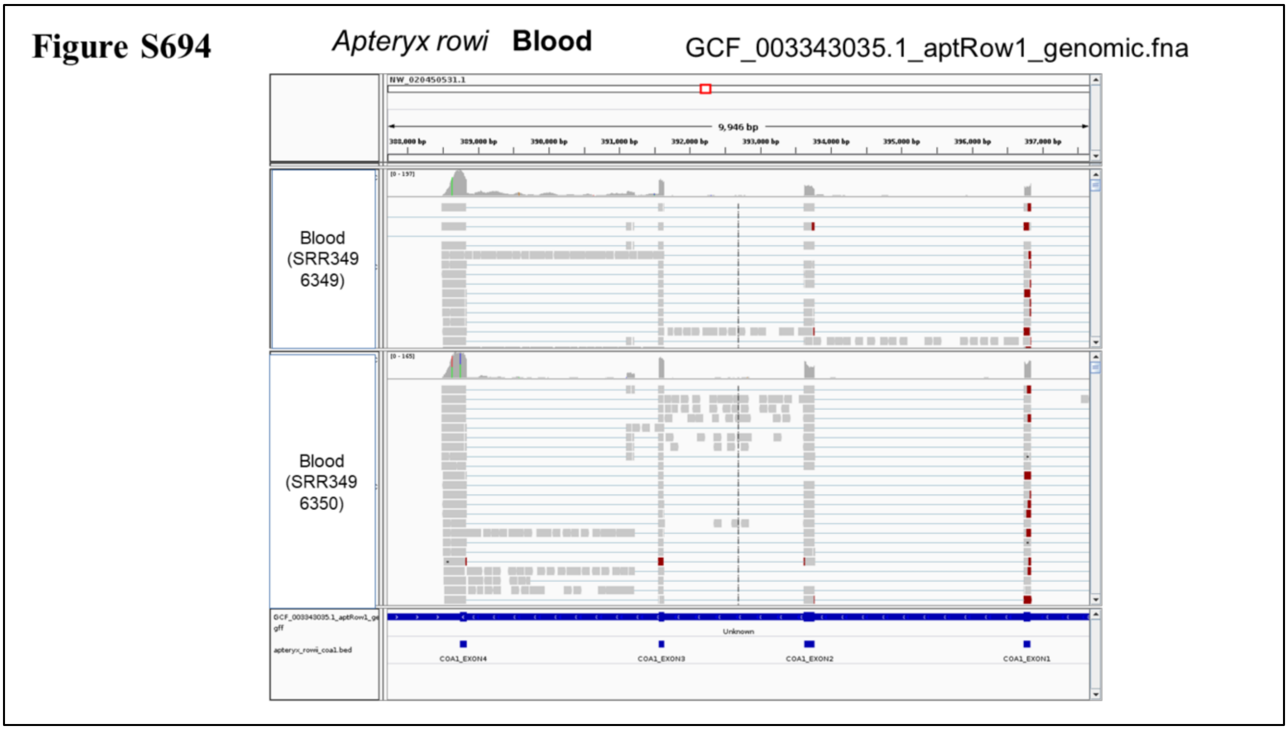

Figure S694: Screenshot of RNA-seq reads aligned to each of the putative locations of the four *COA1* exons in okarito kiwi (*Apteryx rowi*). Gray boxes represent reads aligned to the genome and thin lines connecting these boxes represent the spliced read alignments. Datasets from blood (SRR3496349) and (SRR3496350) have been mapped to the GCF_003343035.1_aptRow1_genomic.fna genome. Locations of each of the exons are depicted as bed record in the bottom row.

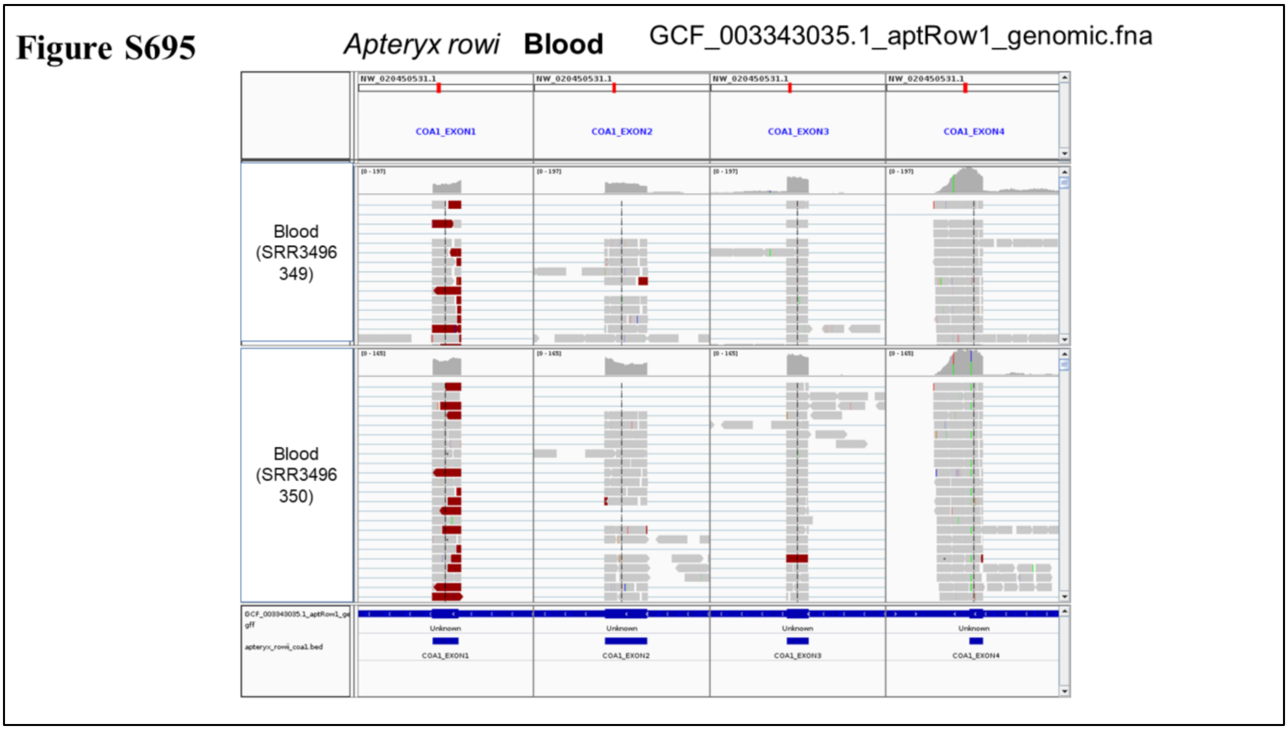

Figure S695: A zoomed in view of each of the *COA1* exons shown in **Supplementary Fig. S694**.

Figure S696 *Aptenodytes forsteri* **Pectoral muscle** GCF_000699145.1_ASM69914v1_genomic.fna

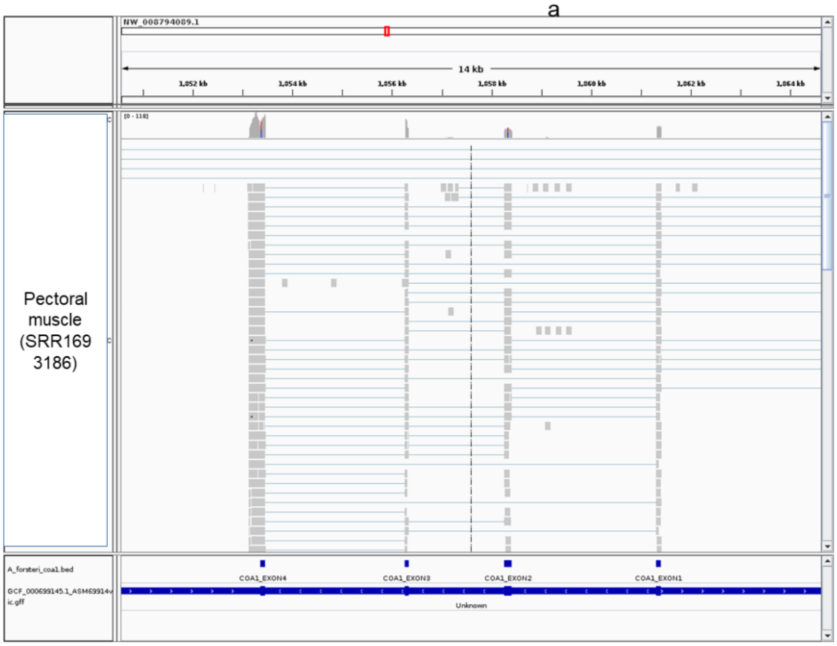

Figure S696: Screenshot of RNA-seq reads aligned to each of the putative locations of the four *COA1* exons in emperor penguin (*Aptenodytes forsteri*). Gray boxes represent reads aligned to the genome and thin lines connecting these boxes represent the spliced read alignments. Datasets from pectoral muscle (SRR1693186) have been mapped to the GCF_000699145.1_ASM69914v1_genomic.fna genome. Locations of each of the exons are depicted as bed record in the bottom row.

Figure S697

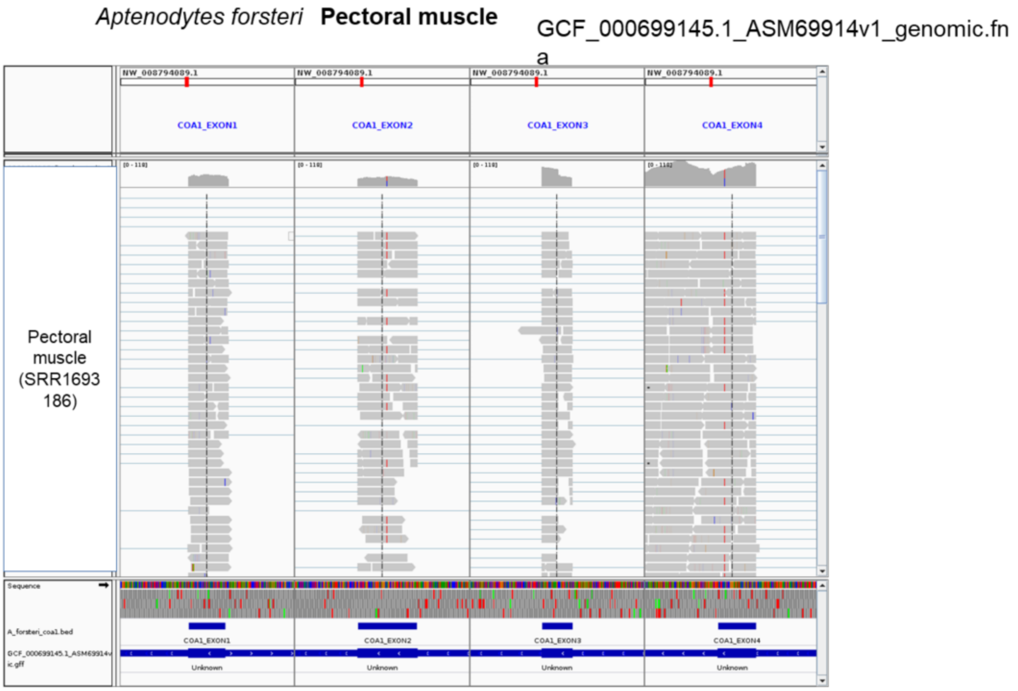

Figure S697: A zoomed in view of each of the *COA1* exons shown in **Supplementary Fig. S696**.

Figure S698 *Oriolus oriolus* **Blood** GCA_013400235.1_ASM1340023v1_genomic.fna

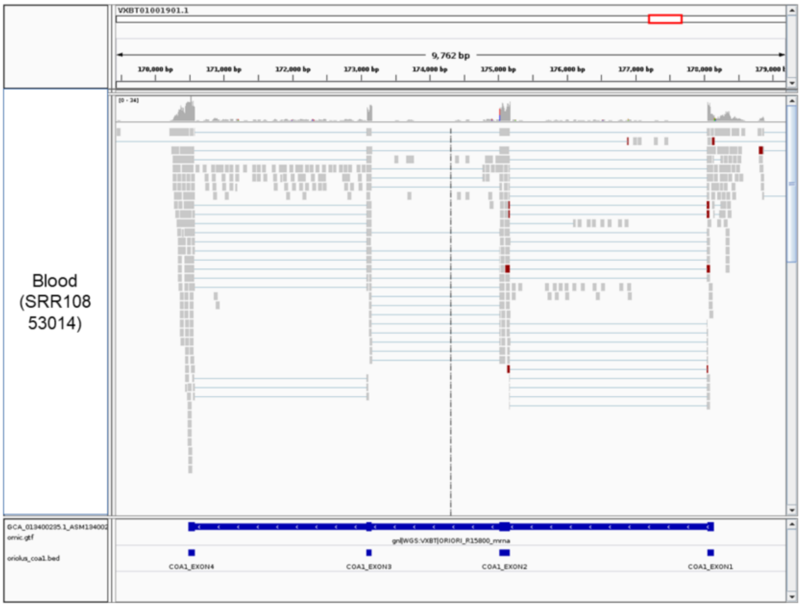

Figure S698: Screenshot of RNA-seq reads aligned to each of the putative locations of the four *COA1* exons in Eurasian golden oriole (*Oriolus oriolus*). Gray boxes represent reads aligned to the genome and thin lines connecting these boxes represent the spliced read alignments. Datasets from blood (SRR10853014) have been mapped to the GCA_013400235.1_ASM1340023v1_genomic.fna genome. Locations of each of the exons are depicted as bed record in the bottom row.

Figure S699 *Oriolus oriolus* **Blood** GCA_013400235.1_ASM1340023v1_genomic.fna

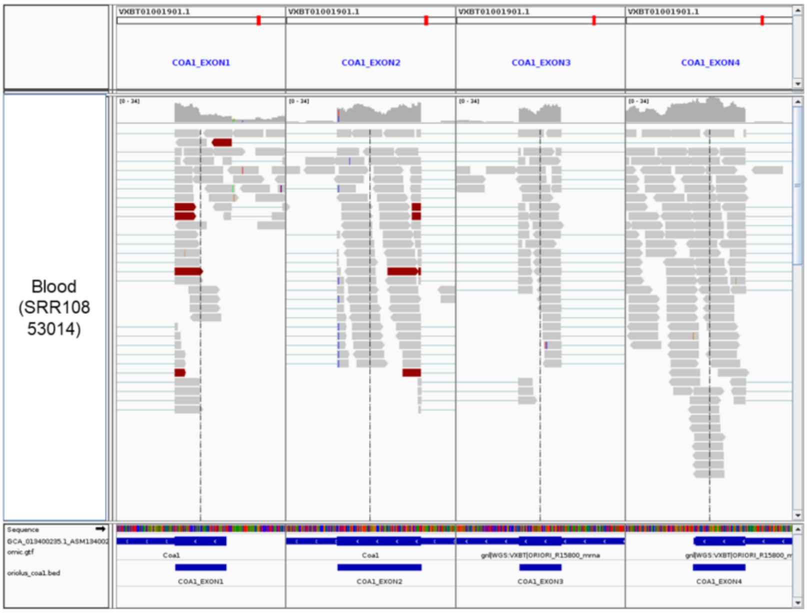

Figure S699: A zoomed in view of each of the *COA1* exons shown in **Supplementary Fig. S688**.

Figure S700 *Manacus vitellinus* **Nidopallium** and **Pectoral muscle** GCF_001715985.3_ASM171598v3_genomic.fna

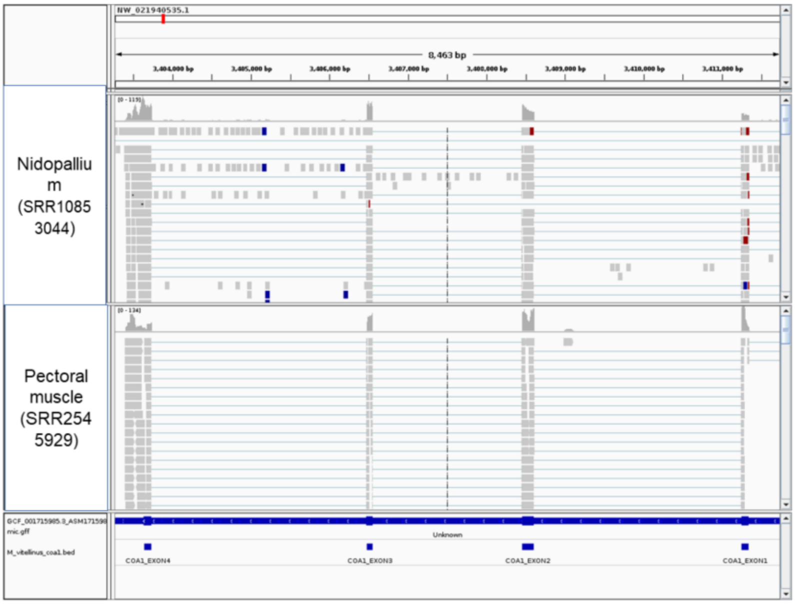

Figure S700: Screenshot of RNA-seq reads aligned to each of the putative locations of the four *COA1* exons in golden-collared manakin (*Manacus vitellinus*). Gray boxes represent reads aligned to the genome and thin lines connecting these boxes represent the spliced read alignments. Datasets from nidopallium (SRR10853044) and pectoral muscle (SRR2545929) have been mapped to the GCF_001715985.3_ASM171598v3_genomic.fna genome. Locations of each of the exons are depicted as bed record in the bottom row.

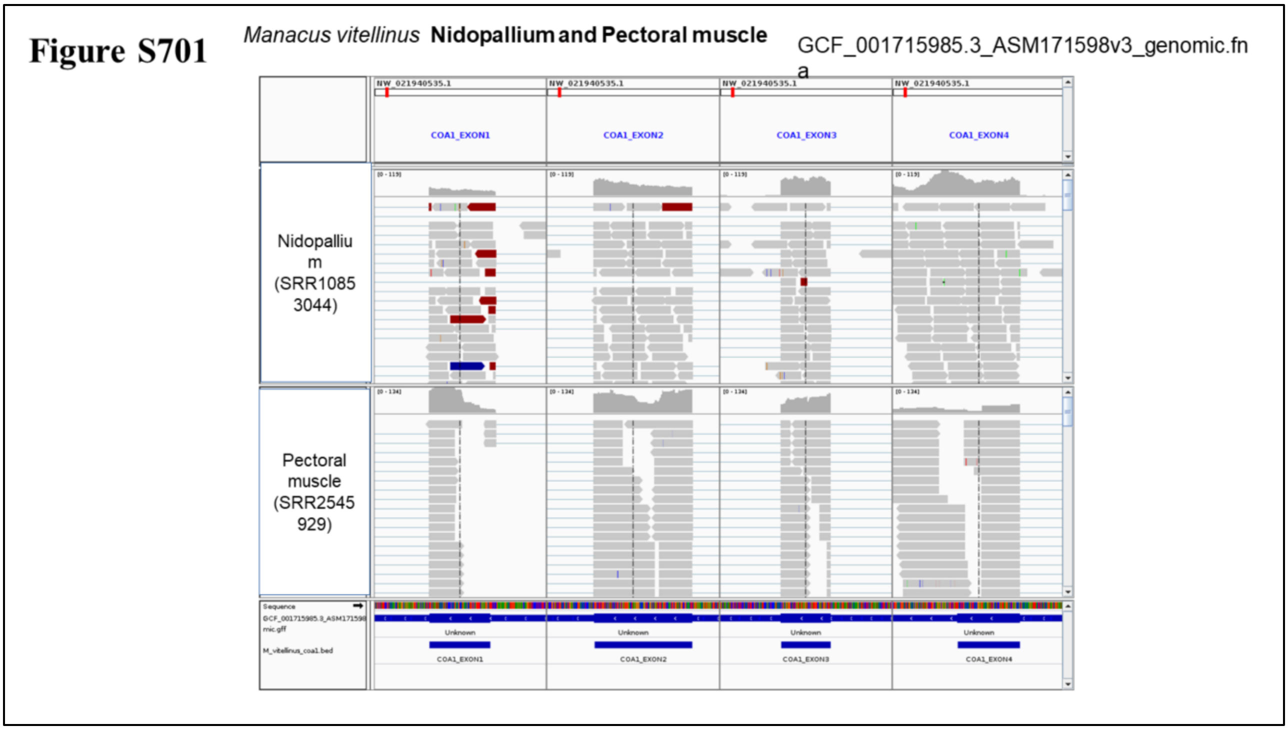

Figure S701: A zoomed in view of each of the *COA1* exons shown in **Supplementary Fig. S700**.

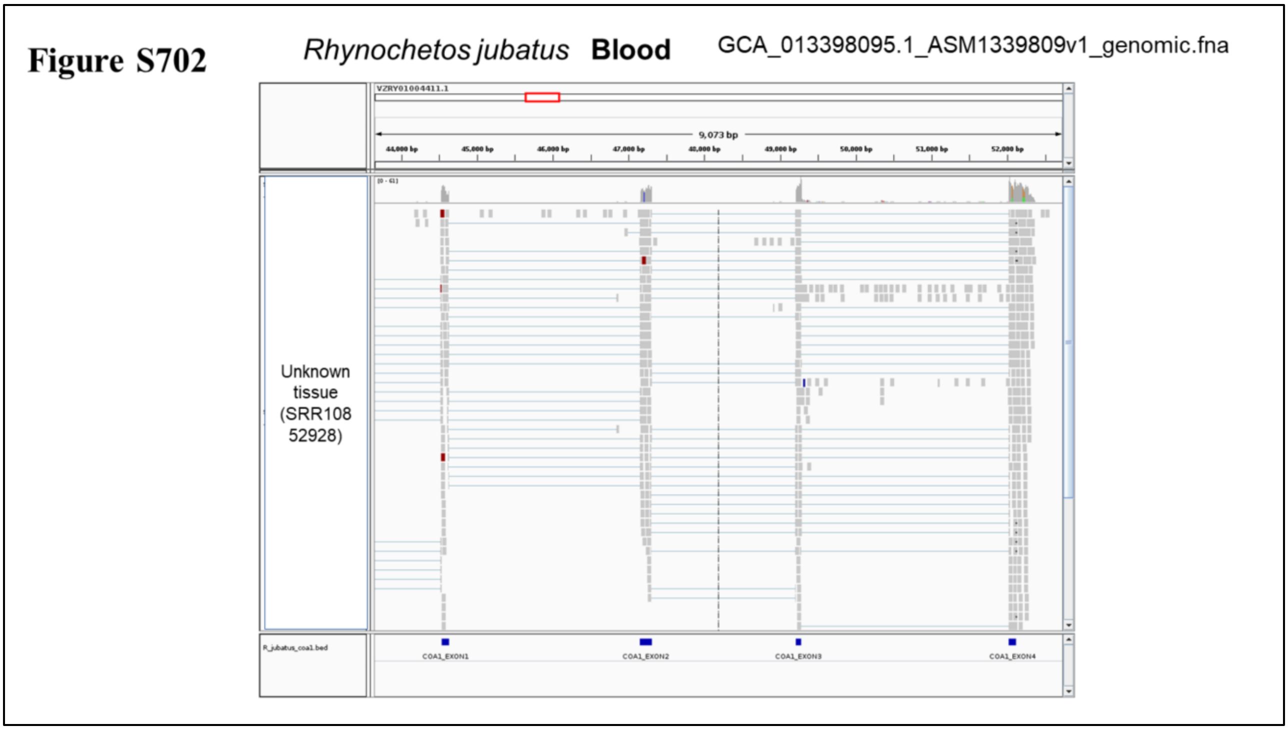

Figure S702: Screenshot of RNA-seq reads aligned to each of the putative locations of the four *COA1* exons in Kagu (*Rhynochetos jubatus*). Gray boxes represent reads aligned to the genome and thin lines connecting these boxes represent the spliced read alignments. Datasets from blood (SRR10852928) have been mapped to the GCA_013398095.1_ASM1339809v1_genomic.fna genome. Locations of each of the exons are depicted as bed record in the bottom row.

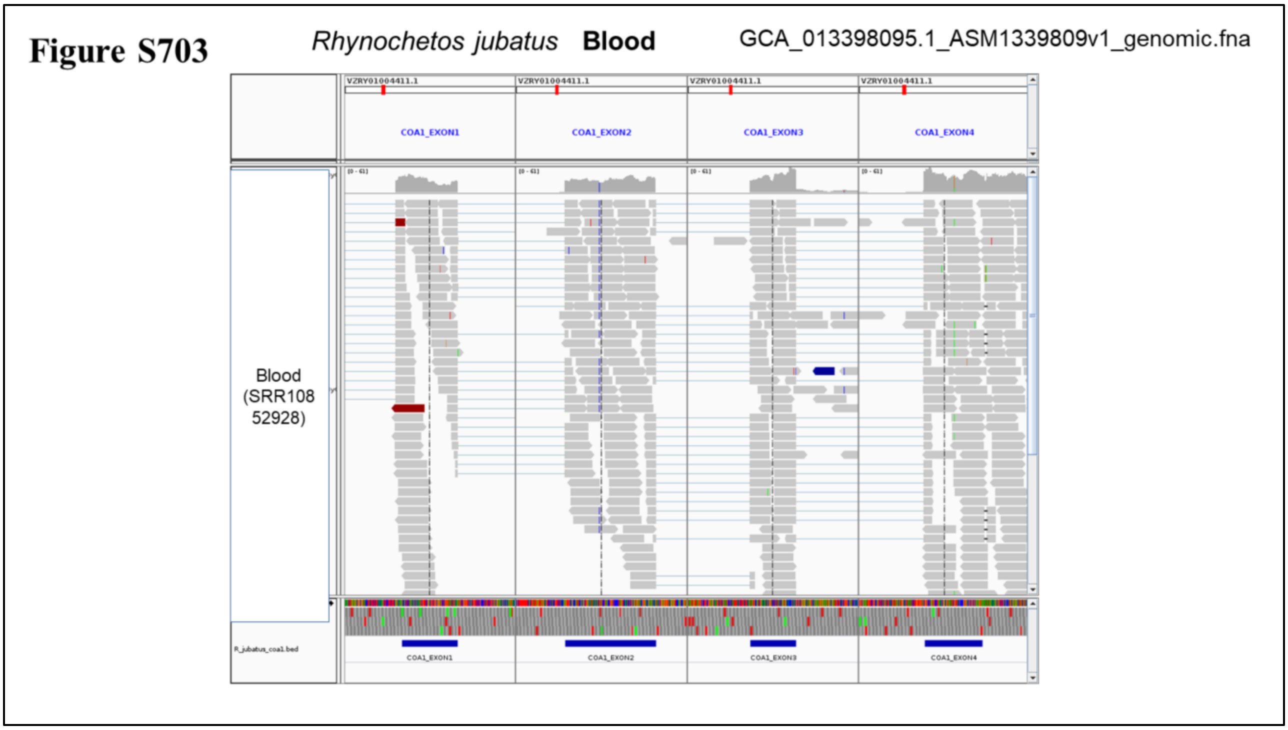

Figure S703: A zoomed in view of each of the *COA1* exons shown in **Supplementary Fig. S702**.

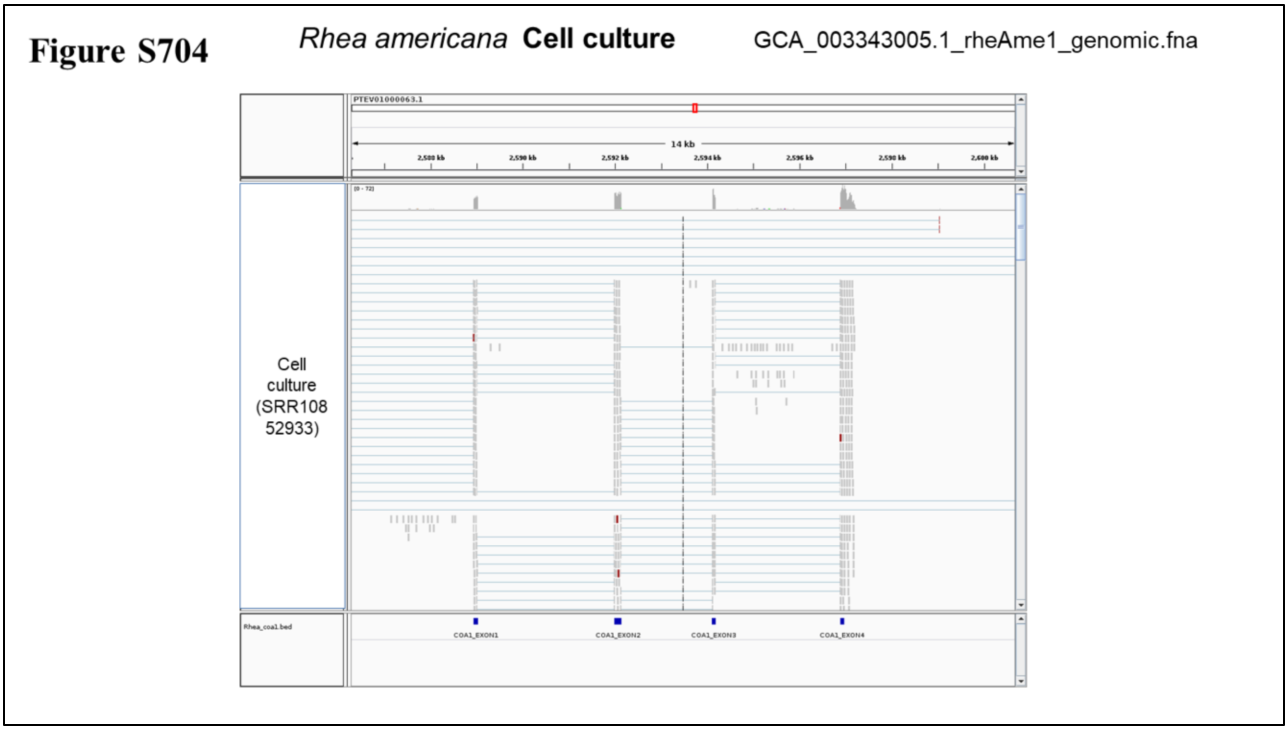

Figure S704: Screenshot of RNA-seq reads aligned to each of the putative locations of the four *COA1* exons in greater rhea (*Rhea americana*). Gray boxes represent reads aligned to the genome and thin lines connecting these boxes represent the spliced read alignments. Datasets from cell culture (SRR10582933) have been mapped to the GCA_003343005.1_rheAme1_genomic.fna genome. Locations of each of the exons are depicted as bed record in the bottom row.

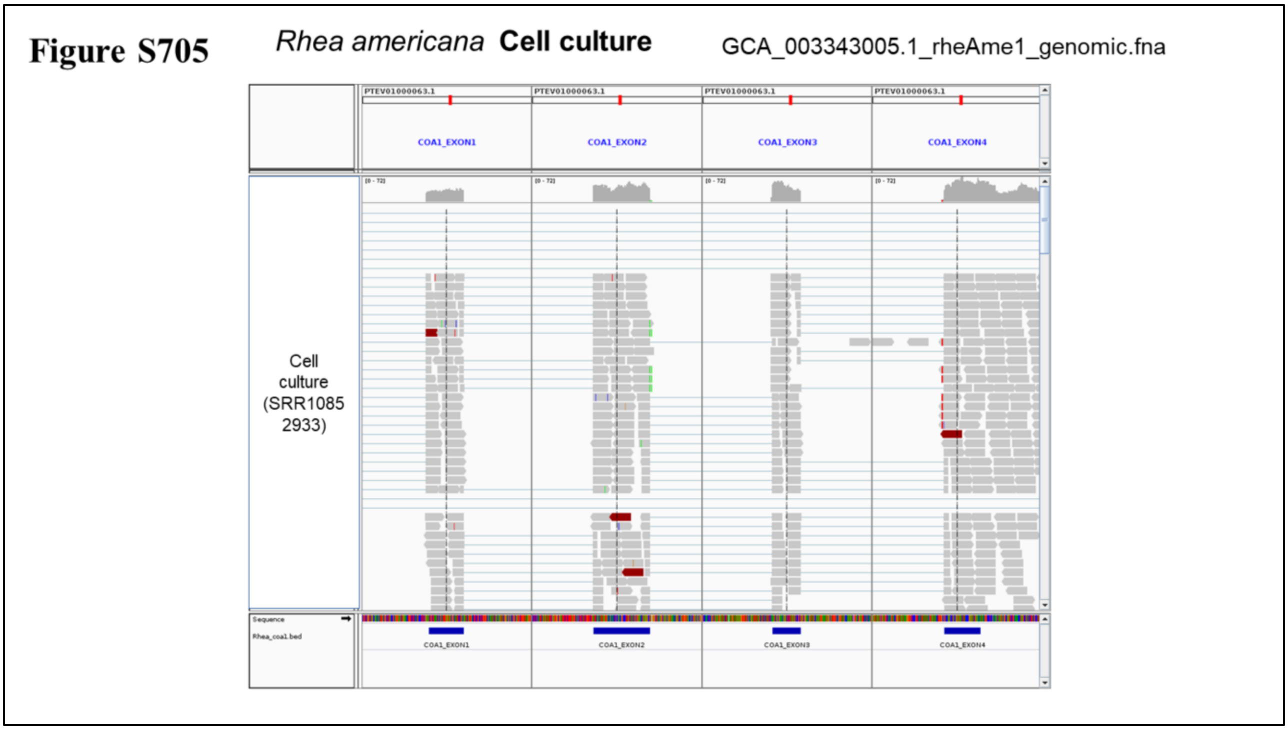

Figure S705: A zoomed in view of each of the *COA1* exons shown in **Supplementary Fig. S704**.

Figure S706

Cyanoderma ruficeps

Blood

GCA_008694505.1_ASM869450v1_genomic.fna

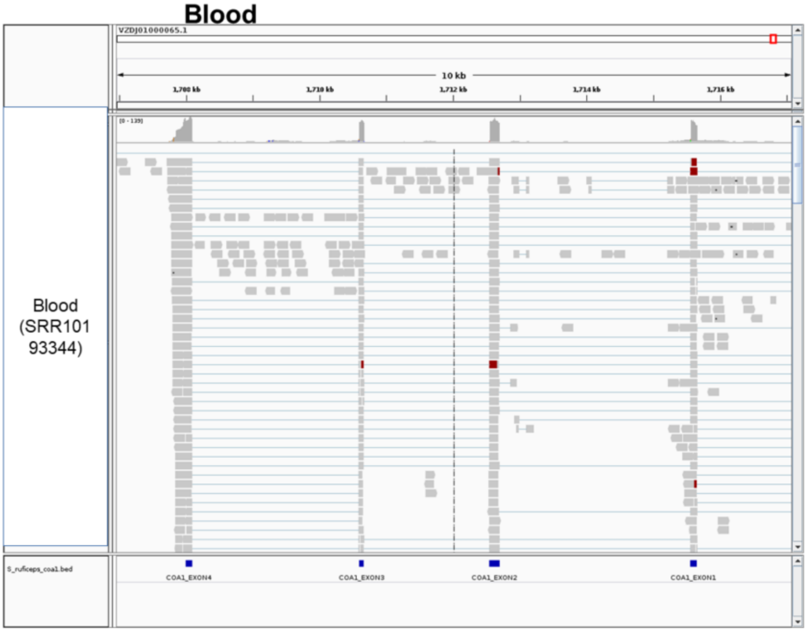

Figure S706: Screenshot of RNA-seq reads aligned to each of the putative locations of the four *COA1* exons in rufous-capped babbler (*Cyanoderma ruficeps*). Gray boxes represent reads aligned to the genome and thin lines connecting these boxes represent the spliced read alignments. Datasets from blood (SRR10193344) have been mapped to the GCA_008694505.1_ASM869450v1_genomic.fna genome. Locations of each of the exons are depicted as bed record in the bottom row.

Figure S707 *Cyanoderma ruficeps* **Blood** GCA_008694505.1_ASM869450v1_genomic.fna

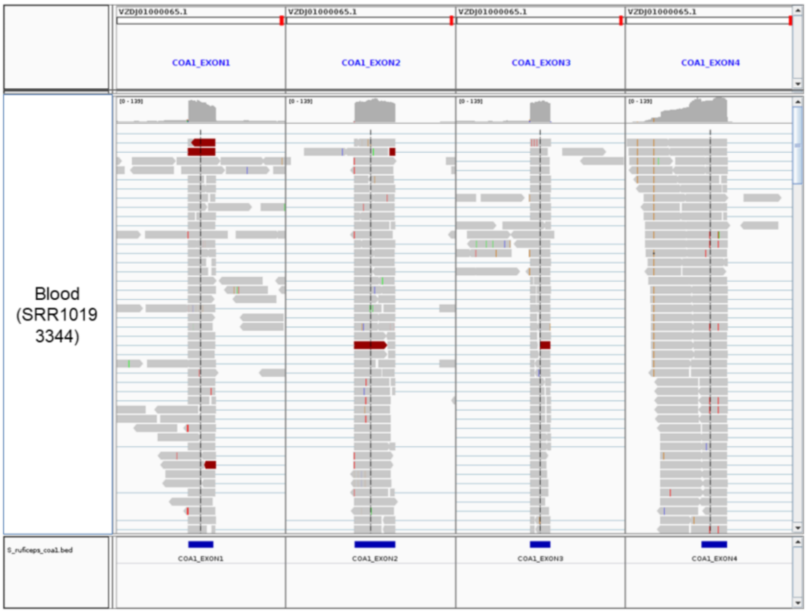

Figure S707: A zoomed in view of each of the *COA1* exons shown in **Supplementary Fig. S706**.

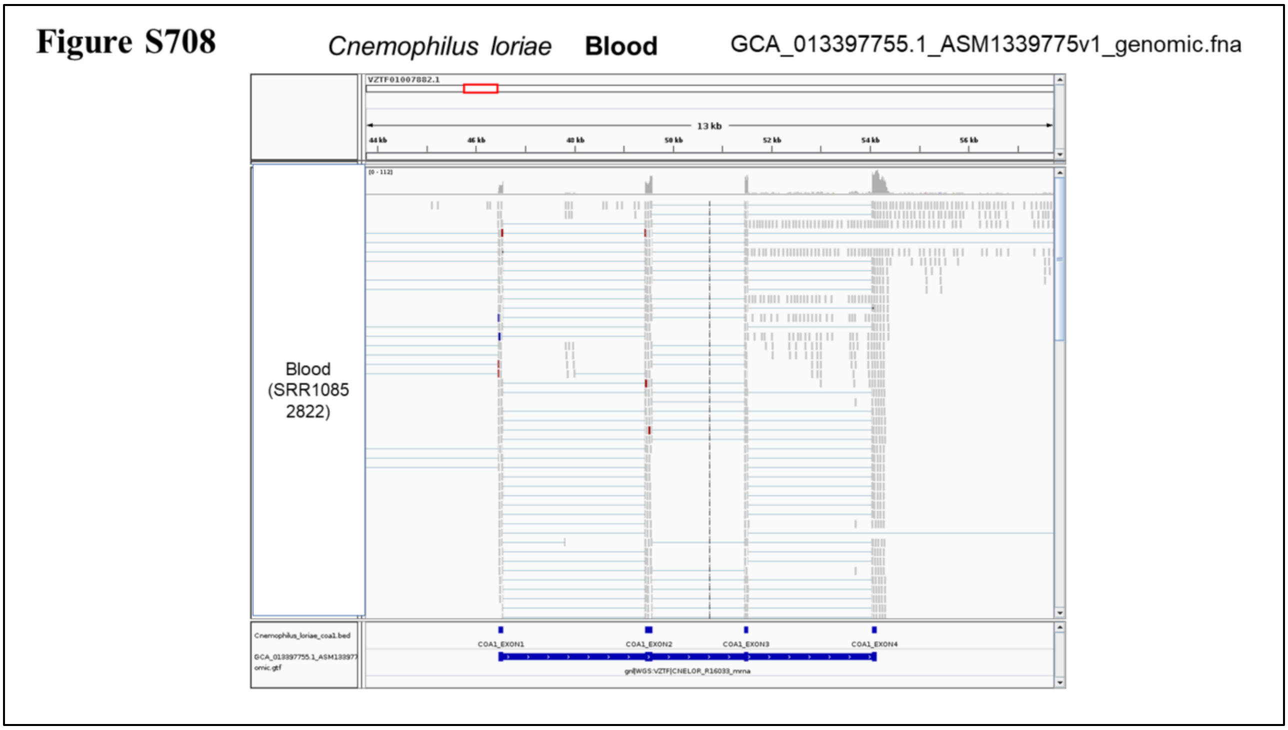

Figure S708: Screenshot of RNA-seq reads aligned to each of the putative locations of the four *COA1* exons in Loria's satinbird (*Cnemophilus loriae*). Gray boxes represent reads aligned to the genome and thin lines connecting these boxes represent the spliced read alignments. Datasets from blood (SRR10852822) have been mapped to the GCA_013397755.1_ASM1339775v1_genomic.fna genome. Locations of each of the exons are depicted as bed record in the bottom row.

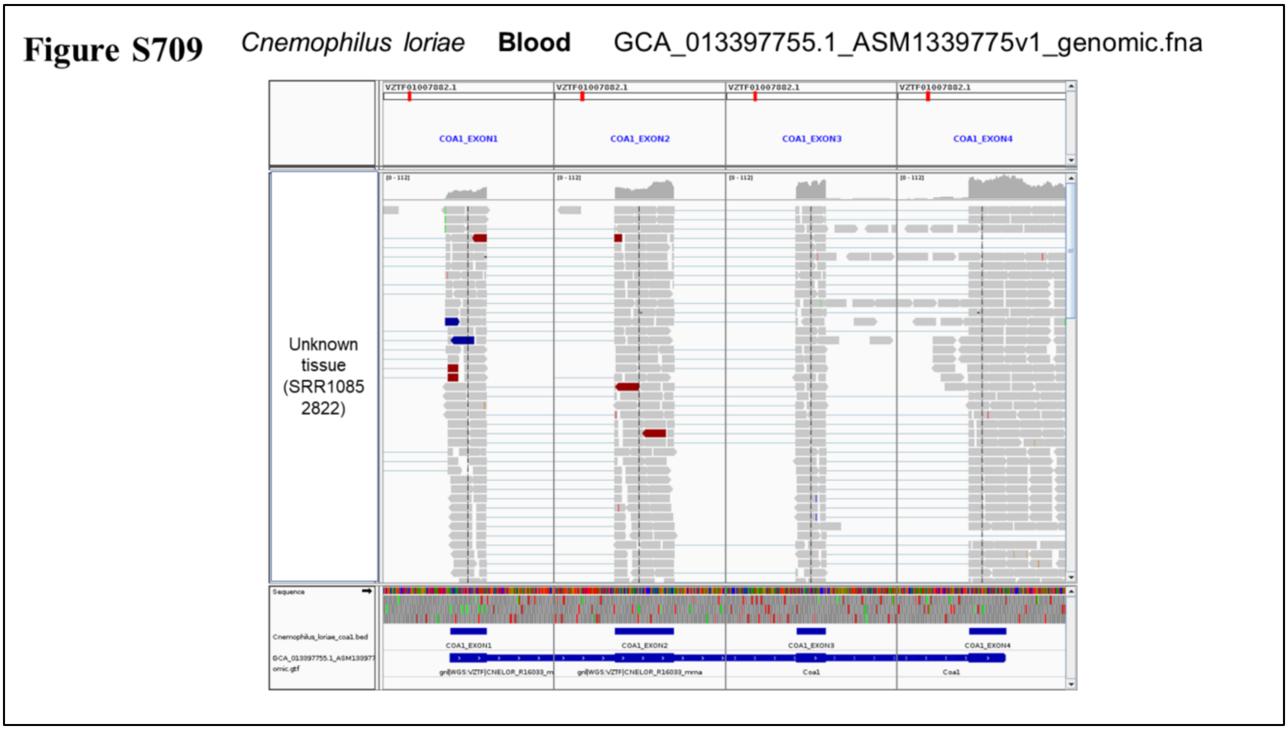

Figure S709: A zoomed in view of each of the *COA1* exons shown in **Supplementary Fig. S708**.

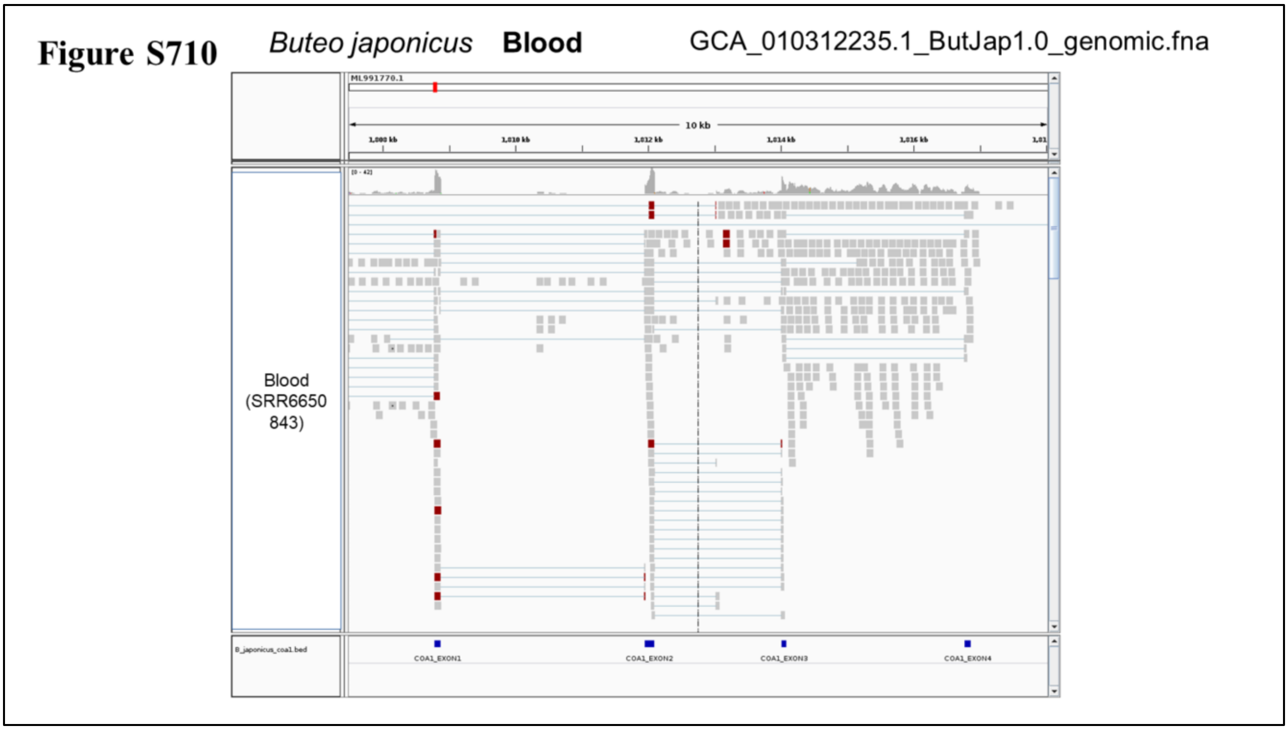

Figure S710: Screenshot of RNA-seq reads aligned to each of the putative locations of the four *COA1* exons in eastern buzzard (*Buteo japonicus*). Gray boxes represent reads aligned to the genome and thin lines connecting these boxes represent the spliced read alignments. Datasets from blood (SRR6650843) have been mapped to the GCA_010312235.1_ButJap1.0_genomic.fna genome. Locations of each of the exons are depicted as bed record in the bottom row.

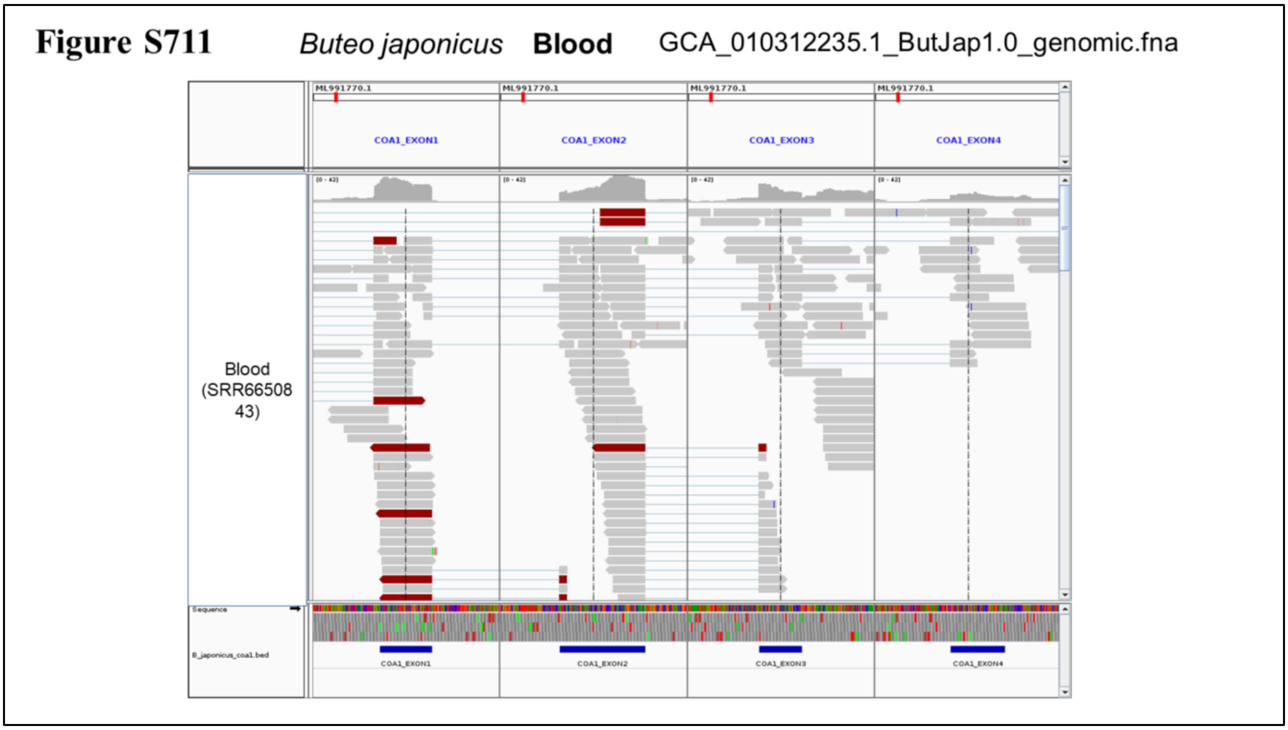

Figure S711: A zoomed in view of each of the *COA1* exons shown in **Supplementary Fig. S710**.

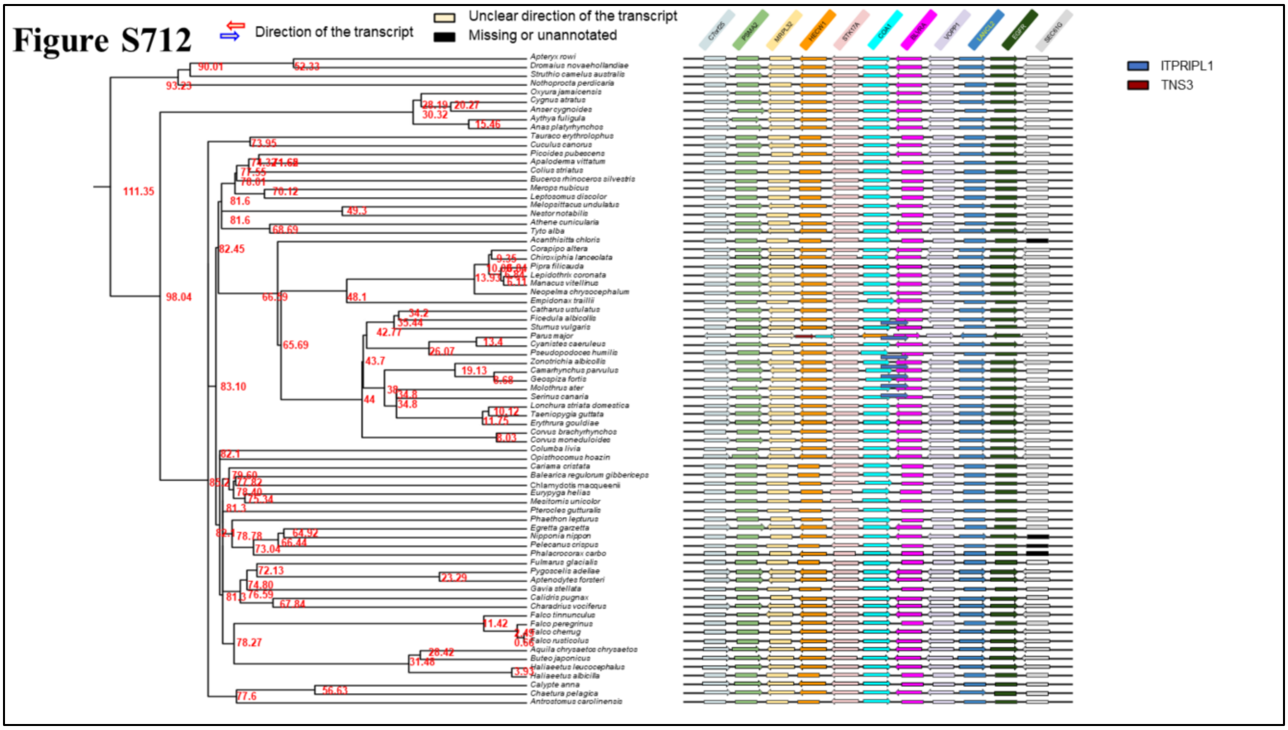

Figure S712: The birds synteny with the time calibrated phylogenetic tree from TimeTree website. The insertion of *TNS3* and rearrangement of *COA1* position occurred in *Parus major* as depicted in figure. The direction of gene transcription is depicted using red and blue arrows relative to the *COA1* gene for consistency across species. Gray boxes represent the genes which are located on short scaffolds and their orientation could not be verified.

Figure S713

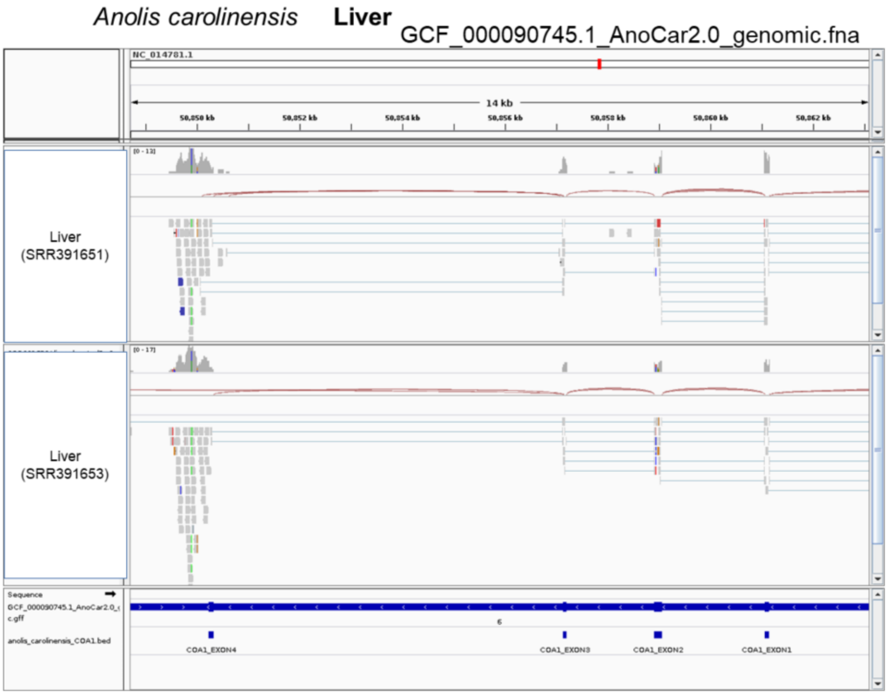

Figure S713: Screenshot of RNA-seq reads aligned to each of the putative locations of the four *COA1* exons in green anole (*Anolis carolinensis*). Gray boxes represent reads aligned to the genome and thin lines connecting these boxes represent the spliced read alignments. Datasets from liver (SRR391651) and (SRR391653) have been mapped to the GCF_000090745.1_AnoCar2.0_genomic.fna genome. Locations of each of the exons are depicted as bed record in the bottom row.

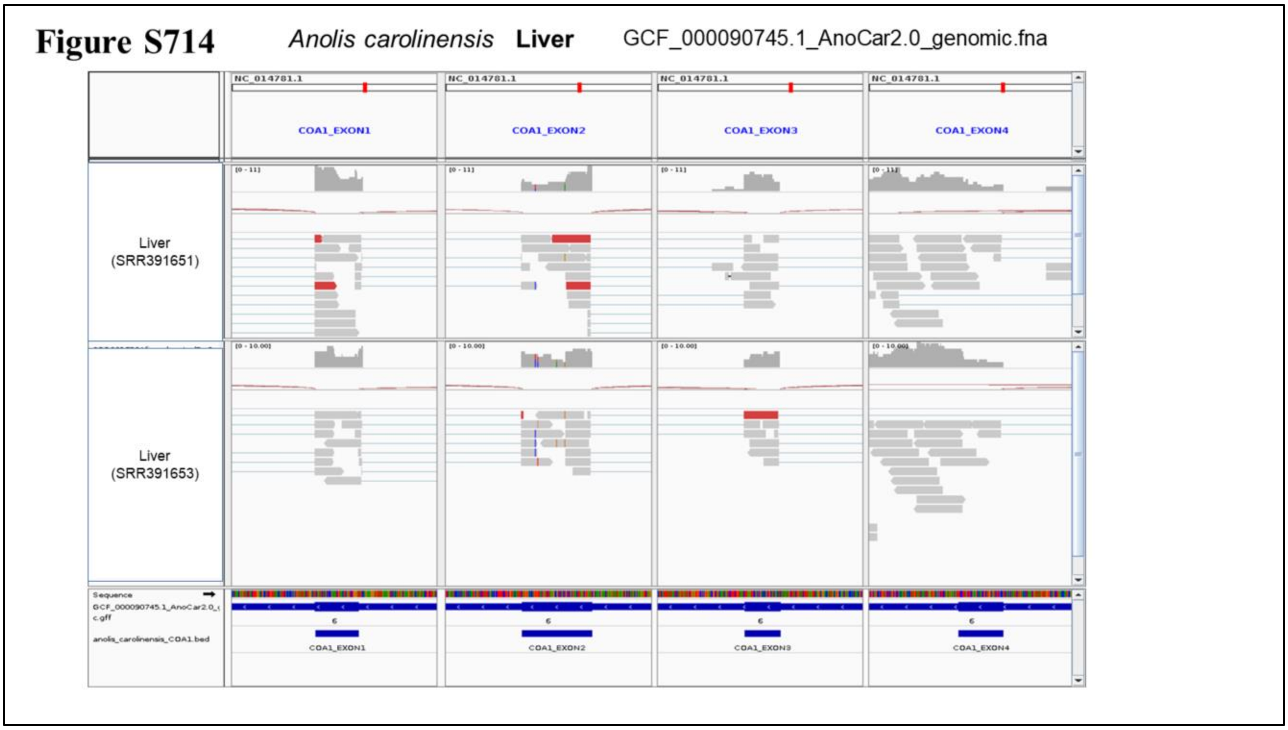

Figure S714: A zoomed in view of each of the *COA1* exons shown in **Supplementary Fig. S484**.

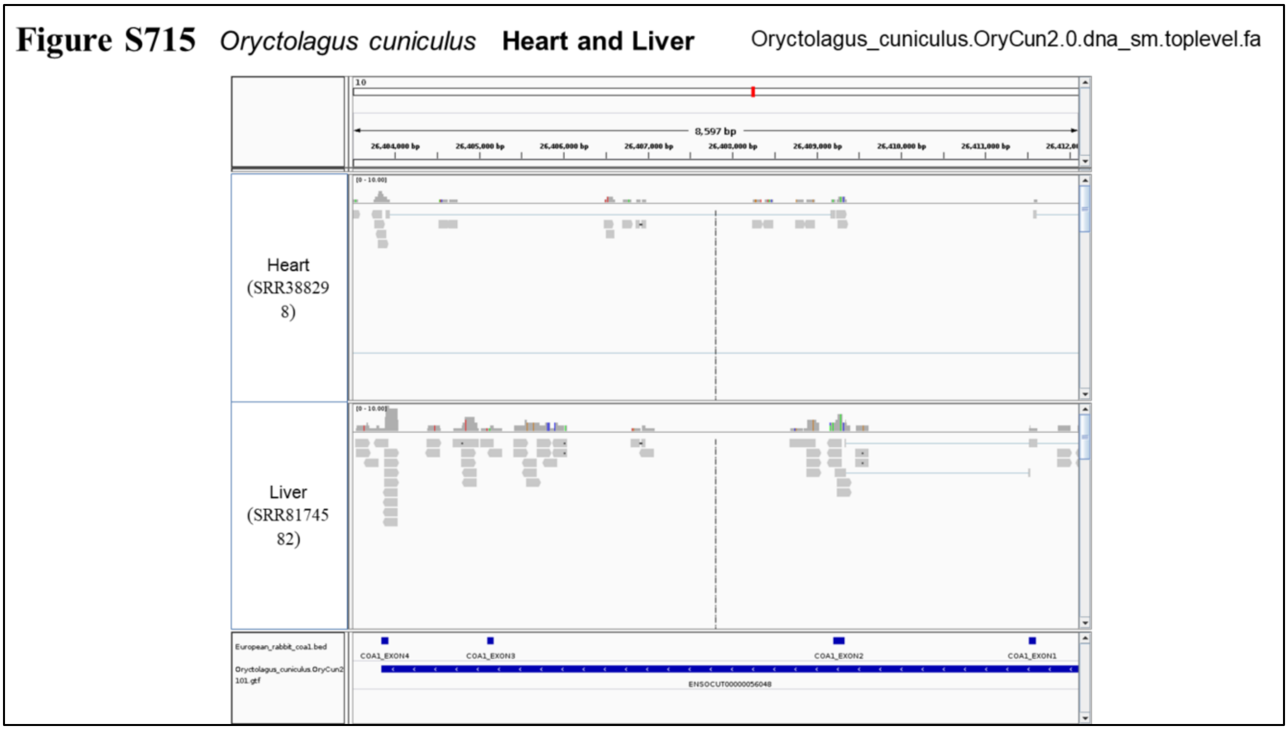

Figure S715: Screenshot of RNA-seq reads aligned to each of the putative locations of the four *COA1* exons in European rabbit (*Oryctolagus cuniculus*). Gray boxes represent reads aligned to the genome and thin lines connecting these boxes represent the spliced read alignments. Datasets from heart (SRR388298) and liver (SRR8174582) have been mapped to the Oryctolagus_cuniculus.OryCun2.0.dna_sm.toplevel.fa. Locations of each of the exons are depicted as bed record in the bottom row.

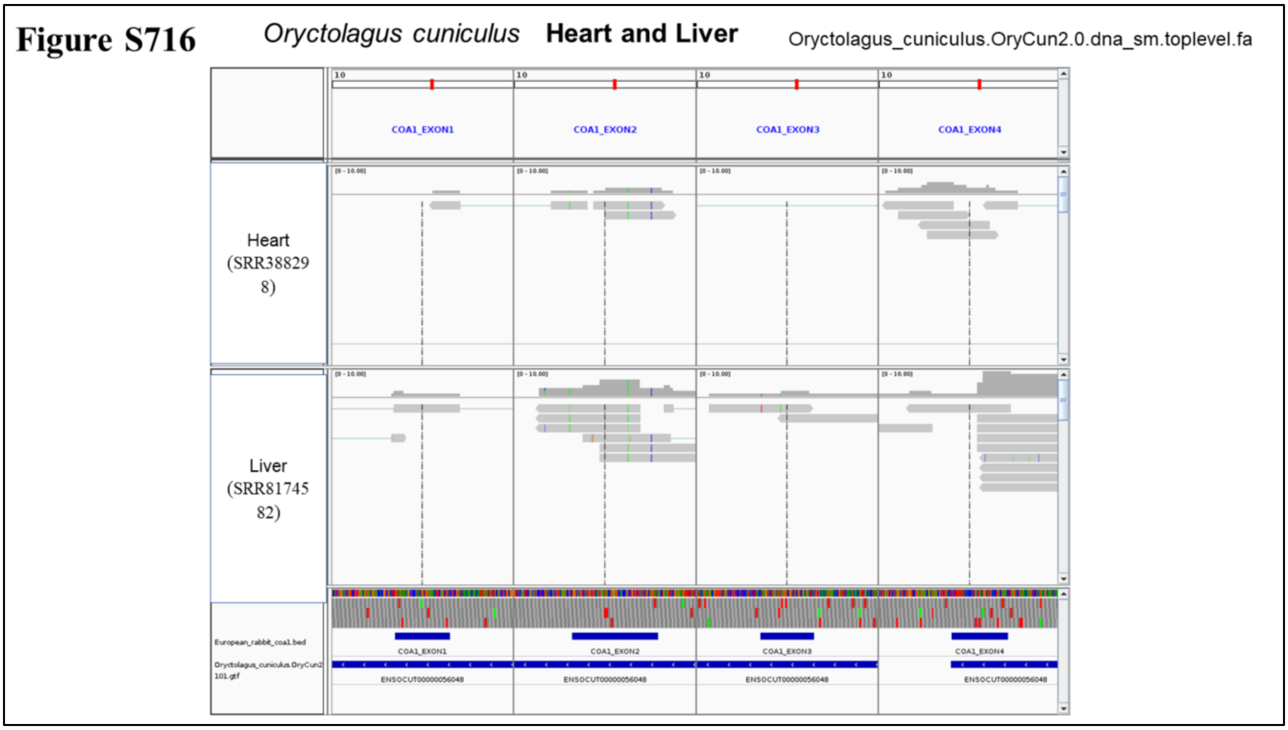

Figure S716: A zoomed in view of each of the *COA1* exons shown in **Supplementary Fig. S715**.

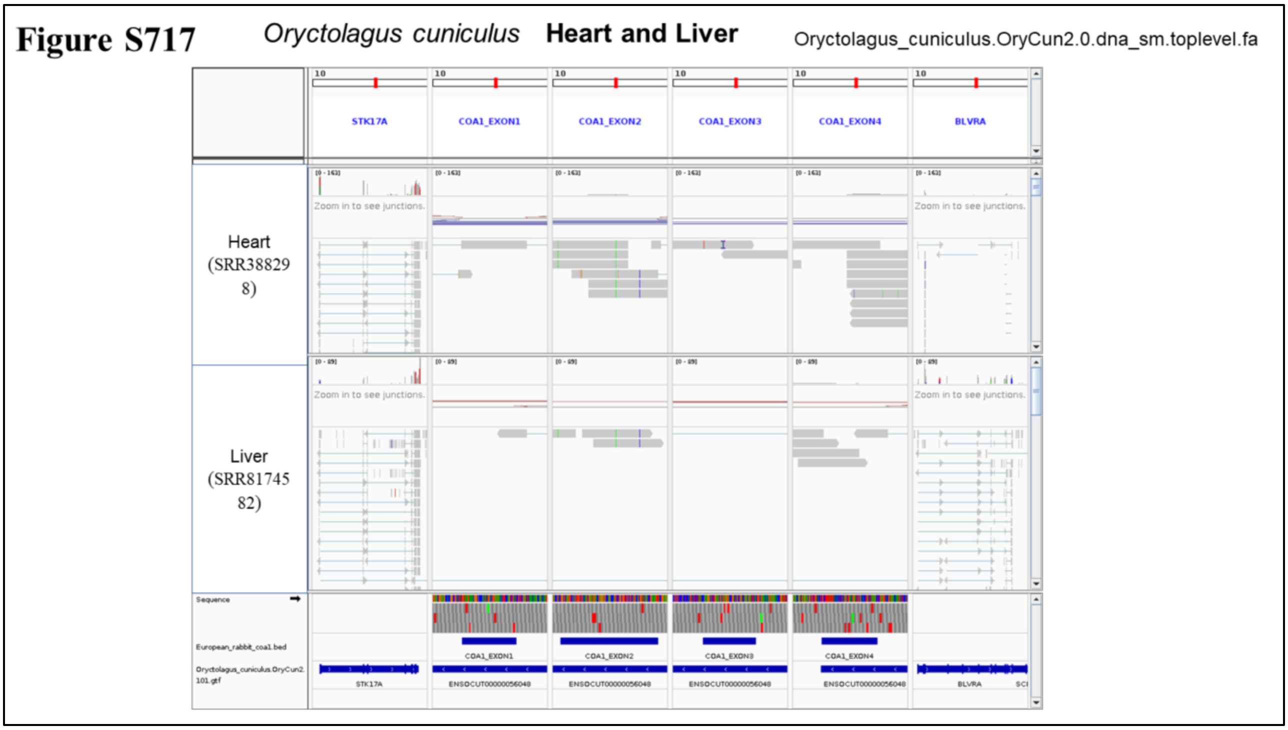

Figure S717: Screenshot of RNA-seq reads aligned to *BLVRA* and *STK17A* to *COA1* exon 1 and 4 respectively in European rabbit (*Oryctolagus cuniculus*). Gray boxes represent reads aligned to the genome and thin lines connecting these boxes represent the spliced read alignments. Datasets from heart (SRR388298) and liver (SRR8174582) have been mapped to the Oryctolagus_cuniculus.OryCun2.0.dna_sm.toplevel.fa. Locations of each of the exons are depicted as bed record in the bottom row.

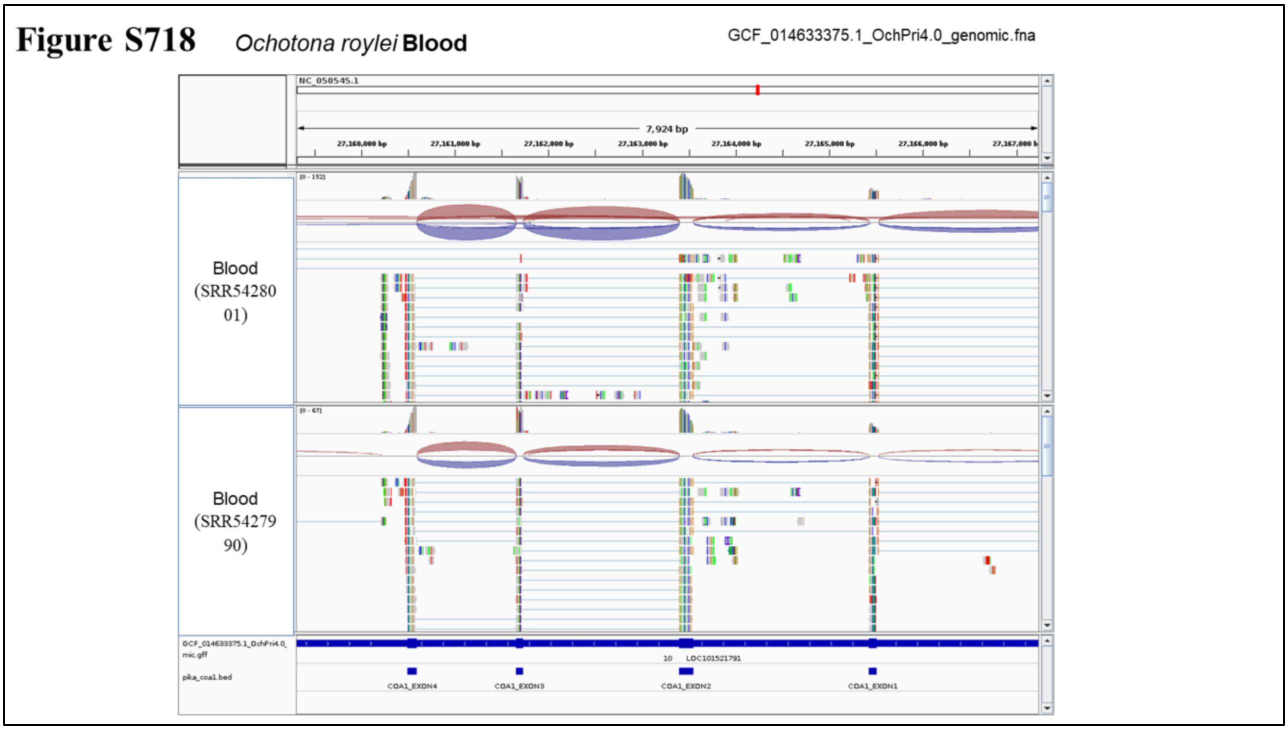

Figure S718: Screenshot of RNA-seq reads aligned to each of the putative locations of the four *COA1* exons in Royle's pika (*Ochotona roylei*). Gray boxes represent reads aligned to the genome and thin lines connecting these boxes represent the spliced read alignments. Datasets from blood (SRR5428001 and SRR5427990) have been mapped to the GCF_014633375.1_OchPri4.0_genomic.fna. Locations of each of the exons are depicted as bed record in the bottom row.

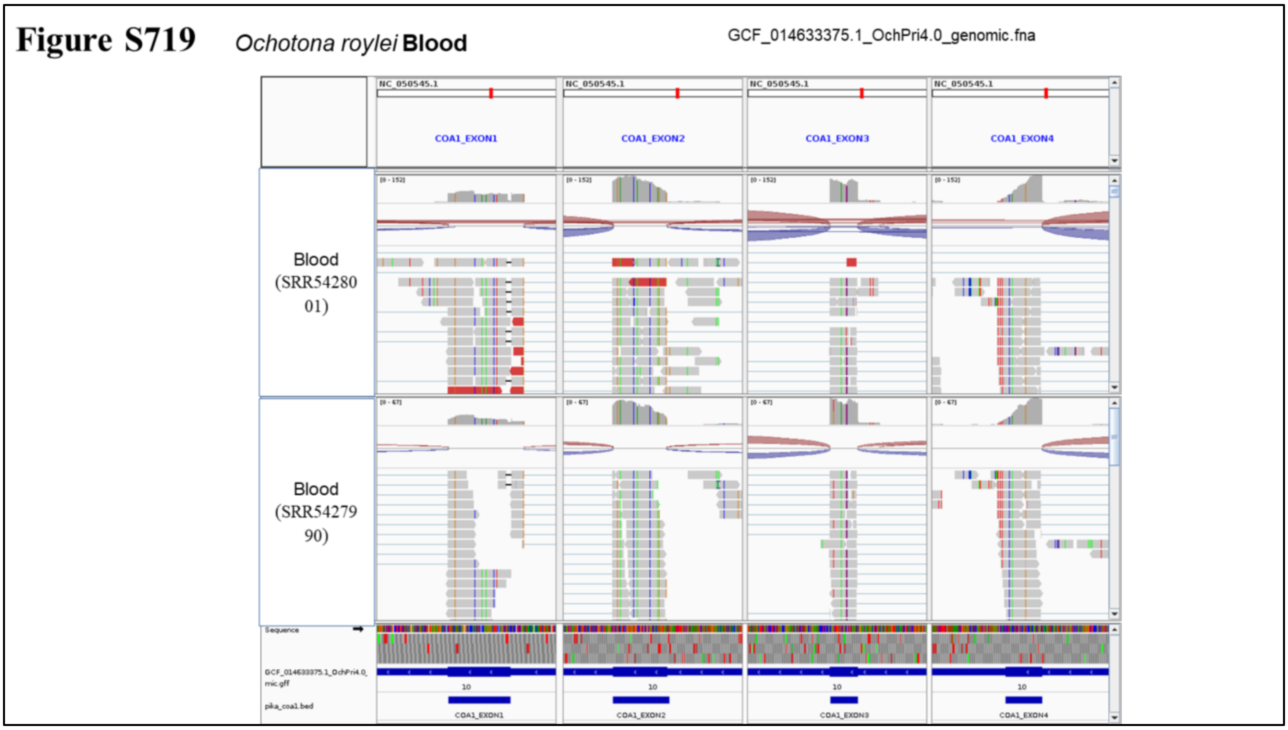

Figure S719: Screenshot of RNA-seq reads aligned to each of the putative locations of the four *COA1* exonwise in Royle's pika (*Ochotona roylei*). Gray boxes represent reads aligned to the genome and thin lines connecting these boxes represent the spliced read alignments. Datasets from blood (SRR5428001 and SRR5427990) have been mapped to the GCF_014633375.1_OchPri4.0_genomic.fna. Locations of each of the exons are depicted as bed record in the bottom row.

Figure S720 (*Heterocephalus glaber)* Ovary and Spleen Heterocephalus_glaber_male.HetGla_1.0.dna_sm.toplevel.fa

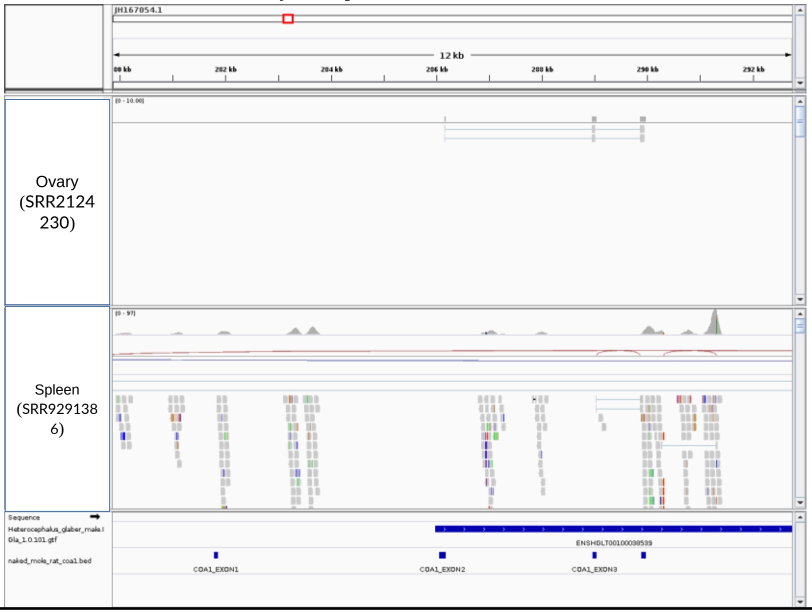

Figure S720: Screenshot of RNA-seq reads aligned to each of the putative locations of the four *COA1* exons in naked mole-rat (*Heterocephalus glaber*). Gray boxes represent reads aligned to the genome and thin lines connecting these boxes represent the spliced read alignments. Datasets from ovary and spleen (SRR2124230 and SRR9291386) respectively have been mapped to the Heterocephalus_glaber_male.HetGla_1.0.dna_sm.toplevel.fa. Locations of each of the exons are depicted as bed record in the bottom row.

Figure S721 (*Heterocephalus glaber)* Ovary and Spleen Heterocephalus_glaber_male.HetGla_1.0.dna_sm.toplevel.fa

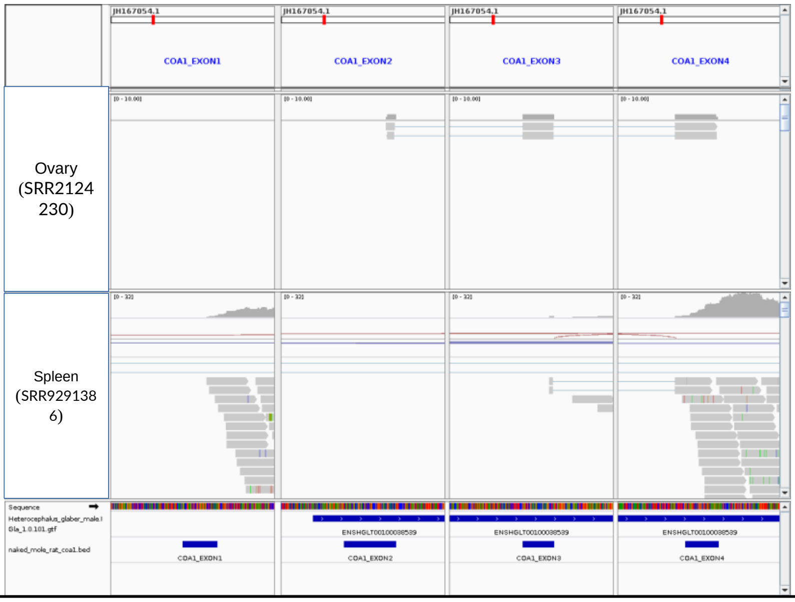

Figure S721: A zoomed in view of each of the *COA1* exons shown in **Supplementary Fig. S720**.

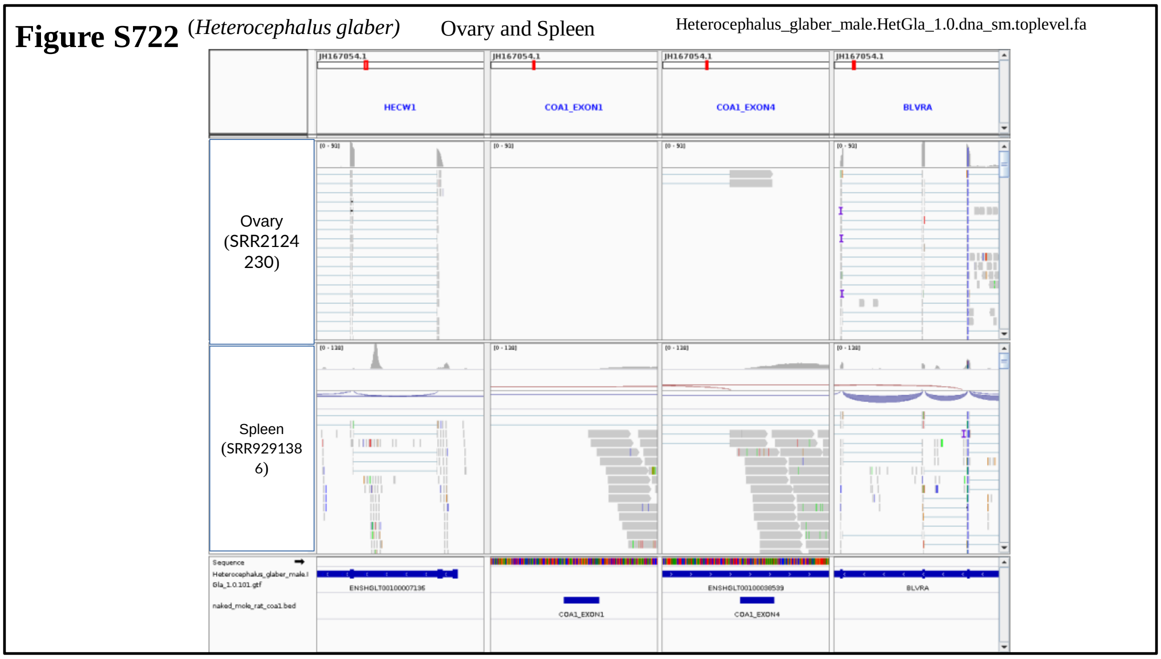

Figure S722: Screenshot of RNA-seq reads aligned to *BLVRA* and *STK17A* to *COA1* exons in naked mole-rat (*Heterocephalus glaber*). Gray boxes represent reads aligned to the genome and thin lines connecting these boxes represent the spliced read alignments. Datasets from ovary and spleen (SRR2124230 and SRR9291386) respectively have been mapped to the Heterocephalus_glaber_male.HetGla_1.0.dna_sm.toplevel.fa. Locations of each of the exons are depicted as bed record in the bottom row.

Figure S723 (*Heterocephalus glaber*) Testis and Liver Heterocephalus_glaber_male.HetGla_1.0.dna_sm.toplevel.fa

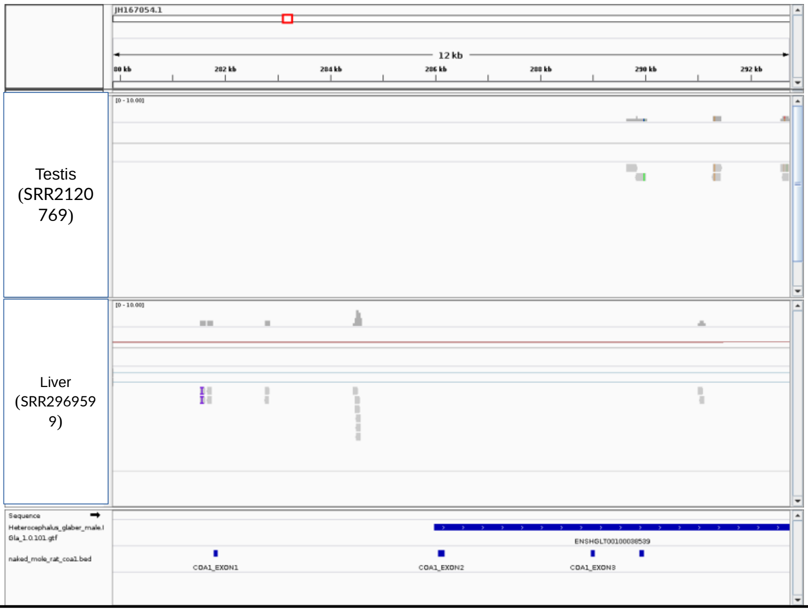

Figure S723: Screenshot of RNA-seq reads aligned to each of the putative locations of the four *COA1* exons in naked mole-rat (*Heterocephalus glaber*). Gray boxes represent reads aligned to the genome and thin lines connecting these boxes represent the spliced read alignments. Datasets from testis and liver (SRR2120769 and SRR2969599) respectively have been mapped to the Heterocephalus_glaber_male.HetGla_1.0.dna_sm.toplevel.fa. Locations of each of the exons are depicted as bed record in the bottom row.

Figure S724 *(Heterocephalus glaber)* Testis and Liver Heterocephalus_glaber_male.HetGla_1.0.dna_sm.toplevel.fa

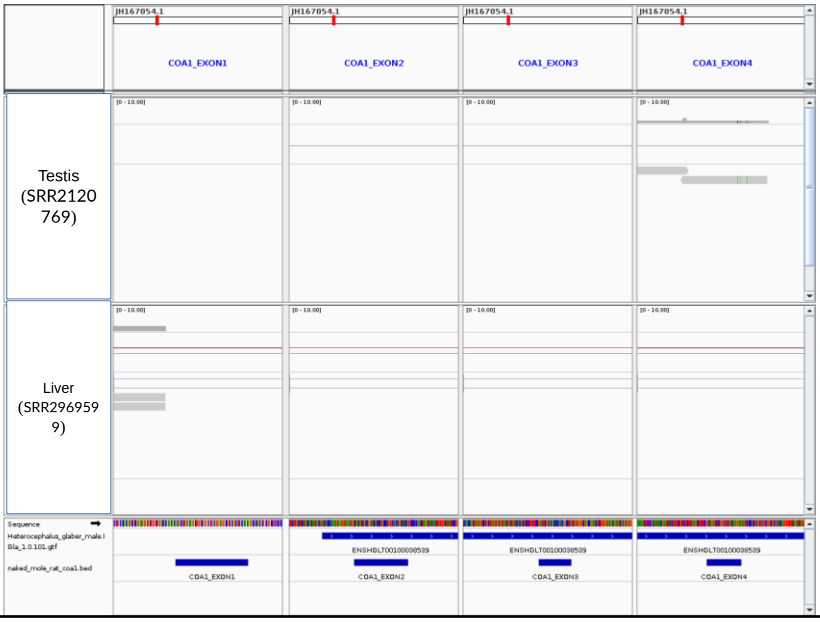

Figure S724: A zoomed in view of each of the *COA1* exons shown in **Supplementary Fig. S723**.

Figure S725 *(Heterocephalus glaber)* Testis and Liver Heterocephalus_glaber_male.HetGla_1.0.dna_sm.toplevel.fa

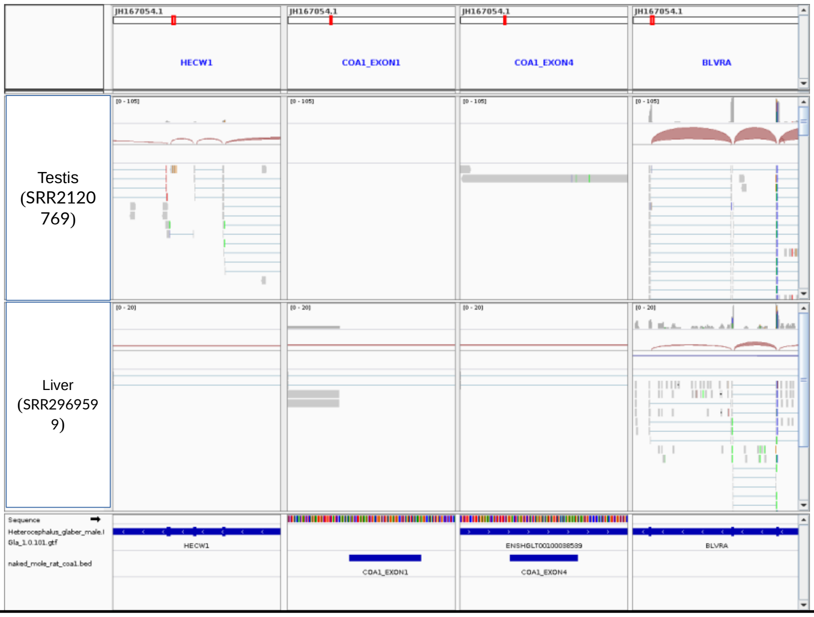

Figure S725: Screenshot of RNA-seq reads aligned to *BLVRA* and *STK17A* to *COA1* exons in naked mole-rat (*Heterocephalus glaber*). Gray boxes represent reads aligned to the genome and thin lines connecting these boxes represent the spliced read alignments. Datasets from testis and liver (SRR2120769 and SRR2969599) respectively have been mapped to the Heterocephalus_glaber_male.HetGla_1.0.dna_sm.toplevel.fa. Locations of each of the exons are depicted as bed record in the bottom row.

Figure S726 (*Heterocephalus glaber)* Root ganglion and Spinal cord Heterocephalus_glaber_male.HetGla_1.0.dna_sm.toplevel.fa

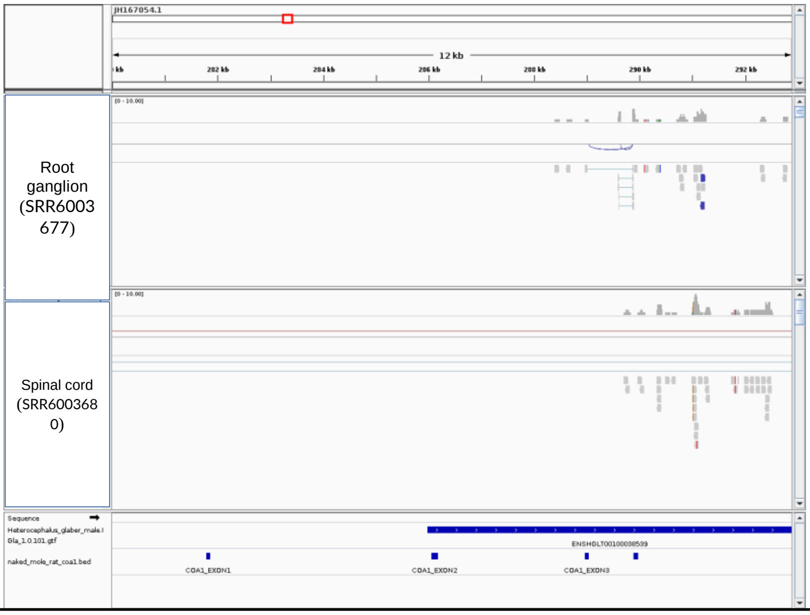

Figure S726: Screenshot of RNA-seq reads aligned to each of the putative locations of the four *COA1* exons in naked mole-rat (*Heterocephalus glaber*). Gray boxes represent reads aligned to the genome and thin lines connecting these boxes represent the spliced read alignments. Datasets from root ganglion and spinal cord (SRR6003677 and SRR6003680) respectively have been mapped to the Heterocephalus_glaber_male.HetGla_1.0.dna_sm.toplevel.fa. Locations of each of the exons are depicted as bed record in the bottom row.

Figure S727 (*Heterocephalus glaber)* Root ganglion and Spinal cord Heterocephalus_glaber_male.HetGla_1.0.dna_sm.toplevel.fa

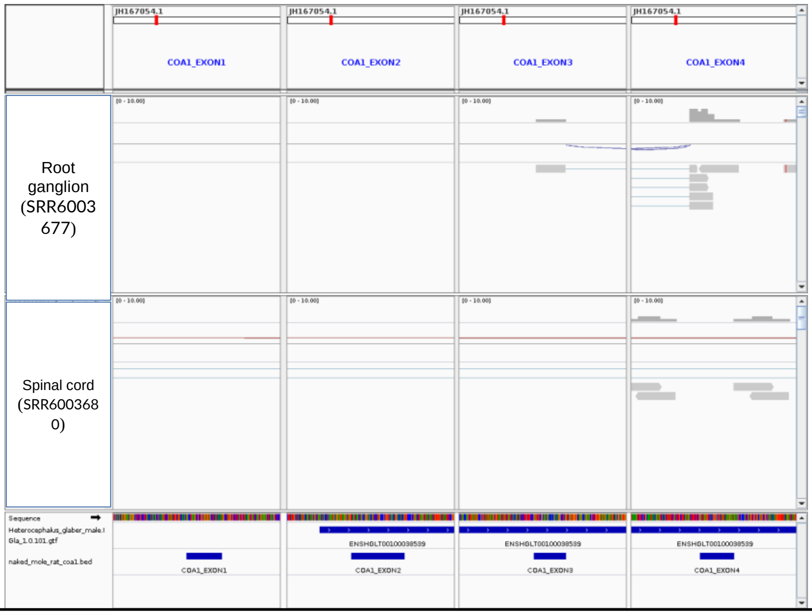

Figure S727: A zoomed in view of each of the *COA1* exons shown in **Supplementary Fig. S726**.

Figure S728 (*Heterocephalus glaber*) Root ganglion and Spinal cord Heterocephalus_glaber_male.HetGla_1.0.dna_sm.toplevel.fa

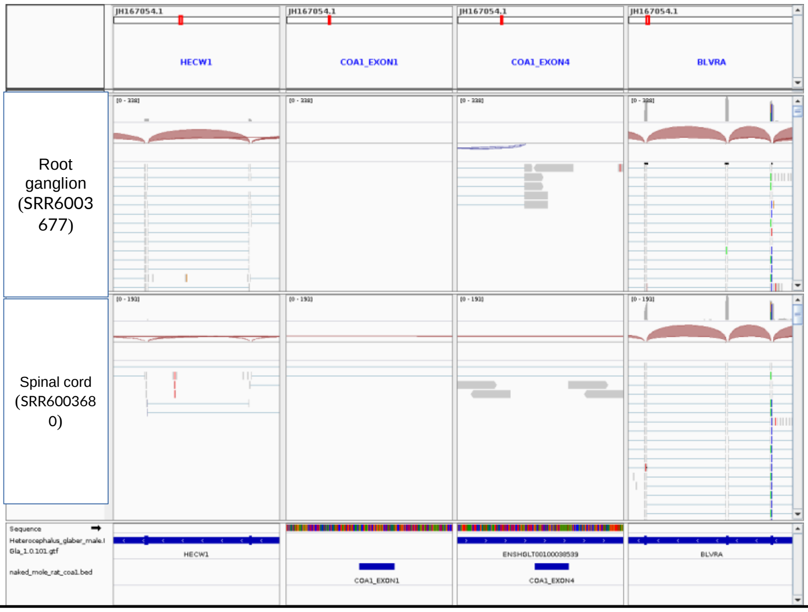

Figure S728: Screenshot of RNA-seq reads aligned to *BLVRA* and *STK17A* to *COA1* exons in naked mole-rat (*Heterocephalus glaber*). Gray boxes represent reads aligned to the genome and thin lines connecting these boxes represent the spliced read alignments. Datasets from root ganglion and spinal cord (SRR6003677 and SRR6003680) respectively have been mapped to the Heterocephalus_glaber_male.HetGla_1.0.dna_sm.toplevel.fa. Locations of each of the exons are depicted as bed record in the bottom row.

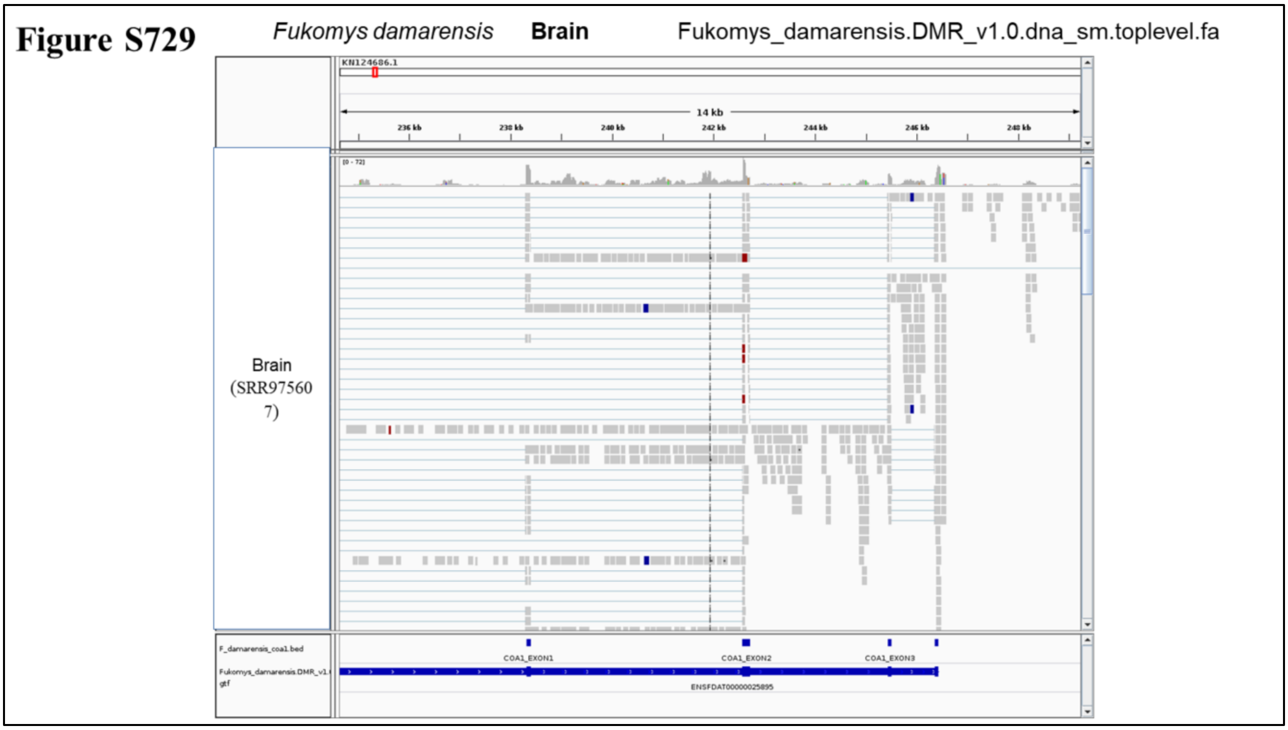

Figure S729: Screenshot of RNA-seq reads aligned to each of the putative locations of the four *COA1* exons in damara mole-rat (*Fukomys damarensis*). Gray boxes represent reads aligned to the genome and thin lines connecting these boxes represent the spliced read alignments. Datasets from brain (SRR975607) have been mapped to the Fukomys_damarensis.DMR_v1.0.dna_sm.toplevel.fa. Locations of each of the exons are depicted as bed record in the bottom row.

Figure S730

Fukomys damarensis **Brain** Fukomys_damarensis.DMR_v1.0.dna_sm.toplevel.fa

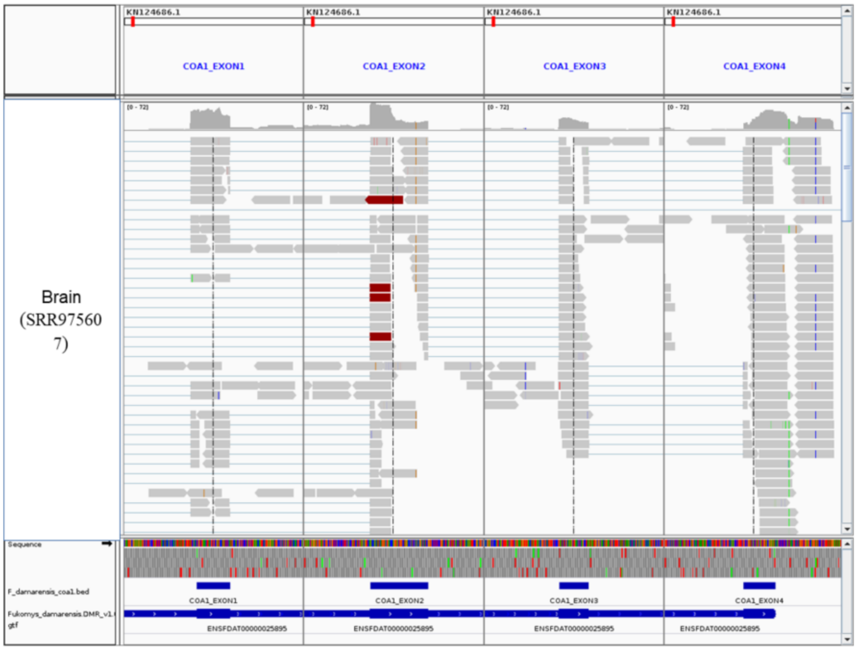

Figure S730: A zoomed in view of each of the *COA1* exons shown in **Supplementary Fig. S729**.

Figure S731 *Fukomys damarensis* **Liver** Fukomys_damarensis.DMR_v1.0.dna_sm.toplevel.fa

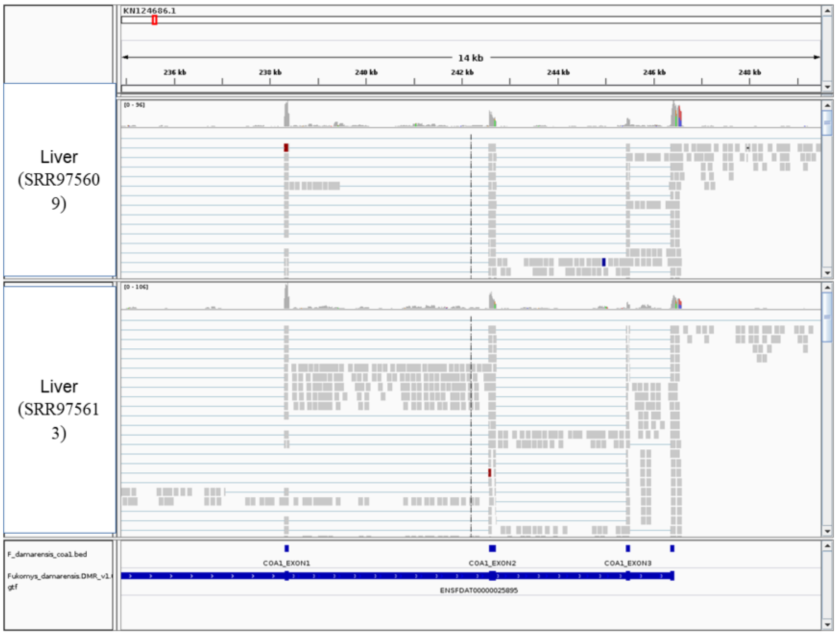

Figure S731: Screenshot of RNA-seq reads aligned to each of the putative locations of the four *COA1* exons in damara mole-rat (*Fukomys damarensis*). Gray boxes represent reads aligned to the genome and thin lines connecting these boxes represent the spliced read alignments. Datasets from liver (SRR975609 and SRR975613)have been mapped to the Fukomys_damarensis.DMR_v1.0.dna_sm.toplevel.fa. Locations of each of the exons are depicted as bed record in the bottom row.

Figure S732 *Fukomys damarensis* **Liver** Fukomys_damarensis.DMR_v1.0.dna_sm.toplevel.fa

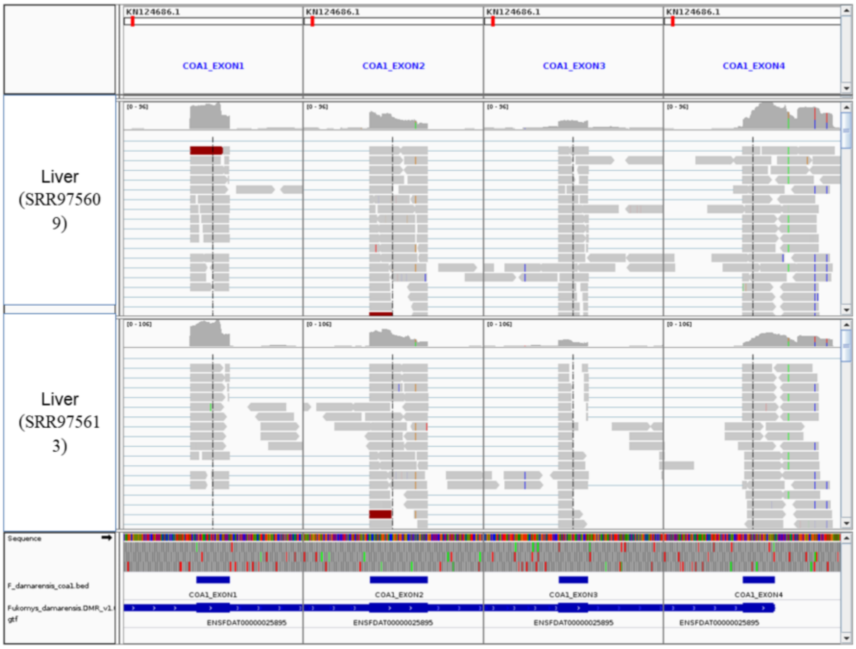

Figure S732: A zoomed in view of each of the *COA1* exons shown in **Supplementary Fig. S731**.

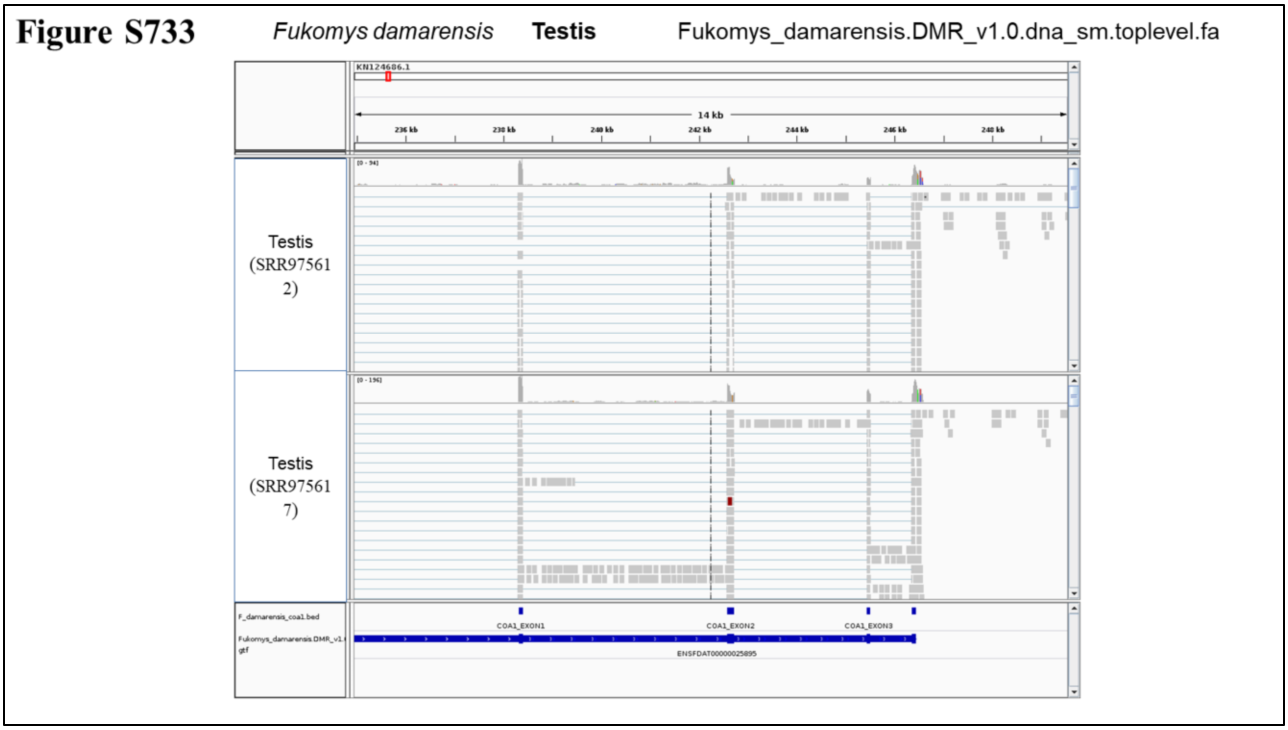

Figure S733: Screenshot of RNA-seq reads aligned to each of the putative locations of the four *COA1* exons in damara mole-rat (*Fukomys damarensis*). Gray boxes represent reads aligned to the genome and thin lines connecting these boxes represent the spliced read alignments. Datasets from testis (SRR975612 and SRR975617) have been mapped to the Fukomys_damarensis.DMR_v1.0.dna_sm.toplevel.fa. Locations of each of the exons are depicted as bed record in the bottom row.

Figure S734

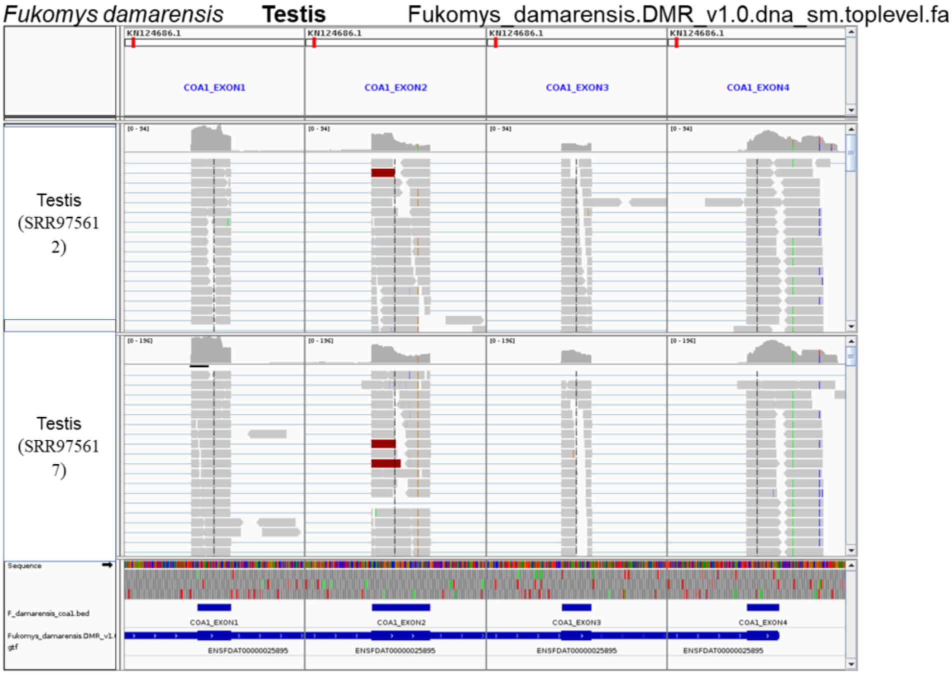

Figure S734: A zoomed in view of each of the *COA1* exons shown in **Supplementary Fig. S733**.

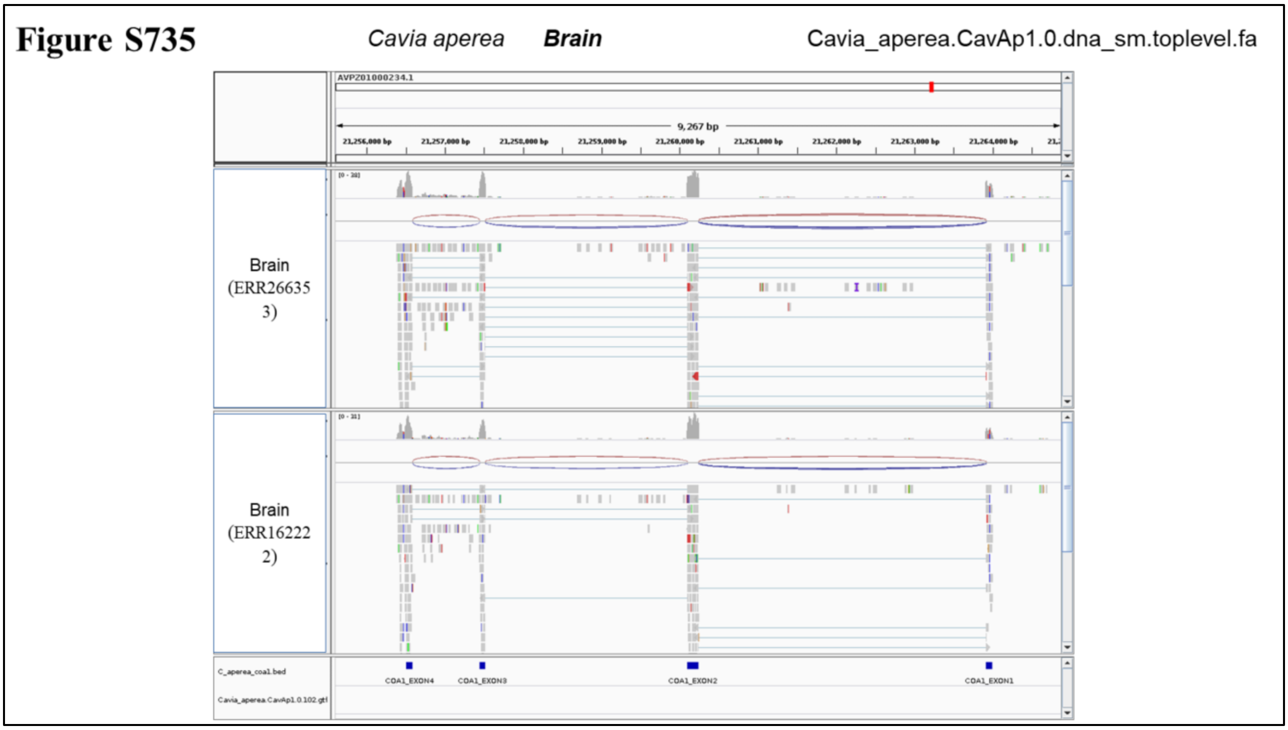

Figure S735: Screenshot of RNA-seq reads aligned to each of the putative locations of the four *COA1* exons in Brazilian guinea pig (*Cavia aperea*). Gray boxes represent reads aligned to the genome and thin lines connecting these boxes represent the spliced read alignments. Datasets from brain (ERR266353 and ERR162222)have been mapped to the Cavia_aperea.CavAp1.0.dna_sm.toplevel.fa. Locations of each of the exons are depicted as bed record in the bottom row.

Figure S736

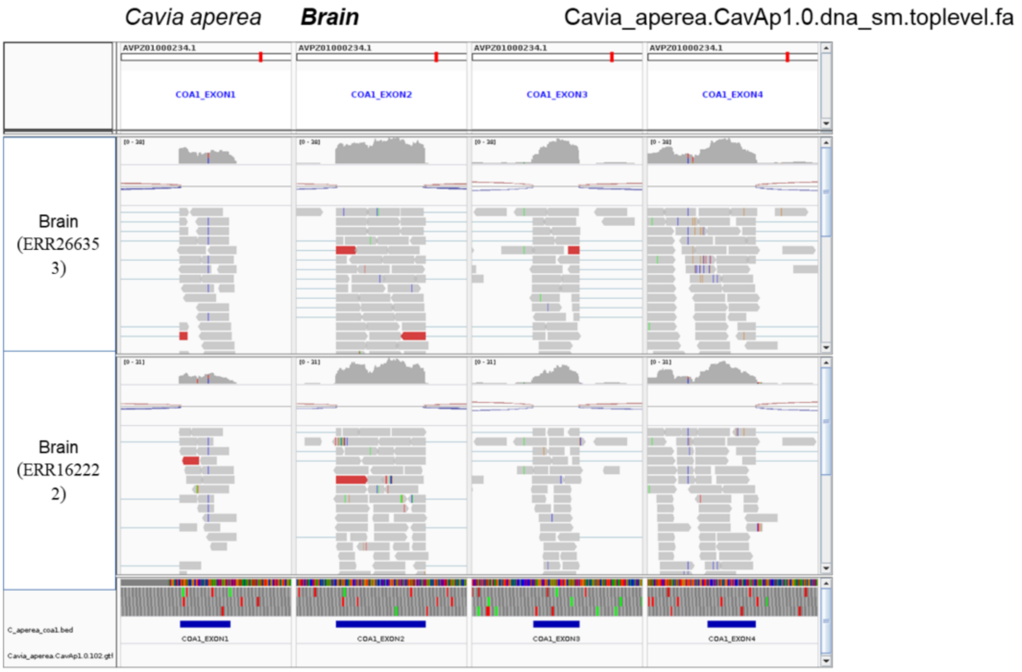

Figure S736: A zoomed in view of each of the *COA1* exons shown in **Supplementary Fig. S735**.

Figure S737

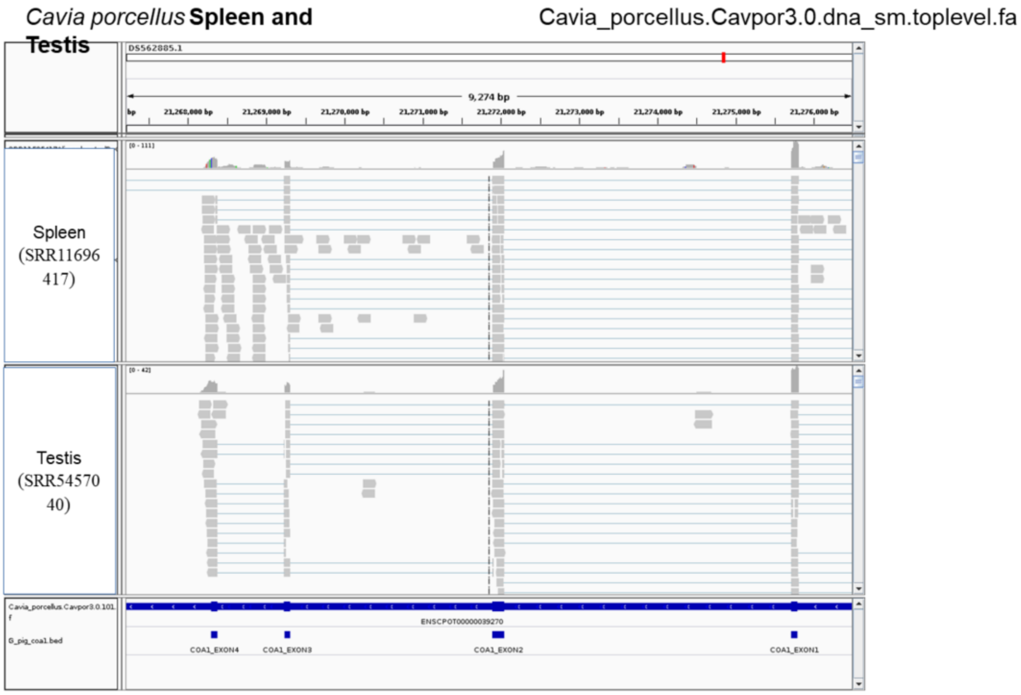

Figure S737: Screenshot of RNA-seq reads aligned to each of the putative locations of the four *COA1* exons in domestic guinea pig (*Cavia porcellus*). Gray boxes represent reads aligned to the genome and thin lines connecting these boxes represent the spliced read alignments. Datasets from spleen (SRR11696417) and testis (SRR5457040) have been mapped to the Cavia_porcellus.Cavpor3.0.dna_sm.toplevel.fa. Locations of each of the exons are depicted as bed record in the bottom row.

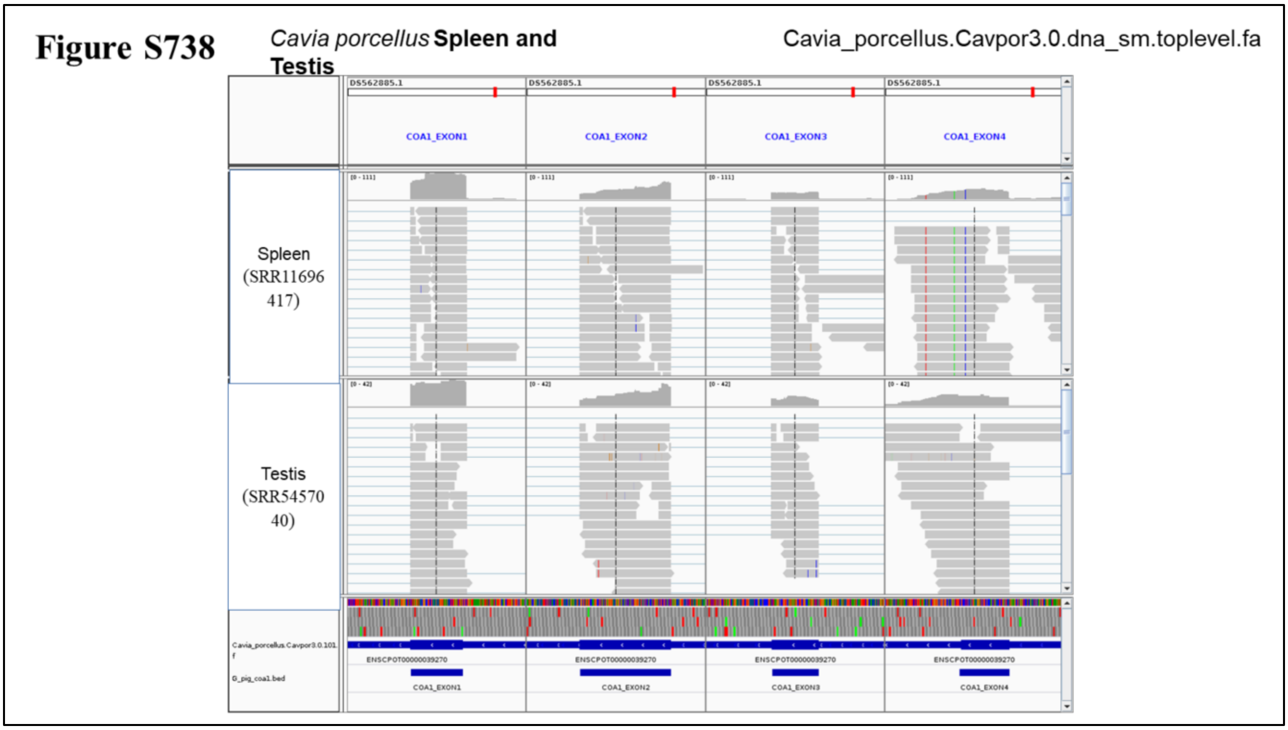

Figure S738: A zoomed in view of each of the *COA1* exons shown in **Supplementary Fig. S737**.

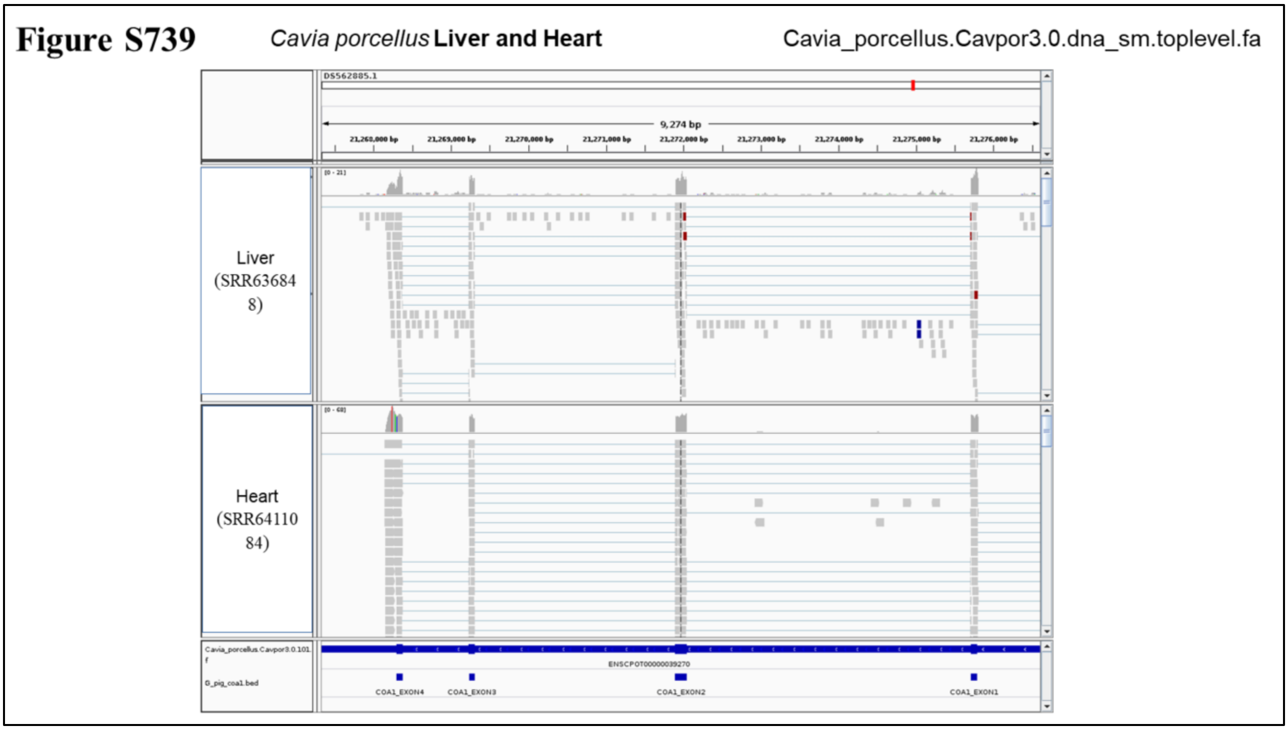

Figure S739: Screenshot of RNA-seq reads aligned to each of the putative locations of the four *COA1* exons in domestic guinea pig (*Cavia porcellus*). Gray boxes represent reads aligned to the genome and thin lines connecting these boxes represent the spliced read alignments. Datasets from liver (SRR636848) and heart (SRR6411084) have been mapped to the Cavia_porcellus.Cavpor3.0.dna_sm.toplevel.fa. Locations of each of the exons are depicted as bed record in the bottom row.

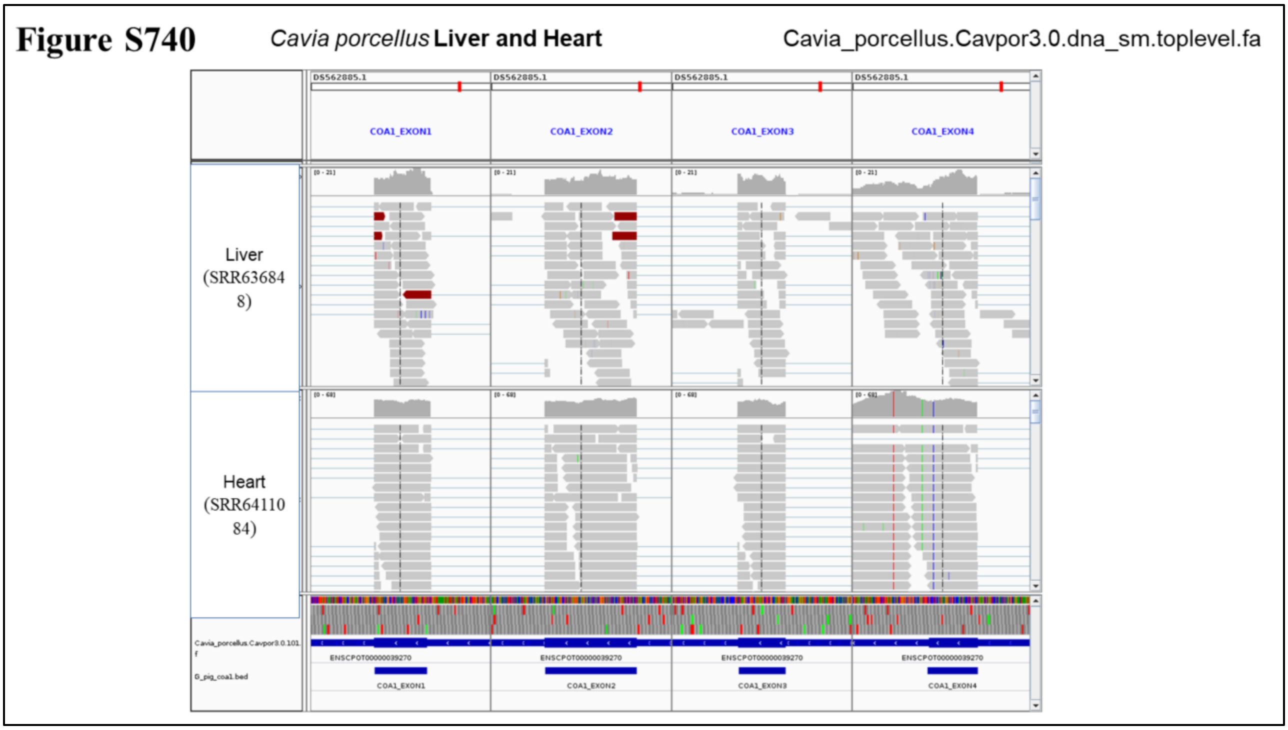

Figure S740: A zoomed in view of each of the *COA1* exons shown in **Supplementary Fig. S739**.

Figure S741 *Chinchilla lanigera* **Brain and Liver** Chinchilla_lanigera.ChiLan1.0.dna_sm.toplevel.fa

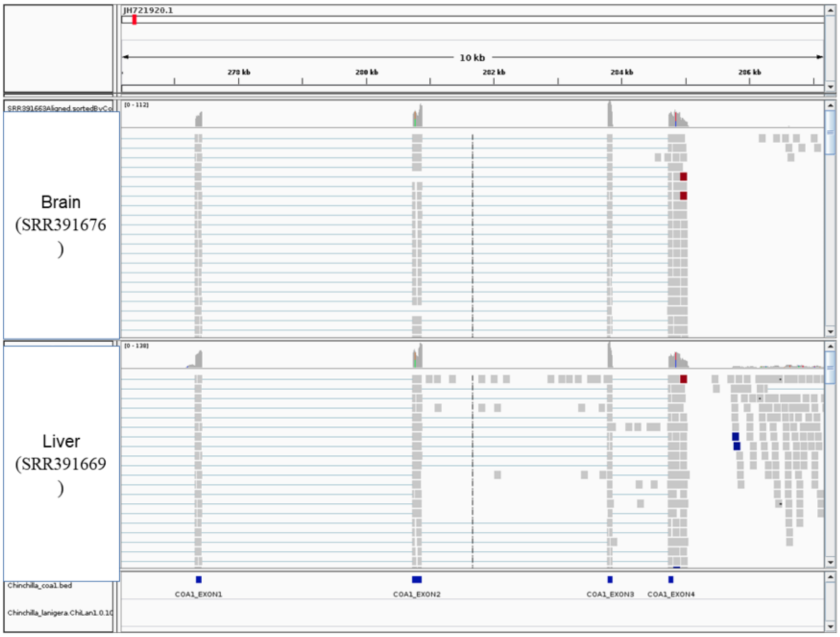

Figure S741: Screenshot of RNA-seq reads aligned to each of the putative locations of the four *COA1* exons in long tailed chinchilla (*Chinchilla lanigera*). Gray boxes represent reads aligned to the genome and thin lines connecting these boxes represent the spliced read alignments. Datasets from brain (SRR391676) and liver (SRR391669) have been mapped to the Chinchilla_lanigera.ChiLan1.0.dna_sm.toplevel.fa. Locations of each of the exons are depicted as bed record in the bottom row.

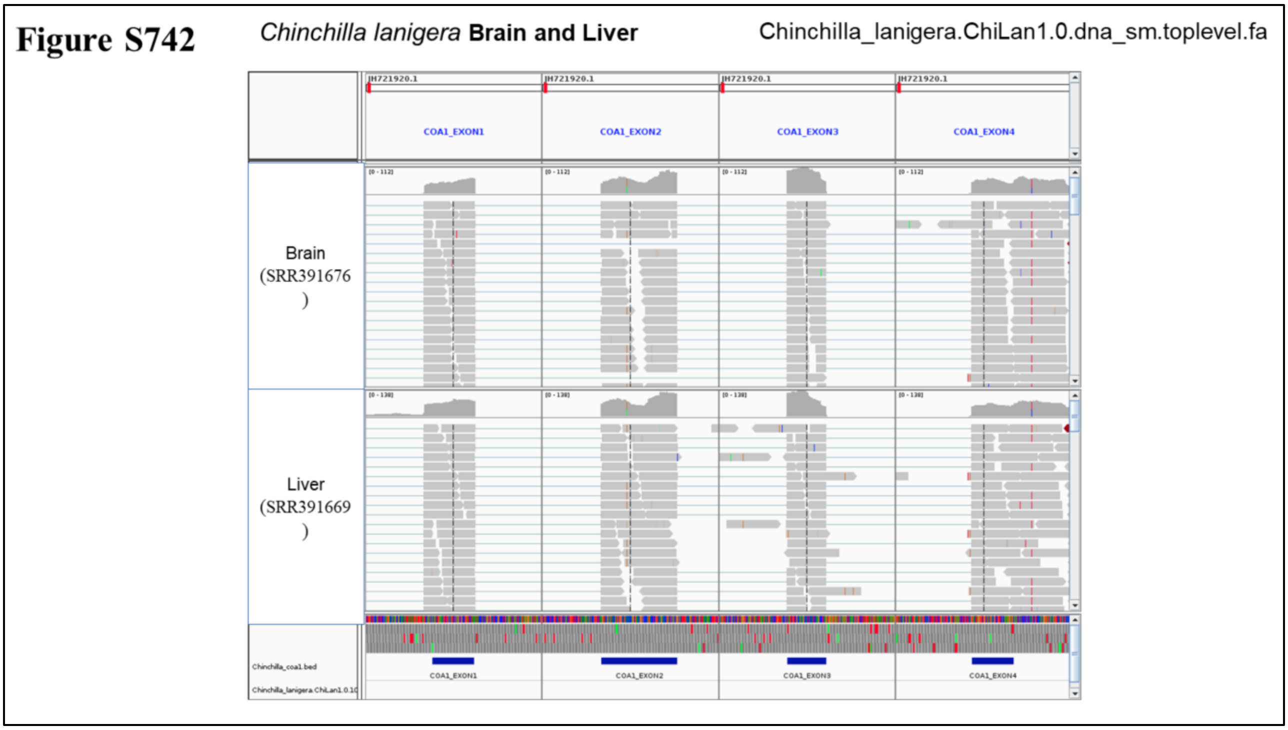

Figure S742: A zoomed in view of each of the *COA1* exons shown in **Supplementary Fig. S741**.

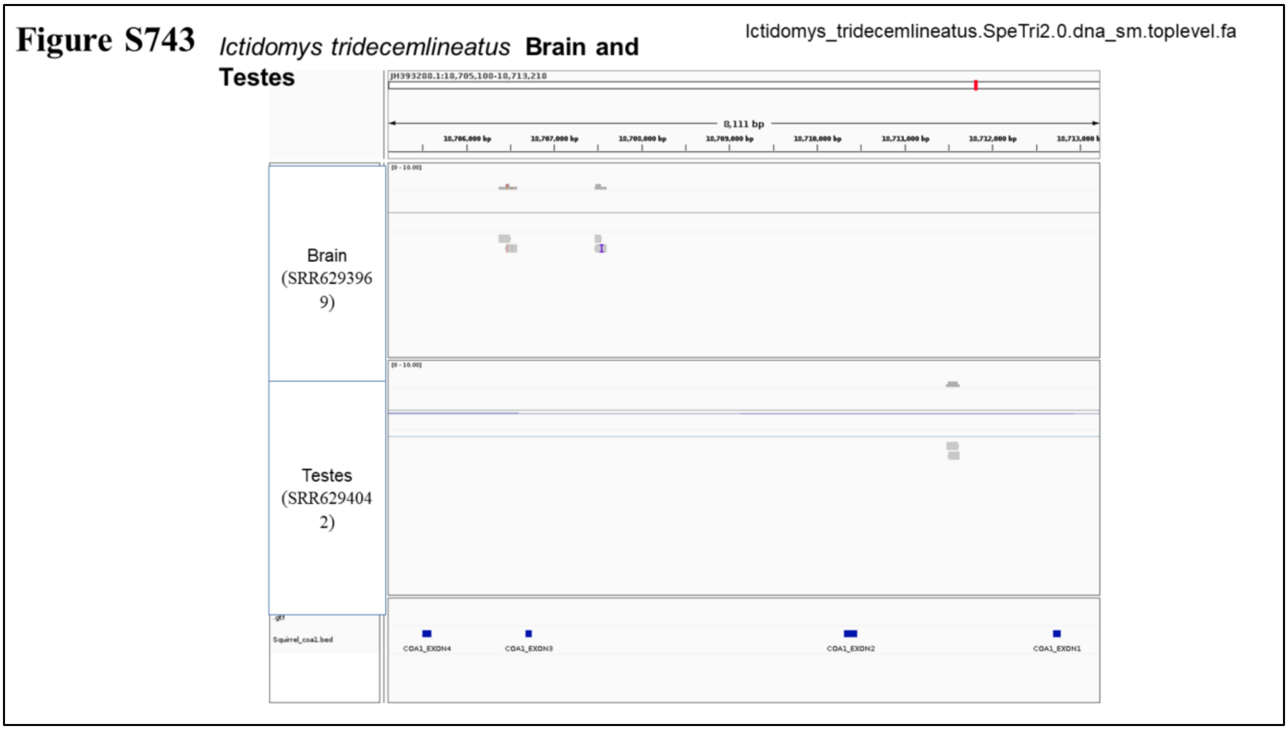

Figure S743: Screenshot of RNA-seq reads aligned to each of the putative locations of the four *COA1* exons in thirteen-lined ground squirrel (*Ictidomys tridecemlineatus*). Gray boxes represent reads aligned to the genome and thin lines connecting these boxes represent the spliced read alignments. Datasets from brain and testes (SRR6293969 and SRR6294042) have been mapped to the Ictidomys_tridecemlineatus.SpeTri2.0.dna_sm.toplevel.fa. Locations of each of the exons are depicted as bed record in the bottom row.

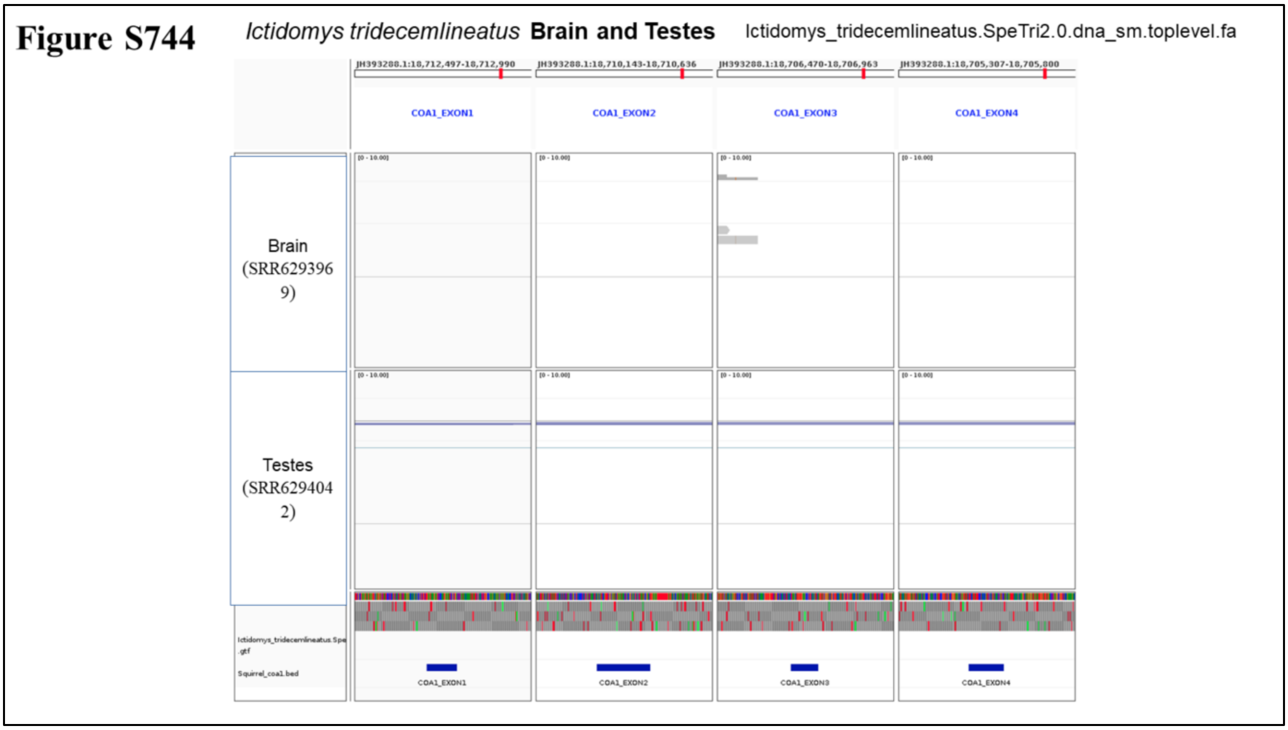

Figure S744: A zoomed in view of each of the *COA1* exons shown in **Supplementary Fig. S743**.

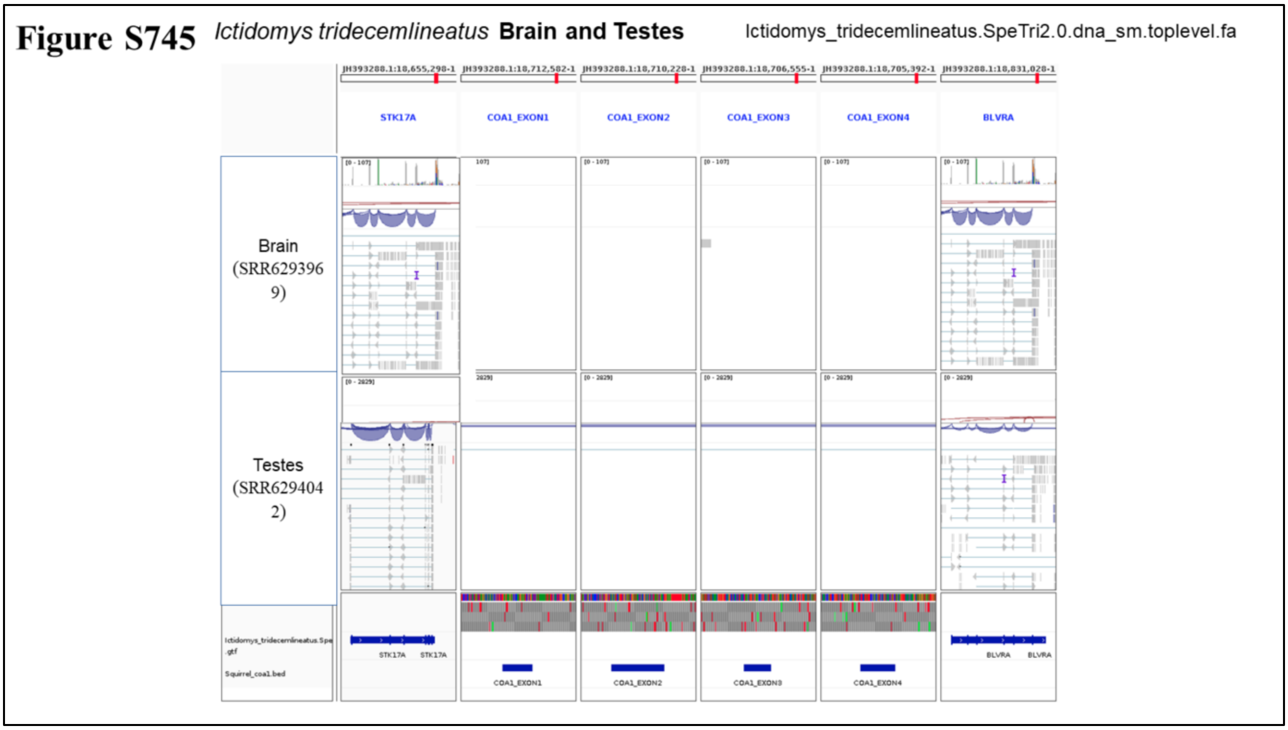

Figure S745: Screenshot of RNA-seq reads aligned to *BLVRA* and *STK17A* to *COA1* exon 1 and 4 respectively in thirteen-lined ground squirrel (*Ictidomys tridecemlineatus*). Gray boxes represent reads aligned to the genome and thin lines connecting these boxes represent the spliced read alignments. Datasets from brain and testes (SRR6293969 and SRR6294042) have been mapped to the Ictidomys_tridecemlineatus.SpeTri2.0.dna_sm.toplevel.fa. Locations of each of the exons are depicted as bed record in the bottom row.

Figure S746 *Ictidomys tridecemlineatus* **Eye** Ictidomys_tridecemlineatus.SpeTri2.0.dna_sm.toplevel.fa

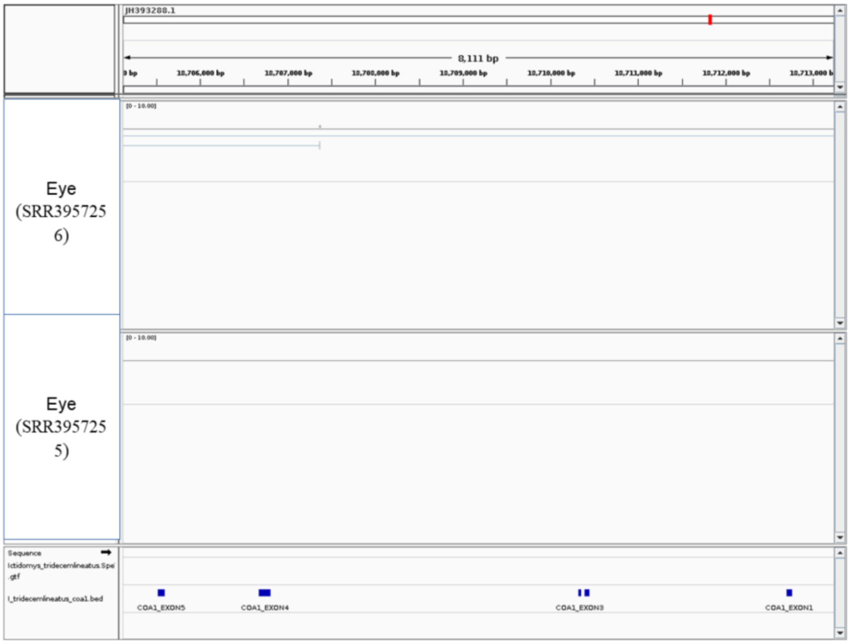

Figure S746: Screenshot of RNA-seq reads aligned to each of the putative locations of the four *COA1* exons in thirteen-lined ground squirrel(*Ictidomys tridecemlineatus*). Gray boxes represent reads aligned to the genome and thin lines connecting these boxes represent the spliced read alignments. Datasets from eye (SRR3957255 and SRR3957256) have been mapped to the Ictidomys_tridecemlineatus.SpeTri2.0.dna_sm.toplevel.fa. Locations of each of the exons are depicted as bed record in the bottom row.

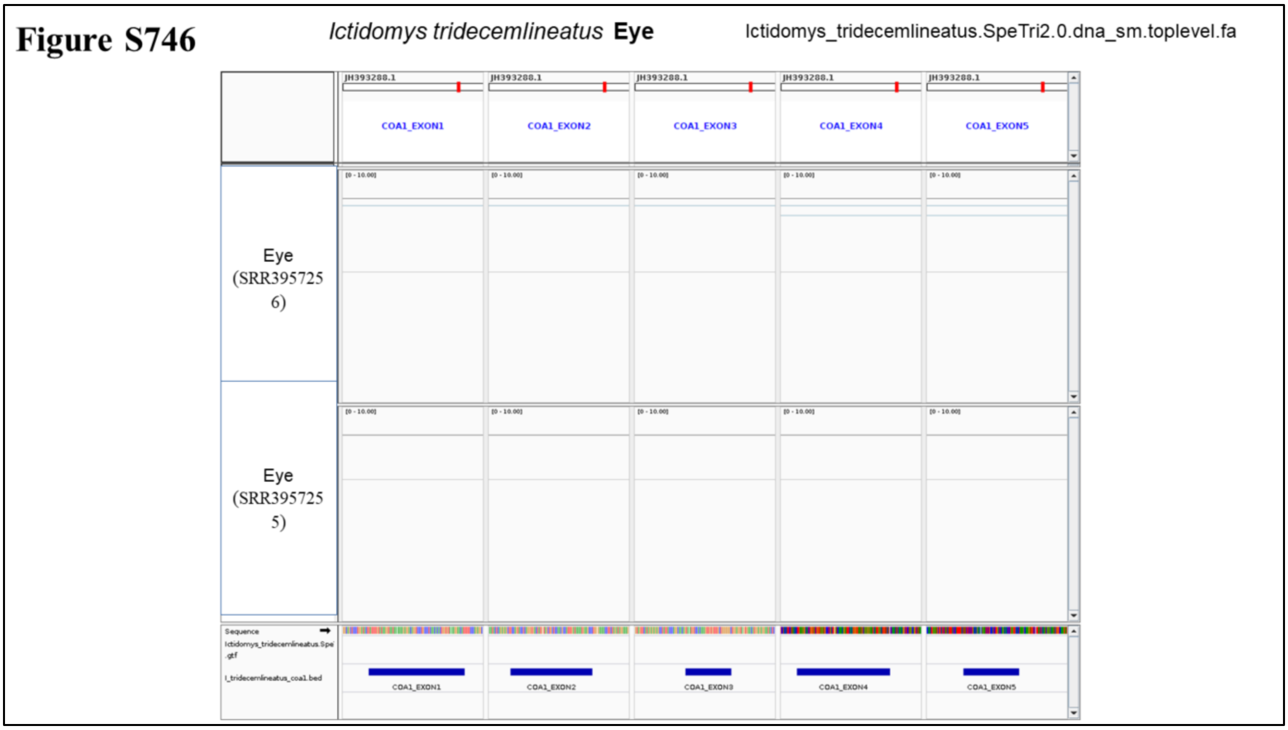

Figure S747: A zoomed in view of each of the *COA1* exons shown in **Supplementary Fig. S745**.

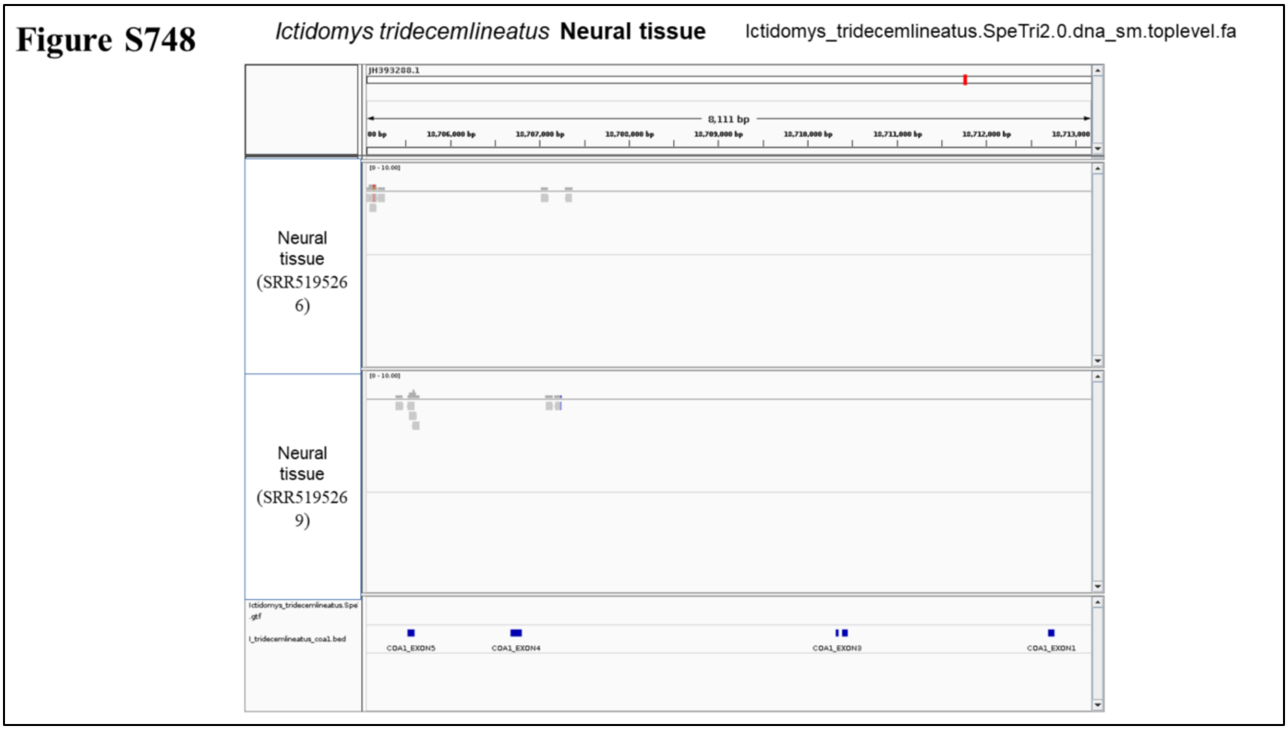

Figure S748: Screenshot of RNA-seq reads aligned to each of the putative locations of the five *COA1* exons in thirteen-lined ground squirrel (*Ictidomys tridecemlineatus*). Gray boxes represent reads aligned to the genome and thin lines connecting these boxes represent the spliced read alignments. Datasets from neural tissue (SRR5195266 and SRR5195269) have been mapped to the Ictidomys_tridecemlineatus.SpeTri2.0.dna_sm.toplevel.fa. Locations of each of the exons are depicted as bed record in the bottom row.

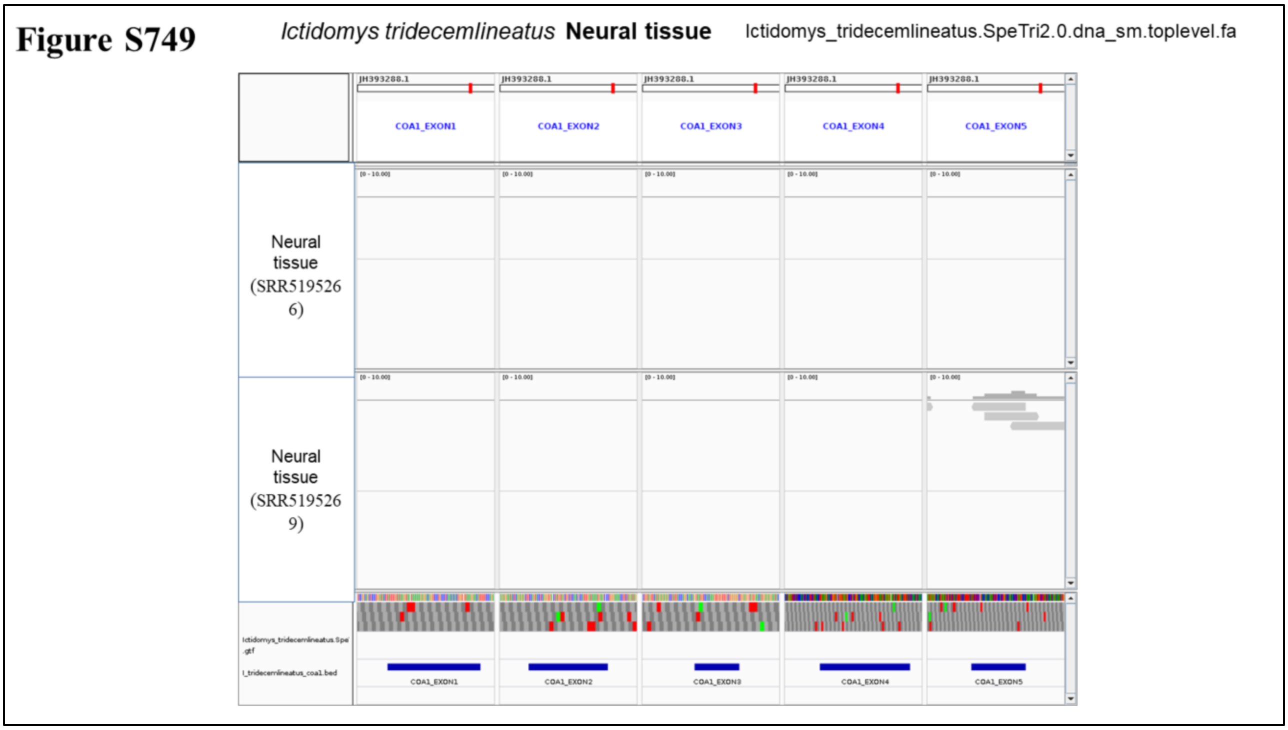

Figure S749: A zoomed in view of each of the *COA1* exons shown in **Supplementary Fig. S748**.

Figure S750 *Urocitellus parryii* **Skeletal muscle** Urocitellus_parryii.ASM342692v1.dna_sm.toplevel.fa

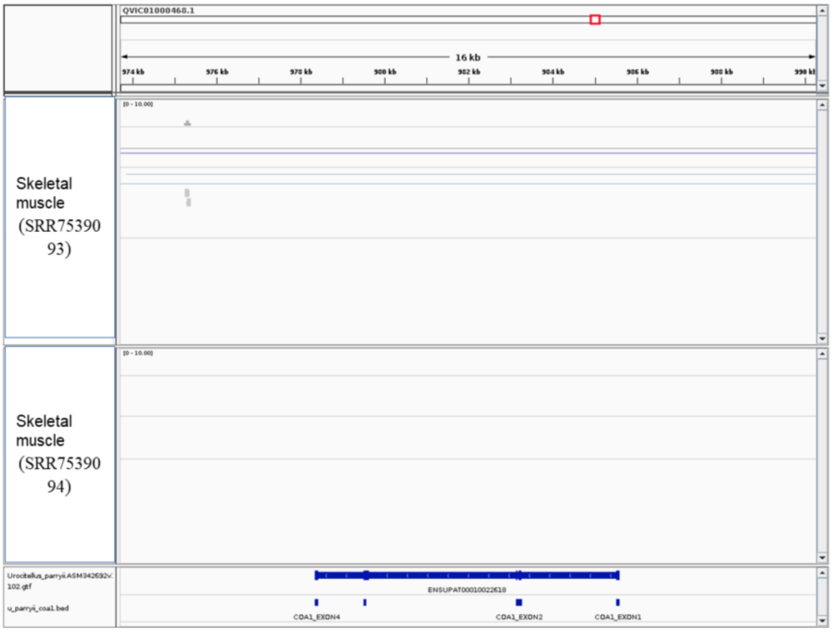

Figure S750: Screenshot of RNA-seq reads aligned to each of the putative locations of the four *COA1* exons in Arctic ground squirrel (*Urocitellus parryii*). Gray boxes represent reads aligned to the genome and thin lines connecting these boxes represent the spliced read alignments. Datasets from skeletal muscle (SRR7539093 and SRR7539094) have been mapped to the Urocitellus_parryii.ASM342692v1.dna_sm.toplevel.fa. Locations of each of the exons are depicted as bed record in the bottom row.

Figure S751 *Urocitellus parryii* **Skeletal muscle** Urocitellus_parryii.ASM342692v1.dna_sm.toplevel.fa

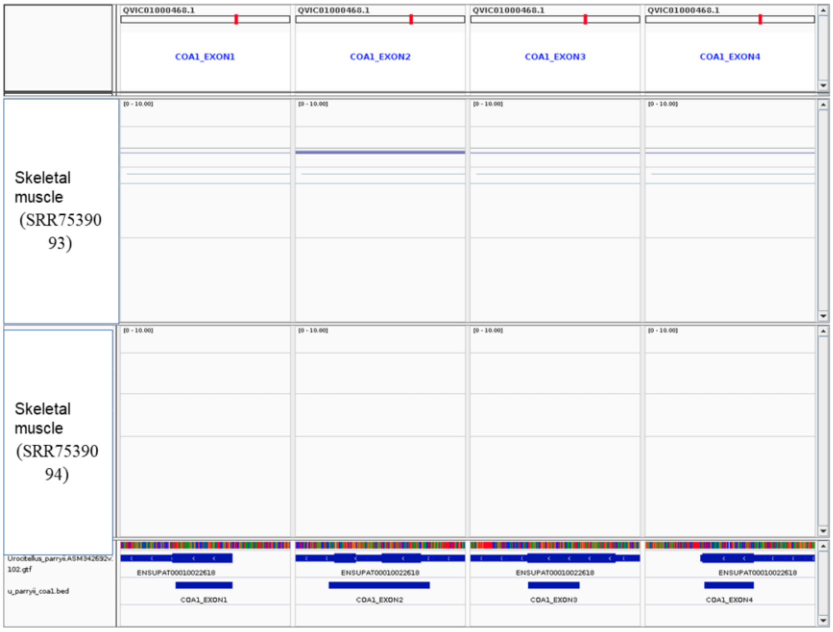

Figure S751: A zoomed in view of each of the *COA1* exons shown in **Supplementary Fig. S750**.

Figure S752 *Urocitellus parryii* **Skeletal muscle** Urocitellus_parryii.ASM342692v1.dna_sm.toplevel.fa

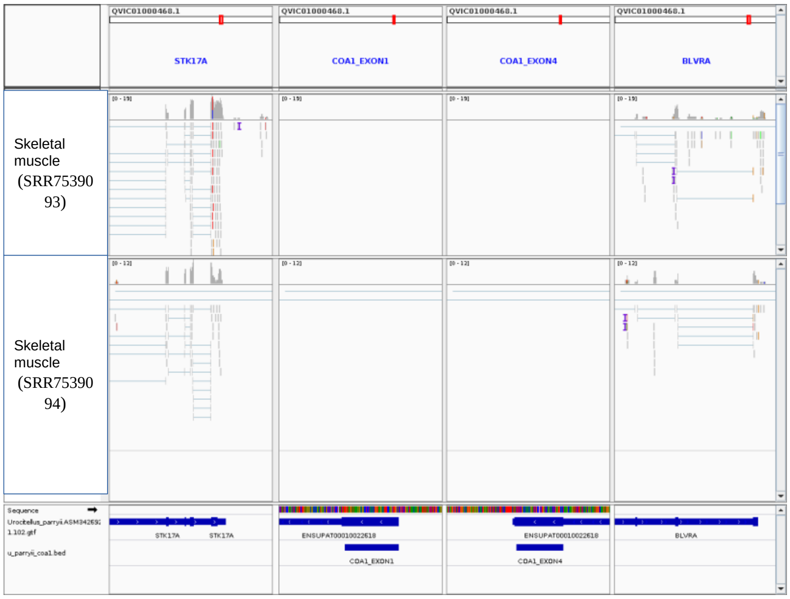

Figure S752: Screenshot of RNA-seq reads aligned to each of the putative locations of the four *COA1* exon-wise with nearby gene region in Arctic ground squirrel (*Urocitellus parryii*). Gray boxes represent reads aligned to the genome and thin lines connecting these boxes represent the spliced read alignments. Datasets from skeletal muscle (SRR7539093 and SRR7539094) have been mapped to the Urocitellus_parryii.ASM342692v1.dna_sm.toplevel.fa. Locations of each of the exons are depicted as bed record in the bottom row.

Figure S753 *Urocitellus parryii* **Skeletal muscle** Urocitellus_parryii.ASM342692v1.dna_sm.toplevel.fa

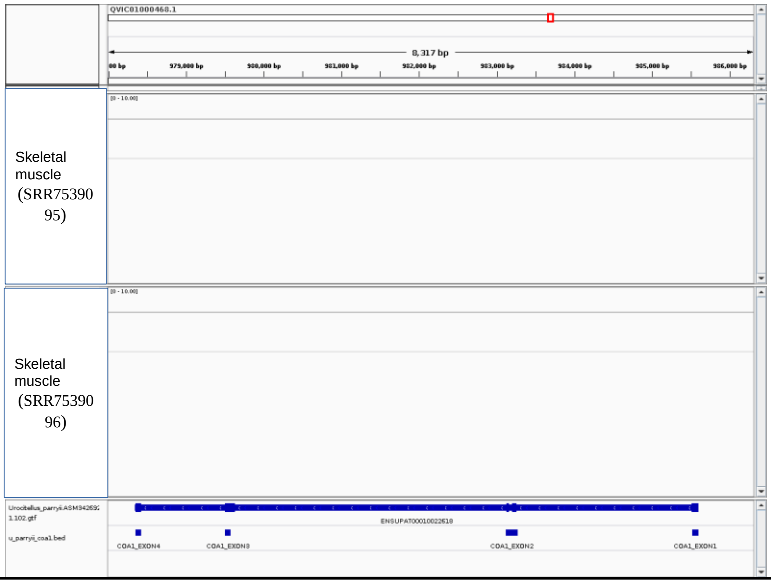

Figure S753: Screenshot of RNA-seq reads aligned to each of the putative locations of the four *COA1* exons in Arctic ground squirrel (*Urocitellus parryii*). Gray boxes represent reads aligned to the genome and thin lines connecting these boxes represent the spliced read alignments. Datasets from skeletal muscle (SRR7539095 and SRR7539096) have been mapped to the Urocitellus_parryii.ASM342692v1.dna_sm.toplevel.fa. Locations of each of the exons are depicted as bed record in the bottom row.

Figure S754 *Urocitellus parryii* **Skeletal muscle** Urocitellus_parryii.ASM342692v1.dna_sm.toplevel.fa

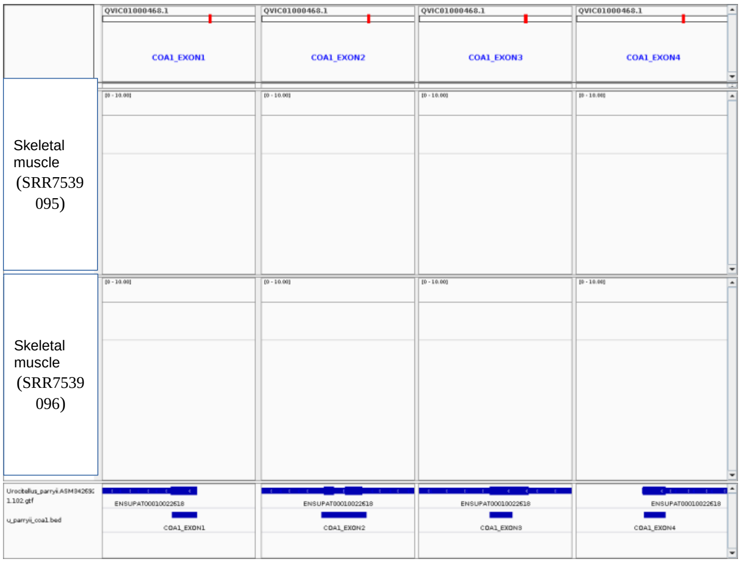

Figure S754: A zoomed in view of each of the *COA1* exons shown in **Supplementary Fig. S753**.

Figure S755 *Urocitellus parryii* **Skeletal muscle** Urocitellus_parryii.ASM342692v1.dna_sm.toplevel.fa

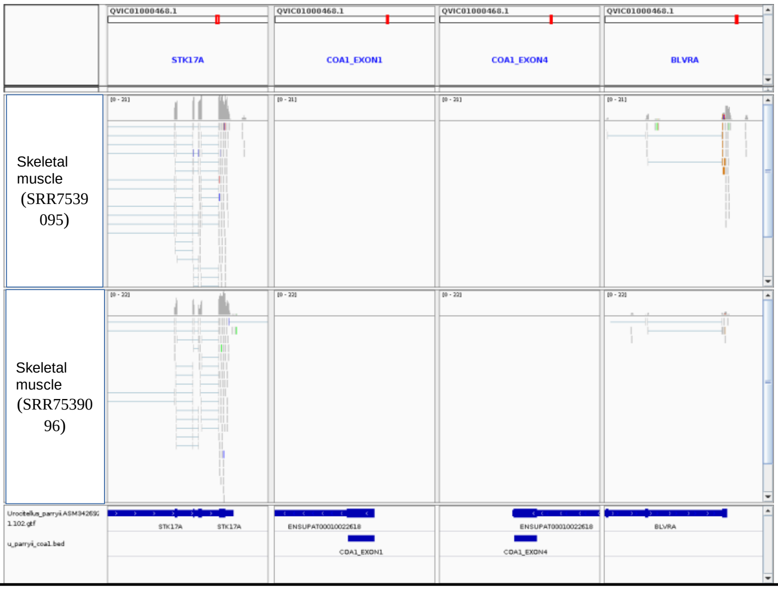

Figure S755: Screenshot of RNA-seq reads aligned to each of the putative locations of the four *COA1* exons with nearby gene region in Arctic ground squirrel (*Urocitellus parryii*). Gray boxes represent reads aligned to the genome and thin lines connecting these boxes represent the spliced read alignments. Datasets from skeletal muscle (SRR7539095 and SRR7539096) have been mapped to the Urocitellus_parryii.ASM342692v1.dna_sm.toplevel.fa. Locations of each of the exons are depicted as bed record in the bottom row.

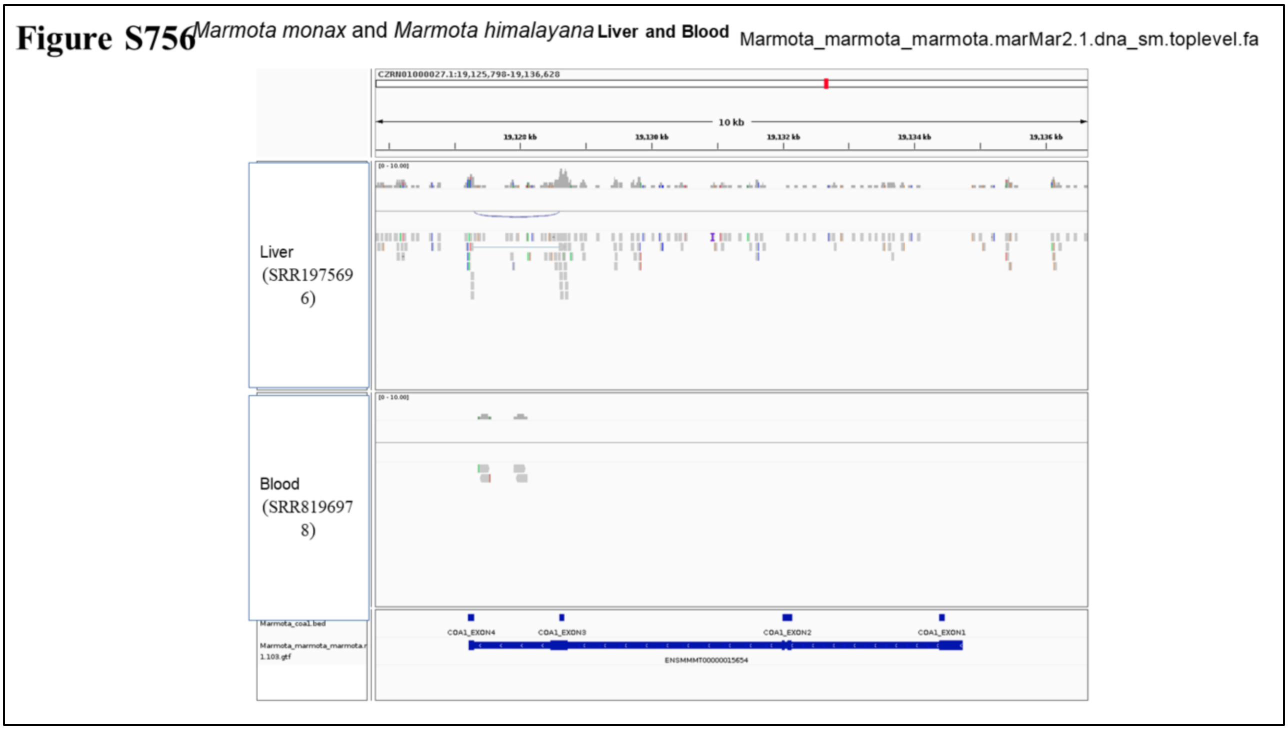

Figure S756: Screenshot of RNA-seq reads aligned to each of the putative locations of the four *COA1* exons in groundhog (*Marmota monax*) and Himalayan marmot(*Marmota himalayana*). Gray boxes represent reads aligned to the genome and thin lines connecting these boxes represent the spliced read alignments. Datasets from liver and blood (SRR1975696 and SRR8196978) have been mapped to the Marmota_marmota_marmota.marMar2.1.dna_sm.toplevel.fa. Locations of each of the exons are depicted as bed record in the bottom row.

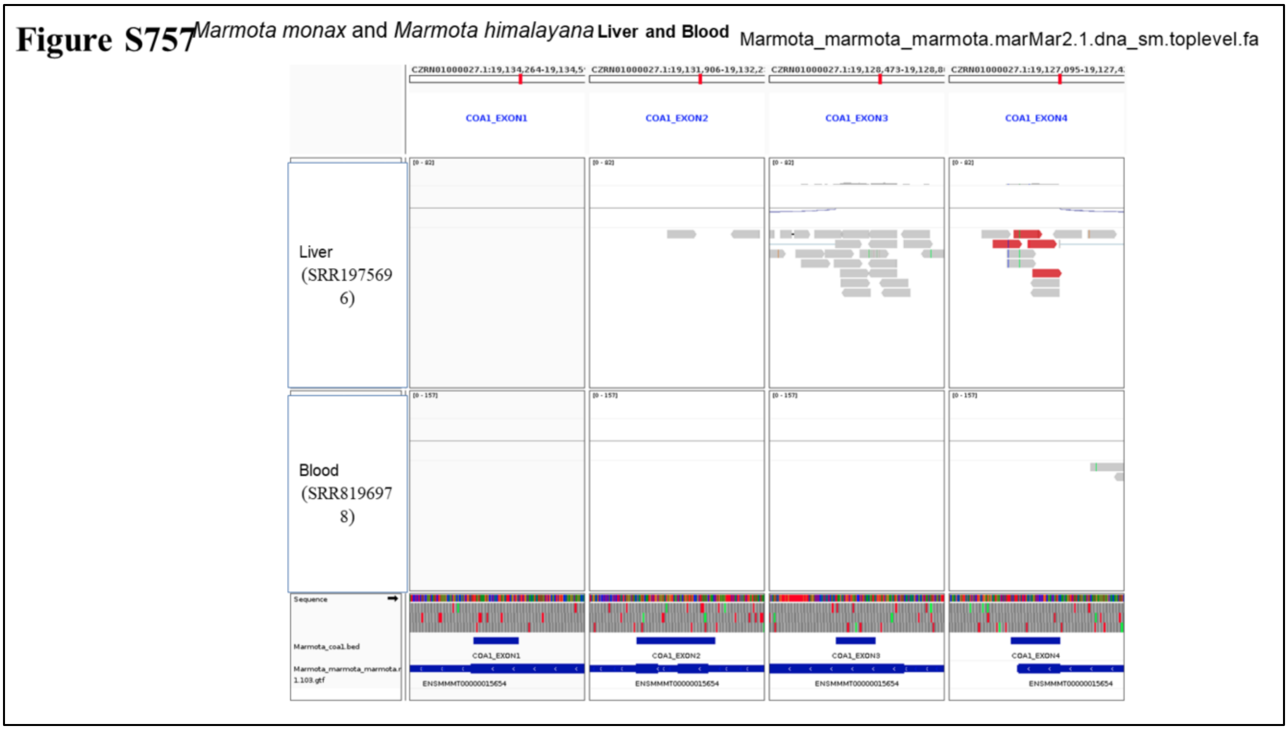

Figure S757: A zoomed in view of each of the *COA1* exons shown in **Supplementary Fig. S756**.

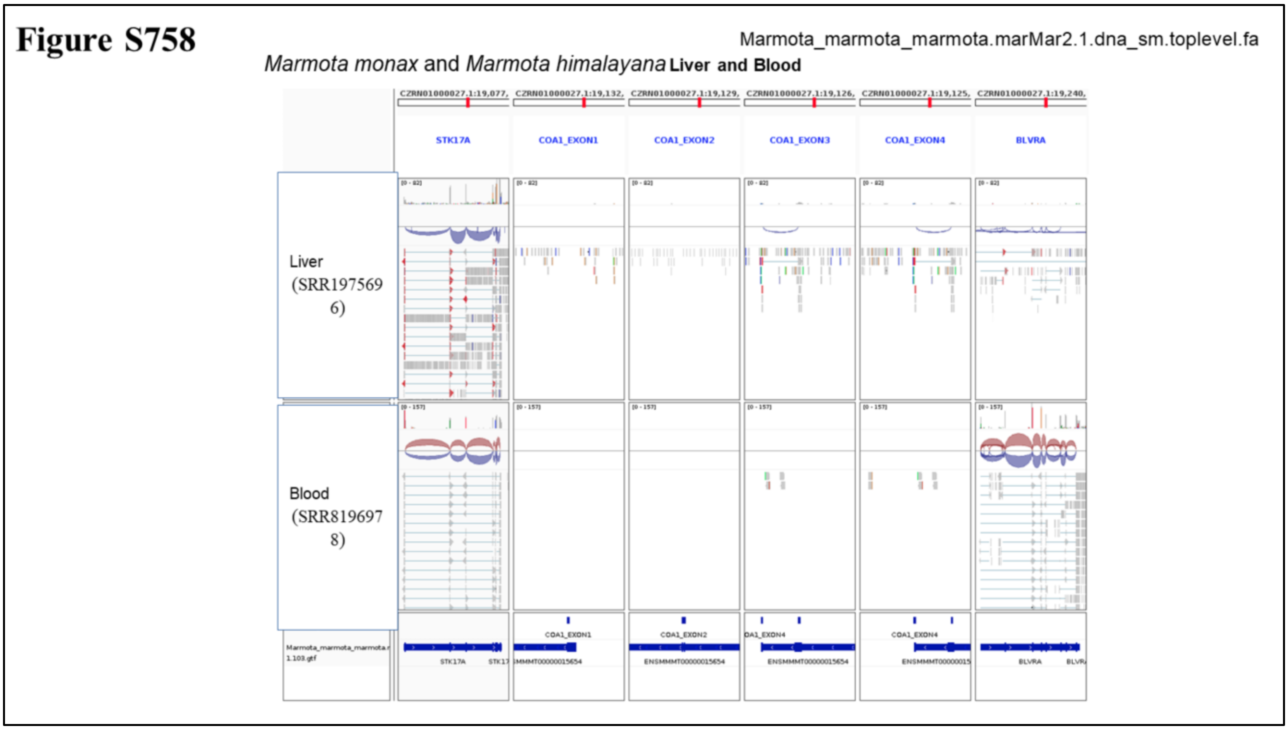

Figure S758: Screenshot of RNA-seq reads aligned to *BLVRA* and *STK17A* to *COA1* exon 1 and 4 respectively in groundhog (*Marmota monax*) and Himalayan marmot(*Marmota himalayana*). Gray boxes represent reads aligned to the genome and thin lines connecting these boxes represent the spliced read alignments. Datasets from liver and blood (SRR1975696 and SRR8196978) have been mapped to the Marmota_marmota_marmota.marMar2.1.dna_sm.toplevel.fa. Locations of each of the exons are depicted as bed record in the bottom row.

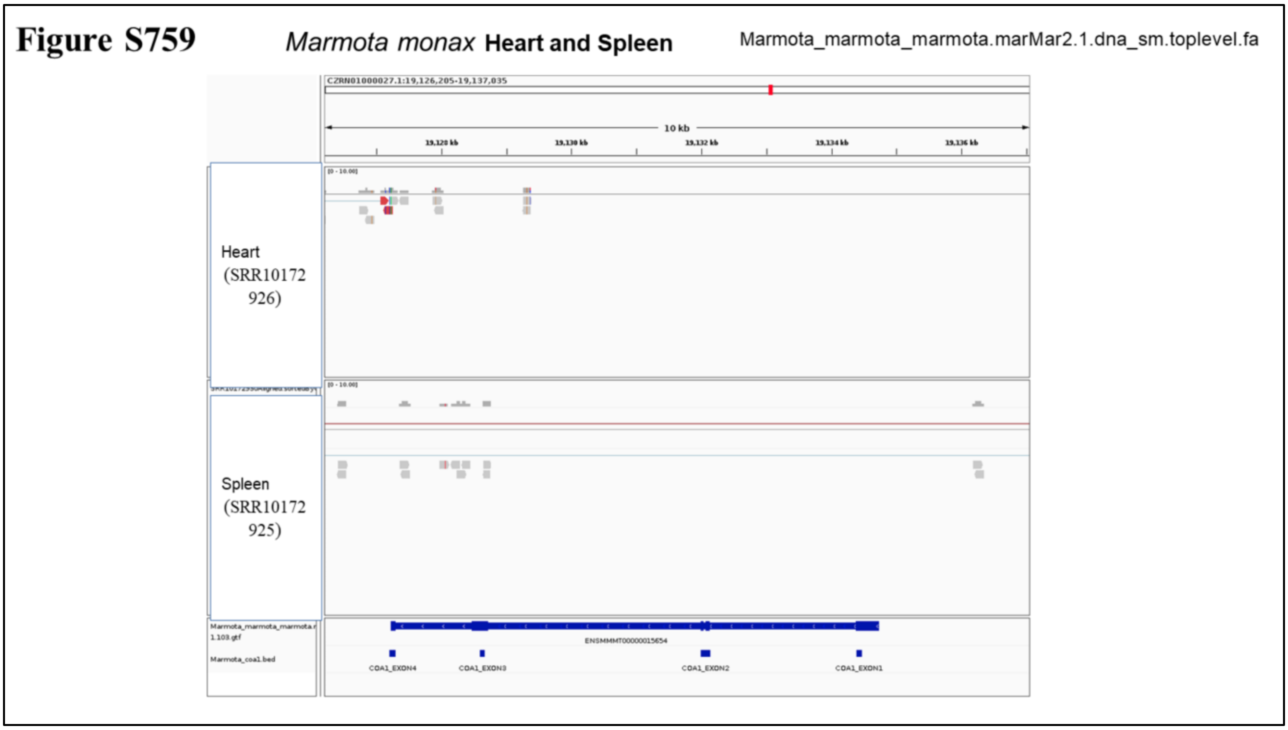

Figure S759: Screenshot of RNA-seq reads aligned to each of the putative locations of the four *COA1* exons in groundhog (*Marmota monax*). Gray boxes represent reads aligned to the genome and thin lines connecting these boxes represent the spliced read alignments. Datasets from heart and spleen (SRR10172926 and SRR10172925) have been mapped to the Marmota_marmota_marmota.marMar2.1.dna_sm.toplevel.fa. Locations of each of the exons are depicted as bed record in the bottom row.

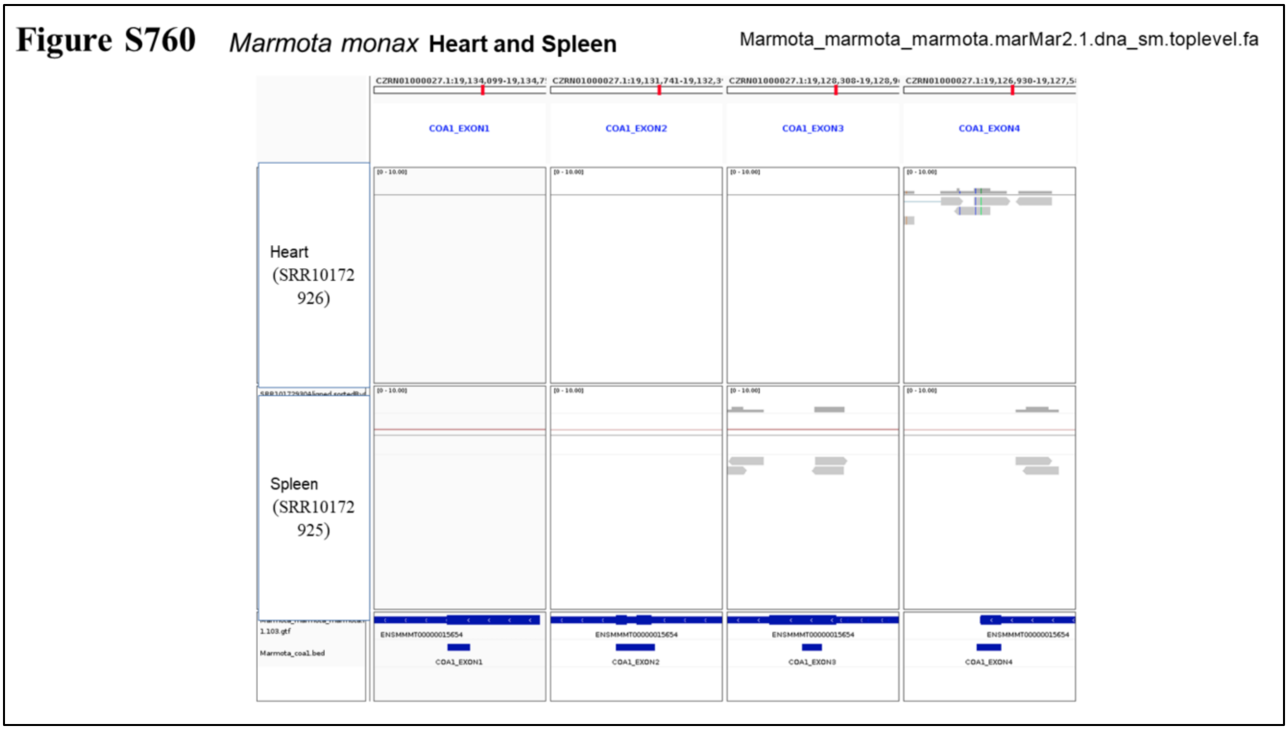

Figure S760: A zoomed in view of each of the *COA1* exons shown in **Supplementary Fig. S759**.

Figure S761 *Marmota monax* **Heart and Spleen** Marmota_marmota_marmota.marMar2.1.dna_sm.toplevel.fa

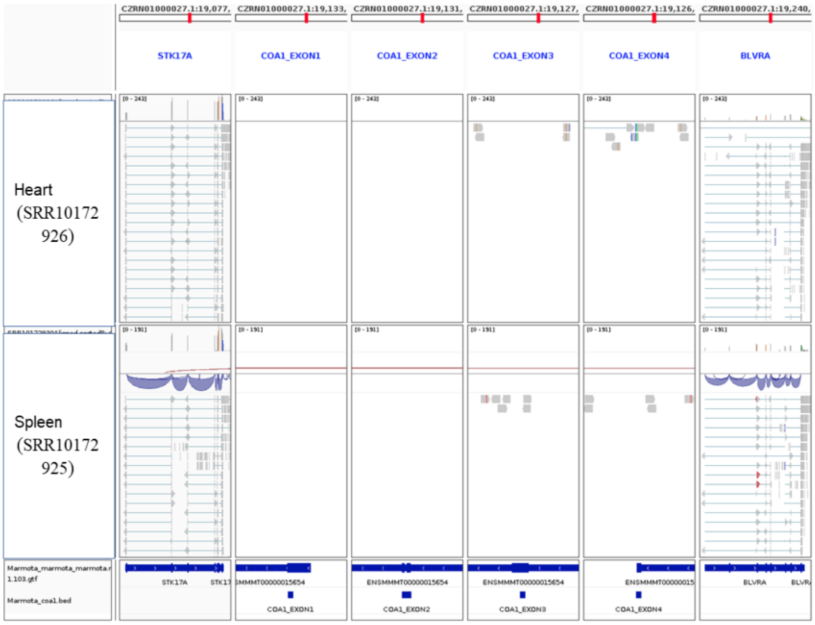

Figure S761: Screenshot of RNA-seq reads aligned to *BLVRA* and *STK17A* to *COA1* exon 1 and 4 respectively in groundhog (*Marmota monax*). Gray boxes represent reads aligned to the genome and thin lines connecting these boxes represent the spliced read alignments. Datasets from heart and spleen (SRR10172926 and SRR10172925) have been mapped to the Marmota_marmota_marmota.marMar2.1.dna_sm.toplevel.fa. Locations of each of the exons are depicted as bed record in the bottom row.

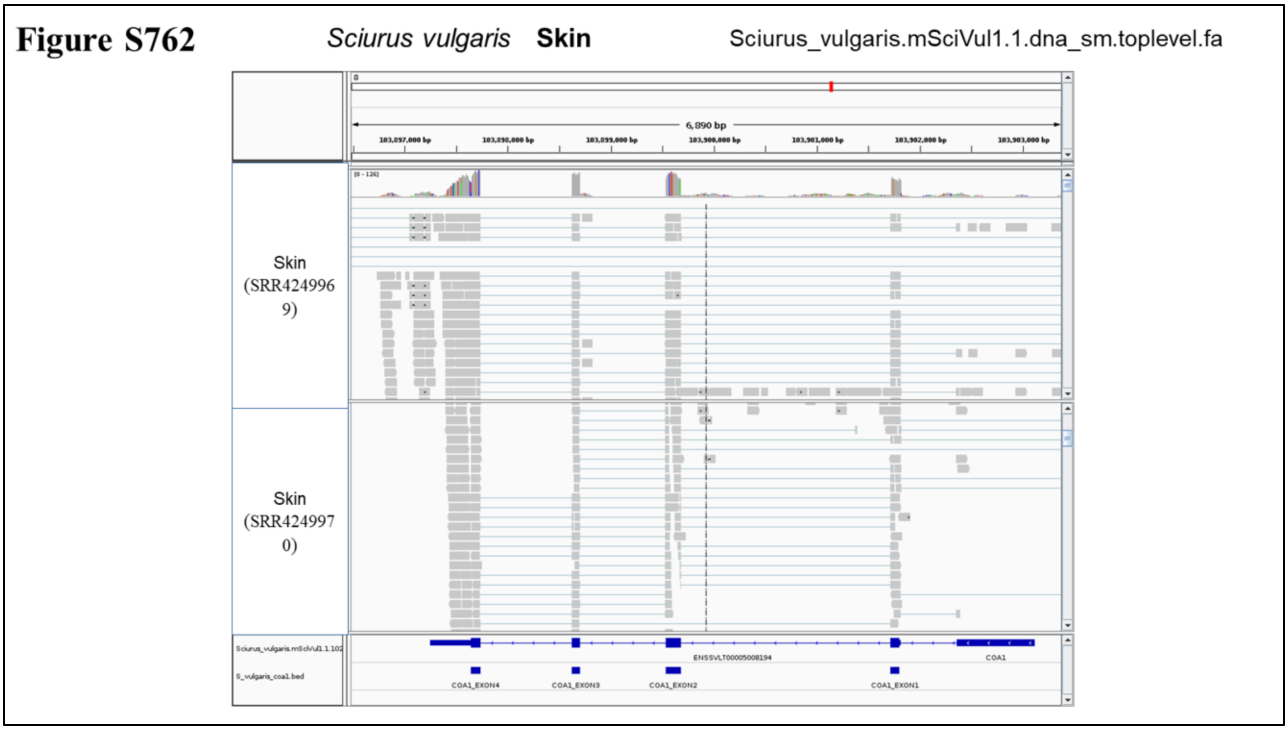

Figure S762: Screenshot of RNA-seq reads aligned to each of the putative locations of the four *COA1* exons in Eurasian red squirrel (*Sciurus vulgaris*). Gray boxes represent reads aligned to the genome and thin lines connecting these boxes represent the spliced read alignments. Datasets from skin (SRR4249969 and SRR4249970) have been mapped to the Sciurus_vulgaris.mSciVul1.1.dna_sm.toplevel.fa. Locations of each of the exons are depicted as bed record in the bottom row.

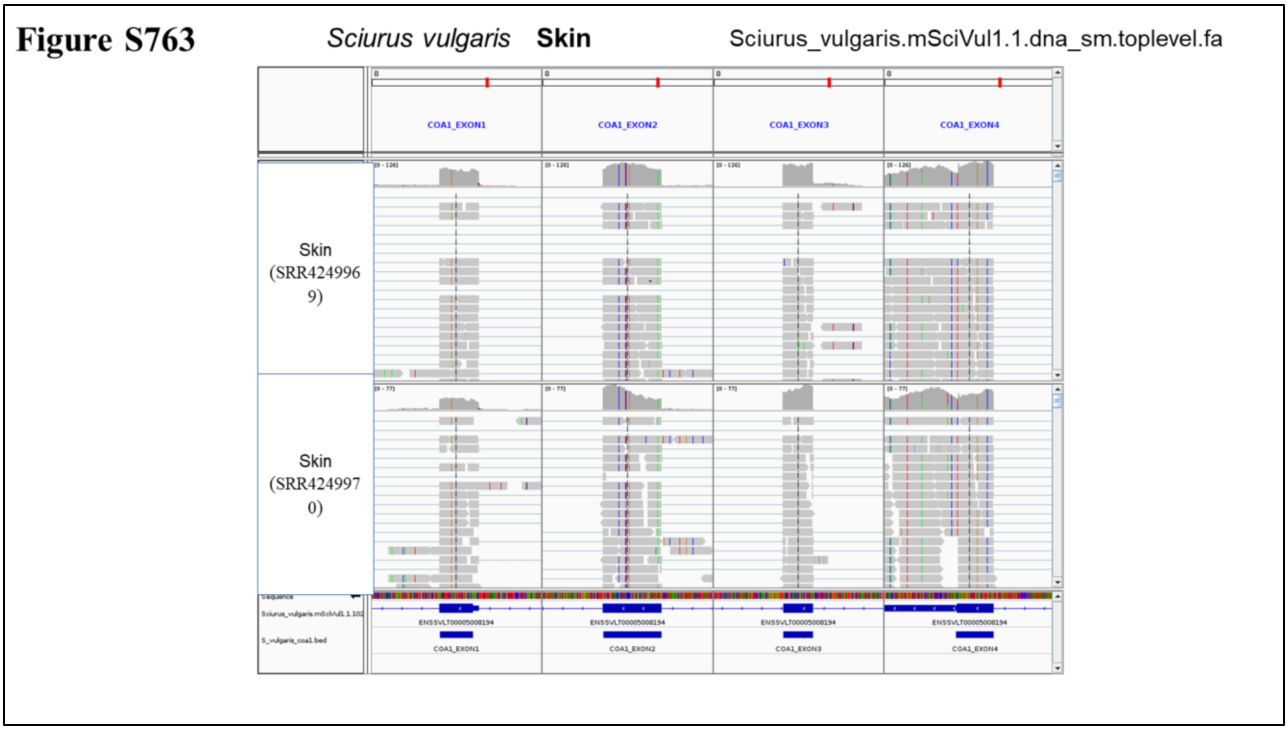

Figure S763: A zoomed in view of each of the *COA1* exons shown in **Supplementary Fig. S762**.

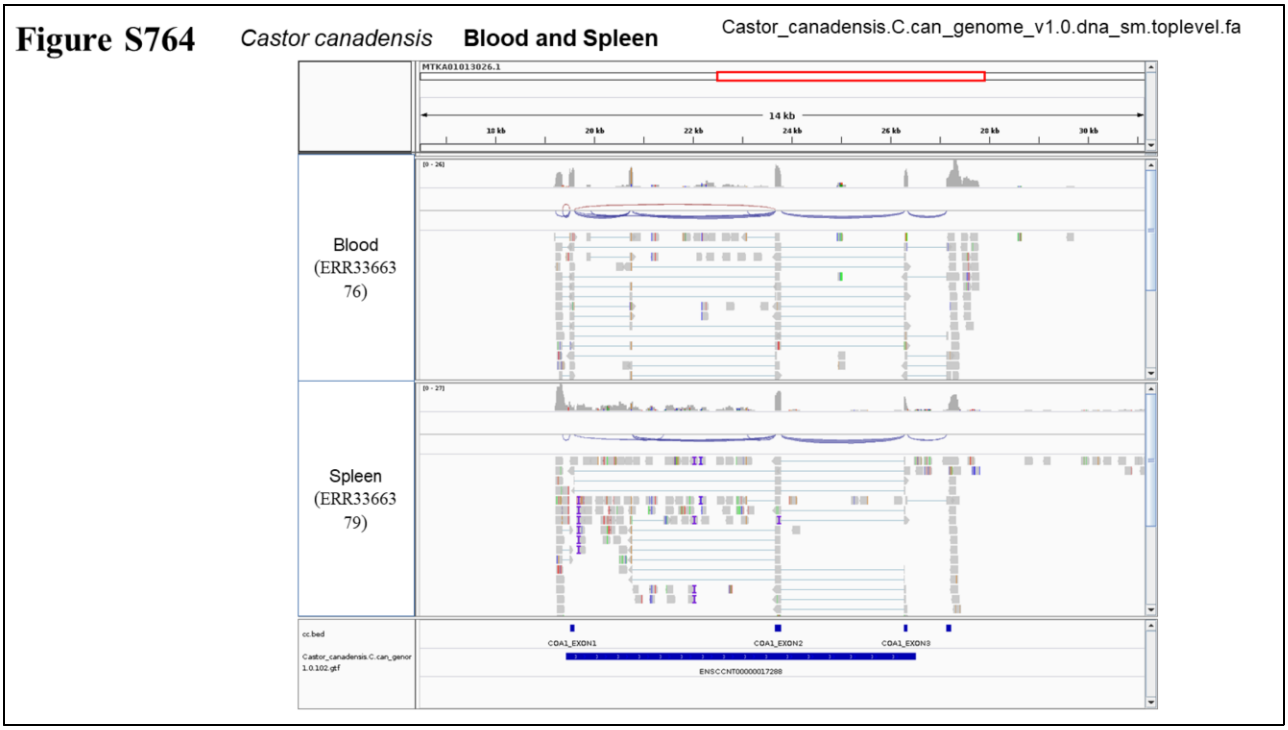

Figure S764: Screenshot of RNA-seq reads aligned to each of the putative locations of the four *COA1* exons in American beaver (*Castor canadensis*). Gray boxes represent reads aligned to the genome and thin lines connecting these boxes represent the spliced read alignments. Datasets from blood (ERR3366376) and spleen (ERR3366379) have been mapped to the Castor_canadensis.C.can_genome_v1.0.dna_sm.toplevel.fa. Locations of each of the exons are depicted as bed record in the bottom row.

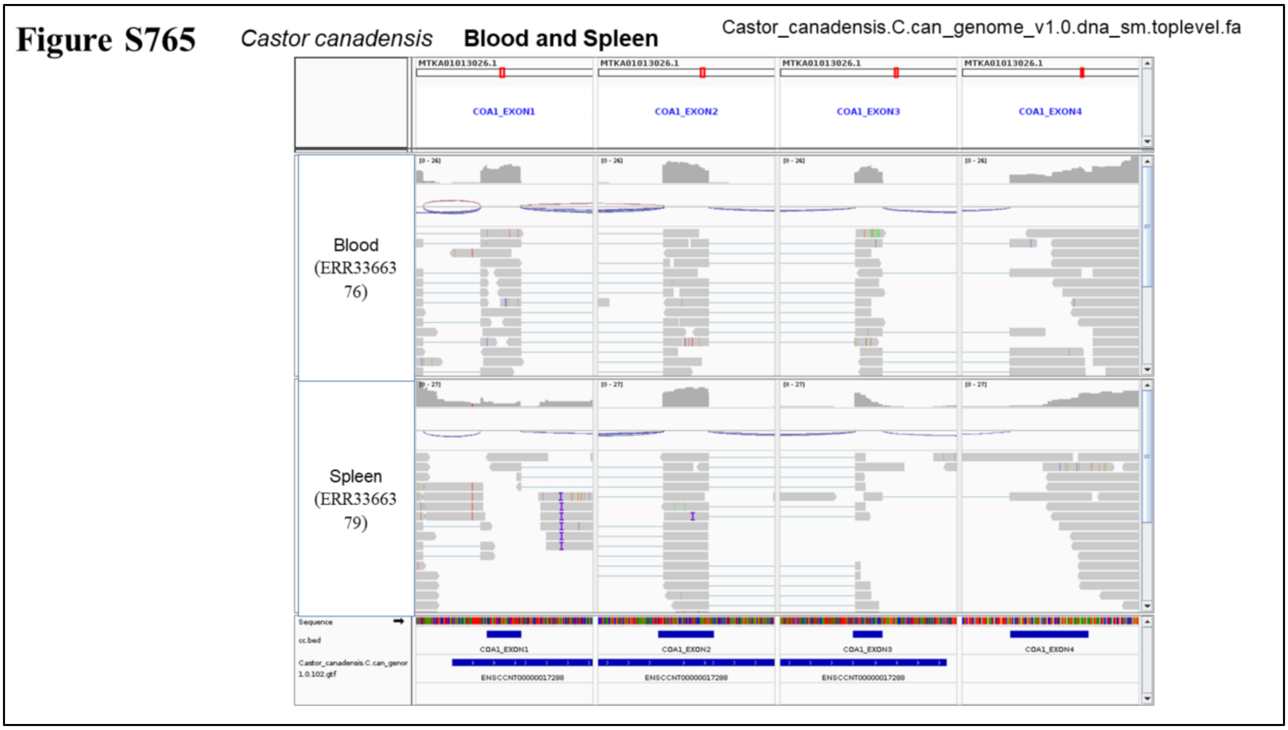

Figure S765: A zoomed in view of each of the *COA1* exons shown in **Supplementary Fig. S764**.

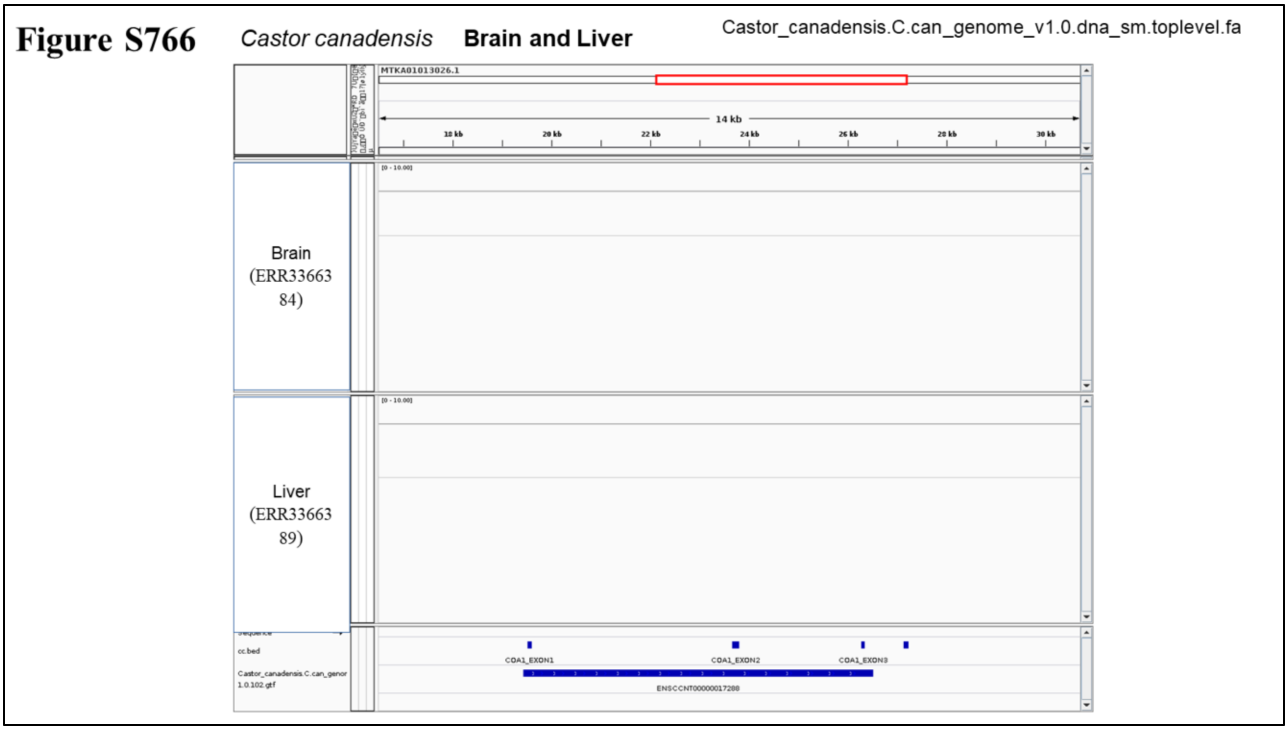

Figure S766: Screenshot of RNA-seq reads aligned to each of the putative locations of the four *COA1* exons in American beaver (*Castor canadensis*). Gray boxes represent reads aligned to the genome and thin lines connecting these boxes represent the spliced read alignments. Datasets from brain (ERR3366384) and liver (ERR3366389) have been mapped to the Castor_canadensis.C.can_genome_v1.0.dna_sm.toplevel.fa. Locations of each of the exons are depicted as bed record in the bottom row.

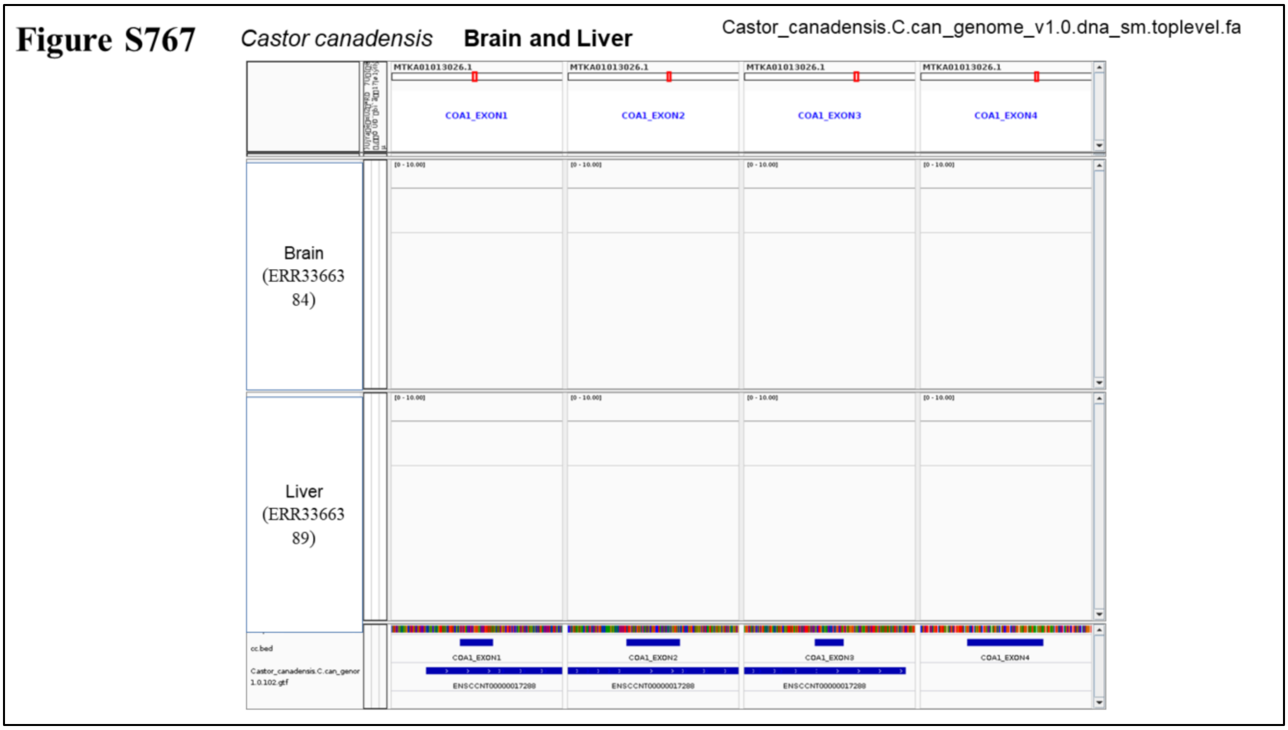

Figure S767: A zoomed in view of each of the *COA1* exons shown in **Supplementary Fig. S765**.

Figure S768 *Castor canadensis* **Stomach and Ovarian follicle** Castor_canadensis.C.can_genome_v1.0.dna_sm.toplevel.fa

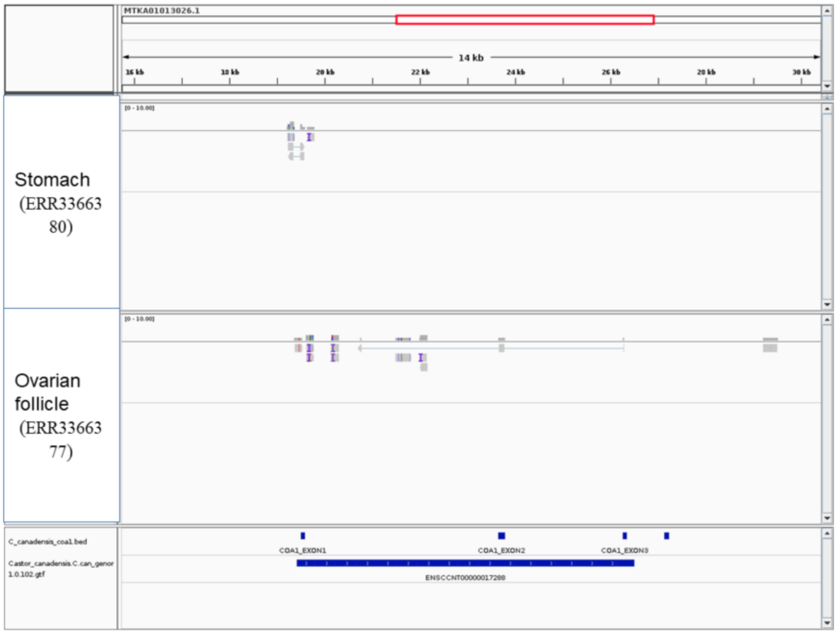

Figure S768: Screenshot of RNA-seq reads aligned to each of the putative locations of the four *COA1* exons in American beaver (*Castor canadensis*). Gray boxes represent reads aligned to the genome and thin lines connecting these boxes represent the spliced read alignments. Datasets from stomach (ERR3366380) and ovarian follicle (ERR3366377) have been mapped to the Castor_canadensis.C.can_genome_v1.0.dna_sm.toplevel.fa. Locations of each of the exons are depicted as bed record in the bottom row.

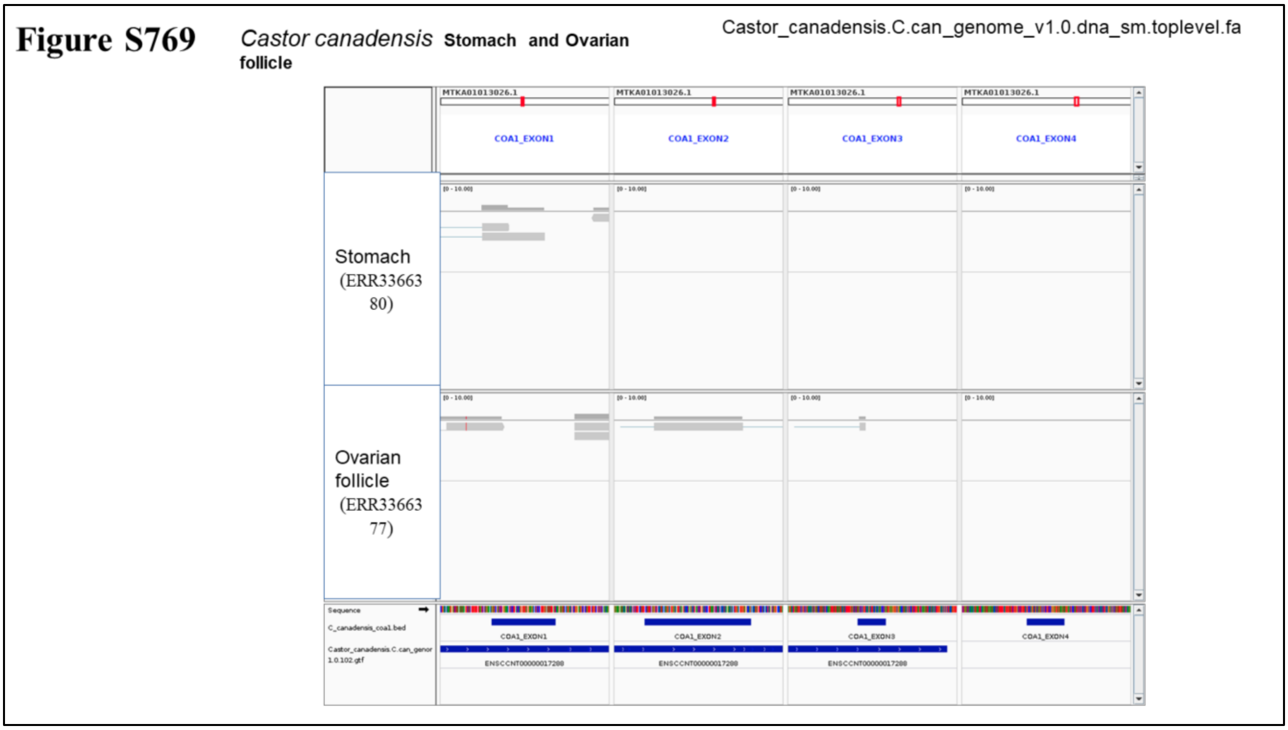

Figure S769: A zoomed in view of each of the *COA1* exons shown in **Supplementary Fig. S768**.

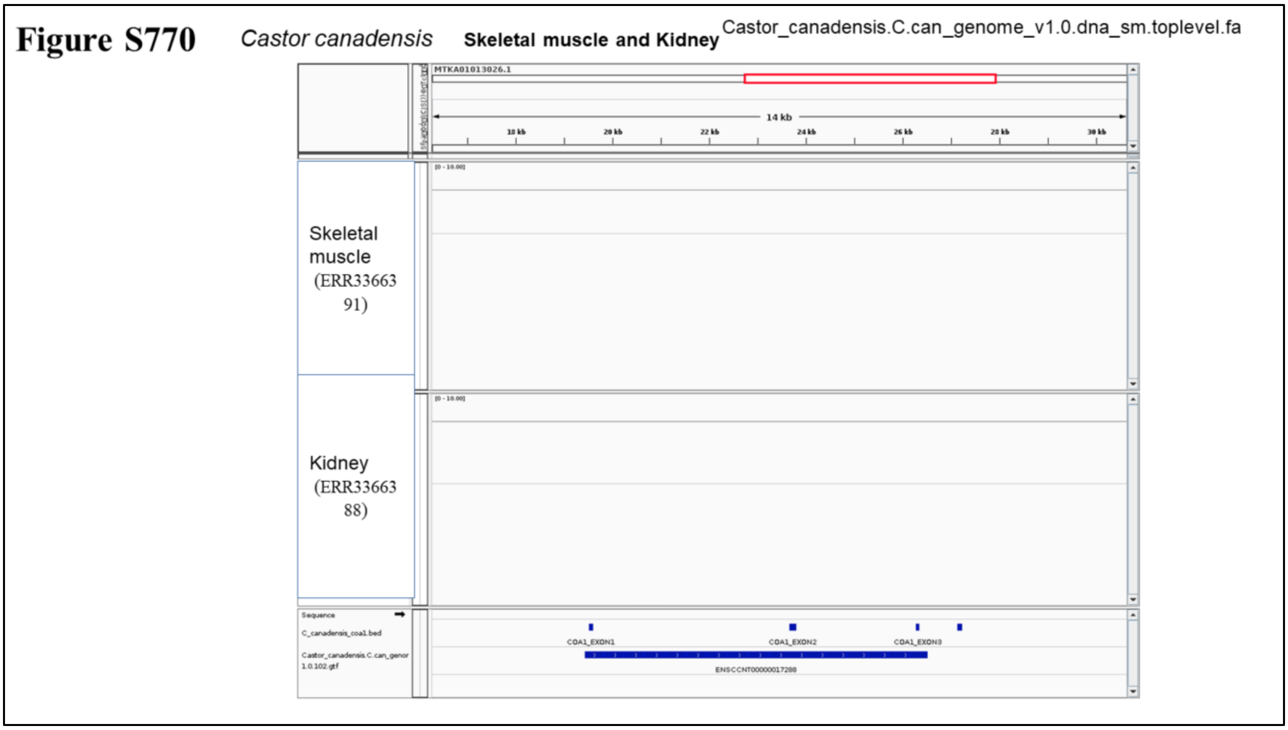

Figure S770: Screenshot of RNA-seq reads aligned to each of the putative locations of the four *COA1* exons in American beaver (*Castor canadensis*). Gray boxes represent reads aligned to the genome and thin lines connecting these boxes represent the spliced read alignments. Datasets from skeletal muscle (ERR3366391) and kidney (ERR3366388) have been mapped to the Castor_canadensis.C.can_genome_v1.0.dna_sm.toplevel.fa. Locations of each of the exons are depicted as bed record in the bottom row.

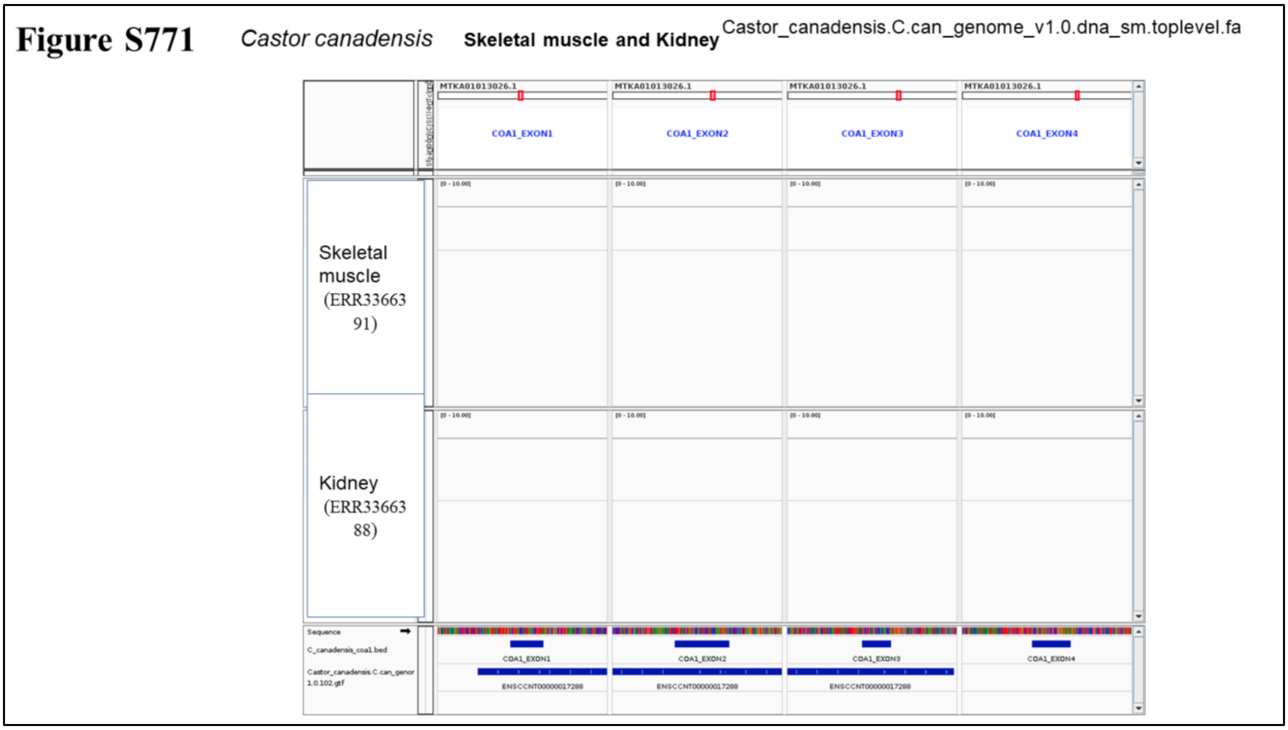

Figure S771: A zoomed in view of each of the *COA1* exons shown in **Supplementary Fig. S770**.

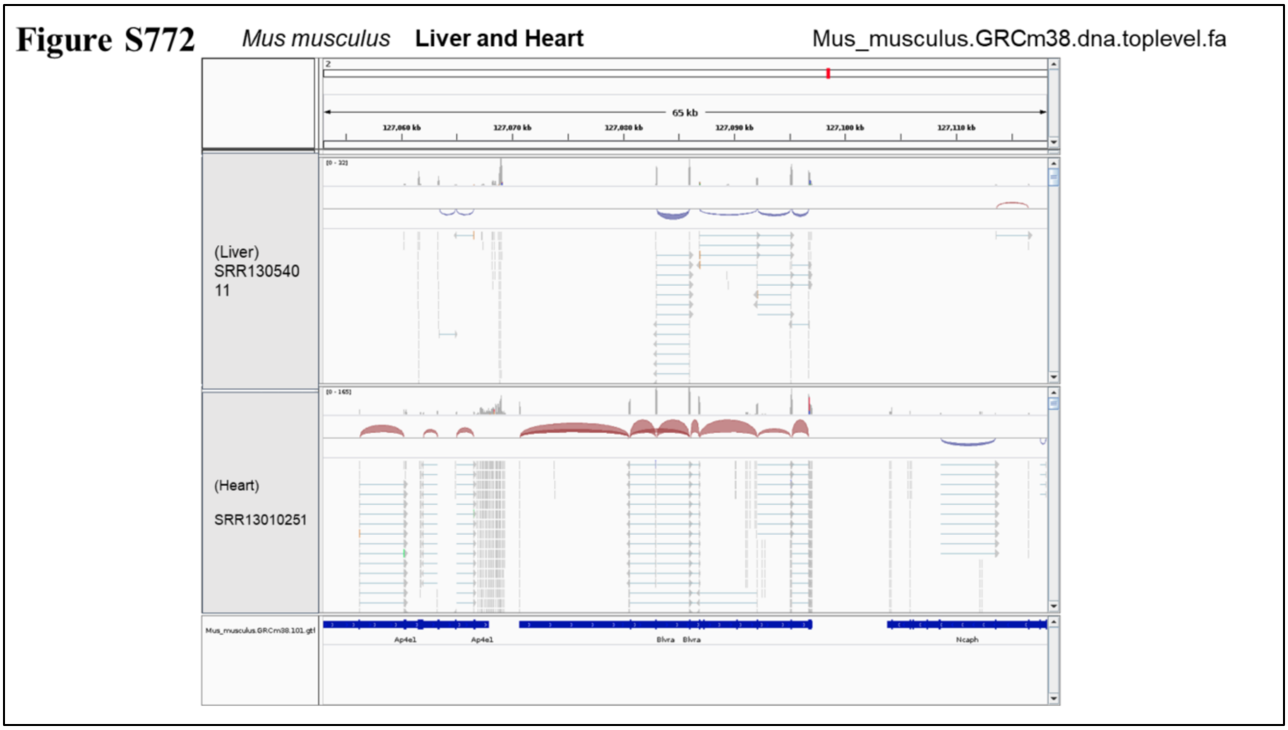

Figure S772: Screenshot of RNA-seq reads aligned each of the putative locations of *AP4E1* and *NCAPH* in mouse (*Mus musculus*). Gray boxes represent reads aligned to the genome and thin lines connecting these boxes represent the spliced read alignments. Datasets from liver (SRR13054011) and heart (SRR13010251) have been mapped to the Mus_musculus.GRCm38.dna.toplevel.fa genome. Locations of *AP4E1* and *NCAPH* are depicted as Mus_musculus.GRCm38.101.gtf record in the bottom row.

Figure S773 *Mus musculus* **Liver and Heart** Mus_musculus.GRCm38.dna.toplevel.fa

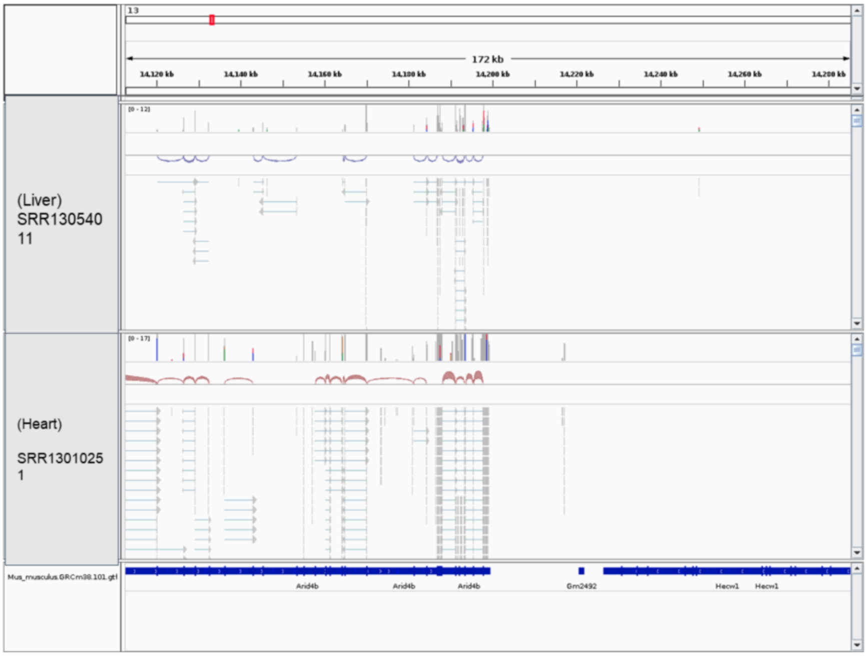

Figure S773: Screenshot of RNA-seq reads aligned each of the putative locations of *HECW1* and *ARID4B* in mouse (*Mus musculus*). Gray boxes represent reads aligned to the genome and thin lines connecting these boxes represent the spliced read alignments. Datasets from liver (SRR13054011) and heart (SRR13010251) have been mapped to the Mus_musculus.GRCm38.dna.toplevel.fa genome. Locations of *HECW1* and *ARID4B* are depicted as Mus_musculus.GRCm38.101.gtf record in the bottom row.

Figure S774 *Mus musculus* **Liver and Heart** Mus_musculus.GRCm38.dna.toplevel.fa

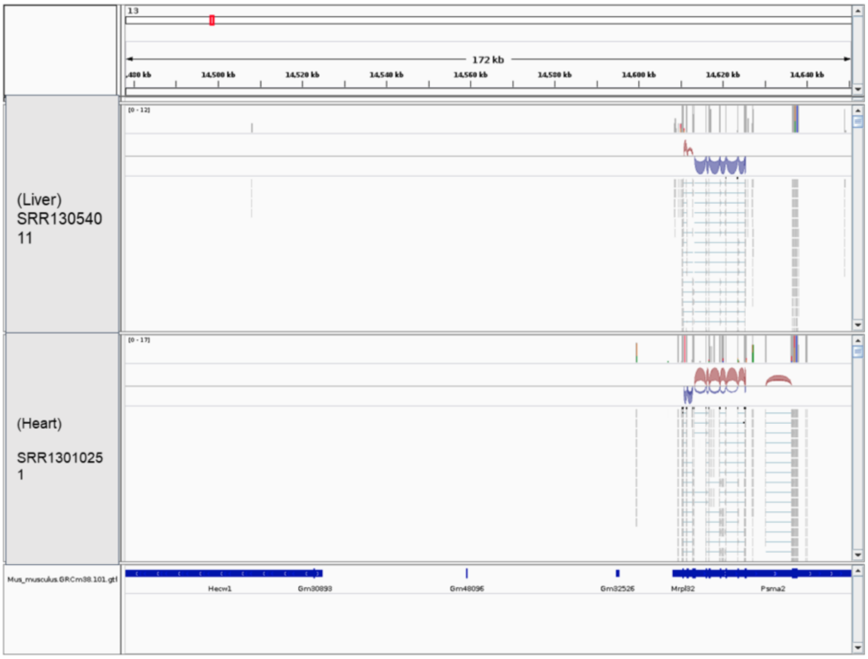

Figure S774: Screenshot of RNA-seq reads aligned each of the putative locations of *HECW1* and *PSMA2* in mouse (*Mus musculus*). Gray boxes represent reads aligned to the genome and thin lines connecting these boxes represent the spliced read alignments. Datasets from liver (SRR13054011) and heart (SRR13010251) have been mapped to the Mus_musculus.GRCm38.dna.toplevel.fa genome. Locations of *HECW1* and *PSMA2* are depicted as Mus_musculus.GRCm38.101.gtf record in the bottom row.

Figure S775

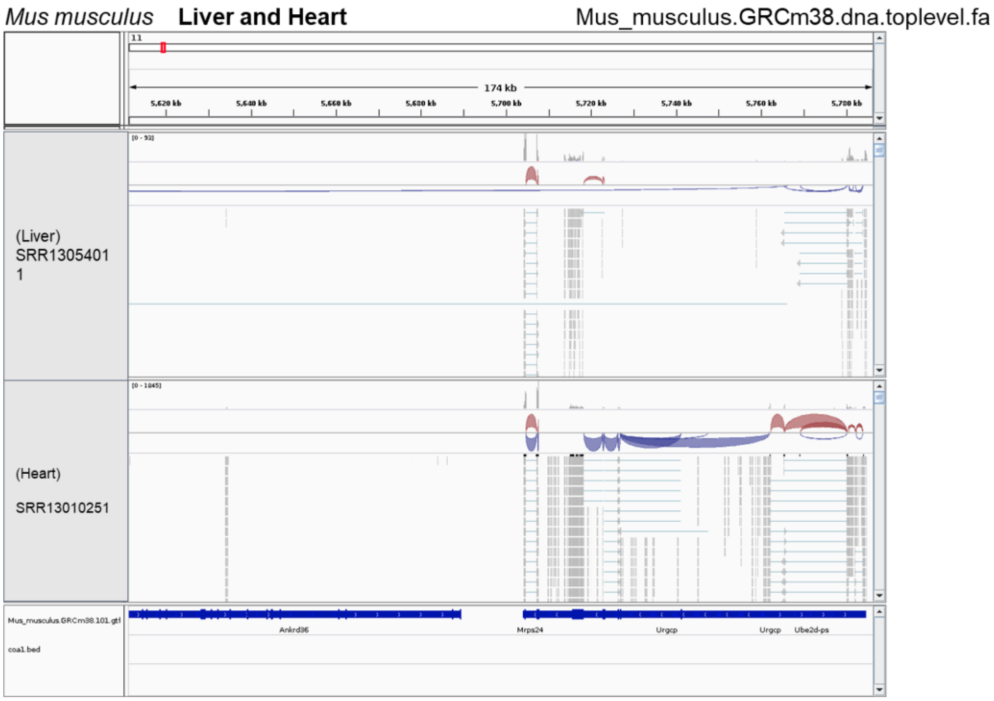

Figure S775: Screenshot of RNA-seq reads aligned each of the putative locations of *ANKRD36* and *URGCP* in mouse (*Mus musculus*). Gray boxes represent reads aligned to the genome and thin lines connecting these boxes represent the spliced read alignments. Datasets from liver (SRR13054011) and heart (SRR13010251) have been mapped to the Mus_musculus.GRCm38.dna.toplevel.fa genome. Locations of *ANKRD36* and *URGCP* are depicted as Mus_musculus.GRCm38.101.gtf record in the bottom row.

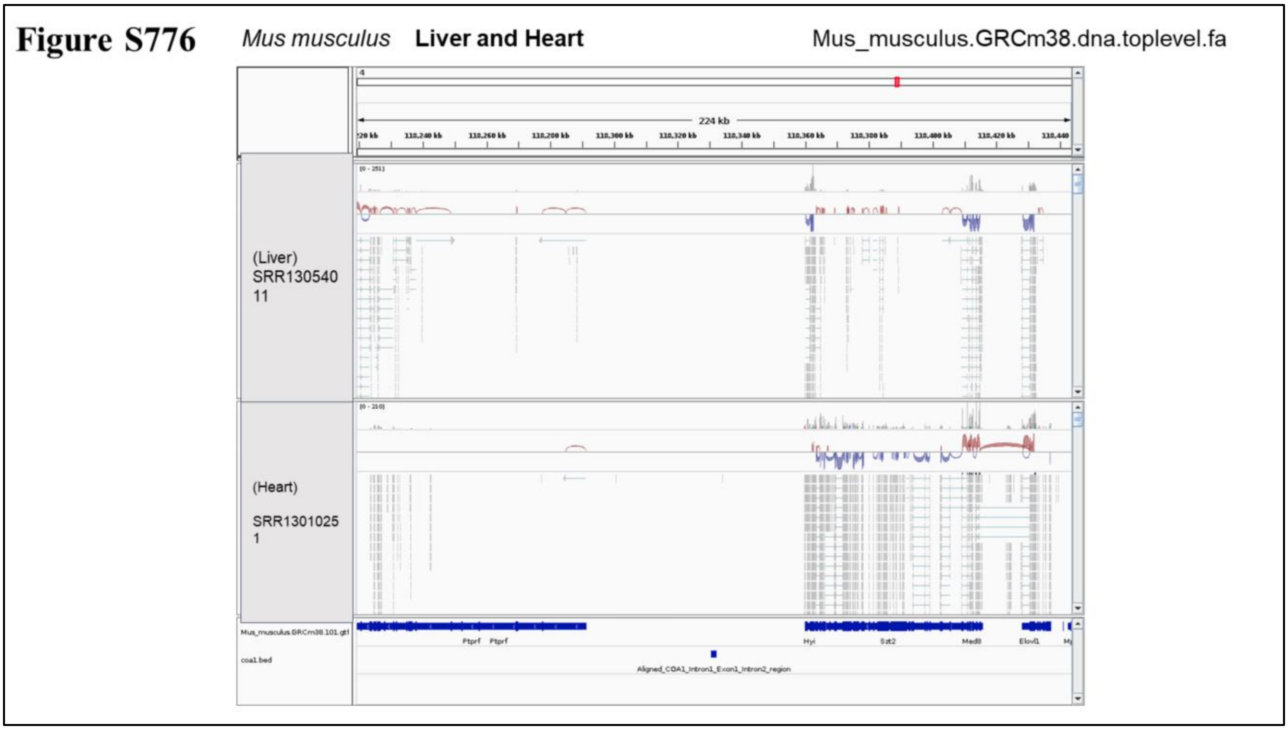

Figure S776: Screenshot of RNA-seq reads aligned each of the putative locations of truncated exon 1, intron-1 and intron-2 of *COA1* in mouse (*Mus musculus*). Gray boxes represent reads aligned to the genome and thin lines connecting these boxes represent the spliced read alignments. Datasets from liver (SRR13054011) and heart (SRR13010251) have been mapped to the Mus_musculus.GRCm38.dna.toplevel.fa genome. Locations of truncated exon 1, intron-1 and intron-2 of *COA1* is depicted as bed record in the bottom row.

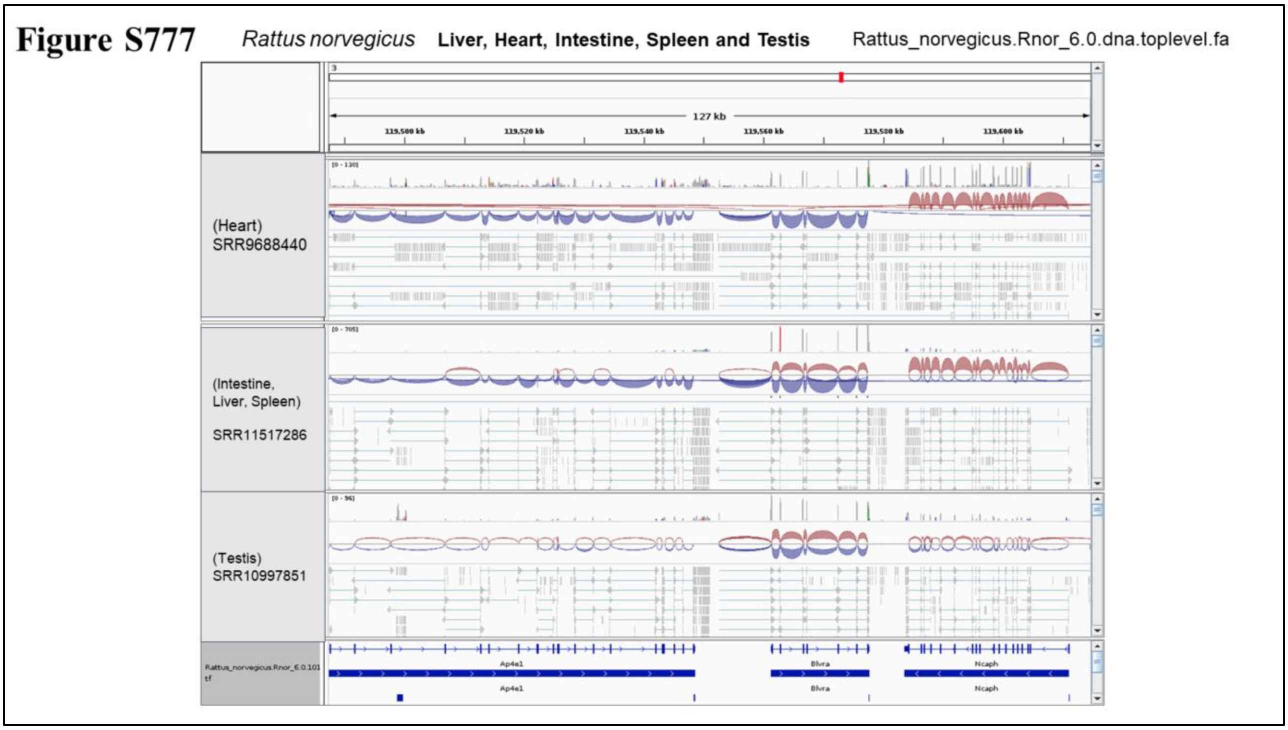

Figure S777: Screenshot of RNA-seq reads aligned each of the putative locations of *AP4E1* and *NCAPH* in rat (*Rattus norvegicus*). Gray boxes represent reads aligned to the genome and thin lines connecting these boxes represent the spliced read alignments. Datasets from liver, spleen and intestine (SRR11517286) and heart (SRR9688440) and testis (SRR10997851) have been mapped to the Rattus_norvegicus.Rnor_6.0.dna.toplevel.fa genome. Locations of *AP4E1* and *NCAPH* are depicted as Rattus_norvegicus.Rnor_6.0.101.gtf record in the bottom row.

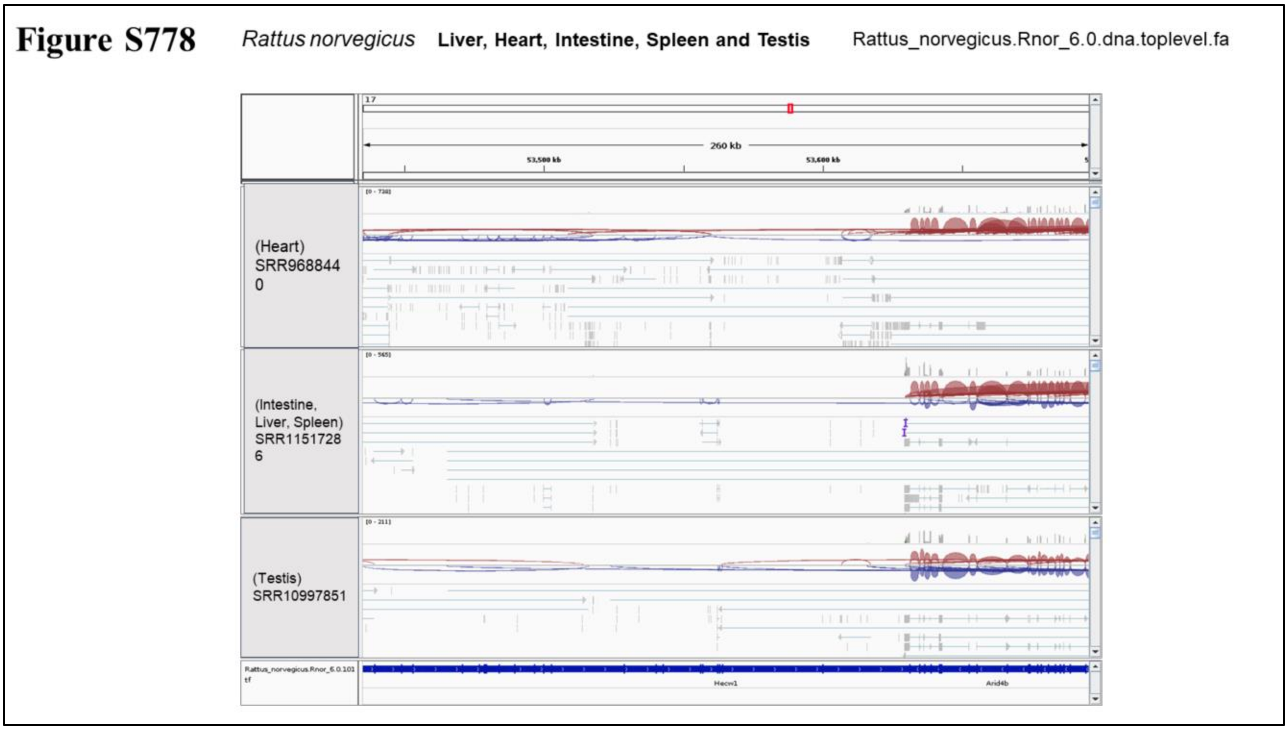

Figure S778: Screenshot of RNA-seq reads aligned each of the putative locations of *HECW1* and *ARID4B* in rat (*Rattus norvegicus*). Gray boxes represent reads aligned to the genome and thin lines connecting these boxes represent the spliced read alignments. Datasets from liver, spleen and intestine (SRR11517286) and heart (SRR9688440) and testis (SRR10997851) have been mapped to the Rattus_norvegicus.Rnor_6.0.dna.toplevel.fa genome. Locations of *HECW1* and *ARID4B* are depicted as Rattus_norvegicus.Rnor_6.0.101.gtf record in the bottom row.

Figure S779 *Rattus norvegicus* **Liver, Heart, Intestine, Spleen and Testis** Rattus_norvegicus.Rnor_6.0.dna.toplevel.fa

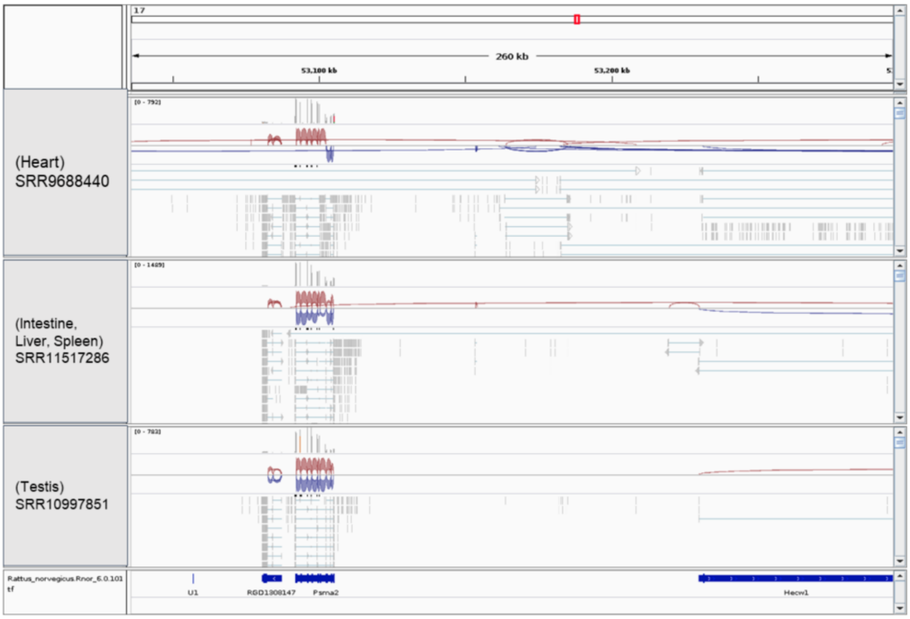

Figure S779: Screenshot of RNA-seq reads aligned each of the putative locations of *PSMA2* and *HECW1* in rat (*Rattus norvegicus*). Gray boxes represent reads aligned to the genome and thin lines connecting these boxes represent the spliced read alignments. Datasets from liver, spleen and intestine (SRR11517286) and heart (SRR9688440) and testis (SRR10997851) have been mapped to the Rattus_norvegicus.Rnor_6.0.dna.toplevel.fa genome. Locations of *PSMA2* and *HECW1* are depicted as Rattus_norvegicus.Rnor_6.0.101.gtf record in the bottom row.

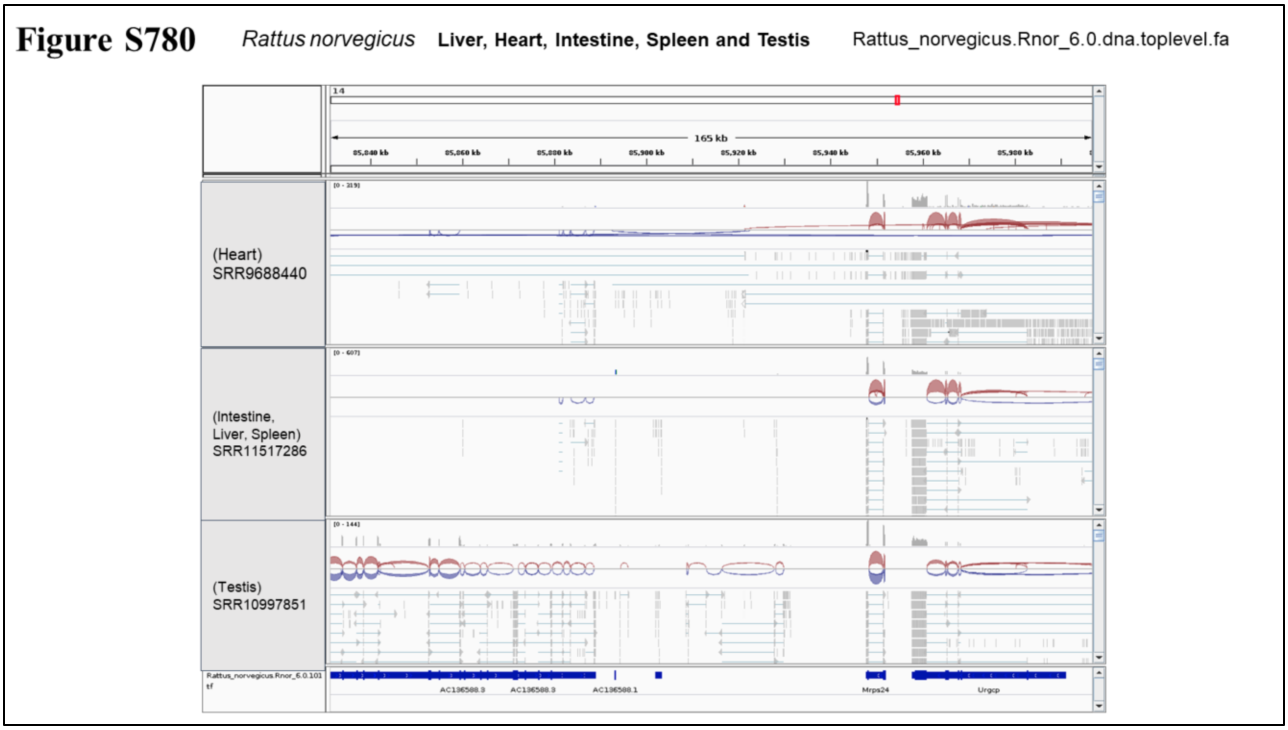

Figure S780: Screenshot of RNA-seq reads aligned each of the putative locations of *ANKRD26* and *URGCP* in rat (*Rattus norvegicus*). Gray boxes represent reads aligned to the genome and thin lines connecting these boxes represent the spliced read alignments. Datasets from liver, spleen and intestine (SRR11517286) and heart (SRR9688440) and testis (SRR10997851) have been mapped to the Rattus_norvegicus.Rnor_6.0.dna.toplevel.fa genome. Locations of *ANKRD26* and *URGCP* are depicted as Rattus_norvegicus.Rnor_6.0.101.gtf record in the bottom row.

Figure S781 *Rattus norvegicus* **Liver, Heart, Intestine, Spleen and Testis** Rattus_norvegicus.Rnor_6.0.dna.toplevel.fa

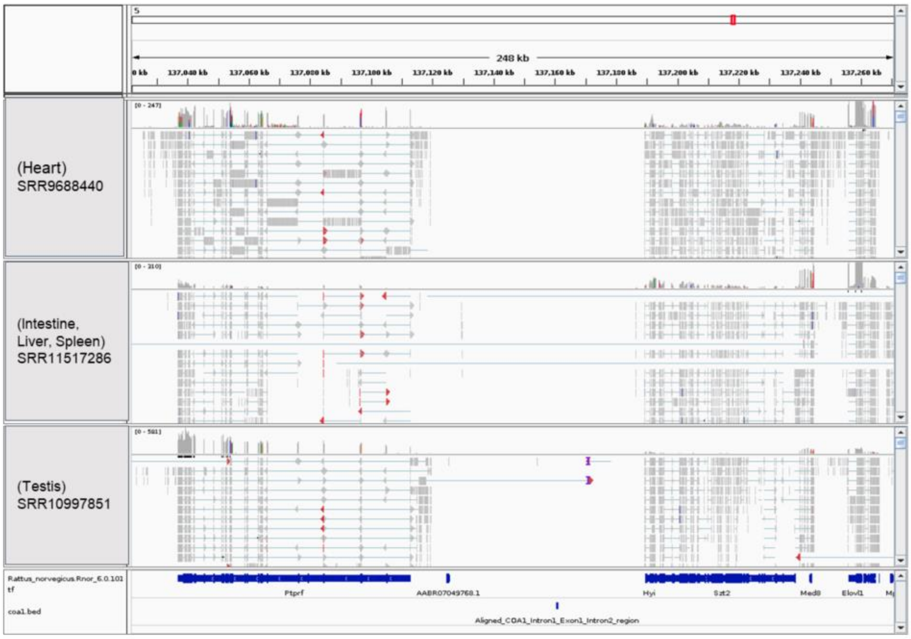

Figure S781: Screenshot of RNA-seq reads aligned each of the putative locations of truncated exon 1, intron-1 and intron-2 in rat (*Rattus norvegicus*). Gray boxes represent reads aligned to the genome and thin lines connecting these boxes represent the spliced read alignments. Datasets from liver, spleen and intestine (SRR11517286) and heart (SRR9688440) and testis (SRR10997851) have been mapped to the Rattus_norvegicus.Rnor_6.0.dna.toplevel.fa genome. Locations of truncated exon 1, intron-1 and intron-2 is depicted as bed record in the bottom row.

Figure S782 *Mus spicilegus* **Testis, Liver and Brain** Mus_spicilegus.MUSP714.dna_sm.toplevel.fa

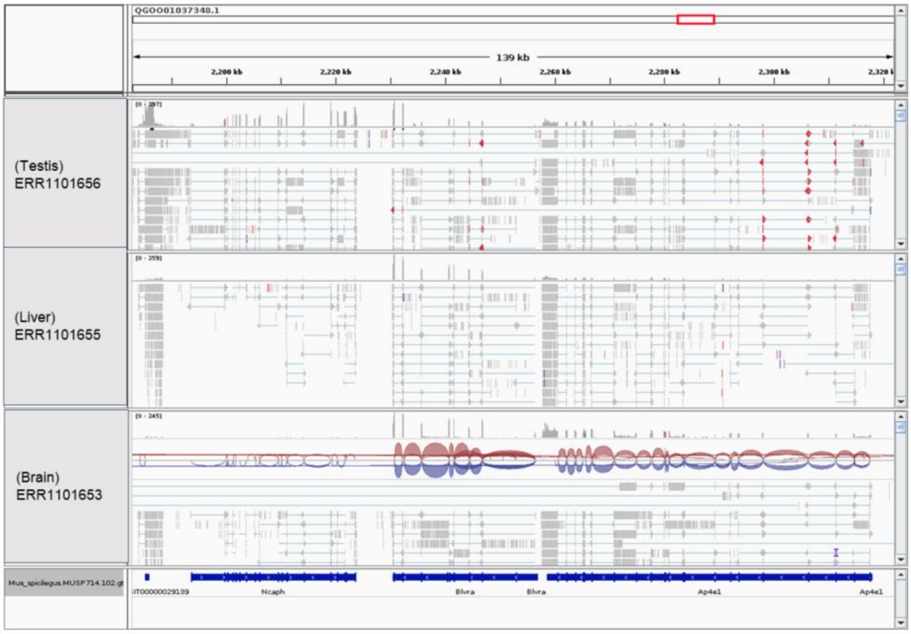

Figure S782: Screenshot of RNA-seq reads aligned each of the putative locations of *AP4E1* and *NCAPH* in Steppe mouse (*Mus spicilegus*). Gray boxes represent reads aligned to the genome and thin lines connecting these boxes represent the spliced read alignments. Datasets from testis (ERR1101656), liver (ERR1101655) and brain (ERR1101653) have been mapped to the Mus_spicilegus.MUSP714.dna_sm.toplevel.fa genome. Locations of *AP4E1* and *NCAPH* are depicted as Mus_spicilegus.MUSP714.102.gtf record in the bottom row.

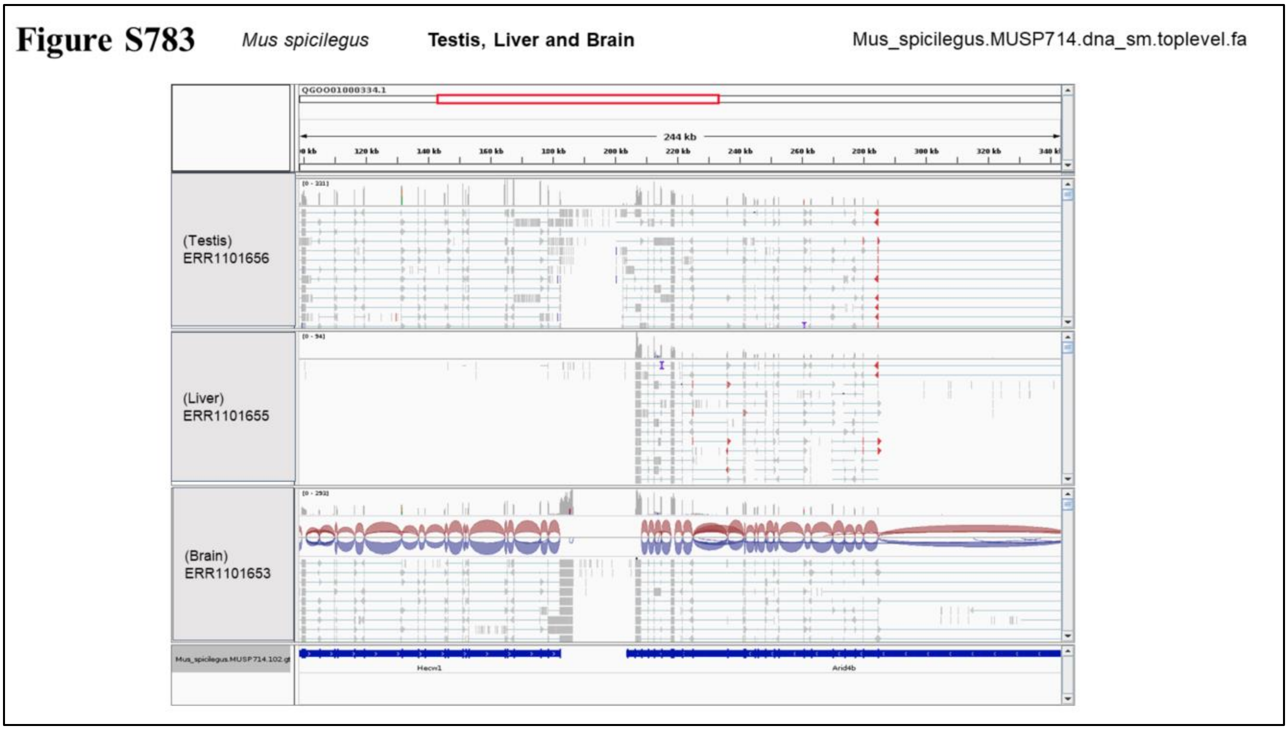

Figure S783: Screenshot of RNA-seq reads aligned each of the putative locations of *HECW1* and *ARID4B* in Steppe mouse (*Mus spicilegus*). Gray boxes represent reads aligned to the genome and thin lines connecting these boxes represent the spliced read alignments. Datasets from testis (ERR1101656), liver (ERR1101655) and brain (ERR1101653) have been mapped to the Mus_spicilegus.MUSP714.dna_sm.toplevel.fa genome. Locations of *HECW1* and *ARID4B* are depicted as Mus_spicilegus.MUSP714.102.gtf record in the bottom row.

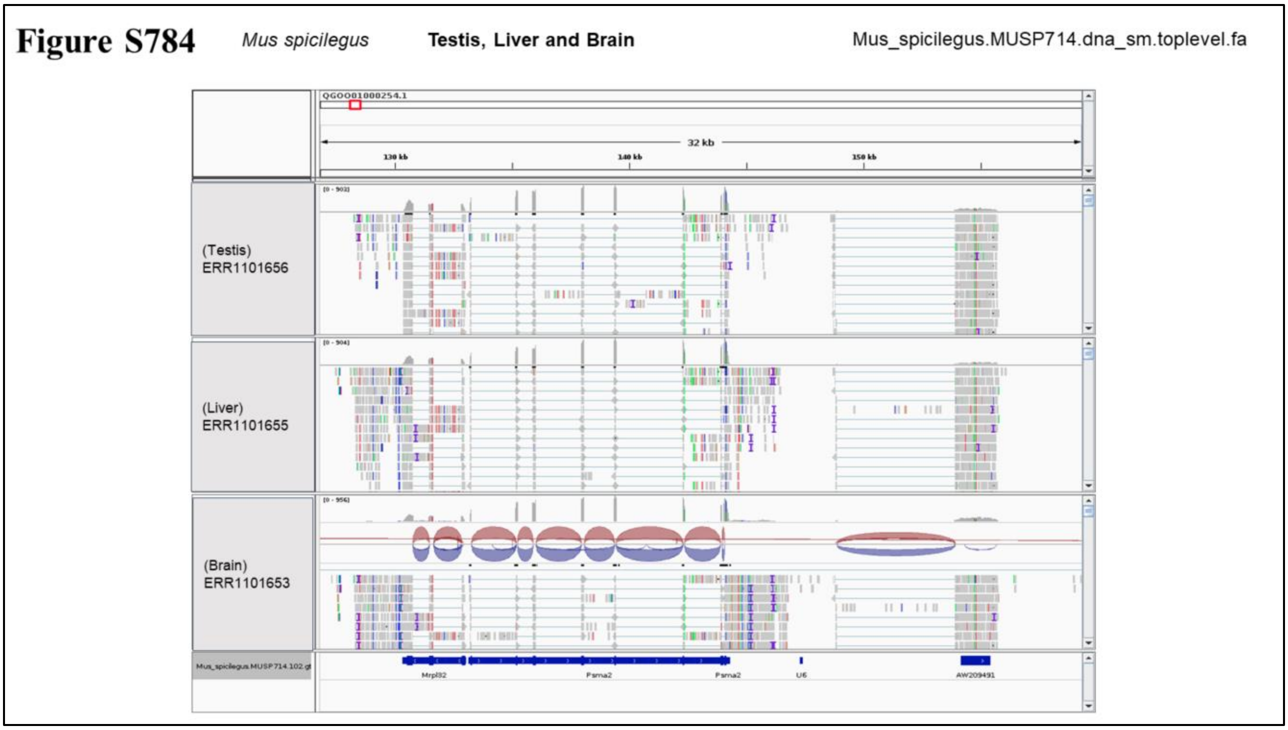

Figure S784: Screenshot of RNA-seq reads aligned each of the putative locations of *MRPL32* and *PSMA2* in Steppe mouse (*Mus spicilegus*). Gray boxes represent reads aligned to the genome and thin lines connecting these boxes represent the spliced read alignments. Datasets from testis (ERR1101656), liver (ERR1101655) and brain (ERR1101653) have been mapped to the Mus_spicilegus.MUSP714.dna_sm.toplevel.fa genome. Locations of *MRPL32* and *PSMA2* are depicted as Mus_spicilegus.MUSP714.102.gtf record in the bottom row.

Figure S785 *Mus spicilegus* **Testis, Liver and Brain** Mus_spicilegus.MUSP714.dna_sm.toplevel.fa

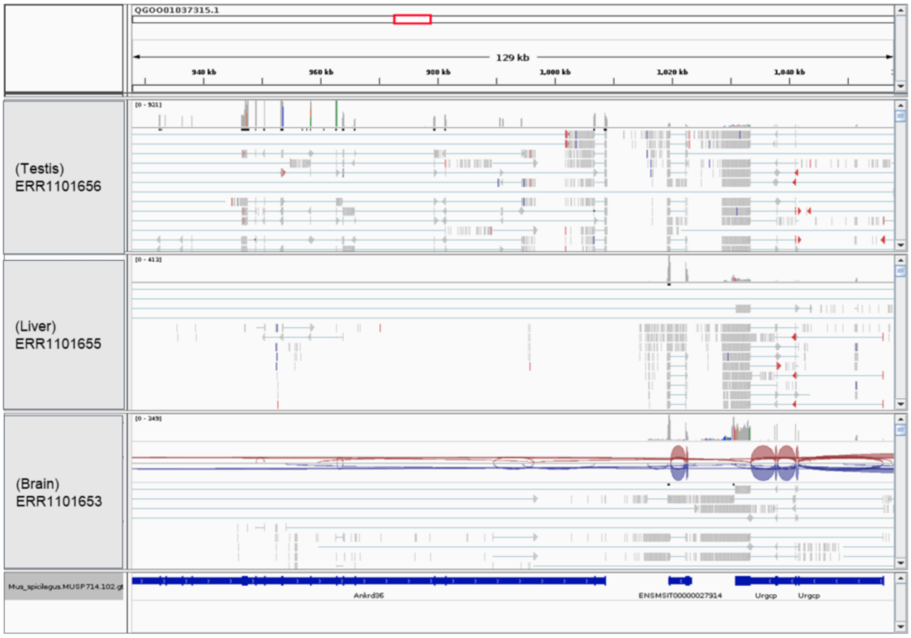

Figure S785: Screenshot of RNA-seq reads aligned each of the putative locations of *ANKRD36* and *URGCP* in Steppe mouse (*Mus spicilegus*). Gray boxes represent reads aligned to the genome and thin lines connecting these boxes represent the spliced read alignments. Datasets from testis (ERR1101656), liver (ERR1101655) and brain (ERR1101653) have been mapped to the Mus_spicilegus.MUSP714.dna_sm.toplevel.fa genome. Locations of *ANKRD36* and *URGCP* are depicted as Mus_spicilegus.MUSP714.102.gtf record in the bottom row.

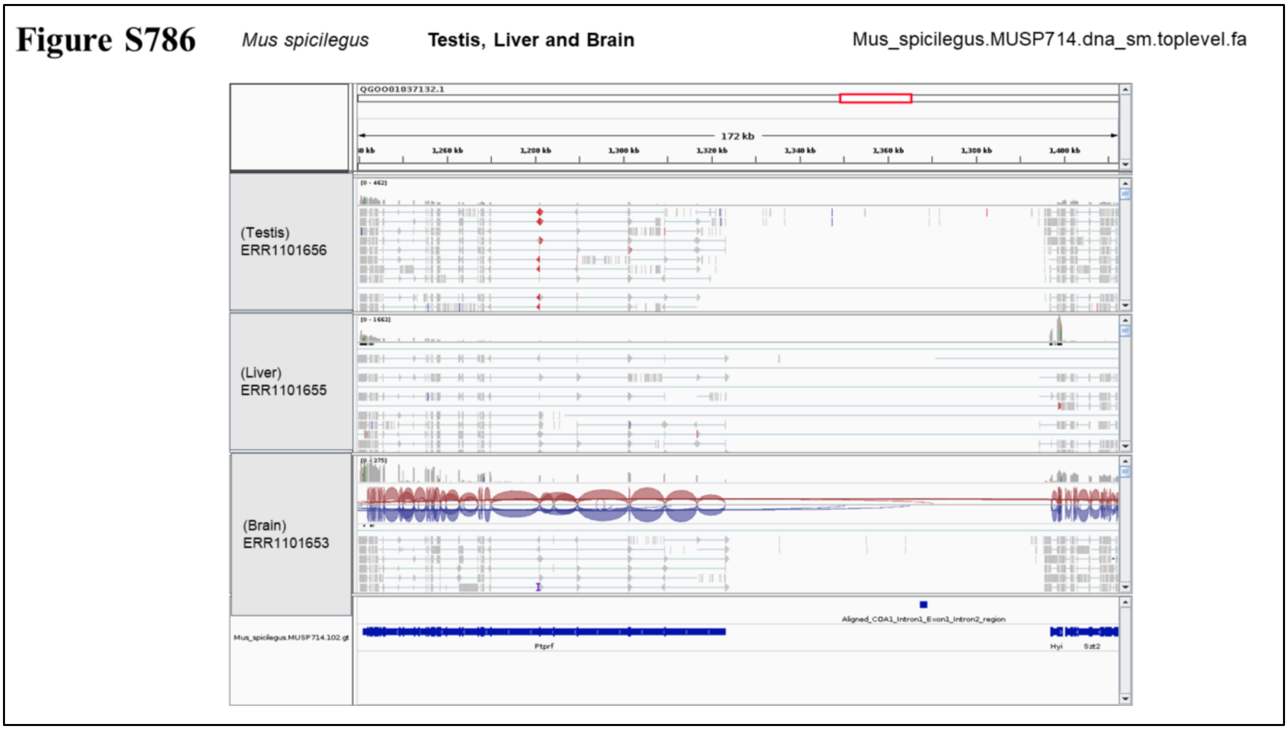

Figure S786: Screenshot of RNA-seq reads aligned each of the putative locations of truncated exon1, intron-1 and intron-2 of *COA1* in Steppe mouse (*Mus spicilegus*). Gray boxes represent reads aligned to the genome and thin lines connecting these boxes represent the spliced read alignments. Datasets from testis (ERR1101656), liver (ERR1101655) and brain (ERR1101653) have been mapped to the Mus_spicilegus.MUSP714.dna_sm.toplevel.fa genome. Locations of truncated exon 1, intron-1 and intron-2 of *COA1* is depicted as bed record in the bottom row.

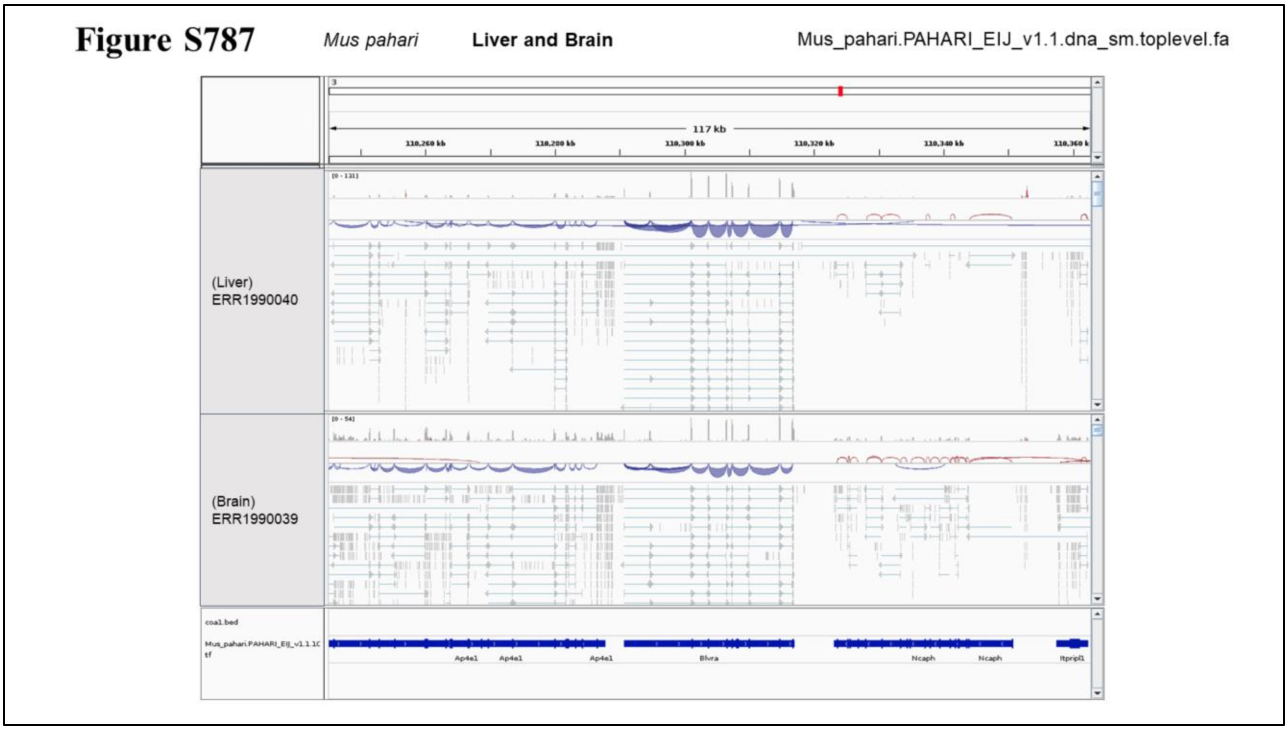

Figure S787: Screenshot of RNA-seq reads aligned each of the putative locations of *AP4E1* and *NCAPH* in Shrew mouse (*Mus pahari*). Gray boxes represent reads aligned to the genome and thin lines connecting these boxes represent the spliced read alignments. Datasets from liver (ERR1990040) and brain (ERR1990039) have been mapped to the Mus_pahari.PAHARI_EIJ_v1.1.dna_sm.toplevel.fa genome. Locations of *AP4E1* and *NCAPH* are depicted as Mus_pahari.PAHARI_EIJ_v1.1.102.gtf record in the bottom row.

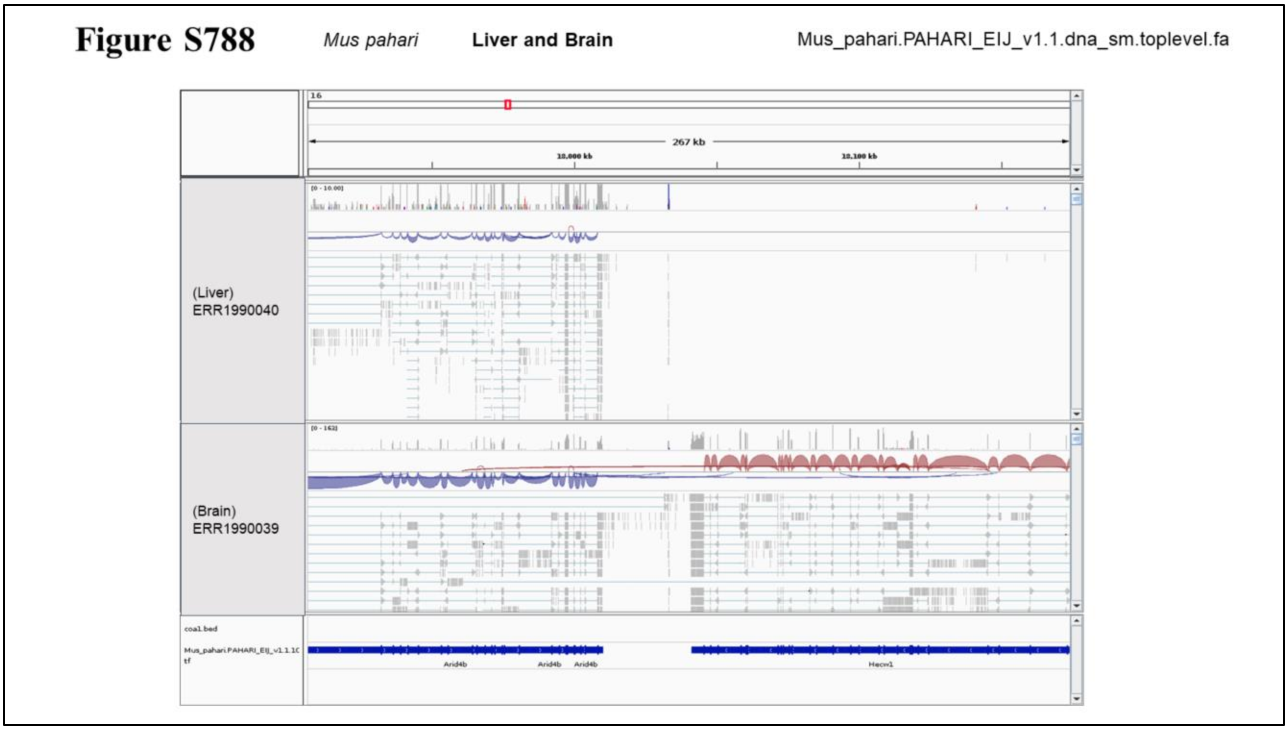

Figure S788: Screenshot of RNA-seq reads aligned each of the putative locations of *ARID4B* and *HECW1* in shrew mouse (*Mus pahari*). Gray boxes represent reads aligned to the genome and thin lines connecting these boxes represent the spliced read alignments. Datasets from liver (ERR1990040) and brain (ERR1990039) have been mapped to the Mus_pahari.PAHARI_EIJ_v1.1.dna_sm.toplevel.fa genome. Locations of *ARID4B* and *HECW1* are depicted as Mus_pahari.PAHARI_EIJ_v1.1.102.gtf record in the bottom row.

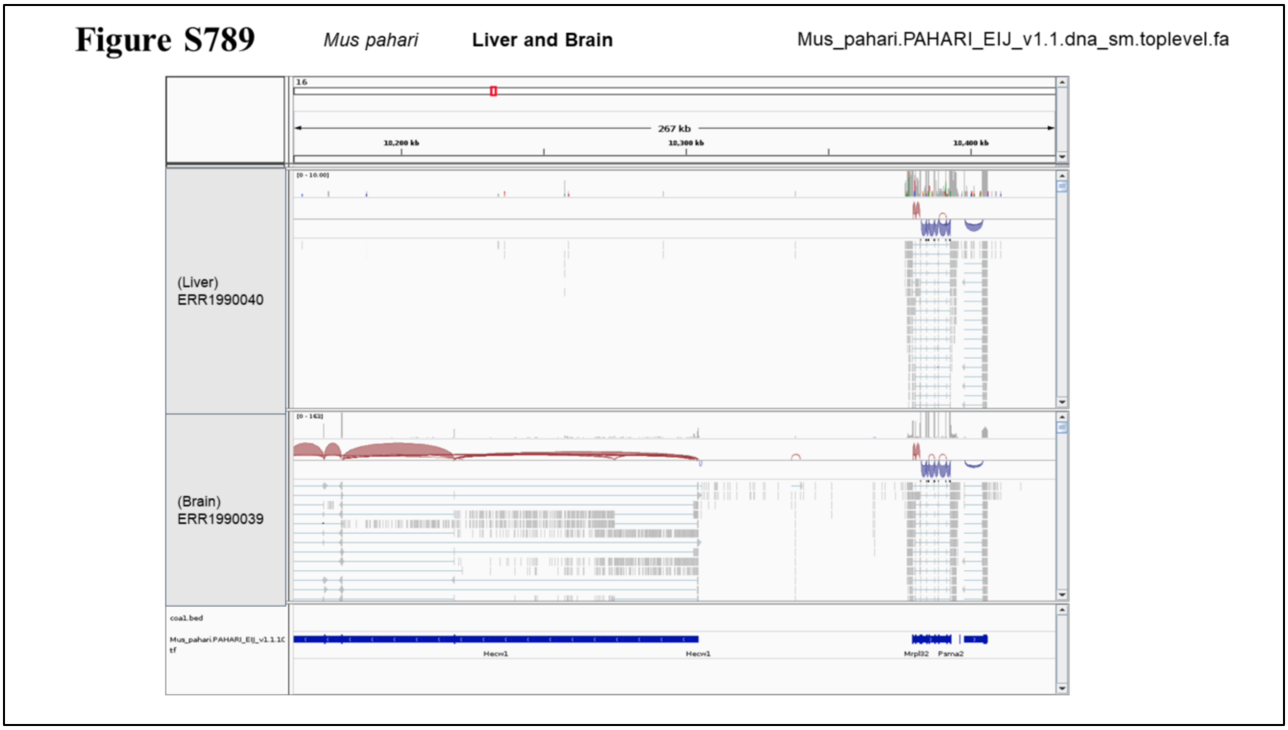

Figure S789: Screenshot of RNA-seq reads aligned each of the putative locations of *PSMA2* and *HECW1* in shrew mouse (*Mus pahari*). Gray boxes represent reads aligned to the genome and thin lines connecting these boxes represent the spliced read alignments. Datasets from liver (ERR1990040) and brain (ERR1990039) have been mapped to the Mus_pahari.PAHARI_EIJ_v1.1.dna_sm.toplevel.fa genome. Locations of *PSMA2* and *HECW1* are depicted as Mus_pahari.PAHARI_EIJ_v1.1.102.gtf record in the bottom row.

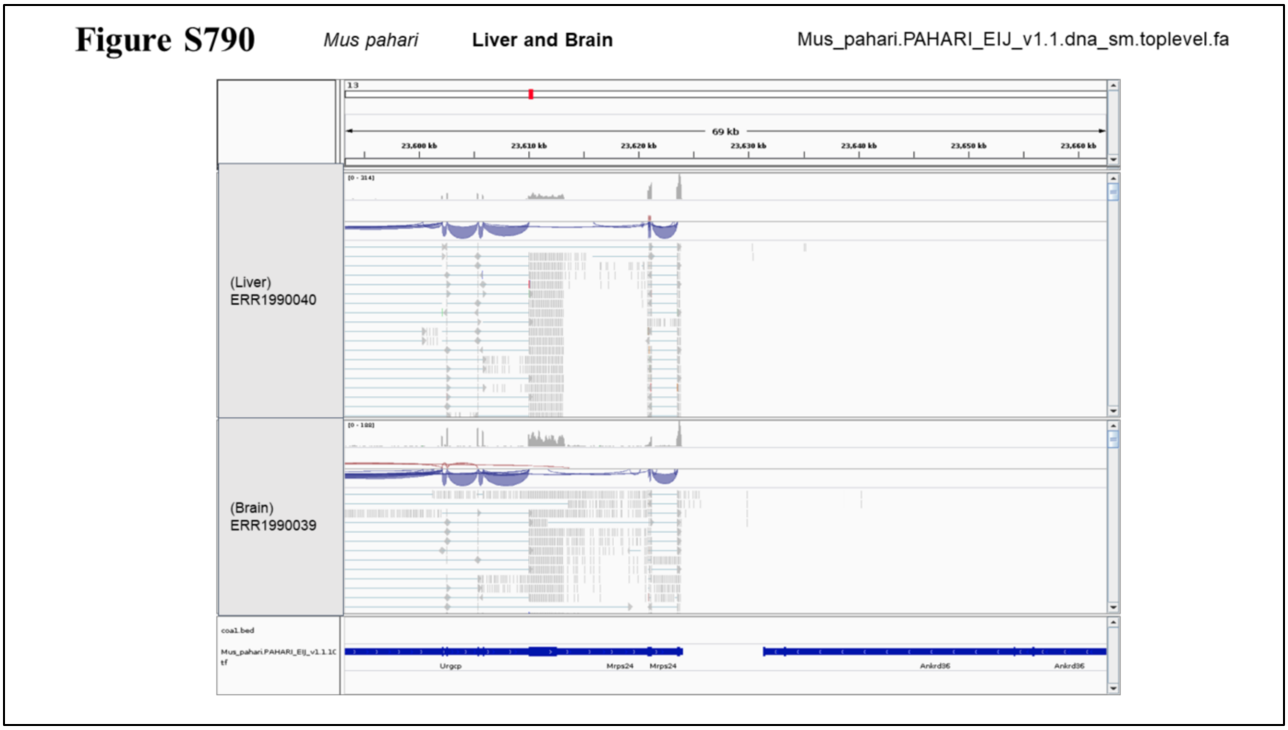

Figure S790: Screenshot of RNA-seq reads aligned each of the putative locations of *URGCP* and *ANKRD36* in shrew mouse (*Mus pahari*). Gray boxes represent reads aligned to the genome and thin lines connecting these boxes represent the spliced read alignments. Datasets from liver (ERR1990040) and brain (ERR1990039) have been mapped to the Mus_pahari.PAHARI_EIJ_v1.1.dna_sm.toplevel.fa genome. Locations of *URGCP* and *ANKRD36* are depicted as Mus_pahari.PAHARI_EIJ_v1.1.102.gtf record in the bottom row.

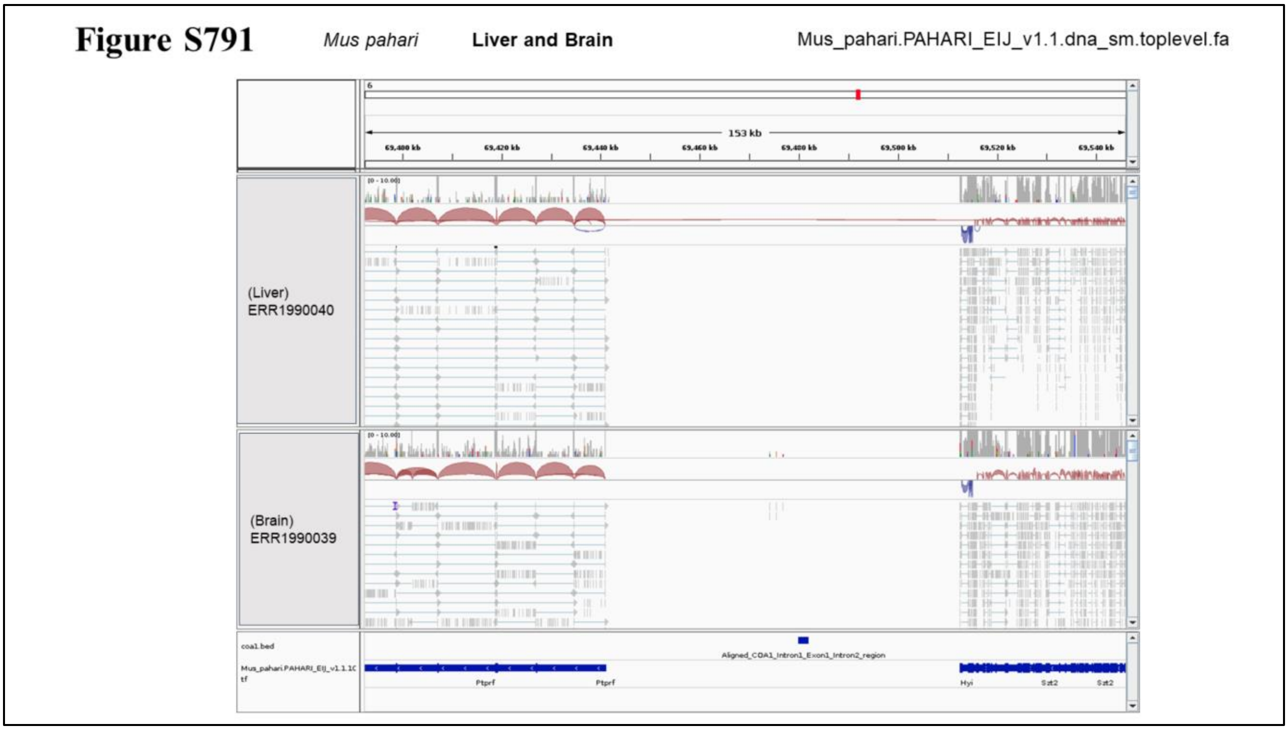

Figure S791: Screenshot of RNA-seq reads aligned each of the putative locations of truncated exon-1, intron-1 and intron-2 of *COA1* in shrew mouse (*Mus pahari*). Gray boxes represent reads aligned to the genome and thin lines connecting these boxes represent the spliced read alignments. Datasets from liver (ERR1990040) and brain (ERR1990039) have been mapped to the Mus_pahari.PAHARI_EIJ_v1.1.dna_sm.toplevel.fa genome. Locations of truncated exon 1, intron-1 and intron-2 of *COA1* is depicted as bed record in the bottom row.

Figure S792 *Mus caroli* **Liver and Brain** Mus_caroli.CAROLI_EIJ_v1.1.dna_sm.toplevel.fa

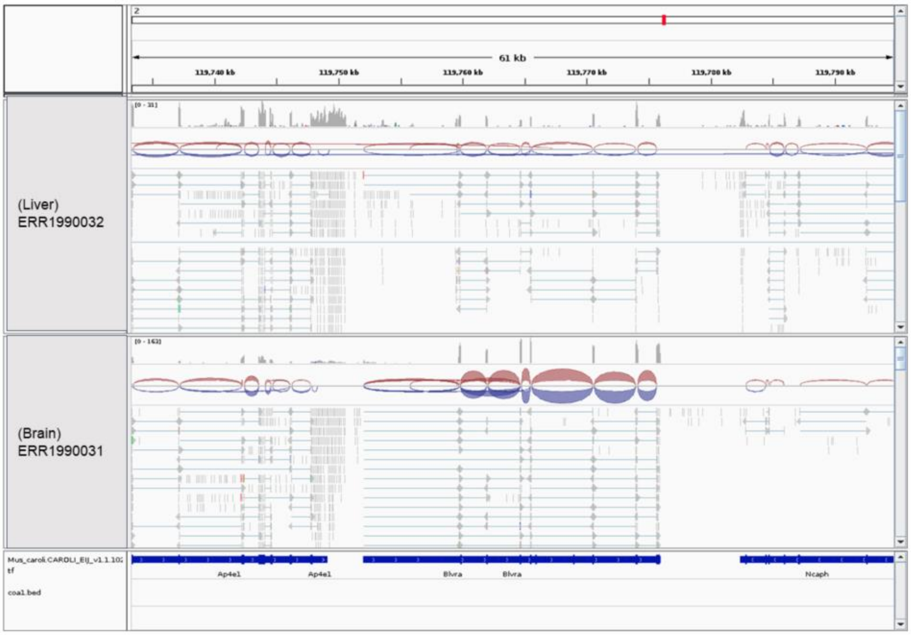

Figure S792: Screenshot of RNA-seq reads aligned each of the putative locations of *AP4E1* and *NCAPH* in Ryukyu mouse (*Mus caroli*). Gray boxes represent reads aligned to the genome and thin lines connecting these boxes represent the spliced read alignments. Datasets from liver (ERR1990032) and brain (ERR1990031) have been mapped to the Mus_caroli.CAROLI_EIJ_v1.1.dna_sm.toplevel.fa genome. Locations of *AP4E1* and *NCAPH* are depicted as Mus_caroli.CAROLI_EIJ_v1.1.102.gtf record in the bottom row.

Figure S793 *Mus caroli* **Liver and Brain** Mus_caroli.CAROLI_EIJ_v1.1.dna_sm.toplevel.fa

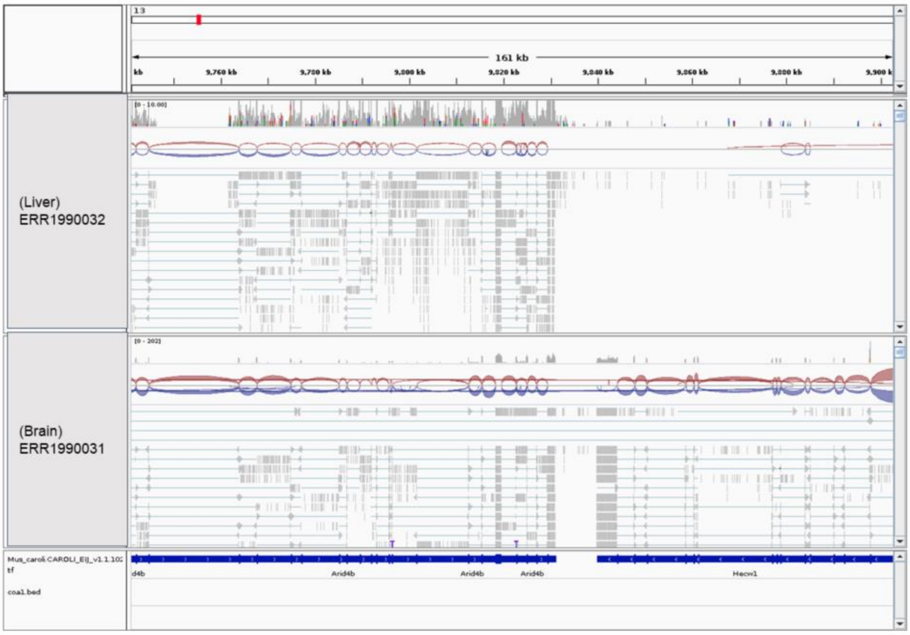

Figure S793: Screenshot of RNA-seq reads aligned each of the putative locations of *ARIDB* and *HECW1* in Ryukyu mouse (*Mus caroli*). Gray boxes represent reads aligned to the genome and thin lines connecting these boxes represent the spliced read alignments. Datasets from liver (ERR1990032) and brain (ERR1990031) have been mapped to the Mus_caroli.CAROLI_EIJ_v1.1.dna_sm.toplevel.fa genome. Locations of *ARID4B* and *HECW1* are depicted as Mus_caroli.CAROLI_EIJ_v1.1.102.gtf record in the bottom row.

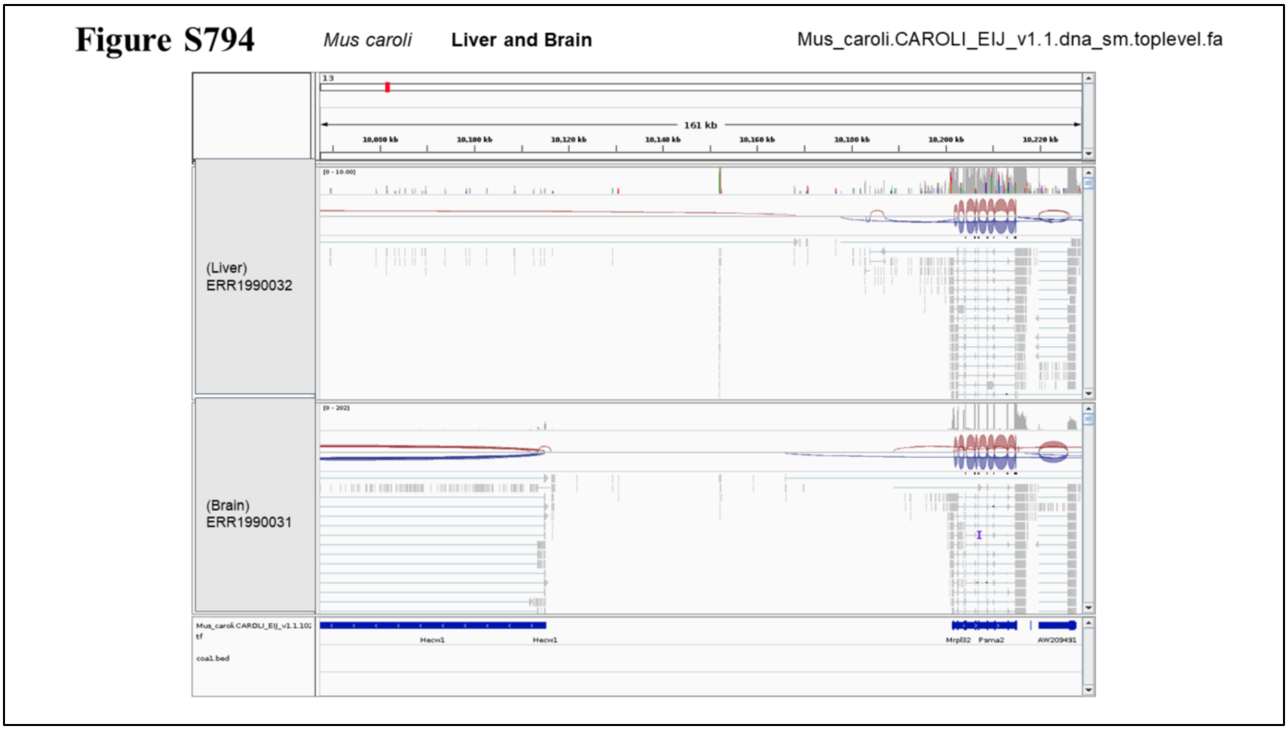

Figure S794: Screenshot of RNA-seq reads aligned each of the putative locations of *HECW1* and *MRPL32* in Ryukyu mouse (*Mus caroli*). Gray boxes represent reads aligned to the genome and thin lines connecting these boxes represent the spliced read alignments. Datasets from liver (ERR1990040) and brain (ERR1990039) have been mapped to the Mus_caroli.CAROLI_EIJ_v1.1.dna_sm.toplevel.fa genome. Locations of *HECW1* and *MRPL32* are depicted as Mus_caroli.CAROLI_EIJ_v1.1.102.gtf record in the bottom row.

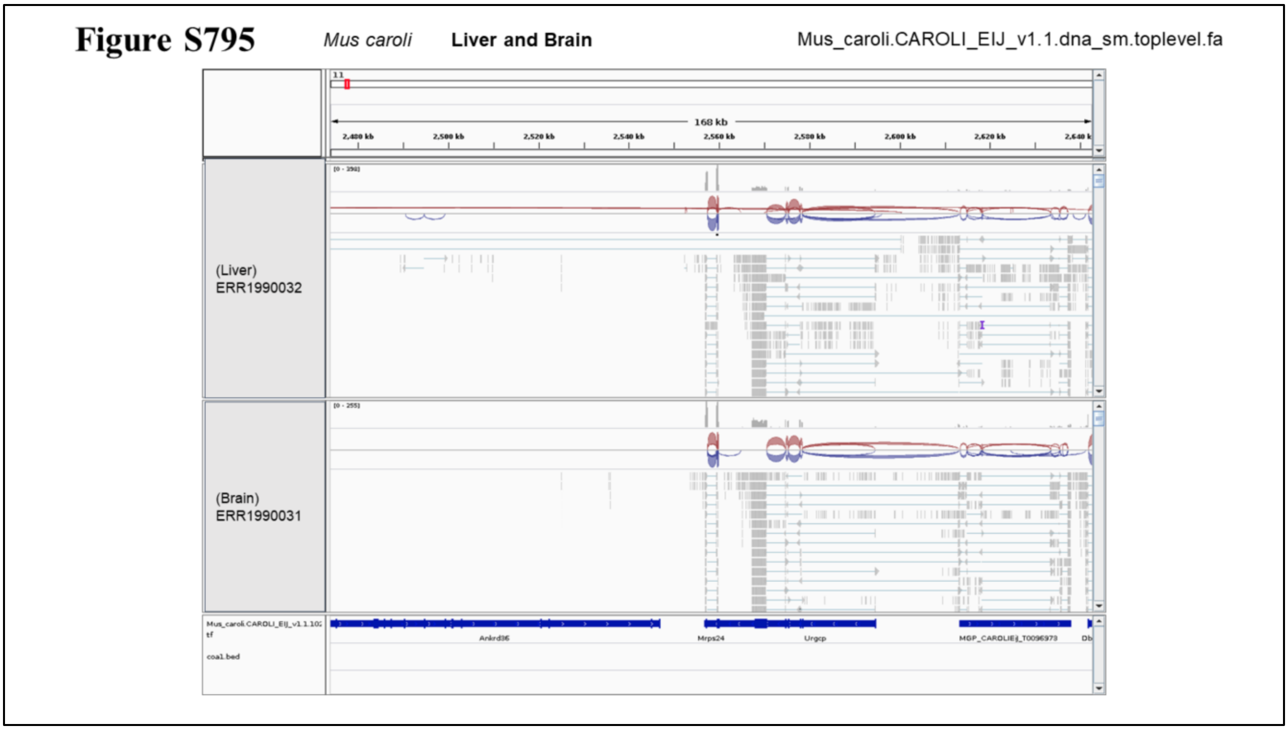

Figure S795: Screenshot of RNA-seq reads aligned each of the putative locations of *ANKRD36* and *URGCP* in Ryukyu mouse (*Mus caroli*). Gray boxes represent reads aligned to the genome and thin lines connecting these boxes represent the spliced read alignments. Datasets from liver (ERR1990040) and brain (ERR1990039) have been mapped to the Mus_caroli.CAROLI_EIJ_v1.1.dna_sm.toplevel.fa genome. Locations of *ANKRD36* and *URGCP* are depicted as Mus_caroli.CAROLI_EIJ_v1.1.102.gtf record in the bottom row.

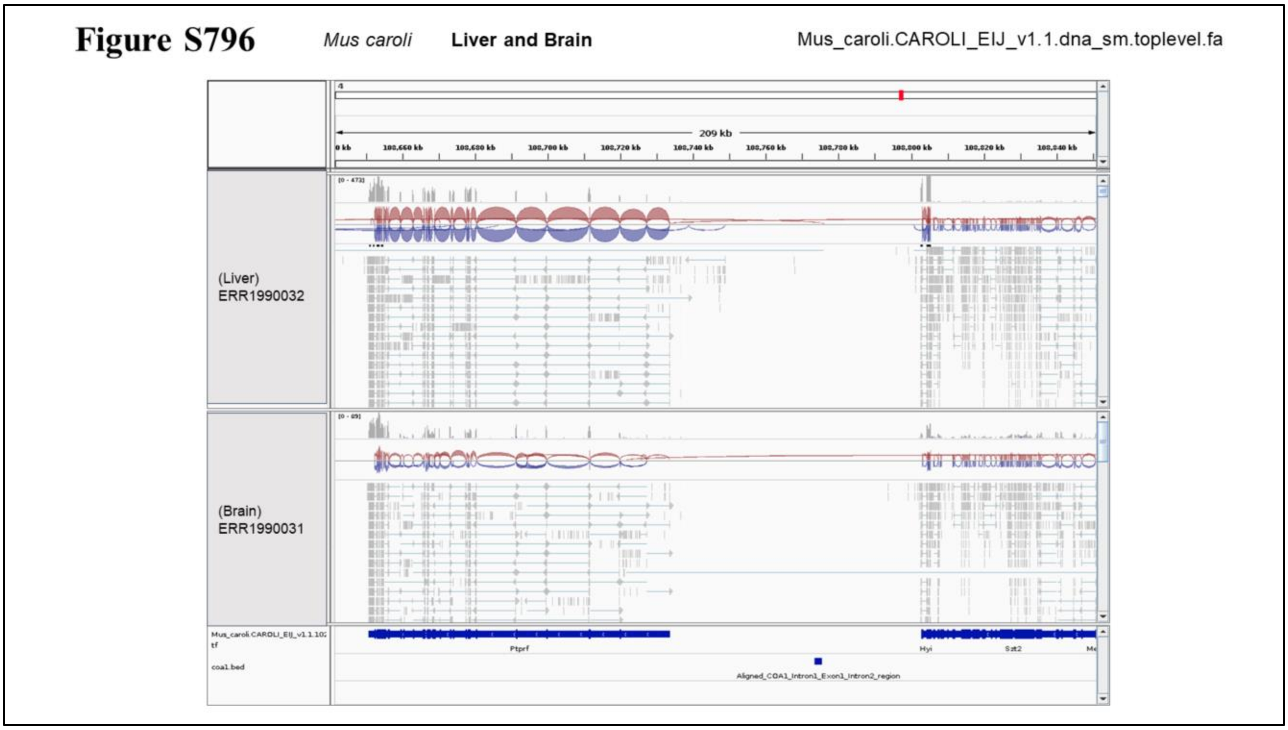

Figure S796: Screenshot of RNA-seq reads aligned each of the putative locations of truncated exon 1, intron-1 and intron-2 of *COA1* in Ryukyu mouse (*Mus caroli*). Gray boxes represent reads aligned to the genome and thin lines connecting these boxes represent the spliced read alignments. Datasets from liver (ERR1990040) and brain (ERR1990039) have been mapped to the Mus_caroli.CAROLI_EIJ_v1.1.dna_sm.toplevel.fa genome. Locations of truncated exon 1, intron-1 and intron-2 of *COA1* is depicted as bed record in the bottom row.

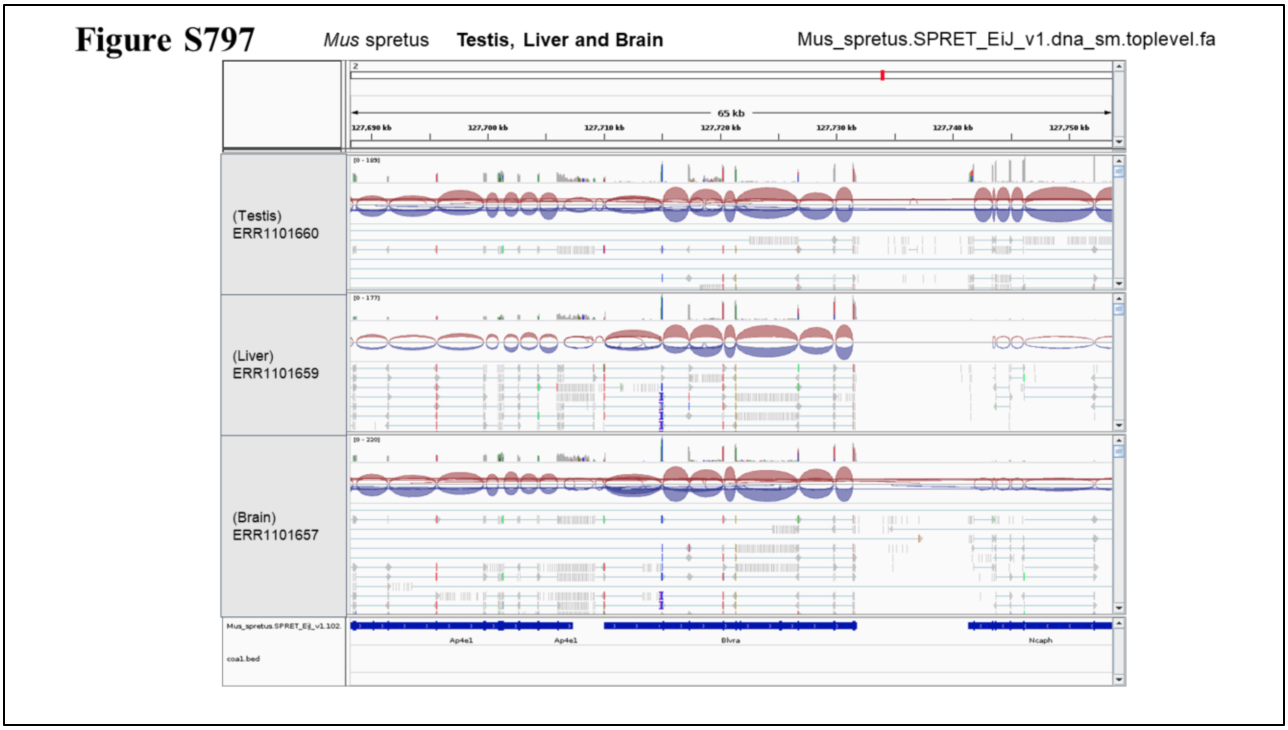

Figure S797: Screenshot of RNA-seq reads aligned each of the putative locations of *AP4E1* and *NCAPH* in Algerian mouse (*Mus spretus*). Gray boxes represent reads aligned to the genome and thin lines connecting these boxes represent the spliced read alignments. Datasets from testis (ERR1101660), liver (ERR1101659) and brain (ERR1101657) have been mapped to the Mus_spretus.SPRET_EiJ_v1.dna_sm.toplevel.fa. Locations of *AP4E1* and *NCAPH* are depicted as Mus_spretus.SPRET_EiJ_v1.102.gtf record in the bottom row.

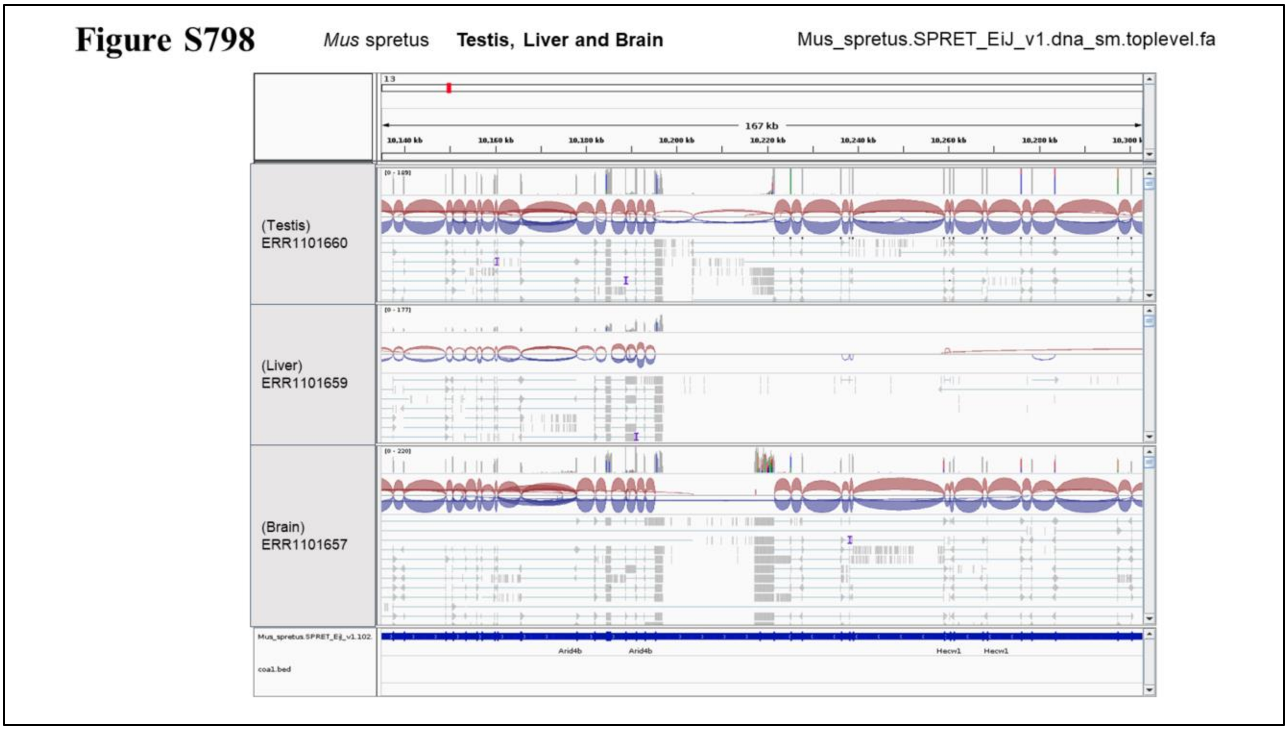

Figure S798: Screenshot of RNA-seq reads aligned each of the putative locations of *ARID4B* and *HECW1* in Algerian mouse (*Mus spretus*). Gray boxes represent reads aligned to the genome and thin lines connecting these boxes represent the spliced read alignments. Datasets from testis (ERR1101660), liver (ERR1101659) and brain (ERR1101657) have been mapped to the Mus_spretus.SPRET_EiJ_v1.dna_sm.toplevel.fa. Locations of *ARID4B* and *HECW1* are depicted as Mus_spretus.SPRET_EiJ_v1.102.gtf record in the bottom row.

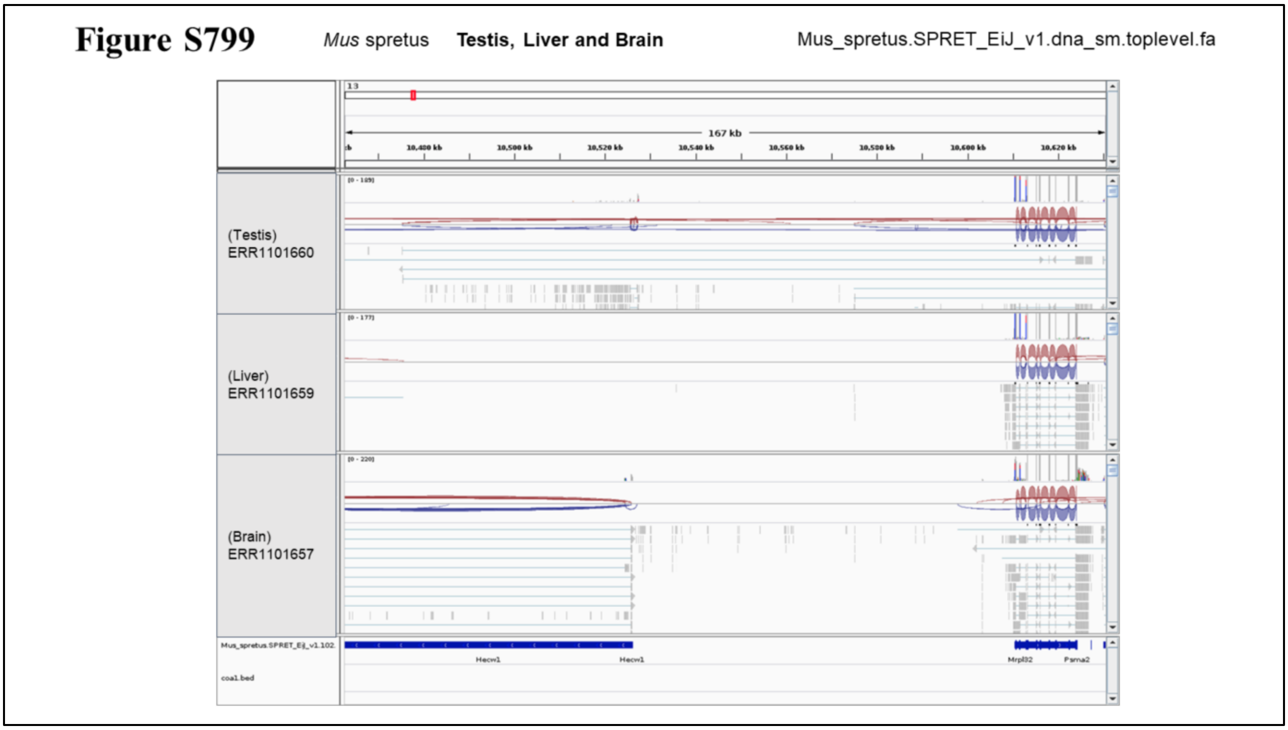

Figure S799: Screenshot of RNA-seq reads aligned each of the putative locations of *HECW1* and *MRPL32* in Algerian mouse (*Mus spretus*). Gray boxes represent reads aligned to the genome and thin lines connecting these boxes represent the spliced read alignments. Datasets from testis (ERR1101660), liver (ERR1101659) and brain (ERR1101657) have been mapped to the Mus_spretus.SPRET_EiJ_v1.dna_sm.toplevel.fa. Locations of *HECW1* and *MRPL32* are depicted as Mus_spretus.SPRET_EiJ_v1.102.gtf record in the bottom row.

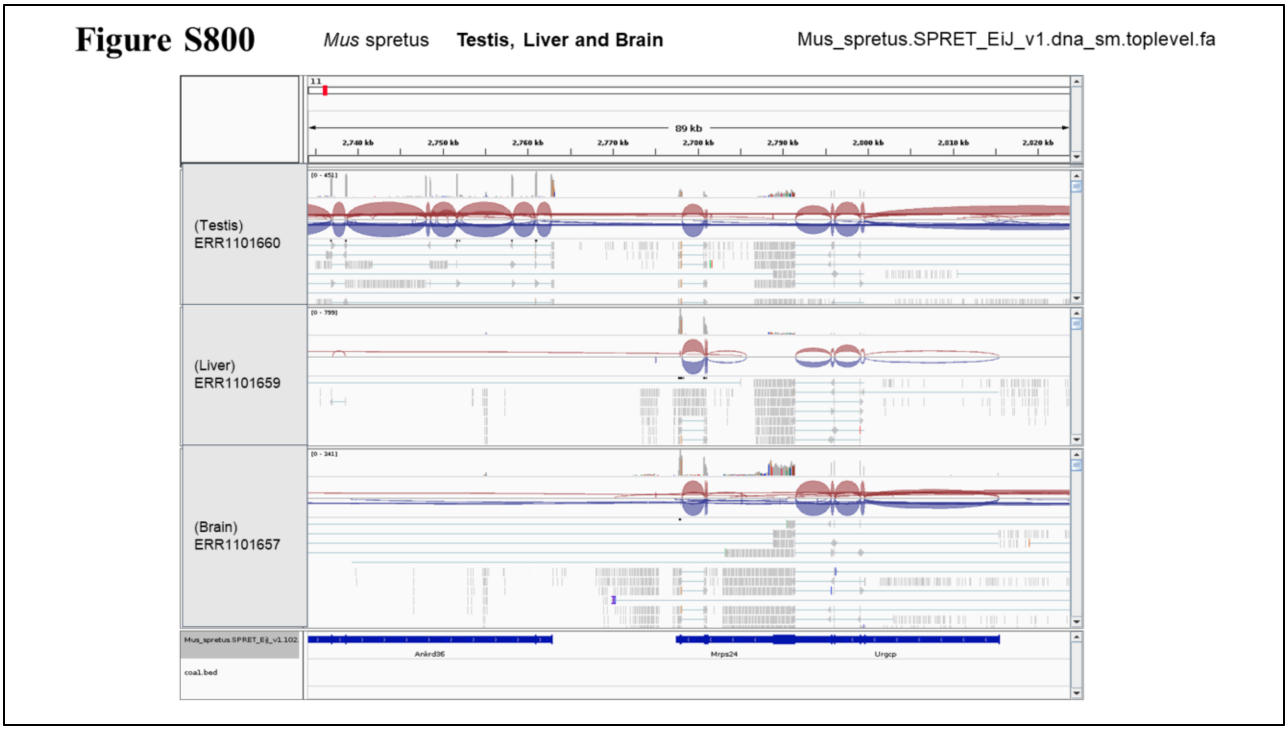

Figure S800: Screenshot of RNA-seq reads aligned each of the putative locations of *ANKRD36* and *URGCP* in Algerian mouse (*Mus spretus*). Gray boxes represent reads aligned to the genome and thin lines connecting these boxes represent the spliced read alignments. Datasets from testis (ERR1101660), liver (ERR1101659) and brain (ERR1101657) have been mapped to the Mus_spretus.SPRET_EiJ_v1.dna_sm.toplevel.fa. Locations of *ANKRD36* and *URGCP* are depicted as Mus_spretus.SPRET_EiJ_v1.102.gtf record in the bottom row.

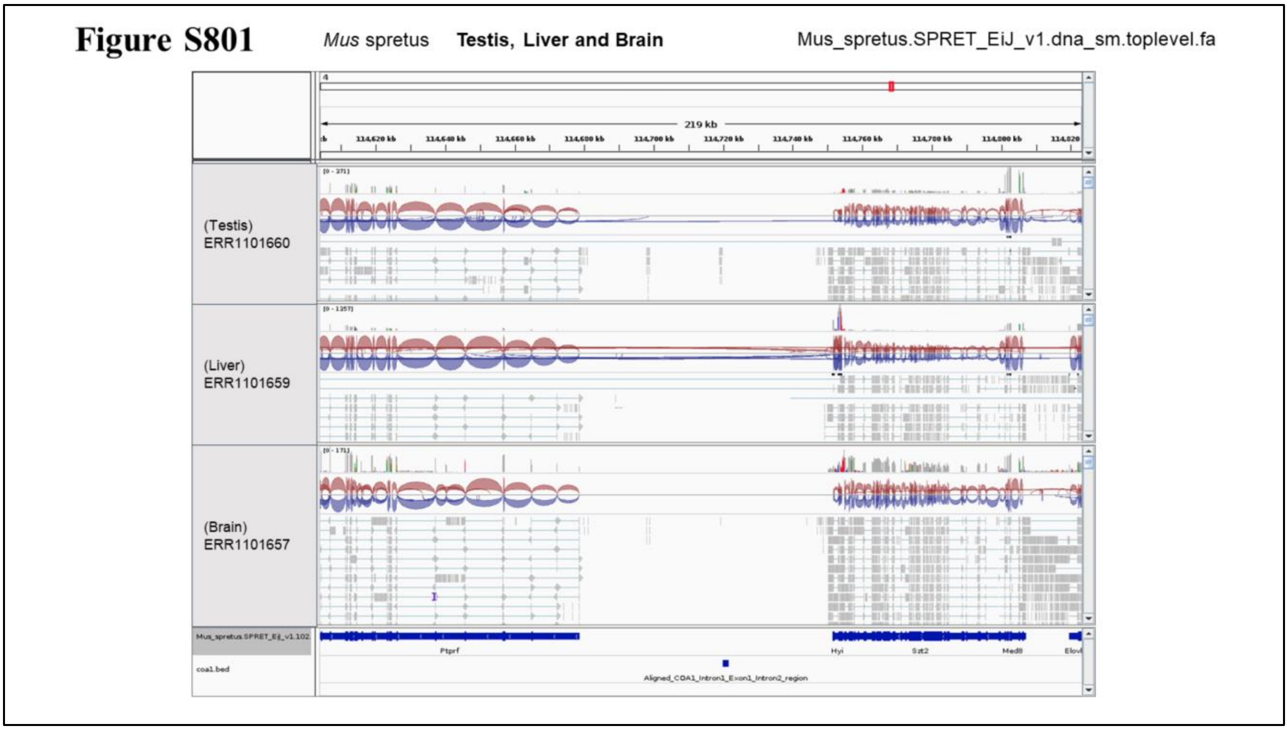

Figure S801: Screenshot of RNA-seq reads aligned each of the putative locations of truncated exon 1, intron-1 and intron-2 of *COA1* in Algerian mouse (*Mus spretus*). Gray boxes represent reads aligned to the genome and thin lines connecting these boxes represent the spliced read alignments. Datasets from testis (ERR1101660), liver (ERR1101659) and brain (ERR1101657) have been mapped to the Mus_spretus.SPRET_EiJ_v1.dna_sm.toplevel.fa. Locations of truncated exon 1, intron-1 and intron-2 of *COA1* is depicted as bed record in the bottom row.

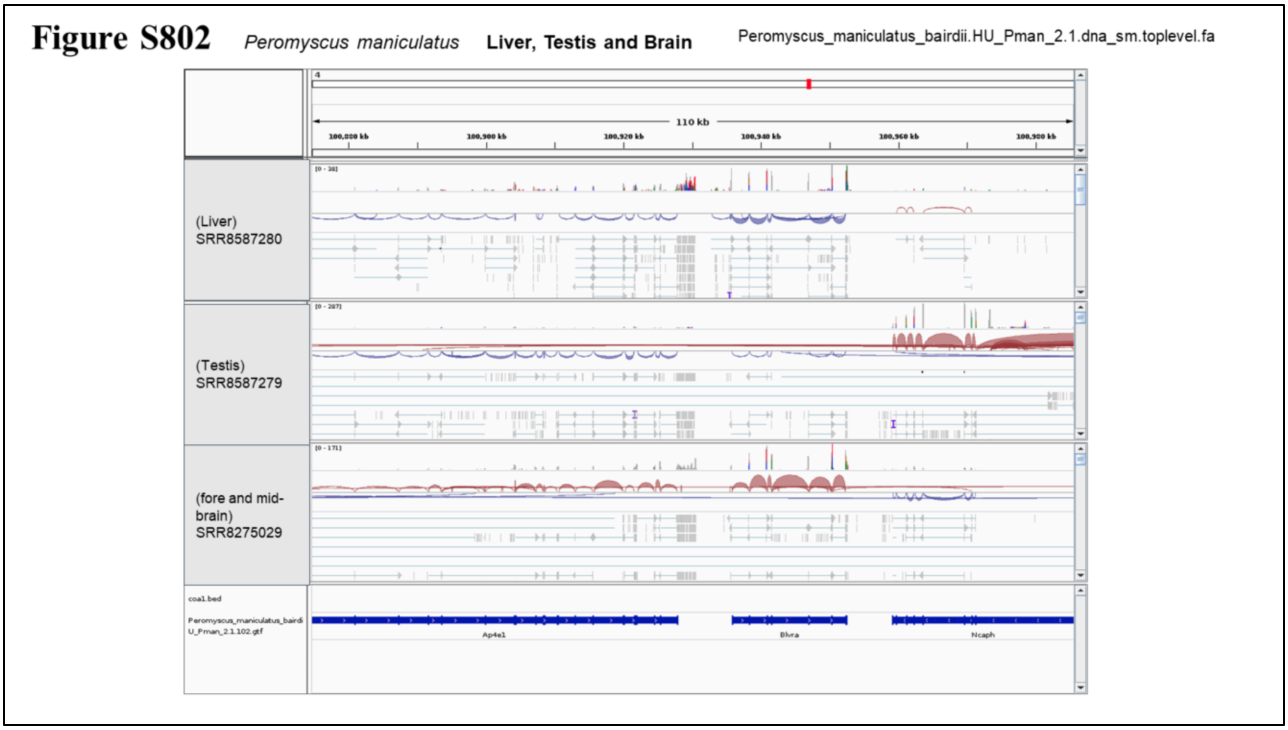

Figure S802: Screenshot of RNA-seq reads aligned each of the putative locations of AP4E1 and NCAPH in Northern American deer mouse (Peromyscus maniculatus). Gray boxes represent reads aligned to the genome and thin lines connecting these boxes represent the spliced read alignments. Datasets from liver (SRR8587280), testis (SRR8587279) and fore and mid-brain (SRR8275029) have been mapped to the Peromyscus_maniculatus_bairdii.HU_Pman_2.1.dna_sm.toplevel.fa. Locations of AP4E1 and NCAPH are depicted as Peromyscus_maniculatus_bairdii.HU_Pman_2.1.102.gtf record in the bottom row.

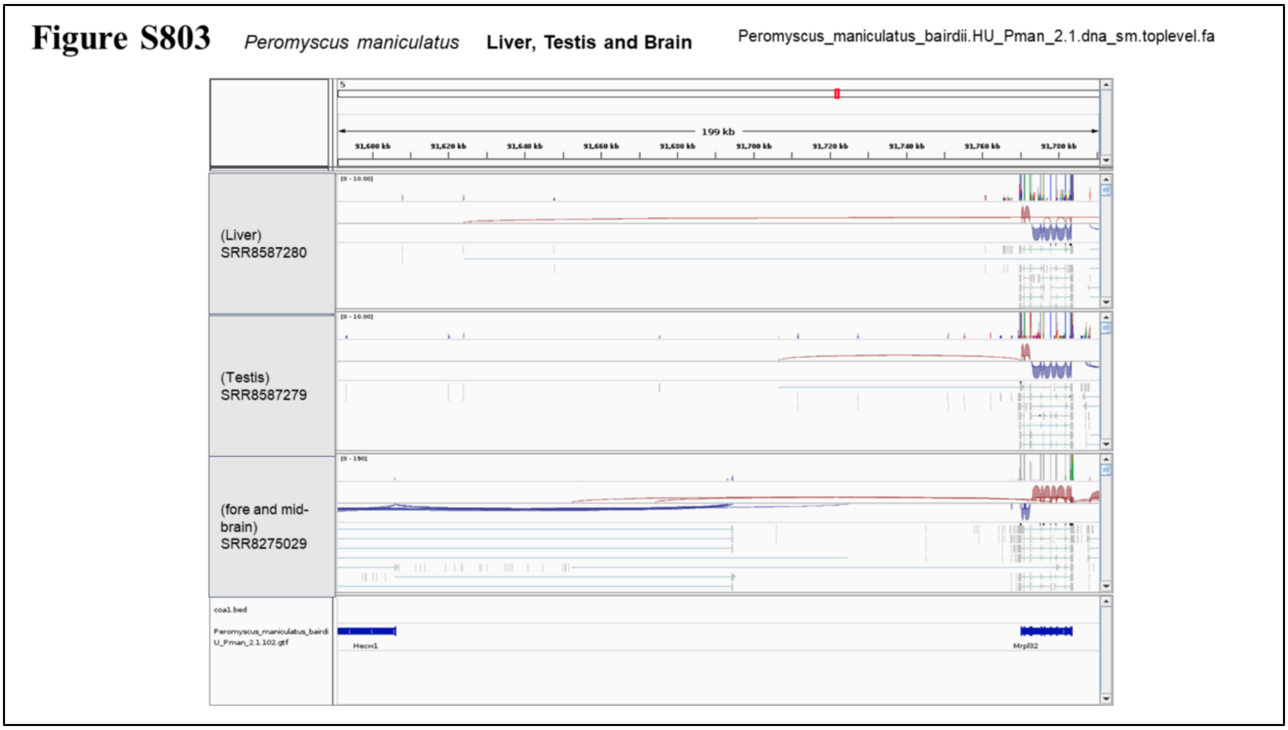

Figure S803: Screenshot of RNA-seq reads aligned each of the putative locations of HECW1 and MRPL32 in Northern American deer mouse (Peromyscus maniculatus). Gray boxes represent reads aligned to the genome and thin lines connecting these boxes represent the spliced read alignments. Datasets from testis (ERR1101660), liver (ERR1101659) and brain (ERR1101657) have been mapped to the Peromyscus_maniculatus_bairdii.HU_Pman_2.1.dna_sm.toplevel.fa. Locations of HECW1 and MRPL32 are depicted as Peromyscus_maniculatus_bairdii.HU_Pman_2.1.102.gtf record in the bottom row.

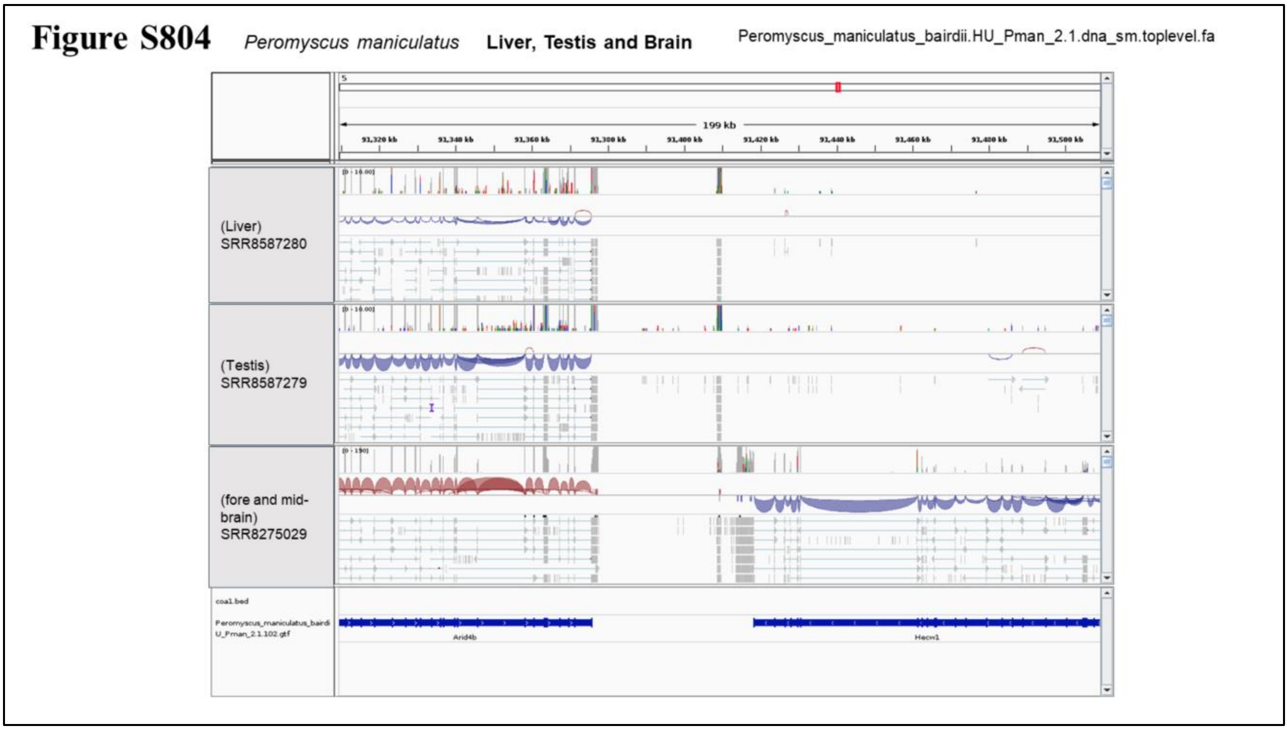

Figure S804: Screenshot of RNA-seq reads aligned each of the putative locations of ARID4B and HECW1 in Northern American deer mouse (Peromyscus maniculatus). Gray boxes represent reads aligned to the genome and thin lines connecting these boxes represent the spliced read alignments. Datasets from testis (ERR1101660), liver (ERR1101659) and brain (ERR1101657) have been mapped to the Peromyscus_maniculatus_bairdii.HU_Pman_2.1.dna_sm.toplevel.fa. Locations of ARID4B and HECW1 are depicted as Peromyscus_maniculatus_bairdii.HU_Pman_2.1.102.gtf record in the bottom row.

Figure S805 *Peromyscus maniculatus* **Liver, Testis and Brain** Peromyscus_maniculatus_bairdii.HU_Pman_2.1.dna_sm.toplevel.fa

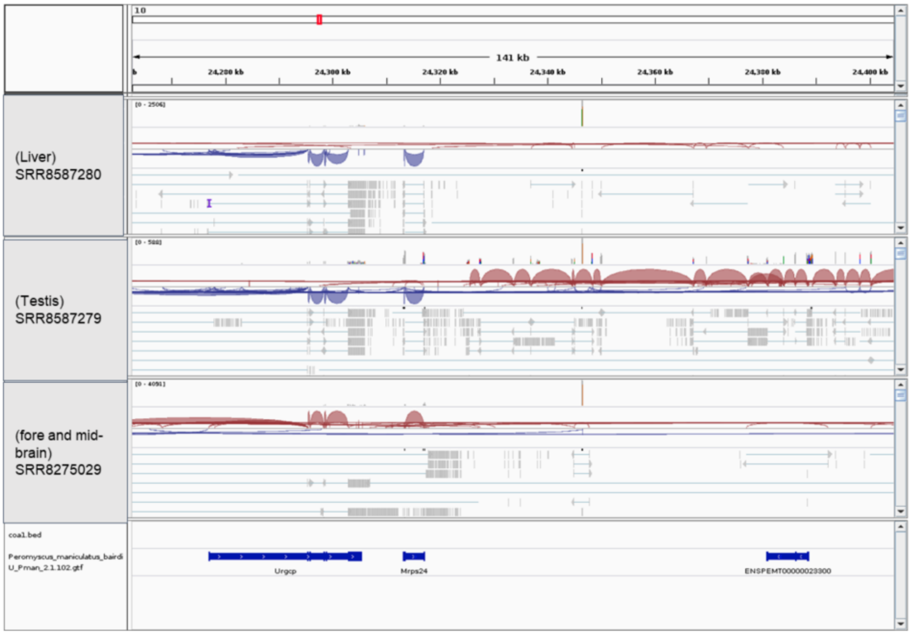

Figure S805: Screenshot of RNA-seq reads aligned each of the putative locations of URGCP and ANKRD36 in Northern American deer mouse (Peromyscus maniculatus). Gray boxes represent reads aligned to the genome and thin lines connecting these boxes represent the spliced read alignments. Datasets from testis (ERR1101660), liver (ERR1101659) and brain (ERR1101657) have been mapped to the Peromyscus_maniculatus_bairdii.HU_Pman_2.1.dna_sm.toplevel.fa. Locations of URGCP and ANKRD36 are depicted as Peromyscus_maniculatus_bairdii.HU_Pman_2.1.102.gtf record in the bottom row.

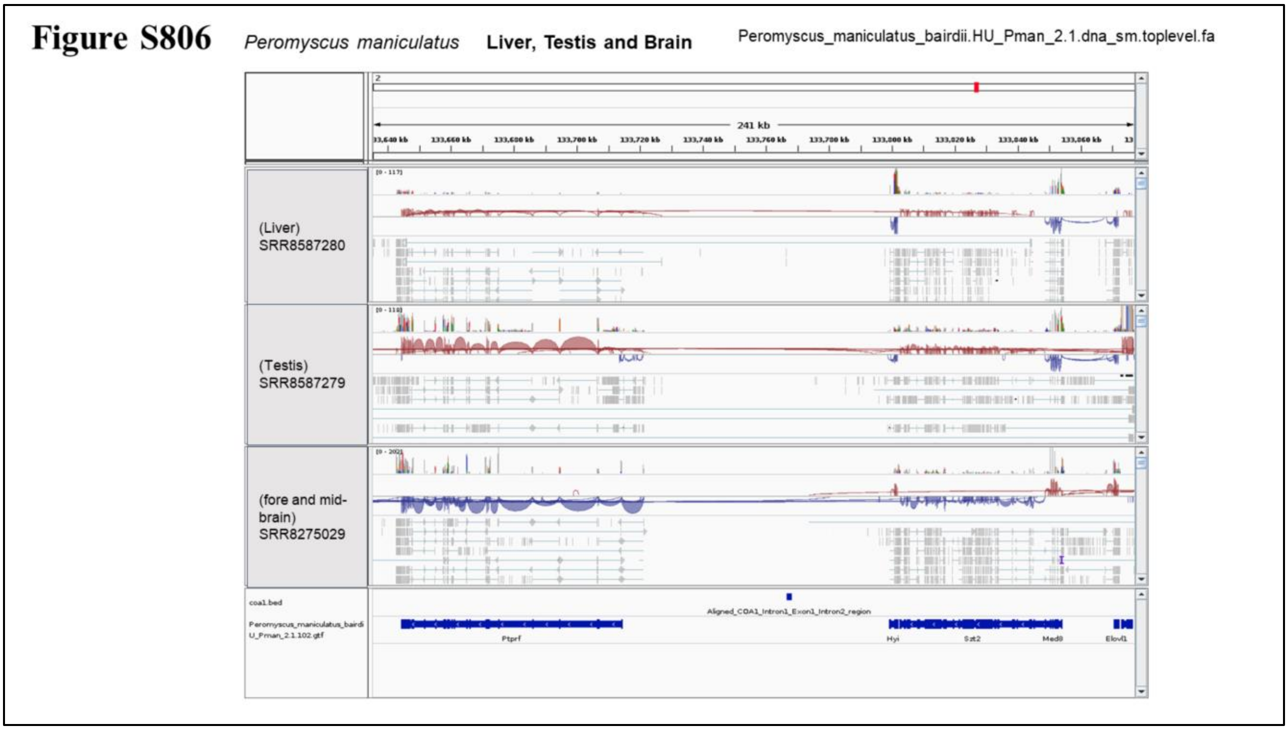

Figure S806: Screenshot of RNA-seq reads aligned each of the putative locations of truncated exon 1 and intron-1 of *COA1* gene in Northern American deer mouse (Peromyscus maniculatus). Gray boxes represent reads aligned to the genome and thin lines connecting these boxes represent the spliced read alignments. Datasets from testis (ERR1101660), liver (ERR1101659) and brain (ERR1101657) have been mapped to the Peromyscus_maniculatus_bairdii.HU_Pman_2.1.dna_sm.toplevel.fa. Locations of truncated exon 1 and intron-1 of *COA1* gene is depicted as bed record in the bottom row.

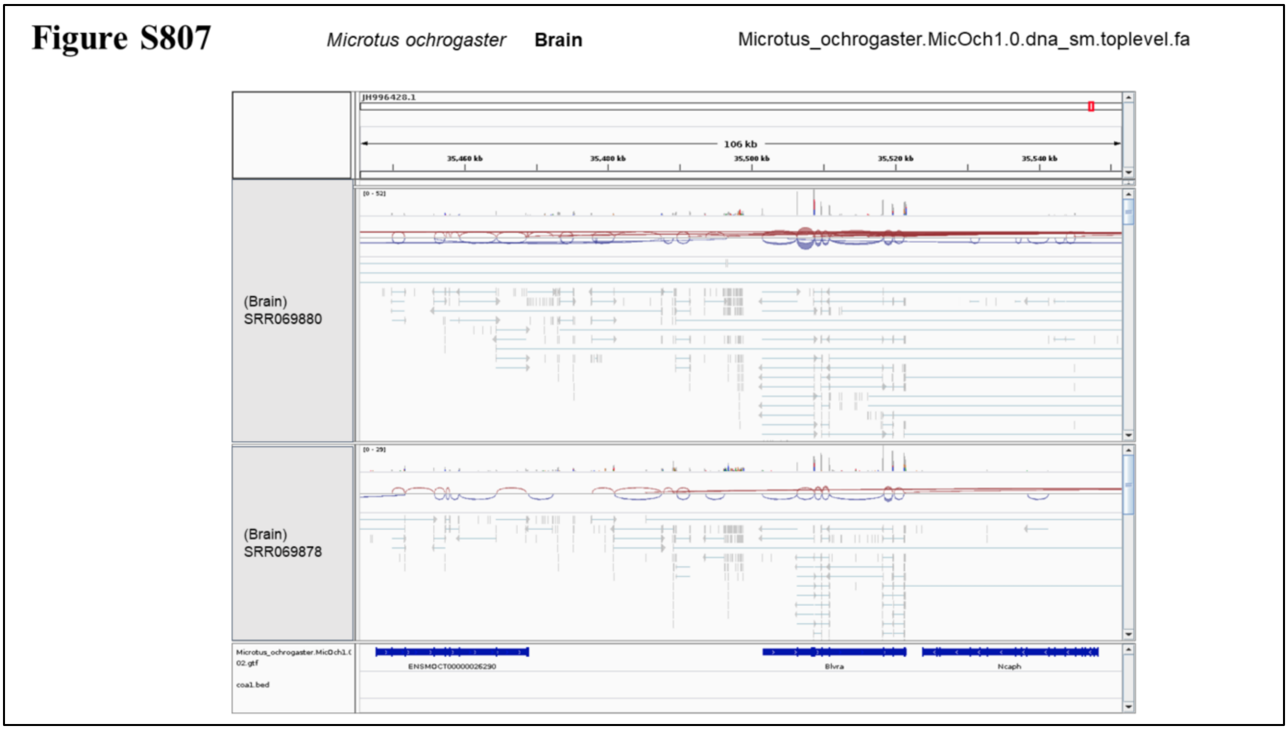

Figure S807: Screenshot of RNA-seq reads aligned each of the putative locations of *AP4E1* and *NCAPH* in Prairie vole (*Microtus ochrogaster*). Gray boxes represent reads aligned to the genome and thin lines connecting these boxes represent the spliced read alignments. Datasets from brain (SRR069880, SRR069878) have been mapped to the Microtus_ochrogaster.MicOch1.0.dna_sm.toplevel.fa. Locations of *AP4E1* and *NCAPH* are depicted as Microtus_ochrogaster.MicOch1.0.102.gtf record in the bottom row.

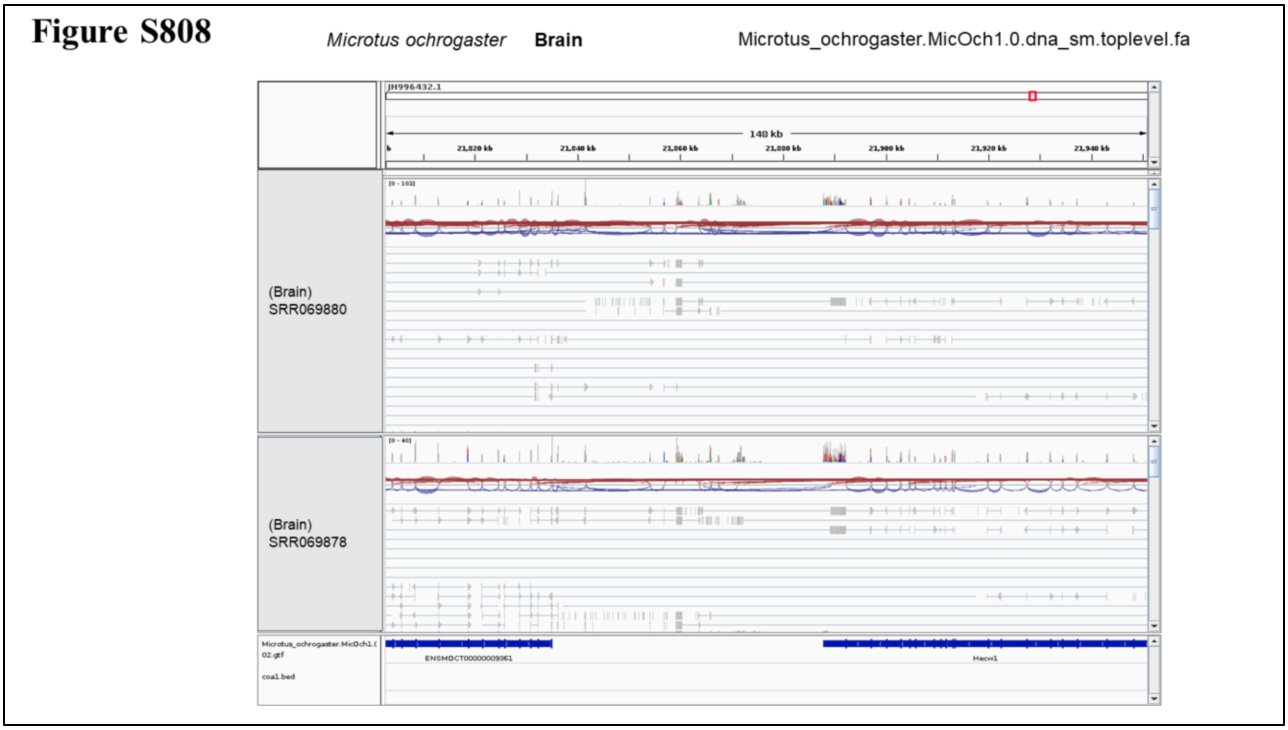

Figure S808: Screenshot of RNA-seq reads aligned each of the putative locations of *ARID4B* and *HECW1* in Prairie vole (*Microtus ochrogaster*). Gray boxes represent reads aligned to the genome and thin lines connecting these boxes represent the spliced read alignments. Datasets from brain (SRR069880, SRR069878) have been mapped to the Microtus_ochrogaster.MicOch1.0.dna_sm.toplevel.fa. Locations of *ARID4B* and *HECW1* are depicted as Microtus_ochrogaster.MicOch1.0.102.gtf record in the bottom row.

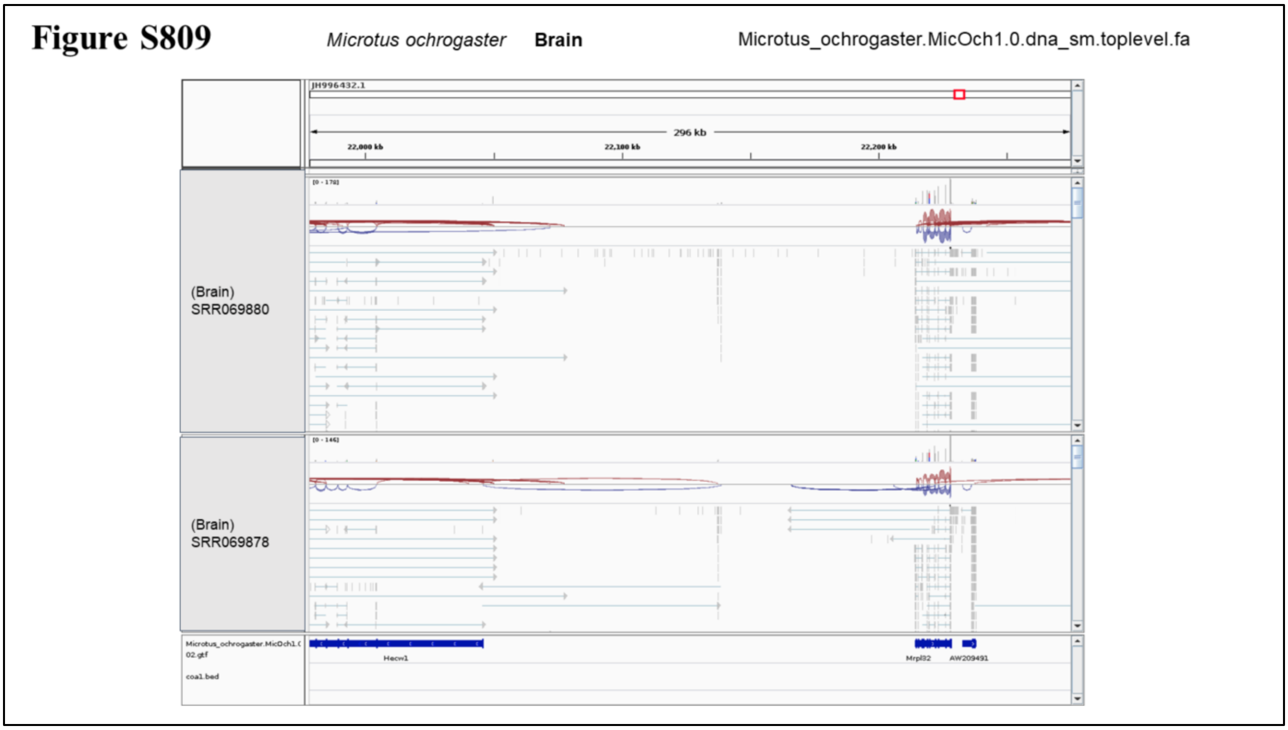

Figure S809: Screenshot of RNA-seq reads aligned each of the putative locations of *MRPL32* and *HECW1* in Prairie vole (*Microtus ochrogaster*). Gray boxes represent reads aligned to the genome and thin lines connecting these boxes represent the spliced read alignments. Datasets from brain (SRR069880, SRR069878) have been mapped to the Microtus_ochrogaster.MicOch1.0.dna_sm.toplevel.fa. Locations of *MRPL32* and *HECW1* are depicted as Microtus_ochrogaster.MicOch1.0.102.gtf record in the bottom row.

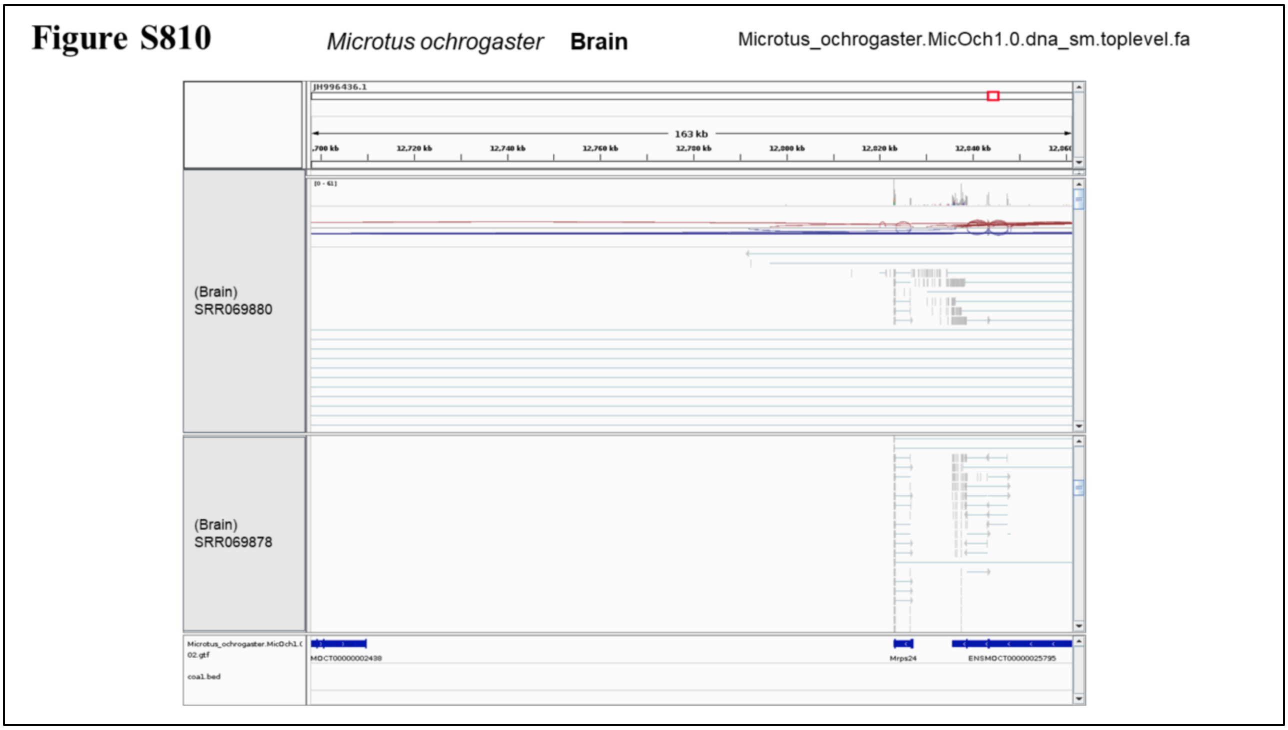

Figure S810: Screenshot of RNA-seq reads aligned each of the putative locations of *ANKRD36* and *MRPS24* in Prairie vole (*Microtus ochrogaster*). Gray boxes represent reads aligned to the genome and thin lines connecting these boxes represent the spliced read alignments. Datasets from brain (SRR069880, SRR069878) have been mapped to the Microtus_ochrogaster.MicOch1.0.dna_sm.toplevel.fa. Locations of *ANKRD36* and *MRPS24* are depicted as Microtus_ochrogaster.MicOch1.0.102.gtf record in the bottom row.

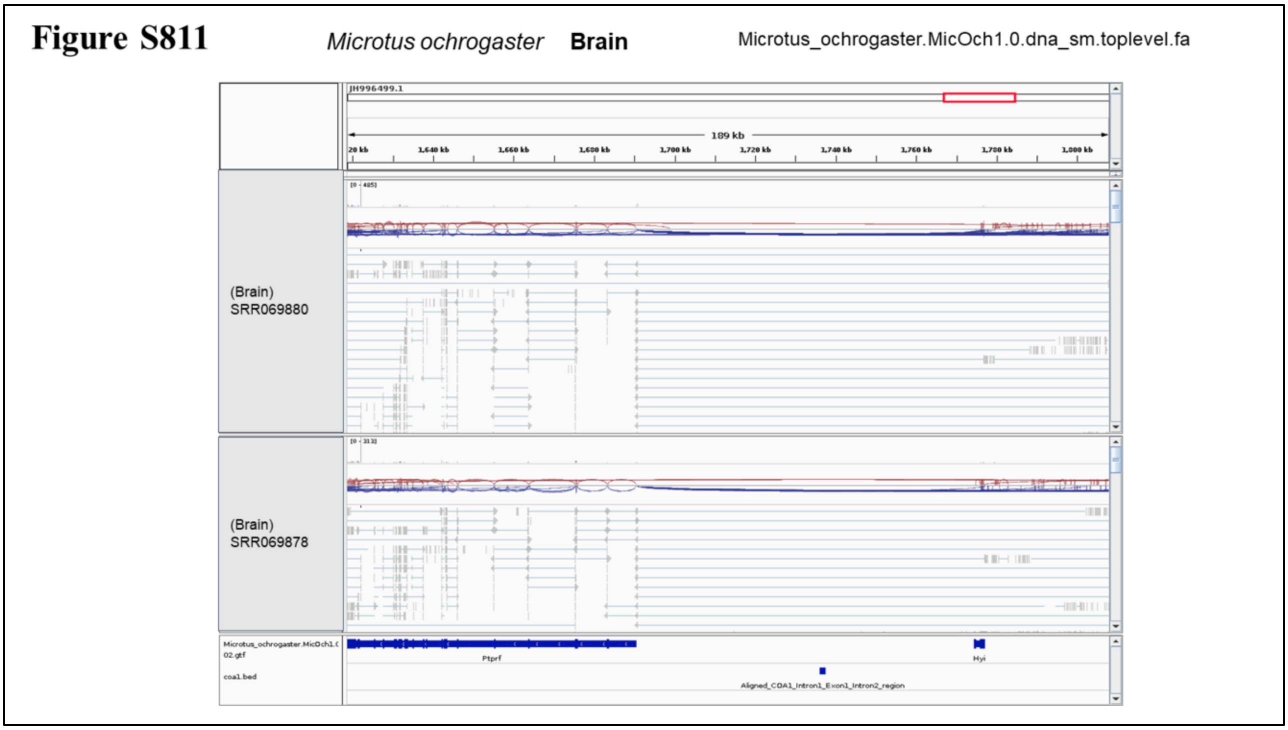

Figure S811: Screenshot of RNA-seq reads aligned each of the putative locations of truncated exon 1, intron-1 and intron-2 of *COA1* in Prairie vole (*Microtus ochrogaster*). Gray boxes represent reads aligned to the genome and thin lines connecting these boxes represent the spliced read alignments. Datasets from brain (SRR069880, SRR069878) have been mapped to the Microtus_ochrogaster.MicOch1.0.dna_sm.toplevel.fa. Locations of exon 1, intron-1 and intron-2 of *COA1* is depicted as bed record in the bottom row.

Figure S812 *Mesocricetus auratus* **Liver and Brain** Mesocricetus_auratus.MesAur1.0.dna_sm.toplevel.fa

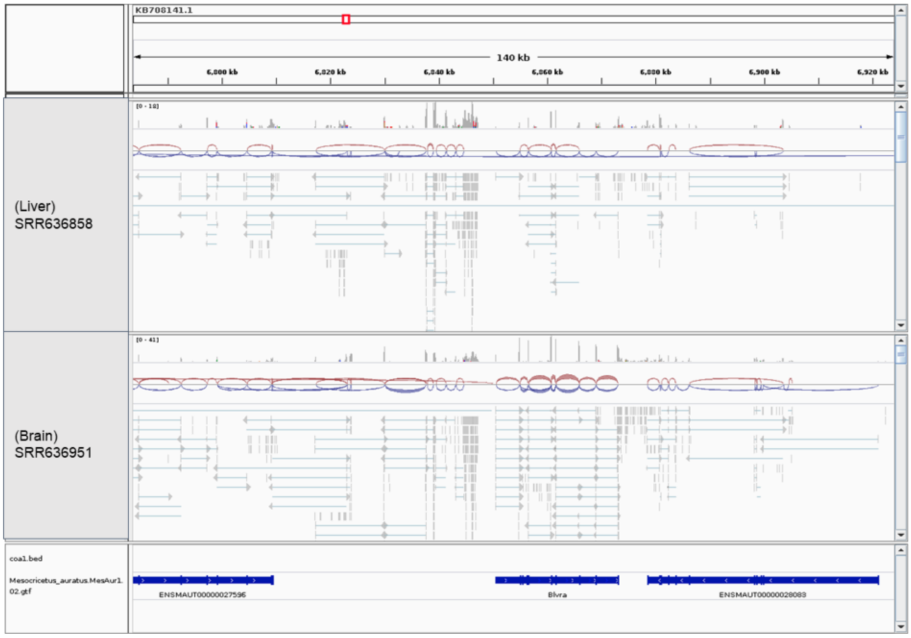

Figure S812: Screenshot of RNA-seq reads aligned each of the putative locations of *AP4E1* and *NCAPH* in golden Hamster (*Mesocricetus auratus*). Gray boxes represent reads aligned to the genome and thin lines connecting these boxes represent the spliced read alignments. Datasets from liver (SRR636858) and brain (SRR636951) have been mapped to the Mesocricetus_auratus.MesAur1.0.dna_sm.toplevel.fa. Locations of *AP4E1* and *NCAPH* are depicted as Mesocricetus_auratus.MesAur1.0.102.gtf record in the bottom row.

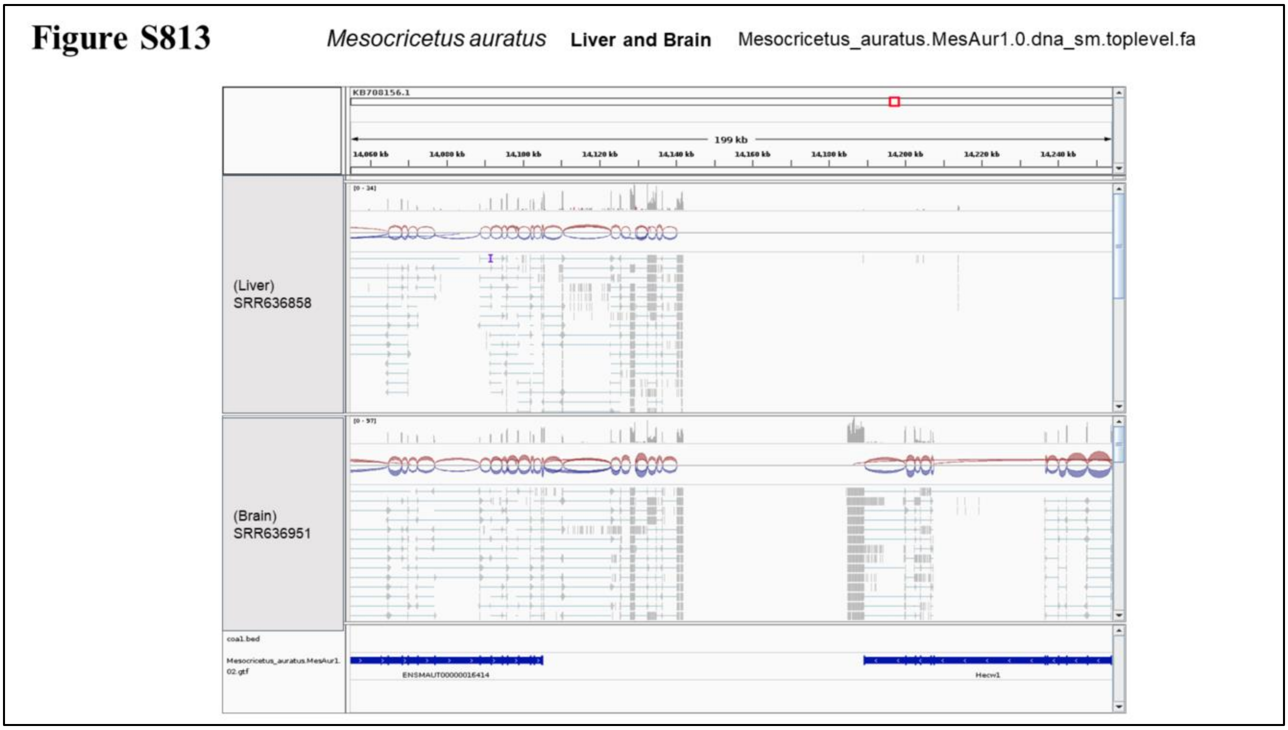

Figure S813: Screenshot of RNA-seq reads aligned each of the putative locations of *ARID4B* and *NCAPH* in golden Hamster (*Mesocricetus auratus*). Gray boxes represent reads aligned to the genome and thin lines connecting these boxes represent the spliced read alignments. Datasets from liver (SRR636858) and brain (SRR636951) have been mapped to the Mesocricetus_auratus.MesAur1.0.dna_sm.toplevel.fa. Locations of *ARID4B* and *NCAPH* are depicted as Mesocricetus_auratus.MesAur1.0.102.gtf record in the bottom row.

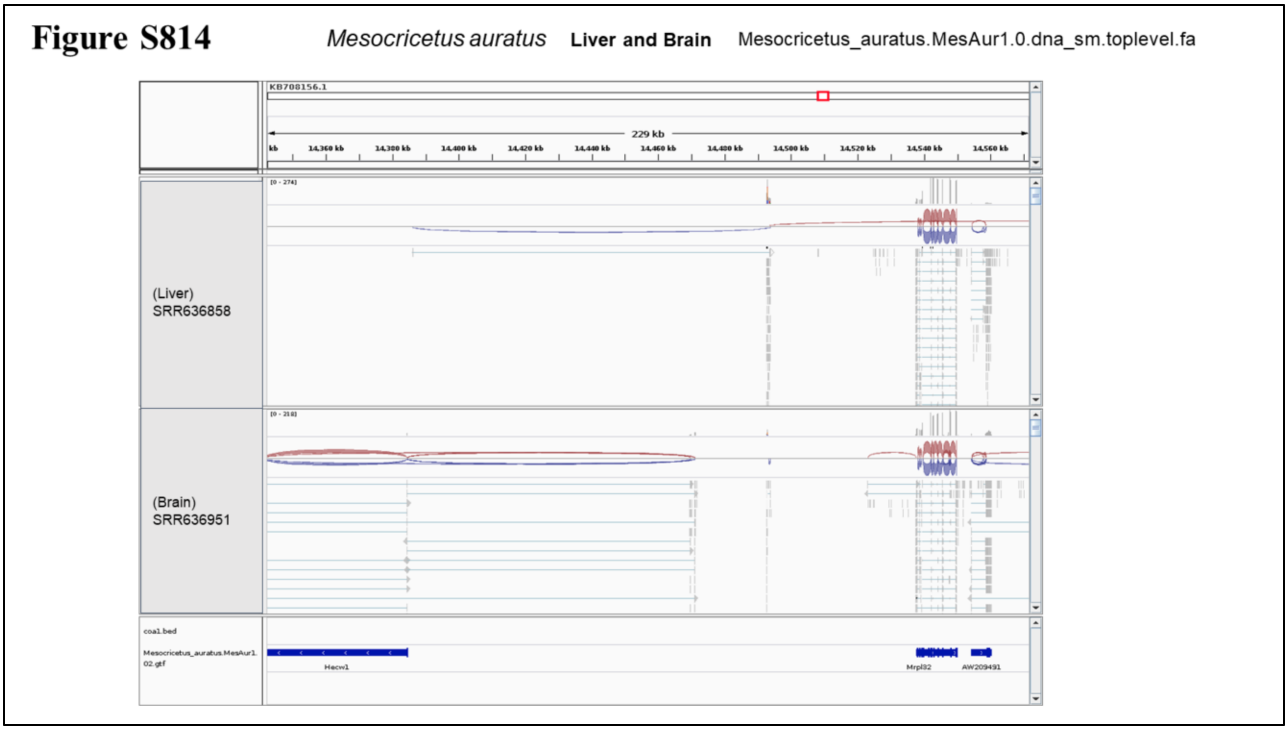

Figure S814: Screenshot of RNA-seq reads aligned each of the putative locations of *HECW1* and *MRPL32* in golden Hamster (*Mesocricetus auratus*). Gray boxes represent reads aligned to the genome and thin lines connecting these boxes represent the spliced read alignments. Datasets from liver (SRR636858) and brain (SRR636951) have been mapped to the Mesocricetus_auratus.MesAur1.0.dna_sm.toplevel.fa. Locations of *HECW1* and *MRPL32* are depicted as Mesocricetus_auratus.MesAur1.0.102.gtf record in the bottom row.

Figure S815 *Mesocricetus auratus* **Liver and Brain** Mesocricetus_auratus.MesAur1.0.dna_sm.toplevel.fa

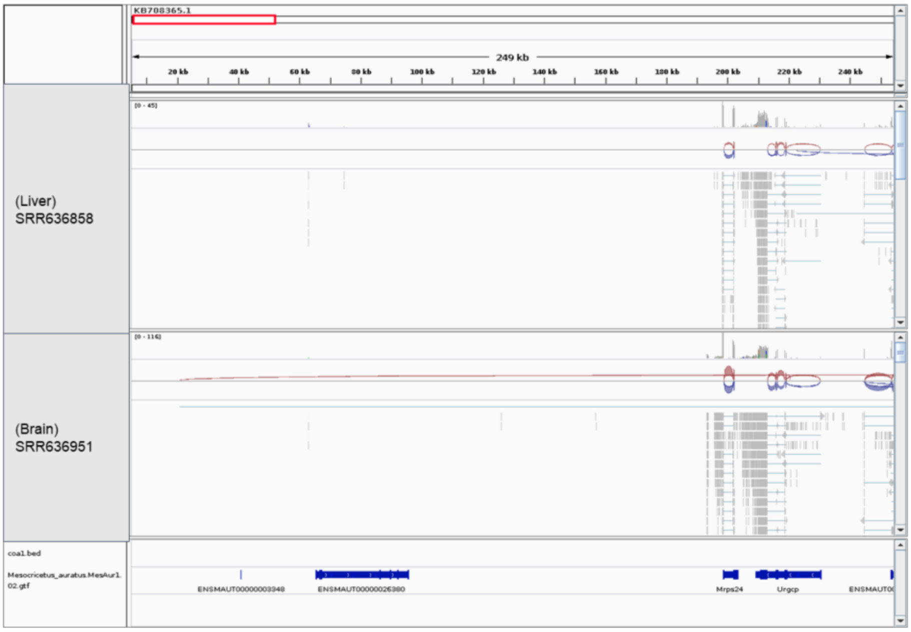

Figure S815: Screenshot of RNA-seq reads aligned each of the putative locations of *ANKRD36* and *MRPS24* in golden Hamster (*Mesocricetus auratus*). Gray boxes represent reads aligned to the genome and thin lines connecting these boxes represent the spliced read alignments. Datasets from liver (SRR636858) and brain (SRR636951) have been mapped to the Mesocricetus_auratus.MesAur1.0.dna_sm.toplevel.fa. Locations of *ANKRD36* and *MRPS24* are depicted as Mesocricetus_auratus.MesAur1.0.102.gtf record in the bottom row.

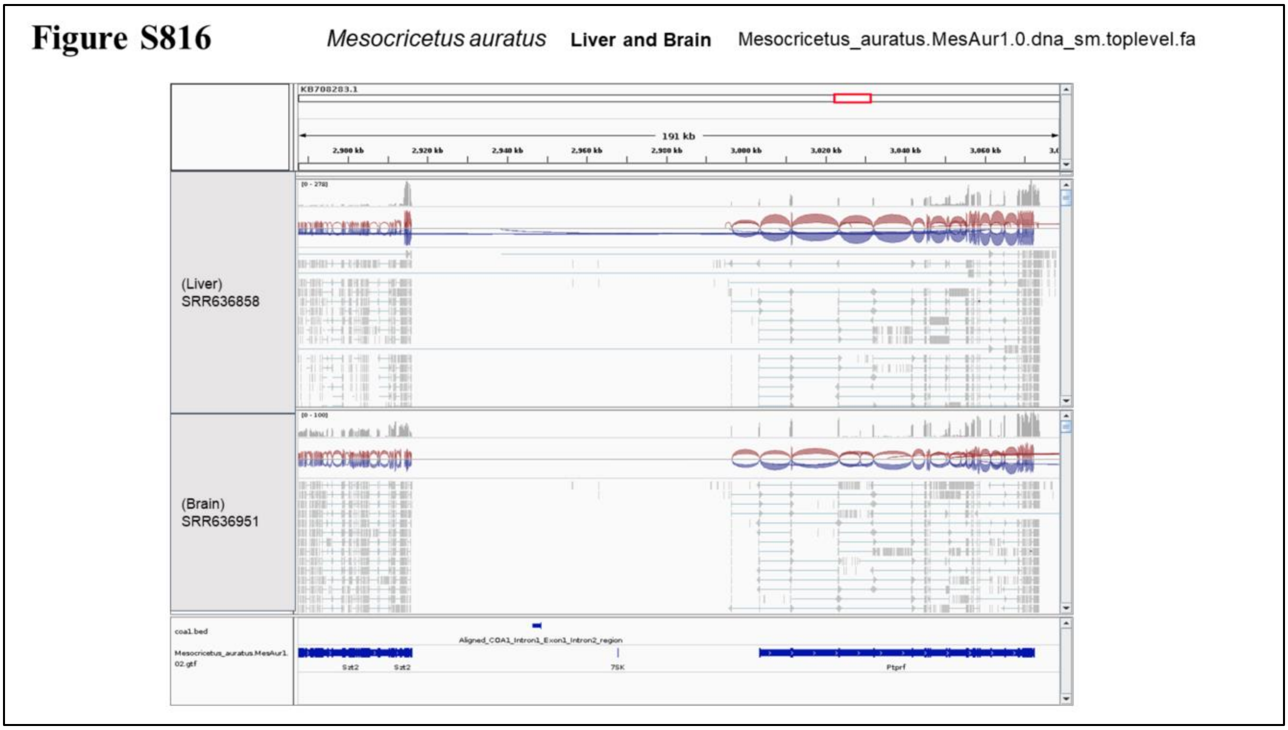

Figure S816: Screenshot of RNA-seq reads aligned each of the putative locations of truncated exon 1, intron-1 and intron-2 of *COA1* in golden Hamster (*Mesocricetus auratus*). Gray boxes represent reads aligned to the genome and thin lines connecting these boxes represent the spliced read alignments. Datasets from liver (SRR636858) and brain (SRR636951) have been mapped to the Mesocricetus_auratus.MesAur1.0.dna_sm.toplevel.fa. Locations of truncated exon 1, intron-1 and intron-2 of *COA1* is depicted as bed record in the bottom row.

Figure S817 *Meriones unguiculatus* **Spleen and Liver**Meriones_unguiculatus.MunDraft-v1.0.dna_sm.toplevel.fa

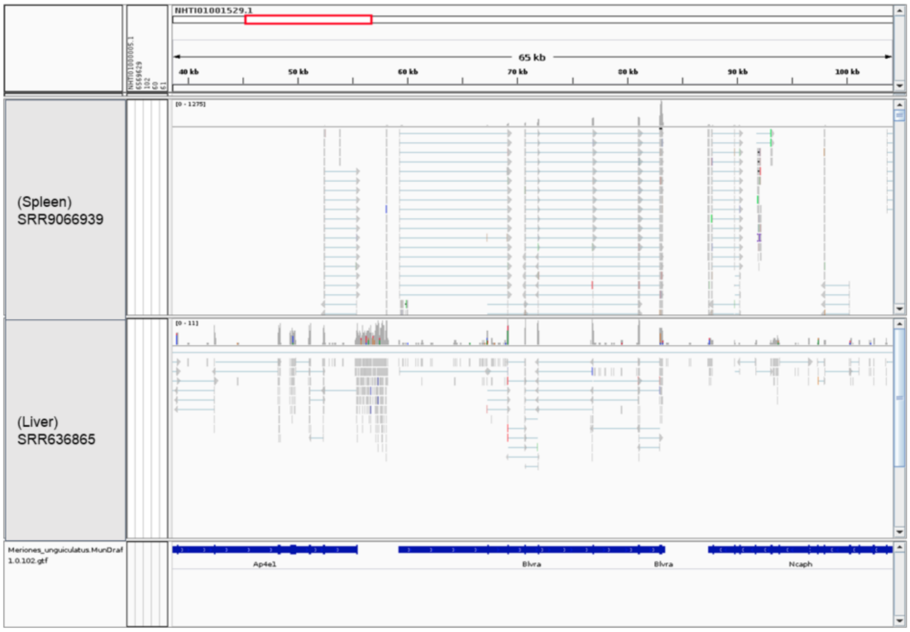

Figure S817: Screenshot of RNA-seq reads aligned each of the putative locations of *AP4E1* and *NCAPH* in Mongolian gerbil (*Meriones unguiculatus*). Gray boxes represent reads aligned to the genome and thin lines connecting these boxes represent the spliced read alignments. Datasets from spleen (SRR9066939) and liver (SRR636865) have been mapped to the Meriones_unguiculatus.MunDraft-v1.0.dna_sm.toplevel.fa. Locations of *AP4E1* and *NCAPH* are depicted as Meriones_unguiculatus.MunDraft-v1.0.102.gtf record in the bottom row.

Figure S818 *Meriones unguiculatus* **Spleen and Liver**Meriones_unguiculatus.MunDraft-v1.0.dna_sm.toplevel.fa

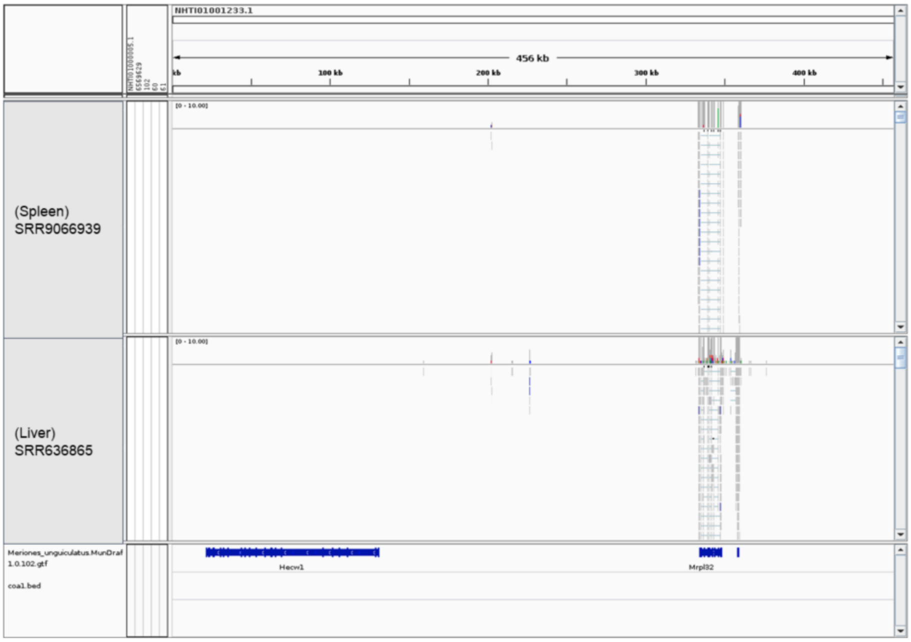

Figure S818: Screenshot of RNA-seq reads aligned each of the putative locations of *HECW1* and *MRPL32* in Mongolian gerbil (*Meriones unguiculatus*). Gray boxes represent reads aligned to the genome and thin lines connecting these boxes represent the spliced read alignments. Datasets from spleen (SRR9066939) and liver (SRR636865) have been mapped to the Meriones_unguiculatus.MunDraft-v1.0.dna_sm.toplevel.fa. Locations of *HECW1* and *MRPL32* are depicted as Meriones_unguiculatus.MunDraft-v1.0.102.gtf record in the bottom row.

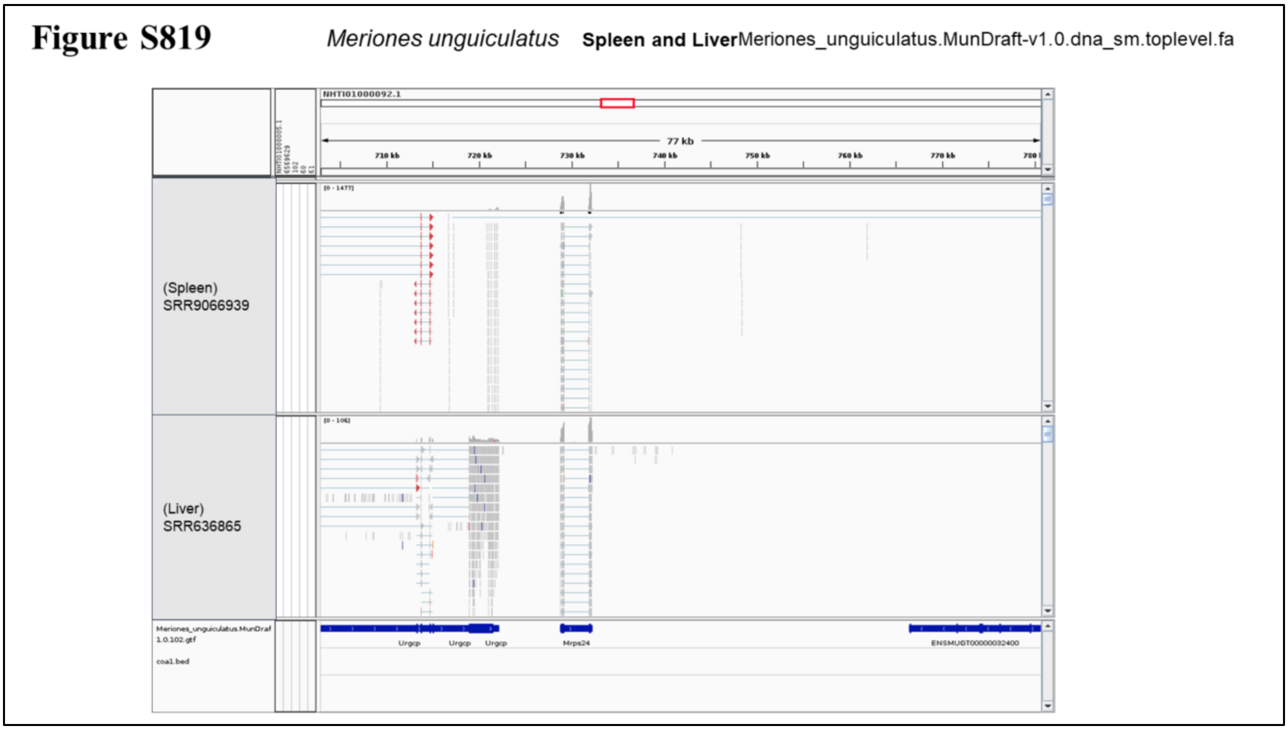

Figure S819: Screenshot of RNA-seq reads aligned each of the putative locations of *ANKRD36* and *MRPL32* in Mongolian gerbil (*Meriones unguiculatus*). Gray boxes represent reads aligned to the genome and thin lines connecting these boxes represent the spliced read alignments. Datasets from spleen (SRR9066939) and liver (SRR636865) have been mapped to the Meriones_unguiculatus.MunDraft-v1.0.dna_sm.toplevel.fa. Locations of *ANKRD36* and *MRPL32* are depicted as Meriones_unguiculatus.MunDraft-v1.0.102.gtf record in the bottom row.

Figure S820 *Meriones unguiculatus* **Spleen and Liver**Meriones_unguiculatus.MunDraft-v1.0.dna_sm.toplevel.fa

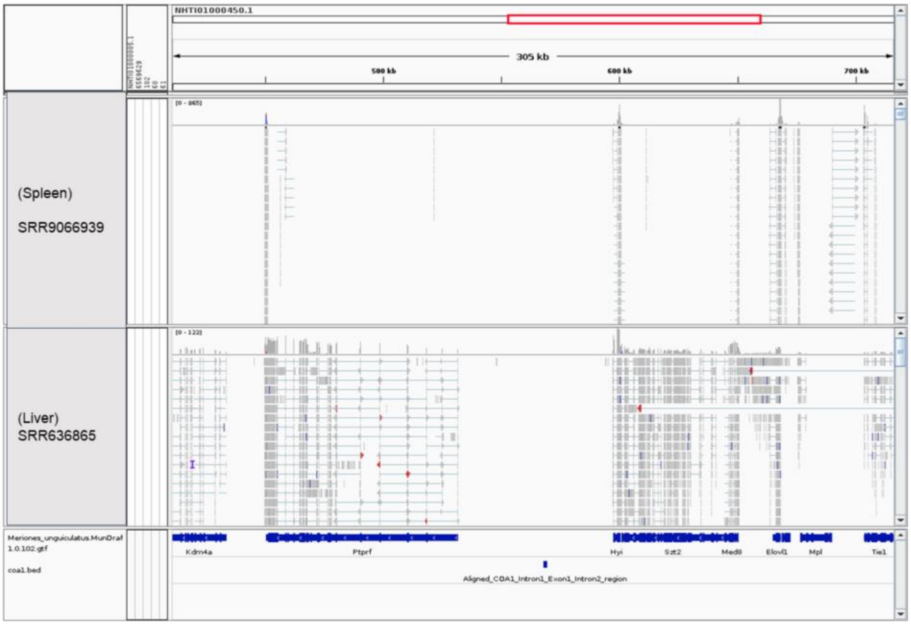

Figure S820: Screenshot of RNA-seq reads aligned each of the putative locations of truncated exon 1, intron-1 and intron-2 of *COA1* in Mongolian gerbil (*Meriones unguiculatus*). Gray boxes represent reads aligned to the genome and thin lines connecting these boxes represent the spliced read alignments. Datasets from spleen (SRR9066939) and liver (SRR636865) have been mapped to the Meriones_unguiculatus.MunDraft-v1.0.dna_sm.toplevel.fa. Locations of truncated exon 1, intron-1 and intron-2 of *COA1* depicted as bed record in the bottom row.

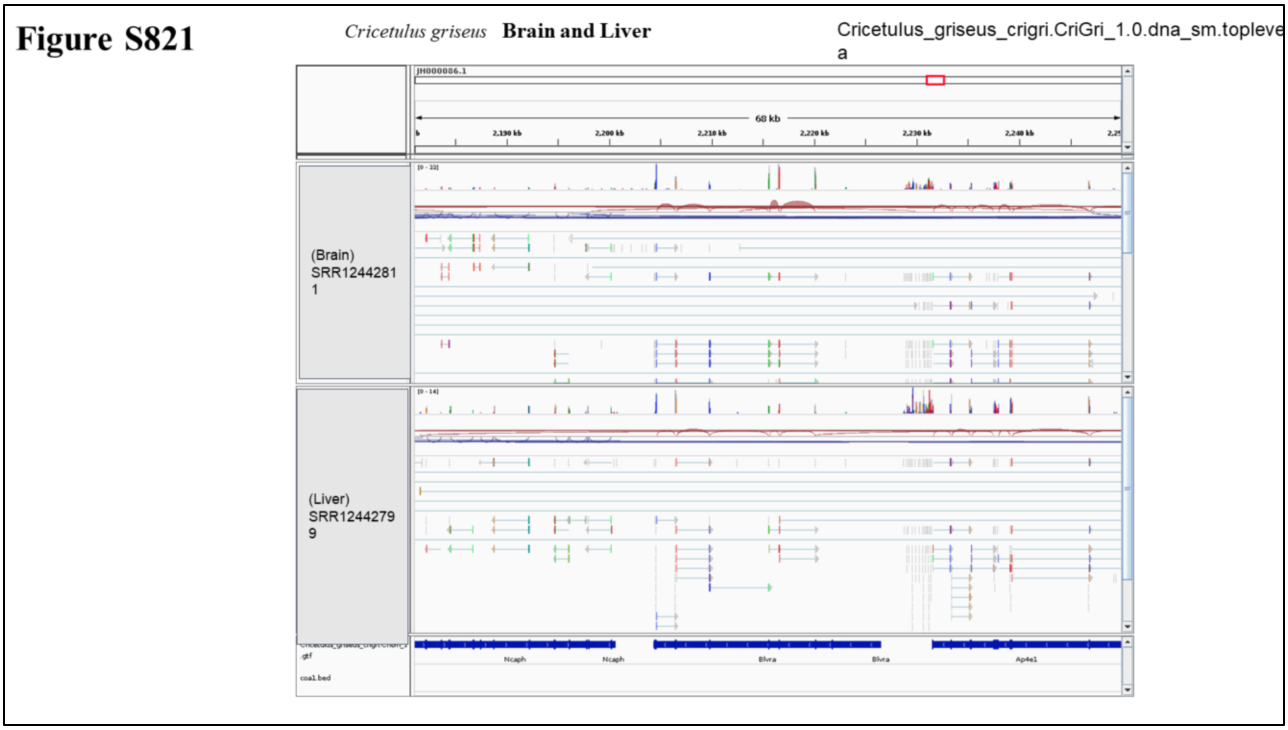

Figure S821: Screenshot of RNA-seq reads aligned each of the putative locations of *NCAPH*, BLVRA and *AP4E1* in Chinese hamster (*Cricetulus griseus*). Gray boxes represent reads aligned to the genome and thin lines connecting these boxes represent the spliced read alignments. Datasets from brain (SRR12442811) and liver (SRR12442799) have been mapped to the Cricetulus_griseus_crigri.CriGri_1.0.dna_sm.toplevel.fa. Locations of *AP4E1* and *NCAPH* are depicted as Cricetulus_griseus_crigri.CriGri_1.0.102.gtf record in the bottom row.

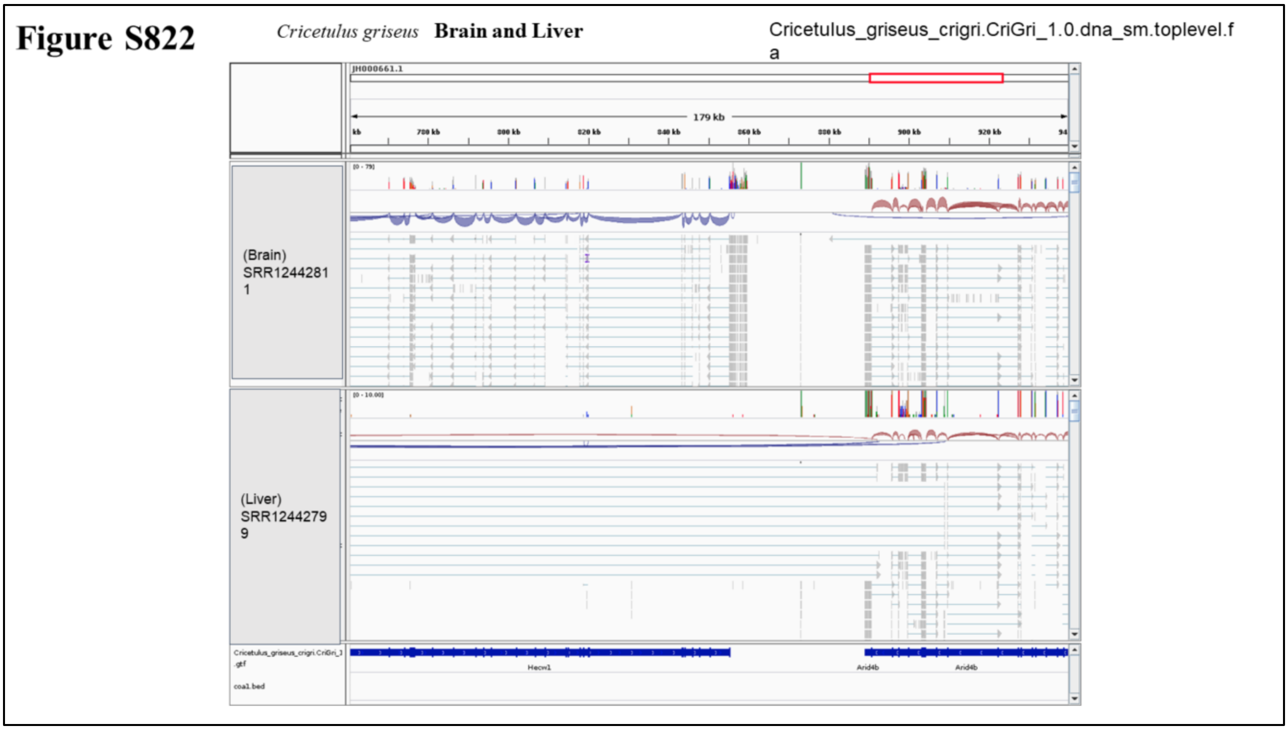

Figure S822: Screenshot of RNA-seq reads aligned each of the putative locations of *HECW1* and *ARID4B* in Chinese hamster (*Cricetulus griseus*). Gray boxes represent reads aligned to the genome and thin lines connecting these boxes represent the spliced read alignments. Datasets from brain (SRR12442811) and liver (SRR12442799) have been mapped to the Cricetulus_griseus_crigri.CriGri_1.0.dna_sm.toplevel.fa. Locations of *AP4E1* and *NCAPH* are depicted as Cricetulus_griseus_crigri.CriGri_1.0.102.gtf record in the bottom row.

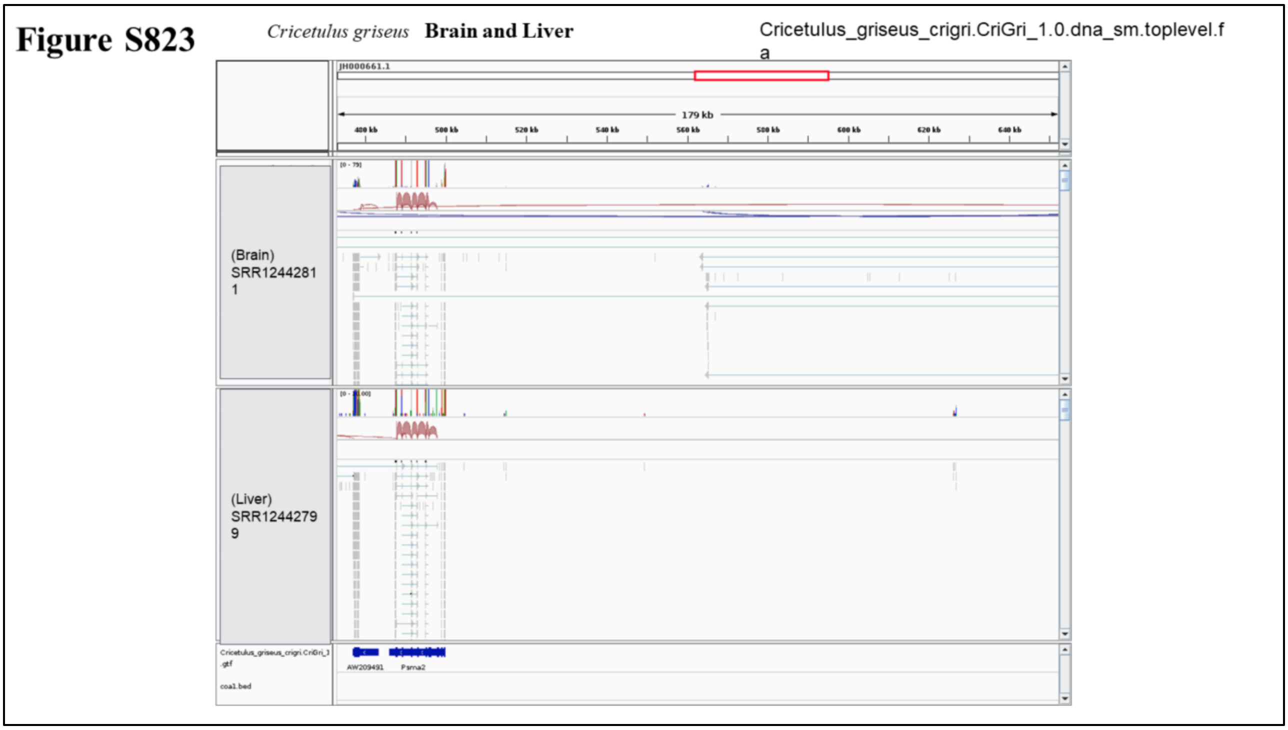

Figure S823: Screenshot of RNA-seq reads aligned each of the putative locations of *HECW1* and *PSMA2* in Chinese hamster (*Cricetulus griseus*). Gray boxes represent reads aligned to the genome and thin lines connecting these boxes represent the spliced read alignments. Datasets from brain (SRR12442811) and liver (SRR12442799) have been mapped to the Cricetulus_griseus_crigri.CriGri_1.0.dna_sm.toplevel.fa. Locations of *AP4E1* and *NCAPH* are depicted as Cricetulus_griseus_crigri.CriGri_1.0.102.gtf record in the bottom row.

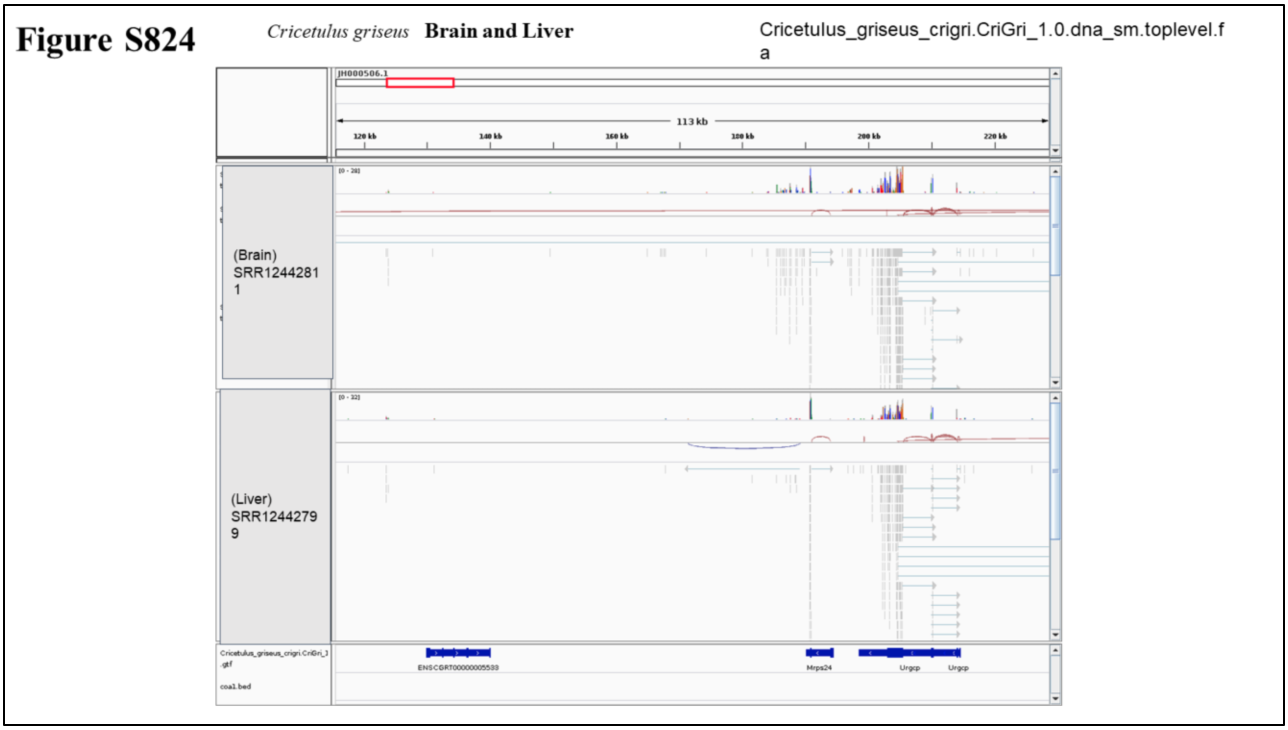

Figure S824: Screenshot of RNA-seq reads aligned each of the putative locations of *MRPS24* and *URGCP* in Chinese hamster (*Cricetulus griseus*). Gray boxes represent reads aligned to the genome and thin lines connecting these boxes represent the spliced read alignments. Datasets from brain (SRR12442811) and liver (SRR12442799) have been mapped to the Cricetulus_griseus_crigri.CriGri_1.0.dna_sm.toplevel.fa. Locations of *AP4E1* and *NCAPH* are depicted as Cricetulus_griseus_crigri.CriGri_1.0.102.gtf record in the bottom row.

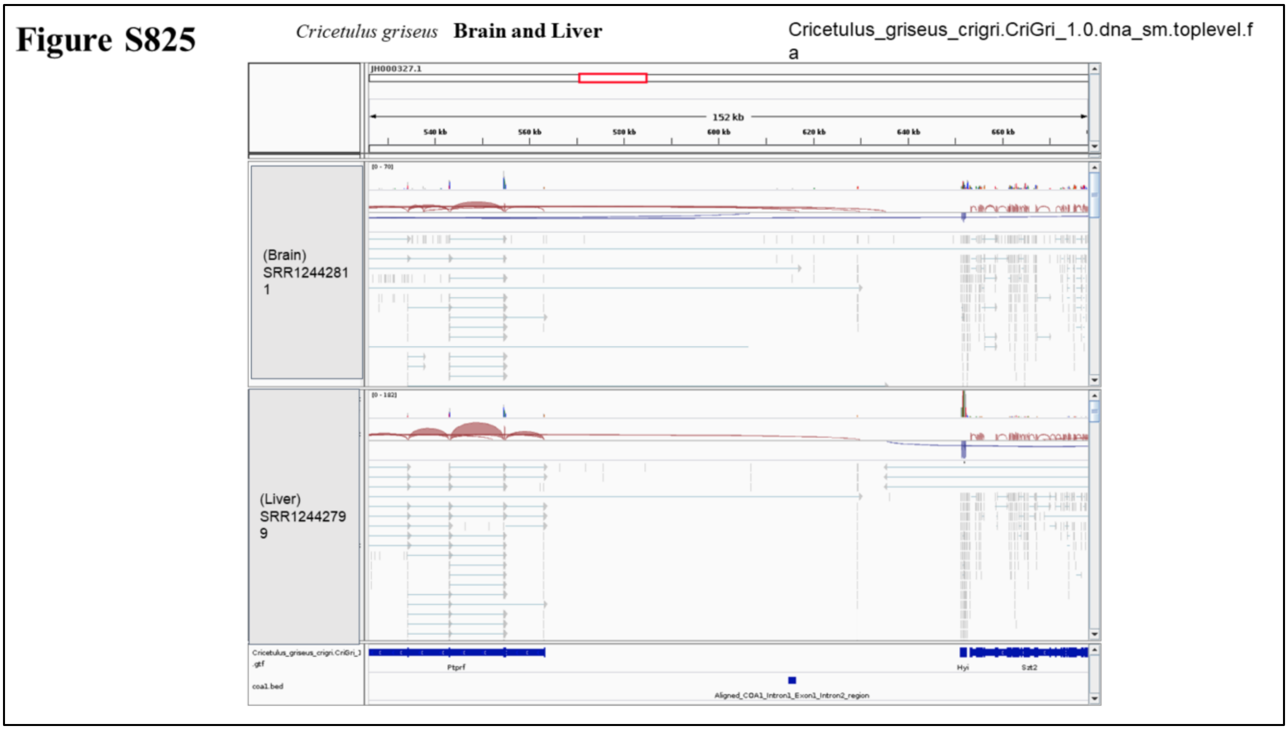

Figure S825: Screenshot of RNA-seq reads aligned each of the putative locations of truncated exon 1, intron-1 and intron-2 of *COA1* between *PTPRF* and *HYI* in Chinese hamster (*Cricetulus griseus*). Gray boxes represent reads aligned to the genome and thin lines connecting these boxes represent the spliced read alignments. Datasets from brain (SRR12442811) and liver (SRR12442799) have been mapped to the Cricetulus_griseus_crigri.CriGri_1.0.dna_sm.toplevel.fa. Locations of *AP4E1* and *NCAPH* are depicted as Cricetulus_griseus_crigri.CriGri_1.0.102.gtf record in the bottom row.

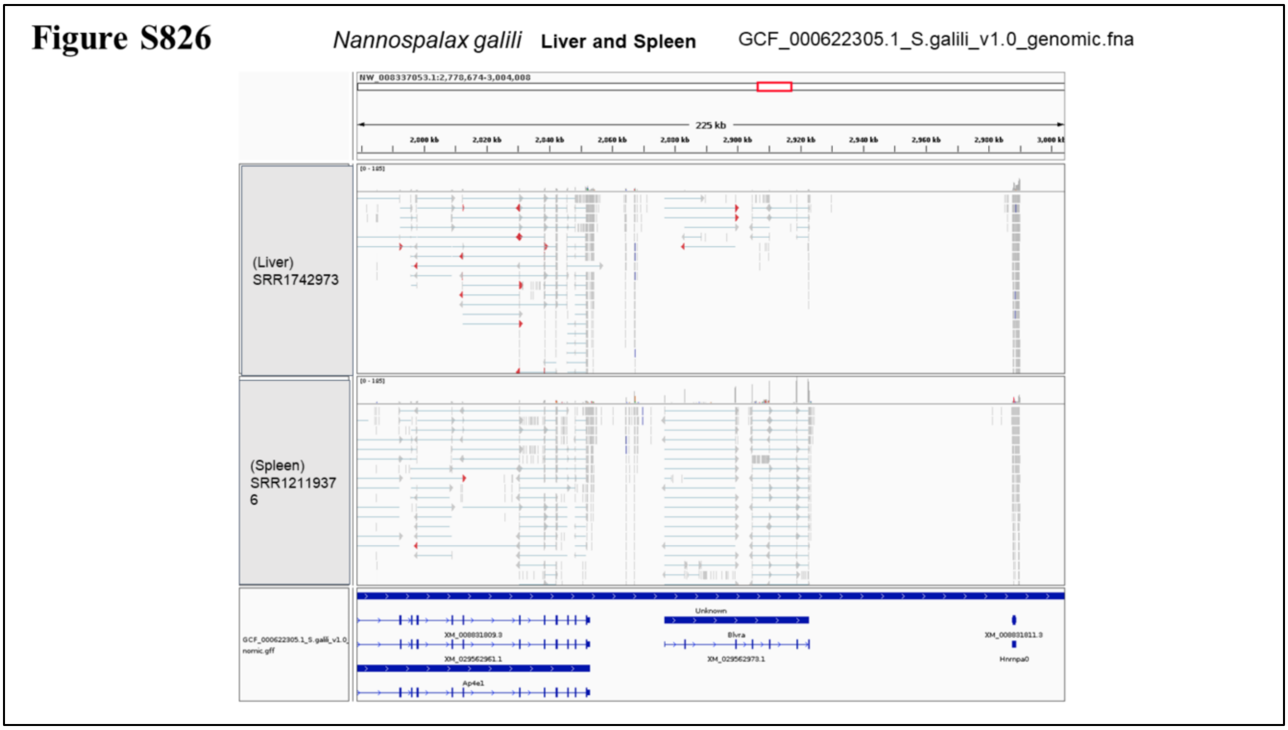

Figure S826: Screenshot of RNA-seq reads aligned each of the putative locations of *AP4E1* and *NCAPH* in Upper Galilee mountains blind mole-rat (*Nannospalax galili*). Gray boxes represent reads aligned to the genome and thin lines connecting these boxes represent the spliced read alignments. Datasets from liver (SRR1742973) and spleen (SRR12119376) have been mapped to the GCF_000622305.1_S.galili_v1.0_genomic.fna. Locations of *AP4E1* and *NCAPH* are depicted as Mesocricetus_auratus.MesAur1.0.102.gtf record in the bottom row.

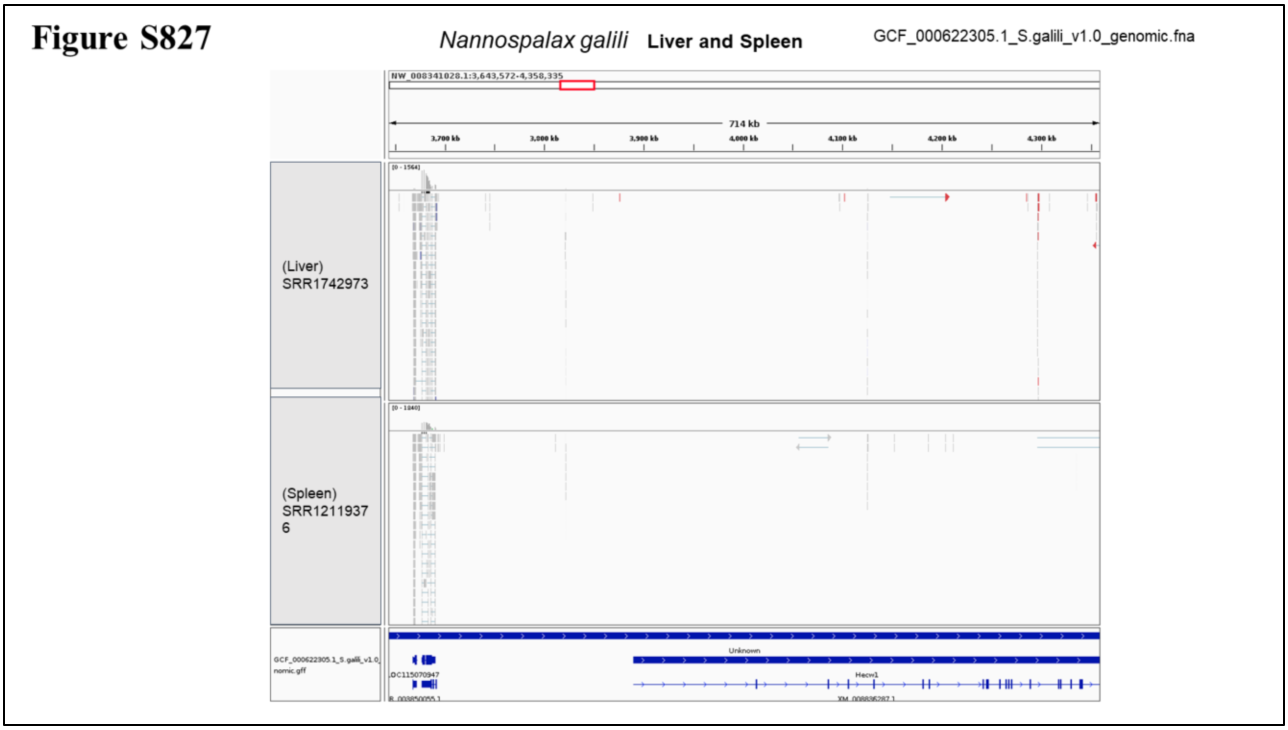

Figure S827: Screenshot of RNA-seq reads aligned each of the putative locations of *HECW1* and *ARID4B* in Upper Galilee mountains blind mole-rat (*Nannospalax galili*). Gray boxes represent reads aligned to the genome and thin lines connecting these boxes represent the spliced read alignments. Datasets from liver (SRR1742973) and spleen (SRR12119376) have been mapped to the GCF_000622305.1_S.galili_v1.0_genomic.fna. Locations of *HECW1* and *ARID4B* are depicted as Mesocricetus_auratus.MesAur1.0.102.gtf record in the bottom row.

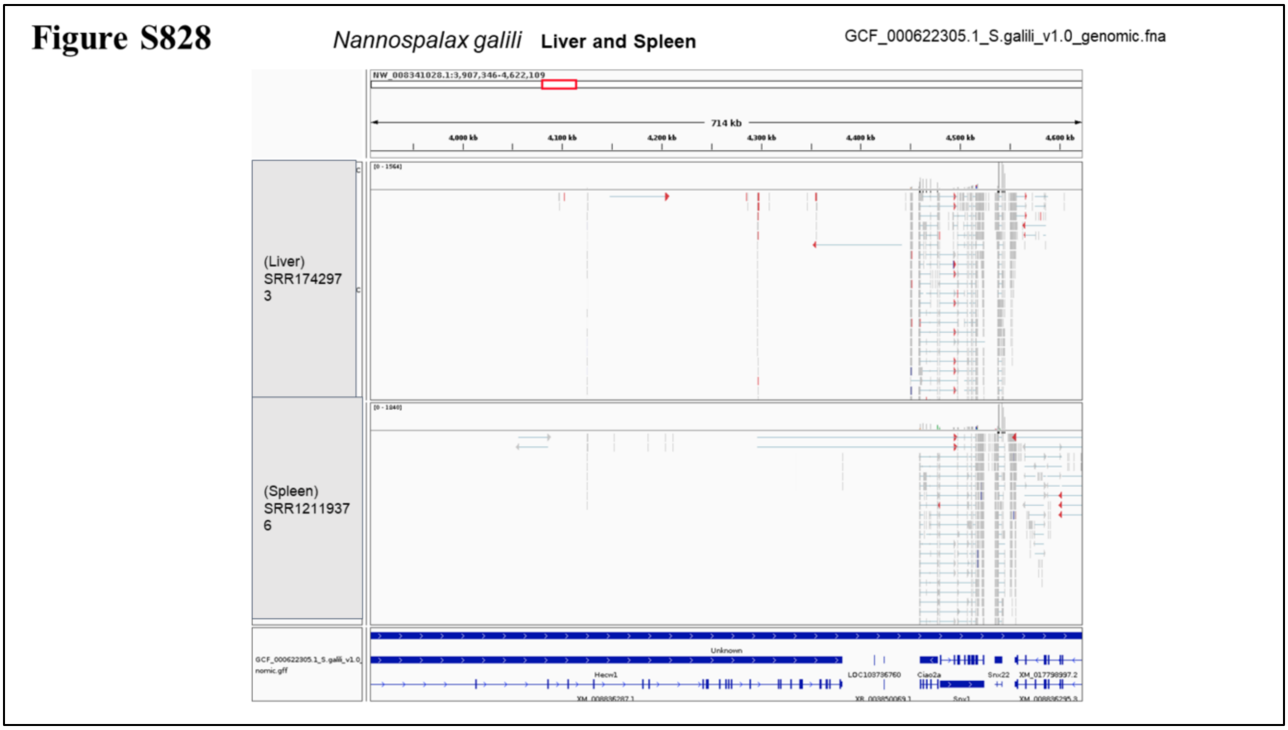

Figure S828: Screenshot of RNA-seq reads aligned each of the putative locations of *HECW1* and *MPRL32* in Upper Galilee mountains blind mole-rat (*Nannospalax galili*). Gray boxes represent reads aligned to the genome and thin lines connecting these boxes represent the spliced read alignments. Datasets from liver (SRR1742973) and spleen (SRR12119376) have been mapped to the GCF_000622305.1_S.galili_v1.0_genomic.fna. Locations of *HECW1* and *MRPL32* are depicted as Mesocricetus_auratus.MesAur1.0.102.gtf record in the bottom row.

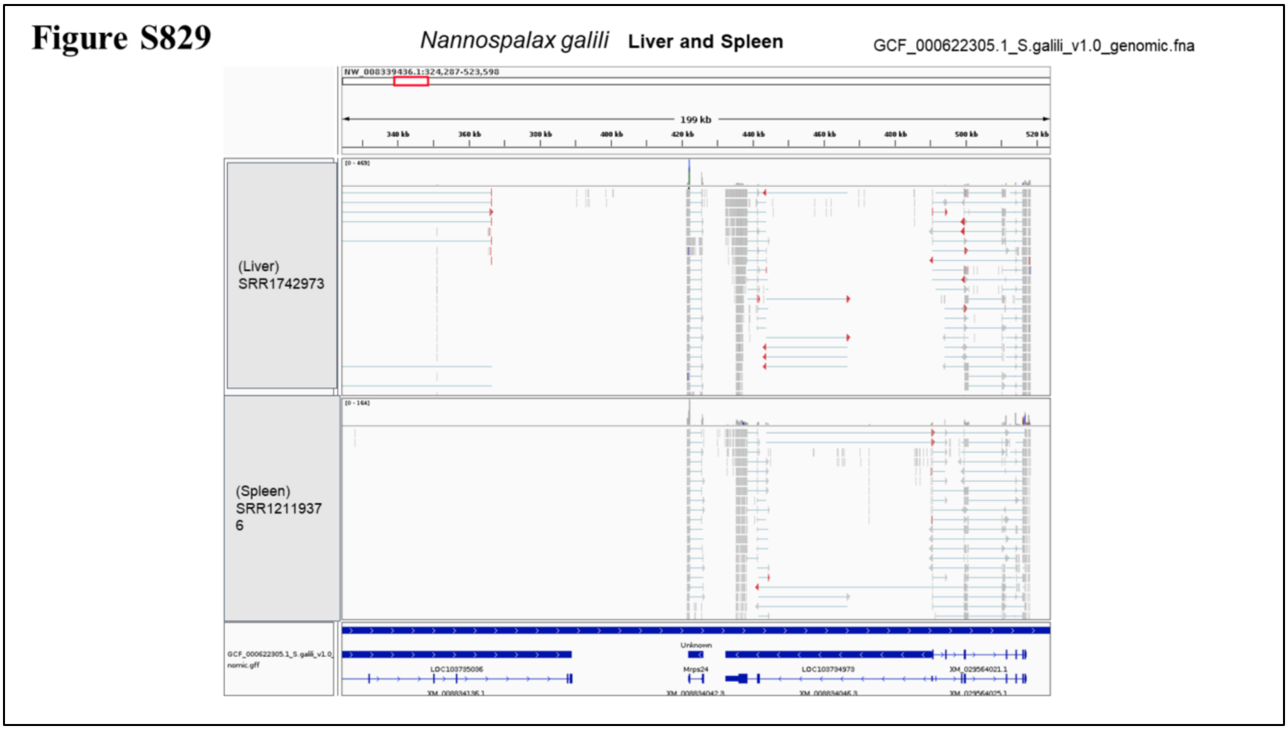

Figure S829: Screenshot of RNA-seq reads aligned each of the putative locations of *ANKRD36* and *MRPS24*in Upper Galilee mountains blind mole-rat (*Nannospalax galili*). Gray boxes represent reads aligned to the genome and thin lines connecting these boxes represent the spliced read alignments. Datasets from liver (SRR1742973) and spleen (SRR12119376) have been mapped to the GCF_000622305.1_S.galili_v1.0_genomic.fna. Locations of *ANKRD36* and *MRPS24* are depicted as Mesocricetus_auratus.MesAur1.0.102.gtf record in the bottom row.

Figure S830 *Nannospalax galili* **Liver and Spleen** GCF_000622305.1_S.galili_v1.0_genomic.fna

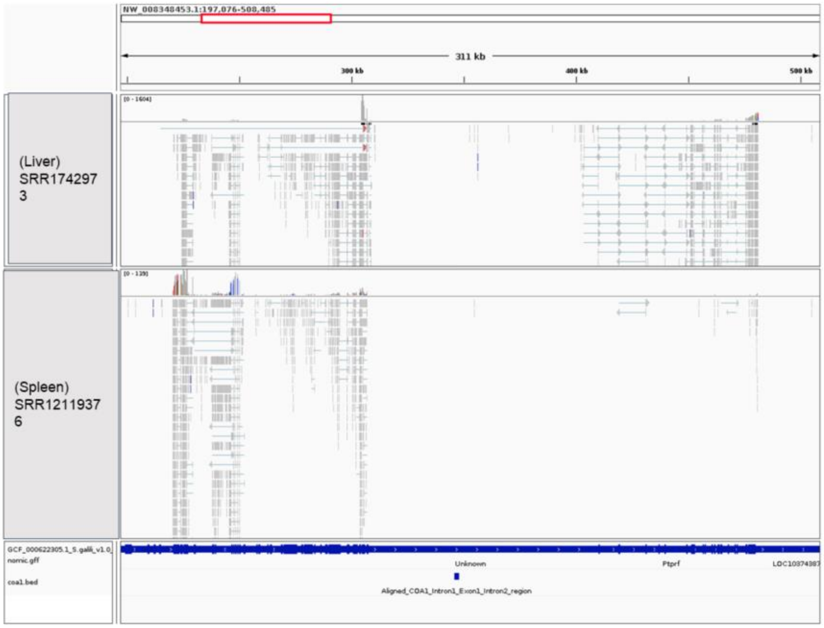

Figure S830: Screenshot of RNA-seq reads aligned each of the putative locations of *PTPRF* and *HYI* in Upper Galilee mountains blind mole-rat (*Nannospalax galili*). Gray boxes represent reads aligned to the genome and thin lines connecting these boxes represent the spliced read alignments. Datasets from liver (SRR1742973) and spleen (SRR12119376) have been mapped to the GCF_000622305.1_S.galili_v1.0_genomic.fna. Locations of *PTPRF* and *HYI* are depicted as Mesocricetus_auratus.MesAur1.0.102.gtf record in the bottom row.

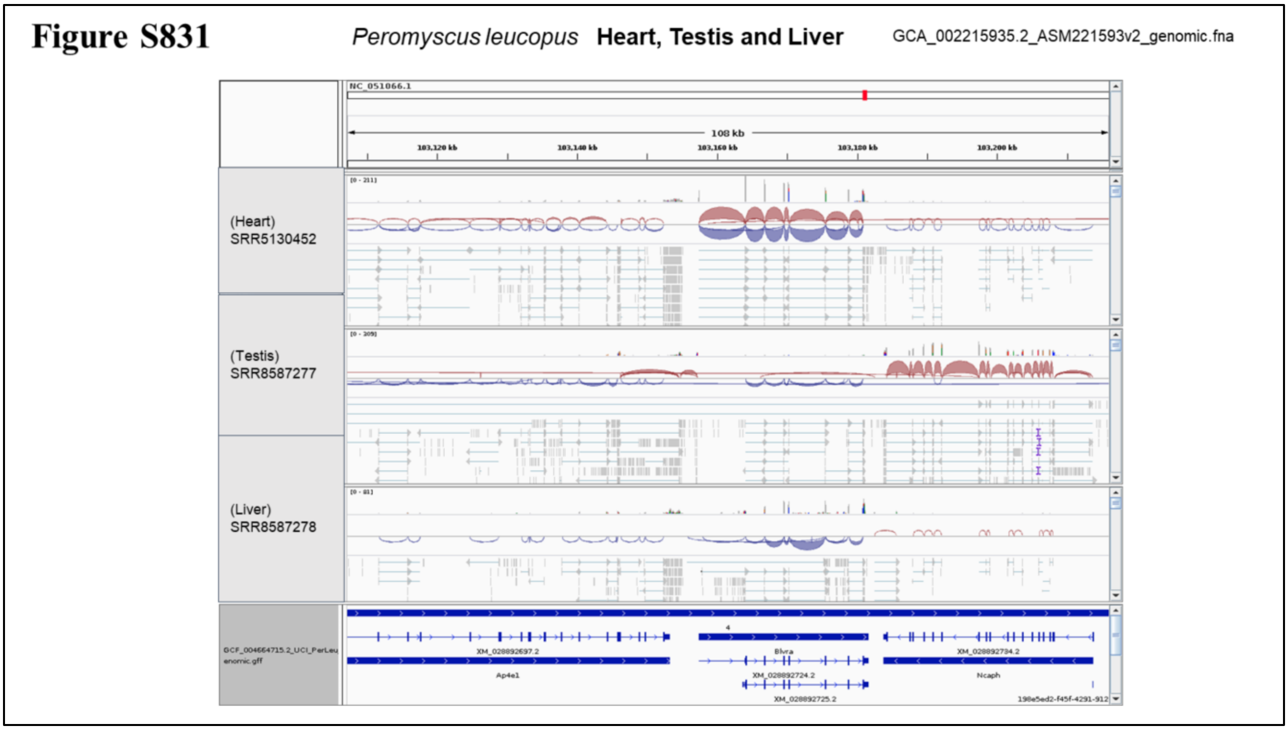

Figure S831: Screenshot of RNA-seq reads aligned each of the putative locations of *AP4E1* and *NCAPH* in White-footed mouse (*Peromyscus leucopus*). Gray boxes represent reads aligned to the genome and thin lines connecting these boxes represent the spliced read alignments. Datasets from heart (SRR5130452), testis (SRR8587277) and liver (SRR8587278) have been mapped to the GCA_002215935.2_ASM221593v2_genomic.fna. Locations of *AP4E1* and *NCAPH* are depicted as GCF_004664715.2_UCI_PerLeu_2.1_genomic.gff record in the bottom row.

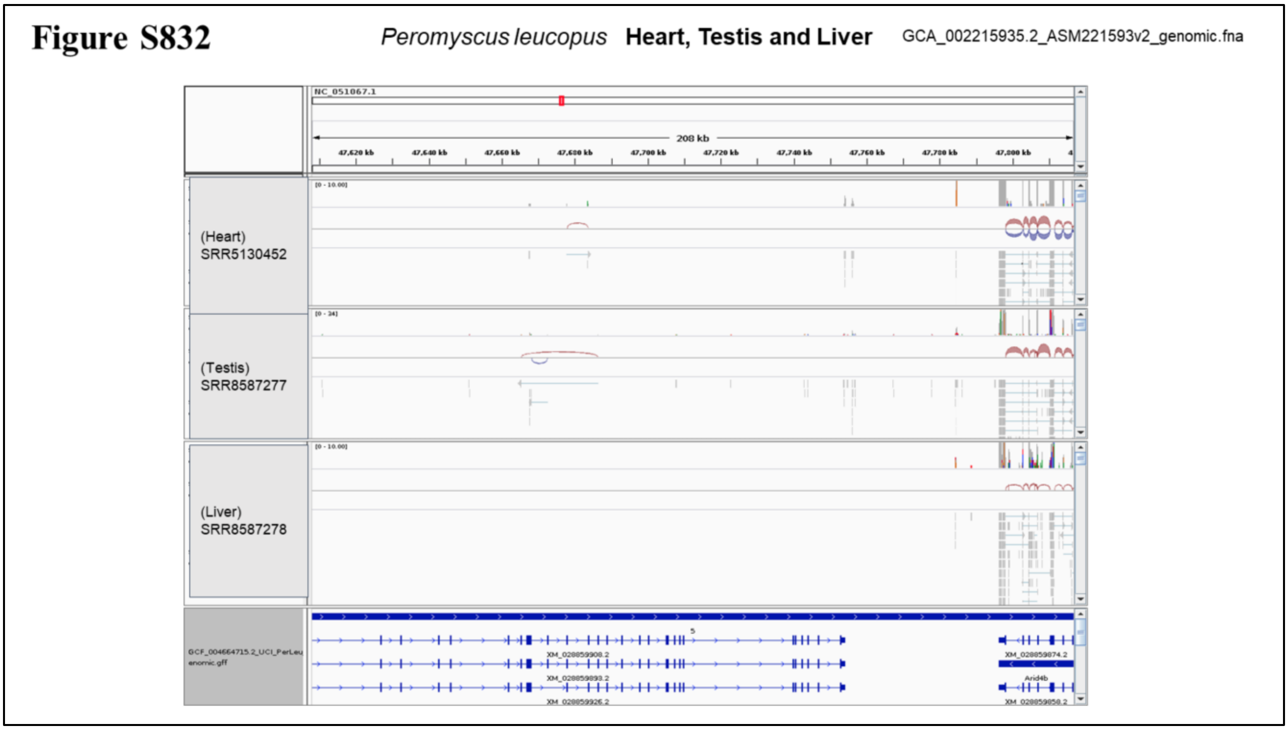

Figure S832: Screenshot of RNA-seq reads aligned each of the putative locations of *HECW1* and *ARID4B* in White-footed mouse (*Peromyscus leucopus*). Gray boxes represent reads aligned to the genome and thin lines connecting these boxes represent the spliced read alignments. Datasets from heart (SRR5130452), testis (SRR8587277) and liver (SRR8587278) have been mapped to the GCA_002215935.2_ASM221593v2_genomic.fna. Locations of *HECW1* and *ARID4B* are depicted as GCF_004664715.2_UCI_PerLeu_2.1_genomic.gff record in the bottom row.

Figure S833 *Peromyscus leucopus* **Heart, Testis and Liver** GCA_002215935.2_ASM221593v2_genomic.fna

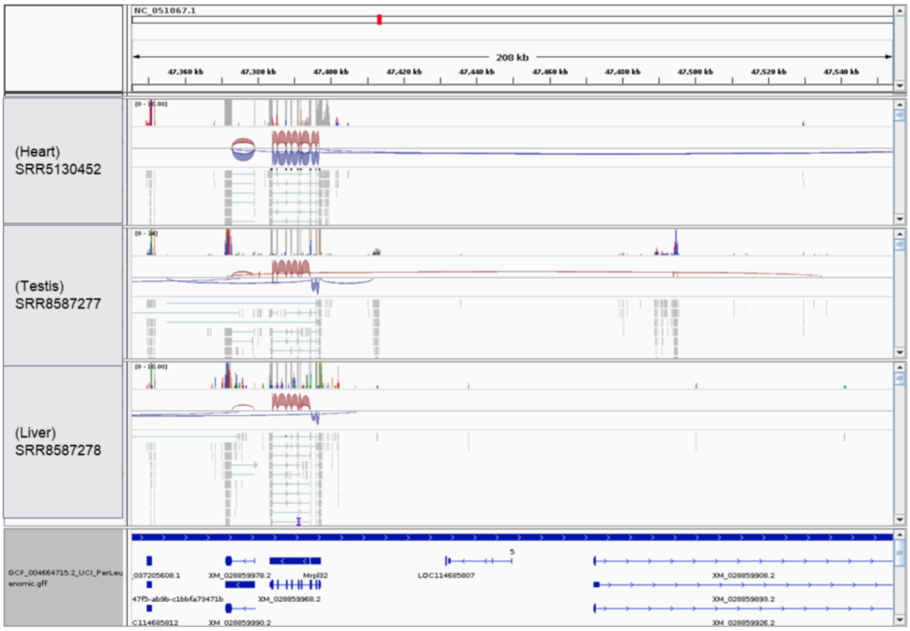

Figure S833: Screenshot of RNA-seq reads aligned each of the putative locations of *HECW1* and *MPRL32* in White-footed mouse (*Peromyscus leucopus*). Gray boxes represent reads aligned to the genome and thin lines connecting these boxes represent the spliced read alignments. Datasets from heart (SRR5130452), testis (SRR8587277) and liver (SRR8587278) have been mapped to the GCA_002215935.2_ASM221593v2_genomic.fna. Locations of *HECW1* and *MRPL32* are depicted as GCF_004664715.2_UCI_PerLeu_2.1_genomic.gff record in the bottom row.

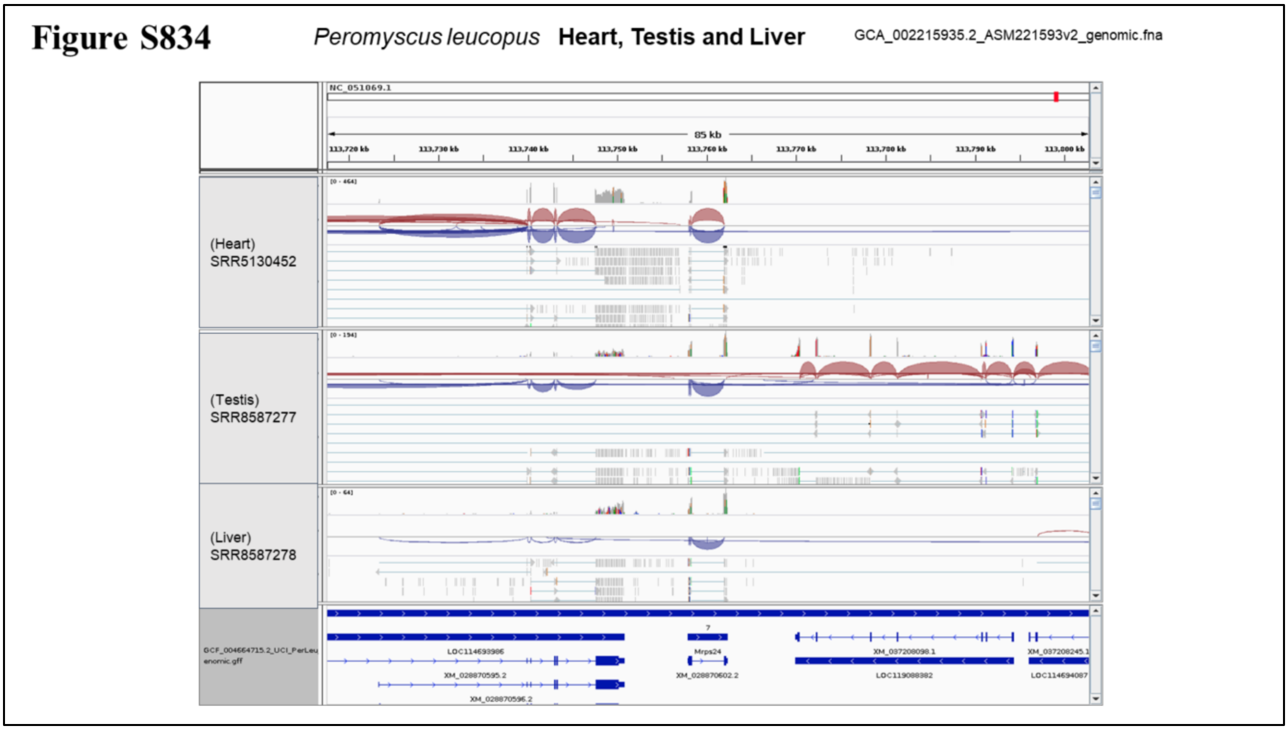

Figure S834: Screenshot of RNA-seq reads aligned each of the putative locations of *ANKRD36* and *MRPS24* in White-footed mouse (*Peromyscus leucopus*). Gray boxes represent reads aligned to the genome and thin lines connecting these boxes represent the spliced read alignments. Datasets from heart (SRR5130452), testis (SRR8587277) and liver (SRR8587278) have been mapped to the GCA_002215935.2_ASM221593v2_genomic.fna. Locations of *ANKRD36* and *MRPS24* are depicted as GCF_004664715.2_UCI_PerLeu_2.1_genomic.gff record in the bottom row.

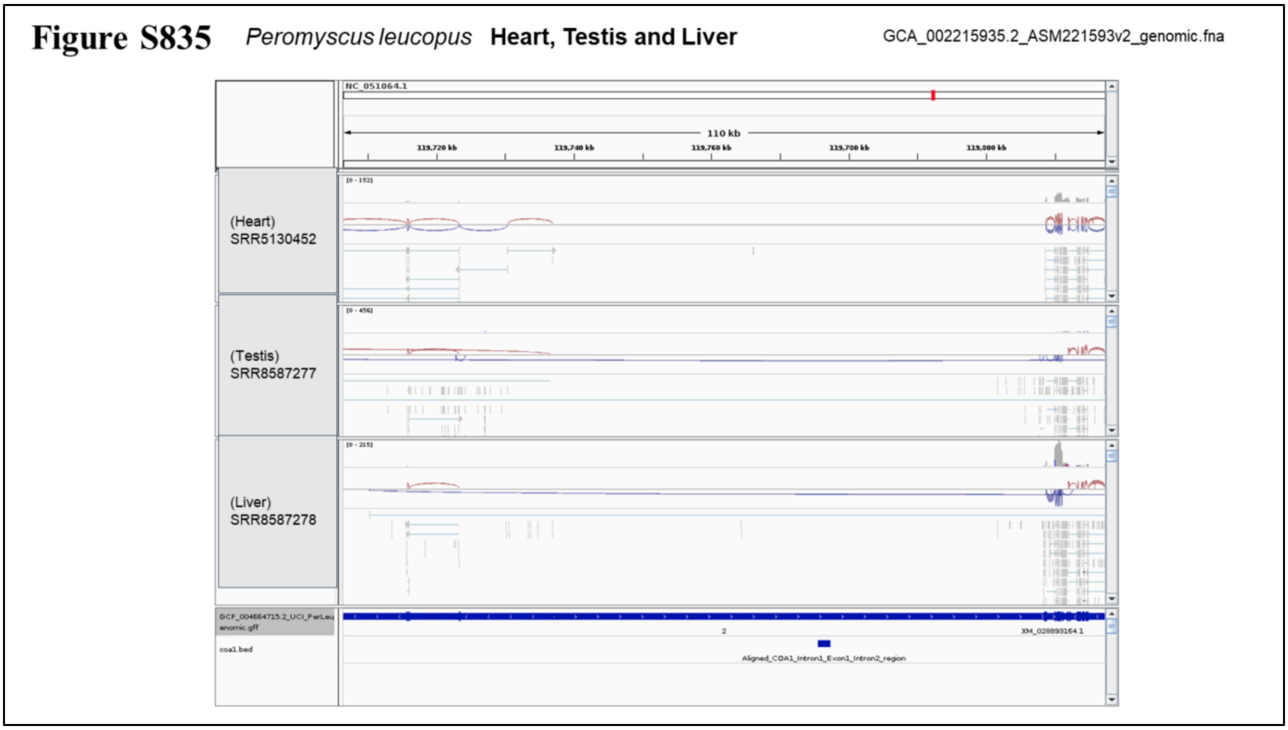

Figure S835: Screenshot of RNA-seq reads aligned each of the putative locations of truncated exon 1, intron-1 and intron-2 of *COA1* in White-footed mouse (*Peromyscus leucopus*). Gray boxes represent reads aligned to the genome and thin lines connecting these boxes represent the spliced read alignments. Datasets from heart (SRR5130452), testis (SRR8587277) and liver (SRR8587278) have been mapped to the GCA_002215935.2_ASM221593v2_genomic.fna. Locations of truncated exon 1, intron-1 and intron-2 of *COA1* is depicted as bed record in the bottom row.

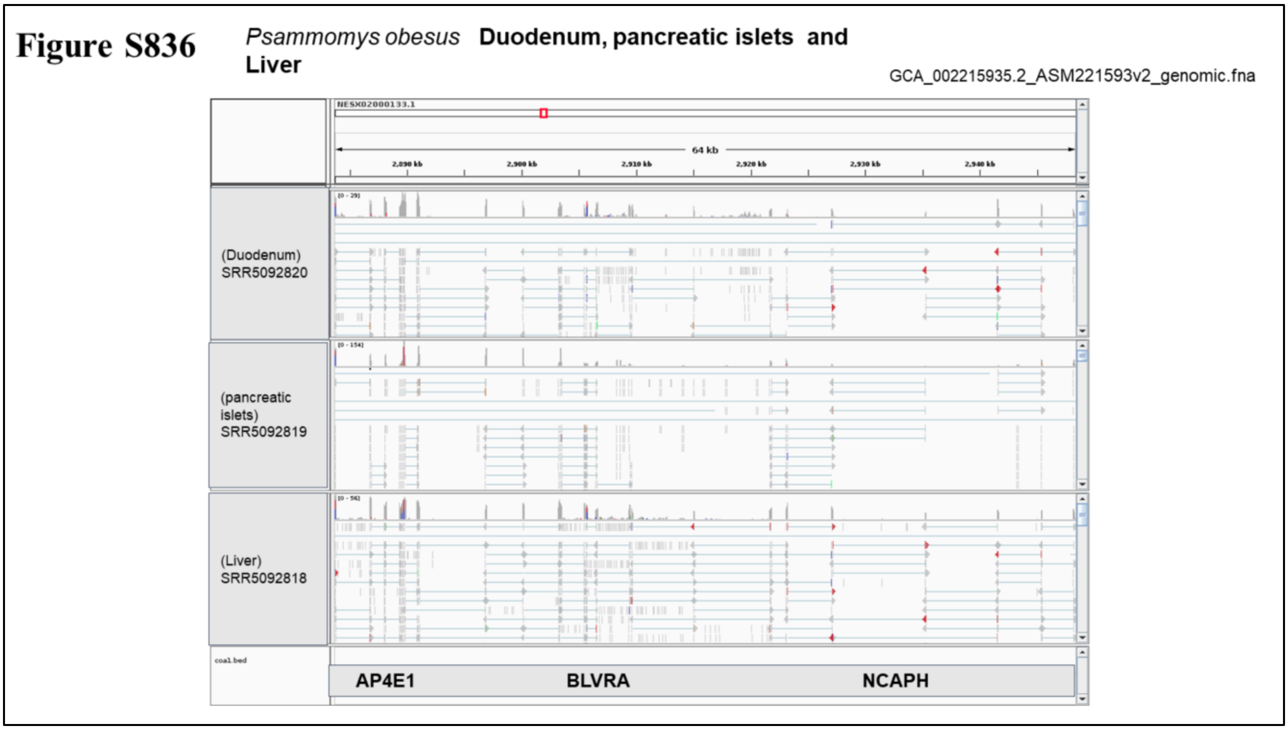

Figure S836: Screenshot of RNA-seq reads aligned each of the putative locations of *AP4E1* and *NCAPH* in fat sand rat (*Psammomys obesus*). Gray boxes represent reads aligned to the genome and thin lines connecting these boxes represent the spliced read alignments. Datasets from duodenum (SRR5092820), pancreatic islets (SRR5092819) and liver (SRR5092818) have been mapped to the GCA_002215935.2_ASM221593v2_genomic.fna. Locations of *AP4E1* and *NCAPH* are depicted as bed record in the bottom row.

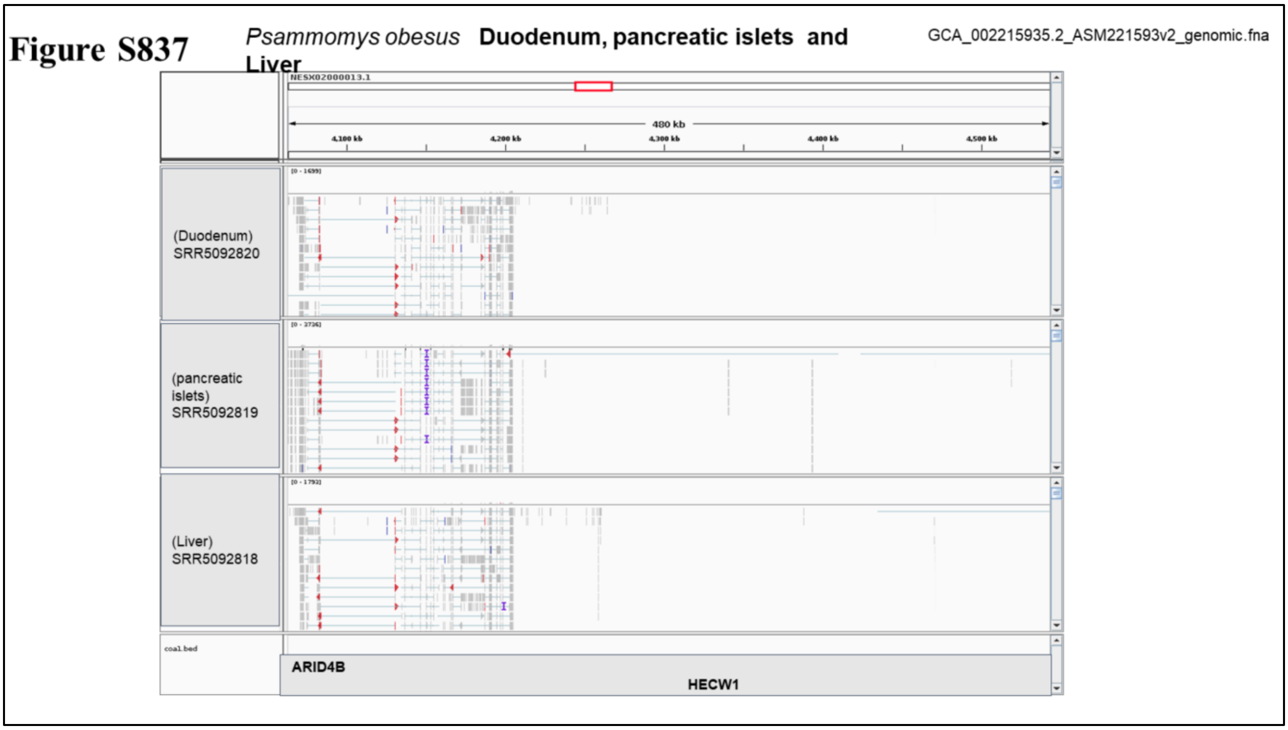

Figure S837: Screenshot of RNA-seq reads aligned each of the putative locations of *ARID4B* and *HECW1* in fat sand rat (*Psammomys obesus*). Gray boxes represent reads aligned to the genome and thin lines connecting these boxes represent the spliced read alignments. Datasets from duodenum (SRR5092820), pancreatic islets (SRR5092819) and liver (SRR5092818) have been mapped to the GCA_002215935.2_ASM221593v2_genomic.fna. Locations of *ARID4B* and *HECW1* are depicted as bed record in the bottom row.

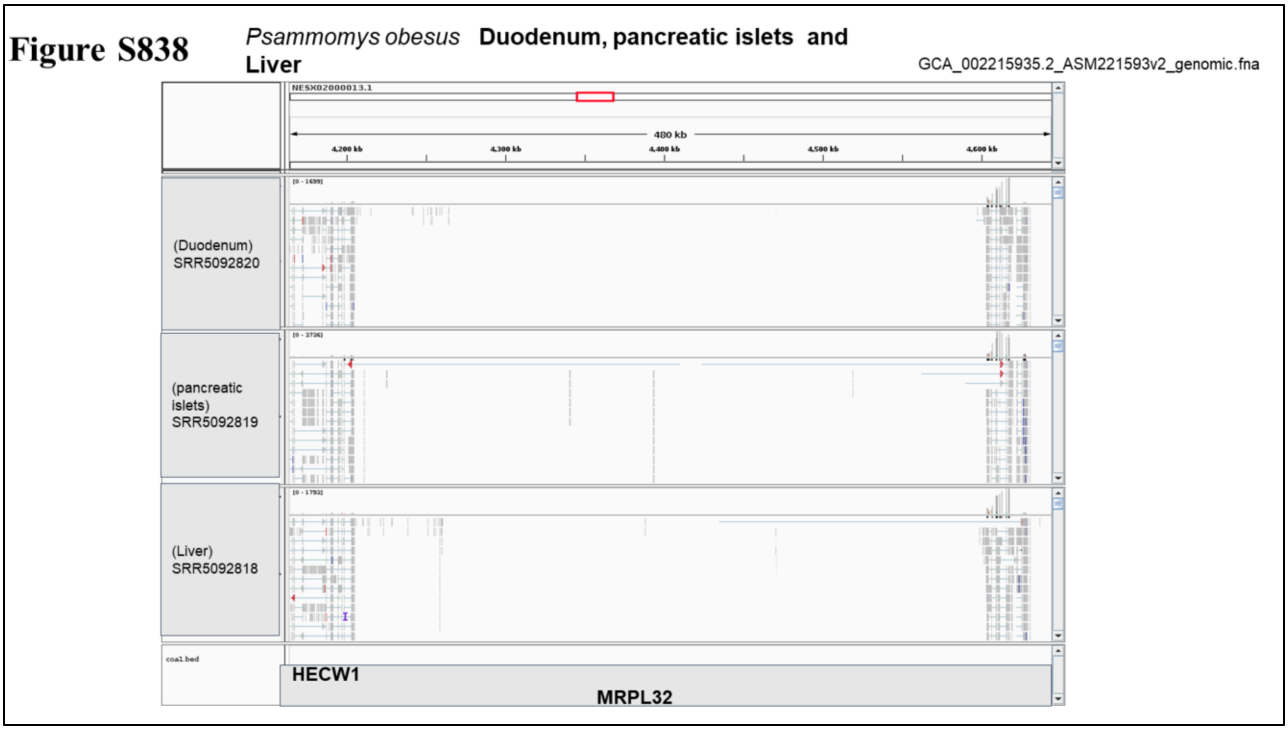

Figure S838: Screenshot of RNA-seq reads aligned each of the putative locations of *MRPL32* and *HECW1* in fat sand rat (*Psammomys obesus*). Gray boxes represent reads aligned to the genome and thin lines connecting these boxes represent the spliced read alignments. Datasets from duodenum (SRR5092820), pancreatic islets (SRR5092819) and liver (SRR5092818) have been mapped to the GCA_002215935.2_ASM221593v2_genomic.fna. Locations of *MRPL32* and *HECW1* are depicted as bed record in the bottom row.

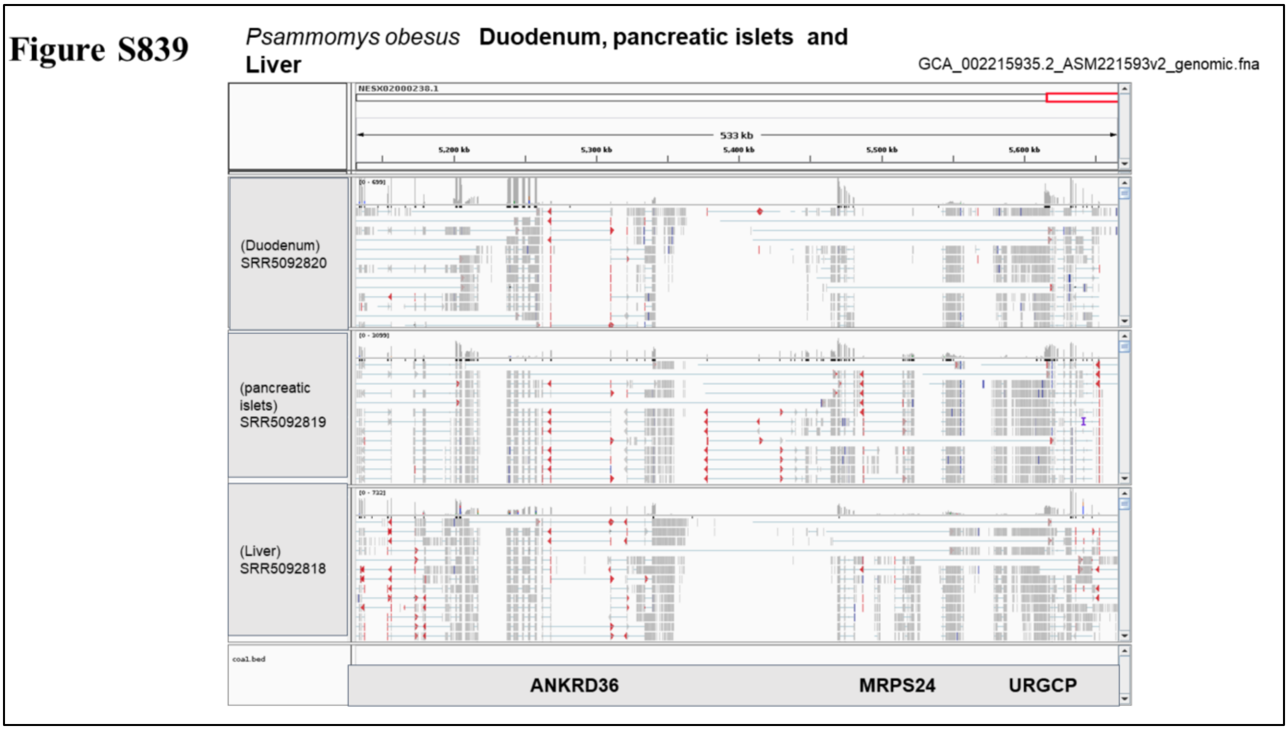

Figure S839: Screenshot of RNA-seq reads aligned each of the putative locations of *ANKRD36* and *URGCP* in fat sand rat (*Psammomys obesus*). Gray boxes represent reads aligned to the genome and thin lines connecting these boxes represent the spliced read alignments. Datasets from duodenum (SRR5092820), pancreatic islets (SRR5092819) and liver (SRR5092818) have been mapped to the GCA_002215935.2_ASM221593v2_genomic.fna. Locations of *ANKRD36* and *URGCP* are depicted as bed record in the bottom row.

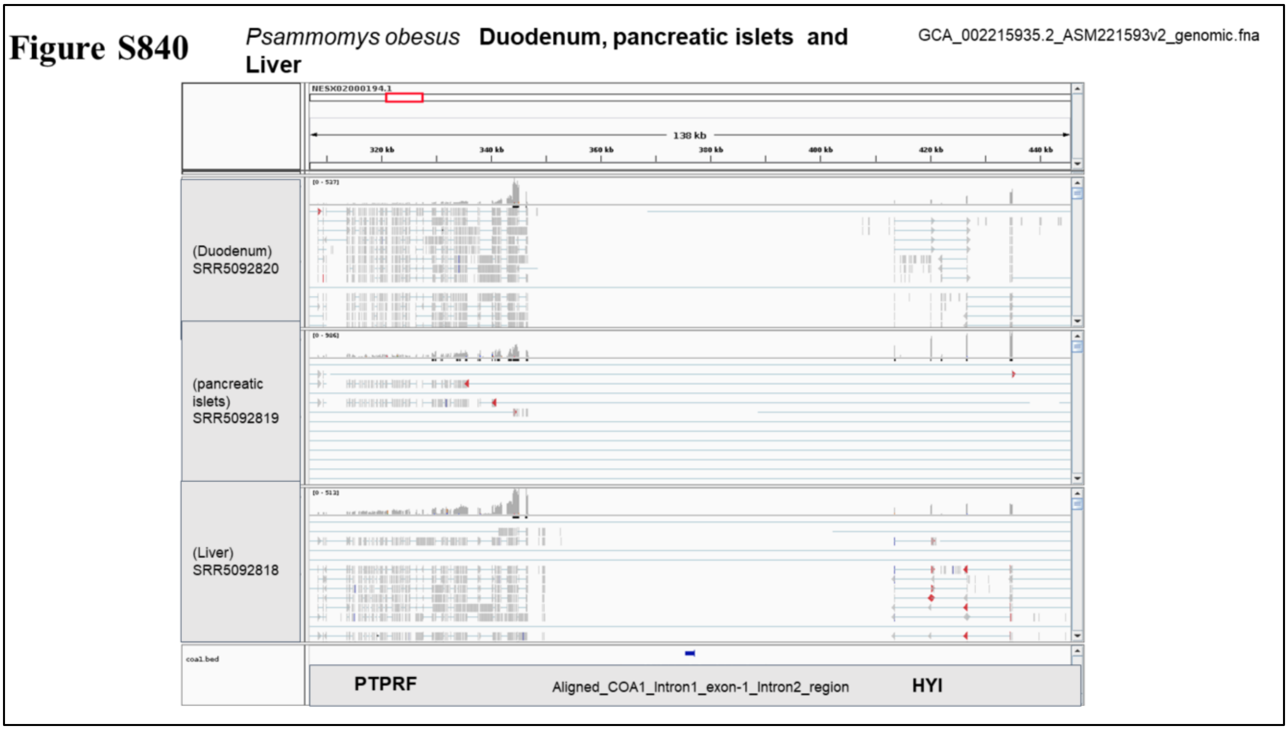

Figure S840: Screenshot of RNA-seq reads aligned each of the putative locations of truncated exon 1, intron-1 and intron-2 of *COA1* in fat sand rat (*Psammomys obesus*). Gray boxes represent reads aligned to the genome and thin lines connecting these boxes represent the spliced read alignments. Datasets from duodenum (SRR5092820), pancreatic islets (SRR5092819) and liver (SRR5092818) have been mapped to the GCA_002215935.2_ASM221593v2_genomic.fna. Locations of truncated exon 1, intron-1 and intron-2 of *COA1* is depicted as bed record in the bottom row.

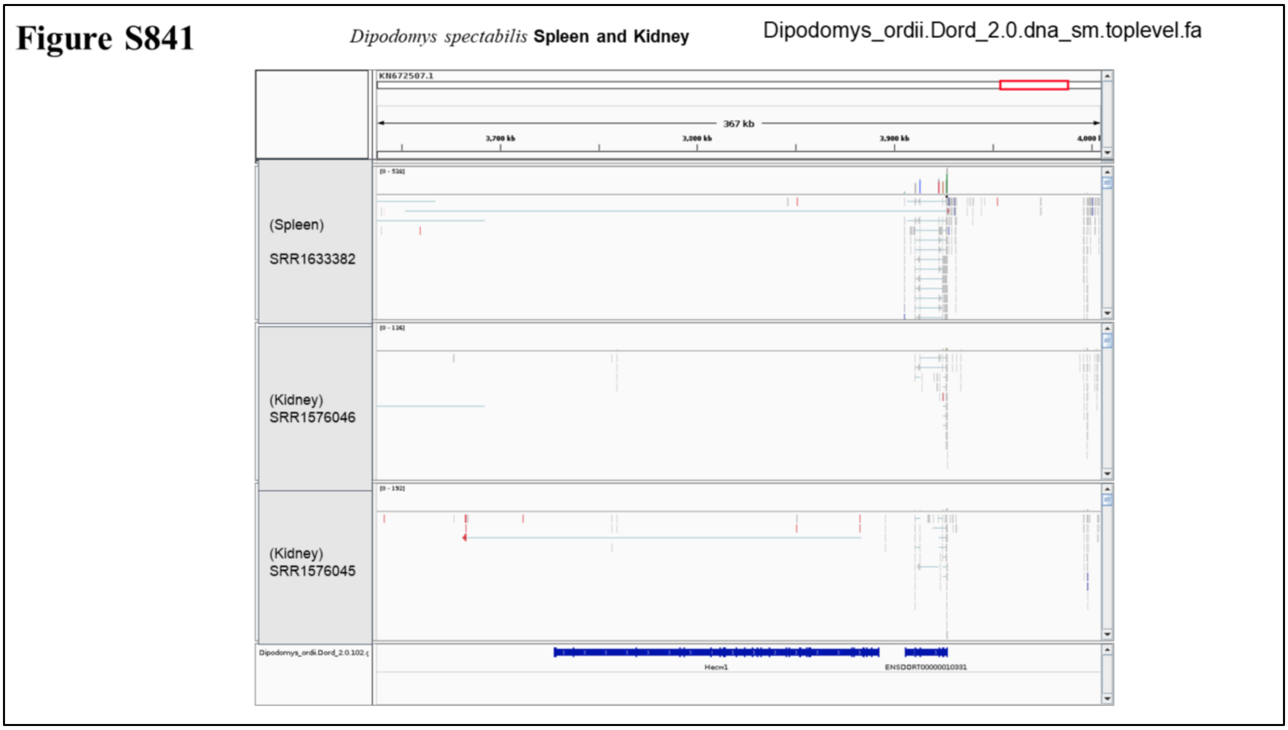

Figure S841: Screenshot of RNA-seq reads aligned each of the putative locations of *STK17A* in kangaroo rat (*Dipodomys spectabilis*). Gray boxes represent reads aligned to the genome and thin lines connecting these boxes represent the spliced read alignments. Datasets from spleen (SRR1633382) and kidney (SRR1576046 and SRR1576046) have been mapped to the Dipodomys_ordii.Dord_2.0.dna_sm.toplevel.fa. Locations of *STK17A* is depicted as Dipodomys_ordii.Dord_2.0.102.gtf record in the bottom row.

Figure S842

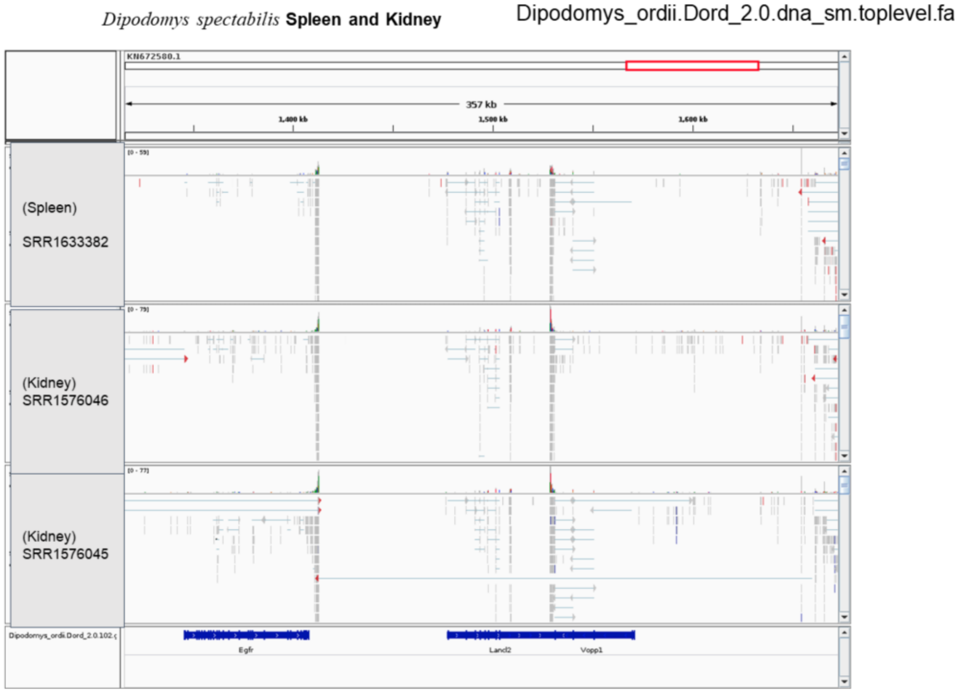

Figure S842: Screenshot of RNA-seq reads aligned each of the putative locations of *VOPP1* in kangaroo rat (*Dipodomys spectabilis*). Gray boxes represent reads aligned to the genome and thin lines connecting these boxes represent the spliced read alignments. Datasets from spleen (SRR1633382) and kidney (SRR1576046 and SRR1576046) have been mapped to the Dipodomys_ordii.Dord_2.0.dna_sm.toplevel.fa. Locations of *VOPP1* is depicted as Dipodomys_ordii.Dord_2.0.102.gtf record in the bottom row.

Figure S843

Mus musculus -PacBio

Mus_musculus.GRCm38.dna.toplevel.fa

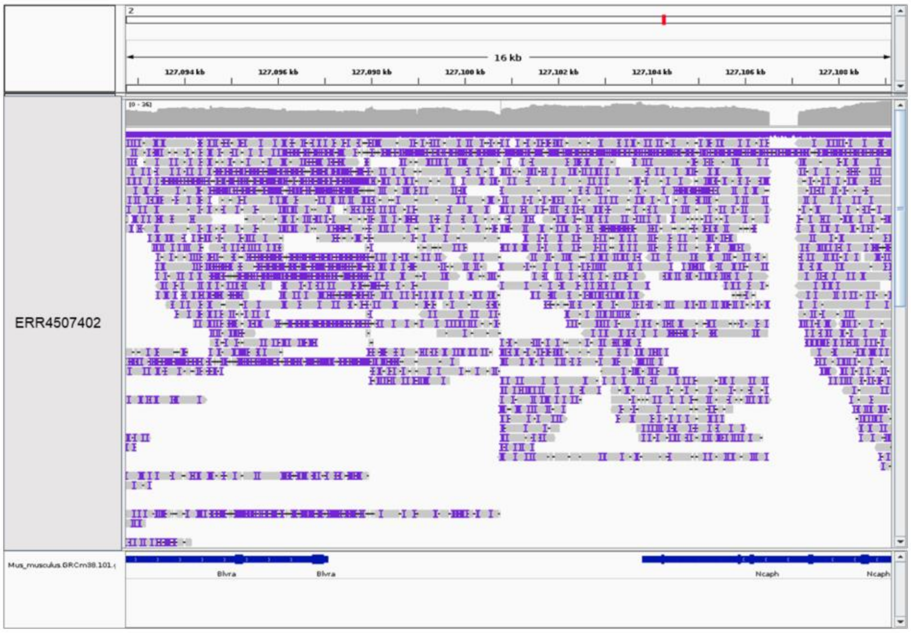

Figure S843: Screenshot of PacBio reads aligned to each of the putative locations of the *BLVRA* and *NCAPH* in mouse (*Mus musculus*). Gray boxes represent reads aligned to the genome. Raw read dataset which is mentioned above have been mapped to the Mus_musculus.GRCm38.dna.toplevel.fa.

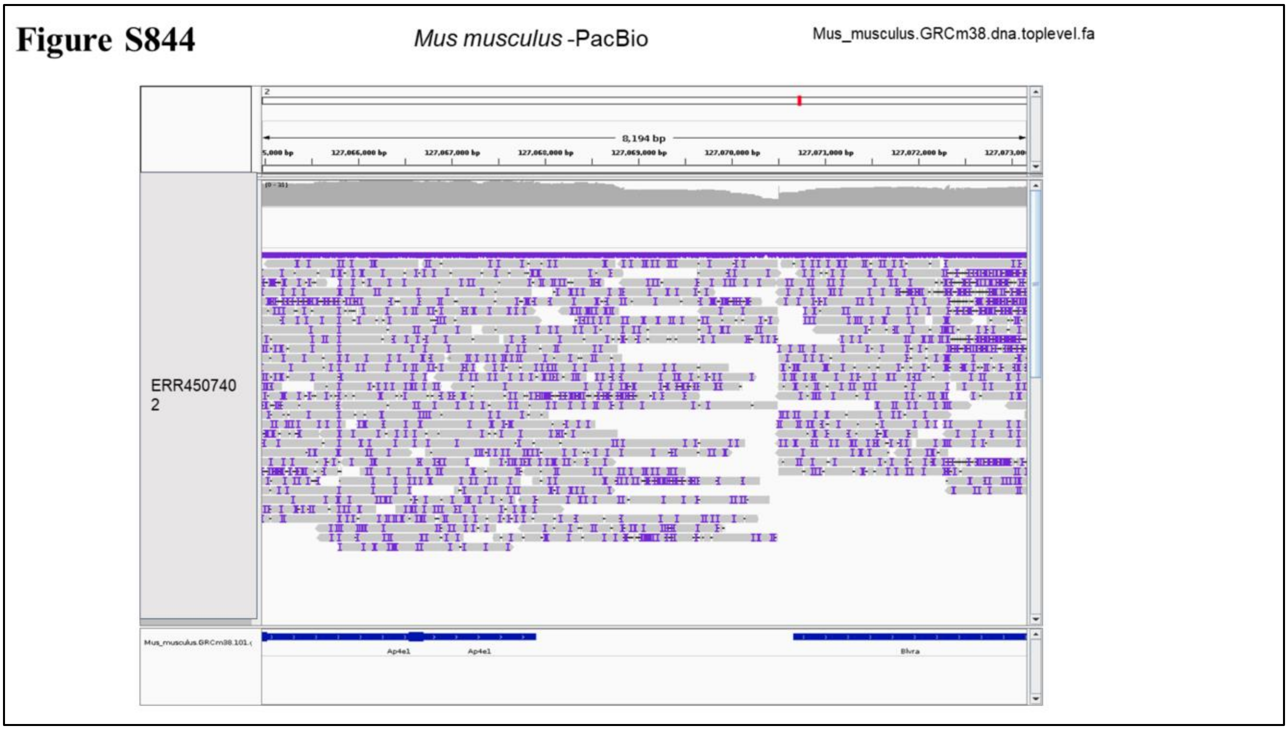

Figure S844: Screenshot of PacBio reads aligned to each of the putative locations of the *BLVRA* and *AP4E1* in mouse (*Mus musculus*). Gray boxes represent reads aligned to the genome. Raw read dataset which is mentioned above have been mapped to the Mus_musculus.GRCm38.dna.toplevel.fa.

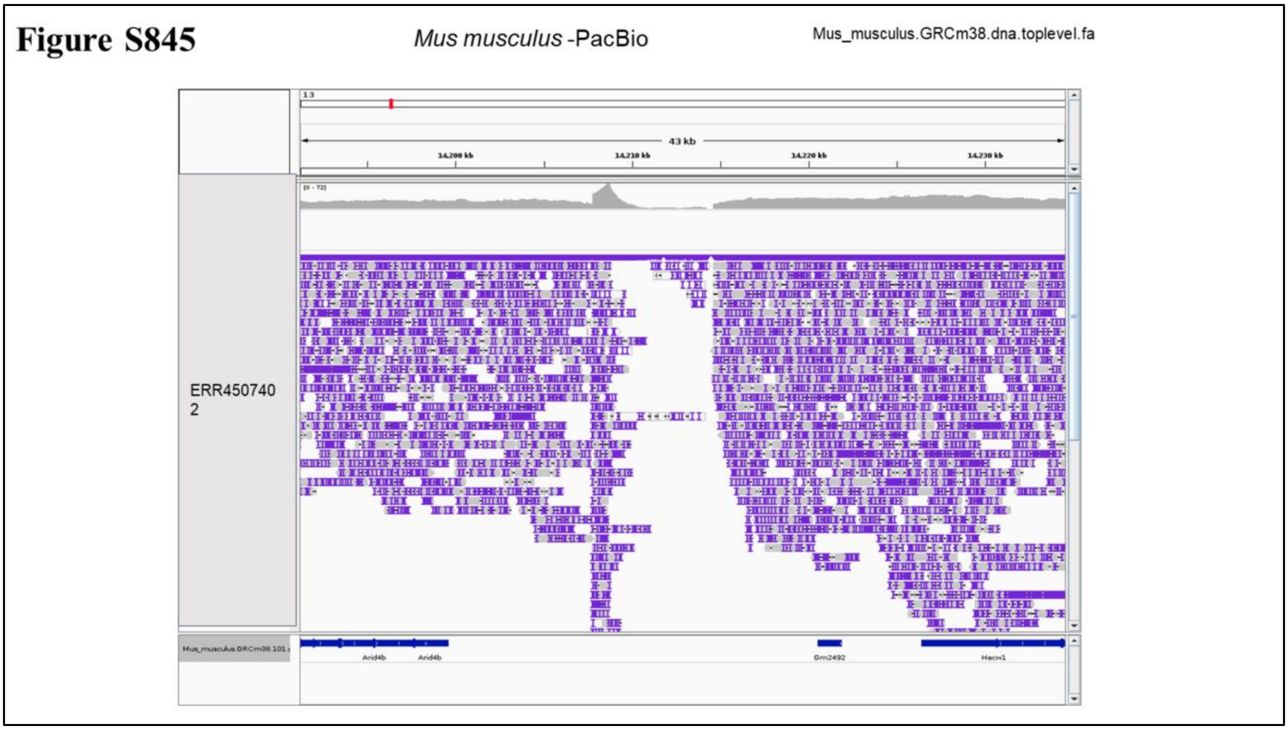

Figure S845: Screenshot of PacBio reads aligned to each of the putative locations of the *HECW1* and *ARID4B* in mouse (*Mus musculus*). Gray boxes represent reads aligned to the genome. Raw read dataset which is mentioned above have been mapped to the Mus_musculus.GRCm38.dna.toplevel.fa.

Figure S846 *Mus musculus* -PacBio Mus_musculus.GRCm38.dna.toplevel.fa

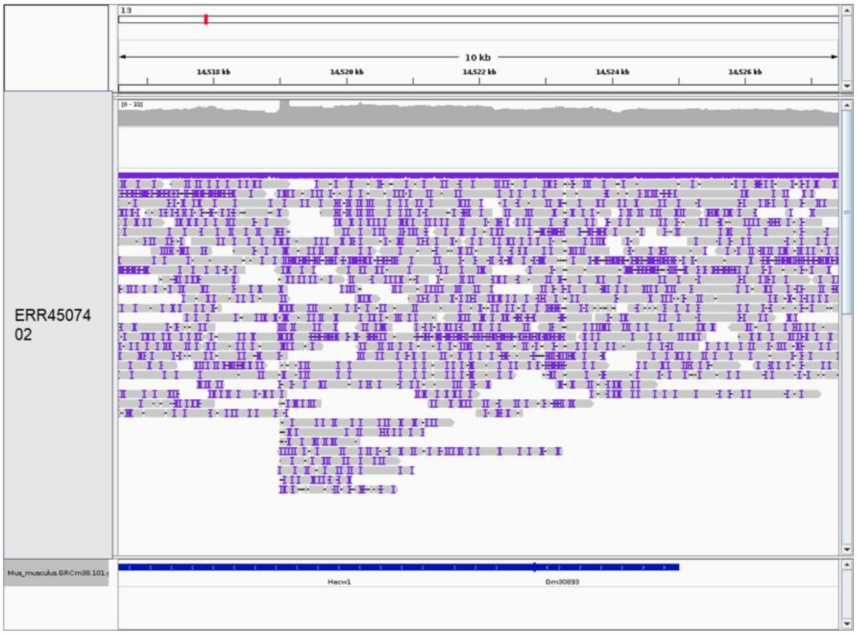

Figure S846: Screenshot of PacBio reads aligned to each of the putative locations of the *HECW1* and *MRPL32* in mouse (*Mus musculus*). Gray boxes represent reads aligned to the genome. Raw read dataset which is mentioned above have been mapped to the Mus_musculus.GRCm38.dna.toplevel.fa.

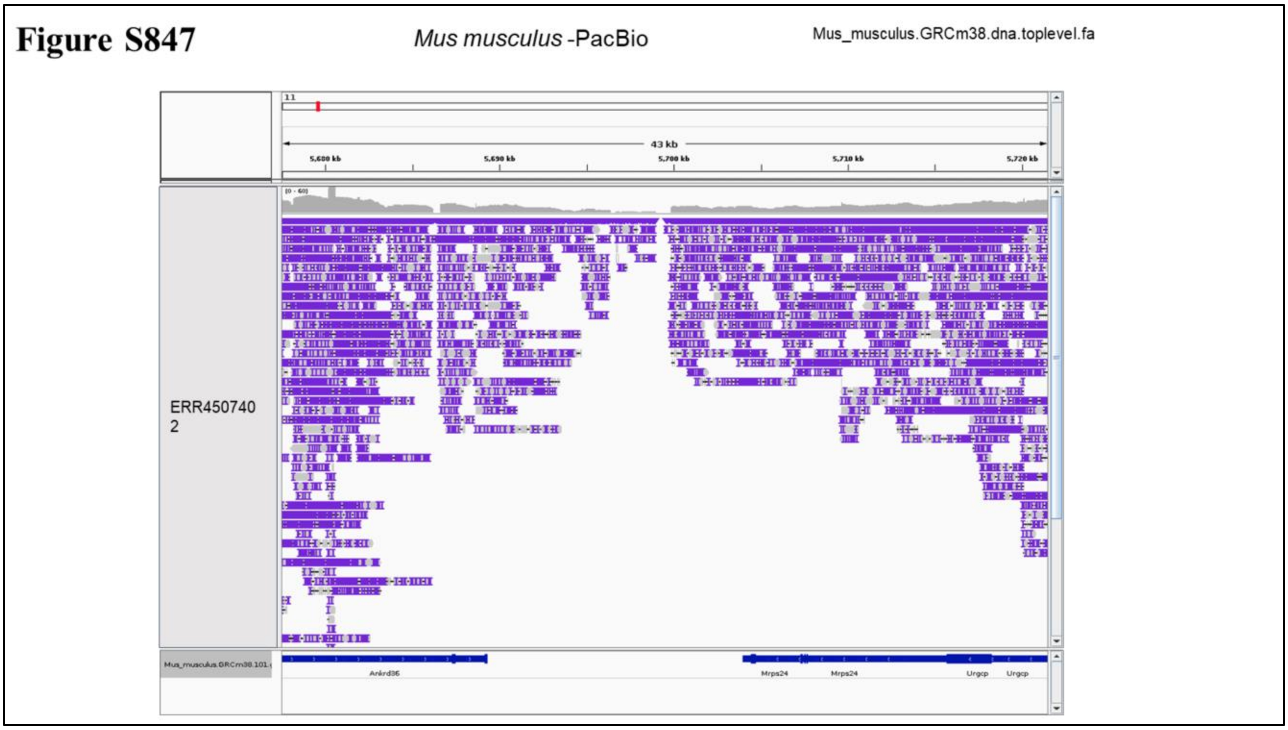

Figure S847: Screenshot of PacBio reads aligned to each of the putative locations of the *ANKRD36*, *MRPS24* and *URGCP* in mouse (*Mus musculus*). Gray boxes represent reads aligned to the genome. Raw read datasets which is mentioned above have been mapped to the Mus_musculus.GRCm38.dna.toplevel.fa.

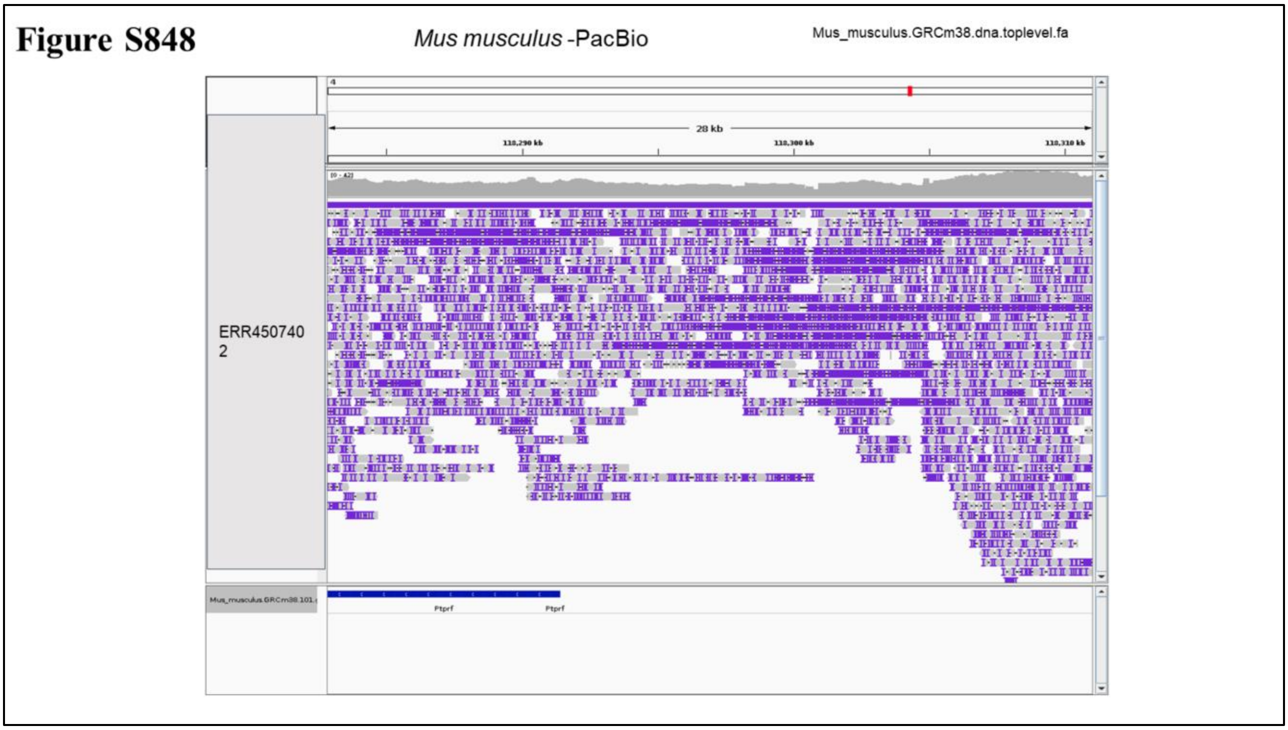

Figure S848: Screenshot of PacBio reads aligned to each of the putative locations of the *PTPRF* and *HYI* in mouse (*Mus musculus*). Gray boxes represent reads aligned to the genome. Raw read datasets which is mentioned above have been mapped to the Mus_musculus.GRCm38.dna.toplevel.fa.

Figure S849 *Mus musculus* -PacBio Mus_musculus.GRCm38.dna.toplevel.fa

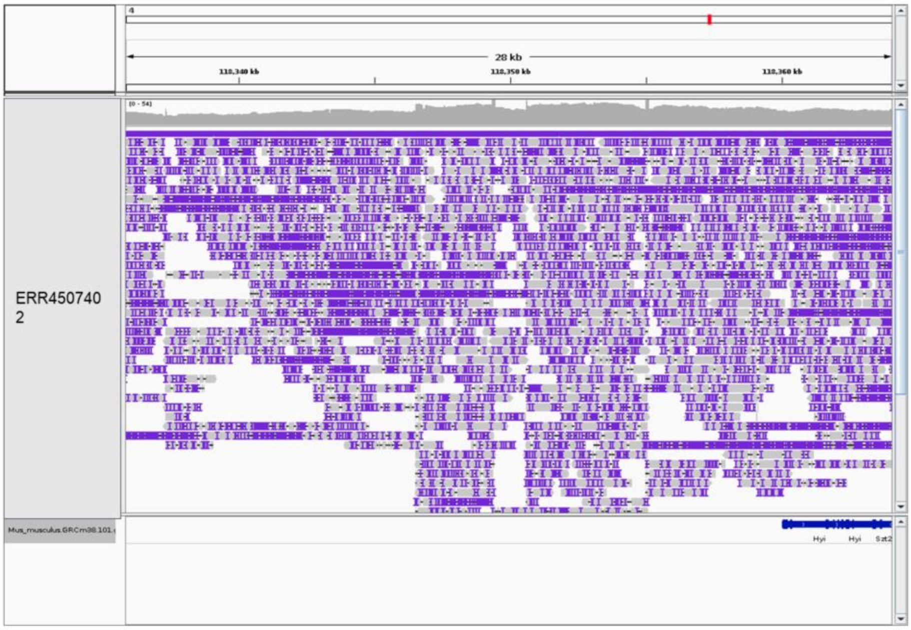

Figure S849: Screenshot of PacBio reads aligned to each of the putative locations of the *PTPRF* and *HYI* in mouse (*Mus musculus*). Gray boxes represent reads aligned to the genome. Raw read datasets which is mentioned above have been mapped to the Mus_musculus.GRCm38.dna.toplevel.fa.

Figure S850

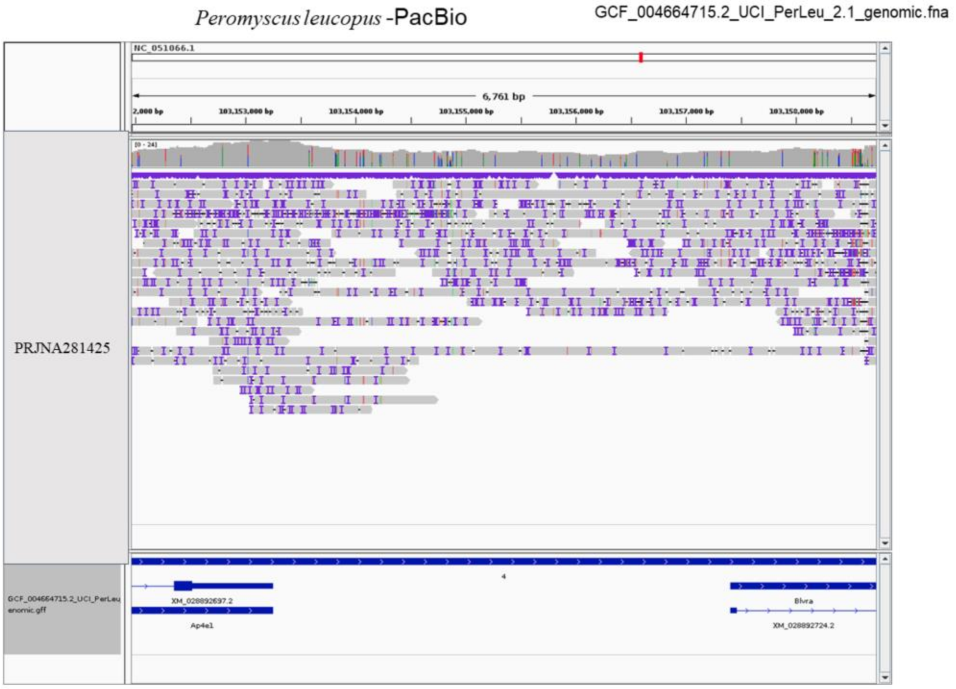

Figure S850: Screenshot of PacBio reads aligned to each of the putative locations of the *AP4E1* and *BLVRA* in white footed mouse (*Peromyscus leucopus*). Gray boxes represent reads aligned to the genome. Raw read datasets which is mentioned above have been mapped to the GCF_004664715.2_UCI_PerLeu_2.1_genomic.fna.

Figure S851

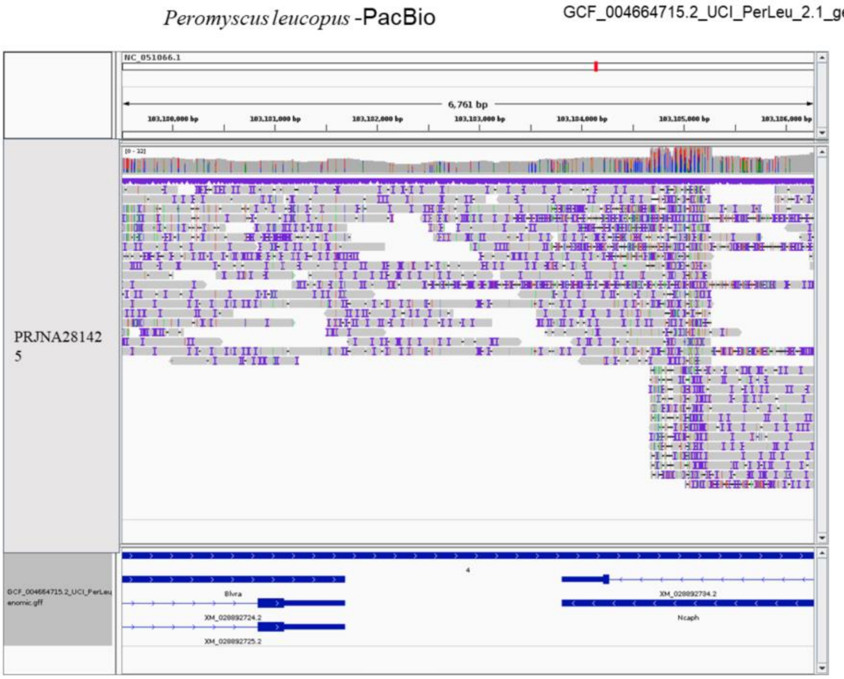

Figure S851: Screenshot of PacBio reads aligned to each of the putative locations of the *BLVRA* and *NCAPH* in white footed mouse (*Peromyscus leucopus*). Gray boxes represent reads aligned to the genome. Raw read datasets which is mentioned above have been mapped to the GCF_004664715.2_UCI_PerLeu_2.1_genomic.fna.

Figure S852

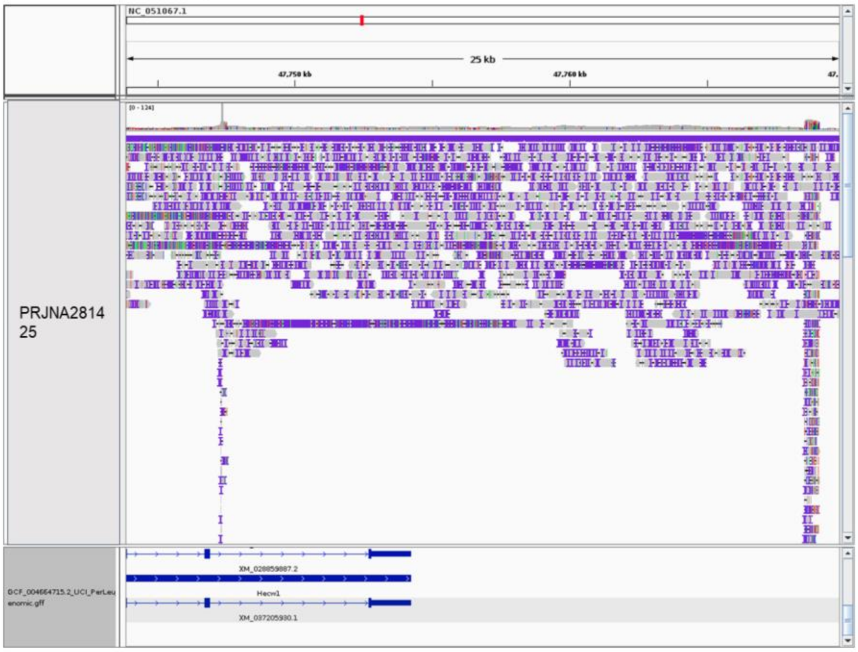

Figure S852: Screenshot of PacBio reads aligned to each of the putative locations of the *HECW1* and *ARID4B* in white footed mouse (*Peromyscus leucopus*). Gray boxes represent reads aligned to the genome. Raw read datasets which is mentioned above have been mapped to the GCF_004664715.2_UCI_PerLeu_2.1_genomic.fna.

Figure S853

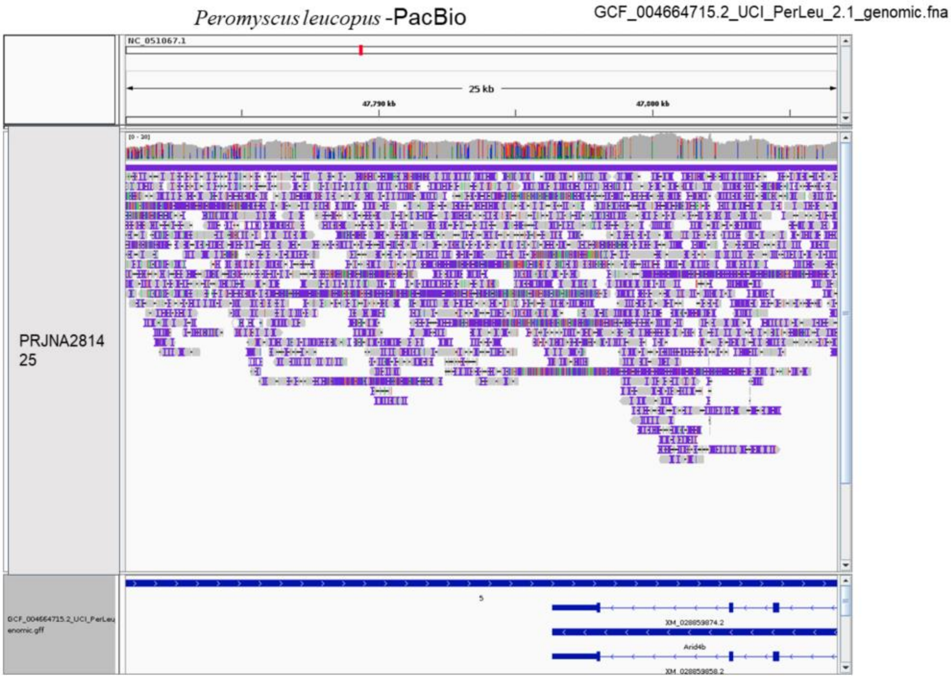

Figure S853: Screenshot of PacBio reads aligned to each of the putative locations of the *HECW1* and *ARID4B* in white footed mouse (*Peromyscus leucopus*). Gray boxes represent reads aligned to the genome. Raw read datasets which is mentioned above have been mapped to the GCF_004664715.2_UCI_PerLeu_2.1_genomic.fna.

Figure S854

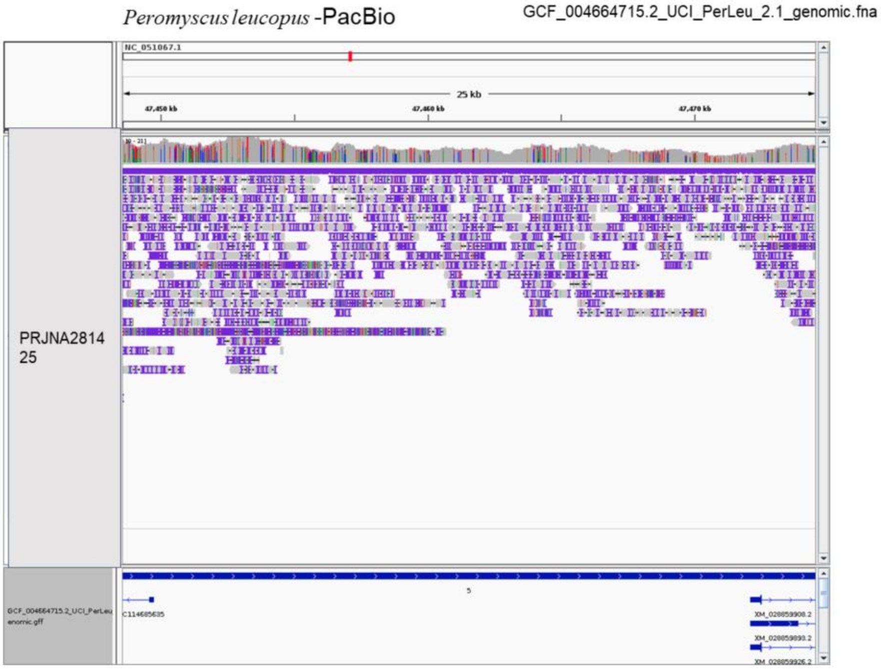

Figure S854: Screenshot of PacBio reads aligned to each of the putative locations of the *HECW1* and *MRPL32* in white footed mouse (*Peromyscus leucopus*). Gray boxes represent reads aligned to the genome. Raw read datasets which is mentioned above have been mapped to the GCF_004664715.2_UCI_PerLeu_2.1_genomic.fna.

Figure S855

Peromyscus leucopus -PacBio GCF_004664715.2_UCI_PerLeu_2.1_genomic.fna

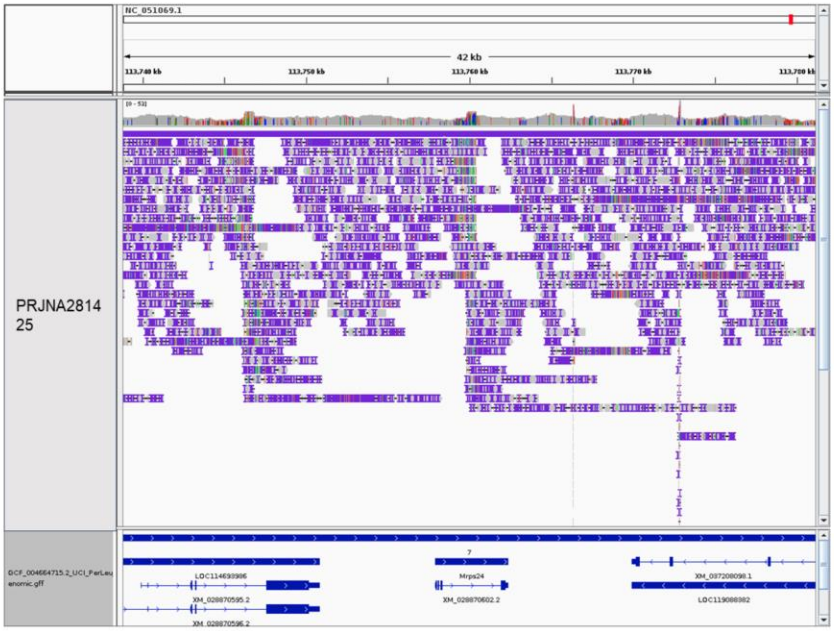

Figure S855: Screenshot of PacBio reads aligned to each of the putative locations of the *ANKRD36*, *MRPS24* and *URGCP* in white footed mouse (*Peromyscus leucopus*). Gray boxes represent reads aligned to the genome. Raw read datasets which is mentioned above have been mapped to the GCF_004664715.2_UCI_PerLeu_2.1_genomic.fna.

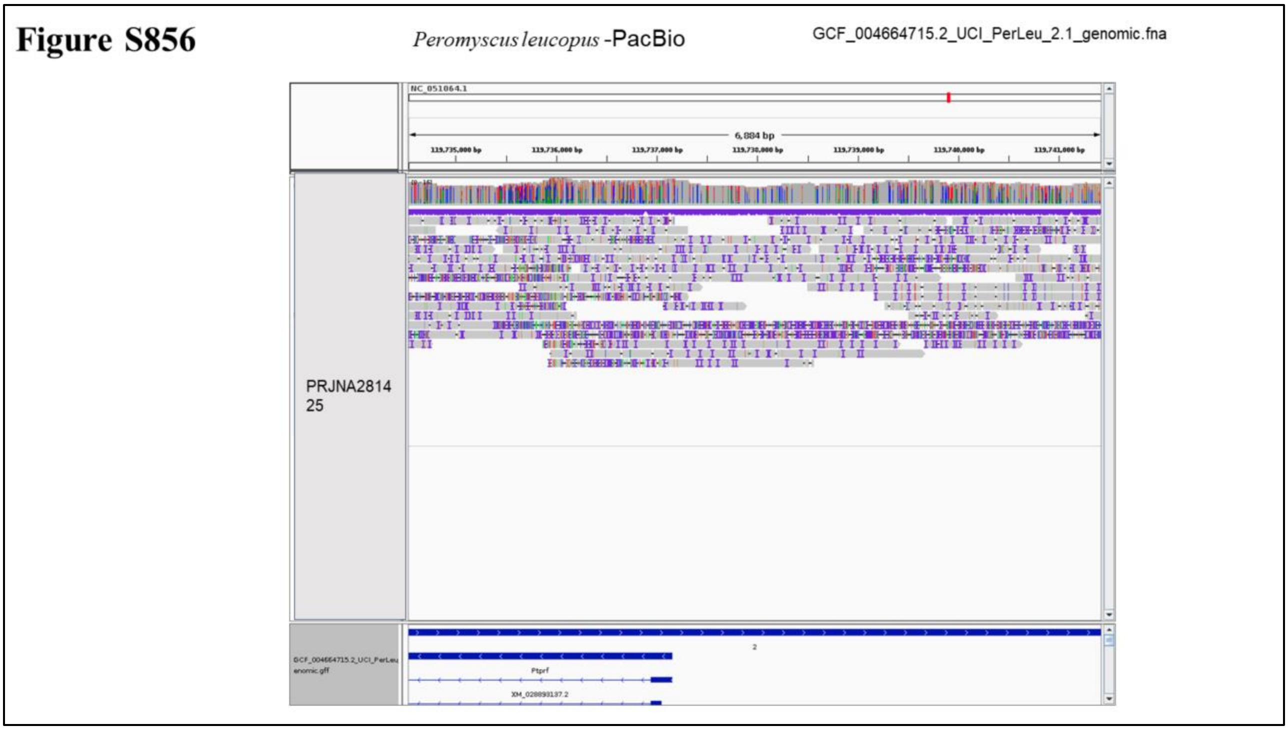

Figure S856: Screenshot of PacBio reads aligned to each of the putative locations of the *PTPRF* and *HYI* in white footed mouse (*Peromyscus leucopus*). Gray boxes represent reads aligned to the genome. Raw read datasets which is mentioned above have been mapped to the GCF_004664715.2_UCI_PerLeu_2.1_genomic.fna.

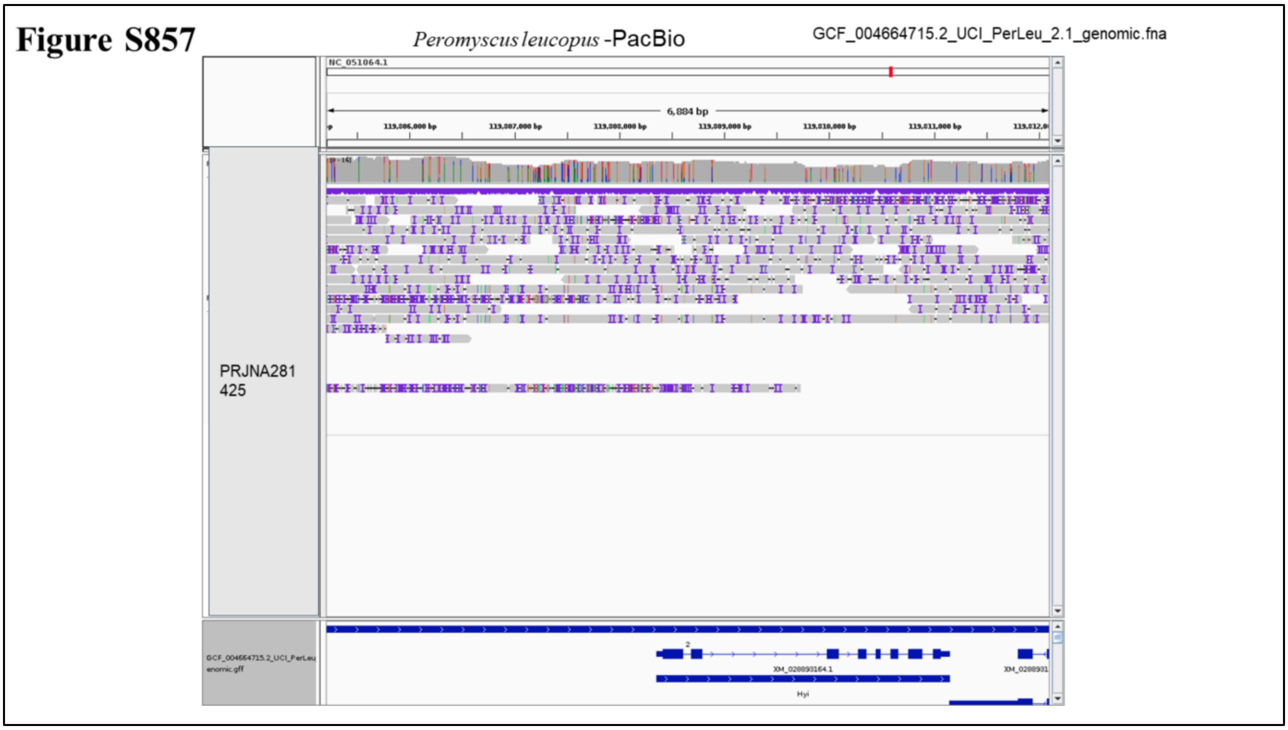

Figure S857: Screenshot of PacBio reads aligned to each of the putative locations of the *PTPRF* and *HYI* in white footed mouse (*Peromyscus leucopus*). Gray boxes represent reads aligned to the genome. Raw read datasets which is mentioned above have been mapped to the GCF_004664715.2_UCI_PerLeu_2.1_genomic.fna.

Figure S858

Mus musculus

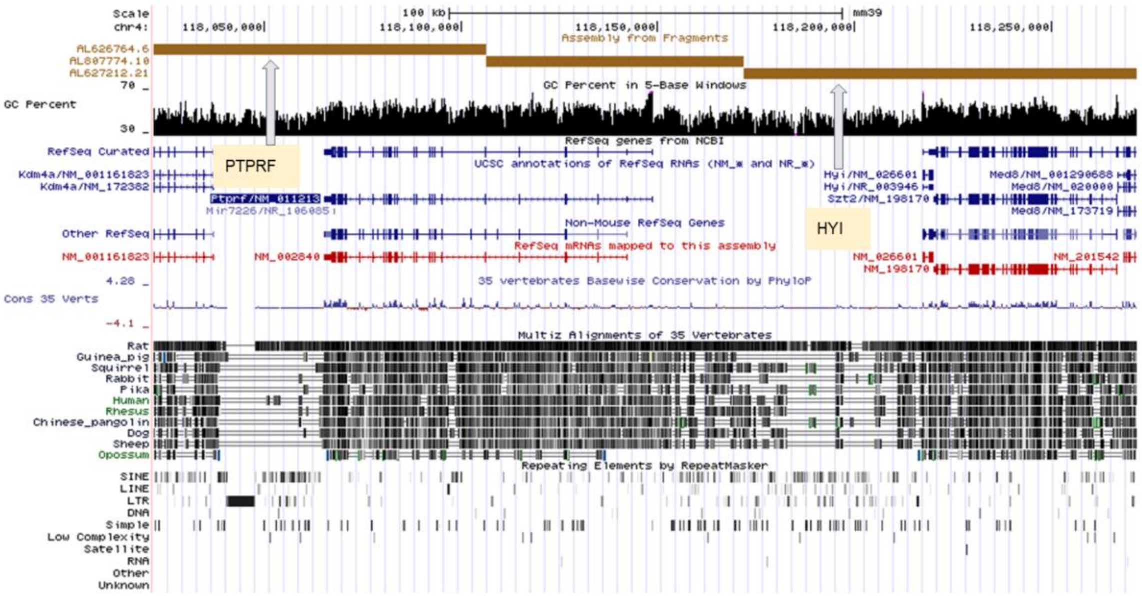

Figure S858: Screenshot of UCSC genome browser of the *PTPRF* and *HYI* in mouse (*Mus musculus*). Arrows are indicating the clones which used in genome assembly and covering these genes.

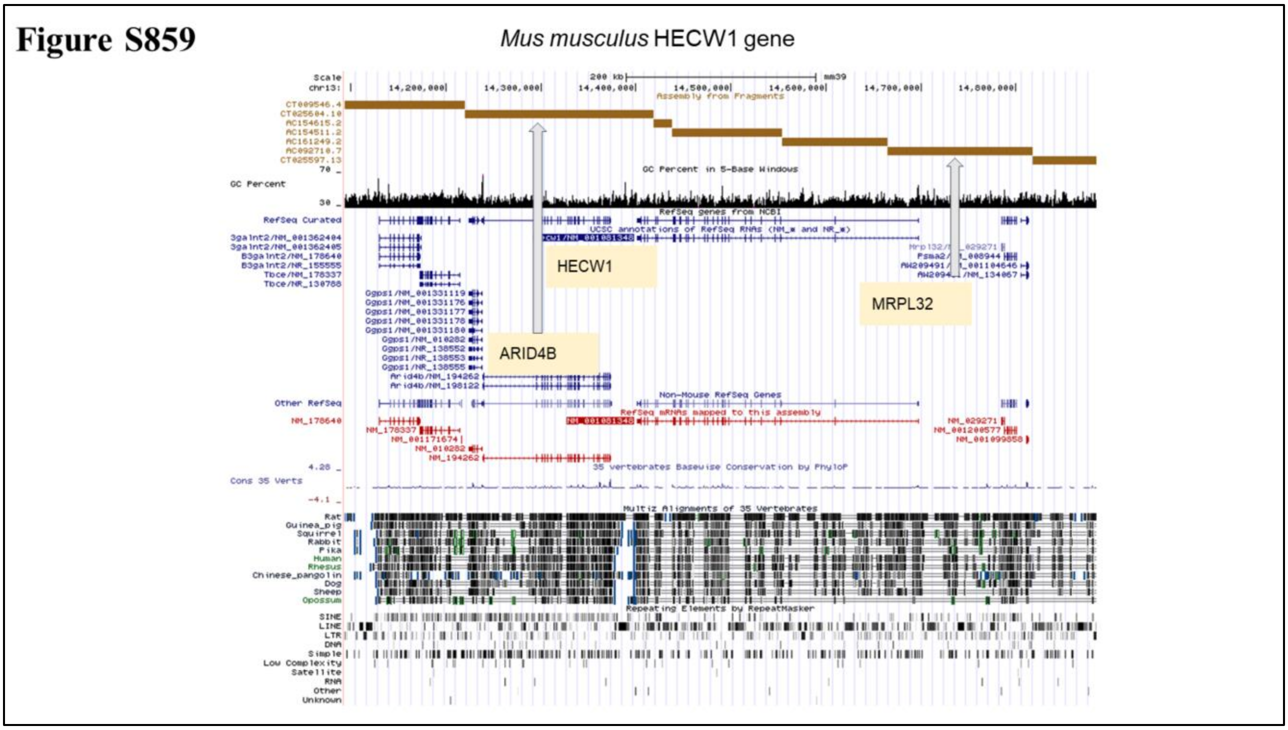

Figure S859: Screenshot of UCSC genome browser of the *ARID4B*, *HECW1* and *MRPL32* in mouse (*Mus musculus*). Arrows are indicating the clones which used in genome assembly and covering these genes.

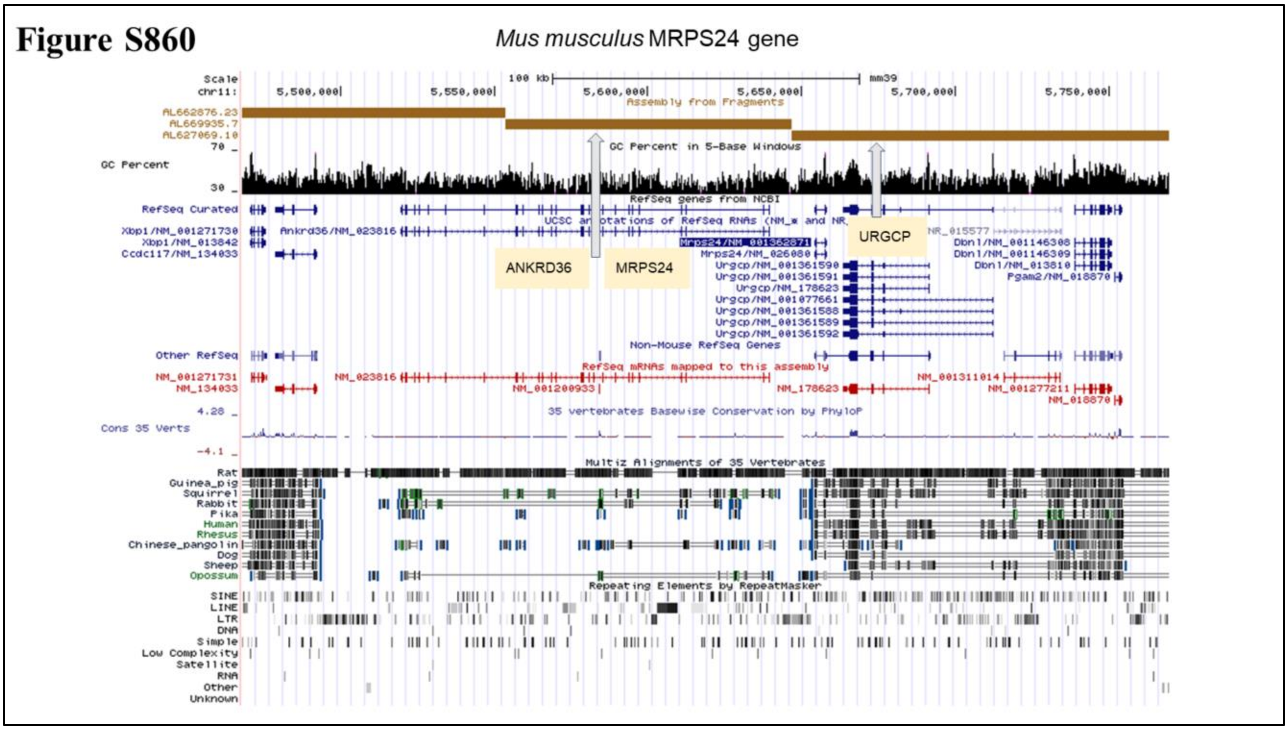

Figure S860: Screenshot of UCSC genome browser of the *ANKRD36*, *MRPS24* and *URGCP* in mouse (*Mus musculus*). Arrows are indicating the clones which used in genome assembly and covering these genes.

Figure S861

Mus musculus BLVRA gene

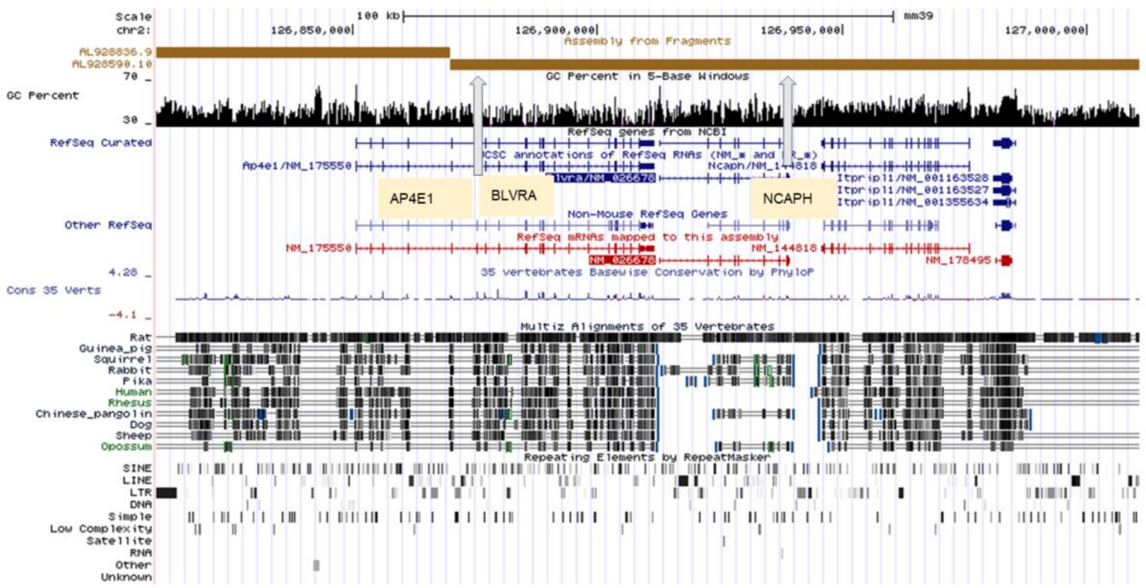

Figure S861: Screenshot of UCSC genome browser of the *AP4E1*, *BLVRA* and *NCAPH* in mouse (*Mus musculus*). Arrows are indicating the clones which used in genome assembly and covering these genes.

Figure S862

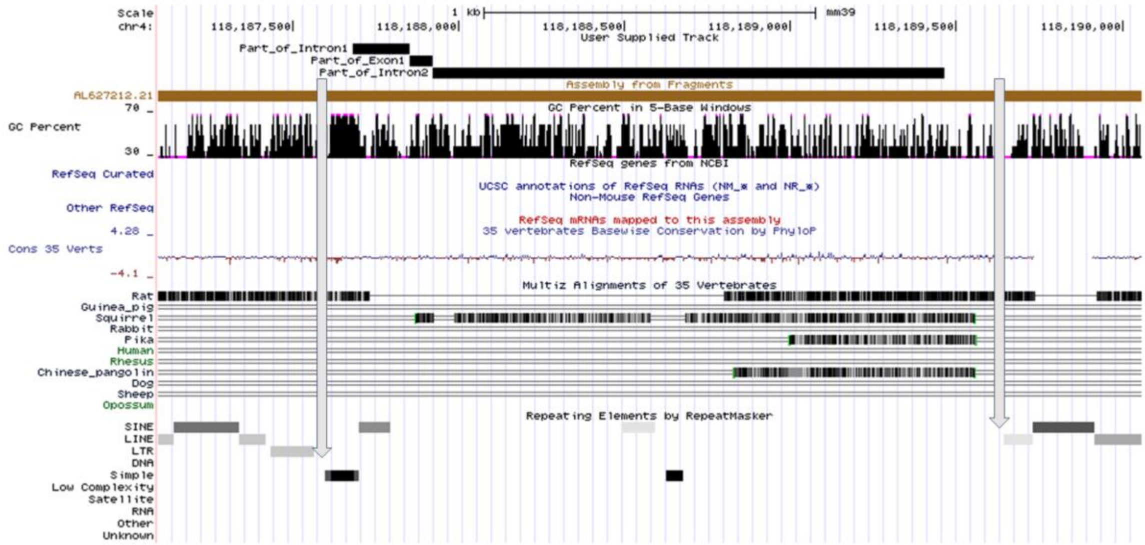

Figure S862: Screenshot of UCSC genome browser of the Intergenic region between *PTPRF* and *HYI* in mouse (*Mus musculus*), where getting the partial hits for red squirrel's exon 1, intron-1 and 2 of *COA1*. Arrows are indicating the repeats which are present at the ends of these regions.

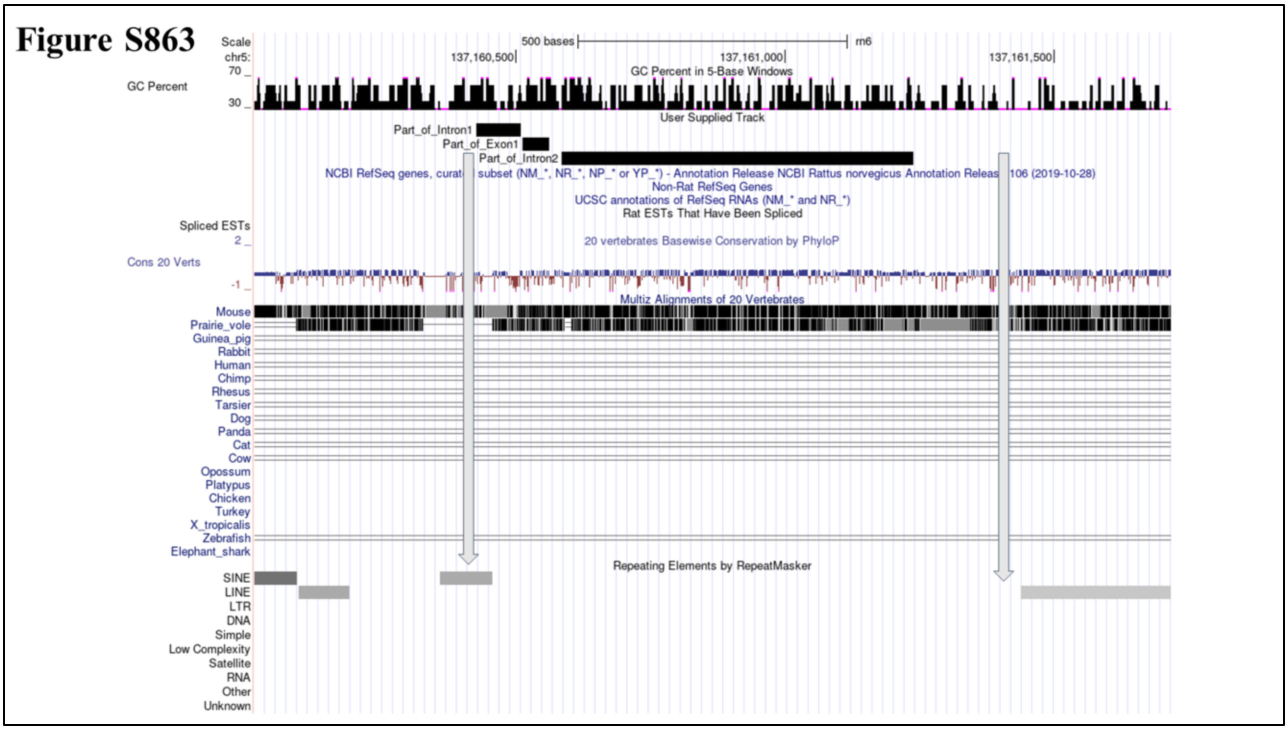

Figure S863: Screenshot of UCSC genome browser of the Intergenic region between *PTPRF* and *HYI* in rat (*Rattus norvegicus*), where getting the partial hits for red squirrel's exon 1, intron-1 and 2 of *COA1*. Arrows are indicating the repeats which are present at the ends of these regions.

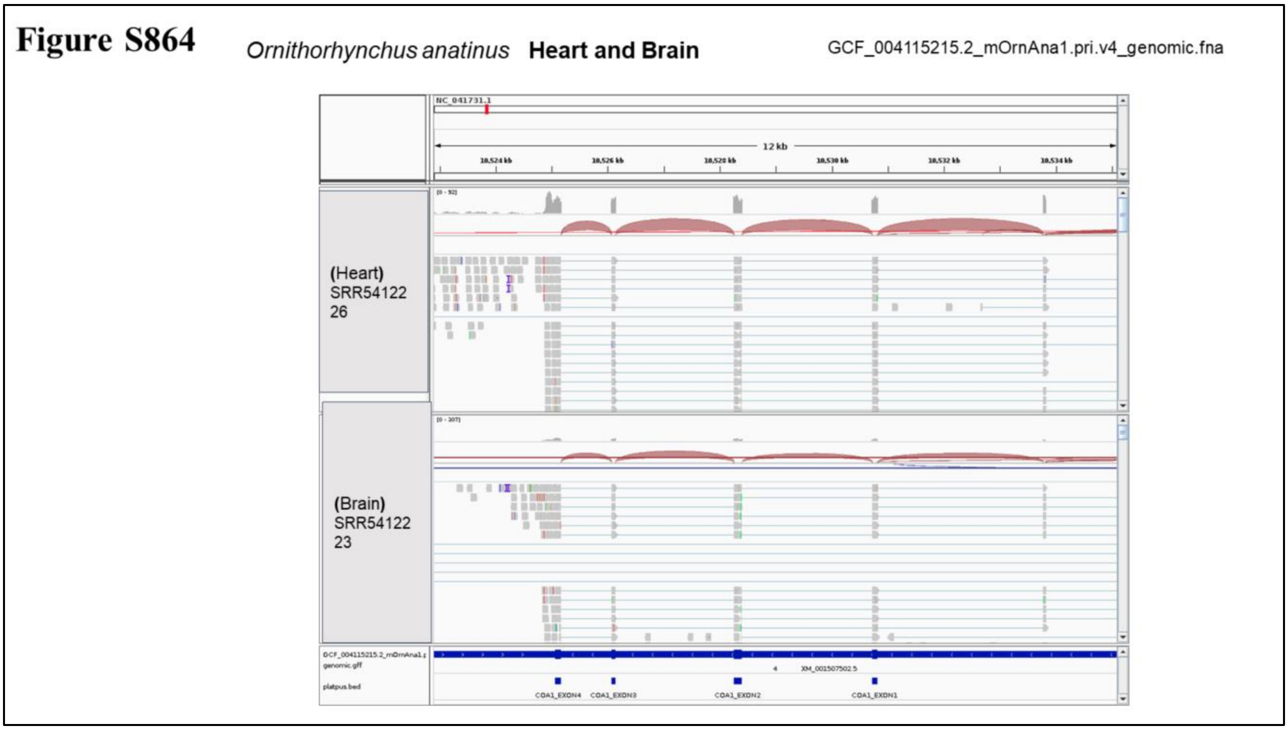

Figure S864: Screenshot of RNA-seq reads aligned to each of the putative locations of the four *COA1* exons in platypus (*Ornithorhynchus anatinus*). Gray boxes represent reads aligned to the genome and thin lines connecting these boxes represent the spliced read alignments. Datasets from heart and brain (SRR5412226 and SRR5412223) have been mapped to the GCF_004115215.2_mOrnAna1.pri.v4_genomic.fna. Locations of each of the exons are depicted as bed record in the bottom row.

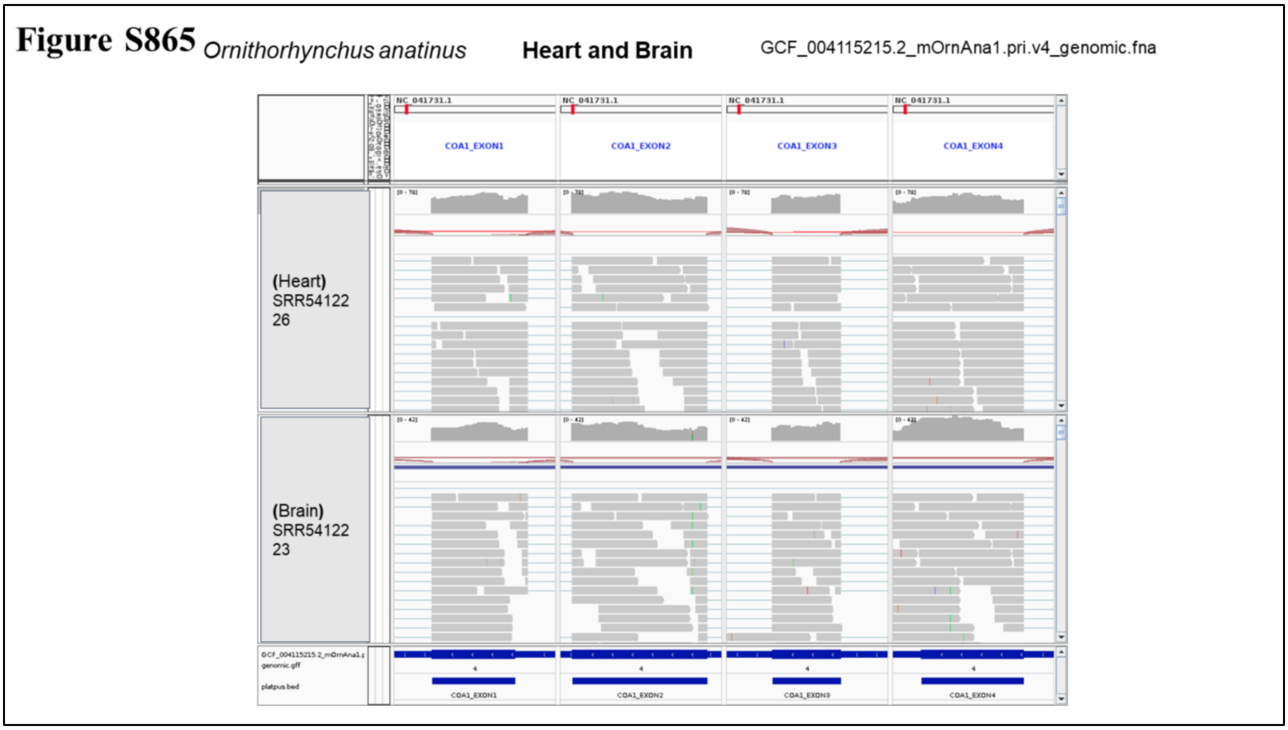

Figure S865: A zoomed in view of each of the *COA1* exons shown in **Supplementary Fig. S864**.

Figure S866 *Ornithorhynchus anatinus* **Heart and Brain** GCF_004115215.2_mOrnAna1.pri.v4_genomic.fna

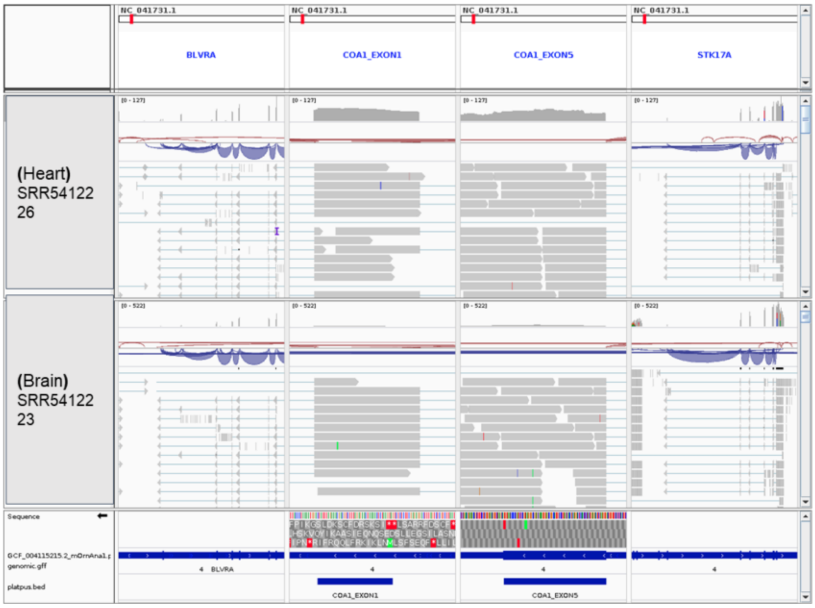

Figure S866: Screenshot of RNA-seq reads aligned to *BLVRA* and *STK17A* to *COA1* exon 1 and 4 respectively in platypus (*Ornithorhynchus anatinus*). Gray boxes represent reads aligned to the genome and thin lines connecting these boxes represent the spliced read alignments. Datasets from heart and brain (SRR5412226 and SRR5412223) have been mapped to the GCF_004115215.2_mOrnAna1.pri.v4_genomic.fna. Locations of each of the exons are depicted as bed record in the bottom row.the bottom row.

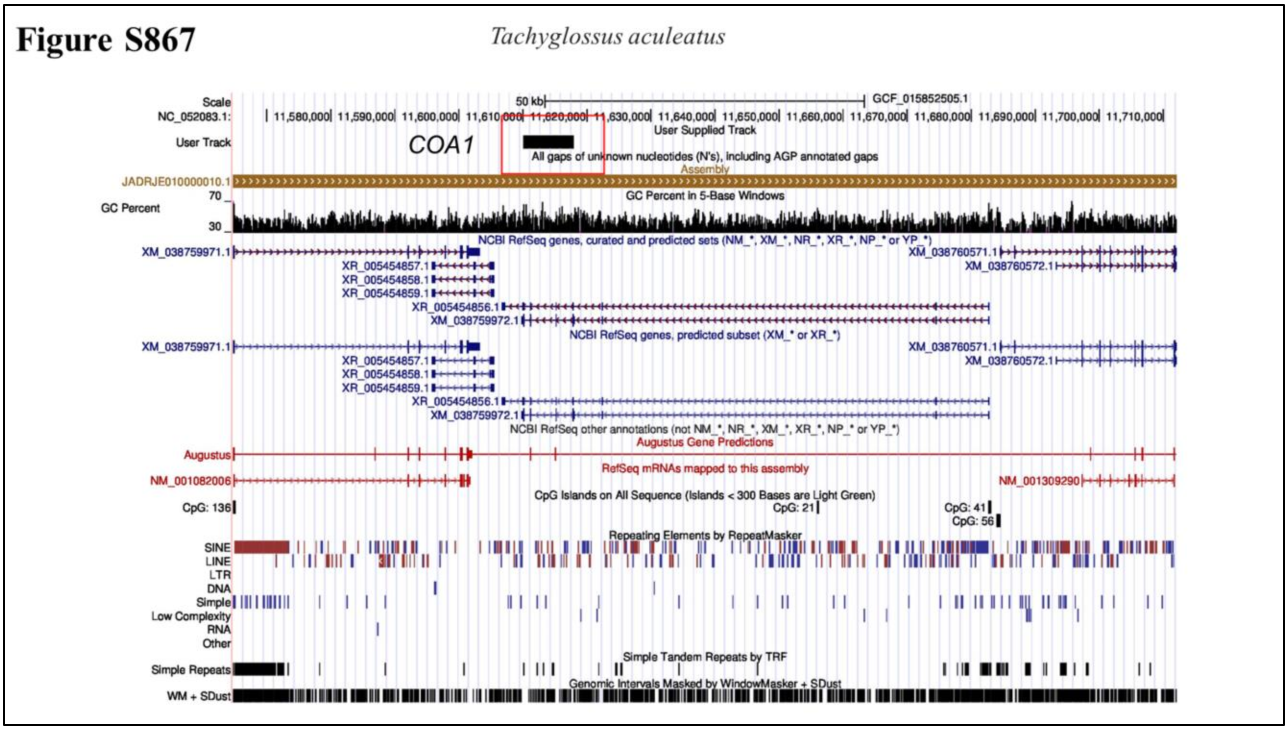

Figure S867: Screenshot of UCSC genome browser of the *COA1* in Australian echidna (*Tachyglossus aculeatus*).

Figure S868

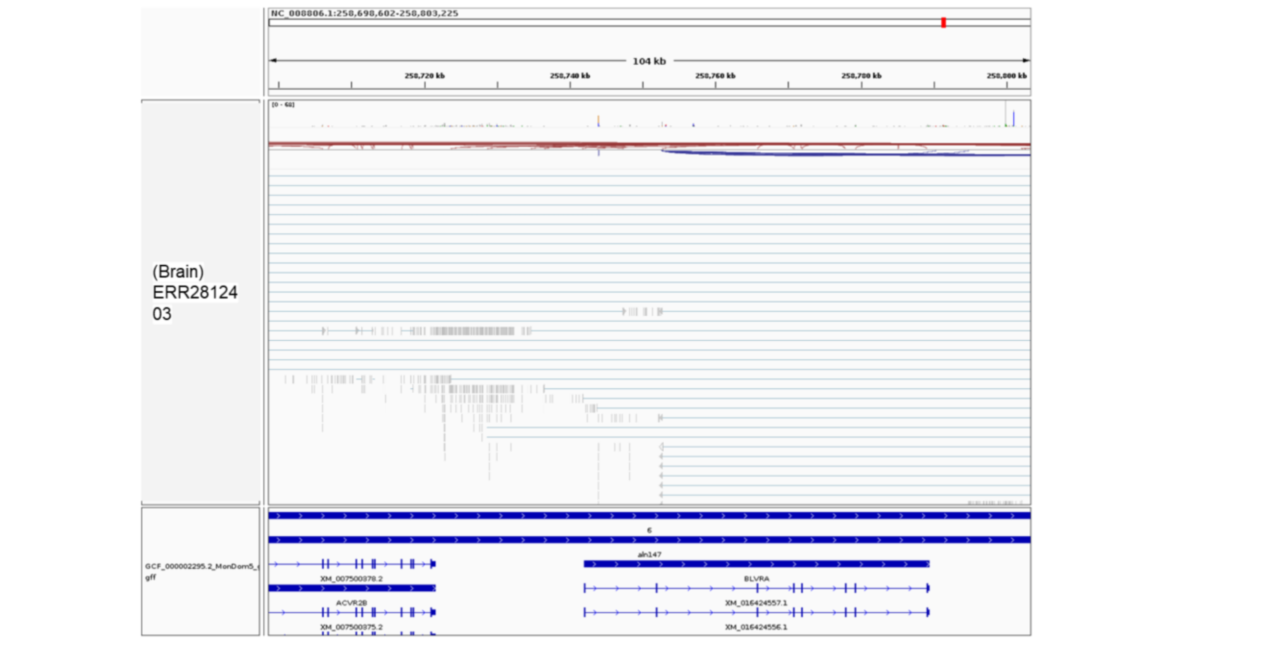

Figure S868: Screenshot of RNA-seq reads aligned each of the putative locations of *ACVR2B* and *BLVRA* in gray short-tailed opossum (*Monodelphis domestica*). Gray boxes represent reads aligned to the genome and thin lines connecting these boxes represent the spliced read alignments. Datasets from brain (ERR2812403) have been mapped to the GCF_000002295.2_MonDom5_genomic.fna. Locations of *ACVR2B* and *BLVRA* are depicted as GCF_000002295.2_MonDom5_genomic.gff record in the bottom row.

Figure S869

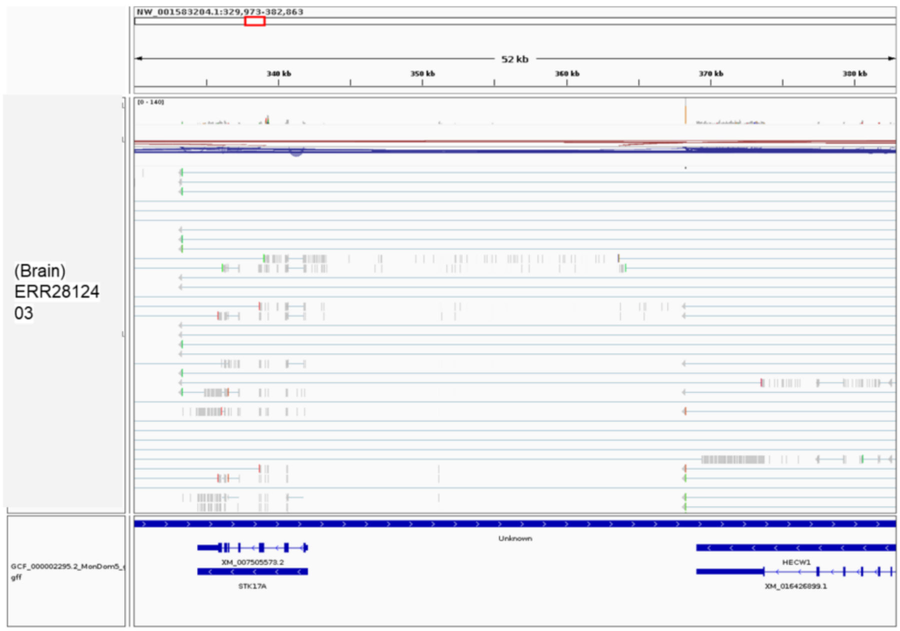

Figure S869: Screenshot of RNA-seq reads aligned each of the putative locations of *STK17A* and *HECW1* in gray short-tailed opossum (*Monodelphis domestica*). Gray boxes represent reads aligned to the genome and thin lines connecting these boxes represent the spliced read alignments. Datasets from brain (ERR2812403) have been mapped to the GCF_000002295.2_MonDom5_genomic.fna. Locations of *STK17A* and *HECW1* are depicted as GCF_000002295.2_MonDom5_genomic.gff record in the bottom row.

Figure S870

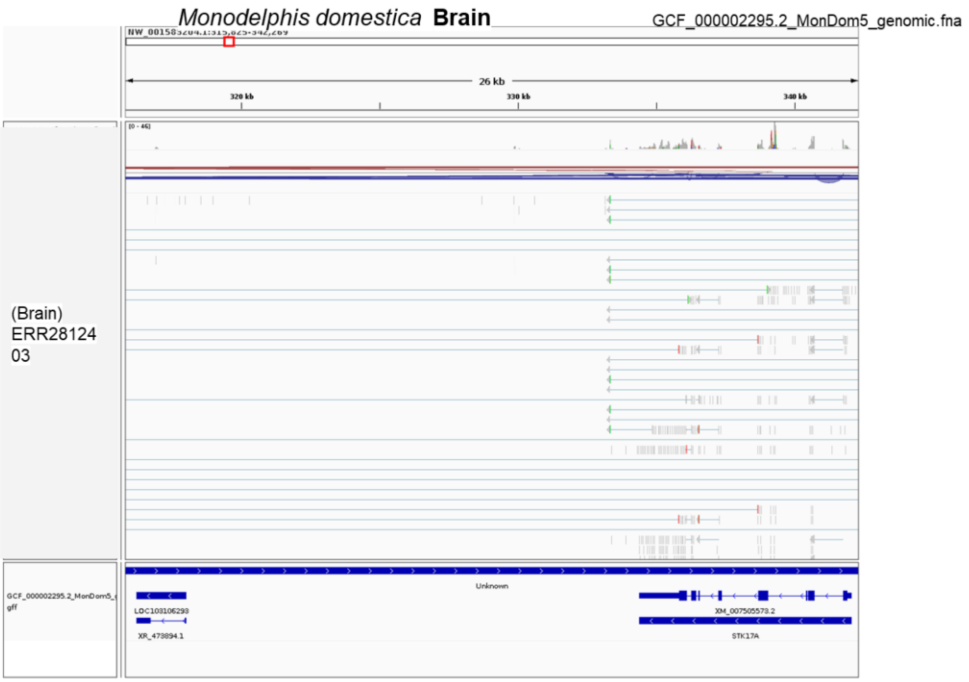

Figure S870: Screenshot of RNA-seq reads aligned each of the putative locations of *STK17A* and *GPR141B* in gray short-tailed opossum (*Monodelphis domestica*). Gray boxes represent reads aligned to the genome and thin lines connecting these boxes represent the spliced read alignments. Datasets from brain (ERR2812403) have been mapped to the GCF_000002295.2_MonDom5_genomic.fna. Locations of *STK17A* and *GPR141B* are depicted as GCF_000002295.2_MonDom5_genomic.gff record in the bottom row.

Figure S871

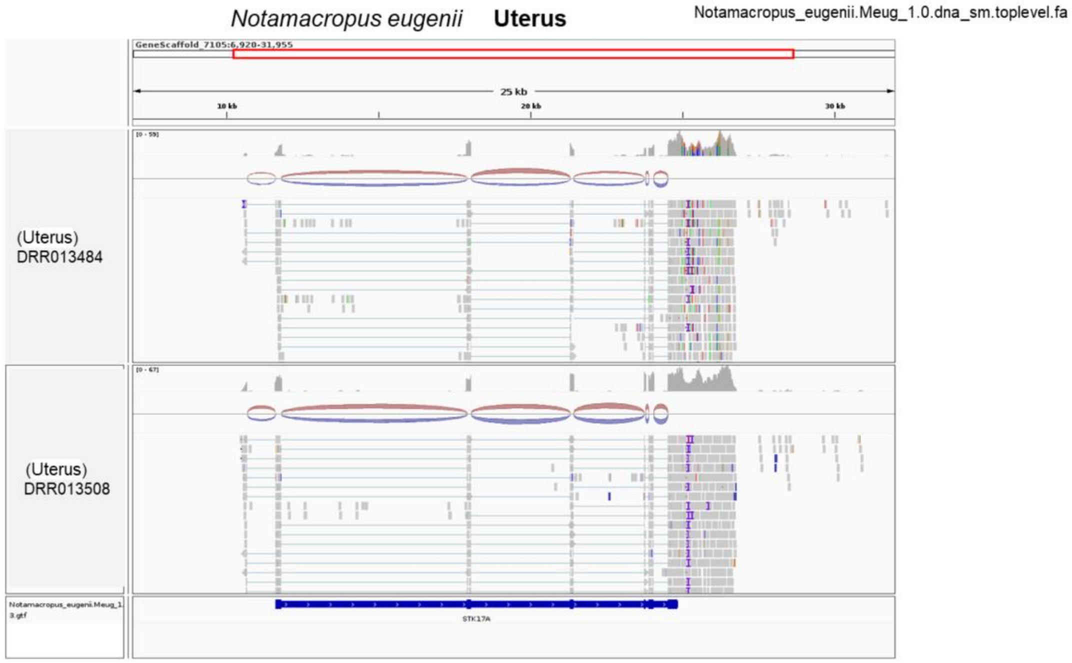

Figure S871: Screenshot of RNA-seq reads aligned each of the putative locations of *STK17A* in tammar wallaby (*Notamacropus eugenii*). Gray boxes represent reads aligned to the genome and thin lines connecting these boxes represent the spliced read alignments. Datasets from uterus (DRR013484 and DRR013508) have been mapped to the Notamacropus_eugenii.Meug_1.0.dna_sm.toplevel.fa. Locations of *STK17A* are depicted as Notamacropus_eugenii.Meug_1.0.103.gtf record in the bottom row.

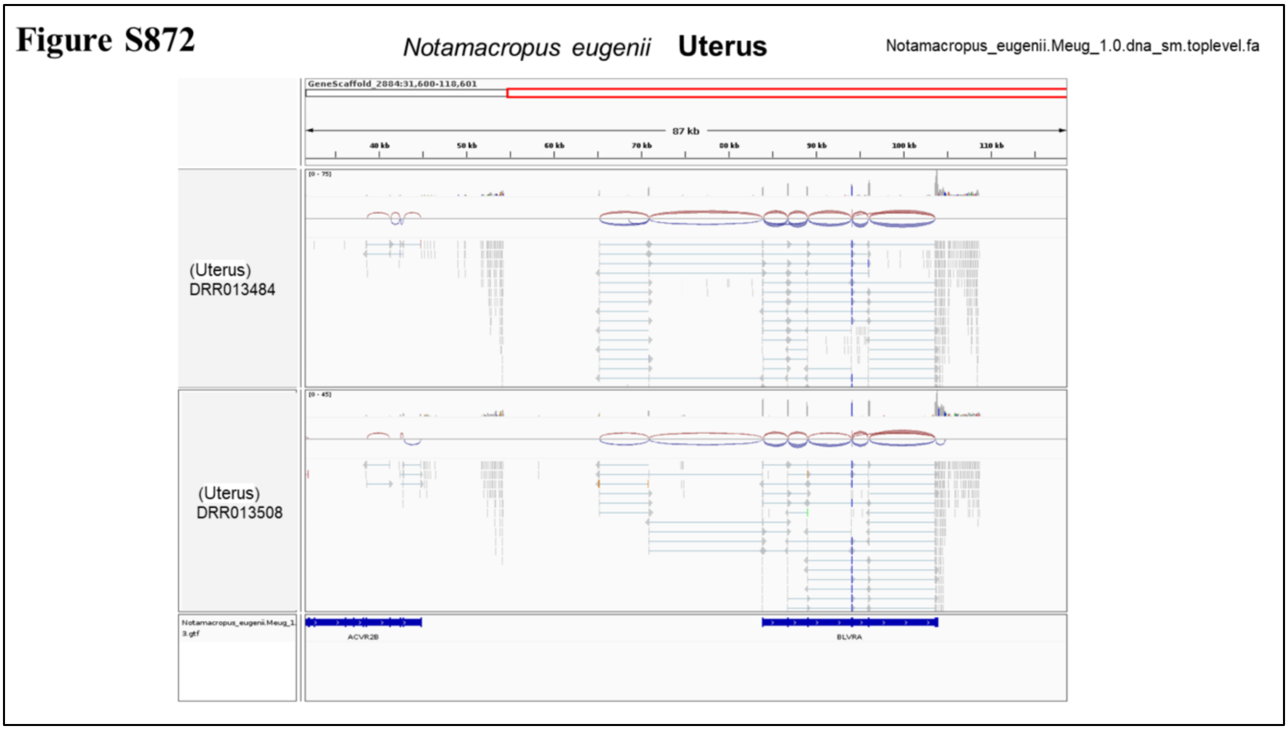

Figure S872: Screenshot of RNA-seq reads aligned each of the putative locations of *ACVR2B* and *BLVRA* in tammar wallaby (*Notamacropus eugenii*). Gray boxes represent reads aligned to the genome and thin lines connecting these boxes represent the spliced read alignments. Datasets from uterus (DRR013484 and DRR013508) have been mapped to the Notamacropus_eugenii.Meug_1.0.dna_sm.toplevel.fa. Locations of *ACVR2B* and *BLVRA* are depicted as Notamacropus_eugenii.Meug_1.0.103.gtf record in the bottom row.

Figure S873

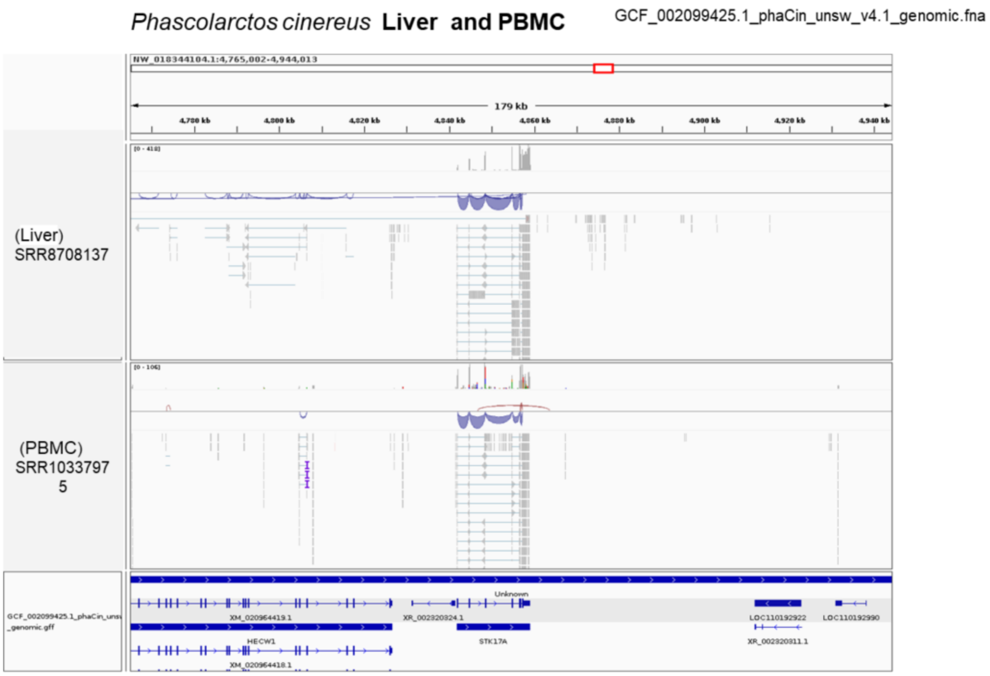

Figure S873: Screenshot of RNA-seq reads aligned each of the putative locations of *HECW1* and *STK17A* in koala (*Phascolarctos cinereus*). Gray boxes represent reads aligned to the genome and thin lines connecting these boxes represent the spliced read alignments. Datasets from liver (SRR8708137) and PBMC (SRR10337975) have been mapped to the GCF_002099425.1_phaCin_unsw_v4.1_genomic.fna. Locations of *HECW1* and *STK17A* are depicted as GCF_002099425.1_phaCin_unsw_v4.1_genomic.gff record in the bottom row.

Figure S874 *Phascolarctos cinereus* **Liver and PBMC** GCF_002099425.1_phaCin_unsw_v4.1_genomic.fna

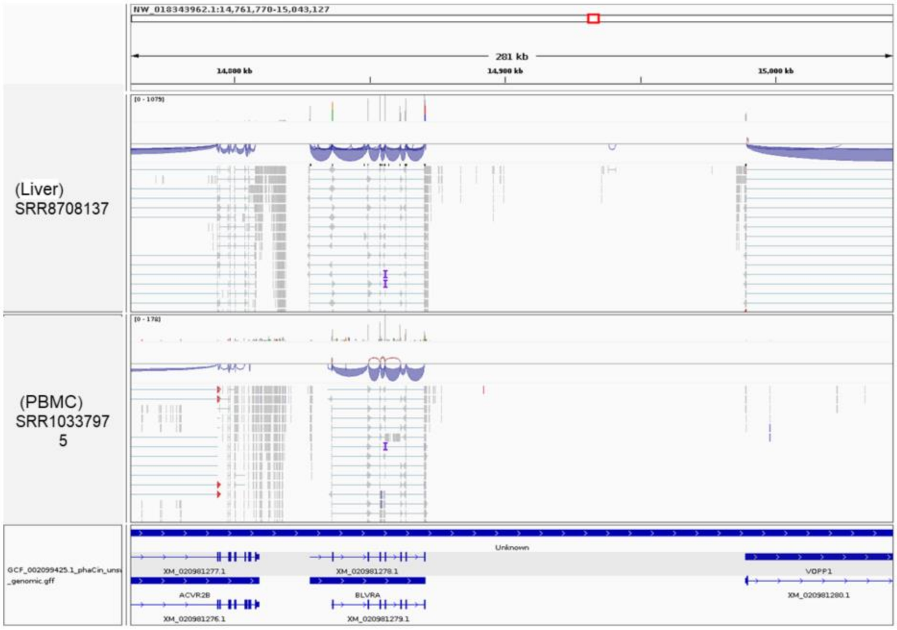

Figure S874: Screenshot of RNA-seq reads aligned each of the putative locations of *ACVR2B* and *VOPP1* in koala (*Phascolarctos cinereus*). Gray boxes represent reads aligned to the genome and thin lines connecting these boxes represent the spliced read alignments. Datasets from liver (SRR8708137) and PBMC (SRR10337975) have been mapped to the GCF_002099425.1_phaCin_unsw_v4.1_genomic.fna. Locations of *ACVR2B* and *VOPP1* are depicted as GCF_002099425.1_phaCin_unsw_v4.1_genomic.gff record in the bottom row.

Figure S875

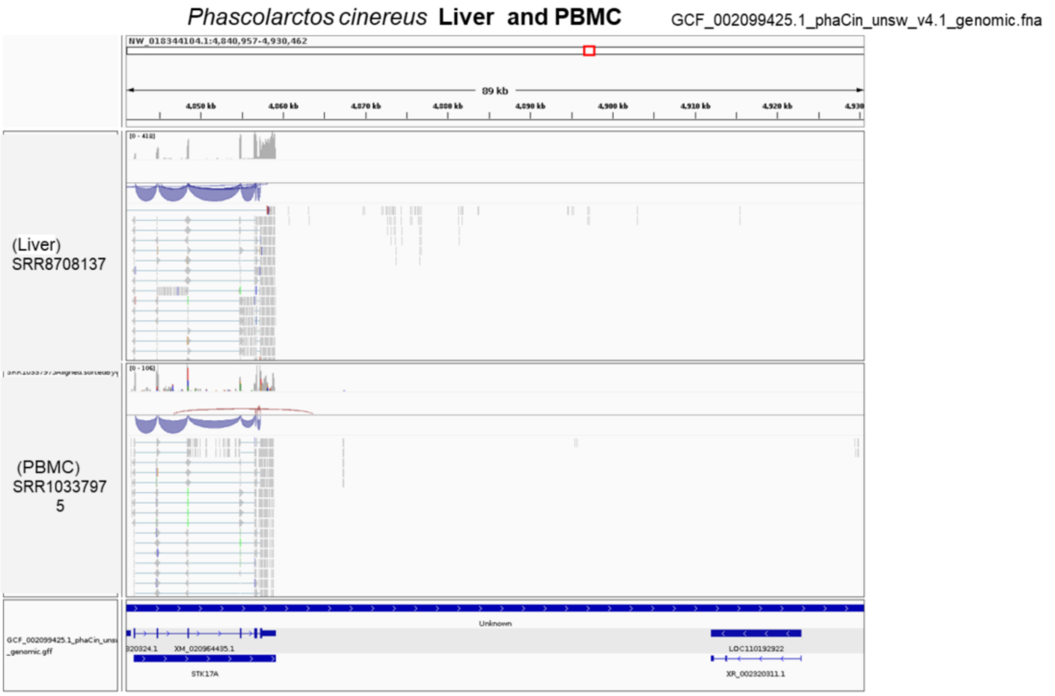

Figure S875: Screenshot of RNA-seq reads aligned each of the putative locations of *STK17A* and *GPR141B* in koala (*Phascolarctos cinereus*). Gray boxes represent reads aligned to the genome and thin lines connecting these boxes represent the spliced read alignments. Datasets from liver (SRR8708137) and PBMC (SRR10337975) have been mapped to the GCF_002099425.1_phaCin_unsw_v4.1_genomic.fna. Locations of *STK17A* and *GPR141B* are depicted as GCF_002099425.1_phaCin_unsw_v4.1_genomic.gff record in the bottom row.

Figure S876

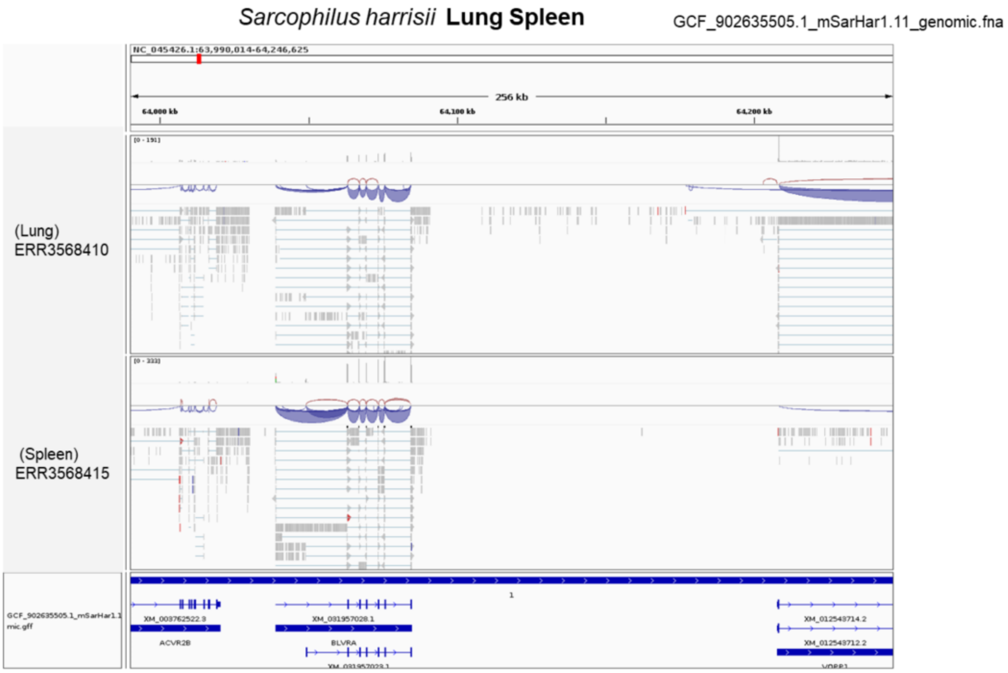

Figure S876: Screenshot of RNA-seq reads aligned each of the putative locations of *ACVR2B* and *VOPP1* in Tasmanian devil (*Sarcophilus harrisii*). Gray boxes represent reads aligned to the genome and thin lines connecting these boxes represent the spliced read alignments. Datasets from lung (ERR3568410) and spleen (ERR3568415) have been mapped to the GCF_902635505.1_mSarHar1.11_genomic.fna. Locations of *ACVR2B* and *VOPP1* are depicted as GCF_902635505.1_mSarHar1.11_genomic.gff record in the bottom row.

Figure S877

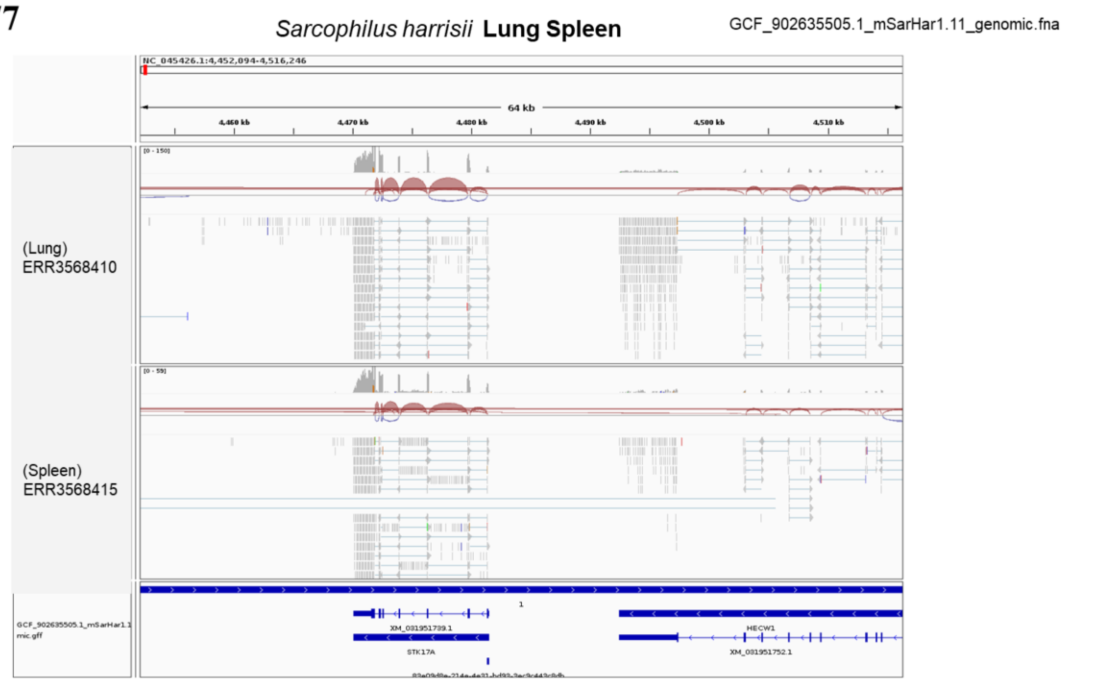

Figure S877: Screenshot of RNA-seq reads aligned each of the putative locations of *STK17A* and *HECW1* in Tasmanian devil (*Sarcophilus harrisii*). Gray boxes represent reads aligned to the genome and thin lines connecting these boxes represent the spliced read alignments. Datasets from lung (ERR3568410) and spleen (ERR3568415) have been mapped to the GCF_902635505.1_mSarHar1.11_genomic.fna. Locations of *STK17A* and *HECW1* are depicted as GCF_902635505.1_mSarHar1.11_genomic.gff record in the bottom row.

Figure S878

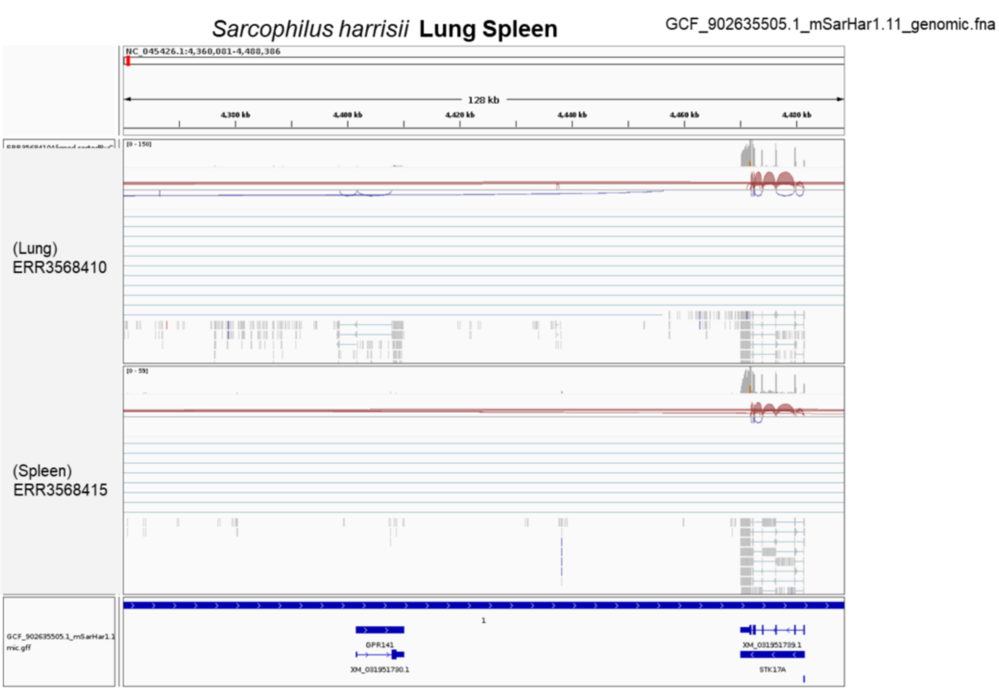

Figure S878: Screenshot of RNA-seq reads aligned each of the putative locations of *STK17A* and *GPR141B* in Tasmanian devil (*Sarcophilus harrisii*). Gray boxes represent reads aligned to the genome and thin lines connecting these boxes represent the spliced read alignments. Datasets from lung (ERR3568410) and spleen (ERR3568415) have been mapped to the GCF_902635505.1_mSarHar1.11_genomic.fna. Locations of *STK17A* and *GPR141B* are depicted as GCF_902635505.1_mSarHar1.11_genomic.gff record in the bottom row.

Figure S879 *Trichosurus vulpecula* **Liver** GCF_011100635.1_mTriVul1.pri_genomic.fna

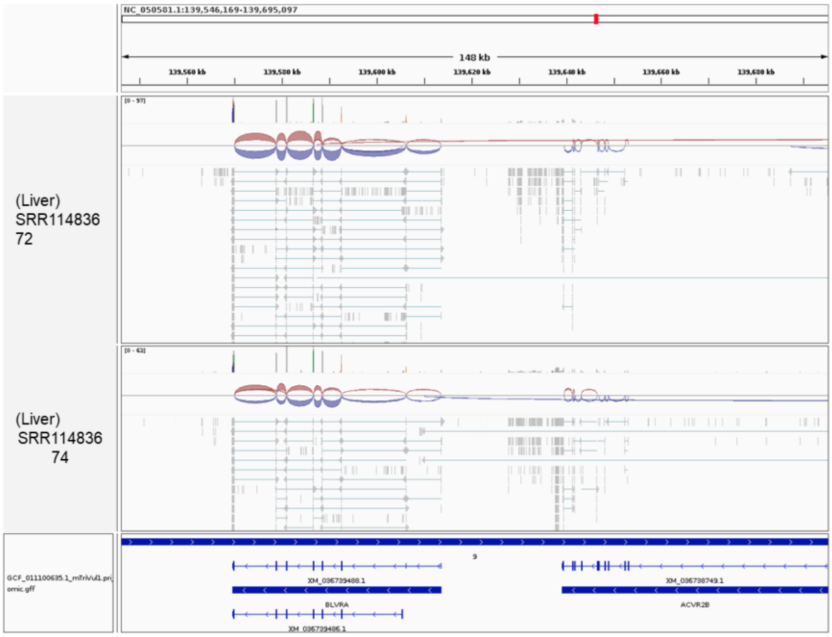

Figure S879: Screenshot of RNA-seq reads aligned each of the putative locations of *ACVR2B* and *BLVRA* in common brushtail (*Trichosurus vulpecula*). Gray boxes represent reads aligned to the genome and thin lines connecting these boxes represent the spliced read alignments. Datasets from liver (SRR11483672 and SRR11483674) have been mapped to the GCF_011100635.1_mTriVul1.pri_genomic.fna. Locations of *ACVR2B* and *BLVRA* are depicted as GCF_011100635.1_mTriVul1.pri_genomic.gff record in the bottom row.

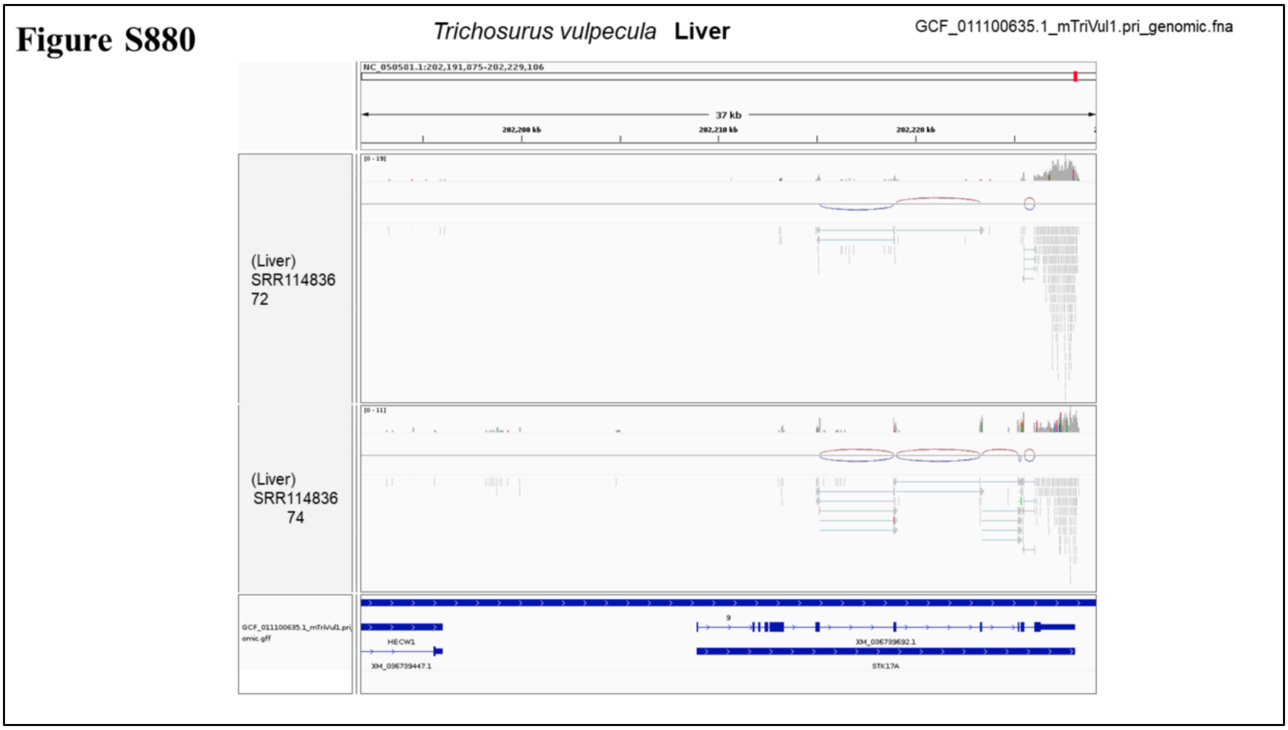

Figure S880: Screenshot of RNA-seq reads aligned each of the putative locations of *HECW1* and *STK17A* in common brushtail (*Trichosurus vulpecula*). Gray boxes represent reads aligned to the genome and thin lines connecting these boxes represent the spliced read alignments. Datasets from liver (SRR11483672 and SRR11483674) have been mapped to the GCF_011100635.1_mTriVul1.pri_genomic.fna. Locations of *HECW1* and *STK17A* are depicted as GCF_011100635.1_mTriVul1.pri_genomic.gff record in the bottom row.

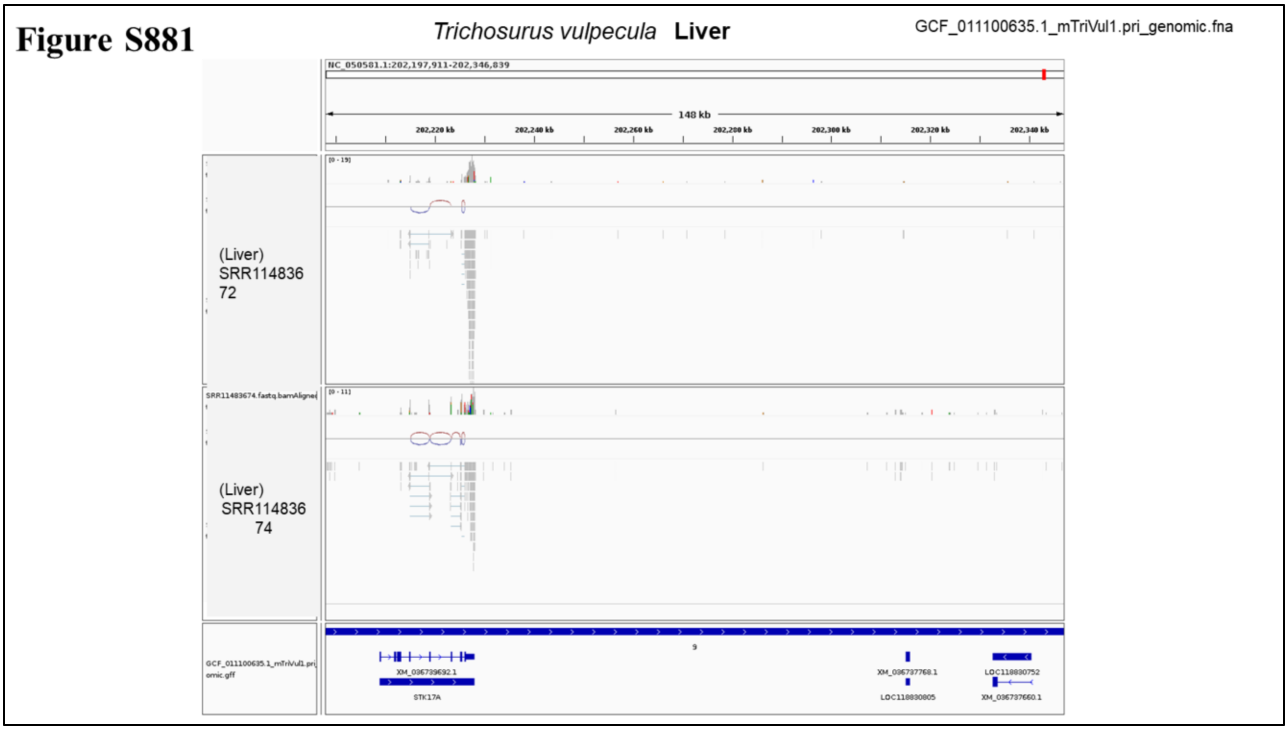

Figure S881: Screenshot of RNA-seq reads aligned each of the putative locations of *STK17A* and *GPR141B* in common brushtail (*Trichosurus vulpecula*). Gray boxes represent reads aligned to the genome and thin lines connecting these boxes represent the spliced read alignments. Datasets from liver (SRR11483672 and SRR11483674) have been mapped to the GCF_011100635.1_mTriVul1.pri_genomic.fna. Locations of *STK17A* and *GPR141B* are depicted as GCF_011100635.1_mTriVul1.pri_genomic.gff record in the bottom row.

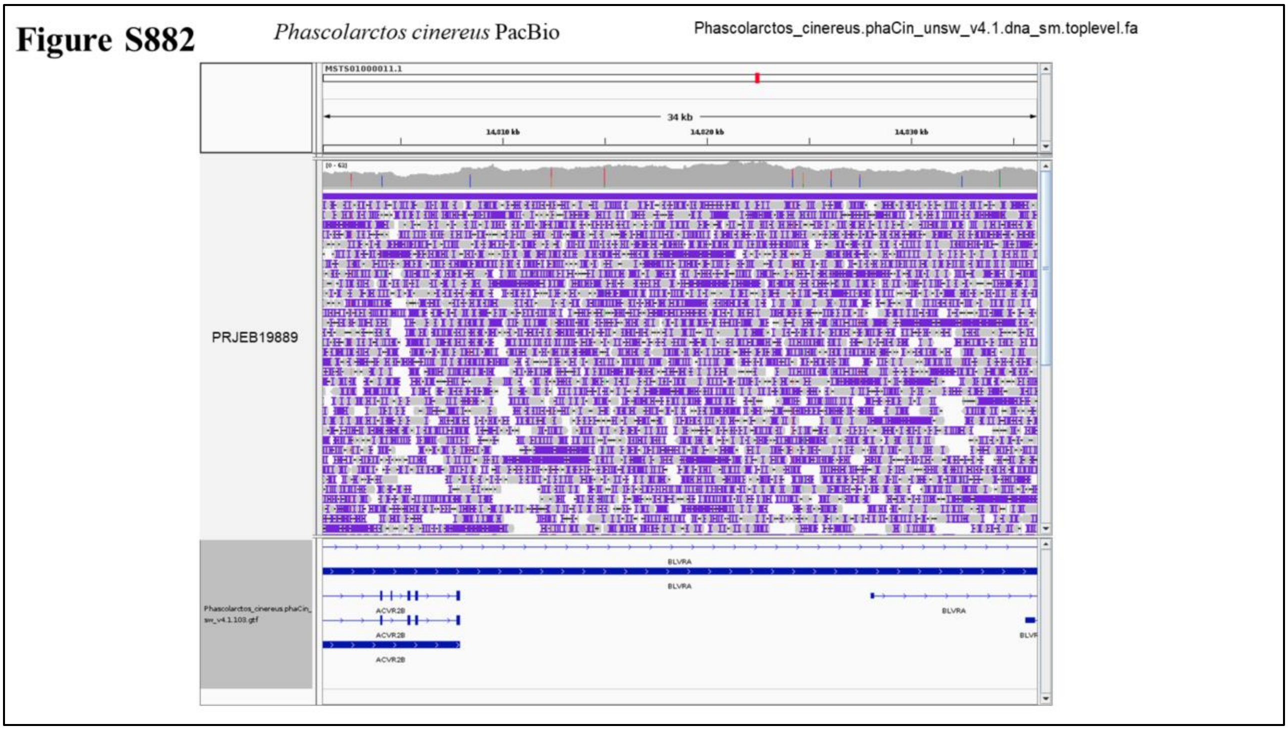

Figure S882: Screenshot of PacBio reads aligned to each of the putative locations of the *ACVR2B* and *BLVRA* in koala (*Phascolarctos cinereus*). Gray boxes represent reads aligned to the genome. Raw read datasets which is mentioned above have been mapped to the Phascolarctos_cinereus.phaCin_unsw_v4.1.dna_sm.toplevel.fa.

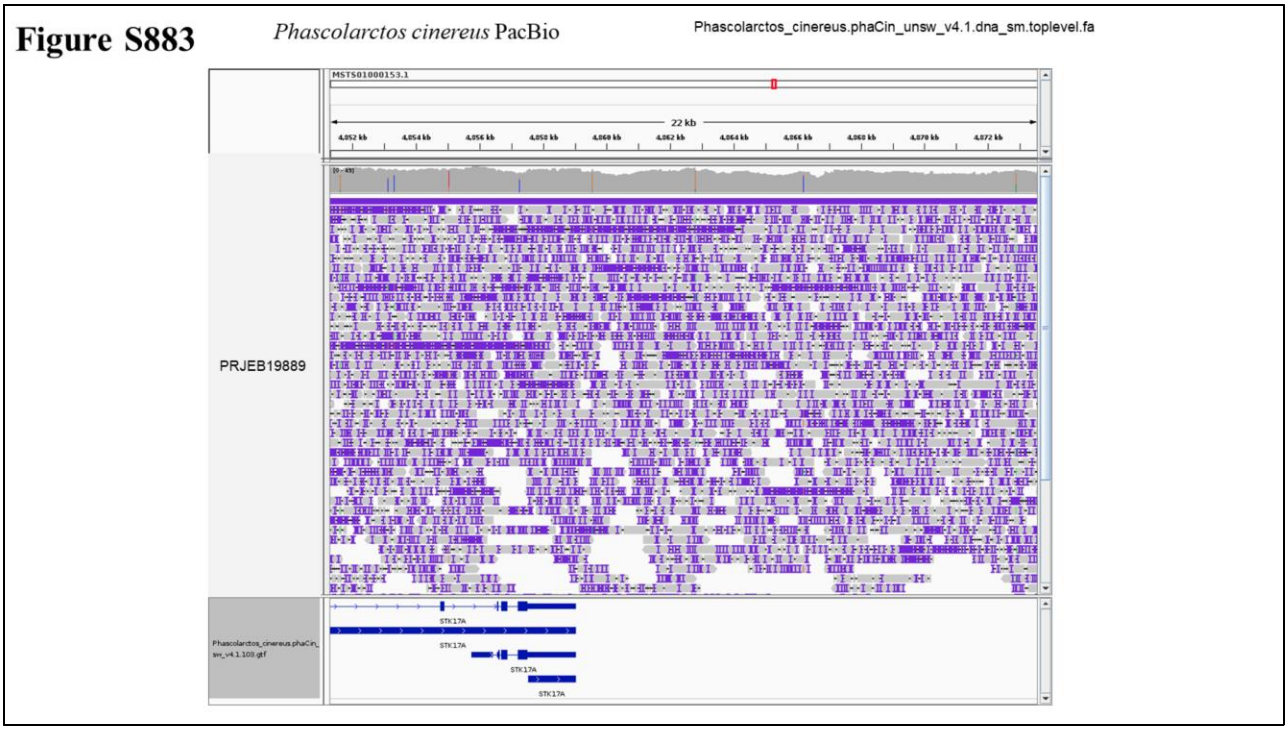

Figure S883: Screenshot of PacBio reads aligned to each of the putative locations of the *STK17A* and *GPR141B* in koala (*Phascolarctos cinereus*). Gray boxes represent reads aligned to the genome. Raw read datasets which is mentioned above have been mapped to the Phascolarctos_cinereus.phaCin_unsw_v4.1.dna_sm.toplevel.fa

.

Figure S884 *Phascolarctos cinereus* PacBio Phascolarctos_cinereus.phaCin_unsw_v4.1.dna_sm.toplevel.fa

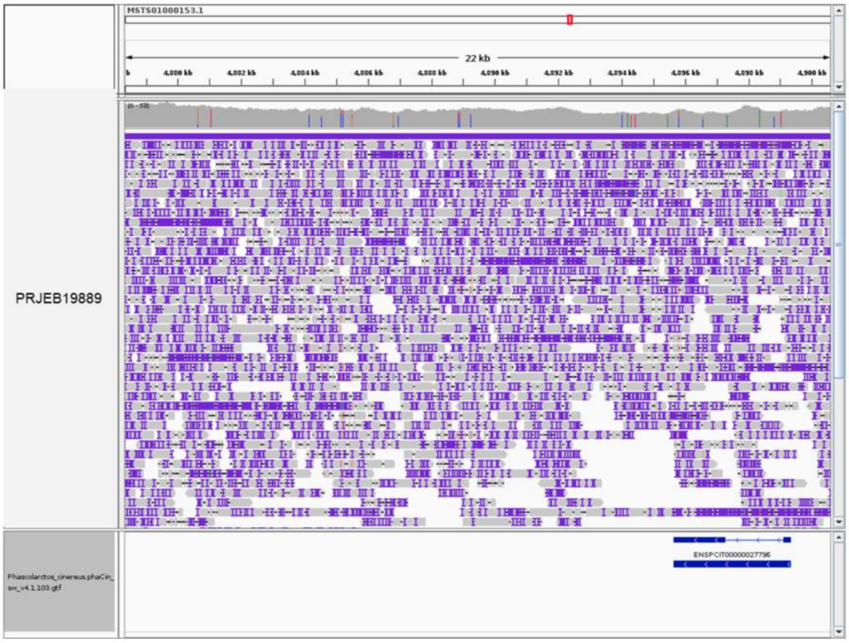

Figure S884: Screenshot of PacBio reads aligned to each of the putative locations of the *STK17A* and *GPR141B* in koala (*Phascolarctos cinereus*). Gray boxes represent reads aligned to the genome. Raw read datasets which is mentioned above have been mapped to the Phascolarctos_cinereus.phaCin_unsw_v4.1.dna_sm.toplevel.fa

.

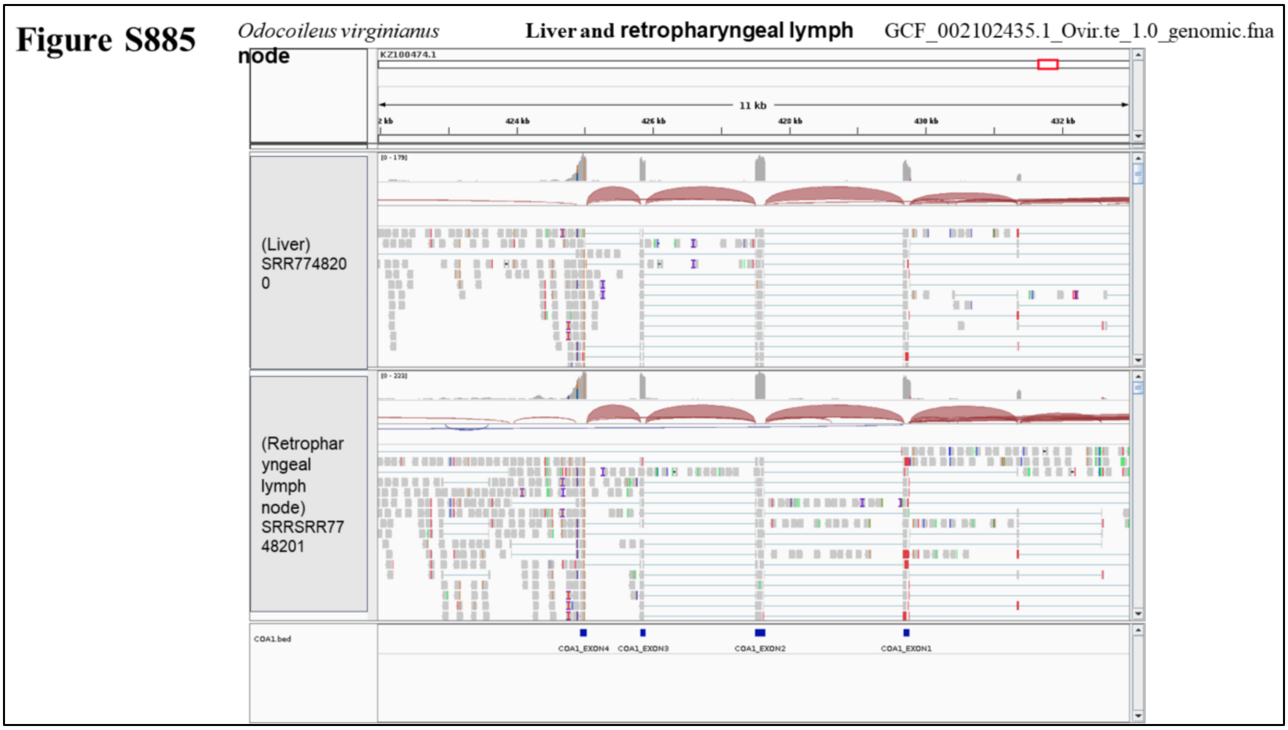

Figure S885: Screenshot of RNA-seq reads aligned to each of the putative locations of the *COA1* in white-tailed deer (*Odocoileus virginianus*). Gray boxes represent reads aligned to the genome and thin lines connecting these boxes represent the spliced read alignments. Datasets from liver and retropharyngeal lymph node (SRR7748200 and SRR7748201) have been mapped to the GCF_002102435.1_Ovir.te_1.0_genomic.fna. Locations of each of the exons are depicted as bed record in the bottom row.

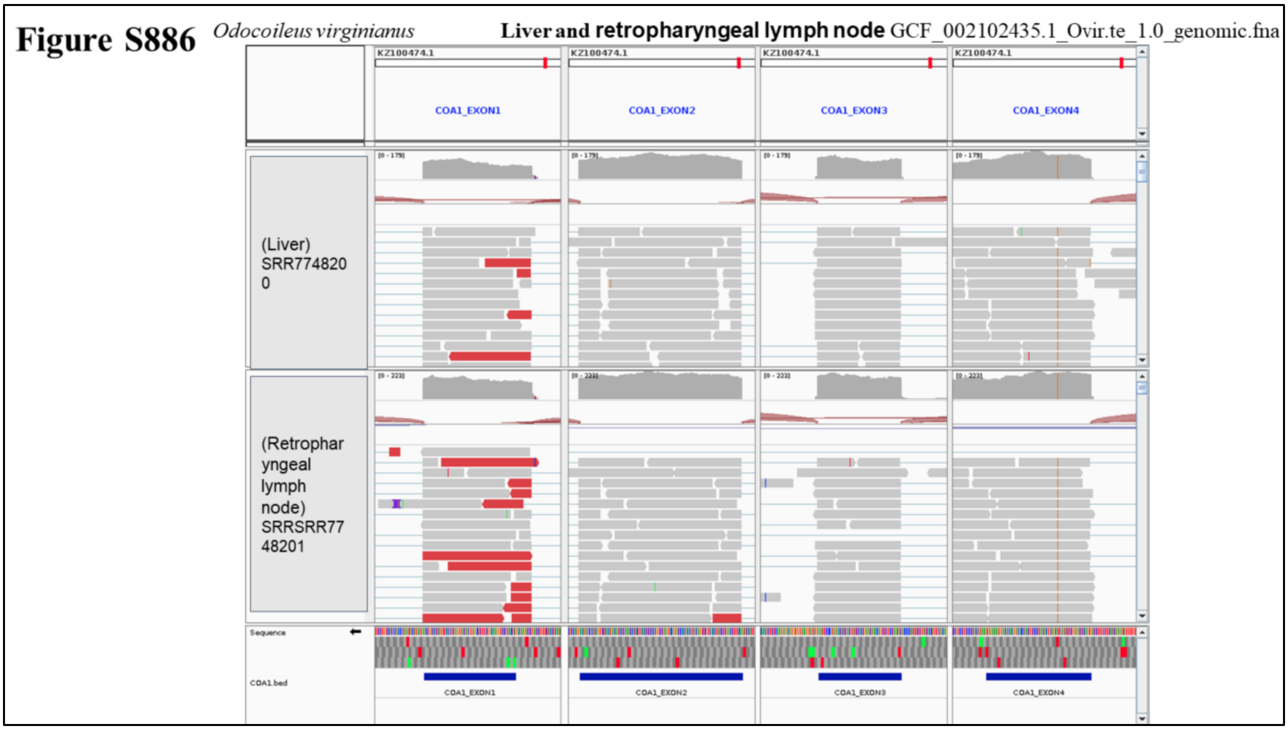

Figure S886: A zoomed in view of each of the *COA1* exons shown in **Supplementary Fig. S885**.

Figure S887 *Cervus elaphus* **Blood** GCA_002197005.1_CerEla1.0_genomic.fna

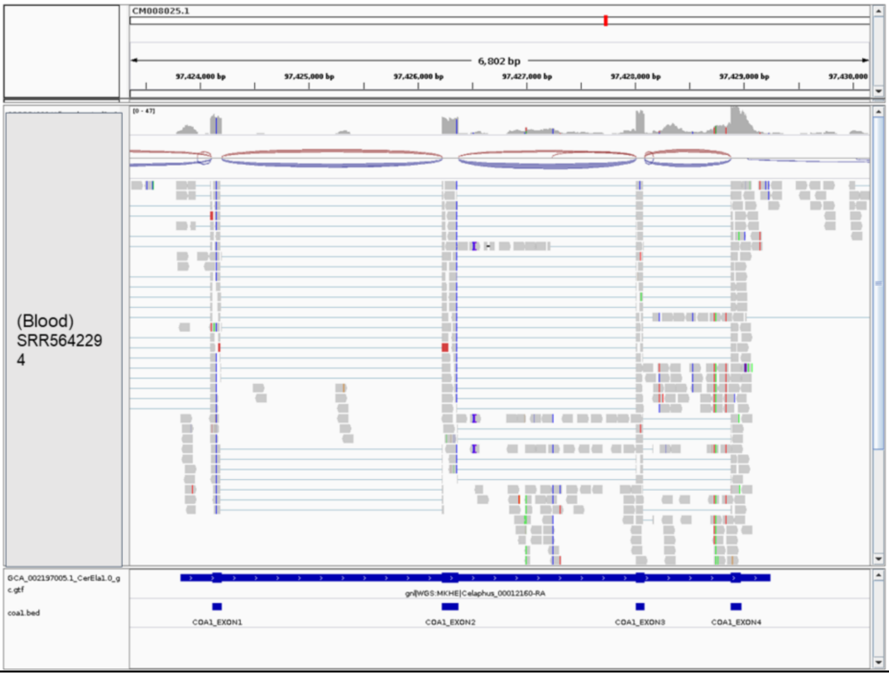

Figure S887: Screenshot of RNA-seq reads aligned to each of the putative locations of the *COA1* in red deer (*Cervus elaphus*). Gray boxes represent reads aligned to the genome and thin lines connecting these boxes represent the spliced read alignments. Datasets from blood (SRR5642294) have been mapped to the GCA_002197005.1_CerEla1.0_genomic.fna. Locations of each of the exons are depicted as bed record in the bottom row.

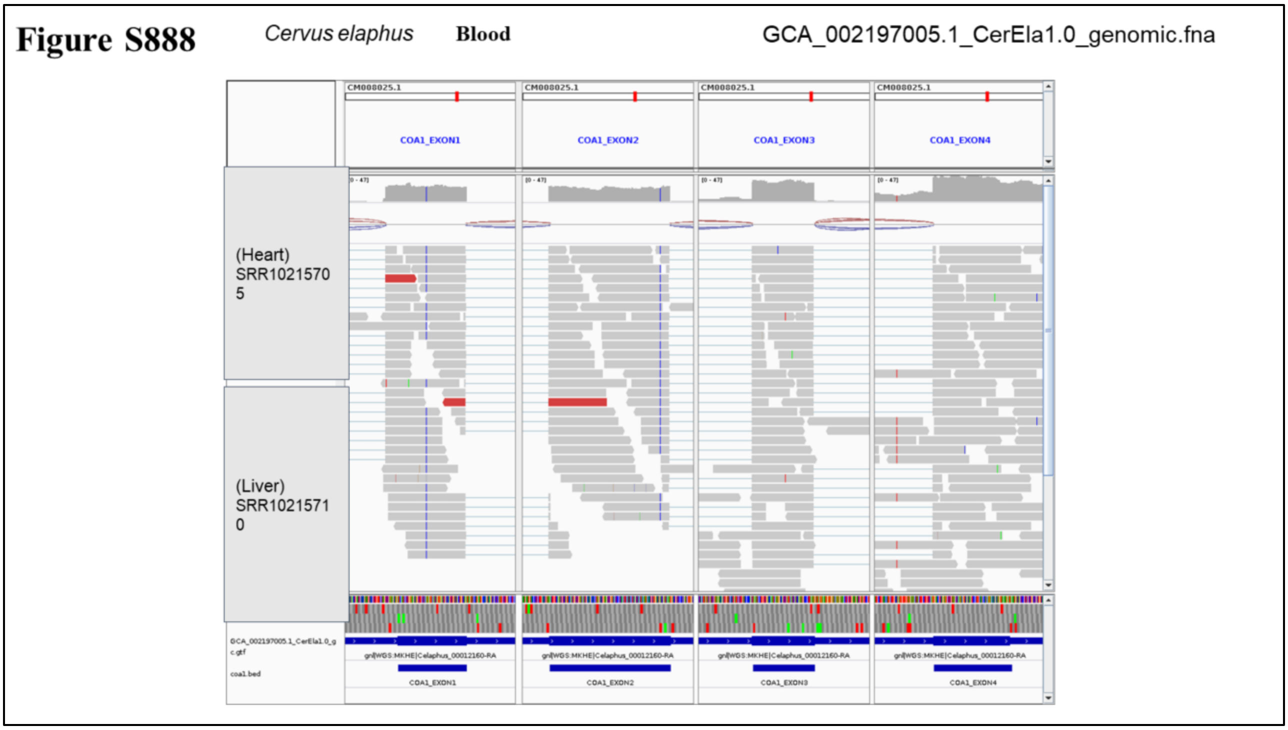

Figure S888: A zoomed in view of each of the *COA1* exons shown in **Supplementary Fig. S887**.

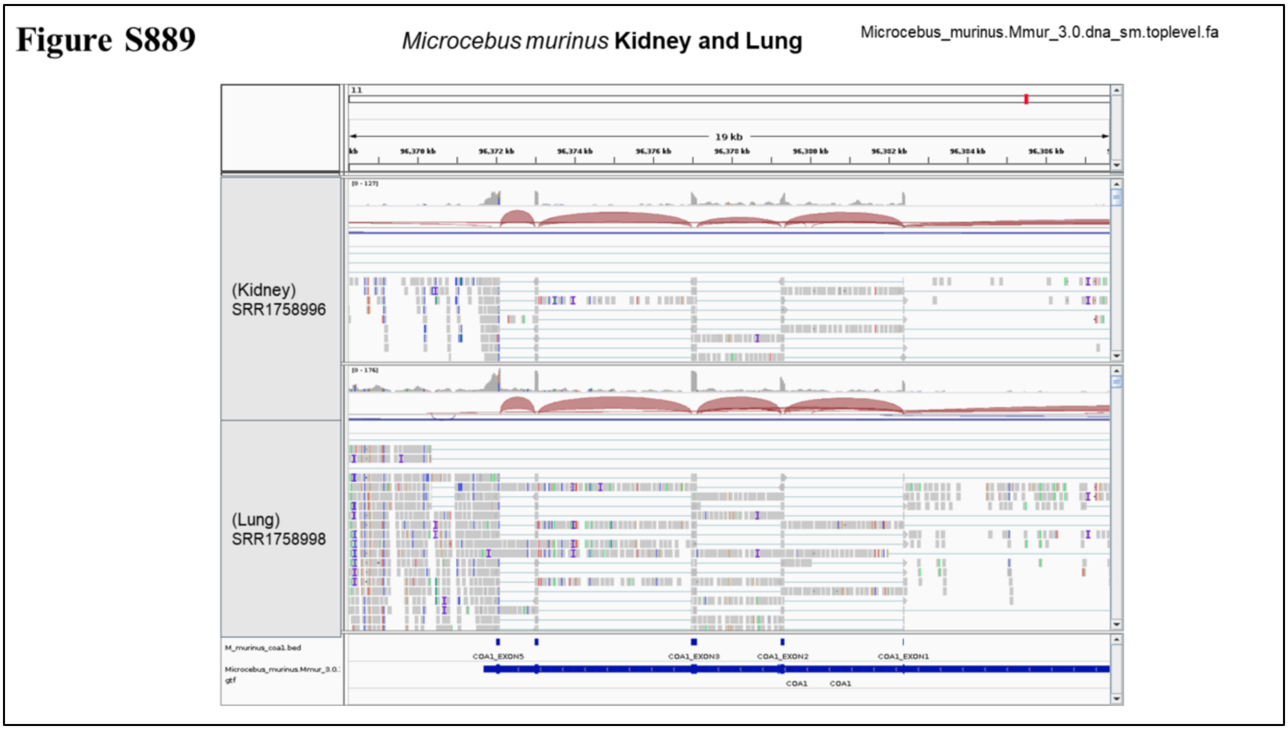

Figure S889: Screenshot of RNA-seq reads aligned to each of the putative locations of the five *COA1* exons in Gray mouse lemur (*Microcebus murinus*). Gray boxes represent reads aligned to the genome and thin lines connecting these boxes represent the spliced read alignments. Datasets from kidney and lung (SRR1758996 and SRR1758998) have been mapped to the Microcebus_murinus.Mmur_3.0.dna_sm.toplevel.fa. Locations of each of the exons are depicted as bed record in the bottom row.

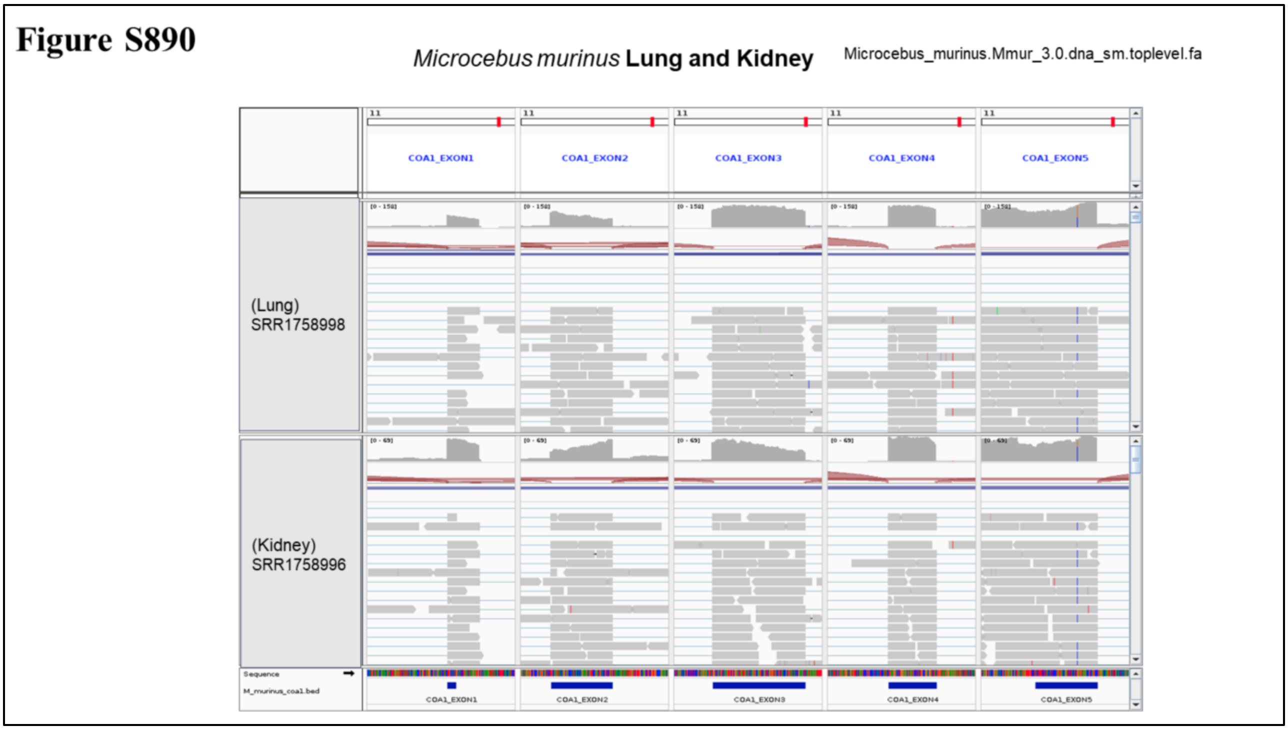

Figure S890: A zoomed in view of each of the *COA1* exons shown in **Supplementary Fig. S889**.

Figure S891

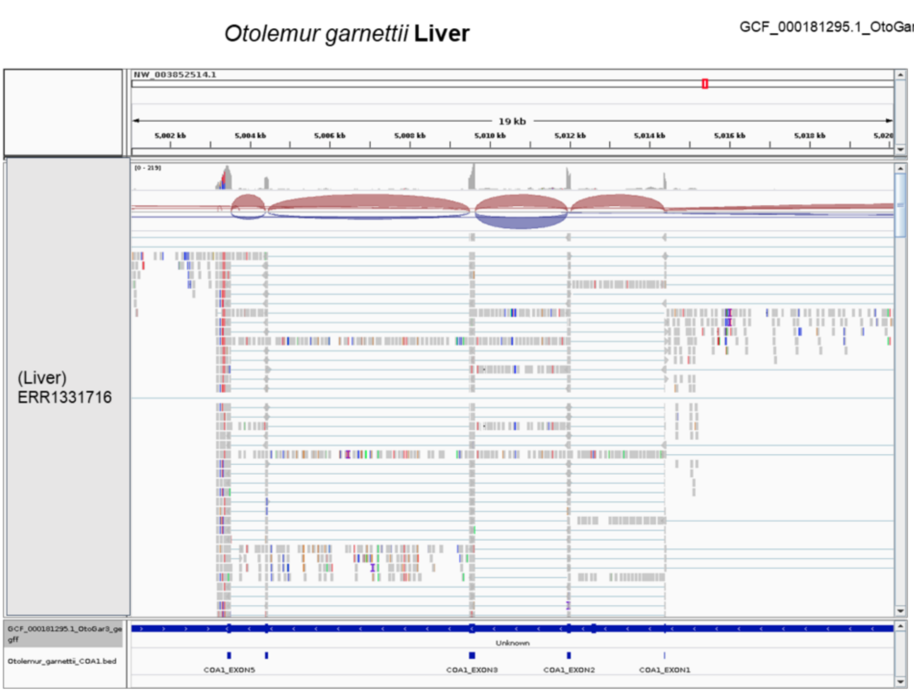

Figure S891: Screenshot of RNA-seq reads aligned to each of the putative locations of the five *COA1* exons in Northern greater galago (*Otolemur garnettii*). Gray boxes represent reads aligned to the genome and thin lines connecting these boxes represent the spliced read alignments. Datasets from liver (ERR1331716) have been mapped to the GCF_000181295.1_OtoGar3_genomic.fna. Locations of each of the exons are depicted as bed record in the bottom row.

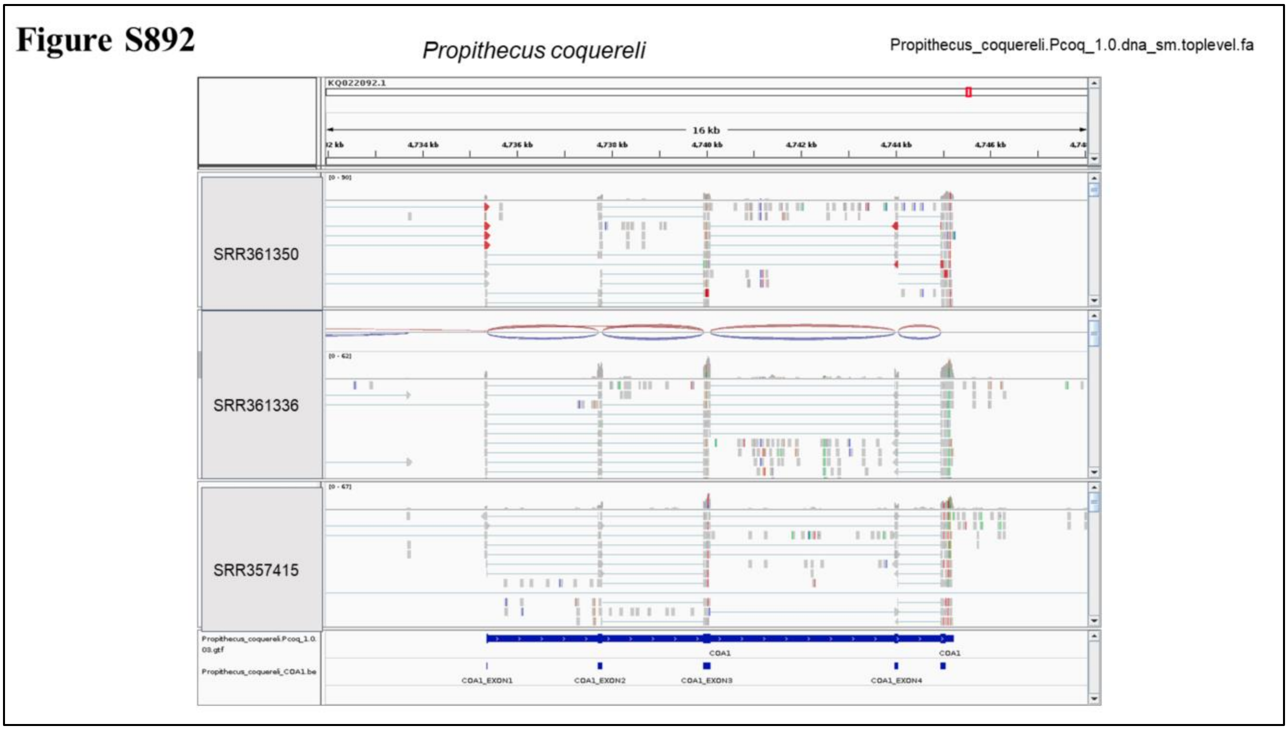

Figure S892: Screenshot of RNA-seq reads aligned to each of the putative locations of the five *COA1* exons in Coquerel's sifaka (*Propithecus coquereli*). Gray boxes represent reads aligned to the genome and thin lines connecting these boxes represent the spliced read alignments. Datasets from unknown tissue (SRR361350, SRR361336 and SRR357415) have been mapped to the Propithecus_coquereli.Pcoq_1.0.dna_sm.toplevel.fa. Locations of each of the exons are depicted as bed record in the bottom row.

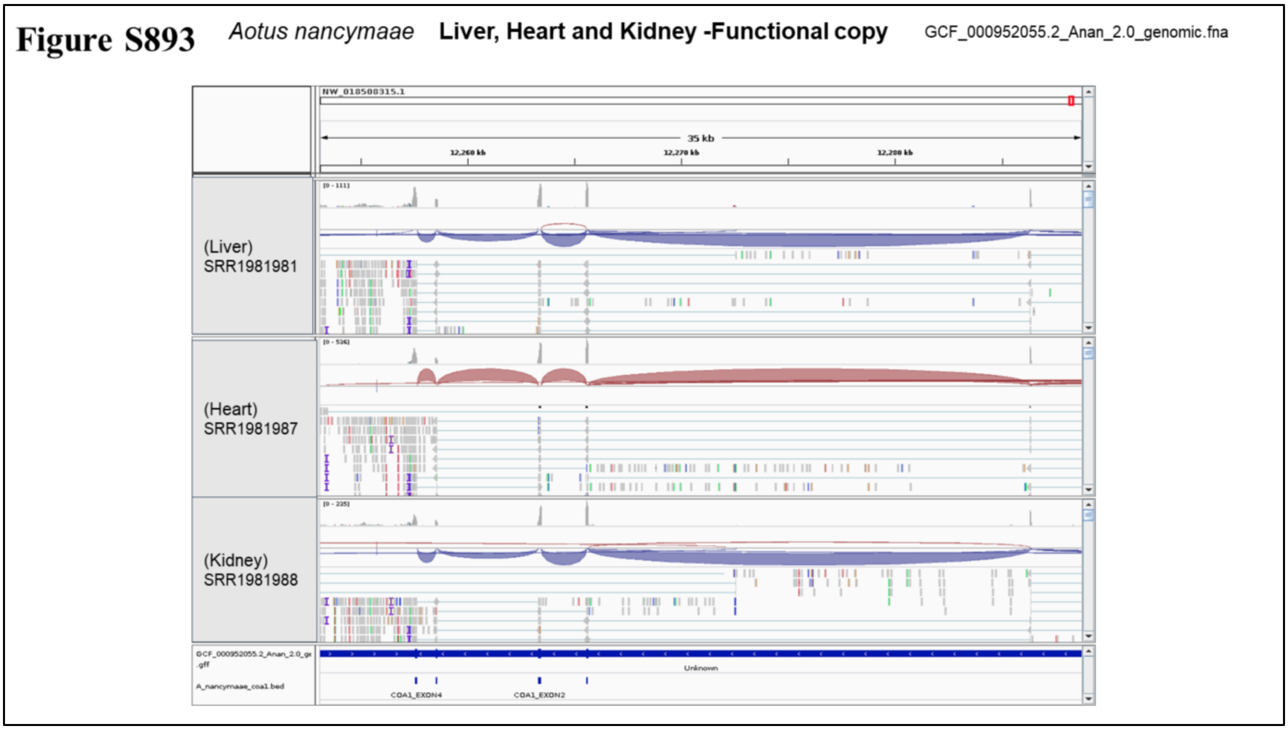

Figure S893: Screenshot of RNA-seq reads aligned to each of the putative locations of the five *COA1* exons in Nancy Ma's night monkey (*Aotus nancymaae*). Gray boxes represent reads aligned to the genome and thin lines connecting these boxes represent the spliced read alignments. Datasets from liver, heart and kidney (SRR1981981, SRR1981987 and SRR1981988) have been mapped to the GCF_000952055.2_Anan_2.0_genomic.fna. Locations of each of the exons are depicted as bed record in the bottom row.

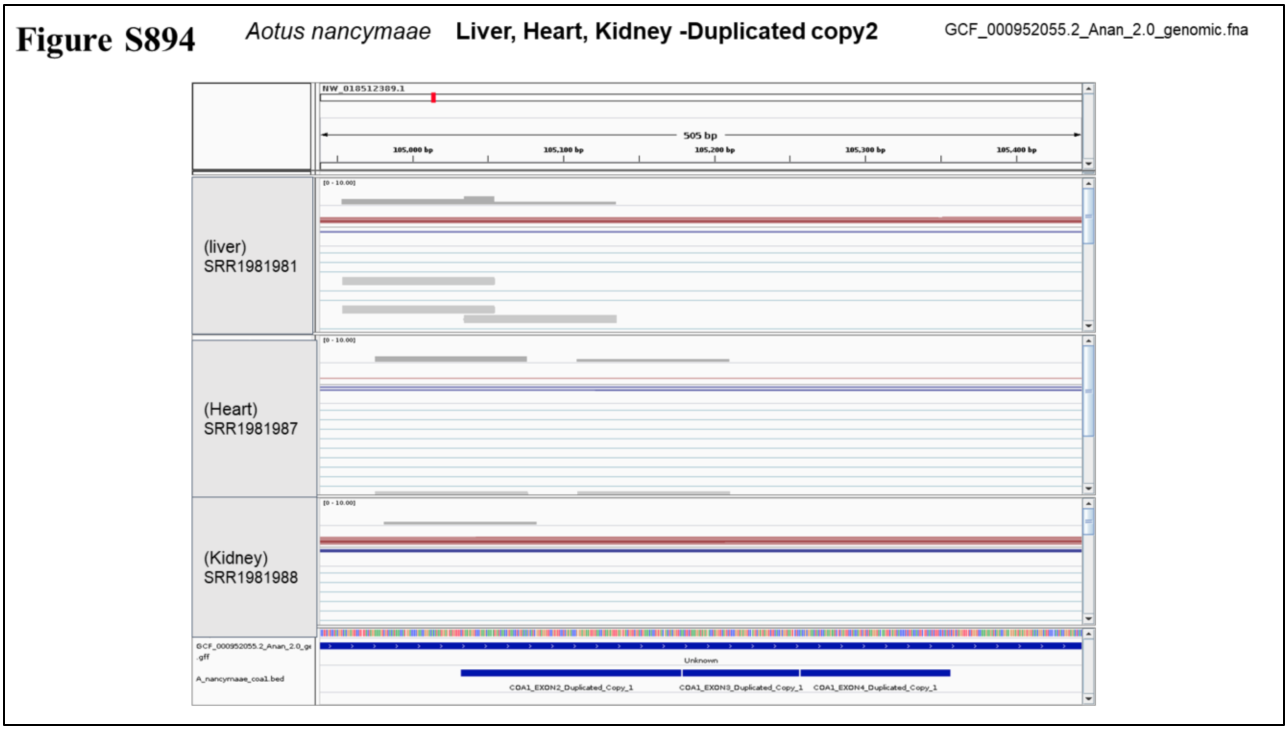

Figure S894: Screenshot of RNA-seq reads aligned to each of the putative locations of the *COA1* duplicated exons in Nancy Ma's night monkey (*Aotus nancymaae*). Gray boxes represent reads aligned to the genome and thin lines connecting these boxes represent the spliced read alignments in *ARFGAP3* gene region. Datasets from liver, heart and kidney (SRR1981981,SRR1981987 and SRR1981988) have been mapped to the GCF_000952055.2_Anan_2.0_genomic.fna. Locations of each of the duplicated exons are depicted as bed record in the bottom row.

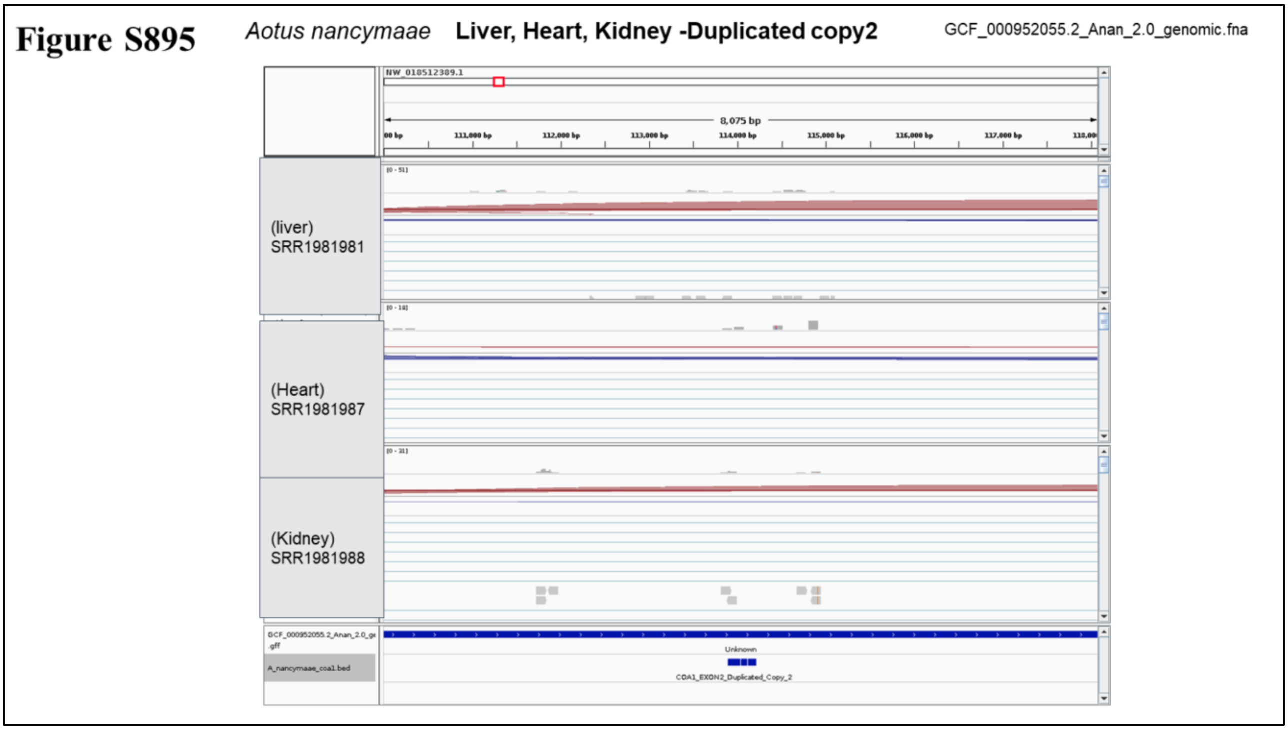

Figure S895: Screenshot of RNA-seq reads aligned to each of the putative locations of the *COA1* duplicated exons in Nancy Ma's night monkey (*Aotus nancymaae*). Gray boxes represent reads aligned to the genome and thin lines connecting these boxes represent the spliced read alignments in *ARFGAP3* gene region. Datasets from liver, heart and kidney (SRR1981981, SRR1981987 and SRR1981988) have been mapped to the GCF_000952055.2_Anan_2.0_genomic.fna. Locations of each of the duplicated exons are depicted as bed record in the bottom row.

Figure S896 *Callithrix jacchus* **Lung,Liver and Kidney -Functional copy** Callithrix_jacchus.ASM275486v1.dna_sm.toplevel.fa

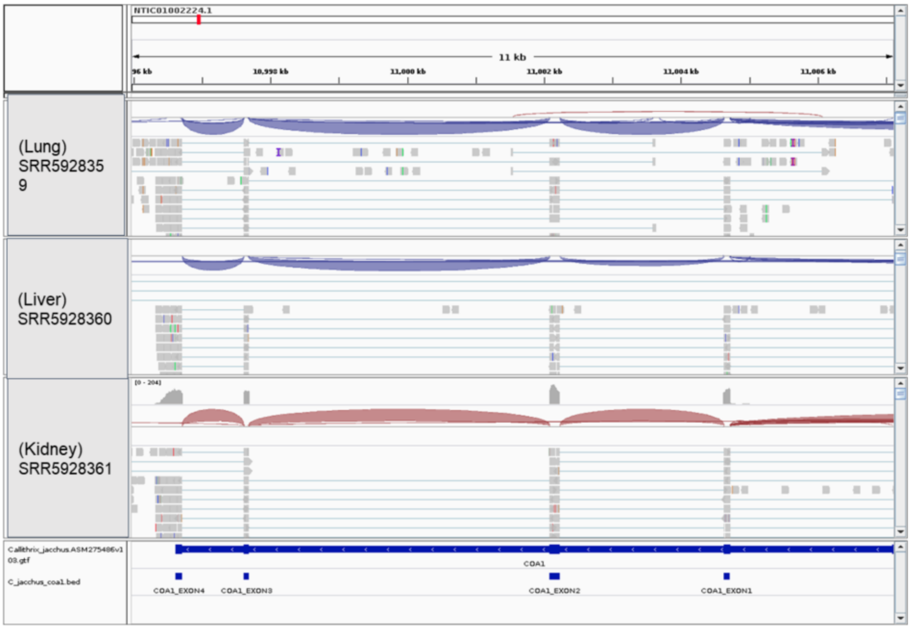

Figure S896: Screenshot of RNA-seq reads aligned to each of the putative locations of the four *COA1* exons in Common marmoset (*Callithrix jacchus*). Gray boxes represent reads aligned to the genome and thin lines connecting these boxes represent the spliced read alignments. Datasets from lung, liver and kidney (SRR5928359, SRR5928360 and SRR5928361) have been mapped to the Callithrix_jacchus.ASM275486v1.dna_sm.toplevel.fa. Locations of each of the exons are depicted as bed record in the bottom row.

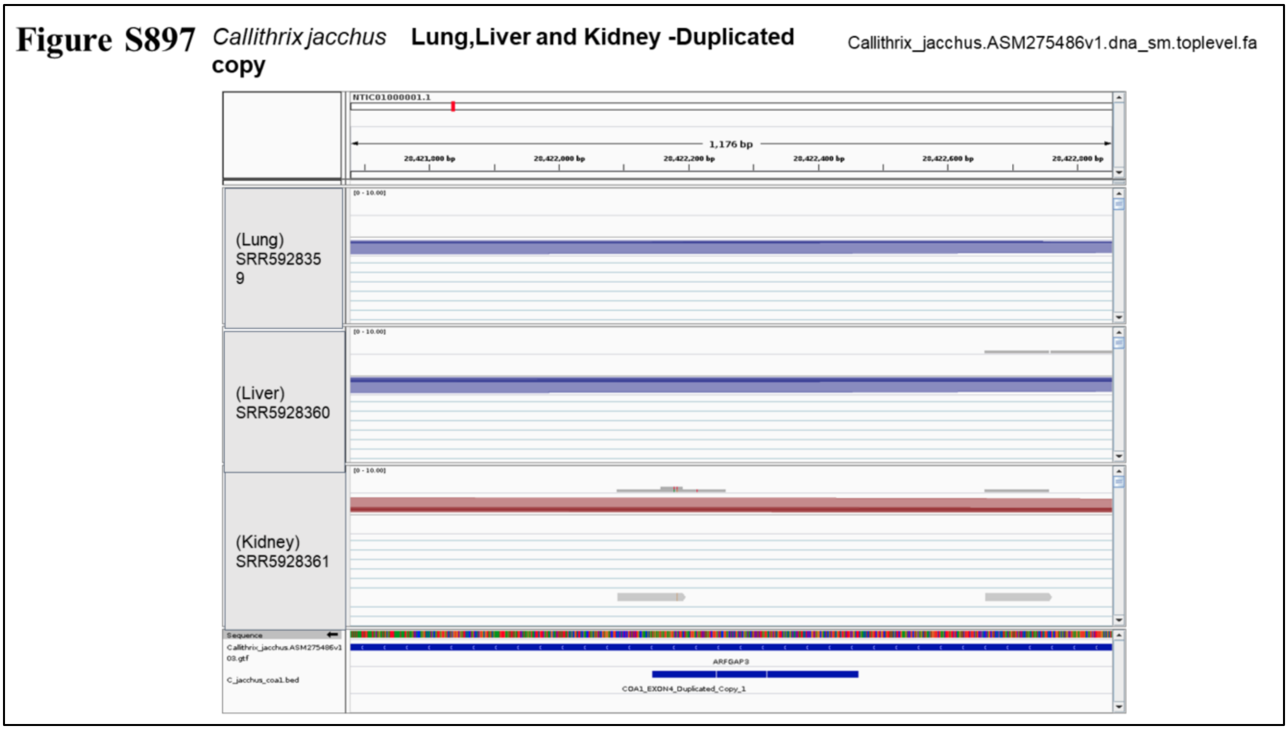

Figure S897: Screenshot of RNA-seq reads aligned to each of the putative locations of the four *COA1* exons in Common marmoset (*Callithrix jacchus*). Gray boxes represent reads aligned to the genome and thin lines connecting these boxes represent the spliced read alignments in *ARFGAP3* gene region. Datasets from lung, liver and kidney (SRR5928359, SRR5928360 and SRR5928361) have been mapped to the Callithrix_jacchus.ASM275486v1.dna_sm.toplevel.fa. Locations of each of the exons are depicted as bed record in the bottom row.

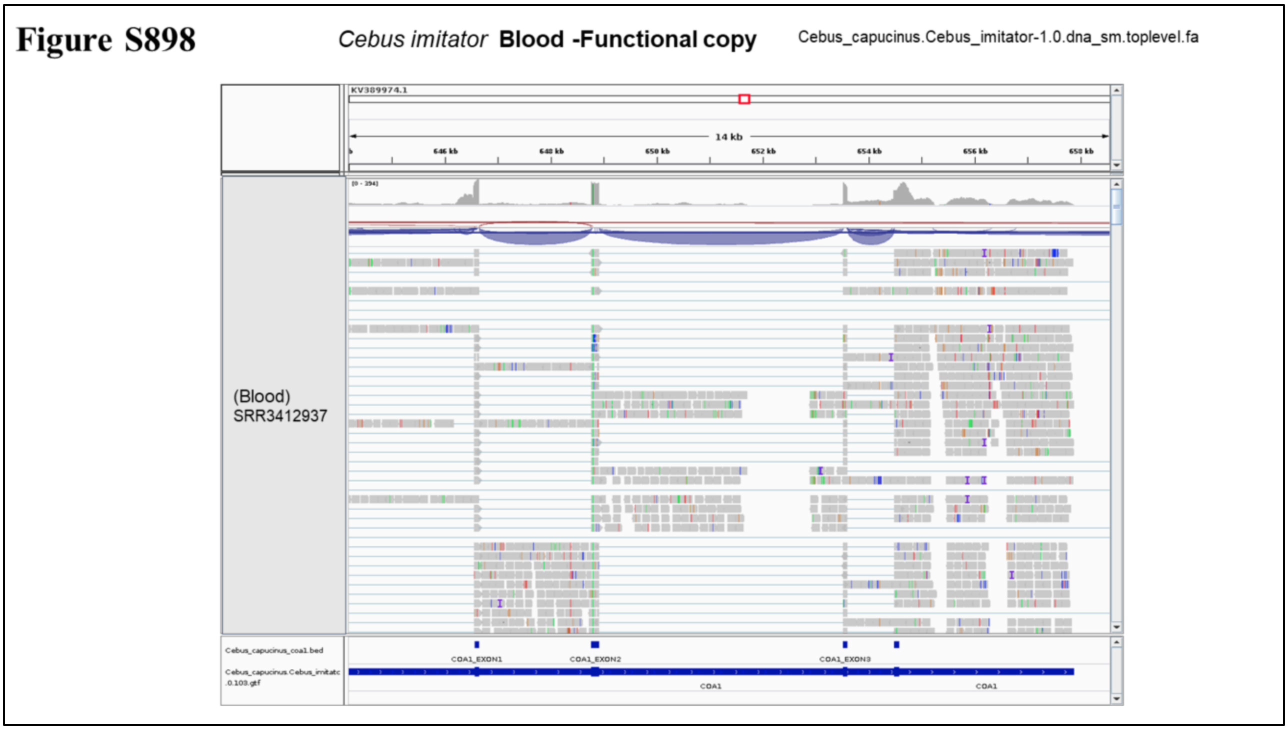

Figure S898: Screenshot of RNA-seq reads aligned to each of the putative locations of the four *COA1* exons in Panamanian White-faced Capuchin (*Cebus imitator*). Gray boxes represent reads aligned to the genome and thin lines connecting these boxes represent the spliced read alignments. Datasets from blood (SRR3412937) have been mapped to the Cebus_capucinus.Cebus_imitator-1.0.dna_sm.toplevel.fa. Locations of each of the exons are depicted as bed record in the bottom row.

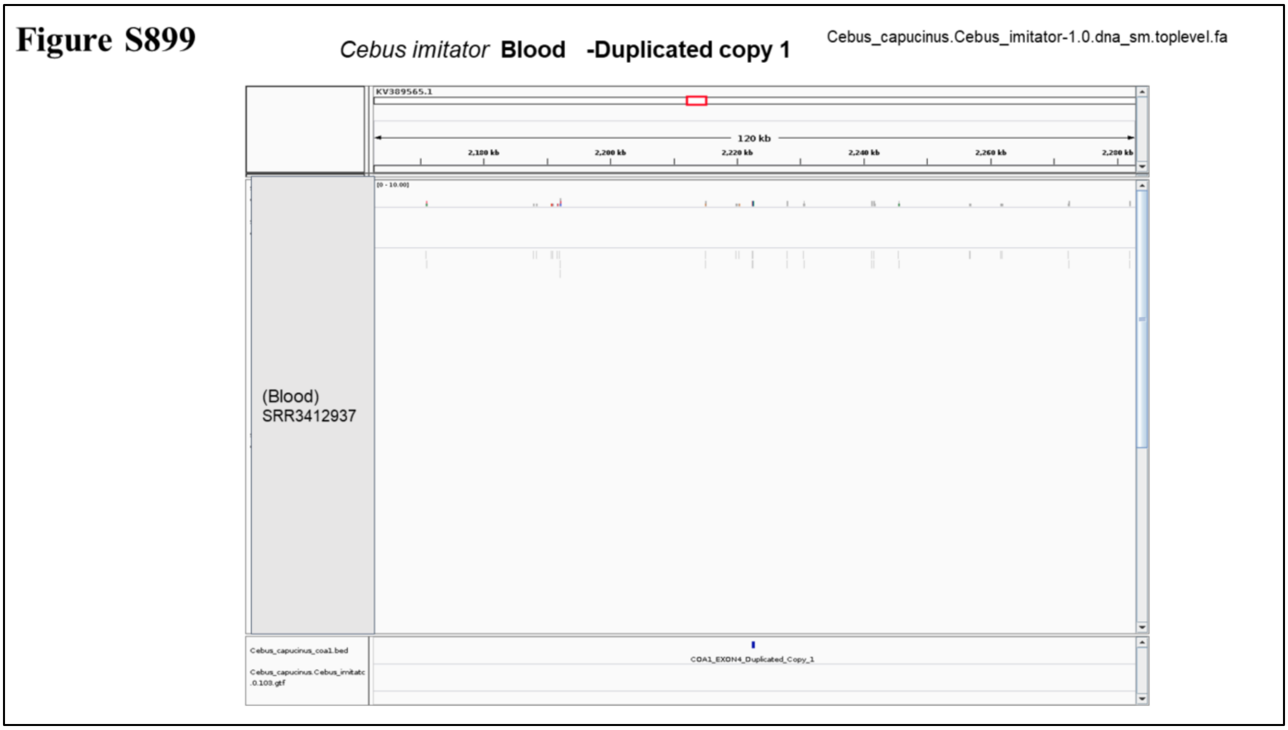

Figure S899: Screenshot of RNA-seq reads aligned to each of the putative locations of the *COA1* duplicated exons in Panamanian White-faced Capuchin (*Cebus imitator*). Gray boxes represent reads aligned to the genome and thin lines connecting these boxes represent the spliced read alignments in this located region. Datasets from blood (SRR3412937) have been mapped to the Cebus_capucinus.Cebus_imitator-1.0.dna_sm.toplevel.fa. Locations of each of the exons are depicted as bed record in the bottom row.

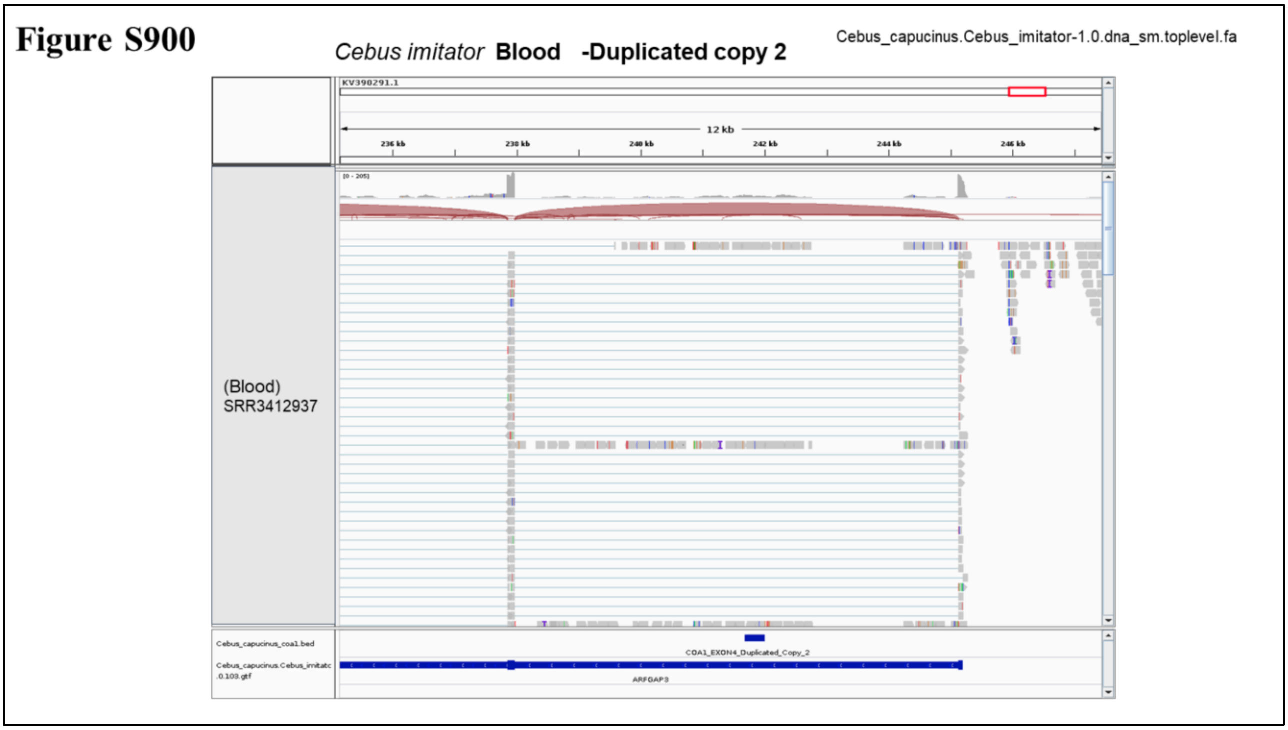

Figure S900: Screenshot of RNA-seq reads aligned to each of the putative locations of the four *COA1* exons in Panamanian White-faced Capuchin (*Cebus imitator*). Gray boxes represent reads aligned to the genome and thin lines connecting these boxes represent the spliced read alignments in ARFGAP3 gene region. Datasets from blood (SRR3412937) have been mapped to the Cebus_capucinus.Cebus_imitator-1.0.dna_sm.toplevel.fa. Locations of each of the exons are depicted as bed record in the bottom row.

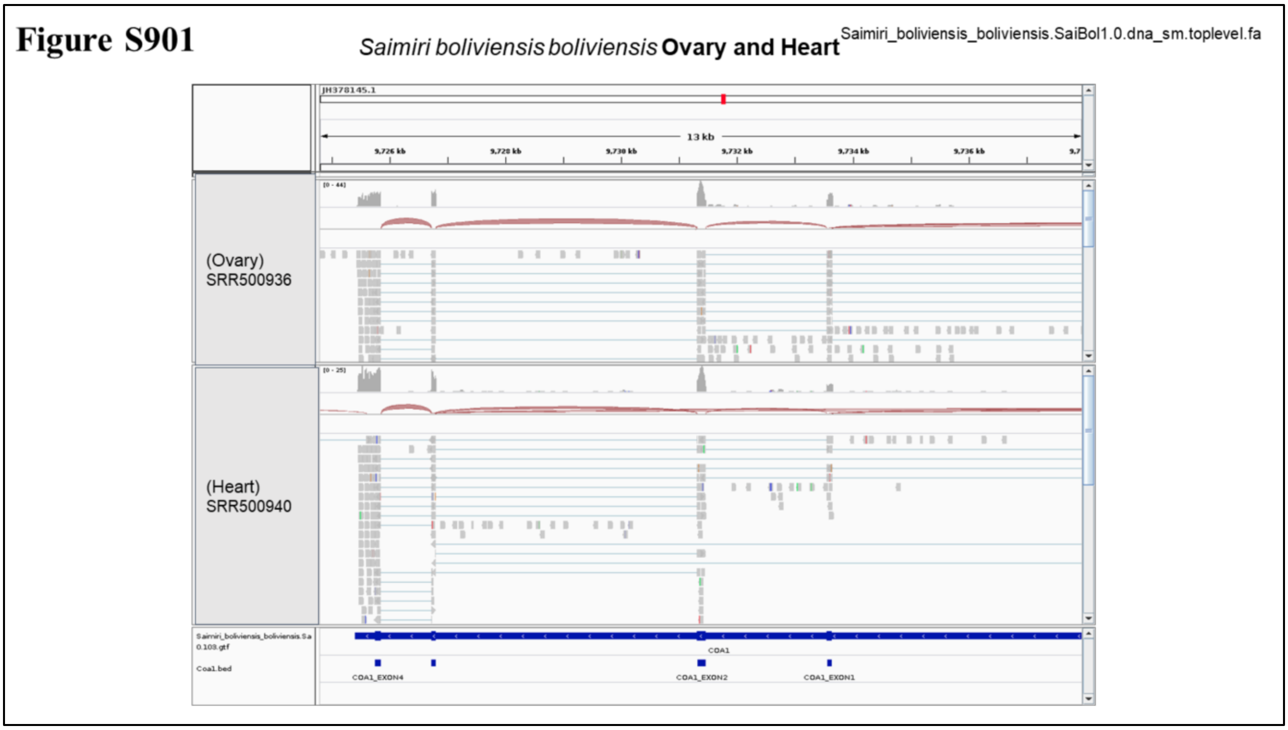

Figure S901: Screenshot of RNA-seq reads aligned to each of the putative locations of the four *COA1* exons in Black-capped squirrel monkey (*Saimiri boliviensis boliviensis*). Gray boxes represent reads aligned to the genome and thin lines connecting these boxes represent the spliced read alignments. Datasets from ovary and heart (SRR500936, SRR500940) have been mapped to the Saimiri_boliviensis_boliviensis.SaiBol1.0.dna_sm.toplevel.fa. Locations of each of the exons are depicted as bed record in the bottom row.

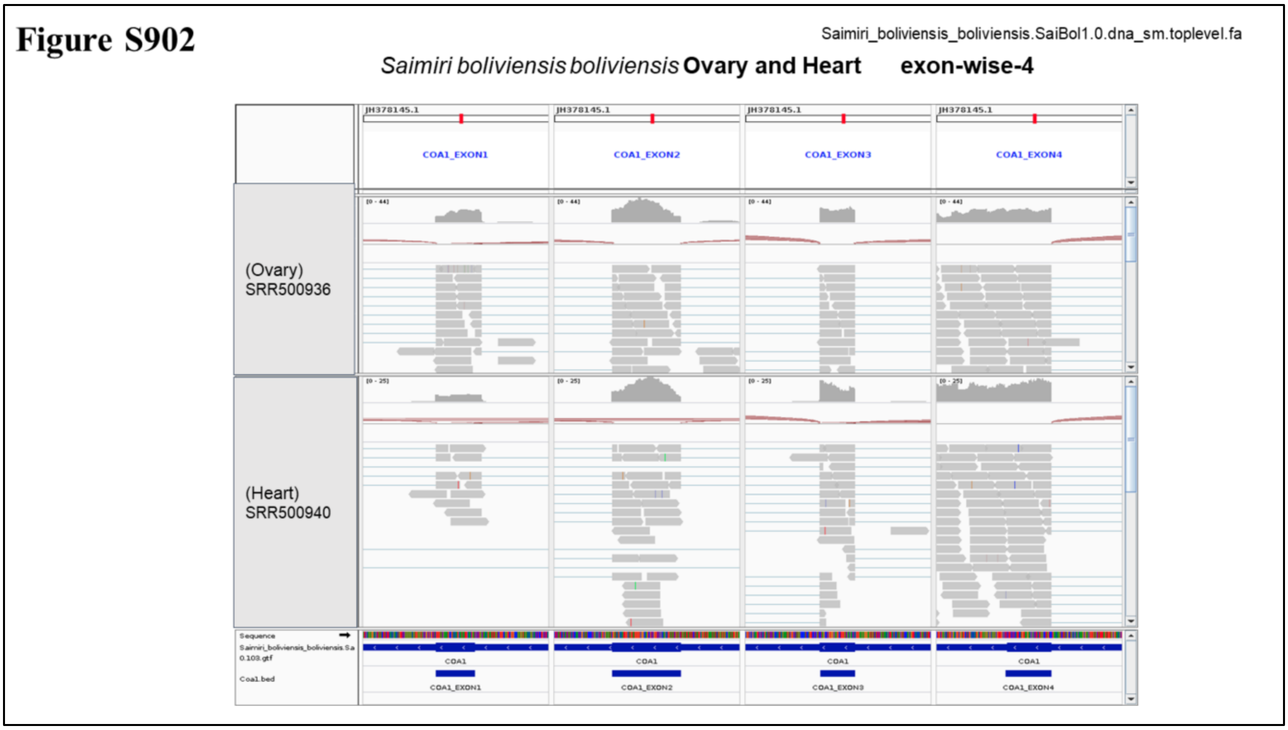

Figure S902: A zoomed in view of each of the *COA1* exons shown in **Supplementary Fig. S901**.

Figure S903

Saimiri boliviensis boliviensis **Ovary and Heart -Duplicated copy1**

Saimiri_boliviensis_boliviensis.SaiBol1.0.dna_sm.toplevel.fa

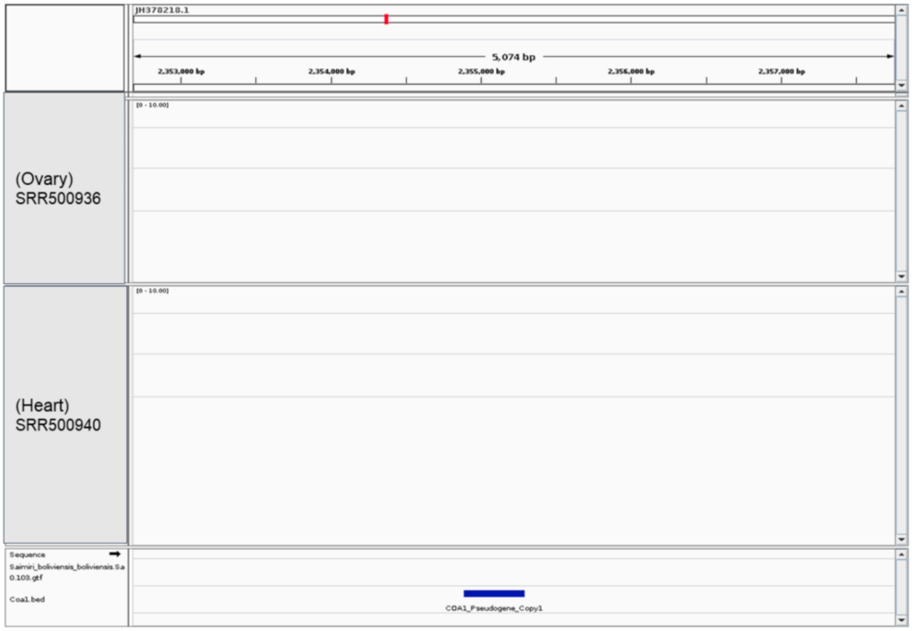

Figure S903: Screenshot of RNA-seq reads aligned to each of the putative locations of the *COA1* duplicated exons in Black-capped squirrel monkey (*Saimiri boliviensis boliviensis*). Gray boxes represent reads aligned to the genome and thin lines connecting these boxes represent the spliced read alignments in this region. Datasets from ovary and heart (SRR500936 and SRR500940) have been mapped to the Saimiri_boliviensis_boliviensis.SaiBol1.0.dna_sm.toplevel.fa. Locations of each of the exons are depicted as bed record in the bottom row.

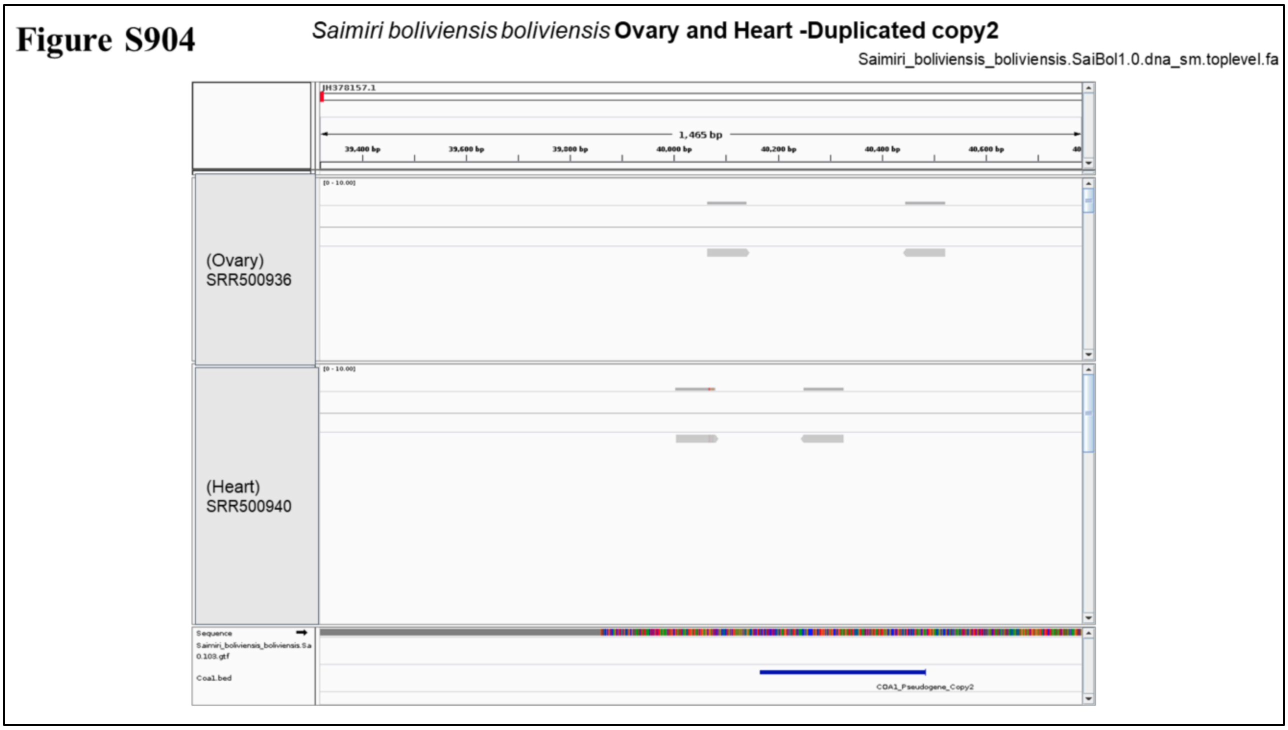

Figure S904: Screenshot of RNA-seq reads aligned to each of the putative locations of the *COA1* duplicated exons in Black-capped squirrel monkey (*Saimiri boliviensis boliviensis*). Gray boxes represent reads aligned to the genome and thin lines connecting these boxes represent the spliced read alignments in *ARFGAP3* gene region. Datasets from ovary and heart (SRR500936 and SRR500940) have been mapped to the Saimiri_boliviensis_boliviensis.SaiBol1.0.dna_sm.toplevel.fa. Locations of each of the exons are depicted as bed record in the bottom row.

Figure S905 *Cercocebus atys* **Liver Functional copy** Cercocebus_atys.Caty_1.0.dna_sm.toplevel.fa

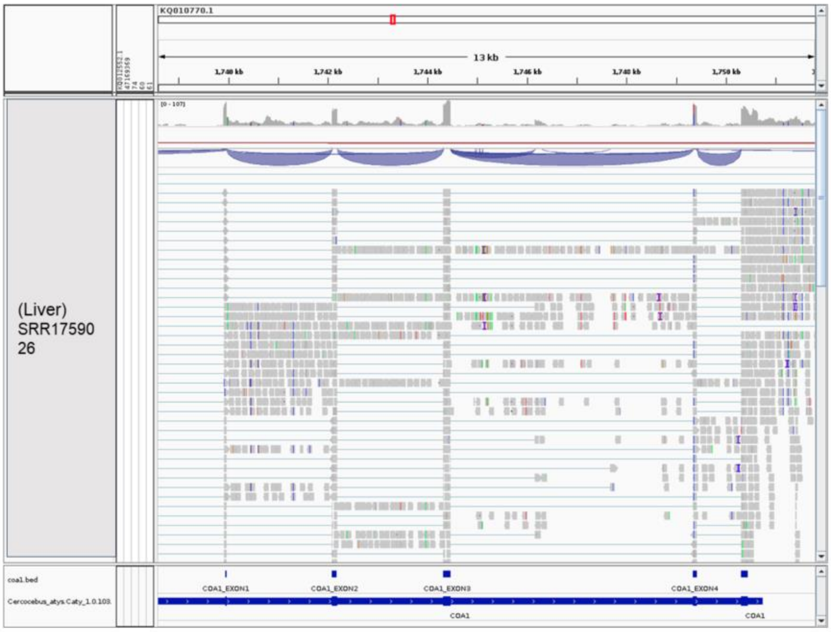

Figure S905: Screenshot of RNA-seq reads aligned to each of the putative locations of the five *COA1* exons in Sooty mangabey (*Cercocebus atys*). Gray boxes represent reads aligned to the genome and thin lines connecting these boxes represent the spliced read alignments. Datasets from liver (SRR1759026) have been mapped to the Cercocebus_atys.Caty_1.0.dna_sm.toplevel.fa. Locations of each of the exons are depicted as coa1.bed record in the bottom row.

Figure S906

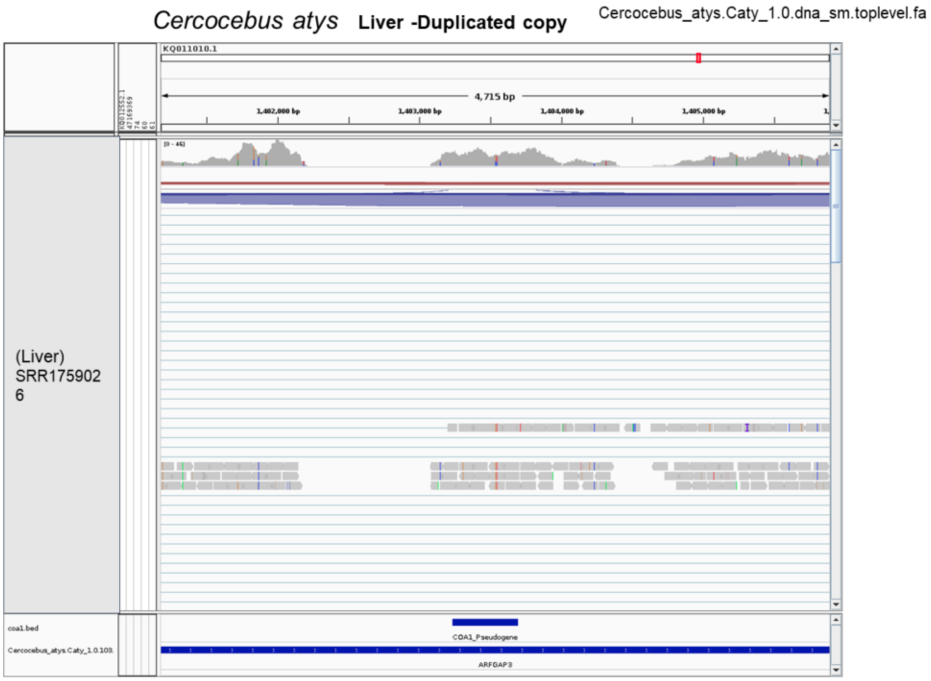

Figure S906: Screenshot of RNA-seq reads aligned to each of the putative locations of the *COA1* duplicated exons in Sooty mangabey (*Cercocebus atys*). Gray boxes represent reads aligned to the genome and thin lines connecting these boxes represent the spliced read alignments in *ARFGAP3* gene region. Datasets from liver (SRR1759026) have been mapped to the Cercocebus_atys.Caty_1.0.dna_sm.toplevel.fa. Locations of each of the exons are depicted as coa1.bed record in the bottom row.

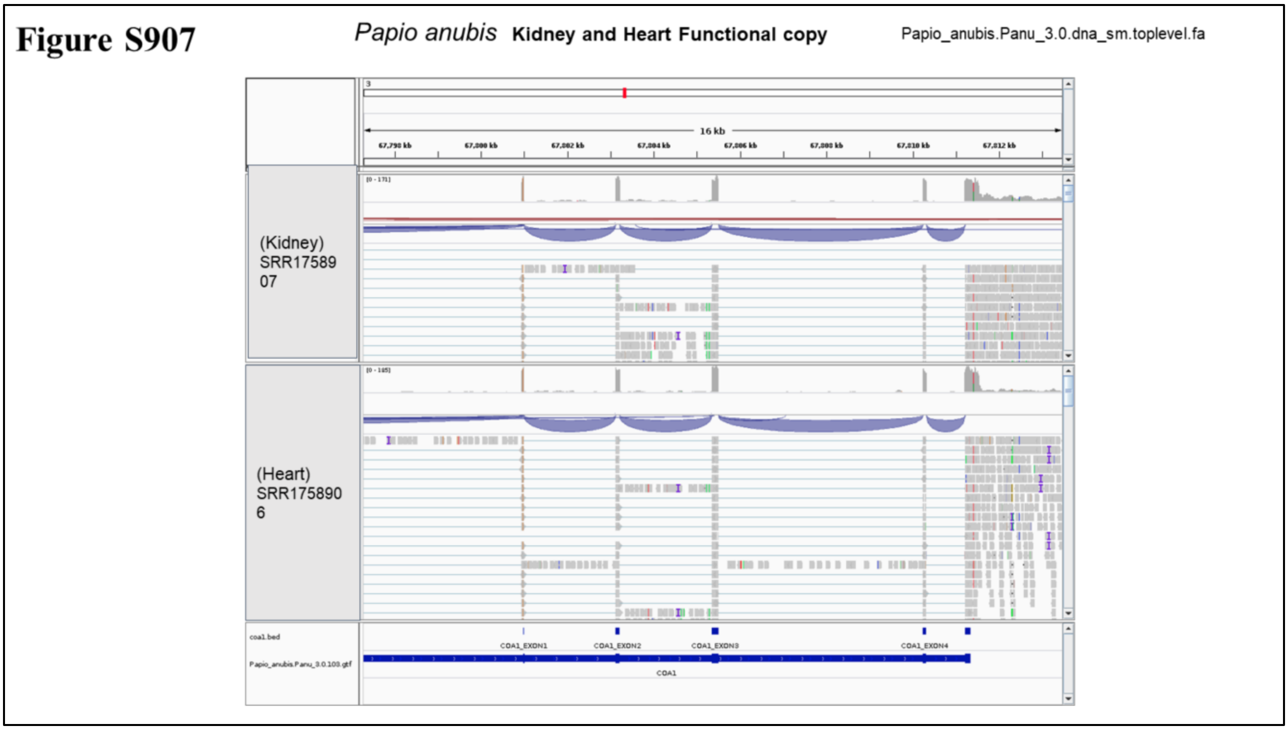

Figure S907: Screenshot of RNA-seq reads aligned to each of the putative locations of the five *COA1* exons in Olive baboon (*Papio anubis*). Gray boxes represent reads aligned to the genome and thin lines connecting these boxes represent the spliced read alignments. Datasets from kidney and heart (SRR1758907, SRR1758906) have been mapped to the Papio_anubis.Panu_3.0.dna_sm.toplevel.fa. Locations of each of the exons are depicted as coa1.bed record in the bottom row.

Figure S908

Papio anubis **Kidney and Heart Duplicated copy**

Papio_anubis.Panu_3.0.dna_sm.toplevel.fa

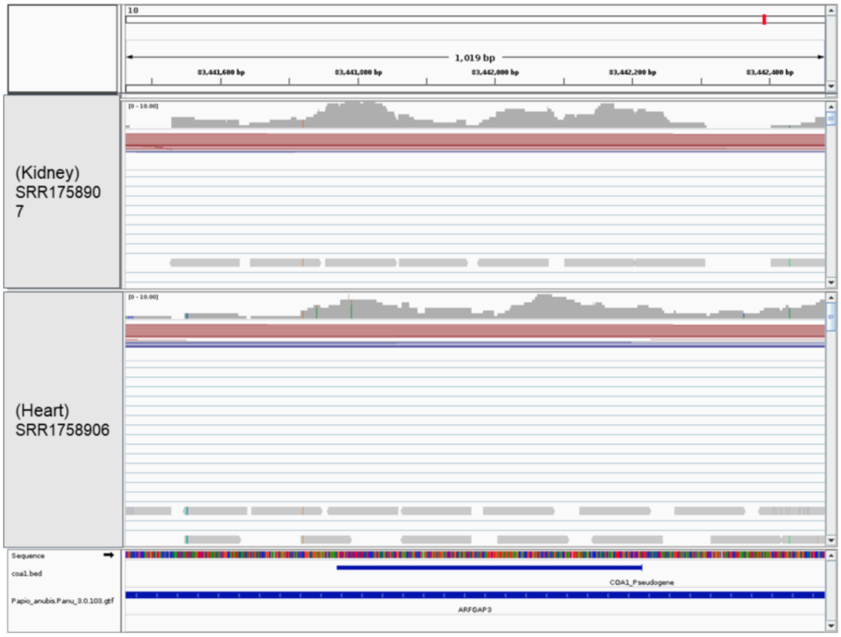

Figure S908: Screenshot of RNA-seq reads aligned to each of the putative locations of the *COA1* duplicated exons in Olive baboon (*Papio anubis*). Gray boxes represent reads aligned to the genome and thin lines connecting these boxes represent the spliced read alignments in *ARFGAP3* gene region. Datasets from kidney and heart (SRR1758907, SRR1758906) have been mapped to the Papio_anubis.Panu_3.0.dna_sm.toplevel.fa. Locations of each of the exons are depicted as coa1.bed record in the bottom row.

Figure S909

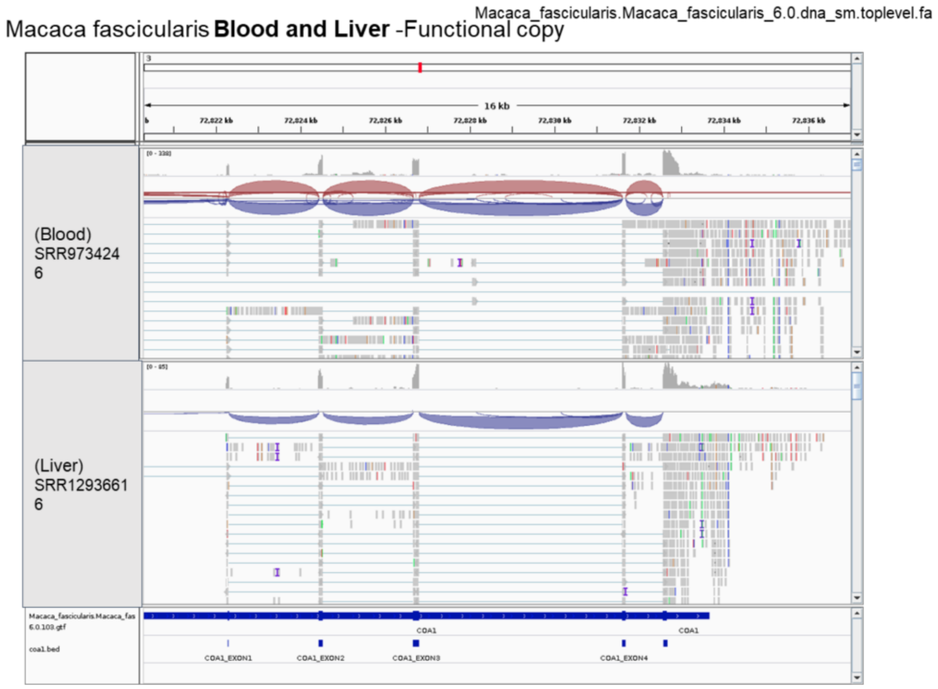

Figure S909: Screenshot of RNA-seq reads aligned to each of the putative locations of the five *COA1* exons in Crab-eating macaque (*Macaca fascicularis*). Gray boxes represent reads aligned to the genome and thin lines connecting these boxes represent the spliced read alignments. Datasets from blood and liver (SRR9734246, SRR12936616) have been mapped to the Macaca_fascicularis.Macaca_fascicularis_6.0.dna_sm.toplevel.fa. Locations of each of the exons are depicted as coa1.bed record in the bottom row.

Figure S910 Macaca fascicularis **Blood and Liver** -Functional copy Macaca_fascicularis.Macaca_fascicularis_6.0.dna_sm.toplevel.fa

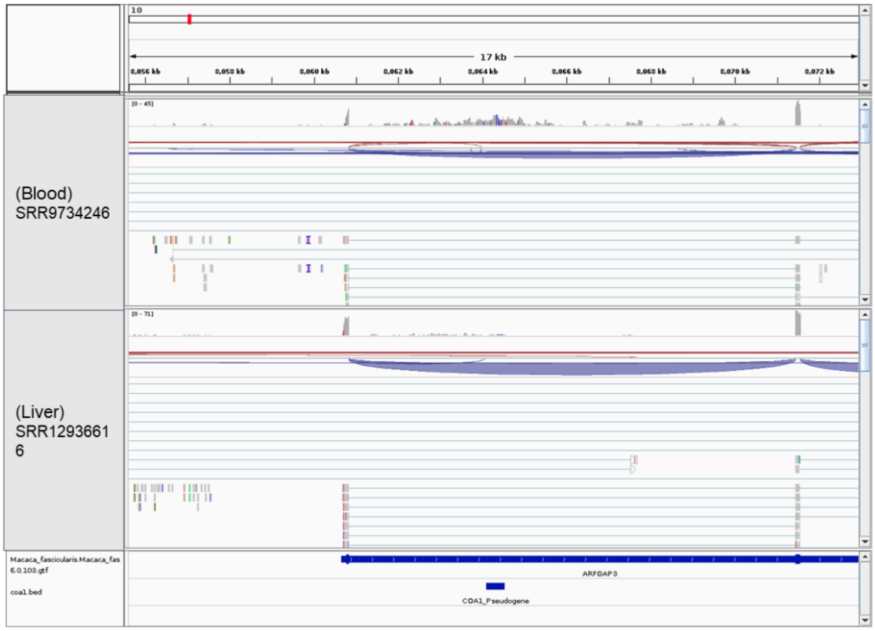

Figure S910: Screenshot of RNA-seq reads aligned to each of the putative locations of the *COA1* duplicated exons in Crab-eating macaque (*Macaca fascicularis*). Gray boxes represent reads aligned to the genome and thin lines connecting these boxes represent the spliced read alignments in *ARFGAP3* gene region. Datasets from blood and liver (SRR9734246, SRR12936616) have been mapped to the Macaca_fascicularis.Macaca_fascicularis_6.0.dna_sm.toplevel.fa. Locations of each of the exons are depicted as coa1.bed record in the bottom row.

Figure S911 *Rhinopithecus roxellana* **Heart and Blood Functional copy** Rhinopithecus_roxellana.Rrox_v1.dna_sm.toplevel.fa

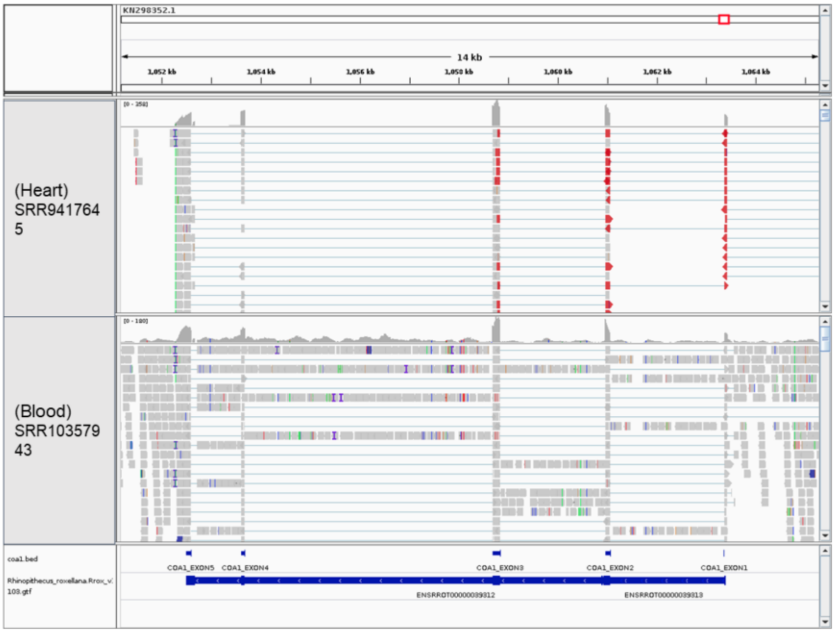

Figure S911: Screenshot of RNA-seq reads aligned to each of the putative locations of the five *COA1* exons in Golden snub-nosed monkey (*Rhinopithecus roxellana*). Gray boxes represent reads aligned to the genome and thin lines connecting these boxes represent the spliced read alignments. Datasets from heart and blood (SRR9417645, SRR10357943) have been mapped to the Rhinopithecus_roxellana.Rrox_v1.dna_sm.toplevel.fa. Locations of each of the exons are depicted as coa1.bed record in the bottom row.

Figure S912 *Rhinopithecus roxellana* **Heart and Blood Duplicated copy** Rhinopithecus_roxellana.Rrox_v1.dna_sm.toplevel.fa

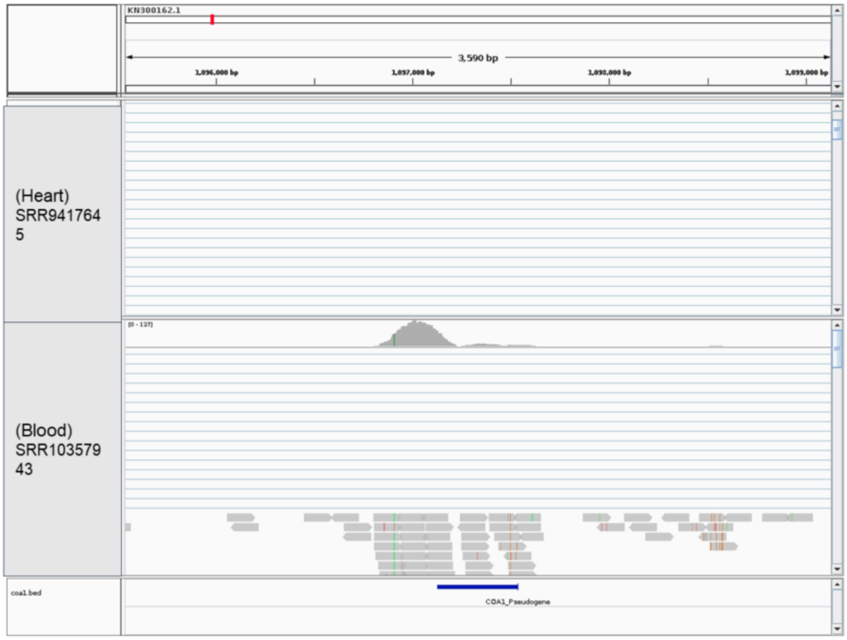

Figure S912: Screenshot of RNA-seq reads aligned to each of the putative locations of the *COA1* duplicated exons in Golden snub-nosed monkey (*Rhinopithecus roxellana*). Gray boxes represent reads aligned to the genome and thin lines connecting these boxes represent the spliced read alignments in *ARFGAP3* gene region. Datasets from heart and blood (SRR9417645, SRR10357943) have been mapped to the Rhinopithecus_roxellana.Rrox_v1.dna_sm.toplevel.fa. Locations of each of the exons are depicted as coa1.bed record in the bottom row.

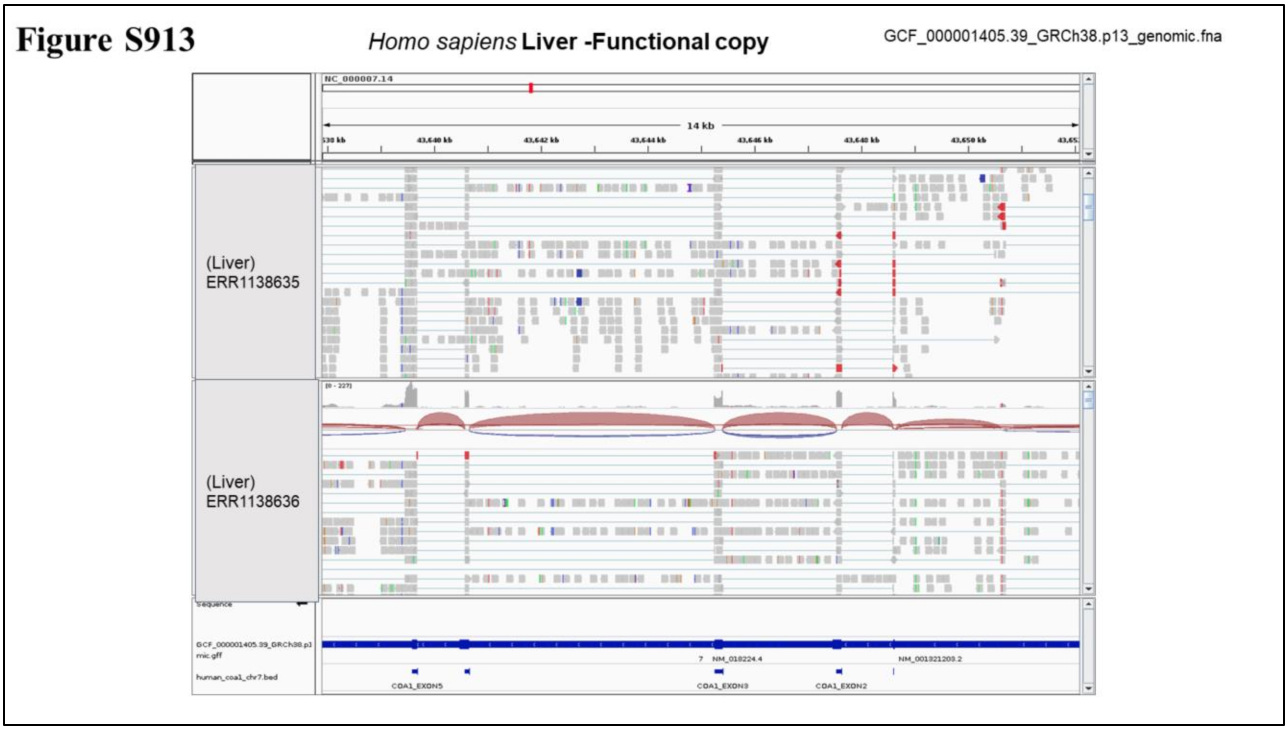

Figure S913: Screenshot of RNA-seq reads aligned to each of the putative locations of the five *COA1* exons in human (*Homo sapiens*). Gray boxes represent reads aligned to the genome and thin lines connecting these boxes represent the spliced read alignments. Datasets from liver (ERR1138635, ERR1138636) have been mapped to the GCF_000001405.39_GRCh38.p13_genomic.fna. Locations of each of the exons are depicted as.bed record in the bottom row.

Figure S914

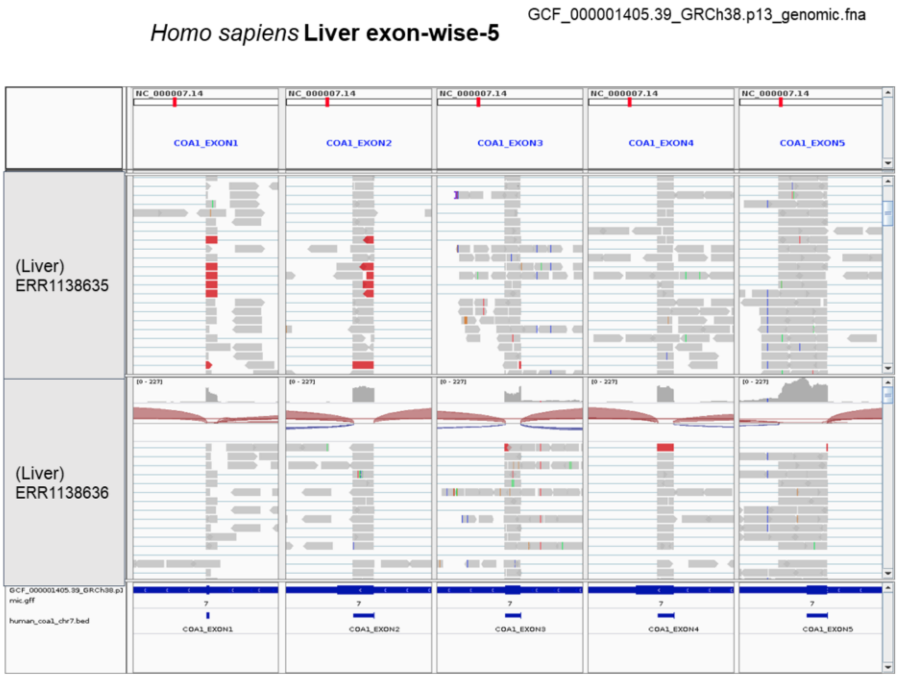

Figure S914: A zoomed in view of each of the *COA1* exons shown in **Supplementary Fig. S913**.

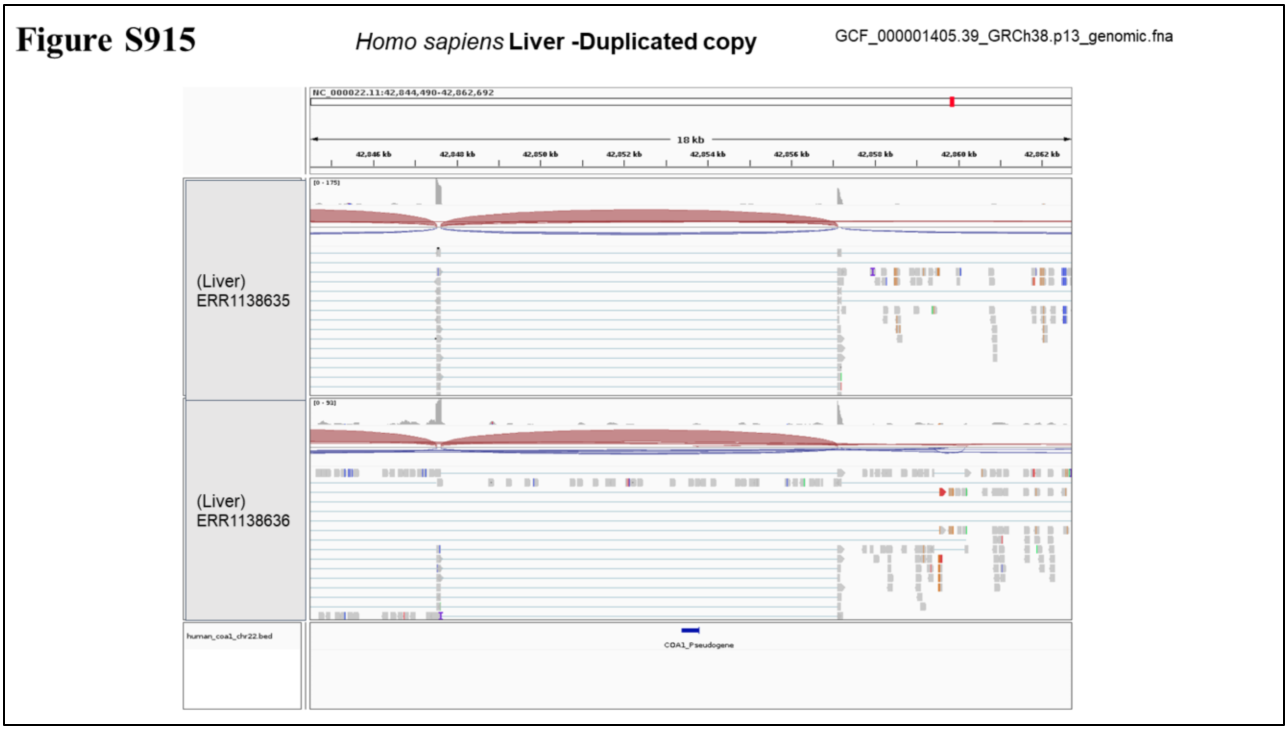

Figure S915: Screenshot of RNA-seq reads aligned to each of the putative locations of the *COA1* duplicated exons in human (*Homo sapiens*). Gray boxes represent reads aligned to the genome and thin lines connecting these boxes represent the spliced read alignments in *ARFGAP3* gene region. Datasets from liver (ERR1138635, ERR1138636) have been mapped to the GCF_000001405.39_GRCh38.p13_genomic.fna. Locations of each of the exons are depicted as.bed record in the bottom row.

Figure S916 *Homo sapiens* **Testis Functional copy** GCF_000001405.39_GRCh38.p13_genomic.fna

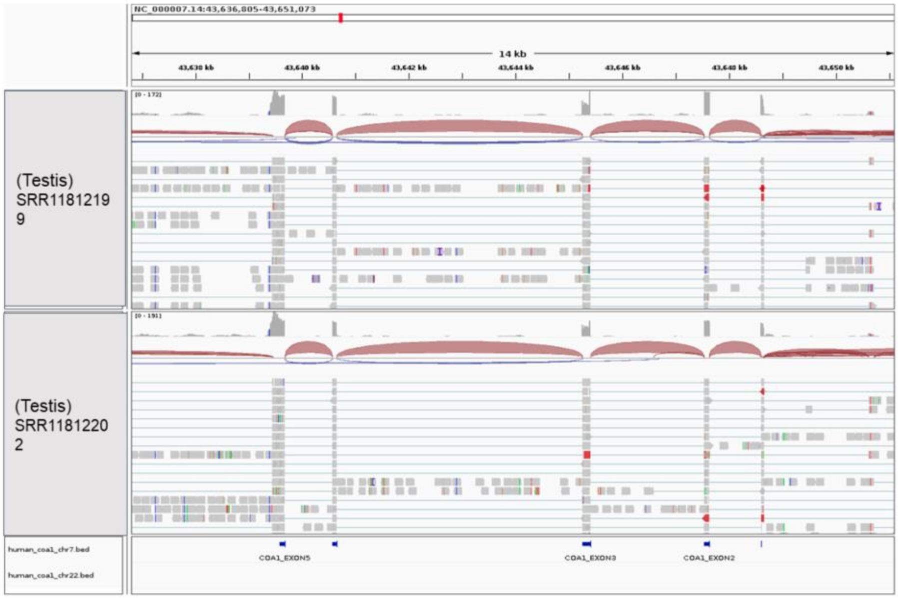

Figure S916: Screenshot of RNA-seq reads aligned to each of the putative locations of the five *COA1* exons in human (*Homo sapiens*). Gray boxes represent reads aligned to the genome and thin lines connecting these boxes represent the spliced read alignments. Datasets from testis (SRR11812199, SRR11812202) have been mapped to the GCF_000001405.39_GRCh38.p13_genomic.fna. Locations of each of the exons are depicted as.bed record in the bottom row.

Figure S917

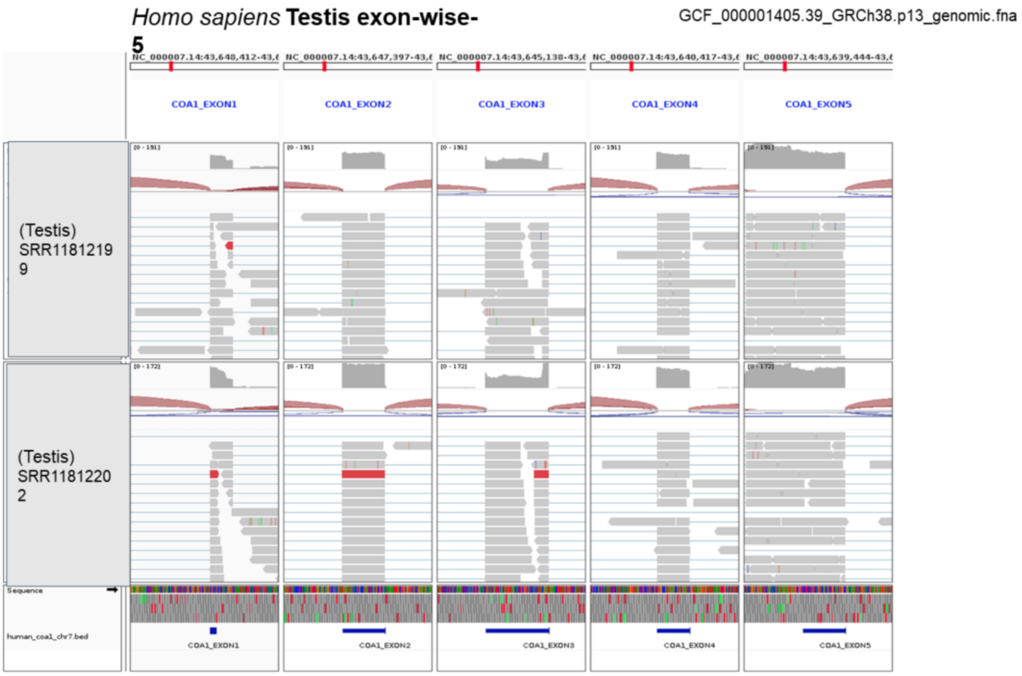

Figure S917: A zoomed in view of each of the *COA1* exons shown in **Supplementary Fig. S916**.

Figure S918

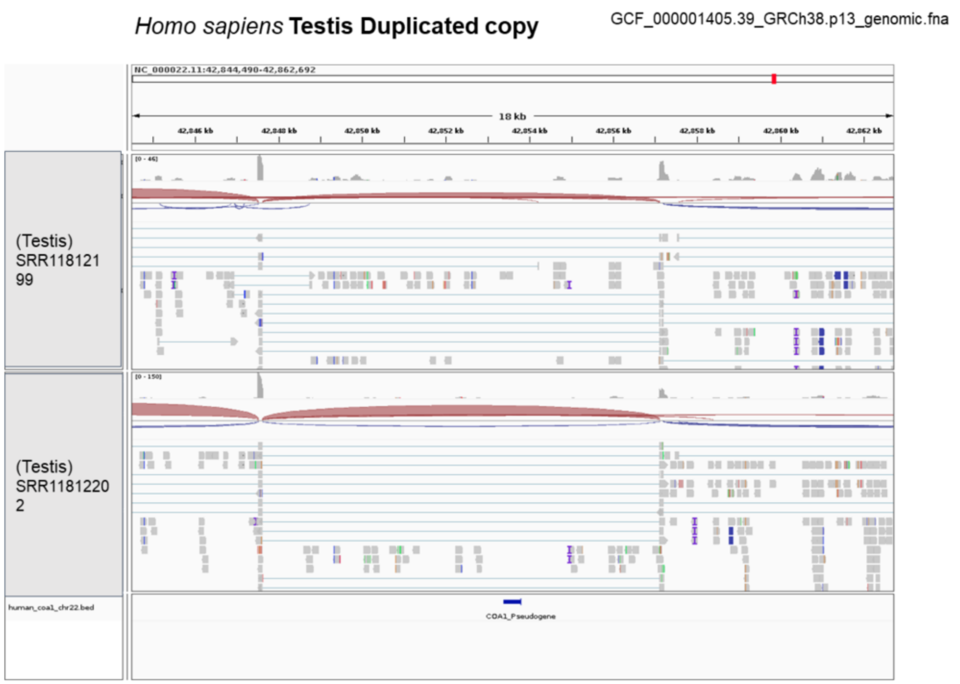

Figure S918: Screenshot of RNA-seq reads aligned to each of the putative locations of the *COA1* duplicated exons in human (*Homo sapiens*). Gray boxes represent reads aligned to the genome and thin lines connecting these boxes represent the spliced read alignments in *ARFGAP3* gene region. Datasets from testis (SRR11812199, SRR11812202) have been mapped to the GCF_000001405.39_GRCh38.p13_genomic.fna. Locations of each of the exons are depicted as.bed record in the bottom row.

Figure S919 *Carlito syrichta* **Unknown tissue**

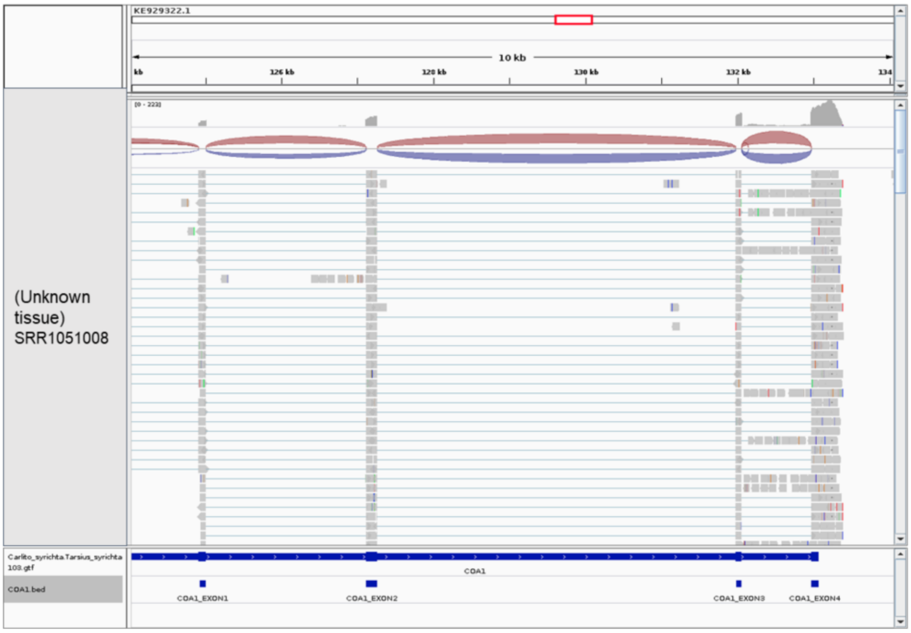

Figure S919: Screenshot of RNA-seq reads aligned to each of the putative locations of the four *COA1* exons in Philippine tarsier (*Carlito syrichta*). Gray boxes represent reads aligned to the genome and thin lines connecting these boxes represent the spliced read alignments. Datasets from Unknown tissue (SRR1051008) have been mapped to its own genome. Locations of each of the exons are depicted as bed record in the bottom row.

Figure S920

Suricata_suricatta.meerkat_22Aug2017_6uvM2_HiC.dna_sm.toplevel.fa

Suricata suricatta **Testis and Liver -Functional copy**

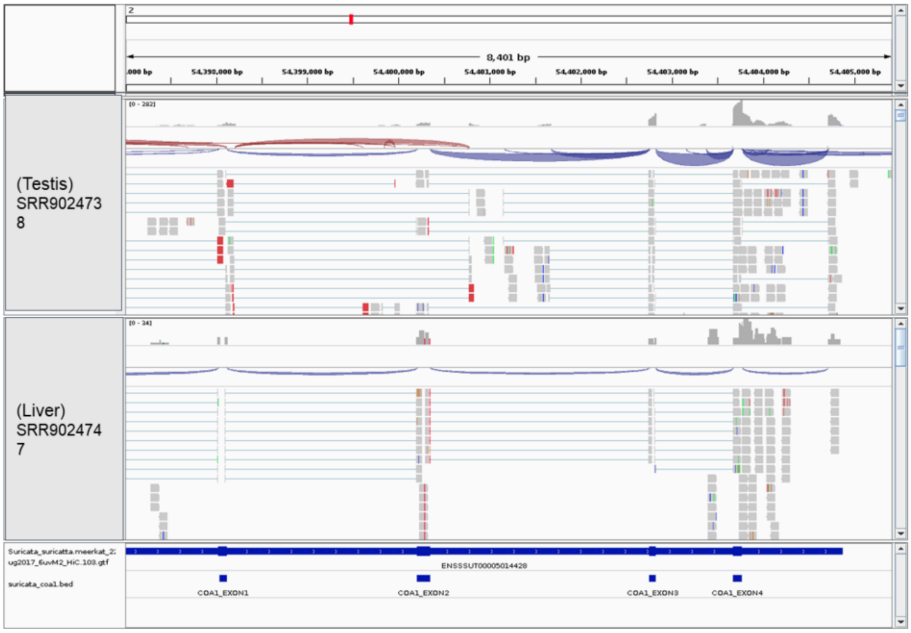

Figure 920: Screenshot of RNA-seq reads aligned to each of the putative locations of the four *COA1* exons in meerkat (*Suricata suricatta*). Gray boxes represent reads aligned to the genome and thin lines connecting these boxes represent the spliced read alignments. Datasets from testis and liver (SRR9024738 and SRR9024747) have been mapped to the Suricata_suricatta.meerkat_22Aug2017_6uvM2_HiC.dna_sm.toplevel.fa. Locations of each of the exons are depicted as bed record in the bottom row.

Figure S921

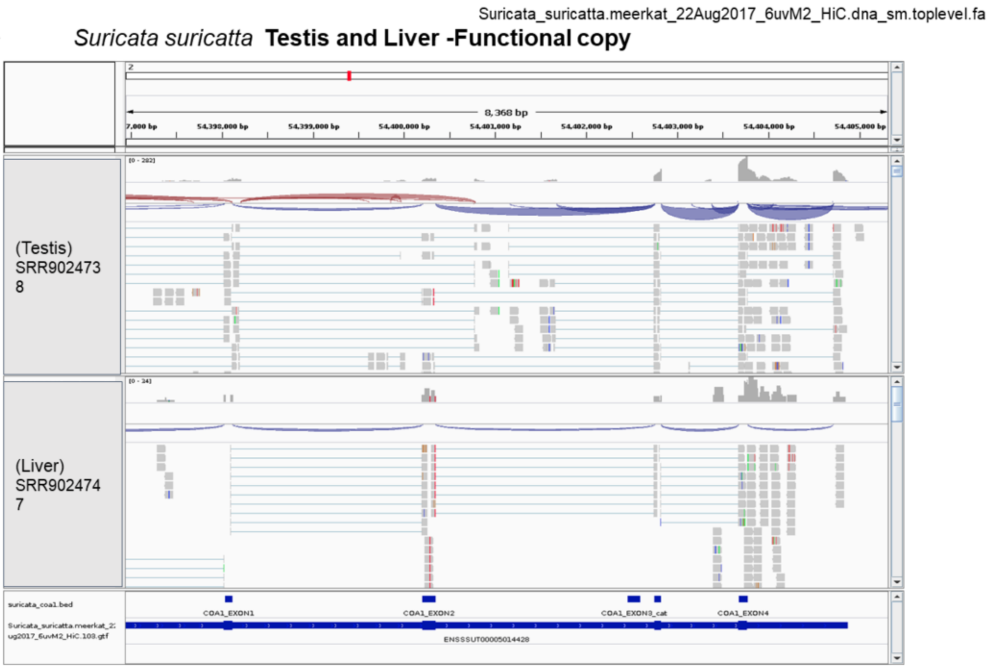

Figure S921: Screenshot of RNA-seq reads aligned to each of the putative locations of the four *COA1* exons with exon 3 cat in meerkat (*Suricata suricatta*). Gray boxes represent reads aligned to the genome and thin lines connecting these boxes represent the spliced read alignments. Datasets from testis and liver (SRR9024738 and SRR9024747) have been mapped to the Suricata_suricatta.meerkat_22Aug2017_6uvM2_HiC.dna_sm.toplevel.fa. Locations of each of the exons are depicted as bed record in the bottom row.

Figure S922

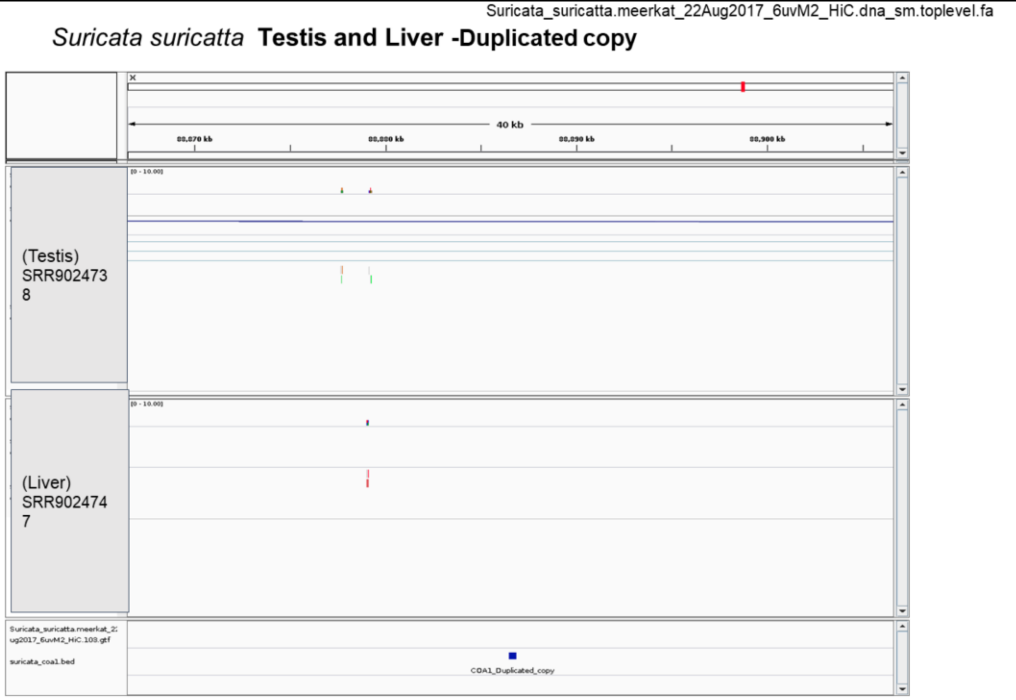

Figure S922: Screenshot of RNA-seq reads aligned to each of the putative locations of the *COA1* duplicated copy in meerkat (*Suricata suricatta*). Datasets from testis and liver (SRR9024738 and SRR9024747) have been mapped to the Suricata_suricatta.meerkat_22Aug2017_6uvM2_HiC.dna_sm.toplevel.fa. Locations of of the *COA1* gene duplicated copy is depicted as bed record in the bottom row.

Figure S923

Canis lupus familiaris **Spleen and Skeletal Muscle -Functional copy**

Canis_lupus_familiaris.CanFam3.1.dna_sm.toplevel.fa

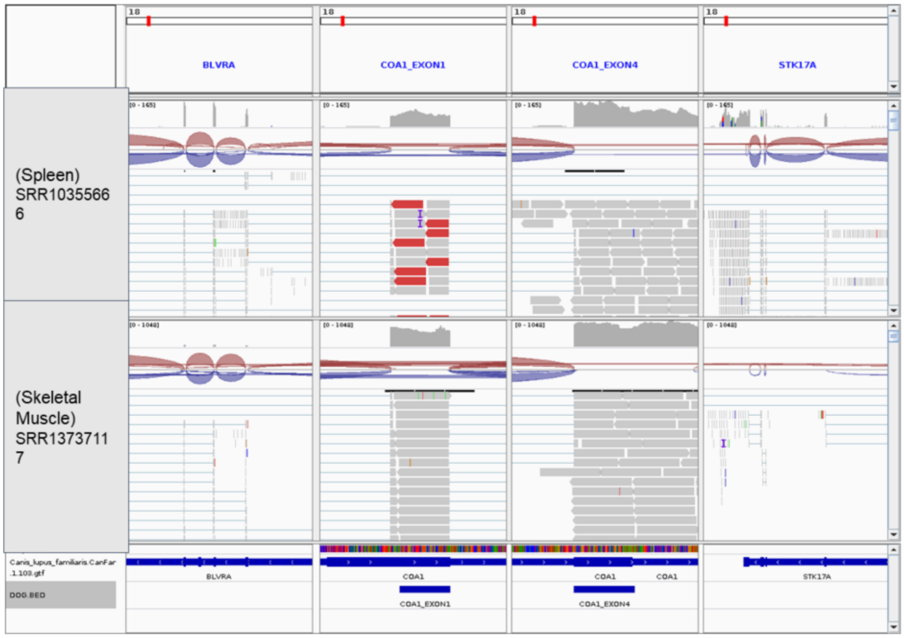

Figure S923: Screenshot of RNA-seq reads aligned to each of the putative locations of *COA1* gene and the flanking genes *BLVRA* and *STK17A* in dog (*Canis lupus familiaris*). Gray boxes represent reads aligned to the genome and thin lines connecting these boxes represent the spliced read alignments. Datasets from spleen and skeletal muscle (SRR10355666 and SRR13737117) have been mapped to the Canis_lupus_familiaris.CanFam3.1.dna_sm.toplevel.fa. Locations of each of the exons are depicted as bed record in the bottom row.

Figure S924

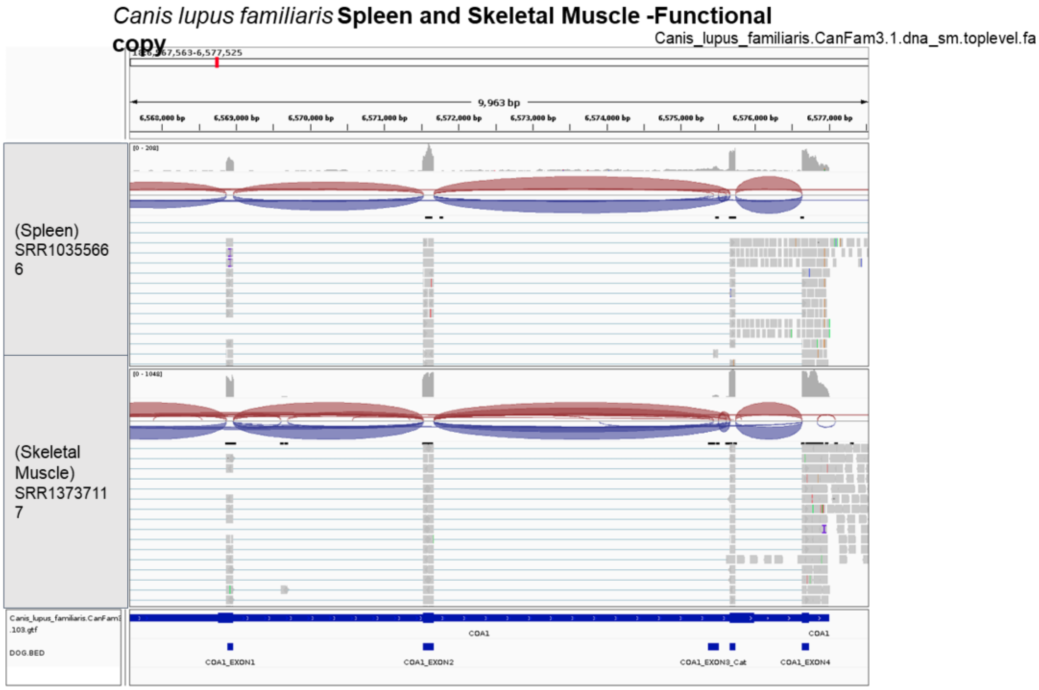

Figure S924: Screenshot of RNA-seq reads aligned to each of the putative locations of the four *COA1* exons in dog (*Canis lupus familiaris*). Gray boxes represent reads aligned to the genome and thin lines connecting these boxes represent the spliced read alignments. Datasets from spleen and skeletal muscle (SRR10355666 and SRR13737117) have been mapped to the Canis_lupus_familiaris.CanFam3.1.dna_sm.toplevel.fa. Locations of each of the exons are depicted as bed record in the bottom row.

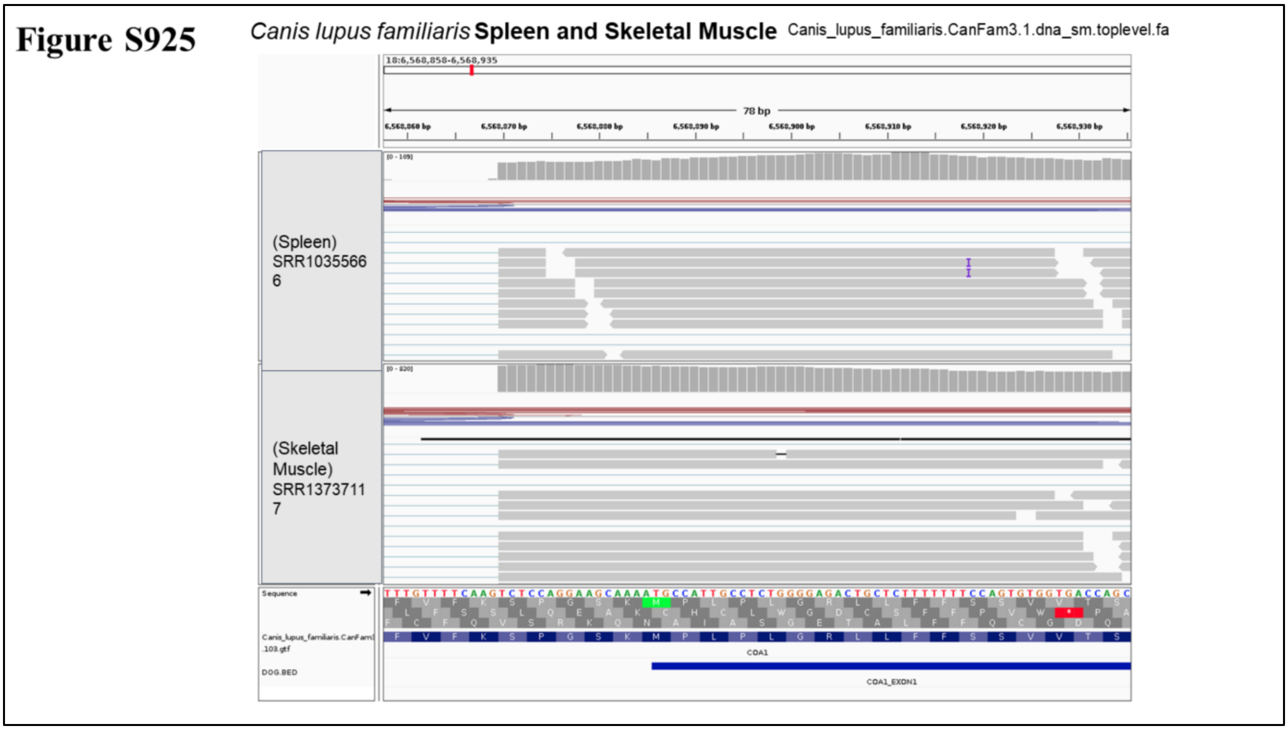

Figure S925: Zoomed view of exon 1 shown in **Supplementary Fig. S924**.

Figure S926

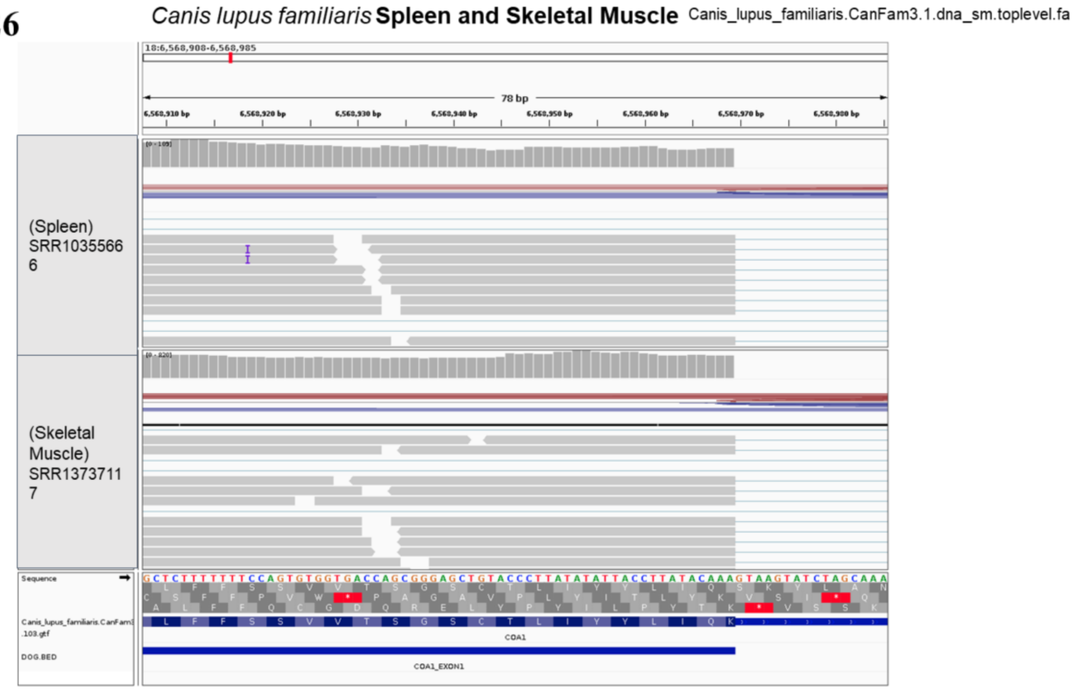

Figure S926: Zoomed view of exon 1 shown in **Supplementary Fig. S924**.

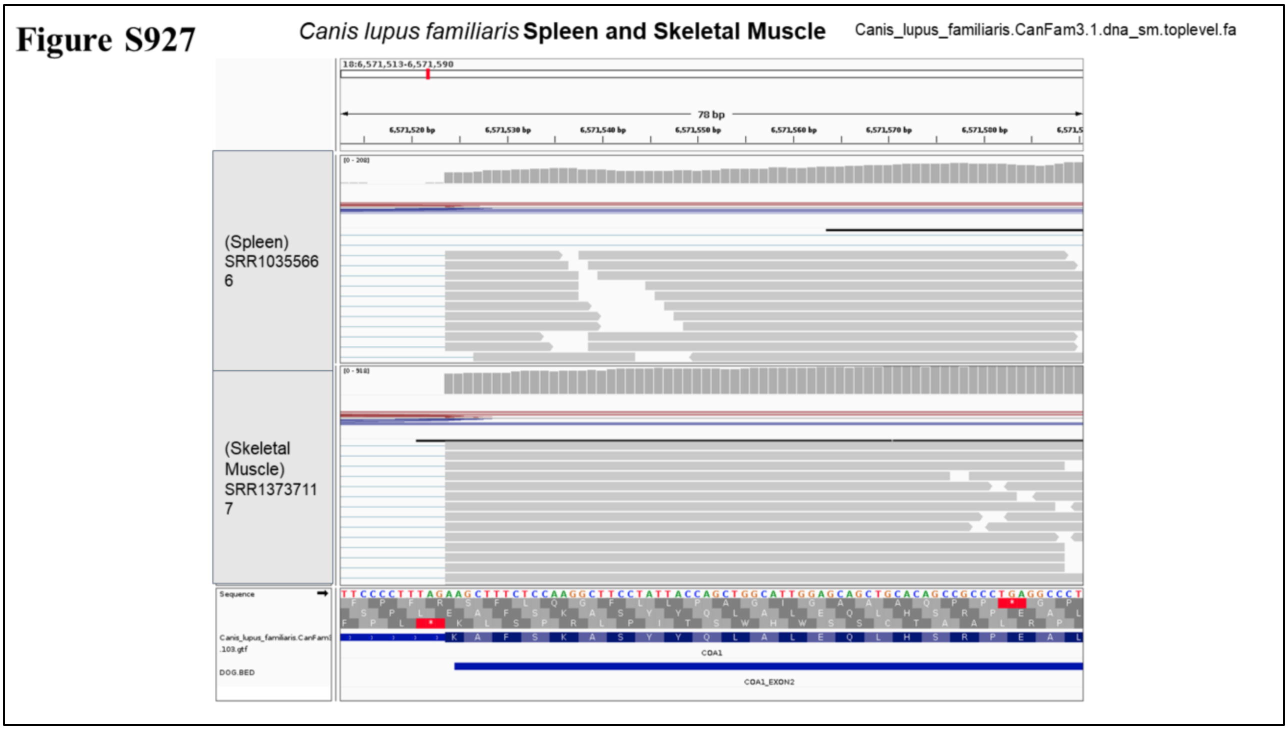

Figure S927: Zoomed view of exon 2 shown in **Supplementary Fig. S924**.

Figure S928

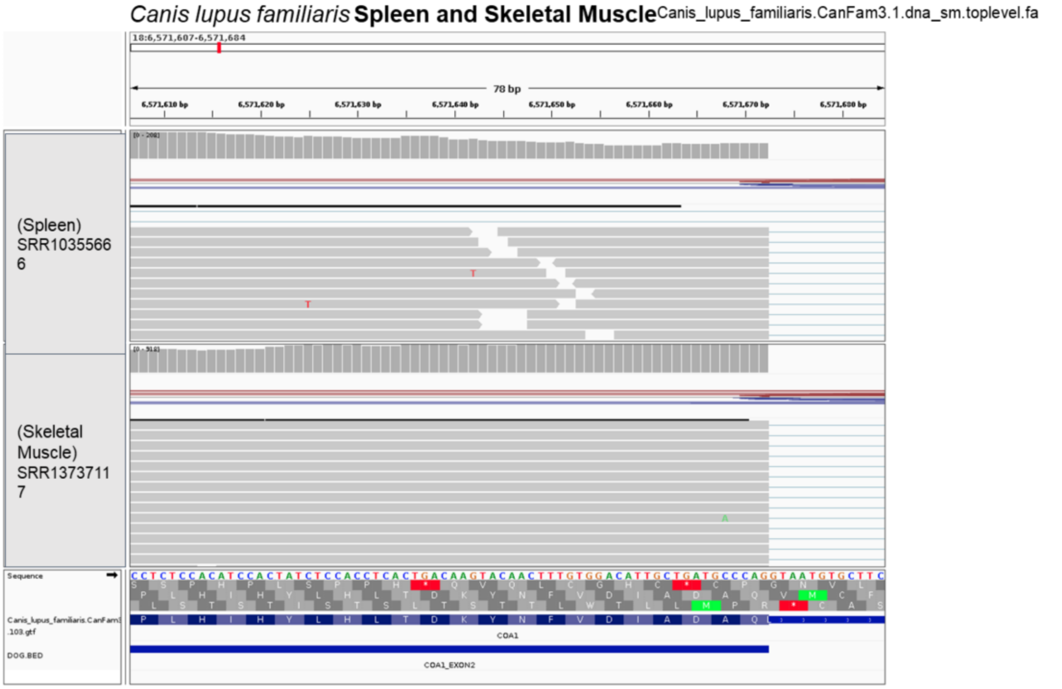

Figure S928: Zoomed view of exon 2 shown in **Supplementary Fig. S924**.

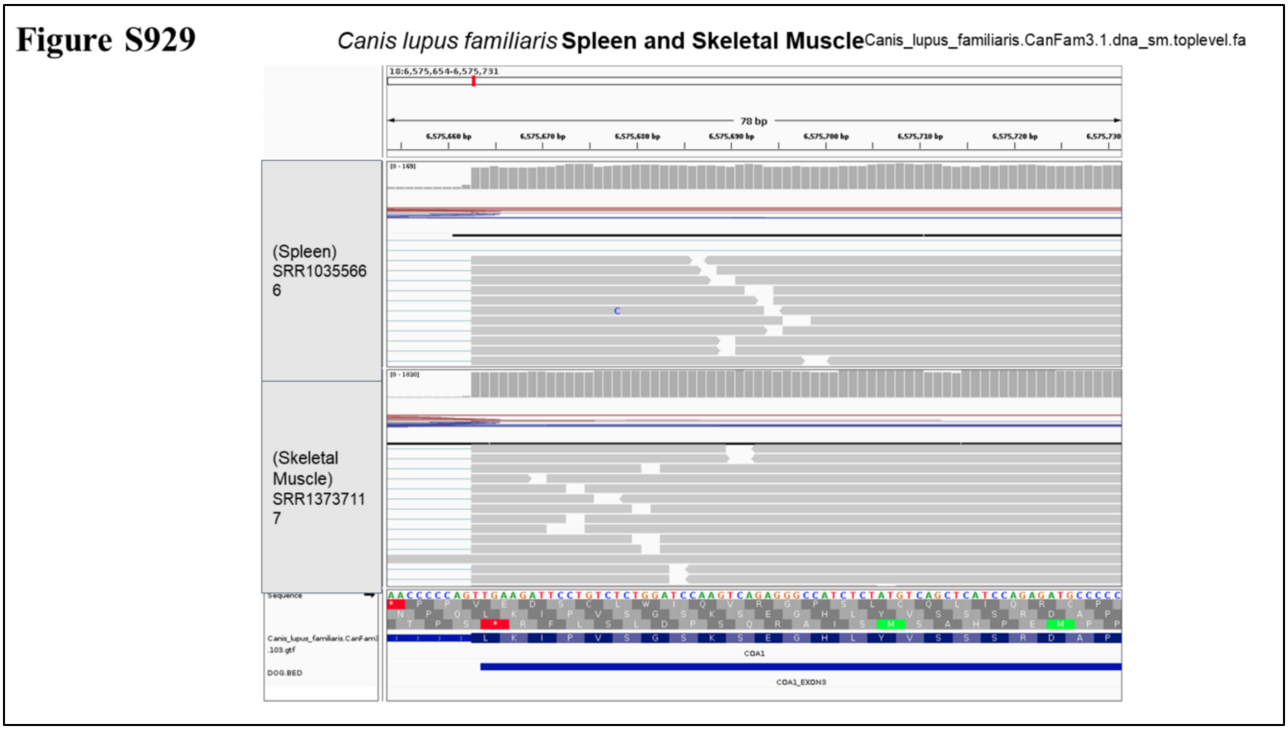

Figure S929: Zoomed view of exon 3 shown in **Supplementary Fig. S924**.

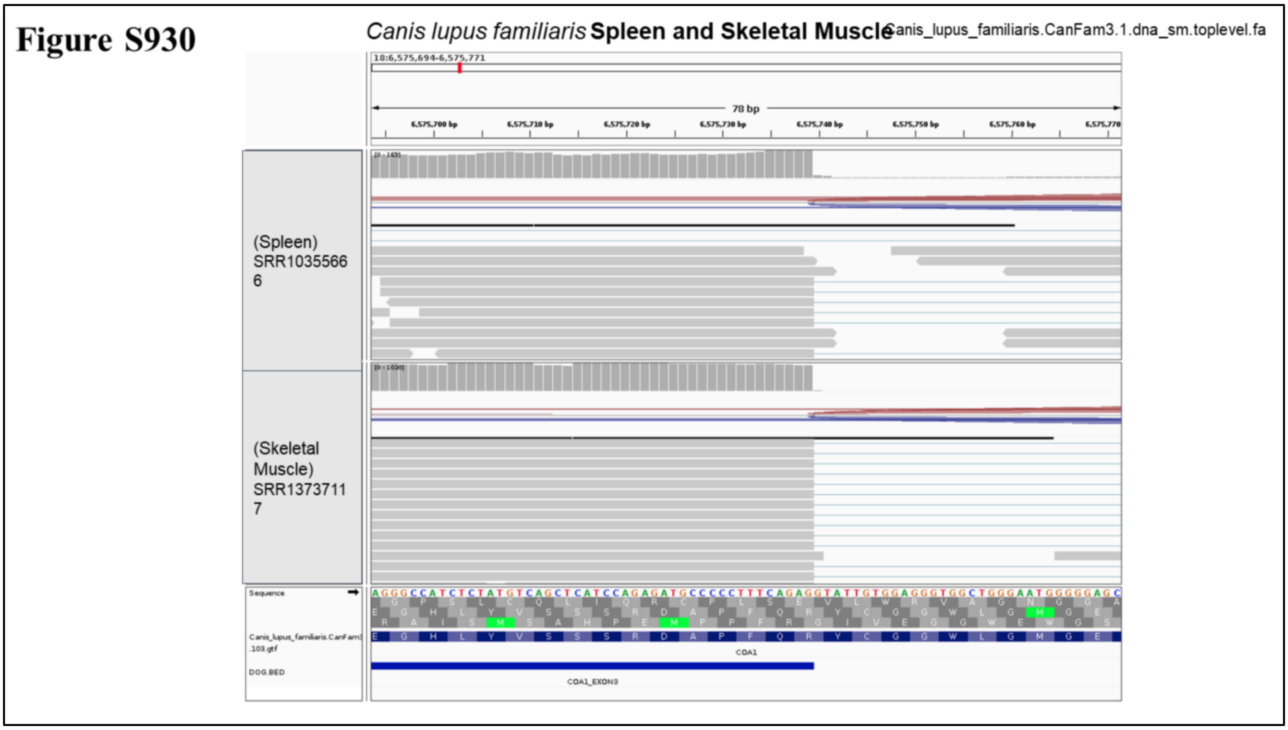

Figure S930: Zoomed view of exon 3 shown in **Supplementary Fig. S924**.

Figure S931

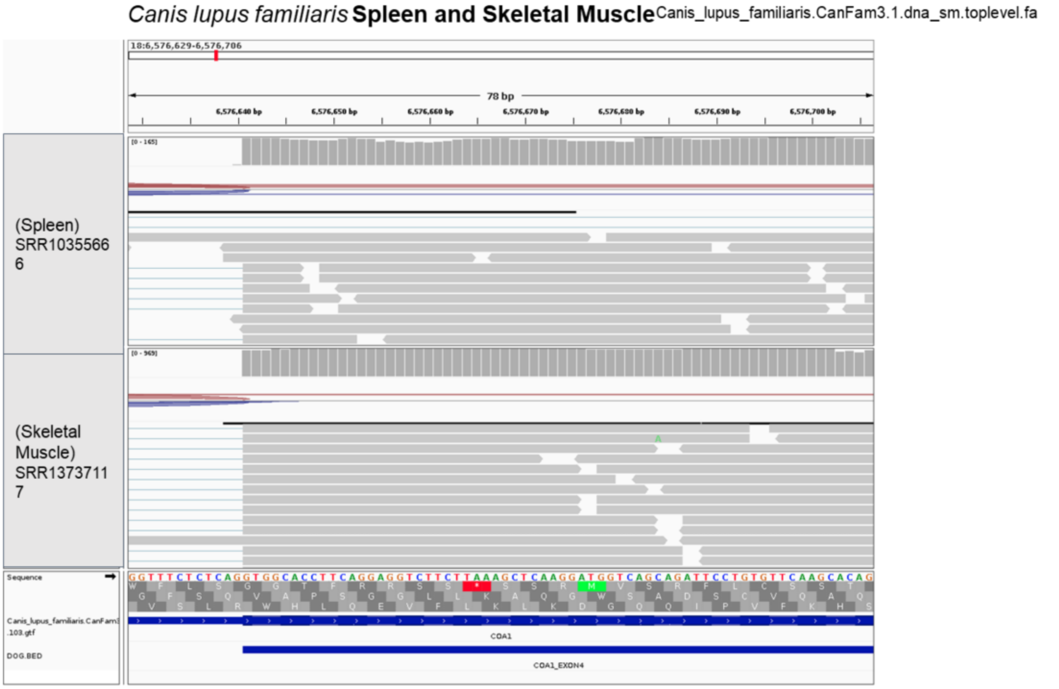

Figure S931: Zoomed view of exon 4 shown in **Supplementary Fig. S924**.

Figure S932

Canis lupus familiaris **Spleen and Skeletal Muscle** Canis_lupus_familiaris.CanFam3.1.dna_sm.toplevel.fa

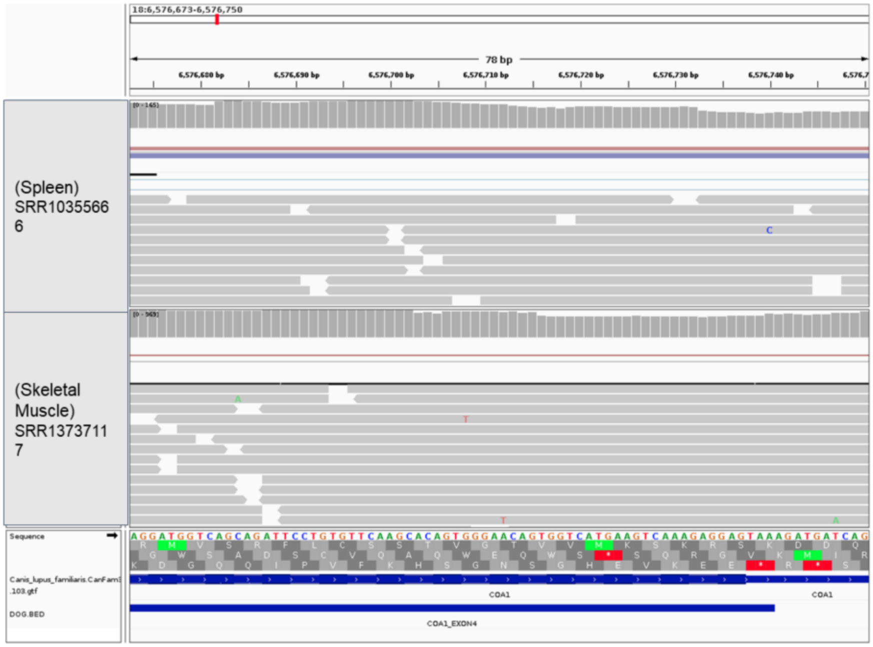

Figure S932: Zoomed view of exon 4 shown in **Supplementary Fig. S924**.

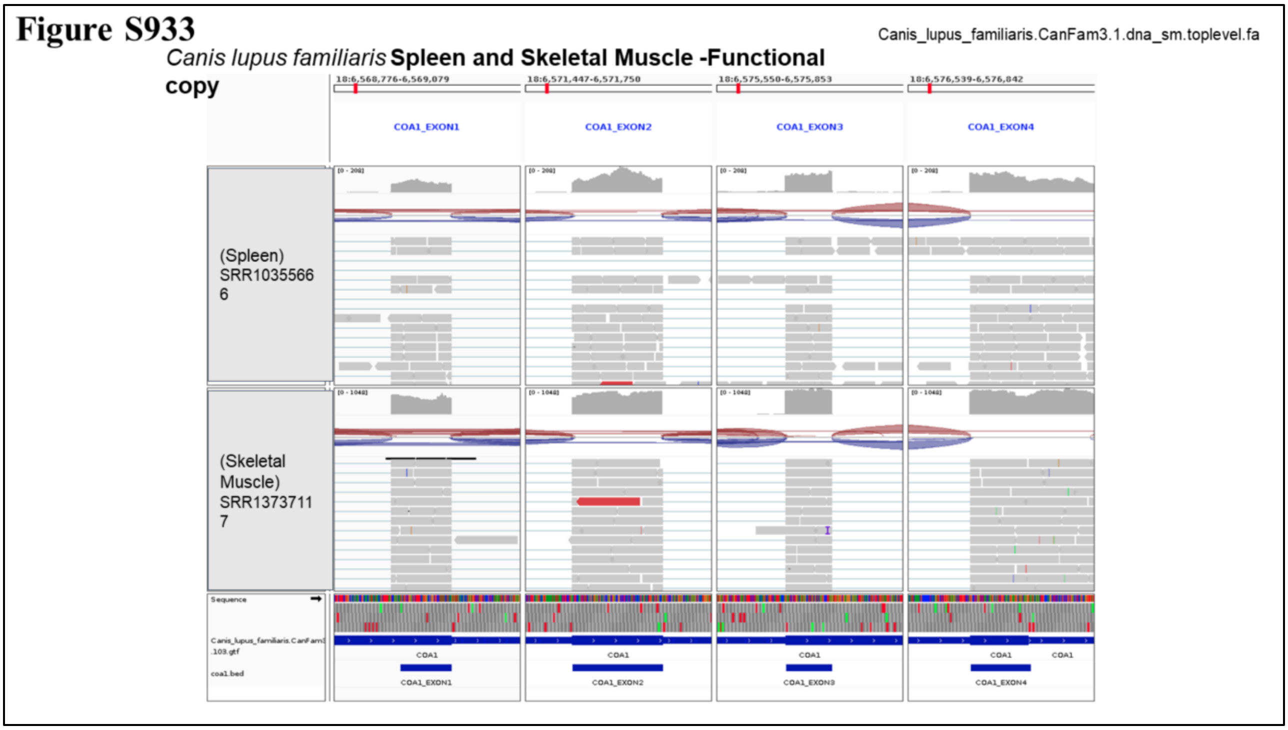

Figure S933: A zoomed in view of each of the *COA1* exons shown in **Supplementary Fig. S924**.

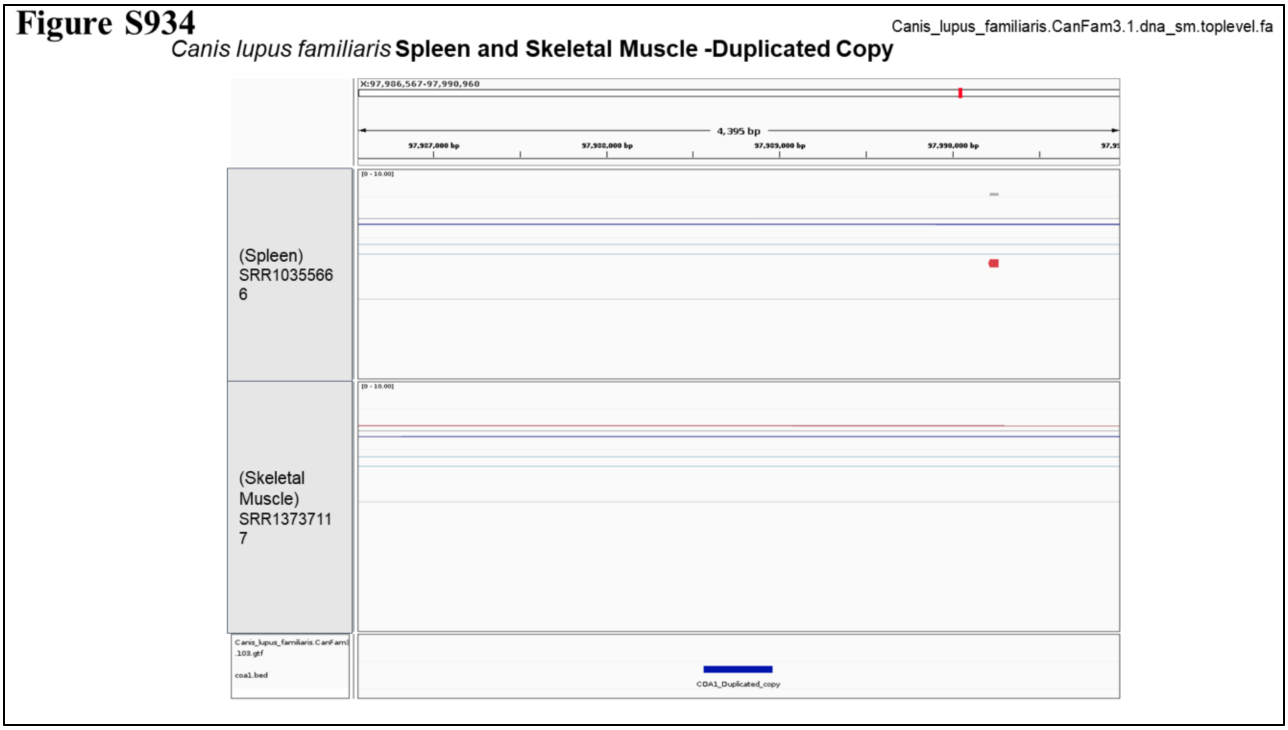

Figure S934: Sreenshot of RNA-seq reads aligned to each of the putative locations of the *COA1* duplicated copy in dog (*Canis lupus familiaris*). Datasets from spleen and skeletal muscle (SRR10355666 and SRR3218716) have been mapped to the Canis_lupus_familiaris.CanFam3.1.dna_sm.toplevel.fa. Locations of of the *COA1* gene duplicated copy is depicted as bed record in the bottom row.

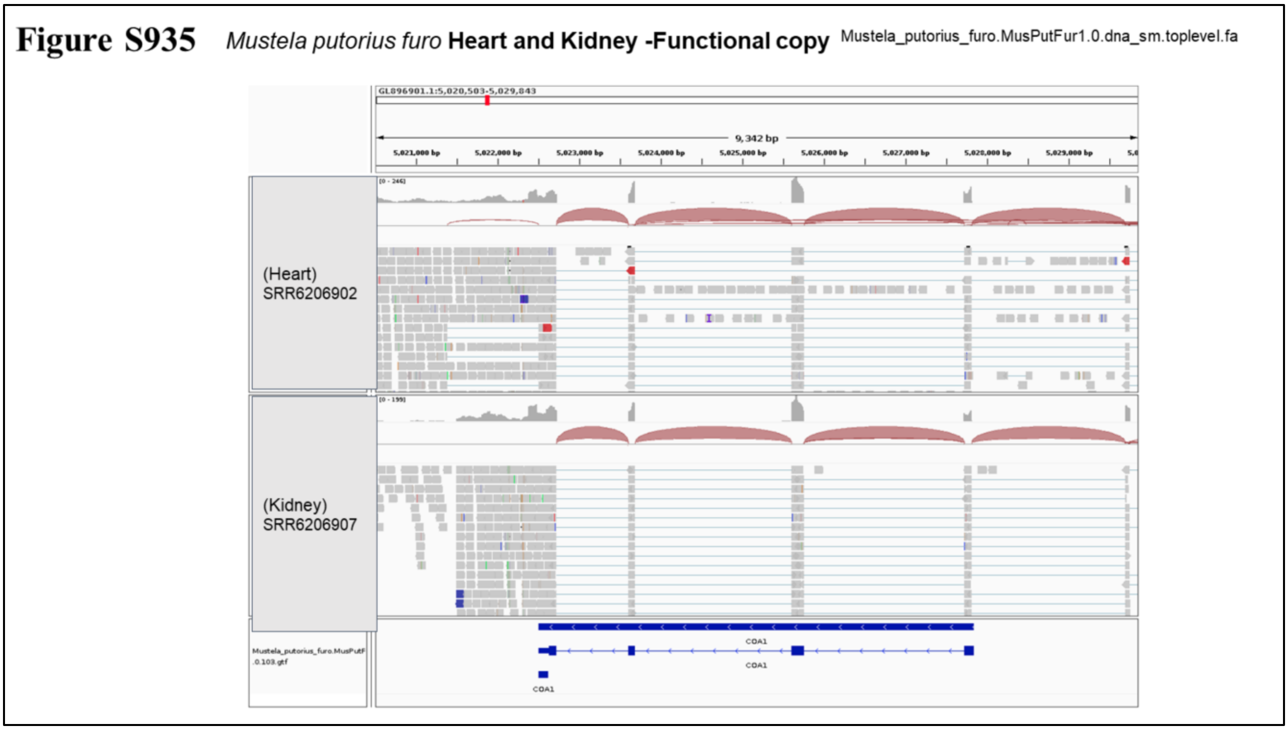

Figure S935: Screenshot of RNA-seq reads aligned to each of the putative locations of the four *COA1* exons in Ferret (*Mustela putorius furo*). Gray boxes represent reads aligned to the genome and thin lines connecting these boxes represent the spliced read alignments. Datasets from heart and kidney (SRR6206902 and SRR6206907) have been mapped to the Mustela_putorius_furo.MusPutFur1.0.dna_sm.toplevel.fa. Locations of each of the exons are depicted as bed record in the bottom row.

Figure S936 *Mustela putorius furo* **Heart and Kidney -Duplicated copy** Mustela_putorius_furo.MusPutFur1.0.dna_sm.toplevel.fa

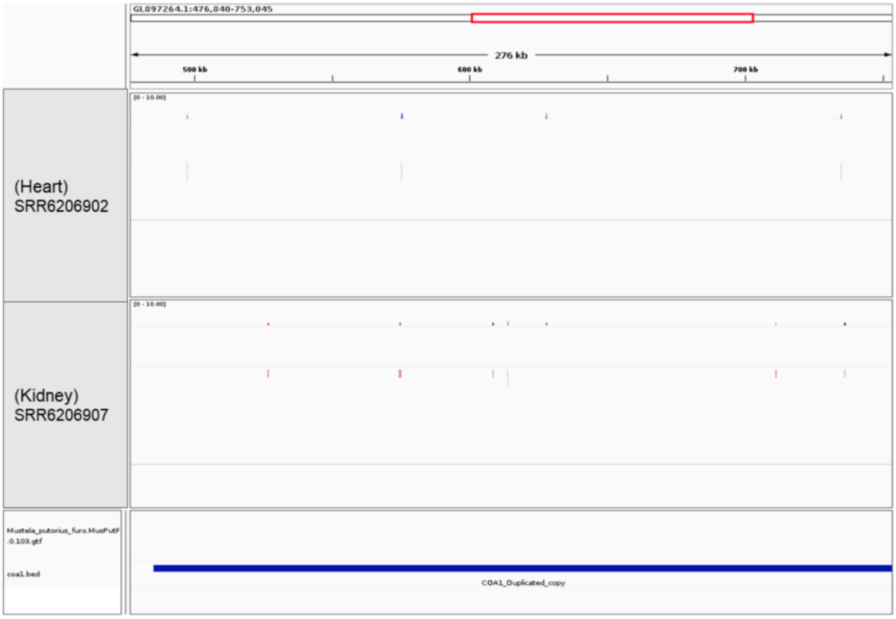

Figure S936: Sreenshot of RNA-seq reads aligned to each of the putative locations of the *COA1* duplicated copy in ferret (*Mustela putorius furo*). Datasets from heart and kidney (SRR6206902 and SRR6206907) have been mapped to the Mustela_putorius_furo.MusPutFur1.0.dna_sm.toplevel.fa. Locations of of the *COA1* gene duplicated copy is depicted as bed record in the bottom row.

Figure S937

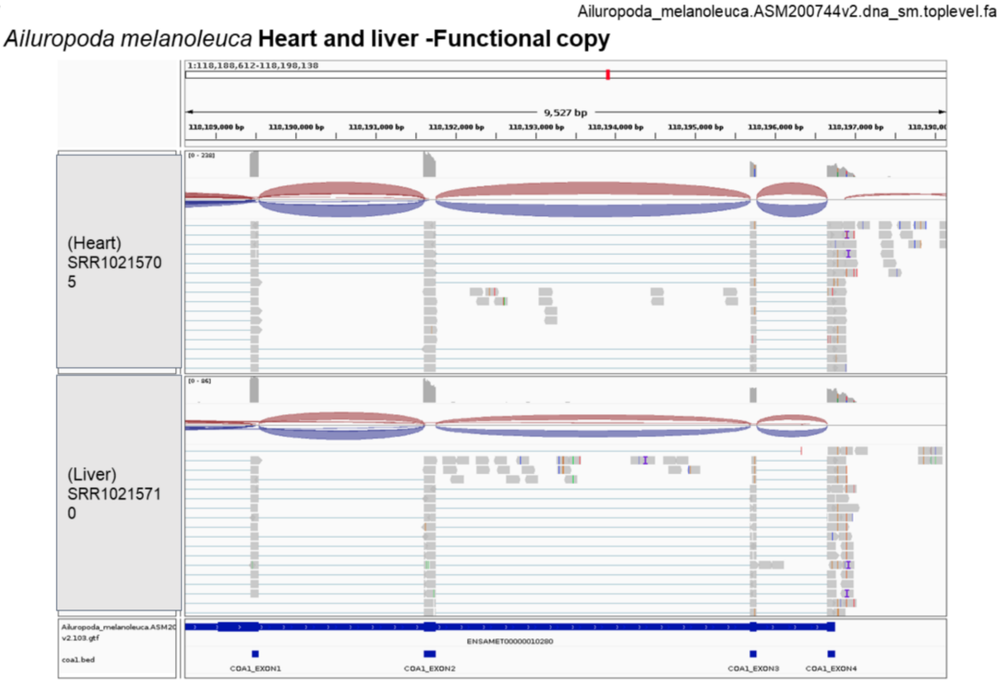

Figure S937: Screenshot of RNA-seq reads aligned to each of the putative locations of the four *COA1* exons in Giant panda (*Ailuropoda melanoleuca*). Gray boxes represent reads aligned to the genome and thin lines connecting these boxes represent the spliced read alignments. Datasets from heart and liver (SRR10215705 and SRR10215710) have been mapped to the Ailuropoda_melanoleuca.ASM200744v2.dna_sm.toplevel.fa. Locations of each of the exons are depicted as bed record in the bottom row.

Figure S938

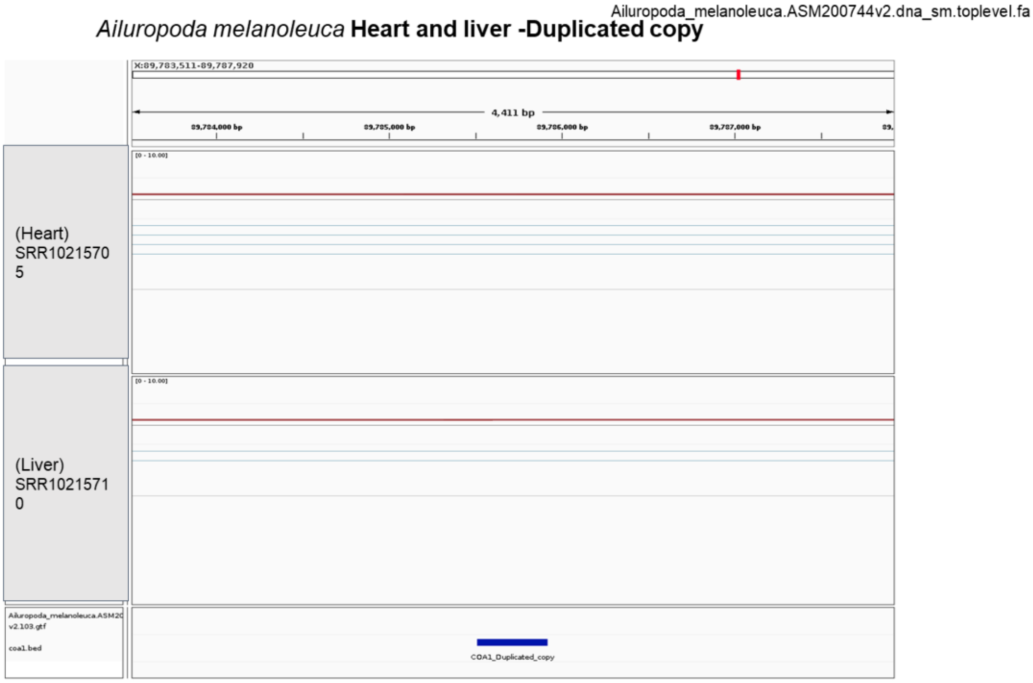

Figure S938: Sreenshot of RNA-seq reads aligned to each of the putative locations of the *COA1* duplicated copy in Giant panda (*Ailuropoda melanoleuca*). Datasets from heart and liver (SRR10215705 and SRR10215710) have been mapped to the Ailuropoda_melanoleuca.ASM200744v2.dna_sm.toplevel.fa. Locations of of the *COA1* gene duplicated copy is depicted as bed record in the bottom row.

Figure S939

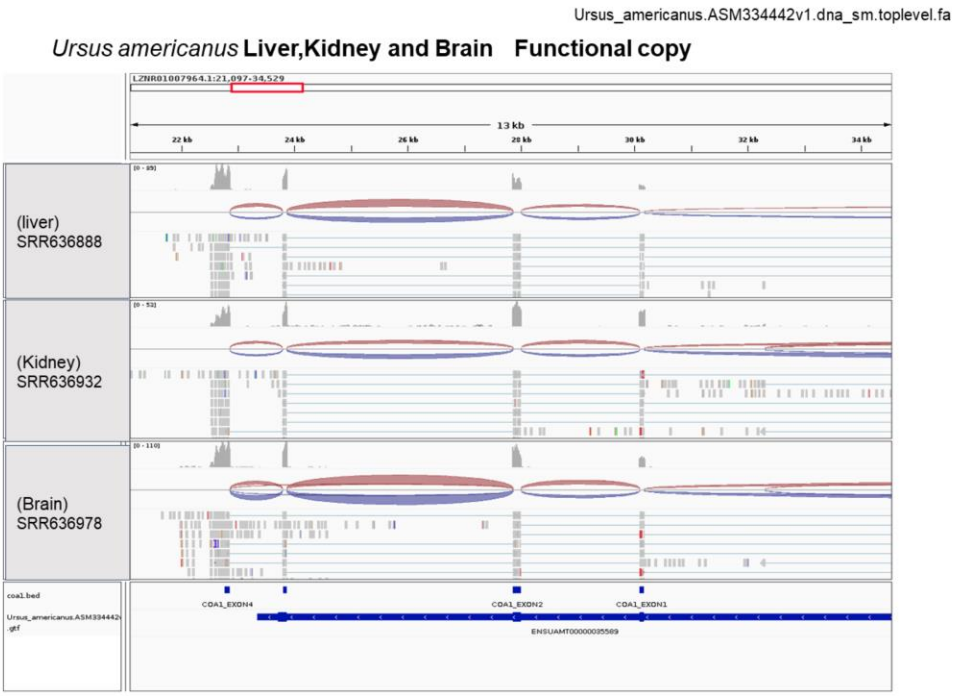

Figure S939: Screenshot of RNA-seq reads aligned to each of the putative locations of the four *COA1* exons in American black bear(*Ursus americanus*). Gray boxes represent reads aligned to the genome and thin lines connecting these boxes represent the spliced read alignments. Datasets from liver, kidney and brain (SRR636888, SRR636932 and SRR636978) have been mapped to the Ursus_americanus.ASM334442v1.dna_sm.toplevel.fa. Locations of each of the exons are depicted as bed record in the bottom row.

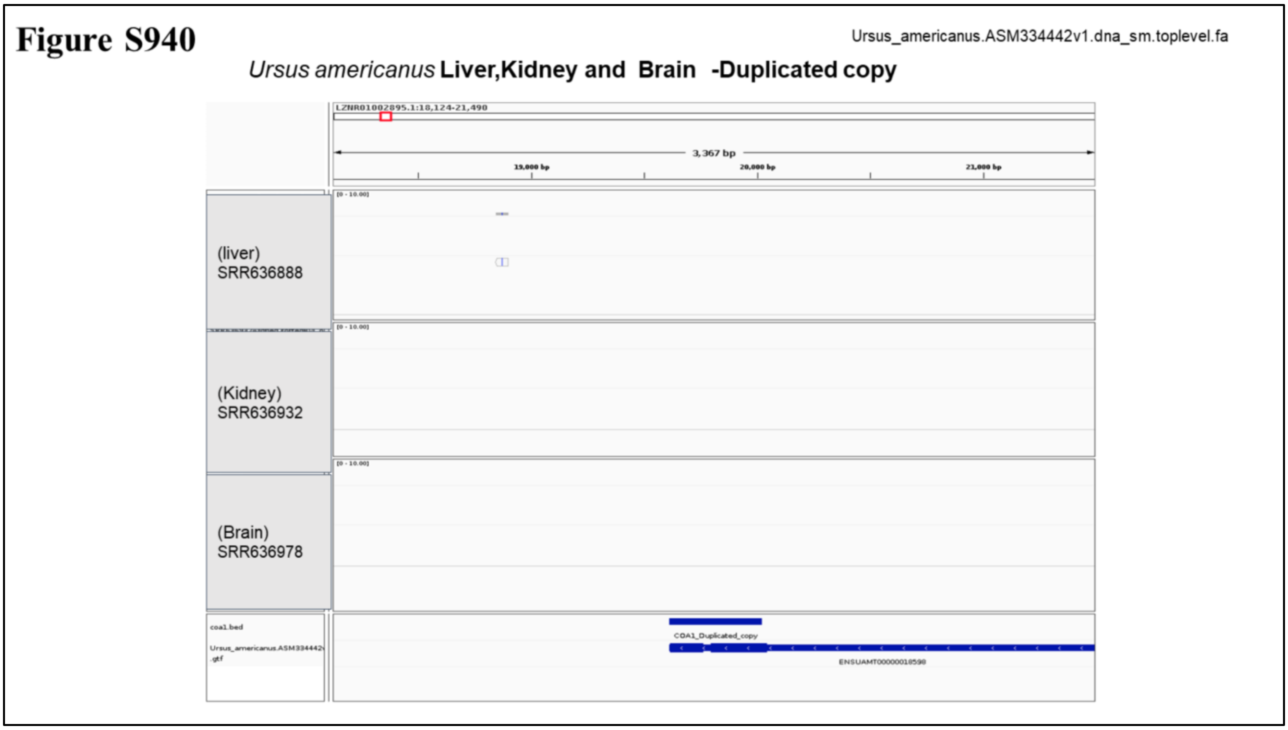

Figure S940: Screenshot of RNA-seq reads aligned to each of the putative locations of the *COA1* duplicated copy in American black bear(*Ursus americanus*). Datasets from liver, kidney and brain (SRR636888, SRR636932 and SRR636978) have been mapped to the Ursus_americanus.ASM334442v1.dna_sm.toplevel.fa. Locations of of the *COA1* gene duplicated copy is depicted as bed record in the bottom row.

Figure S941 *Leptonychotes weddellii* **Lung and Muscle** GCF_000349705.1_LepWed1.0_genomic.fna

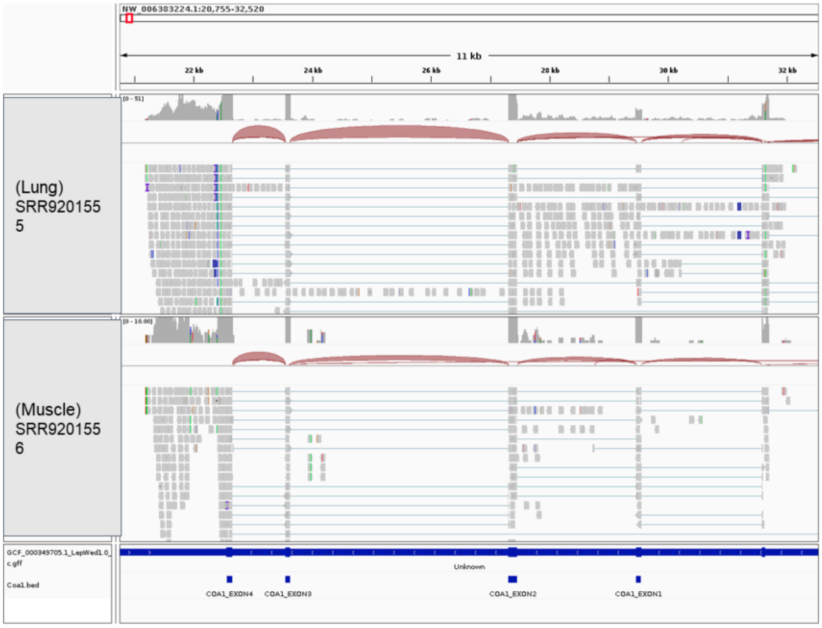

Figure S941: Screenshot of RNA-seq reads aligned to each of the putative locations of the four *COA1* exons in Weddell seal (*Leptonychotes weddellii*). Gray boxes represent reads aligned to the genome and thin lines connecting these boxes represent the spliced read alignments. Datasets from lung and muscle (SRR9201555 and SRR9201556) have been mapped to the GCF_000349705.1_LepWed1.0_genomic.fna. Locations of each of the exons are depicted as bed record in the bottom row.

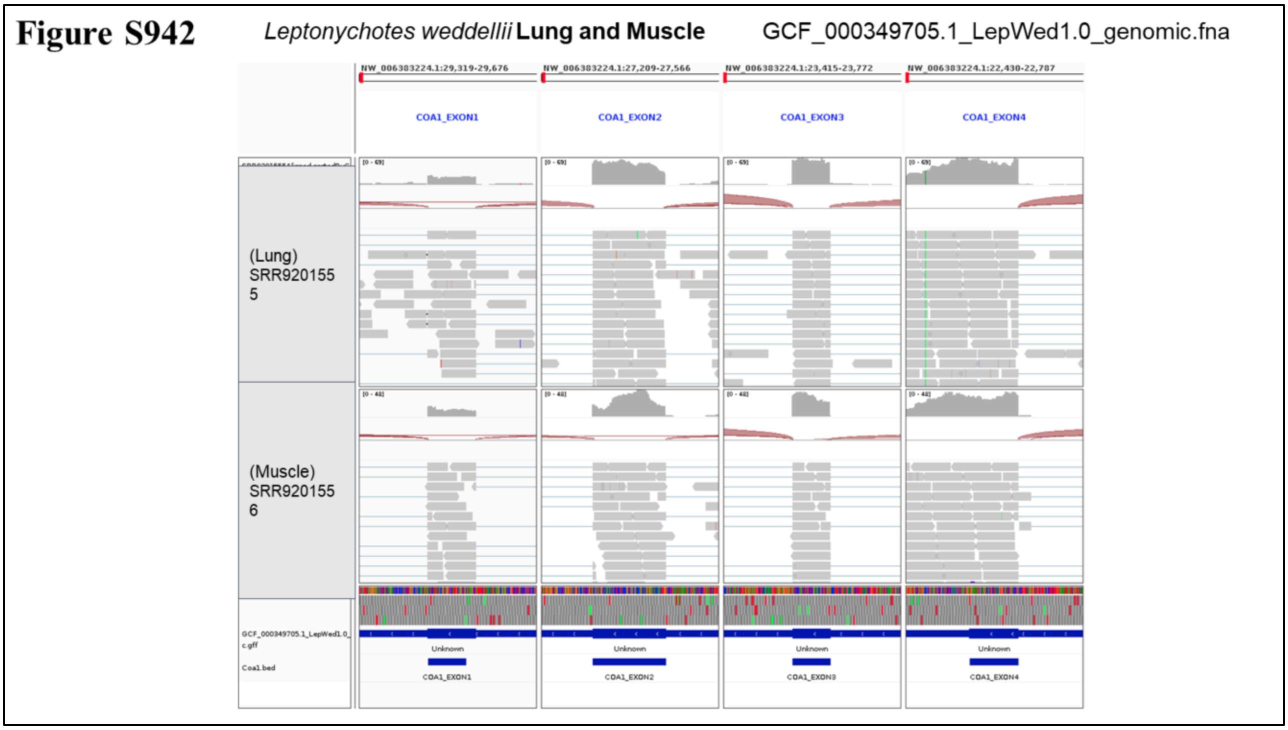

Figure S942: A zoomed in view of each of the *COA1* exons shown in **Supplementary Fig. S941**.

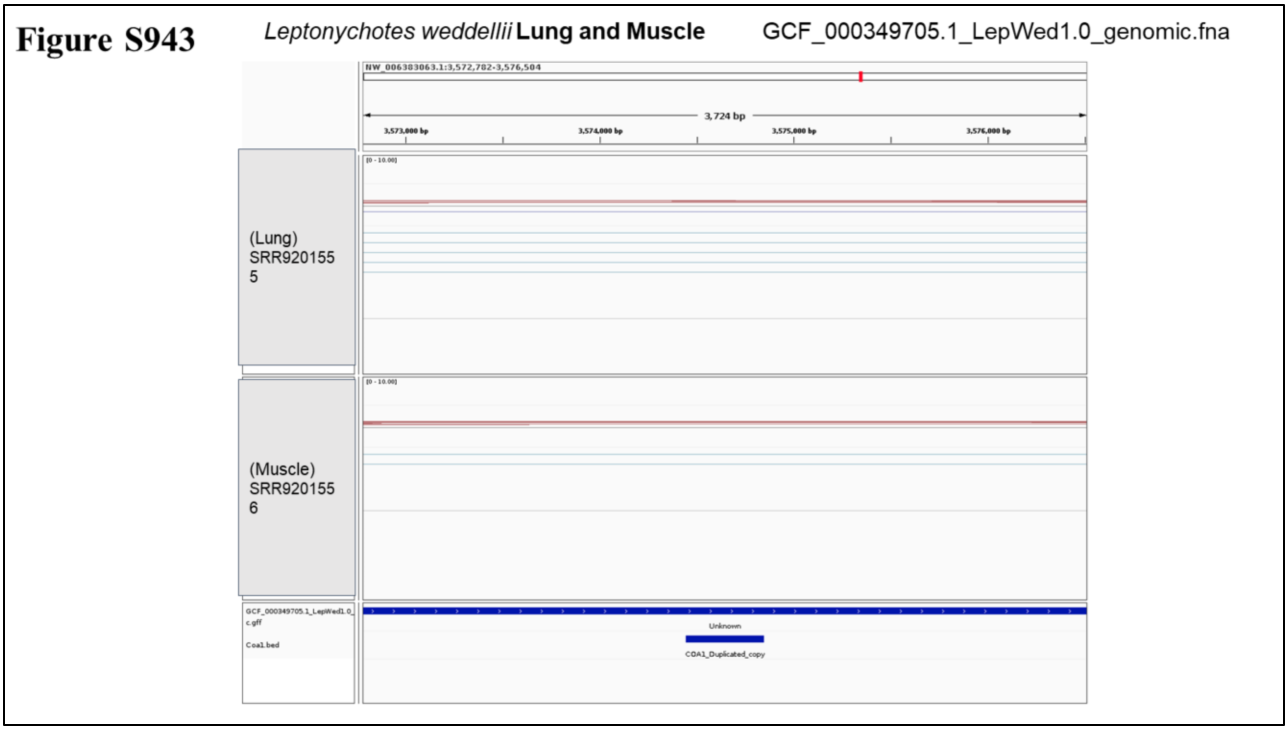

Figure S943: Screenshot of RNA-seq reads aligned to each of the putative locations of the *COA1* duplicated copy in Weddell seal (*Leptonychotes weddellii*). Gray boxes represent reads aligned to the genome and thin lines connecting these boxes represent the spliced read alignments. Datasets from lung and muscle (SRR9201555 and SRR9201556) have been mapped to the GCF_000349705.1_LepWed1.0_genomic.fna. Locations of each of the exons are depicted as bed record in the bottom row.

Figure S944 *Panthera tigris altaica* **Blood -Functional copy** Panthera_tigris_altaica.PanTig1.0.dna_sm.toplevel.fa

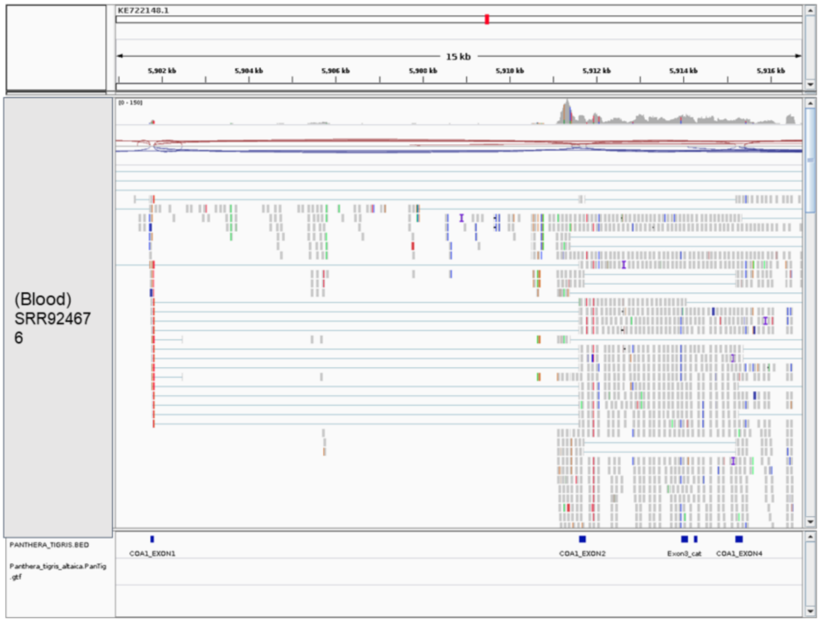

Figure S944: Screenshot of RNA-seq reads aligned to each of the putative locations of the three *COA1* exons in Siberian Tiger (*Panthera tigris altaica*). Gray boxes represent reads aligned to the genome and thin lines connecting these boxes represent the spliced read alignments. Datasets from blood (SRR924676) have been mapped to the Panthera_tigris_altaica.PanTig1.0.dna_sm.toplevel.fa. Locations of each of the exons are depicted as bed record in the bottom row.

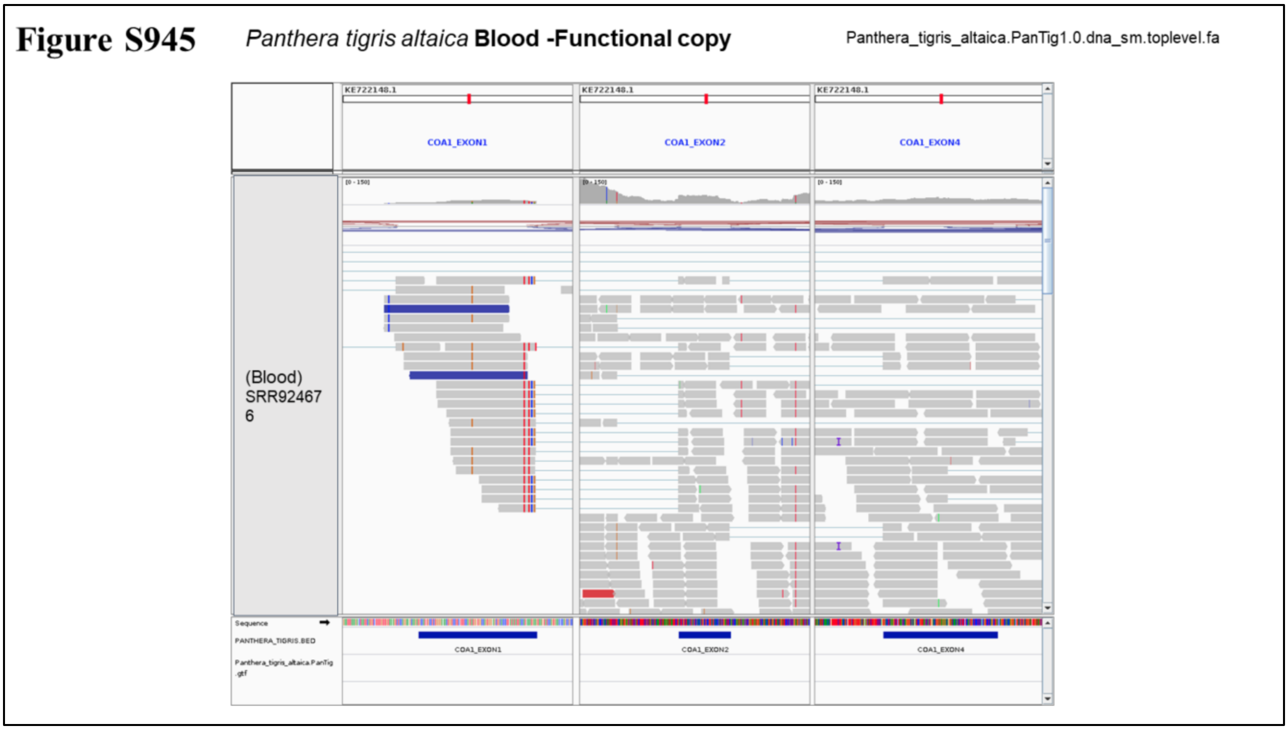

Figure S945: Zoomed view of all three exons 1, 2, and 4 containing cds of *COA1* shown in **Supplementary Fig. S944**.

Figure S946

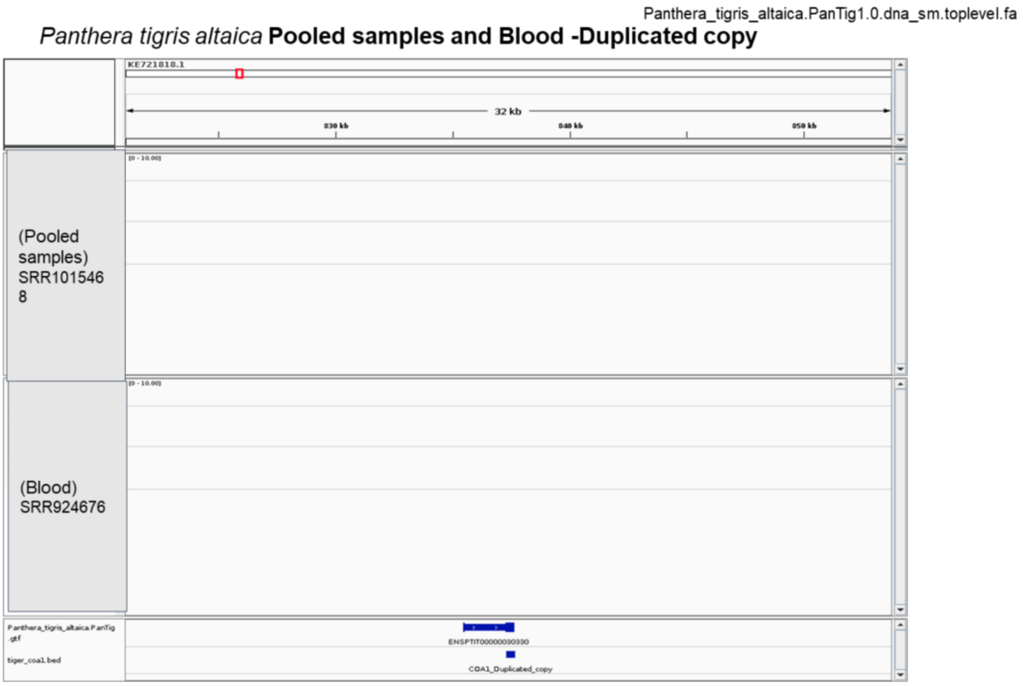

Figure S946: Screenshot of RNA-seq reads aligned to each of the putative locations of the *COA1* duplicated copy in Siberian Tiger (*Panthera tigris altaica*). Datasets from Pooled samples and blood (SRR1015468 and SRR924676) have been mapped to the Panthera_tigris_altaica.PanTig1.0.dna_sm.toplevel.fa. Locations of of the *COA1* duplicated copy is depicted as bed record in the bottom row.

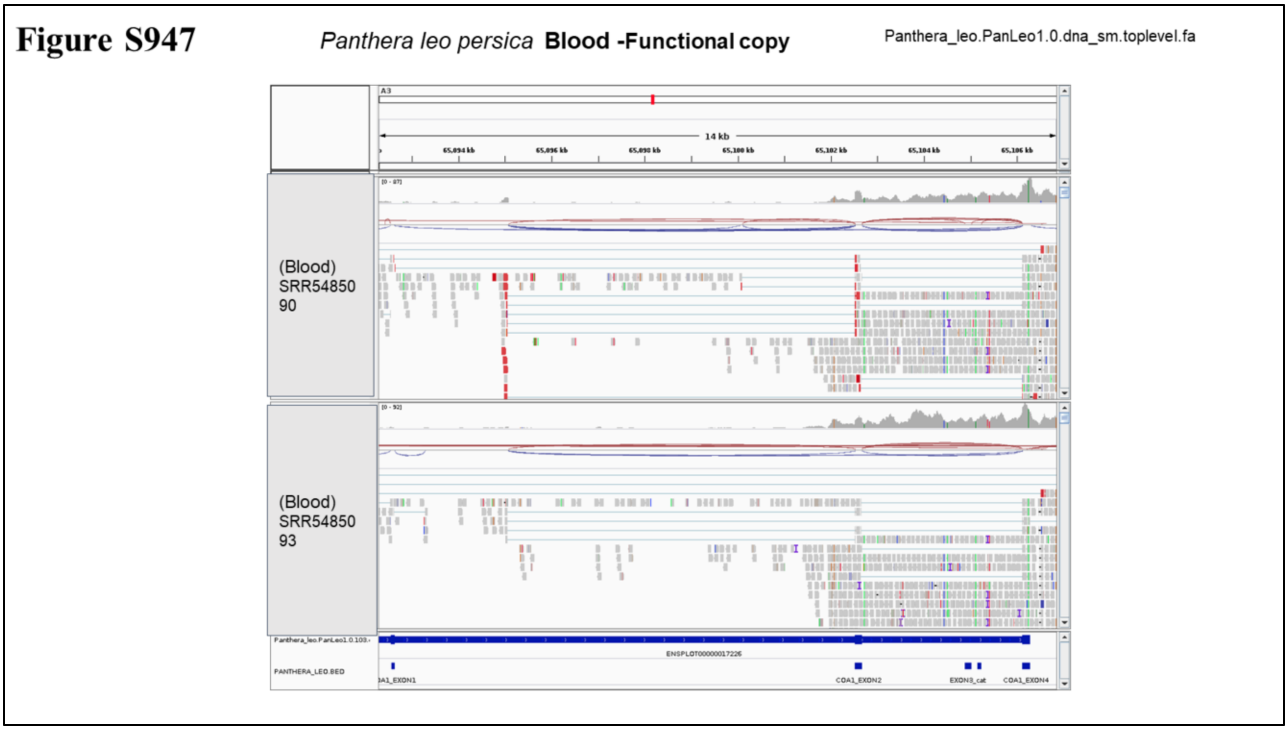

Figure S947: Screenshot of RNA-seq reads aligned to each of the putative locations of the four *COA1* exons in Asiatic lion (*Panthera leo persica*). Gray boxes represent reads aligned to the genome and thin lines connecting these boxes represent the spliced read alignments. Datasets from blood (SRR5485090 and SRR5485093) have been mapped to the Panthera_leo.PanLeo1.0.dna_sm.toplevel.fa. Locations of each of the exons are depicted as bed record in the bottom row.

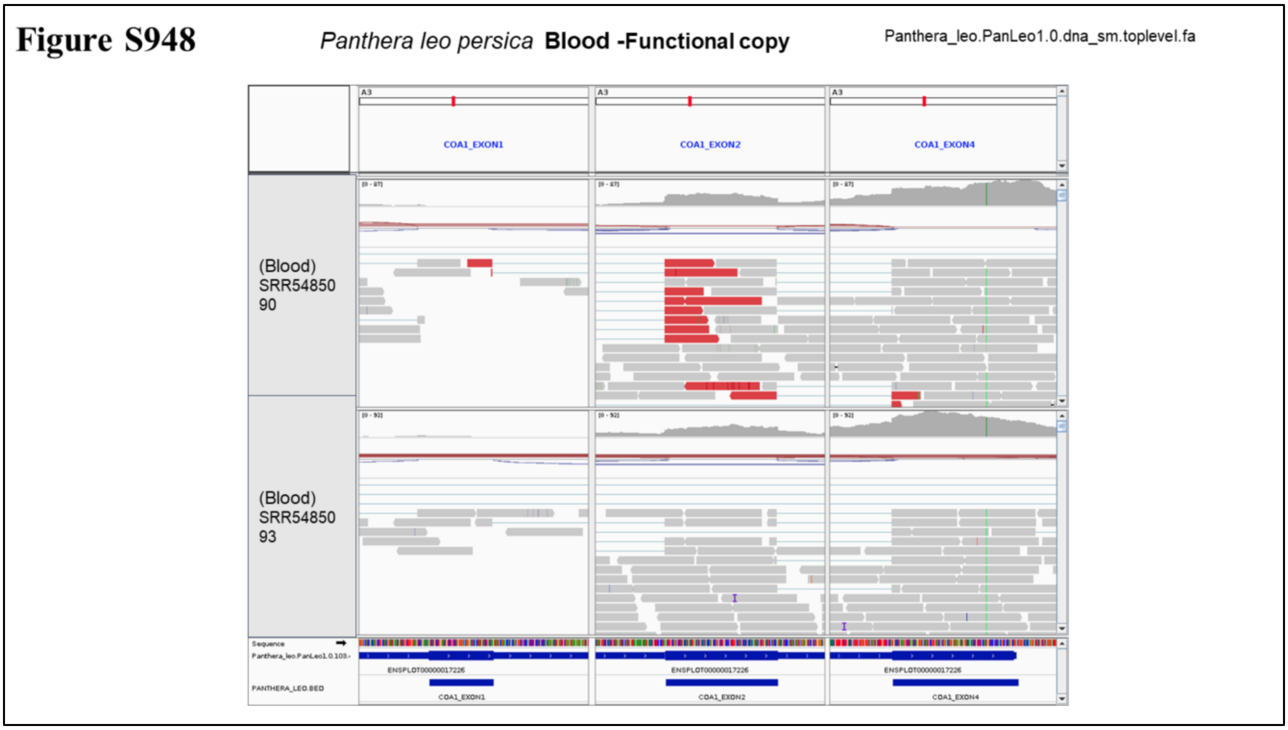

Figure S948: Zoomed view of all CDS containing three exons.

Figure S949

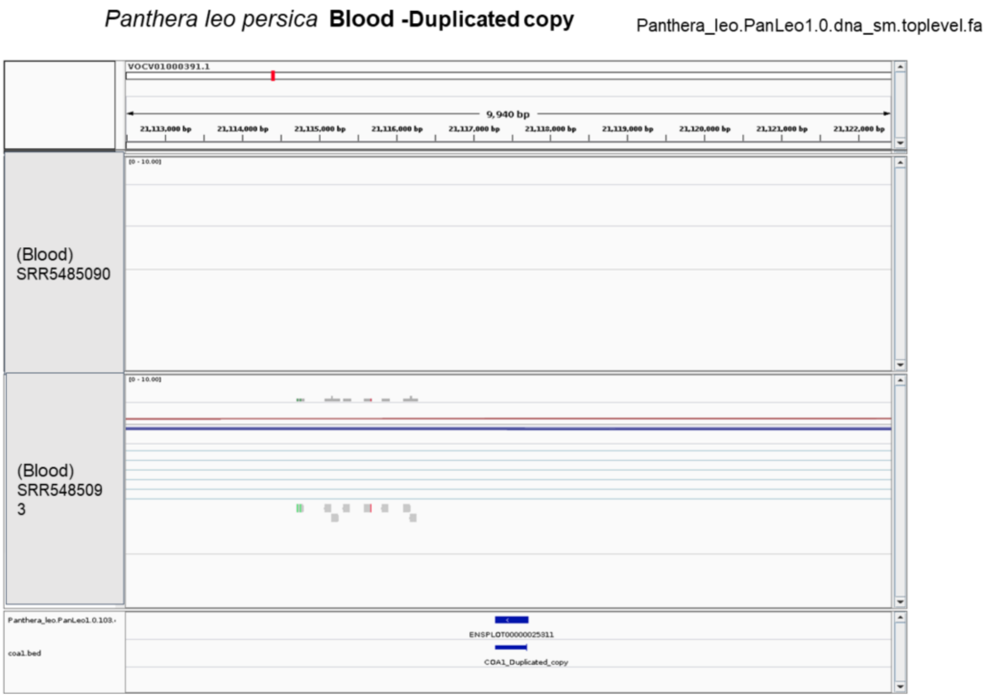

Figure S949: Screenshot of RNA-seq reads aligned to each of the putative locations of the *COA1* duplicated copy in Asiatic lion (*Panthera leo persica*). Datasets from blood (SRR5485090 and SRR5485093) have been mapped to the Panthera_leo.PanLeo1.0.dna_sm.toplevel.fa. Locations of of the *COA1* gene duplicated copy is depicted as bed record in the bottom row.

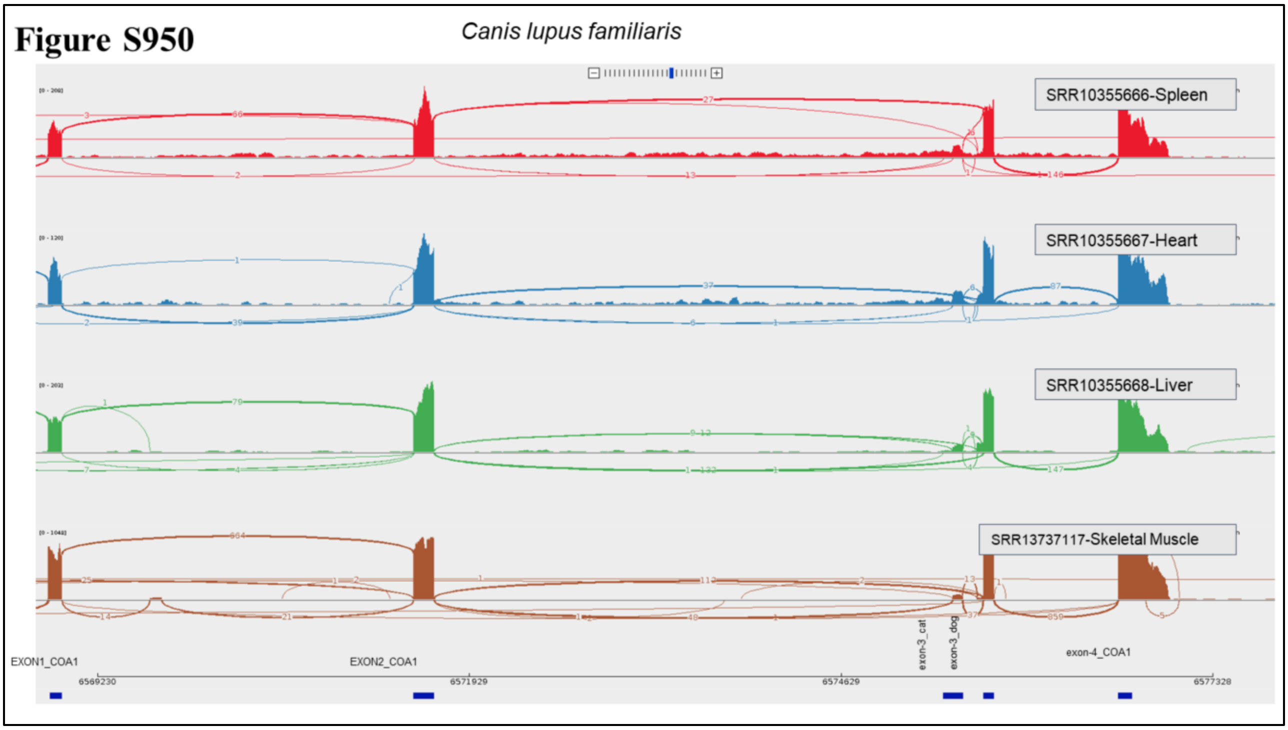

Figure S950: Screenshot of Sashimi plot of *COA1* exon 1, 2, exon-3 cat, exon-3 dog, and exon 4 in dog (*Canis lupus familiaris*). Red, blue, green and brown lines are different tissues and circles represent the spliced reads in different exons with coverage.

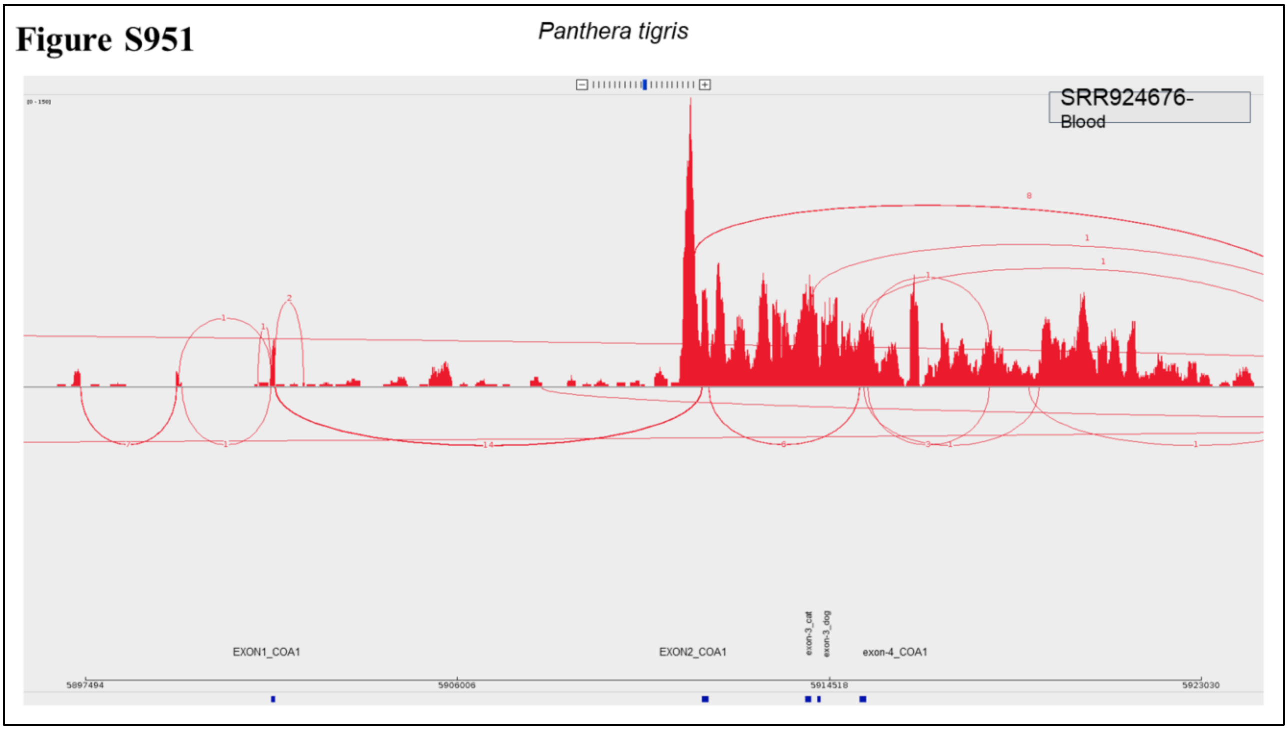

Figure S954: Screenshot of Sashimi plot of *COA1* exon 1, 2, exon-3 cat, exon-3 dog, and exon 4 in *Panthera tigris*. Red circles represent the spliced reads in different exons with coverage.

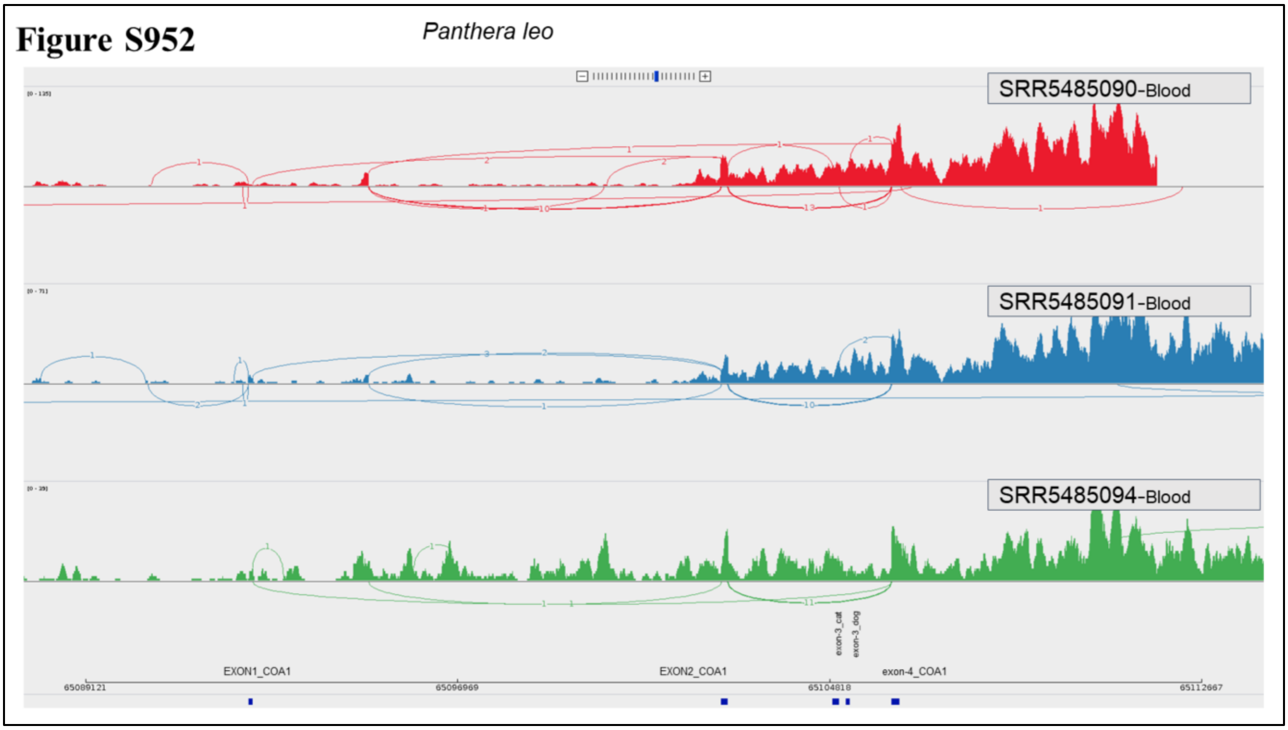

Figure S952: Screenshot of Sashimi plot of *COA1* exon 1, 2, exon-3 cat, exon-3 dog, and exon 4 in *Panthera leo*. Red, blue and green lines are different tissues and circles represent the spliced reads in different exons with coverage.

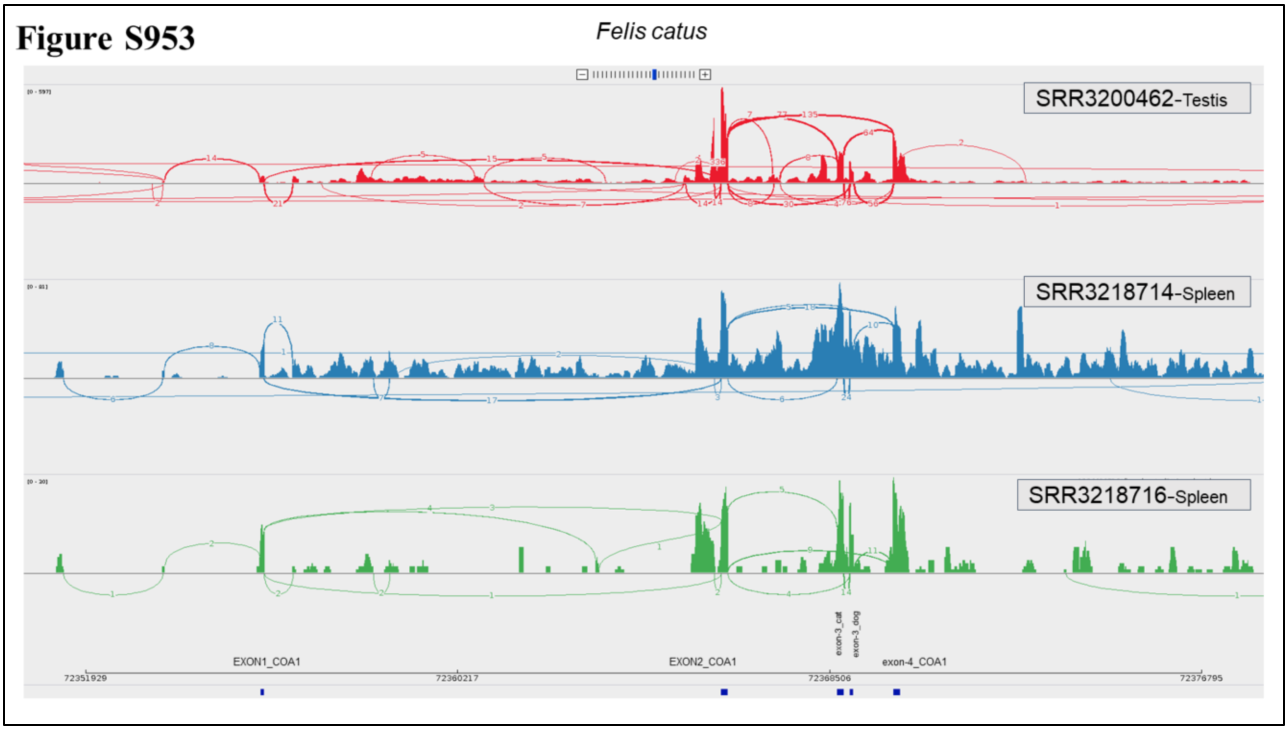

Figure S953: Screenshot of Sashimi plot of *COA1* exon 1, 2, exon-3 cat, exon-3 dog, and exon 4 in cat (*Felis catus*). Red, blue and green lines are different tissues and circles represent the spliced reads in different exons with coverage.

Figure S954

Puma concolor

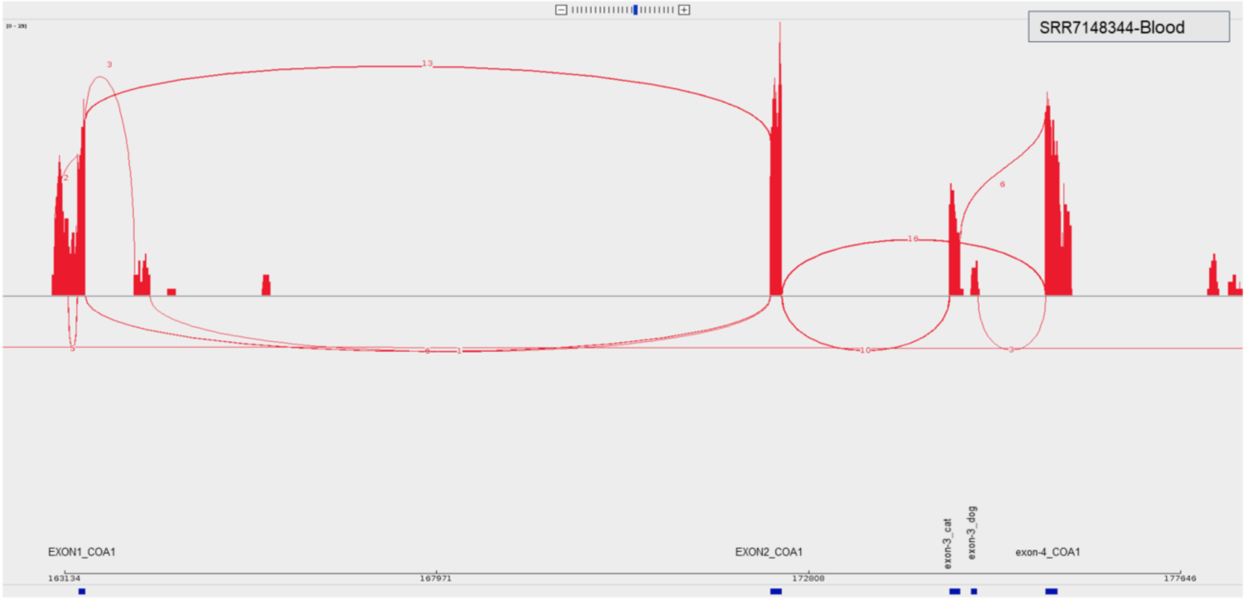

Figure S954: Screenshot of Sashimi plot of *COA1* exon 1, 2, exon-3 cat, exon-3 dog, and exon 4 in *Puma concolor*. Red circles represent the spliced reads in different exons with coverage.

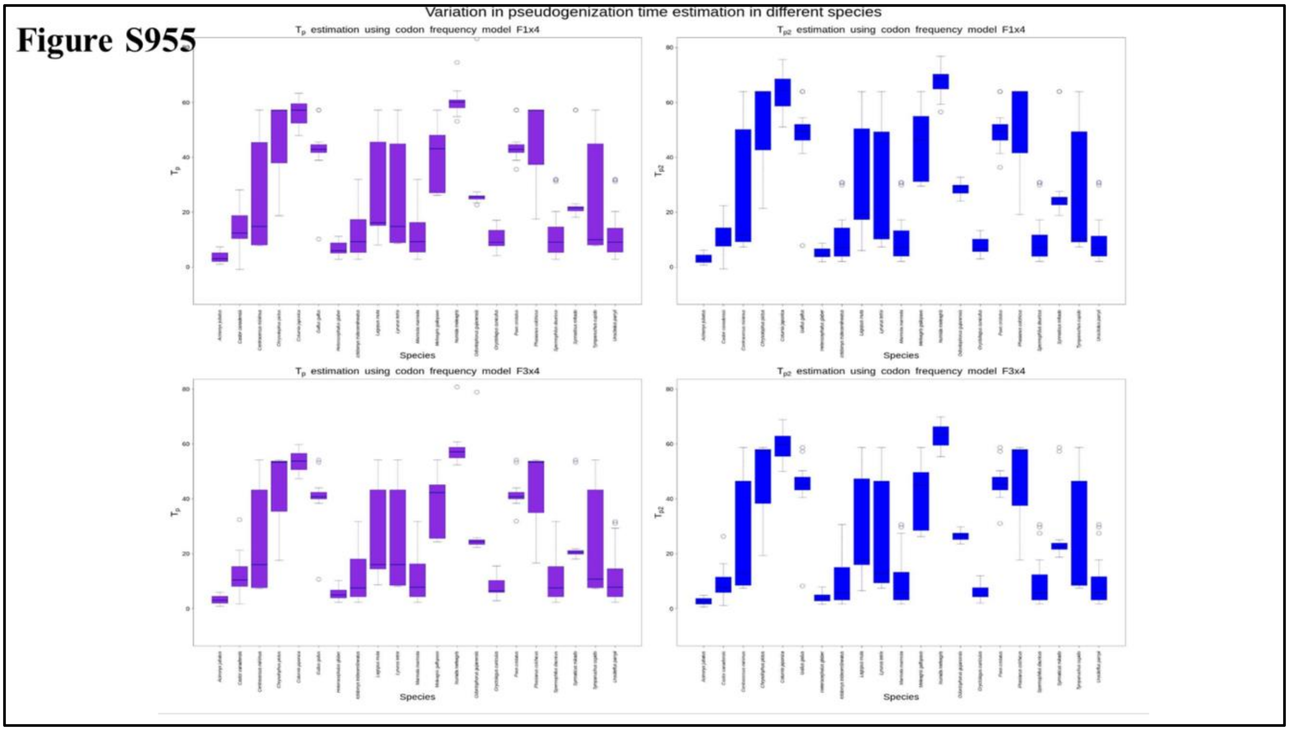

Figure S955: Gene loss timing is estimated using different labellings like pseudogene, mixed and functional. Other than these, we used a combination of background species. All the results were obtained from various combinations plotted in a boxplot. The y-axis represents tp(1ds) tp2 (2ds), and the x-axis contains species names in the boxplot. Based on codon frequency models, different plots are mentioned.

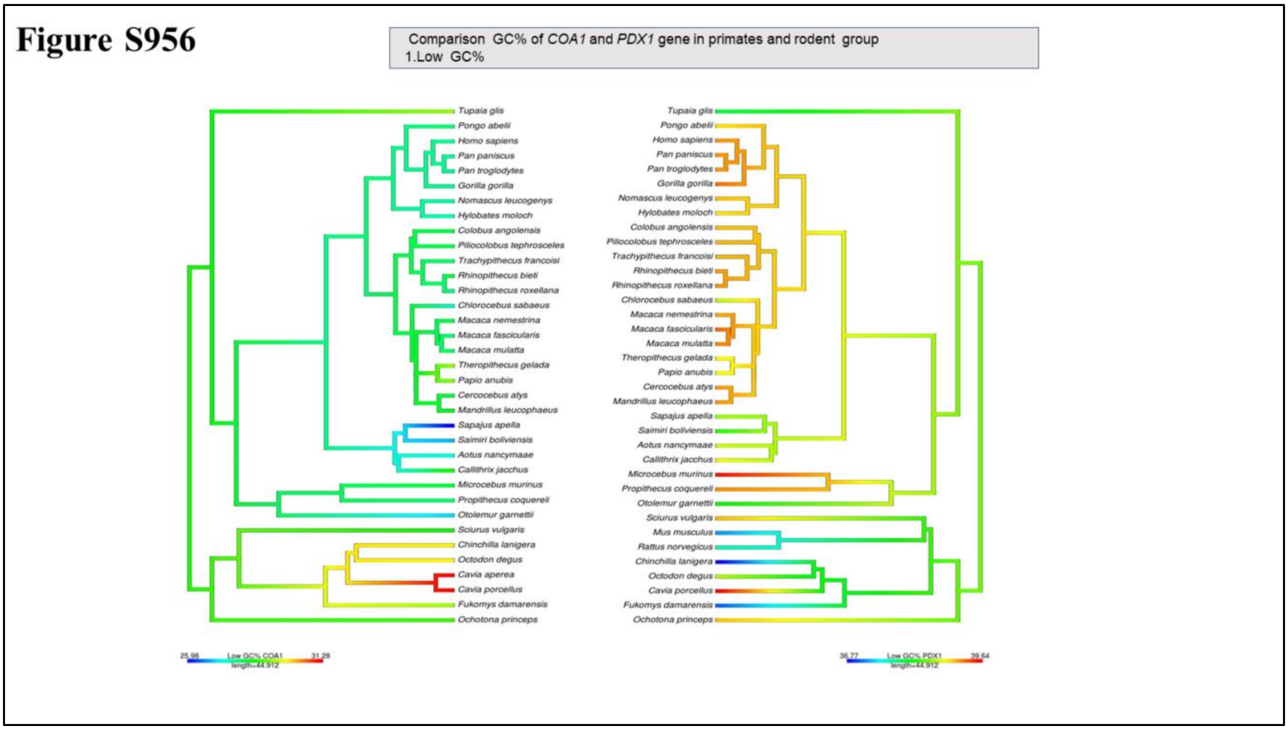

Figure S956: The color represents the GC% of *COA1* and *PDX1* gene using in different species of primates and rodents. The figure showed the possible lowest GC% based synonymous codon using CodSeqGen tools.

Figure S957

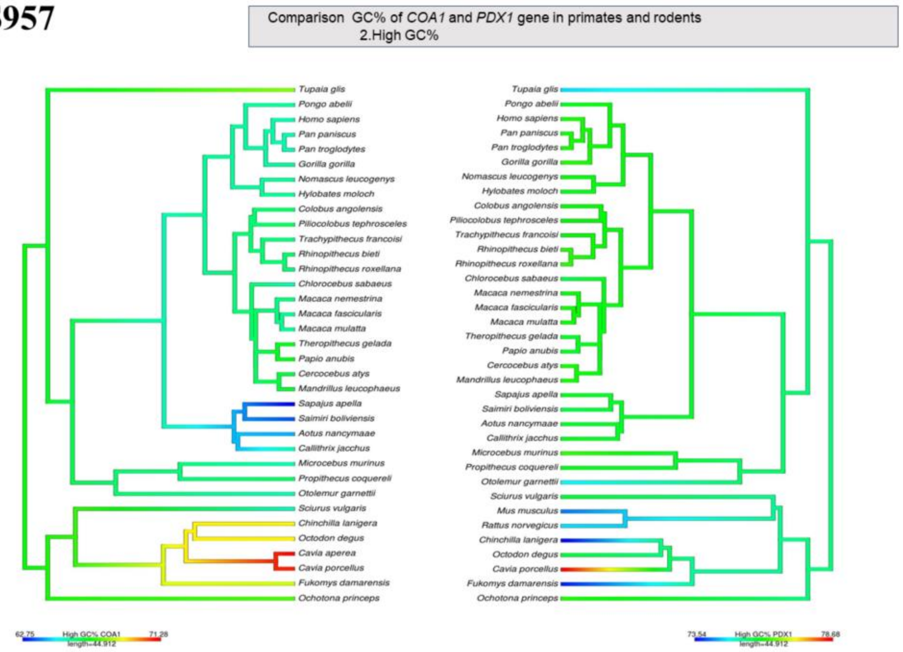

Figure S957: The color represents the GC% of *COA1* and *PDX1* gene using in different species of primates and rodents. The figure showed the possible highest GC% based synonymous codon using CodSeqGen tools.
