# Supplementary material for: Recurrent erosion of COA1/MITRAC15 exemplifies conditional gene dispensability in oxidative phosphorylation
Source: Sci Rep. 2021 Dec 24;11:24437. doi: 10.1038/s41598-021-04077-y (PMC8709867; doi:10.1038/s41598-021-04077-y)
Supplement: Supplementary file 3 — Supplementary Information 3. [file 41598_2021_4077_MOESM3_ESM.zip › Supplementary Dataset/COA1_GENE-main_Github_use_winRAR_to_open.zip!/COA1_GENE/supplementary_figure_data/Pairwise_genome_alignment/Pairwise_alignment_from_ensembl/Mouse_Rat_lastz.odt]

Mouse  mus_musculus: 4:118187691 CCTTCCCTCCCTTCTTTCCTTCCTTCTTCCTCTTTCTTTTTTGTT  mus_musculus: 4:118187735
Rat  rattus_norvegicus: 5:137160555 CCCTCCCTCCC‑‑‑‑‑ACCTCCCTTCTTCCTCTTTT......... rattus_norvegicus: 5:137160585

Mouse  mus_musculus: 4:118187736 GTACTGGCAACTAAATCCGGGGAACTCATGCATGCTGGCTAAGGC  mus_musculus: 4:118187780
Rat 
.............................................  

Mouse  mus_musculus: 4:118187781 TCTACCACCGAGCTAAGAACCAAGGCCTTTTTCAGAGATTTCCAA  mus_musculus: 4:118187825
Rat 
.............................................  

Mouse  mus_musculus: 4:118187826 AATACTTTTGTTACAATGTGACAAAGATCTCTACGAAGTCCTTTG  mus_musculus: 4:118187870
Rat 
.............................................  

Mouse  mus_musculus: 4:118187871 TCGGGGCCTCTTGGAATACTGTTCCTTTATCCCAGTGTATTGGAG  mus_musculus: 4:118187915
Rat 
.............................................  

Mouse  mus_musculus: 4:118187916 GAAGGGTGGGAGAGGTGGAGGATAGGGGATAGGGGTGATGGTGGA  mus_musculus: 4:118187960
Rat 
.............................................  

Mouse  mus_musculus: 4:118187961 GAGTGCTCCTTCTCTTGTTACTTTAAGGAGAGCATAAGTGTGTCA  mus_musculus: 4:118188005
Rat 
.............................................  

Mouse  mus_musculus: 4:118188006 TTCAGCAGACAGACAAACAGCCAACCGTCCTCTCTTCTCACTTTC  mus_musculus: 4:118188050
Rat 
.............................................  

Mouse  mus_musculus: 4:118188051 CTGTAGAAAGTTGAGTCCCTTGAATCTGGAGTGGCCCAGGAGGAC  mus_musculus: 4:118188095
Rat 
.............................................  

Mouse  mus_musculus: 4:118188096 CATTTCCAGGAATGGTTCAGACTGGACTCTAACCCAGCCTCGTCT  mus_musculus: 4:118188140
Rat 
.............................................  

Mouse  mus_musculus: 4:118188141 CCTCTCTGCGGTGAGGACCAGGCAGGACACAGCAGACAGGGCACA  mus_musculus: 4:118188185
Rat 
.............................................  

Mouse  mus_musculus: 4:118188186 TTACGGGGTCAGGATTCAGCCTTCTTGACTGACTGTCTCAGTGCC  mus_musculus: 4:118188230
Rat 
.............................................  

Mouse  mus_musculus: 4:118188231 TTCGAAAGCTGGTGTCTGTCAGCAGAGACCATGGAGCAGTGTGGT  mus_musculus: 4:118188275
Rat 
.............................................  

Mouse  mus_musculus: 4:118188276 CTAGGTTATACAGCCAGATGCCACACCTCAGGGCTAGCTTCTTTA  mus_musculus: 4:118188320
Rat 
.............................................  

Mouse  mus_musculus: 4:118188321 AGCTGTCTCTCCAGGGAAGTCCTTCATCATGTTGGGTCTTAGCTG  mus_musculus: 4:118188365
Rat 
.............................................  

Mouse  mus_musculus: 4:118188366 TCTTCCCTGTGCGGTATGAATCAAGTTCTGCATTCAAGCTATTGC  mus_musculus: 4:118188410
Rat 
.............................................  

Mouse  mus_musculus: 4:118188411 AATACAGAGGATTTCTAGAGATATGTGGAATAGTGAGGCATGTCC  mus_musculus: 4:118188455
Rat 
.............................................  

Mouse  mus_musculus: 4:118188456 CTCACCATAAAAAAAAAAAAAATCCACAGAGTTAGGGCAAGCCAG  mus_musculus: 4:118188500
Rat 
.............................................  

Mouse  mus_musculus: 4:118188501 TTGGCTCAGCCAGCGAAGGTGTTCTGAAGCCTAATGATGAAAGTT  mus_musculus: 4:118188545
Rat 
.............................................  

Mouse  mus_musculus: 4:118188546 CCGATGCTTGGAACTCATGATCGAAGCACTCCTAAAAGCTGCTCT  mus_musculus: 4:118188590
Rat 
.............................................  

Mouse  mus_musculus: 4:118188591 CCACATGCATACCATGGCACATGCCAATTGGAATACACACACACA  mus_musculus: 4:118188635
Rat 
.............................................  

Mouse  mus_musculus: 4:118188636 CACACACACACACACACACACACACACACACACACACACACACTC  mus_musculus: 4:118188680
Rat 
.............................................  

Mouse  mus_musculus: 4:118188681 CACAAGTAGTAAATAAAATAAAAACAAAAACGAAGGAACTCATTT  mus_musculus: 4:118188725
Rat 
.............................................  

Mouse  mus_musculus: 4:118188726 AAAAGTAGCTGCCTAGACCCAGCCTCTCCTTGGCTACCTCTACAG  mus_musculus: 4:118188770
Rat 
.............................................  

Mouse  mus_musculus: 4:118188771 CTCTGTGCCGGAGAAGCAGCATAAAGGTGGCTCTCTGTGGCTTTC  mus_musculus: 4:118188815
Rat  rattus_norvegicus: 5:137160586 
...............................TCGCTGTGGCTTTC rattus_norvegicus: 5:137160599

Mouse  mus_musculus: 4:118188816 CACGCTTGGGCTACACTGTTTCTAC‑‑GACTGGACCTGCCGGAGG  mus_musculus: 4:118188858
Rat  rattus_norvegicus: 5:137160600 CAGGCG‑GGGCTACCCTGTCCCTACCGGATTGGACCTGCAGGAGG rattus_norvegicus: 5:137160643

Mouse  mus_musculus: 4:118188859 GAA‑GCTCTTTCATGCCACAAAACAGTTTATCGTT‑GTTCTGTTT  mus_musculus: 4:118188901
Rat  rattus_norvegicus: 5:137160644 AAAAGCTCTTTCATGCCACAAAACAGTTTATCTTCCGGTCTGTTT rattus_norvegicus: 5:137160688

Mouse  mus_musculus: 4:118188902 CTTGAGCAGCTCGTCTGACCTTA‑CACCTTCAGGGAGAAGCTTTC  mus_musculus: 4:118188945
Rat  rattus_norvegicus: 5:137160689 CTTGAGCAGCTTGTCTGACCTTATCACCTTCAGGGAAAGCTCTCC rattus_norvegicus: 5:137160733

Mouse  mus_musculus: 4:118188946 CCAGGATCCCAATAGCCTTTGGACT‑AGGGAGCGATCTTGGGGTA  mus_musculus: 4:118188989
Rat  rattus_norvegicus: 5:137160734 CAAGGATTCCAATAGCCTTTGGGCCCAAGGAGCAA‑CTTGGGGGA rattus_norvegicus: 5:137160777

Mouse  mus_musculus: 4:118188990 AAAAGCAATTTGGGGTTAAGGAGGTATCTTGCACTGAGAAGCAAC  mus_musculus: 4:118189034
Rat  rattus_norvegicus: 5:137160778 AAAAGCAATCTTGGGT‑AAGAAGGGA‑CTTGGGCAAGAAAG‑‑AC rattus_norvegicus: 5:137160818

Mouse  mus_musculus: 4:118189035 TTTAGTCCGAGAGCTATTCTACTGGAAATGTTAGCTCTGCTCTGT  mus_musculus: 4:118189079
Rat  rattus_norvegicus: 5:137160819 TTGTCGTCGAGA‑CTATTCTACTGGAAATGTTAGCTCTGATCTGT rattus_norvegicus: 5:137160862

Mouse  mus_musculus: 4:118189080 AAACATGACACTCGAGCATTTCCATGCCCTGTTTCTTGG‑CCCAA  mus_musculus: 4:118189123
Rat  rattus_norvegicus: 5:137160863 AAACATGACAC‑CAAGCATTTCCATGCCCTGTTTCTTGGGCCCAA rattus_norvegicus: 5:137160906

Mouse  mus_musculus: 4:118189124 GGGTTTTGTTGTGGTTAATACTGTGGTCTGTCTATTTTATGCTTC  mus_musculus: 4:118189168
Rat  rattus_norvegicus: 5:137160907 GGTTGTTGCTG‑‑GTAATTACTGTG‑‑TCGTCTATTTTATGCT‑C rattus_norvegicus: 5:137160946

Mouse  mus_musculus: 4:118189169 ATCTTCCTGGAAACAGTAAAATGTTTAGCTGTCACACATTCTTGG  mus_musculus: 4:118189213
Rat  rattus_norvegicus: 5:137160947 ATCTCCC‑GGAAACAGTAAAAATGTTAGCTGTCACACATTCTGCT rattus_norvegicus: 5:137160990

Mouse  mus_musculus: 4:118189214 CTCGTGCTTTGCCTTGGGCCAGCTGTGTC‑‑‑‑‑ACTGTGAGCAA  mus_musculus: 4:118189253
Rat  rattus_norvegicus: 5:137160991 CTCA‑GCTTTGCCTTGG‑CCAGCTGTGTCTCACTACNGTGAGCAA rattus_norvegicus: 5:137161033

Mouse  mus_musculus: 4:118189254 CACAATGGAATCTCAGGACTTTAGACCAGAGAGAACCAATACAAT  mus_musculus: 4:118189298
Rat  rattus_norvegicus: 5:137161034 CACAATGGAATCTCGGGACTTTAGACCGGAGAAAGCCAATACAAC rattus_norvegicus: 5:137161078

Mouse  mus_musculus: 4:118189299 CCCTTCACTGAAGGGAAATGAGGCATTGAATGAGGTGAGGAAGAG  mus_musculus: 4:118189343
Rat  rattus_norvegicus: 5:137161079 CCCTTCACTGG‑GGGAAATGAAGCATTGAATGAGGTGAGGAAGAG rattus_norvegicus: 5:137161122

Mouse  mus_musculus: 4:118189344 AGATTGGACCACAGAAATGTCAAAGTGGCCCTTTTCAAGGTCATC  mus_musculus: 4:118189388
Rat  rattus_norvegicus: 5:137161123 AGATTGAACTACAGAAATGTCAAAATGGCCCTTCTCAAGGTCATC rattus_norvegicus: 5:137161167

Mouse  mus_musculus: 4:118189389 TCTGTTCGCCTGTCTCTATAGAATCTGCCTCTCCAGTGTGTAAAA  mus_musculus: 4:118189433
Rat  rattus_norvegicus: 5:137161168 CGAGTTCG‑‑‑‑TCTCTCTAGAATCCGCCTTTCCAGTGTGTAAAA rattus_norvegicus: 5:137161208

Mouse  mus_musculus: 4:118189434 CAAACCTTGGAGTAGGTAGCCACTGGATATCCTTCCATGCACAAG  mus_musculus: 4:118189478
Rat  rattus_norvegicus: 5:137161209 CAAACCTTAGAGTAGATGGCCACTGGATATTCTTCCATGCACACG rattus_norvegicus: 5:137161253

Mouse  mus_musculus: 4:118189479 TTTCACA‑‑‑‑‑‑‑‑‑GCTATTTCTCTGTGTGTAAATCAGCTGTG  mus_musculus: 4:118189514
Rat  rattus_norvegicus: 5:137161254 TTTCACAACTATTTTAGCTATCTCTCTACATGTAAATCCACTGTG rattus_norvegicus: 5:137161298

Mouse  mus_musculus: 4:118189515 
TCATTAAATTCTTTATAGAGAGT‑CCCCCGGTCAAGT  mus_musculus: 4:118189550
Rat  rattus_norvegicus: 5:137161299 
CTATTAAATTCTTTATAGAGTTTGTCCCTGGCCAAGT  rattus_norvegicus: 5:137161335
