# Supplementary material for: Recurrent erosion of COA1/MITRAC15 exemplifies conditional gene dispensability in oxidative phosphorylation
Source: Sci Rep. 2021 Dec 24;11:24437. doi: 10.1038/s41598-021-04077-y (PMC8709867; doi:10.1038/s41598-021-04077-y)
Supplement: Supplementary file 3 — Supplementary Information 3. [file 41598_2021_4077_MOESM3_ESM.zip › Supplementary Dataset/COA1_GENE-main_Github_use_winRAR_to_open.zip!/COA1_GENE/supplementary_figure_data/Pairwise_genome_alignment/Pairwise_alignment_from_ensembl/Mouse_Chinese_hamster_CHOK1GS_lastz.odt]

Mouse  mus_musculus: 4:118187691 CCTTCCCTCCCTTCTTTCCTTCCTTCTTCCTCTTTCTTTTTTGTT  mus_musculus: 4:118187735
Chinese hamster CHOK1GS cricetulus_griseus_chok1gshd: scaffold_2:33925906 CCCTCC‑‑‑‑‑‑‑‑‑‑‑‑‑‑‑‑‑‑‑‑‑‑‑‑‑‑‑‑‑‑‑‑‑TTTGTT cricetulus_griseus_chok1gshd: scaffold_2:33925895

Mouse  mus_musculus: 4:118187736 GTACTGGCAACTAAATCCGGGGAACTCATGCATGCTGGCTAAG‑G  mus_musculus: 4:118187779
Chinese hamster CHOK1GS cricetulus_griseus_chok1gshd: scaffold_2:33925894 GTACCAGCAATTAAACCCAGGGT‑CTCATGCAACCTGGCCAAGTG cricetulus_griseus_chok1gshd: scaffold_2:33925851

Mouse  mus_musculus: 4:118187780 CTCTACCACCGAGCTAAGAACCAAGGCCTTTTTCAGAGATTTCCA  mus_musculus: 4:118187824
Chinese hamster CHOK1GS cricetulus_griseus_chok1gshd: scaffold_2:33925850 CTCTACCACTGAGCTAAGACCCCAGCCCTCTTTCATTGATTCTCA cricetulus_griseus_chok1gshd: scaffold_2:33925806

Mouse  mus_musculus: 4:118187825 AAATACTTTTGTTACAATGTGACAAAGATCTCTACGAAGTCCTTT  mus_musculus: 4:118187869
Chinese hamster CHOK1GS cricetulus_griseus_chok1gshd: scaffold_2:33925805 AGATTGTTTTGCTCTAAAGTGGCAAAGGTCTCTAAGAGGCCATAT cricetulus_griseus_chok1gshd: scaffold_2:33925761

Mouse  mus_musculus: 4:118187870 GTCGGGGCCTCTTGGAATACTGTTCCTTTATCCCAGTGTATTGGA  mus_musculus: 4:118187914
Chinese hamster CHOK1GS cricetulus_griseus_chok1gshd: scaffold_2:33925760 GCCAGGACCTCTGGGAATTCTGGTTCTTTATTCCAGTGTGTTG‑‑ cricetulus_griseus_chok1gshd: scaffold_2:33925718

Mouse  mus_musculus: 4:118187915 GGAAGGGTGGGAGAGGTGGAGGATAGGGGATAGGGGTGATGGTGG  mus_musculus: 4:118187959
Chinese hamster CHOK1GS cricetulus_griseus_chok1gshd: scaffold_2:33925717 ‑‑ATGGCCGGGGGAGG‑‑‑‑‑‑‑‑‑‑‑‑‑‑‑‑‑‑‑‑‑‑‑‑‑‑‑‑‑ cricetulus_griseus_chok1gshd: scaffold_2:33925704

Mouse  mus_musculus: 4:118187960 AGAGTGCTCCTTCTCTTGTTACTTTAAGGAGAGCATAAGTGTGTC  mus_musculus: 4:118188004
Chinese hamster CHOK1GS cricetulus_griseus_chok1gshd: scaffold_2:33925703 ‑GGGTGCTCTTTTTCCTGTTACTGTAAGAAGACC‑‑‑‑‑‑‑‑‑‑‑ cricetulus_griseus_chok1gshd: scaffold_2:33925671

Mouse  mus_musculus: 4:118188005 ATTCAGCAGACAGACAAAC‑‑AGCCAACCGTCCTCTCTTCTCACT  mus_musculus: 4:118188047
Chinese hamster CHOK1GS cricetulus_griseus_chok1gshd: scaffold_2:33925670 ‑‑‑CAGCAGACAGACAAACGGAGCCAATGGTCCTCTCCTCTCACT cricetulus_griseus_chok1gshd: scaffold_2:33925629

Mouse  mus_musculus: 4:118188048 TTCCTGTAGAAAGTTGAGTCCCTTGAATCTGGAGTGGCCCAGGAG  mus_musculus: 4:118188092
Chinese hamster CHOK1GS cricetulus_griseus_chok1gshd: scaffold_2:33925628 TTCCTGTAGAAAACTGGGTCCCTTGAGACTGGAGTGCCCCAAGAA cricetulus_griseus_chok1gshd: scaffold_2:33925584

Mouse  mus_musculus: 4:118188093 GACCATTTCCAGGAATGGTTCAGACTGGACTCTAACCCAGCCTCG  mus_musculus: 4:118188137
Chinese hamster CHOK1GS cricetulus_griseus_chok1gshd: scaffold_2:33925583 GAGCATTTCCAGGAATGTTTCAGAGTTGACTGTAACTTGGCCTCT cricetulus_griseus_chok1gshd: scaffold_2:33925539

Mouse  mus_musculus: 4:118188138 TCTCCTCTCTGCGGTGAGGACCAGGCAGGACACAGCAGACA‑GGG  mus_musculus: 4:118188181
Chinese hamster CHOK1GS cricetulus_griseus_chok1gshd: scaffold_2:33925538 TCTCCCCTCTGCCATGAGAACTGGAAAGGACACAACAGAAACAAG cricetulus_griseus_chok1gshd: scaffold_2:33925494

Mouse  mus_musculus: 4:118188182 CACATTACGGGGT‑‑‑‑‑‑CAGGATTCAGCCTTCTTGACTGACTG  mus_musculus: 4:118188220
Chinese hamster CHOK1GS cricetulus_griseus_chok1gshd: scaffold_2:33925493 AACATTTGGGTGTGTGTGTCAGGATTC‑‑‑‑‑‑‑‑TGTATGAC‑‑ cricetulus_griseus_chok1gshd: scaffold_2:33925459

Mouse  mus_musculus: 4:118188221 TCTCAGTGCCTTCGAAAGCTGGTGTCTGTCAGCAGAGACCATGGA  mus_musculus: 4:118188265
Chinese hamster CHOK1GS cricetulus_griseus_chok1gshd: scaffold_2:33925458 ‑CTCTGTACCTTCGAAAACTGGTGTCTACCAGCAGAGACCATGAA cricetulus_griseus_chok1gshd: scaffold_2:33925415

Mouse  mus_musculus: 4:118188266 G‑CAGTGTGGTCTAGGTTATACAGCCAGATGCC‑‑‑‑‑‑ACACCT  mus_musculus: 4:118188303
Chinese hamster CHOK1GS cricetulus_griseus_chok1gshd: scaffold_2:33925414 GGCAGTGTCATTTAGGCTGTGTAGCCAGATTCCCTCATCACACCT cricetulus_griseus_chok1gshd: scaffold_2:33925370

Mouse  mus_musculus: 4:118188304 CAGGGCTAGCTTCTTTAAGCTGTCTCTCCAGGGAAGTCCTTCATC  mus_musculus: 4:118188348
Chinese hamster CHOK1GS cricetulus_griseus_chok1gshd: scaffold_2:33925369 CAGGTTGAGCTTTTCTAA‑CAATCTCTACAGGGGAGTCCTTCATC cricetulus_griseus_chok1gshd: scaffold_2:33925326

Mouse  mus_musculus: 4:118188349 ATGTTGGGTCTTAGCTGTCTTCCCTGTGCGGTATGA‑‑‑‑‑‑‑‑‑  mus_musculus: 4:118188384
Chinese hamster CHOK1GS cricetulus_griseus_chok1gshd: scaffold_2:33925325 ATGCTGGGTCCTGGCTGCCTTCCCTGTGTGGTAGGAGGTTGTGAG cricetulus_griseus_chok1gshd: scaffold_2:33925281

Mouse  mus_musculus: 4:118188385 ‑ATCAAGTTCTG‑‑CATTCAAGCTATTGCAATACAGAGGATTTCT  mus_musculus: 4:118188426
Chinese hamster CHOK1GS cricetulus_griseus_chok1gshd: scaffold_2:33925280 AATCAAACTGTAGACATTCAAGCTATTGCAATATTGAGGACTTAT cricetulus_griseus_chok1gshd: scaffold_2:33925236

Mouse  mus_musculus: 4:118188427 AGAGATATGTGGAATAGTGAGGCATGT‑‑‑‑CCCTCACCATAAAA  mus_musculus: 4:118188467
Chinese hamster CHOK1GS cricetulus_griseus_chok1gshd: scaffold_2:33925235 AGAGGTATGTGAAATAGTGAGGTACGTACATCCTTTGCCATAAAA cricetulus_griseus_chok1gshd: scaffold_2:33925191

Mouse  mus_musculus: 4:118188468 AAAAAAAAAATCCACAGAGTTAGGGCAAGCCAGTTGGCTCAGCCA  mus_musculus: 4:118188512
Chinese hamster CHOK1GS cricetulus_griseus_chok1gshd: scaffold_2:33925190 AA‑‑‑‑‑‑‑‑‑‑CACCCAGTTAGGGAAAGCCAGATGGTTCAGCCA cricetulus_griseus_chok1gshd: scaffold_2:33925156

Mouse  mus_musculus: 4:118188513 GCGAAGGTGTTCTGAAGCCT‑‑‑‑‑‑‑‑AATGATGAAAGTTCCGA  mus_musculus: 4:118188549
Chinese hamster CHOK1GS cricetulus_griseus_chok1gshd: scaffold_2:33925155 GCAAAGGTGCTCTGAAGCCCTGAAGCCCATTGACTAAAGTTCAGA cricetulus_griseus_chok1gshd: scaffold_2:33925111

Mouse  mus_musculus: 4:118188550 TGCTTGGAACTCATGATCGAAGCACTCCTAAAAGCTGCTCTCCAC  mus_musculus: 4:118188594
Chinese hamster CHOK1GS cricetulus_griseus_chok1gshd: scaffold_2:33925110 TGCCTGGGACTTGTGATGTAAGAATCCCAAAA‑‑‑‑‑‑‑‑‑‑‑‑‑ cricetulus_griseus_chok1gshd: scaffold_2:33925079

Mouse  mus_musculus: 4:118188595 ATGCATACCATGGCACATGCCAATTGGAATACACACACACACACA  mus_musculus: 4:118188639
Chinese hamster CHOK1GS cricetulus_griseus_chok1gshd: scaffold_2:33925078 ‑‑‑‑‑‑‑‑‑‑‑‑GCACATGCCTGCGGGGGTGCTCTCTCTCGCTCT cricetulus_griseus_chok1gshd: scaffold_2:33925046

Mouse  mus_musculus: 4:118188640 CACACACACACAC‑‑‑‑‑‑‑‑‑‑‑‑‑‑‑‑‑‑‑‑‑‑‑‑‑‑‑‑‑‑‑‑  mus_musculus: 4:118188652
Chinese hamster CHOK1GS cricetulus_griseus_chok1gshd: scaffold_2:33925045 CTCTCTCTCTCTCTCTCTCTCTCTCTCTCTCTCTCTCTCTCTCTC cricetulus_griseus_chok1gshd: scaffold_2:33925001

Mouse  mus_musculus: 4:118188653 ‑‑‑‑‑‑‑‑ACACACACACACACACACACACACACTCCACAAGTAG  mus_musculus: 4:118188689
Chinese hamster CHOK1GS cricetulus_griseus_chok1gshd: scaffold_2:33925000 TCTCTCTCACACACACACACACACACACACACACAAAACAAATA‑ cricetulus_griseus_chok1gshd: scaffold_2:33924957

Mouse  mus_musculus: 4:118188690 TAAATAAAA‑‑‑‑‑‑TAAAAACAAAAACGAAGGAACTCATTTAAA  mus_musculus: 4:118188728
Chinese hamster CHOK1GS cricetulus_griseus_chok1gshd: scaffold_2:33924956 ‑AAATAAAACAAAAATAAAAACAAAGACAAAGGAACTCATCT‑‑‑ cricetulus_griseus_chok1gshd: scaffold_2:33924916

Mouse  mus_musculus: 4:118188729 AGTAGCTGCCTAGACCCAGCCTCTCCTTGGCTACCTCTACAGCTC  mus_musculus: 4:118188773
Chinese hamster CHOK1GS cricetulus_griseus_chok1gshd: scaffold_2:33924915 ‑GTAGCGGTCTAGCCCCTGCCTTTCCTTGGCCACATCTACAGCTC cricetulus_griseus_chok1gshd: scaffold_2:33924872

Mouse  mus_musculus: 4:118188774 TGTGCCGGAGAAGCAGCATAAAGGTGGCTCTCTGTGGCTTTCCAC  mus_musculus: 4:118188818
Chinese hamster CHOK1GS cricetulus_griseus_chok1gshd: scaffold_2:33924871 TATGGACCAGAAGCAGCATAAGGGTGGCTCT‑‑GTGGCTCTCCAG cricetulus_griseus_chok1gshd: scaffold_2:33924829

Mouse  mus_musculus: 4:118188819 GCTTGGGCTACACTGTTTCTACGACTGGACCTGCCGGAGGGAAGC  mus_musculus: 4:118188863
Chinese hamster CHOK1GS cricetulus_griseus_chok1gshd: scaffold_2:33924828 TCTTGGGTCATCTTGTTCCTACCATTGGACGTGCAGGACTGAAGT cricetulus_griseus_chok1gshd: scaffold_2:33924784

Mouse  mus_musculus: 4:118188864 TCTTTCATGCCACAAAACA‑‑GTTTATCGTTGTTCTGTTTCTTGA  mus_musculus: 4:118188906
Chinese hamster CHOK1GS cricetulus_griseus_chok1gshd: scaffold_2:33924783 TCTTTTGTGCTATCAGACACAGTTTGTCTTTGCTCTGCTTCTGGA cricetulus_griseus_chok1gshd: scaffold_2:33924739

Mouse  mus_musculus: 4:118188907 GCAGCTCGTCTGACCTTACACCTTCAGGGAGAAGCTTTCCCAGGA  mus_musculus: 4:118188951
Chinese hamster CHOK1GS cricetulus_griseus_chok1gshd: scaffold_2:33924738 GCAGCTTGTCAGTCCCTGCACCTTCAGGGAGAAGCTTTCCCAGGA cricetulus_griseus_chok1gshd: scaffold_2:33924694

Mouse  mus_musculus: 4:118188952 TCCCAATAGCCTTTGGACTAGGGAGC‑‑‑‑GATCTTGGGGTAAAA  mus_musculus: 4:118188992
Chinese hamster CHOK1GS cricetulus_griseus_chok1gshd: scaffold_2:33924693 TTCCAGTAGCCTTTAGGCTAAGAAGCAAGCGATCTCGGGGTAAAC cricetulus_griseus_chok1gshd: scaffold_2:33924649

Mouse  mus_musculus: 4:118188993 AGCAATTTGGGGTTAAGGAGGTATCTTGCACTGAGAAGCAACTTT  mus_musculus: 4:118189037
Chinese hamster CHOK1GS cricetulus_griseus_chok1gshd: scaffold_2:33924648 AACAATCTTGGGCTAAGAAGG‑‑‑‑‑‑‑‑‑‑‑‑‑‑‑‑‑‑‑ATCTT cricetulus_griseus_chok1gshd: scaffold_2:33924623

Mouse  mus_musculus: 4:118189038 AGTCCGAGAGCTATTCTACTGGAAATGTTAGCTCTGCTCTGTAAA  mus_musculus: 4:118189082
Chinese hamster CHOK1GS cricetulus_griseus_chok1gshd: scaffold_2:33924622 GGGCTGAGAGCTGTTCTACTGGAAATGTTAGCTCTGATCTGTAAA cricetulus_griseus_chok1gshd: scaffold_2:33924578

Mouse  mus_musculus: 4:118189083 CATGACACTCGAGCATTTCCATGCCCTGTTTCTTGGCCCAAGGGT  mus_musculus: 4:118189127
Chinese hamster CHOK1GS cricetulus_griseus_chok1gshd: scaffold_2:33924577 CATGACATGTGAGCATTTCCATGCCCTGTTTCTTGGCCCAAGGGT cricetulus_griseus_chok1gshd: scaffold_2:33924533

Mouse  mus_musculus: 4:118189128 TTTGTTGTGGTTAATACTGTGGTCTGTCTATTTTATGCTTCATCT  mus_musculus: 4:118189172
Chinese hamster CHOK1GS cricetulus_griseus_chok1gshd: scaffold_2:33924532 TTTGTTGTGGTTAATACTGTGGTCTGTCTATTTTATGCTTCATCT cricetulus_griseus_chok1gshd: scaffold_2:33924488

Mouse  mus_musculus: 4:118189173 TCCTGGAAACAGTAAAATGTTTAGCTGTCACACATTCTTGGCTCG  mus_musculus: 4:118189217
Chinese hamster CHOK1GS cricetulus_griseus_chok1gshd: scaffold_2:33924487 TCTCGGAAACAGTAAAATGTTTAGCTGTCACACATTCCTGGCTCT cricetulus_griseus_chok1gshd: scaffold_2:33924443

Mouse  mus_musculus: 4:118189218 TGCTTTGCCTTGGGCCAGCTGTGTCACTGTGAGCAACACAATGGA  mus_musculus: 4:118189262
Chinese hamster CHOK1GS cricetulus_griseus_chok1gshd: scaffold_2:33924442 CGCTTTGCCTTGGGCCAGCTGTGTCACTTTGAGCAACACAATGGA cricetulus_griseus_chok1gshd: scaffold_2:33924398

Mouse  mus_musculus: 4:118189263 ATCTCAGGACTTTAGACCAGAGAGAACCAATACAATCCCTTCACT  mus_musculus: 4:118189307
Chinese hamster CHOK1GS cricetulus_griseus_chok1gshd: scaffold_2:33924397 ATCATAGGACTTTAGACCAGAGAGAGCTGATCCGACCCCTTCACT cricetulus_griseus_chok1gshd: scaffold_2:33924353

Mouse  mus_musculus: 4:118189308 GAAGGGAAATGAGGCATTGAATGAGGTGAGG‑‑AAGAGAGATTGG  mus_musculus: 4:118189350
Chinese hamster CHOK1GS cricetulus_griseus_chok1gshd: scaffold_2:33924352 GAAGGGAAATGAGGCAGTGAATGAGGTGAGGAAAAGAGAGACTGA cricetulus_griseus_chok1gshd: scaffold_2:33924308

Mouse  mus_musculus: 4:118189351 ACCACAGAAATGTCAAAGTGGCCCTTTTCAAGGTCATCTCTGTTC  mus_musculus: 4:118189395
Chinese hamster CHOK1GS cricetulus_griseus_chok1gshd: scaffold_2:33924307 ACCACACAGATGTCAAGATGGCCCTTTCCAAGGTCATCTGTGT‑‑ cricetulus_griseus_chok1gshd: scaffold_2:33924265

Mouse  mus_musculus: 4:118189396 GCCTGTCTCTATAGAATCTGCCTCTCCAGTGTGTAAAACAAACCT  mus_musculus: 4:118189440
Chinese hamster CHOK1GS cricetulus_griseus_chok1gshd: scaffold_2:33924264 ‑‑CCATCTCTGCAGAG‑CTGCCTTTCCGGTGTGTAAAACAAGCCT cricetulus_griseus_chok1gshd: scaffold_2:33924223

Mouse  mus_musculus: 4:118189441 TGGAGTAGGTAGCCACTGGATATCCTTC‑‑CATGCACAAGTTTCA  mus_musculus: 4:118189483
Chinese hamster CHOK1GS cricetulus_griseus_chok1gshd: scaffold_2:33924222 TAGAGT‑GGAGGCTGCTGGTTATTCTCCCACATGTGTTTGTTTCA cricetulus_griseus_chok1gshd: scaffold_2:33924179

Mouse  mus_musculus: 4:118189484 CAGCTATTTCTCTGTGTGTAAATCAGCTGTGTCATTAAATTCTTT  mus_musculus: 4:118189528
Chinese hamster CHOK1GS cricetulus_griseus_chok1gshd: scaffold_2:33924178 CAGCTCTTT‑TCTACACGTACGTCAACCGTGTTATTTAACTCTTT cricetulus_griseus_chok1gshd: scaffold_2:33924135

Mouse  mus_musculus: 4:118189529 ATAGAGA‑GTCCCCCGGTCAAGT  mus_musculus: 4:118189550
Chinese hamster CHOK1GS cricetulus_griseus_chok1gshd: scaffold_2:33924134 ATAGAGATTTCCCCTGGGCAAGT  cricetulus_griseus_chok1gshd: scaffold_2:33924112
