# Supplementary material for: Recurrent erosion of COA1/MITRAC15 exemplifies conditional gene dispensability in oxidative phosphorylation
Source: Sci Rep. 2021 Dec 24;11:24437. doi: 10.1038/s41598-021-04077-y (PMC8709867; doi:10.1038/s41598-021-04077-y)
Supplement: Supplementary file 3 — Supplementary Information 3. [file 41598_2021_4077_MOESM3_ESM.zip › Supplementary Dataset/COA1_GENE-main_Github_use_winRAR_to_open.zip!/COA1_GENE/supplementary_figure_data/Pairwise_genome_alignment/Pairwise_alignment_from_ensembl/Mouse_Golden_Hamster_lastz.odt]

Mouse  mus_musculus: 4:118187691 CCTTCCCTCCCTTCTTTCCTTCCTTCTTCCTCTTTCTTTTTTGTT  mus_musculus: 4:118187735
Golden Hamster mesocricetus_auratus: KB708283.1:2948456 ‑‑‑‑‑‑‑‑‑‑‑‑‑‑‑‑‑‑‑‑‑‑‑‑‑‑‑‑‑‑TCTTTCTTCTTTGTT mesocricetus_auratus: KB708283.1:2948442

Mouse  mus_musculus: 4:118187736 GTACTGGCAACTAAATCCGGGGAACTCATGCATGCTGGCTAAG‑G  mus_musculus: 4:118187779
Golden Hamster mesocricetus_auratus: KB708283.1:2948441 ‑TACCAGCAGTGAAACCCAGGGT‑CTCATGCATGCTGGCCAAGTG mesocricetus_auratus: KB708283.1:2948399

Mouse  mus_musculus: 4:118187780 CTCTACCACCGAGCTAAGAACCAAGGCCTTTTTCAGAGATTTCCA  mus_musculus: 4:118187824
Golden Hamster mesocricetus_auratus: KB708283.1:2948398 CTCTACCACTGAGCTAGGACCCCAGCCCTCTTTCATTGATTCTCA mesocricetus_auratus: KB708283.1:2948354

Mouse  mus_musculus: 4:118187825 AAATACTTTTGTTACAATGTGAC‑‑AAAGATCTCTACGAAGTCCT  mus_musculus: 4:118187867
Golden Hamster mesocricetus_auratus: KB708283.1:2948353 AGATTGTTTTGCTATAAAGTGGTTAAAAAGTCTCTAAGAAGCCAT mesocricetus_auratus: KB708283.1:2948309

Mouse  mus_musculus: 4:118187868 TTGTCGGGGCCTCTTGGAATACTGTTCCTTTATCCCAGTGTATTG  mus_musculus: 4:118187912
Golden Hamster mesocricetus_auratus: KB708283.1:2948308 ATGCCAGGGCCTCTGGGAATACTGATGCTTTATTCCAGTGTGCTG mesocricetus_auratus: KB708283.1:2948264

Mouse  mus_musculus: 4:118187913 ‑‑‑GAGGAAGGGTGGGAGA‑‑‑‑GGTGGAGGATAGGGGATAGGG‑  mus_musculus: 4:118187949
Golden Hamster mesocricetus_auratus: KB708283.1:2948263 ATGGGGGTGGGGTGGGGGATGGGAGTGGGGGATGGGGGGTGGGGA mesocricetus_auratus: KB708283.1:2948219

Mouse  mus_musculus: 4:118187950 ‑‑‑‑GTGATGGTGGAGAGTG‑‑‑‑‑‑CTCCTTCTCTTGTTACTTT  mus_musculus: 4:118187984
Golden Hamster mesocricetus_auratus: KB708283.1:2948218 GGTGGAGGGGGTGGAGGGTGTTAATACTCTTTTTCCTGTTACTGT mesocricetus_auratus: KB708283.1:2948174

Mouse  mus_musculus: 4:118187985 AAGGAGAGCATAAGTGTGTCATTCAGCAGACAGACAAACAGCCAA  mus_musculus: 4:118188029
Golden Hamster mesocricetus_auratus: KB708283.1:2948173 CAGAAGAGC‑‑‑‑‑‑‑‑‑‑‑‑‑‑CAACAGACAGACGGA‑‑GCCAA mesocricetus_auratus: KB708283.1:2948145

Mouse  mus_musculus: 4:118188030 CCGTCCTCTCTTCTCACTTTCCTGTAGAAAGTTGAGTCCCTTGAA  mus_musculus: 4:118188074
Golden Hamster mesocricetus_auratus: KB708283.1:2948144 TGGTCCACTCCTCTTACT‑‑‑‑‑GTAGAAAACTGGGTCCCTTGGG mesocricetus_auratus: KB708283.1:2948105

Mouse  mus_musculus: 4:118188075 TCTGGAGTGGCCCAGGAGGACCATTTCCAGGAATGGTTCAGACTG  mus_musculus: 4:118188119
Golden Hamster mesocricetus_auratus: KB708283.1:2948104 TCTGGAGTGGCCCAAGAAGAGCATTTCCAGGAATGTTTCAGACTT mesocricetus_auratus: KB708283.1:2948060

Mouse  mus_musculus: 4:118188120 GACTCTAACCCAGCCTCGTCTCCTCTCTGCGGTGAGGACCAGGCA  mus_musculus: 4:118188164
Golden Hamster mesocricetus_auratus: KB708283.1:2948059 GATTATAATTCAGCCTCTTCTCCCCTCTGCTGTGAGGACCATGAA mesocricetus_auratus: KB708283.1:2948015

Mouse  mus_musculus: 4:118188165 GGACACAGCAGACA‑GGGCACATTACGGG‑‑‑‑‑‑‑‑‑‑‑‑‑‑‑‑  mus_musculus: 4:118188192
Golden Hamster mesocricetus_auratus: KB708283.1:2948014 GGATACAACAGAAACAGGAACAT‑‑‑GGGCTGTGTGTGTGTGTGT mesocricetus_auratus: KB708283.1:2947973

Mouse  mus_musculus: 4:118188193 ‑‑‑‑‑‑‑‑‑‑‑‑‑‑‑‑‑‑‑‑‑‑‑‑‑‑GTCAGGATTCAGCCTTCTT  mus_musculus: 4:118188211
Golden Hamster mesocricetus_auratus: KB708283.1:2947972 GTGTGTGTGTGTGTGTGTGTGTGTGTGTGTGTGTTCAGCCTTCTT mesocricetus_auratus: KB708283.1:2947928

Mouse  mus_musculus: 4:118188212 GACTGACTGTCTCAGTGCCTTCGAAAGCTGGTGTCTGTCAGCAGA  mus_musculus: 4:118188256
Golden Hamster mesocricetus_auratus: KB708283.1:2947927 GACTGATGACCTCTGCGCCTTAGAAAACTAGTGTCTACCAGCAGA mesocricetus_auratus: KB708283.1:2947883

Mouse  mus_musculus: 4:118188257 GACCATGGAG‑CAGTGTGGTCTAGGTTATACAGCCAGATGCC‑‑‑  mus_musculus: 4:118188297
Golden Hamster mesocricetus_auratus: KB708283.1:2947882 AGCCATCGAGGCAGCGTCATTTAGGCTGTGTAGCCAAATGCCCTC mesocricetus_auratus: KB708283.1:2947838

Mouse  mus_musculus: 4:118188298 ‑‑‑ACACCTCAGGGCTAGCTTCTTTAAGCTGTCTCTCCAGGGAAG  mus_musculus: 4:118188339
Golden Hamster mesocricetus_auratus: KB708283.1:2947837 ATCATACCTCAGGTCGAGTTTTCT‑‑AACTGTCTCTATAGGGGAG mesocricetus_auratus: KB708283.1:2947795

Mouse  mus_musculus: 4:118188340 TCCTTCATCATGTTGGGTCTTAGCTGTCTTCCCTGTGCGGTATGA  mus_musculus: 4:118188384
Golden Hamster mesocricetus_auratus: KB708283.1:2947794 TCCTTCATCCTTCTGGGTCCTGGATGTCTTCCCTGTATGGTGGGA mesocricetus_auratus: KB708283.1:2947750

Mouse  mus_musculus: 4:118188385 A‑‑‑‑‑‑‑‑‑‑TCAAGTTCTG‑‑‑CATTCAAGCTATTGCAATACA  mus_musculus: 4:118188416
Golden Hamster mesocricetus_auratus: KB708283.1:2947749 AGTTGTAAGGATCAAGCTGTAGGACATTCAAGCTATTGCAATATT mesocricetus_auratus: KB708283.1:2947705

Mouse  mus_musculus: 4:118188417 GAGGATTTCTAGAGATATGTGGAATAGTGAGGCATGTCCCTCACC  mus_musculus: 4:118188461
Golden Hamster mesocricetus_auratus: KB708283.1:2947704 GAGGATTTCTAGAGGTACGTGAAACAGTGAGGCACATCCCTTGCC mesocricetus_auratus: KB708283.1:2947660

Mouse  mus_musculus: 4:118188462 ATAAAAAAAAAAAAAATCCACAGAGTTAGGGCAAGCCAGTTGGCT  mus_musculus: 4:118188506
Golden Hamster mesocricetus_auratus: KB708283.1:2947659 ATTAAAAA‑‑‑‑‑‑‑‑‑‑CACAGGGTTAGGGCAAGCCAGACGGGT mesocricetus_auratus: KB708283.1:2947625

Mouse  mus_musculus: 4:118188507 CAGCCAGCGAAGGTGTTCTGAAGCCTAATGATGAAAGTTCCGATG  mus_musculus: 4:118188551
Golden Hamster mesocricetus_auratus: KB708283.1:2947624 CAGCCAGCAAAGGTGCTCTGAAGC‑TATTGACCAAAGTTCTGATG mesocricetus_auratus: KB708283.1:2947581

Mouse  mus_musculus: 4:118188552 CTTGGAACTCATGATCGAAGCACTCCTAAAAGCTGCTCTCCACAT  mus_musculus: 4:118188596
Golden Hamster mesocricetus_auratus: KB708283.1:2947580 ‑‑‑‑‑‑‑‑‑‑‑‑‑AAAGAAGAATTCCTGAAATCTGTCCTC..... mesocricetus_auratus: KB708283.1:2947554

Mouse  mus_musculus: 4:118188597 GCATACCATGGCACATGCCAATTGGAATACACACACACACACACA  mus_musculus: 4:118188641
Golden Hamster mesocricetus_auratus: KB708283.1:2947444 .............................CTCTCACACACAGACA mesocricetus_auratus: KB708283.1:2947429

Mouse  mus_musculus: 4:118188642 CACACACACACACACACACACACACACACACACACACTCCACAAG  mus_musculus: 4:118188686
Golden Hamster mesocricetus_auratus: KB708283.1:2947428 CACACACAAATA............................ATAAA mesocricetus_auratus: KB708283.1:2947254

Mouse  mus_musculus: 4:118188687 TAG‑TAAATAAAATAAAAACAAAAACGAAGGAACTCATTTAAAAG  mus_musculus: 4:118188730
Golden Hamster mesocricetus_auratus: KB708283.1:2947253 TAAATAAATAAAATAAAAGCAAAGATAAAGGAACTCATCT‑‑‑‑G mesocricetus_auratus: KB708283.1:2947213

Mouse  mus_musculus: 4:118188731 TAGCTGCCTAGACCCAGCCTCTCCTTGGCTACCTCTACAGCTCTG  mus_musculus: 4:118188775
Golden Hamster mesocricetus_auratus: KB708283.1:2947212 TAGCTGTATAGACCCTGCCTTTCCTTGACCACATCTGCAGCGCTA mesocricetus_auratus: KB708283.1:2947168

Mouse  mus_musculus: 4:118188776 TGCCGGAGAAGCAGCATAAAGGTGGCTCTCTGTGGCTTTCCACGC  mus_musculus: 4:118188820
Golden Hamster mesocricetus_auratus: KB708283.1:2947167 TGGACTAGAAGCAGCATACGGGTGGTTCA‑‑GTGGCTCTCTGGTC mesocricetus_auratus: KB708283.1:2947125

Mouse  mus_musculus: 4:118188821 TTGGGCTACACTGTTTCTACGACTGGACCTGCCGGAGGGAAGCTC  mus_musculus: 4:118188865
Golden Hamster mesocricetus_auratus: KB708283.1:2947124 TTGGGTCATCTTGTTCCTACTATTGGACCTGCAGGACGGAAGCCC mesocricetus_auratus: KB708283.1:2947080

Mouse  mus_musculus: 4:118188866 TTTCATGCCACAAAACAGTTTATCGTTGTTCTGTTTCTTGAGCAG  mus_musculus: 4:118188910
Golden Hamster mesocricetus_auratus: KB708283.1:2947079 CTGTGTGCTATCAGACAGTTTATCTTTGCT‑‑‑‑‑‑‑‑‑‑‑‑‑‑‑ mesocricetus_auratus: KB708283.1:2947050

Mouse  mus_musculus: 4:118188911 CTCGTCTGACCTTACACCTTCAGGGAGAAGCTTTCCCAGGATCCC  mus_musculus: 4:118188955
Golden Hamster mesocricetus_auratus: KB708283.1:2947049 ‑‑‑GTCAGTCCTTACACCTTCGGGGAGAAGCTTTTCCAGGATTCC mesocricetus_auratus: KB708283.1:2947008

Mouse  mus_musculus: 4:118188956 AATAGCCTTTGGACTAGGGAGCGA‑‑‑‑TCTTGGGGTAAAAAGCA  mus_musculus: 4:118188996
Golden Hamster mesocricetus_auratus: KB708283.1:2947007 AGTAGCCTTTAGGCTAAGAAGCAAGCCATCTTGGGGTAAAAAACA mesocricetus_auratus: KB708283.1:2946963

Mouse  mus_musculus: 4:118188997 ATTTGGGGTTAAGGAGGTATCTTGCACTGAGAAGCAACTTTAGTC  mus_musculus: 4:118189041
Golden Hamster mesocricetus_auratus: KB708283.1:2946962 ATCTTGGGCTAAGAAGG‑‑‑‑‑‑‑‑‑‑‑‑‑‑‑‑‑‑‑ATCTTGGGC mesocricetus_auratus: KB708283.1:2946937

Mouse  mus_musculus: 4:118189042 CGAGAGCTATTCTACTGGAAATGTTAGCTCTGCTCTGTAAACATG  mus_musculus: 4:118189086
Golden Hamster mesocricetus_auratus: KB708283.1:2946936 TGAGAGCTGTTCTACTGGAAATGTTAGCTCTGATCTGTAAACGTG mesocricetus_auratus: KB708283.1:2946892

Mouse  mus_musculus: 4:118189087 ACACTCGAGCATTTCCATGCCCTGTTTCTTGGCCCAAGGGTTTTG  mus_musculus: 4:118189131
Golden Hamster mesocricetus_auratus: KB708283.1:2946891 ACATGCGAGCATTTCCATGTCTTGTTTCTTGGCCCAAGGGTTTTG mesocricetus_auratus: KB708283.1:2946847

Mouse  mus_musculus: 4:118189132 TTGTGGTTAATACTGTGGTCTGTCTATTTTATGCTTCATCTTCCT  mus_musculus: 4:118189176
Golden Hamster mesocricetus_auratus: KB708283.1:2946846 TTGTGGTTAATACTGTGGTCTGTCTATTTTATGCTTCATCTTCTC mesocricetus_auratus: KB708283.1:2946802

Mouse  mus_musculus: 4:118189177 GGAAACAGTAAAATGTTTAGCTGTCACACATTCTTGGCTCGTGCT  mus_musculus: 4:118189221
Golden Hamster mesocricetus_auratus: KB708283.1:2946801 GGAACCAGTAAAATGTTTAGCTGTCACACATTCCTGGCTCTCGCT mesocricetus_auratus: KB708283.1:2946757

Mouse  mus_musculus: 4:118189222 TTGCCTTGGGCCAGCTGTGTCACTGTGAGCAACACAATGGAATCT  mus_musculus: 4:118189266
Golden Hamster mesocricetus_auratus: KB708283.1:2946756 TTGCCTGGGGCCAGCTGTGTCACTTTGAGCAACACAATGGAATCA mesocricetus_auratus: KB708283.1:2946712

Mouse  mus_musculus: 4:118189267 CAGGACTTTAGACCAGAGAGAACCAATACAATCCCTTCACTGAAG  mus_musculus: 4:118189311
Golden Hamster mesocricetus_auratus: KB708283.1:2946711 TGGGACTTTAGACCAGAGAGAGCTGATCCGACCCCTTCACTGAAG mesocricetus_auratus: KB708283.1:2946667

Mouse  mus_musculus: 4:118189312 GGAAATGAGGCATTGAATGAGGTGAGG‑‑AAGAGAGATTGGACCA  mus_musculus: 4:118189354
Golden Hamster mesocricetus_auratus: KB708283.1:2946666 GGAAATGAGGCAGTGAAAGAGGTGAGGAAAAGAGAGATTGAACCA mesocricetus_auratus: KB708283.1:2946622

Mouse  mus_musculus: 4:118189355 CAGAAATGTCAAAGTGGCCCTTTTCAAGGTCATCTCTGTTCGCCT  mus_musculus: 4:118189399
Golden Hamster mesocricetus_auratus: KB708283.1:2946621 CACAAATGTCAAGATGGCCCTTTCCAAGGTCATCTGTGTTC‑‑‑‑ mesocricetus_auratus: KB708283.1:2946581

Mouse  mus_musculus: 4:118189400 GTCTCTATAGAATCTGCCTCTCCAGTGTGTAAAACAAACCTTGGA  mus_musculus: 4:118189444
Golden Hamster mesocricetus_auratus: KB708283.1:2946580 ATCTCTGCAGAGTCTGCCTTTCCAGTGTGTAAAGGAAGCCTTAGA mesocricetus_auratus: KB708283.1:2946536

Mouse  mus_musculus: 4:118189445 GTAGGTAGCCACTGGATATCCTTCCATGCACAAGTTTCACAGCTA  mus_musculus: 4:118189489
Golden Hamster mesocricetus_auratus: KB708283.1:2946535 GTAGAGGTCCACAGGTTACTCTTAGACACACATG‑TTCACAACTC mesocricetus_auratus: KB708283.1:2946492

Mouse  mus_musculus: 4:118189490 TTTCTCTGTGTGTAAATCAGCTGTGTCATTAAATTCTTTATAGAG  mus_musculus: 4:118189534
Golden Hamster mesocricetus_auratus: KB708283.1:2946491 TTTTTCTACATGTAAGTCAACTGTGTTATTTAACTCTTTATAGAG mesocricetus_auratus: KB708283.1:2946447

Mouse  mus_musculus: 4:118189535 A‑GTCCCCCGGTCAAGT  mus_musculus: 4:118189550
Golden Hamster mesocricetus_auratus: KB708283.1:2946446 ATTTCCCCTGGGCAAGC  mesocricetus_auratus: KB708283.1:2946430
