# Supplementary material for: Recurrent erosion of COA1/MITRAC15 exemplifies conditional gene dispensability in oxidative phosphorylation
Source: Sci Rep. 2021 Dec 24;11:24437. doi: 10.1038/s41598-021-04077-y (PMC8709867; doi:10.1038/s41598-021-04077-y)
Supplement: Supplementary file 3 — Supplementary Information 3. [file 41598_2021_4077_MOESM3_ESM.zip › Supplementary Dataset/COA1_GENE-main_Github_use_winRAR_to_open.zip!/COA1_GENE/supplementary_figure_data/Pairwise_genome_alignment/Pairwise_alignment_from_ensembl/Mouse_Shrew_mouse_lastz.odt]

Mouse  mus_musculus: 4:118187691 CCTTCCCTCCCTTCTTTCCTTCCTTCTTCCTCTTTCTTTTTTGTT mus_musculus: 4:118187735
Shrew mouse  mus_pahari: 6:69479955 CCCTCCCTCCCTCCTTCCCTCCCTTCTTCCTCTTTCTTTTTTGTT  mus_pahari: 6:69479999

Mouse  mus_musculus: 4:118187736 GTACTGGCAACTAAATCCGGGGAACTCATGCATGCTGGCTAAGGC mus_musculus: 4:118187780
Shrew mouse  mus_pahari: 6:69480000 GTTCTGGCAACTA‑‑‑‑‑‑‑‑‑AACTCATGCATGCTGGCTAAGGC  mus_pahari: 6:69480035

Mouse  mus_musculus: 4:118187781 TCTACCACCGAGCTAAGAACCAAGGCCTTTTTCAGAGATTTCCAA mus_musculus: 4:118187825
Shrew mouse  mus_pahari: 6:69480036 TCTACCACTGAGCTAAGACTCAAGTCCTTTTTCAGAGATTTCCAC  mus_pahari: 6:69480080

Mouse  mus_musculus: 4:118187826 AATACTTTTGTTACAATGTGACAAAGATCTCTACGAAGTCCTTTG mus_musculus: 4:118187870
Shrew mouse  mus_pahari: 6:69480081 CATACTTTTGCTACAATGTGACAAAGATCTCTACTAAGTCCTTTG  mus_pahari: 6:69480125

Mouse  mus_musculus: 4:118187871 TCGGGGCCTCTTGGAATACTGTTCCTTTATCCCAGTGTATTGGAG mus_musculus: 4:118187915
Shrew mouse  mus_pahari: 6:69480126 TCAGGGCCTCTTGGAATACTGTTCCTTTATTCCAGTGTATTAGAG  mus_pahari: 6:69480170

Mouse  mus_musculus: 4:118187916 GAAGGGTGGGAGAGGTGGAGGATAGGGGATA‑GGGGTGATGGTGG mus_musculus: 4:118187959
Shrew mouse  mus_pahari: 6:69480171 GAGGGGTGGGGGA‑‑TGGAGGATAGTGGGTAGGGGGTGATGGTGG  mus_pahari: 6:69480213

Mouse  mus_musculus: 4:118187960 AGAGTGCTCCTTCTCTTGTTACTTTAAGGAGAGCATAAGTGTGTC mus_musculus: 4:118188004
Shrew mouse  mus_pahari: 6:69480214 AGGTTGCTTCTTCTCTTGTTACTTTGAGGAAAGCCTAAGGGTGTC  mus_pahari: 6:69480258

Mouse  mus_musculus: 4:118188005 ATTCAGCAGACAGACAAACAGCCAACCGTCCTCTCTTCTCACTTT mus_musculus: 4:118188049
Shrew mouse  mus_pahari: 6:69480259 A‑TCAGCAGACAGACAAACAGCCAACAGTCCTCTCTTCTCACTTT  mus_pahari: 6:69480302

Mouse  mus_musculus: 4:118188050 CCTGTAGAAAGTTGAGTCCCTTGAATCTGGAGTGGCCCAGGAGGA mus_musculus: 4:118188094
Shrew mouse  mus_pahari: 6:69480303 CCTGTAGAAAGCTGAGTCCCTTGAGTCTGGAGTGGCCCAAGAAGA  mus_pahari: 6:69480347

Mouse  mus_musculus: 4:118188095 CCATTTCCAGGAATGGTTCAGACTGGACTCTAACCCAGCCTCGTC mus_musculus: 4:118188139
Shrew mouse  mus_pahari: 6:69480348 CCATTTCCAGGAATGGTTCAGACCGGACTCTAACTCAGCCTCCTC  mus_pahari: 6:69480392

Mouse  mus_musculus: 4:118188140 TCCTCTCTGCGGTGAGGACCAGGCAGGACACAGCAGACA‑GGGCA mus_musculus: 4:118188183
Shrew mouse  mus_pahari: 6:69480393 TCTTCTCTGCCATGAGGACTGGGCAGGACACAGCAGACAGGGGCA  mus_pahari: 6:69480437

Mouse  mus_musculus: 4:118188184 CATTACGGGGTCAGGATTCAGCCTTCTTGACTGACTGTCTCAGTG mus_musculus: 4:118188228
Shrew mouse  mus_pahari: 6:69480438 CATTACGGGGCCAGGATTCAGCCTTCTTGACTGACTGTCTCAGTG  mus_pahari: 6:69480482

Mouse  mus_musculus: 4:118188229 CCTTCGAAAGCTGGTGTCTGTCAGCAGAGACCATGGAGCAGTGTG mus_musculus: 4:118188273
Shrew mouse  mus_pahari: 6:69480483 CCTTCGAAAGCTGGTGTCTGTCAGTAGAGGCCATGGAGCAGTGTG  mus_pahari: 6:69480527

Mouse  mus_musculus: 4:118188274 GTCTAGGTTATACAGCCAGATGC‑‑‑‑‑‑CACACCTCAGGGCTAG mus_musculus: 4:118188312
Shrew mouse  mus_pahari: 6:69480528 GTCCAGGTTATACAGCCAGATGCCCTGGTCACACCTCAGGGCTAG  mus_pahari: 6:69480572

Mouse  mus_musculus: 4:118188313 CTTCTTTAAGCTGTCTCTCCAGGGAAGTCCTTCATCATGTTGGGT mus_musculus: 4:118188357
Shrew mouse  mus_pahari: 6:69480573 CTTCTTTAAACTGTCTCTCCAGGAAAGTCCTTCATCATGTCAGAT  mus_pahari: 6:69480617

Mouse  mus_musculus: 4:118188358 CTTAGCTGTCTTCCCTG‑‑‑‑‑‑‑‑‑‑‑‑‑‑‑‑TGCGGTATGAAT mus_musculus: 4:118188386
Shrew mouse  mus_pahari: 6:69480618 CTTATCTGTCTTCCCTGTGCTACAGTTTGAAGCTGCTGTGAGAAT  mus_pahari: 6:69480662

Mouse  mus_musculus: 4:118188387 CAAGTTCTGCATTCAAGCTATTGCAATACAGAGGATTTCTAGAGA mus_musculus: 4:118188431
Shrew mouse  mus_pahari: 6:69480663 CAAGTTCTGCATTCAAGCTATTGCAACACTGACAATTTCTAGAGA  mus_pahari: 6:69480707

Mouse  mus_musculus: 4:118188432 TATGTGGAATAGTGAGGCATGTCCCTCACCATAAAAAAAAAAAAA mus_musculus: 4:118188476
Shrew mouse  mus_pahari: 6:69480708 TATGTGGAATAGTGAGGCATGTACCTTACCAT‑TAAAAAAAAAAA  mus_pahari: 6:69480751

Mouse  mus_musculus: 4:118188477 ATCCACAGAGTTAGGGCAAGCCAGTTGGCTCAGCCAGCGAAGGTG mus_musculus: 4:118188521
Shrew mouse  mus_pahari: 6:69480752 ATCCACTGAGTTAGGGCAAGCCAGTTGGCTCAGCCAGCGAAGGTG  mus_pahari: 6:69480796

Mouse  mus_musculus: 4:118188522 TTCTGAAGCCTAATGATGAAAGTTCCGATGCTTGGAACTCATGAT mus_musculus: 4:118188566
Shrew mouse  mus_pahari: 6:69480797 TTCTGAAGCCTAATGACCAAAGTTCAGATGCTTGGAACTCATGAT  mus_pahari: 6:69480841

Mouse  mus_musculus: 4:118188567 CGAAGCACTCCTAAAAGCTGCTCTCCACATGCATACCATGGCACA mus_musculus: 4:118188611
Shrew mouse  mus_pahari: 6:69480842 CGAAGCACTCCTAAAAGCTGTTCTCCACATGCATGCCATGGCACA  mus_pahari: 6:69480886

Mouse  mus_musculus: 4:118188612 TGCCAATTG‑‑‑‑‑‑‑‑‑‑‑‑‑‑‑‑GAATACACACACACACACAC mus_musculus: 4:118188640
Shrew mouse  mus_pahari: 6:69480887 TGCCTATTAGTGCACGTGCGCGCGCGCACACACACACACACACAC  mus_pahari: 6:69480931

Mouse  mus_musculus: 4:118188641 ACACACACACACACACACACACACACACACACACACACTCCACAA mus_musculus: 4:118188685
Shrew mouse  mus_pahari: 6:69480932 ACACACACACACACACACACACACACACACACACACACACCACAA  mus_pahari: 6:69481184

Mouse  mus_musculus: 4:118188686 GTAGTAAATAAAATAAAAACAAAAACGAAGGAACTCATTTAAAAG mus_musculus: 4:118188730
Shrew mouse  mus_pahari: 6:69481185 GTAGTAAATAAAATGAAAACAAAAACGAAGGAACTCATTTAAAAG  mus_pahari: 6:69481229

Mouse  mus_musculus: 4:118188731 TAGCTGCCTAGACCCAGCCTCTCCTTGGCTACCTCTACAGCTCTG mus_musculus: 4:118188775
Shrew mouse  mus_pahari: 6:69481230 TAGCTGCCTAGACCCAGCCTCTCCTTGGCCACCCCTACAGCTCTG  mus_pahari: 6:69481274

Mouse  mus_musculus: 4:118188776 TGCCGGAGAAGCAGCATAAAGG‑‑‑‑TGGCTCTCTGTGGCTTTCC mus_musculus: 4:118188816
Shrew mouse  mus_pahari: 6:69481275 TGCCGGAGAAGCAGCATAAAGGTGGCTGGCTATCTGTGGCTTTCC  mus_pahari: 6:69481319

Mouse  mus_musculus: 4:118188817 ACGCTTGGGCTACACTGTTTCTACGACTGGACCTGCCGGAGGGAA mus_musculus: 4:118188861
Shrew mouse  mus_pahari: 6:69481320 ATGCTTGGGCTAC‑‑‑‑‑‑CCTACGATTGGACCTGTCGGAGGGGA  mus_pahari: 6:69481358

Mouse  mus_musculus: 4:118188862 GCTCTTTCATGCCACAAAACAGTTTATCGTTGTTCTGTTTCTTGA mus_musculus: 4:118188906
Shrew mouse  mus_pahari: 6:69481359 GCTCTTTCATGCCACAAAACAGTTTATTGCTGTTCTGTTTCTTGA  mus_pahari: 6:69481403

Mouse  mus_musculus: 4:118188907 GCAGCTCGTCTGACCTTACACCTTCAGGGAGAAGCTTTCCCAGGA mus_musculus: 4:118188951
Shrew mouse  mus_pahari: 6:69481404 GCAGTTTGTCTGACCTTACACCTTCAGGGAGAAGCTTTCCCAGAA  mus_pahari: 6:69481448

Mouse  mus_musculus: 4:118188952 TCCCAATAGCCTTTGGACTAGGGAGCGATCTTGGGGTAAAAAGCA mus_musculus: 4:118188996
Shrew mouse  mus_pahari: 6:69481449 TTCCAATAGCCTTTGGGCTAGGGAGTGATCTTGGGGTAAAAAGCA  mus_pahari: 6:69481493

Mouse  mus_musculus: 4:118188997 ATTTGGGGTTAAGGAGGTATCTTGCACTGAGAAGCAACTTTAGTC mus_musculus: 4:118189041
Shrew mouse  mus_pahari: 6:69481494 ATCTTGGGTTAAGGAGGTATCTTGCACTAAGAAGCAACTTTAGTC  mus_pahari: 6:69481538

Mouse  mus_musculus: 4:118189042 CGAGAGCTATTCTACTGGAAATGTTAGCTCTGCTCTGTAAACATG mus_musculus: 4:118189086
Shrew mouse  mus_pahari: 6:69481539 TGAGAGCTATTCTACTGAAAATGTTAGCTCTGCTCTGTAAACATG  mus_pahari: 6:69481583

Mouse  mus_musculus: 4:118189087 ACACTCGAGCATTTCCATGCCCTGTTTCTTGGCCCAAGGGTTTTG mus_musculus: 4:118189131
Shrew mouse  mus_pahari: 6:69481584 ACACTCGAGCATTTCCATGCCCTGTTTCTTGGCCCAAGGGTTTTG  mus_pahari: 6:69481628

Mouse  mus_musculus: 4:118189132 TTGTGGTTAATACTGTGGTCTGTCTATTTTATGCTTCATCTTCCT mus_musculus: 4:118189176
Shrew mouse  mus_pahari: 6:69481629 TTGTGGTTAATACTGTGGTCTGTCTATTTTATGCTTCATCTTCCC  mus_pahari: 6:69481673

Mouse  mus_musculus: 4:118189177 GGAAACAGTAAAATGTTTAGCTGTCACACATTCTTGGCTCGTGCT mus_musculus: 4:118189221
Shrew mouse  mus_pahari: 6:69481674 GGAAACAGTAAAATGTTTAGCTGTCACACATTCTTGGCTCGCGCT  mus_pahari: 6:69481718

Mouse  mus_musculus: 4:118189222 TTGCCTTGGGCCAGCTGTGTCACTGTGAGCAACACAATGGAATCT mus_musculus: 4:118189266
Shrew mouse  mus_pahari: 6:69481719 TTGCCTTGGGCCAGCTGTGTCACTGTGAGCAACACAATGGAATCT  mus_pahari: 6:69481763

Mouse  mus_musculus: 4:118189267 CAGGACTTTAGACCAGAGAGAACCAATACAATCCCTTCACTGAAG mus_musculus: 4:118189311
Shrew mouse  mus_pahari: 6:69481764 CGGGACTTTAGACCAGAGAGAGCCGATGCAACCCCTTCACTGAAG  mus_pahari: 6:69481808

Mouse  mus_musculus: 4:118189312 GGAAATGAGGCATTGAATGAGGTGAGGAAGAGAGATTGGACCACA mus_musculus: 4:118189356
Shrew mouse  mus_pahari: 6:69481809 GGAAATGAAGCATTGAATGAGGTGAGGAAGAGAGATTGAACCACA  mus_pahari: 6:69481853

Mouse  mus_musculus: 4:118189357 GAAATGTCAAAGTGGCCCTTTTCAAGGTCATCTCTGTTCGCCTGT mus_musculus: 4:118189401
Shrew mouse  mus_pahari: 6:69481854 GAAATGTCAAAATGGCCCTTCTCAAGGTCATCTCTGTTCGTCTGT  mus_pahari: 6:69481898

Mouse  mus_musculus: 4:118189402 CTCTATAGAATCTGCCTCTCCAGTGTGTAAAACAAACCTTGGAGT mus_musculus: 4:118189446
Shrew mouse  mus_pahari: 6:69481899 CTCTATAGAATCCGCCTCTCCAGTGTGTGAAACAAACCCTGGAGT  mus_pahari: 6:69481943

Mouse  mus_musculus: 4:118189447 AGGTAGCCACTGGATATCCTTCCATGCACAAGTTTCACAGCTATT mus_musculus: 4:118189491
Shrew mouse  mus_pahari: 6:69481944 AGATAGCCGCTGGATATCCTTCCGTGCACAAGTTTCACAGCTATT  mus_pahari: 6:69481988

Mouse  mus_musculus: 4:118189492 TCTCTGTGTGTAAATCAGCTGTGTCATTAAATTCTTTATAGAGAG mus_musculus: 4:118189536
Shrew mouse  mus_pahari: 6:69481989 TCTCTACGGGTAAATCAGCTGTGTCATTAAATTCTTTATAGAAAG  mus_pahari: 6:69482033

Mouse  mus_musculus: 4:118189537 TCCCCCGGTCAAGT  mus_musculus: 4:118189550
Shrew mouse  mus_pahari: 6:69482034 TGTCTCGGTCAAGT  mus_pahari: 6:69482047
