# Supplementary material for: Recurrent erosion of COA1/MITRAC15 exemplifies conditional gene dispensability in oxidative phosphorylation
Source: Sci Rep. 2021 Dec 24;11:24437. doi: 10.1038/s41598-021-04077-y (PMC8709867; doi:10.1038/s41598-021-04077-y)
Supplement: Supplementary file 3 — Supplementary Information 3. [file 41598_2021_4077_MOESM3_ESM.zip › Supplementary Dataset/COA1_GENE-main_Github_use_winRAR_to_open.zip!/COA1_GENE/supplementary_figure_data/Pairwise_genome_alignment/Pairwise_alignment_from_ensembl/Mouse_Prairie_vole_lastz.odt]

Mouse  mus_musculus: 4:118187691 CCTTCCCTCCCTTCTTTCCTTCCTTCTTCCTCTTTCTTTTTTGTT  mus_musculus: 4:118187735
Prairie vole microtus_ochrogaster:  JH996499.1:1736051 CTTTCCCTTCCTCCCTCCCTTCCTTCTT‑‑‑‑‑‑TCTTCCTTCTT microtus_ochrogaster:  JH996499.1:1736089

Mouse  mus_musculus: 4:118187736 GTACTGGCAACTAAATCCGGGGAACTCATGCATGCTGGCTAAG‑G  mus_musculus: 4:118187779
Prairie vole microtus_ochrogaster:  JH996499.1:1736090 GCACTGGCAATTAAACCCAGG‑CTCTCATACCTGATGGCCGAGTG microtus_ochrogaster:  JH996499.1:1736133

Mouse  mus_musculus: 4:118187780 CTCTACCACCGAGCTAAGAACCAAGGCCTTTTTCAGAGATTTCCA  mus_musculus: 4:118187824
Prairie vole microtus_ochrogaster:  JH996499.1:1736134 TTCTACCACTGAGCTAGTACTCCAGCCCTTT‑‑CATTGATTTTCA microtus_ochrogaster:  JH996499.1:1736176

Mouse  mus_musculus: 4:118187825 AAATACTTTTGTTACAATGTGACAAAGATCTCTACGAAGTCCTTT  mus_musculus: 4:118187869
Prairie vole microtus_ochrogaster:  JH996499.1:1736177 AGATTGTTTTGCTACAA‑‑‑‑‑‑‑‑AGGTCTCTAAGAGGCCATAT microtus_ochrogaster:  JH996499.1:1736213

Mouse  mus_musculus: 4:118187870 GTCGGGGCCTCTTGGAATACTGTTCCTTTATCCCAGTGTATTGGA  mus_musculus: 4:118187914
Prairie vole microtus_ochrogaster:  JH996499.1:1736214 GCCAGGGCCTCTGGGAGCACTGCTCCATTATGTCACTGTGTTGAT microtus_ochrogaster:  JH996499.1:1736258

Mouse  mus_musculus: 4:118187915 GGAAGGGTGGGAGAGGTGGAGGATAGGGGATAGGGGTGATGGTG‑  mus_musculus: 4:118187958
Prairie vole microtus_ochrogaster:  JH996499.1:1736259 GGCGGTGTGTGTGTGT‑‑‑‑‑‑‑‑‑‑‑GTGTGTGTGTGTGTGTGT microtus_ochrogaster:  JH996499.1:1736292

Mouse  mus_musculus: 4:118187959 GAGAGTGCTCCTTCTCTTGTTACTTTAAGGAGAGCATAAGTGTGT  mus_musculus: 4:118188003
Prairie vole microtus_ochrogaster:  JH996499.1:1736293 GTGTGTGCTCCTTTTCCTGTTATCT‑‑‑‑‑‑‑‑‑CAGAAGTGTAT microtus_ochrogaster:  JH996499.1:1736328

Mouse  mus_musculus: 4:118188004 CATTCAGCAGACAGACAAACAGCCAACCGTCCTCTCTTCTCACTT  mus_musculus: 4:118188048
Prairie vole microtus_ochrogaster:  JH996499.1:1736329 CAC‑CAGCAGACAGGCAAA‑‑GTCAATGGTCCTCTCCTCTCACTT microtus_ochrogaster:  JH996499.1:1736370

Mouse  mus_musculus: 4:118188049 TCCTGTAGAAAGTTGAGTCCCTTGAATCTGGAGTGGCCCAGGAGG  mus_musculus: 4:118188093
Prairie vole microtus_ochrogaster:  JH996499.1:1736371 TGCTGTAGAAAGCTGAGTCCCTTGAGTCTGGAGTGGCCCAAGAGG microtus_ochrogaster:  JH996499.1:1736415

Mouse  mus_musculus: 4:118188094 ACCATTTCCAGGAATGGTTCAGACTGGACTCTAACCCAGCCTCGT  mus_musculus: 4:118188138
Prairie vole microtus_ochrogaster:  JH996499.1:1736416 AGT‑TTCCCAGGAATGTTTCACACTTGACTATAACTTGGCCTC‑‑ microtus_ochrogaster:  JH996499.1:1736457

Mouse  mus_musculus: 4:118188139 CTCCTCTCTGCGGTGAGGACCAGGCAGGACACAGCAGACA‑GGGC  mus_musculus: 4:118188182
Prairie vole microtus_ochrogaster:  JH996499.1:1736458 ‑‑‑TTCTCTGTCGTGATGACCAGGAAGGACACGACAGAAATGGGC microtus_ochrogaster:  JH996499.1:1736499

Mouse  mus_musculus: 4:118188183 ACATTACGGGGTCAGGATTCAGCCTTCTTGACTGACTGTCTCAGT  mus_musculus: 4:118188227
Prairie vole microtus_ochrogaster:  JH996499.1:1736500 TCATTTAGGGGTCAGGATTCAGCCTTCTTGACTGACATCTTCCGT microtus_ochrogaster:  JH996499.1:1736544

Mouse  mus_musculus: 4:118188228 GCCTTCGAAAGCTGGTGTCTGTCAGCAGAGACCATGGAG‑CAGTG  mus_musculus: 4:118188271
Prairie vole microtus_ochrogaster:  JH996499.1:1736545 GCCTTAGAAAGCTGGTGTCTACTAGCAGAGGCCATGGAGGCAGTG microtus_ochrogaster:  JH996499.1:1736589

Mouse  mus_musculus: 4:118188272 TGGTCTAGGTTATACAGCCAGATGCCACACCTCAGGGCTAGCTTC  mus_musculus: 4:118188316
Prairie vole microtus_ochrogaster:  JH996499.1:1736590 TGT‑‑‑‑GGTTAGATGCCCTG‑‑GTCAAACCTCAGGTTGCGCTTT microtus_ochrogaster:  JH996499.1:1736628

Mouse  mus_musculus: 4:118188317 TTTAAGCTGTCTCTCCAGGGAAGTCCTTCATCATGTTGGGTCTTA  mus_musculus: 4:118188361
Prairie vole microtus_ochrogaster:  JH996499.1:1736629 TCTATG‑TATCTCTACAAAGGAGTCCTTATTGGTGCTGGGTCCTG microtus_ochrogaster:  JH996499.1:1736672

Mouse  mus_musculus: 4:118188362 GCTGTCTTCCCTGTGCGGTATGA‑‑‑‑‑‑‑‑‑‑‑‑ATCAAGTTCT  mus_musculus: 4:118188394
Prairie vole microtus_ochrogaster:  JH996499.1:1736673 GCTGCCTTCCCTGTGTGGTATAACATTGTTGTGAGATCAAACTGT microtus_ochrogaster:  JH996499.1:1736717

Mouse  mus_musculus: 4:118188395 G‑‑‑CATTCAAGCTATTGCAATACAGAGGATTTCTAGAGATATGT  mus_musculus: 4:118188436
Prairie vole microtus_ochrogaster:  JH996499.1:1736718 AGGATATTCAAGCTATTGCATTATTGAGGATTTCTAGAGATTTG‑ microtus_ochrogaster:  JH996499.1:1736761

Mouse  mus_musculus: 4:118188437 GGAATAGTGAGGCATGTCCCTCACCATAAAAAAAAAAAAAATCCA  mus_musculus: 4:118188481
Prairie vole microtus_ochrogaster:  JH996499.1:1736762 GAAATAGTGAGGTACGTCCCTTGCCATAAAAG‑‑‑‑‑‑‑‑‑‑TCA microtus_ochrogaster:  JH996499.1:1736796

Mouse  mus_musculus: 4:118188482 CAGAGTTAGGGCAAGCCAGTTGGCTCAGCCAGCGAAGGTGTTCTG  mus_musculus: 4:118188526
Prairie vole microtus_ochrogaster:  JH996499.1:1736797 CTGAGTTGGGGCAAGCCGGATGACTCAGCCAGTGAAGGTATCCTG microtus_ochrogaster:  JH996499.1:1736841

Mouse  mus_musculus: 4:118188527 AAGCCTAATGATGAAAGTTCCGATGCTTGGAACTCATGATCGAAG  mus_musculus: 4:118188571
Prairie vole microtus_ochrogaster:  JH996499.1:1736842 ACGTCCATTGACCTAAGTTCAGATGCCTGAAACTCGCAATGAAAG microtus_ochrogaster:  JH996499.1:1736886

Mouse  mus_musculus: 4:118188572 CACTCCTAAAAGCTGC‑‑‑‑‑TCTCCACATGCATACCATGGCACA  mus_musculus: 4:118188611
Prairie vole microtus_ochrogaster:  JH996499.1:1736887 AATTCCTAAAAGCTGTCCTCATCTCCATGTGCATGCCACA‑CGCT microtus_ochrogaster:  JH996499.1:1736930

Mouse  mus_musculus: 4:118188612 TGCCAATTGGAA‑TACACACACA‑‑‑‑‑‑‑‑‑‑‑‑‑‑‑‑‑‑‑‑‑‑  mus_musculus: 4:118188633
Prairie vole microtus_ochrogaster:  JH996499.1:1736931 TGCCTGCCAGAACTACACCCCCTTCTCTTTTCTCTCTCTCTCTCT microtus_ochrogaster:  JH996499.1:1736975

Mouse  mus_musculus: 4:118188634 ‑‑‑‑‑‑‑‑‑‑‑‑‑‑‑‑‑‑‑‑‑‑‑‑CACACACACACACACACACAC  mus_musculus: 4:118188654
Prairie vole microtus_ochrogaster:  JH996499.1:1736976 CTCTCTCTCTCTCTCTCTCTCTCTCTCTCTCTCTCACACACACAC microtus_ochrogaster:  JH996499.1:1737020

Mouse  mus_musculus: 4:118188655 ACACACACACACACACACACACACTCCACAAGTAGTAAATAAAA‑  mus_musculus: 4:118188698
Prairie vole microtus_ochrogaster:  JH996499.1:1737021 ACACACACACACACACACACACTGCACACAAATAATAAATAAAAG microtus_ochrogaster:  JH996499.1:1737065

Mouse  mus_musculus: 4:118188699 ‑‑‑‑TAAAAACAAAAACGAAGGAACTCATTTAAAAGTAGCTGCCT  mus_musculus: 4:118188739
Prairie vole microtus_ochrogaster:  JH996499.1:1737066 AAAATAAAAATAAATACAAAGGGGCCCATT‑‑‑‑‑GTAGGTGTCA microtus_ochrogaster:  JH996499.1:1737105

Mouse  mus_musculus: 4:118188740 AGACCCAGCCTCTCCTTGGCTACCTCTACAGCTCTGTGCCGGAGA  mus_musculus: 4:118188784
Prairie vole microtus_ochrogaster:  JH996499.1:1737106 AAACCCAGCCTCTCCTTGGCCATACCTACAGCTCTGTGGTCTGGA microtus_ochrogaster:  JH996499.1:1737150

Mouse  mus_musculus: 4:118188785 AGCAGCATAAAGGTGGCTCTCTGTGGCTTTCCACGCTTGGGCTAC  mus_musculus: 4:118188829
Prairie vole microtus_ochrogaster:  JH996499.1:1737151 AACAGT‑‑‑‑‑‑‑‑‑‑‑‑‑‑‑‑‑‑‑‑‑‑‑‑‑‑‑‑GCTTGGGTCAC microtus_ochrogaster:  JH996499.1:1737167

Mouse  mus_musculus: 4:118188830 ACTGTTTCTACGACTGGACCTGCCGGAGGGAAGCTC‑TTTCATGC  mus_musculus: 4:118188873
Prairie vole microtus_ochrogaster:  JH996499.1:1737168 CTTGTTCCCACGATTGGACCTGCAAGACGGAAGCTCCTTTTGTGC microtus_ochrogaster:  JH996499.1:1737212

Mouse  mus_musculus: 4:118188874 CAC‑‑‑‑‑‑AAAACAGTTTATCGTTGTTCTGTTTCTTGAGCAGCT  mus_musculus: 4:118188912
Prairie vole microtus_ochrogaster:  JH996499.1:1737213 CATCTAGCTAATACAGTTTATCTTTGCTCTGCTTCTTGAACAGCT microtus_ochrogaster:  JH996499.1:1737257

Mouse  mus_musculus: 4:118188913 CGTCTGACCTTACACCTTCAGGGAGAAGCTTTCCCAGGATCCCAA  mus_musculus: 4:118188957
Prairie vole microtus_ochrogaster:  JH996499.1:1737258 TGTCAGTCCCTATACCTTCCGGGAGAACCTATCCAAGAATTCCAG microtus_ochrogaster:  JH996499.1:1737302

Mouse  mus_musculus: 4:118188958 TAGCCTTTGGACTAGG‑‑‑‑‑‑‑‑GAGCGATCTTGGGGTAAAAAG  mus_musculus: 4:118188994
Prairie vole microtus_ochrogaster:  JH996499.1:1737303 TAGCCTTTAGGCTAAGATGCAAGTGAGAGATCTTGGGGTAAAAC‑ microtus_ochrogaster:  JH996499.1:1737346

Mouse  mus_musculus: 4:118188995 CAATTTGGGGTTAAGGAGGTATCTTGCACTGAGAAGCAACTTTAG  mus_musculus: 4:118189039
Prairie vole microtus_ochrogaster:  JH996499.1:1737347 CAATCTTGGGCCAAGAAGGGCTCTTG‑‑‑‑‑‑‑‑‑‑‑‑‑‑‑‑‑‑G microtus_ochrogaster:  JH996499.1:1737373

Mouse  mus_musculus: 4:118189040 TCCGAGAGCTATTCTACTGGAAATGTTAGCTCTGCTCTGTAAACA  mus_musculus: 4:118189084
Prairie vole microtus_ochrogaster:  JH996499.1:1737374 GCGGAGAACTGTTCTACTGGAAATGTTATCTCTGATCCGTAAATG microtus_ochrogaster:  JH996499.1:1737418

Mouse  mus_musculus: 4:118189085 TGACACTCGAGCATTTCCATGCCCTGTTTCTTGGCCCAAGGGTTT  mus_musculus: 4:118189129
Prairie vole microtus_ochrogaster:  JH996499.1:1737419 TGACAC‑‑GAGCATTTCCATGCCCTGCTTCTCGGCCCAAGGGTTT microtus_ochrogaster:  JH996499.1:1737461

Mouse  mus_musculus: 4:118189130 TGTTGTGGTTAATACTGTGGTCTGTCTATTTTATGCTTCATCTTC  mus_musculus: 4:118189174
Prairie vole microtus_ochrogaster:  JH996499.1:1737462 TGTTGTGGTTAATACTGTGGCCTGTCTATTTTATGCTTCATCTTC microtus_ochrogaster:  JH996499.1:1737506

Mouse  mus_musculus: 4:118189175 CTGGAAACAGTAAAATGTTTAGCTGTCACACATTCTTGGCTCGTG  mus_musculus: 4:118189219
Prairie vole microtus_ochrogaster:  JH996499.1:1737507 CTGGAAACAGTGAAATGTTTAGTTGTCACACATTCCTGGCTCGCG microtus_ochrogaster:  JH996499.1:1737551

Mouse  mus_musculus: 4:118189220 CTTTGCCTTGGGCCAGCTGTGTCACTGTGAGCAACACAATGGAAT  mus_musculus: 4:118189264
Prairie vole microtus_ochrogaster:  JH996499.1:1737552 CTTTGCCTTGGGCCAGCTGTGTCACTTTGAGCAACACAATGGAAC microtus_ochrogaster:  JH996499.1:1737596

Mouse  mus_musculus: 4:118189265 CTCAGGACTTTAGACCAGAGAGAACCAATACAATCCCTTCACTGA  mus_musculus: 4:118189309
Prairie vole microtus_ochrogaster:  JH996499.1:1737597 CGTGGGACTTTAGGTCAGAGAGAGCTGATTT‑‑‑‑‑‑‑‑‑‑‑‑‑‑ microtus_ochrogaster:  JH996499.1:1737627

Mouse  mus_musculus: 4:118189310 AGGGAAATGAGGCATTGAATGAGGTGAGG‑‑AAGAGAGATTGGAC  mus_musculus: 4:118189352
Prairie vole microtus_ochrogaster:  JH996499.1:1737628 ‑‑‑‑‑‑‑‑‑‑‑‑‑‑‑‑‑‑‑‑‑‑GGTGAGGAAAAGAGAGGTTGAAC microtus_ochrogaster:  JH996499.1:1737650

Mouse  mus_musculus: 4:118189353 CACAGAAATGTCAAAGTGGCCCTTTTCAAGGTCATCTCTGTTCGC  mus_musculus: 4:118189397
Prairie vole microtus_ochrogaster:  JH996499.1:1737651 ACCAGAAGAGCCAGGACGGCCCTTTTCAAGGTCGTCTGTGTT‑‑‑ microtus_ochrogaster:  JH996499.1:1737692

Mouse  mus_musculus: 4:118189398 CTGTCTCTATAGAATCTGCCTCTCCAGTGTGTAAAACAAACCTTG  mus_musculus: 4:118189442
Prairie vole microtus_ochrogaster:  JH996499.1:1737693 ‑‑‑‑‑‑‑‑‑‑‑‑‑‑‑‑‑‑CCTCTCTGCGGTGTAGA‑‑‑‑‑‑‑‑‑‑ microtus_ochrogaster:  JH996499.1:1737709

Mouse  mus_musculus: 4:118189443 GAGTAGGTAGCCACTGGATATCCTTCCATGCACAAGTTTCACAGC  mus_musculus: 4:118189487
Prairie vole microtus_ochrogaster:  JH996499.1:1737710 ‑‑‑‑‑‑‑‑‑‑‑‑‑‑‑‑‑‑‑TTCCTTCTATG............... microtus_ochrogaster:  JH996499.1:1737720

Mouse  mus_musculus: 4:118189488 TATTTCTCTGTGTGTAAATCAGCTGTGTCATTAAATTCTTTATAG  mus_musculus: 4:118189532
Prairie vole  .............................................  

Mouse  mus_musculus: 4:118189533 AGAGTCCCCCGGTCAAGT  mus_musculus: 4:118189550
Prairie vole  ..................
