# Supplementary material for: Recurrent erosion of COA1/MITRAC15 exemplifies conditional gene dispensability in oxidative phosphorylation
Source: Sci Rep. 2021 Dec 24;11:24437. doi: 10.1038/s41598-021-04077-y (PMC8709867; doi:10.1038/s41598-021-04077-y)
Supplement: Supplementary file 3 — Supplementary Information 3. [file 41598_2021_4077_MOESM3_ESM.zip › Supplementary Dataset/COA1_GENE-main_Github_use_winRAR_to_open.zip!/COA1_GENE/supplementary_figure_data/Pairwise_genome_alignment/Pairwise_alignment_from_ensembl/Mouse_Steppe_mouse_lastz.odt]

Mouse  mus_musculus: 4:118187691 CCTTCCCTCCCTTCTTTCCTTCCTTCTTCCTCTTTCTTTTTTGTT  mus_musculus: 4:118187735
Steppe mouse mus_spicilegus: QGOO01037132.1:1367321 CCTTCCCTCCCTTCTTTCCTTCCTTCTTCCTCTTTCTTTTTTGTT mus_spicilegus: QGOO01037132.1:1367365

Mouse  mus_musculus: 4:118187736 GTACTGGCAACTAAATCCGGGGAACTCATGCATGCTGGCTAAGGC  mus_musculus: 4:118187780
Steppe mouse mus_spicilegus: QGOO01037132.1:1367366 GTACTGGCAACTAAATCCGGGGAACTCATGCATGCTGGCTAAGGC mus_spicilegus: QGOO01037132.1:1367410

Mouse  mus_musculus: 4:118187781 TCTACCACCGAGCTAAGAACCAAGGCCTTTTTCAGAGATTTCCAA  mus_musculus: 4:118187825
Steppe mouse mus_spicilegus: QGOO01037132.1:1367411 TCTACCACTGAGCTAAGAACCAAGCCCTTTCTCAGAGATTTCCAA mus_spicilegus: QGOO01037132.1:1367455

Mouse  mus_musculus: 4:118187826 AATACTTTTGTTACAATGTGACAAAGATCTCTACGAAGTCCTTTG  mus_musculus: 4:118187870
Steppe mouse mus_spicilegus: QGOO01037132.1:1367456 AATACTTTTGTTACAATGTGACAAAGATCTTTACGAAGTCCTTTG mus_spicilegus: QGOO01037132.1:1367500

Mouse  mus_musculus: 4:118187871 TCG‑‑‑‑‑‑GGGCCTCTTGGAATACTGTTCCTTTATCCCAGTGTA  mus_musculus: 4:118187909
Steppe mouse mus_spicilegus: QGOO01037132.1:1367501 TCCTTTGTCGGGCCTCTTGGAATACTGTTCCTTTATCCCAGTGTA mus_spicilegus: QGOO01037132.1:1367545

Mouse  mus_musculus: 4:118187910 TTGGAGGAAGGGTGGGAGAGGTGGAGGATAGGGGATAGGGGTGAT  mus_musculus: 4:118187954
Steppe mouse mus_spicilegus: QGOO01037132.1:1367546 TTGGAGGAGGGGTGGGAGAGGTGGAGGATAGGGGATAGGGGTGAT mus_spicilegus: QGOO01037132.1:1367590

Mouse  mus_musculus: 4:118187955 GGTGGAGAGTGCTCCTTCTCTTGTTACTTTAAGGAGAGCATAAGT  mus_musculus: 4:118187999
Steppe mouse mus_spicilegus: QGOO01037132.1:1367591 GGTGGAGAGTGCTCCTTCTCTTGTTACTTTAAGGAGAGCATAAGT mus_spicilegus: QGOO01037132.1:1367635

Mouse  mus_musculus: 4:118188000 GTGTCATTCAGCAGACAGACAAACAGCCAACCGTCCTCTCTTCTC  mus_musculus: 4:118188044
Steppe mouse mus_spicilegus: QGOO01037132.1:1367636 GTGTCATTCAGCAGACAGACAAACAGCCAACGGTCCTCTCTTCTC mus_spicilegus: QGOO01037132.1:1367680

Mouse  mus_musculus: 4:118188045 ACTTTCCTGTAGAAAGTTGAGTCCCTTGAATCTGGAGTGGCCCAG  mus_musculus: 4:118188089
Steppe mouse mus_spicilegus: QGOO01037132.1:1367681 ACTTTCCTGTAGAAAGCTGAGTCCCTTGAATCTGGAGTGGCCCAG mus_spicilegus: QGOO01037132.1:1367725

Mouse  mus_musculus: 4:118188090 GAGGACCATTTCCAGGAATGGTTCAGACTGGACTCTAACCCAGCC  mus_musculus: 4:118188134
Steppe mouse mus_spicilegus: QGOO01037132.1:1367726 GAAGACCATTTCCAGGAATGGTTCAGACTGGACTCTAACCCAGCC mus_spicilegus: QGOO01037132.1:1367770

Mouse  mus_musculus: 4:118188135 TCGTCTCCTCTCTGCGGTGAGGACCAGGCAGGACACAGCAGACAG  mus_musculus: 4:118188179
Steppe mouse mus_spicilegus: QGOO01037132.1:1367771 TCGCCTCCTCTCTGCGGTGAGGACTGGGCAGGACACAGCAGACAG mus_spicilegus: QGOO01037132.1:1367815

Mouse  mus_musculus: 4:118188180 GGCACATTACGGGGTCAGGATTCAGCCTTCTTGACTGACTGTCTC  mus_musculus: 4:118188224
Steppe mouse mus_spicilegus: QGOO01037132.1:1367816 GGCACATTACGGGGTCAGGATTCAGCCTTCTTGACTGACTGTCTC mus_spicilegus: QGOO01037132.1:1367860

Mouse  mus_musculus: 4:118188225 AGTGCCTTCGAAAGCTGGTGTCTGTCAGCAGAGACCATGGAGCAG  mus_musculus: 4:118188269
Steppe mouse mus_spicilegus: QGOO01037132.1:1367861 AGTGCCTTCAAAAGCTGGTGTCTGTCAGCAGAGACCATGGAGCAG mus_spicilegus: QGOO01037132.1:1367905

Mouse  mus_musculus: 4:118188270 TGTGGTCTAGGTTATACAGCCAGATGCCACACCTCAGGGCTAGCT  mus_musculus: 4:118188314
Steppe mouse mus_spicilegus: QGOO01037132.1:1367906 TGTGGTCTAGGTTATACAGCCAGATGCCACACCTCAGGGCTAGCT mus_spicilegus: QGOO01037132.1:1367950

Mouse  mus_musculus: 4:118188315 TCTTTAAGCTGTCTCTCCAGGGAAGTCCTTCATCATGTTGGGTCT  mus_musculus: 4:118188359
Steppe mouse mus_spicilegus: QGOO01037132.1:1367951 TCTTTAAGCTGTCTCTCCAGGGAAGTCCTTCATCATGTTGGGTCT mus_spicilegus: QGOO01037132.1:1367995

Mouse  mus_musculus: 4:118188360 TAGCTGTCTTCCCTGTGCGGTATGAATCAAGTTCTGCATTCAAGC  mus_musculus: 4:118188404
Steppe mouse mus_spicilegus: QGOO01037132.1:1367996 TAGCTGTTTTCCCTGTGCGGTATGAATCAAGTTCTGCATTCAAGC mus_spicilegus: QGOO01037132.1:1368040

Mouse  mus_musculus: 4:118188405 TATTGCAATACAGAGGATTTCTAGAGATATGTGGAATAGTGAGGC  mus_musculus: 4:118188449
Steppe mouse mus_spicilegus: QGOO01037132.1:1368041 TATTGCAATACAGAGGATTTCTAGAGATATGTGGAATAGTGAGGC mus_spicilegus: QGOO01037132.1:1368085

Mouse  mus_musculus: 4:118188450 ATGTCCCTCACCATAAAAAAAAAAAAAATCCACAGAGTTAGGGCA  mus_musculus: 4:118188494
Steppe mouse mus_spicilegus: QGOO01037132.1:1368086 ATGTCCCTCACCTTAAAAAAAAAAAAAATCCACAGAGTTAGGACA mus_spicilegus: QGOO01037132.1:1368130

Mouse  mus_musculus: 4:118188495 AGCCAGTTGGCTCAGCCAGCGAAGGTGTTCTGAAGCCTAATGATG  mus_musculus: 4:118188539
Steppe mouse mus_spicilegus: QGOO01037132.1:1368131 AGCCAGTTGGCTCAGCCAGCGAAGGTGTTCTGAAGCCTAATGATG mus_spicilegus: QGOO01037132.1:1368175

Mouse  mus_musculus: 4:118188540 AAAGTTCCGATGCTTGGAACTCATGATCGAAGCACTCCTAAAAGC  mus_musculus: 4:118188584
Steppe mouse mus_spicilegus: QGOO01037132.1:1368176 AAAGTTCCGATGCTTGGAACTCATGATCGAAGCACTCCTAAAAGC mus_spicilegus: QGOO01037132.1:1368220

Mouse  mus_musculus: 4:118188585 TGCTCTCCACATGCATACCATGGCACATGCCAATTGGAATACACA  mus_musculus: 4:118188629
Steppe mouse mus_spicilegus: QGOO01037132.1:1368221 TGCTCTCCACATGCATACCATGGCACATGCCAATTGGAATACACA mus_spicilegus: QGOO01037132.1:1368265

Mouse  mus_musculus: 4:118188630 CACACACACACACACACACACACACACACACACACACACACACAC  mus_musculus: 4:118188674
Steppe mouse mus_spicilegus: QGOO01037132.1:1368266 CACACACACACACACACACACAC‑‑‑‑‑‑‑‑‑‑‑‑‑‑‑‑‑‑‑‑‑‑ mus_spicilegus: QGOO01037132.1:1368288

Mouse  mus_musculus: 4:118188675 ACACTCCACAAGTAGTAAATAAAATAAAAACAAAAACGAAGGAAC  mus_musculus: 4:118188719
Steppe mouse mus_spicilegus: QGOO01037132.1:1368289 ‑‑‑‑TCCACAAGTAGTAAATAAAATAAAAACAAAAACGAAGAAAC mus_spicilegus: QGOO01037132.1:1368329

Mouse  mus_musculus: 4:118188720 TCATTTAAAAGTAGCTGCCTAGACCCAGCCTCTCCTTGGCTACCT  mus_musculus: 4:118188764
Steppe mouse mus_spicilegus: QGOO01037132.1:1368330 TCATTTAAAAGTAGCTGCCTAGACCCAGCCTCTCCTTGGCTACCT mus_spicilegus: QGOO01037132.1:1368374

Mouse  mus_musculus: 4:118188765 CTACAGCTCTGTGCCGGAGAAGCAGCATAAAGGTGGCTCTCTGTG  mus_musculus: 4:118188809
Steppe mouse mus_spicilegus: QGOO01037132.1:1368375 CTACAGCTCTGTGCCGGAGAAGCAGCATAAAGGTGGCTCTCTGTG mus_spicilegus: QGOO01037132.1:1368419

Mouse  mus_musculus: 4:118188810 GCTTTCCACGCTTGGGCTACACTGTTTCTACGACTGGACCTGCCG  mus_musculus: 4:118188854
Steppe mouse mus_spicilegus: QGOO01037132.1:1368420 GCTTTCCACGCTTGGGCTACCCTGTTTCTATGACTGGACCTGCCG mus_spicilegus: QGOO01037132.1:1368464

Mouse  mus_musculus: 4:118188855 GAGGGAAGCTCTTTCATGCCACAAAACAGTTTATCGTTGTTCTGT  mus_musculus: 4:118188899
Steppe mouse mus_spicilegus: QGOO01037132.1:1368465 GAGGGAAGCTCTTTCATGCCACAAAACAGTTTATCATTGTTCTGT mus_spicilegus: QGOO01037132.1:1368509

Mouse  mus_musculus: 4:118188900 TTCTTGAGCAGCTCGTCTGACCTTACACCTTCAGGGAGAAGCTTT  mus_musculus: 4:118188944
Steppe mouse mus_spicilegus: QGOO01037132.1:1368510 TTCTTGAGCAGCTCGTCTGACCTTACACCTTCAGGGAGAAGCTTT mus_spicilegus: QGOO01037132.1:1368554

Mouse  mus_musculus: 4:118188945 CCCAGGATCCCAATAGCCTTTGGACTAGGGAGCGATCTTGGGGTA  mus_musculus: 4:118188989
Steppe mouse mus_spicilegus: QGOO01037132.1:1368555 CCCAGGATCCCAATAGCCTTTGGACTAGGGAGCGATCTTGGGGTA mus_spicilegus: QGOO01037132.1:1368599

Mouse  mus_musculus: 4:118188990 AAAAGCAATTTGGGGTTAAGGAGGTATCTTGCACTGAGAAGCAAC  mus_musculus: 4:118189034
Steppe mouse mus_spicilegus: QGOO01037132.1:1368600 AAAAGCAATTTGAGGTTAAGGAGGTATCTTGCACTGAGAAGCAAA mus_spicilegus: QGOO01037132.1:1368644

Mouse  mus_musculus: 4:118189035 TTTAGTCCGAGAGCTATTCTACTGGAAATGTTAGCTCTGCTCTGT  mus_musculus: 4:118189079
Steppe mouse mus_spicilegus: QGOO01037132.1:1368645 TTTAGTCCGAGAGCTATTCTACTGGAAATGTTAGCTCTGCTCTGT mus_spicilegus: QGOO01037132.1:1368689

Mouse  mus_musculus: 4:118189080 AAACATGACACTCGAGCATTTCCATGCCCTGTTTCTTGGCCCAAG  mus_musculus: 4:118189124
Steppe mouse mus_spicilegus: QGOO01037132.1:1368690 AAACATGACACTCGAGCATTTCCATGCCCTGTTTCTTGGCCCAAG mus_spicilegus: QGOO01037132.1:1368734

Mouse  mus_musculus: 4:118189125 GGTTTTGTTGTGGTTAATACTGTGGTCTGTCTATTTTATGCTTCA  mus_musculus: 4:118189169
Steppe mouse mus_spicilegus: QGOO01037132.1:1368735 GGTTTTGTTGTGGTTAATACTGTGGTCTGTCTATTTTATGCTTCA mus_spicilegus: QGOO01037132.1:1368779

Mouse  mus_musculus: 4:118189170 TCTTCCTGGAAACAGTAAAATGTTTAGCTGTCACACATTCTTGGC  mus_musculus: 4:118189214
Steppe mouse mus_spicilegus: QGOO01037132.1:1368780 TCTTCCCGGAAACAGTAAAATGTTTAGCTGTCACACATTCTTGGC mus_spicilegus: QGOO01037132.1:1368824

Mouse  mus_musculus: 4:118189215 TCGTGCTTTGCCTTGGGCCAGCTGTGTCACTGTGAGCAACACAAT  mus_musculus: 4:118189259
Steppe mouse mus_spicilegus: QGOO01037132.1:1368825 TCGCGCTTTGCCTTGGGCCAGCTGTGTCACTGTGAGCAACACAAT mus_spicilegus: QGOO01037132.1:1368869

Mouse  mus_musculus: 4:118189260 GGAATCTCAGGACTTTAGACCAGAGAGAACCAATACAATCCCTTC  mus_musculus: 4:118189304
Steppe mouse mus_spicilegus: QGOO01037132.1:1368870 GGAATCTCAGGACTTTAGACCAGAGAGAACCAATACAACCCCTTC mus_spicilegus: QGOO01037132.1:1368914

Mouse  mus_musculus: 4:118189305 ACTGAAGGGAAATGAGGCATTGAATGAGGTGAGGAAGAGAGATTG  mus_musculus: 4:118189349
Steppe mouse mus_spicilegus: QGOO01037132.1:1368915 ACTGAAGG‑AAATGAGGCATTGAATGAGGTGAGGAAGAGAGATTG mus_spicilegus: QGOO01037132.1:1368958

Mouse  mus_musculus: 4:118189350 GACCACAGAAATGTCAAAGTGGCCCTTTTCAAGGTCATCTCTGTT  mus_musculus: 4:118189394
Steppe mouse mus_spicilegus: QGOO01037132.1:1368959 GACCACAGAAATGTCAAAGTGGCCCTTTTCAAGGTCATCTCTGTT mus_spicilegus: QGOO01037132.1:1369003

Mouse  mus_musculus: 4:118189395 CGCCTGTCTCTATAGAATCTGCCTCTCCAGTGTGTAAAACAAACC  mus_musculus: 4:118189439
Steppe mouse mus_spicilegus: QGOO01037132.1:1369004 CGCCTGTCTCTATAGAATCTGCCTCTCCAGTGTGTAAAACAAACT mus_spicilegus: QGOO01037132.1:1369048

Mouse  mus_musculus: 4:118189440 TTGGAGTAGGTAGCCACTGGATATCCTTCCATGCACAAGTTTCAC  mus_musculus: 4:118189484
Steppe mouse mus_spicilegus: QGOO01037132.1:1369049 TTGGAGTAGGTAGCCACTGGATATCCTTCCATGCACAAGTTTCAC mus_spicilegus: QGOO01037132.1:1369093

Mouse  mus_musculus: 4:118189485 AGCTATTTCTCTGTGTGTAAATCAGCTGTGTCATTAAATTCTTTA  mus_musculus: 4:118189529
Steppe mouse mus_spicilegus: QGOO01037132.1:1369094 AGCTATTTCTCTACGTGTAAATCAGCTGTGTCATTAAATTCTTTA mus_spicilegus: QGOO01037132.1:1369138

Mouse  mus_musculus: 4:118189530 TAGAGAGTCCCCCGGTCAAGT  mus_musculus: 4:118189550
Steppe mouse mus_spicilegus: QGOO01037132.1:1369139 TAGAGAGTCCCCCGGTCAAGT  mus_spicilegus: QGOO01037132.1:1369159
