# Supplementary material for: Recurrent erosion of COA1/MITRAC15 exemplifies conditional gene dispensability in oxidative phosphorylation
Source: Sci Rep. 2021 Dec 24;11:24437. doi: 10.1038/s41598-021-04077-y (PMC8709867; doi:10.1038/s41598-021-04077-y)
Supplement: Supplementary file 3 — Supplementary Information 3. [file 41598_2021_4077_MOESM3_ESM.zip › Supplementary Dataset/COA1_GENE-main_Github_use_winRAR_to_open.zip!/COA1_GENE/supplementary_figure_data/Pairwise_genome_alignment/Pairwise_alignment_from_ensembl/Mouse_Algerian_mouse_lastz.odt]

Mouse  mus_musculus: 4:118187691 CCTTCCCTCCCTTCTTTCCTTCCTTCTTCCTCTTTCTTTTTTGTT mus_musculus: 4:118187735
Algerian mouse  mus_spretus: 4:114718559 NNNNNNNNNNNNNNNNNNNNNNNNNNNNNNNNNNNNNNNNNNNNN  mus_spretus: 4:114718603

Mouse  mus_musculus: 4:118187736 GTACTGGCAACTAAATCCGGGGAACTCATGCATGCTGGCTAAGGC mus_musculus: 4:118187780
Algerian mouse  mus_spretus: 4:114718604 NNNNNNNNNNNNNNNNNNNNNNNNNNNNNNNNNNNNNNNNNNNNN  mus_spretus: 4:114718648

Mouse  mus_musculus: 4:118187781 TCTACCACCGAGCTAAGAACCAAGGCCTTTTTCAGAGATTTCCAA mus_musculus: 4:118187825
Algerian mouse  mus_spretus: 4:114718649 NNNNNNNNNNNNNNNNNNNNNNNNNNNNNNNNNNNNNNNNNNNNN  mus_spretus: 4:114718693

Mouse  mus_musculus: 4:118187826 AATACTTTTGTTACAATGTGACAAAGATCTCTACGAAGTCCTTTG mus_musculus: 4:118187870
Algerian mouse  mus_spretus: 4:114718694 NNNNNNNNNNNNNNNNNNNNNNNNNNNNNNNNNNNNNNNNNNNNN  mus_spretus: 4:114718738

Mouse  mus_musculus: 4:118187871 TCGGGGCCTCTTGGAATACTGTTCCTTTATCCCAGTGTATTGGAG mus_musculus: 4:118187915
Algerian mouse  mus_spretus: 4:114718739 NNNNNNNNNNNNNNNNNNNNNNNNNNNNNNNNCAGTGTATTGGAG  mus_spretus: 4:114720092

Mouse  mus_musculus: 4:118187916 GAAGGGTGGGAGAGGTGGAGGATAGGGGATAGGGGTGATGGTGGA mus_musculus: 4:118187960
Algerian mouse  mus_spretus: 4:114720093 GAGGGGTGGGGGAGGTGCAGGATAGGGGATAGGGGTGATGGTGGA  mus_spretus: 4:114720137

Mouse  mus_musculus: 4:118187961 GAGTGCTCCTTCTCTTGTTACTTTAAGGAGAGCATAAGTGTGTCA mus_musculus: 4:118188005
Algerian mouse  mus_spretus: 4:114720138 GAGTGCTCCTTCTCTTGTTACTTTAAGGAGAGCATAAGTGTGTCA  mus_spretus: 4:114720182

Mouse  mus_musculus: 4:118188006 TTCAGCAGACAGACAAACAGCCAACCGTCCTCTCTTCTCACTTTC mus_musculus: 4:118188050
Algerian mouse  mus_spretus: 4:114720183 TCCAGCAGACAGACAAACAGCCAACGGTCCTCTCTTCTCACTTTC  mus_spretus: 4:114720227

Mouse  mus_musculus: 4:118188051 CTGTAGAAAGTTGAGTCCCTTGAATCTGGAGTGGCCCAGGAGGAC mus_musculus: 4:118188095
Algerian mouse  mus_spretus: 4:114720228 CTGTAGAAAGCTGAGTCCCTTGAATCTGGAGTGGCCCAGGAAGAC  mus_spretus: 4:114720272

Mouse  mus_musculus: 4:118188096 CATTTCCAGGAATGGTTCAGACTGGACTCTAACCCAGCCTCGTCT mus_musculus: 4:118188140
Algerian mouse  mus_spretus: 4:114720273 CATTTCCAGGAATGGTTCAGACTGGACTCTAACTCAGCCTCGCCT  mus_spretus: 4:114720317

Mouse  mus_musculus: 4:118188141 CCTCTCTGCGGTGAGGACCAGGCAGGACACAGCAGACAGGGCACA mus_musculus: 4:118188185
Algerian mouse  mus_spretus: 4:114720318 CCTCTCTGCGGTGAGGACTGGGCAGGACACAGCAGACAGGGCACA  mus_spretus: 4:114720362

Mouse  mus_musculus: 4:118188186 TTACGGGGTCAGGATTCAGCCTTCTTGACTGACTGTCTCAGTGCC mus_musculus: 4:118188230
Algerian mouse  mus_spretus: 4:114720363 TTACGGGGTCAGGATTCAGCCTTCTTGACTGACTGTCTCAGTGCC  mus_spretus: 4:114720407

Mouse  mus_musculus: 4:118188231 TTCGAAAGCTGGTGTCTGTCAGCAGAGACCATGGAGCAGTGTGGT mus_musculus: 4:118188275
Algerian mouse  mus_spretus: 4:114720408 TTCAAAAGCTGGTGTCTGTCAGCAGAAACCATGGAGCAGTGTGGT  mus_spretus: 4:114720452

Mouse  mus_musculus: 4:118188276 CTAGGTTATACAGCCAGATGCCACACCTCAGGGCTAGCTTCTTTA mus_musculus: 4:118188320
Algerian mouse  mus_spretus: 4:114720453 CTAGGTTATACAGCCAGATGCCACACCTCAGGGCTAGCTTCTTTA  mus_spretus: 4:114720497

Mouse  mus_musculus: 4:118188321 AGCTGTCTCTCCAGGGAAGTCCTTCATCATGTTGGGTCTTAGCTG mus_musculus: 4:118188365
Algerian mouse  mus_spretus: 4:114720498 AGCTGTCTCTCCAGGGAAGTCCTTCGTCATGTTGGGTCTTAGCTG  mus_spretus: 4:114720542

Mouse  mus_musculus: 4:118188366 TCTTCCCTGTGCGGTATGAATCAAGTTCTGCATTCAAGCTATTGC mus_musculus: 4:118188410
Algerian mouse  mus_spretus: 4:114720543 TTTTCCCTGTGTGGTATGAATCAAGTTCTGCATTCAAGCTATTGC  mus_spretus: 4:114720587

Mouse  mus_musculus: 4:118188411 AATACAGAGGATTTCTAGAGATATGTGGAATAGTGAGGCATGTCC mus_musculus: 4:118188455
Algerian mouse  mus_spretus: 4:114720588 AATACAGAGGATTTCTAGAGATATGTGGAATAGTGAGGCATGTCC  mus_spretus: 4:114720632

Mouse  mus_musculus: 4:118188456 CTCACCATAAAAAAAAAAAAAATCCACAGAGTTAGGGCAAGCCAG mus_musculus: 4:118188500
Algerian mouse  mus_spretus: 4:114720633 CTCACCATTAAAAAAAAAAA‑‑TCCACAGAGTTAGGGCAAGCCAG  mus_spretus: 4:114720675

Mouse  mus_musculus: 4:118188501 TTGGCTCAGCCAGCGAAGGTGTTCTGAAGCCTAATGATGAAAGTT mus_musculus: 4:118188545
Algerian mouse  mus_spretus: 4:114720676 TTGGCTCAGCCAGCGAAGGTGTTCTGAAGCCTAATGATGAAAGTT  mus_spretus: 4:114720720

Mouse  mus_musculus: 4:118188546 CCGATGCTTGGAACTCATGATCGAAGCACTCCTAAAAGCTGCTCT mus_musculus: 4:118188590
Algerian mouse  mus_spretus: 4:114720721 CCGATGCTTAGAACTCATGATCGAAGCACTCCTAAAAGCTGCTCT  mus_spretus: 4:114720765

Mouse  mus_musculus: 4:118188591 CCACATGCATACCATGGCACATGCCAATTGGAATACACACACACA mus_musculus: 4:118188635
Algerian mouse  mus_spretus: 4:114720766 CCACATGCATACCATGGCACATGCCAATTGGAATACACACACACA  mus_spretus: 4:114720810

Mouse  mus_musculus: 4:118188636 CACACACACACACACACACACACACACACACACACACACACACTC mus_musculus: 4:118188680
Algerian mouse  mus_spretus: 4:114720811 CACACACACACACACACACACACACACACACACACACACAC‑‑TC  mus_spretus: 4:114720853

Mouse  mus_musculus: 4:118188681 CACAAGTAGTAAATAAAATAAAAACAAAAACGAAGGAACTCATTT mus_musculus: 4:118188725
Algerian mouse  mus_spretus: 4:114720854 CACAAGTAGTAAATAAAATAAAAACAAAAACGAAGAAACTCATTT  mus_spretus: 4:114720898

Mouse  mus_musculus: 4:118188726 AAAAGTAGCTGCCTAGACCCAGCCTCTCCTTGGCTACCTCTACAG mus_musculus: 4:118188770
Algerian mouse  mus_spretus: 4:114720899 AAAAGTAGCTGCCTAGACCCAGCCTCTCCTTGGCTACCTCTACAG  mus_spretus: 4:114720943

Mouse  mus_musculus: 4:118188771 CTCTGTGCCGGAGAAGCAGCATAAAGGTGGCTCTCTGTGGCTTTC mus_musculus: 4:118188815
Algerian mouse  mus_spretus: 4:114720944 CTCTGTGCCGGAGAAGCAGCATAAAGGTGGCTCTCTGTGGCTTTC  mus_spretus: 4:114720988

Mouse  mus_musculus: 4:118188816 CACGCTTGGGCTACACTGTTTCTACGACTGGACCTGCCGGAGGGA mus_musculus: 4:118188860
Algerian mouse  mus_spretus: 4:114720989 CACGATTGGGCTACCCTGTTTCTATGACTGGACCTGCCGGAGGGA  mus_spretus: 4:114721033

Mouse  mus_musculus: 4:118188861 AGCTCTTTCATGCCACAAAACAGTTTATCGTTGTTCTGTTTCTTG mus_musculus: 4:118188905
Algerian mouse  mus_spretus: 4:114721034 AGCTCTTTCATGCCACAAAACAGTTTATTGTTGTTCTGTTTCTTG  mus_spretus: 4:114721078

Mouse  mus_musculus: 4:118188906 AGCAGCTCGTCTGACCTTACACCTTCAGGGAGAAGCTTTCCCAGG mus_musculus: 4:118188950
Algerian mouse  mus_spretus: 4:114721079 AGCAGCTCGTCTGACCTTACACCTTCAAGGAGAAGCTTTCCCAGG  mus_spretus: 4:114721123

Mouse  mus_musculus: 4:118188951 ATCCCAATAGCCTTTGGACTAGGGAGCGATCTTGGGGTAAAAAGC mus_musculus: 4:118188995
Algerian mouse  mus_spretus: 4:114721124 ATCCCAATAGCCTTTGGACTAGGGAGCGATCTTGGGGTAAAAAGC  mus_spretus: 4:114721168

Mouse  mus_musculus: 4:118188996 AATTTGGGGTTAAGGAGGTATCTTGCACTGAGAAGCAACTTTAGT mus_musculus: 4:118189040
Algerian mouse  mus_spretus: 4:114721169 AATTTGGGGTTAAGGAGGTATCTTGCACTGAGAAGCAACTTTAGT  mus_spretus: 4:114721213

Mouse  mus_musculus: 4:118189041 CCGAGAGCTATTCTACTGGAAATGTTAGCTCTGCTCTGTAAACAT mus_musculus: 4:118189085
Algerian mouse  mus_spretus: 4:114721214 CCGAGAGCTATTCTACTGGAAATGTTAGCTCTGCTCTGTAAACAT  mus_spretus: 4:114721258

Mouse  mus_musculus: 4:118189086 GACACTCGAGCATTTCCATGCCCTGTTTCTTGGCCCAAGGGTTTT mus_musculus: 4:118189130
Algerian mouse  mus_spretus: 4:114721259 GACACTCGAGCATTTCCATGCCCTGTTTCTTGGCCCAAGGGTTTT  mus_spretus: 4:114721303

Mouse  mus_musculus: 4:118189131 GTTGTGGTTAATACTGTGGTCTGTCTATTTTATGCTTCATCTTCC mus_musculus: 4:118189175
Algerian mouse  mus_spretus: 4:114721304 GTTGTGGTTAATACTGTGGTCTGTCTATTTTATGCTTCATCTTCC  mus_spretus: 4:114721348

Mouse  mus_musculus: 4:118189176 TGGAAACAGTAAAATGTTTAGCTGTCACACATTCTTGGCTCGTGC mus_musculus: 4:118189220
Algerian mouse  mus_spretus: 4:114721349 TGGAAACAGTAAAATGTTTAGCTGTCACACATTCTTGGCTCGCGC  mus_spretus: 4:114721393

Mouse  mus_musculus: 4:118189221 TTTGCCTTGGGCCAGCTGTGTCACTGTGAGCAACACAATGGAATC mus_musculus: 4:118189265
Algerian mouse  mus_spretus: 4:114721394 TTTGCCTTGGGCCAGCTGTGTCACTGTGAGCAACACAATGGAATC  mus_spretus: 4:114721438

Mouse  mus_musculus: 4:118189266 TCAGGACTTTAGACCAGAGAGAACCAATACAATCCCTTCACTGAA mus_musculus: 4:118189310
Algerian mouse  mus_spretus: 4:114721439 TCAGGACTTTAGACCAGAGAGAACCAATACAACCCCTTCACTGAA  mus_spretus: 4:114721483

Mouse  mus_musculus: 4:118189311 GGGAAATGAGGCATTGAATGAGGTGAGGAAGAGAGATTGGACCAC mus_musculus: 4:118189355
Algerian mouse  mus_spretus: 4:114721484 GGGAAATGAGGCATTGAATGAGGTGAGGAAGAGAGATTGGACCAC  mus_spretus: 4:114721528

Mouse  mus_musculus: 4:118189356 AGAAATGTCAAAGTGGCCCTTTTCAAGGTCATCTCTGTTCGCCTG mus_musculus: 4:118189400
Algerian mouse  mus_spretus: 4:114721529 AGAAATGTCAAAGTGGCCCTTTTCAAGGTCATCTCTGTTCGCCTG  mus_spretus: 4:114721573

Mouse  mus_musculus: 4:118189401 TCTCTATAGAATCTGCCTCTCCAGTGTGTAAAACAAACCTTGGAG mus_musculus: 4:118189445
Algerian mouse  mus_spretus: 4:114721574 TCTCTATAGAATCTGCCTCTCCAGGGTGTAAAACAAACCTTGGAG  mus_spretus: 4:114721618

Mouse  mus_musculus: 4:118189446 TAGGTAGCCACTGGATATCCTTCCATGCACAAGTTTCACAGCTAT mus_musculus: 4:118189490
Algerian mouse  mus_spretus: 4:114721619 TAGGTAGCCACTGGATATCCTTCCATGCACAAATTTCACAGCTAT  mus_spretus: 4:114721663

Mouse  mus_musculus: 4:118189491 TTCTCTGTGTGTAAATCAGCTGTGTCATTAAATTCTTTATAGAGA mus_musculus: 4:118189535
Algerian mouse  mus_spretus: 4:114721664 TTCTCTATGTGTAAATCAGCTGTATCATTAAATTCTTTATAGAGA  mus_spretus: 4:114721708

Mouse  mus_musculus: 4:118189536 GTCCCCCGGTCAAGT  mus_musculus: 4:118189550
Algerian mouse  mus_spretus: 4:114721709 GTCCCCTGGTCAAGT  mus_spretus: 4:114721723
