# Supplementary material for: Recurrent erosion of COA1/MITRAC15 exemplifies conditional gene dispensability in oxidative phosphorylation
Source: Sci Rep. 2021 Dec 24;11:24437. doi: 10.1038/s41598-021-04077-y (PMC8709867; doi:10.1038/s41598-021-04077-y)
Supplement: Supplementary file 3 — Supplementary Information 3. [file 41598_2021_4077_MOESM3_ESM.zip › Supplementary Dataset/COA1_GENE-main_Github_use_winRAR_to_open.zip!/COA1_GENE/supplementary_figure_data/Pairwise_genome_alignment/Pairwise_alignment_from_ensembl/Mouse_Northern_American_deer_mouse_lastz.odt]

Mouse  mus_musculus: 4:118187691 CCTTCCCTCCCTTCTTTCCTTCCTTCTTCCTCTTTCTTTTTTGTT  mus_musculus: 4:118187735
Northern American deer mouse peromyscus_maniculatus_bairdii: 2:133766715 TCTT‑‑‑‑‑‑‑‑‑‑‑‑‑‑‑‑‑‑‑‑‑‑‑TCCTTTGTCTCCTTTGTT peromyscus_maniculatus_bairdii: 2:133766736

Mouse  mus_musculus: 4:118187736 GTACTGGCAACTAAATCCGGGGAACTCATGCATGCTGGCTAAG‑G  mus_musculus: 4:118187779
Northern American deer mouse peromyscus_maniculatus_bairdii: 2:133766737 GTACCCCTAGTTGAACCCAGGGC‑CTCATGCATGCTGGCCTGGTG peromyscus_maniculatus_bairdii: 2:133766780

Mouse  mus_musculus: 4:118187780 CTCTACCACCGAGCTAAGAACCAAGGCCTTTTTCAGAGATTTCCA  mus_musculus: 4:118187824
Northern American deer mouse peromyscus_maniculatus_bairdii: 2:133766781 CTCTACCACTGAGTTAGGACCCCAGCTCTCTTCCACTGATTTTCA peromyscus_maniculatus_bairdii: 2:133766825

Mouse  mus_musculus: 4:118187825 AAATACTTTTGTTACAATGTGACAAAGATCTCTACGAAGTCCTTT  mus_musculus: 4:118187869
Northern American deer mouse peromyscus_maniculatus_bairdii: 2:133766826 AGACCGTTTTGCTACAAAGTGGTAAAGGTCTCTAAGAAGCCATAT peromyscus_maniculatus_bairdii: 2:133766870

Mouse  mus_musculus: 4:118187870 GTCGGGGCCTCTTGGAATACTGTTCCTTTATCCCAGTGTATTGGA  mus_musculus: 4:118187914
Northern American deer mouse peromyscus_maniculatus_bairdii: 2:133766871 GCCAGGACCTCTGGGAGTGCTGTTCCTTCATTCCAGTGTGTTGAT peromyscus_maniculatus_bairdii: 2:133766915

Mouse  mus_musculus: 4:118187915 GGAAGGGTGGGAGAGGTGGAGGATAGGGGATAGGGGTGATGGTGG  mus_musculus: 4:118187959
Northern American deer mouse peromyscus_maniculatus_bairdii: 2:133766916 GGCAGGG‑‑‑‑‑‑‑‑‑‑‑‑‑‑‑‑‑‑‑‑‑‑‑‑‑‑‑‑‑‑‑‑‑‑‑‑‑‑ peromyscus_maniculatus_bairdii: 2:133766922

Mouse  mus_musculus: 4:118187960 AGAGTGCTCCTTCTCTTGTTACTTTAAGGAGAGCATAAGTGTGTC  mus_musculus: 4:118188004
Northern American deer mouse peromyscus_maniculatus_bairdii: 2:133766923 ‑GCCTGCTTCTTTTCCTGTTACTTTCAGAAGAGCCTAAGTGTGTC peromyscus_maniculatus_bairdii: 2:133766966

Mouse  mus_musculus: 4:118188005 ATTCAGCAGACAGACAAACAGCCAACCGTCCTCTCTTCTCACTTT  mus_musculus: 4:118188049
Northern American deer mouse peromyscus_maniculatus_bairdii: 2:133766967 GC‑CAGCAGACAGA‑‑‑‑‑‑GCCAATGATCCTCTCCTCTCATTTT peromyscus_maniculatus_bairdii: 2:133767004

Mouse  mus_musculus: 4:118188050 CCTGTAGAAAGTTGAGTCCCTTGAATCTGGAGTGGCCCAGGAGGA  mus_musculus: 4:118188094
Northern American deer mouse peromyscus_maniculatus_bairdii: 2:133767005 CCTGTAGGAAGCTGAGTCCCTTGAGTCTGGAGTG‑‑‑‑‑‑‑‑‑‑‑ peromyscus_maniculatus_bairdii: 2:133767038

Mouse  mus_musculus: 4:118188095 CCATTTCCAGGAATGGTTCAGACTGGACTCTAACCCAGCCTCGTC  mus_musculus: 4:118188139
Northern American deer mouse peromyscus_maniculatus_bairdii: 2:133767039 ‑‑‑‑‑‑‑‑‑‑‑‑‑‑‑‑‑‑‑‑‑ACTGGA‑‑‑‑‑‑‑‑‑‑‑‑‑‑‑‑‑‑ peromyscus_maniculatus_bairdii: 2:133767044

Mouse  mus_musculus: 4:118188140 TCCTCTCTGCGGTGAGGACCAGGCAGGACACAGCAGACA‑GGGCA  mus_musculus: 4:118188183
Northern American deer mouse peromyscus_maniculatus_bairdii: 2:133767045 ‑‑‑‑‑‑‑‑‑‑GAAGAGGACCATGAAGGACATAACAGAAACAGGCA peromyscus_maniculatus_bairdii: 2:133767079

Mouse  mus_musculus: 4:118188184 CATTACGGGGTCAGGATTCAGCCTTCTTGACTGACTGTCTCAGTG  mus_musculus: 4:118188228
Northern American deer mouse peromyscus_maniculatus_bairdii: 2:133767080 CATTTGGGGGTCAGGATTCAGCCTTCTTGATTGATGGCCTCTGTG peromyscus_maniculatus_bairdii: 2:133767124

Mouse  mus_musculus: 4:118188229 CCTTCGAAAGCTGGTGTCTGTCAGCAGAGACCATGGAG‑CAGTGT  mus_musculus: 4:118188272
Northern American deer mouse peromyscus_maniculatus_bairdii: 2:133767125 CCTCAGGAAGCTGGTGTCATCTAGCAGAGGCCATCAAGGCAGCGT peromyscus_maniculatus_bairdii: 2:133767169

Mouse  mus_musculus: 4:118188273 GGTCTAGGTTATACAGCCAGATGCCACACCTCAGGGCTAGCTTCT  mus_musculus: 4:118188317
Northern American deer mouse peromyscus_maniculatus_bairdii: 2:133767170 CATCTAGACTGTGTAGCCCTCT‑TCAGACCTCAGGTCAGACTTTT peromyscus_maniculatus_bairdii: 2:133767213

Mouse  mus_musculus: 4:118188318 TTAAGCTGTCTCTCCAGGGAAGTCCTTCATCATGTTGGGTCTTAG  mus_musculus: 4:118188362
Northern American deer mouse peromyscus_maniculatus_bairdii: 2:133767214 CTA‑GCTGTCTCTACAGGGGAGTCCTTCATCGTGCTGGGTCCTGG peromyscus_maniculatus_bairdii: 2:133767257

Mouse  mus_musculus: 4:118188363 CTGTCTTCCCTGTGCGGTATGAA‑‑‑‑‑‑‑‑‑‑TCAAGTTCTG‑‑  mus_musculus: 4:118188395
Northern American deer mouse peromyscus_maniculatus_bairdii: 2:133767258 CTGCCTTCCCTGTGTGATATGAAGTTGTGAGAATCAAGCTGTAGG peromyscus_maniculatus_bairdii: 2:133767302

Mouse  mus_musculus: 4:118188396 ‑CATTCAAGCTATTGCAATACAGAGGATTTCTAGAGATATGTGGA  mus_musculus: 4:118188439
Northern American deer mouse peromyscus_maniculatus_bairdii: 2:133767303 ACATTCAAGCTATTATA‑TATTGAGGATTTCAAGAGATATGTGAA peromyscus_maniculatus_bairdii: 2:133767346

Mouse  mus_musculus: 4:118188440 ATAGTGAGGCATGTCCCTCACCATAAAAAAAAAAAAAATCCACAG  mus_musculus: 4:118188484
Northern American deer mouse peromyscus_maniculatus_bairdii: 2:133767347 ATAGCCAGGCATGAACCTTGCCATAAAAA‑‑‑‑‑‑‑‑‑‑CTACTG peromyscus_maniculatus_bairdii: 2:133767381

Mouse  mus_musculus: 4:118188485 AGTTAGGGCAAGCCAGTTGGCTCAGCCAGCGAAGGTGTTCTGAAG  mus_musculus: 4:118188529
Northern American deer mouse peromyscus_maniculatus_bairdii: 2:133767382 AGTTAGGGCAAGCCAGAAGGCTCAGCCAGTGAAGGTGTTCCGAAG peromyscus_maniculatus_bairdii: 2:133767426

Mouse  mus_musculus: 4:118188530 CCTAATGATGAAAGTTCCGATGCTTGGAACTCATGATCGAAGCAC  mus_musculus: 4:118188574
Northern American deer mouse peromyscus_maniculatus_bairdii: 2:133767427 CCTGTTAACCAAAGTTCAGGTGCCTGGAACNNNNNNNNNNNNNNN peromyscus_maniculatus_bairdii: 2:133767558

Mouse  mus_musculus: 4:118188575 TCCTAAAAGCTGCTCTCCACATGCATACCATGGCACATGCCAATT  mus_musculus: 4:118188619
Northern American deer mouse peromyscus_maniculatus_bairdii: 2:133767559 NNNNNNNNNNNNNNNNNNNNNNNNNNNNNNNNNNNNNNNNNNNNN peromyscus_maniculatus_bairdii: 2:133767603

Mouse  mus_musculus: 4:118188620 GGAATACACACACACACACACACACACACACACACACACACACAC  mus_musculus: 4:118188664
Northern American deer mouse peromyscus_maniculatus_bairdii: 2:133767604 NNNNNNNNNNNNNNNNNNNNNNNNNNNNNNNNNNNNNNNNNNNNN peromyscus_maniculatus_bairdii: 2:133767648

Mouse  mus_musculus: 4:118188665 ACACACACACACACTCCACAAGTAGTAAATAAAATAAAAACAAAA  mus_musculus: 4:118188709
Northern American deer mouse peromyscus_maniculatus_bairdii: 2:133767649 NNNNNNNNNNNNNNNNNNNNNNNAATAAAGAAAAGAAAAAGAAAT peromyscus_maniculatus_bairdii: 2:133767693

Mouse  mus_musculus: 4:118188710 ACGAAGGAACTCATTTAAAAGTAGCTGCCTAGACCCAGCCTCTCC  mus_musculus: 4:118188754
Northern American deer mouse peromyscus_maniculatus_bairdii: 2:133767694 ACAAAGGAATTCATCT‑‑‑‑GTAGCTGTCTAGACTCAGCCTCTCC peromyscus_maniculatus_bairdii: 2:133767734

Mouse  mus_musculus: 4:118188755 TTGGCTACCTCTACAGCTCTGTGCCGGAGAAGCAGCATAAAGGTG  mus_musculus: 4:118188799
Northern American deer mouse peromyscus_maniculatus_bairdii: 2:133767735 TTGGCCACACCTACAGCTCTATGGTCCCGAAGCAGCTTAAGCGTG peromyscus_maniculatus_bairdii: 2:133767779

Mouse  mus_musculus: 4:118188800 GCTCTCTGTGGCTTTCCACGCTTGGGCTACACTGTTTCTACGACT  mus_musculus: 4:118188844
Northern American deer mouse peromyscus_maniculatus_bairdii: 2:133767780 TCTCTCTGTGGCTCTCCAGGCTTGGGTCATCTTGTTCCCAC‑ATT peromyscus_maniculatus_bairdii: 2:133767823

Mouse  mus_musculus: 4:118188845 GGACCTGCCGGAGGGAAGCTCTTTC‑ATGCCAC‑‑AAAACAGTTT  mus_musculus: 4:118188886
Northern American deer mouse peromyscus_maniculatus_bairdii: 2:133767824 GGACCTGA‑GGATGGCAGCTCTTTTTGTGCTATGGAACACAGTTT peromyscus_maniculatus_bairdii: 2:133767867

Mouse  mus_musculus: 4:118188887 ATCGTTGTTCTGTTTCTTGAGCAGCTCGTCTGACCTTACACCTTC  mus_musculus: 4:118188931
Northern American deer mouse peromyscus_maniculatus_bairdii: 2:133767868 ATCTTTGCTCTGCTTCCCGAGCAGCTTGTCAGTCCTTACACCTTC peromyscus_maniculatus_bairdii: 2:133767912

Mouse  mus_musculus: 4:118188932 AGGGAGAAGCTTTCCCAGGATCCCAATAGCCTTTGGACTAGGGAG  mus_musculus: 4:118188976
Northern American deer mouse peromyscus_maniculatus_bairdii: 2:133767913 AGGGAGAAGCGTCTCCAGGATTCCAGGAGCCTTTAGGCTAAGAAG peromyscus_maniculatus_bairdii: 2:133767957

Mouse  mus_musculus: 4:118188977 C‑‑‑‑‑‑‑‑GATCTTGGGGTAAAAAGCAATTTGGGGTTAAGGAGG  mus_musculus: 4:118189013
Northern American deer mouse peromyscus_maniculatus_bairdii: 2:133767958 CAAGGGACAGATCTTGGGGTAAAAA‑CAATCTCGGGCTCAGAAGG peromyscus_maniculatus_bairdii: 2:133768001

Mouse  mus_musculus: 4:118189014 TATCTTGCACTGAGAAGCAACTTTAGTCCGAGAGCTATTCTACTG  mus_musculus: 4:118189058
Northern American deer mouse peromyscus_maniculatus_bairdii: 2:133768002 AATCTT‑‑‑‑‑‑‑‑‑‑‑‑‑‑‑‑‑‑AGGTTGAGAGCTGTTCTACTG peromyscus_maniculatus_bairdii: 2:133768028

Mouse  mus_musculus: 4:118189059 GAAATGTTAGCTCTGCTCTGTAAACATGACACTCGAGCATTTCCA  mus_musculus: 4:118189103
Northern American deer mouse peromyscus_maniculatus_bairdii: 2:133768029 GAAATGTTAGCTCAGATCTGTAAACGTGACACGAGAGCATTTCCA peromyscus_maniculatus_bairdii: 2:133768073

Mouse  mus_musculus: 4:118189104 TGCCCTGTTTCTTGGCCCAAGGGTTTTGTTGTGGTTAATACTGTG  mus_musculus: 4:118189148
Northern American deer mouse peromyscus_maniculatus_bairdii: 2:133768074 CGCCCTGTTTCTTGGCCCAAGGGTTTTGTTGTGGTTAATACTGTG peromyscus_maniculatus_bairdii: 2:133768118

Mouse  mus_musculus: 4:118189149 GTCTGTCTATTTTATGCTTCATCTTCCTGGAAACAGTAAAATGTT  mus_musculus: 4:118189193
Northern American deer mouse peromyscus_maniculatus_bairdii: 2:133768119 GTCTGTCTATTTTATGCTCCATCTTCTCGGAAACAGTAAAATGTT peromyscus_maniculatus_bairdii: 2:133768163

Mouse  mus_musculus: 4:118189194 TAGCTGTCACACATTCTTGGCTCGTGCTTTGCCTTGGGCCAGCTG  mus_musculus: 4:118189238
Northern American deer mouse peromyscus_maniculatus_bairdii: 2:133768164 TAGCTGTCACACATTCCTGGCTCTCGCTTTGCCTTGGGCCAGCTG peromyscus_maniculatus_bairdii: 2:133768208

Mouse  mus_musculus: 4:118189239 TGTCACTGTGAGCAACACAATGGAATCTCAGGACTTTAGACCAGA  mus_musculus: 4:118189283
Northern American deer mouse peromyscus_maniculatus_bairdii: 2:133768209 TGTCACTTTGAGCCACACGATGGAATCACGGGACTTTAGACCAGG peromyscus_maniculatus_bairdii: 2:133768253

Mouse  mus_musculus: 4:118189284 GAGAACCAATACAATCCCTTCACTGAAGGGAAATGAGGCATTGAA  mus_musculus: 4:118189328
Northern American deer mouse peromyscus_maniculatus_bairdii: 2:133768254 GAGAGCTGATCCAACCCCTTCACTGAAGGGAAATGAGGCGGTGAA peromyscus_maniculatus_bairdii: 2:133768298

Mouse  mus_musculus: 4:118189329 TGAGGTGAGG‑‑AAGAGAGATTGGACCACAGAAATGTCAAAGTGG  mus_musculus: 4:118189371
Northern American deer mouse peromyscus_maniculatus_bairdii: 2:133768299 TGAGGTGAGGAAAAGGGAGATTGAACCACAGAAATGCCAAGATAG peromyscus_maniculatus_bairdii: 2:133768343

Mouse  mus_musculus: 4:118189372 CCCTTTTCAAGGTCATCTCTGTTCGCCTGTCTCTATAGAATCTGC  mus_musculus: 4:118189416
Northern American deer mouse peromyscus_maniculatus_bairdii: 2:133768344 CCCTTTTCAAGGTCATCTGTGTTC‑‑‑‑ATCTCTGTGGAATCTGC peromyscus_maniculatus_bairdii: 2:133768384

Mouse  mus_musculus: 4:118189417 CTCTCCAGTGTGTAAAACAAACCTTGGAGTAGGTAGCCACTGGAT  mus_musculus: 4:118189461
Northern American deer mouse peromyscus_maniculatus_bairdii: 2:133768385 CATTCCAGTGTGT‑AAACAGACCTGAGAGTGGAGGGCCACTGATC peromyscus_maniculatus_bairdii: 2:133768428

Mouse  mus_musculus: 4:118189462 ATCCTTCCATGCACAAG‑TTTCACAGCTATTTCTCTGTGTGTAAA  mus_musculus: 4:118189505
Northern American deer mouse peromyscus_maniculatus_bairdii: 2:133768429 ATTCTTCCGCACCCATGTTTTCACAGCTTTTTTTCTACGTGTAAA peromyscus_maniculatus_bairdii: 2:133768473

Mouse  mus_musculus: 4:118189506 TCAGCTGTGTCATTAAATTCTTTATAGAGAGTCCCCC‑GGTCAAG  mus_musculus: 4:118189549
Northern American deer mouse peromyscus_maniculatus_bairdii: 2:133768474 TCAACTGAGTTATTTAACTCTTTATGGAGATTCCCCCTGGACAAG peromyscus_maniculatus_bairdii: 2:133768518

Mouse  mus_musculus: 4:118189550 T  mus_musculus: 4:118189550
Northern American deer mouse peromyscus_maniculatus_bairdii: 2:133768519 T  peromyscus_maniculatus_bairdii: 2:133768519
