# Supplementary material for: Recurrent erosion of COA1/MITRAC15 exemplifies conditional gene dispensability in oxidative phosphorylation
Source: Sci Rep. 2021 Dec 24;11:24437. doi: 10.1038/s41598-021-04077-y (PMC8709867; doi:10.1038/s41598-021-04077-y)
Supplement: Supplementary file 3 — Supplementary Information 3. [file 41598_2021_4077_MOESM3_ESM.zip › Supplementary Dataset/COA1_GENE-main_Github_use_winRAR_to_open.zip!/COA1_GENE/supplementary_figure_data/Pairwise_genome_alignment/Pairwise_alignment_from_ensembl/Mouse_Ryukyu_mouse_lastz.odt]

Mouse  mus_musculus: 4:118187691 CCTTCCCTCCCTTCTTTCCTTCCTTCTTCCTCTTTCTTTTTTGTT mus_musculus: 4:118187735
Ryukyu mouse  mus_caroli: 4:108773418 CCTTCCCTTCCTTCCTTCCTTCCTTCTTCCTCTTTCTTTTTTGTT  mus_caroli: 4:108773462

Mouse  mus_musculus: 4:118187736 GTACTGGCAACTAAATCCGGGGAACTCATGCATGCTGGCTAAGGC mus_musculus: 4:118187780
Ryukyu mouse  mus_caroli: 4:108773463 GTACTGGCAACTAAATCCAGGGAACTCATGCATGCTGGCTAAGGC  mus_caroli: 4:108773507

Mouse  mus_musculus: 4:118187781 TCTACCACCGAGCTAAGAACCAAGGCCTTTTTCAGAGATTTCCAA mus_musculus: 4:118187825
Ryukyu mouse  mus_caroli: 4:108773508 TCTACCACTGAGCTAAGACCCAAGCCTTTTTTCAGAGATTTCCAC  mus_caroli: 4:108773552

Mouse  mus_musculus: 4:118187826 AATACTTTTGTTACAATGTGACAAAGATCTCTACGAAGTCCTTTG mus_musculus: 4:118187870
Ryukyu mouse  mus_caroli: 4:108773553 CATACTTTTGTTACAATGTGACAAAGATCTCTACCAAGTCCTTTG  mus_caroli: 4:108773597

Mouse  mus_musculus: 4:118187871 TCGGGGCCTCTTGGAATACTGTTCCTTTATCCCAGTGTATTGGAG mus_musculus: 4:118187915
Ryukyu mouse  mus_caroli: 4:108773598 TCGGGGCCTCTTGGAATACTGTTCCTTTATTCCAGTGTATTGGAG  mus_caroli: 4:108773642

Mouse  mus_musculus: 4:118187916 GAAGGGTGGGAGAGGTGGAGGATAGGGGATAGGGGTGATGGTGGA mus_musculus: 4:118187960
Ryukyu mouse  mus_caroli: 4:108773643 GAGGGGTGGGAGAGGTGGAGGATAGGGGATGGGGGGGATGGTGGA  mus_caroli: 4:108773687

Mouse  mus_musculus: 4:118187961 GAGTGCTCCTTCTCTTGTTACTTTAAGGAGAGCATAAGTGTGTCA mus_musculus: 4:118188005
Ryukyu mouse  mus_caroli: 4:108773688 GAGGGCTCCTTCTCTTGTTACTTTAAGGAGAGCATAAATGTGTCA  mus_caroli: 4:108773732

Mouse  mus_musculus: 4:118188006 TTCAGCAGACAGACAAACAGCCAACCGTCCTCTCTTCTCACTTTC mus_musculus: 4:118188050
Ryukyu mouse  mus_caroli: 4:108773733 TTCAGCAGACAGACAAACAGCCAACGGTCCTCTCTTCTCACTTTC  mus_caroli: 4:108773777

Mouse  mus_musculus: 4:118188051 CTGTAGAAAGTTGAGTCCCTTGAATCTGGAGTGGCCCAGGAGGAC mus_musculus: 4:118188095
Ryukyu mouse  mus_caroli: 4:108773778 CTGTAGAAAGCTGAGTCCCTTGAATCTGGAGTGGCCCAGGAAGAC  mus_caroli: 4:108773822

Mouse  mus_musculus: 4:118188096 CATTTCCAGGAATGGTTCAGACTGGACTCTAACCCAGCCTCGTCT mus_musculus: 4:118188140
Ryukyu mouse  mus_caroli: 4:108773823 CATTTCCAGGAATGGTTCAGACTGGACTCTAACCCAGCCTCACCT  mus_caroli: 4:108773867

Mouse  mus_musculus: 4:118188141 CCTCTCTGCGGTGAGGACCAGGCAGGACACAGCAGACAGGGCACA mus_musculus: 4:118188185
Ryukyu mouse  mus_caroli: 4:108773868 CCTCTCTGCCGTGAGGACCGAGCAGGACACAGCAGACAGGGCACC  mus_caroli: 4:108773912

Mouse  mus_musculus: 4:118188186 TTACGGGGTCAGGATTCAGCCTTCTTGACTGACTGTCTCAGTGCC mus_musculus: 4:118188230
Ryukyu mouse  mus_caroli: 4:108773913 TTACGGGGTCAGGATTCAGCCTTCTTGACTGACTGTCTCAGTGCC  mus_caroli: 4:108774034

Mouse  mus_musculus: 4:118188231 TTCGAAAGCTGGTGTCTGTCAGCAGAGACCATGGAGCAGTGTGGT mus_musculus: 4:118188275
Ryukyu mouse  mus_caroli: 4:108774035 TTCAAAAGCTAGTGTCTGTCAGCAGAGGCCATGGAGCAGTATGGT  mus_caroli: 4:108774079

Mouse  mus_musculus: 4:118188276 CTAGGTTATACAGCCAGATGCCACACCTCAGGGCTAGCTTCTTTA mus_musculus: 4:118188320
Ryukyu mouse  mus_caroli: 4:108774080 CTAGGTTATACAGCCAGATGCCACACCTCAGGGCTAGCTTCTTTA  mus_caroli: 4:108774124

Mouse  mus_musculus: 4:118188321 AGCTGTCTCTCCAGGGAAGTCCTTCATCATGTTGGGTCTTAGCTG mus_musculus: 4:118188365
Ryukyu mouse  mus_caroli: 4:108774125 AGCTGTCTCTCCAGGGAAGTCCTTCATCATGTTGGGCCTTAGCTG  mus_caroli: 4:108774169

Mouse  mus_musculus: 4:118188366 TCTTCCCTGTGCGGTATGAA‑‑‑‑‑‑‑‑‑‑‑‑‑TCAAGTTCTGCA mus_musculus: 4:118188397
Ryukyu mouse  mus_caroli: 4:108774170 TCTTCCCTGTGAGGTATGAAGCTGTTGTGAGAATCAAGTTCTGCA  mus_caroli: 4:108774214

Mouse  mus_musculus: 4:118188398 TTCAAGCTATTGCAATACAGAGGATTTCTAGAGATATGTGGAATA mus_musculus: 4:118188442
Ryukyu mouse  mus_caroli: 4:108774215 TTCAAGCTATTGCAATACAGAGGATTTCTAGAGATGTGTGGAATA  mus_caroli: 4:108774259

Mouse  mus_musculus: 4:118188443 GTGAGGCATGTCCCTCACCATAAAAAAAAAAAAAATCCACAGAGT mus_musculus: 4:118188487
Ryukyu mouse  mus_caroli: 4:108774260 GTGAGGCATGTCCCTCACCATAAAAAAAAAAAAAATCCTCAGAGT  mus_caroli: 4:108774304

Mouse  mus_musculus: 4:118188488 TAGGGCAAGCCAGTTGGCTCAGCCAGCGAAGGTGTTCTGAAGCCT mus_musculus: 4:118188532
Ryukyu mouse  mus_caroli: 4:108774305 TAGGGCAAGCCAGTTGGCTCAGCCAGCCAAGGTGTTCTGAAGCCT  mus_caroli: 4:108774349

Mouse  mus_musculus: 4:118188533 AATGATGAAAGTTCCGATGCTTGGAACTCATGATCGAAGCACTCC mus_musculus: 4:118188577
Ryukyu mouse  mus_caroli: 4:108774350 AATGATGAAAGTTCAGATGCTTGGAACTCATGATCGACGCACTCC  mus_caroli: 4:108774594

Mouse  mus_musculus: 4:118188578 TAAAAGCTGCTCTCCACATGCATACCATGGCACATGCCAATTGGA mus_musculus: 4:118188622
Ryukyu mouse  mus_caroli: 4:108774595 TAAAAGCTGTTCTTCACATGCATACCATGGCACGTGCCAATTGGA  mus_caroli: 4:108774639

Mouse  mus_musculus: 4:118188623 ATACACACACACACACACACACACACACACACACACACACACACA mus_musculus: 4:118188667
Ryukyu mouse  mus_caroli: 4:108774640 A‑‑‑‑‑‑‑‑‑‑‑‑‑‑‑‑‑‑‑‑‑‑‑‑‑‑‑‑‑‑‑‑‑‑‑‑‑‑‑‑‑‑CA  mus_caroli: 4:108774642

Mouse  mus_musculus: 4:118188668 CACACACACACTCCACAAGTAGTAAATAAAATAAAAACAAAAACG mus_musculus: 4:118188712
Ryukyu mouse  mus_caroli: 4:108774643 CACACACACACTCCACAAGTAGTAAATAAAATAAAAACAAAAATG  mus_caroli: 4:108774687

Mouse  mus_musculus: 4:118188713 AAGGAACTCATTTAAAAGTAGCTGCCTAGACCCAGCCTCTCCTTG mus_musculus: 4:118188757
Ryukyu mouse  mus_caroli: 4:108774688 AAGGAACTCATTTAAAAGTAGCTGCCTAGACCCAGCCTCTCCTTG  mus_caroli: 4:108774732

Mouse  mus_musculus: 4:118188758 GCTACCTCTACAGC‑‑‑‑‑‑‑‑‑‑‑TCTGTGCCGGAGAAGCAGCA mus_musculus: 4:118188791
Ryukyu mouse  mus_caroli: 4:108774733 ACTACCCCTACAGCACCCCTACAGCTCTGTGCCGGAGAAGCAGCA  mus_caroli: 4:108774777

Mouse  mus_musculus: 4:118188792 TAAAGGTGGCTCTCTGTGGCTTTCCACGCTTGGGCTACACTGTTT mus_musculus: 4:118188836
Ryukyu mouse  mus_caroli: 4:108774778 TAAAGGTGGCTCTCTGTGGCTTTCCACACTTGGGCTACCCTGTTT  mus_caroli: 4:108774822

Mouse  mus_musculus: 4:118188837 CTACGACTGGACCTGCCGGAGGGAAGCTCTTTCATGCCACAAAAC mus_musculus: 4:118188881
Ryukyu mouse  mus_caroli: 4:108774823 CTACGACTGGACCTGCCGGAGGGAAGCTCTTTCATGCCACAAAAC  mus_caroli: 4:108774867

Mouse  mus_musculus: 4:118188882 AGTTTATCGTTGTTCTGTTTCTTGAGCAGCTCGTCTGACCTTACA mus_musculus: 4:118188926
Ryukyu mouse  mus_caroli: 4:108774868 AGTTTATCGTTGTTCTGTTTCTTGAGCAGCTTCTCTGACCTTACA  mus_caroli: 4:108774912

Mouse  mus_musculus: 4:118188927 CCTTCAGGGAGAAGCTTTCCCAGGATCCCAATAGCCTTTGGACTA mus_musculus: 4:118188971
Ryukyu mouse  mus_caroli: 4:108774913 CCTTCAGGGAGAAGCTTTCCCAGGATCCCAATAGCCTTTGGACTA  mus_caroli: 4:108774957

Mouse  mus_musculus: 4:118188972 GGGAGCGATCTTGGGGTAAAAAGCAATTTGGGGTTAAGGAGGTAT mus_musculus: 4:118189016
Ryukyu mouse  mus_caroli: 4:108774958 GGGAGCGATCTTGGGGTAAAAAGCAATTTTGGGTTAAGGAGGTAT  mus_caroli: 4:108775002

Mouse  mus_musculus: 4:118189017 CTTGCACTGAGAAGCAACTTTAGTCCGAGAGCTATTCTACTGGAA mus_musculus: 4:118189061
Ryukyu mouse  mus_caroli: 4:108775003 CTTGCACTGAGAAGCAACTTTAGTCCGAGAGCTATTCTACTGGAA  mus_caroli: 4:108775047

Mouse  mus_musculus: 4:118189062 ATGTTAGCTCTGCTCTGTAAACATGACACTCGAGCATTTCCATGC mus_musculus: 4:118189106
Ryukyu mouse  mus_caroli: 4:108775048 ATGTTAGCTCTGCTCTGTAAACATGACACTCGAGCATTTCCATGC  mus_caroli: 4:108775092

Mouse  mus_musculus: 4:118189107 CCTGTTTCTTGGCCCAAGGGTTTTGTTGTGGTTAATACTGTGGTC mus_musculus: 4:118189151
Ryukyu mouse  mus_caroli: 4:108775093 CCTGTTTCTTGGCCCAAGGGTTTTGTTGTGGTTAATACTGTGGTC  mus_caroli: 4:108775137

Mouse  mus_musculus: 4:118189152 TGTCTATTTTATGCTTCATCTTCCTGGAAACAGTAAAATGTTTAG mus_musculus: 4:118189196
Ryukyu mouse  mus_caroli: 4:108775138 TGTCTATTTTATGCTTCATCTTCCCGGAAACAGTAAAATGTTTAG  mus_caroli: 4:108775182

Mouse  mus_musculus: 4:118189197 CTGTCACACATTCTTGGCTCGTGCTTTGCCTTGGGCCAGCTGTGT mus_musculus: 4:118189241
Ryukyu mouse  mus_caroli: 4:108775183 CTGTCACACATTCTTGGCTCGCGCTTTGCCCTGGGCCAGCTGTGT  mus_caroli: 4:108775227

Mouse  mus_musculus: 4:118189242 CACTGTGAGCAACACAATGGAATCTCAGGACTTTAGACCAGAGAG mus_musculus: 4:118189286
Ryukyu mouse  mus_caroli: 4:108775228 CACTGTGAGCAACACAATGGGATCTCAGGACTTTAGACCAGAGAG  mus_caroli: 4:108775272

Mouse  mus_musculus: 4:118189287 AACCAATACAA‑TCCCTTCACTGAAGGGAAATGAGGCATTGAATG mus_musculus: 4:118189330
Ryukyu mouse  mus_caroli: 4:108775273 AACCGATACAAACCCCTTCACTGAAGGGAAATGAGGCATTGAATG  mus_caroli: 4:108775317

Mouse  mus_musculus: 4:118189331 AGGTGAGGAAGAGAGATTGGACCACAGAAATGTCAAAGTGGCCCT mus_musculus: 4:118189375
Ryukyu mouse  mus_caroli: 4:108775318 AGGTGAGGAAGAGAGATTGGACCACAGAAATGTCAAAGTGGCCCT  mus_caroli: 4:108775362

Mouse  mus_musculus: 4:118189376 TTTCAAGGTCATCTCTGTTCGCCTGTCTCTATAGAATCTGCCTCT mus_musculus: 4:118189420
Ryukyu mouse  mus_caroli: 4:108775363 TTTCAAGGTCATCTCTGTTTGCCTGTCTCTACAGAATCTGCCTCT  mus_caroli: 4:108775407

Mouse  mus_musculus: 4:118189421 CCAGTGTGT‑‑‑‑AAAACAAACCTTGGAGTAGGTAGCCACTGGAT mus_musculus: 4:118189461
Ryukyu mouse  mus_caroli: 4:108775408 CCAGTGTGTGTGTAAAACAAACCTTGGAGTAGGTAGCTACTGGAT  mus_caroli: 4:108775452

Mouse  mus_musculus: 4:118189462 ATCCTTCCATGCACAAGTTTCACAGCTATTTCTCTGTGTGTAAAT mus_musculus: 4:118189506
Ryukyu mouse  mus_caroli: 4:108775453 ATCCTTCCATGCACAAGTTTCACAGCTATTTCTCTACGTGTAAAT  mus_caroli: 4:108775497

Mouse  mus_musculus: 4:118189507 CAGCTGTGTCATTAAATTCTTTATAGAGAGTCCCCCGGTCAAGT  mus_musculus: 4:118189550
Ryukyu mouse  mus_caroli: 4:108775498 CAGCTGTGCCATTAAATTCTTTATAGAGAGTTCCCTGGTCAAGT  mus_caroli: 4:108775541
