# Supplementary material for: Recurrent erosion of COA1/MITRAC15 exemplifies conditional gene dispensability in oxidative phosphorylation
Source: Sci Rep. 2021 Dec 24;11:24437. doi: 10.1038/s41598-021-04077-y (PMC8709867; doi:10.1038/s41598-021-04077-y)
Supplement: Supplementary file 3 — Supplementary Information 3. [file 41598_2021_4077_MOESM3_ESM.zip › Supplementary Dataset/COA1_GENE-main_Github_use_winRAR_to_open.zip!/COA1_GENE/supplementary_figure_data/Pairwise_genome_alignment/Pairwise_alignment_from_ensembl/Mouse_Upper_Galilee_mountains_blind_mole_rat_lastz.odt]

Mouse  mus_musculus: 4:118187691 CCTTCCCTCCCTTCTTTCCTTCCTTCTTCCTCTTTCTTTTTTGTT  mus_musculus: 4:118187735
Upper Galilee mountains blind mole rat  
.............................................  

Mouse  mus_musculus: 4:118187736 GTACTGGCAACTAAATCCGGGGAACTCATGCATGCTGGCTAAGGC  mus_musculus: 4:118187780
Upper Galilee mountains blind mole rat  
.............................................  

Mouse  mus_musculus: 4:118187781 TCTACCACCGAGCTAAGAACCAAGGCCTTTTTCAGAGATTTCCAA  mus_musculus: 4:118187825
Upper Galilee mountains blind mole rat  
.............................................  

Mouse  mus_musculus: 4:118187826 AATACTTTTGTTACAATGTGACAAAGATCTCTACGAAGTCCTTTG  mus_musculus: 4:118187870
Upper Galilee mountains blind mole rat nannospalax_galili: KL202943.1:348331 
......................AAAAGGCTCTAGGAAGCAGGATA nannospalax_galili: KL202943.1:348309

Mouse  mus_musculus: 4:118187871 TCGGGGCCTCTTGGAATACTGTTCCTTTATCCCAGTGTATTGGAG  mus_musculus: 4:118187915
Upper Galilee mountains blind mole rat nannospalax_galili: KL202943.1:348308 TCAGGGCCTCTGGGAAAACTGTTCATTTATACCTGTGTATTGATG nannospalax_galili: KL202943.1:348264

Mouse  mus_musculus: 4:118187916 GAAGGGTGGGAGAGGTGGAGGATAGGGGATAGGGGTGATGGTGGA  mus_musculus: 4:118187960
Upper Galilee mountains blind mole rat nannospalax_galili: KL202943.1:348263 GCAGGGGG‑‑‑‑‑‑‑‑‑‑‑‑‑‑‑‑‑‑‑‑‑‑‑‑‑‑‑‑‑‑‑‑‑‑‑‑‑ nannospalax_galili: KL202943.1:348256

Mouse  mus_musculus: 4:118187961 GAGTGCTCCTTCTCTTGTTACTTTAAGGAGAGCATAAGTGTGTCA  mus_musculus: 4:118188005
Upper Galilee mountains blind mole rat nannospalax_galili: KL202943.1:348255 ‑‑‑‑‑‑‑‑CTTTTTGTATTGCCTTATGCAGAG‑‑‑ACGTGTGTCA nannospalax_galili: KL202943.1:348222

Mouse  mus_musculus: 4:118188006 TTCAGCAGACAGACAAACAG‑‑CCAACCGTCCTCTCTTCTC‑‑‑‑  mus_musculus: 4:118188044
Upper Galilee mountains blind mole rat nannospalax_galili: KL202943.1:348221 C‑CAGCAGGCAATCAAATAGATCCAAGGGTCTTCTCCTCCCCTTC nannospalax_galili: KL202943.1:348178

Mouse  mus_musculus: 4:118188045 ‑‑‑ACTTTCCTGTAGAAAGTTGAGTCCCTTGAATCTGGAGTGGCC  mus_musculus: 4:118188086
Upper Galilee mountains blind mole rat nannospalax_galili: KL202943.1:348177 CTTGCTTTCCTGGAGTTGGCTGAGTCCCTTGAGTCAAGAGTGGCC nannospalax_galili: KL202943.1:348133

Mouse  mus_musculus: 4:118188087 CAGGAGGACCATTTCCAGGAATGGTTCAGACTGGACTCTAACCCA  mus_musculus: 4:118188131
Upper Galilee mountains blind mole rat nannospalax_galili: KL202943.1:348132 AGAAAAGAGGATTCCCAGGAACAATTCACACTGGAATGTAGCTTG nannospalax_galili: KL202943.1:348088

Mouse  mus_musculus: 4:118188132 GCCTCGTCTCCTCTCTGCGGTGAGGACCAGGCAGGACACAGCAGA  mus_musculus: 4:118188176
Upper Galilee mountains blind mole rat nannospalax_galili: KL202943.1:348087 GCCTCCTCGTCCCTCTGCCATGTAGACTGGGCAGGACAGGACAGA nannospalax_galili: KL202943.1:348043

Mouse  mus_musculus: 4:118188177 CA‑GGGCACATTACGGGGTCA‑‑‑‑‑‑‑‑GGATTCA‑‑‑‑‑‑‑‑‑  mus_musculus: 4:118188203
Upper Galilee mountains blind mole rat nannospalax_galili: KL202943.1:348042 AATGGGTACATTCTTGGATCATGTCTGGGGGATGTGGTAATGGGC nannospalax_galili: KL202943.1:347998

Mouse  mus_musculus: 4:118188204 ‑‑GCCTTCTTGACTGACTGTCTCAGTGCCTTCGAAAGCTGGTGTC  mus_musculus: 4:118188246
Upper Galilee mountains blind mole rat nannospalax_galili: KL202943.1:347997 TTGCCTTCTTGTCTGACAGCTTTTTCCCTTAAGGAAGCTGGTATC nannospalax_galili: KL202943.1:347953

Mouse  mus_musculus: 4:118188247 TGTCAGCAGAGACCATGGAGCAGTGTGGTCTAGGTTATACAGCCA  mus_musculus: 4:118188291
Upper Galilee mountains blind mole rat nannospalax_galili: KL202943.1:347952 TGCCAGCAGAGCC‑‑‑‑‑AGTAGTGAGATCTGAGCTGTACAGACA nannospalax_galili: KL202943.1:347913

Mouse  mus_musculus: 4:118188292 GATGCCAC‑‑‑‑‑ACCTCAGGGCTAGCTTCTTTAAGCTGTCTCTC  mus_musculus: 4:118188331
Upper Galilee mountains blind mole rat nannospalax_galili: KL202943.1:347912 TCGGCCCTCACTGACTCTCAGTCTAGCTCAGCT‑AGCTATATGTC nannospalax_galili: KL202943.1:347869

Mouse  mus_musculus: 4:118188332 CAGGGAAGTCCTTCATCATGTTGGGTCTTAGCTGTCTTCCCTGTG  mus_musculus: 4:118188376
Upper Galilee mountains blind mole rat nannospalax_galili: KL202943.1:347868 ‑‑‑‑‑‑‑‑‑CCTTCATCTTCCTGGGACTTAGTGGCCTTCCCTGTG nannospalax_galili: KL202943.1:347833

Mouse  mus_musculus: 4:118188377 CG‑‑‑‑‑‑‑‑‑‑‑‑‑‑‑‑‑‑‑‑‑‑‑‑‑‑‑‑‑‑GTATGAATCAAGT  mus_musculus: 4:118188391
Upper Galilee mountains blind mole rat nannospalax_galili: KL202943.1:347832 TGATGAGGGGTAGAATGAGTAGGTATGAAGTTGTACGAACCAAGT nannospalax_galili: KL202943.1:347788

Mouse  mus_musculus: 4:118188392 T‑‑‑CTGCATTCAAGCTATTGCAATACAGAGGATTTCTAGAGATA  mus_musculus: 4:118188433
Upper Galilee mountains blind mole rat nannospalax_galili: KL202943.1:347787 TGCACAACATTCAAGCTATCACAGTATCGAAGACTCT‑‑‑‑‑‑‑‑ nannospalax_galili: KL202943.1:347751

Mouse  mus_musculus: 4:118188434 TGTGGAATAGTGAGGCATGTCCCTCACCATAAAAAAAAAAAAAAT  mus_musculus: 4:118188478
Upper Galilee mountains blind mole rat nannospalax_galili: KL202943.1:347750 ‑‑‑‑GAGTAGTGAGGCATGGCCCTTGCCATAA‑‑‑‑‑‑‑‑‑‑‑‑‑ nannospalax_galili: KL202943.1:347723

Mouse  mus_musculus: 4:118188479 CCACAGAGTTAGGGCAAGCCAGTTGGCTCAGCCAGCGAAGGTGTT  mus_musculus: 4:118188523
Upper Galilee mountains blind mole rat nannospalax_galili: KL202943.1:347722 ‑‑‑‑‑‑‑‑‑‑‑‑‑‑‑CAGCCA‑‑‑‑‑CTCATCTGGTGAAAGTGCT nannospalax_galili: KL202943.1:347698

Mouse  mus_musculus: 4:118188524 CTGAAGCCTAATGATGAAAGTTCCGATGCTTGGAA‑‑CTCATGAT  mus_musculus: 4:118188566
Upper Galilee mountains blind mole rat nannospalax_galili: KL202943.1:347697 CTGAAGCCTGACAACCTCAGTT‑TGATGTCTGGAACCCCCACAGT nannospalax_galili: KL202943.1:347654

Mouse  mus_musculus: 4:118188567 CGAAGCA‑‑‑‑‑‑‑‑‑CTCCTAAAAG‑‑‑‑‑‑‑CTGCTCTCCACA  mus_musculus: 4:118188595
Upper Galilee mountains blind mole rat nannospalax_galili: KL202943.1:347653 GGAAGGAAAGAACCAGTTCCTGACAATAGTCCTCTGACCTTCATA nannospalax_galili: KL202943.1:347609

Mouse  mus_musculus: 4:118188596 TGCATACCATGGCACATGCCAATTGGAATACACACACACACACAC  mus_musculus: 4:118188640
Upper Galilee mountains blind mole rat nannospalax_galili: KL202943.1:347608 TGCATGTTATGGCAC‑‑‑‑‑‑‑‑‑‑‑‑‑‑‑‑‑‑‑‑‑‑‑‑‑TATGT nannospalax_galili: KL202943.1:347589

Mouse  mus_musculus: 4:118188641 ACACACACACACACACACACACACACACACACACACACTCCACAA  mus_musculus: 4:118188685
Upper Galilee mountains blind mole rat nannospalax_galili: KL202943.1:347588 GTGCTTGCACACACACACACACACACACACGCACACACT‑‑‑‑‑‑ nannospalax_galili: KL202943.1:347550

Mouse  mus_musculus: 4:118188686 GTAGTAAATAAAATAAAAACAAAAACGAAGGAACTCATTTAAAAG  mus_musculus: 4:118188730
Upper Galilee mountains blind mole rat nannospalax_galili: KL202943.1:347549 ‑‑‑‑‑‑‑‑‑‑AAACAACAACAACAACAAAATCACTCAGTTAAGAG nannospalax_galili: KL202943.1:347515

Mouse  mus_musculus: 4:118188731 TAGCTGCCTAGACCCAGCCTCTCCTTGGCTACCTCTACAGCTCTG  mus_musculus: 4:118188775
Upper Galilee mountains blind mole rat nannospalax_galili: KL202943.1:347514 TAGCTGTCTGGACCCACCCTCTTCTTGGCCACGCCTACAGCT‑TA nannospalax_galili: KL202943.1:346547

Mouse  mus_musculus: 4:118188776 TGCCGGAGAAGCAGCATAAAGGTGGCTCTCTGTGGCTT‑TCCACG  mus_musculus: 4:118188819
Upper Galilee mountains blind mole rat nannospalax_galili: KL202943.1:346546 CTCTGGGTTAGCAGTGTTAGGACAGTTCTCTGTGGCTTCTCCAGG nannospalax_galili: KL202943.1:346502

Mouse  mus_musculus: 4:118188820 CTTGGGCTACACTG‑TTTCTACGACTGGACCTGCCGGAGGGAAGC  mus_musculus: 4:118188863
Upper Galilee mountains blind mole rat nannospalax_galili: KL202943.1:346501 CTGAGGCCACCCTAACTTCTGTGACTGGCCCTGCAGGAGGGAAGC nannospalax_galili: KL202943.1:346457

Mouse  mus_musculus: 4:118188864 TCTTTCATGCCACAAAACAG‑‑TTTATCGTTGTTCTGTTTCTTGA  mus_musculus: 4:118188906
Upper Galilee mountains blind mole rat nannospalax_galili: KL202943.1:346456 TCTTAC‑TGTCAAACTGCAGACCTTATC‑TTGCTCTGTTCGCTGA nannospalax_galili: KL202943.1:346414

Mouse  mus_musculus: 4:118188907 GCAGCTCGTCTGACCTTACACCTTCAGGGAGAAGCTTTCCCAGGA  mus_musculus: 4:118188951
Upper Galilee mountains blind mole rat nannospalax_galili: KL202943.1:346413 GCTGCTTCTGGTACCTTGTTCCTTCAGGGAGGAGCTT‑‑CCAGGA nannospalax_galili: KL202943.1:346371

Mouse  mus_musculus: 4:118188952 TCCCAATAGCCTTTGGACTAGGGAGCGATCTTGGGGTAAAAAGCA  mus_musculus: 4:118188996
Upper Galilee mountains blind mole rat nannospalax_galili: KL202943.1:346370 TTCCAAGAGCCTTTGGGCTAAGCAGGGCTCCTG............ nannospalax_galili: KL202943.1:346338

Mouse  mus_musculus: 4:118188997 ATTTGGGGTTAAGGAGGTATCTTGCACTGAGAAGCAACTTTAGTC  mus_musculus: 4:118189041
Upper Galilee mountains blind mole rat nannospalax_galili: KL202943.1:346337 
.........................................AGAC nannospalax_galili: KL202943.1:346334

Mouse  mus_musculus: 4:118189042 CGAGAGCTATTCTACTGGAAATGTTAGCTCTGCTCTGTAAACATG  mus_musculus: 4:118189086
Upper Galilee mountains blind mole rat nannospalax_galili: KL202943.1:346333 CCTGAGCTGTTCTGCTTGAACTGCTGGCTCCGATCTGTAAACTTG nannospalax_galili: KL202943.1:346289

Mouse  mus_musculus: 4:118189087 ACACTCGAGCATTTCCATGCCCTGTTTCTTGGCCCAAGGGTTTTG  mus_musculus: 4:118189131
Upper Galilee mountains blind mole rat nannospalax_galili: KL202943.1:346288 ACAAATGAGCATCTCCAGGTCCTGTTTCTTGGCCCAAGGGTTTTG nannospalax_galili: KL202943.1:346244

Mouse  mus_musculus: 4:118189132 TTGTGGTTAATACTGTGGTCTGTCTATTTTATGCTTCATCTTCCT  mus_musculus: 4:118189176
Upper Galilee mountains blind mole rat nannospalax_galili: KL202943.1:346243 TTGTGGCTAATACTGTG‑‑‑‑GTCTATTTTATGCTGCATCTTTGT nannospalax_galili: KL202943.1:346203

Mouse  mus_musculus: 4:118189177 GGAAACAGTAAAATGTTTAGCTGTCACACATTCTTGGCTCGTGCT  mus_musculus: 4:118189221
Upper Galilee mountains blind mole rat nannospalax_galili: KL202943.1:346202 GG‑AACAGTAAAATGTTTGGCTGTCACACATTCTGGCCT‑‑TGCT nannospalax_galili: KL202943.1:346161

Mouse  mus_musculus: 4:118189222 TTGCCTTGGGCCAGCTGTGTCACTGTGAGCAACACAATGGAATCT  mus_musculus: 4:118189266
Upper Galilee mountains blind mole rat nannospalax_galili: KL202943.1:346160 TTGCCTTGAGCCAGCTGTGTCACTTTGAGCAAGCTAATGGAATCA nannospalax_galili: KL202943.1:346116

Mouse  mus_musculus: 4:118189267 CAGGACTTTAGACCAGAGAG‑‑‑‑‑‑‑‑‑‑‑‑‑AACCAATACAAT  mus_musculus: 4:118189298
Upper Galilee mountains blind mole rat nannospalax_galili: KL202943.1:346115 CAGGACTTTAGAACCAAGAGAGGGTTAGGGAATAGCTGATCAAAC nannospalax_galili: KL202943.1:346071

Mouse  mus_musculus: 4:118189299 CCCTTCACTGAAGGGAAATGAGGCATTGAATGAGGTGAGGA‑‑AG  mus_musculus: 4:118189341
Upper Galilee mountains blind mole rat nannospalax_galili: KL202943.1:346070 CTCTTCATCGAAGGGGAATGAAGCAATGAATGAGGTGAGGAATAA nannospalax_galili: KL202943.1:346026

Mouse  mus_musculus: 4:118189342 AGAGATTGGACCACAGAAATGTCAAAG‑TGGCCCTTTTCAAGGTC  mus_musculus: 4:118189385
Upper Galilee mountains blind mole rat nannospalax_galili: KL202943.1:346025 AGAGGTTGAGTCACAGAAAGGTAACAGATGACCTTCTTCAAGGTT nannospalax_galili: KL202943.1:345981

Mouse  mus_musculus: 4:118189386 ATCTCTGTTCGCCTGTCTCTATAGAATCTGCCTCTCCAGTGTGTA  mus_musculus: 4:118189430
Upper Galilee mountains blind mole rat nannospalax_galili: KL202943.1:345980 A‑‑TCTATCTGCACATCTCTGTAGATTCTGCCTCTTAAGTGTGTA nannospalax_galili: KL202943.1:345938

Mouse  mus_musculus: 4:118189431 AAAC‑AAACCTTGGAGTAGGTAGCCACTGGATATCCTTCCATGCA  mus_musculus: 4:118189474
Upper Galilee mountains blind mole rat nannospalax_galili: KL202943.1:345937 CAATGAAAATTTAGAATAGAT‑GTCACTTGCTATTCTCCCATGCC nannospalax_galili: KL202943.1:345894

Mouse  mus_musculus: 4:118189475 CAAGTTTCACAGCTATTTCTCTGTG‑TGTAAATCAGCTGTGTCAT  mus_musculus: 4:118189518
Upper Galilee mountains blind mole rat nannospalax_galili: KL202943.1:345893 CAAATTTCACACCTCTGCTTCTATACTGCAA‑‑‑AACTGTATTAC nannospalax_galili: KL202943.1:345852

Mouse  mus_musculus: 4:118189519 TAAATTCTTTATAGAGAGTCCCCCGGTCAAGT  mus_musculus: 4:118189550
Upper Galilee mountains blind mole rat nannospalax_galili: KL202943.1:345851 TAAACTTTCCACAGAAATTCCACTTGGC‑TGT  nannospalax_galili: KL202943.1:345821
