# Supplementary material for: Recurrent erosion of COA1/MITRAC15 exemplifies conditional gene dispensability in oxidative phosphorylation
Source: Sci Rep. 2021 Dec 24;11:24437. doi: 10.1038/s41598-021-04077-y (PMC8709867; doi:10.1038/s41598-021-04077-y)
Supplement: Supplementary file 3 — Supplementary Information 3. [file 41598_2021_4077_MOESM3_ESM.zip › Supplementary Dataset/COA1_GENE-main_Github_use_winRAR_to_open.zip!/COA1_GENE/supplementary_figure_data/Pairwise_genome_alignment/Pairwise_alignment_from_ensembl/Eurasian_red_squirrel_Mouse_lastz.odt]

Eurasian red squirrel 8:103903116 GGGGGTGGGGGGACTAGCATTATACCCAGTCCTATGCCACAATGC 8:103903072
Mouse  .............................................  

Eurasian red squirrel 8:103903071 CTCTAACCCCTTTAGGTCATATCAAAGACTTTGTTTTCTAAAGAA 8:103903027
Mouse  .............................................  

Eurasian red squirrel 8:103903026 CAACAATTAAGATTAAATCTTCAAATGGAGACACACATACTTATG 8:103902982
Mouse  .............................................  

Eurasian red squirrel 8:103902981 GAGTTATCTTTTTCTTTATCTAAAGCTGGCATTTCCATCTCTATC 8:103902937
Mouse  .............................................  

Eurasian red squirrel 8:103902936 TAAAATCACCTTCCTTTTTGAAGAAATGTCATTAAAGACAAAGTC 8:103902892
Mouse  .............................................  

Eurasian red squirrel 8:103902891 CTAAATCTCACCTAGGATAAGGATGCCCTGGCAGCCAAAGGAAAC 8:103902847
Mouse  .............................................  

Eurasian red squirrel 8:103902846 CAAAAGCCCATAGAAAGATTTGACCCACGAATGTATTTTGTTAAG 8:103902802
Mouse  .............................................  

Eurasian red squirrel 8:103902801 TCTTTGTTTTTTTTTTTTGTTGGTGGTGGTGGTTGTCAACATTTA 8:103902757
Mouse  .............................................  

Eurasian red squirrel 8:103902756 GAATTCAGGCAGTTTCACATCAAAGATCTGATTCCTGGCACCTTT 8:103902712
Mouse  .............................................  

Eurasian red squirrel 8:103902711 TGAAGATGTAAAGATTCATCGAGACTTGGTCCATATAGCCACAAT 8:103902667
Mouse  .............................................  

Eurasian red squirrel 8:103902666 TAGGTAGAGGCAGGTGAAAGCTTCCCTCTTGGCTGGGTTGTGGTG 8:103902622
Mouse  .............................................  

Eurasian red squirrel 8:103902621 CCTCACTTTTGCAGTATCTGATCTCATGAATGTGATCTGTCTGAC 8:103902577
Mouse  .............................................  

Eurasian red squirrel 8:103902576 CCTATGGGCATTTGAATTGTCCTGTTCTGTCCCCTACAGCCAGCG 8:103902532
Mouse  .............................................  

Eurasian red squirrel 8:103902531 TCTCATTCTGTTTTAATGTCAGGTACTTATAGCTTTCCAGAAAGG 8:103902487
Mouse  .............................................  

Eurasian red squirrel 8:103902486 CTCAGGCTGGTGTGAGTACTGATTAAATCGCTACCAGAAGGAAGA 8:103902442
Mouse  .............................................  

Eurasian red squirrel 8:103902441 GAATTGGCTTCATCGAGGCCCACGTGCTGCTCCTAGCCCCACCCA 8:103902397
Mouse  .............................................  

Eurasian red squirrel 8:103902396 GGCCTTCCAGGGATAGGAGTTAATGCCAGGGCGGATCTGGTATCT 8:103902352
Mouse  .............................................  

Eurasian red squirrel 8:103902351 GTATTTGCTGAGCTGGCACCACTCCATCTGTTCACTCAGCCCAAA 8:103902307
Mouse  .............................................  

Eurasian red squirrel 8:103902306 AGTCAAAACCAAAGTTTCCTCATCCAGGGGGTGGGGCATTTTTAA 8:103902262
Mouse  .............................................  

Eurasian red squirrel 8:103902261 GAATCTGAAAGAAAGAAGCCAGTGACAGCGCTGGCCCATTTGAAG 8:103902217
Mouse  .............................................  

Eurasian red squirrel 8:103902216 TCTTTTATGACCAGCTATTGCCACCAAAGGATAAGTTTCCTGTTT 8:103902172
Mouse  .............................................  

Eurasian red squirrel 8:103902171 ACCAGTGGGCCTGAGACACAGTGGTTGTACTTCCTGACTGTGCTG 8:103902127
Mouse  .............................................  

Eurasian red squirrel 8:103902126 AGCTTGGCACACAGGCCAGGTTCTGTCTGTCAGAATGGTGAGGCT 8:103902082
Mouse  .............................................  

Eurasian red squirrel 8:103902081 GAATGCCCACTCTGTACTTGGCTGGGTGAGGGTGGCAGGGCCCCC 8:103902037
Mouse  .............................................  

Eurasian red squirrel 8:103902036 ATAGCCTGGGTTCTGGATGAGGGCCACCCTCAGGAGGGCACTTAA 8:103901992
Mouse  .............................................  

Eurasian red squirrel 8:103901991 CTTTGGCTCCAGAACTGGCCTTAGCATTCAGAAAGGAAGCAAAAG 8:103901947
Mouse  .............................................  

Eurasian red squirrel 8:103901946 AGGGGAGCGGCCTTGCCTCCTTTCTGGCTCTCCCCTTTAGATTTC 8:103901902
Mouse  4:118187691 ..........CCTTCCCTCCCTTCTTTCCTTCCTTCTTCCTCTTT 4:118187725

Eurasian red squirrel 8:103901901 CTTTTCC‑‑‑‑‑‑‑‑‑‑‑‑‑‑‑‑‑‑TCTGGGGTCC‑‑‑‑‑AATCC 8:103901880
Mouse  4:118187726 CTTTTTTGTTGTACTGGCAACTAAATCCGGGGAACTCATGCATGC 4:118187770

Eurasian red squirrel 8:103901879 AGGCGAAGGCTCT‑‑‑‑‑‑TGGTGCAGAAC‑‑AGAATCTTTTAAT 8:103901843
Mouse  4:118187771 TGGCTAAGGCTCTACCACCGAGCTAAGAACCAAGGCCTTTTTCAG 4:118187815

Eurasian red squirrel 8:103901842 AAATTTGTAT‑‑‑‑CTTTTGTTTTTAAGT‑‑‑‑‑‑‑‑‑CTCCAGG 8:103901811
Mouse  4:118187816 AGATTTCCAAAATACTTTTGTTACAATGTGACAAAGATCTCTACG 4:118187860

Eurasian red squirrel 8:103901810 AAGCAGGATGCCAGAGCCTCTGGGGAAAATGTTCCTTTATACCAG 8:103901766
Mouse  4:118187861 AAGTCCTTTGTCGGGGCCTCTTGGAATACTGTTCCTTTATCCCAG 4:118187905

Eurasian red squirrel 8:103901765 TGTGTTGATGGCAGGGACCTGTCCTCTTTTTTATTACCTTGTACA 8:103901721
Mouse  4:118187906 TGTATTGGAGGAAGGG............................. 4:118187921

Eurasian red squirrel 8:103901720 AAGTAAGTATCCAGGGAGCTGCCTGAGGCATGACTTCCTAGTTCT 8:103901676
Mouse  .............................................  

Eurasian red squirrel 8:103901675 GGTGGTCCTGCTCCTCTCTCAAAGGAAATGCTTGAGATGCTTTCG 8:103901631
Mouse  .............................................  

Eurasian red squirrel 8:103901630 GCAAATTAGAGAAAGGAGGAGAGGACTATTTCAGAAGCCAGTCCA 8:103901586
Mouse  .............................................  

Eurasian red squirrel 8:103901585 CTATCACATTGGCTAAAGCTTCCTTCTTATCCTATAATCCTCCTT 8:103901541
Mouse  .............................................  

Eurasian red squirrel 8:103901540 TTTCCAGGAGCCACAAGTACAAATAAAAGGACTAGGATCCATTTG 8:103901496
Mouse  .............................................  

Eurasian red squirrel 8:103901495 CATAAGAAGCCTTGCAAAGTAAGATAGGCTTTCACTCCAGTGCAC 8:103901451
Mouse  .............................................  

Eurasian red squirrel 8:103901450 ACACAGGGCATCCATCAAGATTTCTGAGATTACATAAAGAAAGAA 8:103901406
Mouse  .............................................  

Eurasian red squirrel 8:103901405 CAGAGAAGGGGAAGACCAGATGGTTCACAGGTGGGGAAAACTGTG 8:103901361
Mouse  .............................................  

Eurasian red squirrel 8:103901360 GCTACTTCTCAAGTGTGGCCAGGCTGCTTGCCCTGCCATCCTCTG 8:103901316
Mouse  .............................................  

Eurasian red squirrel 8:103901315 TTATTGACTGAGTGGAGGAGGGGAACTTGAGTAAGATATGCTGGC 8:103901271
Mouse  .............................................  

Eurasian red squirrel 8:103901270 AGGAGCAGAAAATAAAAGCAGAGTCCAGGATGGCAAAATCCCAAC 8:103901226
Mouse  .............................................  

Eurasian red squirrel 8:103901225 AGAGGCAAAATCCTGACAGATGCAGCTTCCAGCCTCACTGCACCC 8:103901181
Mouse  .............................................  

Eurasian red squirrel 8:103901180 AAATTTGAATTTGTAAAGACAGGCCACAATTATCGTCAGCAGCCA 8:103901136
Mouse  .............................................  

Eurasian red squirrel 8:103901135 AGACATTTAAGGTCATTTCAATGCTACTGGTGGCAAAGCAGCAAC 8:103901091
Mouse  .............................................  

Eurasian red squirrel 8:103901090 ATTTTTGTCTTGGACTGACTGTTTTTGATGCTTCTTTATCCCTCT 8:103901046
Mouse  .............................................  

Eurasian red squirrel 8:103901045 CAGCTTGGCTTCTCAATCTGACTCTGCAGCATTTTCACTTCTTTC 8:103901001
Mouse  .............................................  

Eurasian red squirrel 8:103901000 TAGGATGTGTGTCAC‑CAGCAGACAAGCAAACAGAGCACAGGGGC 8:103900957
Mouse  4:118187998 ......GTGTGTCATTCAGCAGACAGACAAACAGCCAACCGT‑‑C 4:118188034

Eurasian red squirrel 8:103900956 CTCTCCACCCATTTTCACCTTCCTGCAAAGTTGGTCAAGTCCCT‑ 8:103900913
Mouse  4:118188035 CTCTC‑‑‑‑‑‑TTCTCACTTTCCTGTAGAA‑‑AGTTGAGTCCCTT 4:118188071

Eurasian red squirrel 8:103900912 GAATCTGGAGGTGGCCCACGATGAGCATTCCCAAGAATGATTCAG 8:103900868
Mouse  4:118188072 GAATCTGGAG‑TGGCCCAGGAGGACCATTTCCAGGAATGGTTCAG 4:118188115

Eurasian red squirrel 8:103900867 ACTAGAACATAACTCAGCCTCCTCTCTTGTCTGCCTAAAGAATGG 8:103900823
Mouse  4:118188116 ACTGGACTCTAACCCAGCCTCGTCTCCTCTCTGCGGTGAGGAC‑C 4:118188159

Eurasian red squirrel 8:103900822 GGGCAGGACTCAATAGGAACGGGTGCATT‑TTCTATCAGGTTTCA 8:103900779
Mouse  4:118188160 AGGCAGGACACAGCAGACA‑GGGCACATTACGGGGTCAGGATTCA 4:118188203

Eurasian red squirrel 8:103900778 GCCTTCTGGTCTGACAGTCTTTCCCCCTTAGGAAGCTGGAGTTGG 8:103900734
Mouse  4:118188204 GCCTTCTTGACTGACTGTCTCAGTGCCTTCGAAAGCTGGTGTCTG 4:118188248

Eurasian red squirrel 8:103900733 CTAACAGAGACAAGCTGGGTAGTG‑AGACTGGGCTCGGTAGCTGC 8:103900690
Mouse  4:118188249 TCAGCAGAGACCA‑TGGAGCAGTGTGGTCTAGGTTATACAGCCAG 4:118188292

Eurasian red squirrel 8:103900689 ACTGGTTGAATCCCAGATCCAGTTTTTGATTAGCTGTGCAGTCTT 8:103900645
Mouse  4:118188293 AT‑‑GCCACACCTCAGGGCTAGCTTCT‑‑‑‑‑‑TTAAGCTGTCTC 4:118188329

Eurasian red squirrel 8:103900644 ACAAGGGAAA‑‑‑‑‑‑‑‑‑‑‑‑‑‑‑‑‑‑‑‑‑‑‑‑‑‑‑CTTCCC‑‑ 8:103900629
Mouse  4:118188330 TCCAGGGAAGTCCTTCATCATGTTGGGTCTTAGCTGTCTTCCCTG 4:118188374

Eurasian red squirrel 8:103900628 ‑‑‑‑‑‑‑‑‑‑‑‑‑‑‑‑‑‑‑‑‑‑‑‑‑‑‑‑‑‑‑‑GTGTAATGAAGAG 8:103900616
Mouse  4:118188375 TGCGGTATGAATCAAGTTCTGCATTCAAGCTATTGCAATACAGAG 4:118188419

Eurasian red squirrel 8:103900615 TAATATCAG‑TATGTATGAAATTGT‑‑‑‑‑‑TGT‑‑‑‑‑‑‑‑‑‑‑ 8:103900589
Mouse  4:118188420 GATTTCTAGAGATATGTGGAATAGTGAGGCATGTCCCTCACCATA 4:118188464

Eurasian red squirrel 8:103900588 ‑‑‑‑‑‑‑‑GAGAACTAATTGAGTT‑‑‑‑‑‑‑‑‑‑‑‑‑‑‑GCACAA 8:103900567
Mouse  4:118188465 AAAAAAAAAAAAATCCACAGAGTTAGGGCAAGCCAGTTGGCTCAG 4:118188509

Eurasian red squirrel 8:103900566 CTTGTGAGAAGAGATTTTGAGG‑‑‑‑ATTATGAAAGTGTGAAAAT 8:103900526
Mouse  4:118188510 CCAGC‑‑GAAGGTGTTCTGAAGCCTAATGATGAAAGT........ 4:118188544

Eurasian red squirrel 8:103900525 GTTGAGAACAGTTTGTCACCACAGAAATCACACTCAGTCAAGACC 8:103900481
Mouse  4:118188718 ...............................ACTCATTTAAAAGT 4:118188731

Eurasian red squirrel 8:103900480 TGCTGTCTGGGTAAAGCCTCTGCTGCTCCTTTTCCTATTATAGCT 8:103900436
Mouse  4:118188732 AGCTGCCTAGACCCAGCCTCT‑‑‑‑‑‑‑‑‑‑‑‑‑‑‑‑‑‑‑‑‑‑‑‑ 4:118188752

Eurasian red squirrel 8:103900435 GGCACCTTGGTCATGCCTGAAGCTCCATTTCTGGGAAGAAGCAGC 8:103900391
Mouse  4:118188753 ‑‑‑‑CCTTGGCTACCTCTACAGCTCTGTGCCGGAGAAGCAGCATA 4:118188793

Eurasian red squirrel 8:103900390 AGGGGTATATCCTTGGCCTCTCCATGCTCAGGCCTCCTTCATTAC 8:103900346
Mouse  4:118188794 AAGGTGGCTCTCTGTGGCTTTCCACGCTTGGG‑CTACACTGTTTC 4:118188837

Eurasian red squirrel 8:103900345 TGTGATTGGACTTGTAGGAAGAAAGTCCTTTCTGGTTGCCAAACA 8:103900301
Mouse  4:118188838 TACGACTGGACCTGCCGGAGGGAAGCTCTTTCATGCCACAAAACA 4:118188882

Eurasian red squirrel 8:103900300 TTCTACTTAGGATGATCTCCTCCTTGAGCAGGTTGTT‑‑‑CTTTA 8:103900259
Mouse  4:118188883 GTTTATC‑‑‑GTTGTTCTGTTTCTTGAGCAGCTCGTCTGACCTTA 4:118188924

Eurasian red squirrel 8:103900258 TCCCTTTAGGGAGATGCTTCCCAGGGCTCTCAAGTAGCC‑‑‑‑‑‑ 8:103900220
Mouse  4:118188925 CACCTTCAGGGAGAAGCTTTCCCAGGATCCCAA‑TAGCCTTTGGA 4:118188968

Eurasian red squirrel 8:103900219 ‑‑‑‑‑‑‑‑‑‑‑‑‑‑‑‑‑‑‑‑‑‑‑‑‑‑‑‑‑‑TTGGGGTT‑‑‑‑‑‑‑ 8:103900212
Mouse  4:118188969 CTAGGGAGCGATCTTGGGGTAAAAAGCAATTTGGGGTTAAGGAGG 4:118189013

Eurasian red squirrel 8:103900211 ‑‑‑‑‑‑‑‑‑‑‑‑‑‑‑‑‑‑‑‑CCTGAGTGTTAGAG‑‑‑‑‑‑‑‑CTT 8:103900195
Mouse  4:118189014 TATCTTGCACTGAGAAGCAACTTTAGTCCGAGAGCTATTCTACTG 4:118189058

Eurasian red squirrel 8:103900194 GAAATGTTGGCTCAAGCCTGTAAAT‑TGACAAATGAGCATTTCCA 8:103900151
Mouse  4:118189059 GAAATGTTAGCTCTGCTCTGTAAACATGACACTCGAGCATTTCCA 4:118189103

Eurasian red squirrel 8:103900150 TGTCATATT‑‑TTGGCCCAAGGGTTTTCTT‑‑‑GTTAATATTGTG 8:103900111
Mouse  4:118189104 TGCCCTGTTTCTTGGCCCAAGGGTTTTGTTGTGGTTAATACTGTG 4:118189148

Eurasian red squirrel 8:103900110 GTCTATT‑‑TTATATACCTCATTTTAGTGGAAGCAGC‑‑AATGTT 8:103900070
Mouse  4:118189149 GTCTGTCTATTTTATGCTTCATCTTCCTGGAAACAGTAAAATGTT 4:118189193

Eurasian red squirrel 8:103900069 TGGAAATCA‑‑‑‑‑‑GCTGGCCCCTGCTTT‑‑‑‑‑‑‑‑CCAGAAC 8:103900039
Mouse  4:118189194 TAGCTGTCACACATTCTTGGCTCGTGCTTTGCCTTGGGCCAG‑‑C 4:118189236

Eurasian red squirrel 8:103900038 TGTGTCACTTTGAGCAA‑GTTACAGAATCATAGGACTTGAGAACT 8:103899995
Mouse  4:118189237 TGTGTCACTGTGAGCAACACAATGGAATCTCAGGACTTTAGACCA 4:118189281

Eurasian red squirrel 8:103899994 GAGAGGAAACTTAAAGAAGAGCTGATCAAACCCCTTAGTTAAAGG 8:103899950
Mouse  4:118189282 GAG‑‑‑‑‑‑‑‑‑‑‑‑‑‑AGAACCAATACAATCCCTTCACTGAAGG 4:118189312

Eurasian red squirrel 8:103899949 GAAATGAGGTAGTGAGGGAGGTGAGGGAGAGAAGTAGTCGAGGCA 8:103899905
Mouse  4:118189313 GAAATGAGGCATTGAATGAGGTGAGGAAGAGA‑‑‑GATTGGACCA 4:118189354

Eurasian red squirrel 8:103899904 CAGAAAGGTCAGGATGACC‑‑‑‑‑‑‑‑‑‑‑‑‑‑‑‑‑‑‑‑TTGCAC 8:103899880
Mouse  4:118189355 CAGAAATGTCAAAGTGGCCCTTTTCAAGGTCATCTCTGTTCGCCT 4:118189399

Eurasian red squirrel 8:103899879 ATCCCTGTAAATACTGCCTTTTGAGTATGTAAAATGGACAGTTTG 8:103899835
Mouse  4:118189400 GTCTCTATAGAATCTGCCTCTCCAGTGTGTAAAACAAAC‑‑CTTG 4:118189442

Eurasian red squirrel 8:103899834 GAGTAGATGGTCCCCAATTATTCTTCCATCTTTAAATTTCACATT 8:103899790
Mouse  4:118189443 GAGTAGGTAGCCACTGGATATCCTTCCATGCACAAGTTTCACAGC 4:118189487

Eurasian red squirrel 8:103899789 TCTGTTTCTGTTTCCATTCTATCAATTGTATTATTTAACTTTTTA 8:103899745
Mouse  4:118189488 TATTTCTCTGTGTGTA‑‑‑AATCAGCTGTGTCATTAAATTCTTTA 4:118189529

Eurasian red squirrel 8:103899744 CAGAGATTCTGTCTGACCAAGTTCAAGAAAGAAAAAATAACATCT 8:103899700
Mouse  4:118189530 TAGAGAGTCC‑CCCGGTCAAGT....................... 4:118189550

Eurasian red squirrel 8:103899699 GTCTTTCCTATCTAGAAGGTTTTGCCAGGACTTCATTTTATCAAA 8:103899655
Mouse  .............................................  

Eurasian red squirrel 8:103899654 TGGCGTTGGAACAGCTGCATAGTCACCCGGAGGCACTGGAAGCTC 8:103899610
Mouse  .............................................  

Eurasian red squirrel 8:103899609 TGGGCACTCCCCTCCATGTTCACTATCTCCAGCTCCCCAGCAAAC 8:103899565
Mouse  .............................................  

Eurasian red squirrel 8:103899564 ACAACTTTGTGGACATTACCGTTGCCTGGGTAATATGTTCTCCCT 8:103899520
Mouse  .............................................  

Eurasian red squirrel 8:103899519 GTGAACTTTAGGCCAGGTTAGGAAGAGGGAGAGAAGGGGGCCTTG 8:103899475
Mouse  .............................................  

Eurasian red squirrel 8:103899474 TCATCGGGAAAGAGGCTTATAAAAAAGTTGGCATAAAGGGTCTGG 8:103899430
Mouse  .............................................  

Eurasian red squirrel 8:103899429 CATGGCAAAGTTCACTAAGTAAGACTGTCACTAGGCTTAGCACCT 8:103899385
Mouse  .............................................  

Eurasian red squirrel 8:103899384 TAAAAAAGTTTTTATTTTTTATTATGGATTTTAAACATTATACTT 8:103899340
Mouse  .............................................  

Eurasian red squirrel 8:103899339 ATATAAAATATATTTTTGCATTATTATGCATTAATTTATTTTCAT 8:103899295
Mouse  .............................................  

Eurasian red squirrel 8:103899294 ATATTTTGCTTCCTTTCTGGGAGAAGTGGTATAGCAGGAATGTAG 8:103899250
Mouse  .............................................  

Eurasian red squirrel 8:103899249 TTGGTTTGCTAATGTTGCCATCAGTAGAAAAAAAAAAACGATCAG 8:103899205
Mouse  .............................................  

Eurasian red squirrel 8:103899204 TAGTGGTTTGCCACAACTTTGGCTTTGAATTTAAAATGTAAAATG 8:103899160
Mouse  .............................................  

Eurasian red squirrel 8:103899159 TAGCCCTCATTCAGATGTAAGCAGCTCTTTAACCTTTAAATTTTT 8:103899115
Mouse  .............................................  

Eurasian red squirrel 8:103899114 TTTTTTTTTTTTTTGTATAGTTCAGTTTGGTAAAAGCTCACCTGA 8:103899070
Mouse  .............................................  

Eurasian red squirrel 8:103899069 AGATAAAATACATTTGTTCTTCTGTTTAACAAATACATTTTGAGT 8:103899025
Mouse  .............................................  

Eurasian red squirrel 8:103899024 TCATACTCCGGTTGGAGGAATGATTTTAACTTACACAGGGAATGC 8:103898980
Mouse  .............................................  

Eurasian red squirrel 8:103898979 AGACATACAGACATGCAGACATGCAGAACGCTCTTGGGGAGTTTG 8:103898935
Mouse  .............................................  

Eurasian red squirrel 8:103898934 GCACATAAATTCTCCTTGCTAAATGAAAGGACTGCCATTGATCTC 8:103898890
Mouse  .............................................  

Eurasian red squirrel 8:103898889 ACCAAAACATATAGAGGGCAGAACATTTAGAAAAACAGTTGGATC 8:103898845
Mouse  .............................................  

Eurasian red squirrel 8:103898844 TCCAGATGAGCTGTTAGGAAAAAAACTTGATTCTGCAGATGCTCA 8:103898800
Mouse  .............................................  

Eurasian red squirrel 8:103898799 CTGGATTTTATAGACCTGGCTCTAGAAAGCTGTTTTGATAGTTTT 8:103898755
Mouse  .............................................  

Eurasian red squirrel 8:103898754 GTGATGACAACAATGTCTCCAATTACATTTCTCCTGTCATATTAC 8:103898710
Mouse  .............................................  

Eurasian red squirrel 8:103898709 ACCCACAGTTGAAAATTCCTGTTTCTGGATCCAAGTCAGAGGGCC 8:103898665
Mouse  .............................................  

Eurasian red squirrel 8:103898664 ATGTCTACACCAGATCAACCAGAGTTGCCCCCTTTCAGAGGTATT 8:103898620
Mouse  .............................................  

Eurasian red squirrel 8:103898619 ATGGGAAAGTGGTAGGATGTGGGGGAGTGGAGAACCTCCTGATGG 8:103898575
Mouse  .............................................  

Eurasian red squirrel 8:103898574 AAAAAACCCTGATTCTCATTTTCATTTGTCGCTAAATGATTGCAG 8:103898530
Mouse  .............................................  

Eurasian red squirrel 8:103898529 TAATAAGAAATAGTTTTCAAAGCTTGAAGCTGAAAGCTTCATGGC 8:103898485
Mouse  .............................................  

Eurasian red squirrel 8:103898484 TGGGCAGCAAGCCAGTGCCTTGGCTTTTAGCTCAGTCCAGACAAT 8:103898440
Mouse  .............................................  

Eurasian red squirrel 8:103898439 TTATCCAATACAATCTACTGAATTGTCCTCTCTTCCTGCTTCTGA 8:103898395
Mouse  .............................................  

Eurasian red squirrel 8:103898394 ATTTGAAACACTTGATAGTTGTAGTGGTCTAGTCTACAATAATTT 8:103898350
Mouse  .............................................  

Eurasian red squirrel 8:103898349 GATGCTGTGTTCCAGCTTCTAACTCTGCTGCCTCAGTCATTACAA 8:103898305
Mouse  .............................................  

Eurasian red squirrel 8:103898304 ACGAATAAAGGCATGGAGTCCTAATCCAAAATTATTCATCACATT 8:103898260
Mouse  .............................................  

Eurasian red squirrel 8:103898259 CTCAGCATAAGCCCTCCACCCAGTTAATTGGCTTTTCTTGTCTGG 8:103898215
Mouse  .............................................  

Eurasian red squirrel 8:103898214 TTGCCAATAGCTACATCCACTCACTTCCCCCACGGGAAGCCTCTC 8:103898170
Mouse  .............................................  

Eurasian red squirrel 8:103898169 TCAGAGATGTTTGCTGTTTAGCCAGGATGTGTTTTAATTCCTGAG 8:103898125
Mouse  .............................................  

Eurasian red squirrel 8:103898124 GACAAAAGGAACAGGCAGCCCTGAGGCAGCCCCCACCTACCTCAC 8:103898080
Mouse  .............................................  

Eurasian red squirrel 8:103898079 CCTGGGCTCATTGACCTCTGAGCCTGGGATTAGTAGAGGAGCTGG 8:103898035
Mouse  .............................................  

Eurasian red squirrel 8:103898034 CTGGATTCCAAGCAAGGAATTGAAGGTTGATGTGAATTAGGAAAT 8:103897990
Mouse  .............................................  

Eurasian red squirrel 8:103897989 GGGAAGTAAGCTGCTCCCTCTCCTTCCTTATGCCCCTTCCATTCA 8:103897945
Mouse  .............................................  

Eurasian red squirrel 8:103897944 CCTATCAGTCAGCCATCCATAAACCTGCCTTGTAAATGAGAGCCC 8:103897900
Mouse  .............................................  

Eurasian red squirrel 8:103897899 AGTACAGAATTTTAAAACATTATGTTAATACATCACTGATGTAAA 8:103897855
Mouse  .............................................  

Eurasian red squirrel 8:103897854 ATTATGTTTTAAAAAGTAAGACATTATCCTTAACACTGCCCTCCT 8:103897810
Mouse  .............................................  

Eurasian red squirrel 8:103897809 CCCTGTTACTTTTGGATCATGTATTTTGCCTTTAGTTTGCTGATT 8:103897765
Mouse  .............................................  

Eurasian red squirrel 8:103897764 AGAGAGACAATACTTTTGGTTTTCCAGGTGGCAACTTCAGGAAGT 8:103897720
Mouse  .............................................  

Eurasian red squirrel 8:103897719 TGTCTTGGAGCTCAAGGATGGGCAGAAGATTCCCTTGTTCATGGT 8:103897675
Mouse  .............................................  

Eurasian red squirrel 8:103897674 CAGTGAGAAGAATGGTGATATAGTAAACGAGTAAAAGAGATTAGA 8:103897630
Mouse  .............................................  

Eurasian red squirrel 8:103897629 CTATCAGCATTCTTCAAGTTTGGCCTCTCTCATCTCCACCATGTG 8:103897585
Mouse  .............................................  

Eurasian red squirrel 8:103897584 CTCACCAGGAATGACAGCCATCTGCGTGCAGACACTCCTGTGAGC 8:103897540
Mouse  .............................................  

Eurasian red squirrel 8:103897539 CAGTTCTCTTGGCTCTATGTGTAGGCATATGGTAATTTGGGTATA 8:103897495
Mouse  .............................................  

Eurasian red squirrel 8:103897494 ATTCTAATTTGACCACAAACAAATGCTATTGTGGTTTTTAAACTG 8:103897450
Mouse  .............................................  

Eurasian red squirrel 8:103897449 AATCAGAAAAACTTTTATTATAAGTTTATACGGTAATTTATTTAA 8:103897405
Mouse  .............................................  

Eurasian red squirrel 8:103897404 GTGGATGTATGTATATAAAAGCAAAGCCAAGATAAAAGAAATCAG 8:103897360
Mouse  .............................................  

Eurasian red squirrel 8:103897359 TATTAATAATAGAATGTTGATCTCTGCTGGTTCTCATTTTCTTTC 8:103897315
Mouse  .............................................  

Eurasian red squirrel 8:103897314 TTTTCCTCTCCCCACCCCTTCCCCATAATTTTTAGTTCCATCAAT 8:103897270
Mouse  .............................................  

Eurasian red squirrel 8:103897269 AAAAAGGCTGAGGCTGGCTAGCA  8:103897247
Mouse  .......................
