# Supplementary material for: Recurrent erosion of COA1/MITRAC15 exemplifies conditional gene dispensability in oxidative phosphorylation
Source: Sci Rep. 2021 Dec 24;11:24437. doi: 10.1038/s41598-021-04077-y (PMC8709867; doi:10.1038/s41598-021-04077-y)
Supplement: Supplementary file 3 — Supplementary Information 3. [file 41598_2021_4077_MOESM3_ESM.zip › Supplementary Dataset/COA1_GENE-main_Github_use_winRAR_to_open.zip!/COA1_GENE/supplementary_figure_data/supplementary_boxplot_gcpercentage_exonwise/exon3.pdf]

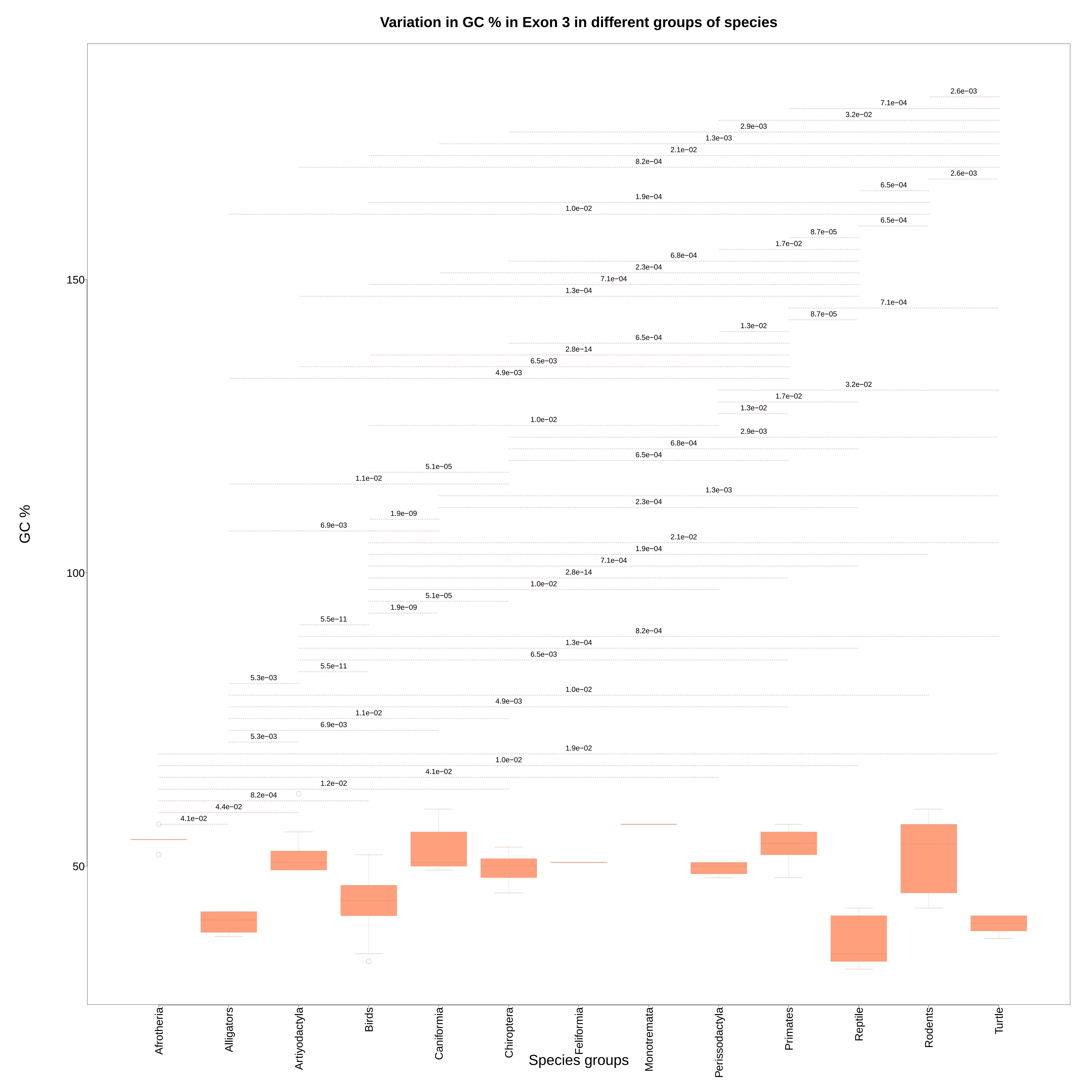

Variation in GC % in Exon 3 in different groups of species
GC %
Species groups
Afrotheria
Alligators
Artiyodactyla
Birds
Caniformia
Chiroptera
Feliformia
Monotremata
Perissodactyla
Primates
Reptile
Rodents
Turtle
50
100
150
