# Supplementary material for: Recurrent erosion of COA1/MITRAC15 exemplifies conditional gene dispensability in oxidative phosphorylation
Source: Sci Rep. 2021 Dec 24;11:24437. doi: 10.1038/s41598-021-04077-y (PMC8709867; doi:10.1038/s41598-021-04077-y)
Supplement: Supplementary file 3 — Supplementary Information 3. [file 41598_2021_4077_MOESM3_ESM.zip › Supplementary Dataset/COA1_GENE-main_Github_use_winRAR_to_open.zip!/COA1_GENE/Geneloss_timing/background_species_effect/rodent_primate_combination_final/background_effect.pdf]

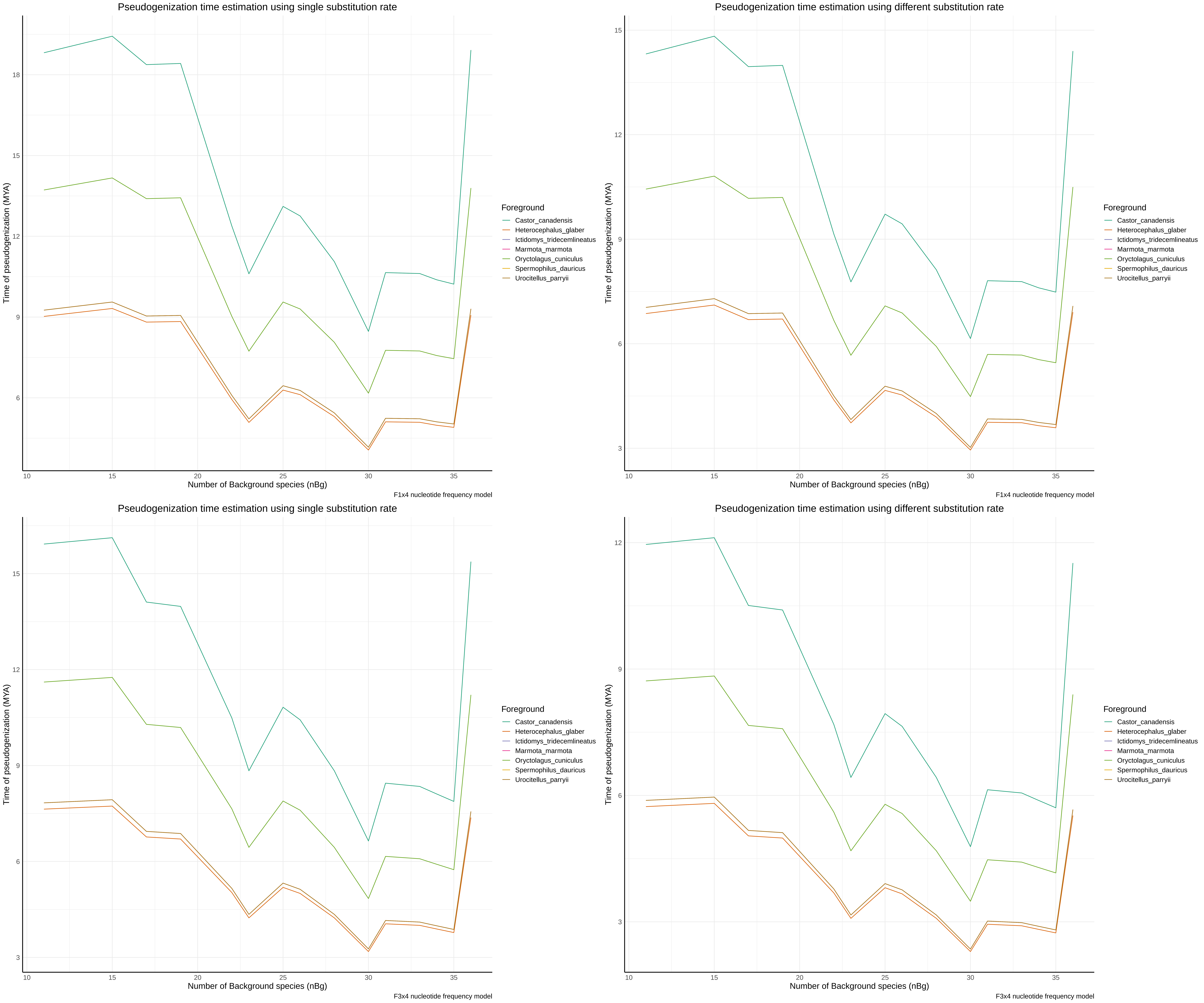
Pseudogenization time estimation using single substitution rate
Time of pseudogenization (MYA)
18
15
12
9
6
10
15
20
25
30
35
Number of Background species (nBg)
F1x4 nucleotide frequency model
Foreground
Castor_canadensis
Heterocephalus_glaber
Ictidomys_tridecemlineatus
Marmota_marmota
Oryctolagus_cuniculus
Spermophilus_dauricus
Urocitellus_parryii
Pseudogenization time estimation using different substitution rate
Time of pseudogenization (MYA)
15
12
9
6
3
10
15
20
25
30
35
Number of Background species (nBg)
F1x4 nucleotide frequency model
Foreground
Castor_canadensis
Heterocephalus_glaber
Ictidomys_tridecemlineatus
Marmota_marmota
Oryctolagus_cuniculus
Spermophilus_dauricus
Urocitellus_parryii
Pseudogenization time estimation using single substitution rate
Time of pseudogenization (MYA)
15
12
9
6
3
10
15
20
25
30
35
Number of Background species (nBg)
F3x4 nucleotide frequency model
Foreground
Castor_canadensis
Heterocephalus_glaber
Ictidomys_tridecemlineatus
Marmota_marmota
Oryctolagus_cuniculus
Spermophilus_dauricus
Urocitellus_parryii
Pseudogenization time estimation using different substitution rate
Time of pseudogenization (MYA)
12
9
6
3
10
15
20
25
30
35
Number of Background species (nBg)
F3x4 nucleotide frequency model
Foreground
Castor_canadensis
Heterocephalus_glaber
Ictidomys_tridecemlineatus
Marmota_marmota
Oryctolagus_cuniculus
Spermophilus_dauricus
Urocitellus_parryii
