# Supplementary material for: Recurrent erosion of COA1/MITRAC15 exemplifies conditional gene dispensability in oxidative phosphorylation
Source: Sci Rep. 2021 Dec 24;11:24437. doi: 10.1038/s41598-021-04077-y (PMC8709867; doi:10.1038/s41598-021-04077-y)
Supplement: Supplementary file 3 — Supplementary Information 3. [file 41598_2021_4077_MOESM3_ESM.zip › Supplementary Dataset/COA1_GENE-main_Github_use_winRAR_to_open.zip!/COA1_GENE/Geneloss_timing/background_species_effect/rodent_primate_combination_final/rodent_primate_combination.nwk.pdf]

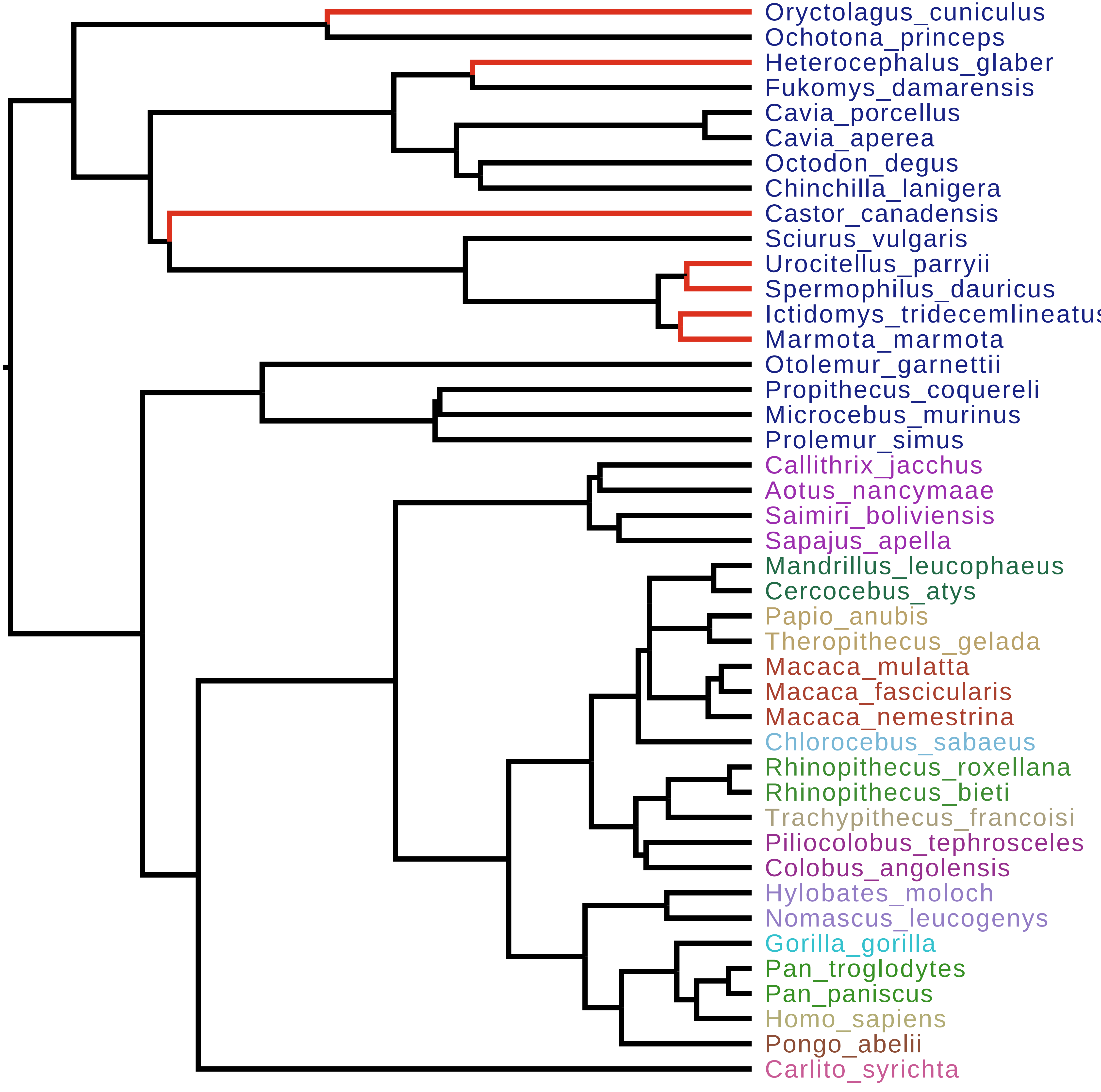
Oryctolagus_cuniculus
Ochotona_princeps
Heterocephalus_glaber
Fukomys_damarensis
Cavia_porcellus
Cavia_aperea
Octodon_degus
Chinchilla_lanigera
Castor_canadensis
Sciurus_vulgaris
Urocitellus_parryii
Spermophilus_dauricus
Ictidomys_tridecemlineatus
Marmota_marmota
Otolemur_garnettii
Propithecus_coquereli
Microcebus_murinus
Prolemur_simus
Callithrix_jacchus
Aotus_nancymaae
Saimiri_boliviensis
Sapajus_apella
Mandrillus_leucophaeus
Cercocebus_atys
Papio_anubis
Theropithecus_gelada
Macaca_mulatta
Macaca_fascicularis
Macaca_nemestrina
Chlorocebus_sabaeus
Rhinopithecus_roxellana
Rhinopithecus_bieti
Trachypithecus_francoisi
Piliocolobus_tephrosceles
Colobus_angolensis
Hylobates_moloch
Nomascus_leucogenys
Gorilla_gorilla
Pan_troglodytes
Pan_paniscus
Homo_sapiens
Pongo_abelii
Carlito_syrichta
