# Supplementary material for: Recurrent erosion of COA1/MITRAC15 exemplifies conditional gene dispensability in oxidative phosphorylation
Source: Sci Rep. 2021 Dec 24;11:24437. doi: 10.1038/s41598-021-04077-y (PMC8709867; doi:10.1038/s41598-021-04077-y)
Supplement: Supplementary file 3 — Supplementary Information 3. [file 41598_2021_4077_MOESM3_ESM.zip › Supplementary Dataset/COA1_GENE-main_Github_use_winRAR_to_open.zip!/COA1_GENE/Geneloss_timing/background_species_effect/birds_bg_effect_rooted_final/background_effect.pdf]

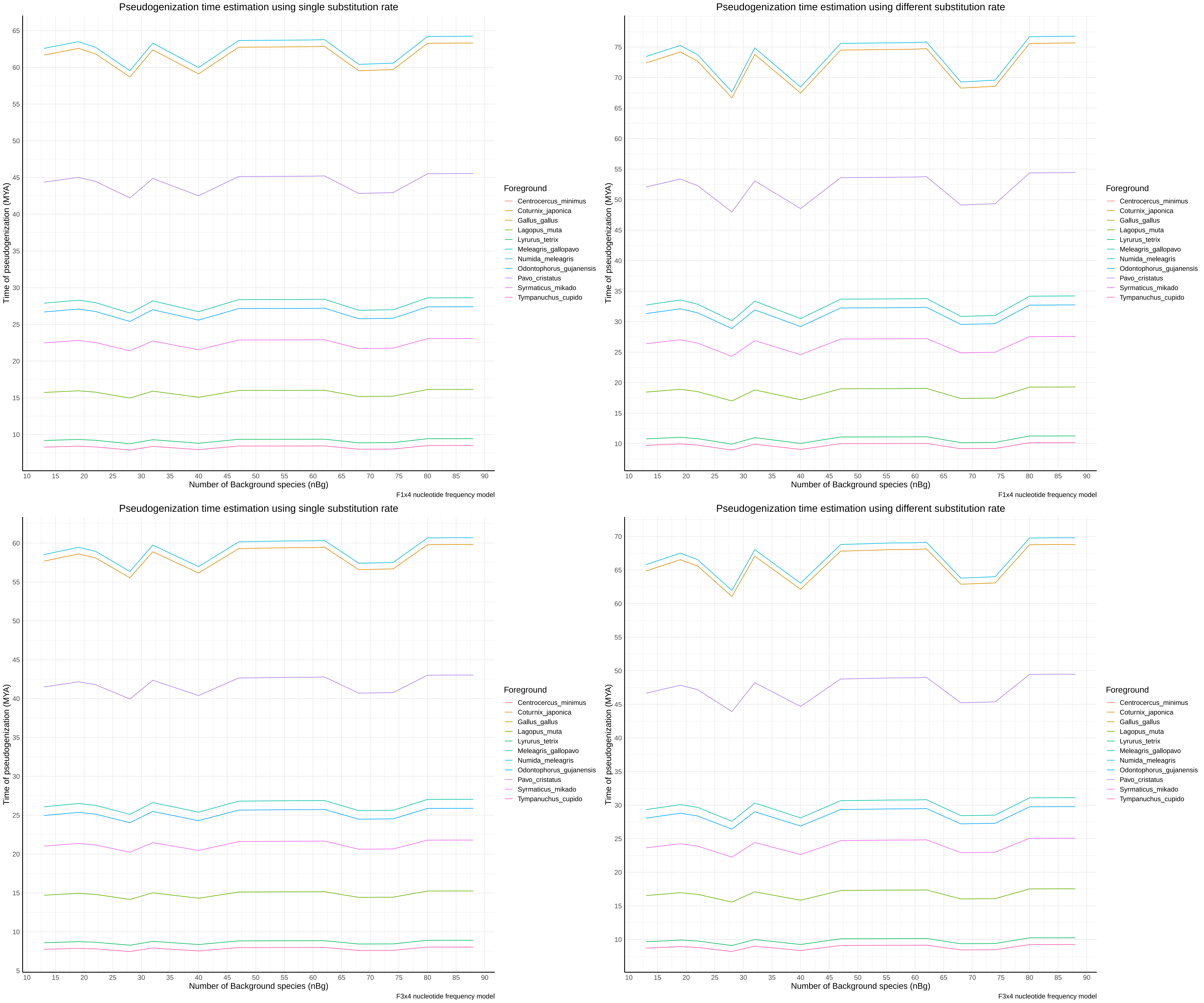
Pseudogenization time estimation using single substitution rate
Time of pseudogenization (MYA)
Number of Background species (nBg)
Foreground
Centrocercus_minimus
Coturnix_japonica
Gallus_gallus
Lagopus_muta
Lyrurus_tetrix
Meleagris_gallopavo
Numida_meleagris
Odontophorus_gujanensis
Pavo_cristatus
Syrmaticus_mikado
Tympanuchus_cupido
F1x4 nucleotide frequency model
Pseudogenization time estimation using different substitution rate
Time of pseudogenization (MYA)
Number of Background species (nBg)
Foreground
Centrocercus_minimus
Coturnix_japonica
Gallus_gallus
Lagopus_muta
Lyrurus_tetrix
Meleagris_gallopavo
Numida_meleagris
Odontophorus_gujanensis
Pavo_cristatus
Syrmaticus_mikado
Tympanuchus_cupido
F1x4 nucleotide frequency model
Pseudogenization time estimation using single substitution rate
Time of pseudogenization (MYA)
Number of Background species (nBg)
Foreground
Centrocercus_minimus
Coturnix_japonica
Gallus_gallus
Lagopus_muta
Lyrurus_tetrix
Meleagris_gallopavo
Numida_meleagris
Odontophorus_gujanensis
Pavo_cristatus
Syrmaticus_mikado
Tympanuchus_cupido
F3x4 nucleotide frequency model
Pseudogenization time estimation using different substitution rate
Time of pseudogenization (MYA)
Number of Background species (nBg)
Foreground
Centrocercus_minimus
Coturnix_japonica
Gallus_gallus
Lagopus_muta
Lyrurus_tetrix
Meleagris_gallopavo
Numida_meleagris
Odontophorus_gujanensis
Pavo_cristatus
Syrmaticus_mikado
Tympanuchus_cupido
F3x4 nucleotide frequency model
