# Supplementary material for: Recurrent erosion of COA1/MITRAC15 exemplifies conditional gene dispensability in oxidative phosphorylation
Source: Sci Rep. 2021 Dec 24;11:24437. doi: 10.1038/s41598-021-04077-y (PMC8709867; doi:10.1038/s41598-021-04077-y)
Supplement: Supplementary file 3 — Supplementary Information 3. [file 41598_2021_4077_MOESM3_ESM.zip › Supplementary Dataset/COA1_GENE-main_Github_use_winRAR_to_open.zip!/COA1_GENE/Geneloss_timing/background_species_effect/birds_bg_effect_rooted_final/birds_background_effect.pdf]

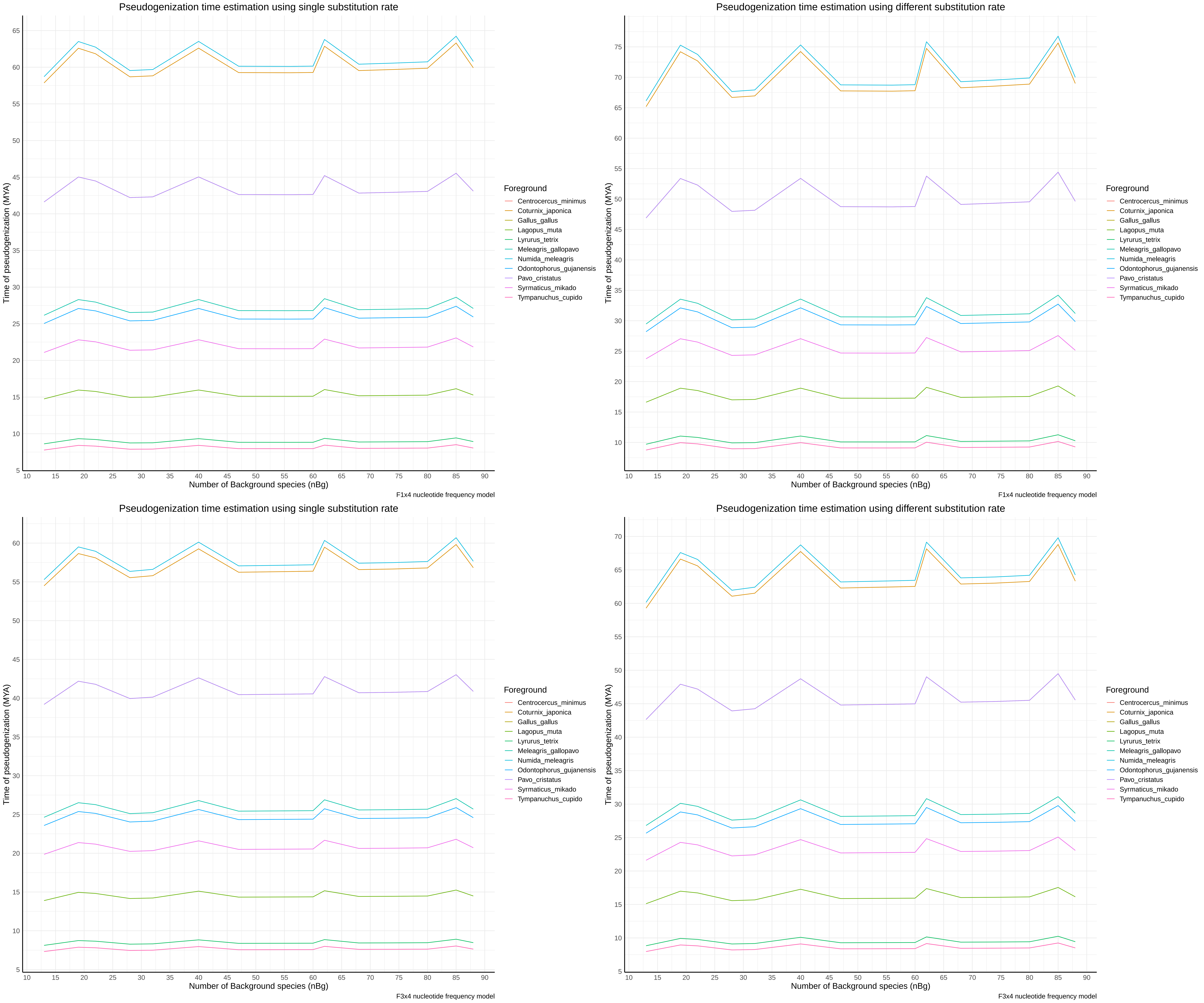
Pseudogenization time estimation using single substitution rate
Time of pseudogenization (MYA)
Number of Background species (nBg)
F1x4 nucleotide frequency model
Pseudogenization time estimation using different substitution rate
Time of pseudogenization (MYA)
Number of Background species (nBg)
F1x4 nucleotide frequency model
Pseudogenization time estimation using single substitution rate
Time of pseudogenization (MYA)
Number of Background species (nBg)
F3x4 nucleotide frequency model
Pseudogenization time estimation using different substitution rate
Time of pseudogenization (MYA)
Number of Background species (nBg)
F3x4 nucleotide frequency model
Foreground
Centrocercus_minimus
Coturnix_japonica
Gallus_gallus
Lagopus_muta
Lyrurus_tetrix
Meleagris_gallopavo
Numida_meleagris
Odontophorus_gujanensis
Pavo_cristatus
Syrmaticus_mikado
Tympanuchus_cupido
