# Supplementary material for: Recurrent erosion of COA1/MITRAC15 exemplifies conditional gene dispensability in oxidative phosphorylation
Source: Sci Rep. 2021 Dec 24;11:24437. doi: 10.1038/s41598-021-04077-y (PMC8709867; doi:10.1038/s41598-021-04077-y)
Supplement: Supplementary file 3 — Supplementary Information 3. [file 41598_2021_4077_MOESM3_ESM.zip › Supplementary Dataset/Supplementary_File_S5.zip!/Phyre2_results_Q9GZY4_COA1_human_secondary_structure.pdf]

| | |
|---|---|
| Email | nagarjun@iiserb.ac.in |
| Description | _sp_Q9GZY4_COA1_HUMAN_Cytochrome_c_oxidase_assembly_factor_1_homolog_OS_Homo_sapiens_OX_9606_GN_COA1_PE_1_SV_1 |
| Date | Fri Mar 26 09:33:32 GMT 2021 |
| Unique Job ID | 3e54bf757caad22f |

## Secondary structure and disorder prediction

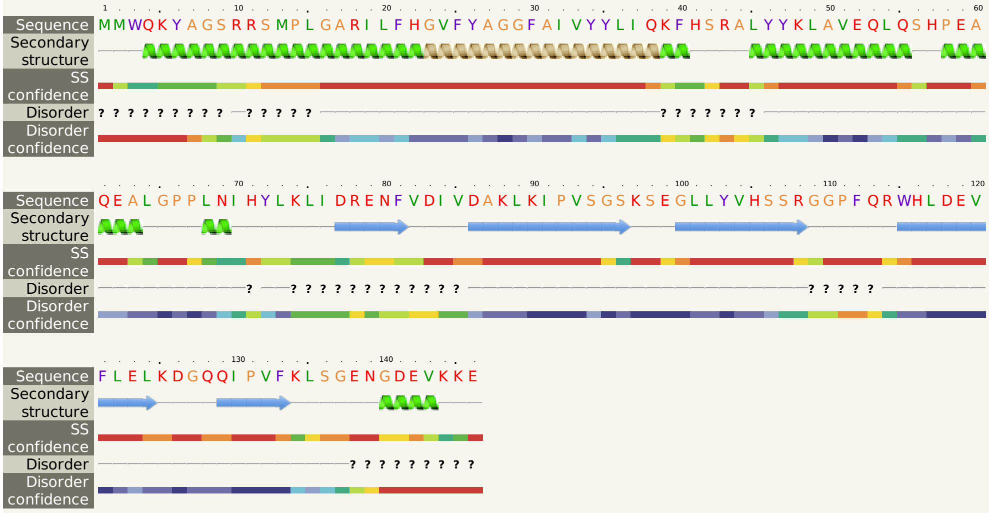


Confidence Key

High(9) Low (0)

? Disordered ( 33%)

Alpha helix ( 41%)

Beta strand ( 27%)

TM helix ( 11%)
