# Supplementary material for: Recurrent erosion of COA1/MITRAC15 exemplifies conditional gene dispensability in oxidative phosphorylation
Source: Sci Rep. 2021 Dec 24;11:24437. doi: 10.1038/s41598-021-04077-y (PMC8709867; doi:10.1038/s41598-021-04077-y)
Supplement: Supplementary file 3 — Supplementary Information 3. [file 41598_2021_4077_MOESM3_ESM.zip › Supplementary Dataset/Supplementary_File_S5.zip!/Phyre2_results_Q9GZY4_COA1_human_domain_analysis.pdf]

Email	nagarjun@iiserb.ac.in
Description	_sp_Q9GZY4_COA1_HUMAN_Cytochrome_c_oxidase_assembly_factor_1_homolog_OS_Homo_sapiens_OX_9606_GN_COA1_PE_1_SV_1
Date	Fri Mar 26 09:33:32 GMT 2021
Unique Job ID	3e54bf757caad22f

Domain analysis

Rank	Aligned region
1	c2ciuA_
2	c6k7dA_
3	c5e06A_
4	c2n2aA_
5	c2lnmA_
6	d2nlua1
7	c5csaA_
8	c2fynO_
9	c2llvA_
10	c5ldwg_
11	c6gvwJ_
12	d1qmga1
13	c3hzpA_
14	d1ppjw_
15	c5iauD_
16	c4b7lB_
17	c5oqtC_
18	c6f34C_
19	c4hppA_
20	c3qajL_
21	
22	
23	
24	
25	
26	
27	
28	
29	
30	
31	
32	
33	
34	
35	
36	
37	
38	
39	
40	
41	
42	
43	
44	
45	
46	
47	
48	
49	
50	
51	
52	
53	
54	
55	
