# Supplementary material for: Recurrent erosion of COA1/MITRAC15 exemplifies conditional gene dispensability in oxidative phosphorylation
Source: Sci Rep. 2021 Dec 24;11:24437. doi: 10.1038/s41598-021-04077-y (PMC8709867; doi:10.1038/s41598-021-04077-y)
Supplement: Supplementary file 3 — Supplementary Information 3. [file 41598_2021_4077_MOESM3_ESM.zip › Supplementary Dataset/Supplementary_File_S5.zip!/Phyre2_results_Q9GZY4_COA1_human.pdf]

Phyre2

Email	nagarjun@iiserb.ac.in
Description	_sp_Q9GZY4_COA1_HUMAN_Cytochrome_c_oxidase_assembly_factor_1_homolog_OS_Homo_sapiens_OX_9606_GN_COA1_PE_1_SV_1
Date	Fri Mar 26 09:33:32 GMT 2021
Unique Job ID	3e54bf757caad22f

Detailed template information

#	Template	Alignment Coverage	3D Model	Confidence	% i.d.	Template Information
1	c2ciuA_	Alignment	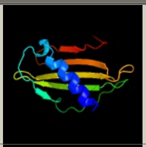	99.8	13	**PDB header:**protein transport<br>**Chain:** A: **PDB Molecule:**import inner membrane translocase subunit tim21<br>**PDBTitle:** structure of the ims domain of the mitochondrial import2 protein tim21 from s. cerevisiae
2	c6k7dA_	Alignment	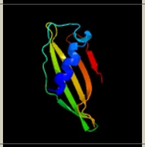	99.6	11	**PDB header:**sugar binding protein, translocase<br>**Chain:** A: **PDB Molecule:**maltose/maltodextrin-binding periplasmic protein,<br>**PDBTitle:** crystal structure of mbpapo-tim21 fusion protein with a 16-residue2 helical linker
3	c5e06A_	Alignment	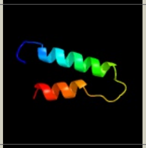	31.4	16	**PDB header:**nuclear protein<br>**Chain:** A: **PDB Molecule:**nucleocapsid protein;<br>**PDBTitle:** structure of sin nombre virus nucleoprotein in long-axis crystal form
4	c2n2aA_	Alignment	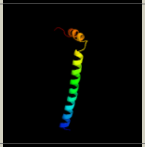	30.6	21	**PDB header:**membrane protein<br>**Chain:** A: **PDB Molecule:**receptor tyrosine-protein kinase erbb-2;<br>**PDBTitle:** spatial structure of her2/erbb2 dimeric transmembrane domain in the2 presence of cytoplasmic juxtamembrane domains
5	c2lnmA_	Alignment	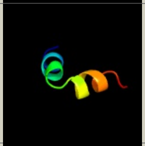	20.5	15	**PDB header:**protein transport<br>**Chain:** A: **PDB Molecule:**protein tic 40, chloroplastic;<br>**PDBTitle:** solution structure of the c-terminal np-repeat domain of tic40, a co-2 chaperone during protein import into chloroplasts
6	d2nlua1	Alignment	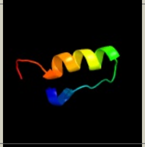	19.3	4	**Fold:**SH3-like barrel<br>**Superfamily:**Tudor/PWWP/MBT<br>**Family:**PWWP domain
7	c5csaA_	Alignment	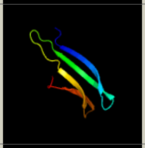	17.2	17	**PDB header:**ligase<br>**Chain:** A: **PDB Molecule:**acetyl-coa carboxylase;<br>**PDBTitle:** crystal structure of domains bt-bccp-ac1-ac5 of yeast acetyl-coa2 carboxylase
8	c2fynO_	Alignment	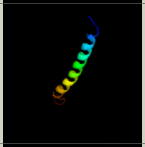	17.1	13	**PDB header:**oxidoreductase<br>**Chain:** O: **PDB Molecule:**ubiquinol-cytochrome c reductase iron-sulfur<br>**PDBTitle:** crystal structure analysis of the double mutant rhodobacter2 sphaeroides bc1 complex
9	c2llvA_	Alignment	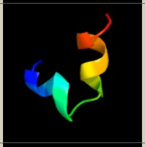	16.9	29	**PDB header:**chaperone<br>**Chain:** A: **PDB Molecule:**heat shock protein sti1;<br>**PDBTitle:** solution structure of the yeast sti1 dp1 domain
10	c5ldwg_	Alignment	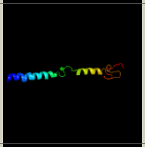	14.8	17	**PDB header:**oxidoreductase<br>**Chain:** G: **PDB Molecule:**nadh-ubiquinone oxidoreductase 75 kda subunit,<br>**PDBTitle:** structure of mammalian respiratory complex i, class1
11	c6gvwJ_	Alignment	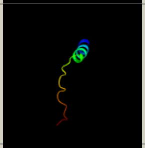	13.3	14	**PDB header:**signaling protein<br>**Chain:** J: **PDB Molecule:**brca1-a complex subunit rap80;<br>**PDBTitle:** crystal structure of the brca1-a complex
12	d1qmga1	Alignment	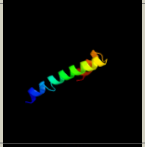	13.1	23	**Fold:**6-phosphogluconate dehydrogenase C-terminal domain-like<br>**Superfamily:**6-phosphogluconate dehydrogenase C-terminal domain-like<br>**Family:**Acetohydroxy acid isomeroreductase (ketol-acid reductoisomerase, KARI)
13	c3hzpA_	Alignment	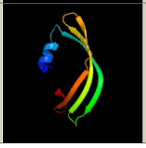	10.3	14	**PDB header:**structural genomics, unknown function<br>**Chain:** A: **PDB Molecule:**ntf2-like protein of unknown function;<br>**PDBTitle:** crystal structure of ntf2-like protein of unknown function mn2a_05052 from prochlorococcus marinus (yp_291699.1) from prochlorococcus sp.3 natl2a at 1.40 a resolution

14	d1ppjw_	Alignment	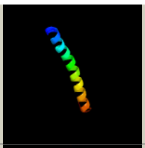	9.9	18	**Fold:**Single transmembrane helix **Superfamily:**Subunit X (non-heme 7 kDa protein) of cytochrome bc1 complex (Ubiquinol-cytochrome c reductase) **Family:**Subunit X (non-heme 7 kDa protein) of cytochrome bc1 complex (Ubiquinol-cytochrome c reductase)
15	c5iauD_	Alignment	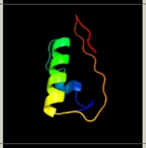	9.2	14	**PDB header:**hydrolase **Chain:** D: **PDB Molecule:**dipeptidase; **PDBTitle:** a c69-family cysteine dipeptidase from lactobacillus farciminis
16	c4b7lB_	Alignment	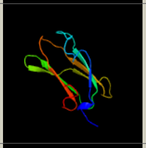	9.2	7	**PDB header:**structural protein **Chain:** B: **PDB Molecule:**filamin-b; **PDBTitle:** crystal structure of human filamin b actin binding domain2 with 1st filamin repeat
17	c5oqtC_	Alignment	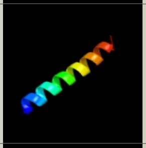	9.2	20	**PDB header:**transport protein **Chain:** C: **PDB Molecule:**uncharacterized protein ynem; **PDBTitle:** crystal structure of a bacterial cationic amino acid transporter (cat)2 homologue
18	c6f34C_	Alignment	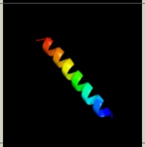	9.2	20	**PDB header:**membrane protein **Chain:** C: **PDB Molecule:**mgts; **PDBTitle:** crystal structure of a bacterial cationic amino acid transporter (cat)2 homologue bound to arginine.
19	c4hppA_	Alignment	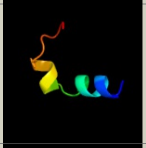	9.0	29	**PDB header:**ligase **Chain:** A: **PDB Molecule:**probable glutamine synthetase; **PDBTitle:** crystal structure of novel glutamine synthase homolog
20	c3qajL_	Alignment	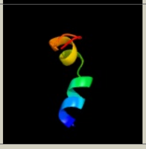	9.0	33	**PDB header:**ligase **Chain:** L: **PDB Molecule:**glutamine synthetase; **PDBTitle:** x-ray crystal structure of glutamine synthetase from bacillus subtilis2 cocrystallized with atp
21	c2kncB_	Alignment	not modelled	9.0	6	**PDB header:**cell adhesion **Chain:** B: **PDB Molecule:**integrin beta-3; **PDBTitle:** platelet integrin alfaiib-beta3 transmembrane-cytoplasmic2 heterocomplex
22	c7jrgV_	Alignment	not modelled	8.9	8	**PDB header:**electron transport **Chain:** V: **PDB Molecule:**cytochrome b-c1 complex subunit 9; **PDBTitle:** plant mitochondrial complex iii2 from vigna radiata
23	c2xxsA_	Alignment	not modelled	8.8	45	**PDB header:**protein binding **Chain:** A: **PDB Molecule:**protein mxig; **PDBTitle:** solution structure of the n-terminal domain of the shigella2 type iii secretion protein mxig
24	c6t9iE_	Alignment	not modelled	8.5	9	**PDB header:**gene regulation **Chain:** E: **PDB Molecule:**transcription initiation factor tfiid subunit 6; **PDBTitle:** cryo-em structure of transcription coactivator saga
25	c4zr7C_	Alignment	not modelled	8.5	17	**PDB header:**transferase **Chain:** C: **PDB Molecule:**sensor histidine kinase rese; **PDBTitle:** the structure of a domain of a functionally unknown protein from2 bacillus subtilis subsp. subtilis str. 168
26	c7kznI_	Alignment	not modelled	8.3	23	**PDB header:**motor protein **Chain:** I: **PDB Molecule:**dynein light chain roadblock lc7a; **PDBTitle:** outer dynein arm core subcomplex from c. reinhardtii
27	c5zazA_	Alignment	not modelled	8.3	15	**PDB header:**cell adhesion **Chain:** A: **PDB Molecule:**integrin beta-2; **PDBTitle:** solution structure of integrin b2 monomer tranmembrane domain in2 bicelle
28	d3cx5i1	Alignment	not modelled	8.0	11	**Fold:**Single transmembrane helix **Superfamily:**Subunit X (non-heme 7 kDa protein) of cytochrome bc1 complex (Ubiquinol-cytochrome c reductase) **Family:**Subunit X (non-heme 7 kDa protein) of cytochrome bc1 complex (Ubiquinol-cytochrome c reductase)
29	c4fu6A_	Alignment	not modelled	7.7	4	**PDB header:**transcription **Chain:** A: **PDB Molecule:**pc4 and sfrs1-interacting protein; **PDBTitle:** crystal structure of the psip1 pwwp domain
30	c6jnfJ_	Alignment	not modelled	7.6	30	**PDB header:**translocase **Chain:** J: **PDB Molecule:**mitochondrial import receptor subunit tom6; **PDBTitle:** cryo-em structure of the translocator of the outer mitochondrial2 membrane
31	c6jnfE_	Alignment	not modelled	7.6	30	**PDB header:**translocase **Chain:** E: **PDB Molecule:**mitochondrial import receptor subunit tom6; **PDBTitle:** cryo-em structure of the translocator of the outer mitochondrial2 membrane
32	c5dm3A_	Alignment	not modelled	7.5	29	**PDB header:**ligase **Chain:** A: **PDB Molecule:**l-glutamine synthetase; **PDBTitle:** crystal structure of glutamine synthetase from chromohalobacter2 salexigens dsm 3043(csal_0679, target efi-550015) with bound adp
33	c3p43A_	Alignment	not modelled	7.2	13	**PDB header:**hydrolase **Chain:** A: **PDB Molecule:**putative uncharacterized protein; **PDBTitle:** structure and activities of archaeal members of the ligd 3'2 phosphoesterase dna repair enzyme superfamily
34	c4a4yA_	Alignment	not modelled	6.8	45	**PDB header:**protein binding **Chain:** A: **PDB Molecule:**protein mxig; **PDBTitle:** structure of the cytosolic domain of the shigella t3ss2 component mxig
35	d1x9ya2	Alignment	not modelled	6.7	19	**Fold:**Cystatin-like **Superfamily:**Cystatin/monellin **Family:**Staphopain B, prodomain

36	d1w6ga3	Alignment	not modelled	6.7	17	**Fold:**Cystatin-like<br>**Superfamily:**Amine oxidase N-terminal region<br>**Family:**Amine oxidase N-terminal region
37	c6zywF_	Alignment	not modelled	6.5	27	**PDB header:**motor protein<br>**Chain:** F: **PDB Molecule:**dynein light chain roadblock-type 2 protein;<br>**PDBTitle:** outer dynein arm-shulin complex - overall structure (tetrahymena2 thermophila)
38	c2j7aC_	Alignment	not modelled	6.3	15	**PDB header:**oxidoreductase<br>**Chain:** C: **PDB Molecule:**cytochrome c quinol dehydrogenase nrfh;<br>**PDBTitle:** crystal structure of cytochrome c nitrite reductase nrfha complex from2 desulfovibrio vulgaris
39	d2hz5a1	Alignment	not modelled	6.3	29	**Fold:**Profilin-like<br>**Superfamily:**Roadblock/LC7 domain<br>**Family:**Roadblock/LC7 domain
40	d2cqla1	Alignment	not modelled	6.3	20	**Fold:**Ribosomal protein L6<br>**Superfamily:**Ribosomal protein L6<br>**Family:**Ribosomal protein L6
41	c2llwA_	Alignment	not modelled	6.3	10	**PDB header:**chaperone<br>**Chain:** A: **PDB Molecule:**heat shock protein sti1;<br>**PDBTitle:** solution structure of the yeast sti1 dp2 domain
42	c3j3vT_	Alignment	not modelled	6.2	21	**PDB header:**ribosome<br>**Chain:** T: **PDB Molecule:**50s ribosomal protein l23;<br>**PDBTitle:** atomic model of the immature 50s subunit from bacillus subtilis (state2 i-a)
43	d1tu2b1	Alignment	not modelled	6.2	15	**Fold:**Common fold of diphtheria toxin/transcription factors/cytochrome f<br>**Superfamily:**Cytochrome f, large domain<br>**Family:**Cytochrome f, large domain
44	c3tj6B_	Alignment	not modelled	6.0	30	**PDB header:**protein binding/toxin<br>**Chain:** B: **PDB Molecule:**antigenic heat-stable 120 kda protein;<br>**PDBTitle:** human vinculin head domain (vh1, residues 1-258) in complex with the2 vinculin binding site of the surface cell antigen 4 (sca4-vbs-c;3 residues 812-835) from rickettsia rickettsii
45	d2dk4a1	Alignment	not modelled	5.8	23	**Fold:**LEM/SAP HeH motif<br>**Superfamily:**PRP4-like<br>**Family:**PRP4-like
46	d1v54i_	Alignment	not modelled	5.8	17	**Fold:**Single transmembrane helix<br>**Superfamily:**Mitochondrial cytochrome c oxidase subunit VIc<br>**Family:**Mitochondrial cytochrome c oxidase subunit VIc
47	c5hizA_	Alignment	not modelled	5.8	19	**PDB header:**rna binding protein<br>**Chain:** A: **PDB Molecule:**non-structural protein 9;<br>**PDBTitle:** the structure of pedv nsp9
48	c6prkA_	Alignment	not modelled	5.7	28	**PDB header:**metal binding protein<br>**Chain:** A: **PDB Molecule:**ricf;<br>**PDBTitle:** x-ray crystal structure of bacillus subtilis rica in complex with ricf
49	c2wvrB_	Alignment	not modelled	5.5	44	**PDB header:**replication<br>**Chain:** B: **PDB Molecule:**geminin;<br>**PDBTitle:** human cdt1:geminin complex
50	d2iaza1	Alignment	not modelled	5.4	22	**Fold:**YheA-like<br>**Superfamily:**YheA/YmcA-like<br>**Family:**YheA-like
51	c7kznJ_	Alignment	not modelled	5.4	23	**PDB header:**motor protein<br>**Chain:** J: **PDB Molecule:**dynein light chain roadblock lc7b;<br>**PDBTitle:** outer dynein arm core subcomplex from c. reinhardtii
52	c3msqC_	Alignment	not modelled	5.2	20	**PDB header:**biosynthetic protein<br>**Chain:** C: **PDB Molecule:**putative ubiquinone biosynthesis protein;<br>**PDBTitle:** crystal structure of a putative ubiquinone biosynthesis protein2 (npun02000094) from nostoc punctiforme pcc 73102 at 2.85 a resolution
53	c2a5hC_	Alignment	not modelled	5.2	22	**PDB header:**isomerase<br>**Chain:** C: **PDB Molecule:**l-lysine 2,3-aminomutase;<br>**PDBTitle:** 2.1 angstrom x-ray crystal structure of lysine-2,3-aminomutase from2 clostridium subterminale sb4, with michaelis analog (l-alpha-lysine3 external aldimine form of pyridoxal-5'-phosphate).
54	c2pihA_	Alignment	not modelled	5.1	17	**PDB header:**structural genomics, unknown function<br>**Chain:** A: **PDB Molecule:**protein ymca;<br>**PDBTitle:** crystal structure of protein ymca from bacillus subtilis, northeast2 structural genomics target sr375
55	d2piha1	Alignment	not modelled	5.1	17	**Fold:**YheA-like<br>**Superfamily:**YheA/YmcA-like<br>**Family:**YmcA-like
