# Supplementary material for: Recurrent erosion of COA1/MITRAC15 exemplifies conditional gene dispensability in oxidative phosphorylation
Source: Sci Rep. 2021 Dec 24;11:24437. doi: 10.1038/s41598-021-04077-y (PMC8709867; doi:10.1038/s41598-021-04077-y)
Supplement: Supplementary file 3 — Supplementary Information 3. [file 41598_2021_4077_MOESM3_ESM.zip › Supplementary Dataset/Supplementary_File_S5.zip!/SwissModel_report_Human_COA1_Q9GZY4.pdf]

SWISS-MODEL Homology Modelling Report

Model Building Report

This document lists the results for the homology modelling project "Untitled Project" submitted to SWISS-MODEL workspace on March 26, 2021, 10:30 a.m..The submitted primary amino acid sequence is given in Table T1.

If you use any results in your research, please cite the relevant publications:

- Waterhouse, A., Bertoni, M., Bienert, S., Studer, G., Tauriello, G., Gumienny, R., Heer, F.T., de Beer, T.A.P., Rempfer, C., Bordoli, L., Lepore, R., Schwede, T. SWISS-MODEL: homology modelling of protein structures and complexes. Nucleic Acids Res. 46(W1), W296-W303 (2018). doi>
- Bienert, S., Waterhouse, A., de Beer, T.A.P., Tauriello, G., Studer, G., Bordoli, L., Schwede, T. The SWISS-MODEL Repository - new features and functionality. Nucleic Acids Res. 45, D313-D319 (2017). doi>
- Studer, G., Tauriello, G., Bienert, S., Biasini, M., Johner, N., Schwede, T. ProMod3 - A versatile homology modelling toolbox. PLOS Comp. Biol. 17(1), e1008667 (2021). doi>
- Studer, G., Rempfer, C., Waterhouse, A.M., Gumienny, G., Haas, J., Schwede, T. QMEANDisCo - distance constraints applied on model quality estimation. Bioinformatics 36, 1765-1771 (2020). doi>
- Bertoni, M., Kiefer, F., Biasini, M., Bordoli, L., Schwede, T. Modeling protein quaternary structure of homo- and hetero-oligomers beyond binary interactions by homology. Scientific Reports 7 (2017). doi>

Results

The SWISS-MODEL template library (SMTL version 2021-03-25, PDB release 2021-03-19) was searched with BLAST (Camacho et al.) and HHblits (Steinegger et al.) for evolutionary related structures matching the target sequence in Table T1. For details on the template search, see Materials and Methods. Overall 28 templates were found (Table T2).

Models

The following models were built (see Materials and Methods "Model Building"):

Model #02	File	Built with	Oligo-State	Ligands	GMQE	QMEAN
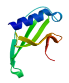	PDB	ProMod3 3.2.0	monomer	None	0.31	-2.28

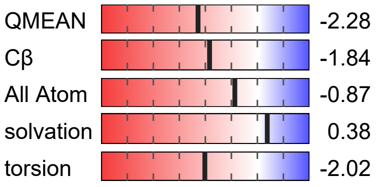

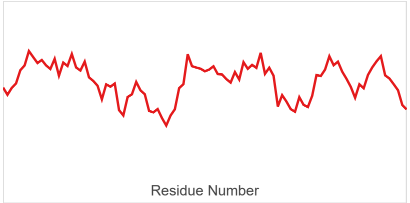

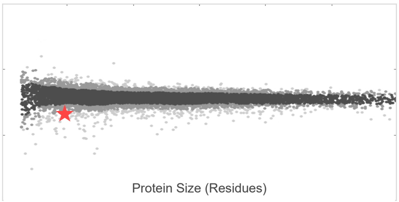

Template	Seq Identity	Oligo-state	QSQE	Found by	Method	Resolution	Seq Similarity	Range	Coverage	Description
2ciu.1.A	10.53	monomer	0.00	HHblits	X-ray	1.60Å	0.27	44 - 138	0.65	IMPORT INNER MEMBRANE TRANSLOCASE SUBUNIT TIM21 MITOCHONDRIAL

The template contained no ligands.

```
Target    MMWQKYAGSRRSMPLGARILFHGVFYAGGFAIVYYLIQKFHSRALYYKLAVEQLQSHPEAQEALG--------PPLNI-H
2ciu.1.A  -------------------------------------------TQLFNRAVSMVEKNKDIRSLLQCDDGITGKERLKAYG

Target    YLKLID------------RENFVDIVDAKLKIPVSGSKSEGLLYVHSSRGGPFQRWHLDEVFLELKDGQQIPVFKLSGEN
2ciu.1.A  ELITNDKWTRNRPIVSTKKLDKEGRTHHYMRFHVESKKKIALVHLEAKESKQNYQPDFINMYVDVPGEKRYYLIKPKLH-

Target    GDEVKKE
2ciu.1.A  -------
```

Model #03	File	Built with	Oligo-State	Ligands	GMQE	QMEAN

Model #03	File	Built with	Oligo-State	Ligands	GMQE	QMEAN
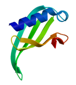	PDB	ProMod3 3.2.0	monomer	None	0.29	-2.60

QMEAN 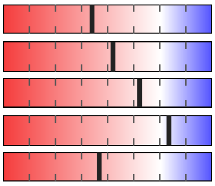 -2.60
Cβ -1.79
All Atom -0.77
solvation 0.35
torsion -2.32

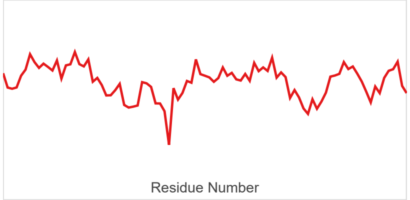

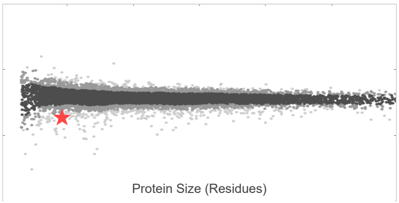

Template	Seq Identity	Oligo-state	QSQE	Found by	Method	Resolution	Seq Similarity	Range	Coverage	Description
6k7f.1.A	10.00	monomer	0.00	HHblits	X-ray	1.80Å	0.27	44 - 134	0.62	Maltose/maltodextrin-binding periplasmic protein,Mitochondrial import inner membrane translocase subunit TIM21

Excluded ligands

Ligand Name.Number	Reason for Exclusion	Description
GLC-GLC.1	Binding site not conserved.	alpha-D-glucopyranose-(1-4)-alpha-D-glucopyranose

```
Target    MMWQKYAGSRRSMPLGARILFHGVFYAGGFAIVYYLIQKFHSRALYYKLAVEQLQSHPEAQEALGP--------PLNI-H
6k7f.1.A  -------------------------------------------TQLFNRAVSMVEKNKDIRSLLQCDDGITGKERLKAYG

Target    YLK-------------LIDRENFVDIVDAKLKIPVSGSKSEGLLYVHSSRGGPFQRWHLDEVFLELKDGQQIPVFKLSGE
6k7f.1.A  ELITNDKWTRNRPIVSTKKLDK-EGRTHHYMRFHVESKKKIALVHLEAKESKQNYQPDFINMYVDVPGEKRYYLIK----

Target    NGDEVKKE
6k7f.1.A  --------
```

Model #01	File	Built with	Oligo-State	Ligands	GMQE	QMEAN
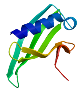	PDB	ProMod3 3.2.0	monomer	None	0.29	-4.16

QMEAN 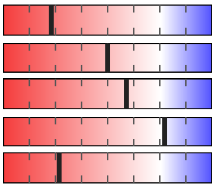 -4.16
Cβ -1.99
All Atom -1.28
solvation 0.18
torsion -3.86

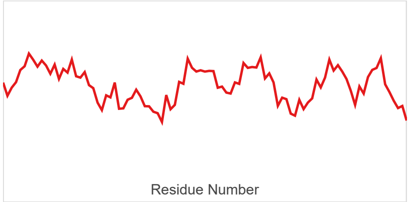

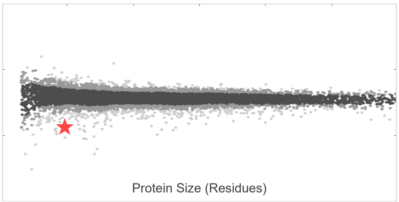

Template	Seq Identity	Oligo-state	QSQE	Found by	Method	Resolution	Seq Similarity	Range	Coverage	Description
2mf7.1.A	10.53	monomer	0.00	HHblits	NMR	-	0.27	44 - 138	0.65	Mitochondrial import inner membrane translocase subunit TIM21

The template contained no ligands.

```
Target    MMWQKYAGSRRSMPLGARILFHGVFYAGGFAIVYYLIQKFHSRALYYKLAVEQLQSHPEAQEALG--------PPLNI-H
2mf7.1.A  -------------------------------------------TQLFNRAVSMVEKNKDIRSLLQCDDGITGKERLKAYG

Target    YLKLID------------RENFVDIVDAKLKIPVSGSKSEGLLYVHSSRGGPFQRWHLDEVFLELKDGQQIPVFKLSGEN
2mf7.1.A  ELITNDKWTRNRPIVSTKKLDKEGRTHHYMRFHVESKKKIALVHLEAKESKQNYQPDFINMYVDVPGEKRYYLIKPKLH-

Target    GDEVKKE
2mf7.1.A  -------
```

Model #04	File	Built with	Oligo-State	Ligands	GMQE	QMEAN
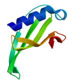	PDB	ProMod3 3.2.0	monomer	None	0.28	-3.19

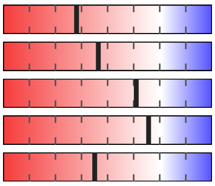

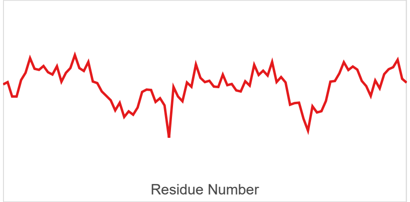

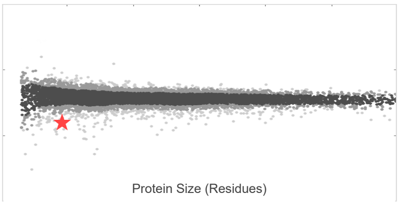

Template	Seq Identity	Oligo-state	QSQE	Found by	Method	Resolution	Seq Similarity	Range	Coverage	Description
6k7d.1.A	10.00	monomer	0.00	HHblits	X-ray	2.00Å	0.27	44 - 134	0.62	Maltose/maltodextrin-binding periplasmic protein,Mitochondrial import inner membrane translocase subunit TIM21

The template contained no ligands.

```
Target     MMWQKYAGSRRSMPLGARILFHGVFYAGGFAIVYYLIQKFHSRALYYKLAVEQLQSHPEAQEALG--------PPLNI-H
6k7d.1.A   -------------------------------------------TQLFNRAVSMVEKNKDIRSLLQCDDGITGKERLKAYG

Target     YLK-L------------IDRENFVDIVDAKLKIPVSGSKSEGLLYVHSSRGGPFQRWHLDEVFLELKDGQQIPVFKLSGE
6k7d.1.A   ELITNDKWTRNRPIVSTKKLDK-EGRTHHYMRFHVESKKKIALVHLEAKESKQNYQPDFINMYVDVPGEKRYYLIK----

Target     NGDEVKKE
6k7d.1.A   --------
```

Model #06	File	Built with	Oligo-State	Ligands	GMQE	QMEAN
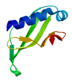	PDB	ProMod3 3.2.0	monomer	None	0.25	-3.74

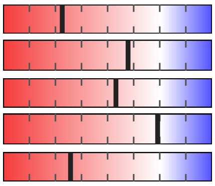

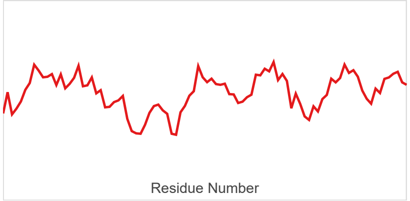

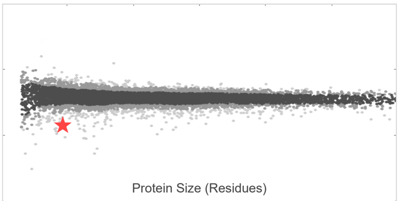

Template	Seq Identity	Oligo-state	QSQE	Found by	Method	Resolution	Seq Similarity	Range	Coverage	Description
6k8q.1.A	10.87	monomer	0.00	HHblits	NMR	-	0.27	43 - 134	0.63	Mitochondrial import inner membrane translocase subunit TIM21

The template contained no ligands.

```
Target     MMWQKYAGSRRSMPLGARILFHGVFYAGGFAIVYYLIQKFHSRALYYKLAVEQLQSHPEAQEALG--------PPLNI-H
6k8q.1.A   ------------------------------------------DTQLFNRAVSMVEKNKDIRSLLQCDDGITGKERLKAYG

Target     YLKLID-----------RE-NFVDIVDAKLKIPVSGSKSEGLLYVHSSRGGPFQRWHLDEVFLELKDGQQIPVFKLSGEN
6k8q.1.A   ELITNDKWTRNRPIVSTKKLDKEGRTHHYMRFHVESKKKIALVHLEAKESKQNYQPDFINMYVDVPGEKRYYLIK-----

Target     GDEVKKE
6k8q.1.A   -------
```

Model #05	File	Built with	Oligo-State	Ligands	GMQE	QMEAN
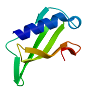	PDB	ProMod3 3.2.0	monomer	None	0.25	-2.37

QMEAN -2.37
Cβ -0.46
All Atom -0.33
solvation 0.06
torsion -2.36

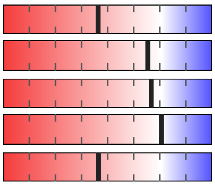

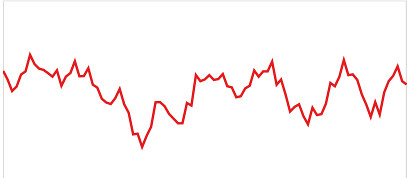

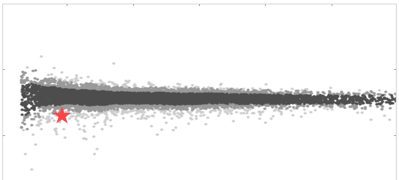

Template	Seq Identity	Oligo-state	QSQE	Found by	Method	Resolution	Seq Similarity	Range	Coverage	Description
6k7e.1.A	10.99	monomer	0.00	HHblits	X-ray	1.53Å	0.27	44 - 134	0.62	Maltose/maltodextrin-binding periplasmic protein,Mitochondrial import inner membrane translocase subunit TIM21

The template contained no ligands.

```
Target     MMWQKYAGSRRSMPLGARILFHGVFYAGGFAIVYYLIQKFHSRALYYKLAVEQLQSHPEAQEALGP--------PLNI-H
6k7e.1.A   -------------------------------------------TQLFNRAVSMVEKNKDIRSLLQCDDGITGKERLKAYG

Target     YLKLID---------RENF---VDIVDAKLKIPVSGSKSEGLLYVHSSRGGPFQRWHLDEVFLELKDGQQIPVFKLSGEN
6k7e.1.A   ELITNDKWTRNRPIVSTKKLDKEGRTHHYMRFHVESKKKIALVHLEAKESKQNYQPDFINMYVDVPGEKRYYLIK-----

Target     GDEVKKE
6k7e.1.A   -------
```

Model #07	File	Built with	Oligo-State	Ligands	GMQE	QMEAN
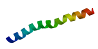	PDB	ProMod3 3.2.0	monomer	None	0.05	-2.05

QMEAN -2.05
Cβ -3.37
All Atom 0.07
solvation -0.86
torsion -1.27

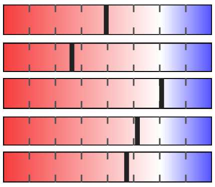

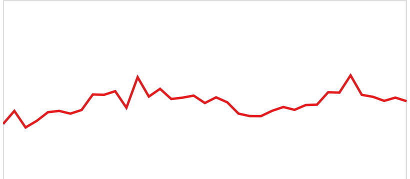

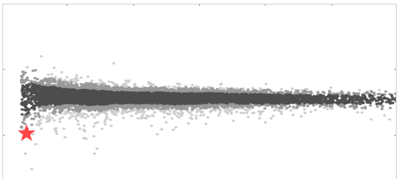

Template	Seq Identity	Oligo-state	QSQE	Found by	Method	Resolution	Seq Similarity	Range	Coverage	Description
2knc.1.B	8.11	monomer	0.00	HHblits	NMR	-	0.25	19 - 55	0.25	Integrin beta-3

The template contained no ligands.

```
Target     MMWQKYAGSRRSMPLGARILFHGVFYAGGFAIVYYLIQKFHSRALYYKLAVEQLQSHPEAQEALGPPLNIHYLKLIDREN
2knc.1.B   ------------------SVMGAILLIGLAALLIWKLLITIHDRKEFAKFEEERA-------------------------

Target     FVDIVDAKLKIPVSGSKSEGLLYVHSSRGGPFQRWHLDEVFLELKDGQQIPVFKLSGENGDEVKKE
2knc.1.B   ------------------------------------------------------------------
```

Materials and Methods

Template Search

Template search with BLAST and HHblits has been performed against the SWISS-MODEL template library (SMTL, last update: 2021-03-25, last included PDB release: 2021-03-19).

The target sequence was searched with BLAST against the primary amino acid sequence contained in the SMTL.

An initial HHblits profile has been built using the procedure outlined in (Steinegger et al.), followed by 1 iteration of HHblits against Uniclust30 (Mirdita, von den Driesch et al.). The obtained profile has then be searched against all profiles of the SMTL. A total of 30 templates were found.

Model Building

Models are built based on the target-template alignment using ProMod3 (Studer et al.). Coordinates which are conserved between the target and the template are copied from the template to the model. Insertions and deletions are remodelled using a fragment library. Side chains are then rebuilt. Finally, the geometry of the resulting model is regularized by using a force field.

Model Quality Estimation

The global and per-residue model quality has been assessed using the QMEAN scoring function (Studer et al.).

Ligand Modelling

Ligands present in the template structure are transferred by homology to the model when the following criteria are met: (a) The ligands are annotated as biologically relevant in the template library, (b) the ligand is in contact with the model, (c) the ligand is not clashing with the protein, (d) the residues in contact with the ligand are conserved between the target and the template. If any of these four criteria is not satisfied, a certain ligand will not be included in the model. The model summary includes information on why and which ligand has not been included.

Oligomeric State Conservation

The quaternary structure annotation of the template is used to model the target sequence in its oligomeric form. The method (Bertoni et al.) is based on a supervised machine learning algorithm, Support Vector Machines (SVM), which combines interface conservation, structural clustering, and other template features to provide a quaternary structure quality estimate (QSQE). The QSQE score is a number between 0 and 1, reflecting the expected accuracy of the interchain contacts for a model built based a given alignment and template. Higher numbers indicate higher reliability. This complements the GMQE score which estimates the accuracy of the tertiary structure of the resulting model.

References

- **BLAST**
  Camacho, C., Coulouris, G., Avagyan, V., Ma, N., Papadopoulos, J., Bealer, K., Madden, T.L. BLAST+: architecture and applications. BMC Bioinformatics 10, 421-430 (2009). doi>
- **HHblits**
  Steinegger, M., Meier, M., Mirdita, M., Vöhringer, H., Haunsberger, S. J., Söding, J. HH-suite3 for fast remote homology detection and deep protein annotation. BMC Bioinformatics 20, 473 (2019). doi>
- **Uniclust30**
  Mirdita, M., von den Driesch, L., Galiez, C., Martin, M.J., Söding, J., Steinegger, M. Uniclust databases of clustered and deeply annotated protein sequences and alignments. Nucleic Acids Research 45, D170–D176 (2016). doi>

Table T1:

Primary amino acid sequence for which templates were searched and models were built.

```
MMWQKYAGSRRSMPLGARILFHGVFYAGGFAIVYYLIQKFHSRALYYKLAVEQLQSHPEAQEALGPPLNIHYLKLIDRENFVDIVDAKLKIPVSGSKSEG
LLYVHSSRGGPFQRWHLDEVFLELKDGQQIPVFKLSGENGDEVKKE
```

Table T2:

Template	Seq Identity	Oligo-state	QSQE	Found by	Method	Resolution	Seq Similarity	Coverage	Description
2ciu.1.A	10.53	monomer	-	HHblits	X-ray	1.60Å	0.27	0.65	IMPORT INNER MEMBRANE TRANSLOCASE SUBUNIT TIM21 MITOCHONDRIAL
6k7f.1.A	10.00	monomer	-	HHblits	X-ray	1.80Å	0.27	0.62	Maltose/maltodextrin-binding periplasmic protein,Mitochondrial import inner membrane translocase subunit TIM21
6k7d.1.A	10.00	monomer	-	HHblits	X-ray	2.00Å	0.27	0.62	Maltose/maltodextrin-binding periplasmic protein,Mitochondrial import inner membrane translocase subunit TIM21
6k7e.1.A	10.99	monomer	-	HHblits	X-ray	1.53Å	0.27	0.62	Maltose/maltodextrin-binding periplasmic protein,Mitochondrial import inner membrane translocase subunit TIM21

Template	Seq Identity	Oligo-state	QSQE	Found by	Method	Resolution	Seq Similarity	Coverage	Description
6k8q.1.A	10.87	monomer	-	HHblits	NMR	NA	0.27	0.63	Mitochondrial import inner membrane translocase subunit TIM21
2mf7.1.A	10.53	monomer	-	HHblits	NMR	NA	0.27	0.65	Mitochondrial import inner membrane translocase subunit TIM21
2knc.1.B	8.11	monomer	-	HHblits	NMR	NA	0.25	0.25	Integrin beta-3
3jcu.1.J	20.00	monomer	-	HHblits	EM	NA	0.28	0.21	Photosystem II reaction center protein J
1s5l.2.I	13.33	monomer	-	HHblits	X-ray	3.50Å	0.25	0.21	Photosystem II reaction center J protein
6kac.1.J	21.88	monomer	-	HHblits	EM	NA	0.29	0.22	Photosystem II reaction center protein J
4rvy.1.Q	13.33	monomer	-	HHblits	X-ray	5.50Å	0.25	0.21	Photosystem II reaction center protein J
5v2c.1.I	13.33	monomer	-	HHblits	X-ray	1.90Å	0.25	0.21	Photosystem II reaction center protein J
4il6.1.I	13.79	monomer	-	HHblits	X-ray	2.10Å	0.26	0.20	Photosystem II reaction center protein J
6yp7.1.O	17.86	monomer	-	HHblits	EM	NA	0.29	0.19	Photosystem II reaction center protein J
5xnl.1.N	16.67	monomer	-	HHblits	EM	NA	0.28	0.21	Photosystem II reaction center protein J
5e79.1.I	13.79	monomer	-	HHblits	X-ray	3.50Å	0.26	0.20	Photosystem II reaction center protein J
4yuu.1.I	20.69	monomer	-	HHblits	X-ray	2.77Å	0.29	0.20	Photosystem II reaction center protein J
6kaf.1.G	20.00	monomer	-	HHblits	EM	NA	0.30	0.21	Photosystem II reaction center protein J, PsbJ
6j3y.1.I	17.24	monomer	-	HHblits	EM	NA	0.27	0.20	Photosystem II reaction center protein J
3a0b.1.I	13.33	monomer	-	HHblits	X-ray	3.70Å	0.25	0.21	Photosystem II reaction center protein J
3kzi.1.I	13.33	monomer	-	HHblits	X-ray	3.60Å	0.25	0.21	Photosystem II reaction center protein J
6xkw.1.D	19.64	monomer	-	HHblits	EM	NA	0.26	0.38	Cytochrome c oxidase, Cbb3-type, biogenesis protein CcoH
3a0h.1.I	13.33	monomer	-	HHblits	X-ray	4.00Å	0.25	0.21	Photosystem II reaction center protein J
6jlu.10.A	18.52	monomer	-	HHblits	EM	NA	0.26	0.18	PsbJ

The table above shows the top 24 filtered templates. A further 4 templates were found which were considered to be less suitable for modelling than the filtered list.
4fby.1.I, 5gti.1.I, 6dhe.1.2, 6dhe.1.I
